# Secretome-Based Identification of ULBP2 as a Novel Serum Marker for Pancreatic Cancer Detection

**DOI:** 10.1371/journal.pone.0020029

**Published:** 2011-05-20

**Authors:** Ya-Ting Chang, Chih-Ching Wu, Yi-Ming Shyr, Tse-Ching Chen, Tsann-Long Hwang, Ta-Sen Yeh, Kai-Ping Chang, Hao-Ping Liu, Yu-Ling Liu, Ming-Hung Tsai, Yu-Sun Chang, Jau-Song Yu

**Affiliations:** 1 Graduate Institute of Biomedical Sciences, Chang Gung University, Tao-Yuan, Taiwan; 2 Molecular Medicine Research Center, Chang Gung University, Tao-Yuan, Taiwan; 3 Department of Medical Biotechnology and Laboratory Science, Chang Gung University, Tao-Yuan, Taiwan; 4 Divisions of General and Transplantation Surgery, Department of Surgery, Taipei Veterans General Hospital, Taipei, Taiwan; 5 Department of Anatomical Pathology, Chang Gung Memorial Hospital, Tao-Yuan, Taiwan; 6 Department of Surgery, Chang Gung Memorial Hospital, Tao-Yuan, Taiwan; 7 Departments of Otolaryngology-Head and Neck Surgery, Chang Gung Memorial Hospital, Tao-Yuan, Taiwan; 8 Department of Cell and Molecular Biology, Chang Gung University, Tao-Yuan, Taiwan; The University of Kansas Medical Center, United States of America

## Abstract

**Background:**

To discover novel markers for improving the efficacy of pancreatic cancer (PC) diagnosis, the secretome of two PC cell lines (BxPC-3 and MIA PaCa-2) was profiled. UL16 binding protein 2 (ULBP2), one of the proteins identified in the PC cell secretome, was selected for evaluation as a biomarker for PC detection because its mRNA level was also found to be significantly elevated in PC tissues.

**Methods:**

ULBP2 expression in PC tissues from 67 patients was studied by immunohistochemistry. ULBP2 serum levels in 154 PC patients and 142 healthy controls were measured by bead-based immunoassay, and the efficacy of serum ULBP2 for PC detection was compared with the widely used serological PC marker carbohydrate antigen 19-9 (CA 19-9).

**Results:**

Immunohistochemical analyses revealed an elevated expression of ULPB2 in PC tissues compared with adjacent non-cancerous tissues. Meanwhile, the serum levels of ULBP2 among all PC patients (n = 154) and in early-stage cancer patients were significantly higher than those in healthy controls (*p*<0.0001). The combination of ULBP2 and CA 19-9 outperformed each marker alone in distinguishing PC patients from healthy individuals. Importantly, an analysis of the area under receiver operating characteristic curves showed that ULBP2 was superior to CA 19-9 in discriminating patients with early-stage PC from healthy controls.

**Conclusions:**

Collectively, our results indicate that ULBP2 may represent a novel and useful serum biomarker for pancreatic cancer primary screening.

## Introduction

Pancreatic cancer (PC) is the fourth-leading cause of cancer-related mortality in the United States with a 5-year survival less than 7% [1], [2]. In 2010, more than 43,000 new PC cases were estimated to develop and 36,800 deaths were expected in the United States [1]. In Taiwan, it is ranked tenth among cancer-related deaths in 2008 and shows increased mortality rate in the last decade [3]. Due to the early spread of PC and the late onset of apparent symptoms, less than 8% of PC patients are diagnosed at the localized stage when a surgical cure is possible [1], [4], [5]. Accordingly, there is an urgent need to develop improved strategies for early detection of PC.

Current approaches for PC diagnosis are mainly based on imaging and endoscopic methods [6], which, because of the retroperitoneal location of the pancreas, have a limited probability of early diagnosis [7], [8]. An elevation in serum levels of carbohydrate antigen 19-9 (CA 19-9) has been widely used for PC detection; however, this approach is insufficient with respect to both specificity and sensitivity [6], [9], [10]. In addition to CA 19-9, more than 40 proteins have been reported as potential serological biomarkers for PC detection [11]. Unfortunately, most either have limited specificity and/or sensitivity, or await validation with a large-scale cohort of specimens [11]–[17], despite evidence that the use of a combinatory biomarker panel improves the accuracy of PC diagnosis [12]. Therefore, discovery of novel and useful serum markers could facilitate the improvement of PC diagnosis and/or prognosis.

Recently, the secretome-based approaches have been widely applied in the identification of potential cancer biomarkers [18], [19]. In the present study, we analyzed the secretome of two PC cell lines, BxPC-3 and MIA PaCa-2 and evaluated one of the identified proteins, UL16 binding protein 2 (ULBP2), as a potential PC biomarker. Immunohistochemical staining results confirmed the elevated levels of ULBP2 in PC tissues compared with adjacent non-cancerous counterparts. Bead-based immunoassays further validated the elevated serum levels of ULBP2 in PC patients versus healthy individuals. Most importantly, the combined use of ULBP2 with CA 19-9 improved the sensitivity and accuracy of PC early detection in this case control study, providing a promising approach for PC diagnosis at an early stage.

## Materials and Methods

### Patient population and clinical specimens

Tumor specimens from 67 PC patients (median age, 64; range: 35–82) were collected in the Chang Gung Memorial Hospital (CGMH), Lin-Kou, Taiwan. The tissue samples were collected at surgery, evaluated by pathologists, and stored in the CGMH Tissue Bank until use. Serum samples from 154 PC patients (median age, 70; range, 31–87) were collected in Taipei Veterans General Hospital, Taipei, Taiwan. Additional blood samples, including 142 serum samples from healthy donors (median age, 58; range, 34–86), 25 plasma samples from healthy donors (median age, 54; range, 29–79), 28 serum samples from nasopharyngeal carcinoma (NPC) patients (median age, 46; range, 31–80), 29 serum samples from colorectal carcinoma (CRC) patients (median age, 61; range, 30–83), and 30 plasma samples from gastric cancer (GC) patients (median age, 66; range, 33–83), were collected in the CGMH, Lin-Kou, Taiwan. Aliquots of these samples were stored at −80°C until use. This research followed the tenets of the Declaration of Helsinki and all subjects signed an informed consent approved by Institutional Review Board of Chang Gung Memorial Hospital or Taipei Veterans General Hospital, Taiwan before their participation in this study and for the use of tissue or blood samples collected before treatment.

### Cell culture

The PC cell lines BxPC-3, MIA PaCa-2, PANC-1 and AsPC-1 were purchased from Bioresource Collection and Research Center (Hsinchu, Taiwan). BxPC-3 and AsPC-1 were maintained in 10% fetal bovine serum (FBS, Biological Industries, Israel)-supplemented RPMI-1640 medium (Invitrogen, CA, USA). MIA PaCa-2 and PANC-1 were maintained in Dulbecco's modified Eagle's medium (DMEM; Invitrogen) with 10% FBS plus 2.5% horse serum (Biological Industries) and 10% FBS, respectively. The four cell lines were cultured at 37°C in a humidified 5% CO_2_ environment.

### Generation of secretome dataset of cancer cell lines

The methods used for preparation of cancer cell conditioned media (CM) and secretome profiling were described previously [20] and detailed in Materials and Methods S1.

### Public domain database search for expression profiles of selected target genes

The expression profiles of the secreted proteins in PC tissues were searched in the National Center for Biotechnology Information (NCBI) Gene Expression Omnibus database (http://www.ncbi.nlm.nih.gov/geo/). The GSE1542 dataset was selected for identifying candidate genes with elevated expression levels in PC cells [21]. The GSE1542 dataset contains the gene expression profiles of pancreatic ductal cells isolated from 24 patients with pancreatic ductal adenocarcinoma and 25 donors with a normal pancreas [21]. The mean intensities of each gene probe in healthy and cancerous groups were calculated to obtain the Tumor/Normal (T/N) ratio; genes with T/N ratios ≥2 were considered up-regulated candidates. Among them, those with *p*-values less than 0.05 calculated by *t*-test were further selected as the most promising candidates for elevated expression in cancerous tissues.

### Western blot analysis

Proteins in cell extracts and CM were separated by SDS-PAGE, transferred onto PVDF membranes (Millipore, MA, USA), and probed with antibodies against transforming growth factor-β-induced protein ig-h3 (BIGH3) (1∶1000 dilution; Santa Cruz Biotechnology, CA, USA), ULBP2 (1∶1000 dilution; R&D Systems, MN, USA) or α-tubulin (1∶10000 dilution; Millipore). Proteins of interest were detected by incubating for 1 hour with the appropriate horseradish peroxidase (HRP)-conjugated secondary antibodies (Santa Cruz Biotechnology), and visualized using an enhanced chemiluminescence system (PerkinElmer, MA, USA).

### Immunohistochemistry

The immunohistochemical staining was performed with antibodies against BIGH3 (1∶100 dilution; Santa Cruz Biotechnology) and ULBP2 (1∶20 dilution; R&D Systems). The staining protocols are described in the Materials and Methods S1. Expression of target proteins was evaluated according to the simplified H score system, which is based on the intensity of cell staining (3, strong; 2, moderate; 1, weak; 0, no cell staining) and the percentage of cell staining [3, ≥90%; 2, 50–89%; 1, 10–49%; 0, 0–9%)]. The two scores were multiplied and then divided by 3 to get the final score. Positive staining was defined as a final score ≥0.67 [22], [23].

### Serum analyses

Serum BIGH3 and CA 19-9 levels were determined using the ELISA kits from R&D Systems and Alpha Diagnostic (TX, USA), respectively, according to the respective manufacturer's instructions. To determine the serum ULBP2 concentration, the in-house sandwich ELISA and bead-based immunoassay were established in our laboratory (see Materials and Methods S1 for details).

### Statistical analysis

Differences in immunohistochemical scores and serum levels of target proteins between groups were tested using either the Wilcoxon test or Kruskal-Wallis test. Correlations between serum levels of target proteins were calculated using the Pearson correlation, and immunohistochemical scores of paired samples from the same subject were compared using paired *t*-tests. Receiver operator characteristic (ROC) curves were constructed by plotting sensitivity versus 1-specificity and areas under the ROC curves (AUCs) were analyzed using the Hanley and McNeil method. All tests were two-sided, and a *p*-value<0.05 was considered statistically significant. All data were processed using SPSS software version 13.0 (IL, USA).

## Results

### Secretome analysis of two pancreatic cancer cell lines

**Figure 1:**
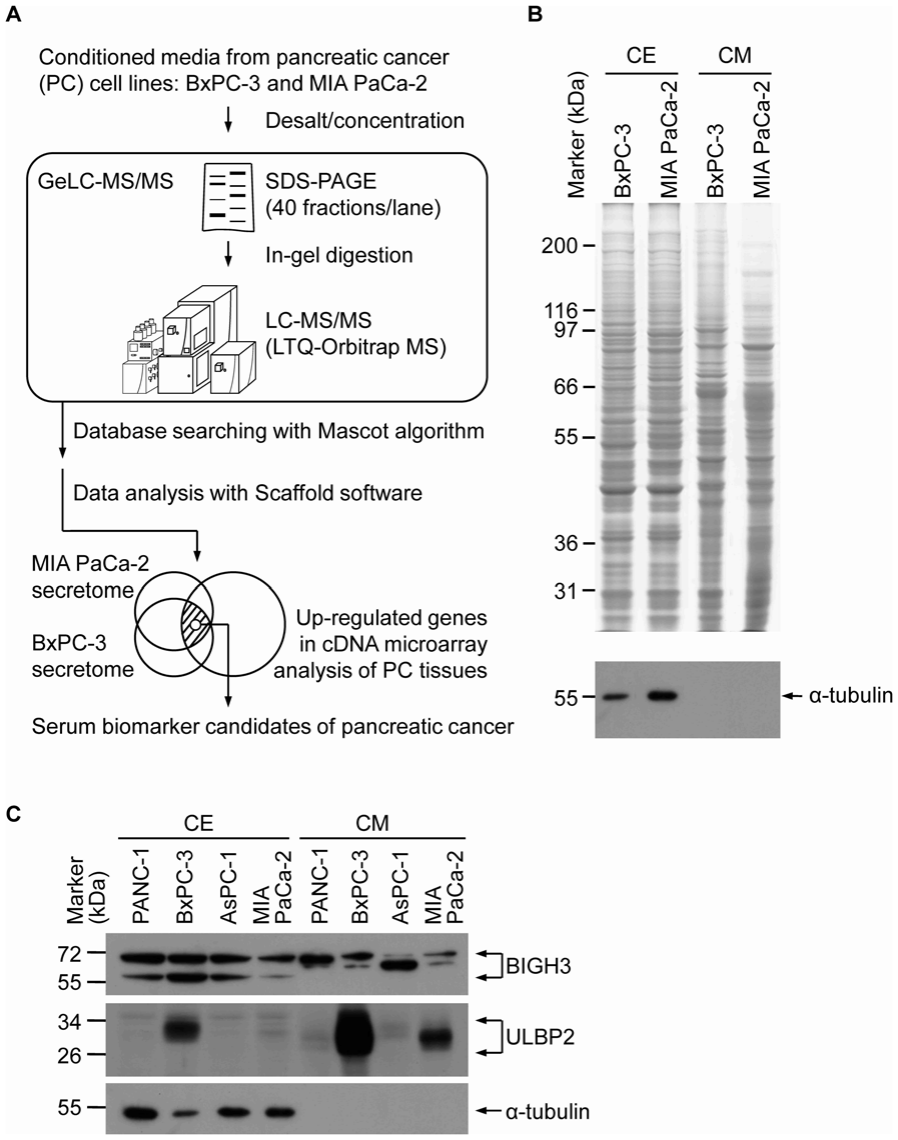
Strategy for identification of potential PC serum biomarkers. (A), The strategy consists of cancer secretome and tissue tranacriptome analysis. (B), The proteins (50 µg) in CM and cell extracts (CE) of the cancer cells were resolved by 10% SDS-PAGE and stained with Coomassie blue (upper panel) or underwent immunoblot analysis with alpha-tubulin (lower panel). (C), Immunoblot analysis with anti-BIGH3 and ULBP2 antibodies.

To discover potential serum PC biomarkers, the secreted proteins of PC cell lines were systemically analyzed. The schematic representation of the flowchart used is illustrated in Fig. 1A. The proteins present in the 24 h serum-free CM of BxPC-3 and MIA PaCa-2 cells were separated by SDS-PAGE, visualized by Coomassie blue staining (Fig. 1B, upper panel), sliced into 40 fractions, digested individually with trypsin and analyzed by C-18 reversed-phase liquid chromatography-tandem mass spectrometry (LC-MS/MS). The protein staining pattern of the cell lysate is also shown in parallel to demonstrate that the secreted proteins were enriched in the CM, and its pattern was quite different from that generated by intracellular proteins (Fig. 1B, upper panel). The distribution of the cytosolic protein, alpha-tubulin, was further examined as a control. Alpha-tubulin was clearly detected in the cell extracts, but not in the CM (Fig. 1B, lower panel), indicating that proteins recovered from the CM was not due to cell death.

After searching with Mascot algorithm and setting a cutoff of 95.0% peptide probability and 95.0% protein probability in Scaffold software, 1011 and 1141 proteins with multiple (≥2) peptide hits were found in the BxPC-3 and MIA PaCA-2 CM, respectively (Supporting Tables S1 and S2), resulting in 1427 non-redundant and 725 overlap proteins.

### Generation of candidate biomarker list for PC detection

To narrow down the candidate list, expression levels of the common 725 proteins in pancreatic ductal cells were examined in an array-based analysis published by Ishikawa et al. [21], in which they compared gene expression in pancreatic ductal cells derived from 24 pancreatic ductal adenocarcinoma patients and 25 donors with a normal pancreas (Fig. 1A). This analysis revealed that 10 of the 725 proteins were significantly upregulated (≥2-fold over-expression, *p*<0.05) in pancreatic ductal carcinoma compared with non-tumor pancreatic ductal cells (Table 1). Among them, ceruloplasmin has been confirmed to be overexpressed in PC tissues [24], and elevated serum level of ERO1-like protein alpha has been reported in PC patients comparing to healthy controls [17], indicating that the combined analysis of the secretome and the transcriptome is a feasible strategy for discovering novel candidate PC markers.

**Table 1:** List of pancreatic cancer cell-secreted proteins that are overexpressed in pancreatic cancer tissue transcriptome.

Protein name (Gene symbol)	NCBI GEO GSE1542^a^		
	Probe ID^b^	T/N ratio^c^	*p*-value^d^
Alpha-soluble NSF attachment protein (NAPA)	208751_at	3.016	0.017
Transforming growth factor-β-induced protein ig-h3 (BIGH3)	201506_at	2.962	0.047
Ras-related protein Rab-14 (RAB14)	200928_s_at	2.300	0.020
UL16 binding protein 2 (ULBP2)	238542_at	2.282	0.019
Ceruloplasmin (CP)	227253_at	2.234	0.012
60S ribosomal protein L22 (RPL22)	238370_x_at	2.099	0.018
Transcriptional activator protein Pur-beta (PURB)	235711_at	2.098	0.006
Complement C1s subcomponent (C1S)	208747_s_at	2.073	0.002
Annexin A11 (ANXA11)	228727_at	2.005	0.002
ERO1-like protein alpha (ERO1L)	218498_s_at	2.001	<0.001

a Data obtained from the NCBI GEO dataset GSE1542 (Ref. 21).

b Probe identities of Affymetrix Human Genome U133A and B arrays.

c The expression ratios are shown as pancreatic ductal carcinoma (T) versus non-cancerous pancreatic ductal cells (N).

d The p-values were determined using t-tests.

### Overexpression of ULBP2 and BIGH3 in PC tissues

**Figure 2:**
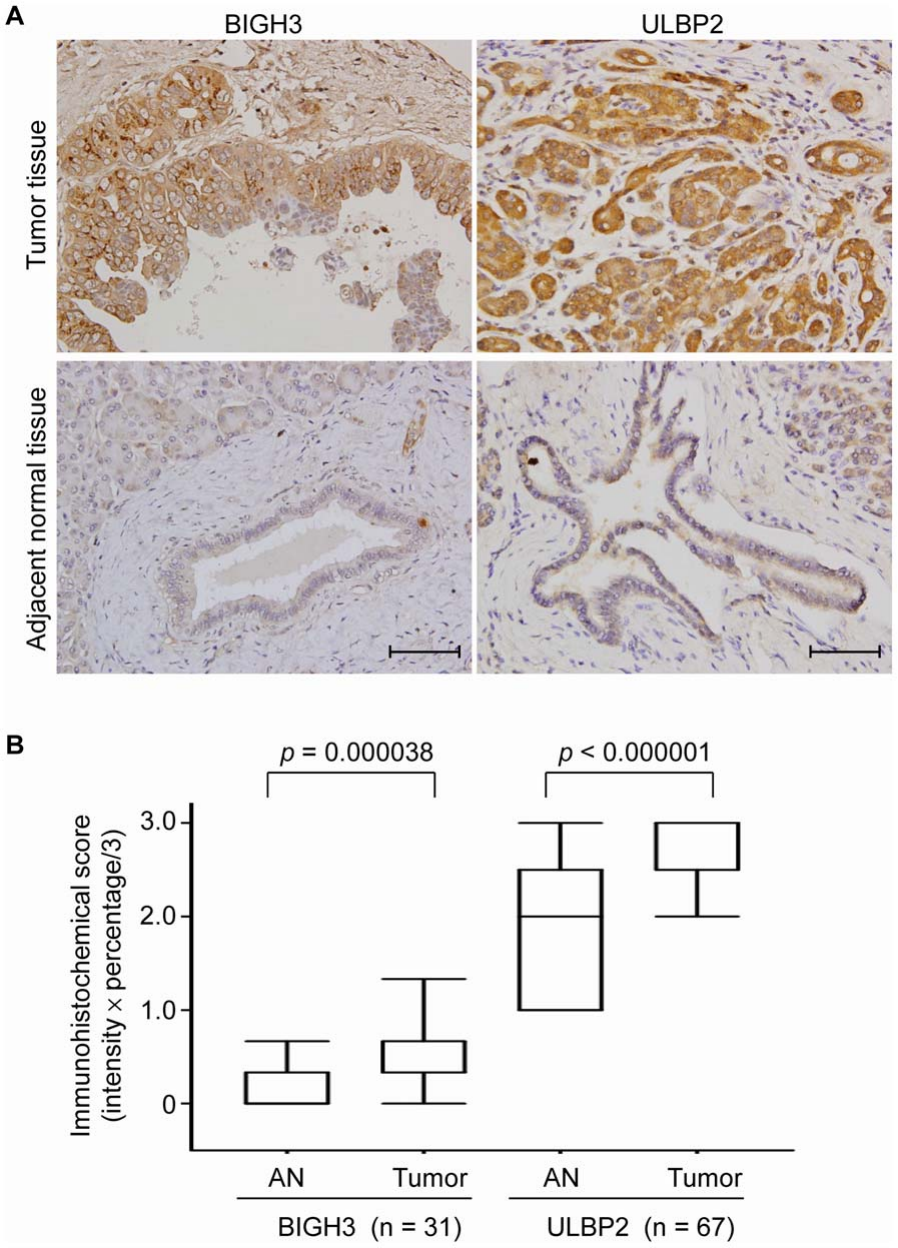
Elevated expression of BIGH3 and ULBP2 in PC tissues. (A), Immunohistochemical staining for BIGH3 (left panel) and ULBP2 (right panel) in paired pericancerous adjacent non-cancerous (lower panel) and tumor (upper panel) tissues. Scale bar, 100 µm. Original magnification, ×400. (B), Box-plot analysis of the immunohistochemical staining scores in paired adjacent non-cancerous (AN) and tumor tissues. The box indicates the 25^th^ and 75^th^ percentiles of the data range; the middle line indicates the median; the dashed line shows the middle 90% distribution.

We focused our attention on ULBP2 and BIGH3 as potential PC markers (Table 1) because another array-based analysis had also shown that their mRNA levels were significantly increased in PC tissues [25], and neither had yet been studied in PC. We confirmed the presence of BIGH3 and ULBP2 in the CM collected from four PC cell lines (BxPC-3, MIA PaCA-2, PANC-1 and AsPC-1) by Western blot analysis (Fig. 1C). Further immunohistochemical analyses showed positive staining for BIGH3 and ULBP2 in 41.9% (13/31) and 100.0% (67/67), respectively, of PC tissue sections. Inspection of each tissue section containing both non-cancerous and cancerous ductal tissues revealed higher expression of both proteins in the cancerous tissues than in the non-cancerous counterparts in most of the tissue sections examined (Fig. 2A; Supporting Figures S1 and S2). The immunohistochemical scores of both proteins in tumor tissues were statistically higher than those in non-cancerous counterparts: 0.54±0.46 versus 0.14±0.27 for BIGH3 (*p*<0.0001) and 2.71±0.49 versus 1.89±0.74 for ULBP2 (*p*<0.0001) (Fig. 2B). Further analyses showed that their expression levels in tumor tissues were not statistically associated with age, gender, histological grade, overall tumor stage, or tumor-node-metastasis (TNM) classification of pancreatic cancer (Supporting Tables S3 and S4).

### Serum levels of BIGH3 and ULBP2 in PC patients

**Figure 3:**
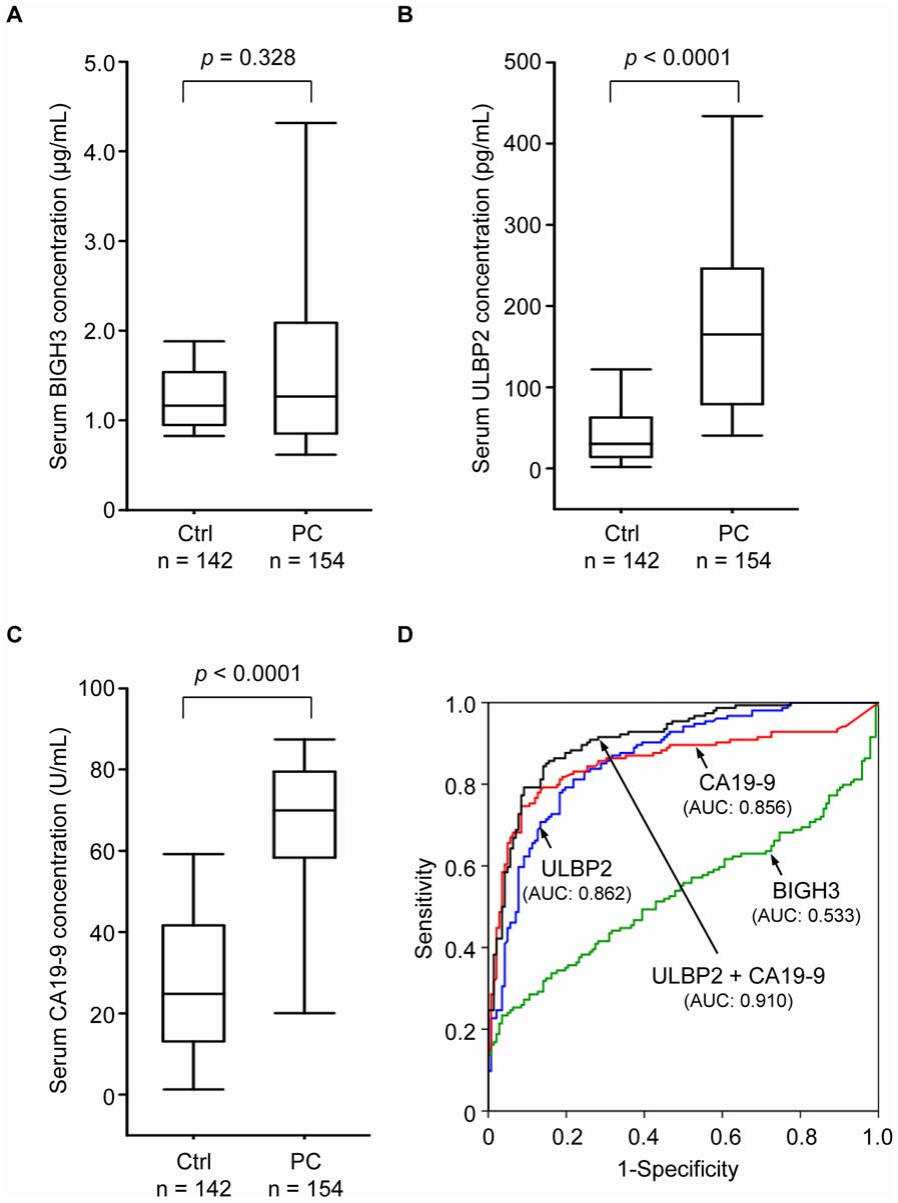
Serum levels of BIGH3, ULBP2, and CA 19-9 in PC patients. The serum levels of BIGH3 (A), ULBP2 (B), and CA 19-9 (C) were measured in 154 pancreatic cancer patients (PC) and 142 healthy controls (Ctrl). Data are presented as Box plots. (D), ROC curve analyses of the ability of BIGH3, ULBP2, CA 19-9, and the two-marker panel comprising ULBP2 and CA 19-9 to discriminate PC patients from controls.

To evaluate the potential of BIGH3 and ULBP2 as serum PC markers, we examined their levels in sera from PC patients (n = 154) and healthy controls (n = 142). We measured serum BIGH3 levels using a commercial ELISA kit and determined serum ULBP2 levels using a bead-based immunoassay developed in our laboratory that is capable of detecting the typically very low levels of serum ULBP2 (<1 ng/mL) that could not be measured precisely using our in-house sandwich ELISA. Our bead-based immunoassay could accurately detect ULBP2 levels over a range of 4.3 pg/mL to 31.9 ng/mL (Supporting Figure S3). We found that the serum levels of ULBP2, but not BIGH3, were significantly elevated in PC patients compared to those in the healthy controls: 1.87±1.67 versus 1.29±0.49 µg/mL for BIGH3 (*p* = 0.328; Fig. 3A) and 200.2±168.6 versus 51.4±64.6 pg/mL for ULBP2 (*p*<0.0001; Fig. 3B). Using a cutoff value of 60 pg/mL for ULBP2, we found that the sensitivity and specificity values for cancer detection were 83.8% and 73.9%, respectively. Serum BIGH3 and ULBP2 levels were not statistically correlated with age, gender, histological grade, overall tumor stage, or TNM classification of PC in this case-control study (Supporting Table S5). These findings indicate that ULBP2 might be a novel serum marker for PC detection.

### The efficacy of ULBP2 and CA 19-9 for PC screening

Serum levels of CA 19-9, a currently used serum PC biomarker, were additionally assayed in the same samples. We found that serum CA 19-9 levels were higher in PC patients than in healthy controls (63.4±24.4 versus 28.3±20.2 U/mL, *p*<0.0001, Fig. 3C); like ULBP2, CA 19-9 levels were not correlated with clinicopathological characteristics (Supporting Table S5). Further analysis showed that there was no correlation between the two molecules in the patient cohort (r = 0.061, *p* = 0.453 by Pearson correlation; Supporting Figure S4). At 40 U/mL, the cutoff value currently applied for PC screening, the sensitivity and specificity values for CA 19-9 were 84.4% and 74.6%, respectively. Notably, applying a cutoff value of 60 pg/mL for ULBP2, we were able to discriminate 21 of 24 PC patients with CA 19-9 levels <40 U/mL from healthy individuals. In addition, 24 of 36 healthy individuals with CA 19-9 levels >40 U/mL could be further distinguished from patients based on ULBP2 levels <60 pg/mL.

The utility of ULBP2 and CA 19-9 as detection markers was further tested by applying a ROC curve analysis. This analysis demonstrated that ULBP2 (AUC = 0.862; 95% confidence interval [CI], 0.821–0.904) performed slightly better than CA 19-9 (AUC = 0.856; 95% CI, 0.809–0.902) as a screening marker (Fig. 3D). Most importantly, a logistic regression model [26] showed that the diagnostic capacity of the combination of ULBP2 and CA 19-9 was greater than that of either marker alone (AUC = 0.910; 95% CI, 0.877–0.943; Fig. 3D). Collectively, these results indicate that ULBP2 might be a useful serum PC marker, especially when used together with CA 19-9.

### The suitability of ULBP2 and CA 19-9 for PC early detection

**Figure 4:**
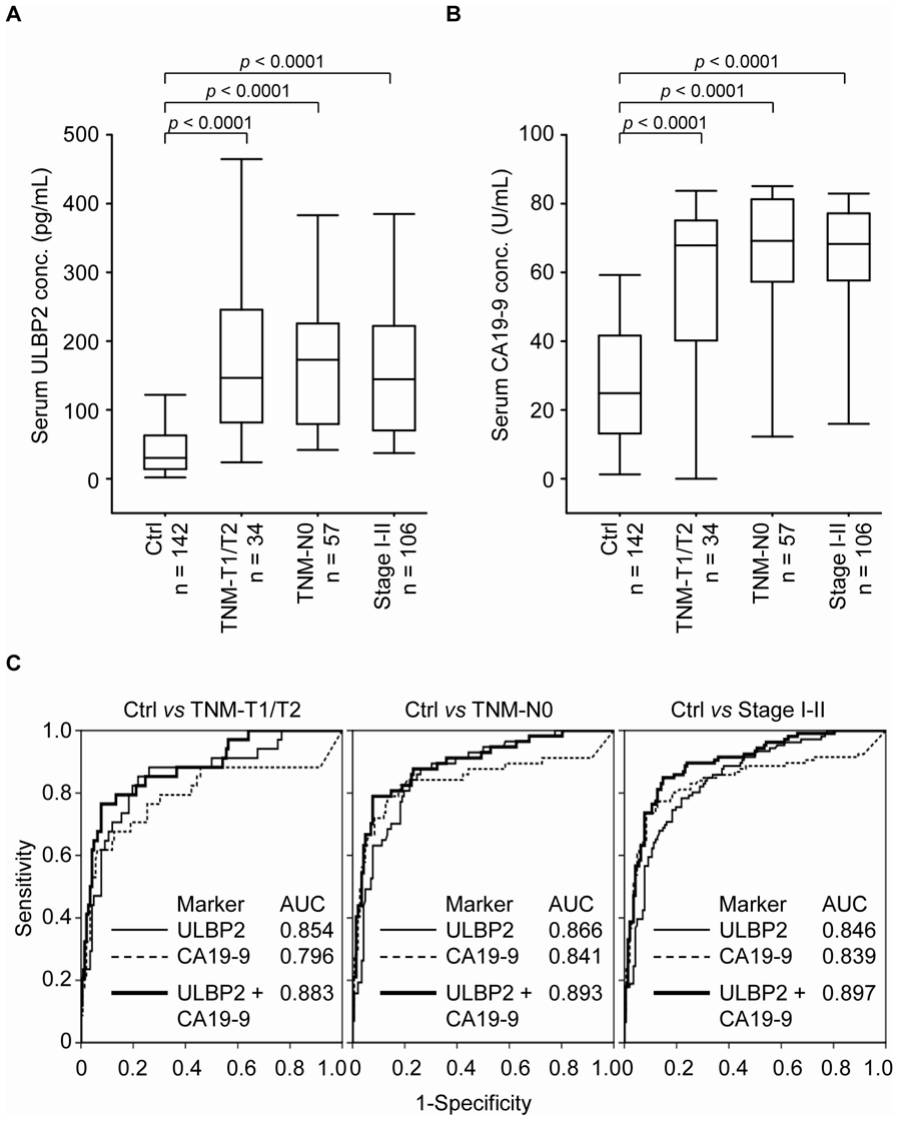
Efficacy of ULBP2 and CA 19-9 for early detection of pancreatic cancer. Serum levels of ULBP2 (A) and CA 19-9 (B) in healthy controls (Ctrl) were compared with those in patients with early-stage PC. Data are presented as Box plots. (C), ROC curve analyses of the ability of ULBP2 (black line), CA 19-9 (dashed line), and a combination of both proteins (thick line) to discriminate early-stage PC patients from controls.

We then evaluated the suitability of ULBP2 as an early detection marker of PC by testing serum ULBP2 levels in patients with early-stage primary tumors (TNM-T1/T2), no lymph node metastasis (TNM-N0), and at early overall tumor stages (stage I–II). We found that serum ULBP2 levels were significantly higher in patients with early-stage primary tumors (205.7±184.3 pg/mL, *p*<0.0001, n = 34), no lymph node metastasis (191.6±155.2 pg/mL, *p*<0.0001, n = 57), and at an early overall tumor stage (181.2±158.8 pg/mL, *p*<0.0001, n = 106) than in healthy controls (51.4±64.6 pg/mL; n = 142; Fig. 4A). Serum CA 19-9 levels were also elevated in the early stages of primary tumor (56.3±26.9 U/mL, *p*<0.0001), lymph node metastasis (62.1±25.5 U/mL, *p*<0.0001), and overall tumor stage (60.7±24.4 U/mL, *p*<0.0001) compared to healthy controls (28.3±20.2 U/mL, n = 142,) (Fig. 4B). ROC curve analyses further showed that ULBP2 was more appropriate than CA 19-9 for discriminating healthy controls from patients with PC diagnosed as TNM-T1/T2 (AUC = 0.854 [95% CI, 0.778–0.930] versus AUC = 0.796 [95% CI, 0.690–0.901]), TNM-N0 (AUC = 0.866 [95% CI, 0.811–0.920] versus AUC = 0.841 [95% CI, 0.764–0.917]) or stage I/II (AUC = 0.846 [95% CI, 0.798–0.895] versus AUC = 0.839 [95% CI, 0.782–0.896]; Fig. 4C). More importantly, the combination of the two markers was better for distinguishing between healthy individuals and TNM-T1/T2- (AUC = 0.883; 95% CI, 0.816–0.949), TNM-N0- (AUC = 0.893; 95% CI, 0.841–0.946), or stage I/II-PC patients (AUC = 0.897; 95% CI, 0.856–0.937) than either marker alone (Fig. 4C). These results imply that ULBP2 is a potentially useful serum marker for PC early detection, particularly in conjunction with CA 19-9.

### Blood ULBP2 levels in patients with other cancer types

**Figure 5:**
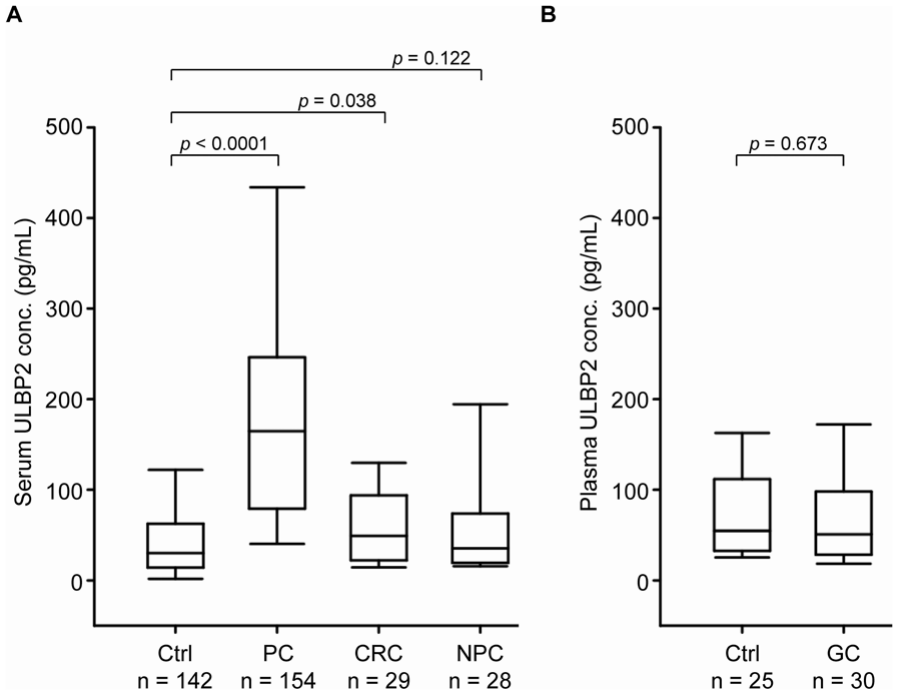
ULBP2 levels in blood specimens from other cancers. (A), Serum ULBP2 levels in healthy controls (Ctrl) were compared to those in patients with nasopharyngeal carcinoma (NPC, n = 28) and colorectal carcinoma (CRC, n = 29). (B), Plasma ULBP2 levels in controls (Ctrl, n = 25) were compared to those in gastric cancer patients (GC, n = 30).

To test whether ULBP2 is also a marker for other malignant diseases, we determined ULBP2 levels in the blood of patients with CRC, NPC, and GC. We found that serum ULBP2 levels trended to be higher in patients suffering from NPC (65.5±74.3 pg/mL, *p* = 0.122, n = 28) compared with healthy controls (51.4±64.6 pg/mL) and were moderately, but significantly, higher in CRC patients (70.6±73.8 pg/mL, *p* = 0.038, n = 29; Fig. 5A); plasma levels of ULPB2 were not significantly different between healthy individuals (86.1±101.2, n = 25) and GC patients (78.1±79.7 pg/mL, n = 30, *p* = 0.673; Fig. 5B). However, serum ULBP2 levels were strikingly elevated in PC patients compared to those in CRC (200.2±168.6 versus 70.6±73.8 pg/mL, *p*<0.0001) and NPC (200.2±168.6 versus 65.5±74.3 pg/mL, *p*<0.0001) patients (Fig. 5A). The observation that ULBP2 levels were unaltered or only marginally elevated in two other gastrointestinal cancers, CRC and GC, suggests that ULBP2 might represent a relatively specific PC marker.

## Discussion

CA 19-9 is currently regarded as the most practicable serum PC marker [27], [28]. However, because CA 19-9 is also elevated in other gastrointestinal diseases, including pancreatitis, hepatitis, biliary obstruction, and other gastrointestinal cancers [9], [27], [28], its specificity is not reliable. Furthermore, it is not expressed in subjects with Lewis a-b- genotype [29], and poorly differentiated PC appears to produce less CA 19-9 than moderately or well differentiated cancers [28], thus limiting its detection sensitivity. Clearly, the identification of novel PC markers that circumvented these limitations would be a welcomed development.

In this study, we used an integrated approach, combined analyses of the PC cell secretome and transcriptome to identify novel PC marker candidates, and provide the first evidence that ULBP2 is a potential novel candidate serum marker for PC diagnosis. ULBP2 was overexpressed in PC cells compared to adjacent non-cancerous tissues (Fig. 2). Bead-based immunoassays performed with more than 150 serum specimens from PC patients revealed that serum ULBP2 levels not only discriminated patients from healthy donors, but also demonstrated great potential for PC early detection (Figs. 3 and 4). Although several potential serum PC markers have been reported, very few have been shown to be superior to CA 19-9 [30], [31]. Importantly, our comparison of serum ULBP2 with CA 19-9 in this case-control study showed that serum ULBP2 is superior to CA 19-9 as an early diagnostic marker (Fig. 4). This finding is especially significant in light of the fact that most PC patients die within one year because they are not diagnosed until the cancer reaches an advanced stage [1]. The diagnostic and prognostic potential of ULBP2 in a clinical setting will need to be verified using a larger series of serum specimens. Moreover, additional candidates listed in Table 1 have not been studied in detail for PC, but their expression levels could be examined in PC tissues using Human Protein Atlas (HPA) database, which contains the immunohistochemical (IHC) staining profiles of 10118 proteins in a variety of cancerous and non-cancerous tissues based on 13154 antibodies (version 7.1, http://www.proteinatlas.org/) [32]. We searched 8 proteins in the HPA database and found 5 of them were detected in more than 50% of the PC sections examined. Noteworthily, alpha-soluble NSF attachment protein, annexin A11, and ERO1-like protein alpha were expressed in more than 50% of the PC sections with moderate to strong IHC staining (Supporting Table S6). These three proteins may represent as potential PC biomarkers that warrant further investigation.

A consensus has coalesced around the idea that effective and accurate detection of early-stage cancer will likely rely on marker panels that possess better specificity and sensitivity than each marker alone [26], [33]. As noted above, CA 19-9 is currently used for PC detection, but its use as a primary screening agent is problematic. Hence, a marker panel that combined CA 19-9 with other useful markers, such as ULBP2, could enhance the ability to detect and monitor cancer. Indeed, the combination of both markers evinced an improved diagnostic efficacy compared with the traditional approach using CA 19-9 alone, especially with respect to the detection of early-stage PC (Fig. 4).

Although ULBP2 has been reported as a tissue and serum prognostic marker for ovarian cancer [34] and melanoma [35], respectively, we believe that ULBP2 still has potential as a relatively specific and useful serum test for PC diagnosis. A number of lines of evidence support this: (a) ULBP2 serum/plasma levels were not significantly elevated in GC, CRC or NPC patients compared to healthy individuals in this study; (b) Li et al. have reported that ULBP2 was not detectable in the sera of ovarian cancer patients [34]; (c) Paschen et al. showed that ULBP2 was not measurable in more than 20% of tested sera from melanoma patients, particularly in early-stage patients [35]; and (d) ULBP2, unlike CA 19-9, does not appear to be expressed at lower levels in poorly differentiated PC than in moderately or well differentiated cancers (Supporting Tables S4 and S5). However, evaluation of ULBP2 as a pancreatic cancer marker will require large-scale counter-screening, particularly using serum samples from pancreatitis patients.

ULBPs and the major histocompatibility complex class I-related chain (MIC) are cell surface ligands for NK and T cell-expressed immunoreceptor (NKG2D) that are rarely expressed by normal cells, but appear in a broad variety of malignancies [36], [37]. These ligands can activate natural killer (NK) and cytotoxic T cells by binding to the NKG2D, and subsequently contribute to cell-mediated cytolysis and clearance of cancer [38], suggesting that tumor cells overexpressing these ligands are more susceptible to cell-mediated immune surveillance. Indeed, MIC expression has been described as an indicator of better prognosis in CRC patients [39], and the release of NKG2D ligands from tumor cells has been previously demonstrated as a novel cancer immunoevasion mechanism [40], [41]. It has recently been proposed that proteolytic shedding of ULBP2 from tumor cells is mediated by matrix metalloproteases (MMP), and may impair the immunogenicity of tumor cells [41]. Via the secretome analysis in this study, several MMPs, including MMP-1, -7, -9, -11, -13, -14, and -28 could be detected in CM from PC cell lines (Supporting Tables S1 and S2). Moreover, elevated expression of MMP-7, -8, -9, and -11 have been described in PC tissues; two of these, MMP-7 and MMP-11, are strongly associated with poor cancer prognosis [42]. These observations collectively suggest that elevated serum ULBP2 levels in PC patients could reflect the involvement of proteolytic cleavage by MMPs released from the cancer itself. By activating NK and cytotoxic T cell-mediated immunity, elimination of the ULBP2 soluble form might be an effective therapeutic strategy in PC. This intriguing possibility warrants further investigation.

In conclusion, we herein report that ULBP2 serum levels in PC patients are significantly higher than those in healthy controls. The combination of ULBP2 and CA 19-9 outperformed each marker alone in distinguishing PC patients from healthy persons. More importantly, an analysis of the area under the ROC curve showed that ULBP2 was superior to CA 19-9 in discriminating patients with early-stage pancreatic cancer from healthy controls. Thus, ULBP2 may represent a novel and useful serum biomarker for primary screening for pancreatic cancer.

## Supporting Information

Figure S1 **Detection of BIGH3 expression in 31 pancreatic cancer tissues by immunohistochemistry.**
(PDF)

Figure S2 **Detection of ULBP2 expression in 67 pancreatic cancer tissues by immunohistochemistry.**
(PDF)

Figure S3 **Standard curve of ULBP2 determined by the bead-based immunoassay developed in house.**
(PDF)

Figure S4 **Correlation of serum ULBP2 and serum CA 19-9 levels.**
(PDF)

Table S1 **List of proteins identified in the BxPC-3 conditioned medium.**
(PDF)

Table S2 **List of proteins identified in the MIA PaCa-2 conditioned medium.**
(PDF)

Table S3 **Correlation between clinicopathological features and BIGH3 expression in tissue sections from 31 pancreatic cancer patients.**
(PDF)

Table S4 **Correlation between clinicopathological features and ULBP2 expression in tissue sections from 67 pancreatic cancer patients.**
(PDF)

Table S5 **Correlation of serum BIGH3, ULBP2, and CA19-9 levels with clinicopathologic characteristics in 154 pancreatic cancer patients.**
(PDF)

Table S6 **Expression profiles of candidate PC markers in the Human Protein Atlas (HPA) database.**
(PDF)

Materials and Methods S1 (PDF)
