# Supplementary material for: Secretome-Based Identification of ULBP2 as a Novel Serum Marker for Pancreatic Cancer Detection
Source: PLoS One. 2011 May 20;6(5):e20029. doi: 10.1371/journal.pone.0020029 (PMC3098863; doi:10.1371/journal.pone.0020029)
Supplement: Figure S1 — Detection of BIGH3 expression in 31 pancreatic cancer tissues by immunohistochemistry. (PDF) [file pone.0020029.s001.pdf]

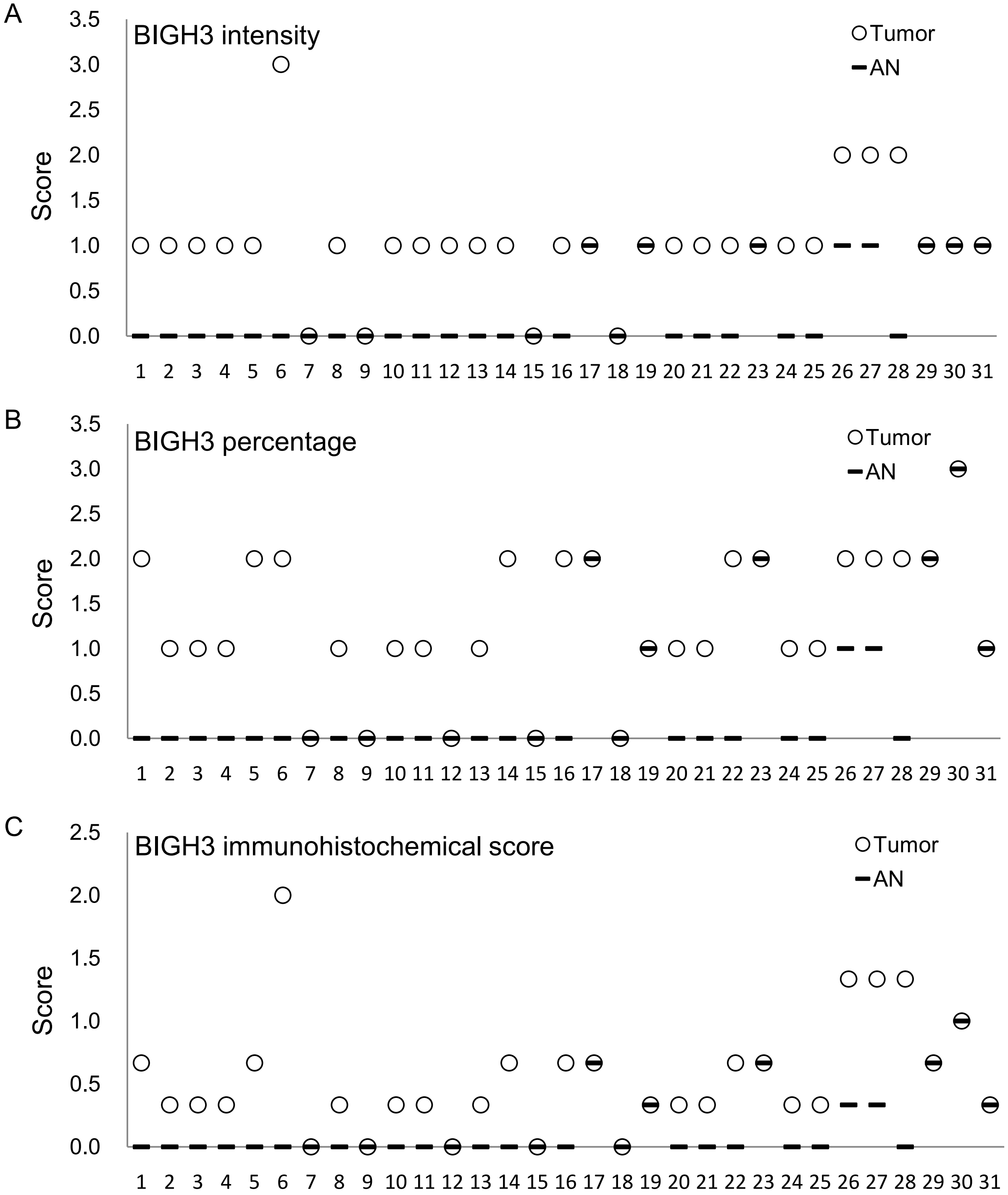

Supporting Figure S1. Detection of BIGH3 expression in 31 pancreatic cancer tissues by immunohistochemistry. The expression patterns of BIGH3 in individual pancreatic cancer tissue in comparison with adjacent normal (AN) tissue are illustrated by (A) BIGH3 intensity, (B) BIGH3 percentage, and (C) BIGH3 immunohistochemical score (calculated as intensity $\times$ percentage/3). The *p*-values in (A), (B), and (C), determined using paired *t*-tests, are 4.05×10^{-7}, 5.23×10^{-7}, and 2.00×10^{-5}, respectively.
