# Supplementary material for: Secretome-Based Identification of ULBP2 as a Novel Serum Marker for Pancreatic Cancer Detection
Source: PLoS One. 2011 May 20;6(5):e20029. doi: 10.1371/journal.pone.0020029 (PMC3098863; doi:10.1371/journal.pone.0020029)
Supplement: Figure S2 — Detection of ULBP2 expression in 67 pancreatic cancer tissues by immunohistochemistry. (PDF) [file pone.0020029.s002.pdf]

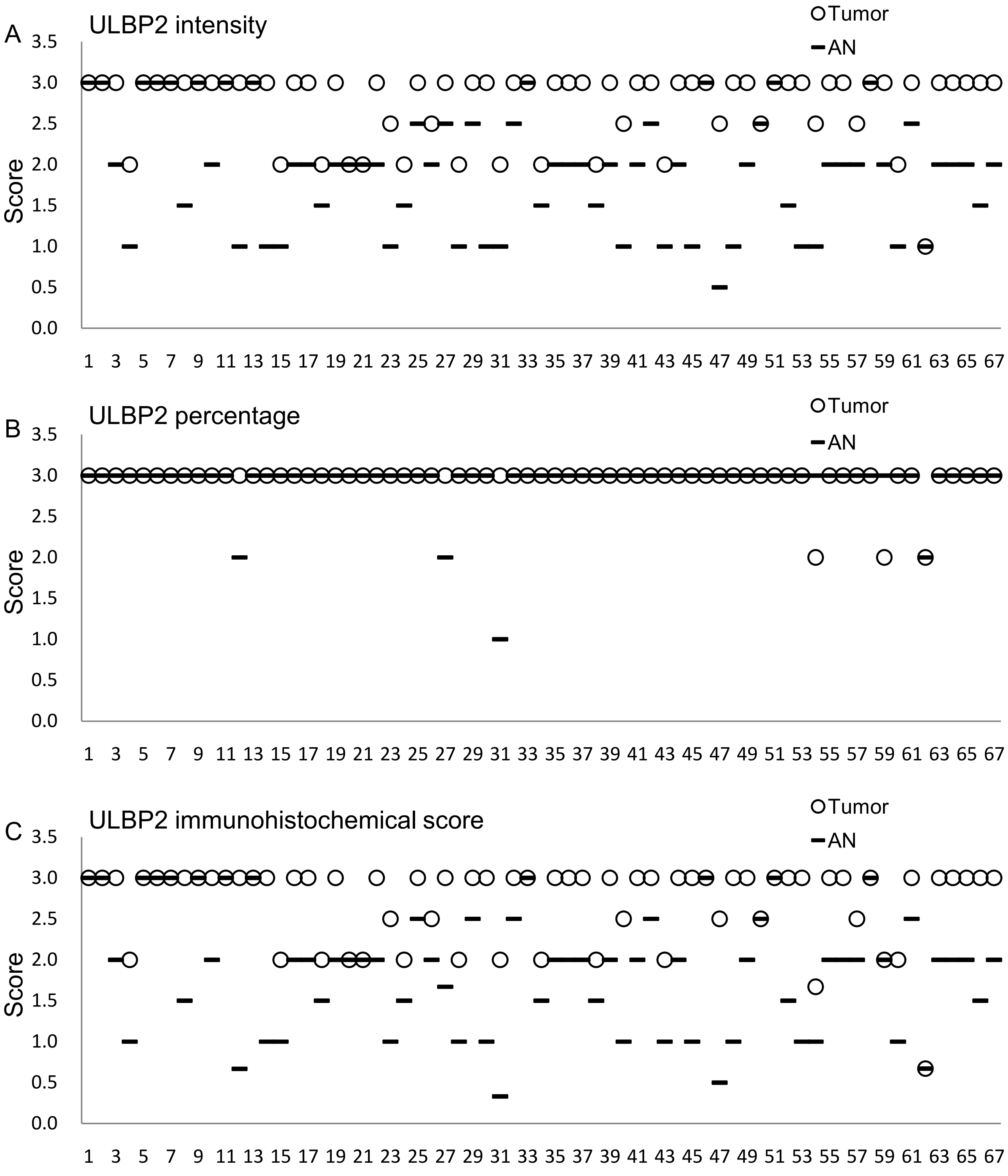

Supporting Figure S2. Detection of ULBP2 expression in 67 pancreatic cancer tissues by immunohistochemistry. The expression patterns of ULBP2 in individual pancreatic cancer tissue in comparison with adjacent normal (AN) tissue are illustrated by (A) ULBP2 intensity, (B) ULBP2 percentage, and (C) ULBP2 immunohistochemical score (calculated as intensity $\times$ percentage/3). The *p*-values in (A), (B), and (C), determined using paired *t*-tests, are 2.66×10^{-16}, 0.48, and 1.26×10^{-15}, respectively.
