# Supplementary material for: Secretome-Based Identification of ULBP2 as a Novel Serum Marker for Pancreatic Cancer Detection
Source: PLoS One. 2011 May 20;6(5):e20029. doi: 10.1371/journal.pone.0020029 (PMC3098863; doi:10.1371/journal.pone.0020029)
Supplement: Figure S3 — Standard curve of ULBP2 determined by the bead-based immunoassay developed in house. (PDF) [file pone.0020029.s003.pdf]

Standard curve (5 parameter logistic regression):
FI = -7.90692 + (30289 + 7.90692) / ((1 + (Conc / 12805.3)^-1.34681))^0.702052
Fit Probability = 0.9589

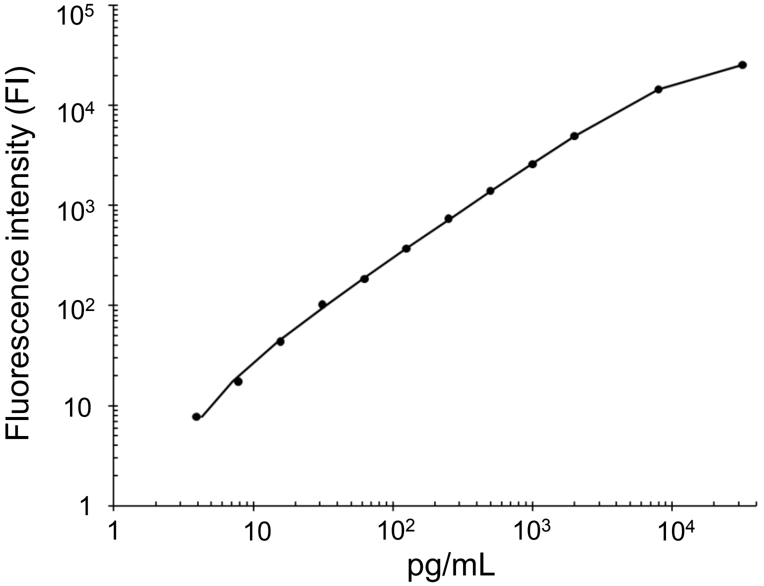


Raw data of standard curve

| Exp. Conc.[a] (pg/mL) | Fluorescence intensity (FI)[b] | | | Obs. Conc.[c] (pg/ml) | %Recovery (Obs./Exp.) |
|---|---|---|---|---|---|
| | mean | SD | %CV | | |
| 0 | 22.8 | 1.77 | 7.77 | 0 | 100% |
| 3.91 | 30.5 | 4.95 | 63.87 | 4.28 | 110% |
| 7.81 | 40.3 | 2.47 | 14.14 | 7.14 | 91% |
| 15.63 | 66.5 | 9.19 | 21.01 | 15.12 | 97% |
| 31.25 | 125 | 9.9 | 9.68 | 33.7 | 108% |
| 62.5 | 207.5 | 7.78 | 4.21 | 60.88 | 97% |
| 125 | 393.8 | 16.62 | 4.48 | 124.6 | 100% |
| 250 | 760.5 | 80.61 | 10.93 | 255.56 | 102% |
| 500 | 1427.5 | 84.85 | 6.04 | 505.18 | 101% |
| 1000 | 2620.8 | 240.06 | 9.24 | 978.4 | 98% |
| 2000 | 4972.5 | 431.34 | 8.71 | 2001.86 | 100% |
| 8000 | 14451 | 42.43 | 0.29 | 8030.71 | 100% |
| 32000 | 25319 | 0.71 | 0 | 31907.98 | 100% |

[a]Expected concentration, the standard concentration we determined.

[b]Data from duplicated points.

[c]Observed concentration, the concentration calculated from standard curve.

**Supporting Figure S3. Standard curve of ULBP2 determined by the bead-based immunoassay developed in house.** The fluorescence intensity of standard protein (from 3.91 pg/mL to 32 ng/mL) was measured by the bead-based immunoassay developed in house and the curve was fitted in 5 parameter logistic regression to obtain the standard curve. Upper figure denotes the standard curve; bottom table shows the raw data of each standard point.
