# Supplementary material for: Secretome-Based Identification of ULBP2 as a Novel Serum Marker for Pancreatic Cancer Detection
Source: PLoS One. 2011 May 20;6(5):e20029. doi: 10.1371/journal.pone.0020029 (PMC3098863; doi:10.1371/journal.pone.0020029)
Supplement: Figure S4 — Correlation of serum ULBP2 and serum CA 19-9 levels. (PDF) [file pone.0020029.s004.pdf]

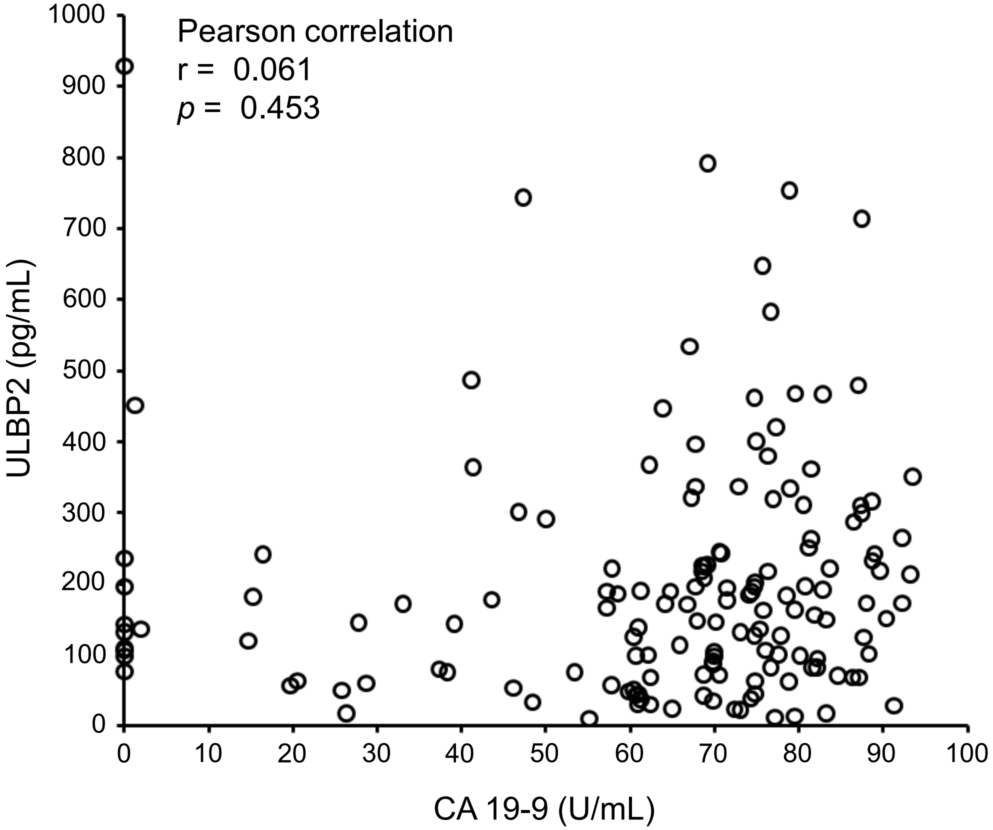

Supporting Figure S4. Correlation of serum ULBP2 and serum CA 19-9 levels. Lack of a significant correlation between the serum levels of ULBP2 and CA 19-9 in 154 pancreatic patients (r = 0.061, p = 0.453, by Pearson correlation test).
