# Supplementary material for: Secretome-Based Identification of ULBP2 as a Novel Serum Marker for Pancreatic Cancer Detection
Source: PLoS One. 2011 May 20;6(5):e20029. doi: 10.1371/journal.pone.0020029 (PMC3098863; doi:10.1371/journal.pone.0020029)
Supplement: Table S1 — List of proteins identified in the BxPC-3 conditioned medium. (PDF) [file pone.0020029.s005.pdf]

Supporting Table S1. List of proteins identified in the BxPC-3 conditioned medium

Protein name	Accession No.	Gene symbol	Molecular weight (Da)	Protein probability	No. of unique peptides	No. of unique spectra	Spectral counts	Sequence coverage	Peptide sequence	Peptide probability	Mascot ion score	Mascot identity score	No. of identified spectra			No. of tryptic termini	Calculated MH+
													2+ spectra	3+ spectra	4+ spectra		
Apolipoprotein A-I-binding protein	AIBP_HUMAN	APOA1BP	31,657	100.00%	4	4	8	20.10%	GLTVPIASIDIPSGWDVEK	95.0%	47.3	20.1	3	0	0	2	1,997.06
									GNAGGIQPDLLISLTAPK	95.0%	64.2	17.8	2	0	0	2	1,764.99
									KSATQFTGR	95.0%	36.0	21.6	1	0	0	2	995.53
									LFGYEPTIYYPK	95.0%	51.2	23.9	2	0	0	2	1,490.76
Kunitz-type protease inhibitor 1	SPIT1_HUMAN	SPINT1	58,379	100.00%	10	11	77	20.20%	AWAGIDLK	95.0%	31.2	24.8	1	0	0	2	873.48
									DPNQVELWGLK	95.0%	45.8	21.3	2	0	0	2	1,298.67
									EGFINYLTR	95.0%	53.5	22.0	26	0	0	2	1,112.57
									GSSGAQATFPQGPSMER	95.0%	67.5	18.5	16	0	0	2	1,723.77
									KDPNQVELWGLK	95.0%	69.1	20.6	7	4	0	2	1,426.77
									SFVYGGCLGNK	95.0%	49.7	21.8	2	0	0	2	1,201.57
									TQGFGGSGIPK	95.0%	37.8	23.4	4	0	0	2	1,048.54
									VQPQEPLVLKDVENTDWR	95.0%	94.9	21.5	1	0	0	2	2,166.12
									WYYDPTEQICK	95.0%	37.0	19.0	2	0	0	2	1,502.66
									YTSGFDELQR	95.0%	61.5	20.6	12	0	0	2	1,215.56
DNA replication licensing factor MCM2	MCM2_HUMAN	MCM2	101,880	100.00%	2	2	4	2.65%	GLALALFGGEPK	95.0%	68.1	21.6	2	0	0	2	1,172.67
									VAVGELTDEDVK	95.0%	64.2	23.0	2	0	0	2	1,274.65
Multifunctional protein ADE2	PUR6_HUMAN	PAICS	47,062	99.50%	2	2	3	6.35%	EIVLADVIDNDSWR	95.0%	36.8	22.4	1	0	0	2	1,644.82
									VTSAHKGPDETLR	95.0%	23.8	21.9	0	0	2	2	1,410.73
Matrilysin	MMP7_HUMAN	MMP7	29,660	100.00%	3	3	4	9.74%	DLPHITVDR	95.0%	31.8	21.6	1	0	0	2	1,065.57
									FYLYDSETK	94.8%	30.2	22.6	1	0	0	2	1,165.54
									NANSLEAK	95.0%	40.7	22.4	2	0	0	2	846.43
Antithrombin-III	ANT3_HUMAN	SERPINC1	52,586	100.00%	7	7	43	10.60%	FDTISEK	95.0%	44.1	22.4	11	0	0	2	839.42
									FSPENTR	95.0%	32.0	23.8	2	0	0	2	850.41
									FSPENTRK	95.0%	32.2	23.6	1	0	0	2	978.50
									LPGIVAEGR	95.0%	49.2	19.0	13	0	0	2	911.53
									LQPLDFK	95.0%	34.7	23.9	2	0	0	2	860.49
									RVWELSK	95.0%	33.6	22.4	4	0	0	2	917.52
									TSDQIHFFFAK	95.0%	57.3	22.1	10	0	0	2	1,340.66
Tumor protein D52	TPD52_HUMAN	TPD52	24,310	100.00%	3	3	5	16.10%	LGINSLQELK	95.0%	40.3	21.5	1	0	0	2	1,114.65
									TSETLSQAGQK	95.0%	62.2	23.1	2	0	0	2	1,149.58
									VEEEIQTLSQVLAAK	95.0%	55.2	20.9	2	0	0	2	1,657.90
Histone H2B type 1-C/E/F/G/I	H2B1C_HUMAN	HIST1H2BC	13,889	100.00%	5	5	17	38.90%	AMGIMNSFVNDIFER	95.0%	114.0	21.8	13	0	0	2	1,743.82
									EIQTAVR	95.0%	34.3	23.8	1	0	0	2	816.46
									IAGEASR	95.0%	41.5	24.5	1	0	0	2	703.37
									LLLPGELAK	95.0%	32.5	13.8	1	0	0	2	953.60
									QVHPDTGISSK	95.0%	45.9	22.5	0	1	0	2	1,168.60
Kynureninase	KYNU_HUMAN	KYNU	52,335	100.00%	5	5	12	11.40%	FTNLLTSILDSAETK	95.0%	101.0	21.6	4	0	0	2	1,652.87
									FTNLLTSILDSAETKN	95.0%	62.8	21.4	4	0	0	2	1,766.92
									IAAYGHEVGK	95.0%	64.8	22.5	2	0	0	2	1,044.55
									TYLEEELDKWAK	95.0%	38.9	23.1	1	0	0	2	1,524.76
									VAPVPLYNSFHDVYK	95.0%	44.0	22.5	1	0	0	2	1,748.90
Serine/threonine-protein phosphatase 2B catalytic subunit alpha isoform	PP2BA_HUMAN	PPP3CA	58,672	100.00%	4	4	7	10.20%	DAMPSDANLNSINK	95.0%	39.2	19.9	2	0	0	2	1,505.69
									GLTPTGMLPSGVLSGGK	95.0%	61.0	22.2	2	0	0	2	1,587.84
									IITEGASILR	95.0%	45.4	19.3	1	0	0	2	1,072.64
									LFEVGGSPANTR	95.0%	48.4	23.8	2	0	0	2	1,247.64
Heterogeneous nuclear ribonucleoproteins A2/B1	ROA2_HUMAN	HNRNPA2B1	37,412	100.00%	17	20	314	43.60%	DYFEEYGK	95.0%	33.2	15.4	2	0	0	2	1,050.44
									EDTEEHHLR	95.0%	44.3	20.9	5	0	0	2	1,165.52

									EESGKPGAHVTVK	95.0%	55.6	22.6	8	0	0	2	1,338.70
									GFGFVTFDDHDPVDK	95.0%	86.5	20.5	23	14	0	2	1,695.77
									GFGFVTFDDHDPVDKIVLQK	95.0%	39.2	21.3	0	9	0	2	2,277.16
									GGGGNFGPGPGSNFR	95.0%	66.3	21.1	24	0	0	2	1,377.63
									GGNFGFGDSR	95.0%	63.1	17.4	19	0	0	2	1,013.44
									GGSDGYGSGR	95.0%	74.0	16.1	2	0	0	2	912.38
									IDTIEIITDR	95.0%	101.0	23.1	30	0	0	2	1,188.65
									KLFIGGLSFETTEESLR	95.0%	80.3	20.7	2	1	0	2	1,927.02
									LFIGGLSFETTEESLR	95.0%	109.0	21.8	112	0	0	2	1,798.92
									LFVGGIK	95.0%	34.6	16.5	1	0	0	2	733.46
									LFVGGIKEDTEEHHLR	95.0%	41.6	21.8	0	17	1	2	1,879.97
									LTDCVVMR	95.0%	35.6	22.9	2	0	0	2	993.49
									NYYEQWGK	95.0%	37.4	19.1	2	0	0	2	1,087.48
									QEMQEVQSSR	95.0%	68.4	19.7	29	0	0	2	1,237.55
									TLETVPLER	95.0%	40.4	20.9	11	0	0	2	1,057.59
Glutaredoxin-3	GLRX3_HUMAN	GLRX3	37,415	100.00%	5	5	8	17.90%	AYSNWPTYPQLYVK	95.0%	32.0	22.2	1	0	0	2	1,729.86
									ENGELLPILR	95.0%	46.4	19.1	2	0	0	2	1,153.66
									GELVGGLDIVK	95.0%	35.5	21.6	2	0	0	2	1,099.64
									LEAEGVPEVSEK	95.0%	43.9	22.8	2	0	0	2	1,286.65
									YEISSVPTFLFFK	95.0%	44.0	22.6	1	0	0	2	1,577.83
Laminin subunit gamma-1	LAMC1_HUMAN	LAMC1	177,583	100.00%	26	29	355	19.20%	AFDITYVR	95.0%	54.3	21.6	12	0	0	2	984.52
									AHVENTER	95.0%	34.5	20.7	1	0	0	2	955.46
									ALAEEAAK	95.0%	48.1	21.6	9	0	0	2	802.43
									CELCDDGYFGDPLGR	95.0%	71.6	14.1	2	0	0	2	1,773.72
									CIYNTAGFYCDR	95.0%	33.8	14.6	1	0	0	2	1,539.64
									DGFFGNPLAPNPADK	95.0%	71.3	21.8	16	0	0	2	1,559.75
									DGSEASLEWSSER	95.0%	73.9	16.5	5	0	0	2	1,452.62
									DTLQEANDILNNLK	95.0%	79.1	21.6	9	0	0	2	1,600.82
									EAQDVKDVDQNLMDR	95.0%	45.7	22.1	3	2	0	2	1,791.82
									EAQQALGSAAADATEAK	95.0%	133.0	22.7	26	6	0	2	1,631.79
									EVVCTNCPTGTTGK	95.0%	41.7	19.8	1	0	0	2	1,523.68
									GKTEQQTADQLLAR	95.0%	88.3	22.9	5	0	0	2	1,558.82
									HKQEADDIVR	95.0%	47.0	23.1	0	23	0	2	1,210.62
									LIEIASR	95.0%	53.3	22.3	4	0	0	2	801.48
									LNEIEGTLNK	95.0%	52.3	24.2	9	0	0	2	1,130.61
									LNTFGDEVFNDPK	95.0%	88.2	22.7	36	0	0	2	1,495.71
									LSAEDLVLEGAGLR	95.0%	123.0	21.8	58	2	0	2	1,442.79
									MEAENLEQLIDQK	95.0%	92.1	21.8	20	0	0	2	1,576.75
									NTIEETGNLAEQAR	95.0%	98.8	22.3	21	0	0	2	1,545.75
									QDIAVISDSYFPR	95.0%	88.3	22.8	20	0	0	2	1,510.75
									QLQEAEK	95.0%	39.5	22.0	1	0	0	2	845.44
									TEQQTADQLLAR	95.0%	60.2	22.6	2	0	0	2	1,373.70
									TFAEVTDLDNEVNNMLK	95.0%	110.0	21.6	13	0	0	2	1,968.92
									TGQCECQPGITGQHCER	95.0%	69.2	14.5	0	11	0	2	2,017.83
									TKEVVCTNCPTGTTGK	95.0%	57.1	21.7	1	0	0	2	1,752.83
									VSVPLIAQGNSYPSETTVK	95.0%	109.0	20.5	36	0	0	2	1,990.05
Flap endonuclease 1	FEN1_HUMAN	FEN1	42,576	100.00%	4	4	9	15.30%	EAHQLFLEPEVLDPESVELK	95.0%	43.2	20.8	0	2	0	2	2,322.19
									KLPIQEFHLSR	95.0%	27.6	17.6	0	1	0	2	1,367.78
									LIADVAPSAIR	95.0%	54.0	17.2	3	0	0	2	1,125.66
									QLQQAQAAGAEQEVEK	95.0%	107.0	22.1	3	0	0	2	1,727.86
Phosphoserine phosphatase	SERB_HUMAN	PSPH	24,990	100.00%	2	2	2	11.60%	LIAEQPPHLTPGIR	95.0%	32.1	17.6	0	1	0	2	1,541.88

									LNIPATNVFANR	95.0%	50.7	21.6	1	0	0	2	1,329.73
Complement C1r subcomponent	C1R_HUMAN	C1R	80,102	100.00%	14	14	67	22.60%	FCGQLGSPLGNPPGK	95.0%	82.3	22.4	3	0	0	2	1,528.76
									FCGQLGSPLGNPPGKK	95.0%	44.7	21.8	0	4	0	2	1,656.85
									GGGALLGDR	95.0%	53.4	21.5	4	0	0	2	815.44
									IAHDLR	95.0%	35.3	19.2	2	0	0	2	724.41
									LGNHPIR	95.0%	40.9	20.5	2	0	0	2	806.46
									LPVANPQACENWLR	95.0%	77.4	22.4	4	0	0	2	1,667.83
									MGNFPWQVFTNIHGR	95.0%	62.2	23.2	0	12	0	2	1,819.87
									QDACQGDSGGVFAVR	95.0%	39.2	19.2	1	0	0	2	1,566.70
									QRPPDLDTSSNAVDLLFFTDESGDSR	95.0%	71.1	20.3	0	5	0	2	2,882.34
									TLDEFTIIQNLQPQYQFR	95.0%	88.4	22.2	10	0	0	2	2,254.15
									VLNYVDWIK	95.0%	50.3	21.5	5	0	0	2	1,149.63
									VLNYVDWIKK	95.0%	29.4	18.8	0	1	0	2	1,277.73
									WILTAAHTLYPK	95.0%	67.2	20.0	2	0	0	2	1,413.79
									YTTTMGVNTYK	95.0%	54.9	21.2	12	0	0	2	1,294.60
Eukaryotic translation initiation factor 3 subunit A	EIF3A_HUMAN	EIF3A	166,557	100.00%	3	3	4	3.62%	EQPEKEPELQQYVPQLQNNTILR	95.0%	27.4	20.6	0	1	0	2	2,794.44
									FNVLQYVVPEVK	95.0%	50.5	19.0	2	0	0	2	1,434.80
									LTSLVPFVDAFQLER	95.0%	33.4	20.2	1	0	0	2	1,734.94
Crk-like protein	CRKL_HUMAN	CRKL	33,759	100.00%	2	2	3	8.91%	IHYLDTTTLIEPAPR	95.0%	32.4	20.9	0	2	0	2	1,739.93
									TALALEVGDIVK	95.0%	45.0	19.7	1	0	0	2	1,228.72
Rap1 GTPase-GDP dissociation stimulator 1	GDS1_HUMAN	RAP1GDS1	66,300	99.90%	2	2	3	4.28%	EQFASTNIAEELVK	95.0%	54.1	22.6	2	0	0	2	1,578.80
									SVAQQASLTEQR	95.0%	73.8	23.3	1	0	0	2	1,317.68
Eukaryotic initiation factor 4A-III	IF4A3_HUMAN	EIF4A3	46,854	100.00%	4	4	6	16.50%	ETQALILAPTR	95.0%	48.5	19.8	2	0	0	2	1,212.70
									GIYAYGFEKPSAIQQR	95.0%	71.7	21.2	3	2	0	2	1,827.94
									GRDVIAQSQSGTGK	95.0%	66.6	23.3	1	0	0	2	1,403.72
									LDYGQHVVAGTPGR	95.0%	28.4	22.1	0	1	0	2	1,469.75
									MLVLDEADEMLNK	95.0%	64.5	22.1	2	0	0	2	1,552.72
T-complex protein 1 subunit alpha	TCPA_HUMAN	TCP1	60,327	100.00%	8	9	17	18.50%	AFHNEAQVNPER	95.0%	39.6	21.3	2	0	0	2	1,411.67
									AFHNEAQVNPERK	95.0%	28.8	22.1	0	1	1	2	1,539.77
									FATEAAITILR	95.0%	35.5	19.2	2	0	0	2	1,205.69
									MLVDDIGDVTITNDGATILK	95.0%	127.0	21.5	2	0	0	2	2,120.08
									QAGVFEPTIVK	95.0%	38.5	21.8	2	0	0	2	1,188.66
									SLLVIPNTLAVNAAQDSTDLVAK	95.0%	95.7	15.4	4	0	0	2	2,353.30
									SSLGPVGLDK	95.0%	32.7	21.5	1	0	0	2	972.54
									YINENLIVNTDELGR	95.0%	61.0	22.5	2	0	0	2	1,762.90
Serpin B9	SPB9_HUMAN	SERPINB9	42,386	100.00%	13	15	57	41.20%	ADLSAMSAER	95.0%	76.8	20.8	9	0	0	2	1,066.48
									AFQSLLTEVNK	95.0%	77.6	21.1	5	0	0	2	1,249.68
									AQLLELPYAR	95.0%	59.0	22.1	7	0	0	2	1,173.66
									GKWNEPFDETYTR	95.0%	51.8	20.4	1	2	0	2	1,642.75
									GNTATQMAQALSLNTEEDIHR	95.0%	28.7	21.5	0	2	0	2	2,300.09
									HLGIVDAFQQGK	95.0%	67.4	21.4	4	1	0	2	1,312.70
									IEELLPGSSIDAETR	95.0%	85.9	23.1	3	0	0	2	1,629.83
									INQEEQRPVQMMYQEATFK	95.0%	63.3	21.6	0	7	0	2	2,370.12
									LAHVGEVR	95.0%	55.9	19.0	4	0	0	2	880.50
									LQEDYDMESVLR	95.0%	65.1	22.0	3	0	0	2	1,497.69
									LVLVNAIYFK	95.0%	32.0	14.0	4	0	0	2	1,179.71
									STEVEVLLPK	95.0%	61.4	21.8	5	0	0	2	1,114.64
									TEGKIEELLPGSSIDAETR	95.0%	50.0	22.3	0	2	0	2	2,045.04
									WNEPFDETYTR	95.0%	35.5	19.3	2	0	0	2	1,457.63
Neutral amino acid transporter B(0)	AAAT_HUMAN	SLC1A5	56,582	100.00%	3	3	31	8.13%	EVLDSFLDLAR	95.0%	69.9	22.7	9	0	0	2	1,277.67
									NIFPSNLVSAAFR	95.0%	35.7	21.7	2	0	0	2	1,435.77

									SELPLDPLPVPTEEGNPLLK	95.0%	69.7	18.7	20	0	0	2	2,158.17
Integrin alpha-3	ITA3_HUMAN	ITGA3	118,740	100.00%	9	9	25	9.57%	AAFVSEQQQK	95.0%	50.1	23.2	6	0	0	2	1,135.57
									ARPVINIVHK	95.0%	34.1	11.5	0	3	0	2	1,146.71
									EAGNPGSLFGYSVALHR	95.0%	72.3	22.6	2	0	0	2	1,774.89
									GNSYMIQR	95.0%	39.7	20.2	2	0	0	2	984.46
									LQSFFGGTVMGESGMK	95.0%	91.2	21.9	4	0	0	2	1,691.78
									TVEDVGSPLK	95.0%	47.0	22.6	1	0	0	2	1,044.56
									VYIYHSSSK	95.0%	30.5	21.9	1	0	0	2	1,083.55
									YLLLAGAPR	95.0%	60.5	19.5	4	0	0	2	973.58
									YTQVLWSGSEDQR	95.0%	67.9	22.0	2	0	0	2	1,568.73
Glia-derived nexin	GDN_HUMAN	SERPINE2	43,985	100.00%	4	4	20	14.10%	DMIDNLLSPDLIDGVLTR	95.0%	69.7	22.2	9	0	0	2	2,016.03
									SYQVPMLAQLSVFR	95.0%	64.0	21.3	4	0	0	2	1,654.86
									TIDSWMSIMVPK	95.0%	64.9	23.0	2	0	0	2	1,439.69
									VLGITDMFDSSK	95.0%	74.6	22.3	5	0	0	2	1,328.64
Major vault protein	MVP_HUMAN	MVP	99,308	100.00%	12	12	22	18.90%	ALQPLEEGEDEEK	95.0%	40.0	21.3	2	0	0	2	1,486.69
									DITPLQVVLPNTALHLK	95.0%	45.3	13.6	2	0	0	2	1,872.10
									DLAVAGPEMQVK	95.0%	58.1	22.1	4	0	0	2	1,257.65
									DQAVFPQNGLVVSSVDVQSVEPVDQR	95.0%	37.1	21.2	0	1	0	2	2,812.41
									HYCTVANPVSR	95.0%	53.1	22.5	1	0	0	2	1,303.62
									LAQDPFPLYPGEVLEK	95.0%	60.8	20.6	3	0	0	2	1,815.95
									LFSVPDFVGDACK	95.0%	35.7	22.1	1	0	0	2	1,454.70
									QAIPLDENEGIYVQDVK	95.0%	74.8	21.9	2	0	0	2	1,930.98
									QMTEAIGPSTIR	95.0%	40.3	23.9	2	0	0	2	1,303.67
									TAVFGFETSEAK	95.0%	49.7	22.0	2	0	0	2	1,286.63
									VLFAPMR	94.6%	30.1	22.6	1	0	0	2	849.47
									VPHNAAVQVYDYR	95.0%	26.8	22.8	0	1	0	2	1,531.77
Receptor-type tyrosine-protein phosphatase kappa	PTPRK_HUMAN	PTPRK	162,085	100.00%	13	15	95	10.60%	CVTQSER	95.0%	35.2	21.7	1	0	0	2	879.40
									ESEETIIQTDEDVPGPVPVK	95.0%	82.2	22.0	4	0	0	2	2,182.08
									GLNPGTLNILVR	95.0%	65.7	16.3	13	0	0	2	1,266.75
									GSGVSNFAQLIVR	95.0%	73.5	21.4	22	0	0	2	1,347.74
									IAEIQAR	95.0%	41.5	21.7	2	0	0	2	800.46
									KESEETIIQTDEDVPGPVPVK	95.0%	46.9	21.9	0	5	0	2	2,310.17
									LWHLDPDTEYEIR	95.0%	45.2	21.8	2	8	0	2	1,686.81
									MILTNPEGR	95.0%	33.3	23.9	1	0	0	2	1,030.54
									MTSGSWTETHAVNAPTYK	95.0%	55.3	20.2	3	0	0	2	1,996.91
									NGEDIPVAQTK	95.0%	38.7	22.5	4	0	0	2	1,171.60
									TDQDLYR	95.0%	35.1	21.3	5	0	0	2	910.43
									TYQGFWNPPLAPR	95.0%	53.1	23.0	2	0	0	2	1,546.78
									VLLTRPGEGGTGLPGPPLITR	95.0%	58.2	12.8	3	20	0	2	2,101.21
Ras-related protein Rap-1b	RAP1B_HUMAN	RAP1B	20,807	100.00%	3	3	5	16.80%	EQGQNLAR	95.0%	45.7	20.6	1	0	0	2	915.46
									INVNEIFYDLVR	95.0%	90.8	21.8	3	0	0	2	1,494.80
									LVVLGSGGVGK	95.0%	45.1	17.8	1	0	0	2	985.60
Ras-related protein Rab-11B	RB11B_HUMAN	RAB11B	24,471	100.00%	5	5	20	30.30%	AQIWDTAGQER	95.0%	54.2	21.8	3	0	0	2	1,274.61
									GAVGALLVYDIAK	95.0%	70.3	17.9	9	0	0	2	1,289.75
									NNLSFIETSALDSTNVEEAFK	95.0%	117.0	21.4	2	0	0	2	2,329.12
									STIGVEFATR	95.0%	53.8	21.4	2	0	0	2	1,080.57
									VVLIGDSGVGK	95.0%	64.8	19.8	4	0	0	2	1,043.61
10 kDa heat shock protein, mitochondrial	CH10_HUMAN	HSPE1	10,914	100.00%	6	7	25	60.80%	GGIMLPEK	95.0%	51.9	25.3	2	0	0	2	860.46
									GKGGEIQPVSVK	95.0%	41.9	21.6	1	0	0	2	1,198.68
									KFLPLFDR	95.0%	30.9	19.3	1	0	0	2	1,035.60
									SAAETVTK	95.0%	39.8	24.1	2	0	0	2	806.43

									VLQATVVAVGSGSK	95.0%	97.7	19.1	2	0	0	2	1,315.76
									VVLDDKDYFLFR	95.0%	71.9	22.1	4	13	0	2	1,529.80
Insulin-degrading enzyme	IDE_HUMAN	IDE	117,956	99.50%	2	2	3	2.65%	ESLDDLTNLVVK	95.0%	38.1	22.8	1	0	0	2	1,345.72
									NEFIPTNFEILPLEK	95.0%	47.5	21.4	2	0	0	2	1,803.95
Fermitin family homolog 3	URP2_HUMAN	FERMT3	75,937	100.00%	3	3	5	7.35%	LEGSAPTDVLDSLTTIPELK	95.0%	68.2	20.4	2	0	0	2	2,099.11
									SQDEAPGDPIQQLNLK	95.0%	32.1	22.4	1	0	0	2	1,752.88
									VVLAGGVAPALFR	95.0%	55.1	15.4	2	0	0	2	1,269.77
Vasodilator-stimulated phosphoprotein	VASP_HUMAN	VASP	39,811	100.00%	5	6	18	12.10%	DESANQEEPEAR	95.0%	43.7	16.2	2	0	0	2	1,374.58
									QEEASGGPTAPK	94.8%	30.2	21.4	1	0	0	2	1,171.56
									QVWGLNFGSK	95.0%	43.2	23.0	1	0	0	2	1,135.59
									TPKDESANQEEPEAR	95.0%	60.3	20.3	3	9	0	2	1,700.77
									VPAQSESVR	95.0%	37.2	21.5	2	0	0	2	972.51
Electron transfer flavoprotein subunit alpha, mitochondrial	ETFA_HUMAN	ETFA	35,062	99.90%	2	2	3	8.71%	GLLPEELTPLILATQK	95.0%	46.2	12.8	2	0	0	2	1,736.02
									LEVAPISDIIAIK	95.0%	34.0	12.3	1	0	0	2	1,381.83
UPF0587 protein C1orf123	CA123_HUMAN	C1orf123	18,031	99.50%	2	2	7	25.00%	ATLENITNLRPVGEDFR	95.0%	40.0	21.3	0	3	0	2	1,945.01
									ENSIEILSSTIKPYNAEDNENFK	95.0%	63.2	21.4	0	4	0	2	2,655.28
Podocalyxin-like protein 1	PODXL_HUMAN	PODXL	58,617	100.00%	2	2	12	4.66%	LASVPGSQTVVVK	95.0%	49.7	18.3	8	0	0	2	1,284.75
									LGDQGPPEEAEDR	95.0%	57.9	20.3	4	0	0	2	1,412.63
Biotinidase	BTD_HUMAN	BTD	61,115	100.00%	3	3	15	9.94%	LSSGLVTAALYGR	95.0%	46.8	19.7	4	0	0	2	1,307.73
									QEALELMNQNLDIYEQQVMTAAQK	95.0%	39.6	21.9	0	2	0	2	2,808.36
									TSIYPFLDFMPSPQVVR	95.0%	56.0	21.8	9	0	0	2	2,013.02
Epididymis-specific alpha-mannosidase	MA2B2_HUMAN	MAN2B2	113,961	100.00%	3	3	4	3.96%	IEQEYQAGPLELNR	95.0%	50.7	22.8	1	0	0	2	1,659.83
									LWWDGVASDQQK	95.0%	31.9	22.0	1	0	0	2	1,432.69
									QGPISDNYLFTPGK	95.0%	60.4	22.7	2	0	0	2	1,536.77
Membrane-bound transcription factor site-1 protease	MBTP1_HUMAN	MBTPS1	117,732	99.90%	2	2	6	2.38%	QRPPSGAGSVTPER	95.0%	39.3	22.4	0	4	0	2	1,438.74
									VAVFDTGLSEK	95.0%	31.6	22.4	2	0	0	2	1,165.61
Desmoglein-1	DSG1_HUMAN	DSG1	113,731	100.00%	6	6	52	8.67%	EMQDLGGGER	95.0%	56.9	17.2	10	0	0	2	1,107.47
									ISGVGIDQPPYGIFVINQK	95.0%	58.2	18.6	22	0	0	2	2,045.11
									QEPSDSPMFIINR	95.0%	59.3	22.7	7	0	0	2	1,549.73
									VGDFVATDLDTGRPSTTVR	95.0%	31.2	21.8	0	1	0	2	2,007.02
									YQGTILSIDDNLQR	95.0%	67.7	22.7	1	0	0	2	1,635.83
									YVMGNNPADLLAVDSR	95.0%	69.9	22.6	11	0	0	2	1,750.84
40S ribosomal protein S19	RS19_HUMAN	RPS19	16,043	99.50%	2	2	3	13.10%	IAGQVAAANK	95.0%	51.8	20.0	2	0	0	2	942.54
									VLQALEGLK	95.0%	35.2	16.2	1	0	0	2	970.59
Poliovirus receptor-related protein 2	PVRL2_HUMAN	PVRL2	57,724	100.00%	3	3	4	7.62%	MGPSFPSPKPGSER	95.0%	27.2	22.0	0	1	0	2	1,473.72
									VEHESFEEPALIPVTLSVR	95.0%	33.9	20.3	0	1	0	2	2,152.13
									VQVLPEVR	95.0%	33.9	16.6	2	0	0	2	939.56
Protein-glutamine gamma-glutamyltransferase 2	TGM2_HUMAN	TGM2	77,311	100.00%	10	11	49	18.00%	ALLVEPVINSYLLAER	95.0%	91.4	15.3	18	2	0	2	1,800.03
									CLGIPTR	95.0%	37.5	24.7	1	0	0	2	816.44
									DLYLENPEIK	95.0%	49.4	22.9	2	0	0	2	1,233.64
									LAEKEETGMAMR	95.0%	43.5	21.0	0	6	0	2	1,397.64
									LVVNFESDKLK	95.0%	36.6	19.6	2	0	0	2	1,291.73
									NEFGEIQGDK	95.0%	52.0	22.4	2	0	0	2	1,136.52
									TVEIPDPVEAGEEVK	95.0%	36.6	22.7	2	0	0	2	1,611.81
									VVTNYNSAHDQNSNLLIEYFR	95.0%	39.5	21.4	0	7	0	2	2,497.21
									YLLNLNLEPFSEK	95.0%	88.8	21.7	6	0	0	2	1,579.84
									YPEGSSEER	95.0%	58.2	17.7	1	0	0	2	1,053.45
Bleomycin hydrolase	BLMH_HUMAN	BLMH	52,545	100.00%	3	3	5	8.13%	IGPITPLEFYR	95.0%	38.0	20.4	2	0	0	2	1,305.72
									LYTVEYLSNMVGGR	95.0%	37.3	22.7	2	0	0	2	1,617.79
									TLYNNQPIDFLK	95.0%	36.1	22.1	1	0	0	2	1,465.77
Urokinase-type plasminogen	UROK_HUMAN	PLAU	48,490	100.00%	10	11	45	21.80%	DKPGVYTR	95.0%	31.4	23.4	1	0	0	2	935.50

activator									DYSADTLAHHNDIALLK	95.0%	32.3	22.6	0	2	0	2	1,896.95
									EDYIVYLGR	95.0%	48.3	21.1	2	0	0	2	1,127.57
									FEVENLILHK	95.0%	36.3	20.8	4	0	0	2	1,241.69
									KEDYIVYLGR	95.0%	54.6	22.0	5	0	0	2	1,255.67
									KPSSPPEELK	95.0%	55.3	21.8	10	3	0	2	1,111.60
									LNSNTQGEMK	95.0%	69.7	22.2	4	0	0	2	1,121.53
									MLCAADPQWK	95.0%	37.1	19.5	1	0	0	2	1,235.56
									SDALQLGLGK	95.0%	86.8	22.2	12	0	0	2	1,001.56
									VSHFLPWIR	95.0%	32.3	19.9	0	1	0	2	1,154.65
Latent-transforming growth factor	LTBP1_HUMAN	LTBP1	186,769	99.50%	2	2	4	1.57%	EEPVEALTFSR	95.0%	40.2	23.6	2	0	0	2	1,277.64
beta-binding protein 1									TSTDLDVDVDQPKEEK	95.0%	72.0	22.1	2	0	0	2	1,818.86
Heterogeneous nuclear	HNRPL_HUMAN	HNRNPL	64,115	100.00%	6	8	27	15.60%	ISRPGDSDDSR	95.0%	42.9	20.6	1	2	0	2	1,204.56
ribonucleoprotein L									NDQDTWDYTNPNLSGQGDPGSNPNKR	95.0%	79.9	17.6	0	2	0	2	2,890.26
									SDALETLGFLNHYQMK	95.0%	61.3	22.1	8	3	0	2	1,866.91
									SKPGAAMVEMADGYAVDR	95.0%	29.5	21.7	0	1	0	2	1,867.87
									SSSGLLEWESK	95.0%	62.5	23.0	8	0	0	2	1,222.60
									TPASPVVHIR	95.0%	41.0	20.1	0	2	0	2	1,076.62
Golgi membrane protein 1	GOLM1_HUMAN	GOLM1	45,315	100.00%	10	11	71	29.90%	DLSENNDQR	95.0%	40.0	18.3	4	0	0	2	1,090.48
									DQLVIPDGQEEEQEAAGEGR	95.0%	103.0	21.0	6	0	0	2	2,169.99
									DTINLLDQR	95.0%	59.8	23.4	10	0	0	2	1,087.58
									GNVLGNSK	94.7%	30.1	24.3	1	0	0	2	788.43
									IQSSHNFQLESVNK	95.0%	88.2	22.5	4	11	0	2	1,630.82
									LPQEPGR	95.0%	38.6	18.9	8	0	0	2	796.43
									LQQDVLQFQK	95.0%	42.6	22.1	6	0	0	2	1,246.68
									NIDVFNVEDQKR	95.0%	45.1	23.0	1	0	0	2	1,476.74
									QQLQALSEPQPR	95.0%	58.9	22.4	14	0	0	2	1,394.74
									QVEKEETNEIQVVNEEPQR	95.0%	40.8	21.9	0	6	0	2	2,298.12
Septin-2	SEPT2_HUMAN	SEPT2	41,470	100.00%	6	6	25	19.70%	ASIPFSVVGSNQLIEAK	95.0%	74.9	19.9	8	0	0	2	1,759.96
									HIIDNR	95.0%	30.8	20.6	1	0	0	2	767.42
									ILDEIEEHNIK	95.0%	45.5	22.2	3	0	0	2	1,352.71
									STLINSLFLTDLYPER	95.0%	51.9	20.5	4	0	0	2	1,882.00
									TIISYIDEQFER	95.0%	66.1	22.5	6	0	0	2	1,513.75
									VNIVPVIAK	95.0%	36.9	10.4	3	0	0	2	952.62
Peptidyl-prolyl cis-trans isomerase	FKB1A_HUMAN	FKBP1A	11,933	99.50%	2	2	7	25.00%	GVQVETISPGDGR	95.0%	61.7	21.8	2	0	0	1	1,314.67
FKBP1A									GWEEGVAQMSVGQR	95.0%	107.0	21.5	5	0	0	2	1,533.71
Protein disulfide-isomerase A3	PDIA3_HUMAN	PDIA3	56,767	100.00%	32	40	439	59.60%	DASIVGFFDDSFSEAHSEFLK	95.0%	36.7	20.5	1	0	0	2	2,348.07
									DGEEAGAYDGPR	95.0%	66.1	14.9	17	0	0	2	1,236.51
									DLLIAYYDVDYEK	95.0%	88.1	22.2	53	0	0	2	1,619.78
									DPNIVIAK	95.0%	45.8	16.4	4	0	0	2	869.51
									EATNPPVIQEEKPK	95.0%	80.2	21.6	17	0	0	2	1,579.83
									EATNPPVIQEEKPKK	95.0%	42.3	20.3	1	0	0	2	1,707.93
									ELSDFISYLQR	95.0%	64.7	22.5	33	0	0	2	1,370.70
									FEDKTVAYTEQK	95.0%	45.8	22.3	0	1	0	2	1,458.71
									FIQENIFGICPHMTEDNKDLIQGK	95.0%	43.0	21.6	0	2	1	2	2,863.38
									FISDKDASIVGFFDDSFSEAHSEFLK	95.0%	28.6	20.8	0	1	0	2	2,938.38
									FLDAGHK	95.0%	31.2	21.6	1	0	0	2	787.41
									FLQDYFDGNLK	95.0%	71.8	22.1	9	0	0	2	1,359.66
									FLQDYFDGNLKR	95.0%	58.2	22.5	12	6	0	2	1,515.76
									FVMQEEFSR	95.0%	53.2	18.5	38	0	0	2	1,188.54
									GFPTIYFSPANK	95.0%	61.3	22.5	18	0	0	2	1,341.68
									GFPTIYFSPANKK	95.0%	65.6	21.2	4	0	0	2	1,469.78

									IFRDGEEAGAYDGPR	95.0%	80.0	22.5	5	12	0	2	1,652.77
									KAQEDL	94.8%	30.2	22.9	1	0	0	2	703.36
									KTFSHELSDFGLESTAGEIPVVAIR	95.0%	54.3	20.3	0	10	12	2	2,703.40
									LAPEYEAAATR	95.0%	97.0	23.5	24	0	0	2	1,191.60
									LNFAVASR	95.0%	57.6	22.6	34	0	0	2	877.49
									LSKDPNIVIAK	95.0%	68.8	16.6	5	18	0	2	1,197.72
									MDATANDVPSPYEVR	95.0%	87.5	20.8	24	0	0	2	1,680.75
									QAGPASVPLR	95.0%	50.8	18.9	4	0	0	2	995.56
									RLAPEYEAAATR	95.0%	51.7	23.2	2	1	0	2	1,347.70
									SEPIPESNDGPVK	95.0%	57.8	21.9	10	0	0	2	1,368.66
									TADGIVSHLKK	95.0%	44.4	18.8	2	1	0	2	1,168.67
									TEEEFKK	95.0%	30.7	21.9	1	0	0	2	910.45
									TFSHELSDFGLESTAGEIPVVAIR	95.0%	99.2	21.5	2	7	0	2	2,575.30
									TVAYTEQK	95.0%	50.9	21.9	5	0	0	2	939.48
									VVVAENFDEIVNNENK	95.0%	129.0	22.6	7	0	0	2	1,832.90
									YGVSGYPTLK	95.0%	56.8	21.9	33	0	0	2	1,084.57
Periplakin	PEPL_HUMAN	PPL	204,660	100.00%	24	24	123	15.20%	AGLIDWNMFVK	95.0%	46.3	23.4	2	0	0	2	1,293.67
									AITGILRPPLEQGR	95.0%	41.9	15.1	2	0	0	2	1,520.89
									ALEQETR	95.0%	41.8	22.4	3	0	0	2	846.43
									ASQEEQIAR	95.0%	46.8	22.2	4	0	0	2	1,031.51
									AVQDSAER	95.0%	42.8	22.0	1	0	0	2	875.42
									EAEVLLLQQR	95.0%	63.7	21.1	6	0	0	2	1,198.68
									ELDEETER	95.0%	30.6	18.3	1	0	0	2	1,020.45
									ELSELIEQLQK	95.0%	68.0	21.7	9	0	0	2	1,329.73
									EVDPQVNWAALVEEK	95.0%	83.2	22.4	4	0	0	2	1,726.87
									FTEVYAINR	95.0%	49.2	21.9	8	0	0	2	1,112.57
									GAEEQLR	95.0%	46.5	22.0	2	0	0	2	802.41
									KVPDPVLEESFQQLQR	95.0%	30.5	20.5	0	2	0	2	1,913.01
									LAALEQEEAEAR	95.0%	73.8	22.9	1	0	0	2	1,329.67
									LELVEQER	95.0%	32.0	22.4	1	0	0	2	1,015.54
									LLAASQAR	95.0%	41.2	22.2	3	0	0	2	829.49
									LQNLEFALNLLR	95.0%	78.8	17.6	2	0	0	2	1,443.83
									NLLDEIASR	95.0%	43.9	23.6	9	0	0	2	1,030.55
									SLLGEVEQNLQAAK	95.0%	85.9	21.5	19	0	0	2	1,499.81
									SSLEEESR	95.0%	52.6	20.6	2	0	0	2	936.43
									STAEGEAFIQALPGSGTTPLLR	95.0%	69.8	20.4	10	0	0	2	2,216.16
									TENPGDASDLQGR	95.0%	64.2	19.6	21	0	0	2	1,359.61
									VKEEEAALAAK	95.0%	27.8	23.5	0	1	0	2	1,158.64
									VLDKYEDVVQGLQK	95.0%	83.5	21.4	2	0	0	2	1,633.88
									VVLQQDPQQAR	95.0%	52.6	21.2	8	0	0	2	1,281.69
14-3-3 protein gamma	1433G_HUMAN	YWHAG	28,285	100.00%	11	14	101	59.90%	ATVVESSEK	95.0%	58.0	23.5	5	0	0	2	949.48
									AYSEAHEISK	95.0%	55.2	22.8	6	0	0	2	1,134.54
									DNLTLWTSDQQDDDGGEGNN	95.0%	80.8	12.8	4	0	0	2	2,193.88
									DSTLIMQLLR	95.0%	79.3	21.3	113	0	0	2	1,189.66
									LAEQAER	95.0%	59.4	21.6	36	0	0	2	816.42
									LAEQAERYDDMAAAMK	95.0%	52.0	20.8	4	1	0	2	1,812.83
									MKGDYYR	95.0%	42.7	19.2	6	0	0	2	932.43
									NCSETQYESK	95.0%	42.2	14.3	2	0	0	2	1,245.51
									NLLSVAYK	95.0%	51.4	19.1	49	0	0	2	907.53
									NLLSVAYKNVVGAR	95.0%	79.1	17.7	4	2	0	2	1,503.86
									NVTELNEPLSNEER	95.0%	94.6	21.6	23	0	0	2	1,643.79

									RATVVESSEK	95.0%	40.3	23.5	2	0	0	2	1,105.59
									TAFDDAIAELDTLNEDSYK	95.0%	121.0	20.4	12	2	0	2	2,130.97
									VISSIEQK	95.0%	65.9	22.9	27	0	0	2	903.52
									YDDMAAAMK	95.0%	70.3	14.5	11	0	0	2	1,015.42
									YLAEVATGEK	95.0%	64.8	22.1	19	0	0	2	1,080.56
									YLAEVATGEKR	95.0%	64.8	22.3	8	2	0	2	1,236.66
Deoxyuridine 5'-triphosphate nucleotidohydrolase, mitochondrial	DUT_HUMAN	DUT	26,689	100.00%	4	5	13	20.20%	ARPAEVGGMQLR	95.0%	37.6	22.2	0	1	0	2	1,300.68
									GNVGVVLFNFGK	95.0%	79.5	20.9	2	0	0	2	1,250.69
									IFYPEIEEVQALDDTER	95.0%	106.0	21.6	2	0	0	2	2,066.99
									LSEHATAPTR	95.0%	47.8	21.8	3	5	0	2	1,082.56
Regulation of nuclear pre-mRNA domain-containing protein 1B	RPR1B_HUMAN	RPRD1B	36,883	100.00%	5	6	14	22.70%	ALQDLENAASGDATVR	95.0%	89.5	22.3	2	0	0	2	1,630.80
									EFESVLVDAFSHVAR	95.0%	54.2	22.9	1	4	0	2	1,705.85
									IASLPQEVQDVSLLEK	95.0%	88.5	19.5	2	0	0	2	1,768.97
									LTFLYLANDVIQNSK	95.0%	73.0	20.1	2	0	0	2	1,738.94
									SVYGGEFIQQLK	95.0%	69.4	21.1	3	0	0	2	1,368.72
Far upstream element-binding protein 2	FUBP2_HUMAN	KHSRP	73,129	100.00%	4	4	11	8.87%	IGGGIDVPVPR	95.0%	60.3	18.6	3	0	0	2	1,079.62
									IGQQPQQPGAPPQQDYTK	95.0%	66.6	22.3	2	0	0	2	1,980.98
									IQNDAGVR	95.0%	35.0	21.3	2	0	0	2	872.46
									SVSLTGAPESVQK	95.0%	63.8	23.2	4	0	0	2	1,302.69
									VQISPDSGGLPER	95.0%	49.6	22.1	2	0	0	2	1,354.70
Sialate O-acetylesterase	SIAE_HUMAN	SIAE	58,297	100.00%	4	4	11	10.30%	ELSNTAAYQSVR	95.0%	61.6	22.2	2	0	0	2	1,338.67
									FASYINNDMVLQK	95.0%	34.5	22.2	1	0	0	2	1,558.76
									FFPFGLVQLSSDLSK	95.0%	98.2	21.7	6	0	0	2	1,684.90
									QGSIPYDSVTGPSK	95.0%	54.1	22.8	2	0	0	2	1,435.71
Myristoylated alanine-rich C-kinase substrate	MARCS_HUMAN	MARCKS	31,536	100.00%	6	7	32	25.30%	AAEEPSKVEEK	95.0%	36.3	22.5	0	2	0	2	1,216.61
									AEDGATPSPSNETPK	95.0%	55.1	20.8	11	0	0	2	1,500.68
									EAGEGGEAEAPAAEGGK	95.0%	76.0	19.3	2	0	0	2	1,529.67
									GEAAAERPGEAAVASSPSK	95.0%	90.9	22.1	4	6	0	2	1,784.88
									GEPAAAAAPEAGASPVEK	95.0%	93.4	22.9	6	0	0	2	1,622.80
									TAAKGEAAAERPGEAAVASSPSK	95.0%	39.2	21.7	0	1	0	2	2,156.09
Histone H4	H4_HUMAN	HIST1H4A	11,350	100.00%	6	8	42	51.50%	DAVTYTEHAK	95.0%	35.1	22.8	3	0	0	2	1,134.54
									DNIQGITKPAIR	95.0%	50.4	18.8	2	2	0	2	1,325.75
									ISGLIYEETR	95.0%	41.5	22.5	6	0	0	2	1,180.62
									TVTAMDVVYALK	95.0%	56.5	20.8	4	0	0	2	1,310.70
									TVTAMDVVYALKR	95.0%	79.4	20.0	2	2	0	2	1,466.80
									VFLENVIR	95.0%	52.8	20.3	21	0	0	2	989.58
Spectrin beta chain, brain 1	SPTB2_HUMAN	SPTBN1	274,595	100.00%	28	28	80	16.00%	AFEDEMSGR	95.0%	40.9	14.0	2	0	0	2	1,057.43
									ALVADSHPESER	95.0%	42.9	22.4	2	0	0	2	1,310.63
									DASVAEAWLLGQEPYLSSR	95.0%	72.2	21.8	1	0	0	2	2,092.04
									DTGNIGQER	95.0%	52.8	20.2	2	0	0	2	989.47
									EIEELQSQAQALSQEGK	95.0%	98.2	21.9	2	0	0	2	1,887.93
									EQWANLEQLSAIR	95.0%	57.8	22.5	2	0	0	2	1,557.80
									EVDDLEQWIAER	95.0%	64.0	20.8	5	0	0	2	1,502.71
									FESLEPEMNNQASR	95.0%	47.0	18.3	4	0	0	2	1,667.73
									FMELLEPLNER	95.0%	51.5	22.5	2	0	0	2	1,406.70
									HQILEQAVEDYAETVHQLSK	95.0%	49.1	22.2	0	2	0	2	2,338.17
									ITDLYTDLR	95.0%	31.9	22.3	1	0	0	2	1,109.58
									IVSSSDVGHDEYSTQSLVK	95.0%	41.3	22.8	0	4	0	2	2,050.99
									LAEISDVWEEMK	95.0%	36.0	21.9	1	0	0	2	1,465.69
									LLEVLSGER	95.0%	48.8	21.5	4	0	0	2	1,015.58
									LQALDTGWNELHK	95.0%	55.7	22.6	2	0	0	2	1,524.78

									LQQFLR	95.0%	31.1	21.2	5	0	0	2	804.47
									LVSDGNINSDR	95.0%	74.3	23.2	3	0	0	2	1,189.58
									LVSQDNFGFDLPAVEAATK	95.0%	92.1	22.1	4	0	0	2	2,022.02
									MLTAQDMSYDEAR	95.0%	31.2	14.3	2	0	0	2	1,562.65
									QALQDTLALYK	95.0%	50.6	21.4	2	0	0	2	1,263.70
									SQNIVTDSSSLSAEAIR	95.0%	118.0	22.8	4	0	0	2	1,777.89
									TALPAQSAATLPAR	95.0%	57.0	18.8	5	0	0	2	1,367.76
									TQETPSAQMEGFLNR	95.0%	56.3	22.0	1	0	0	2	1,708.80
									TQTAIASEDMPNTLTEAEK	95.0%	129.0	21.4	6	0	0	2	2,065.96
									VAVVNQIAR	95.0%	53.0	16.0	3	0	0	2	969.58
									VDSIDDR	95.0%	43.6	22.5	2	0	0	2	819.39
									VIESTQDLGNDLAGVMALQR	95.0%	104.0	21.9	2	0	0	2	2,146.08
									VLDNAIETEK	95.0%	50.6	24.2	6	0	0	2	1,131.59
									VLVLSQDYGK	95.0%	43.0	22.0	4	0	0	2	1,121.62
Serine-threonine kinase receptor-associated protein	STRAP_HUMAN	STRAP	38,421	100.00%	5	5	8	21.70%	FSPDGELYASGSEDGTLR	95.0%	66.2	18.7	1	0	0	2	1,900.86
									IYDLNKPEAEPK	94.6%	30.0	21.7	1	0	0	2	1,416.74
									QGDTGDWIGTFLGHK	95.0%	49.8	22.6	0	2	0	2	1,631.78
									TVDFTQDSNYLLTGGQDK	95.0%	107.0	21.7	2	0	0	2	2,001.94
									YDYNSGEELESYK	95.0%	70.8	15.1	2	0	0	2	1,596.67
Annexin A8	ANXA8_HUMAN	ANXA8	36,865	100.00%	18	22	225	66.40%	AYEEDYGSSLEEDIQADTSGYLER	95.0%	33.7	16.5	1	0	0	2	2,740.18
									CTQNLHSYFAER	95.0%	48.5	20.8	2	2	0	2	1,525.69
									DDVSSFVDPGLALQDAQDLYAAGEK	95.0%	92.5	20.9	6	4	0	2	2,624.24
									EGVIIEILASR	95.0%	87.1	20.8	59	0	0	2	1,199.70
									FITILCTR	95.0%	66.6	20.8	11	0	0	2	1,023.57
									GIGTNEQAIIDVLTK	95.0%	105.0	20.6	24	0	0	2	1,571.86
									GIGTNEQAIIDVLTKR	95.0%	104.0	18.3	4	0	0	2	1,727.97
									ILVCLLQGSR	95.0%	77.7	21.2	6	0	0	2	1,158.67
									LIVALMYPPYR	95.0%	64.5	20.6	20	0	0	2	1,335.75
									NALLSLVGSDP	95.0%	35.3	22.9	1	0	0	2	1,085.58
									SATHLLR	95.0%	32.1	18.7	1	0	0	2	797.46
									SEIDLNLIK	95.0%	58.1	21.7	7	0	0	2	1,044.59
									SELSGKFER	95.0%	34.5	21.8	2	0	0	2	1,052.54
									SETHGSLEEAMLTVVK	95.0%	73.3	21.7	13	31	0	2	1,730.86
									SSSHFNPDPDAETLYK	95.0%	68.7	21.8	5	3	0	2	1,807.81
									SWIEQEGVTVK	95.0%	55.7	22.5	6	0	0	2	1,275.66
									TKNQLR	95.0%	31.5	22.7	1	0	0	2	759.45
									TLSSMIMEDTSGDYK	95.0%	106.0	19.3	16	0	0	2	1,693.73
Protein transport protein Sec23A	SC23A_HUMAN	SEC23A	86,145	100.00%	3	3	12	5.62%	GPQVQQPPPSNR	95.0%	62.3	23.7	8	0	0	2	1,304.67
									HLLQAPVDDAQEILHSR	95.0%	60.0	21.5	0	2	0	2	1,942.01
									IDMNLTDLLGELQR	95.0%	61.3	22.3	2	0	0	2	1,630.85
Destrin	DEST_HUMAN	DSTN	18,488	99.50%	2	2	4	18.80%	HECQANGPEDLNR	95.0%	54.6	17.7	2	0	0	2	1,539.66
									MIYASSK	95.0%	37.6	20.3	1	0	0	2	815.40
									YALYDASFETK	95.0%	59.8	22.6	2	0	0	2	1,307.62
Transmembrane protein 132A	T132A_HUMAN	TMEM132A	110,089	100.00%	7	7	20	9.48%	AEELVNTAPLTGVPQHVPVR	95.0%	37.1	18.0	0	4	0	2	2,127.16
									EPGVTSIEVR	95.0%	45.4	22.7	4	0	0	2	1,086.58
									IELTDTTLEQVR	95.0%	55.1	22.8	4	0	0	2	1,417.75
									QVAGSVGGNTGVR	95.0%	41.4	22.4	2	0	0	2	1,201.63
									SETFLLLQPWPR	95.0%	45.4	21.5	2	0	0	2	1,486.81
									VASLEGGR	95.0%	41.4	24.3	2	0	0	2	788.43
									VPGPAEGPAEPAAEASDEAERR	95.0%	37.9	21.6	0	2	0	2	2,206.04
Interstitial collagenase	MMP1_HUMAN	MMP1	53,990	100.00%	13	13	96	27.10%	ADVDHAIEK	95.0%	31.3	21.0	1	0	0	2	997.50

									AFQLWSNVTPLTFTK	95.0%	78.7	20.1	9	0	0	2	1,752.93
									DIYSSFGFPR	95.0%	67.5	21.3	18	0	0	2	1,188.57
									EYNLHR	95.0%	33.6	21.0	2	0	0	2	831.41
									HIDAALSEENTGK	95.0%	82.8	22.5	15	0	0	2	1,384.67
									IENYTPDLPR	95.0%	50.8	23.0	7	0	0	2	1,217.62
									NSGPVVEK	95.0%	36.8	21.0	1	0	0	2	829.44
									QMQEFFGLK	95.0%	33.9	21.0	4	0	0	2	1,127.56
									SMDPGYPK	95.0%	34.2	18.5	1	0	0	2	910.40
									SQNPVQPIGPQTPK	95.0%	78.8	21.8	26	0	0	2	1,490.80
									TYFFVANK	95.0%	38.3	23.1	5	0	0	2	989.51
									VTGKPDAETLK	95.0%	42.5	23.4	5	0	0	2	1,158.64
									YDEYKR	95.0%	34.7	19.6	2	0	0	2	873.41
Myosin regulatory light chain 12A	ML12A_HUMAN	MYL12A	19,777	100.00%	5	6	12	39.80%	ATSNVFAMFDQSQIQEFK	95.0%	101.0	21.5	4	0	0	2	2,090.99
									DGFIDKEDLHDMLASLGK	95.0%	70.1	22.1	2	0	1	2	2,003.98
									FTDEEVDELYR	95.0%	72.9	19.4	2	0	0	2	1,415.63
									GNFNYIEFTR	95.0%	67.0	21.7	2	0	0	2	1,260.60
									LNGTDPEDVIR	95.0%	60.9	22.8	1	0	0	2	1,228.62
ATP-dependent DNA helicase Q1	RECQ1_HUMAN	RECQL	73,441	100.00%	2	2	4	3.85%	ISSMVVMENVGQQK	95.0%	44.7	22.9	1	0	0	2	1,581.76
									VAGVVAPTLPR	95.0%	52.0	14.8	3	0	0	2	1,079.66
ADP-ribosylation factor 1	ARF1_HUMAN	ARF1	20,680	100.00%	3	3	6	25.40%	ILMVGLDAAGK	95.0%	44.1	23.1	2	0	0	2	1,087.62
									LGEIVTTIPTIGFNVETVEYK	95.0%	57.3	18.5	2	0	0	2	2,323.24
									NISFTVWDVGGQDK	95.0%	77.2	23.3	2	0	0	2	1,565.76
Beta-mannosidase	MANBA_HUMAN	MANBA	100,879	100.00%	13	13	46	18.30%	FASEYGYQSWPSFSTLEK	95.0%	94.2	21.2	3	0	0	2	2,126.97
									FQSAVLYAAQQSK	95.0%	109.0	22.1	6	0	0	2	1,440.75
									FSDNGFLMTEK	95.0%	69.9	19.5	3	0	0	2	1,288.59
									GSNWIPADSFQDR	95.0%	34.3	21.0	1	0	0	2	1,492.68
									GSPGLSFYFK	95.0%	37.8	23.1	3	0	0	2	1,102.56
									ILFNEVTIGETDNMFNR	95.0%	107.0	22.1	3	0	0	2	2,028.97
									LLLQSVVDANMNTLR	95.0%	79.6	21.0	7	0	0	2	1,702.92
									TILFYPWEPTSK	95.0%	34.2	23.1	1	0	0	2	1,481.77
									TVELIEEPIK	95.0%	55.9	20.8	5	0	0	2	1,170.66
									VNLILEGVDTVSK	95.0%	99.4	19.5	5	0	0	2	1,386.78
									VSSTEDWSFNSK	95.0%	72.5	20.1	4	0	0	2	1,386.62
									VTSELLR	95.0%	33.9	22.3	1	0	0	2	817.48
									YSFDITNVVR	95.0%	72.1	21.4	4	0	0	2	1,213.62
Low-density lipoprotein receptor	LDLR_HUMAN	LDLR	95,357	100.00%	4	4	11	5.35%	AHGVSSYDTVISR	95.0%	45.0	23.3	1	0	0	2	1,391.69
									IYWSDLSQR	94.9%	30.3	22.4	1	0	0	2	1,167.58
									NVVALDTEVASNR	95.0%	89.7	22.1	3	0	0	2	1,387.72
									SEYTSLIPNLR	95.0%	51.8	23.0	6	0	0	2	1,292.69
Plasminogen activator inhibitor 1 RNA-binding protein	PAIRB_HUMAN	SERBP1	44,948	100.00%	4	4	30	13.70%	EAGGGGVGGPGAK	95.0%	78.6	21.0	4	0	0	2	1,013.50
									FDQLFDDESDPFEVLK	95.0%	97.2	20.3	8	0	0	2	1,943.89
									RPDQQLQGEGK	95.0%	55.5	22.1	8	0	0	2	1,255.64
									SAAQAAAQTNSNAAGK	95.0%	91.7	22.9	10	0	0	2	1,460.71
Cold-inducible RNA-binding protein	CIRBP_HUMAN	CIRBP	18,630	99.50%	2	2	4	20.30%	GFGFVTFENIDDAK	95.0%	55.2	21.5	2	0	0	2	1,559.74
									LFVGGLSFDTNEQSLEQVFSK	95.0%	123.0	22.0	2	0	0	2	2,345.17
Protein S100-A9	S10A9_HUMAN	S100A9	13,224	99.90%	2	2	2	18.40%	MSQLER	95.0%	35.5	24.1	1	0	0	2	763.38
									NIETIINTFHQYSVK	95.0%	47.7	21.5	1	0	0	2	1,806.94
Syndecan-4	SDC4_HUMAN	SDC4	21,624	100.00%	6	8	435	25.30%	AGSGSQVPTEPK	95.0%	77.2	22.3	99	0	0	2	1,157.58
									AGSGSQVPTEPKK	95.0%	39.5	22.0	3	0	0	2	1,285.68
									ETEVIDPQDLLEGR	95.0%	89.2	22.5	134	0	0	2	1,613.80
									ISPVEESEDVSNK	95.0%	101.0	22.0	33	0	0	2	1,432.68

									KLEENEVIPK	95.0%	57.7	21.6	78	29	0	2	1,198.67
									RISPVEESEDVSNK	95.0%	78.5	23.1	33	26	0	2	1,588.78
Protein transport protein Sec24C	SC24C_HUMAN	SEC24C	118,307	100.00%	2	2	2	3.02%	AVITSLLDQIPEMFADTR	95.0%	55.9	21.6	1	0	0	2	2,036.04
									TLFQPQTGAYQTLAK	95.0%	35.7	20.7	1	0	0	2	1,666.88
Transketolase	TKT_HUMAN	TKT	67,861	100.00%	29	39	526	50.70%	AFDQIR	95.0%	35.9	25.6	8	0	0	2	749.39
									AVELAANTK	95.0%	55.9	23.0	4	0	0	2	916.51
									AYGQALAK	95.0%	42.9	22.7	3	0	0	2	821.45
									DAIAQAVR	95.0%	37.0	22.7	1	0	0	2	843.47
									HQPTAIIAK	95.0%	37.1	17.4	3	0	0	2	978.57
									IIALDGDTK	95.0%	49.7	23.1	10	0	0	2	945.53
									ILATPPQEDAPSVDIANIR	95.0%	99.8	20.1	68	30	0	2	2,020.07
									ILTVEDHYYEGGIGEAVSSAVVGEPGITVTHLAVNR	95.0%	86.0	18.9	0	2	28	2	3,752.91
									ISSDLDGHPVPK	95.0%	55.6	22.7	9	11	0	2	1,264.65
									KAYGQALAK	95.0%	51.4	20.5	3	0	0	2	949.55
									KILATPPQEDAPSVDIANIR	95.0%	54.1	17.8	0	2	0	2	2,148.17
									KISSDLDGHPVPK	95.0%	83.2	20.7	3	4	0	2	1,392.75
									KLILDSAR	95.0%	53.6	19.8	7	0	0	2	915.56
									LDNLVAILDINR	95.0%	86.8	17.6	21	0	0	2	1,368.79
									LGHASDR	95.0%	31.3	21.3	1	0	0	2	755.38
									LGQSDPAPLQHQMDIYQK	95.0%	76.0	22.1	8	11	0	2	2,069.01
									LQALKDTANR	95.0%	38.6	21.6	1	0	0	2	1,129.63
									MFGIDRDAIAQAVR	95.0%	57.1	22.8	2	11	0	2	1,562.81
									MPSLPSYK	95.0%	43.0	21.8	5	0	0	2	938.47
									NMAEQIIQEIYSQIQSK	95.0%	113.0	21.7	28	57	0	2	2,039.01
									NSTFSEIFK	95.0%	38.8	20.6	2	0	0	2	1,072.53
									NSTFSEIFKK	95.0%	44.5	22.6	7	0	0	2	1,200.63
									QAFTDVATGSLGQGLGAACGMAYTGK	95.0%	91.2	21.0	4	2	0	2	2,532.19
									SGKPAELLK	95.0%	46.1	19.6	1	0	0	2	942.56
									SVPTSTVFYPSDGVATEK	95.0%	94.8	22.3	60	0	0	2	1,884.92
									TSRPENAIIYNNNEDFQVGQAK	95.0%	84.7	21.6	6	71	0	2	2,508.21
									TVPFCSTFAAFFTR	95.0%	64.8	22.1	6	0	0	2	1,651.79
									VLDPFTIKPLDR	95.0%	62.8	19.2	20	2	0	2	1,413.81
									VLDPFTIKPLDRK	95.0%	27.9	14.9	0	0	4	2	1,541.91
Splicing factor, arginine/serine-rich 9	SFRS9_HUMAN	SFRS9	25,525	100.00%	4	5	9	21.30%	DGVGMVEYLR	95.0%	57.9	22.6	1	0	0	2	1,138.56
									EKDLEDLFYK	95.0%	60.4	21.9	3	1	0	2	1,299.65
									IYVGNLPTDVR	95.0%	50.9	22.0	2	0	0	2	1,246.68
									VLVSGLPPSGSWQDLK	95.0%	50.8	20.0	2	0	0	2	1,682.91
Cathepsin S	CATS_HUMAN	CTSS	37,478	99.50%	2	2	4	6.04%	GIDSDASYPYK	95.0%	45.5	20.9	2	0	0	2	1,215.55
									ILPDSVDWR	95.0%	30.5	23.5	2	0	0	2	1,100.57
Integrin alpha-6	ITA6_HUMAN	ITGA6	126,618	100.00%	8	8	36	9.91%	LETTSNQDNLAPITAK	95.0%	82.8	21.9	3	0	0	2	1,715.88
									LIATFPDTLTYSAYR	95.0%	73.0	21.5	8	0	0	2	1,731.90
									LLLVGAPR	95.0%	34.5	12.6	1	0	0	2	838.55
									LNYLDILMR	95.0%	73.7	21.7	9	0	0	2	1,150.63
									NIGDINQDGYPDIAVGAPYDDLGK	95.0%	90.2	21.5	2	0	0	2	2,520.19
									SEDEVGSLIEYEFR	95.0%	66.7	21.0	2	0	0	2	1,672.77
									SRPVINIQK	95.0%	34.9	16.1	3	0	0	2	1,054.64
									VNSLPEVLPILNSDEPK	95.0%	56.5	19.6	8	0	0	2	1,864.01
Prostaglandin F2 receptor negative regulator	FPRP_HUMAN	PTGFRN	98,538	100.00%	15	15	47	17.50%	ALSADQGSYR	95.0%	62.2	22.1	2	0	0	2	1,067.51
									AQDGDFIFSK	95.0%	40.9	21.4	4	0	0	2	1,127.54
									EGEPFELR	95.0%	36.5	22.3	1	0	0	2	976.47
									EHTDTFNFR	95.0%	35.3	20.3	1	0	0	2	1,166.52

									GEILLR	95.0%	32.3	22.0	1	0	0	2	700.44
									LDTVGSDAYR	95.0%	62.8	21.7	8	0	0	2	1,096.53
									MDVLNAFK	95.0%	50.7	22.8	2	0	0	2	937.48
									MPDSTLPGSR	95.0%	56.4	21.8	9	0	0	2	1,060.51
									MYQTQVSDAGLYR	95.0%	102.0	20.6	5	0	0	2	1,531.72
									NVQPSDQGHYK	95.0%	32.8	20.9	1	0	0	2	1,272.60
									SDLSLER	95.0%	46.8	24.7	4	0	0	2	819.42
									TANDAVELHIK	95.0%	33.1	22.4	0	2	0	2	1,210.64
									VLADSLHVGPSARPPPSLSLR	95.0%	51.5	15.4	0	2	0	2	2,169.21
									VPTATLVR	95.0%	36.9	18.9	3	0	0	2	856.53
									YIISLDQDSVVK	95.0%	44.7	22.1	2	0	0	2	1,379.74
Eukaryotic translation initiation factor 6	IF6_HUMAN	EIF6	26,580	100.00%	6	8	31	37.10%	ASFENNCEIGCFAK	95.0%	52.3	15.8	2	0	0	2	1,646.69
									ETEEILADVLK	95.0%	59.7	22.1	2	0	0	2	1,259.67
									HGLLVPNNTTDQELQHIR	95.0%	67.8	20.6	0	3	2	2	2,085.08
									LNEAQPSTIATSMR	95.0%	72.1	23.1	6	0	0	2	1,534.75
									NSLPDTVQIR	95.0%	35.5	22.4	1	0	0	2	1,142.62
									TSIEDQDELSSLLQVPLVAGTVNR	95.0%	73.3	19.0	8	7	0	2	2,584.35
Cytosolic non-specific dipeptidase	CNDP2_HUMAN	CNDP2	52,862	100.00%	12	14	40	38.10%	EGGSIPVTLTFQEATGK	95.0%	83.1	22.7	4	0	0	2	1,734.89
									GNILIPGINEAVAAVTEEEHK	95.0%	45.9	20.9	2	0	0	2	2,204.16
									GSTDDKGPVAGWINALEAYQK	95.0%	29.3	22.4	0	1	0	2	2,220.09
									LPDGSEIPLPPILLGR	95.0%	56.4	13.4	4	0	0	2	1,686.98
									LVPNMTPEVVGEQVTSYLTK	95.0%	81.5	21.5	4	0	0	2	2,221.14
									NVMLLPVGSADDGAHSQNEK	95.0%	90.0	21.8	3	3	0	2	2,097.99
									QKLPDGSEIPLPPILLGR	95.0%	47.3	13.0	3	5	0	2	1,943.13
									QLGGSVELVDIGK	95.0%	87.9	22.4	3	0	0	2	1,314.73
									TVFGVEPDLTR	95.0%	48.0	22.9	4	0	0	2	1,233.65
									WVAIQSVSAWPEK	95.0%	77.1	22.7	2	0	0	2	1,500.79
									YIDENQDR	95.0%	42.8	20.1	1	0	0	2	1,052.47
									YPSLSLHGIEGAFSGSGAK	95.0%	36.4	21.7	0	1	0	2	1,877.94
Aspartate aminotransferase, cytoplasmic	AATC_HUMAN	GOT1	46,230	100.00%	14	17	85	42.90%	APPSVFAEVPQAQPVLVFK	95.0%	87.1	17.8	10	3	0	1	2,024.12
									EPESILQVLSQMEK	95.0%	73.5	22.2	14	0	0	2	1,630.84
									HIYLLPSGR	95.0%	33.6	18.8	2	0	0	2	1,055.60
									IANDNSLNHEYLPILGLAEFR	95.0%	29.8	20.8	0	5	0	2	2,399.24
									IGADFLAR	95.0%	65.7	24.4	9	0	0	2	862.48
									INVSGLTTK	95.0%	35.8	20.4	3	0	0	2	932.54
									ITWSNPPAQGAR	95.0%	68.0	22.3	10	0	0	2	1,297.67
									IVASTLSNPELFEEWTGNVK	95.0%	102.0	21.2	9	1	0	2	2,234.13
									KVNLGVGAYR	95.0%	33.9	17.9	0	1	0	2	1,076.62
									LALGDDSPALK	95.0%	47.6	21.9	4	0	0	2	1,099.60
									NFGLYNER	95.0%	30.6	21.4	1	0	0	2	1,012.49
									NLDYVATSIHEAVTK	95.0%	83.2	21.4	4	2	0	2	1,660.85
									QIASVMK	95.0%	32.1	22.6	1	0	0	2	792.43
									VGGVQSLGGTGALR	95.0%	76.6	22.2	6	0	0	2	1,271.71
Ubiquitin-like modifier-activating enzyme 1	UBA1_HUMAN	UBA1	117,832	100.00%	20	24	171	26.50%	AAVATFLQSVQVPEFTPK	95.0%	73.1	19.3	20	2	0	2	1,933.04
									AENYDIPSADR	95.0%	44.4	21.2	3	0	0	2	1,250.57
									AEVSQPR	95.0%	31.7	20.5	2	0	0	2	786.41
									ALPAVQQNNLDEDLIR	95.0%	95.9	22.0	6	0	0	2	1,808.95
									ALPAVQQNNLDEDLIRK	95.0%	50.8	19.2	1	0	0	2	1,937.05
									ATLPSPDKLPGFK	95.0%	31.8	20.1	1	0	0	2	1,370.77
									GNVQVVIPFLTESYSSSQDPPEK	95.0%	75.7	21.8	4	0	0	2	2,521.25
									IYDDDFFQNLDGVANALDNVDAR	95.0%	53.6	19.7	4	0	0	2	2,600.19

									LAGTQPLEVLEAVQR	95.0%	92.1	17.9	23	9	0	2	1,623.91
									LAYVAAGDLAPINAFIGGLAAQEVMK	95.0%	42.4	18.7	0	4	0	2	2,619.39
									LDQPMTEIVSR	95.0%	61.0	23.5	12	0	0	2	1,304.65
									LKSDTAAAAVR	95.0%	63.2	21.1	4	4	0	2	1,102.62
									LQTSSVLVSGLR	95.0%	81.7	19.1	10	0	0	2	1,259.73
									LVVADTR	95.0%	35.2	24.6	1	0	0	2	773.45
									NEEDAAELVALAQAVNAR	95.0%	110.0	21.8	14	9	0	2	1,883.95
									NFPNAIEHTLQWAR	95.0%	55.8	22.3	1	0	0	2	1,696.86
									QPAENVNQYLTDPK	95.0%	76.0	22.6	8	0	0	2	1,616.79
									SLVASLAEPDFVVTDFAK	95.0%	93.3	22.0	19	0	0	2	1,909.00
									VGPDTER	95.0%	53.2	20.1	3	0	0	2	773.38
									YDGQVAVFGSDLQEK	95.0%	97.5	21.6	7	0	0	2	1,655.79
Proteasome subunit beta type-5	PSB5_HUMAN	PSMB5	28,463	100.00%	9	12	69	31.20%	ATAGAYIASQTVK	95.0%	110.0	21.0	13	0	0	2	1,280.69
									ATAGAYIASQTVKK	95.0%	83.7	19.3	2	0	0	2	1,408.78
									DAYSGGAVNLYHVR	95.0%	72.1	23.2	7	2	0	2	1,521.75
									GPGLYYVDSEGNR	95.0%	71.3	21.4	4	0	0	2	1,426.66
									HGVIVAADSR	95.0%	56.1	21.2	17	0	0	2	1,024.55
									ISVAAASK	95.0%	34.9	22.4	1	0	0	2	746.44
									LLANMVYQYK	95.0%	67.5	21.2	9	0	0	2	1,242.66
									RGPGLYYVDSEGNR	95.0%	67.8	22.4	2	1	0	2	1,582.76
									VSSDNVADLHEK	95.0%	84.0	22.5	8	3	0	2	1,313.63
Golgi apparatus protein 1	GSLG1_HUMAN	GLG1	134,536	100.00%	3	3	5	2.97%	FCENTQAGEGR	95.0%	49.2	17.0	1	0	0	2	1,268.53
									IIIQESALDYR	95.0%	66.4	22.0	2	0	0	2	1,320.72
									VAELSSDDFHLDR	95.0%	36.9	21.0	0	2	0	2	1,503.71
Coactosin-like protein	COTL1_HUMAN	COTL1	15,927	100.00%	11	12	40	70.40%	AGGANYDAQTE	95.0%	52.1	17.0	2	0	0	2	1,096.45
									ELEEDFIK	95.0%	38.4	22.8	3	0	0	2	1,022.50
									ELEEDFIKSELK	95.0%	58.2	22.8	2	0	0	2	1,479.76
									EVVQNFAK	95.0%	33.7	23.4	2	0	0	2	934.50
									FALITWIGENVSGLQR	95.0%	81.7	20.8	14	1	0	2	1,803.98
									FTTGDAMSK	95.0%	39.2	17.2	2	0	0	2	973.43
									FTTGDAMSKR	95.0%	46.6	21.6	2	0	0	2	1,113.54
									KAGGANYDAQTE	95.0%	36.2	20.1	1	0	0	2	1,224.55
									LFAFVR	95.0%	34.8	16.3	8	0	0	2	752.45
									TGTDKTLVK	95.0%	41.8	19.1	1	0	0	2	962.55
									YDGSTIVPGEQGAEYQHFIQQCTDDVR	95.0%	39.1	17.3	0	2	0	2	3,113.39
Aspartate aminotransferase, mitochondrial	AATM_HUMAN	GOT2	47,459	100.00%	11	12	78	28.80%	ASAELALGENSEVLK	95.0%	59.8	22.6	8	0	0	2	1,530.80
									DAGMQLQGYR	95.0%	76.5	21.2	10	0	0	2	1,138.53
									EIATVVK	95.0%	40.3	22.8	2	0	0	2	759.46
									FVTVQTISGTGALR	95.0%	107.0	20.9	35	0	0	2	1,449.81
									IAAAILNTPDLR	95.0%	74.9	18.0	8	0	0	2	1,267.74
									IAAAILNTPDLRK	95.0%	49.8	15.9	3	1	0	2	1,395.83
									IGASFLQR	95.0%	70.4	21.7	4	0	0	2	891.51
									ISVAGVTSSNVGYLAHAIHQVTK	95.0%	24.3	17.8	0	0	1	2	2,352.27
									MNLGVGAYR	95.0%	34.1	23.3	1	0	0	2	980.50
									NLDKEYLPIGGLAEFCK	95.0%	30.4	22.0	0	1	0	2	1,967.00
									NMGLYGER	95.0%	46.1	20.5	4	0	0	2	955.43
Nascent polypeptide-associated complex subunit alpha	NACA_HUMAN	NACA	23,365	100.00%	6	6	44	30.70%	IEDLSQQAQLAAAEK	95.0%	128.0	22.3	14	0	0	2	1,614.83
									NILFVITKPDVYK	95.0%	77.0	15.8	10	0	0	2	1,549.90
									NNSNDIVNAIMELTM	95.0%	40.6	20.3	3	0	0	2	1,710.77
									QVTGVTR	95.0%	35.5	24.2	1	0	0	2	760.43
									SKNILFVITKPDVYK	95.0%	40.7	13.4	0	2	0	2	1,765.03

									SPASDTYIVFGEAK	95.0%	107.0	22.5	14	0	0	2	1,484.73
Coatomer subunit beta'	COPB2_HUMAN	COPB2	102,471	100.00%	3	3	10	4.53%	AAESLADPTEYENLFPGLK	95.0%	78.6	22.0	6	0	0	2	2,065.01
									LPEAAFLAR	95.0%	64.0	22.5	3	0	0	2	987.56
									NVMEEGKDFQPSR	95.0%	37.0	20.9	1	0	0	2	1,552.71
G-protein coupled receptor 126	GP126_HUMAN	GPR126	136,681	99.50%	2	2	19	2.46%	LLLGSNQNEIVSLK	95.0%	87.7	17.9	8	0	0	2	1,527.87
									VILPQTSDAYQVSVAK	95.0%	71.2	20.5	11	0	0	2	1,718.93
Prefoldin subunit 2	PFD2_HUMAN	PFDN2	16,630	99.50%	2	2	3	16.90%	GAVSAEQVIAGFNR	95.0%	68.6	22.9	2	0	0	2	1,418.74
									IIETLTQQLQAK	95.0%	43.9	18.5	1	0	0	2	1,385.80
26S proteasome non-ATPase regulatory subunit 7	PSD7_HUMAN	PSMD7	37,008	99.50%	2	2	4	7.72%	DIKDTTVGTLSQR	95.0%	50.7	22.1	2	0	0	2	1,433.76
									SVVALHNLINNK	95.0%	62.6	18.9	2	0	0	2	1,321.76
Poly(ADP-ribose) glycohydrolase ARH3	ARHL2_HUMAN	ADPRHL2	38,929	100.00%	3	3	8	10.70%	ALVQSLLAK	95.0%	56.7	15.3	4	0	0	2	942.60
									HVQSLEPDPGTPGSER	95.0%	52.2	23.4	2	0	0	2	1,705.81
									TEALYYTDDTAMAR	95.0%	83.8	18.9	2	0	0	2	1,636.72
Methionine adenosyltransferase 2 subunit beta	MAT2B_HUMAN	MAT2B	37,534	100.00%	6	7	20	24.30%	AVLENNLGAAVLR	95.0%	90.4	19.1	3	0	0	2	1,339.77
									IPILYGEVEK	95.0%	36.8	21.0	1	0	0	2	1,160.66
									LETLGIGQR	95.0%	65.2	22.0	4	0	0	2	986.56
									LVEEEVNIPNRR	95.0%	42.4	21.0	2	1	0	2	1,467.79
									RPDVVENQPDAASQLNVDASGNLAK	95.0%	64.6	22.0	0	3	0	2	2,608.30
									VLVTGATGLLGR	95.0%	85.1	16.0	6	0	0	2	1,156.71
Brain acid soluble protein 1	BASP1_HUMAN	BASP1	22,675	100.00%	6	7	20	50.70%	AAEAAAAPAESAAPAAGEEPSKEEGEPK	95.0%	35.6	20.5	0	2	0	2	2,636.23
									AEPPKAPEQEQAAPGPAAGGEAPK	95.0%	46.4	21.8	0	2	0	2	2,298.14
									ESEPQAAAEPAEAK	95.0%	54.5	20.0	4	0	0	2	1,427.67
									ETPAATEAPSSTPK	95.0%	41.3	21.7	4	0	0	2	1,386.68
									KTEAPAAPAAQETK	95.0%	66.8	21.7	3	0	0	2	1,412.74
									SDGAPASDSKPGSSEAAPSSK	95.0%	50.1	21.2	3	2	0	2	1,932.88
Calcyclin-binding protein	CYBP_HUMAN	CACYBP	26,192	100.00%	2	2	2	19.30%	KAELLDNEKPAAVVAPITTGYTVK	95.0%	33.1	14.9	0	1	0	2	2,528.40
									SYSMIVNNLLKPISVEGSSK	95.0%	28.3	20.1	0	1	0	2	2,182.14
Proteasome subunit alpha type-6	PSA6_HUMAN	PSMA6	27,382	100.00%	8	10	75	37.40%	AINQGGLTSVAVR	95.0%	96.8	20.8	20	0	0	2	1,285.72
									GSSAGFDR	95.0%	46.3	18.3	2	0	0	2	796.36
									HITIFSPEGR	95.0%	60.8	21.5	12	0	0	2	1,156.61
									ILTEAEIDAHLVALAERD	95.0%	123.0	20.1	6	2	0	2	1,979.05
									LLDSSTVTHLFK	95.0%	73.4	21.6	10	5	0	2	1,360.75
									LYQVEYAFK	95.0%	54.5	23.7	10	0	0	2	1,160.60
									QTESTSFLEK	95.0%	45.3	22.4	4	0	0	2	1,169.57
									YGYEIPVDMLCK	95.0%	50.6	20.6	4	0	0	2	1,503.69
26S proteasome non-ATPase regulatory subunit 2	PSMD2_HUMAN	PSMD2	100,184	100.00%	6	6	14	9.69%	APVQPQQSPAAAPGGTDEKPSGK	95.0%	56.1	21.6	0	4	0	2	2,218.11
									AVPLALALISVSNPR	95.0%	33.1	12.0	0	1	0	2	1,520.92
									EDVLTLLLPVMGDSK	95.0%	63.4	20.9	3	0	0	2	1,629.88
									FGGSGSQVDSAR	95.0%	79.8	21.6	2	0	0	2	1,167.54
									HLAGEVAK	95.0%	37.7	18.9	1	0	0	2	824.46
									VGQAVDVVGQAGKPK	95.0%	84.5	18.1	3	0	0	2	1,452.82
Lactadherin	MFGM_HUMAN	MFGE8	43,105	100.00%	3	3	6	10.90%	EVTGIITQGAR	95.0%	83.5	23.1	4	0	0	2	1,144.63
									MWVTGVVTQGASR	95.0%	46.0	22.5	1	0	0	2	1,407.71
									NAVHVNLFETPVEAQYVR	95.0%	27.4	21.5	0	1	0	2	2,086.07
Myotrophin	MTPN_HUMAN	MTPN	12,877	100.00%	2	2	4	25.40%	GPDGLTAFEATDNQAIK	95.0%	103.0	22.9	2	0	0	2	1,747.85
									NGDLDEVKDYVAK	95.0%	49.4	22.7	2	0	0	2	1,465.72
Probable ATP-dependent RNA helicase DDX6	DDX6_HUMAN	DDX6	54,401	99.50%	2	2	6	7.25%	GPVKPTGGPGGGGTQTQQQMNQLK	95.0%	64.6	22.0	0	4	0	2	2,382.18
									SGAYLIPLLER	95.0%	40.5	19.2	2	0	0	2	1,231.71
Ubiquitin thioesterase OTUB1	OTUB1_HUMAN	OTUB1	31,267	100.00%	5	5	13	23.60%	EYAEDDNIYQQK	95.0%	68.4	18.7	4	0	0	2	1,515.66
									FFEHFIEGGR	95.0%	41.0	22.3	2	0	0	2	1,238.60
									GEGGTTNPHIFPEGSEPK	95.0%	38.3	21.5	0	1	0	2	1,853.87

									IQQEIAVQNPLVSER	95.0%	108.0	20.4	4	0	0	2	1,723.93
									LLTSGYLQR	95.0%	44.2	19.2	2	0	0	2	1,050.59
Integrin beta-4	ITB4_HUMAN	ITGB4	202,133	100.00%	9	9	38	5.98%	DVVSFEQPEFSVSR	95.0%	62.7	22.3	4	0	0	2	1,625.78
									EAIINLATQPK	95.0%	72.8	19.4	9	0	0	2	1,197.68
									LLELQEVDSLLR	95.0%	70.7	20.4	4	0	0	2	1,427.81
									LVFSALGPTSLR	95.0%	46.1	18.1	4	0	0	2	1,260.73
									MLLIENLR	95.0%	37.7	22.8	5	0	0	2	1,001.58
									NVISLTEDVDEFR	95.0%	40.6	22.3	2	0	0	2	1,536.75
									QEVEENLNEVYR	95.0%	64.9	22.1	2	0	0	2	1,521.72
									TQDYPSVPTLVR	95.0%	31.8	22.2	3	0	0	2	1,375.72
									VAPGYYTLTADQDAR	95.0%	110.0	22.6	5	0	0	2	1,640.79
60S ribosomal protein L10a	RL10A_HUMAN	RPL10A	24,814	100.00%	8	11	26	27.60%	AGKFPSLLTHNENMVAK	95.0%	36.3	20.8	0	2	1	2	1,856.97
									AVDIPHMDIEALK	95.0%	27.0	22.6	0	1	0	2	1,451.76
									AVDIPHMDIEALKK	95.0%	42.4	20.7	2	1	0	2	1,595.85
									DTLYEAVR	95.0%	52.1	22.5	6	0	0	2	966.49
									FPSLLTHNENMVAK	95.0%	49.5	22.9	2	0	0	2	1,616.81
									KYDAFLASESLIK	95.0%	70.5	21.2	3	5	0	2	1,484.80
									STMGKPQR	95.0%	30.7	24.1	1	0	0	2	904.47
									YDAFLASESLIK	95.0%	72.0	21.5	2	0	0	2	1,356.71
Clusterin	CLUS_HUMAN	CLU	52,477	100.00%	14	17	904	30.30%	ALQEYR	95.0%	45.8	24.6	2	0	0	2	779.41
									ASSIIDELFQDR	95.0%	105.0	23.5	188	0	0	2	1,393.70
									ELDESLQVAER	95.0%	65.7	22.5	242	0	0	2	1,288.64
									FMETVAEK	95.0%	57.8	19.0	83	0	0	2	970.46
									IDSLLENDR	95.0%	53.6	22.9	26	0	0	2	1,074.54
									KTLLSNLEEAK	95.0%	47.9	20.4	3	1	0	2	1,245.71
									KYNELLK	95.0%	43.6	19.1	28	0	0	2	907.53
									LFDSDPITVTVPVEVSR	95.0%	102.0	21.7	88	0	0	2	1,873.99
									QQTHMLDVMQDHFSR	95.0%	43.0	19.9	0	9	17	2	1,872.85
									RPHFFFPK	95.0%	35.2	23.9	0	57	0	2	1,075.58
									SGSGLVGR	95.0%	85.3	23.2	21	0	0	2	732.40
									TLLSNLEEAK	95.0%	71.8	23.1	30	0	0	2	1,117.61
									TLLSNLEEAKK	95.0%	52.6	20.4	11	0	0	2	1,245.71
									VTTVASHTSDSDVPSGVTEVVVK	95.0%	84.9	21.6	34	64	0	2	2,314.18
ATP-citrate synthase	ACLY_HUMAN	ACLY	120,825	100.00%	13	13	23	15.10%	AFDSGIIPMEFVNK	95.0%	47.2	23.0	2	0	0	2	1,567.78
									DLVSSLTSGLLTIGDR	95.0%	58.7	20.8	2	0	0	2	1,646.90
									EAYPEEAYIADLDAK	95.0%	59.5	21.0	1	0	0	2	1,697.79
									FICTTSAIQNR	95.0%	39.8	22.8	1	0	0	2	1,310.65
									IGNTGGMLDNILASK	95.0%	90.9	22.1	4	0	0	2	1,503.78
									LGQEATVGK	95.0%	54.2	23.9	2	0	0	2	902.49
									LYRPGSVAYVSR	95.0%	28.5	20.7	0	1	0	2	1,367.74
									QHFPATPLLDYALEVEK	95.0%	27.2	21.2	0	2	0	2	1,971.02
									SGGMSNELNNIISR	95.0%	58.6	21.5	2	0	0	2	1,507.72
									SINNPDMR	95.0%	39.1	20.6	1	0	0	2	962.44
									TIAIIAEGIPEALTR	95.0%	73.2	17.0	3	0	0	2	1,567.91
									TILSLMTR	95.0%	34.4	21.4	1	0	0	2	934.54
									WGDIEFPPPFGR	95.0%	30.4	22.5	1	0	0	2	1,417.69
N-acetylgalactosaminyltransferase 7	GALT7_HUMAN	GALNT7	75,373	99.90%	2	2	4	5.02%	LEGWQGNPPPIYVGSSPTLK	95.0%	67.1	20.9	2	0	0	2	2,140.11
									SPAMAGGLFAIER	95.0%	73.2	23.0	2	0	0	2	1,335.67
Ras GTPase-activating protein-binding protein 1	G3BP1_HUMAN	G3BP1	52,145	100.00%	3	3	14	9.87%	EAGEQGDIEPR	95.0%	42.3	20.8	3	0	0	2	1,200.55
									LPNFGFVVFDDSEPVQK	95.0%	80.2	22.1	6	0	0	2	1,937.97
									SSSPAPADIAQTVQEDLR	95.0%	92.7	22.0	5	0	0	2	1,884.93

U1 small nuclear ribonucleoprotein A	SNRPA_HUMAN	SNRPA	31,262	100.00%	4	4	10	17.00%	AVQGGGATPVVGAVQGPVPGMPPMTQAPR	95.0%	36.5	21.2	0	2	0	2	2,759.40
									EVSSATNALR	95.0%	56.3	23.1	4	0	0	2	1,047.54
									GQAFVIFK	95.0%	38.9	20.3	3	0	0	2	909.52
									KAVQGGGATPVVGAVQGPVPGMPPMTQAPR	95.0%	44.3	20.2	0	1	0	2	2,887.49
Dipeptidyl peptidase 1	CATC_HUMAN	CTSC	51,824	100.00%	2	3	8	4.10%	AINAIQK	95.0%	42.1	21.8	4	0	0	2	757.46
									NVHGINFVSPVR	95.0%	49.5	20.3	2	2	0	2	1,338.73
Dynactin subunit 2	DCTN2_HUMAN	DCTN2	44,214	100.00%	4	4	7	12.70%	ASVEDADTQSK	95.0%	69.6	20.5	2	0	0	2	1,150.52
									LLGPDAAINLTDPDGALAK	95.0%	90.6	20.0	2	0	0	2	1,865.00
									VAELEKR	95.0%	51.5	23.7	1	0	0	2	844.49
									WSPIASTLPELVQR	95.0%	80.7	19.2	2	0	0	2	1,596.88
Transferrin receptor protein 1	TFR1_HUMAN	TFRC	84,856	100.00%	11	12	42	17.00%	AAAEVAGQFVIK	95.0%	53.7	21.3	4	0	0	2	1,203.67
									DSAQNSVIIVDK	95.0%	67.3	23.4	2	0	0	2	1,288.68
									GFVEPDHYVVVGAQR	95.0%	69.6	22.7	1	2	0	2	1,672.84
									ILNIFGVIK	95.0%	51.1	12.3	7	0	0	2	1,016.65
									LAQMFSDMVLK	95.0%	43.2	21.6	2	0	0	2	1,314.64
									LLNENSYVPR	95.0%	77.2	23.9	2	0	0	2	1,204.63
									SGVGTALLLK	95.0%	43.9	17.6	3	0	0	2	958.59
									SSGLPNIPVQTISR	95.0%	55.0	19.4	6	0	0	2	1,468.81
									VEYHFLSPYVSPK	95.0%	30.7	22.2	0	1	0	2	1,565.80
									VSASPLLYTLIEK	95.0%	102.0	17.0	9	0	0	2	1,433.83
									YNSQLLSFVR	95.0%	62.3	21.7	3	0	0	2	1,226.65
Involucrin	INVO_HUMAN	IVL	68,457	100.00%	5	5	7	12.00%	HLEEQEGQLK	95.0%	41.5	22.9	2	0	0	2	1,210.61
									HLEQQQGQLEVPEQQVGQPK	95.0%	33.8	21.5	0	1	0	2	2,300.16
									HLVQQEGQLEQQER	95.0%	29.3	21.9	0	2	0	2	1,721.86
									QEAQLELPEQQVGQPK	95.0%	63.9	22.9	1	0	0	2	1,821.94
									YLEQQEGQLK	95.0%	34.1	22.9	1	0	0	2	1,235.63
Glucosidase 2 subunit beta	GLU2B_HUMAN	PRKCSH	59,408	100.00%	17	19	142	32.20%	AQQEQELAADAFK	95.0%	93.1	23.1	11	0	0	2	1,448.70
									ERESLQQMAEVTR	95.0%	27.0	22.6	0	1	0	2	1,576.78
									ESLQQMAEVTR	95.0%	62.1	22.8	10	0	0	2	1,291.63
									ETMVTSTTEPSR	95.0%	72.4	21.1	35	0	0	2	1,354.62
									ILIEDWK	95.0%	32.9	24.2	1	0	0	2	916.51
									ILIEDWKK	95.0%	31.3	21.0	1	0	0	2	1,044.61
									LGGSPTSLGTWGSWIGPDHDK	95.0%	51.2	21.7	0	2	0	2	2,168.04
									LGGSPTSLGTWGSWIGPDHDKFSAMK	95.0%	23.8	21.0	0	0	1	2	2,748.31
									LIELQAGKK	95.0%	33.5	18.3	2	0	0	2	999.62
									LVSQKPK	95.0%	36.2	18.6	2	0	0	2	799.50
									LWEEQLAAAK	95.0%	61.3	23.7	11	0	0	2	1,158.62
									NKFEEAER	95.0%	34.1	21.8	2	0	0	2	1,022.49
									SEALPTDLPAPSAPDLTEPK	95.0%	82.2	21.6	4	0	0	2	2,049.04
									SLEDQVEMLR	95.0%	74.8	22.8	10	0	0	2	1,219.60
									SLKDMEESIR	95.0%	56.1	22.7	2	6	0	2	1,207.60
									TVKEEAEKPER	95.0%	46.2	23.0	7	32	0	2	1,315.69
									YEQGTGCWQGPNR	95.0%	70.4	18.0	2	0	0	2	1,552.66
Ras-related protein Rab-1A	RAB1A_HUMAN	RAB1A	22,660	100.00%	5	5	15	36.10%	EFADSLGIPFLETSAK	95.0%	77.3	23.1	7	0	0	2	1,724.87
									IQSTPVK	95.0%	31.5	20.7	2	0	0	2	772.46
									LLLIGDSGVGK	95.0%	58.7	20.3	2	0	0	2	1,071.64
									LQIWDTAGQER	95.0%	61.5	22.2	7	0	0	2	1,316.66
									MGPGATAGGAEK	95.0%	55.5	20.3	2	0	0	2	1,062.49
									NATNVEQSFMTMAAEIK	95.0%	103.0	20.3	2	0	0	2	1,916.87
Heterogeneous nuclear ribonucleoprotein K	HNRPK_HUMAN	HNRNPK	50,961	100.00%	9	11	68	26.30%	ENTQTTIK	95.0%	31.6	23.3	1	0	0	2	934.48
									GSYGDLGGPIITTQVTIPK	95.0%	97.7	19.2	5	0	0	2	1,917.03

									IDEPLEGSEDR	95.0%	78.2	19.5	2	0	0	2	1,259.58
									IILDLISESPIK	95.0%	99.9	14.9	36	0	0	2	1,340.80
									IITITGTQDQIQNAQYLLQNSVK	95.0%	59.6	17.2	1	7	0	2	2,589.39
									NAGAVIGK	95.0%	34.0	23.3	2	0	0	2	729.43
									RPAEDMEEEQAFKR	95.0%	52.2	21.5	0	2	0	2	1,751.80
									TDYNASVSVPDSSGPER	95.0%	81.8	19.4	8	0	0	2	1,780.80
									VVLIGGKPDR	95.0%	33.2	12.6	2	2	0	2	1,053.64
Collagen alpha-1(XII) chain	COCA1_HUMAN	COL12A1	333,127	100.00%	56	58	484	24.50%	ALALGALQNIR	95.0%	91.3	11.1	24	0	0	2	1,139.69
									DITDTSIGAYWTSAPGMVR	95.0%	45.6	21.8	2	0	0	2	2,056.97
									DYKPQVGVIADPSSK	95.0%	50.1	22.6	2	0	0	2	1,603.83
									FNQMLNQIPNDYQSSR	95.0%	92.1	20.5	7	0	0	2	1,954.91
									GDTTNTVLQGLK	95.0%	57.4	22.9	14	0	0	2	1,246.66
									GEVQTVTFDTEEVK	95.0%	96.3	22.9	12	0	0	2	1,581.77
									GGNTLTGMALNFIR	95.0%	46.7	22.1	2	0	0	2	1,464.76
									GGNTMTGDAIDYLVK	95.0%	89.9	22.6	20	0	0	2	1,570.74
									GMTSSEPISIMEK	95.0%	76.9	20.5	12	0	0	2	1,441.66
									GPGDLEAPSNLVISER	95.0%	87.6	22.5	25	0	0	2	1,653.85
									GQEITVR	94.6%	30.5	25.5	1	0	0	2	802.44
									IEQELAAIK	95.0%	47.8	20.4	2	0	0	2	1,014.58
									IIYRPVAGGESR	95.0%	32.6	21.7	1	4	0	2	1,317.73
									IIYSPTVGDPIDEYTTVPGR	95.0%	65.1	21.7	4	0	0	2	2,193.11
									ITEVTSEGFR	95.0%	72.8	23.2	14	0	0	2	1,138.57
									ITVDPTTDGPTK	95.0%	41.6	23.4	6	0	0	2	1,244.64
									ITWTQAPGR	95.0%	32.9	22.0	2	0	0	2	1,029.55
									ITYQPSTGEGNEQTTTIGGR	95.0%	124.0	21.1	17	0	0	2	2,110.01
									IVEVFDIGPK	95.0%	62.0	21.3	28	0	0	2	1,116.63
									LGELVVGPYDNTVVLEELR	95.0%	75.2	20.2	6	0	0	2	2,115.13
									LKPDTPYTITVSSLYPDGEGGR	95.0%	37.6	21.3	0	6	0	2	2,366.19
									LLPETPSDPFAIWQITDR	95.0%	36.0	21.2	4	0	0	2	2,099.08
									LNWNPSPSPVTGYK	95.0%	62.1	23.0	10	0	0	2	1,559.79
									LQPQTTYDITVLPIYK	95.0%	69.4	17.7	20	0	0	2	1,893.04
									LSPADGTR	95.0%	34.2	21.6	2	0	0	2	816.42
									MIATDPDDTHAYNVADFESLSR	95.0%	55.0	18.1	0	3	0	2	2,484.10
									NLQPDTSYTVTVVPVYTEGDGGR	95.0%	79.1	21.3	6	0	0	2	2,468.19
									NNVILQPLQPDTPYK	95.0%	62.1	21.0	4	0	0	2	1,739.93
									NSDVEIFAVGVK	95.0%	66.7	22.7	3	0	0	2	1,277.67
									NTFTESAGAR	95.0%	40.7	22.5	6	0	0	2	1,053.50
									NVGVEVFSLGIK	95.0%	62.3	20.6	20	0	0	2	1,261.72
									NVQVYNPTPNSLDVR	95.0%	50.6	22.0	2	0	0	2	1,715.87
									QHALSVGPQTTTLSVR	95.0%	31.9	20.2	0	3	0	2	1,694.92
									QVCEQLISGQMNR	95.0%	63.1	22.4	2	0	0	2	1,562.74
									SLLQAVANLPYK	95.0%	68.4	18.3	37	0	0	2	1,316.76
									SLYDDVDTGEK	95.0%	39.5	21.3	6	0	0	2	1,241.55
									SQDDVEAPSKK	95.0%	44.4	22.8	1	0	0	2	1,203.59
									SQDEVEIPAR	95.0%	60.3	21.4	16	0	0	2	1,143.56
									TEFNLNQYYQR	95.0%	48.3	21.3	4	0	0	2	1,475.69
									TLENLIPDTK	95.0%	38.4	23.6	2	0	0	2	1,143.63
									TLKVDEETENTMR	95.0%	40.5	22.0	2	0	0	2	1,581.74
									TSDPTMSSFR	95.0%	32.2	19.7	1	0	0	2	1,144.49
									VEDIIEAINTFPYR	95.0%	62.9	22.3	11	6	0	2	1,679.86
									VGVVQYSSDTR	95.0%	88.7	23.1	31	0	0	2	1,210.61

									VILTPMTAGSR	95.0%	33.3	22.7	1	0	0	2	1,145.64
									VLVVVTDGR	95.0%	58.0	20.0	8	0	0	2	957.57
									VPPTVTSTVLK	95.0%	34.9	18.1	2	0	0	2	1,141.68
									VQIALAQYSGDPR	95.0%	82.7	22.5	6	0	0	2	1,417.74
									VSDPTTSTMK	95.0%	49.5	21.6	6	0	0	2	1,082.50
									VSWDPSPSPVLGYK	95.0%	69.6	23.2	13	0	0	2	1,531.78
									VSWTPPSDSVDR	95.0%	33.0	21.2	2	0	0	2	1,345.64
									VTDETTDSFK	95.0%	64.2	20.0	10	0	0	2	1,142.52
									VTWEPAPGEVK	95.0%	33.2	21.3	1	0	0	2	1,212.63
									VVYSPVDGTRPSESIVVPGNTR	95.0%	42.5	20.4	0	8	0	2	2,329.22
									VYDPSTSTLNVR	95.0%	80.8	22.6	12	0	0	2	1,351.69
									WDPAPGPVLQYR	95.0%	53.7	22.0	2	0	0	2	1,398.72
Dipeptidyl peptidase 3	DPP3_HUMAN	DPP3	82,574	100.00%	12	14	69	23.50%	AQDPDQLR	95.0%	34.1	20.5	1	0	0	2	942.46
									EVDGEGKPYYEVR	95.0%	51.4	22.7	4	2	0	2	1,540.73
									FPEDGPELEEILTQLATADAR	95.0%	33.3	21.5	0	1	0	2	2,315.14
									FSTIASSYEECR	95.0%	69.1	18.4	4	0	0	2	1,449.63
									GEFEGFVAVVNK	95.0%	48.4	22.1	2	0	0	2	1,295.66
									LAQDFLDSQNLSAYNTR	95.0%	125.0	21.5	10	0	0	2	1,955.95
									LASVLGSEPSLDSEVTSK	95.0%	65.7	22.7	8	0	0	2	1,818.93
									NVSLGNVLAVAYATQR	95.0%	117.0	20.0	8	0	0	2	1,675.91
									SGETWDSK	95.0%	60.8	20.0	3	0	0	2	909.40
									VILGSEAAQQHPEEVR	95.0%	90.8	21.7	4	4	0	2	1,762.91
									VLLEAGEGLVTITPTTGSDGRPDAR	95.0%	70.2	19.8	0	15	0	2	2,525.32
									VVEQLEK	95.0%	40.9	20.9	3	0	0	2	844.48
Branched-chain-amino-acid aminotransferase, mitochondrial	BCAT2_HUMAN	BCAT2	44,270	100.00%	6	6	14	21.20%	AADLQLEMTQKPHK	95.0%	37.5	22.2	1	0	0	2	1,625.83
									KPGPGEPLVFGK	95.0%	43.7	19.2	2	0	0	2	1,225.69
									LFRPWLNMDR	95.0%	26.9	23.5	0	1	0	2	1,363.69
									LGGNYGPTVLVQQEALK	95.0%	70.2	19.5	2	0	0	2	1,786.97
									NLHIPTMENGPELILR	95.0%	42.6	21.2	0	3	0	2	1,862.98
									QSLLDMAQTWGEFR	95.0%	88.7	21.8	5	0	0	2	1,681.80
Keratinocyte proline-rich protein	KPRP_HUMAN	KPRP	64,115	100.00%	4	4	48	6.74%	GRPAVCQPQGR	95.0%	41.6	22.6	0	43	0	2	1,225.62
									IEISSPCCPR	95.0%	37.3	20.1	1	0	0	2	1,218.56
									LQLFPR	95.0%	35.0	23.2	3	0	0	2	773.47
									RLDQCPESPLQR	95.0%	52.8	21.6	1	0	0	2	1,498.74
Granulins	GRN_HUMAN	GRN	63,522	100.00%	16	20	423	26.30%	APAHLSLPDPQALK	95.0%	57.7	20.4	11	5	0	2	1,457.81
									APAHLSLPDPQALKR	95.0%	64.5	18.8	8	51	29	2	1,613.91
									ASLSHPR	95.0%	58.1	20.6	43	0	0	2	767.42
									AVALSSSVMCPDAR	95.0%	81.5	21.7	25	0	0	2	1,479.69
									CITPTGTHPLAK	95.0%	58.6	21.7	15	4	0	2	1,295.68
									EVVSAQPATFLAR	95.0%	87.5	22.3	99	0	0	2	1,388.75
									GSEIVAGLEK	95.0%	66.4	22.8	77	0	0	2	1,002.55
									LPAHTVGDVK	95.0%	53.1	20.0	22	0	0	2	1,036.58
									QGVCCADR	95.0%	46.8	14.9	1	0	0	2	965.39
									QGWACCPYR	95.0%	44.3	15.8	8	0	0	2	1,197.49
									QHCCPAGYTCNVK	95.0%	51.9	15.8	6	0	0	2	1,594.66
									SCEKEVVSAQPATFLAR	95.0%	43.4	22.0	0	3	0	2	1,892.95
									SPHVGVK	95.0%	39.2	17.8	10	0	0	2	723.42
									VHCCPHGAFCDLVHTR	95.0%	20.5	18.3	0	0	1	2	1,965.86
									WDAPLRDPALR	95.0%	28.9	21.6	0	3	0	2	1,309.70
									WPTTLSR	95.0%	32.6	24.1	2	0	0	2	860.46
Prostasin	PRSS8_HUMAN	PRSS8	36,413	99.50%	2	2	4	6.12%	LGAHQLDSYSEDAK	95.0%	36.3	22.0	0	1	0	2	1,533.72

									VTELQPR	95.0%	40.4	20.4	3	0	0	2	842.47
Polyadenylate-binding protein 1	PABP1_HUMAN	PABPC1	70,653	100.00%	17	19	73	29.90%	ALDTMNFDVIK	95.0%	73.5	21.7	12	0	0	2	1,282.64
									EFSPFGTITSAK	95.0%	49.8	22.1	6	0	0	2	1,284.65
									FSPAGPILSIR	95.0%	62.9	21.0	11	0	0	2	1,157.67
									GFGFVCFSSPEEATK	95.0%	61.6	19.8	1	0	0	2	1,662.75
									GFGFVSFER	95.0%	34.4	22.3	3	0	0	2	1,045.51
									IVATKPLYVALAQR	95.0%	63.1	10.8	2	7	0	2	1,542.94
									KEFSPFGTITSAK	95.0%	67.8	21.4	2	0	0	2	1,412.74
									NFGEDMDDER	95.0%	45.2	8.5	3	0	0	2	1,243.45
									NFGEDMDDERLK	95.0%	39.2	18.3	1	0	0	2	1,484.63
									NLDDGIDDERLR	95.0%	36.6	22.4	2	0	0	2	1,430.69
									QAHLTNQYMQR	95.0%	39.9	22.1	2	3	0	2	1,405.66
									QTELKR	95.0%	32.2	24.1	1	0	0	2	774.45
									SGVGNIFIK	95.0%	67.8	19.9	6	0	0	2	934.54
									SKVDEAVAVLQAHQAK	95.0%	46.0	19.5	0	2	0	2	1,693.92
									SLGYAYVNFQQPADAER	95.0%	97.2	21.3	5	0	0	2	1,928.91
									VANTSTQTMGPRPAAAAAAATPAVR	95.0%	43.8	21.0	0	2	0	2	2,397.23
									YQGVNLYVK	95.0%	43.2	20.8	2	0	0	2	1,083.58
FACT complex subunit SSRP1	SSRP1_HUMAN	SSRP1	81,060	99.90%	2	2	3	2.68%	ASSGLLYPLER	95.0%	37.3	22.7	1	0	0	2	1,205.65
									LFDFVNAK	95.0%	36.4	20.9	2	0	0	2	953.51
Protein SET	SET_HUMAN	SET	33,471	100.00%	8	12	148	31.40%	EFHLNESGDPSSK	95.0%	77.4	20.5	29	1	0	2	1,446.65
									EQQEAIEHIDEVQNEIDR	95.0%	96.5	21.7	2	2	0	2	2,195.02
									EQQEAIEHIDEVQNEIDRLNEQASEEILK	95.0%	60.4	20.5	0	1	1	2	3,449.67
									IDFYFDENPYFENK	95.0%	86.6	19.1	32	0	0	2	1,840.81
									KPRPPPALGPEETSASAGLPK	95.0%	79.9	19.6	0	2	0	2	2,100.15
									LNEQASEEILK	95.0%	59.6	23.3	13	0	0	2	1,273.66
									LNEQASEEILKVEQK	95.0%	96.7	21.1	3	5	0	2	1,757.93
									VEVTEFEDIK	95.0%	76.7	23.6	57	0	0	2	1,208.61
Poly(rC)-binding protein 1	PCBP1_HUMAN	PCBP1	37,480	100.00%	2	2	12	9.27%	ESTGAQVQVAGDMLPNSTER	95.0%	107.0	21.7	2	0	0	2	2,105.98
									IITLTGPTNAIFK	95.0%	53.3	16.4	10	0	0	2	1,388.82
Flavin reductase	BLVRB_HUMAN	BLVRB	22,101	100.00%	5	6	28	42.70%	LPSEGPRPAHVVVGDVLQAADVDK	95.0%	88.9	19.8	0	4	1	2	2,469.31
									LQAVTDDHIR	95.0%	43.7	23.2	4	0	0	2	1,167.61
									NDLSPTTVMSEGAR	95.0%	73.1	21.5	6	0	0	2	1,493.69
									TVAGQDAVIVLLGTR	95.0%	121.0	17.9	9	0	0	2	1,512.88
									YVAVMPPHIGDQPLTGAYTVTLDGR	95.0%	67.4	21.2	0	4	0	2	2,687.35
HLA class I histocompatibility antigen, A-11 alpha chain	1A11_HUMAN	HLA-A	40,919	100.00%	4	4	33	18.40%	APWIEQEGPEYWDQETR	95.0%	97.9	18.7	2	0	0	2	2,133.95
									DGEDQTQDTELVETRPAGDGTFQK	95.0%	67.8	20.8	0	12	0	2	2,637.19
									SWTAADMAAQITK	95.0%	76.3	22.7	5	0	0	2	1,393.68
									WAAVVVPSGEEQR	95.0%	55.7	23.0	14	0	0	2	1,427.73
CD109 antigen	CD109_HUMAN	CD109	161,674	100.00%	33	41	575	24.40%	ADGNQLTLEER	95.0%	78.1	22.5	32	0	0	2	1,245.61
									ADGNQLTLEERR	95.0%	68.6	22.2	0	2	0	2	1,401.71
									AEQEGGMQFWVSSESK	95.0%	93.8	19.0	16	0	0	2	1,815.79
									AKEALNMLTWR	95.0%	56.7	22.6	2	0	0	2	1,348.70
									ALSEFAALMNTER	95.0%	98.9	22.0	38	0	0	2	1,468.71
									AYFLGSK	95.0%	44.2	19.7	7	0	0	2	785.42
									DYIDGVYDNAEYAER	95.0%	98.4	18.8	8	0	0	2	1,792.77
									EALNMLTWR	95.0%	60.8	23.0	14	0	0	2	1,133.58
									ELLYQR	95.0%	32.0	22.7	4	0	0	2	821.45
									ELSYMVVSR	95.0%	54.0	21.8	10	0	0	2	1,083.55
									FLIDTHNR	95.0%	55.2	22.9	13	0	0	2	1,015.53
									GDVTLTFLPLSFWGK	95.0%	47.6	20.7	2	0	0	2	1,680.90

									IEFPILEDSSELQLK	95.0%	104.0	21.5	50	2	0	2	1,760.93
									IPVQLVFK	95.0%	57.2	17.0	22	0	0	2	943.60
									ISVFIQTDK	95.0%	52.2	21.5	7	0	0	2	1,050.58
									ISVTQPDSIVGIVAVDK	95.0%	99.5	18.3	42	1	0	2	1,740.97
									IVTLFSDFKPYK	95.0%	59.6	20.9	23	1	0	2	1,457.80
									LKELSYMVVSR	95.0%	46.1	20.9	2	2	0	2	1,340.72
									LSDSWQPR	95.0%	68.6	21.7	15	0	0	2	988.49
									NNVVITVTQR	95.0%	52.6	20.7	3	0	0	2	1,143.65
									NSLGGFASTQDTTVALK	95.0%	101.0	21.9	25	0	0	2	1,709.87
									SNGEFWDPGR	95.0%	50.9	17.9	6	0	0	2	1,164.51
									SNLIQQWLSQQSDLGVISK	95.0%	122.0	20.3	37	4	0	2	2,144.14
									SSMAVHSLFK	95.0%	39.9	24.1	4	0	0	2	1,106.57
									SYSQSILLDLTDNR	95.0%	92.4	22.0	26	0	0	2	1,624.82
									TLSFSFPPNTVTGSER	95.0%	79.8	22.0	49	0	0	2	1,739.86
									TLTLPSLPLNSADEIYELR	95.0%	101.0	20.0	46	2	0	2	2,145.14
									TNIQVTVTGPSSPSPVK	95.0%	105.0	20.4	8	0	0	2	1,711.92
									TRDENIK	95.0%	35.9	24.5	3	0	0	2	875.46
									TYIQLK	95.0%	33.1	19.1	2	0	0	2	765.45
									VGSPFELVVSGNK	95.0%	90.7	22.3	15	0	0	2	1,332.72
									VGSPFELVVSGNKR	95.0%	81.5	20.3	17	9	0	2	1,488.82
									VIHSELQGGNK	95.0%	35.3	22.6	1	3	0	2	1,181.63
Serine/threonine-protein phosphatase 2A activator	PTPA_HUMAN	PPP2R4	40,650	100.00%	6	7	19	20.90%	FGSLLPIHPVTSG	95.0%	63.3	20.5	6	0	0	2	1,324.73
									FPVIQHFK	95.0%	33.1	23.0	1	0	0	2	1,015.57
									LVALLNTLDR	95.0%	76.9	19.1	5	0	0	2	1,127.68
									QPPPDSSEEAPPATQNFIIPK	95.0%	47.0	21.3	1	1	0	2	2,263.13
									VDDQIAIVFK	95.0%	49.0	21.8	2	0	0	2	1,147.64
									WIDETPPVDQPSR	95.0%	51.2	23.2	3	0	0	2	1,539.74
Heat shock cognate 71 kDa protein	HSP7C_HUMAN	HSPA8	70,882	100.00%	42	60	1243	61.00%	ARFEELNADLFR	95.0%	53.8	22.1	2	21	0	2	1,480.75
									ATVEDEK	95.0%	32.9	23.2	2	0	0	2	791.38
									CNEIINWLDK	95.0%	51.4	24.2	2	0	0	2	1,304.63
									DAGTIAGLNVLR	95.0%	109.0	22.0	100	0	0	2	1,199.68
									EIAEAYLGK	95.0%	50.7	21.7	10	0	0	2	993.53
									FDDAVVQSDMK	95.0%	88.0	19.8	12	0	0	2	1,270.56
									FEELNADLFR	95.0%	56.2	22.7	31	0	0	2	1,253.62
									FELTGIPPAPR	95.0%	35.9	19.7	1	0	0	2	1,197.66
									GPAVGIDLGTTYSCVGVFQHGK	95.0%	52.1	21.4	0	24	0	2	2,263.12
									GTLDPVEK	95.0%	46.9	21.5	10	0	0	2	858.46
									HWPFMVVNDAGRPK	95.0%	61.0	22.6	0	53	0	2	1,669.83
									IINEPTAAAIAYGLDK	95.0%	114.0	21.5	30	4	0	2	1,659.90
									IINEPTAAAIAYGLDKK	95.0%	129.0	17.0	25	21	0	2	1,787.99
									ITITNDKGR	95.0%	64.4	22.3	20	0	0	2	1,017.57
									LDKSQIHDIVLVGGSTR	95.0%	86.0	19.3	6	27	1	2	1,838.01
									LLQDFFNGK	95.0%	50.0	22.1	13	0	0	2	1,081.57
									LSKEDIER	95.0%	62.1	23.4	19	0	0	2	989.53
									MKEIAEAYLGK	95.0%	71.7	22.1	26	52	0	2	1,268.66
									MVNHFIAEFK	95.0%	46.7	22.9	21	5	0	2	1,235.62
									MVNHFIAEFKR	95.0%	34.3	22.7	1	1	0	2	1,407.72
									MVQEAEK	95.0%	49.8	23.2	3	0	0	2	834.40
									MVQEAEKYK	95.0%	32.2	23.3	3	0	0	2	1,125.56
									NQTAEKEEFEHQQK	95.0%	69.7	20.9	6	8	0	2	1,745.81
									NQVAMNPTNTVFDAK	95.0%	106.0	22.6	60	0	0	2	1,665.79

									NQVAMNPTNTVFDAKR	95.0%	83.9	22.6	4	1	0	2	1,821.89
									NSLESYAFNMK	95.0%	89.2	20.6	50	0	0	2	1,303.60
									NTTIPTK	95.0%	36.0	24.6	1	0	0	2	774.44
									QATKDAGTIAGLNVLR	95.0%	58.2	19.0	0	2	0	2	1,627.91
									QTQTFTTYSDNQPGVLIQVYEGER	95.0%	75.4	21.2	13	43	0	2	2,774.33
									RFDDAVVQSDMK	95.0%	68.8	21.3	20	6	0	2	1,426.66
									RNTTIPTK	95.0%	39.2	22.1	2	0	0	2	930.54
									SFYPEEVSSMVLTK	95.0%	102.0	22.3	83	1	0	2	1,632.78
									SINPDEAVAYGAAVQAAILSGDK	95.0%	134.0	21.7	38	4	0	2	2,260.15
									SQIHDIVLVGGSTR	95.0%	87.1	21.0	38	16	0	2	1,481.81
									STAGDTHLGGEDFDNR	95.0%	75.2	17.4	27	26	0	2	1,691.73
									TTPSYVAFTDTER	95.0%	106.0	21.6	97	0	0	2	1,487.70
									TVTNAVVTVPAYFNDSQR	95.0%	89.9	21.8	52	9	0	2	1,982.00
									VCNPIITK	95.0%	38.8	24.7	5	0	0	2	944.52
									VEIIANDQGNR	95.0%	80.0	22.6	56	0	0	2	1,228.63
									VQVEYK	95.0%	37.3	22.1	2	0	0	2	765.41
									VQVEYKGETK	95.0%	54.7	22.5	24	1	0	2	1,180.62
									YKAEDEK	95.0%	36.8	22.0	2	0	0	2	882.42
Heterogeneous nuclear ribonucleoprotein U-like protein 1	HNRL1_HUMAN	HNRNPUL1	95,722	100.00%	5	5	16	8.88%	EALGGQALYPHVLVK	95.0%	63.0	18.1	2	0	0	2	1,594.90
									HLPSTEPDPHVVR	95.0%	43.4	22.1	0	8	0	2	1,483.77
									NYILDQTNVYGSAQR	95.0%	94.8	21.6	2	0	0	2	1,741.85
									QGAPTSFLPPEASQLKPDR	95.0%	34.7	21.1	0	3	0	2	2,039.06
									WDVLIQQATQCLNR	95.0%	86.9	22.7	1	0	0	2	1,744.88
Chromobox protein homolog 3	CBX3_HUMAN	CBX3	20,794	100.00%	6	7	19	45.40%	GFTDADNTWEPEENLDCPELIEAFLNSQK	95.0%	46.2	17.9	0	1	0	2	3,382.51
									IIGATDSSGELMFLMK	95.0%	97.2	22.3	6	0	0	2	1,744.85
									KVEEAEPEEFVVEK	95.0%	84.4	23.0	3	2	0	2	1,661.83
									SLSDSESDDSK	95.0%	63.7	17.0	2	0	0	2	1,169.48
									VEEAEPEEFVVEK	95.0%	58.1	22.7	3	0	0	2	1,533.73
									WKDSDEADLVLAK	95.0%	58.2	23.1	2	0	0	2	1,489.75
Elongation factor 1-gamma	EF1G_HUMAN	EEF1G	50,101	100.00%	12	13	79	25.60%	AKDPFAHLPK	95.0%	42.1	21.0	0	11	0	2	1,123.63
									ALIAAQYSGAQVR	95.0%	92.8	21.2	11	0	0	2	1,347.74
									EYFSWEGAFQHVGK	95.0%	48.2	21.2	2	0	0	2	1,684.78
									FAETQPK	95.0%	33.4	23.8	4	0	0	2	820.42
									ILGLLDAYLK	95.0%	63.5	13.0	19	0	0	2	1,118.68
									KLDPGSEETQTLVR	95.0%	76.4	22.2	6	7	0	2	1,572.82
									LDPGSEETQTLVR	95.0%	66.9	22.3	4	0	0	2	1,444.73
									MAQFDAK	95.0%	31.1	18.9	1	0	0	2	826.38
									QAFPNTNR	95.0%	32.1	22.6	1	0	0	2	947.47
									STFVLDEFKR	95.0%	51.7	21.3	9	0	0	2	1,241.65
									TFLVGER	95.0%	35.5	22.7	1	0	0	2	821.45
									WFLTCINQPQFR	95.0%	46.2	22.2	3	0	0	2	1,609.80
Purine nucleoside phosphorylase	PNPH_HUMAN	PNP	32,100	100.00%	14	17	116	51.20%	ANHEEVLAAGK	95.0%	63.4	23.4	8	7	0	2	1,138.59
									DHINLPGFSGQNPLR	95.0%	64.7	22.8	6	9	0	2	1,664.85
									DHINLPGFSGQNPLRGPNDER	95.0%	22.6	20.9	0	0	1	2	2,333.14
									FEVGDIMLIR	95.0%	58.2	22.1	17	0	0	2	1,192.64
									FGDRFPAMSDAYDR	95.0%	46.7	19.2	0	5	0	2	1,647.72
									FPAMSDAYDR	95.0%	55.8	16.5	4	0	0	2	1,172.50
									LEQFVSILMASIPLPDK	95.0%	53.6	19.2	2	0	0	2	1,917.04
									LEQFVSILMASIPLPDKAS	95.0%	32.2	20.2	1	0	0	2	2,075.11
									LGADAVGMSTVPEVIVAR	95.0%	79.8	21.1	19	0	0	2	1,800.95
									LTQAQIFDYGEIPNFPR	95.0%	69.4	21.7	5	1	0	2	2,009.01

									LVFGFLNGR	95.0%	62.5	21.0	4	0	0	2	1,022.58
									STVPGHAGR	95.0%	39.3	22.1	1	0	0	2	881.46
									VFGFSLITNK	95.0%	75.8	20.0	19	0	0	2	1,125.63
									VIMDYESLEK	95.0%	47.7	22.2	7	0	0	2	1,242.59
Vimentin	VIME_HUMAN	VIM	53,635	100.00%	3	3	63	3.43%	FLEQQNK	95.0%	47.6	24.2	58	0	0	2	906.47
									KLLEGEESR	95.0%	56.2	23.9	1	0	0	2	1,060.56
									LLEGEESR	95.0%	43.0	23.5	4	0	0	2	932.47
Asparaginyl-tRNA synthetase, cytoplasmic	SYNC_HUMAN	NARS	62,926	100.00%	10	11	21	23.40%	EDGTFYEFGEDIPEAPER	95.0%	68.1	18.3	2	0	0	2	2,100.90
									EGIDPTPYYWYTDQR	95.0%	52.6	20.2	2	0	0	2	1,903.85
									EPFPTIYVDSQKENER	95.0%	29.8	22.0	0	1	0	2	1,951.94
									FLTWILNR	95.0%	41.5	19.8	2	0	0	2	1,062.61
									IFDSEEILAGYK	95.0%	38.0	22.7	1	0	0	2	1,384.70
									KEDGTFYEFGEDIPEAPER	95.0%	34.7	19.7	0	1	0	2	2,229.00
									LTESVDVLMPNVGEIVGGSMR	95.0%	77.0	22.3	2	0	0	2	2,235.10
									MNYSDAIVWLK	95.0%	57.1	22.4	4	0	0	2	1,339.67
									NLMFLVLR	95.0%	40.9	18.9	2	0	0	2	1,021.59
									SPAGSIVHELNPNFQPPK	95.0%	70.8	22.7	2	2	0	2	1,932.00
UTP--glucose-1-phosphate uridylyltransferase	UGPA_HUMAN	UGP2	56,924	100.00%	3	4	9	7.48%	GLPDNISSVLNK	95.0%	31.7	22.2	1	0	0	2	1,256.69
									IQRPPEDSIQPYEK	95.0%	57.5	22.0	2	2	0	2	1,699.87
									SFENSLGINVPR	95.0%	67.0	23.1	4	0	0	2	1,332.69
Cullin-4B	CUL4B_HUMAN	CUL4B	103,969	100.00%	4	4	5	4.93%	ETVEEQASTTER	95.0%	31.7	20.7	1	0	0	2	1,379.63
									LPENYTDETWQK	95.0%	48.6	21.4	1	0	0	2	1,523.70
									QYQIDAAIVR	95.0%	49.8	23.0	2	0	0	2	1,176.64
									TIDGILLLIER	95.0%	40.1	14.9	1	0	0	2	1,255.76
tRNA-nucleotidyltransferase 1, mitochondrial	TRNT1_HUMAN	TRNT1	50,112	100.00%	2	2	3	5.76%	EIGALLQQLR	95.0%	51.3	18.1	2	0	0	2	1,140.67
									LQSPEFQSLFTEGLK	95.0%	58.4	21.2	1	0	0	2	1,723.89
Ras-related protein Rab-5C	RAB5C_HUMAN	RAB5C	23,465	100.00%	3	3	6	17.10%	GVDLQENNPASR	95.0%	73.8	21.7	2	0	0	2	1,299.63
									LVLLGESAVGK	95.0%	43.1	20.1	2	0	0	2	1,085.66
									QASPNIVIALAGNK	95.0%	43.5	18.2	2	0	0	2	1,395.80
Ras-related C3 botulinum toxin substrate 1	RAC1_HUMAN	RAC1	21,433	100.00%	2	2	5	12.50%	LTPITYPQGLAMAK	95.0%	55.1	20.8	4	0	0	2	1,519.82
									YLECSALTQR	95.0%	46.2	21.9	1	0	0	2	1,240.60
6-phosphofructokinase type C	K6PP_HUMAN	PFKP	85,579	100.00%	3	3	4	6.51%	AIGVLTSGGDAQGMNAAVR	95.0%	80.9	22.8	1	0	0	2	1,803.90
									EIGWTDVGGWTGQGGSILGTK	95.0%	100.0	22.0	2	0	0	2	2,119.05
									NVIFQPVAELK	95.0%	34.8	20.3	1	0	0	2	1,257.72
Annexin A2	ANXA2_HUMAN	ANXA2	38,588	100.00%	28	37	986	69.90%	AEDGSVIDYELIDQDAR	95.0%	101.0	21.0	31	2	0	2	1,908.88
									AYTNFDAER	95.0%	66.7	19.4	14	0	0	2	1,086.49
									AYTNFDAERDALNIETAIK	95.0%	72.7	22.1	38	34	0	2	2,155.07
									DALNIETAIK	95.0%	74.5	24.5	23	0	0	2	1,087.60
									DIISDTSGDFR	95.0%	75.3	20.8	6	0	0	2	1,225.57
									DIISDTSGDFRK	95.0%	57.6	22.4	6	0	0	2	1,353.67
									DLYDAGVKR	95.0%	47.6	23.2	4	0	0	2	1,036.54
									ELASALK	95.0%	37.7	23.5	1	0	0	2	731.43
									GLGTDEDSLIEIICSR	95.0%	98.8	22.4	74	0	0	2	1,777.86
									GVDEVTIVNILTNR	95.0%	105.0	21.3	116	30	0	2	1,542.85
									LMVALAK	95.0%	44.8	24.2	12	0	0	2	745.46
									LSLEGDHSTPPSAYGSVK	95.0%	77.1	22.5	15	3	0	2	1,844.90
									LYDSMK	95.0%	34.8	18.9	1	0	0	2	756.36
									QDIAFAYQR	95.0%	82.0	22.3	79	0	0	2	1,111.55
									RAEDGSVIDYELIDQDAR	95.0%	99.0	22.3	22	82	0	2	2,064.98
									SALSGHLETVILGLLK	95.0%	89.0	11.1	36	36	0	2	1,650.98
									SEVDMLK	95.0%	35.4	22.8	4	0	0	2	837.40

									SLYYYIQQDTK	95.0%	74.2	23.0	86	0	0	2	1,421.70
									SLYYYIQQDTKGDYQK	95.0%	71.4	21.7	3	2	0	2	2,012.96
									SVPHLQK	95.0%	30.7	19.2	1	0	0	2	808.47
									SYSPYDMLESIR	95.0%	76.7	21.1	28	0	0	2	1,460.67
									SYSPYDMLESIRK	95.0%	64.6	23.0	21	0	0	2	1,588.77
									TDLEKDIISDTSGDFR	95.0%	87.7	22.2	16	25	0	2	1,811.87
									TKGVDEVTIVNILTNR	95.0%	92.7	18.1	6	10	0	2	1,771.99
									TNQELQEINR	95.0%	69.0	23.6	71	0	0	2	1,244.62
									TPAQYDASELK	95.0%	75.0	23.1	39	0	0	2	1,222.60
									VYKEMYK	95.0%	38.2	22.8	4	0	0	2	976.48
									WISIMTER	95.0%	37.0	22.2	5	0	0	2	1,051.52
Profilin-2	PROF2_HUMAN	PFN2	15,028	99.50%	2	2	3	19.30%	EGFFTNGLTLGAK	95.0%	35.5	22.6	1	0	0	2	1,354.70
									SQGGEPTYNVAVGR	95.0%	58.2	22.2	2	0	0	2	1,434.70
Aldo-keto reductase family 1 member C1	AK1C1_HUMAN	AKR1C1	36,771	100.00%	4	5	27	45.50%	AIDGLNR	95.0%	37.0	22.6	2	0	0	2	758.42
									EEPWVDPNSPVLLEDPVLCALAK	95.0%	29.4	21.3	0	1	0	2	2,591.31
									HIDSAHLYNNEEQVGLAIR	95.0%	82.9	22.0	0	56	21	2	2,179.09
									IADGSVKR	95.0%	45.2	23.3	1	0	0	2	845.48
									LAIEAGFR	95.0%	46.2	23.1	52	0	0	2	876.49
									LNDGHFMPVLGFGTYAPAEVPK	95.0%	59.8	21.7	3	16	0	2	2,360.18
									QLEMILNKPGLK	95.0%	56.0	17.6	9	0	0	2	1,383.80
									QNVQVFEFQLTSEEMK	95.0%	91.6	21.3	5	0	0	2	1,972.93
									REDIFYTSK	95.0%	38.6	21.6	15	0	0	2	1,158.58
									RQLEMILNKPGLK	95.0%	28.1	14.5	0	2	0	2	1,539.90
									RTPALIALR	95.0%	37.6	10.0	2	0	0	2	1,010.65
									SIGVSNFNR	95.0%	53.8	22.6	6	0	0	2	993.51
									TPALIALR	95.0%	70.5	14.8	31	0	0	2	854.55
									YQLQR	95.0%	30.5	22.5	2	0	0	2	707.38
Actin-related protein 2/3 complex subunit 5	ARPC5_HUMAN	ARPC5	16,303	99.50%	2	2	3	16.60%	ALAAGGVGSIVR	95.0%	83.8	19.8	2	0	0	2	1,070.63
									QGNMTAALQAALK	95.0%	56.9	23.0	1	0	0	2	1,316.70
Aldo-keto reductase family 1 member B10	AK1BA_HUMAN	AKR1B10	36,003	100.00%	16	17	117	55.70%	ACNVLQSSHLEDYPFDAEY	95.0%	53.9	17.4	2	0	0	2	2,257.97
									AKMPIVGLGTWK	95.0%	29.8	19.2	0	1	0	2	1,300.75
									ALGVSNFSHFQIEK	95.0%	83.6	23.0	10	2	0	2	1,576.81
									ATFLDAWEAMEELVDEGLVK	95.0%	101.0	21.5	2	0	0	2	2,282.09
									DDKGNAIGGK	95.0%	35.9	22.0	2	0	0	2	974.49
									HIDCAYVYQNEHEVGEAIQEK	95.0%	38.9	20.3	0	1	0	2	2,532.15
									IKEIAAK	95.0%	40.3	19.8	2	0	0	2	772.49
									IVENIQVFDFK	95.0%	99.3	21.2	24	0	0	2	1,351.73
									LLNKPGLK	95.0%	32.4	11.5	2	0	0	2	882.58
									LSDEEMATILSFNR	95.0%	92.3	22.4	23	0	0	2	1,625.78
									MPIVGLGTWK	95.0%	59.7	21.4	11	0	0	2	1,101.61
									NVIVIPK	95.0%	41.1	13.6	2	0	0	2	782.51
									REDLFIVSK	95.0%	50.8	21.1	11	0	0	2	1,106.62
									SGDDLFPK	95.0%	60.7	22.3	10	0	0	2	878.43
									TAAQVLIR	95.0%	61.2	21.0	11	0	0	2	871.54
									VAIDAGYR	95.0%	33.0	23.8	1	0	0	2	864.46
Desmocollin-3	DSC3_HUMAN	DSC3	99,953	100.00%	11	11	29	10.50%	AVALSDK	95.0%	40.4	25.8	1	0	0	2	703.40
									AVALSDKK	95.0%	35.3	21.9	2	0	0	2	831.49
									EVTVLLEHQK	95.0%	53.3	19.2	4	0	0	2	1,195.67
									GVDKEPLNLFYIER	95.0%	31.6	21.7	0	1	0	2	1,692.90
									NAGFQEYTIPITVK	95.0%	74.8	21.6	2	0	0	2	1,580.83
									SFTIWLSDK	95.0%	52.5	21.6	5	0	0	2	1,096.57

									SSDPDFR	95.0%	49.2	20.1	2	0	0	2	823.36
									VILNVPSK	95.0%	36.7	14.0	2	0	0	2	869.55
									VILNVPSKLEADK	95.0%	54.3	16.6	2	0	0	2	1,425.83
									VLNDGSVYTAR	95.0%	91.4	23.6	4	0	0	2	1,194.61
									VNLEECFR	95.0%	38.2	22.2	4	0	0	2	1,066.50
60S ribosomal protein L12	RL12_HUMAN	RPL12	17,801	100.00%	5	5	13	38.20%	CTGGEVGATSALAPK	95.0%	94.4	22.6	2	0	0	2	1,418.70
									HSGNITFDEIVNIAR	95.0%	40.3	22.1	1	0	0	2	1,685.86
									IGPLGLSPK	95.0%	50.5	11.5	4	0	0	2	881.55
									KVGDDIAK	95.0%	40.0	23.4	2	0	0	2	845.47
									QAQIEVVPSASALIIK	95.0%	49.5	12.8	4	0	0	2	1,666.97
Deleted in malignant brain tumors 1 protein	DMBT1_HUMAN	DMBT1	260,708	100.00%	4	4	31	1.78%	FGQGSGPIVLDDVR	95.0%	93.7	22.5	26	0	0	1	1,459.75
									LVNGDGR	95.0%	37.0	21.9	1	0	0	2	730.38
									LVNGGDR	95.0%	37.0	21.9	1	0	0	2	730.38
									QLGCGWATSAPGNAR	95.0%	43.9	21.1	3	0	0	2	1,545.72
Follistatin	FST_HUMAN	FST	37,989	100.00%	9	9	248	28.80%	CKEQPELEVQYQGR	95.0%	38.9	24.6	0	1	0	2	1,763.84
									CVCAPDCSNITWK	95.0%	61.2	17.2	5	0	0	2	1,610.68
									EQPELEVQYQGR	95.0%	81.1	22.2	10	0	0	2	1,475.71
									GPVCGLDGK	95.0%	33.6	21.6	1	0	0	2	902.44
									GRCSLCDELCPDSK	95.0%	34.7	16.6	12	0	0	2	1,525.65
									LSTSWTEEDVNDNTLFK	95.0%	110.0	21.6	174	0	0	2	1,998.93
									SIGLAYEGK	95.0%	57.8	21.3	41	0	0	2	937.50
									TELSKEECCSTGR	95.0%	32.3	16.9	1	0	0	2	1,556.67
									TYRNECALLK	95.0%	33.0	22.6	0	3	0	2	1,267.65
Inositol monophosphatase 1	IMPA1_HUMAN	IMPA1	30,171	100.00%	2	2	4	8.66%	SLLVTELGSSR	95.0%	64.5	21.9	2	0	0	2	1,161.65
									SSPVDLVTATDQK	95.0%	74.2	24.0	2	0	0	2	1,360.70
Actin-related protein 2/3 complex subunit 1B	ARC1B_HUMAN	ARPC1B	40,932	100.00%	3	4	19	11.30%	ASSEGGTAAGAGLDSLHK	95.0%	95.1	21.9	6	2	0	2	1,628.79
									EVEERPAPTPWGSK	95.0%	49.1	22.4	1	0	0	2	1,582.79
									TWKPTLVILR	95.0%	48.2	14.8	0	10	0	2	1,226.76
Adapter molecule crk	CRK_HUMAN	CRK	33,813	100.00%	5	5	11	25.30%	ALFDFNGNDEEDLPFK	95.0%	83.1	20.8	2	0	0	2	1,870.85
									DSSTSPGDYVLSVSENSR	95.0%	74.0	19.2	2	0	0	2	1,899.86
									IGDQEFDSLPALLEFYK	95.0%	91.9	22.5	2	0	0	2	1,984.99
									IHYLDTTTLIEPVSR	95.0%	37.3	21.0	0	1	0	2	1,757.94
									QEAVALLQGQR	95.0%	73.4	20.4	4	0	0	2	1,212.67
Proline synthase co-transcribed bacterial homolog protein	PROSC_HUMAN	PROSC	30,326	100.00%	7	7	20	32.40%	APLEVAQEH	95.0%	33.3	23.3	1	0	0	2	993.50
									HGLPPSETIAIVEHINAK	95.0%	34.5	20.0	0	2	0	2	1,926.05
									IGSTIFGER	95.0%	63.7	22.0	2	0	0	2	979.52
									LMAVPNLFMLETVDSVK	95.0%	63.8	21.8	5	0	0	2	1,938.99
									TFGENYVQELLEK	95.0%	79.1	23.3	7	0	0	2	1,569.78
									TKPADMVIEAYGHGQR	95.0%	62.7	22.0	0	1	0	2	1,788.87
									VQQAVAR	95.0%	45.2	18.9	2	0	0	2	771.45
Nucleolin	NUCL_HUMAN	NCL	76,598	100.00%	28	33	416	35.20%	ALELTGLK	95.0%	58.9	20.7	20	0	0	2	844.51
									ALVATPGKK	95.0%	32.0	16.3	1	0	0	2	884.56
									EALNSCNKR	95.0%	38.2	21.8	1	0	0	2	1,091.53
									EAMEDGEIDGNK	95.0%	72.2	14.1	13	0	0	2	1,323.54
									EAMEDGEIDGNKVTLDWAKPK	95.0%	48.7	21.3	0	4	0	2	2,362.12
									EVFEDAAEIR	95.0%	70.9	23.5	8	0	0	2	1,178.57
									FGYVDFESAEDLEK	95.0%	97.9	19.2	13	0	0	2	1,648.74
									GFGFVDFNSEEDAK	95.0%	81.0	17.4	59	0	0	2	1,561.68
									GIAYIEFK	95.0%	45.0	20.2	18	0	0	2	940.51
									GLSEDTTEETLK	95.0%	71.5	22.7	13	0	0	2	1,322.63
									GLSEDTTEETLKESFDGSVR	95.0%	94.7	21.3	5	22	0	2	2,200.03

									IVTDRETGSSK	95.0%	49.8	22.9	1	0	0	2	1,192.62
									KFGYVDFESAEDLEK	95.0%	77.6	22.1	2	0	0	2	1,776.83
									KGAAIPAK	95.0%	40.9	18.1	1	0	0	2	755.48
									NDLAVVDVR	95.0%	70.8	21.6	24	0	0	2	1,000.54
									NSTWSGESK	95.0%	53.8	20.4	12	0	0	2	995.44
									QGTEIDGR	95.0%	58.5	22.0	19	0	0	2	875.42
									QKVEGTEPTTAFNLFVGNLNFNK	95.0%	49.0	20.8	0	2	0	2	2,568.31
									SISLYYTGEK	95.0%	52.0	23.7	19	0	0	2	1,160.58
									TEADAEKTFEEK	95.0%	65.8	21.4	9	4	0	2	1,397.64
									TGISDVFAK	95.0%	55.6	20.9	34	0	0	2	937.50
									TLVLSNLSYSATEETLQEVFEK	95.0%	129.0	21.1	17	3	0	2	2,501.27
									VAVATPAK	95.0%	47.7	15.4	3	0	0	2	756.46
									VAVATPAKK	95.0%	33.5	16.3	1	0	0	2	884.56
									VEGTEPTTAFNLFVGNLNFNK	95.0%	109.0	21.6	42	12	0	2	2,312.16
									VTLDWAKPK	95.0%	48.6	21.4	3	0	0	2	1,057.60
									VTQDELK	95.0%	40.7	24.5	12	0	0	2	832.44
									VTQDELKEVFEDAAEIR	95.0%	94.3	21.9	7	12	0	2	1,991.99
Triosephosphate isomerase	TPIS_HUMAN	TPI1	26,651	100.00%	19	30	637	89.60%	DCGATWVVLGHSER	95.0%	88.2	21.5	15	12	0	2	1,586.74
									EAGITEK	95.0%	32.2	24.5	1	0	0	2	747.39
									ELASQPDVDGFLVGGASLKPEFVDIINAK	95.0%	67.6	18.4	0	51	0	2	3,029.58
									FFVGGNWK	95.0%	32.3	20.7	4	0	0	2	954.48
									HVFGESDELIGQK	95.0%	89.2	22.9	38	37	0	2	1,458.72
									IAVAAQNCYK	95.0%	58.8	23.2	18	0	0	2	1,137.57
									IIYGGSVTGATCK	95.0%	115.0	22.5	16	0	0	2	1,326.67
									KFFVGGNWK	95.0%	38.0	21.3	3	2	0	2	1,082.58
									KQSLGELIGTLNAAK	95.0%	99.7	16.5	21	22	0	2	1,542.89
									QSLGELIGTLNAAK	95.0%	102.0	21.0	28	1	0	2	1,414.79
									RHVFGESDELIGQK	95.0%	64.9	22.5	3	8	0	2	1,614.82
									SNVSDAVAQSTR	95.0%	98.2	22.7	42	0	0	2	1,234.60
									TATPQQAQEVHEK	95.0%	77.8	22.7	64	5	0	2	1,466.72
									VAHALAEGLGVIACIGEK	95.0%	119.0	20.2	30	45	0	2	1,807.97
									VIADNVKDWSK	95.0%	57.7	23.4	11	0	0	2	1,274.67
									VPADTEVVCAPPTAYIDFAR	95.0%	78.0	21.8	6	2	0	2	2,192.07
									VTNGAFTGEISPGMIK	95.0%	99.0	22.4	14	5	0	2	1,621.83
									VVFEQTK	95.0%	45.1	21.9	5	0	0	2	850.47
									VVLAYEPVWAIGTGK	95.0%	112.0	19.4	124	4	0	2	1,602.89
RNA-binding protein FUS	FUS_HUMAN	FUS	53,408	100.00%	5	6	11	12.70%	AAIDWFDGK	95.0%	62.3	22.3	2	0	0	2	1,022.49
									AAIDWFDGKEFSGNPIK	95.0%	37.3	22.6	1	1	0	2	1,894.93
									APKPDGPGGGPGGSHMGGNYGDDR	95.0%	28.0	16.8	0	0	2	2	2,268.97
									GEATVSFDDPPSAK	95.0%	76.7	21.7	3	0	0	2	1,420.66
									TGQPMINLYTDR	95.0%	41.0	22.7	2	0	0	2	1,424.68
Heterogeneous nuclear ribonucleoproteins C1/C2	HNRPC_HUMAN	HNRNPC	33,653	100.00%	9	10	94	30.40%	AAVAGEDGR	95.0%	53.5	20.6	2	0	0	2	845.41
									GFAFVQYVNER	95.0%	85.8	22.3	12	0	0	2	1,329.66
									LKGDDLQAIK	95.0%	37.5	21.3	1	0	0	2	1,100.63
									MIAGQVLDINLAAEPK	95.0%	99.1	19.7	16	0	0	2	1,682.92
									NDKSEEEQSSSSVK	95.0%	72.4	19.8	6	0	0	2	1,553.69
									QKVDSLLENLEK	95.0%	54.0	21.3	4	1	0	2	1,415.77
									VDSLLENLEK	95.0%	48.2	24.0	4	0	0	2	1,159.62
									VFIGNLNTLVVK	95.0%	85.2	13.6	46	0	0	2	1,316.79
									VPPPPPIAR	95.0%	32.4	18.1	2	0	0	2	943.57
Protocadherin-1	PCDH1_HUMAN	PCDH1	114,726	100.00%	10	11	114	11.60%	DMNDNAPTIEIR	95.0%	76.7	20.3	16	0	0	2	1,404.64

									EQQSTYTFQLK	95.0%	42.1	21.6	5	0	0	2	1,372.68
									GLFTISPETGEIQVK	95.0%	59.7	22.0	13	0	0	2	1,618.87
									LEVGAPYLR	95.0%	32.1	21.8	6	0	0	2	1,017.57
									NTGLITVQGPVDREDLSTLR	95.0%	59.3	19.6	2	17	0	2	2,184.16
									TGDIFTTETSIDR	95.0%	81.5	22.3	2	0	0	2	1,455.70
									VQDGGSPPR	95.0%	52.3	21.1	13	0	0	2	912.45
									VTVLDTNDNAPK	95.0%	89.0	22.9	20	0	0	2	1,286.66
									WDSYDLTIK	95.0%	32.3	23.2	1	0	0	2	1,140.56
									YFLQTTTPLDYEK	95.0%	60.6	22.8	19	0	0	2	1,618.80
Poliovirus receptor	PVR_HUMAN	PVR	45,284	100.00%	2	3	32	6.00%	VLAKPQNTAEVQK	95.0%	60.2	18.8	16	3	0	2	1,425.81
									VQLTGEPVPMAR	95.0%	59.6	21.2	13	0	0	2	1,297.69
S-adenosylmethionine synthase isoform type-2	METK2_HUMAN	MAT2A	43,643	100.00%	6	7	25	16.70%	AAVDYQK	95.0%	35.8	22.5	3	0	0	2	794.40
									DSFPWEVPK	95.0%	30.5	22.3	1	0	0	2	1,104.54
									FVIGGPQGDAGLTGR	95.0%	88.8	22.7	6	0	0	2	1,444.76
									HIGYDDSSK	95.0%	43.1	19.8	6	0	0	2	1,021.46
									TQVTVQYMQDR	95.0%	60.7	22.1	2	0	0	2	1,384.65
									YLDEDTIYHLQPSGR	95.0%	61.5	22.1	4	3	0	2	1,806.87
Filaggrin-2	FILA2_HUMAN	FLG2	248,034	100.00%	3	3	24	1.71%	FSNSSSSNEFSK	95.0%	71.1	16.1	22	0	0	2	1,320.57
									HQEEESETEEDEEDTPGHK	95.0%	62.0	10.0	0	1	0	2	2,254.89
									QDGECGTLSK	95.0%	32.2	18.2	1	0	0	2	1,094.48
Putative pre-mRNA-splicing factor ATP-dependent RNA helicase	DHX15_HUMAN	DHX15	90,917	99.90%	2	2	6	3.14%	EVDDLGPEVGDIK	95.0%	49.4	22.4	1	0	0	2	1,385.68
									TLATDILMGVLK	95.0%	60.7	19.0	5	0	0	2	1,290.73
Aldehyde dehydrogenase family 1 member A3	AL1A3_HUMAN	ALDH1A3	56,091	100.00%	15	19	60	35.20%	ANSTDYGLTAAVFTK	95.0%	112.0	23.1	3	0	0	2	1,558.78
									AVEAAQVAFQR	95.0%	56.3	23.9	2	0	0	2	1,189.63
									EAGFPPGVVNIVPGFGPTVGAAISSHPQINK	95.0%	36.7	18.8	0	4	0	2	3,057.62
									EEIFGPVQPILK	95.0%	54.8	18.8	2	0	0	2	1,369.77
									ELGEYALAEYTEVK	95.0%	71.8	22.3	4	0	0	2	1,614.79
									GLFIKPTVFSEVTDNMR	95.0%	43.8	21.4	1	3	0	2	1,970.01
									IAFTGSTEVGK	95.0%	52.9	21.1	4	0	0	2	1,109.58
									IAKEEIFGPVQPILK	95.0%	65.7	14.0	2	2	0	2	1,681.99
									IFINNEWHESK	95.0%	60.8	21.9	2	0	0	2	1,416.69
									ILELIESGK	95.0%	36.5	19.3	2	0	0	2	1,001.59
									ILELIESGKK	95.0%	51.0	18.2	2	2	0	2	1,129.68
									KRPVGDPFDVK	95.0%	33.9	22.0	0	2	0	2	1,257.70
									LLHQLADLVER	95.0%	47.0	17.9	2	2	0	2	1,306.75
									TEQGPQIDQK	95.0%	52.2	21.5	5	0	0	2	1,143.56
									VFVEEQVYSEFVR	95.0%	79.6	22.3	14	0	0	2	1,630.81
Alpha-actinin-4	ACTN4_HUMAN	ACTN4	104,839	100.00%	61	80	2114	70.70%	AGTQIENIDEDFR	95.0%	72.7	20.7	5	0	0	2	1,507.70
									AGTQIENIDEDFRDGLK	95.0%	87.9	22.0	11	16	0	2	1,920.93
									AIMTYVSSFYHAFSGAQK	95.0%	87.6	22.3	31	13	0	2	2,023.96
									ALDFIASK	95.0%	64.7	21.2	17	0	0	2	864.48
									ASFNHFDKDHGGALGPEEFK	95.0%	45.0	20.3	0	17	25	2	2,203.02
									ASIHEAWTDGK	95.0%	48.0	20.6	20	0	0	2	1,214.58
									CQLEINFNTLQTK	95.0%	92.7	22.9	4	0	0	2	1,608.81
									DDPVTNLNNAFEVAEK	95.0%	86.6	22.0	14	0	0	2	1,775.85
									DGLAFNALIHR	95.0%	60.7	21.6	26	4	0	2	1,226.66
									DHALLEEQSK	95.0%	59.8	23.2	8	0	0	2	1,169.58
									DHGGALGPEEFK	95.0%	44.6	21.1	2	0	0	2	1,256.59
									DLLLDPAWEK	95.0%	50.4	22.0	7	0	0	2	1,199.63
									DYETATLSDIK	95.0%	70.9	21.7	22	0	0	2	1,255.61
									EAILAIHK	95.0%	55.5	15.2	14	0	0	2	894.54

									EGLLLWCQR	95.0%	48.2	23.4	26	0	0	2	1,174.60
									ELPPDQAEYCIAR	95.0%	73.8	21.1	14	0	0	2	1,561.73
									ETTDTDTADQVIASFK	95.0%	114.0	21.2	78	3	0	2	1,741.81
									FAIQDISVEETSAK	95.0%	104.0	22.3	225	0	0	2	1,537.78
									GISQEQMQEFR	95.0%	72.7	21.0	48	0	0	2	1,368.62
									GYEEWLLNEIR	95.0%	101.0	23.5	73	0	0	2	1,421.71
									HEAFESDLAAHQDR	95.0%	51.6	20.1	0	4	0	2	1,625.73
									HRDYETATLSDIK	95.0%	71.0	22.8	6	34	0	2	1,548.77
									HRPELIEYDK	95.0%	41.8	22.3	0	44	0	2	1,299.67
									HRPELIEYDKLR	95.0%	39.2	20.4	0	2	0	2	1,568.86
									HTNYTMEHIR	95.0%	41.5	20.6	0	15	0	2	1,301.61
									IAESNHIK	95.0%	56.8	20.1	13	0	0	2	911.50
									ICDQWDALGSLTHSR	95.0%	97.7	20.7	8	27	0	2	1,758.82
									ISIEMNGTLEDQLSHLK	95.0%	42.6	22.4	0	9	0	2	1,943.98
									KDDPVTNLNNAFEVAEK	95.0%	109.0	21.8	29	66	0	2	1,903.94
									KHEAFESDLAAHQDR	95.0%	48.4	21.5	0	6	74	2	1,753.83
									LASDLLEWIR	95.0%	101.0	22.0	38	0	0	2	1,215.67
									LDHLAEK	95.0%	45.8	19.3	13	0	0	2	825.45
									LMLLLEVISGER	95.0%	43.2	19.6	1	0	0	2	1,388.78
									LSGSNPYTTVTPQIINSK	95.0%	104.0	21.3	57	4	0	2	1,920.01
									LSNRPAFMPSEGK	95.0%	57.7	23.1	19	31	0	2	1,433.72
									LVSIGAEEIVDGNAK	95.0%	114.0	22.0	180	0	0	2	1,514.81
									MAPYQGPDAVPGALDYK	95.0%	102.0	22.2	53	0	0	2	1,808.85
									MEEIGR	95.0%	30.8	23.0	3	0	0	2	734.35
									MLDAEDIVNTARPDEK	95.0%	94.6	22.0	20	38	0	2	1,832.87
									MTLGMIWTIILR	95.0%	97.4	20.2	49	0	0	2	1,479.81
									MVSDINNGWQHLEQAEK	95.0%	83.7	21.1	8	6	0	2	2,014.93
									NFITAEELR	95.0%	47.7	23.3	7	0	0	2	1,092.57
									NVNVQNFHISWK	95.0%	59.0	22.2	8	0	0	2	1,485.76
									QFASQANVVGPWIQTK	95.0%	111.0	21.9	39	2	0	2	1,773.93
									QGEAEFNR	95.0%	67.1	21.5	5	0	0	2	950.43
									QLEAIDQLHLEYAK	95.0%	86.0	21.7	52	4	0	2	1,670.88
									QQSNEHLR	95.0%	30.4	20.8	1	0	0	2	1,011.50
									RQFASQANVVGPWIQTK	95.0%	50.7	20.6	0	4	0	2	1,930.03
									STLPDADR	95.0%	34.4	21.7	1	0	0	2	874.43
									STLPDADRER	95.0%	42.1	22.3	7	17	0	2	1,159.57
									TFTAWCNSHLR	95.0%	33.2	22.0	1	0	0	2	1,392.65
									TINEVENQILTR	95.0%	86.4	22.5	61	0	0	2	1,429.77
									TIPWLEDR	95.0%	30.7	22.7	1	0	0	2	1,029.54
									TIPWLEDRVPQK	95.0%	39.9	20.7	9	0	0	2	1,481.81
									TIQEMQQK	95.0%	39.8	22.5	4	0	0	2	1,021.50
									VEQIAAIAQELNELDYYDSHNVNTR	95.0%	107.0	20.7	0	44	3	2	2,905.40
									VGWEQLLTTIAR	95.0%	92.8	20.5	180	0	0	2	1,386.77
									VLAGDKNFITAEELR	95.0%	117.0	20.5	12	21	0	2	1,675.90
									VLAVNQENEHLMEDYEK	95.0%	88.6	21.8	8	17	0	2	2,060.96
									VQQLVPK	95.0%	53.3	15.4	12	0	0	2	811.50
									YLDIPK	95.0%	35.7	19.0	14	0	0	2	748.42
Splicing factor, arginine/serine-rich 2	SFRS2_HUMAN	SFRS2	25,459	100.00%	3	3	33	14.50%	DAEDAMDAMDGAVLDGR	95.0%	122.0	13.8	15	0	0	2	1,783.71
									VDNLTYR	95.0%	31.0	23.1	1	0	0	2	880.45
									VGDVYIPR	95.0%	65.1	22.8	17	0	0	2	918.51
3-hydroxyisobutyrate	3HIDH_HUMAN	HIBADH	35,312	100.00%	7	8	18	31.80%	DFSSVFQFLR	95.0%	36.7	22.7	1	0	0	2	1,245.63

dehydrogenase, mitochondrial									DLGLAQDSATSTK	95.0%	67.3	22.7	2	0	0	2	1,306.65
									EFQDAGEQVVSSPADVAEK	95.0%	90.3	20.0	2	0	0	2	2,005.94
									GSLLIDSSTIDPAVSK	95.0%	48.3	21.0	2	0	0	2	1,602.86
									MGAVFMDAPVSGGVGAAR	95.0%	87.7	21.4	5	0	0	2	1,724.81
									SPILLGSLAHQIYR	95.0%	51.0	18.5	2	1	0	2	1,567.90
									TPVGFIGLGNMGNPMAK	95.0%	63.3	22.2	3	0	0	2	1,735.85
Bone morphogenetic protein 1	BMP1_HUMAN	BMP1	111,231	100.00%	13	13	73	15.70%	AAAFLGDIALDEEDLR	95.0%	88.4	22.3	3	0	0	2	1,718.86
									AGFAVNFFK	95.0%	63.7	21.6	18	0	0	2	1,000.53
									DYGHIQSPNYPDDYRPSK	95.0%	20.4	20.2	0	0	1	2	2,151.97
									ENIQPGQEYNFLK	95.0%	72.3	22.8	12	0	0	2	1,579.78
									FVSDGSINK	95.0%	31.3	22.5	2	0	0	2	966.49
									FYSDNSVQR	95.0%	49.6	20.0	2	0	0	2	1,115.51
									GDIAQAR	95.0%	45.5	21.9	6	0	0	2	730.38
									GGGPQAISIGK	95.0%	59.6	18.9	2	0	0	2	984.55
									GIFLDTIVPK	95.0%	46.8	18.2	10	0	0	2	1,102.65
									KPEPVLATGSR	95.0%	39.2	19.9	2	0	0	2	1,154.65
									LPEPIVSTDSR	95.0%	60.0	22.3	12	0	0	2	1,213.64
									YCGSGPPEEVYSAGDSVLVK	95.0%	111.0	21.0	2	0	0	2	2,113.98
									YCGYEKPDDIK	95.0%	42.3	19.1	1	0	0	2	1,387.62
UPF0568 protein C14orf166	CN166_HUMAN	C14orf166	28,051	100.00%	3	3	6	15.60%	AGVMALANLLQIQR	95.0%	58.5	19.5	3	0	0	2	1,513.85
									LTQDAVAK	95.0%	30.4	23.4	1	0	0	2	845.47
									NAEPLINLDVNNPDFK	95.0%	74.2	21.9	2	0	0	2	1,812.91
Peptidyl-prolyl cis-trans isomerase F,	PPIF_HUMAN	PPIF	22,022	99.50%	2	2	4	18.40%	FPDENFTLK	95.0%	42.8	22.5	2	0	0	2	1,110.55
mitochondrial									GSGDPSSSSSSGNPLVYLDVDANGKPLGR	95.0%	73.8	20.7	0	2	0	2	2,833.36
Tripeptidyl-peptidase 2	TPP2_HUMAN	TPP2	138,335	100.00%	2	2	4	1.84%	GTLTEAFPVLGGK	95.0%	56.4	21.1	2	0	0	2	1,289.71
									VPITAVIAAK	95.0%	37.5	7.8	2	0	0	2	982.63
Heterogeneous nuclear	HNRDL_HUMAN	HNRPDL	46,421	99.50%	2	2	2	10.00%	DLTEYLSR	95.0%	31.1	21.8	1	0	0	2	996.50
ribonucleoprotein D-like									FGEVVDCTIK	95.0%	55.7	22.7	13	0	0	2	1,167.57
									GFGFVLFK	95.0%	40.5	22.9	13	0	0	2	914.51
									VFVGGLSPDTSEEQIK	95.0%	35.2	22.6	1	0	0	2	1,705.87
Polypeptide N-	GALT2_HUMAN	GALNT2	64,715	100.00%	12	12	31	25.20%	AAEVWMDEYK	95.0%	44.1	19.1	2	0	0	2	1,257.55
acetylgalactosaminyltransferase 2									EIILVDDYSNDPEDGALLGK	95.0%	127.0	22.1	7	0	0	2	2,176.07
									FNQVESDKLR	95.0%	29.8	23.5	0	2	0	2	1,235.64
									FYFEELGK	94.8%	30.2	22.3	1	0	0	2	1,032.50
									GGFDWNLVFK	95.0%	43.6	21.7	2	0	0	2	1,182.59
									NFYYAAVPSAR	95.0%	53.8	21.9	2	0	0	2	1,258.62
									QHPYTFPGGSGTVFAR	95.0%	55.8	22.6	0	2	0	2	1,721.84
									QKWEQIEGNSK	95.0%	41.3	22.9	2	0	0	2	1,346.67
									TPMIAGGLFVMDK	95.0%	63.1	21.9	5	0	0	2	1,411.70
									VRGADAAQAK	95.0%	32.0	21.3	1	0	0	2	986.54
									WDYMTPEQR	95.0%	48.5	16.9	2	0	0	2	1,241.53
									WPDFNQEAYVGGTMVR	95.0%	61.7	20.6	3	0	0	2	1,885.85
Inositol-3-phosphate synthase 1	INO1_HUMAN	ISYNA1	61,050	100.00%	4	4	11	8.78%	APLVPPGSPVVNALFR	95.0%	32.4	15.8	2	0	0	2	1,633.94
									VFVGGDDFK	95.0%	31.2	21.6	1	0	0	2	983.48
									VGPVAATYPMLNK	95.0%	48.7	22.4	4	0	0	2	1,360.73
									VIVLWTANTER	95.0%	72.8	21.4	4	0	0	2	1,301.72
Translin	TSN_HUMAN	TSN	26,165	99.50%	2	4	6	15.40%	EAVTEILGIEPDREK	95.0%	59.4	21.5	1	2	0	2	1,698.89
									EILTLLQGVHQGAGFQDIPK	95.0%	75.8	17.9	1	2	0	2	2,164.18
SUMO-conjugating enzyme UBC9	UBC9_HUMAN	UBE2I	17,990	100.00%	3	4	10	20.30%	KDHPFGFVAVPTK	95.0%	47.1	21.4	2	4	0	2	1,442.78
									MLFKDDYPSSPPK	95.0%	81.1	22.7	2	0	0	2	1,540.74
									VEYEKR	95.0%	31.4	22.0	2	0	0	2	823.43

Cleavage and polyadenylation specificity factor subunit 6	CPSF6_HUMAN	CPSF6	59,193	99.50%	2	2	2	7.08%	AVSDASAGDYGSAIETLVTAISLIK	95.0%	51.5	19.6	1	0	0	2	2,452.28
									TPLSEAEFEEIMNR	95.0%	51.9	22.6	1	0	0	2	1,665.78
Complement factor D	CFAD_HUMAN	CFD	27,014	100.00%	3	4	23	22.10%	AVPHPDSQPDTIDHDLLLLQLSEK	95.0%	41.9	20.6	0	3	0	2	2,681.38
									RPDSLQHVLLPVLDR	95.0%	44.9	16.6	0	16	2	2	1,758.00
									VQVLLGAHSLSQPEPSK	95.0%	32.0	19.0	0	2	0	2	1,789.98
Lysyl oxidase homolog 4	LOXL4_HUMAN	LOXL4	84,463	100.00%	10	13	53	17.10%	AQEVVMSGVR	95.0%	65.3	23.7	7	0	0	2	1,091.55
									FSTQIYNLGR	95.0%	67.2	21.0	8	0	0	2	1,198.62
									GSWAEEPR	95.0%	42.4	17.6	4	0	0	2	931.43
									GYLSETVSNALGPQGR	95.0%	88.6	23.3	7	0	0	2	1,648.83
									LGQGLGPIHLSEVR	95.0%	48.7	19.5	2	8	0	2	1,475.83
									LVGPESKPEEGR	95.0%	41.1	22.0	1	2	0	2	1,297.68
									QHSPVTEGAVEVK	95.0%	49.5	22.2	2	2	0	2	1,380.71
									SADHMDWPYGYR	95.0%	34.2	15.9	0	1	0	2	1,497.62
									VVCGMLGFPSEVPVDSHYYR	95.0%	46.4	21.0	0	2	0	2	2,328.08
									YGQGEGPIWLDNVR	95.0%	70.6	22.0	7	0	0	2	1,603.79
Nucleosome assembly protein 1-like 4	NP1L4_HUMAN	NAP1L4	42,806	99.50%	2	2	3	10.10%	AAATAEEPDPK	95.0%	45.1	21.8	2	0	0	2	1,099.53
									FYEEVHDLER	95.0%	48.4	21.0	4	0	0	2	1,336.62
									LDNVPHTPSSYIETLPK	95.0%	28.3	21.0	0	1	0	2	1,910.99
Hexokinase-1	HXK1_HUMAN	HK1	102,470	100.00%	12	13	28	16.00%	ASGVEGADVVK	95.0%	35.8	23.3	2	0	0	2	1,031.54
									ATDCVGHDVVTLLR	95.0%	67.0	22.1	1	0	0	2	1,555.79
									GAALITAVGVR	95.0%	38.3	18.6	1	0	0	2	1,027.63
									GAAMVTAVAYR	95.0%	54.4	22.5	1	0	0	2	1,125.57
									GDFIALDLGGSSFR	95.0%	35.0	22.3	2	0	0	2	1,454.73
									GKFNTSDVSAIEK	95.0%	63.2	22.0	2	0	0	2	1,395.71
									HIDLVEGDEGR	95.0%	53.3	21.9	3	0	0	2	1,239.60
									ITPELLTR	95.0%	35.0	18.2	1	0	0	2	942.56
									LRTEASS	95.0%	37.3	22.5	1	0	0	2	763.40
									LVDEYSLNAGK	95.0%	74.7	23.2	2	0	0	2	1,208.62
									SANLVAATLGAILNR	95.0%	77.9	17.9	4	7	0	2	1,483.86
									TPDGTENGDFLALDLGGTNFR	95.0%	109.0	21.5	1	0	0	2	2,210.04
Thioredoxin reductase 1, cytoplasmic	TRXR1_HUMAN	TXNRD1	70,889	100.00%	9	11	19	17.60%	FLIATGERPR	95.0%	34.8	22.0	2	0	0	2	1,159.66
									KLMHQAALLGQALQDSR	95.0%	34.0	20.5	0	1	0	2	1,896.01
									LMHQAALLGQALQDSR	95.0%	39.7	21.3	0	2	0	2	1,767.92
									MIEAVQNHIGSLNWGYR	95.0%	44.5	22.2	0	1	0	2	2,003.98
									QFVPIKVEQIEAGTPGR	95.0%	62.3	18.4	2	1	0	2	1,869.02
									VEQIEAGTPGR	95.0%	58.0	22.1	2	0	0	2	1,156.60
									VMVLDFVTPTPLGTR	95.0%	99.9	21.0	4	0	0	2	1,661.89
									VVGFHVLGPNAGEVTQGFAAALK	95.0%	69.9	18.1	0	2	0	2	2,282.23
									VVYENAYGQFIGPHR	95.0%	61.4	22.5	1	1	0	2	1,749.87
UPF0027 protein C22orf28	CV028_HUMAN	C22orf28	55,192	99.50%	2	2	4	4.95%	QIGNVAALPGIVHR	95.0%	31.4	17.2	0	2	0	2	1,444.84
									SYNDELQFLEK	95.0%	35.8	20.5	2	0	0	2	1,385.66
Plakophilin-1	PKP1_HUMAN	PKP1	82,845	100.00%	3	3	5	5.09%	LLQSGNSDVVR	95.0%	48.2	22.2	2	0	0	2	1,187.64
									SPNQNVQQAAAGALR	95.0%	104.0	22.1	2	0	0	2	1,524.79
									VMGNQVFPEVTR	95.0%	32.2	22.7	1	0	0	2	1,376.70
Insulin-like growth factor-binding protein 5	IBP5_HUMAN	IGFBP5	30,552	100.00%	5	7	61	22.80%	EHEEPTTSEMAEETYSPK	95.0%	92.6	15.2	4	6	0	2	2,094.88
									FVGGAENTAHPR	95.0%	76.8	22.1	18	0	0	2	1,255.62
									HMEASLQELK	95.0%	44.7	21.4	11	0	0	2	1,201.59
									IISAPEMR	95.0%	37.7	23.6	6	0	0	2	932.49
									QDEEKPLHALLHGR	95.0%	42.8	20.9	0	7	9	2	1,642.87
Complement factor B	CFAB_HUMAN	CFB	85,515	100.00%	6	6	11	9.42%	EAGIPEFYDYDVALIK	95.0%	61.4	22.4	2	0	0	2	1,842.92
									EKLQDEDLGFL	95.0%	44.5	23.3	2	0	0	2	1,306.65

									QLNEINYEDHK	95.0%	33.7	21.9	1	0	0	2	1,402.66
									VASYGVKPR	95.0%	43.4	20.8	2	0	0	2	976.56
									VSEADSSNADWVTK	95.0%	94.2	20.6	2	0	0	2	1,508.69
									YGLVTYATYPK	95.0%	67.7	22.8	2	0	0	2	1,275.66
F-actin-capping protein subunit alpha-1	CAZA1_HUMAN	CAPZA1	32,905	100.00%	13	16	83	67.50%	DVQDSLTVSNEAQTAK	95.0%	98.7	23.4	6	0	0	2	1,705.82
									EASDPQPEEADGGLK	95.0%	90.3	19.5	14	0	0	2	1,542.69
									EGAAHAFAQYNMDQFTPVK	95.0%	30.5	20.6	0	3	0	2	2,140.98
									ESCDSALR	95.0%	43.4	18.3	1	0	0	2	937.41
									FITHAPPGEFNEVFNDVR	95.0%	51.1	21.7	2	4	0	2	2,089.01
									FTITPPTAQVVGVLK	95.0%	65.7	14.8	10	0	0	2	1,570.92
									IEGYEDQVLITEHGDLGNSR	95.0%	61.2	22.1	0	5	0	2	2,245.07
									IIENAENEYQTAISENYQTMSDTTFK	95.0%	61.3	18.5	0	3	0	2	3,056.37
									IQVHYYEDGNVQLVSHK	95.0%	40.7	22.2	0	11	4	2	2,029.01
									KEASDPQPEEADGGLK	95.0%	73.6	22.0	2	1	0	2	1,670.79
									LLLNNDNLLR	95.0%	72.5	19.2	14	0	0	2	1,197.70
									TIDGQQTIIACIESHQFQPK	95.0%	66.1	22.1	1	0	0	2	2,314.15
									VSDEEKVR	95.0%	59.3	24.0	2	0	0	2	961.50
HLA class I histocompatibility antigen, Cw-12 alpha chain	1C12_HUMAN	HLA-C	40,867	99.50%	2	2	12	16.40%	DGEDQTQDTELVETRPAGDGTFQK	95.0%	67.8	20.8	0	12	0	2	2,637.19
									FDSDAASPR	95.0%	63.5	22.1	6	0	0	2	965.43
									SWTAADTAAQITQR	95.0%	81.4	23.7	6	0	0	2	1,519.75
									WAAVVVPSGEEQR	95.0%	55.7	23.0	14	0	0	2	1,427.73
Cystatin-C	CYTC_HUMAN	CST3	15,781	100.00%	9	12	108	62.30%	AFCSFQIYAVPWQGTMTLSK	95.0%	57.9	21.2	6	0	0	2	2,351.12
									ALDFAVGEYNK	95.0%	70.7	22.9	38	0	0	2	1,226.61
									ASNDMYHSR	95.0%	73.0	18.1	8	0	0	2	1,080.45
									KQIVAGVNYFLDVELGR	95.0%	102.0	18.1	6	3	0	2	1,921.05
									LVGGPMDASVEEEGVR	95.0%	106.0	21.2	12	0	0	2	1,660.79
									LVGGPMDASVEEEGVRR	95.0%	37.8	21.3	5	4	0	2	1,800.89
									QIVAGVNYFLDVELGR	95.0%	115.0	20.7	19	3	0	2	1,792.96
									RALDFAVGEYNK	95.0%	38.5	22.9	2	0	0	2	1,382.71
									TQPNLDNCPFHDQPHLK	95.0%	71.2	21.7	2	0	0	2	2,060.96
Insulin-like growth factor-binding protein 6	IBP6_HUMAN	IGFBP6	25,304	100.00%	4	6	732	16.20%	APAVAEENPK	95.0%	73.9	20.9	393	0	0	2	1,025.53
									GAQTLYVPNCDHR	95.0%	43.4	20.8	6	3	0	2	1,530.71
									HLDSVLQQLQTEVYR	95.0%	129.0	21.8	238	91	0	2	1,828.96
									RHLDSVLQQLQTEVYR	95.0%	53.7	20.2	0	1	0	2	1,985.06
Small nuclear ribonucleoprotein Sm D2	SMD2_HUMAN	SNRPD2	13,509	100.00%	3	3	5	24.60%	EEEEFNTGPLSVLTQSVK	95.0%	89.0	22.1	2	0	0	2	2,006.99
									NNTQVLINCR	95.0%	62.3	23.4	2	0	0	2	1,231.62
									REEEEFNTGPLSVLTQSVK	95.0%	34.5	22.2	0	1	0	2	2,163.09
Mesothelin	MSLN_HUMAN	MSLN	68,970	100.00%	13	15	123	20.80%	ANVDLLPR	95.0%	31.3	17.6	2	0	0	2	897.52
									EIDESLIFYK	95.0%	56.9	22.4	9	0	0	2	1,256.64
									FVAESAEVLLPR	95.0%	55.6	21.6	4	0	0	2	1,330.74
									GLLPVLGQPIIR	95.0%	50.5	9.0	6	0	0	2	1,275.82
									GSLLSEADVR	95.0%	53.9	23.7	8	0	0	2	1,046.55
									IQSFLGGAPTEDLK	95.0%	84.0	23.3	30	0	0	2	1,475.77
									LLGPHVEGLK	95.0%	43.8	17.0	2	4	0	2	1,062.63
									LLGPHVEGLKAEER	95.0%	48.4	19.6	0	2	0	2	1,547.85
									LLPAALACWGVR	95.0%	40.0	20.5	3	0	0	2	1,326.74
									LRTDAVLPLTVAEVQK	95.0%	96.4	14.0	10	11	0	2	1,753.02
									QLDVLYPK	95.0%	47.3	21.1	6	0	0	2	975.55
									TDAVLPLTVAEVQK	95.0%	101.0	19.5	10	0	0	2	1,483.84
									VNAIPFTYEQLDVLK	95.0%	67.5	20.0	16	0	0	2	1,749.94
Drebrin-like protein	DBNL_HUMAN	DBNL	48,188	100.00%	4	4	12	13.50%	FQDVGPQAPVGSVYQK	95.0%	102.0	22.1	4	0	0	2	1,719.87

									VAGTGEGGLEEMVEELNSGK	95.0%	87.5	21.4	2	0	0	2	2,021.93
									VKDPNSGLPK	95.0%	45.1	20.0	0	2	0	2	1,054.59
									YQEQGGEASPQR	95.0%	65.5	23.3	4	0	0	2	1,349.61
Protein S100-A16	S10AG_HUMAN	S100A16	11,784	100.00%	4	4	6	36.90%	AADKLIQNLDANHDGR	95.0%	60.6	22.7	0	2	0	2	1,750.88
									AVIVLVENFYK	95.0%	65.6	17.4	2	0	0	2	1,294.74
									LIHEQEQQSSS	95.0%	39.6	20.9	1	0	0	2	1,285.60
									LIQNLDANHDGR	95.0%	28.5	22.6	0	1	0	2	1,365.69
Small nuclear ribonucleoprotein-associated proteins B and B'	RSMB_HUMAN	SNRPB	24,593	100.00%	2	2	11	9.58%	GENLVSMTVEGPPPK	95.0%	70.4	23.2	6	0	0	2	1,554.78
									VLGLVLLR	95.0%	52.1	6.0	5	0	0	2	882.61
Thrombospondin-1	TSP1_HUMAN	THBS1	129,364	100.00%	25	28	433	25.00%	AQGYSGLSVK	95.0%	37.2	20.2	2	0	0	2	1,009.53
									CEGSSVQTR	95.0%	55.8	20.3	1	0	0	2	1,023.45
									DHSGQVFSVVSNGK	95.0%	61.7	22.7	1	0	0	2	1,460.71
									DLASIAR	95.0%	41.6	25.5	4	0	0	2	745.42
									FQMIPLDPK	95.0%	44.9	24.0	11	0	0	2	1,088.58
									FTGSQPFGQGVEHATANK	95.0%	83.3	22.3	17	4	0	2	1,875.90
									FVFGTTPEDILR	95.0%	69.2	22.6	79	0	0	2	1,394.73
									FYVVMWK	95.0%	43.3	23.3	1	0	0	2	988.50
									GFLLLASLR	95.0%	61.6	13.8	8	0	0	2	989.61
									GGVNDNFQGVLQNVR	95.0%	93.9	23.5	56	1	0	2	1,616.81
									GPDPSSPAFR	95.0%	50.2	20.3	7	0	0	2	1,030.50
									GTLLALER	95.0%	59.8	21.8	8	0	0	2	872.52
									GTSQNDPNWVVR	95.0%	52.2	21.2	22	0	0	2	1,372.66
									IEDANLIPPVPDDKFQDLVDAVR	95.0%	56.0	20.0	0	26	0	2	2,579.34
									IMADSGPIYDK	95.0%	55.2	23.2	12	0	0	2	1,209.58
									IPESGGDNSVFDIFELTGAAR	95.0%	133.0	22.2	7	0	0	2	2,195.06
									KIMADSGPIYDK	95.0%	51.8	22.7	2	0	0	2	1,353.67
									LCNNPTPQFGGK	95.0%	62.0	22.6	15	0	0	2	1,332.64
									LVPNPDQK	95.0%	38.2	19.6	3	0	0	2	910.50
									MENAELDVPIQSVFTR	95.0%	117.0	22.6	51	0	0	2	1,864.91
									NALWHTGNTPGQVR	95.0%	55.9	22.3	5	1	0	2	1,550.78
									QVTQSYWDTNPTR	95.0%	97.2	22.1	21	0	0	2	1,595.75
									SITLFVQEDR	95.0%	53.9	23.3	2	0	0	2	1,207.63
									TIVTTLQDSIR	95.0%	66.4	21.2	64	0	0	2	1,246.70
									VTEENKELANELR	95.0%	53.7	23.1	2	0	0	2	1,544.79
Nucleoprotein TPR	TPR_HUMAN	TPR	267,271	100.00%	11	11	20	5.80%	ASTALSNEQQAR	95.0%	53.3	22.6	4	0	0	2	1,275.63
									EGVQGPLNVSLSEEGK	95.0%	41.7	22.4	1	0	0	2	1,642.83
									ELENANDLLSATK	95.0%	53.1	22.6	1	0	0	2	1,417.72
									LDELQASDVSVK	95.0%	63.4	24.4	2	0	0	2	1,303.67
									LQEQVTDLR	95.0%	59.3	23.1	2	0	0	2	1,101.59
									NIEELQQQNQR	95.0%	61.4	21.3	2	0	0	2	1,399.69
									NLQEQTVQLQSELSR	95.0%	96.3	21.8	1	0	0	2	1,772.91
									QTEEQVNDLKER	95.0%	33.7	22.2	1	0	0	2	1,488.73
									SQEQILEILR	95.0%	54.5	24.8	3	0	0	2	1,228.69
									TETMNVVMETNK	95.0%	64.6	20.0	1	0	0	2	1,428.64
									TLSSVQNEVQEALQR	95.0%	86.7	22.2	2	0	0	2	1,701.88
Tripartite motif-containing protein 29	TRI29_HUMAN	TRIM29	65,818	100.00%	2	2	3	3.06%	EQDAVDQVK	95.0%	39.4	21.9	1	0	0	2	1,031.50
									VIMDALDER	95.0%	33.0	23.6	2	0	0	2	1,077.53
Acidic leucine-rich nuclear phosphoprotein 32 family member B	AN32B_HUMAN	ANP32B	28,771	100.00%	3	4	40	28.70%	DISTLEPLK	95.0%	33.0	21.5	3	0	0	2	1,015.57
									IFGGLDMLAEK	95.0%	62.3	22.4	21	0	0	2	1,209.62
									KLELSENR	95.0%	46.1	23.4	6	0	0	2	988.54
									LKDISTLEPLK	95.0%	50.2	17.5	6	3	0	2	1,256.75

									LLPQLTYLDGYDR	95.0%	69.2	21.9	4	0	0	2	1,566.82
									LPNLTHLNLSGNK	95.0%	76.2	19.9	6	7	0	2	1,420.79
									SLDLFNCEVTNLNDYR	95.0%	103.0	20.7	5	0	0	2	1,972.91
NEDD8-activating enzyme E1 catalytic subunit	UBA3_HUMAN	UBA3	51,835	99.50%	2	2	2	5.83%	IQDFNDTFYR	95.0%	49.3	20.5	1	0	0	2	1,318.61
									LQEVLDYLTNSASLQMK	95.0%	86.5	22.0	1	0	0	2	1,969.00
Puromycin-sensitive aminopeptidase	PSA_HUMAN	NPEPPS	103,261	100.00%	22	25	86	28.10%	AFFESHPAPSAER	95.0%	39.4	21.1	2	0	0	2	1,445.68
									AGIISTVEVLK	95.0%	62.0	18.2	5	0	0	2	1,129.68
									ATLEEAR	95.0%	47.2	24.9	2	0	0	2	789.41
									DAESIHQYLLQR	95.0%	64.8	21.6	2	2	0	2	1,472.75
									DLSLPPVDR	94.6%	30.0	21.6	1	0	0	2	1,011.55
									DNWEELYNR	95.0%	49.9	19.8	2	0	0	2	1,238.54
									DYFNVPYPLPK	95.0%	47.8	22.7	6	0	0	2	1,352.69
									ETALLIDPK	95.0%	34.1	20.5	2	0	0	2	999.57
									IDFVGELNDK	95.0%	56.2	23.8	2	0	0	2	1,149.58
									KPYPDDENLVEVK	95.0%	43.0	23.1	2	0	0	2	1,545.78
									LGLQNDLFSLAR	95.0%	86.9	21.6	7	0	0	2	1,346.74
									LGWDPKPGEGHLDALLR	95.0%	34.1	21.1	0	6	1	2	1,873.99
									LNLGTVGFYR	95.0%	61.2	21.6	3	0	0	2	1,139.62
									LSVEGFAVDK	95.0%	56.3	23.5	2	0	0	2	1,064.56
									SPVYLTVLK	95.0%	48.9	14.0	3	0	0	2	1,019.61
									TIQQCCENILLNAAWLK	95.0%	68.2	22.3	2	0	0	2	2,075.04
									TQYSSAMLESLLPGIR	95.0%	69.0	22.5	2	1	0	2	1,781.91
									VALSNMNVIDR	95.0%	74.2	23.8	4	0	0	2	1,231.65
									VLGATLLPDLIQK	95.0%	91.2	11.8	18	0	0	2	1,380.85
									VTLSFPSTLQTGTGTLK	95.0%	76.0	17.9	2	0	0	2	1,750.96
									YAAVTQFEATDAR	95.0%	99.6	21.2	3	0	0	2	1,442.69
									YQGGFLISR	95.0%	54.2	21.0	4	0	0	2	1,040.55
Nuclear autoantigenic sperm protein	NASP_HUMAN	NASP	85,218	100.00%	4	5	16	7.49%	EAEGSSAEYKK	95.0%	39.0	21.5	1	0	0	2	1,198.56
									EAQLYAAQAHLK	95.0%	47.8	21.8	2	0	0	2	1,342.71
									EIEELKELLPEIR	95.0%	66.4	18.8	5	1	0	2	1,610.90
									HLVMGDIPAAVNAFQEAASLLGK	95.0%	38.8	20.8	0	7	0	2	2,368.23
Small proline-rich protein 2E	SPR2E_HUMAN	SPRR2E	7,837	100.00%	4	4	88	66.70%	CPEPCPPPK	95.0%	52.2	20.0	69	0	0	2	1,081.48
									CPPVTPSPPCQPK	95.0%	32.6	22.0	1	0	0	2	1,464.70
									CPQPCPPQQCQQK	95.0%	54.9	18.1	2	0	0	2	1,655.71
									QPCQPPPVCPTPK	95.0%	47.2	23.1	16	0	0	2	1,505.72
Transitional endoplasmic reticulum ATPase	TERA_HUMAN	VCP	89,307	100.00%	28	34	195	42.80%	AFEEAEK	95.0%	35.8	20.0	2	0	0	2	823.38
									AIANECQANFISIK	95.0%	63.5	22.6	2	0	0	2	1,578.80
									DVDLEFLAK	95.0%	63.3	23.0	6	0	0	2	1,049.55
									ELQELVQYPVEHPDK	95.0%	73.4	22.3	4	2	0	2	1,823.92
									ETVVEVPQVTWEDIGGLEDVKR	95.0%	81.2	20.5	2	2	0	2	2,498.28
									EVDIGIPDATGR	95.0%	71.2	21.9	9	0	0	2	1,242.63
									GGNIGDGGGAADR	95.0%	75.1	20.1	5	0	0	2	1,116.50
									GILLYGPPGTGK	95.0%	58.7	21.2	9	0	0	2	1,172.67
									GPELLTMWFGESEANVR	95.0%	55.2	21.6	2	0	0	2	1,951.92
									GVLFYGPPGCGK	95.0%	35.2	22.8	1	0	0	2	1,251.62
									IVSQLLTLMDGLK	95.0%	112.0	17.9	20	0	0	2	1,446.82
									KAFEEAEK	95.0%	32.0	23.0	1	0	0	2	951.48
									KGDIFLVR	95.0%	45.2	17.6	11	0	0	2	947.57
									KYEMFAQTLQQSR	95.0%	95.2	22.1	3	2	0	2	1,629.81
									LAGESESNLR	95.0%	73.9	22.7	4	0	0	2	1,075.54
									LAGESESNLRK	95.0%	54.4	23.6	6	0	0	2	1,203.63

									LDQLIYIPLPDEK	95.0%	78.0	20.3	14	0	0	2	1,556.86
									LGDVISIQPCPDVK	95.0%	52.9	22.0	1	0	0	2	1,540.80
									LIVDEAINEDNSVVSLSQPK	95.0%	110.0	20.8	5	0	0	2	2,170.12
									MDELQLFR	95.0%	63.1	23.0	6	0	0	2	1,051.52
									NAPAIIFIDELDAIAPK	95.0%	95.9	18.6	34	2	0	2	1,811.00
									NVFIIGATNRPDIIDPAILRPGR	95.0%	30.1	12.8	0	1	0	2	2,518.43
									QAAPCVLFFDELDSIAK	95.0%	69.4	21.6	8	2	0	2	1,923.95
									QTNPSAMEVEEDDPVPEIR	95.0%	76.8	20.3	4	0	0	2	2,171.98
									RIVSQLLTLMDGLK	95.0%	45.5	14.8	2	2	0	2	1,586.93
									SVSDNDIR	95.0%	36.7	22.5	2	0	0	2	905.43
									VINQILTEMDGMSTK	95.0%	94.9	22.7	9	0	0	2	1,679.84
									WALSQSNPSALR	95.0%	77.9	22.9	10	0	0	2	1,329.69
Peptidyl-prolyl cis-trans isomerase FKBP4	FKBP4_HUMAN	FKBP4	51,788	100.00%	10	10	49	29.00%	AEASSGDHPTDTEMKEEQK	95.0%	45.0	16.6	0	10	0	2	2,105.89
									ATESGAQSAPLPMEGVDISPK	95.0%	72.8	21.6	7	0	0	2	2,101.01
									AWDIAIATMK	95.0%	62.8	23.3	2	0	0	2	1,135.58
									EGTGTEMPMIGDR	95.0%	52.4	17.1	9	0	0	2	1,425.60
									FEIGEGENLDLPYGLER	95.0%	104.0	22.1	8	0	0	2	1,950.95
									LAEEENKAK	95.0%	40.0	24.0	1	0	0	2	1,031.54
									LYANMFER	95.0%	46.6	19.4	2	0	0	2	1,059.49
									QDEGVLK	95.0%	35.9	23.0	2	0	0	2	788.42
									RGEAHLAVNDFELAR	95.0%	45.2	22.0	0	2	0	2	1,697.87
									SNTAGSQSQVETEA	95.0%	75.9	17.4	6	0	0	2	1,408.62
Eukaryotic initiation factor 4A-I	IF4A1_HUMAN	EIF4A1	46,137	100.00%	15	18	65	39.40%	ATQALVLAPTR	95.0%	55.3	18.1	3	0	0	2	1,140.67
									DFTVSAMHGDMDQK	95.0%	43.4	14.1	2	0	0	2	1,613.66
									DQIYDIFQK	95.0%	32.3	23.4	1	0	0	2	1,169.58
									EELTLEGIR	95.0%	32.5	23.7	1	0	0	2	1,059.57
									ELAQQIQK	95.0%	39.3	20.5	5	0	0	2	957.54
									ENYIHR	95.0%	34.0	21.0	1	0	0	2	831.41
									GFKDQIYDIFQK	95.0%	67.4	22.8	4	0	0	2	1,501.77
									GIYAYGFEKPSAIQQR	95.0%	71.7	21.2	3	2	0	2	1,827.94
									GYDVIAQAQSGTGK	95.0%	121.0	23.1	4	0	0	2	1,394.69
									KEELTLEGIR	95.0%	48.1	22.7	2	1	0	2	1,187.66
									LNSNTQVVLLSATMPSDVLEVTK	95.0%	64.9	19.5	2	0	0	2	2,475.30
									LQMEAPHIIVGTPGR	95.0%	58.5	21.3	4	7	0	2	1,618.87
									MFVLDEADEMLSR	95.0%	105.0	20.5	11	0	0	2	1,571.71
									QFYINVER	95.0%	38.2	22.6	2	0	0	2	1,068.55
									VLITTDLLAR	95.0%	55.7	17.6	10	0	0	2	1,114.68
SAP domain-containing ribonucleoprotein	SARNP_HUMAN	SARNP	23,653	100.00%	6	6	12	34.30%	FGISSVPTK	95.0%	49.7	20.9	3	0	0	2	935.52
									FGIVTSSAGTGTTEDTEAK	95.0%	98.5	21.6	2	0	0	2	1,871.89
									FGLNVSSISR	95.0%	47.7	22.0	2	0	0	2	1,079.58
									FNVPVSLESK	95.0%	31.8	23.6	2	0	0	2	1,119.60
									GLSSDNKPMVNLDK	95.0%	69.6	22.4	2	0	0	2	1,533.76
									ITSEIPQTER	95.0%	48.8	23.5	1	0	0	2	1,173.61
Mucin-2	MUC2_HUMAN	MUC2	540,252	100.00%	6	6	37	1.35%	HETQEVLIK	95.0%	38.2	19.6	5	0	0	2	1,096.60
									SNNDFTTR	95.0%	39.2	18.8	2	0	0	2	954.43
									TASGLVEATGAGFANTWK	95.0%	102.0	22.8	20	0	0	2	1,780.89
									TCGCVGPDNVPR	95.0%	35.6	19.0	1	0	0	2	1,331.58
									TFDGDVFR	95.0%	45.5	20.3	5	0	0	2	956.45
									TVHMMPMQVQVQVNR	95.0%	41.2	21.8	0	4	0	2	1,845.88
Eukaryotic translation initiation factor 3 subunit G	EIF3G_HUMAN	EIF3G	35,594	100.00%	3	3	4	7.19%	ADDNATIR	95.0%	47.5	22.0	2	0	0	2	875.42
									LPGELEPVQATQNK	95.0%	39.6	21.4	1	0	0	2	1,523.81

									RADDNATIR	95.0%	33.8	22.4	1	0	0	2	1,031.52
Eukaryotic translation initiation factor 5A-1	IF5A1_HUMAN	EIF5A	16,815	100.00%	8	11	27	55.20%	EDLRLPEGDLGK	95.0%	43.7	22.5	2	2	0	2	1,341.70
									IVEMSTSK	95.0%	42.8	21.5	1	0	0	2	910.46
									KYEDICPSTHNMDVPNIK	95.0%	33.5	21.5	0	2	0	2	2,161.01
									LPEGDLGKEIEQK	95.0%	55.9	21.4	2	0	0	2	1,455.77
									NDFQLIGIQDGYLSLLQDSGEVR	95.0%	74.3	21.0	4	2	0	2	2,580.30
									NGFVVLK	95.0%	51.4	19.9	2	0	0	2	776.47
									VHLVGIDIFTGK	95.0%	57.5	19.2	4	4	0	2	1,298.75
									VHLVGIDIFTGKK	95.0%	46.7	15.6	0	2	0	2	1,426.84
Tripeptidyl-peptidase 1	TPP1_HUMAN	TPP1	61,230	100.00%	6	6	13	16.00%	ILSGRPPLGFLNPR	95.0%	30.4	13.8	0	1	0	2	1,536.90
									LFGGNFAHQASVAR	95.0%	87.2	23.1	1	0	0	2	1,474.76
									LSELVQAVSDPSSPQYGK	95.0%	95.5	22.0	6	0	0	2	1,904.96
									LYQQHGAGLFDVTR	95.0%	54.9	22.3	0	2	0	2	1,604.82
									VNTELMK	95.0%	44.4	23.8	2	0	0	2	850.43
									YLTLENVADLVRPSPLTLHTVQK	95.0%	41.5	14.8	0	1	0	2	2,607.45
6-phosphogluconate dehydrogenase, decarboxylating	6PGD_HUMAN	PGD	53,124	100.00%	11	12	103	26.90%	AGQAVDDFIEK	95.0%	61.5	23.1	7	0	0	2	1,192.59
									FQDTDGK	95.0%	31.1	19.3	1	0	0	2	810.36
									GILFVGSGVSGGEEGAR	95.0%	107.0	23.0	54	0	0	2	1,591.81
									HEMLPASLIQAQR	95.0%	53.7	22.3	4	0	0	2	1,509.78
									IISYAQGFMLLR	95.0%	59.8	22.3	3	0	0	2	1,427.77
									LVPLLDTGDIIIDGGNSEYR	95.0%	116.0	21.5	13	1	0	2	2,160.12
									NPELQNLLLDDFFK	95.0%	80.4	22.4	2	0	0	2	1,705.88
									SAVENCQDSWR	95.0%	48.0	16.1	2	0	0	2	1,351.57
									TVSKVDDFLANEAK	95.0%	78.2	22.7	2	0	0	2	1,536.79
									VDDFLANEAK	95.0%	77.1	23.6	8	0	0	2	1,121.55
									YGPSLMPGGNK	95.0%	75.6	22.3	6	0	0	2	1,136.54
Serpin H1	SERPH_HUMAN	SERPINH1	46,424	100.00%	14	15	46	42.30%	AATLAER	95.0%	31.9	25.1	1	0	0	2	731.41
									AVAISLPK	95.0%	37.1	15.8	1	0	0	2	798.51
									AVLSAEQLR	95.0%	37.3	22.0	2	0	0	2	986.56
									AVLSAEQLRDEEVHAGLGELLR	95.0%	53.0	19.2	0	4	0	2	2,405.28
									DQAVENILVSPVVVASSLGLVSLGGK	95.0%	73.1	14.3	1	6	0	2	2,551.43
									DTQSGSLLFIGR	95.0%	79.8	22.9	9	0	0	2	1,293.68
									GVVEVTHDLQK	95.0%	51.0	21.1	4	0	0	2	1,224.66
									HLAGLGLTEAIDK	95.0%	46.5	19.2	2	0	0	2	1,337.74
									HLAGLGLTEAIDKNK	95.0%	57.2	18.5	0	2	0	2	1,579.88
									LSSLIILMPHHVEPLER	94.7%	18.5	17.2	0	0	1	2	1,984.11
									LYGPSSVSFADDFVR	95.0%	71.9	21.8	7	0	0	2	1,659.80
									SAGLAFSLYQAMAK	95.0%	58.1	22.4	2	0	0	2	1,457.75
									SALQSINEWAAQTTDGK	95.0%	90.5	23.3	2	0	0	2	1,819.88
									TGLYNYYDDEKEK	95.0%	51.4	20.9	2	0	0	2	1,637.73
Chloride intracellular channel protein 4	CLIC4_HUMAN	CLIC4	28,756	100.00%	9	11	32	47.80%	AGSDGESIGNCPFSQR	95.0%	94.2	18.6	2	0	0	2	1,681.72
									FLDGNEMTLADCNLLPK	95.0%	70.9	21.8	4	0	0	2	1,950.93
									FSAYIK	95.0%	34.2	19.5	4	0	0	2	728.40
									GVVFSVTTVDLK	95.0%	56.0	19.0	2	0	0	2	1,264.72
									HPESNTAGMDIFAK	95.0%	34.4	20.4	2	0	0	2	1,533.70
									LDEYLNSPLPDEIDENSMEDIK	95.0%	51.9	20.4	5	2	0	2	2,579.17
									LFMILWLK	95.0%	40.8	16.6	4	0	0	2	1,079.63
									NFDIPK	95.0%	37.2	24.5	1	0	0	2	733.39
									NSRPEANEALER	95.0%	39.8	21.9	5	3	0	2	1,385.68
									YLTNAYSR	95.0%	40.1	20.5	2	0	0	2	987.49
Phosphoglycerate mutase 1	PGAM1_HUMAN	PGAM1	28,787	100.00%	17	24	165	69.70%	ALPFWNEEIVPQIK	95.0%	74.7	20.6	34	0	0	2	1,683.91

									AMEAVAAQGK	95.0%	68.3	23.2	3	0	0	2	991.49
									FSGWYDADLSPAGHEEAK	95.0%	82.3	19.8	8	19	0	2	1,979.88
									FSGWYDADLSPAGHEEAKR	95.0%	41.9	20.5	0	6	0	2	2,135.98
									HGEAQVK	95.0%	47.5	19.5	2	0	0	2	768.40
									HGESAWNLENR	95.0%	79.2	21.2	12	1	0	2	1,312.60
									HLEGLSEEAIMELNLPTGIPIVYELDK	95.0%	34.3	19.8	0	1	0	2	3,039.56
									HYGGLTGLNK	95.0%	49.6	23.0	10	0	0	2	1,059.56
									KAMEAVAAQGK	95.0%	73.1	23.5	5	1	0	2	1,103.59
									NLKPIKPMQFLGDEETVR	95.0%	54.3	20.5	4	5	6	2	2,115.13
									NLKPIKPMQFLGDEETVRK	95.0%	30.3	18.5	0	0	10	2	2,243.22
									RGGQALR	94.7%	30.1	20.9	1	0	0	2	757.44
									RSYDVPPPPMEPDHPFYSNISK	95.0%	43.2	20.6	0	3	3	2	2,589.21
									SYDVPPPPMEPDHPFYSNISK	95.0%	43.7	19.9	4	0	0	2	2,433.11
									VLIAAHGNSLR	95.0%	58.0	18.2	12	9	0	2	1,150.67
									YADLTEDQLPSCESLK	95.0%	87.8	20.5	2	0	0	2	1,868.86
									YADLTEDQLPSCESLKDTIAR	95.0%	47.0	21.0	0	4	0	2	2,425.16
Serine/threonine-protein phosphatase 6 catalytic subunit	PPP6_HUMAN	PPP6C	35,127	100.00%	3	4	7	11.10%	APLDLDKYVEIAR	95.0%	68.6	21.8	1	2	0	1	1,502.82
									GAGWLFGAK	95.0%	47.8	21.7	2	0	0	2	906.48
									VTNEFVHINNLK	95.0%	70.8	21.5	2	0	0	2	1,427.76
Ras-related protein Rab-2A	RAB2A_HUMAN	RAB2A	23,528	100.00%	3	3	8	19.30%	IQEGVFDINNEANGIK	95.0%	102.0	23.0	2	0	0	2	1,760.88
									TASNVEEAFINTAK	95.0%	101.0	23.4	4	0	0	2	1,494.74
									YIIIGDTGVGK	95.0%	43.7	21.7	2	0	0	2	1,135.64
Tyrosyl-tRNA synthetase, cytoplasmic	SYYC_HUMAN	YARS	59,127	100.00%	7	7	14	15.30%	AMLESIGVPLEK	95.0%	35.8	22.6	2	0	0	2	1,286.70
									APWELLELR	95.0%	41.9	20.7	2	0	0	2	1,126.63
									IDVGEAEPR	95.0%	53.7	20.8	2	0	0	2	985.50
									NSEPEEVIPSR	95.0%	39.4	21.8	2	0	0	2	1,256.61
									QVEPLDPPAGSAPGEHVFVK	95.0%	30.7	22.0	0	2	0	2	2,074.06
									VDAQFGGIDQR	95.0%	79.3	23.7	2	0	0	2	1,205.59
									VSYYENVIK	95.0%	37.1	22.8	2	0	0	2	1,114.58
Ferritin light chain	FRIL_HUMAN	FTL	20,003	100.00%	3	3	5	21.70%	ELAEEKR	95.0%	36.2	24.4	1	0	0	2	874.46
									KLNQALLDLHALGSAR	95.0%	35.3	14.8	0	2	0	2	1,719.99
									LGGPEAGLGEYLFER	95.0%	59.2	23.2	2	0	0	2	1,607.81
Proteasome subunit alpha type-2	PSA2_HUMAN	PSMA2	25,881	100.00%	4	8	47	32.10%	HIGLVYSGMGPDYR	95.0%	73.1	22.3	6	5	0	2	1,580.75
									LAQQYYLVYQEPIPTAQLVQR	95.0%	89.0	18.8	4	1	0	2	2,521.35
									LVQIEYALAAVAGGAPSVGIK	95.0%	44.2	14.0	1	18	0	2	2,027.15
									YNEDLELEDAIHTAILTLK	95.0%	119.0	20.9	2	10	0	2	2,201.13
Glutathione synthetase	GSHB_HUMAN	GSS	52,368	100.00%	18	18	99	42.80%	AIEHADGGVAAGVAVLDNPYPV	95.0%	65.2	21.9	15	0	0	2	2,135.08
									AIENELLAR	95.0%	53.8	21.4	4	0	0	2	1,028.57
									ALAEGVLLR	95.0%	73.2	16.5	16	0	0	2	941.58
									ASYILMEK	95.0%	61.6	20.2	8	0	0	2	954.50
									CPDIATQLAGTK	95.0%	71.6	22.8	3	0	0	2	1,274.64
									EGGGNNLYGEEMVQALK	95.0%	72.5	21.9	8	0	0	2	1,808.85
									EGIAQTVFLGLNR	95.0%	80.6	21.1	9	0	0	2	1,417.78
									GLALGIAK	95.0%	40.3	18.2	2	0	0	2	742.48
									GSLDQDR	95.0%	33.9	19.9	2	0	0	2	790.37
									ILSNNPSK	95.0%	33.0	22.3	2	0	0	2	872.48
									QDDFTAR	95.0%	36.4	21.0	4	0	0	2	852.39
									QIEINTISASFGGLASR	95.0%	124.0	21.6	6	0	0	2	1,763.93
									QQLEELAR	95.0%	43.1	21.4	3	0	0	2	986.53
									QYSLQNWEAR	95.0%	60.7	22.5	6	0	0	2	1,294.62
									SADGSPALK	95.0%	44.2	22.0	4	0	0	2	845.44

									SDYMFQR	95.0%	30.9	13.0	1	0	0	2	962.40
									TFEDISEK	95.0%	43.2	21.1	5	0	0	2	968.46
									VQQELSRPGMLEMLLPGQPEAVAR	94.6%	26.2	20.8	0	1	0	2	2,681.38
N-acetyl-D-glucosamine kinase	NAGK_HUMAN	NAGK	37,359	99.90%	2	2	3	7.85%	AGVDPLVPLR	95.0%	45.2	17.8	1	0	0	2	1,036.62
									HIVAVLPEIDPVLFQGK	95.0%	46.9	12.3	0	2	0	2	1,875.07
Mucin-4	MUC4_HUMAN	MUC4	231,563	100.00%	3	3	4	1.89%	IGLASALQPR	95.0%	65.0	16.9	1	0	0	2	1,025.61
									NDVVFQPISGEDVR	95.0%	41.5	22.1	2	0	0	2	1,574.78
									TEGLLGVWNNNPEDDFR	95.0%	77.3	20.5	1	0	0	2	1,975.92
Exostosin-1	EXT1_HUMAN	EXT1	86,239	100.00%	5	6	23	9.25%	ASISTENFRPNFDVSIPLFSK	95.0%	26.9	20.8	0	1	0	2	2,369.21
									IAESYQNILAAIEGSR	95.0%	92.8	22.6	7	6	0	2	1,734.90
									IVLTTLEIIQDR	94.6%	30.0	17.1	1	0	0	2	1,413.83
									LLLQIPSTIR	95.0%	45.7	9.5	4	0	0	2	1,153.73
									YLTGIGSDTR	95.0%	44.7	22.0	4	0	0	2	1,082.55
Catenin alpha-1	CTNA1_HUMAN	CTNNA1	100,055	100.00%	4	5	17	6.73%	LLEPLVTQVTTLVNTNSK	95.0%	98.2	15.8	6	1	0	2	1,970.12
									NAGNEQDLGIQYK	95.0%	63.0	22.5	4	0	0	2	1,449.70
									TSVQTEDDQLIAGQSAR	95.0%	84.4	22.3	4	0	0	2	1,818.88
									WDDSGNDIIVLAK	95.0%	73.4	22.8	2	0	0	2	1,445.73
Coatomer subunit epsilon	COPE_HUMAN	COPE	34,465	100.00%	5	5	11	20.50%	APPAPGPASGGSGEVDELFDVK	95.0%	88.3	22.3	2	0	0	1	2,097.01
									AYLAQR	95.0%	31.1	23.7	1	0	0	2	721.40
									FGVVLDEIKPSSAPELQAVR	95.0%	52.8	18.2	0	3	0	2	2,155.18
									KFGVVLDEIKPSSAPELQAVR	95.0%	33.0	16.0	0	1	0	2	2,283.27
									LQDAYYIFQEMADK	95.0%	86.2	22.6	4	0	0	2	1,734.81
Glutamine synthetase	GLNA_HUMAN	GLUL	42,047	100.00%	4	4	6	11.30%	GGLDNAR	95.0%	30.7	19.3	1	0	0	2	702.35
									QVYMSLPQGEK	95.0%	42.8	21.8	2	0	0	2	1,295.63
									RPAETNLR	95.0%	38.5	19.8	2	0	0	2	956.53
									VQAMYIWIDGTGEGLR	95.0%	54.3	22.5	1	0	0	2	1,824.90
Cleavage and polyadenylation specificity factor subunit 5	CPSF5_HUMAN	NUDT21	26,210	100.00%	5	6	14	26.90%	LFLVQLQEK	95.0%	66.4	18.8	2	0	0	2	1,117.66
									LMTEILGR	95.0%	41.0	21.4	2	0	0	2	932.52
									LPGGELNPGEDEVEGLK	95.0%	76.8	22.3	2	0	0	2	1,752.87
									TINLYPLTNYTFGTK	95.0%	74.2	22.3	4	0	0	2	1,745.91
									YIQQTKPLTLER	95.0%	52.2	19.4	2	2	0	2	1,489.84
Eukaryotic translation initiation factor 3 subunit C	EIF3C_HUMAN	EIF3C	105,329	100.00%	3	3	6	3.18%	ELLGQGLLLR	95.0%	48.0	16.7	2	0	0	2	1,111.68
									FEELTNLIR	95.0%	48.4	22.5	2	0	0	2	1,134.62
									LGSLVENNER	95.0%	57.1	22.6	2	0	0	2	1,130.58
Ig kappa chain C region	IGKC_HUMAN	IGKC	11,591	99.50%	2	2	15	35.80%	TVAAPSVFIFPPSDEQLK	95.0%	68.6	20.3	11	0	0	2	1,946.03
									VDNALQSGNSQESVTEQDSK	95.0%	140.0	20.1	4	0	0	2	2,135.97
Integrin alpha-2	ITA2_HUMAN	ITGA2	129,280	100.00%	4	4	6	5.17%	FVQGLDIGPTK	95.0%	55.4	22.6	2	0	0	2	1,174.65
									IGQTSSSVSFK	95.0%	36.6	21.7	2	0	0	2	1,140.59
									QIPAAQEQPFIVSNQNK	95.0%	31.7	21.8	1	0	0	2	1,911.99
									VDISLENPGTSPALEAYSETAK	95.0%	84.3	22.4	1	0	0	2	2,292.12
Dipeptidyl peptidase 9	DPP9_HUMAN	DPP9	98,246	100.00%	5	5	7	7.65%	AGKPYQLQIYPNER	95.0%	31.0	22.4	0	1	0	2	1,676.88
									ELVQPFSSLFPK	95.0%	39.0	20.9	2	0	0	2	1,391.76
									LSGPDDDPLHK	95.0%	33.7	23.1	1	0	0	2	1,193.58
									LYYLGMPYGSR	95.0%	38.7	22.2	2	0	0	2	1,335.64
									SQGYDWSEPFSPGEDEFK	95.0%	54.1	16.0	1	0	0	2	2,104.88
Heterogeneous nuclear ribonucleoprotein Q	HNRPQ_HUMAN	SYNCRIP	69,586	100.00%	10	10	52	20.70%	AGPIWDLR	95.0%	39.9	21.3	2	0	0	2	927.51
									DLFEDELVPLFEK	95.0%	75.9	23.0	23	0	0	2	1,593.81
									DSDLSHVQNK	95.0%	51.8	21.1	6	0	0	2	1,142.54
									EAAQEAVK	95.0%	37.7	22.0	5	0	0	2	845.44
									EFNEDGALAVLQQFK	95.0%	44.6	23.1	3	0	0	2	1,708.85
									LMMDPLTGLNR	95.0%	50.8	23.2	5	0	0	2	1,292.63

									TGYTLDVTTGQR	95.0%	43.9	22.5	2	0	0	2	1,311.65
									TKEQILEEFSK	95.0%	58.2	22.3	2	0	0	2	1,351.71
									VTEGLTDVILYHQPDDK	95.0%	42.5	22.5	0	3	0	2	1,942.98
									VWGNVGTVEWADPIEDPDPEVMAK	95.0%	43.8	21.0	1	0	0	2	2,670.24
5'-nucleotidase domain-containing protein 1	NT5D1_HUMAN	NT5DC1	51,830	100.00%	5	6	10	16.00%	DIVAAIQHNYK	95.0%	39.7	21.7	2	0	0	2	1,271.67
									MMTPEVLAEAYGK	95.0%	50.1	21.0	2	0	0	2	1,471.68
									SQRPEESEPLEK	95.0%	46.0	22.3	2	1	0	2	1,428.70
									TAGYYPNPPLVLSSDETLISK	95.0%	74.0	20.8	2	0	0	2	2,265.17
									VVYFGDSMHSDIFPAR	95.0%	32.6	21.2	0	1	0	2	1,856.86
Nardilysin	NRDC_HUMAN	NRD1	131,558	100.00%	5	5	11	4.09%	ANLVLLSGANEGK	95.0%	104.0	21.9	4	0	0	2	1,285.71
									GSLSNAGDPEIVK	95.0%	59.2	22.9	2	0	0	2	1,286.66
									LGADESEEEGR	95.0%	66.3	17.9	2	0	0	2	1,191.51
									LGADESEEEGRR	95.0%	50.0	19.4	0	2	0	2	1,347.61
									TVFQFDVQR	95.0%	35.7	22.2	1	0	0	2	1,139.59
Aldose reductase	ALDR_HUMAN	AKR1B1	35,836	100.00%	7	7	66	26.30%	EELFIVSK	95.0%	41.2	19.9	2	0	0	2	964.54
									GIVVTAYSPLGSPDRPWAKPEDPSLLEDPR	95.0%	33.9	18.7	0	2	0	2	3,262.68
									MPILGLGTWK	95.0%	53.7	20.5	28	0	0	2	1,115.63
									NLVVIPK	95.0%	37.2	13.6	4	0	0	2	782.51
									REELFIVSK	95.0%	45.2	20.6	16	0	0	2	1,120.64
									SPPGQVTEAVK	95.0%	75.6	20.5	23	0	0	2	1,112.60
									TTAQVLIR	95.0%	64.5	18.5	12	0	0	2	901.55
									VAIDVGYR	95.0%	47.0	22.9	7	0	0	2	892.49
Osteopontin	OSTP_HUMAN	SPP1	35,405	100.00%	9	10	52	37.60%	AIPVAQDLNAPSDWDSR	95.0%	98.1	22.7	18	0	0	2	1,854.90
									ANDESNEHSDVIDSQELSK	95.0%	26.9	18.5	0	1	0	2	2,116.93
									DSYETSQLDDQSAETHSHK	95.0%	43.4	16.8	0	4	0	2	2,177.92
									GKDSYETSQLDDQSAETHSHK	95.0%	52.4	18.1	0	3	1	2	2,363.04
									ISHELDSASSEVN	95.0%	56.6	20.5	6	0	0	2	1,387.63
									KANDESNEHSDVIDSQELSK	95.0%	43.5	20.2	0	3	0	2	2,245.02
									QLYNKYPDAVATWLNPDPSQK	95.0%	35.5	21.1	0	2	0	2	2,448.22
									QNLLAPQNAVSSEETNDFKQETLPSK	95.0%	58.7	21.3	0	8	0	2	2,888.43
									YPDAVATWLNPDPSQK	95.0%	81.2	22.4	6	0	0	2	1,801.88
Isoleucyl-tRNA synthetase, mitochondrial	SYIM_HUMAN	IARS2	113,776	100.00%	2	2	3	1.98%	DSFLGSIPGK	95.0%	35.1	22.5	2	0	0	2	1,020.54
									EMTADVIELK	95.0%	34.0	23.1	1	0	0	2	1,164.58
Tumor necrosis factor receptor superfamily member 6B	TNF6B_HUMAN	TNFRSF6B	32,661	99.50%	2	2	18	10.30%	LLQALEAPEGWGPTPR	95.0%	54.1	21.4	7	0	0	2	1,734.92
									LTELLGAQDGALLVR	95.0%	91.7	17.7	11	0	0	2	1,568.90
Neuroblast differentiation-associated protein AHNAK	AHNK_HUMAN	AHNAK	629,086	100.00%	10	10	85	2.19%	ADIDVSGPK	95.0%	47.5	22.1	28	0	0	2	901.46
									GEGPDVDVNLPK	95.0%	55.0	22.6	15	0	0	2	1,239.62
									GEGPEVDVNLPK	95.0%	43.2	22.3	8	0	0	2	1,253.64
									VDIDVPDVNIEGPDAK	95.0%	56.3	22.6	8	0	0	2	1,695.84
									VDIDVPDVNIEGPEGK	95.0%	50.9	22.4	4	0	0	2	1,695.84
									VDIEGPDVNIEGPEGK	95.0%	57.3	22.4	1	0	0	2	1,667.81
									VDVDIPDVNIEGPDAK	95.0%	43.6	22.4	2	0	0	2	1,695.84
									VDVDVPDVNIEGPDAK	95.0%	72.0	22.6	13	0	0	2	1,681.83
									VDVEVPDVSLEGPEGK	95.0%	49.0	21.6	6	0	0	2	1,668.83
Ig alpha-1 chain C region	IGHA1_HUMAN	IGHA1	37,636	99.50%	2	2	3	7.37%	QEPSQGTTTFAVTSILR	95.0%	46.9	22.0	1	0	0	2	1,835.95
									SAVQGPPER	95.0%	37.3	21.9	2	0	0	2	940.49
Aconitate hydratase, mitochondrial	ACON_HUMAN	ACO2	85,410	100.00%	10	11	21	17.20%	DGYAQILR	95.0%	47.6	23.3	2	0	0	2	935.50
									DINQEVYNFLATAGAK	95.0%	79.6	22.0	2	0	0	2	1,753.88
									DSSGQHVDVSPTSQR	95.0%	43.7	21.1	2	1	0	2	1,599.74
									EGWPLDIR	95.0%	33.2	20.8	1	0	0	2	985.51
									IVYGHLDDPASQEIER	95.0%	35.1	21.9	0	2	0	2	1,841.90

									LTGSLSGWSSPK	95.0%	54.7	23.1	2	0	0	2	1,219.63
									NAVTQEFGPVPDTAR	95.0%	67.6	22.6	2	0	0	2	1,601.79
									QGLLPLTFADPADYNK	95.0%	69.0	22.3	3	0	0	2	1,762.90
									SQFTITPGSEQIR	95.0%	41.4	23.2	2	0	0	2	1,463.75
									WVVIGDENYGEGSSR	95.0%	54.3	21.4	2	0	0	2	1,667.77
Cathepsin B	CATB_HUMAN	CTSB	37,803	100.00%	10	13	86	26.30%	DIMAEIYK	95.0%	33.1	21.6	3	0	0	2	998.49
									HYGYNSYSVSNSEK	95.0%	80.1	19.1	5	0	0	2	1,634.71
									ICEPGYSPTYK	95.0%	52.7	20.5	6	0	0	2	1,314.60
									LCGTFLGGPKPPQR	95.0%	53.6	21.8	3	2	0	2	1,527.81
									LPASFDAR	95.0%	51.1	24.5	7	0	0	2	876.46
									SGVYQHVTGEMMGGHAIR	95.0%	65.4	21.5	1	15	0	2	1,961.90
									TDQYWEK	95.0%	38.1	19.4	3	0	0	2	969.43
									TDQYWEKI	95.0%	62.5	21.5	26	0	0	2	1,082.52
									VMFTEDLK	95.0%	35.0	22.0	2	0	0	2	982.49
									VMFTEDLKLPASFDAR	95.0%	45.5	22.6	1	12	0	2	1,855.93
Protein-arginine deiminase type-3	PADI3_HUMAN	PADI3	74,725	100.00%	9	9	19	17.50%	ALLFQGVVDDEQVK	95.0%	63.1	22.5	2	0	0	2	1,560.83
									ATAFFPDLVNMLVLGK	95.0%	61.7	20.4	4	0	0	2	1,751.94
									HVLGQDK	95.0%	36.7	20.1	2	0	0	2	796.43
									ILGPDFGYVTR	95.0%	38.2	21.7	1	0	0	2	1,237.66
									ILIGGNLPGSSGR	95.0%	75.1	20.2	3	0	0	2	1,240.70
									SVSGLDSFGNLEVSPPVVANGK	95.0%	34.4	21.6	1	0	0	2	2,173.11
									TISINQVLSNK	95.0%	39.2	21.6	2	0	0	2	1,216.69
									TLPVVFDSPR	95.0%	44.0	23.2	2	0	0	2	1,130.62
									TQGPAALFDDHK	95.0%	36.4	21.7	0	2	0	2	1,299.63
Lamin-B1	LMNB1_HUMAN	LMNB1	66,392	100.00%	6	6	13	12.80%	ALYETELADAR	95.0%	53.2	22.8	4	0	0	2	1,251.62
									IESLSSQLSNLQK	95.0%	38.3	21.7	1	0	0	2	1,446.78
									IGDTSVSYK	95.0%	30.9	20.4	1	0	0	2	969.49
									IQELEDLLAK	95.0%	36.4	22.9	2	0	0	2	1,171.66
									LLEGEEER	95.0%	39.2	21.6	2	0	0	2	974.48
									LSSEMNTSTVNSAR	95.0%	67.2	22.1	2	0	0	2	1,512.70
									NSQGEEVAQR	95.0%	66.6	20.4	3	0	0	2	1,117.52
Endoplasmic reticulum resident protein 29	ERP29_HUMAN	ERP29	28,977	100.00%	6	6	17	27.60%	DGDFENPVPYTGAVK	95.0%	72.1	22.1	2	0	0	2	1,608.75
									ESYPVFYLFR	95.0%	43.5	23.7	2	0	0	2	1,320.66
									FDTQYPYGEK	95.0%	31.5	18.9	1	0	0	2	1,247.56
									GALPLDTVTFYK	95.0%	56.7	20.6	5	0	0	2	1,324.72
									ILDQGEDFPASEMTR	95.0%	92.5	21.1	3	0	0	2	1,724.78
									SLNILTAFQK	95.0%	68.3	18.1	4	0	0	2	1,134.65
NSFL1 cofactor p47	NSF1C_HUMAN	NSFL1C	40,555	100.00%	9	12	24	33.80%	ASSSILIDESEPTTNIQIR	95.0%	78.2	22.1	1	0	0	2	2,074.07
									DLIHDQDEDEEEEEGQR	95.0%	105.0	14.3	1	2	0	2	2,085.85
									EANLLNAVIVQR	95.0%	51.8	18.9	2	0	0	2	1,339.77
									LGAAPEEESAYVAGEK	95.0%	63.2	22.7	2	0	0	2	1,620.78
									LGAAPEEESAYVAGEKR	95.0%	67.1	22.0	1	1	0	2	1,776.88
									LGSTAPQVLSTSSPAQQAENEAK	95.0%	74.0	22.1	2	1	0	2	2,314.15
									SGFSLDNGELR	95.0%	51.0	23.0	2	0	0	2	1,194.58
									SPNELVDDLFK	95.0%	62.1	23.4	4	0	0	2	1,276.64
									SYQDPSNAQFLESIR	95.0%	108.0	21.5	5	0	0	2	1,754.84
Spectrin alpha chain, brain	SPTA2_HUMAN	SPTAN1	284,525	100.00%	59	66	256	29.40%	AALLELWELR	95.0%	53.1	20.0	5	0	0	2	1,213.69
									ADVVESWIGEK	95.0%	43.1	23.8	4	0	0	2	1,232.62
									ALINADELASDVAGAEALLDR	95.0%	114.0	21.4	4	2	0	2	2,127.09
									DLAALEDKVK	95.0%	45.5	21.4	3	0	0	2	1,101.62
									DLAALGDKVNSLGETAER	95.0%	43.6	22.1	0	1	0	2	1,858.95

DLASVQALLR	95.0%	72.8	21.2	8	0	0	2	1,085.63
DLIGVQNLLK	95.0%	50.5	18.6	3	0	0	2	1,112.67
DLSSVQTLLTK	95.0%	72.3	21.0	7	0	0	2	1,204.68
DLTGVQNLR	95.0%	59.4	22.4	3	0	0	2	1,015.55
DLTNVQNLQK	95.0%	50.9	22.3	4	0	0	2	1,172.63
DLTSWVTEMK	95.0%	37.1	23.2	6	0	0	2	1,209.58
DTEQVDNWMSK	95.0%	44.0	17.8	1	0	0	2	1,352.58
DVDEIEAWISEK	95.0%	39.0	22.4	1	0	0	2	1,433.68
DVDETISWIK	95.0%	52.7	23.9	1	0	0	2	1,205.61
DVEDEETWIR	95.0%	72.2	20.2	9	0	0	2	1,291.58
DVTGAEALLER	95.0%	72.3	23.5	9	0	0	2	1,173.61
EAALTSEEVGADLEQVEVLQK	95.0%	80.6	21.2	1	0	0	2	2,258.14
EANELQQWINEK	95.0%	69.7	21.6	4	0	0	2	1,501.73
EANQQQQFNR	95.0%	45.0	21.6	8	0	0	2	1,262.59
EELYQNLTR	95.0%	31.4	23.1	2	0	0	2	1,165.59
EKEPIAASTNR	95.0%	26.6	23.7	0	1	0	2	1,215.63
ELPTAFDYVEFTR	95.0%	56.6	22.6	5	0	0	2	1,587.77
GKDLIGVQNLLK	95.0%	40.5	14.9	4	0	0	2	1,297.78
GLVSSDELAK	95.0%	32.6	24.0	2	0	0	2	1,018.54
GNAMVEEGHFAAEDVK	95.0%	64.2	20.1	2	4	0	2	1,719.76
GVIDMGNSLIER	95.0%	49.7	22.8	4	0	0	2	1,319.66
HQAFEAEVQANSGAIVK	95.0%	29.1	22.0	0	1	0	2	1,798.91
IAALQAFADQLIAAGHYAK	95.0%	35.3	19.8	0	2	0	2	1,972.07
IDGITIQAR	95.0%	45.6	22.0	2	0	0	2	986.56
ITALDEFATK	95.0%	45.8	22.4	6	0	0	2	1,108.59
KVEDLFLTFAK	95.0%	45.0	18.8	4	0	0	2	1,310.74
LAALADQWQFLVQK	95.0%	60.3	20.7	4	0	0	2	1,630.90
LDILDQER	95.0%	35.7	21.2	1	0	0	2	1,001.53
LFGAAEVQR	95.0%	68.3	24.1	13	0	0	2	990.54
LGESQTLQQFSR	95.0%	64.5	22.9	4	0	0	2	1,393.71
LIQEQHPEEELIK	95.0%	54.8	21.6	5	0	0	2	1,605.85
LIQNNHYAMEDVATR	95.0%	43.5	22.4	0	4	0	2	1,790.85
LIQSHPESAEDLQEK	95.0%	72.2	22.2	7	2	0	2	1,723.85
LLEATELK	95.0%	31.0	20.9	1	0	0	2	916.54
LLVGSEDYGR	95.0%	49.3	22.3	4	0	0	2	1,108.56
LNDSYR	95.0%	36.1	21.8	3	0	0	2	767.37
LQIASDENYKDPTNLQGK	95.0%	77.5	22.5	1	3	0	2	2,034.01
LQQLFR	95.0%	30.8	21.2	2	0	0	2	804.47
LQQSHPLSATQIQVK	95.0%	50.9	18.2	0	4	0	2	1,677.93
LQTASDESYKDPTNIQSK	95.0%	76.0	21.8	3	3	0	2	2,024.98
LSDDNTIGKEEIQQR	95.0%	76.9	22.7	1	6	0	2	1,745.87
LTVLSEER	95.0%	47.8	22.9	4	0	0	2	946.52
MNEVISLWK	95.0%	43.2	23.3	5	0	0	2	1,119.59
MTLVASEDYGDTLAAIQGLLK	95.0%	82.8	21.3	2	0	0	2	2,225.14
NQALNTDNYGHDLASVQALQR	95.0%	47.8	22.4	0	2	0	2	2,328.13
NTTGVTEEALK	95.0%	31.1	23.8	1	0	0	2	1,162.60
QEQIDNQTR	95.0%	44.2	20.7	2	0	0	2	1,131.54
REELITNWEQIR	95.0%	37.9	22.2	0	2	0	2	1,586.83
SADESGQALLAAGHYASDEVR	95.0%	61.9	21.6	0	8	0	2	2,147.00
SQLLGSAHEVQR	95.0%	66.2	22.2	5	0	0	2	1,324.70
SSLSSAQADFNQLAELDR	95.0%	133.0	21.7	7	0	0	2	1,951.94

									TATDEAYKDPSNLQGK	95.0%	73.9	22.3	8	4	0	2	1,737.83
									VLETAEDIQER	95.0%	71.1	22.5	6	0	0	2	1,302.65
									VNEVNQFAAK	95.0%	72.5	23.6	6	0	0	2	1,119.58
Serpin B13	SPB13_HUMAN	SERPINB13	44,259	100.00%	5	5	8	19.40%	ADYSGMSSGSGLYAQK	95.0%	64.8	19.0	2	0	0	2	1,637.71
									IKDLFPDGSISSSTK	95.0%	30.6	21.8	1	0	0	2	1,594.83
									LGFDLFK	95.0%	42.8	19.8	2	0	0	2	839.47
									NNDLSMFVLLPNDIDGLEK	95.0%	86.6	22.3	2	0	0	2	2,163.06
									YYHASLEPVDFVNAADESR	95.0%	37.4	21.5	0	1	0	2	2,183.00
Growth/differentiation factor 15	GDF15_HUMAN	GDF15	34,137	100.00%	4	4	6	17.20%	AALPEGLPEASR	95.0%	38.8	22.4	1	0	0	2	1,210.64
									AANMHAQIK	95.0%	40.4	20.9	1	0	0	2	999.50
									ASLEDLGWADWVLSPR	95.0%	78.5	22.0	2	0	0	2	1,814.91
									TDTGVSLQTYDDLLAK	95.0%	57.7	22.1	2	0	0	2	1,739.87
LanC-like protein 2	LANC2_HUMAN	LANCL2	50,838	99.50%	2	2	2	7.11%	SGNYPSSLSNETDR	95.0%	59.0	17.4	1	0	0	2	1,526.67
									VDQETLTEMVKPSIDYVR	95.0%	29.2	22.0	0	1	0	2	2,139.06
Stress-70 protein, mitochondrial	GRP75_HUMAN	HSPA9	73,663	100.00%	15	15	58	28.60%	AQFEGIVTDLIR	95.0%	70.7	21.4	3	0	0	2	1,361.74
									DAGQISGLNVLR	95.0%	65.2	22.9	7	0	0	2	1,242.68
									DSETGENIR	95.0%	49.8	19.1	2	0	0	2	1,020.46
									KDSETGENIR	95.0%	63.7	22.9	2	0	0	2	1,148.55
									LLGQFTLIGIPPAPR	95.0%	42.3	10.4	4	0	0	2	1,592.95
									LYSPSQIGAFVLMK	95.0%	43.0	21.8	2	0	0	2	1,569.84
									NAVITVPAYFNDSQR	95.0%	52.0	22.9	2	0	0	2	1,694.85
									NTTIPTK	95.0%	36.0	24.6	1	0	0	2	774.44
									QAVTNPNNTFYATK	95.0%	88.5	22.7	3	0	0	2	1,568.77
									RYDDPEVQK	95.0%	44.4	22.8	3	0	0	2	1,149.55
									SQVFSTAADGQTQVEIK	95.0%	70.0	22.2	8	0	0	2	1,808.90
									STNGDTFLGGEDFDQALLR	95.0%	97.8	21.8	2	0	0	2	2,055.96
									TTPSVVAFTADGER	95.0%	88.8	22.7	4	0	0	2	1,450.72
									VINEPTAAALAYGLDK	95.0%	98.2	21.2	2	0	0	2	1,645.88
									VLENAEGAR	95.0%	61.5	21.0	9	0	0	2	958.50
									VQQTVQDLFGR	95.0%	77.0	23.4	5	0	0	2	1,290.68
Myelin protein zero-like protein 2	MPZL2_HUMAN	MPZL2	24,466	99.50%	2	2	6	10.20%	NPPDVDGVIGEIR	95.0%	69.1	22.2	4	0	0	2	1,380.71
									YDASILLWK	95.0%	40.3	21.2	2	0	0	2	1,108.60
Claudin-1	CLD1_HUMAN	CLDN1	22,726	99.50%	2	2	5	14.70%	IVQEFYDPMTPVNAR	95.0%	62.1	21.8	3	0	0	2	1,779.87
									VFDSLLNLSSTLQATR	95.0%	74.0	19.2	2	0	0	2	1,764.95
Splicing factor 3A subunit 1	SF3A1_HUMAN	SF3A1	88,868	100.00%	4	4	10	8.07%	ASKPLPPAPAPDEYLVSPITGEK	95.0%	29.6	19.4	0	2	0	2	2,377.27
									IGEEEIQKPEEK	95.0%	55.0	22.1	2	0	0	2	1,428.72
									VMQQQQQTTQQQLPQK	95.0%	71.6	21.8	4	0	0	2	1,957.98
									VQAQVIQETIVPK	95.0%	55.5	16.8	2	0	0	2	1,452.84
Heterogeneous nuclear ribonucleoprotein G	HNRPG_HUMAN	RBMX	42,316	100.00%	5	7	19	13.00%	AIKVEQATKPSFESGR	95.0%	38.5	21.5	0	1	2	2	1,747.93
									GFAFVTFESPADAK	95.0%	76.1	22.3	3	0	0	2	1,486.72
									IVEVLLMK	95.0%	32.0	18.8	1	0	0	2	944.59
									LFIGGLNTETNEK	95.0%	80.0	22.3	9	0	0	2	1,435.74
									VEQATKPSFESGR	95.0%	60.5	23.1	2	1	0	2	1,435.72
Eukaryotic translation initiation factor 4H	IF4H_HUMAN	EIF4H	27,368	100.00%	4	5	15	23.80%	EALTYDGALLGDR	95.0%	82.7	23.4	6	0	0	2	1,393.70
									EEVVQKEQE	95.0%	46.9	22.3	3	0	0	2	1,117.54
									GSNMDFREPTEEER	95.0%	37.4	16.3	1	2	0	2	1,712.72
									TVATPLNQVANPNSAIFGGARPR	95.0%	43.9	18.8	0	3	0	2	2,351.26
Splicing factor, arginine/serine-rich 1	SFRS1_HUMAN	SFRS1	27,727	100.00%	10	11	90	44.40%	DGTGVVEFVR	95.0%	66.8	22.9	22	0	0	2	1,078.55
									DGYDYDGYR	95.0%	60.4	11.5	6	0	0	2	1,123.43
									EAGDVCYADVYR	95.0%	68.1	17.0	4	0	0	2	1,417.61
									GGPPFAFVEFEDPR	95.0%	65.2	21.3	7	0	0	2	1,564.74

									GGPPFAFVEFEDPRDAEDAVYGR	95.0%	37.0	20.5	0	3	0	2	2,541.17
									IYVGNLPPDIR	95.0%	54.8	21.6	17	0	0	2	1,256.70
									KEDMTYAVR	95.0%	43.4	21.1	8	0	0	2	1,128.54
									SHEGETAYIR	95.0%	42.9	22.0	6	0	0	2	1,162.55
									TKDIEDVFYK	95.0%	62.2	22.2	8	4	0	2	1,257.64
									VVVSGLPPSGSWQDLK	95.0%	39.5	21.0	5	0	0	2	1,668.90
Haloacid dehalogenase-like hydrolase domain-containing protein	HDHD2_HUMAN	HDHD2	28,519	100.00%	2	2	4	13.10%	DGLALGPGPFVTALEYATDTK	95.0%	70.3	21.8	2	0	0	2	2,136.09
									LLLDGAPLIAIHK	95.0%	45.4	7.0	2	0	0	2	1,373.85
Junction plakoglobin	PLAK_HUMAN	JUP	81,728	100.00%	10	10	55	17.00%	ALMGSPQLVAAVVR	95.0%	73.5	20.1	17	0	0	2	1,427.80
									HPEAEMAQNSVR	95.0%	42.3	20.4	2	0	0	2	1,384.63
									ISEDKNPDYR	95.0%	27.4	22.0	0	1	0	2	1,236.59
									LAEPSQLLK	95.0%	35.7	17.5	1	0	0	2	998.59
									LLNDEDPVVVTK	95.0%	72.9	21.3	7	0	0	2	1,341.73
									LNYGIPAIVK	95.0%	52.9	18.1	16	0	0	2	1,087.65
									LVQNCLWTLR	95.0%	60.4	23.1	6	0	0	2	1,302.70
									NLALCPANHAPLQEAAVIPR	95.0%	46.1	19.4	0	2	0	2	2,155.14
									SAIVHLINYQDDAELATR	95.0%	27.0	21.8	0	1	0	2	2,029.04
									TMQNTSDLDTAR	95.0%	56.2	19.9	2	0	0	2	1,352.61
Cell adhesion molecule 4	CADM4_HUMAN	CADM4	42,766	99.90%	2	2	9	6.44%	FQLEEFSPR	95.0%	55.8	22.2	7	0	0	2	1,152.57
									LHQYDGSIVVIQNPAR	95.0%	35.9	20.4	0	2	0	2	1,809.96
Alpha-galactosidase A	AGAL_HUMAN	GLA	48,750	100.00%	5	5	19	13.50%	ALLQDKDVIAINQDPLGK	95.0%	43.7	17.5	0	4	0	2	1,951.09
									NFADIDDSWK	95.0%	38.2	19.7	2	0	0	2	1,210.54
									QEIGGPR	95.0%	38.7	20.7	2	0	0	2	756.40
									SILDWTSFNQER	95.0%	72.2	22.1	7	0	0	2	1,495.72
									SYTIAVASLGK	95.0%	34.2	19.6	4	0	0	2	1,109.62
CD44 antigen	CD44_HUMAN	CD44	81,535	100.00%	4	4	24	5.80%	ALSIGFETCR	95.0%	58.0	22.6	6	0	0	2	1,153.57
									ESSETPDQFMTADETR	95.0%	83.6	10.4	1	0	0	2	1,859.76
									FAGVFHVEK	95.0%	42.3	22.9	8	0	0	2	1,033.55
									TEAADLCK	95.0%	41.4	20.9	9	0	0	2	907.42
40S ribosomal protein S25	RS25_HUMAN	RPS25	13,725	99.50%	2	2	2	15.20%	AALQELLSK	95.0%	39.3	20.0	1	0	0	2	972.57
									LITPAVVSER	95.0%	35.6	18.6	1	0	0	2	1,084.64
Calpain-1 catalytic subunit	CAN1_HUMAN	CAPN1	81,875	100.00%	9	10	50	12.30%	APSDLYQIILK	95.0%	63.3	18.9	12	0	0	2	1,260.72
									GQVVSLIR	95.0%	43.3	21.0	3	0	0	2	871.54
									KAPSDLYQIILK	95.0%	72.3	16.7	12	0	0	2	1,388.82
									LYELIITR	95.0%	45.5	14.6	5	0	0	2	1,020.61
									NYLSIFR	95.0%	31.5	20.5	1	0	0	2	912.49
									NYPATFWVNPQFK	95.0%	70.7	22.6	6	0	0	2	1,611.80
									QLAGEDMEISVK	95.0%	59.1	22.4	2	0	0	2	1,335.65
									RPTELLSNPQFIVDGATR	95.0%	52.0	21.2	1	6	0	2	2,014.07
									YLGQDYEQLR	95.0%	58.2	21.4	2	0	0	2	1,284.62
Serpin B8	SPB8_HUMAN	SERPINB8	42,750	100.00%	5	5	12	18.40%	ADFSGMSTEK	95.0%	52.9	14.0	1	0	0	2	1,088.46
									GFQSLLSEVNR	95.0%	57.5	23.3	3	0	0	2	1,249.65
									ISEVLDAGTVDPLTK	95.0%	105.0	22.2	2	0	0	2	1,557.84
									LEESYDLEPFLR	95.0%	48.0	22.9	2	0	0	2	1,510.74
									LGMIDAFDEAK	95.0%	58.0	23.2	4	0	0	2	1,209.58
									LVLVNAIYFK	95.0%	32.0	14.0	4	0	0	2	1,179.71
Cytochrome c	CYC_HUMAN	CYCS	11,731	100.00%	6	7	20	49.50%	ADLIAYLK	95.0%	62.3	17.4	4	0	0	2	906.53
									GIIWGEDTLMEYLENPK	95.0%	93.6	22.5	4	0	0	2	2,023.97
									GIIWGEDTLMEYLENPKK	95.0%	98.9	22.0	2	0	0	2	2,136.07
									KTGQAPGYSYTAANK	95.0%	98.8	22.6	2	0	0	2	1,556.77
									TGPNLHGLFGR	95.0%	70.8	21.7	3	1	0	2	1,168.62

									TGQAPGYSYTAANK	95.0%	56.6	21.2	4	0	0	2	1,428.68
Superoxide dismutase [Cu-Zn]	SODC_HUMAN	SOD1	15,917	100.00%	2	2	5	16.90%	GDGPVQGIINFEQK	95.0%	69.4	23.0	3	0	0	2	1,501.77
									HVGDLGNVTADK	95.0%	71.4	22.4	2	0	0	2	1,225.62
Amidophosphoribosyltransferase	PUR1_HUMAN	PPAT	57,381	99.50%	2	2	4	4.26%	EVLPGEIVEISR	95.0%	61.1	20.1	2	0	0	2	1,340.74
									IVLVDDSIVR	95.0%	44.3	21.2	2	0	0	2	1,128.66
26S proteasome non-ATPase regulatory subunit 9	PSMD9_HUMAN	PSMD9	24,635	99.50%	2	3	5	9.42%	KLGQSESQGPPR	95.0%	39.4	21.9	2	1	0	2	1,283.67
									SDVDLYQVR	95.0%	55.9	21.7	2	0	0	2	1,094.55
Collagenase 3	MMP13_HUMAN	MMP13	53,804	100.00%	7	7	14	22.70%	DPGALMFPIYTYTGK	95.0%	48.5	23.1	4	0	0	2	1,689.82
									ENAASSMTER	94.5%	30.0	17.6	1	0	0	2	1,095.47
									IVNYTPDMTHSEVEK	95.0%	65.2	25.3	2	0	0	2	1,778.83
									LDDNTLDVMK	95.0%	34.8	22.9	1	0	0	2	1,163.56
									LIEEDFPGIGDKVDAVYEK	95.0%	27.2	21.8	0	1	0	2	2,137.07
									SHFMLPDDDVQGIQSLYGPGDEDPNPK	95.0%	54.0	18.5	0	1	0	2	2,987.34
									TLLFSGNQVWR	95.0%	56.5	22.8	4	0	0	2	1,320.71
UPF0424 protein C1orf128	CA128_HUMAN	C1orf128	24,160	100.00%	3	3	7	12.80%	DLTGELEYATK	94.9%	30.3	22.6	1	0	0	2	1,239.61
									GLAYGLYLR	95.0%	45.7	19.0	4	0	0	2	1,025.58
									VFYIGLR	95.0%	39.8	19.7	2	0	0	2	867.51
GDP-fucose protein O-fucosyltransferase 1	OFUT1_HUMAN	POFUT1	43,938	99.90%	2	2	3	6.70%	FGNQADHFLGSLAFAK	95.0%	48.4	22.7	0	2	0	2	1,722.86
									VISLEDFMEK	95.0%	38.8	22.8	1	0	0	2	1,210.60
Programmed cell death protein 5	PDCD5_HUMAN	PDCD5	14,267	99.50%	2	2	2	19.20%	AVENYLIQMAR	95.0%	40.3	22.7	1	0	0	2	1,307.68
									NSILAQVLDQSAR	95.0%	69.2	21.7	1	0	0	2	1,414.77
Partner of Y14 and mago	WIBG_HUMAN	WIBG	22,638	99.50%	2	2	6	14.20%	AAPTAASDQPDSAATTEK	95.0%	139.0	20.4	4	0	0	2	1,731.80
									IQAGEVSQPSK	95.0%	44.1	22.0	2	0	0	2	1,143.60
Apoptosis-inducing factor 1, mitochondrial	AIFM1_HUMAN	AIFM1	66,884	100.00%	4	4	10	8.48%	ALGTEVIQLFPEK	95.0%	38.2	20.1	3	0	0	2	1,444.81
									ELWFSDDPNVTK	95.0%	33.4	21.2	1	0	0	2	1,450.69
									SATEQSGTGIR	95.0%	59.8	23.1	4	0	0	2	1,106.54
									VMPNAIVQSVGVSSGK	95.0%	49.9	22.8	2	0	0	2	1,588.84
Glia maturation factor beta	GMFB_HUMAN	GMFB	16,696	99.50%	2	2	3	23.90%	LVVLDEELEGISPDELKDELPER	95.0%	64.2	21.4	0	2	0	2	2,637.35
									NTEDLTEEWLR	95.0%	63.8	21.5	1	0	0	2	1,405.66
Lupus La protein	LA_HUMAN	SSB	46,821	100.00%	7	7	25	19.90%	EVTWEVLEGEVEK	95.0%	57.3	22.4	4	0	0	2	1,546.76
									GSIFVVFDSIESAK	95.0%	68.4	21.6	8	0	0	2	1,498.78
									IIEDQQESLNK	95.0%	70.6	23.6	6	0	0	2	1,316.67
									LDEGWVPLEIMIK	95.0%	44.4	21.8	2	0	0	2	1,542.82
									NKEVTWEVLEGEVEK	95.0%	27.8	22.4	0	1	0	2	1,788.90
									QKLEEDAEMK	95.0%	39.0	21.7	0	2	0	2	1,236.58
									SPSKPLPEVTDEYKNDVK	95.0%	39.6	22.0	0	2	0	2	2,046.04
Platelet-activating factor acetylhydrolase IB subunit gamma	PA1B3_HUMAN	PAFAH1B3	25,716	100.00%	2	2	11	7.79%	AIVQLVNER	95.0%	45.2	20.0	2	0	0	2	1,041.61
									VVVLGLLPR	95.0%	71.8	3.0	9	0	0	2	965.65
Catalase	CATA_HUMAN	CAT	59,739	100.00%	4	4	8	11.40%	ADVLTTGAGNPVGDKLNVITVGPR	95.0%	50.0	17.2	0	2	0	2	2,364.29
									AFYVNVLNEEQR	95.0%	71.1	22.3	2	0	0	2	1,481.74
									FNTANDDNVTQVR	95.0%	72.0	22.4	2	0	0	2	1,493.70
									LSQEDPDYGIR	95.0%	40.2	22.1	2	0	0	2	1,292.61
Olfactomedin-like protein 2A	OLM2A_HUMAN	OLFML2A	73,037	100.00%	4	4	51	6.75%	LDPGDLSVHR	95.0%	58.1	22.3	4	0	0	2	1,108.58
									LGLQLLQK	95.0%	54.5	13.0	4	0	0	2	912.59
									SNSAEPNSAEQDEAEPR	95.0%	136.0	15.4	39	0	0	2	1,830.77
									VFGDLDQVR	95.0%	56.9	24.0	4	0	0	2	1,048.54
Gelsolin	GELS_HUMAN	GSN	85,680	100.00%	28	41	685	43.20%	AGALNSNDAFVLK	95.0%	84.6	23.0	66	0	0	2	1,319.70
									AGKEPGLQIWR	95.0%	57.0	19.8	3	26	0	2	1,254.70
									AQPVQVAEGSEPDGFWEALGGK	95.0%	117.0	21.4	53	5	0	2	2,272.09
									AVEVLPK	95.0%	56.8	14.5	20	0	0	2	755.47
									DPDQTDGLGLSYLSSHIANVER	95.0%	121.0	21.7	2	2	0	2	2,387.15

									DSQEEEKTEALTSAK	95.0%	101.0	22.8	38	18	0	2	1,665.78
									EPGLQIWR	95.0%	37.9	20.8	3	0	0	2	998.54
									EVQGFESATFLGYFK	95.0%	110.0	22.3	95	0	0	2	1,722.84
									GASQAGAPQGR	95.0%	69.2	20.8	11	0	0	2	999.50
									GGVASGFK	95.0%	43.4	22.5	7	0	0	2	722.38
									HVVPNEVVVQR	95.0%	57.0	20.5	20	11	0	2	1,275.72
									KGGVASGFK	95.0%	47.9	21.0	2	0	0	2	850.48
									LKATQVSK	95.0%	38.1	17.8	2	0	0	2	874.54
									NWRDPDQTDGLGLSYLSSHIANVER	95.0%	72.8	21.2	0	6	15	2	2,843.37
									QTQVSVLPEGGETPLFK	95.0%	91.1	21.2	61	1	0	2	1,829.97
									RTPITVVK	95.0%	31.0	17.0	2	0	0	2	913.58
									SEDCFILDHGK	95.0%	65.6	19.6	3	1	0	2	1,320.59
									SEDCFILDHGKDGK	95.0%	27.1	19.6	0	1	0	2	1,620.73
									TASDFITK	95.0%	46.1	20.8	2	0	0	2	882.46
									TGAQELLR	95.0%	74.2	23.7	18	0	0	2	887.50
									TPITVVK	95.0%	35.2	14.9	2	0	0	2	757.48
									TPSAAYLWVGTGASEAEK	95.0%	122.0	22.4	116	1	0	2	1,837.90
									VHVSEEGTEPEAMLQVLGPKPALPAGTEDTAK	95.0%	70.1	19.6	0	5	2	2	3,317.66
									VHVSEEGTEPEAMLQVLGPKPALPAGTEDTAKEDAANR	95.0%	48.7	19.2	0	0	5	2	3,957.95
									VPEARPNSMVVEHPEFLK	95.0%	61.4	21.9	0	10	1	2	2,095.06
									VPFDAATLHTSTAMAAQHGMDDDGTGQK	95.0%	65.3	18.5	0	20	9	2	2,889.28
									VSNGAGTMSVSLVADENPFAQGALK	95.0%	113.0	21.9	4	7	0	2	2,479.21
									YIETDPANR	95.0%	48.1	23.0	9	0	0	2	1,078.52
Peptidyl-prolyl cis-trans isomerase A	PPIA_HUMAN	PPIA	17,995	100.00%	13	17	257	72.70%	ALSTGEK	95.0%	35.6	26.1	1	0	0	2	705.38
									EGMNIVEAMER	95.0%	58.7	21.3	15	0	0	2	1,278.58
									FEDENFILK	95.0%	56.8	21.7	6	0	0	2	1,154.57
									HTGPGILSMANAGPNTNGSQFFICTAK	95.0%	76.5	21.5	0	4	0	2	2,791.33
									IIPGFMCQGGDFTR	95.0%	88.7	22.0	7	0	0	2	1,598.75
									MVNPTVFFDIAVDGEPLGR	95.0%	138.0	22.6	13	0	0	2	2,077.04
									SIYGEKFEDENFILK	95.0%	82.9	22.6	37	4	0	2	1,831.91
									TEWLDGK	95.0%	41.3	23.0	14	0	0	2	848.42
									TEWLDGKHVVFGK	95.0%	29.4	22.3	0	1	0	2	1,515.80
									VKEGMNIVEAMER	95.0%	86.5	22.7	21	12	0	2	1,505.75
									VNPTVFFDIAVDGEPLGR	95.0%	106.0	21.5	38	22	0	1	1,946.00
									VSFELFADK	95.0%	65.3	21.4	30	0	0	2	1,055.54
									VSFELFADKVPK	95.0%	66.9	20.3	19	13	0	2	1,379.76
Growth factor receptor-bound protein 2	GRB2_HUMAN	GRB2	25,189	100.00%	2	2	3	11.10%	ATADDELSFK	95.0%	30.5	22.4	1	0	0	2	1,096.52
									ESESAPGDFSLSVK	95.0%	49.4	22.1	2	0	0	2	1,452.69
Transcription intermediary factor 1-beta	TIF1B_HUMAN	TRIM28	88,531	100.00%	3	3	9	6.23%	IVAERPGTNSTGPAPMAPPR	95.0%	53.6	22.5	0	4	0	2	2,035.04
									LDLDLTADSQPPVFK	95.0%	69.6	22.0	2	0	0	2	1,658.86
									LSPPYSSPQEFAQDVGR	95.0%	84.4	23.1	3	0	0	2	1,877.90
26S proteasome non-ATPase regulatory subunit 3	PSMD3_HUMAN	PSMD3	60,962	100.00%	6	6	8	14.40%	AVQGFFTSNNATR	95.0%	90.8	22.2	1	0	0	2	1,412.69
									FNQVLDQFGEK	95.0%	49.1	22.3	1	0	0	2	1,324.65
									LQLDSPEDAEFIVAK	95.0%	39.1	22.9	2	0	0	2	1,674.86
									SLMPYFLLTQAVR	94.7%	30.1	19.8	1	0	0	2	1,538.84
									SVFPEQANNNEWAR	95.0%	38.8	21.0	1	0	0	2	1,661.77
									TAAAAAEHSQR	95.0%	61.7	21.1	2	0	0	2	1,112.54
Proprotein convertase subtilisin/kexin type 9	PCSK9_HUMAN	PCSK9	74,353	100.00%	19	26	261	35.00%	AGVVLVTAAGNFR	95.0%	48.4	19.7	8	0	0	2	1,274.72
									AHNAFGGEGVYAIAR	95.0%	79.6	22.5	9	12	0	2	1,532.76
									CAPDEELLSCSSFSR	95.0%	94.7	16.8	2	0	0	2	1,757.75
									DVINEAWFPEDQR	95.0%	74.4	21.0	22	0	0	2	1,618.75

									GTVSGTLIGLEFIR	95.0%	62.4	19.4	22	2	0	2	1,462.83
									HLAQASQELQ	95.0%	52.4	22.4	7	0	0	2	1,124.57
									ILHVFHGLLPGFLVK	95.0%	46.2	7.8	0	7	9	2	1,690.02
									LPGTYVVVLK	95.0%	38.2	12.8	3	0	0	2	1,088.67
									LPGTYVVVLKEETHLSQSER	95.0%	34.7	19.6	0	4	1	2	2,285.21
									LQAQAAR	95.0%	45.8	19.4	2	0	0	2	757.43
									MATAIAR	95.0%	44.8	24.2	6	0	0	2	749.40
									MSGDLLELALK	95.0%	88.1	23.0	12	0	0	2	1,189.65
									RLQAQAAR	95.0%	44.4	20.5	1	0	0	2	913.53
									SEEDGLAEAPEHGTTATFHR	95.0%	79.3	20.5	2	2	2	2	2,154.97
									SQLVQPVGPLVVLLPLAGGYSR	95.0%	108.0	10.4	68	19	0	2	2,263.32
									TVWSAHSGPTR	95.0%	59.0	22.3	8	0	0	2	1,198.60
									VLNAACQR	95.0%	43.5	24.2	3	0	0	2	931.48
									VLTPNLVAALPPSTHGAGWQLFCR	95.0%	29.5	19.4	0	3	0	2	2,605.37
									VMVTDFENVPEEDGTR	95.0%	108.0	20.8	25	0	0	2	1,837.83
Proteasome subunit alpha type-4	PSA4_HUMAN	PSMA4	29,467	100.00%	9	10	49	36.80%	ATCIGNNSAAAVSMLK	95.0%	80.9	22.7	4	0	0	2	1,623.78
									EGEMTLK	95.0%	31.1	21.0	1	0	0	2	823.39
									LLDEVFFSEK	95.0%	75.0	22.6	15	0	0	2	1,226.63
									LNEDMACSVAGITSDANVLTNELR	95.0%	128.0	21.5	2	0	0	2	2,609.22
									LSAEKVEIATLTR	95.0%	77.6	18.5	2	3	0	2	1,430.82
									QAYTQFGGK	95.0%	34.3	20.8	1	0	0	2	999.49
									SALALAIK	95.0%	62.0	17.9	9	0	0	2	786.51
									TTIFSPEGR	95.0%	42.0	23.3	8	0	0	2	1,007.52
									VEIATLTR	95.0%	54.7	20.5	4	0	0	2	902.53
Deoxyribonuclease-2-alpha	DNS2A_HUMAN	DNASE2	39,563	99.90%	2	2	5	6.11%	ALINSPEGAVGR	95.0%	64.5	21.4	3	0	0	2	1,183.64
									YLDESSGGWR	95.0%	76.3	21.4	2	0	0	2	1,169.52
N(G),N(G)-dimethylarginine dimethylaminohydrolase 2	DDAH2_HUMAN	DDAH2	29,626	100.00%	6	7	12	33.70%	DFAVSTVPVSGPSHLR	95.0%	50.8	21.5	2	1	0	2	1,668.87
									GAEIVADTFR	95.0%	51.6	23.6	2	0	0	2	1,078.55
									GGGDLPNSQEALQK	95.0%	52.2	22.5	2	0	0	2	1,413.70
									GVPESLASGEGAGAGLPALDLAK	95.0%	102.0	21.0	2	0	0	2	2,080.09
									IVEIGDENATLDGTDVLFTGR	95.0%	129.0	22.6	2	0	0	2	2,235.11
									TVVAGSSDAAQK	95.0%	64.5	23.1	1	0	0	2	1,133.58
Peptidyl-prolyl cis-trans isomerase B	PPIB_HUMAN	PPIB	23,725	100.00%	13	17	92	54.60%	DFMIQGGDFTR	95.0%	51.0	19.3	5	0	0	2	1,286.58
									DKPLKDVIIADCGK	95.0%	68.5	20.9	2	1	0	2	1,571.85
									DTNGSQFFITTVK	95.0%	96.1	22.0	4	0	0	2	1,457.73
									FPDENFK	95.0%	32.9	20.6	2	0	0	2	896.42
									HYGPGWVSMANAGK	95.0%	75.9	21.4	4	4	0	2	1,474.69
									IEVEKPFAIAK	95.0%	42.3	17.3	2	0	0	2	1,244.73
									IEVEKPFAIAKE	95.0%	70.6	20.4	7	4	0	2	1,373.77
									IGDEDVGR	95.0%	50.4	20.2	2	0	0	2	860.41
									SIYGERFPDENFK	95.0%	53.6	22.0	2	0	0	2	1,601.76
									TVDNFVALATGEK	95.0%	98.9	22.8	20	0	0	2	1,364.71
									VIFGLFGK	95.0%	52.6	18.7	13	0	0	2	880.53
									VIKDFMIQGGDFTR	95.0%	91.2	22.4	4	7	0	2	1,642.83
									VLEGMEVVR	95.0%	56.4	23.1	9	0	0	2	1,031.56
Beta-1,3-N-acetylglucosaminyltransferase	LFNG_HUMAN	LFNG	41,756	99.90%	2	3	11	5.28%	GPFSVEADPSR	95.0%	67.1	22.1	5	0	0	2	1,161.55
									LLASYPHTR	95.0%	45.0	22.1	4	2	0	2	1,057.58
Thioredoxin	THIO_HUMAN	TXN	11,719	100.00%	6	6	22	42.90%	EKLEATINELV	95.0%	49.1	22.7	8	0	0	2	1,258.69
									LEATINELV	95.0%	35.8	23.6	1	0	0	2	1,001.55
									MIKPFFHSLSEK	95.0%	65.2	22.6	4	0	0	2	1,479.77
									TAFQEALDAAGDK	95.0%	99.2	22.6	4	0	0	2	1,336.64

									VGEFSGANK	95.0%	56.0	22.5	3	0	0	2	908.45
									VGEFSGANKEK	95.0%	37.8	23.5	2	0	0	2	1,165.59
Transportin-1	TNPO1_HUMAN	TNPO1	102,341	100.00%	3	3	3	4.68%	ESQSPDTTIQR	94.7%	30.1	22.2	1	0	0	2	1,261.60
									GDVEEDETIPDSEQDIRPR	95.0%	40.7	20.5	0	1	0	2	2,200.00
									QSSFALLGDLTK	95.0%	60.1	21.9	1	0	0	2	1,279.69
Serine/threonine-protein phosphatase 5	PPP5_HUMAN	PPP5C	56,862	100.00%	9	9	16	23.80%	AALRDYETVVK	95.0%	37.3	20.8	1	0	0	2	1,264.69
									AASNMALGK	95.0%	39.6	23.4	2	0	0	2	878.44
									AEGYEVAHGGR	95.0%	41.2	19.8	1	0	0	2	1,145.53
									AFLEENNLDYIIR	95.0%	89.2	21.9	4	0	0	2	1,609.82
									AIAGDEHKR	95.0%	38.5	21.0	1	0	0	2	996.52
									AIELDKK	95.0%	39.4	22.6	2	0	0	2	816.48
									FYSQAIELNPSNAIYYGNR	95.0%	115.0	21.8	2	0	0	2	2,220.07
									GNHETDNMNQIYGFEGEVK	95.0%	41.3	17.1	0	1	0	2	2,197.95
									SVVDSLDIESMTIEDEYSGPK	95.0%	118.0	19.8	2	0	0	2	2,330.06
Small nuclear ribonucleoprotein Sm D1	SMD1_HUMAN	SNRPD1	13,264	99.50%	2	3	8	27.70%	NREPVQLETLSIR	95.0%	53.8	20.0	2	2	0	2	1,554.86
									YFILPDSLPLDTLLVDVEPK	95.0%	87.8	17.0	4	0	0	2	2,287.25
Guanine nucleotide-binding protein G(I)/G(S)/G(O) subunit gamma-12	GBG12_HUMAN	GNG12	7,989	99.50%	2	2	2	37.50%	SDPLLIGIPTSENPFK	95.0%	30.5	21.1	1	0	0	2	1,727.92
									TASTNNIAQAR	95.0%	49.0	23.5	1	0	0	2	1,146.59
Iron/zinc purple acid phosphatase-like protein	PAPL_HUMAN	PAPL	50,462	100.00%	3	3	6	10.00%	AQGTFVPFVDGGILR	95.0%	73.9	21.1	3	0	0	2	1,576.85
									LAVFGDLGADNPK	95.0%	45.9	23.4	1	0	0	2	1,316.69
									SEVQFGLQPSGPLPLR	95.0%	54.2	19.7	2	0	0	2	1,724.93
Ephrin-B1	EFNB1_HUMAN	EFNB1	37,989	100.00%	6	8	38	24.60%	AEAGRPYEYYK	95.0%	30.5	21.9	1	0	0	2	1,346.64
									FQEFSPNYMGLEFK	95.0%	73.7	21.1	8	0	0	2	1,752.79
									GGSGTAGTEPSDIIIPLR	95.0%	47.0	21.8	2	0	0	2	1,740.91
									GSLGDSDGKHETVNQEEK	95.0%	43.5	20.0	1	6	0	2	1,929.88
									IGDKLDIICPR	95.0%	42.1	21.1	1	7	0	2	1,299.71
									NLEPVSWSSLNPK	95.0%	89.0	22.7	12	0	0	2	1,470.76
L-lactate dehydrogenase A chain	LDHA_HUMAN	LDHA	36,671	100.00%	14	16	150	38.60%	DLADELALVDVIEDK	95.0%	106.0	21.3	25	0	0	2	1,657.85
									DQLIYNLLK	95.0%	64.7	20.4	6	0	0	2	1,119.64
									DQLIYNLLKEEQTPQNK	95.0%	76.1	21.9	2	2	0	2	2,074.08
									DYNVTANSK	95.0%	62.2	20.6	2	0	0	2	1,011.47
									FIIPNVVK	95.0%	36.9	17.9	3	0	0	2	929.58
									GEMMDLQHGSLFLR	95.0%	43.2	22.0	4	4	0	2	1,649.78
									KSADTLWGIQK	95.0%	35.2	21.7	2	0	0	2	1,246.68
									LNLVQR	95.0%	48.6	18.8	25	0	0	2	742.46
									LVIITAGAR	95.0%	73.5	17.0	22	0	0	2	913.58
									NVNIFK	95.0%	31.4	22.0	1	0	0	2	734.42
									QVVESAYEVIK	95.0%	56.5	21.8	4	0	0	2	1,264.68
									SADTLWGIQK	95.0%	64.5	23.0	11	0	0	2	1,118.58
									VIGSGCNLDSAR	95.0%	73.9	21.7	3	0	0	2	1,248.60
									VTLTSEEEAR	95.0%	66.1	23.7	34	0	0	2	1,134.56
Nidogen-2	NID2_HUMAN	NID2	151,235	100.00%	19	20	147	17.10%	AGLELGAEPETIVNSGLISPEGLAIDHIR	95.0%	48.4	17.7	0	2	0	2	2,971.57
									AIAVDPIR	95.0%	31.6	18.7	1	0	0	2	854.51
									DGVVSVNK	95.0%	38.5	24.1	2	0	0	2	817.44
									EDTSPAVLGLAAR	95.0%	73.1	21.3	16	0	0	2	1,299.69
									ELYHYSDSTVTSTSSR	95.0%	72.8	20.6	4	1	0	2	1,832.83
									ESYNVQLQLPAR	95.0%	57.1	22.5	9	0	0	2	1,417.74
									GEADDLKSEGPYFSLTSTEQSVK	95.0%	37.0	20.3	0	2	0	2	2,488.17
									GNLYWTDWNR	95.0%	36.9	21.4	4	0	0	2	1,324.61
									HAQAQYAYPGAR	95.0%	54.1	23.2	14	0	0	2	1,332.64
									HPSFPTTQQLNVDR	95.0%	53.7	22.5	0	16	0	2	1,639.82

									HSGQFTDEYLPEQR	95.0%	69.7	21.9	3	0	0	2	1,706.78
									IETSSLDGENRR	95.0%	29.0	21.8	0	2	0	2	1,376.68
									ITQTAEGLDPENYLSIK	95.0%	121.0	22.0	29	0	0	2	1,891.97
									KVLFYTDLVNPR	95.0%	79.4	18.3	1	0	0	2	1,464.82
									LANPLHFYEAR	95.0%	51.2	23.2	4	0	0	2	1,330.69
									MVYWTDVAGR	95.0%	54.0	22.0	7	0	0	2	1,197.57
									SEGPYFSLTSTEQSVK	95.0%	96.3	21.6	6	0	0	2	1,759.84
									VFALYNDEER	95.0%	57.1	21.2	17	0	0	2	1,255.60
									VLFYTDLVNPR	95.0%	64.7	20.8	10	0	0	2	1,336.73
									VLYREDTSPAVLGLAAR	95.0%	35.6	19.4	1	0	0	2	1,831.01
Nucleophosmin	NPM_HUMAN	NPM1	32,557	100.00%	9	10	143	38.80%	GPSSVEDIK	95.0%	67.2	21.7	31	0	0	2	931.47
									LLSISGK	95.0%	44.5	22.0	1	0	0	2	717.45
									MQASIEK	95.0%	40.3	22.8	7	0	0	2	822.40
									MSVQPTVSLGGFEITPPVVLR	95.0%	126.0	19.2	49	6	0	2	2,243.21
									MTDQEAIQDLWQWR	95.0%	86.8	24.6	26	0	0	2	1,835.84
									SKGQESFK	95.0%	34.3	21.5	1	0	0	2	910.46
									TVSLGAGAKDELHIVEAEAMNYEGSPIK	95.0%	69.0	21.1	0	2	0	2	2,929.46
									VDNDENEHQLSLR	95.0%	89.0	21.7	15	0	0	2	1,568.73
									VTLATLK	95.0%	37.8	15.1	5	0	0	2	745.48
Collagen alpha-6(IV) chain	CO4A6_HUMAN	COL4A6	163,794	100.00%	3	3	7	2.25%	GDSGSQGFR	95.0%	48.9	19.3	1	0	0	2	910.40
									GNPGPVGIPSPR	95.0%	39.2	22.8	2	0	0	2	1,147.62
									GPMGSEGVQGPPGQQGK	95.0%	79.3	21.0	4	0	0	2	1,626.76
Tropomodulin-3	TMOD3_HUMAN	TMOD3	39,578	99.90%	2	2	6	7.10%	FGYQFTQQGPR	95.0%	50.3	22.4	2	0	0	2	1,328.64
									SNDPVATAFAEMLK	95.0%	63.4	23.2	4	0	0	2	1,493.73
Aspartyl aminopeptidase	DNPEP_HUMAN	DNPEP	52,411	100.00%	3	3	4	4.84%	VKVPLQDLMVR	95.0%	30.4	19.1	0	1	0	2	1,313.76
									VPLQDLMVR	95.0%	37.8	22.7	2	0	0	2	1,086.60
									YASNAVSEALIR	95.0%	46.1	22.9	1	0	0	2	1,293.68
Xaa-Pro dipeptidase	PEPD_HUMAN	PEPD	54,530	100.00%	6	6	29	14.40%	AVYEAVLR	95.0%	33.9	21.0	1	0	0	2	920.52
									FEVNNTILHPEIVECR	95.0%	43.4	21.7	0	2	0	2	1,969.98
									KNPAVQAGSIVVLQGGEETQR	95.0%	61.1	19.0	0	2	0	2	2,181.16
									STLFVPR	95.0%	33.8	20.5	3	0	0	2	819.47
									TDMELEVLR	95.0%	63.4	23.7	3	0	0	2	1,121.55
									VPLALFALNR	95.0%	87.8	16.9	18	0	0	2	1,113.68
Filamin-B	FLNB_HUMAN	FLNB	278,141	100.00%	83	99	1267	41.70%	AAGSGELGVTMK	95.0%	70.7	23.0	24	0	0	2	1,136.56
									ADIEMPFDPSK	95.0%	73.7	19.9	30	0	0	2	1,265.57
									AEVSIQNNK	95.0%	41.1	22.9	5	0	0	2	1,002.52
									AGGPGLER	95.0%	79.5	20.7	30	0	0	2	756.40
									AGLAPLEVR	95.0%	49.7	17.9	15	0	0	2	925.55
									AGPGTLSVTIEGPSK	95.0%	70.1	22.1	18	0	0	2	1,413.76
									AHGPGLEGGLVGKPAEFTIDTK	95.0%	36.9	19.0	0	0	10	2	2,194.15
									APLNVQFNSPLPGDAVK	95.0%	79.2	21.3	35	0	0	2	1,766.94
									APSVATVGSICDLNLK	95.0%	106.0	21.6	2	0	0	2	1,644.86
									AWGPGLHGGIVGR	95.0%	48.6	22.7	2	0	0	2	1,276.69
									CLATGPGIASTVK	95.0%	60.6	23.6	7	0	0	2	1,274.68
									DAGEGLLAVQITDQEGKPK	95.0%	65.0	21.6	5	2	0	2	1,969.02
									DAGYGGISLAVEGPSK	95.0%	55.8	23.0	3	0	0	2	1,520.76
									DGTYAVTYIPDK	95.0%	41.7	21.7	3	0	0	2	1,342.65
									DGTYAVTYVPLTAGMYTLTMK	95.0%	40.9	21.3	1	1	0	2	2,328.11
									DLAEDAPWK	95.0%	31.6	20.5	1	0	0	2	1,044.50
									DLAEDAPWKK	95.0%	40.9	22.5	2	0	0	2	1,172.60
									DLDIIDNYDYSHTVK	95.0%	81.1	21.0	18	0	0	2	1,810.85

EAFTNKPNVFTVVTR	95.0%	63.6	20.9	11	43	0	2	1,722.92
EAGAGGLSIAVEGPSK	95.0%	80.8	22.5	7	0	0	2	1,442.75
EATTDFTVDSRPLTQVGGDHIK	95.0%	52.5	21.7	0	12	0	2	2,387.18
FADEHVPGSPFTVK	95.0%	41.6	22.1	3	6	0	2	1,530.76
FNDEHIPESPYLVPVIAPSDDAR	95.0%	36.7	21.4	1	3	0	2	2,581.26
FNGSHVVGSPFK	94.8%	26.4	22.9	0	1	0	2	1,275.65
FVPQEMGVHTVSVK	95.0%	65.5	23.6	6	0	0	2	1,573.80
GAGIGGLGITVEGPSESK	95.0%	85.3	22.0	25	0	0	2	1,628.85
GEAGVPAEFSIWTR	95.0%	75.9	23.6	17	0	0	2	1,519.75
GIEPTGNMVK	95.0%	37.8	22.4	5	0	0	2	1,061.53
GLEELVK	95.0%	31.9	21.8	1	0	0	2	787.46
GLVEPVNVVDNGDGTHTVTYTPSQEGPYMVSVK	95.0%	90.7	19.2	0	10	0	2	3,505.68
GQHVTGSPFQFTVGPLGEGGAHK	95.0%	43.6	21.8	0	4	10	2	2,308.15
HVGNQQYNVTYVVK	95.0%	52.0	23.3	2	0	0	2	1,648.84
IAGPGLGSGVR	95.0%	79.6	18.8	29	0	0	2	983.56
IFAQDGEGQR	95.0%	63.3	22.9	52	0	0	2	1,120.54
IFFAGDTIPK	95.0%	59.8	21.2	55	0	0	2	1,108.60
IGNLQTDLSDGLR	95.0%	72.4	22.5	17	0	0	2	1,401.73
IKVFGPGIEGK	95.0%	46.6	18.6	0	9	0	2	1,144.67
IPEINSSDMSAHVTSPSGR	95.0%	98.2	22.0	6	20	0	2	1,984.94
IPYLPITNFNQNWQDGK	95.0%	82.1	22.6	31	0	0	2	2,048.02
IQQNTFTR	95.0%	42.6	22.3	12	0	0	2	1,007.53
KGEITGEVHMPSGK	95.0%	40.4	21.8	1	4	0	2	1,485.74
LDVTILSPSR	95.0%	77.0	22.7	17	0	0	2	1,100.63
LDVTILSPSRK	95.0%	32.4	18.7	0	1	0	2	1,228.73
LIALLEVLSQK	95.0%	65.7	13.4	17	0	0	2	1,226.77
LKPGAPLKPK	95.0%	32.5	0.0	0	3	0	2	1,048.69
LLGWIQNK	95.0%	65.3	22.2	37	0	0	2	971.57
LNGLENR	95.0%	44.8	21.5	2	0	0	2	815.44
LPNNHIGISFIPR	95.0%	44.3	17.7	0	10	0	2	1,477.83
LTVMSLQESGLK	95.0%	58.7	22.5	8	0	0	2	1,321.70
LVSPGSANETSSILVESVTR	95.0%	129.0	21.3	35	3	0	2	2,046.07
NTVELLVEDK	95.0%	32.6	24.0	1	0	0	2	1,159.62
NTVELLVEDKGNQVYR	95.0%	33.3	21.8	0	3	0	2	1,876.98
SPFEVQVGPEAGMQK	95.0%	86.5	22.1	36	0	0	2	1,603.78
SPFEVSVDK	95.0%	41.2	21.8	7	0	0	2	1,007.51
SPFTVGVAAPLDLSK	95.0%	101.0	21.5	57	0	0	2	1,501.83
TATPEIVDNKDGTVTVR	95.0%	79.9	20.9	7	2	0	2	1,815.95
TFEMSDFIVDTR	95.0%	92.5	21.1	19	0	0	2	1,460.67
TGEEVGFVVDAK	95.0%	46.2	22.6	15	0	0	2	1,250.63
TYSVEYLPK	95.0%	41.8	22.3	3	0	0	2	1,099.57
VDPSHDASK	95.0%	35.2	20.1	1	0	0	2	955.45
VEVGKDQEFTVDTR	95.0%	87.8	22.9	12	13	0	2	1,622.80
VFGPGIEGK	95.0%	37.5	22.7	2	0	0	2	903.49
VKAEGPGLSK	95.0%	34.6	18.6	4	0	0	2	985.57
VKVDPSHDASK	95.0%	27.5	21.9	0	1	0	2	1,182.61
VKVEPAVDTSR	95.0%	45.3	22.3	3	14	0	2	1,200.66
VLFASQEIPASPFR	95.0%	85.8	22.5	38	0	0	2	1,561.84
VLQSFTVDSSK	95.0%	69.1	22.5	6	0	0	2	1,210.63
VLSEDEEDVDFDIIHNANDTFTVK	95.0%	31.5	20.5	0	1	0	2	2,765.28
VMYTPMAPGNYLISVK	95.0%	74.2	22.4	21	0	0	2	1,799.91

									VNIGQGSHPQK	95.0%	44.6	22.2	13	13	0	2	1,164.61
									VNQPASFAIR	95.0%	70.9	23.5	25	0	0	2	1,102.60
									VPVKDVVDPSK	95.0%	64.6	19.4	17	0	0	2	1,182.67
									VQAQGPGLK	95.0%	52.9	17.6	13	0	0	2	897.52
									VSYFPTVPGVYIVSTK	95.0%	58.5	20.3	30	0	0	2	1,756.95
									VTASGPGLSSYGVPASLPVDFAIDAR	95.0%	52.0	20.3	6	0	0	2	2,547.31
									VTEAEIVPMGK	95.0%	77.4	23.3	24	0	0	2	1,173.62
									VVASGPGLEHGK	95.0%	78.2	22.5	22	21	0	2	1,150.62
									VVPCLVTPVTGR	95.0%	45.7	19.3	6	0	0	2	1,297.73
									YADEEIPR	95.0%	52.8	22.1	7	0	0	2	992.47
									YGGELVPHFPAR	95.0%	41.9	22.1	3	30	0	2	1,342.69
									YGGPNHIVGSPFK	95.0%	59.3	22.3	4	2	0	2	1,372.70
									YMIGVTYGGDDIPLSPYR	95.0%	62.4	21.0	6	0	0	2	2,032.97
									YTPTQQGNMQVLVTYGGDPIPK	95.0%	92.5	21.2	9	0	0	2	2,423.19
Echinoderm microtubule-associated protein-like 2	EMAL2_HUMAN	EML2	70,659	100.00%	5	5	11	8.63%	DGTLVSGGGR	95.0%	61.7	23.9	3	0	0	2	918.46
									IIEDPAR	95.0%	35.8	19.7	1	0	0	2	813.45
									LQEVEVPEDFGPVR	95.0%	85.5	22.1	4	0	0	2	1,613.82
									QITSADAVR	95.0%	34.8	24.0	1	0	0	2	960.51
									TVAEGHGDTLYVGTTR	95.0%	74.3	22.4	2	0	0	2	1,676.82
Heterogeneous nuclear ribonucleoprotein D0	HNRPD_HUMAN	HNRNPD	38,417	100.00%	8	10	116	26.50%	ESESVDKVMDQK	95.0%	43.1	21.8	4	2	0	2	1,394.65
									EYFGGFGEVESIELPMDNK	95.0%	73.6	20.2	7	0	0	2	2,176.98
									FGEVVDCTLK	95.0%	55.7	22.7	13	0	0	2	1,167.57
									GFGFVLFK	95.0%	40.5	22.9	13	0	0	2	914.51
									IDASKNEEDEGHSNSSPR	95.0%	68.2	18.9	3	21	0	2	1,971.86
									IFVGGLSPDTPEEK	95.0%	91.8	23.0	45	0	0	2	1,488.76
									MFIGGLSWDTTK	95.0%	71.1	22.4	6	0	0	2	1,355.67
									MFIGGLSWDTTKK	95.0%	32.9	22.1	2	0	0	2	1,499.76
Single-stranded DNA-binding protein, mitochondrial	SSBP_HUMAN	SSBP1	17,242	99.50%	2	2	5	20.30%	NPVTIFSLATNEMWR	95.0%	81.9	22.4	3	0	0	2	1,778.89
									SGDSEVYQLGDVSQK	95.0%	100.0	21.4	2	0	0	2	1,611.75
Endoplasmic reticulum resident protein 44	ERP44_HUMAN	ERP44	46,955	100.00%	3	3	6	7.88%	DLAEITTLDR	95.0%	50.1	23.8	4	0	0	2	1,146.60
									SDPIQEIR	95.0%	31.9	20.8	1	0	0	2	957.50
									TPADCPVIAIDSFR	95.0%	37.0	22.8	1	0	0	2	1,561.77
Ubiquitin-conjugating enzyme E2 N	UBE2N_HUMAN	UBE2N	17,121	100.00%	3	3	5	23.70%	LELFLPEEYPMAAPK	95.0%	32.4	22.4	1	0	0	2	1,763.89
									LLAEPVPGIK	95.0%	34.7	11.8	2	0	0	2	1,036.64
									TNEAQAIETAR	95.0%	54.5	23.2	2	0	0	2	1,203.60
Transcriptional activator protein Pur-alpha	PURA_HUMAN	PURA	34,893	100.00%	2	2	3	11.20%	IAEVGAGGNK	95.0%	46.8	21.6	2	0	0	2	915.49
									LIDDYGVEEEPAELPEGTSLTVDNKR	95.0%	67.1	21.3	0	1	0	2	2,889.40
Cystatin-SN	CYTN_HUMAN	CST1	16,344	99.50%	2	2	3	24.80%	IIPGGIYNADLNDEWVQR	95.0%	89.7	22.4	2	0	0	2	2,073.04
									QQTVGGVNYFFDVEVGR	95.0%	33.1	21.6	1	0	0	2	1,914.94
Ceruloplasmin	CERU_HUMAN	CP	122,190	100.00%	9	9	29	11.50%	ALYLQYTDETFR	95.0%	66.1	23.4	2	0	0	2	1,519.74
									DIASGLIGPLIICK	95.0%	53.3	19.3	2	0	0	2	1,469.84
									DIFTGLIGPMK	95.0%	33.7	23.0	1	0	0	2	1,207.64
									EYTDASFTNR	95.0%	51.8	17.0	3	0	0	2	1,203.53
									GAYPLSIEPIGVR	95.0%	56.2	22.0	9	0	0	2	1,371.76
									MFTTAPDQVDKEDEDFQESNK	95.0%	38.4	15.7	0	2	0	2	2,490.06
									NNEGTYYSPNYNPQSR	95.0%	71.4	17.5	4	0	0	2	1,903.82
									QSEDSTFYLGER	95.0%	69.1	20.2	2	0	0	2	1,431.64
									VNKDDEEFIESNK	95.0%	88.5	21.3	4	0	0	2	1,566.73
Beta-2-microglobulin	B2MG_HUMAN	B2M	13,697	100.00%	5	10	224	42.90%	IEKVEHSDLSFSK	95.0%	92.9	23.0	5	3	9	2	1,518.78
									IQVYSR	95.0%	39.4	21.9	12	0	0	2	765.43
									SNFLNCYVSGFHPSDIEVDLLK	95.0%	44.9	21.4	7	37	0	2	2,554.23

									VEHSDLSFSK	95.0%	60.7	23.1	30	17	0	2	1,148.56
									VNHVTLSQPK	95.0%	59.6	20.3	46	58	0	2	1,122.63
Adenosylhomocysteinase	SAHH_HUMAN	AHCY	47,699	100.00%	18	24	142	39.60%	AGIPVYAWK	95.0%	42.1	22.9	1	0	0	2	1,004.56
									ALDIAENEMPGLMR	95.0%	77.5	22.4	11	0	0	2	1,575.75
									ATDVMIAGK	95.0%	35.1	24.0	1	0	0	2	905.48
									DGPLNMILDDGGDLTNLIHTK	95.0%	111.0	22.6	4	29	0	2	2,268.12
									ESLIDGIK	95.0%	41.5	23.6	1	0	0	2	874.49
									ESLIDGIKR	95.0%	40.9	20.9	3	0	0	2	1,030.59
									GISEETTTGVHNLYK	95.0%	80.5	23.2	7	1	0	2	1,648.82
									IILLAEGR	95.0%	41.5	16.3	6	0	0	2	884.56
									KALDIAENEMPGLMR	95.0%	71.3	22.1	2	4	0	2	1,719.84
									KLDEAVAEAHLGK	95.0%	91.3	21.4	2	3	0	2	1,380.75
									LDEAVAEAHLGK	95.0%	59.9	22.1	2	2	0	2	1,252.65
									VADIGLAAWGR	95.0%	93.8	22.3	13	0	0	2	1,128.62
									VAVVAGYGDVGK	95.0%	73.9	22.7	4	0	0	2	1,134.62
									VNIKPQVDR	95.0%	33.1	18.7	1	0	0	2	1,068.62
									VPAINVNDSVTK	95.0%	76.6	22.2	15	0	0	2	1,256.69
									WLNENAVEK	95.0%	50.6	22.6	7	0	0	2	1,102.55
									YPQLLPGIR	95.0%	51.3	20.4	17	0	0	2	1,056.62
									YPVGVHFLPK	95.0%	40.6	21.0	2	4	0	2	1,156.65
Aspartyl-tRNA synthetase, cytoplasmic	SYDC_HUMAN	DARS	57,119	100.00%	5	5	10	14.20%	ATVNQDTR	95.0%	34.7	23.1	3	0	0	2	904.45
									EAGVEMGDEDDLSTPNEK	95.0%	40.6	14.9	1	0	0	2	1,951.81
									FGAPPHAGGGIGLER	95.0%	44.9	22.3	0	2	0	2	1,435.74
									IYVISLAEPR	95.0%	52.1	19.7	2	0	0	2	1,160.67
									LPLQLDDAVRPEAEGEEEGR	95.0%	36.1	22.4	0	2	0	2	2,223.09
Nuclear migration protein nudC	NUDC_HUMAN	NUDC	38,226	100.00%	8	8	20	27.50%	ELTDEEAER	95.0%	55.5	19.2	6	0	0	2	1,091.49
									FMDQHPEMDFSK	95.0%	44.4	12.3	2	0	0	2	1,543.62
									GQPAIIDGELYNEVK	95.0%	75.5	22.0	2	0	0	2	1,645.84
									LKPNLGNGADLPNYR	95.0%	27.0	21.2	0	1	0	2	1,641.87
									LSDLDSETR	95.0%	36.0	22.4	2	0	0	2	1,035.50
									LVSSDPEINTK	95.0%	61.6	23.9	3	0	0	2	1,202.63
									SETSGPQIK	95.0%	48.3	23.8	1	0	0	2	946.48
									SMGLPTSDEQK	95.0%	34.9	21.0	3	0	0	2	1,208.55
Procollagen-lysine,2-oxoglutarate 5-dioxygenase 3	PLOD3_HUMAN	PLOD3	84,769	100.00%	16	16	77	27.00%	AVMNFVVR	95.0%	71.5	22.8	4	0	0	2	935.51
									DVFSGSDTDPDMAFCK	95.0%	51.7	14.1	1	0	0	2	1,791.72
									IFQNLNGALDEVVLK	95.0%	124.0	19.6	5	0	0	2	1,672.93
									KFVQSGSR	95.0%	38.1	21.1	1	0	0	2	908.50
									LAGGYENVPTVDIHMK	95.0%	38.5	22.1	0	6	0	2	1,743.87
									LLLLDYPPDR	95.0%	56.1	22.3	17	0	0	2	1,214.68
									LSLNLDHK	95.0%	31.7	17.4	1	0	0	2	939.53
									LVGPEEALSPGEAR	95.0%	95.0	21.9	13	0	0	2	1,424.74
									LYLDPGLR	95.0%	53.6	21.7	4	0	0	2	946.54
									NVAYDTLPIVVHGNGPTK	95.0%	69.0	21.0	3	0	0	2	1,895.00
									QVGYEDQWLQLLR	95.0%	55.4	22.9	5	0	0	2	1,647.85
									SEDYVELVQR	95.0%	70.9	22.2	6	0	0	2	1,237.61
									TLGLGEEWR	95.0%	55.4	25.1	4	0	0	2	1,060.54
									TYVGPMTESLFPGYHTK	95.0%	50.8	22.2	1	0	0	2	1,927.93
									VGVWNVPYISQAYVIR	95.0%	59.7	19.3	5	0	0	2	1,864.01
									YKDDDDDQLFYTR	95.0%	75.3	19.9	1	0	0	2	1,693.73
Talin-1	TLN1_HUMAN	TLN1	269,747	100.00%	6	6	11	3.19%	AVAEQIPLLVQGVR	95.0%	39.6	14.5	1	0	0	2	1,492.89
									GLAGAVSELLR	95.0%	46.0	21.2	4	0	0	2	1,085.63

									ILAQATSDLVNAIK	95.0%	49.6	17.9	2	0	0	2	1,456.84
									LNEAAAGLNQAATELVQASR	95.0%	84.8	20.8	1	0	0	2	2,027.05
									NLGTALAELR	95.0%	47.1	21.6	2	0	0	2	1,057.60
									VLVQNAAGSQEK	95.0%	40.0	22.2	1	0	0	2	1,243.66
DNA replication licensing factor MCM6	MCM6_HUMAN	MCM6	92,873	100.00%	6	6	8	7.92%	ESEDFIVEQYK	95.0%	32.0	21.2	1	0	0	2	1,386.64
									GVLLMLFGGVPK	94.5%	30.0	18.4	1	0	0	2	1,246.72
									IQETQAELPR	95.0%	61.6	22.3	2	0	0	2	1,184.63
									ISGQVVR	95.0%	46.3	23.8	1	0	0	2	758.45
									SELVNWYLK	95.0%	38.6	22.4	2	0	0	2	1,151.61
									TSILAAANPISGHYDR	94.6%	26.2	22.1	0	1	0	2	1,685.86
Sodium-coupled neutral amino acid transporter 2	S38A2_HUMAN	SLC38A2	56,011	99.50%	2	2	4	6.32%	TANEGGSLLYEQLGYK	95.0%	107.0	22.3	2	0	0	2	1,742.86
									YELPLVIQALTNIEDK	95.0%	96.1	19.9	2	0	0	2	1,859.02
RuvB-like 1	RUVB1_HUMAN	RUVBL1	50,211	100.00%	7	8	22	20.80%	ALESSIAPIVIFASNR	95.0%	71.9	19.8	6	3	0	2	1,687.94
									AVLLAGPPGTGK	95.0%	33.0	16.7	1	0	0	2	1,080.64
									GLGLDESGLAK	95.0%	45.4	23.1	1	0	0	2	1,059.57
									GTEDITSPHGIPLDLLDR	95.0%	39.5	22.3	0	4	0	2	1,949.00
									LDPSIFESLQK	95.0%	50.7	23.2	2	0	0	2	1,276.68
									QAASGLVGQENAR	95.0%	57.9	22.1	3	0	0	2	1,300.66
									YSVQLLTPANLLAK	95.0%	55.1	16.2	2	0	0	2	1,530.89
Protein S100-P	S100P_HUMAN	S100P	10,382	99.50%	2	2	10	31.60%	MTELETAMGMIIDVFSR	95.0%	77.8	20.4	4	0	0	2	1,991.91
									YSGSEGSTQTLTK	95.0%	97.0	21.8	6	0	0	2	1,358.64
Cysteinyl-tRNA synthetase, cytoplasmic	SYCC_HUMAN	CARS	85,458	100.00%	5	5	8	7.62%	APVDITGQFEK	95.0%	39.0	23.1	2	0	0	2	1,204.62
									DNILPELGVR	95.0%	32.9	20.8	1	0	0	2	1,125.63
									FEDHEGLPTVVK	95.0%	31.5	21.8	0	2	0	2	1,370.70
									IQHAVQLATEPLEK	95.0%	39.9	20.2	0	1	0	2	1,576.87
									WGEEEAELNK	95.0%	45.6	20.2	2	0	0	2	1,204.55
V-type proton ATPase catalytic subunit A	VATA_HUMAN	ATP6V1A	68,287	100.00%	2	2	4	5.19%	LAEMPADSGYPAYLGAR	95.0%	50.9	21.8	2	0	0	2	1,797.85
									TALVANTSNMPVAAR	95.0%	93.9	23.3	2	0	0	2	1,531.79
Elongation factor 1-delta	EF1D_HUMAN	EEF1D	31,104	100.00%	8	10	63	37.00%	ATAPQTQHVSPMR	95.0%	44.4	23.4	7	2	0	2	1,439.71
									FYEQMNGPVAGASR	95.0%	90.3	20.5	9	0	0	2	1,542.70
									GVVQELQQAISK	95.0%	79.0	20.9	13	0	0	2	1,299.73
									IASLEVENQSLR	95.0%	90.1	22.1	10	0	0	2	1,358.73
									KPALVAK	95.0%	39.0	12.0	1	0	0	2	726.49
									LVPVGYGIR	95.0%	47.2	19.5	4	0	0	2	973.58
									SIQLDGLVWGASK	95.0%	82.6	22.0	8	0	0	2	1,373.74
									SLAGSSGPGASSGTSGDHGELVVR	95.0%	108.0	21.3	1	8	0	2	2,185.05
14-3-3 protein eta	1433F_HUMAN	YWHAH	28,202	100.00%	9	10	41	58.90%	AVTELNEPLSNEDR	95.0%	86.6	22.5	4	0	0	2	1,586.77
									AVTELNEPLSNEDRNLLSVAYK	95.0%	32.5	20.8	0	3	0	2	2,475.27
									DNLTLWTSDQQDEEAGEGN	95.0%	32.5	15.6	1	0	0	2	2,121.89
									DSTLIMQLLR	95.0%	79.3	21.3	113	0	0	2	1,189.66
									ELETVCNDVLSLLDK	95.0%	96.4	22.6	6	0	0	2	1,747.88
									KNSVVEASEAAYK	95.0%	53.8	22.5	2	0	0	2	1,395.71
									LAEQAER	95.0%	59.4	21.6	36	0	0	2	816.42
									MKGDYYR	95.0%	42.7	19.2	6	0	0	2	932.43
									NLLSVAYK	95.0%	51.4	19.1	49	0	0	2	907.53
									NLLSVAYKNVVGAR	95.0%	79.1	17.7	4	2	0	2	1,503.86
									NSVVEASEAAYK	95.0%	71.3	22.3	7	0	0	2	1,267.62
									QAFDDAIAELDTLNEDSYK	95.0%	113.0	21.6	4	0	0	2	2,157.98
									VISSIEQK	95.0%	65.9	22.9	27	0	0	2	903.52
									YDDMASAMK	95.0%	53.8	9.5	9	0	0	2	1,063.41
									YLAEVASGEKK	95.0%	46.7	21.2	4	1	0	2	1,194.64

Early endosome antigen 1	EEA1_HUMAN	EEA1	162,450	100.00%	5	5	12	3.90%	AAQLATEIADIK	95.0%	54.7	22.3	3	0	0	2	1,243.69
									IQNLEALLQK	95.0%	51.3	18.1	4	0	0	2	1,169.69
									ITTQLDQVTAK	95.0%	47.0	22.1	2	0	0	2	1,217.67
									LSASETSLHR	95.0%	46.5	22.6	1	0	0	2	1,100.57
									LSLAQEDLISNR	95.0%	56.7	21.8	2	0	0	2	1,358.73
Protein S100-A8	S10A8_HUMAN	S100A8	10,817	99.50%	2	2	9	23.70%	ALNSIIDVYHK	95.0%	36.6	21.9	1	0	0	2	1,272.70
									LLETECPQYIR	95.0%	72.0	23.3	8	0	0	2	1,421.71
Interleukin enhancer-binding factor 2	ILF2_HUMAN	ILF2	43,045	100.00%	7	9	27	23.80%	ILITTVPPNLR	95.0%	36.2	11.5	3	0	0	2	1,236.77
									ILPTLEAVAALGNK	95.0%	90.0	14.1	9	0	0	2	1,409.84
									NQDLAPNSAEQASILSLVTK	95.0%	130.0	20.6	4	2	0	2	2,099.10
									QPLALNVAYR	95.0%	37.5	21.8	2	0	0	2	1,144.65
									VKPAPDETSFSEALLK	95.0%	60.0	21.6	2	1	0	2	1,731.92
									VLQSALAAIR	95.0%	68.8	18.1	2	0	0	2	1,041.64
									WFEENASQSTVK	95.0%	72.7	21.8	2	0	0	2	1,425.67
Ephrin type-B receptor 4	EPHB4_HUMAN	EPHB4	108,252	99.50%	2	2	2	2.53%	SSPSSLSLAWAVPR	95.0%	39.7	21.8	1	0	0	2	1,457.78
									VDTVAAEHLTR	95.0%	47.2	21.5	1	0	0	2	1,211.64
Pyruvate kinase isozymes M1/M2	KPYM_HUMAN	PKM2	57,920	100.00%	38	50	933	67.60%	AGKPVICATQMLESMIK	95.0%	48.7	22.4	1	7	0	2	1,908.96
									APIIAVTR	95.0%	59.2	14.3	18	0	0	2	840.53
									ASDVHEVR	95.0%	60.2	21.1	10	0	0	2	912.45
									CCSGAIIVLTK	95.0%	52.2	22.8	2	0	0	2	1,221.63
									CDENILWLDYK	95.0%	57.7	22.0	5	0	0	2	1,468.68
									DIQDLK	95.0%	42.7	24.4	2	0	0	2	731.39
									DPVQEAWAEDVDLR	95.0%	103.0	21.9	15	0	0	2	1,642.77
									FDEILEASDGIMVAR	95.0%	41.4	21.9	1	0	0	2	1,665.82
									FGVEQDVDMVFASFIR	95.0%	120.0	22.4	24	16	0	2	1,875.90
									GADFLVTEVENGGSLGSK	95.0%	124.0	21.8	66	0	0	2	1,779.88
									GADFLVTEVENGGSLGSKK	95.0%	54.9	22.2	2	2	0	2	1,907.97
									GDLGIEIPAEK	95.0%	67.3	21.5	32	0	0	2	1,141.61
									GDYPLEAVR	95.0%	50.4	22.7	22	0	0	2	1,019.52
									GIFPVLCKDPVQEAWAEDVDLR	95.0%	50.7	20.5	0	3	0	2	2,557.28
									GSGTAEVELK	95.0%	60.2	23.3	8	0	0	2	990.51
									GSGTAEVELKK	95.0%	65.3	22.3	28	4	0	2	1,118.61
									GVNLPGAAVDLPAVSEK	95.0%	96.0	20.1	15	0	0	2	1,636.89
									GVNLPGAAVDLPAVSEKDIQDLK	95.0%	81.9	17.8	6	14	0	2	2,349.27
									IENHEGVR	95.0%	54.0	22.5	15	0	0	2	953.48
									ITLDNAYMEK	95.0%	68.6	22.6	40	0	0	2	1,197.58
									IYVDDGLISLQVK	95.0%	109.0	19.4	87	0	0	2	1,462.82
									KGVNLPGAAVDLPAVSEK	95.0%	84.7	17.8	6	8	0	2	1,764.99
									LAPITSDPTEATAVGAVEASFK	95.0%	159.0	21.2	87	45	0	2	2,175.12
									LDIDSPPITAR	95.0%	69.5	21.5	60	0	0	2	1,197.65
									LNFSHGTHEYHAETIK	95.0%	35.6	22.2	0	1	1	2	1,883.90
									MQHLIAR	95.0%	41.5	20.8	8	0	0	2	884.48
									NTGIICTIGPASR	95.0%	75.5	23.2	10	0	0	2	1,359.71
									QKGADFLVTEVENGGSLGSK	95.0%	86.0	22.2	2	4	0	2	2,036.03
									RFDEILEASDGIMVAR	95.0%	96.0	22.5	35	84	0	2	1,821.92
									RLAPITSDPTEATAVGAVEASFK	94.8%	26.5	20.5	0	1	0	2	2,331.22
									SAHQVAR	95.0%	33.1	20.0	2	0	0	2	768.41
									SGMNVAR	95.0%	70.1	23.8	4	0	0	2	734.36
									SVETLKEMIK	95.0%	42.2	21.2	7	1	0	2	1,193.65
									TATESFASDPILYRPVAVALDTK	95.0%	63.6	19.1	10	77	0	2	2,465.29
									TATESFASDPILYRPVAVALDTKGPEIR	95.0%	58.4	17.9	0	5	0	2	3,017.60

									VFLAQK	95.0%	35.6	19.8	2	0	0	2	705.43
									VNFAMNVGK	95.0%	61.9	22.0	27	0	0	2	995.50
									VVEVGSK	95.0%	32.8	25.5	1	0	0	2	717.41
Hepatocyte growth factor receptor	MET_HUMAN	MET	155,525	100.00%	19	20	90	14.50%	AFFMLDGILSK	95.0%	78.7	21.1	12	0	0	2	1,241.66
									EDPIVYEIHPTK	95.0%	69.7	22.7	2	0	0	2	1,440.74
									ETLDAQTFHTR	95.0%	68.7	22.2	2	0	0	2	1,318.64
									ETSIFSYR	95.0%	44.2	20.5	2	0	0	2	1,002.49
									ETSIFSYREDPIVYEIHPTK	95.0%	55.3	20.9	0	4	0	2	2,424.21
									EVFNILQAAYVSKPGAQLAR	95.0%	83.9	18.2	1	8	0	2	2,175.19
									FMQVVVSR	95.0%	57.0	22.6	8	0	0	2	965.52
									GDLTIANLGTSEGR	95.0%	106.0	22.6	14	0	0	2	1,403.71
									GNDIDPEAVKGEVLK	95.0%	74.4	21.3	6	0	0	2	1,583.83
									IDLANR	95.0%	39.5	22.7	2	0	0	2	701.39
									KEVFNILQAAYVSKPGAQLAR	95.0%	31.4	14.1	0	0	2	2	2,303.29
									MVINVHEAGR	95.0%	40.6	22.6	2	0	0	2	1,141.58
									NLNSVSVPR	95.0%	65.2	20.6	6	0	0	2	985.54
									QAISSTVLGK	95.0%	50.1	19.4	1	0	0	2	1,003.58
									SFISGGSTITGVGK	95.0%	107.0	21.3	4	0	0	2	1,310.70
									SVSNSILECYTPAQTISTEFAVK	95.0%	45.9	21.6	2	0	0	2	2,545.25
									TEFTTALQR	95.0%	56.9	22.3	7	0	0	2	1,066.55
									VFPNSAPLEGGTR	95.0%	39.5	22.3	2	0	0	2	1,344.69
									YVNDFFNK	95.0%	42.0	22.0	3	0	0	2	1,046.49
Sulfatase-modifying factor 2	SUMF2_HUMAN	SUMF2	33,825	100.00%	2	2	3	8.31%	MGNTPDSASDNLGFR	95.0%	56.5	19.2	1	0	0	2	1,581.70
									QPAGPGSGIR	95.0%	41.1	21.8	2	0	0	2	939.50
Tyrosine-protein kinase receptor UFO	UFO_HUMAN	AXL	98,317	100.00%	3	4	59	4.59%	APLQGTLLGYR	95.0%	90.9	21.2	26	0	0	2	1,188.67
									LAYQGQDTPEVLMDIGLR	95.0%	102.0	22.9	21	1	0	2	2,035.02
									TATITVLPQQPR	95.0%	50.3	16.8	11	0	0	2	1,324.76
Omega-amidase NIT2	NIT2_HUMAN	NIT2	30,591	100.00%	3	3	6	16.30%	AVDNQVYVATASPAR	95.0%	92.6	22.1	4	0	0	2	1,561.80
									ECSIYLIGGSIPEEDAGK	95.0%	66.7	21.7	1	0	0	2	1,937.92
									LALIQLQISSIK	95.0%	33.5	10.8	1	0	0	2	1,326.84
Exosome complex exonuclease RRP4	EXOS2_HUMAN	EXOSC2	32,771	99.90%	2	2	2	11.30%	LDSVLLLSSMNLPGGELR	95.0%	33.4	20.6	1	0	0	2	1,930.03
									LGQGVLVQVSPSLVK	95.0%	50.2	13.4	1	0	0	2	1,523.92
Ephrin-A1	EFNA1_HUMAN	EFNA1	23,769	99.90%	2	2	13	11.20%	ITHSPQAHDNPQEK	95.0%	26.1	22.3	0	0	3	2	1,601.77
									LAADDPEVR	95.0%	54.3	20.5	10	0	0	2	985.50
GTP-binding nuclear protein Ran	RAN_HUMAN	RAN	24,405	100.00%	6	6	27	35.60%	FNVWDTAGQEK	95.0%	79.9	21.6	13	0	0	2	1,294.61
									LVLVGDGGTGK	95.0%	58.2	21.5	6	0	0	2	1,015.58
									NLQYYDISAK	95.0%	33.7	22.4	3	0	0	2	1,214.61
									SNYNFEKPFLWLAR	95.0%	35.9	21.3	1	0	0	2	1,784.91
									VCENIPIVLCGNK	95.0%	73.2	22.5	2	0	0	2	1,515.77
									YVATLGVEVHPLVFHTNR	95.0%	22.0	19.8	0	0	2	2	2,052.10
Dipeptidyl peptidase 2	DPP2_HUMAN	DPP7	54,325	99.50%	2	2	6	4.88%	DLFLQGAYDTVR	95.0%	48.5	22.1	3	0	0	2	1,397.71
									DVTADFEGQSPK	95.0%	61.6	21.4	3	0	0	2	1,293.60
DnaJ homolog subfamily B member 11	DJB11_HUMAN	DNAJB11	40,497	100.00%	6	6	13	21.80%	DGMEYPFIGEGEPHVDGEPGDLR	94.9%	26.6	17.2	0	1	0	2	2,532.10
									FQDLGAAYEVLSDSEK	95.0%	128.0	21.8	4	0	0	2	1,771.84
									FQDLGAAYEVLSDSEKR	95.0%	32.0	22.0	0	2	0	2	1,927.94
									LALQLHPDRNPDDPQAQEK	95.0%	34.8	22.1	0	1	0	2	2,185.10
									TLEVEIEPGVR	95.0%	51.0	20.9	2	0	0	2	1,241.67
									TTQLGPGR	95.0%	34.4	21.6	3	0	0	2	829.45
Proteasome activator complex subunit 2	PSME2_HUMAN	PSME2	27,384	100.00%	4	4	12	23.00%	AFYAELYHIISSNLEK	95.0%	56.2	21.9	2	0	0	2	1,897.97
									IEDGNDFGVAIQEK	95.0%	110.0	22.7	3	0	0	2	1,534.74
									QNLFQEAEEFLYR	95.0%	76.2	21.6	4	0	0	2	1,686.81

									TKVEAFQTTISK	95.0%	76.5	19.5	3	0	0	2	1,352.74
Perilipin-3	PLIN3_HUMAN	PLIN3	47,028	100.00%	3	3	8	10.40%	IATSLDGFDVASVQQQR	95.0%	89.0	22.1	3	0	0	2	1,834.93
									TLTAAAVSGAQPILSK	95.0%	42.1	18.3	2	0	0	2	1,527.87
									VSGAQEMVSSAK	95.0%	56.8	22.6	3	0	0	2	1,209.58
Phospholipid transfer protein	PLTP_HUMAN	PLTP	54,723	100.00%	9	11	112	24.70%	AGALQLLLVGDK	95.0%	86.8	16.6	16	0	0	2	1,197.72
									ATYFGSIVLLSPAVIDSPLK	95.0%	63.0	14.1	6	0	0	2	2,091.17
									AVEPQLQEEER	95.0%	64.2	22.7	31	0	0	2	1,327.65
									DPVASTSNLDMDFR	95.0%	71.0	18.3	3	0	0	2	1,583.70
									FLEQELETITIPDLR	95.0%	64.4	21.5	9	2	0	2	1,816.97
									TGLELSRDPAGR	95.0%	38.3	21.7	1	4	0	2	1,271.67
									TMLQIGVMPMLNER	95.0%	85.6	23.2	11	0	0	2	1,632.83
									VPHDLDMLLR	95.0%	36.2	22.4	0	7	0	2	1,208.65
									VYDFLSTFITSGMR	95.0%	77.6	22.7	22	0	0	2	1,636.80
Argininosuccinate lyase	ARLY_HUMAN	ASL	51,641	99.50%	2	2	3	7.33%	INVLPLGSGAIAGNPLGVDR	95.0%	101.0	16.8	2	0	0	2	1,933.09
									LNSNDEDIHTANER	95.0%	36.0	19.6	0	1	0	2	1,627.73
Complement factor H	CFAH_HUMAN	CFH	139,078	100.00%	35	40	244	34.20%	AGEQVTYTCATYYK	95.0%	54.7	20.8	5	0	0	2	1,654.74
									AQTTVTCMENGWSPTPR	95.0%	38.6	20.0	1	0	0	2	1,951.86
									CFEGFGIDGPAIAK	95.0%	83.5	22.3	9	0	0	2	1,481.71
									CNMGYEYSER	95.0%	58.2	7.8	4	0	0	2	1,324.49
									CTSTGWIPAPR	95.0%	51.9	22.1	2	0	0	2	1,245.61
									DGWSAQPTCIK	95.0%	34.1	21.0	1	0	0	2	1,262.58
									DTSCVNPPTVQNAYIVSR	95.0%	82.4	22.3	4	0	0	2	2,020.98
									ECDTDGWTNDIPICEVVK	95.0%	60.8	17.6	1	0	0	2	2,150.94
									ECELPK	95.0%	30.4	20.5	1	0	0	2	775.37
									EFDHNSNIR	95.0%	57.0	20.3	7	0	0	2	1,131.52
									EIMENYNIALR	95.0%	85.2	22.7	20	0	0	2	1,365.68
									EQVQSCGPPPELLNGNVK	95.0%	74.1	22.2	4	0	0	2	1,965.97
									FVCNSGYK	95.0%	34.0	18.6	2	0	0	2	974.44
									GEWVALNPLR	95.0%	70.8	20.4	7	0	0	2	1,154.63
									HGGLYHENMR	95.0%	37.1	19.4	0	1	0	2	1,229.55
									IDVHLVPDR	95.0%	34.5	20.8	5	3	0	2	1,063.59
									IDVHLVPDRK	94.9%	26.5	18.7	0	1	0	2	1,191.69
									IVSSAMEPDR	95.0%	60.5	22.9	9	0	0	2	1,120.53
									IVSSAMEPDREYHFGQAVR	95.0%	42.7	21.8	0	4	0	2	2,192.06
									KGEWVALNPLR	95.0%	42.6	18.5	6	0	0	2	1,282.73
									LGYVTADGETSGSITCGK	95.0%	43.7	20.8	1	0	0	2	1,815.84
									LSYTCEGGFR	95.0%	65.3	19.5	3	0	0	2	1,189.53
									NTEILTGSWSDQTYPEGTQAIYK	95.0%	70.8	21.0	5	4	0	2	2,602.23
									RPYFPVAVGK	95.0%	42.8	20.5	3	28	0	2	1,133.65
									SCDIPVFMNAR	95.0%	56.1	21.3	8	0	0	2	1,309.60
									SIDVACHPGYALPK	95.0%	65.7	22.6	4	0	0	2	1,527.76
									SLGNVIMVCR	95.0%	59.5	22.6	12	0	0	2	1,164.59
									SPDVINGSPISQK	95.0%	71.8	22.5	18	0	0	2	1,341.70
									SPPEISHGVVAHMSDSYQYGEEVTYK	95.0%	41.4	19.0	0	2	1	2	2,926.32
									SSNLIILEEHLK	95.0%	64.5	18.9	16	17	0	2	1,395.78
									SSQESYAHGTK	95.0%	41.0	19.7	1	0	0	2	1,194.54
									TDCLSLPSFENAIPMGEK	95.0%	71.3	21.5	8	0	0	2	2,008.94
									TGDEITYQCR	95.0%	49.7	18.3	9	0	0	2	1,242.54
									TGESVEFVCK	95.0%	35.8	21.4	1	0	0	2	1,155.54
									WQSIPLCVEK	95.0%	42.7	22.8	6	0	0	2	1,259.65
UPF0556 protein C19orf10	CS010_HUMAN	C19orf10	18,777	100.00%	2	3	5	13.90%	ESDVPLKTEEFEVTK	95.0%	71.1	22.7	2	1	0	2	1,750.88

									SYLYFTQFK	95.0%	46.7	22.6	2	0	0	2	1,196.60
Platelet-derived growth factor subunit A	PDGFA_HUMAN	PDGFA	24,025	99.50%	2	2	3	13.30%	SQVDPTSANFLIWPPCVEVK	95.0%	42.0	21.8	1	0	0	2	2,287.14
									TVIYEIPR	95.0%	36.3	20.2	2	0	0	2	990.56
Annexin A7	ANXA7_HUMAN	ANXA7	52,723	99.50%	2	2	11	5.12%	GFGTDEQAIVDVVANR	95.0%	89.0	22.6	2	0	0	2	1,690.84
									SEIDLVQIK	95.0%	52.5	19.8	9	0	0	2	1,044.59
LIM domain and actin-binding protein 1	LIMA1_HUMAN	LIMA1	85,208	100.00%	3	3	5	6.46%	IAWPPPTELGSSGSALEEGIK	95.0%	41.7	21.7	2	0	0	2	2,139.10
									ISANENSLAVR	95.0%	60.9	22.6	2	0	0	2	1,173.62
									NENEEILERPAQLANAR	95.0%	32.4	22.0	0	1	0	2	1,966.99
Nuclear transport factor 2	NTF2_HUMAN	NUTF2	14,461	100.00%	3	3	7	29.90%	ADEDPIMGFHQMFLLK	95.0%	49.2	22.9	4	0	0	2	1,891.91
									LSSLPFQK	95.0%	31.0	20.6	1	0	0	2	919.53
									NINDAWVCTNDMFR	95.0%	85.7	19.3	2	0	0	2	1,755.76
Tropomyosin beta chain	TPM2_HUMAN	TPM2	32,834	100.00%	13	16	65	32.00%	AEFAER	95.0%	30.5	22.0	1	0	0	2	722.35
									GTEDEVEK	95.0%	33.2	21.2	1	0	0	2	906.41
									IQLVEEELDR	95.0%	69.4	22.3	10	0	0	2	1,243.65
									IQLVEEELDRAQER	95.0%	50.9	21.9	7	6	0	2	1,727.89
									KATDAEADVASLNR	95.0%	110.0	22.6	2	0	0	2	1,460.73
									KYEEVAR	95.0%	30.6	22.4	1	0	0	2	894.47
									LDKENAIDR	95.0%	32.3	23.4	0	2	0	2	1,073.56
									LEEAEKAADESER	95.0%	77.8	21.9	11	7	0	2	1,476.68
									LKEAETR	95.0%	42.4	24.5	6	0	0	2	846.47
									LKGTEDEVEK	95.0%	30.2	23.5	0	2	0	2	1,147.58
									LVILEGELER	95.0%	41.3	20.3	2	0	0	2	1,170.67
									RIQLVEEELDR	95.0%	32.9	21.9	2	2	0	2	1,399.75
									YEEVAR	95.0%	38.1	22.0	3	0	0	2	766.37
Phosphoglucomutase-2	PGM2_HUMAN	PGM2	68,268	100.00%	5	5	8	8.01%	AAMGPGISR	95.0%	43.8	23.7	2	0	0	2	875.44
									DLTTGYDDSQPDKK	95.0%	64.8	20.5	2	0	0	2	1,582.72
									LDQETAQWLR	95.0%	45.9	22.2	2	0	0	2	1,259.64
									MEFGTAGLR	95.0%	50.9	21.8	1	0	0	2	981.48
									QLIDQGK	95.0%	31.8	21.7	1	0	0	2	801.45
EF-hand domain-containing protein D2	EFHD2_HUMAN	EFHD2	26,680	100.00%	7	7	24	24.60%	ADLNQGIGEPQSPSR	95.0%	68.1	22.3	4	0	0	2	1,568.77
									ADLNQGIGEPQSPSRR	95.0%	35.8	23.1	0	1	0	2	1,724.87
									DGFIDLMELK	95.0%	44.8	23.4	6	0	0	2	1,180.59
									LGAPQTHLGLK	95.0%	30.7	18.1	0	2	0	2	1,134.66
									LSEIDVSSEGVK	95.0%	82.5	23.2	7	0	0	2	1,262.65
									RADLNQGIGEPQSPSR	95.0%	44.5	23.1	3	0	0	2	1,724.87
									VQAINVSSR	95.0%	50.9	22.5	1	0	0	2	973.54
14-3-3 protein theta	1433T_HUMAN	YWHAQ	27,747	100.00%	11	11	63	59.20%	AVTEQGAELSNEER	95.0%	104.0	21.8	8	0	0	2	1,532.72
									DSTLIMQLLR	95.0%	79.3	21.3	113	0	0	2	1,189.66
									EMQPTHPIR	95.0%	67.1	22.4	13	0	0	2	1,108.56
									KEMQPTHPIR	95.0%	43.9	22.5	0	11	0	2	1,252.65
									LAEQAER	95.0%	59.4	21.6	36	0	0	2	816.42
									NLLSVAYK	95.0%	51.4	19.1	49	0	0	2	907.53
									QTIDNSQGAYQEAFDISK	95.0%	119.0	21.2	4	0	0	2	2,014.94
									QTIDNSQGAYQEAFDISKK	95.0%	69.4	22.0	0	4	0	2	2,143.03
									SICTTVLELLDK	95.0%	74.9	21.6	6	0	0	2	1,391.75
									TAFDEAIAELDTLNEDSYK	95.0%	107.0	21.6	4	0	0	2	2,144.99
									TELIQK	95.0%	36.0	23.5	4	0	0	2	731.43
									VISSIEQK	95.0%	65.9	22.9	27	0	0	2	903.52
									YDDMATCMK	95.0%	32.0	10.4	1	0	0	2	1,134.43
									YLAEVACGDDR	95.0%	35.0	18.5	1	0	0	2	1,268.56
									YLAEVACGDDRK	95.0%	56.2	21.5	2	0	0	2	1,396.65

									YLIANATNPESK	95.0%	74.4	23.2	2	0	0	2	1,320.68
Protein mago nashi homolog 2	MGN2_HUMAN	MAGOHB	17,259	100.00%	5	5	8	35.80%	IGSLIDVNQSK	95.0%	62.3	23.3	2	0	0	2	1,173.65
									IGSLIDVNQSKDPEGLR	95.0%	42.7	20.5	0	1	0	2	1,840.98
									IIDDSEITKEDDALWPPPDR	95.0%	57.1	22.1	1	0	0	2	2,325.13
									NDVMIR	95.0%	36.7	24.1	2	0	0	2	763.38
									VFYYLVQDLK	95.0%	51.5	22.7	2	0	0	2	1,287.70
Ubiquitin-conjugating enzyme E2 variant 1	UB2V1_HUMAN	UBE2V1	16,477	100.00%	2	2	4	13.60%	LLEELEEGQK	95.0%	46.1	22.8	2	0	0	2	1,187.62
									WTGMIIGPPR	95.0%	43.4	20.6	2	0	0	2	1,127.60
Platelet-activating factor acetylhydrolase IB subunit alpha	LIS1_HUMAN	PAFAH1B1	46,619	100.00%	8	10	15	22.20%	EEFTSGGPLGQK	95.0%	37.0	22.5	1	0	0	2	1,249.61
									GHTDSVQDISFDHSGK	95.0%	70.9	20.5	1	2	0	2	1,729.78
									LNEAKEEFTSGGPLGQK	95.0%	51.3	22.5	1	2	0	2	1,804.91
									SGKPGPFLLSGSR	95.0%	31.4	22.0	0	1	0	2	1,302.72
									SNGYEEAYSVFK	95.0%	41.4	20.5	2	0	0	2	1,393.63
									TAPYVVTGSVDQTVK	95.0%	75.7	22.1	2	0	0	2	1,564.82
									VWDYETGDFER	95.0%	76.9	17.6	2	0	0	2	1,416.61
									YALSGHR	95.0%	33.8	22.6	1	0	0	2	803.42
Aldehyde dehydrogenase, dimeric NADP-preferring	AL3A1_HUMAN	ALDH3A1	50,362	100.00%	17	21	200	41.90%	DLYPVINGGVPETTELLK	95.0%	67.8	20.0	3	0	0	2	1,958.05
									EKPLALYMFSSNDK	95.0%	48.8	22.3	5	1	0	2	1,642.82
									ERFDHILYTGSTGVGK	95.0%	26.6	22.4	0	1	0	2	1,779.90
									FDHILYTGSTGVGK	95.0%	66.7	22.9	6	0	0	2	1,494.76
									HLTPVTLELGGK	95.0%	56.7	18.9	2	4	0	2	1,264.73
									IIMTAAAK	95.0%	40.0	22.9	2	0	0	2	834.48
									IQQLEALQR	95.0%	68.4	18.6	16	0	0	2	1,098.63
									ISEAVKR	95.0%	34.5	22.5	3	0	0	2	802.48
									LIQEQEQELVGALAADLHK	95.0%	104.0	19.5	4	52	0	2	2,105.12
									LPEWAADEPVEK	95.0%	68.1	22.3	11	0	0	2	1,383.68
									SCLVRPLMNDEGLK	95.0%	53.0	23.3	2	0	0	2	1,647.82
									SFETFSHR	95.0%	36.8	21.8	5	0	0	2	1,010.47
									SLEEAIQFINQR	95.0%	82.0	22.7	15	6	0	2	1,447.75
									SLKEFYGEDAK	95.0%	49.3	22.0	2	0	0	2	1,286.63
									VAYGGTGDAATR	95.0%	95.2	22.4	51	0	0	2	1,138.55
									VMGLIEGQK	95.0%	48.5	22.7	8	0	0	2	974.53
									VRYPPSPAK	95.0%	31.3	22.3	1	0	0	2	1,014.57
Trifunctional purine biosynthetic protein adenosine-3	PUR2_HUMAN	GART	107,750	100.00%	3	3	5	3.86%	ENLISALEEAKK	95.0%	52.6	21.6	1	0	0	2	1,344.74
									FGDPECQVILPLLK	95.0%	55.8	21.4	2	0	0	2	1,628.87
									VDLGGFAGLFDLK	95.0%	72.9	21.2	2	0	0	2	1,351.73
3-ketoacyl-CoA thiolase, peroxisomal	THIK_HUMAN	ACAA1	44,274	100.00%	4	4	6	12.00%	AEELGLPILGVLR	95.0%	76.8	14.3	2	0	0	2	1,379.83
									GNPGNITSR	95.0%	36.0	20.6	1	0	0	2	915.46
									IAQFLSDIPETVPLSTVNR	95.0%	79.4	19.7	2	0	0	2	2,100.13
									QVITLLNELK	95.0%	48.2	17.8	1	0	0	2	1,170.71
Spermidine synthase	SPEE_HUMAN	SRM	33,807	99.50%	2	2	3	7.62%	AAFVLPEFAR	95.0%	35.9	21.6	2	0	0	2	1,120.62
									VLIIGGGDGGVLR	95.0%	45.7	17.8	1	0	0	2	1,225.73
Laminin subunit alpha-3	LAMA3_HUMAN	LAMA3	366,625	100.00%	43	52	416	15.50%	AASASESALQTVIK	95.0%	84.6	22.1	8	0	0	2	1,375.74
									AASASESALQTVIKEDLPR	95.0%	94.9	21.0	12	26	0	2	1,986.05
									AGYTGTQCER	95.0%	74.0	18.1	3	0	0	2	1,142.49
									ALGAIQR	95.0%	40.0	20.9	2	0	0	2	728.44
									ALTDADNSVNK	95.0%	76.9	22.8	30	0	0	2	1,147.56
									ANDITDEVLDGLNPIQTDVER	95.0%	98.2	21.6	2	0	0	2	2,327.14
									APVYLGSPPSGKPK	95.0%	56.3	20.0	9	1	0	2	1,397.78
									AQTLNNNVNR	95.0%	52.6	21.7	3	0	0	2	1,143.59
									CAPGYFGNPQK	95.0%	40.3	20.5	2	0	0	2	1,238.56

									CAVDAATAYENILNAIK	95.0%	102.0	22.9	2	0	0	2	1,836.92
									EAELQVDQILTK	95.0%	87.7	22.6	7	0	0	2	1,386.75
									EINSLQSDFTK	95.0%	58.5	22.1	15	0	0	2	1,281.63
									ELTDLNQEFETLQEK	95.0%	119.0	22.6	29	0	0	2	1,836.89
									ESQLLLNR	95.0%	51.8	23.0	7	0	0	2	972.55
									FANSPRPDLWVLER	95.0%	30.8	21.2	0	4	0	2	1,699.89
									FISLNIEDGK	95.0%	47.6	23.1	4	0	0	2	1,135.60
									GATSSKPETPGVYDMDGR	95.0%	67.8	20.5	5	9	0	2	1,867.85
									GDIDAMISSAK	95.0%	65.7	23.5	10	0	0	2	1,107.54
									GLLFFAENGDR	95.0%	51.7	22.5	6	0	0	2	1,238.62
									IKDTYGR	95.0%	30.5	21.5	1	0	0	2	852.46
									INQLLQDTPVASPR	95.0%	102.0	20.6	8	0	0	2	1,551.85
									KANDITDEVLDGLNPIQTDVER	95.0%	100.0	20.4	2	17	0	2	2,455.23
									KAQTLNNNVNR	95.0%	63.0	21.1	2	3	0	2	1,271.68
									LAASLNEAR	95.0%	66.7	23.4	12	0	0	2	944.52
									LGGSNFEGCISNVFVQR	95.0%	92.0	22.2	2	0	0	2	1,883.91
									LKQEVSPALNNLQQTLNIVTVQK	95.0%	50.1	11.1	0	11	0	2	2,578.46
									LLIDDQLLR	95.0%	68.7	20.3	33	0	0	2	1,098.65
									LPDLWR	95.0%	33.0	20.7	1	0	0	2	799.45
									LPQELLKPR	95.0%	39.7	12.6	1	0	0	2	1,093.67
									LQEAAAQAK	95.0%	65.2	21.6	4	0	0	2	929.51
									LSLSPEVLDLTSNSLK	95.0%	110.0	20.9	15	1	0	2	1,715.94
									LSLSPEVLDLTSNSLKR	95.0%	78.1	17.2	8	2	0	2	1,872.04
									NSFMALYLSK	95.0%	35.5	22.7	1	0	0	2	1,189.59
									QANGLNQENER	95.0%	60.6	20.7	2	0	0	2	1,272.59
									QEVSPALNNLQQTLNIVTVQK	95.0%	121.0	16.6	7	2	0	2	2,337.28
									QISGTDGEGNNVPSGDFSR	95.0%	98.8	20.4	12	0	0	2	1,936.86
									SAISNHGSK	95.0%	44.2	20.5	4	0	0	2	900.45
									SPQTYMDGLLHYVSVISDNSGLR	95.0%	44.8	21.5	0	2	0	2	2,568.24
									SQFAVDMQTTSSR	95.0%	74.7	19.9	7	0	0	2	1,473.66
									SQLQGLSASAGLLEQMR	95.0%	108.0	22.6	11	2	0	2	1,804.92
									TLSSNSDKLLNEAK	95.0%	59.5	22.0	1	0	0	2	1,519.80
									VPTQPHAPIPTFGQTIQTTVDR	95.0%	79.3	19.9	0	37	0	2	2,404.26
									VTASMDSGAGGTSTSVTPK	95.0%	97.0	21.4	12	0	0	2	1,769.82
									YKLNSELPK	95.0%	29.8	20.8	0	1	0	2	1,091.61
Sulfhydryl oxidase 1	QSOX1_HUMAN	QSOX1	82,561	100.00%	21	24	234	32.80%	AAPGQEPPEHMAELQR	95.0%	70.3	22.1	6	0	0	2	1,776.83
									AHFSPSNIILDFPAAGSAAR	95.0%	97.2	21.6	11	27	0	2	2,042.05
									DCASHFEQMAAASMHR	95.0%	27.1	13.0	0	1	0	2	1,864.75
									DFNIPGFPTVR	95.0%	44.5	22.8	11	0	0	2	1,262.65
									DVQNVAAAPELAMGALELESR	95.0%	98.4	22.2	3	0	0	2	2,184.10
									EVALDLSQHK	95.0%	55.9	22.1	9	0	0	2	1,139.61
									FPVLEGQR	95.0%	42.2	22.9	11	0	0	2	945.52
									IEVGRFPVLEGQR	95.0%	46.9	19.9	2	2	0	2	1,499.83
									IYMADLESALHYILR	95.0%	36.6	22.2	1	0	0	2	1,823.94
									KEGAVLAK	95.0%	31.4	21.0	1	0	0	2	815.50
									LAGAPSEDPQFPK	95.0%	71.9	22.0	20	0	0	2	1,356.68
									LEEIDGFFAR	95.0%	51.0	22.8	8	0	0	2	1,196.60
									NGSGAVFPVAGADVQTLR	95.0%	84.9	22.4	8	0	0	2	1,758.91
									NKIPYSFFK	95.0%	28.7	22.9	0	2	0	2	1,143.62
									NNEEYLALIFEK	95.0%	92.0	22.3	10	0	0	2	1,482.75
									RVLNTEANVVR	95.0%	45.6	20.1	6	0	0	2	1,270.72

									SALYSPSDPLTLLQADTVR	95.0%	121.0	21.4	64	2	0	2	2,047.07
									SFYTAYLQR	95.0%	51.0	23.3	10	0	0	2	1,148.57
									TALDDRK	95.0%	36.0	24.3	4	0	0	2	818.44
									VLNTEANVVR	95.0%	51.0	22.9	12	0	0	2	1,114.62
									VPVLMESR	95.0%	50.2	23.1	3	0	0	2	946.50
Acidic leucine-rich nuclear phosphoprotein 32 family member E	AN32E_HUMAN	ANP32E	30,675	100.00%	3	3	4	7.46%	DLSTVEALQNLK	95.0%	47.3	22.7	1	0	0	2	1,330.72
									IKDLSTVEALQNLK	95.0%	49.1	17.9	1	0	0	2	1,571.90
									INLELR	95.0%	39.6	21.8	2	0	0	2	757.46
Nuclease-sensitive element-binding protein 1	YBOX1_HUMAN	YBX1	35,906	100.00%	9	10	63	47.20%	AADPPAENSSAPEAEQGGAE	95.0%	36.1	17.7	2	0	0	2	1,897.81
									EDGNEEDKENQGDETQGQQPPQR	95.0%	43.3	15.8	0	18	0	2	2,628.11
									GAEAANVTGPGGVPVQGSK	95.0%	115.0	22.3	25	0	0	2	1,695.87
									NDTKEDVFVHQTAIK	95.0%	35.6	22.7	0	2	0	2	1,744.89
									NEGSESAPEGQAQQR	95.0%	70.5	19.2	6	0	0	2	1,587.70
									NYQQNYQNSESGEK	95.0%	37.8	17.7	1	0	0	2	1,688.72
									NYQQNYQNSESGEKNEGSESAPEGQAQQR	95.0%	85.1	15.6	0	5	0	2	3,257.40
									RPQYSNPPVQGEVMEGADNQGAGEQGRPVR	95.0%	61.5	20.6	0	1	1	2	3,239.53
									SVGDGETVEFDVVEGEK	95.0%	45.0	21.7	2	0	0	2	1,795.82
Eukaryotic peptide chain release factor subunit 1	ERF1_HUMAN	ETF1	49,015	100.00%	3	3	8	7.78%	GFGGIGGILR	95.0%	40.9	20.1	2	0	0	2	946.55
									LSVLGAITSVQQR	95.0%	65.0	17.8	3	0	0	2	1,371.80
									YFDEISQDTGK	95.0%	62.0	18.5	3	0	0	2	1,302.59
Dihydrolipoyllysine-residue succinyltransferase component of 2-oxoglutarate dehydrogenase	ODO2_HUMAN	DLST	48,711	100.00%	3	3	9	9.93%	AKPAEAPAAAAPK	95.0%	39.8	21.6	0	5	0	2	1,192.67
									ASAFALQEQPVVNAVIDDTTK	95.0%	108.0	20.9	2	0	0	2	2,217.14
									VEGGTPLFTLR	95.0%	52.8	21.6	2	0	0	2	1,189.66
Splicing factor 3A subunit 3	SF3A3_HUMAN	SF3A3	58,833	100.00%	4	4	7	12.80%	EELNAISGPNEFAEFYNR	95.0%	73.8	20.6	1	0	0	2	2,099.97
									ENPSEEAQNLVEFTDEEGYGR	95.0%	119.0	17.8	2	0	0	2	2,413.04
									SLESLDTSLFAK	95.0%	65.8	22.1	2	0	0	2	1,310.68
									VKPLQDQNELFGK	95.0%	46.2	21.5	2	0	0	2	1,515.82
Proliferating cell nuclear antigen	PCNA_HUMAN	PCNA	28,751	100.00%	12	14	53	49.00%	AEDNADTLALVFEAPNQEK	95.0%	90.9	21.7	19	1	0	2	2,074.99
									ATPLSSTVTLSMSADVPLVVEYK	95.0%	50.8	20.4	2	0	0	2	2,424.26
									DLSHIGDAVVISCAK	95.0%	65.1	22.5	2	1	0	2	1,584.81
									FSASGELGNGNIK	95.0%	98.0	23.2	3	0	0	2	1,293.64
									LVQGSILK	95.0%	32.1	19.2	2	0	0	2	857.55
									LVQGSILKK	95.0%	43.5	11.5	2	0	0	2	985.64
									MPSGEFAR	95.0%	43.7	20.6	7	0	0	2	910.41
									NLAMGVNLTSMSK	95.0%	83.3	23.3	7	0	0	2	1,365.69
									SEGFDTYR	95.0%	47.9	15.7	2	0	0	2	974.42
									VSDYEMK	95.0%	49.0	17.1	2	0	0	2	871.39
									YLNFFTK	95.0%	37.8	23.6	1	0	0	2	932.49
									YYLAPK	95.0%	31.5	19.6	2	0	0	2	754.41
Actin-related protein 2/3 complex subunit 4	ARPC4_HUMAN	ARPC4	19,649	100.00%	3	3	9	17.90%	AENFFILR	95.0%	49.8	21.7	5	0	0	2	1,009.55
									ELLLQPVTISR	95.0%	66.4	17.1	2	0	0	2	1,268.76
									VLIEGSINSVR	95.0%	58.2	21.2	2	0	0	2	1,186.68
Actin-related protein 3	ARP3_HUMAN	ACTR3	47,354	100.00%	8	9	23	30.90%	AEPEDHYFLLTEPPLNTPENR	95.0%	28.8	21.8	0	1	0	2	2,482.19
									DITYFIQQLLR	95.0%	46.9	19.1	2	0	0	2	1,409.78
									DREVGIPPEQSLETAK	95.0%	81.2	21.9	4	1	0	2	1,768.91
									LGYAGNTEPQFIIPSCIAIK	95.0%	76.7	21.3	2	0	0	2	2,192.14
									LSEELSGGR	95.0%	59.3	23.5	5	0	0	2	947.48
									NIVLSGGSTMFR	95.0%	66.4	22.3	5	0	0	2	1,297.66
									TLTGTVIDSGDGVTHVIPVAEGYVIGSCIK	95.0%	33.3	19.2	0	1	0	2	3,058.58
									YSYVCPDLVK	95.0%	38.8	20.2	2	0	0	2	1,243.60
Adenylyl cyclase-associated protein	CAP1_HUMAN	CAP1	51,838	100.00%	8	10	45	23.40%	AGAAPYVQAFDSLLAGPVAEYLK	95.0%	77.6	20.0	2	0	0	2	2,351.23

1									EIGGDVQK	95.0%	35.0	22.0	3	0	0	2	845.44
									EMNDAAMFYTNR	95.0%	76.7	14.5	5	0	0	2	1,494.60
									LSDLLAPISEQIK	95.0%	70.9	18.0	15	0	0	2	1,426.82
									NSLDCEIVSAK	95.0%	35.6	22.5	2	0	0	2	1,235.59
									SGPKPFSAPKPQTSPSPK	95.0%	50.7	20.5	0	2	1	2	1,837.98
									VENQENVSNLVIEDTELK	95.0%	116.0	22.4	10	2	0	2	2,073.04
									VPTISINK	95.0%	33.9	20.4	3	0	0	2	871.53
Oncostatin-M-specific receptor subunit beta	OSMR_HUMAN	OSMR	110,492	99.90%	2	2	2	2.55%	LPLTPVSLK	95.0%	34.6	6.0	1	0	0	2	967.62
									QPSQSYTLFESFSGEK	95.0%	41.4	22.9	1	0	0	2	1,834.85
SUMO-activating enzyme subunit 1	SAE1_HUMAN	SAE1	38,432	100.00%	3	3	6	9.83%	AQNLNPMVDVK	95.0%	44.9	23.7	2	0	0	2	1,244.63
									LDSSETTMVK	95.0%	39.1	20.9	2	0	0	2	1,126.53
									VSQGVEDGPDTKR	95.0%	41.4	22.2	2	0	0	2	1,387.68
Insulin-like growth factor-binding protein 4	IBP4_HUMAN	IGFBP4	27,916	100.00%	5	5	25	20.90%	EDARPVPQGSCQSELHR	95.0%	55.8	21.1	0	7	0	2	1,965.92
									LPGGLEPK	95.0%	35.2	16.2	1	0	0	2	810.47
									QCHPALDGQR	95.0%	40.0	21.0	0	8	0	2	1,181.55
									TGVKLPGGLEPK	95.0%	49.0	15.7	0	4	0	2	1,195.71
									THEDLYIIPIPNCDR	95.0%	76.3	21.8	5	0	0	2	1,855.90
Prothymosin alpha	PTMA_HUMAN	PTMA	12,185	100.00%	3	3	8	21.60%	AAEDDEDDDVDTK	95.0%	67.4	10.0	2	0	0	2	1,437.55
									AAEDDEDDDVDTKK	95.0%	89.5	16.6	2	0	0	2	1,565.65
									EVVEEAENGR	95.0%	63.3	20.8	4	0	0	2	1,131.53
Importin-7	IPO7_HUMAN	IPO7	119,502	100.00%	10	10	17	10.60%	AFAVGVQQVLLK	95.0%	70.8	14.3	2	0	0	2	1,272.77
									AIFQTIQNR	95.0%	49.3	22.2	2	0	0	2	1,090.60
									ENIVEAIIHSPELIR	95.0%	42.8	20.1	2	0	0	2	1,732.96
									ETENDDLTNVIQK	95.0%	66.5	22.3	2	0	0	2	1,518.73
									EYNEFAEVFLK	95.0%	36.1	21.0	2	0	0	2	1,388.67
									GIDQCIPLFVEAALER	95.0%	38.2	22.3	1	0	0	2	1,830.94
									GTMDPALR	95.0%	43.7	23.1	1	0	0	2	876.43
									QLQDIATLADQR	95.0%	53.7	22.7	2	0	0	2	1,371.72
									QLQDIATLADQRR	95.0%	28.3	21.9	0	1	0	2	1,527.82
									SDQNLQTALELTR	95.0%	77.4	22.9	2	0	0	2	1,488.77
Apoptosis inhibitor 5	API5_HUMAN	API5	57,545	99.50%	2	2	8	6.08%	ELPQFATGENLPR	95.0%	60.8	22.4	6	0	0	2	1,471.75
									GTLGGLFSQILQGEDIVR	95.0%	53.1	19.9	2	0	0	2	1,903.03
UPF0553 protein C9orf64	CI064_HUMAN	C9orf64	39,012	100.00%	5	6	9	17.00%	DCLLELIEQK	95.0%	47.6	23.6	1	0	0	2	1,260.65
									FGGSFLNCVR	95.0%	42.4	21.3	2	0	0	2	1,156.56
									ILNETGK	95.0%	37.1	24.6	1	0	0	2	774.44
									LPQVLAHLGALK	95.0%	58.2	10.4	2	2	0	2	1,259.78
									QEVEIR	95.0%	37.8	23.5	1	0	0	2	773.42
									SDTDVSMPLVEER	95.0%	48.4	20.9	1	0	0	2	1,493.68
NADPH--cytochrome P450 reductase	NCPR_HUMAN	POR	76,673	99.50%	2	2	3	5.17%	SYENQKPPFDAK	95.0%	43.1	21.9	2	0	0	2	1,423.69
									YESGDHVAVYPANDSALVNQLGK	95.0%	27.8	21.6	0	1	0	2	2,447.18
Lactoylglutathione lyase	LGUL_HUMAN	GLO1	20,761	100.00%	4	4	26	25.00%	DFLLQQTMLR	95.0%	77.6	22.4	8	0	0	2	1,280.67
									GLAFIQDPDGYWIEILNPNK	95.0%	112.0	20.5	4	0	0	2	2,303.17
									IAWALSR	95.0%	45.1	22.3	7	0	0	2	816.47
									VLGMTLIQK	95.0%	59.2	18.5	7	0	0	2	1,002.60
PEST proteolytic signal-containing nuclear protein	PCNP_HUMAN	PCNP	18,907	100.00%	4	5	10	29.80%	AGAAGGPEEEAEKPVK	95.0%	76.2	21.9	2	0	0	2	1,539.77
									FGFAIGSQTTK	95.0%	61.0	22.2	2	0	0	2	1,156.60
									SAEEEAADLPTKPTK	95.0%	71.6	22.4	2	2	0	2	1,586.79
									SHLGNVHDQDN	95.0%	39.0	18.2	2	0	0	2	1,235.54
Rab GDP dissociation inhibitor alpha	GDIA_HUMAN	GDI1	50,566	100.00%	10	10	30	45.00%	DWNVDLIPK	95.0%	43.3	21.6	2	0	0	2	1,099.58
									FLMANGQLVK	95.0%	43.4	22.8	6	0	0	2	1,136.61
									FQLLEGPPESMGR	95.0%	78.3	23.0	4	0	0	2	1,460.72

									GRDWNVDLIPK	95.0%	36.0	21.5	1	0	0	2	1,312.70
									IICILSHPIK	94.9%	26.5	16.9	0	1	0	2	1,193.71
									IYKVPSTETEALASNLMGMFEK	95.0%	32.2	21.6	0	2	0	2	2,491.21
									KQNDVFGEAEQ	95.0%	43.0	20.9	4	0	0	2	1,264.58
									LYSESLAR	95.0%	55.9	20.3	6	0	0	2	938.49
									MAGTAFDFENMKR	95.0%	36.4	18.3	2	0	0	2	1,549.68
									MLLYTEVTR	95.0%	53.4	23.0	13	0	0	2	1,125.60
									NPYYGGESSSITPLEELYK	95.0%	108.0	22.1	2	0	0	2	2,147.02
									NTNDANSCQIIIPQNQVNR	95.0%	59.4	21.9	1	0	0	2	2,199.06
									SPYLYPLYGLGELPQGFAR	95.0%	98.6	21.6	8	0	0	2	2,141.11
									TDDYLDQPCLETVNR	95.0%	79.5	20.2	2	0	0	2	1,838.82
									TFEGVDPQTTSMR	95.0%	79.6	21.4	4	0	0	2	1,468.67
									VPSTETEALASNLMGMFEK	95.0%	104.0	21.2	8	0	0	2	2,070.97
									VVEGSFVYK	95.0%	30.8	20.9	1	0	0	2	1,027.55
Calcium-binding protein 39	CAB39_HUMAN	CAB39	39,853	100.00%	4	4	4	12.90%	FQNDRTEDEQFNDEK	94.6%	26.2	16.5	0	1	0	2	1,914.81
									LIEFLSK	95.0%	31.9	14.1	1	0	0	2	849.51
									LLGELLLDR	95.0%	57.2	19.1	1	0	0	2	1,041.63
									LLSAEFLEQHYDR	95.0%	27.4	22.6	0	1	0	2	1,620.80
Arginyl-tRNA synthetase, cytoplasmic	SYRC_HUMAN	RARS	75,364	100.00%	5	5	13	10.60%	AAYPDLENPPLLVTPSQQAK	95.0%	79.9	20.5	4	0	0	2	2,152.13
									GFDILGIKPVQR	95.0%	35.2	18.6	0	1	0	2	1,342.79
									SDGGYTYDTSDLAAIK	95.0%	81.8	21.2	2	0	0	2	1,676.77
									STIIGESISR	95.0%	31.7	22.2	2	0	0	2	1,062.58
									VIVDFSSPNIAK	95.0%	67.7	21.1	4	0	0	2	1,289.71
Fibulin-1	FBLN1_HUMAN	FBLN1	77,194	100.00%	13	13	113	21.50%	AITPPHPASQANIIFDITEGNLR	95.0%	59.1	19.7	0	28	0	2	2,475.30
									CLAFECPENYR	95.0%	53.0	16.6	3	0	0	2	1,458.61
									DSFDIIKR	95.0%	40.1	21.6	5	0	0	2	993.54
									EFTRPEEIIFLR	95.0%	44.5	20.4	5	0	0	2	1,549.84
									GYHLNEEGTR	95.0%	39.8	19.5	12	0	0	2	1,175.54
									IIEVEEEQEDPYLNDR	95.0%	98.8	21.1	6	0	0	2	1,990.92
									LEMNYVVGGVVSHR	95.0%	28.3	23.0	0	2	0	2	1,575.80
									MCVDVNECQR	95.0%	53.3	15.2	10	0	0	2	1,326.52
									SAATLQQEK	95.0%	64.8	23.1	3	0	0	2	975.51
									SQETGDLDVGGLQETDK	95.0%	137.0	22.4	8	0	0	2	1,791.83
									SQETGDLDVGGLQETDKIIEVEEEQEDPYLNDR	95.0%	71.0	19.4	0	8	0	2	3,763.73
									TGYYFDGISR	95.0%	76.1	21.7	18	0	0	2	1,178.55
									YMDGMTVGVVR	95.0%	73.4	21.0	5	0	0	2	1,227.59
Leucine-rich PPR motif-containing protein, mitochondrial	LPPRC_HUMAN	LRPPRC	157,894	100.00%	6	6	9	5.09%	LQDAINILK	95.0%	32.8	17.8	1	0	0	2	1,027.62
									NVQGIIEILK	95.0%	31.1	17.2	1	0	0	2	1,126.68
									SNTLPISLQSIR	95.0%	40.3	20.7	1	0	0	2	1,328.75
									SYVSEKDVTSAK	95.0%	44.3	22.1	1	0	0	2	1,313.66
									TVLDQQQTPSR	95.0%	55.6	22.7	4	0	0	2	1,272.66
									TVQLTSSELESTLETLK	95.0%	46.0	20.2	1	0	0	2	1,878.99
Inorganic pyrophosphatase	IPYR_HUMAN	PPA1	32,643	100.00%	4	4	7	19.40%	AAPFSLEYR	95.0%	35.7	22.0	2	0	0	2	1,053.54
									DKDFAIDIIK	95.0%	51.6	21.7	2	0	0	2	1,177.65
									VIAINVDDPDAANYNDINDVK	95.0%	110.0	22.2	2	0	0	2	2,288.10
									VLGILAMIDEGETDWK	95.0%	50.7	22.1	1	0	0	2	1,805.90
Translationally-controlled tumor protein	TCTP_HUMAN	TPT1	19,578	100.00%	5	7	51	33.10%	DGLEMEKC	95.0%	34.8	12.3	1	0	0	2	997.40
									DLISHDEMFSDIYK	95.0%	88.5	21.5	17	7	0	2	1,712.78
									EDGVTPYMIFFK	95.0%	65.4	22.4	10	0	0	2	1,446.70
									GKLEEQRPER	95.0%	39.9	21.1	2	0	0	2	1,241.66
									VKPFMTGAAEQIK	95.0%	56.1	21.2	9	5	0	2	1,419.77

Ras-related protein Rab-1B	RAB1B_HUMAN	RAB1B	22,154	99.50%	2	3	5	34.80%	EFADSLGIPFLETSAK	95.0%	77.3	23.1	7	0	0	2	1,724.87
									LLLIGDSGVGK	95.0%	58.7	20.3	2	0	0	2	1,071.64
									LQIWDTAGQER	95.0%	61.5	22.2	7	0	0	2	1,316.66
									MGPGAASGGERPNLK	95.0%	51.6	21.6	2	1	0	2	1,457.72
									NATNVEQAFMTMAAEIK	95.0%	84.4	19.7	2	0	0	2	1,900.88
Actin, cytoplasmic 1	ACTB_HUMAN	ACTB	41,720	100.00%	19	28	1414	58.70%	AGFAGDDAPR	95.0%	81.5	21.2	149	0	0	2	976.45
									AVFPSIVGRPR	95.0%	65.7	18.6	47	0	0	2	1,198.71
									CDVDIRK	95.0%	34.7	23.7	1	0	0	2	905.45
									DLTDYLMK	95.0%	51.3	20.6	26	0	0	2	1,014.48
									DLYANTVLSGGTTMYPGIADR	95.0%	125.0	21.7	149	6	0	2	2,231.07
									DSYVGDEAQSK	95.0%	81.6	19.0	88	0	0	2	1,198.52
									DSYVGDEAQSKR	95.0%	63.0	21.6	43	7	0	2	1,354.62
									EITALAPSTMK	95.0%	68.7	23.5	60	0	0	2	1,177.61
									GYSFTTTAER	95.0%	76.4	20.0	154	0	0	2	1,132.53
									HQGVMVGMGQK	95.0%	48.0	21.7	19	13	0	2	1,171.57
									IIAPPER	95.0%	42.4	17.9	6	0	0	2	795.47
									IIAPPERK	95.0%	39.0	16.0	5	0	0	2	923.57
									IWHHTFYNELR	95.0%	55.8	22.4	0	53	0	2	1,515.75
									KDLYANTVLSGGTTMYPGIADR	95.0%	96.1	21.4	2	10	0	2	2,359.16
									LCYVALDFEQEMATAASSSSLEK	95.0%	99.6	20.3	26	22	0	2	2,566.17
									QEYDESGPSIVHR	95.0%	58.9	21.4	36	53	0	2	1,516.70
									SYELPDGQVITIGNER	95.0%	125.0	22.9	175	2	0	2	1,790.89
									TTGIVMDSGDGVTHTVPIYEGYALPHAILR	95.0%	74.2	20.0	0	81	77	2	3,183.62
									VAPEEHPVLLTEAPLNPK	95.0%	85.4	18.4	60	44	0	2	1,954.07
ATP synthase subunit alpha, mitochondrial	ATPA_HUMAN	ATP5A1	59,734	100.00%	4	4	6	9.22%	AVDSLVPIGR	95.0%	31.1	19.3	2	0	0	2	1,026.59
									TGAIVDVPVGEELLGR	95.0%	30.5	20.2	1	0	0	2	1,624.89
									TGTAEMSSILEER	95.0%	67.5	20.6	1	0	0	2	1,439.67
									VVDALGNAIDGK	95.0%	56.7	23.1	2	0	0	2	1,171.63
Serine/threonine-protein phosphatase 2A 65 kDa regulatory subunit A alpha isoform	2AAA_HUMAN	PPP2R1A	65,292	100.00%	10	13	31	25.00%	AISHEHSPSDLEAHFVPLVK	95.0%	22.0	21.0	0	0	3	2	2,213.14
									IGPILDNSTLQSEVKPILEK	95.0%	84.4	14.6	2	1	0	2	2,194.23
									LAGGDWFTSR	95.0%	55.2	21.7	2	0	0	2	1,109.54
									LTQDQDVDVK	95.0%	37.1	23.6	2	0	0	2	1,160.58
									MAGDPVANVR	95.0%	66.6	22.0	2	0	0	2	1,045.51
									NLCSDDTPMVR	95.0%	39.0	18.1	1	0	0	2	1,323.57
									QLSQSLLPAIVELAEDAK	95.0%	84.9	18.6	7	1	0	2	1,925.06
									SALASVIMGLSPILGK	95.0%	37.8	15.7	2	0	0	2	1,572.90
									SEIIPMFSNLASDEQDSVR	95.0%	105.0	22.4	4	0	0	2	2,138.01
									VLAMSGDPNYLHR	95.0%	73.9	21.8	2	2	0	2	1,488.73
Galectin-3	LEG3_HUMAN	LGALS3	26,135	100.00%	6	10	65	28.40%	GNDVAFHFNPR	95.0%	66.6	21.8	10	0	0	2	1,273.61
									IALDFQR	95.0%	43.1	24.4	4	0	0	2	862.48
									IQVLVEPDHFK	95.0%	54.1	20.6	7	11	0	2	1,324.73
									MLITILGTVKPNANR	95.0%	86.9	14.8	9	12	0	2	1,640.95
									QSVFPFESGKPFK	95.0%	41.8	22.1	1	5	0	2	1,497.77
									VAVNDAHLLQYNHR	95.0%	75.1	22.6	2	4	0	2	1,649.85
Serine protease HTRA1	HTRA1_HUMAN	HTRA1	51,269	100.00%	12	13	78	27.70%	ADIALIK	95.0%	35.5	19.8	3	0	0	2	743.47
									ESTLNMVVR	95.0%	33.6	23.5	2	0	0	2	1,064.54
									EVPVASGSGFIVSEDGLIVTNAHVVTNK	95.0%	29.9	19.2	0	2	0	2	2,839.48
									GACGQGQEDPNSLR	95.0%	38.2	18.6	1	0	0	2	1,488.65
									IAPAVVHIELFR	95.0%	51.1	15.2	2	0	0	2	1,364.81
									IDHQGKLPVLLLGR	95.0%	50.7	10.8	0	2	0	2	1,558.94
									IKDVDEK	95.0%	35.9	24.7	1	0	0	2	846.46

									LHRPPVIVLQR	95.0%	41.1	9.0	2	6	0	2	1,327.83
									LPVLLLGR	95.0%	57.6	6.0	45	0	0	2	880.60
									MMSLTSSK	95.0%	50.2	16.8	3	0	0	2	916.41
									VTAGISFAIPSDK	95.0%	42.6	22.0	1	0	0	2	1,305.71
									YNFIADVVEK	95.0%	55.8	22.0	8	0	0	2	1,197.62
Dermcidin	DCD_HUMAN	DCD	11,266	99.50%	2	2	69	20.00%	DAVEDLESVGK	95.0%	50.7	23.0	1	0	0	2	1,161.56
									ENAGEDPGLAR	95.0%	90.3	20.9	68	0	0	2	1,128.53
Vacuolar protein sorting-associated protein 35	VPS35_HUMAN	VPS35	91,692	100.00%	11	11	30	17.60%	AELAELPLR	95.0%	46.3	17.7	4	0	0	2	1,011.58
									ENDAVTIQVLNQLIQK	95.0%	79.1	19.1	2	0	0	2	1,826.00
									GVQHPLR	95.0%	30.5	20.5	1	0	0	2	806.46
									ILVGTNLVR	95.0%	63.1	16.7	1	0	0	2	984.62
									IPVDTYNNILTVLK	95.0%	79.8	17.6	10	0	0	2	1,602.91
									IREDLPNLESSEETEQINK	95.0%	45.7	21.8	0	2	0	2	2,244.10
									LLDEAIQAVK	95.0%	54.4	21.6	2	0	0	2	1,099.64
									LSQLEGVNVER	95.0%	70.8	22.3	3	0	0	2	1,243.66
									PTTQQSPQDEQEKLLDEAIQAVK	95.0%	48.8	21.4	0	2	0	1	2,596.31
									SEDPDQQYLILNTAR	95.0%	73.2	22.5	2	0	0	2	1,762.86
									VADLYELVQYAGNIIPR	95.0%	34.0	20.8	0	1	0	2	1,934.04
Eukaryotic translation initiation factor 5	IF5_HUMAN	EIF5	49,205	99.50%	2	2	4	5.80%	AMGPLVLTEVLFNEK	95.0%	75.7	21.3	3	0	0	2	1,676.89
									VLTLSDDLER	95.0%	35.8	23.5	1	0	0	2	1,160.62
Protein S100-A10	S10AA_HUMAN	S100A10	11,186	100.00%	3	5	9	45.40%	EFPGFLENQKDPLAVDK	95.0%	64.8	22.5	4	1	0	2	1,946.99
									FAGDKGYLTK	95.0%	33.6	22.6	2	1	0	2	1,099.58
									PSQMEHAMETMMFTFHK	95.0%	20.0	17.5	0	0	1	1	2,082.89
Ras-related protein Rab-7a	RAB7A_HUMAN	RAB7A	23,472	100.00%	7	8	27	39.60%	ATIGADFLTK	95.0%	59.5	20.2	2	0	0	2	1,036.57
									DPENFPFVVLGNK	95.0%	64.4	22.5	4	0	0	2	1,475.75
									EAINVEQAFQTIAR	95.0%	89.0	22.4	12	1	0	2	1,589.83
									FQSLGVAFYR	95.0%	67.9	22.0	2	0	0	2	1,187.62
									LVTMQIWDTAGQER	95.0%	83.8	23.3	4	0	0	2	1,647.82
									NNIPYFETSAK	95.0%	32.1	21.7	1	0	0	2	1,283.63
									TSLMNQYVNK	95.0%	31.5	21.7	1	0	0	2	1,213.59
Polyadenylate-binding protein 4	PABP4_HUMAN	PABPC4	70,766	99.50%	2	2	5	10.20%	ALDTMNFDVIK	95.0%	73.5	21.7	12	0	0	2	1,282.64
									EFSPFGSITSAK	95.0%	41.4	21.6	1	0	0	2	1,270.63
									FSPAGPVLSIR	95.0%	48.9	21.1	4	0	0	2	1,143.65
									GFGFVCFSSPEEATK	95.0%	61.6	19.8	1	0	0	2	1,662.75
									SLGYAYVNFQQPADAER	95.0%	97.2	21.3	5	0	0	2	1,928.91
Legumain	LGMN_HUMAN	LGMN	49,393	100.00%	7	7	41	21.00%	ASSPVPLPPVTHLDLTPSPDVPLTIMK	95.0%	50.0	17.4	0	6	0	2	2,838.53
									DYTGEDVTPQNFLAVLR	95.0%	78.1	22.1	17	0	0	2	1,937.96
									IVSLLAASEAEVEQLLSER	95.0%	64.7	18.4	1	0	0	2	2,057.11
									KLMNTNDLEESR	95.0%	75.1	21.1	5	0	0	2	1,465.70
									LMNTNDLEESR	95.0%	65.0	20.1	6	0	0	2	1,337.60
									QLTEEIQR	95.0%	47.0	22.3	5	0	0	2	1,016.54
									VMQFQGMK	95.0%	31.1	18.7	1	0	0	2	1,000.46
Coronin-1B	COR1B_HUMAN	CORO1B	54,217	99.50%	2	2	5	4.29%	DADPILISLR	95.0%	57.1	19.9	3	0	0	2	1,112.63
									VGIIAWHPTAR	95.0%	34.2	20.5	0	2	0	2	1,220.69
ATP-dependent RNA helicase DDX1	DDX1_HUMAN	DDX1	82,415	100.00%	2	2	3	2.57%	ELAEQTLNNIK	95.0%	46.1	22.7	2	0	0	2	1,272.68
									TGASVLNK	95.0%	41.0	22.9	1	0	0	2	789.45
Myosin-9	MYH9_HUMAN	MYH9	226,520	100.00%	38	44	163	24.50%	AGVLAHLEEER	95.0%	48.5	23.8	1	1	0	2	1,223.64
									ALEEAMEQK	95.0%	48.8	22.2	3	0	0	2	1,064.49
									ALELDSNLYR	95.0%	69.8	23.2	5	0	0	2	1,193.62
									ANLQIDQINTDLNLER	95.0%	133.0	22.0	4	0	0	2	1,869.97
									DFSALESQLQDTQELLQEENR	95.0%	125.0	20.8	5	2	0	2	2,493.17

									ELEDATETADAMNR	95.0%	88.5	17.0	8	0	0	2	1,581.67
									ELESQISELQEDLESER	95.0%	107.0	21.4	6	0	0	2	2,033.95
									EMEAELEDERK	95.0%	45.1	20.0	3	0	0	2	1,394.61
									FVSELWK	95.0%	33.0	22.0	1	0	0	2	908.49
									HSQAVEELAEQLEQTKR	95.0%	50.3	21.9	0	2	0	2	1,996.01
									IAEFTTNLTEEEEK	95.0%	101.0	22.0	6	0	0	2	1,653.79
									IAEFTTNLTEEEEKSK	95.0%	87.6	22.3	1	0	0	2	1,868.91
									IAQLEEELEEEQGNTELINDR	95.0%	98.9	20.6	2	2	0	2	2,472.17
									IAQLEEQLDNETK	95.0%	83.1	22.3	8	0	0	2	1,530.77
									IAQLEEQLDNETKER	95.0%	27.0	22.3	0	1	0	2	1,815.91
									IIGLDQVAGMSETALPGAFK	95.0%	92.1	21.3	12	0	0	2	2,018.06
									IMGIPEEEQMGLLR	95.0%	64.0	23.3	3	0	0	2	1,647.81
									IRELESQISELQEDLESER	95.0%	47.9	21.6	0	2	0	2	2,303.14
									KEEELQAALAR	95.0%	66.7	22.8	5	0	0	2	1,257.68
									KFDQLLAEEK	95.0%	35.6	22.6	1	0	0	2	1,220.65
									KQELEEICHDLEAR	95.0%	31.3	21.8	0	1	0	2	1,769.85
									LDPHLVLDQLR	95.0%	50.3	18.9	4	5	0	2	1,318.75
									LQVELDNVTGLLSQSDSK	95.0%	65.3	21.4	3	0	0	2	1,946.01
									MQQNIQELEEQLEEEESAR	95.0%	137.0	20.3	1	3	0	2	2,333.06
									NFINNPLAQADWAAK	95.0%	82.4	22.6	6	0	0	2	1,672.84
									NLPIYSEEIVEMYK	95.0%	33.7	20.6	2	0	0	2	1,743.85
									NRDEAIK	95.0%	37.2	23.0	1	0	0	2	845.45
									NTDQASMPDNTAAQK	95.0%	94.1	18.1	5	0	0	2	1,591.70
									QAQQERDELADEIANSSGK	95.0%	55.2	20.7	0	3	0	2	2,088.98
									QLEEAEEEAQR	95.0%	70.5	20.4	3	0	0	2	1,331.61
									QLLQANPILEAFGNAK	95.0%	81.5	19.4	28	0	0	2	1,726.95
									QQQLTAMK	95.0%	36.3	23.6	1	0	0	2	963.49
									RGDLPFVVPR	94.8%	26.4	19.6	0	1	0	2	1,155.66
									TEMEDLMSSKDDVGK	95.0%	57.4	17.0	2	1	0	2	1,716.73
									THEAQIQEMR	95.0%	38.7	21.5	2	0	0	2	1,258.59
									VEAQLQELQVK	95.0%	37.6	21.1	2	0	0	2	1,284.72
									VISGVLQLGNIVFK	95.0%	60.0	12.0	3	0	0	2	1,486.90
									VKLQEMEGTVK	95.0%	31.1	22.8	0	2	0	2	1,277.68
Glypican-1	GPC1_HUMAN	GPC1	61,663	100.00%	18	22	271	37.30%	DVQDFWISLPGTLCSEK	95.0%	50.6	22.1	4	0	0	2	1,994.95
									ERPPSGTLEK	95.0%	41.6	21.2	9	0	0	2	1,113.59
									GCLANQADLDAEWR	95.0%	62.1	20.3	2	0	0	2	1,618.73
									GFSLSDVPQAEISGEHLR	95.0%	56.1	21.8	6	9	0	2	1,941.97
									KVAQVPLGPECSR	95.0%	45.6	21.6	2	7	0	2	1,440.76
									MALSTASDDR	95.0%	61.9	20.5	9	0	0	2	1,082.48
									QAEALRPFGEAPR	95.0%	32.8	21.9	0	4	0	2	1,441.76
									QQIMQLK	95.0%	32.8	23.4	3	0	0	2	904.49
									SFVQGLGVASDVVR	95.0%	97.2	21.3	61	0	0	2	1,433.78
									SFVQGLGVASDVVRK	95.0%	74.7	18.8	2	0	0	2	1,561.87
									SHAELETALR	95.0%	40.2	22.7	5	0	0	2	1,126.59
									TLQATFPGAFGELYTQNAR	95.0%	105.0	23.2	54	12	0	2	2,085.04
									VAQVPLGPECSR	95.0%	48.5	22.3	8	0	0	2	1,312.67
									VIQGCGNPK	95.0%	38.4	21.3	4	0	0	2	972.49
									VLQAMLATQLR	95.0%	51.7	20.4	13	0	0	2	1,259.71
									VNPQGPGPEEK	95.0%	44.8	23.0	35	0	0	2	1,151.57
									VNPQGPGPEEKR	95.0%	52.9	22.7	13	7	0	2	1,307.67
									YLPEVMGDGLANQINNPEVEVDITKPDMTIR	95.0%	80.9	19.8	0	2	0	2	3,503.70

Profilin-1	PROF1_HUMAN	PFN1	15,036	100.00%	8	13	157	55.70%	CYEMASHLR	95.0%	44.5	18.5	3	2	0	2	1,182.50
									DSLLQDGEFSMDLR	95.0%	105.0	21.4	78	2	0	2	1,625.75
									DSPSVWAAVPGK	95.0%	61.2	21.4	4	0	0	2	1,213.62
									STGGAPTFNVTVTK	95.0%	78.5	23.1	8	0	0	2	1,379.72
									TDKTLVLLMGK	95.0%	65.1	17.6	10	7	0	2	1,218.71
									TFVNITPAEVGVLVGK	95.0%	102.0	17.7	22	2	0	2	1,643.94
									TFVNITPAEVGVLVGKDR	95.0%	86.8	17.2	2	3	0	2	1,915.07
									TLVLLMGK	95.0%	56.4	17.6	14	0	0	2	874.54
Chromobox protein homolog 5	CBX5_HUMAN	CBX5	22,208	100.00%	2	2	4	14.70%	SNFSNSADDIK	95.0%	42.9	20.9	2	0	0	2	1,197.54
									TADSSSSEDEEEYVVEK	95.0%	130.0	14.3	2	0	0	2	1,903.79
Macrophage migration inhibitory factor	MIF_HUMAN	MIF	12,459	99.50%	2	2	8	17.40%	LLCGLLAER	95.0%	66.2	22.2	4	0	0	2	1,044.59
									PMFIVNTNVPR	95.0%	72.6	23.9	4	0	0	1	1,303.68
Pyridoxal kinase	PDXK_HUMAN	PDXK	35,084	100.00%	5	5	9	24.00%	AQAGEGVRPSPMQLELR	95.0%	26.8	21.4	0	1	0	2	1,838.95
									GQVLNSDELQELYEGLR	95.0%	103.0	22.5	3	0	0	2	1,962.98
									NPAGSVVMER	95.0%	46.3	21.7	2	0	0	2	1,059.53
									VVPLADIITPNQFEAELLSGR	95.0%	56.6	17.7	1	0	0	2	2,282.24
									YDYVLTGYTR	95.0%	61.9	22.2	2	0	0	2	1,250.61
Coproporphyrinogen-III oxidase, mitochondrial	HEM6_HUMAN	CPOX	50,134	100.00%	2	2	6	6.61%	ATSLGRPEEEEDELAHR	95.0%	32.6	21.8	0	2	0	2	1,938.92
									IESILMSLPLTAR	95.0%	78.5	19.6	4	0	0	2	1,459.82
5'(3')-deoxyribonucleotidase, cytosolic type	NT5C_HUMAN	NT5C	23,365	100.00%	3	3	6	19.90%	LLSWSDNWR	95.0%	37.5	22.5	2	0	0	2	1,176.58
									VLVDMDGVLADFEAGLLR	95.0%	111.0	21.7	2	0	0	2	1,949.01
									WVEQHLGPQFVER	95.0%	32.8	21.6	0	2	0	2	1,624.82
Beta-galactosidase	BGAL_HUMAN	GLB1	76,060	100.00%	2	2	9	4.28%	AYVAVDGIPQGVLER	95.0%	38.4	22.1	2	0	0	2	1,586.85
									TEAVASSLYDILAR	95.0%	80.9	21.6	7	0	0	2	1,508.80
Metastasis-associated protein MTA2	MTA2_HUMAN	MTA2	75,007	99.50%	2	2	2	7.04%	LNPADAPNPVVFVATK	95.0%	63.7	20.0	1	0	0	2	1,652.90
									VKPTLIAVRPPVPLPAPSHPASTNEPIVLED	95.0%	30.7	13.6	0	1	0	2	3,254.82
Nucleosome assembly protein 1-like 1	NP1L1_HUMAN	NAP1L1	45,357	100.00%	2	2	10	6.91%	FYEEVHDLER	95.0%	48.4	21.0	4	0	0	2	1,336.62
									LDGLVETPTGYIESLPR	95.0%	80.5	21.1	6	0	0	2	1,859.98
26S proteasome non-ATPase regulatory subunit 11	PSD11_HUMAN	PSMD11	47,448	100.00%	4	4	6	12.30%	EASIDILHSIVK	95.0%	34.7	18.8	1	0	0	2	1,324.75
									IMLNTPEDVQALVSGK	95.0%	65.0	22.2	1	0	0	2	1,730.90
									LYDNLLEQNLIR	95.0%	83.4	20.8	2	0	0	2	1,503.82
									TGQAAELGGLLK	95.0%	68.5	22.4	2	0	0	2	1,157.65
Apolipoprotein A-I	APOA1_HUMAN	APOA1	30,761	100.00%	3	3	7	15.40%	DYVSQFEGSALGK	95.0%	41.7	21.1	1	0	0	2	1,400.67
									EQLGPVTQEFWDNLEK	95.0%	78.3	22.7	4	0	0	2	1,932.93
									VSFLSALEEYTK	95.0%	85.9	21.5	2	0	0	2	1,386.72
Thioredoxin-like protein 1	TXNL1_HUMAN	TXNL1	32,233	100.00%	4	5	7	17.00%	IFINLPR	95.0%	44.0	20.3	2	0	0	2	872.54
									IKQHLENDPGSNEDTDIPK	95.0%	48.1	22.0	0	1	0	2	2,150.04
									QHLENDPGSNEDTDIPK	95.0%	48.8	19.6	1	1	0	2	1,908.86
									SEPTQALELTEDDIKEDGIVPLR	95.0%	38.7	21.0	0	2	0	2	2,568.31
Radixin	RADI_HUMAN	RDX	68,548	100.00%	9	10	24	36.20%	AFAAQEDLEK	95.0%	40.5	23.6	3	0	0	2	1,121.55
									ALELDQER	95.0%	34.1	21.5	1	0	0	2	973.50
									APDFVFYAPR	95.0%	80.6	21.7	84	0	0	2	1,182.59
									EKEELMER	95.0%	33.2	21.6	2	0	0	2	1,079.50
									ENPLQFK	95.0%	35.5	24.5	1	0	0	2	875.46
									FVIKPIDK	95.0%	41.1	17.2	6	0	0	2	959.59
									IAQDLEMYGVNYFEIK	95.0%	55.0	23.2	2	0	0	2	1,948.94
									IGFPWSEIR	95.0%	68.8	23.4	121	0	0	2	1,104.58
									ILALCMGNHELYMR	95.0%	40.7	21.7	2	0	0	2	1,752.82
									KALELDQER	95.0%	31.0	23.2	1	0	0	2	1,101.59
									KAPDFVFYAPR	95.0%	47.6	22.3	25	2	0	2	1,310.69
									KENPLQFK	95.0%	31.1	21.9	1	0	0	2	1,003.56

									KPDTIEVQQMK	95.0%	64.0	23.1	5	0	0	2	1,316.69
									KTQNDVLHAENVK	95.0%	27.7	22.8	0	1	0	2	1,495.79
									LFFLQVK	95.0%	42.2	18.9	11	0	0	2	894.55
									LKQIEEQTIK	95.0%	28.2	20.0	0	2	0	2	1,229.71
									NISFNDKK	95.0%	31.2	22.7	2	0	0	2	965.51
									NQEQLAAELAEFTAK	95.0%	109.0	23.0	8	2	0	2	1,662.83
									QAADQMKNQEQLAAELAEFTAK	94.8%	26.4	21.8	0	1	0	2	2,451.18
									QLFDQVVK	95.0%	58.8	21.4	19	0	0	2	976.55
									QLQALSSELAQAR	95.0%	101.0	22.0	3	0	0	2	1,414.77
									RKPDTIEVQQMK	95.0%	51.6	22.3	12	33	0	2	1,488.78
									VLEQHK	95.0%	34.7	19.1	2	0	0	2	753.43
									VTTMDAELEFAIQPNTTGK	95.0%	106.0	22.4	27	0	0	2	2,082.01
S-formylglutathione hydrolase	ESTD_HUMAN	ESD	31,446	100.00%	8	8	28	38.30%	AFSGYLGTDQSK	95.0%	70.0	21.8	8	0	0	2	1,273.61
									AYDATHLVK	95.0%	48.9	23.3	4	0	0	2	1,017.54
									FAVYLPPK	95.0%	51.0	21.4	6	0	0	2	934.54
									KAFSGYLGTDQSK	95.0%	64.6	22.7	2	0	0	2	1,401.70
									MYSYVTEELPQLINANFPVDPQR	95.0%	46.7	21.8	0	2	0	2	2,740.33
									SGYHQSASEHGLVVIAPDTSPR	95.0%	31.5	21.8	0	2	0	2	2,308.13
									SVSAFAPICNPVLCPWGK	95.0%	61.5	22.7	2	0	0	2	2,002.99
									SYPGSQLDILIDQGK	95.0%	76.5	22.3	2	0	0	2	1,633.84
Alpha-2-macroglobulin	A2MG_HUMAN	A2M	163,273	100.00%	4	4	12	2.51%	ATVLNYLPK	95.0%	46.2	18.4	2	0	0	2	1,018.59
									GPTQEFK	95.0%	31.5	23.7	2	0	0	2	806.41
									LPPNVVEESAR	95.0%	40.0	22.4	1	0	0	2	1,210.64
									QTVSWAVTPK	95.0%	43.8	23.0	7	0	0	2	1,116.61
Coronin-1A	COR1A_HUMAN	CORO1A	51,008	100.00%	3	3	7	7.38%	AAPEASGTPSSDAVSR	95.0%	82.2	20.3	4	0	0	2	1,502.71
									DAGPLLISLK	95.0%	48.2	16.0	2	0	0	2	1,026.62
									KGTVVAEK	94.7%	30.1	21.9	1	0	0	2	831.49
Eukaryotic translation initiation factor 2 subunit 3	IF2G_HUMAN	EIF2S3	51,092	100.00%	2	3	5	5.93%	IVLTNPVCTEVGEK	95.0%	32.8	22.8	1	0	0	2	1,558.82
									VGQEIEVRPGIVSK	95.0%	45.8	18.3	2	2	0	2	1,510.86
Peptidyl-prolyl cis-trans isomerase NIMA-interacting 4	PIN4_HUMAN	PIN4	13,792	99.50%	2	2	5	32.10%	FNEVAAQYSEDK	95.0%	85.7	18.6	2	0	0	2	1,400.63
									GSMVGPFQEAAFALPVSGMDKPVFTDPPVK	95.0%	44.4	20.7	0	3	0	2	3,151.55
Renin receptor	RENR_HUMAN	ATP6AP2	38,991	100.00%	2	2	4	7.43%	LFQENSVLSSLPLNSLSR	95.0%	92.5	19.4	3	0	0	2	2,004.08
									SFDTSLIR	95.0%	32.9	20.3	1	0	0	2	938.49
D-dopachrome decarboxylase	DOPD_HUMAN	DDT	12,694	100.00%	4	4	9	36.40%	FFPLESWQIGK	95.0%	61.0	21.8	3	0	0	2	1,351.71
									PFLELDTNLPANR	95.0%	81.2	22.1	2	0	0	1	1,499.79
									PFLELDTNLPANRVPAGLEK	95.0%	38.8	18.1	0	2	0	1	2,194.19
									SHSAHFFEFLTK	95.0%	39.8	22.8	0	2	0	2	1,450.71
Syntaxin-binding protein 2	STXB2_HUMAN	STXBP2	66,423	99.50%	2	2	2	3.88%	ILSGVIR	95.0%	33.9	21.8	1	0	0	2	757.49
									LIVPVLLDAAVPAYDK	94.6%	30.0	14.6	1	0	0	2	1,696.99
V-set and transmembrane domain-containing protein 2-like protein	VTM2L_HUMAN	VSTM2L	22,331	99.50%	2	2	2	18.10%	VQPGENSVLHLPEAPPAAPAPPPPKPGK	95.0%	42.4	16.5	0	1	0	2	2,792.51
									VVGSNISHK	95.0%	35.6	20.0	1	0	0	2	940.52
Platelet-activating factor acetylhydrolase IB subunit beta	PA1B2_HUMAN	PAFAH1B2	25,552	99.90%	2	3	28	12.20%	ELFSPLHALNFGIGGDTTR	95.0%	74.1	22.1	2	2	0	2	2,045.05
									IIVLGLLPR	95.0%	55.9	4.8	24	0	0	2	993.68
T-complex protein 1 subunit theta	TCPQ_HUMAN	CCT8	59,603	100.00%	15	15	33	31.60%	AIADTGANVVVTGGK	95.0%	83.2	22.1	2	0	0	2	1,372.74
									ANEVISK	95.0%	52.5	25.0	2	0	0	2	760.42
									APGFAQMLK	95.0%	39.6	23.4	2	0	0	2	962.51
									AVDDGVNTFK	94.8%	30.2	22.3	1	0	0	2	1,065.52
									DIDEVSSLLR	95.0%	54.0	23.8	2	0	0	2	1,146.60
									DMLEAGILDTYLGK	95.0%	70.1	22.6	4	0	0	2	1,538.78
									EDGAISTIVLR	95.0%	40.1	23.0	2	0	0	2	1,173.65
									FAEAFEAIPR	95.0%	78.6	21.2	4	0	0	2	1,150.59

									GSTDNLMDDIER	95.0%	59.3	18.9	2	0	0	2	1,365.60
									LFVTNDAATILR	95.0%	82.9	20.1	3	0	0	2	1,333.75
									LVPGGGATEIELAK	95.0%	35.1	19.5	2	0	0	2	1,354.76
									QYGNEVFLAK	95.0%	40.3	22.5	2	0	0	2	1,168.60
									TAEELMNFSKGEENLMDAQVK	95.0%	29.7	20.9	0	1	0	2	2,416.10
									VADMALHYANK	95.0%	39.3	23.3	2	0	0	2	1,232.61
									YNIMLVR	95.0%	48.1	21.1	2	0	0	2	908.50
Erythrocyte band 7 integral membrane protein	STOM_HUMAN	STOM	31,714	100.00%	10	11	58	46.50%	AMAAEAEASR	95.0%	51.8	20.0	3	0	0	2	1,022.46
									EASMVITESPAALQLR	95.0%	93.6	21.8	9	0	0	2	1,731.90
									GPGLFFILPCTDSFIK	95.0%	53.3	21.1	2	0	0	2	1,811.94
									LLAQTTLR	95.0%	42.7	19.8	2	0	0	2	915.56
									LPVQLQR	95.0%	42.1	15.8	2	0	0	2	853.53
									NSTIVFPLPIDMLQGIIGAK	95.0%	51.7	17.1	1	0	0	2	2,143.18
									TISFDIPPQEILTK	95.0%	53.9	19.9	4	0	0	2	1,601.88
									VIAAEGEMNASR	95.0%	76.7	22.6	21	0	0	2	1,263.60
									VQNATLAVANITNADSATR	95.0%	153.0	21.3	7	1	0	2	1,930.00
									YLQTLTTIAAEK	95.0%	74.8	21.5	6	0	0	2	1,351.75
EH domain-containing protein 1	EHD1_HUMAN	EHD1	60,611	100.00%	3	3	4	7.30%	ELVNNLGEIYQK	95.0%	72.6	22.4	2	0	0	2	1,419.75
									LLDTVDDMLANDIAR	95.0%	60.5	22.6	1	0	0	2	1,690.83
									QEESLMPSQVVK	95.0%	41.4	22.9	1	0	0	2	1,390.69
Citrate synthase, mitochondrial	CISY_HUMAN	CS	51,696	100.00%	9	9	23	20.00%	ALGFPLERPK	95.0%	28.0	20.0	0	1	0	2	1,127.66
									AYAQGISR	95.0%	43.2	23.0	3	0	0	2	865.45
									DILADLIPK	95.0%	46.6	14.3	5	0	0	2	997.59
									DVSDEKLR	95.0%	47.5	24.0	1	0	0	2	961.50
									DYIWNTLNSGR	95.0%	66.3	22.5	5	0	0	2	1,338.64
									GLVYETSVLDPDEGIR	95.0%	114.0	22.7	4	0	0	2	1,762.89
									HLPNDPMFK	94.7%	30.1	21.8	1	0	0	2	1,098.54
									IVPNVLLEQGK	95.0%	38.8	15.3	1	0	0	2	1,209.72
									VVPGYGHAVLR	95.0%	35.6	20.5	2	0	0	2	1,167.66
Tryptophanyl-tRNA synthetase, cytoplasmic	SYWC_HUMAN	WARS	53,150	100.00%	8	11	45	27.40%	AGNASKDEIDSAVK	95.0%	53.9	22.0	2	1	0	2	1,404.70
									ALIEVLQPLIAEHQAR	95.0%	59.7	16.4	5	6	0	2	1,801.03
									DLTLDQAYSYAVENAK	95.0%	107.0	21.3	11	0	0	2	1,800.87
									DMNQVLDAYENK	95.0%	93.8	19.4	4	0	0	2	1,455.64
									GIDYDKLIVR	95.0%	56.8	20.3	2	1	0	2	1,191.67
									ISFPAIQAAPSFSNSFPQIFR	95.0%	57.6	21.5	2	0	0	2	2,325.20
									MSASDPNSSIFLTDTAK	95.0%	109.0	21.6	9	0	0	2	1,800.83
									PNSEPASLLELFNSIATQGELVR	95.0%	135.0	20.7	2	0	0	1	2,485.29
Leukocyte elastase inhibitor	ILEU_HUMAN	SERPINB1	42,726	100.00%	14	16	62	38.30%	ADLSGMSGAR	95.0%	68.3	21.0	6	0	0	2	964.45
									EATTNAPFR	94.5%	30.0	23.3	1	0	0	2	1,006.50
									FAYGYIEDLK	95.0%	51.7	23.3	2	0	0	2	1,218.60
									FQSLNADINKR	95.0%	63.6	22.3	4	1	0	2	1,305.69
									HNSSGSILFLGR	95.0%	49.4	23.3	1	0	0	2	1,287.68
									IEEQLTLEK	95.0%	43.3	22.4	2	0	0	2	1,102.60
									IPELLASGMVDNMTK	95.0%	92.8	22.7	9	0	0	2	1,650.81
									KIEEQLTLEK	94.8%	30.2	21.7	1	0	0	2	1,230.69
									LGVQDLFNSSK	95.0%	74.2	23.3	11	0	0	2	1,207.63
									LVLVNAIYFK	95.0%	32.0	14.0	4	0	0	2	1,179.71
									TFHFNTVEEVHSR	95.0%	20.2	22.3	0	0	1	2	1,602.77
									TYGADLASVDFQHASEDAR	95.0%	102.0	19.9	2	4	0	2	2,052.93
									TYGADLASVDFQHASEDARK	95.0%	55.1	20.7	0	2	0	2	2,181.02
									TYNFLPEFLVSTQK	95.0%	64.6	22.7	11	0	0	2	1,686.87

Cellular retinoic acid-binding protein 2	RABP2_HUMAN	CRABP2	15,675	100.00%	3	3	5	28.30%	IAVAAASKPAVEIK	95.0%	27.4	14.0	0	1	0	2	1,367.83
									SENFEELLK	95.0%	40.4	22.5	2	0	0	2	1,108.55
									VGEEFEEQTVDGRPCK	95.0%	50.2	21.1	0	2	0	2	1,879.85
Calmodulin	CALM_HUMAN	CALM1	16,820	100.00%	5	7	12	50.30%	DTDSEEEIREAFR	95.0%	40.3	18.8	1	0	0	2	1,596.71
									EADIDGDGQVNYEEFVQMMTAK	95.0%	32.2	17.6	1	0	0	2	2,490.08
									EAFSLFDKDGDGTITTK	95.0%	68.8	22.4	2	2	0	2	1,844.89
									ELGTVMR	95.0%	35.7	23.8	2	0	0	2	821.42
									VFDKDGNGYISAAELR	95.0%	109.0	22.2	2	2	0	2	1,754.87
Lysosomal protective protein	PPGB_HUMAN	CTSA	54,450	100.00%	3	3	16	6.25%	DTVVVQDLGNIFTR	95.0%	88.6	22.6	8	0	0	2	1,576.83
									YEKDTVVVQDLGNIFTR	95.0%	28.0	21.3	0	1	0	2	1,997.03
									YGDSGEQIAGFVK	95.0%	83.1	21.3	7	0	0	2	1,370.66
Hornerin	HORN_HUMAN	HRNR	282,355	100.00%	9	11	106	4.49%	GPYESGSGHSSGLGHQESR	95.0%	56.7	19.3	0	9	0	2	1,928.85
									GPYESGSGHSSGLGHR	95.0%	90.5	20.4	2	19	5	2	1,584.72
									GSGSGQSPSSGQHGTGFGR	95.0%	28.7	18.8	0	1	0	2	1,747.78
									GSGSGQSPSYGR	95.0%	62.5	20.8	11	0	0	2	1,139.51
									HGSSSGSSSSYGQHGSGSR	95.0%	29.1	18.6	0	1	0	1	1,808.75
									QSLGHGQHGSGSGQSPSPSR	95.0%	45.5	21.6	0	47	0	2	1,947.90
									QSPSYGR	95.0%	37.1	21.3	8	0	0	1	794.38
									SGSGWSSSR	95.0%	42.0	19.3	2	0	0	2	910.40
									YGQQGSGSGQSPSR	95.0%	43.3	20.5	1	0	0	2	1,395.63
Intercellular adhesion molecule 1	ICAM1_HUMAN	ICAM1	57,807	100.00%	3	3	19	7.52%	DGTFPLPIGESVTVTR	95.0%	56.5	22.6	7	0	0	2	1,688.89
									LLGIETPLPK	95.0%	46.2	12.6	5	0	0	2	1,080.67
									VELAPLPSWQPVGK	95.0%	60.5	19.6	7	0	0	2	1,520.85
Phosphoserine aminotransferase	SERC_HUMAN	PSAT1	40,405	100.00%	9	11	33	24.10%	ALELNMLSLK	95.0%	74.6	21.5	7	0	0	2	1,147.64
									DDLLGFALR	95.0%	42.9	23.9	6	0	0	2	1,019.55
									ELLAVPDNYK	95.0%	44.0	23.4	1	0	0	2	1,161.62
									FGTINIVHPK	95.0%	37.2	19.4	1	2	0	2	1,125.64
									FGVIFAGAQK	95.0%	54.3	20.2	1	0	0	2	1,037.58
									GDDALEKR	95.0%	35.1	22.3	1	0	0	2	903.45
									IINNTENLVR	95.0%	72.1	23.8	4	0	0	2	1,185.66
									LPHSVLLEIQK	95.0%	36.0	16.4	2	4	0	2	1,276.76
									QVVNFGPGPAK	95.0%	40.0	21.0	4	0	0	2	1,113.61
Scavenger mRNA-decapping enzyme DcpS	DCPS_HUMAN	DCPS	38,592	99.90%	2	2	3	11.30%	IVFENPDPSDGFVLIPDLK	95.0%	60.5	20.2	2	0	0	2	2,115.10
									VNEASGDGDGEDAVVILEK	95.0%	132.0	21.4	1	0	0	2	1,916.91
Acylamino-acid-releasing enzyme	ACPH_HUMAN	APEH	81,206	100.00%	14	16	92	23.10%	AESFFQTK	95.0%	53.0	20.6	2	0	0	2	957.47
									ALDVSASDDEIAR	95.0%	94.4	24.3	12	0	0	2	1,361.66
									CELLSDDSLAVSSPR	95.0%	79.7	22.2	1	0	0	2	1,648.79
									GSTGFGQDSILSLPGNVGHQDVK	95.0%	68.9	21.7	2	6	0	2	2,313.15
									MGFAVLLVNYR	95.0%	32.9	21.8	1	0	0	2	1,298.69
									QPALSAACLGPEVTTQYGGQYR	95.0%	81.7	22.3	1	2	0	2	2,367.14
									QVLLSEPEEAAALYR	95.0%	96.5	22.8	12	0	0	2	1,688.89
									SALYYVDLIGGK	95.0%	79.1	21.6	15	0	0	2	1,298.70
									SFNLSALEK	95.0%	30.4	21.4	1	0	0	2	1,008.54
									TPLLLMLGQEDR	95.0%	89.4	22.5	25	0	0	2	1,401.74
									TPLLLMLGQEDRR	95.0%	41.9	19.9	0	2	0	2	1,541.85
									TVHTEWTQR	95.0%	32.4	21.6	1	0	0	2	1,157.57
									VTSVVVDVVPR	95.0%	90.4	18.1	5	0	0	2	1,169.69
									VVFDSAQR	95.0%	40.2	23.1	4	0	0	2	921.48
Hydroxyacyl-coenzyme A dehydrogenase, mitochondrial	HCDH_HUMAN	HADH	34,260	100.00%	4	5	14	12.10%	DTPGFIVNR	95.0%	32.3	24.0	2	0	0	2	1,018.53
									GDASKEDIDTAMK	95.0%	55.0	21.2	5	2	0	2	1,380.63
									LVEVIK	95.0%	31.8	14.8	1	0	0	2	700.46

									TFESLVDFSK	95.0%	61.5	22.4	4	0	0	2	1,172.58
Kinectin	KTN1_HUMAN	KTN1	156,258	100.00%	30	33	68	25.20%	AHVQEVAQHNLK	95.0%	40.1	22.2	0	1	0	2	1,373.73
									ALKEEIGNVQLEK	95.0%	58.8	21.1	2	2	0	2	1,470.82
									AQQSLELIQSK	95.0%	68.5	22.6	5	0	0	2	1,244.69
									DAVSNTTNQLESK	95.0%	90.9	22.0	4	0	0	2	1,406.68
									EVIDLLKPDQVEGIQK	95.0%	58.9	17.9	2	1	0	2	1,824.01
									GELTTLIHQLQEK	95.0%	34.5	20.3	1	0	0	2	1,509.83
									ILNDQNK	95.0%	30.7	22.5	1	0	0	2	844.45
									LLEEQLQHEISNK	95.0%	75.1	21.6	2	0	0	2	1,580.83
									LMQLMESEQK	95.0%	33.2	21.2	2	0	0	2	1,268.59
									LQALANEQAAAAHELEK	95.0%	69.9	21.8	1	1	0	2	1,806.94
									LQQEEVQKK	95.0%	49.2	23.6	1	0	0	2	1,129.62
									LQTLVSEQPNKDVVEQMEK	95.0%	42.2	22.0	0	3	0	2	2,231.12
									NAEQAATQLK	95.0%	50.8	22.9	2	0	0	2	1,073.56
									QMQSSFTSSEQELER	95.0%	112.0	19.3	3	0	0	2	1,802.79
									QQNYQQASSFPPHEELLK	95.0%	35.7	22.7	0	1	0	2	2,144.04
									QQQVEAVELEAK	95.0%	59.5	22.4	1	0	0	2	1,371.71
									QSAELNK	95.0%	41.7	24.9	1	0	0	2	789.41
									QTEDSLASER	95.0%	47.4	21.2	2	0	0	2	1,135.52
									QTEDSLASERDR	95.0%	28.9	20.9	0	1	0	2	1,406.65
									STYVTEVR	95.0%	33.4	19.5	1	0	0	2	954.49
									SVEELLEAELLK	95.0%	76.0	21.6	2	0	0	2	1,372.76
									SVEQEENKWK	95.0%	41.6	22.5	2	0	0	2	1,276.62
									SVLAETEGILQK	95.0%	89.2	22.4	4	0	0	2	1,287.72
									TAEHEAAQQDLQSK	95.0%	69.7	22.1	5	0	0	2	1,555.74
									TENSSLTK	95.0%	33.4	22.3	1	0	0	2	879.44
									TQLLQDVQDENK	95.0%	78.3	22.3	2	0	0	2	1,430.71
									VQELQNLLK	95.0%	38.4	19.9	3	0	0	2	1,084.64
									VQLQEAER	95.0%	50.3	21.4	4	0	0	2	972.51
									WEEVQSYIR	95.0%	50.4	22.8	2	0	0	2	1,209.59
									WLQDLQEENESLK	95.0%	63.6	23.0	2	0	0	2	1,631.79
Attractin	ATRN_HUMAN	ATRN	158,518	100.00%	12	13	76	8.96%	CTWLIEGQPNR	95.0%	67.2	22.0	4	0	0	2	1,373.66
									DLDMFINASK	95.0%	40.8	21.8	2	0	0	2	1,169.55
									EEYSNLKLPR	95.0%	35.9	22.9	1	0	0	2	1,248.66
									IMQSSQSMSK	95.0%	53.3	19.5	6	0	0	2	1,142.52
									LTGSSGFVTDGPGNYK	95.0%	95.5	22.1	19	0	0	2	1,599.77
									LTLTPWVGLR	95.0%	61.7	19.1	12	0	0	2	1,155.69
									NHNALLASLTTQK	95.0%	81.3	20.4	5	7	0	2	1,410.77
									SEAACLAAGPGIR	95.0%	64.7	21.8	8	0	0	2	1,272.64
									SVNNVVVR	95.0%	38.1	22.9	3	0	0	2	886.51
									TLDHDR	95.0%	30.5	19.2	1	0	0	2	756.36
									YDVDTQMWTILK	95.0%	77.1	22.0	7	0	0	2	1,528.74
									YGHSLALYK	95.0%	27.3	21.4	0	1	0	2	1,051.56
Fibroblast growth factor-binding protein 1	FGFP1_HUMAN	FGFBP1	26,246	100.00%	5	7	160	21.40%	DTLGNTQIK	95.0%	63.2	24.0	20	0	0	2	989.53
									KDFPESSLK	95.0%	33.0	22.5	3	0	0	2	1,050.55
									LVSSTLFGNTKPR	95.0%	91.8	20.1	38	11	0	2	1,419.80
									VVSEQKDTLGNTQIK	95.0%	96.9	20.9	8	5	0	2	1,659.89
									WAATEQEEGISLK	95.0%	95.1	23.5	75	0	0	2	1,461.72
CAD protein	PYR1_HUMAN	CAD	242,965	100.00%	4	4	5	2.20%	EATAGNPGGQTVR	95.0%	36.7	21.8	1	0	0	2	1,257.62
									LALGIPLPELR	95.0%	31.8	7.8	1	0	0	2	1,191.75
									MALLATVLGR	95.0%	32.2	20.3	1	0	0	2	1,060.62

									VLGTSPEAIDSAENR	95.0%	98.5	22.7	2	0	0	2	1,558.77
Serum albumin	ALBU_HUMAN	ALB	69,349	100.00%	23	27	1376	38.40%	AACLLPK	95.0%	36.6	24.7	11	0	0	2	772.44
									AAFTECCQAADK	95.0%	53.6	14.6	4	0	0	2	1,371.57
									AVMDDFAAFVEK	95.0%	68.9	20.8	22	0	0	2	1,342.64
									CCTESLVNR	95.0%	53.0	18.1	36	0	0	2	1,138.50
									DDNPNLPR	95.0%	32.7	22.0	3	0	0	2	940.45
									DVFLGMFLYEYAR	95.0%	39.2	22.3	2	0	0	2	1,639.78
									FKDLGEENFK	95.0%	52.4	22.8	8	4	0	2	1,226.61
									FQNALLVR	95.0%	53.2	19.8	38	0	0	2	960.56
									KVPQVSTPTLVEVSR	95.0%	104.0	16.1	264	444	0	2	1,639.94
									LKECCEKPLLEK	95.0%	56.1	22.5	11	4	0	2	1,546.80
									LVAASQAALGL	95.0%	69.3	17.6	2	0	0	2	1,013.60
									LVNEVTEFAK	95.0%	64.2	22.8	44	0	0	2	1,149.62
									LVTDLTK	95.0%	55.6	21.1	70	0	0	2	789.47
									QNCELFEQLGEYK	95.0%	75.9	21.1	2	0	0	2	1,657.75
									QTALVELVK	95.0%	54.3	21.2	9	0	0	2	1,000.60
									RHPDYSVVLLLR	95.0%	39.1	17.6	0	8	0	2	1,467.84
									SLHTLFGDK	95.0%	32.1	23.9	1	0	0	2	1,017.54
									TCVADESAENCDK	95.0%	96.6	8.5	3	0	0	2	1,498.58
									TYETTLEK	95.0%	34.8	21.1	1	0	0	2	984.49
									VFDEFKPLVEEPQNLIK	95.0%	58.5	20.0	17	34	0	2	2,045.10
									VPQVSTPTLVEVSR	95.0%	84.9	20.3	88	0	0	2	1,511.84
									YICENQDSISSK	95.0%	51.9	19.4	52	0	0	2	1,443.64
									YLYEIAR	95.0%	46.6	21.0	194	0	0	2	927.49
Transaldolase	TALDO_HUMAN	TALDO1	37,524	100.00%	20	24	197	48.10%	AAQASDLEK	95.0%	68.2	23.6	4	0	0	2	932.47
									ALAGCDFLTISPK	95.0%	83.2	22.4	13	0	0	2	1,392.72
									EAGISKDR	95.0%	47.0	24.5	5	0	0	2	875.46
									FAADAVK	95.0%	49.4	23.0	3	0	0	2	721.39
									IHLDEK	95.0%	33.2	18.8	1	0	0	2	754.41
									ILDWHVANTDK	95.0%	65.4	22.7	2	0	0	2	1,311.67
									ILDWHVANTDKK	95.0%	64.2	20.2	5	2	0	2	1,439.76
									KFAADAVK	95.0%	31.2	20.6	1	0	0	2	849.48
									KLGGSQEDQIK	95.0%	32.9	23.9	1	1	0	2	1,202.64
									LGGSQEDQIK	95.0%	56.5	23.9	8	0	0	2	1,074.54
									LIELYK	95.0%	46.9	13.8	9	0	0	2	778.47
									LLGELLQDNAK	95.0%	77.8	21.3	45	0	0	2	1,213.68
									LSDGIRK	95.0%	46.9	22.2	4	0	0	2	788.46
									LSFDKDAMVAR	95.0%	62.5	22.7	12	2	0	2	1,252.64
									LSSTWEGIQAGK	95.0%	88.6	23.3	24	0	0	2	1,276.65
									MESALDQLK	95.0%	43.9	23.1	8	0	0	2	1,050.51
									SYEPLEDPGVK	95.0%	36.0	22.6	6	0	0	2	1,233.60
									TIVMGASFR	95.0%	37.7	22.3	14	0	0	2	981.52
									VSTEVDAR	95.0%	59.2	23.6	10	0	0	2	876.44
									WLHNEDQMAVEK	95.0%	61.3	21.5	10	7	0	2	1,499.70
Actin-like protein 6A	ACL6A_HUMAN	ACTL6A	47,443	100.00%	4	4	10	11.40%	IPEGLFDPSNVK	95.0%	31.1	22.9	1	0	0	2	1,315.69
									LIANNTTVER	95.0%	55.6	23.8	4	0	0	2	1,130.62
									QGGPTYYIDTNALR	95.0%	74.9	22.6	4	0	0	2	1,568.77
									VDFPTAIGMVVER	95.0%	41.6	22.8	1	0	0	2	1,433.75
Protein S100-A2	S10A2_HUMAN	S100A2	11,099	100.00%	4	5	8	27.60%	ELPSFVGEK	95.0%	36.6	21.9	1	0	0	2	1,005.53
									ELPSFVGEKVDEEGLK	95.0%	86.8	22.6	2	1	0	2	1,775.91
									VDEEGLKK	95.0%	40.1	23.6	2	0	0	2	917.49

									YSCQEGDKFK	95.0%	35.1	18.4	2	0	0	2	1,261.55
Peptidyl-prolyl cis-trans isomerase C	PPIC_HUMAN	PPIC	22,746	99.50%	2	2	14	9.91%	IVIGLFGK	95.0%	40.9	9.5	2	0	0	2	846.55
									TVENFVALATGEK	95.0%	106.0	22.2	12	0	0	2	1,378.72
Receptor-type tyrosine-protein phosphatase U	PTPRU_HUMAN	PTPRU	162,407	100.00%	4	4	19	3.32%	FLATFPLAAVSR	95.0%	56.1	18.9	9	0	0	2	1,292.74
									GAPISVYQVIVEEER	95.0%	97.5	22.6	6	0	0	2	1,688.89
									LVLTNPEGR	95.0%	37.6	18.8	1	0	0	2	998.56
									QSGALVPAAGVR	95.0%	38.1	19.3	3	0	0	2	1,125.64
Transportin-3	TNPO3_HUMAN	TNPO3	104,186	100.00%	2	3	6	3.90%	SLDSFLLSPEAAVGLLK	95.0%	47.6	17.5	2	0	0	2	1,759.98
									SVDPENNPTLVEVLEGVVR	95.0%	74.4	20.2	2	2	0	2	2,066.08
Elongation factor 1-beta	EF1B_HUMAN	EEF1B2	24,746	100.00%	3	3	19	18.20%	KPALVAK	95.0%	39.0	12.0	1	0	0	2	726.49
									LEECVR	95.0%	37.8	22.9	1	0	0	2	805.39
									SIQADGLVWGSSK	95.0%	70.8	22.5	6	0	0	2	1,347.69
									SPAGLQVLNDYLADK	95.0%	120.0	22.6	12	0	0	2	1,603.83
Serine protease 23	PRS23_HUMAN	PRSS23	42,984	100.00%	4	5	13	17.20%	DFLLNYPFSTSVK	95.0%	54.7	22.5	4	0	0	2	1,530.78
									GNANDIGMDYDYALLELK	95.0%	80.6	20.6	4	0	0	2	2,030.94
									LEVSSSCGPQCHK	95.0%	52.0	19.0	2	0	0	2	1,488.66
									LPVVLPQSTLNLAKPDFGAEAK	95.0%	73.4	14.0	2	1	0	2	2,308.29
Soluble calcium-activated nucleotidase 1	CANT1_HUMAN	CANT1	44,822	100.00%	7	7	24	23.20%	AEWLAVKDER	95.0%	31.4	23.4	1	0	0	2	1,216.63
									AQEENTWFSYLK	95.0%	57.8	21.1	3	0	0	2	1,515.71
									AVPWVILSDGDGTVEK	95.0%	98.1	22.2	9	0	0	2	1,685.88
									EWTTTTGDVVNENPEWVK	95.0%	102.0	21.3	4	0	0	2	2,104.98
									GMELSDLIVFNGK	95.0%	37.6	22.9	1	0	0	2	1,438.73
									TGVVYQIEGSK	95.0%	54.8	22.5	4	0	0	2	1,180.62
									VASYIMAFTLDGR	95.0%	62.0	23.4	2	0	0	2	1,459.73
Carcinoembryonic antigen-related cell adhesion molecule 1	CEAM1_HUMAN	CEACAM1	57,542	100.00%	3	3	32	8.94%	QIVGYAIGTQQATPGPANSGR	95.0%	113.0	22.1	6	0	0	2	2,086.07
									TIIVTELSPVVAKPQIK	95.0%	37.0	9.5	1	0	0	2	1,836.12
									TLTLLSVTR	95.0%	64.0	13.2	25	0	0	2	1,003.62
Brain-specific serine protease 4	BSSP4_HUMAN	PRSS22	33,713	100.00%	3	3	22	13.60%	IVQGVQLR	95.0%	40.8	15.2	9	0	0	2	912.56
									VGVAWVEPHPVYSWK	95.0%	57.7	21.9	0	7	0	2	1,753.91
									VVGGEDSTDSEWPWIVSIQK	95.0%	84.2	21.5	6	0	0	2	2,232.08
Protein TFG	TFG_HUMAN	TFG	43,430	99.50%	2	2	4	9.75%	LLDSLEPPGEPGPSTNIPENDTVDGREEK	95.0%	38.7	19.9	0	2	0	2	3,105.49
									LLSNDEVTIK	95.0%	65.8	23.0	2	0	0	2	1,131.63
Protein-L-isoaspartate(D-aspartate) O-methyltransferase	PIMT_HUMAN	PCMT1	24,633	100.00%	5	5	9	33.50%	ALDVGSGSGILTACFAR	95.0%	57.6	22.4	2	0	0	2	1,694.85
									LILPVGPAGGNQMLEQYDK	95.0%	59.4	21.4	2	0	0	2	2,059.05
									MKPLMGVIYVPLTDKEK	95.0%	29.3	20.0	0	2	0	2	1,994.07
									SGGASHSELIHNLR	95.0%	48.3	23.5	0	2	0	2	1,477.75
									VQLVVGDGR	95.0%	33.2	20.0	1	0	0	2	942.54
Dickkopf-related protein 1	DKK1_HUMAN	DKK1	28,653	100.00%	4	5	16	12.00%	GQEGSVCLR	95.0%	33.3	22.2	2	0	0	2	1,005.48
									GSHGLEIFQR	95.0%	41.5	22.7	2	3	0	2	1,143.59
									KGSHGLEIFQR	95.0%	41.1	21.7	0	6	0	2	1,271.69
									SSDCASGLCCAR	95.0%	40.7	9.0	3	0	0	2	1,343.51
Apoptosis-associated speck-like protein containing a CARD	ASC_HUMAN	PYCARD	21,610	100.00%	6	6	11	27.20%	DLLLQALR	95.0%	39.9	16.5	2	0	0	2	941.58
									ESQSYLVEDLER	95.0%	65.9	22.5	2	0	0	2	1,467.70
									ESQSYLVEDLERS	95.0%	57.8	21.0	2	0	0	2	1,554.73
									LLSVPLR	94.6%	30.0	12.3	1	0	0	2	797.53
									VLTDEQYQAVR	95.0%	84.0	22.6	2	0	0	2	1,321.68
									VTNVEWLLDALYGK	95.0%	61.8	21.2	2	0	0	2	1,620.86
Tropomyosin alpha-4 chain	TPM4_HUMAN	TPM4	28,504	100.00%	3	3	6	39.90%	AEFAER	95.0%	30.5	22.0	1	0	0	2	722.35
									AEVSELK	95.0%	33.9	25.1	1	0	0	2	775.42
									EKAEGDVAALNR	95.0%	63.8	22.7	2	0	0	2	1,272.65
									HIAEEADR	95.0%	38.5	22.0	4	0	0	2	940.45

									IQALQQQADEAEDR	95.0%	75.6	21.9	3	0	0	2	1,614.77
									IQLVEEELDR	95.0%	69.4	22.3	10	0	0	2	1,243.65
									IQLVEEELDRAQER	95.0%	50.9	21.9	7	6	0	2	1,727.89
									KYEEVAR	95.0%	30.6	22.4	1	0	0	2	894.47
									LEEAEKAADESER	95.0%	77.8	21.9	11	7	0	2	1,476.68
									LKEAETR	95.0%	42.4	24.5	6	0	0	2	846.47
									LVILEGELER	95.0%	41.3	20.3	2	0	0	2	1,170.67
									RIQLVEEELDR	95.0%	32.9	21.9	2	2	0	2	1,399.75
									YEEVAR	95.0%	38.1	22.0	3	0	0	2	766.37
Phosphoglycerate kinase 1	PGK1_HUMAN	PGK1	44,597	100.00%	32	45	935	72.90%	ACANPAAGSVILLENLR	95.0%	106.0	20.4	5	1	0	2	1,768.94
									AGGFLMK	95.0%	51.7	22.2	22	0	0	2	739.38
									AHSSMVGVNLPQK	95.0%	64.1	22.6	24	10	0	2	1,383.71
									ALESPERPFLAILGGAK	95.0%	82.0	17.3	38	52	0	2	1,769.00
									ALMDEVVK	95.0%	51.0	23.9	7	0	0	2	904.48
									DCVGPEVEK	95.0%	49.2	18.8	10	0	0	2	1,032.47
									DVLFLK	95.0%	42.5	15.7	2	0	0	2	734.45
									ELNYFAK	95.0%	38.3	21.5	6	0	0	2	884.45
									FHVEEEGKGK	95.0%	36.8	22.2	3	18	0	2	1,159.57
									IQLINNMLDK	95.0%	52.5	23.4	6	0	0	2	1,217.66
									IQLINNMLDKVNEMIIGGGMAFTFLK	95.0%	50.3	20.0	0	3	0	2	2,958.51
									ITLPVDFVTADK	95.0%	54.8	20.3	1	0	0	2	1,318.73
									ITLPVDFVTADKFDENAK	95.0%	100.0	21.4	67	40	0	2	2,023.04
									KYAEAVTR	95.0%	51.4	20.8	2	0	0	2	937.51
									LGDVYVNDAFGTAHR	95.0%	105.0	22.5	25	76	0	2	1,634.79
									LTLDKLDVK	95.0%	45.5	17.3	6	0	0	2	1,044.63
									NNQITNNQR	95.0%	52.8	21.5	15	0	0	2	1,101.54
									SLLGKDVLFLK	95.0%	44.0	9.0	2	0	0	2	1,232.76
									SVVLMSHLGRPDGVPMPDK	95.0%	42.9	22.4	0	8	0	2	2,035.05
									SVVLMSHLGRPDGVPMPDKYSLEPVAVELK	95.0%	26.2	18.5	0	0	5	2	3,295.71
									TGQATVASGIPAGWMGLDCGPESSK	95.0%	80.3	20.3	1	2	0	2	2,493.14
									TGQATVASGIPAGWMGLDCGPESSKK	95.0%	50.2	21.4	0	5	0	2	2,621.23
									VADKIQLINNMLDK	95.0%	70.8	21.0	4	4	0	2	1,630.88
									VDFNVPMK	95.0%	55.8	22.8	13	0	0	2	965.48
									VDFNVPMKNNQITNNQR	95.0%	40.3	22.7	2	2	0	2	2,048.00
									VLNNMEIGTSLFDEEGAK	95.0%	101.0	22.0	153	4	0	2	1,966.94
									VLPGVDALSNI	95.0%	66.6	18.7	39	0	0	2	1,097.62
									VNEMIIGGGMAFTFLK	95.0%	76.2	22.5	40	1	0	2	1,759.88
									VSHVSTGGGASLELLEGK	95.0%	106.0	21.6	47	77	0	2	1,740.91
									WNTEDKVSHVSTGGGASLELLEGK	95.0%	61.5	21.1	0	33	7	2	2,514.25
									YAEAVTR	95.0%	53.1	22.5	25	0	0	2	809.42
									YSLEPVAVELK	95.0%	65.8	21.6	22	0	0	2	1,247.69
Junctional adhesion molecule A	JAM1_HUMAN	F11R	32,565	100.00%	6	6	27	22.70%	DGIVMPTNPK	95.0%	30.8	22.0	1	0	0	2	1,087.55
									FDQGDTTR	95.0%	43.3	18.6	3	0	0	2	939.42
									IPENNPVK	95.0%	38.2	19.6	5	0	0	2	910.50
									ITASYEDR	95.0%	51.5	20.6	5	0	0	2	954.45
									LIVLVPPSKPTVNIPSSATIGNR	95.0%	45.7	4.8	0	9	0	2	2,373.39
									VTFLPTGITFK	95.0%	40.0	16.8	4	0	0	2	1,223.70
Procollagen-lysine,2-oxoglutarate 5-dioxygenase 1	PLOD1_HUMAN	PLOD1	83,535	100.00%	18	18	85	33.40%	AQVEEFLAQHGSEYQSVK	95.0%	94.9	22.3	6	0	0	2	2,049.99
									FLGSGGFIGYAPNLSK	95.0%	107.0	21.9	5	0	0	2	1,627.85
									FLLEYIAPMTEK	95.0%	69.9	22.4	7	0	0	2	1,470.76
									IFLDPEKR	95.0%	38.1	21.9	1	0	0	2	1,017.57

									IFQNLDGALDEVVLK	95.0%	109.0	20.5	13	0	0	2	1,673.91
									IQALGLGEDWNVEK	95.0%	88.5	21.3	4	0	0	2	1,571.81
									IQGGYENVPTIDIHMNQIGFER	95.0%	54.3	21.5	0	4	0	2	2,531.24
									LDPDMAFCANIR	95.0%	77.1	20.8	2	0	0	2	1,438.65
									LQLNYLGNYIPR	95.0%	67.4	20.7	7	0	0	2	1,463.80
									LVAEWEGQDSDSDQLFYTK	95.0%	74.0	21.1	11	0	0	2	2,231.01
									LVGPEVR	95.0%	33.1	17.2	2	0	0	2	769.46
									NLAYDTLPVLIHGNGPTK	95.0%	89.7	19.8	3	0	0	2	1,923.03
									NVIAPLMTR	94.7%	30.2	23.4	1	0	0	2	1,030.57
									QQDVFMFLTNR	95.0%	62.6	22.7	8	0	0	2	1,398.68
									SAQFFNYK	95.0%	36.1	22.6	1	0	0	2	1,004.48
									SEDYVDIVQGR	95.0%	82.0	22.3	8	0	0	2	1,280.61
									SQVVFSAEELIYPDR	95.0%	40.8	22.0	1	0	0	2	1,752.88
									VGVWNVPYISNIYLIK	95.0%	50.0	16.1	1	0	0	2	1,878.05
Threonyl-tRNA synthetase, cytoplasmic	SYTC_HUMAN	TARS	83,420	100.00%	33	37	191	45.50%	ADMETLQR	95.0%	64.4	22.0	6	0	0	2	963.46
									AEHDSILAEK	95.0%	49.9	21.4	5	0	0	2	1,112.56
									AELNPWPEYIYTR	95.0%	57.3	22.7	8	0	0	2	1,651.81
									AILGSVER	95.0%	56.8	23.7	3	0	0	2	844.49
									CGPLIDLCR	95.0%	60.3	22.5	2	0	0	2	1,103.53
									ETLLAMFK	95.0%	42.7	20.7	4	0	0	2	968.51
									FLGDIEVWDQAEK	95.0%	68.7	22.7	13	0	0	2	1,549.75
									FNLTYVSHDGDDK	95.0%	67.1	19.6	2	2	0	2	1,510.68
									FQEEAK	95.0%	32.3	23.2	1	0	0	2	751.36
									GAYIYNALIEFIR	95.0%	78.3	20.9	9	0	0	2	1,542.83
									GCLDFLR	95.0%	31.5	23.2	1	0	0	2	880.44
									GFQEVVTPNIFNSR	95.0%	71.8	23.2	12	0	0	2	1,607.82
									IDIQIK	95.0%	32.3	20.5	2	0	0	2	729.45
									IYGISFPDPK	95.0%	63.7	22.2	25	0	0	2	1,136.60
									KETLLAMFK	95.0%	37.6	19.7	2	1	0	2	1,080.61
									LADFGVLHR	95.0%	60.4	20.3	2	0	0	2	1,027.57
									LEMYNILK	95.0%	39.0	21.1	3	0	0	2	1,023.56
									LNLSTRPEK	95.0%	32.9	22.2	0	2	0	2	1,057.60
									MGGEEKPIGAGEEK	95.0%	59.1	20.6	10	8	0	2	1,447.67
									MIAILTENYGGK	95.0%	62.2	22.5	6	0	0	2	1,309.68
									NELSGALTGLTR	95.0%	97.8	22.5	17	0	0	2	1,231.66
									NSSTYWEGK	95.0%	39.7	18.9	2	0	0	2	1,071.47
									QLENSLNEFGEK	95.0%	67.8	22.1	2	0	0	2	1,407.68
									QLENSLNEFGEKWELNSGDGAFYGPK	95.0%	90.1	20.1	0	4	0	2	2,929.36
									QVDAESWK	95.0%	41.4	21.3	3	0	0	2	962.46
									QVMVVPVGPTCDEYAQK	95.0%	67.5	21.8	4	0	0	2	1,936.92
									TISETIER	95.0%	39.7	23.8	5	0	0	2	948.50
									TTPYQIACGISQGLADNTVIAK	95.0%	103.0	20.9	4	2	0	2	2,321.18
									TVYSVFGFSFK	95.0%	35.9	22.7	2	0	0	2	1,281.65
									VNTPTTTVYR	95.0%	38.7	22.5	1	0	0	2	1,151.61
									VTLPDGK	95.0%	32.5	21.1	3	0	0	2	729.41
									WELNSGDGAFYGPK	95.0%	60.9	20.8	2	0	0	2	1,540.71
									WPFWLSPR	95.0%	46.6	23.9	11	0	0	2	1,088.57
Nuclear mitotic apparatus protein 1	NUMA1_HUMAN	NUMA1	238,242	100.00%	6	6	10	3.55%	AALMESQGQQQEER	95.0%	67.6	19.7	4	0	0	2	1,620.73
									ALQQVQEK	94.6%	30.1	20.6	1	0	0	2	943.52
									AVQAQGGESQQEAQR	95.0%	86.3	22.2	1	0	0	2	1,586.75
									DSALETLQGQLEEK	95.0%	48.0	23.0	1	0	0	2	1,560.78

									DSAQTSVTQAQR	95.0%	38.2	22.6	2	0	0	2	1,291.62
									LLQAETASNSAR	95.0%	40.5	23.3	1	0	0	2	1,260.65
Disintegrin and metalloproteinase domain-containing protein 15	ADA15_HUMAN	ADAM15	87,726	99.50%	2	2	4	3.69%	SYTLEQGPGDLQGPPIISR	95.0%	74.7	21.0	3	0	0	2	2,028.04
									VLQTSLPEPLR	95.0%	31.0	17.7	1	0	0	2	1,252.73
von Willebrand factor A domain-containing protein 1	VWA1_HUMAN	VWA1	46,786	100.00%	4	4	5	15.70%	EFVGQLVAPLPLGTGALR	95.0%	38.0	15.6	2	0	0	2	1,838.05
									EQLFAEASGARPGVPK	95.0%	29.0	21.8	0	1	0	2	1,656.87
									MGDTHTGLALVYAK	95.0%	31.6	22.7	0	1	0	2	1,492.75
									VLVWVTDGGSSDPVGPPMQELK	95.0%	69.8	21.4	1	0	0	2	2,327.16
Eukaryotic translation initiation factor 3 subunit I	EIF3I_HUMAN	EIF3I	36,484	100.00%	4	4	7	13.80%	DPSQIDNNEPYMK	95.0%	51.0	16.6	2	0	0	2	1,566.67
									LFDSTTLEHQK	95.0%	44.7	23.1	2	0	0	2	1,318.66
									QINDIQLSR	95.0%	34.4	22.0	1	0	0	2	1,086.59
									SYSSGGEDGYVR	95.0%	54.2	16.7	2	0	0	2	1,276.54
Epithelial discoidin domain-containing receptor 1	DDR1_HUMAN	DDR1	101,112	99.90%	2	2	5	2.19%	DRWGQEVISGNEDPEGVVLK	95.0%	42.2	21.7	0	1	0	2	2,227.10
									WGQEVISGNEDPEGVVLK	95.0%	92.0	22.2	4	0	0	2	1,955.97
Septin-7	SEPT7_HUMAN	SEPT7	50,662	100.00%	6	6	13	15.30%	DVTNNVHYENYR	95.0%	71.4	20.9	2	0	0	2	1,523.69
									FEDYLNAESR	95.0%	66.7	20.3	2	0	0	2	1,243.56
									NLEGYVGFANLPNQVYR	95.0%	61.7	21.6	1	0	0	2	1,953.98
									SPLAQMEEERR	94.8%	26.4	24.2	0	1	0	2	1,361.65
									TVQVEQSK	95.0%	33.8	23.7	1	0	0	2	918.49
									VNIIPLIAK	95.0%	39.9	9.5	6	0	0	2	980.65
Tropomyosin alpha-3 chain	TPM3_HUMAN	TPM3	32,802	100.00%	4	4	15	34.50%	AADAEAEVASLNR	95.0%	60.6	21.8	5	0	0	2	1,316.64
									AEFAER	95.0%	30.5	22.0	1	0	0	2	722.35
									HIAEEADR	95.0%	38.5	22.0	4	0	0	2	940.45
									IQLVEEELDR	95.0%	69.4	22.3	10	0	0	2	1,243.65
									IQLVEEELDRAQER	95.0%	50.9	21.9	7	6	0	2	1,727.89
									KLVIIEGDLER	95.0%	41.8	18.3	1	0	0	2	1,284.75
									KYEEVAR	95.0%	30.6	22.4	1	0	0	2	894.47
									LDKENALDR	95.0%	32.3	23.4	0	2	0	2	1,073.56
									LEEAEKAADESER	95.0%	77.8	21.9	11	7	0	2	1,476.68
									LKEAETR	95.0%	42.4	24.5	6	0	0	2	846.47
									MELQEIQLK	95.0%	56.2	23.9	5	0	0	2	1,131.61
									RIQLVEEELDR	95.0%	32.9	21.9	2	2	0	2	1,399.75
									YEEVAR	95.0%	38.1	22.0	3	0	0	2	766.37
Four and a half LIM domains protein 1	FHL1_HUMAN	FHL1	36,244	99.50%	2	2	3	7.12%	AIVAGDQNVEYK	95.0%	44.2	23.2	2	0	0	2	1,306.66
									QVIGTGSFFPK	95.0%	36.8	21.6	1	0	0	2	1,180.64
Tumor necrosis factor receptor superfamily member 21	TNR21_HUMAN	TNFRSF21	71,827	100.00%	3	3	7	5.19%	GPEASLAQLISALR	95.0%	50.8	19.3	2	0	0	2	1,425.81
									GTETEDVR	95.0%	33.1	21.1	2	0	0	2	906.42
									GTFSDVPSSVMK	95.0%	45.8	21.4	3	0	0	2	1,270.60
Importin-9	IPO9_HUMAN	IPO9	115,946	99.90%	2	2	3	3.27%	EALVDTLTGILSPVQEVR	95.0%	48.8	18.5	2	0	0	2	1,940.07
									YSNDPVVASLAQDIFK	95.0%	38.1	21.8	1	0	0	2	1,766.90
Collagen alpha-1(VII) chain	CO7A1_HUMAN	COL7A1	295,200	100.00%	14	15	84	6.56%	DLVLSEPSSQSLR	95.0%	54.3	21.8	9	0	0	2	1,430.75
									EGSASVLTVR	95.0%	37.2	23.4	1	0	0	2	1,018.55
									FATVQYSDDPR	95.0%	37.3	20.8	2	0	0	2	1,298.60
									GATGVQGER	95.0%	41.6	21.7	1	0	0	2	874.44
									GPGQEVPGSPQTLPGISSSQR	95.0%	73.3	22.6	6	0	0	2	2,079.05
									LSVLGPAGEGPSAEVTAR	95.0%	70.1	21.0	14	0	0	2	1,710.90
									QEVNVPAGETSVR	95.0%	59.4	22.3	13	0	0	2	1,385.70
									SFLEGLVLPFSGAASAQGVR	95.0%	55.8	19.7	1	0	0	2	2,006.07
									SQDLVDTAAQR	95.0%	56.4	23.2	11	0	0	2	1,203.60
									VLSGGPTQQQELGPGQGSVLLR	95.0%	89.5	19.5	4	2	0	2	2,221.19
									VPSIELR	95.0%	36.3	17.9	3	0	0	2	813.48

									VQYTPLTGLGQPLPSER	95.0%	95.4	19.9	12	0	0	2	1,855.99
									VSWSPVPGATQYR	95.0%	45.1	23.4	3	0	0	2	1,447.73
									VVVSDATR	95.0%	40.4	24.5	2	0	0	2	846.47
Alpha-mannosidase 2	MA2A1_HUMAN	MAN2A1	131,128	100.00%	10	10	21	10.50%	FLSSSLYTALTEAR	95.0%	82.8	23.2	2	0	0	2	1,558.81
									FYTDLNGYQIQPR	95.0%	42.7	22.2	2	0	0	2	1,614.79
									GLEQGIQDNK	95.0%	51.1	22.2	4	0	0	2	1,101.55
									ILESASSNSHLADYVLYK	95.0%	30.9	21.5	0	1	0	2	2,010.02
									ITANLFR	95.0%	30.5	21.8	1	0	0	2	834.48
									LLAENNEIISNIR	95.0%	75.2	21.3	4	0	0	2	1,498.82
									NYQQLFDYMNSQSK	95.0%	66.6	20.0	1	0	0	2	1,781.78
									SQDSLPQK	95.0%	33.2	22.5	1	0	0	2	902.46
									VLLAPLGDDFR	95.0%	42.6	22.0	3	0	0	2	1,215.67
									YLVVYNPLEQDR	95.0%	46.4	23.0	2	0	0	2	1,508.78
Phosphoglycerate kinase 2	PGK2_HUMAN	PGK2	44,779	99.50%	2	2	13	19.70%	DVLFLK	95.0%	42.5	15.7	2	0	0	2	734.45
									LGDVYVNDAFGTAHR	95.0%	105.0	22.5	25	76	0	2	1,634.79
									NQITNNQR	95.0%	32.9	20.9	1	0	0	2	987.50
									SLLGKDVLFLK	95.0%	44.0	9.0	2	0	0	2	1,232.76
									VDFNVPMK	95.0%	55.8	22.8	13	0	0	2	965.48
									VNEMIIGGGMAYTFLK	95.0%	46.3	22.5	12	0	0	2	1,759.88
									VSHVSTGGGASLELLEGK	95.0%	106.0	21.6	47	77	0	2	1,740.91
									WNTEDKVSHVSTGGGASLELLEGK	95.0%	61.5	21.1	0	33	7	2	2,514.25
Glucose-6-phosphate isomerase	G6PI_HUMAN	GPI	63,130	100.00%	23	29	381	42.50%	AVLHVALR	95.0%	41.5	9.0	3	0	0	2	878.56
									DPQFQK	95.0%	35.9	23.3	4	0	0	2	762.38
									DVMPEVNK	95.0%	31.9	22.3	1	0	0	2	947.45
									ELQAAGK	95.0%	33.0	23.6	1	0	0	2	716.39
									EWFLQAAK	95.0%	48.4	23.1	12	0	0	2	992.52
									FAAYFQQGDMESNGK	95.0%	86.3	19.3	6	0	0	2	1,692.73
									HFVALSTNTTK	95.0%	55.4	23.1	10	0	0	2	1,218.65
									ILLANFLAQTEALMR	95.0%	97.3	19.2	69	28	0	2	1,719.95
									INYTEGR	95.0%	39.8	22.7	7	0	0	2	852.42
									KELQAAGK	95.0%	45.9	23.7	3	0	0	2	844.49
									KIEPELDGSAQVTSHDASTNGLINFIK	95.0%	51.9	19.2	0	16	0	2	2,884.47
									LFDANKDR	95.0%	33.3	24.0	2	0	0	2	978.50
									LTPFMLGALVAMYEHK	95.0%	56.9	21.9	4	0	0	2	1,852.93
									MLVDLAK	95.0%	37.7	21.0	4	0	0	2	805.45
									NLVTEDVMR	95.0%	52.5	22.6	12	0	0	2	1,092.54
									SELNLR	95.0%	33.3	25.1	5	0	0	2	731.41
									SNTPILVDGKDVMPEVNK	95.0%	58.6	22.3	8	7	0	2	1,972.01
									SPEDLER	95.0%	48.9	19.5	15	0	0	2	845.40
									TFTTQETITNAETAK	95.0%	119.0	21.9	69	1	0	2	1,655.81
									TFTTQETITNAETAKEWFLQAAK	95.0%	66.2	21.1	1	8	0	2	2,629.32
									TLAQLNPESSLFIIASK	95.0%	108.0	16.8	57	2	0	2	1,832.02
									VFEGNRPTNSIVFTK	95.0%	42.3	21.4	6	0	0	2	1,708.90
									VWYVSNIDGTHIAK	95.0%	72.5	22.8	14	6	0	2	1,602.83
Acidic leucine-rich nuclear phosphoprotein 32 family member A	AN32A_HUMAN	ANP32A	28,568	100.00%	10	11	42	35.70%	CPNLTHLNLSGNK	95.0%	39.5	23.2	0	2	0	2	1,467.74
									DLSTIEPLK	95.0%	33.0	21.5	3	0	0	2	1,015.57
									ELVLDNSR	95.0%	41.8	23.2	5	0	0	2	945.50
									IKDLSTIEPLK	95.0%	50.2	17.5	6	3	0	2	1,256.75
									KLELSDNR	95.0%	51.6	22.8	3	0	0	2	974.53
									LLPQLTYLDGYDR	95.0%	69.2	21.9	4	0	0	2	1,566.82
									LLPQLTYLDGYDRDDK	95.0%	35.1	22.8	1	0	0	2	1,924.97

									SLDLFNCEVTNLNDYR	95.0%	103.0	20.7	5	0	0	2	1,972.91
									TPSDVKELVLDNSR	95.0%	27.4	22.2	0	2	0	2	1,572.82
									VSGGLEVLAEK	95.0%	78.7	22.9	8	0	0	2	1,101.62
Hypoxia up-regulated protein 1	HYOU1_HUMAN	HYOU1	111,319	100.00%	4	4	7	5.81%	AANSLEAFIFETQDK	95.0%	46.8	22.5	0	2	0	2	1,683.82
									DAVVYPILVEFTR	95.0%	41.9	20.6	2	0	0	2	1,521.83
									LGNTISSLFGGGTTPDAK	95.0%	55.3	21.9	1	0	0	2	1,735.89
									LPATEKPVLLSK	95.0%	46.6	14.0	2	0	0	2	1,295.79
Phosphoglycolate phosphatase	PGP_HUMAN	PGP	33,989	100.00%	3	3	6	11.80%	GETAVPGAPEALR	95.0%	36.5	21.9	1	0	0	2	1,267.66
									LGFITNNSSK	95.0%	39.2	21.7	2	0	0	2	1,080.57
									TILTLTGVSTLGDVK	95.0%	65.0	16.8	3	0	0	2	1,517.88
14-3-3 protein beta/alpha	1433B_HUMAN	YWHAB	28,065	100.00%	7	11	180	63.00%	AVTEQGHELSNEER	95.0%	105.0	21.6	27	9	0	2	1,598.74
									DNLTLWTSENQGDEGDAGEGEN	95.0%	122.0	14.3	4	0	0	2	2,350.96
									DSTLIMQLLR	95.0%	79.3	21.3	113	0	0	2	1,189.66
									EMQPTHPIR	95.0%	67.1	22.4	13	0	0	2	1,108.56
									KEMQPTHPIR	95.0%	43.9	22.5	0	11	0	2	1,252.65
									LAEQAER	95.0%	59.4	21.6	36	0	0	2	816.42
									LAEQAERYDDMAAAMK	95.0%	52.0	20.8	4	1	0	2	1,812.83
									NLLSVAYK	95.0%	51.4	19.1	49	0	0	2	907.53
									NLLSVAYKNVVGAR	95.0%	79.1	17.7	4	2	0	2	1,503.86
									QTTVSNSQQAYQEAFEISK	95.0%	140.0	22.0	65	2	0	2	2,159.03
									QTTVSNSQQAYQEAFEISKK	95.0%	78.3	22.2	2	11	0	2	2,287.12
									TAFDEAIAELDTLNEESYK	95.0%	96.7	21.5	14	6	0	2	2,159.00
									VISSIEQK	95.0%	65.9	22.9	27	0	0	2	903.52
									YDDMAAAMK	95.0%	70.3	14.5	11	0	0	2	1,015.42
									YLIPNATQPESK	95.0%	71.3	23.7	14	0	0	2	1,360.71
									YLSEVASGDNK	95.0%	76.9	21.7	26	0	0	2	1,182.56
Biliverdin reductase A	BIEA_HUMAN	BLVRA	33,411	100.00%	4	4	11	16.20%	FGFPAFSGISR	95.0%	53.9	22.0	2	0	0	2	1,185.61
									GSLLFTAGPLEEER	95.0%	44.2	23.0	3	0	0	2	1,518.78
									SGSLENVPNVGVNK	95.0%	61.4	21.6	4	0	0	2	1,413.73
									SPLSWIEEK	95.0%	36.6	23.1	2	0	0	2	1,088.56
Ras-related protein Rab-10	RAB10_HUMAN	RAB10	22,524	99.50%	2	2	4	22.50%	AFLTLAEDILR	95.0%	49.9	20.6	3	0	0	2	1,261.72
									LLLIGDSGVGK	95.0%	58.7	20.3	2	0	0	2	1,071.64
									LQIWDTAGQER	95.0%	61.5	22.2	7	0	0	2	1,316.66
									NIDEHANEDVER	95.0%	38.0	18.6	1	0	0	2	1,440.64
Plectin-1	PLEC1_HUMAN	PLEC1	531,766	100.00%	106	109	722	24.20%	AALAHSEEVTASQVAATK	95.0%	28.9	21.5	0	1	0	2	1,783.92
									AALEEVER	95.0%	52.1	22.2	7	0	0	2	916.47
									AGVAAPATQVAQVTLQSVQR	95.0%	78.0	17.1	2	10	0	2	1,995.10
									AGVVGPELHEQLLSAEK	94.7%	26.4	20.9	0	1	0	2	1,776.95
									AKLEQLFQDEVAK	95.0%	53.2	22.0	2	0	0	2	1,518.82
									ALQALEELR	95.0%	59.2	21.5	14	0	0	2	1,042.59
									APVPASELLASGVLSR	95.0%	112.0	18.0	22	1	0	2	1,566.89
									AQAEQAALR	95.0%	53.9	21.1	2	0	0	2	957.51
									AQLEPVASPAK	95.0%	48.8	19.8	5	0	0	2	1,110.62
									AQVEQELTTLR	95.0%	75.7	23.1	13	0	0	2	1,287.69
									ASDSELER	95.0%	57.2	21.1	5	0	0	2	906.42
									DDGTGQLLLPLSDAR	95.0%	57.4	22.0	6	0	0	2	1,570.81
									DPYSGSTISLFQAMQK	95.0%	88.4	21.8	3	0	0	2	1,772.85
									DSQDAGGFGPEDR	95.0%	61.0	14.9	13	0	0	2	1,350.56
									DTHDQLSEPSEVR	95.0%	46.5	21.2	1	0	0	2	1,512.69
									EAEGQLQK	95.0%	36.2	22.5	5	0	0	2	902.46
									EAQAVPATLPELEATK	95.0%	53.1	19.9	5	0	0	2	1,667.89

ELAQEQAR	95.0%	56.9	21.0	14	0	0	2	944.48
ELQNAGDR	95.0%	50.4	20.8	5	0	0	2	902.43
EQELQQTLQQEQSVLDQLR	95.0%	124.0	21.4	6	0	0	2	2,313.17
ESADPLGAWLQDAR	95.0%	87.9	22.0	29	0	0	2	1,528.74
FLEVQYLTGGLIEPDTPGR	95.0%	84.1	21.2	4	0	0	2	2,105.09
FPVTDAVNK	95.0%	31.8	23.3	2	0	0	2	990.53
FRELAEEAAR	95.0%	29.4	23.7	0	1	0	2	1,191.61
GAQEVGER	95.0%	59.1	20.7	5	0	0	2	845.41
GGAEGELQALR	95.0%	90.5	22.5	11	0	0	2	1,100.57
GGELVYTDSEAR	95.0%	59.1	22.2	2	0	0	2	1,296.61
GIYQSLEGAVQAGQLK	95.0%	81.3	21.0	6	0	0	2	1,661.89
GKAEEQAVR	95.0%	34.5	22.4	1	0	0	2	987.52
GTQGAEEVLR	95.0%	40.6	22.1	2	0	0	2	1,059.54
GYFSEEMNR	95.0%	35.0	12.6	1	0	0	2	1,148.47
GYYSPYSVSGSGSTAGSR	95.0%	81.6	19.0	1	0	0	2	1,782.79
HISDLYEDLR	95.0%	39.1	22.0	2	0	0	2	1,260.62
LAAEQELIR	95.0%	40.0	20.1	2	0	0	2	1,042.59
LAAIGEATR	95.0%	46.2	24.1	11	0	0	2	901.51
LAEDEAFQR	95.0%	64.9	22.7	13	0	0	2	1,078.52
LEDLLQDAQDEKEQLNEYK	95.0%	48.9	21.5	0	10	0	2	2,321.12
LEQLFQDEVAK	95.0%	49.6	23.0	1	0	0	2	1,319.68
LFNAIIHR	95.0%	54.7	16.0	4	0	0	2	983.58
LGFHLPLEVAYQR	95.0%	29.5	21.1	0	1	0	2	1,542.84
LISLFQAMK	95.0%	36.1	19.7	1	0	0	2	1,050.60
LKQSAEEQAQAR	95.0%	60.9	22.9	0	17	0	2	1,358.70
LLDAQLATGGIVDPR	95.0%	59.4	19.1	4	0	0	2	1,538.85
LLDPEDVDVPQPDEK	95.0%	82.8	22.5	11	0	0	2	1,708.83
LLEAAAQSTK	95.0%	46.5	23.3	3	0	0	2	1,031.57
LLFNDVQTLK	95.0%	53.8	20.0	6	0	0	2	1,190.68
LLLWSQR	95.0%	41.9	20.3	5	0	0	2	915.54
LQAEEAER	95.0%	46.6	20.1	8	0	0	2	945.46
LQAEEVAQQK	95.0%	48.4	23.0	16	0	0	2	1,143.60
LQEAGILSAEELQR	95.0%	87.2	22.2	4	0	0	2	1,556.83
LQEALR	95.0%	32.0	23.3	1	0	0	2	729.43
LQLEATER	95.0%	41.5	22.5	3	0	0	2	959.52
LQNVQIALDYLR	95.0%	77.8	19.2	4	0	0	2	1,445.81
LRAETEQGEQQR	95.0%	54.8	22.7	6	17	0	2	1,444.71
LSVAAQEAAR	95.0%	65.2	22.4	5	0	0	2	1,015.55
LSYTQLLR	95.0%	62.8	17.2	3	0	0	2	993.57
LTAEDLFEAR	95.0%	59.6	23.5	4	0	0	2	1,164.59
LVASMEEAR	95.0%	42.4	22.5	3	0	0	2	1,021.50
MQAVQEATR	95.0%	62.2	22.1	11	0	0	2	1,049.51
MQEEVVR	95.0%	42.9	22.2	5	0	0	2	906.44
NDDIADGNPK	95.0%	55.8	17.6	7	0	0	2	1,058.48
NLLDEELQR	95.0%	53.1	23.2	6	0	0	2	1,129.59
NLVDNITGQR	95.0%	54.5	23.0	2	0	0	2	1,129.60
QAEEIGEK	95.0%	40.1	22.1	4	0	0	2	903.44
QEQIQAMPLADSQAVR	95.0%	69.7	21.3	1	0	0	2	1,800.89
QLAAEEER	95.0%	45.4	20.1	6	0	0	2	945.46
QLAEEDAAR	95.0%	65.0	20.0	7	0	0	2	1,002.49
QLAEEDLAQQR	95.0%	75.8	21.9	19	0	0	2	1,300.65

									QLAEGTAQQR	95.0%	64.7	20.5	12	0	0	2	1,101.57
									QLEMSAEAER	95.0%	53.5	20.4	9	0	0	2	1,179.53
									QLLEEELAR	95.0%	40.4	23.1	8	0	0	2	1,100.60
									QLQLAQEAAQK	95.0%	74.8	21.7	12	0	0	2	1,227.67
									QLQLAQEAAQKR	95.0%	45.3	20.3	1	0	0	2	1,383.77
									QQEELLAEENQR	95.0%	85.0	22.1	5	0	0	2	1,486.71
									QQGLASYDYVR	95.0%	38.6	21.7	2	0	0	2	1,299.63
									QSAEEQAQAR	95.0%	48.9	20.4	2	0	0	2	1,117.52
									QSSEAEIQAK	95.0%	61.3	23.7	6	0	0	2	1,090.54
									QTNLENLDQAFSVAER	95.0%	134.0	23.6	16	0	0	2	1,834.89
									QVAEEAAR	95.0%	38.6	21.8	6	0	0	2	873.44
									QVEEEILALK	95.0%	64.6	22.7	15	0	0	2	1,171.66
									QVQVALETAQR	95.0%	46.3	22.9	5	0	0	2	1,242.68
									RAAEEAEEAR	95.0%	35.6	20.5	1	0	0	2	1,131.54
									RPELEDSTLR	95.0%	41.9	23.3	4	0	0	2	1,215.63
									RQELEAELAK	95.0%	33.1	22.4	2	0	0	2	1,186.64
									RQEQIQAMPLADSQAVR	95.0%	46.7	22.1	0	1	0	2	1,956.99
									SAEAELQSK	95.0%	63.2	23.5	15	0	0	2	962.48
									SELELTLGK	95.0%	33.4	22.0	1	0	0	2	989.55
									SIITYVSSLYDAMPR	95.0%	63.7	21.8	9	0	0	2	1,731.86
									SIQEELQQLR	95.0%	75.4	22.3	18	0	0	2	1,243.66
									SKEQAELEAAR	95.0%	37.1	23.3	0	6	0	2	1,231.63
									SLAAEEEAAR	95.0%	72.1	22.1	24	0	0	2	1,046.51
									SLESLHSFVAAATK	95.0%	83.3	22.6	4	0	0	2	1,460.77
									SLQEEHVAVAQLR	95.0%	40.4	22.1	0	10	0	2	1,479.79
									SLSAIYLEK	95.0%	40.7	19.4	5	0	0	2	1,023.57
									SLVPAAELLESR	95.0%	45.9	21.1	4	0	0	2	1,284.72
									SMVEEGTGLR	95.0%	47.4	21.3	2	0	0	2	1,094.52
									SNAEDTLR	95.0%	46.2	22.5	3	0	0	2	905.43
									SQVEEELFSVR	95.0%	86.4	22.9	9	0	0	2	1,322.66
									SSIAGLLLK	95.0%	46.9	15.8	7	0	0	2	901.57
									SWSLATFR	95.0%	37.6	22.9	4	0	0	2	967.50
									VAQLLER	95.0%	46.6	20.7	4	0	0	2	828.49
									VKAEAEAAR	95.0%	38.8	23.1	3	0	0	2	944.52
									VLALPEPSPAAPTLR	95.0%	58.3	16.7	22	0	0	2	1,531.89
									VPDVQDGVR	95.0%	34.3	21.3	2	0	0	2	984.51
									VQSGSESVIQEYVDLR	95.0%	101.0	22.5	6	0	0	2	1,808.90
									WQAVLAQTDVR	95.0%	61.0	23.2	16	0	0	2	1,286.69
Hypoxanthine-guanine phosphoribosyltransferase	HPRT_HUMAN	HPRT1	24,562	100.00%	8	8	32	39.40%	FFADLLDYIK	95.0%	60.9	22.4	5	0	0	2	1,244.66
									NVLIVEDIIDTGK	95.0%	72.9	21.2	4	0	0	2	1,428.80
									SIPMTVDFIR	95.0%	32.0	23.2	2	0	0	2	1,178.62
									SYCNDQSTGDIK	95.0%	45.5	16.6	2	0	0	2	1,387.58
									TMQTLLSLVR	95.0%	64.5	19.8	9	0	0	2	1,161.67
									VASLLVK	95.0%	32.6	19.1	2	0	0	2	729.49
									VFIPHGLIMDR	95.0%	41.0	20.5	0	5	0	2	1,297.71
									VIGGDDLSTLTGK	95.0%	93.2	22.6	3	0	0	2	1,275.68
Receptor-type tyrosine-protein phosphatase S	PTPRS_HUMAN	PTPRS	217,075	100.00%	4	4	12	2.77%	SPQGLGAFTPVVR	95.0%	53.6	20.7	2	0	0	2	1,328.73
									TDEDVPSAPPR	95.0%	32.6	21.5	1	0	0	2	1,183.56
									TGEQAPASAPR	95.0%	66.8	22.4	6	0	0	2	1,084.54
									VLAFTSVGDGPLSDPIQVK	95.0%	85.9	20.3	3	0	0	2	1,943.05
Malate dehydrogenase,	MDHM_HUMAN	MDH2	35,486	100.00%	17	21	365	62.70%	AGAGSATLSMAYAGAR	95.0%	112.0	22.0	74	0	0	2	1,454.71

mitochondrial									ANTFVAELK	95.0%	83.3	21.7	15	0	0	2	992.54
									FVFSLVDAMNGK	95.0%	87.8	22.4	11	0	0	2	1,343.67
									GCDVVVIPAGVPR	95.0%	71.3	21.4	5	0	0	2	1,338.72
									GYLGPEQLPDCLK	95.0%	54.5	23.2	6	0	0	2	1,489.74
									HGVYNPNK	95.0%	39.7	21.1	2	0	0	2	928.46
									IFGVTTLDIVR	95.0%	66.0	17.3	36	0	0	2	1,233.72
									IQEAGTEVVK	95.0%	46.8	23.5	9	0	0	2	1,073.58
									KGEDFVK	95.0%	48.3	22.4	2	0	0	2	822.44
									LTLYDIAHTPGVAADLSHIETK	95.0%	99.3	18.9	2	50	1	2	2,365.24
									MISDAIPELK	95.0%	72.9	23.6	24	0	0	2	1,116.60
									SQETECTYFSTPLLLGK	95.0%	106.0	22.1	3	0	0	2	1,973.95
									TIIPLISQCTPK	95.0%	65.3	19.9	2	0	0	2	1,370.77
									VAVLGASGGIGQPLSLLLK	95.0%	129.0	3.0	61	2	0	2	1,793.09
									VDFPQDQLTALTGR	95.0%	108.0	22.9	32	0	0	2	1,560.80
									VNVPVIGGHAGK	95.0%	52.3	17.7	9	15	0	2	1,147.66
									VSSFEEK	95.0%	35.8	21.5	4	0	0	2	825.40
Protein jagged-1	JAG1_HUMAN	JAG1	133,778	100.00%	8	9	26	7.96%	ASHSGMINPSR	95.0%	45.4	20.7	2	3	0	2	1,172.55
									CVCPPQWTGK	95.0%	36.2	18.2	2	0	0	2	1,232.56
									DDGNPIKEITDK	95.0%	34.9	22.6	0	1	0	2	1,344.67
									DLVNDFYCDCK	95.0%	75.6	13.2	6	0	0	2	1,448.58
									IIDLVSKR	95.0%	40.6	17.1	2	0	0	2	943.59
									SSSLQPVK	95.0%	58.8	23.4	6	0	0	2	845.47
									VTAGGPCSFGSGSTPVIGGNTFNLK	95.0%	98.7	22.1	2	0	0	2	2,425.18
									YISSNVCGPHGK	95.0%	52.0	21.3	2	0	0	2	1,318.62
Cadherin-3	CADH3_HUMAN	CDH3	91,401	100.00%	6	7	58	14.70%	DPAGWLAMDPDSGQVTAVGTLDREDEQFVR	95.0%	56.6	19.3	0	1	0	2	3,291.52
									ETGWLLLNKPLDREEIAK	95.0%	29.2	17.6	0	2	0	2	2,125.17
									GLDFEAK	95.0%	41.4	23.6	2	0	0	2	779.39
									IFYSITGPGADSPPEGVFAVEK	95.0%	123.0	21.3	16	0	0	2	2,281.14
									LTVTDLDAPNSPAWR	95.0%	77.7	21.7	13	0	0	2	1,655.84
									STGTISVISSGLDR	95.0%	96.4	21.8	22	0	0	2	1,392.73
									YEAHVPENAVGHEVQR	95.0%	67.3	23.4	0	2	2	2	1,834.88
Cytoplasmic aconitate hydratase	ACOC_HUMAN	ACO1	98,383	100.00%	9	11	24	17.40%	FVEFFGPGVAQLSIADR	95.0%	74.2	21.3	2	0	0	2	1,852.96
									GPFLLGIK	95.0%	44.1	21.2	3	0	0	2	844.53
									IDFEKEPLGVNAK	95.0%	52.5	22.6	2	2	0	2	1,459.78
									IETVNESWNALATPSDK	95.0%	84.6	22.1	2	0	0	2	1,874.91
									QAPQTIHLPSGEILDVFDAAER	95.0%	38.2	20.8	0	3	0	2	2,407.23
									SNLVGMGVIPLEYLPGENADALGLTGQER	95.0%	30.1	20.5	0	1	0	2	3,029.53
									SPPFFENLTLDLQPPK	95.0%	67.1	21.3	2	0	0	2	1,842.96
									VILQDFTGVPAVVDFAAMR	95.0%	99.2	21.0	2	2	0	2	2,065.08
									YQQAGLPLIVLAGK	95.0%	65.1	14.9	3	0	0	2	1,470.87
Elongation factor 2	EF2_HUMAN	EEF2	95,322	100.00%	25	27	126	31.90%	ALLELQLEPEELYQTFQR	95.0%	94.1	20.8	3	1	0	2	2,220.16
									ARPFPDGLAEDIDKGEVSAR	95.0%	60.9	22.1	0	3	4	2	2,143.08
									CLYASVLTAQPR	95.0%	55.6	22.5	2	0	0	2	1,378.72
									EDLYLKPIQR	95.0%	35.8	19.9	1	0	0	2	1,274.71
									EGIPALDNFLDKL	95.0%	69.5	22.4	24	0	0	2	1,444.77
									FSVSPVVR	95.0%	35.2	20.6	2	0	0	2	890.51
									GEGQLGPAER	95.0%	46.4	21.1	10	0	0	2	1,013.50
									GGGQIIPTAR	95.0%	38.3	20.3	4	0	0	2	969.55
									GHVFEESQVAGTPMFVVK	95.0%	39.9	22.4	0	2	0	2	1,977.97
									GPLMMYISK	95.0%	63.4	22.0	8	0	0	2	1,055.53
									IKPVLMMNK	94.8%	26.4	22.0	0	1	0	2	1,089.62

									IMGPNYTPGKK	95.0%	30.3	22.4	0	3	0	2	1,221.63
									IWCFGPDGTGPNILTDITK	95.0%	89.0	21.8	2	0	0	2	2,105.04
									KEDLYLKPIQR	95.0%	45.7	18.3	0	2	0	2	1,402.81
									KIWCFGPDGTGPNILTDITK	95.0%	29.0	21.5	0	1	0	2	2,233.13
									MVNFTVDQIR	95.0%	46.7	22.8	2	0	0	2	1,238.62
									QFAEMYVAK	95.0%	41.7	21.1	3	0	0	2	1,102.52
									SDPVVSYR	95.0%	32.2	22.6	2	0	0	2	922.46
									TFCQLILDPIFK	95.0%	58.5	21.5	5	0	0	2	1,494.80
									TGTITTFEHAHNMR	95.0%	38.9	20.4	0	4	0	2	1,615.77
									VFDAIMNFK	95.0%	43.0	20.8	2	0	0	2	1,100.55
									VFSGLVSTGLK	95.0%	84.6	18.8	17	0	0	2	1,107.64
									VNFTVDQIR	95.0%	80.2	23.8	12	0	0	1	1,091.59
									WLPAGDALLQMITIHLPSPVTAQK	95.0%	44.4	16.3	0	1	0	2	2,616.42
									YEWDVAEAR	95.0%	63.6	19.4	5	0	0	2	1,138.52
Cullin-associated NEDD8-dissociated protein 1	CAND1_HUMAN	CAND1	136,363	100.00%	12	12	26	12.80%	AADIDQEVKER	95.0%	38.1	23.3	1	0	0	2	1,273.64
									ALTLIAGSPLK	95.0%	57.0	17.4	3	0	0	2	1,083.68
									DLLDTVLPHLYNETK	95.0%	39.0	22.0	1	0	0	2	1,770.93
									EGPAVVGQFIQDVK	95.0%	49.1	21.9	2	0	0	2	1,486.79
									ITSEALLVTQQLVK	95.0%	73.6	14.3	2	0	0	2	1,542.91
									LGTLSALDILIK	95.0%	33.6	13.0	1	0	0	2	1,256.78
									LTLIDPETLLPR	95.0%	67.4	15.3	8	0	0	2	1,380.81
									MLTGPVYSQSTALTHK	95.0%	27.9	22.8	0	1	0	2	1,749.88
									SVILEAFSSPSEEVK	95.0%	65.8	22.1	2	0	0	2	1,621.83
									TVSPALISR	95.0%	30.9	21.4	1	0	0	2	943.56
									TYIQCIAAISR	95.0%	48.5	21.6	2	0	0	2	1,295.68
									VIRPLDQPSSFDATPYIK	95.0%	44.6	20.2	0	2	0	2	2,047.09
Proteasome subunit beta type-8	PSB8_HUMAN	PSMB8	30,337	100.00%	6	7	15	24.30%	AIAYATHR	95.0%	33.4	22.2	1	0	0	2	902.48
									ASAGSYISALR	95.0%	91.5	21.8	2	0	0	2	1,095.58
									FQHGVIAAVDSR	95.0%	50.7	22.2	2	0	0	2	1,299.68
									ISVSAASK	95.0%	51.7	21.8	2	0	0	2	762.44
									KGPGLYYVDEHGTR	95.0%	28.0	22.7	0	2	0	2	1,591.79
									VESTDVSDLLHQYR	95.0%	75.9	22.8	2	4	0	2	1,661.81
Exostosin-2	EXT2_HUMAN	EXT2	82,238	100.00%	5	5	11	7.52%	ADPVLYKDDFPEK	95.0%	51.5	22.4	2	0	0	2	1,536.76
									FASVFGTMPLK	95.0%	50.4	21.9	3	0	0	2	1,197.63
									HGESVLVLDK	95.0%	44.6	21.0	1	0	0	2	1,096.60
									LLVVWNNQNK	95.0%	38.4	20.8	1	0	0	2	1,227.69
									LPADSPIPER	95.0%	61.5	20.9	4	0	0	2	1,094.58
Nucleoredoxin	NXN_HUMAN	NXN	48,376	100.00%	4	4	8	15.90%	DYTNLPEAAPLLTILDMSAR	95.0%	50.8	22.4	1	0	0	2	2,220.12
									EVIAGPLLR	95.0%	41.6	13.0	1	0	0	2	967.59
									ISNIPSLIFLDATTGK	95.0%	72.6	18.1	4	0	0	2	1,689.94
									LRGDAAAGPGPGAGAGAAAEPEPR	95.0%	62.5	21.8	0	2	0	2	2,116.05
Protein MEMO1	MEMO1_HUMAN	MEMO1	33,716	100.00%	2	2	2	6.40%	TGMFER	95.0%	34.2	16.7	1	0	0	2	756.34
									YSYYDESQGEIYR	95.0%	52.4	16.6	1	0	0	2	1,672.71
Glycogen phosphorylase, liver form	PYGL_HUMAN	PYGL	97,134	100.00%	11	11	21	19.40%	DFNVGDYIQAVLDR	95.0%	87.8	22.8	3	0	0	2	1,624.80
									EYAQNIWNVEPSDLK	95.0%	48.0	22.8	2	0	0	2	1,805.87
									EYYEALPELK	95.0%	30.9	22.6	1	0	0	2	1,254.63
									FSQFLETEYK	95.0%	68.3	22.8	2	0	0	2	1,291.62
									IDDVAALDKK	95.0%	28.3	23.4	0	1	0	2	1,087.60
									INPSSMFDVQVK	95.0%	52.3	22.9	2	0	0	2	1,364.69
									LITSVADVVNNDPMVGSK	95.0%	108.0	21.8	4	0	0	2	1,874.95
									LVIDQIDNGFFSPK	95.0%	65.4	22.2	1	0	0	2	1,592.83

									TFAYTNHTVLPEALER	95.0%	44.5	22.1	0	2	0	2	1,861.94
									VIFLENYR	95.0%	50.2	21.6	5	0	0	2	1,053.57
									VLYPNDNFFEGK	95.0%	55.6	21.8	3	0	0	2	1,442.70
									WLLLCNPGLAELIAEK	95.0%	48.8	19.2	2	0	0	2	1,840.00
									YEYGIFNQK	95.0%	35.8	22.1	1	0	0	2	1,161.56
40S ribosomal protein S12	RS12_HUMAN	RPS12	14,497	99.50%	2	2	4	18.90%	LVEALCAEHQINLIK	95.0%	35.8	18.4	0	2	0	2	1,750.95
									TALIHDGLAR	95.0%	30.0	20.5	0	2	0	2	1,066.60
Moesin	MOES_HUMAN	MSN	67,804	100.00%	49	60	875	69.30%	AKFYPEDVSEELIQDITQR	95.0%	30.5	21.4	0	5	0	2	2,281.14
									ALELEQER	95.0%	53.2	23.3	22	0	0	2	987.51
									ALTSELANAR	95.0%	81.8	22.9	34	0	0	2	1,045.56
									ALTSELANARDESK	95.0%	83.4	23.0	6	0	0	2	1,504.76
									AMLENEK	95.0%	36.3	22.5	3	0	0	2	850.40
									AMLENEKK	95.0%	42.0	23.6	1	0	0	2	962.50
									APDFVFYAPR	95.0%	80.6	21.7	84	0	0	2	1,182.59
									AQMVQEDLEK	95.0%	64.3	21.6	41	0	0	2	1,206.57
									AQQELEEQTR	95.0%	66.8	22.4	6	0	0	2	1,231.59
									AQQELEEQTRR	95.0%	38.5	22.6	0	1	0	2	1,387.69
									EALLQASR	95.0%	68.0	23.7	10	0	0	2	887.50
									EDAVLEYLK	95.0%	41.3	23.2	7	0	0	2	1,079.56
									EGILNDDIYCPPETAVLLASYAVQSK	95.0%	36.2	21.6	0	2	0	2	2,866.42
									EKEELMER	95.0%	33.2	21.6	2	0	0	2	1,079.50
									ESEAVEWQQK	95.0%	53.0	20.5	8	0	0	2	1,233.58
									ESPLLFK	95.0%	30.4	20.7	1	0	0	2	833.48
									EVWFFGLQYQDTK	95.0%	72.8	22.1	8	0	0	2	1,660.80
									FVIKPIDK	95.0%	41.1	17.2	6	0	0	2	959.59
									FYPEDVSEELIQDITQR	95.0%	96.5	22.3	37	17	0	2	2,082.00
									GFSTWLK	95.0%	37.8	21.2	4	0	0	2	838.45
									GMLREDAVLEYLK	95.0%	45.5	21.3	2	7	0	2	1,552.80
									IAQDLEMYGVNYFSIK	95.0%	102.0	22.4	60	0	0	2	1,906.93
									IGFPWSEIR	95.0%	68.8	23.4	121	0	0	2	1,104.58
									ILALCMGNHELYMR	95.0%	40.7	21.7	2	0	0	2	1,752.82
									ISQLEMAR	95.0%	64.0	23.4	18	0	0	2	963.49
									KAPDFVFYAPR	95.0%	47.6	22.3	25	2	0	2	1,310.69
									KAQQELEEQTR	95.0%	79.5	22.5	9	10	0	2	1,359.69
									KESPLLFK	95.0%	42.6	17.6	17	0	0	2	961.57
									KPDTIEVQQMK	95.0%	64.0	23.1	5	0	0	2	1,316.69
									KTANDMIHAENMR	95.0%	61.2	20.1	5	6	0	2	1,546.71
									KTQEQLALEMAELTAR	95.0%	113.0	21.5	18	18	0	2	1,831.96
									KVTAQDVR	95.0%	39.5	22.5	1	0	0	2	916.52
									LFFLQVK	95.0%	42.2	18.9	11	0	0	2	894.55
									LKQIEEQTK	95.0%	31.0	22.3	3	0	0	2	1,116.63
									LNKDQWEER	95.0%	58.2	22.2	8	1	0	2	1,217.59
									NISFNDKK	95.0%	31.2	22.7	2	0	0	2	965.51
									QEAEEAKEALLQASR	95.0%	80.9	22.7	4	4	0	2	1,672.85
									QIEEQTK	95.0%	39.4	23.5	1	0	0	2	875.45
									QLFDQVVK	95.0%	58.8	21.4	19	0	0	2	976.55
									QRIDEFESM	95.0%	54.3	18.1	10	0	0	2	1,170.51
									RALELEQER	95.0%	46.7	22.7	9	0	0	2	1,143.61
									RKPDTIEVQQMK	95.0%	51.6	22.3	12	33	0	2	1,488.78
									SGYLAGDK	95.0%	45.3	23.4	9	0	0	2	810.40
									TAMSTPHVAEPAENEQDEQDENGAEASADLR	95.0%	95.4	14.3	0	21	0	2	3,328.42

									TANDMIHAENMR	95.0%	82.1	19.2	21	3	0	2	1,402.62
									TQEQLALEMAELTAR	95.0%	122.0	22.8	29	9	0	2	1,703.86
									VLEQHK	95.0%	34.7	19.1	2	0	0	2	753.43
									VTAQDVR	95.0%	35.1	24.3	6	0	0	2	788.43
									VTTMDAELEFAIQPNTTGK	95.0%	106.0	22.4	27	0	0	2	2,082.01
Splicing factor 3B subunit 3	SF3B3_HUMAN	SF3B3	135,561	100.00%	7	7	35	7.97%	DYIVVGSDSGR	95.0%	38.6	22.0	1	0	0	2	1,167.56
									FLAVGLVDNTVR	95.0%	64.4	20.3	3	0	0	2	1,303.74
									IVILEYQPSK	95.0%	39.3	19.1	1	0	0	2	1,189.68
									LGAVFNQVAFPLQYTPR	95.0%	74.4	20.2	4	0	0	2	1,921.03
									LPPNTNDEVDEDPTGNK	95.0%	79.4	19.3	6	0	0	2	1,854.84
									TPVEEVPAAIAPFQGR	95.0%	80.3	21.9	11	0	0	2	1,681.89
									TVLDPVTGDLSDTR	95.0%	96.9	22.8	9	0	0	2	1,488.75
Nucleoporin Nup37	NUP37_HUMAN	NUP37	36,689	99.50%	2	2	3	8.28%	VDGIAWSPETR	95.0%	56.4	22.8	2	0	0	2	1,230.61
									VGAVAGNDWLIWDITR	95.0%	42.3	21.7	1	0	0	2	1,785.93
Ig lambda chain C regions	LAC_HUMAN	IGLC1	11,218	100.00%	3	3	9	41.90%	AAPSVTLFPPSSEELQANK	95.0%	47.4	21.4	1	0	0	2	1,986.02
									AGVETTTPSK	95.0%	59.7	23.3	4	0	0	2	990.51
									YAASSYLSLTPEQWK	95.0%	74.6	21.4	4	0	0	2	1,743.86
Protein Daple	DAPLE_HUMAN	CCDC88C	228,201	99.50%	2	2	2	0.84%	KAELEER	95.0%	36.5	24.4	1	0	0	2	874.46
									NADLSDASRK	95.0%	33.3	23.4	1	0	0	2	1,076.53
Lamina-associated polypeptide 2, isoform alpha	LAP2A_HUMAN	TMPO	75,476	100.00%	3	3	8	5.91%	PEFLEDPSVLTK	95.0%	42.9	23.2	2	0	0	1	1,374.72
									SSTPLPTISSSAENTR	95.0%	64.6	23.1	4	0	0	2	1,647.82
									YGVNPGPIVGTTR	95.0%	45.5	22.5	2	0	0	2	1,330.71
Bile salt-activated lipase	CEL_HUMAN	CEL	79,304	100.00%	4	4	16	7.84%	AISQSGVALSPWVIQK	95.0%	71.9	19.2	4	0	0	2	1,683.94
									LGLLGDSVDIFK	95.0%	76.7	20.0	6	0	0	2	1,276.72
									VGCPVGDAAR	95.0%	39.5	21.6	2	0	0	2	1,001.48
									VGPLGFLSTGDANLPGNYGLR	95.0%	83.0	21.6	4	0	0	2	2,118.10
LIM and SH3 domain protein 1	LASP1_HUMAN	LASP1	29,699	100.00%	4	5	18	20.30%	GFSVVADTPELQR	95.0%	64.3	23.4	4	0	0	2	1,418.73
									LKQQSELQSQVR	95.0%	43.2	22.1	2	4	0	2	1,443.79
									MGPSGGEGMEPERR	95.0%	27.3	16.3	0	2	0	2	1,521.64
									QSFTMVADTPENLR	95.0%	81.8	23.2	6	0	0	2	1,608.77
Protocadherin Fat 2	FAT2_HUMAN	FAT2	479,291	100.00%	10	10	33	3.04%	ATDSGQPPLSASVR	95.0%	41.3	22.3	2	0	0	2	1,385.70
									AVAAQDPVIYSLVR	95.0%	87.4	20.3	6	0	0	2	1,501.84
									DVIEINPVTGVVK	95.0%	51.5	16.9	2	0	0	2	1,382.79
									GSVVENSEPGELVATLK	95.0%	60.2	21.8	2	0	0	2	1,728.90
									IDPYLGDISLK	95.0%	33.0	22.0	1	0	0	2	1,233.67
									IILTDENDNPPQFK	95.0%	54.3	23.1	2	0	0	2	1,643.83
									TGVLTVTGPLDYESK	95.0%	62.0	22.3	4	0	0	2	1,579.82
									VPQDTVPGVELLR	95.0%	53.2	19.1	8	0	0	2	1,422.80
									VQAIDPDSR	95.0%	30.9	20.6	1	0	0	2	1,000.51
									VSIEDVNDNPPK	95.0%	59.4	23.0	5	0	0	2	1,326.65
Elongation factor 1-alpha 1	EF1A1_HUMAN	EEF1A1	50,123	100.00%	12	13	134	27.90%	EHALLAYTLGVK	95.0%	59.2	20.5	7	0	0	2	1,314.74
									EVSTYIK	94.6%	30.0	21.6	1	0	0	2	839.45
									IGGIGTVPVGR	95.0%	84.2	16.1	37	0	0	2	1,025.61
									KLEDGPK	94.6%	30.0	19.8	1	0	0	2	786.44
									LPLQDVYK	95.0%	48.0	21.1	11	0	0	2	975.55
									QLIVGVNK	95.0%	45.9	17.1	2	0	0	2	870.54
									QTVAVGVIK	95.0%	33.2	19.4	1	0	0	2	914.57
									STTTGHLIYK	95.0%	38.5	21.3	5	0	0	2	1,120.60
									THINIVVIGHVDSGK	95.0%	48.2	20.0	0	14	0	2	1,588.88
									VETGVLKPGMVVTFAPVNVTTEVK	95.0%	43.5	16.0	0	12	0	2	2,531.38
									YEEIVK	95.0%	30.9	20.6	1	0	0	2	780.41

									YYVTIIDAPGHR	95.0%	64.3	22.8	16	26	0	2	1,404.73
Geranylgeranyl transferase type-2 subunit beta	PGTB2_HUMAN	RABGGTB	36,907	99.50%	2	2	2	6.65%	HADYIASYGSK	94.8%	30.2	20.9	1	0	0	2	1,211.57
									SDAPDTLLLEK	95.0%	38.9	22.3	1	0	0	2	1,201.63
26S proteasome non-ATPase regulatory subunit 6	PSMD6_HUMAN	PSMD6	45,515	100.00%	6	6	10	15.20%	ANEDELK	95.0%	30.4	20.5	2	0	0	2	818.39
									IDKVNEIVETNRPDSK	95.0%	20.4	21.0	0	0	1	2	1,856.97
									IGLFYMDNDLITR	95.0%	70.7	22.3	2	0	0	2	1,586.79
									PLENLEEEGLPK	95.0%	56.0	22.0	2	0	0	1	1,367.71
									RLDEELEDAEK	95.0%	52.3	22.7	2	0	0	2	1,346.64
									VNEIVETNRPDSK	95.0%	27.6	22.9	0	1	0	2	1,500.77
Protein disulfide-isomerase A4	PDIA4_HUMAN	PDIA4	72,916	100.00%	28	32	291	42.20%	DKDPPIPVAK	95.0%	41.0	20.0	4	0	0	2	1,079.61
									DLGLSESGEDVNAAILDESGK	95.0%	114.0	22.2	2	0	0	2	2,119.00
									DLGLSESGEDVNAAILDESGKK	95.0%	123.0	21.5	2	7	0	2	2,247.10
									EFVTAFK	95.0%	33.3	21.5	1	0	0	2	841.45
									EKYGIVDYMIEQSGPPSK	95.0%	42.1	21.8	0	3	0	2	2,041.00
									EVSQPDWTPPPEVTLVLTK	95.0%	57.7	20.3	16	0	0	2	2,136.12
									FAMEPEEFDSDTLR	95.0%	70.8	17.2	18	0	0	2	1,702.73
									FDVSGYPTIK	95.0%	67.2	22.1	27	0	0	2	1,126.58
									FEGGDRDLEHLSK	95.0%	37.6	21.7	2	0	0	2	1,502.72
									FIEEHATK	95.0%	51.4	22.7	4	0	0	2	974.49
									GESDPAYQQYQDAANNLR	95.0%	113.0	19.2	10	0	0	2	2,039.91
									GQAVDYEGSR	95.0%	59.1	20.8	6	0	0	2	1,081.49
									IDATSASVLASR	95.0%	112.0	22.5	24	0	0	2	1,190.64
									KGQAVDYEGSR	95.0%	83.8	22.9	5	7	0	2	1,209.59
									MDATANDVPSDR	95.0%	83.3	17.9	46	0	0	2	1,307.55
									RFDVSGYPTIK	95.0%	44.3	21.7	4	1	0	1	1,282.68
									RSPPIPLAK	95.0%	41.0	9.0	1	0	0	2	978.61
									SHMMDVQGSTQDSAIK	95.0%	41.5	20.8	2	0	0	2	1,766.77
									SHMMDVQGSTQDSAIKDFVLK	95.0%	38.7	21.5	0	5	0	2	2,369.11
									TFDSIVMDPK	95.0%	60.4	22.1	18	0	0	2	1,152.56
									TQEEIVAK	95.0%	40.7	23.6	5	0	0	2	917.49
									VDATAETDLAK	95.0%	67.7	22.9	14	0	0	2	1,133.57
									VDATAETDLAKR	95.0%	72.4	24.2	7	2	0	2	1,289.67
									VEGFPTIYFAPSGDK	95.0%	56.8	22.1	5	0	0	2	1,627.80
									VEGFPTIYFAPSGDKK	95.0%	57.5	22.3	2	0	0	2	1,755.90
									VSQGQLVVMQPEK	95.0%	80.7	22.4	24	0	0	2	1,458.76
									YALPLVGHR	95.0%	51.0	18.1	4	0	0	2	1,025.59
									YGIVDYMIEQSGPPSK	95.0%	98.9	22.3	13	0	0	2	1,783.86
Macrophage-capping protein	CAPG_HUMAN	CAPG	38,500	100.00%	8	8	31	32.80%	AQVEIVTDGEEPAEMIQVLGPKPALK	95.0%	38.5	19.6	0	3	0	2	2,778.46
									DLALAIR	95.0%	48.8	20.0	4	0	0	2	771.47
									EGNPEEDLTADK	95.0%	64.1	19.2	2	0	0	2	1,317.58
									EVQGNESDLFMSYFPR	95.0%	70.8	20.9	5	0	0	2	1,918.86
									MQYAPNTQVEILPQGR	95.0%	76.6	22.6	4	0	0	2	1,844.93
									QAALQVAEGFISR	95.0%	86.5	22.5	6	0	0	2	1,389.75
									VSDATGQMNLTK	95.0%	66.4	22.4	4	0	0	2	1,280.62
									YQEGGVESAFHK	95.0%	60.2	21.6	3	0	0	2	1,351.63
Laminin subunit alpha-5	LAMA5_HUMAN	LAMA5	399,769	100.00%	57	61	662	18.70%	AGALLPAIHEQLR	95.0%	45.1	19.2	6	0	0	2	1,388.80
									AHPASNAIDGTER	95.0%	43.7	22.6	4	0	0	2	1,338.64
									AIEASNAYSR	95.0%	72.8	22.0	31	0	0	2	1,081.53
									AIQVFLLGGSR	95.0%	76.4	18.1	18	0	0	2	1,160.68
									ASPDGLCQVSLQQGR	95.0%	94.6	22.0	5	0	0	2	1,615.79
									AVAAEAQDTATR	95.0%	119.0	23.6	63	0	0	2	1,203.60

AVPLQPPPPLTSASK	95.0%	42.8	18.0	2	0	0	2	1,502.86
CDQCSLGTFSLDAANPK	95.0%	70.9	19.5	2	0	0	2	1,883.83
CNCPPGLSGER	95.0%	35.7	17.0	2	0	0	2	1,246.53
DLADLAAYTALK	95.0%	80.4	21.8	17	0	0	2	1,264.68
DLGAPQAAAEAELAAAQR	95.0%	115.0	22.2	24	0	0	2	1,752.89
DQASQLLAGTEATLGHAK	95.0%	106.0	21.4	12	2	0	2	1,810.93
DTLASVFR	95.0%	49.4	21.9	22	0	0	2	908.48
EAQELNSR	95.0%	53.6	21.8	9	0	0	2	946.46
ECAPGYWGLPEQGCR	95.0%	56.2	18.6	2	0	0	2	1,779.76
ENVQGPK	95.0%	33.3	20.8	2	0	0	2	771.40
FANSPRPDLWVLER	95.0%	30.8	21.2	0	4	0	2	1,699.89
FPKPPQPIILR	95.0%	33.8	10.8	0	4	0	2	1,305.81
FYLQGPEPEPGQGTEDR	95.0%	92.6	20.8	13	0	0	2	1,919.88
GALDQLCGAGGLCR	95.0%	90.9	20.7	11	0	0	2	1,447.68
GAMSVSGR	95.0%	52.3	22.3	2	0	0	2	764.37
GDSCQECAPGFYR	95.0%	34.7	10.4	2	0	0	2	1,546.61
GFGEPFVLNPGTWALR	95.0%	40.1	22.3	1	0	0	2	1,760.91
GPPPELQPQPEGPPR	95.0%	81.6	22.1	29	0	0	2	1,595.82
GQDLGQAVLDAGHSVSTLEK	95.0%	104.0	22.1	11	31	0	2	2,025.03
GQLQLVEGNFR	95.0%	81.8	23.5	22	0	0	2	1,260.67
GQYCDICTAANSNK	95.0%	98.4	16.4	2	0	0	2	1,601.67
GYAQMAPVQPR	95.0%	61.1	22.9	10	0	0	2	1,217.61
HETAQQLEVLEQQSTSLGQDAR	95.0%	36.8	21.4	0	2	0	2	2,468.20
IAASATCGEEAPAR	95.0%	90.3	21.6	4	0	0	2	1,403.66
ILLVTDGAR	95.0%	41.4	20.0	3	0	0	2	957.57
ISFDSQISTTK	95.0%	66.4	22.2	13	0	0	2	1,226.63
LAASLDGAR	95.0%	58.0	24.0	13	0	0	2	873.48
LAQHEAGLMDLR	95.0%	34.7	22.2	0	4	0	2	1,353.70
LEEALQR	94.8%	30.2	22.9	1	0	0	2	858.47
LELEEAATPEGHAVR	95.0%	83.6	22.4	14	25	0	2	1,621.82
LGLVWAALQGAR	95.0%	78.4	17.8	52	0	0	2	1,254.73
LLWEMR	95.0%	38.7	24.3	2	0	0	2	847.45
LTAEQAR	95.0%	44.6	24.3	6	0	0	2	788.43
LVGGPVAGGDPNQTIR	95.0%	78.7	21.4	23	0	0	2	1,550.83
MQTFSPAGSK	95.0%	43.4	22.3	2	0	0	2	1,053.50
QATGDYMGVSLR	95.0%	51.8	22.2	8	0	0	2	1,297.62
QEVGVAVHTPQR	95.0%	32.8	22.4	3	0	0	2	1,320.70
QMIQETK	95.0%	36.2	23.8	3	0	0	2	877.45
STGDPWLTDGSYLDGTGFAR	95.0%	132.0	20.9	10	0	0	2	2,115.96
TLAEVER	95.0%	39.5	24.1	4	0	0	2	817.44
TLLAAIR	95.0%	38.8	21.8	10	0	0	2	757.49
TPLLQR	95.0%	44.8	19.0	13	0	0	2	727.45
TYQPWQFFASSK	95.0%	57.2	22.5	5	0	0	2	1,489.71
VQEQLSSLWEENQALATQTR	95.0%	98.7	21.6	7	1	0	2	2,331.16
VQSQLQAMQENVER	95.0%	110.0	22.7	37	0	0	2	1,659.81
VSLQLLR	95.0%	45.7	21.8	14	0	0	2	828.53
VSSYGGTLR	95.0%	46.4	21.1	11	0	0	2	939.49
WQGQYEGLR	95.0%	38.7	22.6	4	0	0	2	1,136.55
WWQSPPLSR	94.9%	30.3	21.9	1	0	0	2	1,156.59
YALVVEYANEDAR	95.0%	36.7	22.3	1	0	0	2	1,512.73
YELHSETQR	95.0%	34.4	21.9	0	1	0	2	1,162.55

Metalloproteinase inhibitor 1	TIMP1_HUMAN	TIMP1	23,153	100.00%	5	7	431	32.40%	EPGLCTWQSLR	95.0%	38.2	22.8	6	0	0	2	1,346.65
									FVYTPAMESVCGYFHR	95.0%	97.1	20.1	18	1	0	2	1,963.88
									GFQALGDAADIR	95.0%	106.0	23.5	259	0	0	2	1,233.62
									LQSGTHCLWTDQLLQGSEK	95.0%	133.0	21.9	9	38	0	2	2,201.07
									SEEFLIAGK	95.0%	65.6	21.7	100	0	0	2	993.53
D-tyrosyl-tRNA(Tyr) deacylase 1	DTD1_HUMAN	DTD1	23,406	100.00%	3	3	6	14.80%	ASVTVGGEQISAIGR	95.0%	36.6	21.9	1	0	0	2	1,444.78
									SASSGAEGDVSSER	95.0%	36.7	18.8	1	0	0	2	1,338.58
									SASSGAEGDVSSEREP	95.0%	53.7	17.9	4	0	0	2	1,564.67
Fatty acid-binding protein, epidermal	FABP5_HUMAN	FABP5	15,146	100.00%	3	3	10	23.70%	ELGVGIALR	95.0%	37.2	17.1	2	0	0	2	927.56
									FEETTADGR	95.0%	52.5	19.8	4	0	0	2	1,025.45
									LVVECVMNNVTCTR	95.0%	71.1	22.3	4	0	0	2	1,694.80
Lysyl-tRNA synthetase	SYK_HUMAN	KARS	68,032	100.00%	8	8	13	11.70%	INMVEELEK	95.0%	32.4	24.2	1	0	0	2	1,104.56
									LIFYDLR	95.0%	38.7	19.0	1	0	0	2	939.53
									LPETNLFETEETR	95.0%	72.5	22.6	2	0	0	2	1,578.77
									LPETNLFETEETRK	95.0%	44.3	23.1	0	2	0	2	1,706.86
									LQVMANSR	95.0%	35.8	23.3	1	0	0	2	934.48
									MLVVGGIDR	95.0%	31.4	23.7	2	0	0	2	975.53
									VAMFLTDSNNIK	95.0%	40.9	23.3	2	0	0	2	1,368.68
									YLDLILNDFVR	95.0%	45.6	20.8	2	0	0	2	1,380.75
Pre-mRNA-splicing factor SPF27	SPF27_HUMAN	BCAS2	26,113	99.50%	2	2	6	10.70%	TIVQLENEIYQIK	95.0%	56.3	20.3	2	0	0	2	1,590.87
									YELPAPSSGQK	95.0%	29.5	22.7	0	4	0	2	1,176.59
Ezrin	EZRI_HUMAN	EZR	69,397	100.00%	29	36	330	53.90%	AKEAQDDLVK	95.0%	57.1	24.0	5	0	0	2	1,116.59
									AKEELER	95.0%	40.5	24.4	4	0	0	2	874.46
									ALQLEEER	95.0%	45.8	21.6	17	0	0	2	987.51
									APDFVFYAPR	95.0%	80.6	21.7	84	0	0	2	1,182.59
									AQEEAERLEADR	95.0%	42.2	21.3	12	6	0	2	1,416.67
									DNAMLEYLK	95.0%	47.8	22.9	15	0	0	2	1,096.54
									EAQDDLVK	95.0%	42.5	22.2	1	0	0	2	917.46
									EKEELMLR	95.0%	32.8	23.9	1	0	0	2	1,063.55
									ELSEQIQR	95.0%	49.8	22.9	10	0	0	2	1,002.52
									ENPLQFK	95.0%	35.5	24.5	1	0	0	2	875.46
									FVIKPIDK	95.0%	41.1	17.2	6	0	0	2	959.59
									FYPEDVAEELIQDITQK	95.0%	82.5	22.0	5	5	0	2	2,038.00
									GFPTWLK	95.0%	30.7	23.9	1	0	0	2	848.47
									GMLKDNAMLEYLK	95.0%	65.8	22.3	5	4	0	2	1,525.78
									IALLEEAR	95.0%	56.8	18.6	34	0	0	2	914.53
									IAQDLEMYGINYFEIK	95.0%	79.9	22.0	25	0	0	2	1,962.95
									IGFPWSEIR	95.0%	68.8	23.4	121	0	0	2	1,104.58
									ILQLCMGNHELYMR	95.0%	45.8	21.6	2	1	0	2	1,809.85
									IQVWHAEHR	95.0%	33.2	23.1	1	0	0	2	1,175.61
									ITEAEKNER	95.0%	51.7	23.5	3	2	0	2	1,089.55
									KAPDFVFYAPR	95.0%	47.6	22.3	25	2	0	2	1,310.69
									KENPLQFK	95.0%	31.1	21.9	1	0	0	2	1,003.56
									KPDTIEVQQMK	95.0%	64.0	23.1	5	0	0	2	1,316.69
									KVSAQEVR	95.0%	51.1	22.5	4	0	0	2	916.52
									LFFLQVK	95.0%	42.2	18.9	11	0	0	2	894.55
									NISFNDKK	95.0%	31.2	22.7	2	0	0	2	965.51
									QAVDQIK	95.0%	48.6	21.7	7	0	0	2	801.45
									QLFDQVVK	95.0%	58.8	21.4	19	0	0	2	976.55
									QLLTLSSELSQAR	95.0%	113.0	21.4	45	0	0	2	1,445.80
									QQLETEK	95.0%	40.8	23.5	6	0	0	2	875.45

									QQLETEKK	95.0%	33.7	22.9	1	0	0	2	1,003.54
									QRIDEFEAL	95.0%	43.5	23.6	5	0	0	2	1,120.56
									RKPDTIEVQQMK	95.0%	51.6	22.3	12	33	0	2	1,488.78
									SGYLSSER	95.0%	61.3	20.5	17	0	0	2	898.43
									SQEQLAAELAEYTAK	95.0%	125.0	22.5	68	5	0	2	1,651.82
									THNDIIHNENMR	95.0%	55.5	21.5	3	2	0	2	1,509.69
									VSAQEVR	95.0%	40.3	24.7	4	0	0	2	788.43
									VSAQEVRK	95.0%	36.8	22.5	2	0	0	2	916.52
									VTTMDAELEFAIQPNTTGK	95.0%	106.0	22.4	27	0	0	2	2,082.01
Thioredoxin-dependent peroxide reductase, mitochondrial	PRDX3_HUMAN	PRDX3	27,675	100.00%	4	4	22	18.40%	DYGVLLEGSGLALR	95.0%	103.0	22.0	10	0	0	2	1,462.79
									GLFIIDPNGVIK	95.0%	58.5	18.3	8	0	0	2	1,285.75
									GTAVVNGEFK	95.0%	35.2	22.6	1	0	0	2	1,021.53
									HLSVNDLPVGR	95.0%	55.4	21.9	3	0	0	2	1,206.66
Latent-transforming growth factor beta-binding protein 3	LTBP3_HUMAN	LTBP3	139,337	100.00%	5	6	18	6.52%	CTCAQGYAPAPDGR	95.0%	59.6	15.2	2	0	0	2	1,523.64
									FCQVPAGGAGGGTGGSGPGLSR	95.0%	106.0	21.6	1	1	0	2	1,946.91
									GAGGGGALAR	95.0%	55.8	19.0	2	0	0	2	786.42
									GYTQDNNIVNYGIPAHR	95.0%	32.0	22.8	0	3	0	2	1,931.94
									TGALSTGALPPLAPEGDSVASK	95.0%	65.9	21.2	9	0	0	2	2,039.07
Annexin A1	ANXA1_HUMAN	ANXA1	38,698	100.00%	18	25	353	58.10%	AAYLQETGKPLDETLK	95.0%	65.7	21.4	2	1	0	2	1,776.94
									AAYLQETGKPLDETLKK	95.0%	68.1	18.2	4	9	0	2	1,905.03
									ALYEAGER	95.0%	55.8	22.5	8	0	0	2	908.45
									CATSKPAFFAEK	95.0%	48.4	21.0	4	2	0	2	1,356.66
									DITSDTSGDFR	95.0%	82.2	19.3	21	0	0	2	1,213.53
									GDRSEDFGVNEDLADSDAR	95.0%	95.4	17.8	2	9	0	2	2,067.89
									GGPGSAVSPYPTFNPSSDVAALHK	95.0%	67.4	22.1	3	29	0	2	2,356.16
									GLGTDEDTLIEILASR	95.0%	122.0	21.8	54	2	0	2	1,702.89
									GTDVNVFNTILTTR	95.0%	102.0	21.8	40	0	0	2	1,550.82
									GVDEATIIDILTK	95.0%	98.1	20.9	69	0	0	2	1,387.77
									GVDEATIIDILTKR	95.0%	52.0	19.1	3	0	0	2	1,543.87
									KGTDVNVFNTILTTR	95.0%	114.0	20.3	9	3	0	2	1,678.91
									MYGISLCQAILDETKGDYEK	95.0%	32.8	20.6	0	1	0	2	2,350.10
									NALLSLAK	95.0%	40.7	20.9	3	0	0	2	829.51
									QAWFIENEEQEYVQTVK	95.0%	98.3	22.2	25	0	0	2	2,141.02
									SEDFGVNEDLADSDAR	95.0%	122.0	18.0	9	0	0	2	1,739.74
									SEIDMNDIK	95.0%	48.6	21.3	11	0	0	2	1,064.49
									TPAQFDADELR	95.0%	88.1	22.1	30	0	0	2	1,262.60
Bifunctional purine biosynthesis protein PURH	PUR9_HUMAN	ATIC	64,599	100.00%	6	6	10	16.40%	AEISNAIDQYVTGTIGEDEDLIK	95.0%	80.7	21.9	1	0	0	2	2,494.22
									ALFEEVPELLTEAEKK	95.0%	40.5	20.9	2	0	0	2	1,845.99
									APGQLALFSVSDK	95.0%	72.3	22.3	2	0	0	1	1,332.72
									DVSELTGFPEMLGGR	95.0%	36.8	21.8	2	0	0	2	1,623.77
									EVSDGIIAPGYEEEALTILSK	95.0%	90.1	20.9	2	0	0	2	2,234.14
									SLFSNVVTK	95.0%	31.0	19.5	1	0	0	2	994.56
Reticulocalbin-1	RCN1_HUMAN	RCN1	38,873	100.00%	4	4	11	12.10%	AADLNGDLTATR	95.0%	81.2	23.0	2	0	0	2	1,217.61
									IDNDGDGFVTTEELK	95.0%	114.0	22.6	4	0	0	2	1,652.77
									TFDQLTPDESK	95.0%	34.6	22.1	1	0	0	2	1,280.60
									TFDQLTPDESKER	95.0%	57.9	22.7	4	0	0	2	1,565.75
Caveolin-1	CAV1_HUMAN	CAV1	20,454	100.00%	2	2	2	19.70%	IDFEDVIAEPEGTHSFDGIWK	95.0%	62.7	21.4	1	0	0	2	2,405.13
									YVDSEGHLYTVPIR	95.0%	30.5	23.5	0	1	0	2	1,648.83
Nucleoside diphosphate kinase B	NDKB_HUMAN	NME2	17,280	100.00%	11	14	74	73.70%	ASEEHLK	95.0%	48.6	20.3	5	0	0	2	813.41
									DRPFFPGLVK	95.0%	38.5	20.6	3	0	0	2	1,175.66
									EISLWFKPEELVDYK	95.0%	44.1	21.1	3	0	0	2	1,895.98

									GDFCIQVGR	95.0%	51.1	22.8	4	0	0	2	1,051.50
									GLVGEIIK	95.0%	36.3	16.7	2	0	0	2	828.52
									GLVGEIIKR	95.0%	52.3	16.7	5	0	0	2	984.62
									NIIHGSDSVK	95.0%	40.6	21.7	1	0	0	2	1,069.56
									SAEKEISLWFKPEELVDYK	95.0%	31.4	20.7	0	2	4	2	2,311.19
									TFIAIKPDGVQR	95.0%	34.9	19.8	2	0	0	2	1,344.76
									VMLGETNPADSKPGTIR	95.0%	88.7	22.3	4	8	0	2	1,785.92
									YMNSGPVVAMVWEGLNVVK	95.0%	118.0	22.3	19	12	0	2	2,125.05
28 kDa heat- and acid-stable phosphoprotein	HAP28_HUMAN	PDAP1	20,613	100.00%	4	4	10	22.10%	GVEGLIDIENPNR	95.0%	72.9	22.6	2	0	0	2	1,425.73
									KVTQLDLDGPK	95.0%	49.5	21.3	2	0	0	2	1,213.68
									QYTSPEEIDAQLQAEK	95.0%	98.7	22.0	4	0	0	2	1,849.88
									VTQLDLDGPK	95.0%	41.8	21.8	2	0	0	2	1,085.58
Septin-11	SEP11_HUMAN	SEPT11	49,381	100.00%	4	5	7	14.70%	AAAQLLQSQAQQSGAQQTK	95.0%	135.0	21.5	2	2	0	2	1,957.01
									FESDPATHNEPGVR	95.0%	65.3	21.7	1	0	0	2	1,555.71
									STLMDTLFNTK	95.0%	35.8	22.0	1	0	0	2	1,286.63
									SYELQESNVR	95.0%	44.9	21.0	1	0	0	2	1,224.59
									VNIIPIIAK	95.0%	39.9	9.5	6	0	0	2	980.65
Protein S100-A11	S10AB_HUMAN	S100A11	11,723	100.00%	7	8	100	56.20%	CIESLIAVFQK	95.0%	70.9	21.2	6	0	0	2	1,307.70
									DGYNYTLSK	95.0%	54.8	20.7	6	0	0	2	1,060.50
									DPGVLDR	95.0%	45.5	20.5	2	0	0	2	771.40
									ISSPTETER	95.0%	34.5	22.4	2	0	0	2	1,019.50
									NQKDPGVLDR	95.0%	39.7	21.6	0	2	0	2	1,141.60
									TEFLSFMNTELAAFTK	95.0%	104.0	22.4	79	1	0	2	1,849.90
									YAGKDGYNYTLSK	95.0%	51.9	22.5	2	0	0	2	1,479.71
Carbonyl reductase [NADPH] 1	CBR1_HUMAN	CBR1	30,357	100.00%	10	11	70	53.80%	DVCTELLPLIKPQGR	95.0%	62.0	19.1	1	0	0	2	1,738.95
									EGWPSSAYGVTK	95.0%	58.1	21.4	5	0	0	2	1,281.61
									EYGGLDVLVNNAGIAFK	95.0%	74.9	21.3	6	0	0	2	1,779.93
									FHQLDIDDLQSIR	95.0%	40.5	21.8	0	10	0	2	1,599.81
									GIGLAIVR	95.0%	48.8	14.8	5	0	0	2	798.52
									GQAAVQQLQAEGLSPR	95.0%	96.8	21.8	8	4	0	2	1,652.87
									ILLNACCPGWVR	95.0%	58.2	22.9	4	0	0	2	1,458.74
									SETITEEELVGLMNK	95.0%	106.0	22.0	9	0	0	2	1,692.84
									SPEEGAETPVYLALLPPDAEGPHGQFVSEK	95.0%	84.2	20.3	0	10	0	2	3,164.54
									VVNVSSIMSVR	95.0%	87.9	22.4	8	0	0	2	1,206.65
Lysosomal Pro-X carboxypeptidase	PCP_HUMAN	PRCP	55,783	99.50%	2	2	7	5.24%	NALDPMSVLLAR	95.0%	58.2	21.1	5	0	0	2	1,299.71
									YYGESLPFGDNSFK	95.0%	52.9	20.8	2	0	0	2	1,623.73
Ras-related protein R-Ras	RRAS_HUMAN	RRAS	23,463	100.00%	3	3	7	16.10%	ADLESQR	95.0%	38.6	23.3	1	0	0	2	818.40
									LDILDTAGQEEFGAMR	95.0%	79.5	22.1	3	0	0	2	1,765.84
									LNVDEAFEQLVR	95.0%	114.0	23.0	3	0	0	2	1,432.74
Heat shock protein HSP 90-beta	HS90B_HUMAN	HSP90AB1	83,249	100.00%	38	49	810	46.00%	ADHGEPIGR	95.0%	35.9	22.0	3	0	0	2	951.47
									ADLINNLGTIAK	95.0%	74.8	20.9	86	0	0	2	1,242.71
									ALLFIPR	95.0%	51.5	17.1	11	0	0	2	829.53
									APFDLFENK	95.0%	37.6	22.7	4	0	0	2	1,080.54
									DNSTMGYMMAK	95.0%	55.5	11.8	6	0	0	2	1,280.50
									EDQTEYLEER	95.0%	49.1	18.8	12	0	0	2	1,311.57
									EGLELPEDEEEK	95.0%	58.8	20.1	3	0	0	2	1,416.64
									EGLELPEDEEEKK	95.0%	43.1	20.8	2	0	0	2	1,544.73
									EKYIDQEELNK	95.0%	55.1	23.3	4	0	0	2	1,408.70
									ELISNASDALDK	95.0%	92.5	23.1	15	0	0	2	1,275.64
									ELISNASDALDKIR	95.0%	108.0	21.4	10	2	0	2	1,544.83
									ELKIDIIPNPQER	95.0%	37.3	18.5	2	0	0	2	1,564.87

									EMLQQSK	95.0%	50.8	23.4	6	0	0	2	879.42
									EQVANSAFVER	95.0%	76.7	23.5	18	0	0	2	1,249.62
									GVVDSEDLPLNISR	95.0%	113.0	22.4	54	0	0	2	1,513.79
									HFSVEGQLEFR	95.0%	64.6	23.7	7	4	0	2	1,348.67
									HLEINPDHPIVETLR	95.0%	69.1	21.0	2	8	1	2	1,782.95
									HNDDEQYAWESSAGGSFTVR	95.0%	133.0	17.3	7	54	0	2	2,255.96
									HSQFIGYPITLYLEK	95.0%	73.6	21.8	5	9	0	2	1,808.96
									IDIIPNPQER	95.0%	57.1	21.4	13	0	0	2	1,194.65
									IEDVGSDEEDDSGK	95.0%	62.6	15.1	1	0	0	2	1,494.61
									IEDVGSDEEDDSGKDK	95.0%	32.8	17.3	0	2	0	2	1,737.73
									IRYESLTDPSK	95.0%	47.5	22.3	2	0	0	2	1,308.68
									KHLEINPDHPIVETLR	95.0%	30.3	18.4	0	0	11	2	1,911.05
									LGIHEDSTNR	95.0%	53.3	22.5	9	13	0	2	1,141.56
									LSELLR	95.0%	44.8	23.2	17	0	0	2	730.45
									NPDDITQEEYGEFYK	95.0%	91.9	17.8	21	0	0	2	1,847.80
									RAPFDLFENK	95.0%	49.7	22.6	6	0	0	2	1,236.64
									SIYYITGESK	95.0%	54.9	23.7	6	0	0	2	1,160.58
									SLTNDWEDHLAVK	95.0%	83.6	21.8	97	7	0	2	1,527.74
									SLVSVTK	95.0%	43.2	18.8	2	0	0	2	733.45
									TLTLVDTGIGMTK	95.0%	109.0	22.1	57	0	0	2	1,365.73
									VILHLK	95.0%	35.5	7.8	7	0	0	2	722.49
									VILHLKEDQTEYLEER	95.0%	69.3	22.0	7	102	0	2	2,015.05
									YESLTDPSK	95.0%	43.0	23.0	9	0	0	2	1,039.49
									YESLTDPSKLDSGK	95.0%	67.9	22.6	14	3	0	2	1,539.75
									YHTSQSGDEMTSLSEYVSR	95.0%	75.7	17.9	3	45	0	2	2,192.94
									YIDQEELNK	95.0%	60.6	23.1	21	0	0	2	1,151.56
Polypeptide N-acetylgalactosaminyltransferase 12	GLT12_HUMAN	GALNT12	66,921	100.00%	3	3	4	5.16%	ESSDSFVPLLR	95.0%	33.7	23.1	1	0	0	2	1,249.64
									LANELSGLPK	95.0%	35.1	17.5	1	0	0	2	1,041.59
									LQLQGEELR	95.0%	45.2	21.6	2	0	0	2	1,085.60
60 kDa heat shock protein, mitochondrial	CH60_HUMAN	HSPD1	61,038	100.00%	11	14	57	25.10%	ALMLQGVDLLADAVAVTMGPK	95.0%	73.6	20.9	2	14	0	2	2,145.13
									APGFGDNRK	95.0%	34.4	24.4	1	0	0	2	961.49
									GYISPYFINTSK	95.0%	68.3	23.4	4	0	0	2	1,389.71
									ISSIQSIVPALEIANAHR	95.0%	60.0	17.7	2	4	0	2	1,919.07
									KISSIQSIVPALEIANAHR	95.0%	26.8	14.6	0	1	0	2	2,047.17
									LSDGVAVLK	95.0%	48.1	20.6	1	0	0	2	901.54
									LVQDVANNTNEEAGDGTTTATVLAR	95.0%	139.0	21.8	4	3	0	2	2,560.25
									TLNDELEIIEGMK	95.0%	72.3	23.3	8	0	0	2	1,504.76
									TVIIEQSWGSPK	95.0%	69.6	22.1	4	0	0	2	1,344.72
									VGEVIVTKDDAMLLK	95.0%	31.0	20.2	0	1	0	2	1,646.90
									VTDALNATR	95.0%	58.2	24.0	8	0	0	2	960.51
High mobility group protein B1	HMGB1_HUMAN	HMGB1	24,877	100.00%	9	16	121	40.50%	FKDPNAPK	95.0%	42.0	23.5	9	0	0	2	916.49
									GEHPGLSIGDVAK	95.0%	45.4	22.8	2	0	0	2	1,279.66
									GEHPGLSIGDVAKK	95.0%	47.6	21.1	0	1	1	2	1,407.76
									HPDASVNFSEFSK	95.0%	52.8	21.1	3	2	0	2	1,464.68
									IKGEHPGLSIGDVAK	95.0%	62.7	19.7	14	30	15	2	1,520.84
									KHPDASVNFSEFSK	95.0%	40.2	23.1	2	15	0	2	1,592.77
									LGEMWNNTAADDKQPYEK	95.0%	76.8	20.8	7	15	0	2	2,109.96
									MSSYAFFVQTCR	95.0%	104.0	19.7	19	0	0	2	1,512.66
									TYIPPK	95.0%	39.9	22.7	1	0	0	2	718.41
									TYIPPKGETK	95.0%	35.2	21.6	2	2	0	2	1,133.62
									YEKDIAAYR	95.0%	50.4	22.0	5	2	0	2	1,128.57

Prostaglandin reductase 1	PTGR1_HUMAN	PTGR1	35,853	100.00%	6	7	14	26.40%	EYIIEGFENMPAAFMGMLK	95.0%	58.6	21.0	2	0	0	2	2,239.01
									HFVGYPTNSDFELK	95.0%	41.9	22.0	2	0	0	2	1,653.79
									LKEGDTMMGQQVAK	95.0%	39.0	22.2	2	2	0	2	1,567.75
									TGPLPPGPPPEIVIYQELR	95.0%	59.2	18.1	4	0	0	2	2,073.14
									TVESLEETLKK	95.0%	40.7	21.7	0	1	0	2	1,276.70
									VVGAVGSDEK	95.0%	40.1	23.7	1	0	0	2	960.50
Ras GTPase-activating-like protein IQGAP1	IQGA1_HUMAN	IQGAP1	189,241	100.00%	30	32	145	24.60%	ALESGDVNTVWK	95.0%	75.9	23.1	6	0	0	2	1,318.66
									ALQSPALGLR	95.0%	54.2	16.1	3	0	0	2	1,025.61
									ATFYGEQVDYYK	95.0%	68.1	20.0	4	0	0	2	1,483.67
									EEIQSSISGVTAAYNR	95.0%	100.0	22.1	3	0	0	2	1,724.85
									EQLWLANEGLITR	95.0%	94.1	21.2	6	0	0	2	1,542.83
									FLSAIVSSVDK	95.0%	42.3	20.8	1	0	0	2	1,165.65
									FQPGETLTEILETPATSEQEAEHQR	95.0%	51.2	20.3	0	9	0	2	2,841.35
									GLQQQNSDWYLK	95.0%	31.2	22.8	1	0	0	2	1,479.72
									IFYPETTDIYDR	95.0%	50.3	22.4	2	0	0	2	1,532.73
									IFYPETTDIYDRK	95.0%	27.3	22.9	0	1	0	2	1,660.82
									IIGNLLYYR	95.0%	61.9	20.0	4	0	0	2	1,124.65
									ILAIGLINEALDEGDAQK	95.0%	118.0	19.9	16	9	0	2	1,883.01
									ITLQDVVSHSK	95.0%	38.2	21.4	0	2	0	2	1,226.67
									LAAVALINAAIQK	95.0%	76.0	13.2	4	0	0	2	1,295.81
									LEGVLAEVAQHYQDTLIR	95.0%	78.1	19.6	4	3	0	2	2,055.09
									LGLAPQIQDLYGK	95.0%	47.5	20.3	4	0	0	2	1,415.79
									LGNFFSPK	95.0%	44.0	21.8	5	0	0	2	909.48
									LPYDVTPEQALAHEEVK	95.0%	54.1	21.5	0	6	0	2	1,938.98
									LQQTYAALNSK	95.0%	51.7	22.4	4	0	0	2	1,236.66
									LTAEEMDER	95.0%	70.4	18.8	5	0	0	2	1,109.48
									NPNAMLVNLEEPLASTYQDILYQAK	95.0%	44.6	20.6	0	4	0	2	2,851.42
									QSGQTDPLQKEELQSGVDAANSAAQQYQR	95.0%	97.1	20.6	0	4	0	2	3,147.49
									SNQQLENDLNLMDIK	95.0%	87.4	22.6	2	0	0	2	1,790.86
									SWVNQMESQTGEASK	95.0%	109.0	18.9	2	0	0	2	1,697.74
									TLINAEDPPMVVVR	95.0%	61.0	20.7	8	0	0	2	1,553.84
									TLQALQIPAAK	95.0%	72.2	15.3	6	0	0	2	1,153.69
									VDFTEEEINNMK	95.0%	59.5	20.0	6	0	0	2	1,468.66
									VDQIQEIVTGNPTVIK	95.0%	78.1	18.7	4	0	0	2	1,753.97
									YGIQMPAFSK	95.0%	47.8	21.8	4	0	0	2	1,141.57
									YQELINDIAR	95.0%	36.5	23.7	3	0	0	2	1,234.64
Glutathione S-transferase P	GSTP1_HUMAN	GSTP1	23,339	100.00%	15	19	715	70.00%	AFLASPEYVNLPINGNGK	95.0%	90.3	21.6	4	4	0	2	1,903.99
									AFLASPEYVNLPINGNGKQ	95.0%	86.5	21.6	3	0	0	2	2,032.05
									ALPGQLKPFETLLSQNQGGK	95.0%	84.9	18.5	29	73	0	2	2,126.16
									ASCLYGQLPK	95.0%	57.3	22.5	10	0	0	2	1,136.58
									DQQEAALVDMVNDGVEDLR	95.0%	129.0	21.8	78	9	0	2	2,116.98
									EEVVTVETWQEGSLK	95.0%	102.0	23.2	81	0	0	2	1,733.86
									FQDGDLTLYQSNTILR	95.0%	141.0	21.8	136	0	0	2	1,883.95
									MLLADQGQSWK	95.0%	67.4	23.1	14	0	0	2	1,276.64
									MLLADQGQSWKEEVVTVETWQEGSLK	95.0%	50.8	21.6	0	4	0	2	3,007.47
									MPPYTVVYFPVR	95.0%	76.9	22.1	4	0	0	2	1,484.76
									PPYTVVYFPVR	95.0%	73.2	21.2	119	0	0	1	1,337.73
									TLGLYGK	95.0%	41.0	21.1	27	0	0	2	751.44
									TLGLYGKDQQEAALVDMVNDGVEDLR	95.0%	75.3	21.3	0	22	0	2	2,849.40
									YISLIYTNYEAGK	95.0%	55.5	22.7	4	0	0	2	1,534.78
									YISLIYTNYEAGKDDYVK	95.0%	121.0	22.1	84	10	0	2	2,155.06

Fibronectin	FINC_HUMAN	FN1	262,581	100.00%	5	5	18	3.31%	ITYGETGGNSPVQEFTVPGSK	95.0%	58.0	21.7	2	0	0	2	2,168.05
									LGVRPSQGGEAPR	95.0%	44.8	21.1	0	8	0	2	1,323.71
									NLQPASEYTVSLVAIK	95.0%	49.4	20.4	1	0	0	2	1,732.95
									SYTITGLQPGTDYK	95.0%	45.4	21.8	2	0	0	2	1,543.76
									VPGTSTSATLTGLTR	95.0%	80.8	21.6	5	0	0	2	1,461.79
14-3-3 protein zeta/delta	1433Z_HUMAN	YWHAZ	27,728	100.00%	21	27	754	73.90%	DICNDVLSLLEK	95.0%	82.6	23.7	60	0	0	2	1,418.72
									DNLTLWTSDTQGDEAEAGEGGEN	95.0%	129.0	13.8	14	0	0	2	2,409.00
									DSTLIMQLLR	95.0%	79.3	21.3	113	0	0	2	1,189.66
									EKIETELR	95.0%	47.8	22.3	5	0	0	2	1,017.56
									EMQPTHPIR	95.0%	67.1	22.4	13	0	0	2	1,108.56
									FLIPNASQAESK	95.0%	62.7	23.7	21	0	0	2	1,304.69
									GIVDQSQQAYQEAFEISK	95.0%	134.0	21.8	169	8	0	2	2,040.99
									GIVDQSQQAYQEAFEISKK	95.0%	74.9	22.5	6	7	0	2	2,169.08
									KEMQPTHPIR	95.0%	43.9	22.5	0	11	0	2	1,252.65
									KGIVDQSQQAYQEAFEISK	95.0%	115.0	22.5	3	4	0	2	2,169.08
									LAEQAER	95.0%	59.4	21.6	36	0	0	2	816.42
									LAEQAERYDDMAACMK	95.0%	50.3	16.1	0	2	0	2	1,933.81
									MKGDYYR	95.0%	42.7	19.2	6	0	0	2	932.43
									NELVQK	95.0%	31.1	22.9	2	0	0	2	730.41
									NLLSVAYK	95.0%	51.4	19.1	49	0	0	2	907.53
									NLLSVAYKNVVGAR	95.0%	79.1	17.7	4	2	0	2	1,503.86
									SVTEQGAELSNEER	95.0%	112.0	20.7	64	0	0	2	1,548.71
									TAFDEAIAELDTLSEESYK	95.0%	114.0	20.6	34	10	0	2	2,131.99
									YDDMAACMK	95.0%	59.2	10.0	21	0	0	2	1,104.42
									YLAEVAAGDDK	95.0%	47.0	22.5	8	0	0	2	1,151.56
									YLAEVAAGDDKK	95.0%	79.5	22.7	53	29	0	2	1,279.65
Agrin	AGRIN_HUMAN	AGRN	214,820	100.00%	70	86	1629	41.60%	AAAVSSGFDGAIQLVSLGGR	95.0%	106.0	21.1	109	0	0	2	1,875.99
									AIVDVHFDPTTAFR	95.0%	56.0	22.2	5	1	0	2	1,588.81
									ALEPQGLLLYNGNAR	95.0%	36.1	21.0	0	2	0	2	1,628.88
									ALGPAGCEADASAPATCAEMR	95.0%	119.0	17.2	7	4	0	2	2,105.91
									ALQSNHFELSLR	95.0%	66.7	22.1	6	6	0	2	1,414.74
									ASCYNSALGCCSDGK	95.0%	109.0	10.4	4	0	0	2	1,649.64
									AYGTGFVGCLR	95.0%	64.2	21.2	16	0	0	2	1,200.58
									AYHTLR	95.0%	37.0	24.2	6	0	0	2	760.41
									CEFGAR	95.0%	35.5	18.8	1	0	0	2	739.32
									CEHPPPGPVCGSDGVTYGSACELR	95.0%	38.6	16.2	0	1	0	2	2,602.11
									DDCEQMTGLCSCKPGVAGPK	95.0%	41.4	14.5	0	4	0	2	2,225.93
									DFLALALLDGR	95.0%	49.9	21.5	3	0	0	2	1,203.67
									DQCPEPCR	95.0%	35.0	11.5	4	0	0	2	1,061.41
									EAACLQQTQIEEAR	95.0%	105.0	21.8	11	0	0	2	1,646.78
									EPLYVGGAPDFSK	95.0%	63.3	22.7	16	0	0	2	1,379.68
									EPVTLGAWTR	94.6%	30.0	23.4	1	0	0	2	1,129.60
									ESLLDGGNK	95.0%	38.5	23.5	1	0	0	2	932.47
									FDGPCDPCQGALPDPSR	95.0%	50.9	16.6	2	0	0	2	1,888.80
									FDTGSGPAVLTSAVPVEPGQWHR	95.0%	93.8	20.8	5	101	0	2	2,408.20
									FGALCEAETGR	95.0%	87.4	20.8	35	0	0	2	1,210.55
									FHCQCPPGR	95.0%	29.5	18.0	0	2	0	2	1,158.49
									FNAVCLSR	95.0%	72.7	23.2	32	0	0	2	966.48
									GAPEGTVCGSDGADYPGECQLLR	95.0%	123.0	16.3	10	3	0	2	2,409.05
									GDFVSLALR	95.0%	62.2	21.4	24	0	0	2	977.54
									GIVTDGR	95.0%	33.1	24.0	2	0	0	2	717.39

									GKDFLALALLDGR	95.0%	103.0	19.9	32	0	0	2	1,388.79
									GLYVAAQGACR	95.0%	69.6	23.5	22	0	0	2	1,165.58
									GMLCGFGAVCEPNAEGPGR	95.0%	94.2	17.4	15	0	0	2	1,994.85
									GPSGLLLYNGQK	95.0%	57.2	22.0	8	0	0	2	1,246.68
									GSGVGECGDHPCLPNPCHGGAPCQNLEAGR	95.0%	35.9	14.5	0	2	0	2	3,160.32
									HQGPCDQAPSPCLGVQCAFGATCAVK	95.0%	55.0	18.8	0	4	0	2	2,816.24
									IFFVNPAPPYLWPAHK	95.0%	29.5	20.1	0	7	0	2	1,897.02
									KFDGPCDPCQGALPDPSR	95.0%	34.3	19.3	0	1	0	2	2,016.89
									KSPCPSVVAPVCGSDASTYSNECELQR	95.0%	57.7	18.6	0	2	0	2	2,998.33
									LALEFR	95.0%	40.6	21.6	24	0	0	2	748.44
									LELGIGPGAATR	95.0%	79.9	20.2	94	0	0	2	1,154.65
									LLDVNNQR	95.0%	57.4	20.9	54	0	0	2	971.53
									LRDLGPGK	95.0%	50.7	18.1	17	0	0	2	855.51
									MALEVVFLAR	95.0%	62.6	20.4	16	0	0	2	1,164.65
									QAPVCGDDGVTYENDCVMGR	95.0%	119.0	14.8	4	2	0	2	2,242.92
									QENVFK	95.0%	33.2	24.0	1	0	0	2	764.39
									QIQVSR	95.0%	31.6	24.0	2	0	0	2	730.42
									QLLTPEHVLR	95.0%	43.5	15.2	8	2	0	2	1,205.70
									QVDVTSFAGHPCTR	95.0%	69.8	21.1	16	17	0	2	1,574.74
									RLEFR	95.0%	33.2	21.3	18	0	0	2	720.42
									SADGLTASCLCPATCR	95.0%	89.9	18.9	19	0	0	2	1,739.75
									SAGDVDTLAFDGR	95.0%	115.0	21.5	116	0	0	2	1,323.62
									SELFGETAR	95.0%	57.2	22.2	25	0	0	2	1,009.50
									SFLAFPTLR	95.0%	58.0	20.3	95	0	0	2	1,051.59
									SGCTPCSCDPQGAVR	95.0%	82.9	13.6	4	0	0	2	1,651.66
									SIESTLDDLFR	95.0%	70.9	22.0	34	0	0	2	1,295.65
									SPCPSVVAPVCGSDASTYSNECELQR	95.0%	67.8	17.1	0	2	0	2	2,870.24
									SPCQPNPCHGAAPCR	95.0%	63.2	17.1	5	3	0	2	1,708.71
									SREPVTLGAWTR	95.0%	51.4	22.5	25	32	0	2	1,372.73
									STVPVNTNR	95.0%	53.1	22.4	17	0	0	2	987.52
									TDGKGDFVSLALR	95.0%	76.5	22.2	12	3	0	2	1,378.73
									TEATQGLVLWSGK	95.0%	103.0	22.3	84	0	0	2	1,389.74
									TFVEYLNAVTESEK	95.0%	124.0	22.4	164	0	0	2	1,629.80
									TFVGAGLR	95.0%	55.5	23.1	21	0	0	2	820.47
									TPSLDAEGSNCPATK	95.0%	97.3	20.8	8	0	0	2	1,547.70
									TYDSDCWR	95.0%	49.4	13.4	11	0	0	2	1,102.43
									VCGSDGVTYGNECQLK	95.0%	106.0	18.4	31	0	0	2	1,786.77
									VGPTCADEK	95.0%	51.0	19.4	2	0	0	2	976.44
									VGPTCADEKSPCQPNPCHGAAPCR	95.0%	70.6	15.6	0	2	1	2	2,666.13
									VLGAPVPAFEGR	95.0%	69.0	21.0	44	0	0	2	1,212.67
									VLGESPVPHTVLNLK	95.0%	71.0	16.3	23	4	0	2	1,602.92
									VLGESPVPHTVLNLKEPLYVGGAPDFSK	95.0%	60.9	18.0	0	12	3	2	2,963.59
									VLPEGGAQCECPLGR	95.0%	81.1	21.6	7	0	0	2	1,642.77
									VTCDGAYRPVCAQDGR	95.0%	37.7	20.0	2	10	0	2	1,824.81
									VVISGFGDPLICDNQVSTGDTR	95.0%	108.0	20.9	14	2	0	2	2,350.14
Ribosome-binding protein 1	RRBP1_HUMAN	RRBP1	152,453	100.00%	4	4	11	4.33%	DALNQATSQVESK	95.0%	72.8	22.3	3	0	0	2	1,390.68
									DAQDVQASQAEADQQQTR	95.0%	139.0	20.3	6	0	0	2	1,988.89
									GNTPATGTTQGK	95.0%	38.5	22.4	1	0	0	2	1,132.56
									VEPAVSSVVNSIQVLTSK	95.0%	61.8	17.9	1	0	0	2	1,857.03
Kinesin-1 heavy chain	KINH_HUMAN	KIF5B	109,668	100.00%	4	4	8	5.30%	ISFLENNLEQLTK	95.0%	54.2	22.3	2	0	0	2	1,548.83
									LITDLQDQNQK	95.0%	45.7	22.9	2	0	0	2	1,315.69

									QAVEQQIQSHR	95.0%	31.7	22.6	0	1	0	2	1,323.68
									SAEIDSDDTGGSAAQK	95.0%	71.5	18.8	3	0	0	2	1,551.68
Rab GDP dissociation inhibitor beta	GDIB_HUMAN	GDI2	50,648	100.00%	23	27	108	60.00%	DLGTESQIFISR	95.0%	80.0	22.6	6	0	0	2	1,365.70
									DWNVDLIPK	95.0%	43.3	21.6	2	0	0	2	1,099.58
									EIRPALELLEPIEQK	95.0%	44.8	16.1	0	2	0	2	1,778.01
									EPEKEIRPALELLEPIEQK	95.0%	23.5	17.9	0	0	2	2	2,261.24
									FKIPGSPPESMGR	95.0%	39.5	23.4	3	0	0	2	1,418.71
									FLMANGQLVK	95.0%	43.4	22.8	6	0	0	2	1,136.61
									FVSISDLLVPK	95.0%	65.7	16.5	6	0	0	2	1,217.71
									GRDWNVDLIPK	95.0%	36.0	21.5	1	0	0	2	1,312.70
									IPGSPPESMGR	95.0%	43.3	21.1	2	0	0	2	1,143.55
									LSAIYGGTYMLNKPIEEIIVQNGK	95.0%	53.7	19.3	0	1	0	2	2,667.41
									LYSESLAR	95.0%	55.9	20.3	6	0	0	2	938.49
									MLLYTEVTR	95.0%	53.4	23.0	13	0	0	2	1,125.60
									MTGSEFDFEEMKR	95.0%	62.2	17.3	3	0	0	2	1,622.68
									NPYYGGESASITPLEDLYK	95.0%	119.0	22.1	6	0	0	2	2,117.01
									NPYYGGESASITPLEDLYKR	95.0%	113.0	21.6	2	2	0	2	2,273.11
									NTNDANSCQIIIPQNQVNR	95.0%	59.4	21.9	1	0	0	2	2,199.06
									QLICDPSYVK	95.0%	32.8	23.3	1	0	0	2	1,222.61
									SPYLYPLYGLGELPQGFAR	95.0%	98.6	21.6	8	0	0	2	2,141.11
									TDDYLDQPCYETINR	95.0%	97.4	18.1	2	0	0	2	1,902.82
									TYDATTHFETTCDDIK	95.0%	66.4	17.8	2	2	0	2	1,917.82
									VICILSHPIK	95.0%	37.9	18.4	2	1	0	2	1,179.69
									VPSTEAEALASSLMGLFEK	95.0%	103.0	22.6	14	9	0	2	1,980.00
									VTEGSFVYK	95.0%	44.1	21.8	3	0	0	2	1,029.53
Prefoldin subunit 3	PFD3_HUMAN	VBP1	22,641	100.00%	3	3	4	16.80%	KLDEQYQK	95.0%	33.5	21.9	2	0	0	2	1,051.54
									NLDSLEEDLDFLR	95.0%	45.3	22.6	1	0	0	2	1,578.77
									QPGNETADTVLK	95.0%	43.2	21.9	1	0	0	2	1,272.64
DNA-(apurinic or apyrimidinic site) lyase	APEX1_HUMAN	APEX1	35,538	100.00%	12	14	96	47.20%	EAAGEGPALYEDPPDQK	95.0%	76.3	20.6	5	0	0	2	1,786.81
									EGYSGVGLLSR	95.0%	70.3	22.8	11	0	0	2	1,137.59
									GAVAEDGDELR	95.0%	36.2	20.2	2	0	0	2	1,131.53
									GAVAEDGDELRTEPEAK	95.0%	85.5	21.5	4	3	0	2	1,786.85
									ICSWNVDGLR	95.0%	39.8	21.8	2	0	0	2	1,219.59
									LDYFLLSHSLLPALCDSK	95.0%	45.9	21.1	1	0	0	2	2,092.08
									LPAELQELPGLSHQYWSAPSDK	95.0%	57.0	20.9	0	4	0	2	2,466.23
									NAGFTPQER	95.0%	45.0	22.8	8	0	0	2	1,019.49
									NDKEAAGEGPALYEDPPDQK	95.0%	44.8	21.0	0	2	0	2	2,143.98
									QGFGELLQAVPLADSFR	95.0%	89.8	21.6	41	0	0	2	1,847.97
									TSPSGKPATLK	95.0%	31.5	21.2	2	0	0	2	1,086.62
									VSYGIGDEEHDQEGR	95.0%	81.7	17.4	10	1	0	2	1,690.73
Calnexin	CALX_HUMAN	CANX	67,552	100.00%	2	2	2	5.24%	APVPTGEVYFADSFDR	95.0%	82.3	21.5	1	0	0	2	1,770.83
									KIPNPDFFEDLEPFR	95.0%	32.5	22.6	0	1	0	2	1,863.93
High mobility group protein B3	HMGB3_HUMAN	HMGB3	22,963	100.00%	6	6	21	29.50%	KLGEMWNNLNDSEK	95.0%	73.0	22.7	2	0	0	2	1,677.79
									KNPEVPVNFAEFSK	95.0%	40.0	22.3	2	0	0	2	1,605.83
									LGEMWNNLNDSEK	95.0%	74.0	19.5	5	0	0	2	1,565.69
									LGEMWNNLNDSEKQPYITK	95.0%	40.7	22.1	0	2	0	2	2,280.10
									MSAYAFFVQTCR	95.0%	91.7	21.0	2	0	0	2	1,496.67
									STNPGISIGDVAK	95.0%	97.2	23.0	8	0	0	2	1,258.66
DnaJ homolog subfamily C member 8	DNJC8_HUMAN	DNAJC8	29,824	99.90%	2	2	6	10.70%	EGKPTIVEEDDPELFK	95.0%	57.9	22.3	2	0	0	2	1,845.91
									QREEEIEAQEK	95.0%	38.6	20.8	4	0	0	2	1,388.67
Cofilin-1	COF1_HUMAN	CFL1	18,485	100.00%	9	11	172	66.30%	AVLFCLSEDKK	95.0%	65.1	22.5	2	0	0	2	1,309.68

									EILVGDVGQTVDDPYATFVK	95.0%	113.0	21.6	10	0	0	2	2,166.10
									HELQANCYEEVKDR	95.0%	58.2	21.4	0	13	0	2	1,790.81
									KEDLVFIFWAPESAPLK	95.0%	79.6	19.5	7	11	0	2	1,990.07
									LGGSAVISLEGKPL	95.0%	91.4	18.0	61	0	0	2	1,340.78
									MIYASSK	95.0%	37.6	20.3	1	0	0	2	815.40
									MLPDKDCR	95.0%	33.2	20.8	1	0	0	2	1,050.47
									NIILEEGKEILVGDVGQTVDDPYATFVK	95.0%	107.0	19.1	0	57	1	2	3,062.59
									YALYDATYETK	95.0%	72.4	22.2	8	0	0	2	1,337.63
WD repeat-containing protein 5	WDR5_HUMAN	WDR5	36,571	100.00%	4	4	6	17.10%	FSPNGEWLASSSADK	95.0%	76.9	21.3	2	0	0	2	1,595.73
									TLIDDDNPPVSFVK	95.0%	77.7	23.2	2	0	0	2	1,559.80
									TLPAHSDPVSAVHFNR	95.0%	38.3	22.5	0	1	0	2	1,747.89
									YILAATLDNTLK	95.0%	69.5	20.0	1	0	0	2	1,335.75
Eukaryotic translation initiation factor 3 subunit K	EIF3K_HUMAN	EIF3K	25,042	100.00%	3	3	6	18.80%	ENAYDLEANLAVLK	95.0%	38.8	23.0	2	0	0	2	1,562.81
									WLLAEMLGDLSDSQLK	95.0%	80.8	22.4	2	0	0	2	1,834.93
									YNPENLATLER	95.0%	56.2	22.7	2	0	0	2	1,319.66
60S acidic ribosomal protein P2	RLA2_HUMAN	RPLP2	11,647	100.00%	2	2	4	39.10%	LASVPAGGAVAVSAAPGSAAPAAGSAPAAAEEK	95.0%	33.5	20.0	0	2	0	2	2,774.43
									NIEDVIAQGIGK	95.0%	77.3	22.2	2	0	0	2	1,256.69
UV excision repair protein RAD23 homolog B	RD23B_HUMAN	RAD23B	43,153	100.00%	3	5	11	13.90%	NQPQFQQMR	95.0%	33.0	22.0	2	0	0	2	1,192.55
									QEKPAEKPAETPVATSPTATDSTSGDSSR	95.0%	64.8	20.9	0	2	1	2	2,945.40
									QIIQQNPSLLPALLQQIGR	95.0%	97.4	11.1	5	1	0	2	2,130.24
Serglycin	SRGN_HUMAN	SRGN	17,635	100.00%	3	3	11	20.90%	CNPDSNSANCLEEK	95.0%	74.7	13.4	4	0	0	2	1,637.65
									GPMFELLPGESNK	95.0%	57.5	22.9	6	0	0	2	1,418.70
									IQDLNR	94.9%	30.3	22.6	1	0	0	2	758.42
Alpha-soluble NSF attachment protein	SNAA_HUMAN	NAPA	33,216	100.00%	3	3	6	13.90%	EAEAMALLAEAER	95.0%	77.4	22.3	2	0	0	2	1,403.68
									NSQSFFSGLFGGSSK	95.0%	115.0	22.4	2	0	0	2	1,549.73
									VAGYAALLEQYQK	95.0%	66.1	21.9	2	0	0	2	1,453.77
Zyxin	ZYX_HUMAN	ZYX	61,258	100.00%	3	3	6	7.52%	FSPGAPGGSGSQPNQK	95.0%	53.9	21.4	1	0	0	2	1,515.72
									GPPASSPAPAPK	95.0%	39.3	23.3	1	0	0	2	1,076.57
									QHPVPPPAQNQNQVR	95.0%	57.2	21.2	0	4	0	2	1,709.88
Anterior gradient protein 2 homolog	AGR2_HUMAN	AGR2	19,962	100.00%	5	5	13	26.90%	HLSPDGQYVPR	95.0%	38.7	22.2	2	0	0	2	1,268.64
									IMFVDPSLTVR	95.0%	56.0	22.3	4	0	0	2	1,293.69
									LPQTLSR	95.0%	35.4	20.8	2	0	0	2	814.48
									LYAYEPADTALLLDNMK	95.0%	94.2	22.5	4	0	0	2	1,940.97
									LYAYEPADTALLLDNMKK	95.0%	75.9	22.5	1	0	0	2	2,069.06
26S proteasome non-ATPase regulatory subunit 5	PSMD5_HUMAN	PSMD5	56,179	100.00%	3	3	8	10.70%	IVENSDAVTEILNNAELLK	95.0%	96.4	19.9	4	0	0	2	2,085.11
									TYLSEGPYYVKPVSTTAVEGAE	95.0%	50.4	21.6	2	0	0	2	2,361.15
									VFTAIANQPWAQK	95.0%	85.0	22.0	2	0	0	2	1,473.79
General vesicular transport factor p115	USO1_HUMAN	USO1	107,880	100.00%	2	2	4	2.29%	IVAFENAFER	95.0%	36.3	22.1	2	0	0	2	1,195.61
									NDGVLLLQALTR	95.0%	61.5	19.3	2	0	0	2	1,312.76
Epidermal growth factor receptor	EGFR_HUMAN	EGFR	134,261	100.00%	7	9	36	7.19%	EISDGDVIISGNK	95.0%	43.2	23.9	5	0	0	2	1,346.68
									GDSFTHTPPLDPQELDILK	95.0%	45.7	21.6	2	9	0	2	2,123.07
									IICAQQCSGR	95.0%	42.7	21.8	2	0	0	2	1,192.56
									IPLENLQIIR	95.0%	64.8	14.3	9	0	0	2	1,208.74
									LTQLGTFEDHFLSLQR	95.0%	46.2	22.2	2	1	0	2	1,904.99
									NLQEILHGAVR	95.0%	53.7	20.1	2	0	0	2	1,249.70
									NYDLSFLK	95.0%	47.5	20.9	4	0	0	2	999.52
Alpha-N-acetylglucosaminidase	ANAG_HUMAN	NAGLU	82,150	100.00%	5	6	12	10.20%	AGGVLAYELLPALDEVLASDSR	95.0%	51.0	19.7	2	1	0	2	2,259.19
									FLLGSWLEQAR	95.0%	53.8	22.1	4	0	0	2	1,319.71
									LLLTSAPSLATSPAFR	95.0%	65.7	17.9	3	0	0	2	1,644.93
									LPRPLPAVPGELTEATPNR	95.0%	32.8	17.0	0	1	0	2	2,028.12
									YDLLDLTR	95.0%	31.2	21.4	1	0	0	2	1,008.54

PDZ and LIM domain protein 1	PDLI1_HUMAN	PDLIM1	36,053	100.00%	11	12	28	48.60%	CGTGIVGVFVK	95.0%	58.0	22.7	2	0	0	2	1,136.61
									DFEQPLAISR	95.0%	43.0	23.6	3	0	0	2	1,175.61
									ERVTPPEGYEVVTVFPK	95.0%	39.9	21.5	0	1	0	2	1,947.02
									LVGGKDFEQPLAISR	95.0%	74.2	20.9	2	2	0	2	1,629.90
									MNLASEPQEVLHIGSAHNR	95.0%	35.2	22.2	0	0	2	2	2,119.04
									SAMPFTASPASSTTAR	95.0%	91.2	22.2	5	0	0	2	1,598.75
									TAASGVEANSRPLDHAQPPSSLVIDKESEVYK	95.0%	22.4	20.3	0	0	1	2	3,395.71
									VAASIGNAQK	95.0%	63.2	22.5	3	0	0	2	958.53
									VITNQYNNPAGLYSSENISNFNNALESK	95.0%	51.0	21.0	0	2	0	2	3,101.48
									VTPPEGYEVVTVFPK	95.0%	43.6	21.0	3	0	0	2	1,661.88
									VWSPLVTEEGKR	95.0%	57.6	21.9	2	0	0	2	1,400.75
Lysozyme C	LYSC_HUMAN	LYZ	16,519	100.00%	2	2	9	27.00%	STDYGIFQINSR	95.0%	86.2	21.3	6	0	0	2	1,400.68
									TPGAVNACHLSCSALLQDNIADAVACAK	95.0%	58.8	20.6	0	3	0	2	2,927.38
Stress-induced-phosphoprotein 1	STIP1_HUMAN	STIP1	62,624	100.00%	21	24	67	40.70%	AAALEFLNR	95.0%	59.5	23.5	2	0	0	2	1,004.55
									ALSVGNIDDALQCYSEAIK	95.0%	110.0	21.8	2	0	0	2	2,067.01
									AMADPEVQQIMSDPAMR	95.0%	95.9	17.9	2	0	0	2	1,937.84
									AMDVYQK	95.0%	33.4	19.5	1	0	0	2	870.40
									DPQALSEHLKNPVIAQK	95.0%	39.4	19.2	0	2	0	2	1,888.03
									EAADGYQR	95.0%	38.0	20.9	2	0	0	2	909.41
									EGLQNMEAR	95.0%	44.3	19.3	5	0	0	2	1,063.48
									ELDPTNMTYITNQAAVYFEK	95.0%	82.4	20.5	4	0	0	2	2,364.11
									ELGNDAYK	95.0%	33.8	21.8	1	0	0	2	909.43
									ETKPEPMEEDLPENKK	95.0%	38.1	21.6	1	3	0	2	1,913.92
									FMNPFNMPNLYQK	95.0%	33.2	21.0	1	0	0	2	1,659.77
									HDSPEDVKR	95.0%	36.3	22.6	0	2	0	2	1,082.52
									HEANNPQLK	95.0%	41.6	23.2	2	0	0	2	1,050.53
									IGNSYFKEEK	95.0%	56.4	22.5	2	0	0	2	1,214.61
									ILKEQER	95.0%	38.5	23.4	2	0	0	2	915.53
									KAAALEFLNR	95.0%	29.9	20.6	0	1	0	2	1,132.65
									LAYINPDLALEEK	95.0%	70.5	22.5	12	0	0	2	1,488.80
									LDPHNHVLYSNR	95.0%	38.3	22.4	2	0	2	2	1,464.73
									LMDVGLIAIR	95.0%	70.8	20.8	10	0	0	2	1,100.65
									NPVIAQK	95.0%	45.0	17.2	2	0	0	2	769.46
									TYEEGLKHEANNPQLK	95.0%	52.6	22.0	2	2	0	2	1,870.93
ADP-sugar pyrophosphatase	NUDT5_HUMAN	NUDT5	24,310	100.00%	5	6	15	23.30%	EQTADGVAVIPVLQR	95.0%	89.5	19.5	2	0	0	2	1,595.88
									HANAKPFEVPFLK	95.0%	33.4	20.5	0	2	0	2	1,497.82
									KEQTADGVAVIPVLQR	95.0%	85.6	16.9	3	4	0	2	1,723.97
									QYIISEELISEGK	95.0%	59.6	22.6	3	0	0	2	1,508.79
									TTYMDPTGK	95.0%	35.3	18.6	1	0	0	2	1,029.46
EH domain-containing protein 4	EHD4_HUMAN	EHD4	61,160	100.00%	3	3	5	5.91%	EYQISAGDFPEVK	95.0%	35.1	22.6	2	0	0	2	1,482.71
									FGNAFLNR	95.0%	40.3	22.3	1	0	0	2	938.48
									LFEAEAQDLFR	95.0%	50.0	21.8	2	0	0	2	1,338.67
Carcinoembryonic antigen-related cell adhesion molecule 5	CEAM5_HUMAN	CEACAM5	76,778	99.50%	2	2	12	6.13%	QIIGYVIGTQQATPGPAYSGR	95.0%	102.0	20.1	4	0	0	2	2,177.14
									SDLVNEEATGQFR	95.0%	68.5	21.5	8	0	0	2	1,465.69
									TLTLLSVTR	95.0%	64.0	13.2	25	0	0	2	1,003.62
Lamin-A/C	LMNA_HUMAN	LMNA	74,123	100.00%	27	32	213	43.50%	AAYEAELGDAR	95.0%	59.7	22.3	2	0	0	2	1,165.55
									AAYEAELGDARK	95.0%	39.3	23.2	2	0	0	2	1,293.64
									AGQVVTIWAAGAGATHSPPTDLVWK	95.0%	53.4	19.5	0	2	0	2	2,533.32
									AQHEDQVEQYKK	95.0%	46.5	21.8	0	5	0	2	1,502.72
									EAALSTALSEKR	95.0%	57.5	22.0	6	0	0	2	1,275.69
									IDSLSAQLSQLQK	95.0%	84.6	20.7	15	0	0	2	1,430.79

									IRIDSLSAQLSQLQK	95.0%	40.4	16.9	0	2	0	2	1,699.97
									ITESEEVVSR	95.0%	90.0	23.4	19	0	0	2	1,148.58
									KLESTESR	95.0%	59.2	22.1	5	0	0	2	949.50
									LADALQELR	95.0%	76.1	22.6	4	0	0	2	1,028.57
									LAVYIDR	94.8%	30.2	20.6	1	0	0	2	849.48
									LEAALGEAK	95.0%	37.0	21.7	2	0	0	2	901.50
									LKDLEALLNSK	95.0%	60.2	19.0	11	2	0	2	1,243.73
									LLEGEEER	95.0%	39.2	21.6	2	0	0	2	974.48
									LQEKEDLQELNDR	95.0%	87.4	22.1	15	17	0	2	1,629.81
									LQTMKEELDFQK	95.0%	44.9	22.7	4	0	0	2	1,525.76
									LRDLEDSLAR	95.0%	32.6	22.8	1	1	0	2	1,187.64
									LSPSPTSQR	95.0%	36.3	21.4	2	0	0	2	972.51
									MQQQLDEYQELLDIK	95.0%	105.0	22.6	23	0	0	2	1,909.92
									NSNLVGAAHEELQQSR	95.0%	101.0	22.1	8	10	0	2	1,752.86
									QNGDDPLLTYR	95.0%	42.9	22.6	1	0	0	2	1,291.63
									SGAQASSTPLSPTR	95.0%	71.2	22.6	15	0	0	2	1,359.69
									SNEDQSMGNWQIK	95.0%	54.1	19.2	2	0	0	2	1,552.67
									TALINSTGEEVAMR	95.0%	77.5	22.9	13	0	0	2	1,507.74
									TLEGELHDLR	95.0%	47.2	22.4	7	0	0	2	1,182.61
									VAVEEVDEEGK	95.0%	62.5	21.6	4	0	0	2	1,203.57
									VAVEEVDEEGKFVR	95.0%	77.9	22.5	6	4	0	2	1,605.81
N-acetylglucosamine-6-sulfatase	GNS_HUMAN	GNS	62,066	100.00%	5	5	21	10.10%	AFQNVFAPR	95.0%	51.9	23.2	2	0	0	2	1,049.55
									IQEPNTFPAILR	95.0%	59.9	20.3	10	0	0	2	1,398.77
									SDVLVEYQGEGR	95.0%	47.5	22.3	2	0	0	2	1,351.65
									TPGVFDPGYR	95.0%	37.6	22.4	3	0	0	2	1,108.54
									WQTLLSVDDLVEK	95.0%	79.1	23.3	4	0	0	2	1,545.82
COP9 signalosome complex subunit 4	CSN4_HUMAN	COPS4	46,252	100.00%	3	3	6	9.36%	IASQMITEGR	95.0%	46.6	24.1	2	0	0	2	1,121.56
									NAAQVLVGIPLETGQK	95.0%	58.1	18.2	2	0	0	2	1,637.92
									VISFEEQVASIR	95.0%	61.4	22.0	2	0	0	2	1,377.74
Vacuolar protein sorting-associated protein 29	VPS29_HUMAN	VPS29	20,488	99.50%	2	3	5	12.60%	GDFDENLNYPEQK	95.0%	43.7	19.5	2	0	0	2	1,568.69
									TLAGDVHIVR	95.0%	44.5	19.1	2	1	0	2	1,080.62
Clathrin heavy chain 1	CLH1_HUMAN	CLTC	191,601	100.00%	32	35	130	24.30%	ADDPSSYMEVVQAANTSGNWEELVK	95.0%	40.7	19.1	1	0	0	2	2,756.24
									AFMTADLPNELIELLEK	95.0%	40.7	22.3	2	0	0	2	1,963.01
									ALEHFTDLYDIKR	95.0%	38.4	22.8	0	2	0	2	1,620.84
									AYEFAER	95.0%	39.5	19.1	2	0	0	2	885.41
									DAMQYASESK	95.0%	47.5	15.2	2	0	0	2	1,145.48
									ENPYYDSR	95.0%	30.9	17.9	1	0	0	2	1,043.44
									FNALFAQGNYSEAAK	95.0%	56.5	22.2	1	0	0	2	1,630.79
									GQCDLELINVCNENSLFK	95.0%	45.5	21.7	1	0	0	2	2,153.00
									GQFSTDELVAEVEK	95.0%	98.2	23.4	2	0	0	2	1,551.75
									HELIEFR	95.0%	39.3	20.5	1	0	0	2	943.50
									IAAYLFK	95.0%	31.8	19.0	1	0	0	2	825.49
									ISGETIFVTAPHEATAGIIGVNR	95.0%	52.4	18.9	0	10	0	2	2,353.25
									IVLDNSVFSEHR	95.0%	89.7	21.6	1	4	0	2	1,415.73
									IYIDSNNNPER	95.0%	53.9	22.4	12	0	0	2	1,334.63
									KFNALFAQGNYSEAAK	95.0%	37.2	22.6	0	1	0	2	1,758.88
									LAELEEFINGPNNAHIQQVGDR	95.0%	57.2	21.5	0	5	0	2	2,464.22
									LASTLVHLGEYQAAVDGAR	95.0%	49.2	21.4	0	11	0	2	1,971.03
									LEKHELIEFR	95.0%	33.3	20.8	0	2	0	2	1,313.72
									LLLPWLEAR	95.0%	40.3	15.8	7	0	0	2	1,110.67
									LLYNNVSNFGR	95.0%	72.7	22.4	7	0	0	2	1,296.67

									NLQNLLILTAIK	95.0%	87.7	10.0	10	0	0	2	1,353.85
									NNLAGAEELFAR	95.0%	93.1	23.5	7	0	0	2	1,304.66
									NNRPSEGPLQTR	95.0%	44.4	23.3	2	7	0	2	1,368.70
									RPISADSAIMNPASK	95.0%	59.4	23.3	2	2	0	2	1,573.80
									SVNESLNNLFITEEDYQALR	95.0%	113.0	21.6	2	0	0	2	2,355.15
									TLQIFNIEMK	95.0%	53.5	22.0	6	0	0	2	1,252.66
									TSIDAYDNFDNISLAQR	95.0%	114.0	22.0	3	0	0	2	1,942.91
									VGYTPDWIFLLR	95.0%	32.9	21.4	1	0	0	2	1,479.80
									VIQCFAETGQVQK	95.0%	91.1	22.6	2	0	0	2	1,507.76
									VMEYINR	95.0%	38.2	22.1	2	0	0	2	940.46
									VVGAMQLYSVDR	95.0%	67.8	22.5	4	0	0	2	1,337.69
									WLLLTGISAQQNR	95.0%	87.9	20.0	4	0	0	2	1,499.83
Annexin A4	ANXA4_HUMAN	ANXA4	35,866	100.00%	11	11	54	42.00%	AASGFNAMEDAQTLR	95.0%	119.0	21.8	4	0	0	2	1,581.73
									AASGFNAMEDAQTLRK	95.0%	75.2	22.2	2	0	0	2	1,725.82
									AEIDMLDIR	95.0%	59.3	24.4	4	0	0	2	1,091.54
									DEGNYLDDALVR	95.0%	60.7	21.3	2	0	0	2	1,379.64
									GAGTDEGCLIEILASR	95.0%	103.0	22.9	2	0	0	2	1,661.82
									GLGTDDNTLIR	95.0%	55.4	23.5	2	0	0	2	1,174.61
									GLGTDEDAIISVLAYR	95.0%	103.0	21.4	10	0	0	2	1,692.88
									ISQTYQQQYGR	95.0%	63.6	21.6	5	0	0	2	1,371.67
									QDAQDLYEAGEK	95.0%	55.8	19.1	2	0	0	2	1,366.61
									SDTSFMFQR	95.0%	65.0	18.5	5	0	0	2	1,118.49
									SETSGSFEDALLAIVK	95.0%	107.0	21.7	16	0	0	2	1,666.85
									TPEEIR	95.0%	40.6	20.7	1	0	0	2	744.39
Fumarate hydratase, mitochondrial	FUMH_HUMAN	FH	54,620	100.00%	4	4	7	9.41%	AAAEVNQDYGLDPK	95.0%	62.8	22.0	2	0	0	2	1,490.71
									IANDIR	95.0%	32.4	22.7	2	0	0	2	701.39
									IGGVTER	95.0%	42.7	25.1	1	0	0	2	731.41
									SGLGELILPENEPGSSIMPGK	95.0%	46.1	22.2	2	0	0	2	2,141.08
Proteasome subunit beta type-7	PSB7_HUMAN	PSMB7	29,948	100.00%	6	8	24	22.70%	ATEGMVVADK	95.0%	33.7	23.1	2	0	0	2	1,020.50
									DGIVLGADTR	95.0%	33.7	22.3	3	0	0	2	1,016.54
									FRPDMEEEEAK	95.0%	50.1	18.2	10	2	0	2	1,396.61
									ITPLEIEVLEETVQTMDTS	95.0%	58.4	22.1	2	0	0	2	2,164.06
									LDFLRPYTVPNK	95.0%	35.5	20.4	1	3	0	2	1,462.81
									LDFLRPYTVPNKK	95.0%	30.6	15.8	0	1	0	2	1,590.90
Coatomer subunit alpha	COPA_HUMAN	COPA	138,331	100.00%	4	4	6	4.08%	DADSITLFDVQQK	95.0%	51.0	22.7	1	0	0	2	1,479.73
									LLELGPKPEVAQQTR	95.0%	32.1	16.6	0	2	0	2	1,678.95
									SILLSVPLLVVDNK	95.0%	47.5	11.5	1	0	0	2	1,509.93
									TALNLFFK	95.0%	37.9	20.2	2	0	0	2	953.55
Beta-hexosaminidase subunit beta	HEXB_HUMAN	HEXB	63,095	100.00%	8	10	34	18.90%	EISEVFPDQFIHLGGDEVEFK	95.0%	52.4	21.4	1	1	0	2	2,435.18
									GSIVWQEVFDDK	95.0%	56.6	22.0	6	0	0	2	1,422.69
									GSYSLSHVYTPNDVR	95.0%	63.3	22.5	6	1	0	2	1,694.81
									KLESFYIQK	95.0%	30.5	20.6	1	0	0	2	1,155.64
									LAPGTIVEVWK	95.0%	49.0	18.3	6	0	0	2	1,212.70
									VEPLDFGGTQK	95.0%	30.5	23.7	1	0	0	2	1,190.61
									VLDIIATINK	95.0%	62.2	18.0	7	0	0	2	1,099.67
									VLPEFDTPGHTLSWGK	95.0%	61.7	21.8	4	0	0	2	1,783.90
DNA-binding protein A	DBPA_HUMAN	CSDA	40,071	100.00%	3	3	6	20.70%	AGEAPTENPAPPTQQSSAE	95.0%	64.4	20.3	1	0	0	2	1,881.85
									GAEAANVTGPDGVPVEGSR	95.0%	65.3	22.9	4	0	0	2	1,782.86
									NDTKEDVFVHQTAIK	95.0%	35.6	22.7	0	2	0	2	1,744.89
									NPTYRPR	95.0%	31.9	23.9	1	0	0	2	903.48
									SVGDGETVEFDVVEGEK	95.0%	45.0	21.7	2	0	0	2	1,795.82

F-actin-capping protein subunit beta	CAPZB_HUMAN	CAPZB	31,334	100.00%	6	6	51	23.80%	LEVEANNAFDQYR	95.0%	94.7	21.9	4	0	0	2	1,568.73
									RLPPQQIEK	95.0%	44.1	18.8	4	0	0	2	1,108.65
									SGSGTMNLGGSLTR	95.0%	89.8	21.5	17	0	0	2	1,337.65
									SPWSNKYDPPLEDGAMPSAR	95.0%	41.4	20.5	0	7	0	2	2,234.02
									STLNEIYFGK	95.0%	76.9	22.8	18	0	0	2	1,171.60
									YDPPLEDGAMPSAR	95.0%	50.1	20.0	1	0	0	2	1,518.69
Superoxide dismutase [Mn], mitochondrial	SODM_HUMAN	SOD2	24,705	100.00%	4	4	19	19.80%	AIWNVINWENVTER	95.0%	71.2	22.6	8	0	0	2	1,743.88
									GDVTAQIALQPALK	95.0%	70.7	16.7	4	0	0	2	1,424.81
									GELLEAIKR	95.0%	50.6	19.6	5	0	0	2	1,028.61
									YQEALAK	95.0%	43.4	22.4	2	0	0	2	822.44
Heat shock protein 105 kDa	HS105_HUMAN	HSPH1	96,848	100.00%	19	22	52	27.00%	AFNDPFIQK	95.0%	42.6	23.1	4	0	0	2	1,079.55
									DLLNMYIETEGK	95.0%	52.4	21.6	2	0	0	2	1,441.69
									EKENLSYDLVPLK	95.0%	76.8	21.4	2	0	0	2	1,547.83
									ENLSYDLVPLK	95.0%	53.1	22.3	1	0	0	2	1,290.69
									FQEAEERPK	95.0%	42.6	23.1	5	4	0	2	1,133.56
									FVVQNVSAQK	95.0%	68.8	23.0	2	0	0	2	1,119.62
									GCALQCAILSPAFK	95.0%	37.1	22.6	1	0	0	2	1,535.77
									IEVPLYSLLEQTHLK	95.0%	61.3	18.0	2	0	0	2	1,783.00
									KVDQPPEAK	95.0%	36.9	20.5	2	0	0	2	1,011.55
									LKETAENSLK	95.0%	32.6	22.6	1	1	0	2	1,132.62
									LLTETEDWLYEEGEDQAK	95.0%	115.0	21.1	2	0	0	2	2,168.99
									MFEELGQR	95.0%	37.5	21.6	2	0	0	2	1,009.48
									NAVEEYVYEFR	95.0%	32.6	21.4	2	0	0	2	1,418.66
									NQQITHANNTVSNFK	95.0%	88.2	22.0	2	1	0	2	1,715.85
									QAYVDKLEELMK	95.0%	51.6	22.3	2	0	0	2	1,482.75
									QDLPSLDEKPR	95.0%	29.9	22.0	0	1	0	2	1,297.68
									SQFEELCAELLQK	95.0%	40.1	22.7	2	0	0	2	1,594.78
									SVLDAAQIVGLNCLR	95.0%	81.1	20.8	2	0	0	2	1,628.88
									VEDVSAVEIVGGATR	95.0%	89.5	22.9	4	0	0	2	1,501.79
									VLGTAFDPFLGGK	95.0%	67.8	21.2	6	0	0	2	1,321.72
Proteasome-associated protein ECM29 homolog	ECM29_HUMAN	ECM29	204,278	99.90%	2	2	3	1.63%	AGEQLAPFLPQLVPR	95.0%	37.2	16.8	2	0	0	2	1,635.92
									TLMSSGQMAPSSSNK	95.0%	72.0	18.3	1	0	0	2	1,557.69
Farnesyl pyrophosphate synthase	FPPS_HUMAN	FDPS	48,259	100.00%	8	10	41	19.60%	ATPEQYQILK	95.0%	43.5	22.5	4	0	0	2	1,190.64
									CSWLVVQCLQR	95.0%	44.5	23.2	2	0	0	2	1,448.71
									EVLEYNAIGGK	95.0%	47.2	23.3	2	0	0	2	1,192.62
									IGTDIQDNK	95.0%	44.6	22.8	4	0	0	2	1,003.51
									KQDADSLQR	95.0%	51.2	24.4	3	0	0	2	1,060.54
									LKEVLEYNAIGGK	95.0%	53.4	19.7	2	2	0	2	1,433.80
									QDFVQHFSQIVR	95.0%	42.3	22.9	0	9	0	2	1,503.77
									VLTEDEMGHPEIGDAIAR	95.0%	75.2	22.0	5	8	0	2	1,952.94
Sorbitol dehydrogenase	DHSO_HUMAN	SORD	38,307	100.00%	3	3	7	11.80%	AMGAAQVVVTDLSATR	95.0%	120.0	22.4	4	0	0	2	1,605.83
									LENYPIPEPGPNEVLLR	95.0%	67.0	20.6	2	0	0	2	1,950.03
									VAIEPGAPR	95.0%	33.3	19.2	1	0	0	2	909.52
Disintegrin and metalloproteinase domain-containing protein 10	ADA10_HUMAN	ADAM10	84,125	100.00%	3	3	7	4.55%	AIDTIYQTTDFSGIR	95.0%	74.4	22.4	4	0	0	2	1,700.85
									FPNIGVEK	95.0%	49.0	22.7	2	0	0	2	903.49
									GGTFYVEPAER	95.0%	36.4	21.9	1	0	0	2	1,225.59
DCN1-like protein 1	DCNL1_HUMAN	DCUN1D1	30,108	100.00%	5	5	7	20.80%	FLDLWNK	95.0%	30.4	23.3	1	0	0	2	935.50
									LDVATDNFFQNPELYIR	95.0%	81.3	23.4	2	0	0	2	2,055.02
									MEQELKEPGR	95.0%	31.7	21.5	0	1	0	2	1,232.59
									QFMIFTQSSEK	95.0%	50.5	22.5	2	0	0	2	1,345.65
									YKDPQDENK	95.0%	30.4	22.4	1	0	0	2	1,136.52

Serpin B5	SPB5_HUMAN	SERPINB5	42,084	100.00%	14	16	63	48.50%	ACLENLGLK	95.0%	31.7	23.8	1	0	0	2	1,017.54
									DELNADHPFIYIIR	95.0%	71.1	21.9	2	0	0	2	1,715.88
									DLTDGHFENILADNSVNDQTK	95.0%	80.4	20.6	2	6	0	2	2,346.09
									DVEDESTGLEK	95.0%	63.3	19.7	4	0	0	2	1,221.55
									DVPFGFQTVTSDVNK	95.0%	105.0	22.4	14	0	0	2	1,653.81
									ELETVDFK	95.0%	34.2	22.1	1	0	0	2	980.49
									GDTANEIGQVLHFENVK	95.0%	74.5	22.4	6	8	0	2	1,870.93
									GQINNSIK	95.0%	36.6	24.0	1	0	0	2	873.48
									HIFSEDTSDFSGMSETK	95.0%	65.0	16.1	3	0	0	2	1,933.81
									IIELPFQNK	95.0%	46.8	21.4	6	0	0	2	1,101.63
									ILVVNAAYFVGK	95.0%	39.1	16.0	2	0	0	2	1,293.76
									LSSFYSLK	94.7%	30.1	23.8	1	0	0	2	944.51
									QLNSESLSQWTNPSTMANAK	95.0%	106.0	21.2	4	0	0	2	2,207.04
									SLNLSTEFISSTK	95.0%	82.6	22.0	2	0	0	2	1,426.74
Alcohol dehydrogenase [NADP+]	AK1A1_HUMAN	AKR1A1	36,556	100.00%	11	13	94	36.60%	ALEALVAK	95.0%	50.8	17.2	2	0	0	2	814.50
									AVPREELFVTSK	95.0%	35.8	21.2	2	0	0	2	1,375.76
									AWRDPDEPVLLEEPVVLALAEK	95.0%	71.4	19.4	0	18	0	2	2,489.33
									DPDEPVLLEEPVVLALAEK	95.0%	109.0	20.7	9	2	0	2	2,076.11
									GLEVTAYSPLGSSDR	95.0%	90.2	23.3	10	0	0	2	1,551.77
									GLVQALGLSNFNSR	95.0%	72.9	22.0	2	1	0	2	1,475.80
									MPLIGLGTWK	95.0%	53.7	20.5	28	0	0	2	1,115.63
									QLNALNK	95.0%	42.7	21.7	2	0	0	2	800.46
									SPAQILLR	95.0%	60.5	15.3	8	0	0	2	897.55
									VFDFTFSPEEMK	95.0%	71.4	20.2	8	0	0	2	1,492.67
									YIVPMLTVDGK	95.0%	35.7	21.7	2	0	0	2	1,251.67
Laminin subunit beta-1	LAMB1_HUMAN	LAMB1	198,045	100.00%	28	31	217	17.40%	AAQNSGEAEYIEK	95.0%	83.1	20.8	25	0	0	2	1,409.65
									AEMLLEEAKR	95.0%	46.8	23.9	2	0	0	2	1,205.62
									AMDLDQDVLSALAEVEQLSK	95.0%	90.1	22.2	2	2	0	2	2,191.08
									ATGQCLCLPNVIGQNCDR	95.0%	95.3	20.4	7	0	0	2	2,075.94
									AVITVQRPGR	95.0%	29.9	17.6	0	6	0	2	1,096.66
									DILAQSPAAEPLK	95.0%	62.0	19.5	4	0	0	2	1,352.74
									DRVEDVMMER	95.0%	28.4	18.1	0	2	0	2	1,311.57
									DVTEMMAQVEVK	95.0%	48.2	20.5	3	0	0	2	1,411.65
									EALEEAEK	95.0%	32.0	21.4	2	0	0	2	918.44
									ELAEQLEFIK	95.0%	57.1	22.8	13	0	0	2	1,219.66
									ELDSLQTEAESLDNTVK	95.0%	142.0	23.0	20	0	0	2	1,891.91
									ETVDSVER	95.0%	60.0	23.1	17	0	0	2	934.45
									IEDPYSPR	95.0%	35.7	22.3	4	0	0	2	976.47
									IPSWTGAGFVR	95.0%	47.8	22.0	12	0	0	2	1,190.63
									IQNLLK	95.0%	35.8	19.0	3	0	0	2	728.47
									ISELER	95.0%	35.8	24.3	2	0	0	2	746.40
									ISGVIGPYR	95.0%	58.3	21.2	20	0	0	2	961.55
									KAAQNSGEAEYIEK	95.0%	61.9	22.8	4	10	0	2	1,537.75
									LEGEVR	95.0%	33.4	22.7	4	0	0	2	702.38
									LHTLGDNLLDSR	95.0%	61.1	22.0	5	2	0	2	1,353.71
									NFLTQDSADLDSIEAVANEVLK	95.0%	88.2	21.4	2	0	0	2	2,392.19
									QSAEDILLK	95.0%	52.5	22.3	9	0	0	2	1,016.56
									QYIQDR	95.0%	30.4	23.4	1	0	0	2	822.41
									TLDGELDEKYK	95.0%	38.6	22.7	2	0	0	2	1,310.65
									TPMTDVCR	95.0%	53.5	18.6	5	0	0	2	995.43
									VESLSQVEVILQHSAADIAR	95.0%	54.2	18.3	0	6	0	2	2,165.16

									YEPQLPDHWEK	95.0%	32.8	21.5	2	0	0	2	1,441.68
									YSDIEPSTEGEVIFR	95.0%	68.5	21.7	19	0	0	2	1,741.83
Disintegrin and metalloproteinase domain-containing protein 9	ADAM9_HUMAN	ADAM9	90,538	100.00%	3	4	14	3.17%	DLLPEDFVVYTYNK	95.0%	51.3	22.0	6	0	0	2	1,715.85
									MDDVYKEPLK	95.0%	48.5	23.0	3	0	0	2	1,237.61
									NKDLLPEDFVVYTYNK	95.0%	71.4	21.6	4	1	0	2	1,957.99
Matrix metalloproteinase-14	MMP14_HUMAN	MMP14	65,868	99.90%	2	2	2	4.30%	SPQSLSAAIAAMQK	95.0%	70.8	23.0	1	0	0	2	1,402.74
									VGEYATYEAIR	95.0%	65.9	21.9	1	0	0	2	1,271.63
Proteasome subunit alpha type-5	PSA5_HUMAN	PSMA5	26,393	100.00%	8	9	73	46.90%	AIGSASEGAQSSLQEVYHK	95.0%	125.0	21.8	7	5	0	2	1,961.96
									EELEEVIKDI	95.0%	57.6	23.4	3	0	0	2	1,216.63
									GVNTFSPEGR	95.0%	52.4	23.3	4	0	0	2	1,063.52
									ITSPLMEPSSIEK	95.0%	73.7	23.8	11	0	0	2	1,431.74
									LFQVEYAIEAIK	95.0%	89.6	19.5	23	0	0	2	1,423.78
									LGSTAIGIQTSEGVCLAVEK	95.0%	40.8	21.1	2	0	0	2	2,033.06
									LNATNIELATVQPGQNFHMFTK	95.0%	71.0	21.2	0	6	0	2	2,474.25
									SSLIILK	95.0%	67.5	12.0	12	0	0	2	773.51
Annexin A11	ANX11_HUMAN	ANXA11	54,374	100.00%	4	4	10	7.92%	DAQELYAAGENR	95.0%	49.8	21.1	1	0	0	2	1,336.61
									FNAVLCSR	95.0%	59.5	23.2	6	0	0	2	966.48
									SETDLLDIR	95.0%	38.0	23.9	1	0	0	2	1,061.55
									TPVLFDIYEIK	95.0%	66.2	20.4	2	0	0	2	1,337.74
78 kDa glucose-regulated protein	GRP78_HUMAN	HSPA5	72,317	100.00%	34	42	564	54.40%	AKFEELNMDLFR	95.0%	84.0	22.4	22	12	0	2	1,512.75
									ALSSQHQAR	95.0%	61.1	21.6	4	0	0	2	997.52
									DAGTIAGLNVMR	95.0%	106.0	23.6	19	0	0	2	1,217.63
									DNHLLGTFDLTGIPPAPR	95.0%	97.6	22.2	20	18	0	2	1,934.01
									EFFNGKEPSR	95.0%	54.1	22.4	3	0	0	2	1,210.59
									ELEEIVQPIISK	95.0%	73.3	18.9	26	0	0	2	1,397.79
									FEELNMDLFR	95.0%	49.5	20.8	7	0	0	2	1,329.62
									IEIESFYEGEDFSETLTR	95.0%	99.4	20.4	9	0	0	2	2,164.99
									IEWLESHQDADIEDFK	95.0%	97.4	20.9	5	4	0	2	1,974.91
									IEWLESHQDADIEDFKAK	95.0%	60.3	20.8	0	10	0	2	2,174.04
									IINEPTAAAIAYGLDK	95.0%	114.0	21.5	30	4	0	2	1,659.90
									IINEPTAAAIAYGLDKR	95.0%	110.0	19.3	21	19	0	2	1,816.00
									ITITNDQNR	95.0%	63.0	23.3	14	0	0	2	1,074.55
									ITPSYVAFTPEGER	95.0%	85.5	22.4	33	0	0	2	1,566.78
									KSDIDEIVLVGGSTR	95.0%	124.0	22.3	21	13	0	2	1,588.85
									KSQIFSTASDNQPTVTIK	95.0%	90.9	20.5	2	2	0	2	1,965.03
									KVTHAVVTVPAYFNDAQR	95.0%	29.0	20.0	0	2	4	2	2,016.07
									LSSEDKETMEK	95.0%	52.5	21.6	6	0	0	2	1,296.60
									LYGSAGPPPTGEEDTAEKDEL	95.0%	110.0	21.0	26	0	0	2	2,175.99
									MKETAEAYLGK	95.0%	59.6	22.0	9	0	0	2	1,256.62
									NELESYAYSLK	95.0%	77.4	22.1	22	0	0	2	1,316.64
									NQLTSNPENTVFDAK	95.0%	104.0	22.9	26	0	0	2	1,677.81
									NQLTSNPENTVFDAKR	95.0%	93.9	21.8	2	0	0	2	1,833.91
									RALSSQHQAR	95.0%	50.3	21.7	0	2	0	2	1,153.62
									SDIDEIVLVGGSTR	95.0%	77.2	22.6	10	0	0	2	1,460.76
									SQIFSTASDNQPTVTIK	95.0%	103.0	22.0	39	0	0	2	1,836.93
									STMKPVQK	95.0%	31.9	23.1	1	0	0	2	918.51
									TFAPEEISAMVLTK	95.0%	99.5	22.8	44	0	0	2	1,536.80
									TKPYIQVDIGGGQTK	95.0%	65.5	20.9	4	0	0	2	1,604.87
									TWNDPSVQQDIK	95.0%	71.4	22.4	24	0	0	2	1,430.69
									VEIIANDQGNR	95.0%	80.0	22.6	56	0	0	2	1,228.63
									VLEDSDLK	95.0%	30.6	23.9	1	0	0	2	918.48

									VLEDSDLKK	95.0%	42.3	23.4	6	0	0	2	1,046.57
									VMEHFIK	95.0%	33.3	24.2	1	0	0	2	903.48
									VTHAVVTVPAYFNDAQR	95.0%	94.0	22.2	9	27	0	2	1,887.97
									VYEGERPLTK	95.0%	53.5	22.7	15	0	0	2	1,191.64
Aminoacyl tRNA synthase complex-interacting multifunctional protein 1	AIMP1_HUMAN	AIMP1	34,335	100.00%	6	8	18	25.60%	GAEADQIIEYLK	95.0%	66.0	22.8	2	0	0	2	1,349.70
									IEILAPPNGSVPGDR	95.0%	47.3	21.7	2	0	0	2	1,534.82
									ITFDAFPGEPDKELNPK	95.0%	62.8	22.4	2	2	0	2	1,917.96
									KQQSIAGSADSKPIDVSR	95.0%	54.0	21.0	0	2	0	2	1,886.99
									QQSIAGSADSKPIDVSR	95.0%	94.7	22.3	1	3	0	2	1,758.90
									TVVSGLVNHVPLEQMQNR	95.0%	52.9	21.2	0	4	0	2	2,021.06
Peroxiredoxin-2	PRDX2_HUMAN	PRDX2	21,874	100.00%	7	8	42	35.90%	ATAVVDGAFK	95.0%	40.6	22.9	4	0	0	2	978.53
									EGGLGPLNIPLLADVTR	95.0%	80.7	16.3	10	0	0	2	1,734.98
									GLFIIDGK	95.0%	33.1	18.1	2	0	0	2	862.50
									KEGGLGPLNIPLLADVTR	95.0%	86.1	14.9	5	11	0	2	1,863.07
									LSEDYGVLK	95.0%	43.1	22.7	4	0	0	2	1,023.54
									QITVNDLPVGR	95.0%	82.9	21.0	23	0	0	2	1,211.68
									SVDEALR	95.0%	44.8	24.9	2	0	0	2	789.41
									TDEGIAYR	95.0%	47.9	21.2	4	0	0	2	924.44
Gamma-glutamyl hydrolase	GGH_HUMAN	GGH	35,948	100.00%	7	9	56	26.10%	FFNVLTTNTDGK	95.0%	72.1	22.1	10	0	0	2	1,356.68
									KPIIGILMQK	95.0%	33.4	15.1	2	2	0	2	1,156.71
									LDLTEKDYEILFK	95.0%	62.3	21.0	8	13	0	2	1,626.86
									NLDGISHAPNAVK	95.0%	78.0	22.2	11	0	0	2	1,335.70
									SINGILFPGGSVDLR	95.0%	46.5	20.8	7	0	0	2	1,544.84
									YLESAGAR	95.0%	32.3	23.2	1	0	0	2	866.44
									YPVYGVQWHPEK	95.0%	33.0	22.8	0	2	0	2	1,502.74
Enoyl-CoA hydratase, mitochondrial	ECHM_HUMAN	ECHS1	31,370	100.00%	4	4	11	21.70%	ALNALCDGLIDELNQALK	95.0%	141.0	21.8	2	0	0	2	1,971.02
									AQFAQPEILIGTIPGAGGTQR	95.0%	91.7	19.1	4	0	0	2	2,125.14
									ICPVETLVEEAIQCAEK	95.0%	97.4	21.7	4	0	0	2	1,988.97
									ISAQDAK	95.0%	41.4	25.4	1	0	0	2	732.39
26S proteasome non-ATPase regulatory subunit 12	PSD12_HUMAN	PSMD12	52,888	100.00%	2	2	2	4.82%	DPNNLLNDWSQK	95.0%	51.9	21.3	1	0	0	2	1,443.69
									LNSLMSLVNK	95.0%	31.6	21.9	1	0	0	2	1,118.62
CD166 antigen	CD166_HUMAN	ALCAM	65,086	100.00%	13	14	95	25.20%	ADIQMPFTCSVTYYGPSGQK	95.0%	87.3	19.4	5	0	0	2	2,266.02
									ALFLETEQLK	95.0%	74.0	20.7	8	0	0	2	1,191.66
									ALFLETEQLKK	95.0%	37.8	17.7	3	0	0	2	1,319.76
									EMDPVTQLYTMTSTLEYK	95.0%	109.0	21.0	29	0	0	2	2,166.00
									FVCMLVTEDNVFEAPTIVK	95.0%	86.3	22.1	4	0	0	2	2,228.10
									LDVPQNLMFGK	95.0%	59.8	22.4	11	0	0	2	1,261.66
									QIGDALPVSCTISASR	95.0%	103.0	22.7	5	0	0	2	1,674.85
									SSNTYTLTDVR	95.0%	67.4	21.8	16	0	0	2	1,256.61
									SSNTYTLTDVRR	95.0%	40.5	22.5	1	0	0	2	1,412.71
									SVQYDDVPEYK	95.0%	31.7	20.6	1	0	0	2	1,342.62
									SVQYDDVPEYKDR	95.0%	44.5	21.2	2	0	0	2	1,613.74
									VFKQPSKPEIVSK	95.0%	37.2	17.6	0	3	0	2	1,486.86
									YEKPDGSPVFIAFR	95.0%	48.8	22.3	2	5	0	2	1,625.83
UMP-CMP kinase	KCY_HUMAN	CMPK1	22,205	100.00%	8	9	32	55.60%	EMDQTMAANAQK	95.0%	57.4	18.9	2	0	0	2	1,337.58
									FLIDGFPR	95.0%	58.9	22.0	11	0	0	2	964.53
									IQTYLQSTKPIIDLYEEMGK	95.0%	46.0	20.9	0	6	0	2	2,386.22
									KNPDSQYGELIEK	95.0%	72.9	23.1	2	0	0	2	1,520.76
									MKPLVVFVLGGPGAGK	95.0%	71.4	16.8	1	4	0	2	1,585.91
									NQDNLQGWNK	95.0%	36.0	20.6	2	0	0	2	1,216.57
									SVDEVFDEVVQIFDKEG	95.0%	39.3	21.6	1	0	0	2	1,954.93

									YGYTHLSAGELLR	95.0%	29.9	22.7	0	3	0	2	1,479.76
Glucosamine-6-phosphate isomerase 1	GNPI1_HUMAN	GNPDA1	32,651	100.00%	6	7	18	30.10%	AIEEGVNHMWTVSAFQQHPR	95.0%	32.0	21.8	0	2	0	2	2,353.11
									LVDPLYSIK	94.9%	30.3	16.1	1	0	0	2	1,047.61
									TFNMDEYVGLPR	95.0%	75.3	20.9	8	0	0	2	1,457.67
									TLAMDTILANAR	95.0%	93.6	23.3	2	0	0	2	1,289.69
									VPTMALTVGVGTVMDAR	95.0%	91.8	22.6	2	1	0	2	1,749.89
									YFTLGLPTGSTPLGCYK	95.0%	72.6	22.0	2	0	0	2	1,874.94
Galactocerebrosidase	GALC_HUMAN	GALC	77,019	99.90%	2	2	3	4.09%	EFDGIGAVSGGGATSR	95.0%	70.7	22.0	1	0	0	2	1,480.70
									SLWTDIPVNFPK	95.0%	37.7	22.1	2	0	0	2	1,416.75
Eukaryotic translation initiation factor 3 subunit E	EIF3E_HUMAN	EIF3E	52,205	100.00%	5	5	9	15.30%	LGHVVMGNNAVSPYQQVIEK	95.0%	32.0	21.4	0	2	0	2	2,199.12
									LKETIDNNSVSSPLQSLQQR	95.0%	39.6	20.6	0	2	0	2	2,257.18
									LNMTPEEAER	95.0%	50.1	18.9	2	0	0	2	1,205.55
									MLFDYLADK	95.0%	38.9	20.9	2	0	0	2	1,131.54
									QEYLDTLYR	95.0%	33.3	21.8	1	0	0	2	1,200.59
45 kDa calcium-binding protein	CAB45_HUMAN	SDF4	41,789	100.00%	12	14	73	31.20%	AVDPDGDGHVSWDEYK	95.0%	90.1	17.5	4	0	0	2	1,789.77
									DLGGFDEDAEPR	95.0%	82.3	16.0	13	0	0	2	1,320.57
									DLGGFDEDAEPRR	95.0%	39.9	21.2	1	0	0	2	1,476.67
									EVADAIR	95.0%	31.5	23.5	1	0	0	2	773.42
									GFHQEVFLGK	95.0%	40.3	24.2	2	0	0	2	1,161.61
									LEMDGHLNR	95.0%	51.8	19.9	4	2	0	2	1,100.52
									LMVIFSK	95.0%	33.1	20.2	1	0	0	2	853.49
									QMIAVADENQNHHLEPEEVLK	95.0%	48.2	22.5	0	7	0	2	2,444.19
									TAEHFQEAMEESK	95.0%	78.9	17.8	15	8	0	2	1,536.66
									VDVNTDR	95.0%	45.7	23.3	11	0	0	2	818.40
									VDVNTDRK	95.0%	34.0	23.4	3	0	0	2	946.50
									YSEFFTGSK	95.0%	32.3	21.2	1	0	0	2	1,065.49
Plasminogen activator inhibitor 1	PAI1_HUMAN	SERPINE1	45,042	100.00%	17	22	726	48.30%	ELMGPWNKDEISTTDAIFVQR	95.0%	45.4	20.8	0	30	0	2	2,466.20
									FIINDWVK	95.0%	51.7	22.5	24	0	0	2	1,034.57
									FSLETEVDLR	95.0%	55.6	23.2	21	0	0	2	1,208.62
									GAVDQLTR	95.0%	58.5	23.4	19	0	0	2	859.46
									GMAPALR	95.0%	35.2	25.7	3	0	0	2	731.39
									GMISNLLGK	95.0%	54.8	21.4	41	0	0	2	932.52
									HNPTGTVLFMGQVMEP	95.0%	89.0	21.1	113	0	0	2	1,789.83
									IDDKGMAPALR	95.0%	57.9	23.9	13	9	0	2	1,202.62
									KPLENLGMTDMFR	95.0%	65.8	23.2	34	24	0	2	1,551.77
									LVQGFMPHFFR	95.0%	47.5	23.0	13	3	0	2	1,394.70
									MAPEEIIMDRPFLFVVR	95.0%	36.6	21.8	0	17	0	2	2,095.07
									QFQADFTSLSDQEPLHVAQALQK	95.0%	90.9	21.1	1	130	0	2	2,601.30
									QVDFSEVER	95.0%	80.7	22.3	52	0	0	2	1,108.53
									SDGSTVSVPMMAQTNK	95.0%	60.6	20.1	57	0	0	2	1,684.75
									TPFPDSSTHR	95.0%	49.4	20.6	29	38	0	2	1,144.54
									VFQQVAQASK	95.0%	78.7	22.3	53	0	0	2	1,105.60
									VFQQVAQASKDR	95.0%	50.6	22.8	2	0	0	2	1,376.73
Ras-related protein Ral-A	RALA_HUMAN	RALA	23,549	100.00%	2	2	2	14.10%	AEQWNVNYVETSAK	95.0%	57.4	21.9	1	0	0	2	1,638.78
									VKEDENVPFLLVGNK	95.0%	33.8	20.4	1	0	0	2	1,700.92
Peroxiredoxin-6	PRDX6_HUMAN	PRDX6	25,018	100.00%	8	11	38	41.50%	DGDSVMVLPTIPEEEAK	95.0%	54.7	22.0	5	0	0	2	1,829.88
									ELAILLGMLDPAEKDEK	95.0%	67.6	21.2	5	2	0	2	1,885.00
									LPFPIIDDR	95.0%	59.3	20.9	8	0	0	2	1,085.60
									LSILYPATTGR	95.0%	59.0	19.5	3	0	0	2	1,191.67
									PGGLLLGDVAPNFEANTTVGR	95.0%	77.2	20.3	4	2	0	1	2,098.09
									VATPVDWKDGDSVMVLPTIPEEEAK	95.0%	35.6	21.1	0	2	0	2	2,742.36

									VVFVFGPDK	95.0%	30.5	21.8	1	0	0	2	1,007.56
									VVFVFGPDKK	95.0%	41.2	18.9	4	2	0	2	1,135.65
Pyridoxine-5'-phosphate oxidase	PNPO_HUMAN	PNPO	29,970	100.00%	3	3	4	12.30%	EAFEETHLTSLDPVK	95.0%	58.2	22.0	2	0	0	2	1,715.85
									FFTNFESR	95.0%	52.2	21.5	1	0	0	2	1,047.49
									GEEDWLYER	95.0%	33.7	19.0	1	0	0	2	1,196.52
Complement C1s subcomponent	C1S_HUMAN	C1S	76,666	100.00%	7	7	22	13.50%	EDTPNSVWEPAK	95.0%	42.2	21.1	3	0	0	2	1,372.64
									EPTMYVGSTSVQTSR	95.0%	78.8	21.6	3	0	0	2	1,642.77
									GDSGGAFAVQDPNDKTK	95.0%	45.7	24.1	1	0	0	2	1,706.80
									SDFSNEER	95.0%	46.5	14.9	4	0	0	2	983.41
									SSNNPHSPIVEEFQVPYNK	95.0%	31.0	21.2	0	3	0	2	2,186.05
									TMQENSTPRED	95.0%	41.0	16.1	3	0	0	2	1,323.55
									TNFDNDIALVR	95.0%	68.8	23.5	5	0	0	2	1,277.65
Vesicle-associated membrane protein-associated protein B/C	VAPB_HUMAN	VAPB	27,211	100.00%	3	3	3	8.64%	FRGPFTDVVTTNLK	95.0%	29.8	20.4	0	1	0	2	1,594.86
									GPFTDVVTTNLK	95.0%	59.7	22.0	1	0	0	2	1,291.69
									LGNPTDR	95.0%	32.6	20.9	1	0	0	2	772.40
Cytoplasmic dynein 1 intermediate chain 2	DC1I2_HUMAN	DYNC1I2	71,438	100.00%	4	4	8	9.25%	ADAEEEAATR	95.0%	74.7	18.3	2	0	0	2	1,062.47
									DLEDKEGEIQAGAK	95.0%	65.3	22.4	2	0	0	2	1,502.73
									EAVAPVQEESDLEK	95.0%	55.6	21.6	2	0	0	2	1,543.75
									SVSTPSEAGSQDSGDGAVGSR	95.0%	103.0	20.0	2	0	0	2	1,950.86
Staphylococcal nuclease domain-containing protein 1	SND1_HUMAN	SND1	101,981	100.00%	16	18	44	23.50%	DTNGENIAESLVAEGLATR	95.0%	98.2	21.8	1	2	0	2	1,959.96
									DTPDEPWAFPAR	95.0%	34.9	19.3	2	0	0	2	1,401.64
									DYVAPTANLDQK	95.0%	45.8	23.5	2	0	0	2	1,334.66
									EADGSETPEPFAAEAK	95.0%	79.3	19.2	4	0	0	2	1,648.73
									GDVGLGLVK	95.0%	35.3	20.3	1	0	0	2	857.51
									LGTLSPAFSTR	95.0%	37.3	21.8	2	0	0	2	1,149.63
									NDIASHPPVEGSYAPR	95.0%	69.0	21.6	2	0	0	2	1,709.82
									NLPGLVQEGEPFSEEATLFTK	95.0%	88.1	21.7	6	0	0	2	2,306.16
									QFLPFLQR	95.0%	39.2	20.3	6	0	0	2	1,048.59
									QINLSNIR	95.0%	40.3	21.8	2	0	0	2	957.55
									SLLSAEEAAK	95.0%	37.2	24.0	2	0	0	2	1,018.54
									SSHYDELLAAEAR	95.0%	46.5	22.0	2	1	0	2	1,461.70
									TDAVDSVVR	95.0%	34.4	24.7	1	0	0	2	961.50
									VITEYLNAQESAK	95.0%	63.2	22.1	2	0	0	2	1,465.75
									VMQVLNADAIVVK	95.0%	76.3	19.6	4	0	0	2	1,415.79
									VNVTVDYIRPASPATETVPAFSER	95.0%	42.2	21.1	0	2	0	2	2,619.34
Heme-binding protein 2	HEBP2_HUMAN	HEBP2	22,858	100.00%	4	5	24	23.90%	APEDAGPQPGSYEIR	95.0%	78.0	21.9	4	0	0	2	1,586.75
									NNEVWLIQK	95.0%	55.5	22.8	4	0	0	2	1,143.62
									NQEQLLTLASILR	95.0%	108.0	18.5	11	1	0	2	1,498.86
									VYYTAGYNSPVK	95.0%	75.2	22.6	4	0	0	2	1,361.67
Latent-transforming growth factor beta-binding protein 2	LTBP2_HUMAN	LTBP2	195,025	100.00%	7	7	29	4.78%	EQDAPVAGLQPVER	95.0%	52.8	23.3	3	0	0	2	1,508.77
									GAGGQSMSEAPTGDHAPAPTR	95.0%	58.8	19.7	0	19	0	2	2,010.89
									QICCCSR	95.0%	34.2	13.0	1	0	0	2	983.39
									SSAAGEGTLAR	95.0%	59.4	22.8	3	0	0	2	1,019.51
									SSGALPGPAER	95.0%	32.1	22.0	1	0	0	2	1,041.53
									STPLGQQQPAPR	95.0%	33.9	22.4	1	0	0	2	1,279.68
									VTNDVCSEPLR	95.0%	36.3	22.0	1	0	0	2	1,289.62
Protein CYR61	CYR61_HUMAN	CYR61	42,008	100.00%	4	4	14	13.10%	ELGFDASEVELTR	95.0%	98.3	22.6	8	0	0	2	1,465.72
									GLECNFGASSTALK	95.0%	74.0	22.0	4	0	0	2	1,454.70
									ILYNPLQGQK	95.0%	35.4	21.9	1	0	0	2	1,173.66
									IYQNGESFQPNCK	95.0%	73.1	20.5	1	0	0	2	1,584.71
Chloride intracellular channel protein	CLIC1_HUMAN	CLIC1	26,905	100.00%	14	14	149	56.80%	FLDGNELTLADCNLLPK	95.0%	72.0	21.8	7	0	0	2	1,932.97

1									FSAYIK	95.0%	34.2	19.5	4	0	0	2	728.40
									GFTIPEAFR	95.0%	44.0	22.1	4	0	0	2	1,037.54
									GVTFNVTTVDTK	95.0%	101.0	21.6	21	0	0	2	1,281.67
									GVTFNVTTVDTKR	95.0%	35.3	21.1	1	0	0	2	1,437.77
									IGNCPFSQR	95.0%	53.3	23.3	6	0	0	2	1,078.51
									KFLDGNELTLADCNLLPK	95.0%	41.1	21.5	1	0	0	2	2,061.07
									LAALNPESNTAGLDIFAK	95.0%	110.0	21.5	50	0	0	2	1,844.98
									LFMVLWLK	95.0%	52.5	18.3	9	0	0	2	1,065.62
									LHIVQVVCK	95.0%	47.3	17.9	4	0	0	2	1,095.64
									NSNPALNDNLEK	95.0%	78.8	22.4	27	0	0	2	1,328.64
									VLDNYLTSPLPEEVDETSAEDEGVSQR	95.0%	44.2	19.3	0	7	0	2	2,992.39
									VLDNYLTSPLPEEVDETSAEDEGVSQRK	95.0%	51.0	20.1	0	2	0	2	3,120.49
									YLSNAYAR	95.0%	45.4	20.3	6	0	0	2	957.48
Basal cell adhesion molecule	BCAM_HUMAN	BCAM	67,386	100.00%	15	18	174	28.70%	AGAAGTAEATAR	95.0%	104.0	23.2	36	0	0	2	1,046.52
									EGDEVTLICSAR	95.0%	54.6	22.2	3	0	0	2	1,349.64
									EGDTVQLLCR	95.0%	48.8	22.8	2	0	0	2	1,190.58
									GDGSPSPEYTLFR	95.0%	63.1	21.9	13	0	0	2	1,425.67
									GRSPPYQLDSQGR	95.0%	43.0	22.9	2	2	0	2	1,460.72
									LASAEMQGSELQVTMHDTR	95.0%	34.5	20.7	0	4	0	2	2,119.98
									LEVPVEMNPEGYMTSR	95.0%	86.6	20.2	16	0	0	2	1,883.85
									LNVFAKPEATEVSPNK	95.0%	92.1	21.4	8	7	0	2	1,743.93
									LSVPPLVEVMR	95.0%	46.1	18.3	6	0	0	2	1,239.71
									LSWSQLGGSPAEPIPGR	95.0%	85.8	21.6	10	1	0	2	1,751.91
									SPPYQLDSQGR	95.0%	63.1	22.9	14	0	0	2	1,247.60
									TAEIEPK	95.0%	37.9	21.9	4	0	0	2	787.42
									VAYLDPLELSEGK	95.0%	61.4	22.5	28	0	0	2	1,433.75
									VEDYDAADDVQLSK	95.0%	129.0	20.3	14	0	0	2	1,567.71
									VTSALSR	95.0%	37.8	22.2	4	0	0	2	733.42
Transgelin-2	TAGL2_HUMAN	TAGLN2	22,374	100.00%	12	15	86	67.30%	DDGLFSGDPNWFPK	95.0%	58.5	20.0	6	0	0	2	1,594.72
									DDGLFSGDPNWFPKK	95.0%	31.6	22.2	1	1	0	2	1,722.81
									DGTVLCELINALYPEGQAPVK	95.0%	72.6	20.5	1	0	0	2	2,287.16
									DGTVLCELINALYPEGQAPVKK	95.0%	48.4	21.1	1	4	0	2	2,415.26
									GASQAGMTGYGMPR	95.0%	71.7	18.1	14	0	0	2	1,415.60
									GPAYGLSR	95.0%	42.1	25.0	5	0	0	2	820.43
									IQASTMAFK	95.0%	41.1	21.6	2	0	0	2	1,012.51
									NFSDNQLQEGK	95.0%	75.7	21.0	10	0	0	2	1,279.59
									NVIGLQMGTNR	95.0%	62.2	23.6	6	0	0	2	1,202.63
									QMEQISQFLQAAER	95.0%	95.1	22.4	8	8	0	2	1,694.82
									TLMNLGGLAVAR	95.0%	86.6	21.5	11	0	0	2	1,215.69
									YGINTTDIFQTVDLWEGK	95.0%	111.0	22.3	8	0	0	2	2,100.03
Exportin-T	XPOT_HUMAN	XPOT	109,949	100.00%	3	3	6	4.26%	DLQEFIPLINQITAK	95.0%	39.8	18.7	1	0	0	2	1,742.97
									LAQVSPELLLASVR	95.0%	52.3	14.3	4	0	0	2	1,495.88
									NAQEALQAIETK	95.0%	45.2	22.9	1	0	0	2	1,315.69
1,4-alpha-glucan-branching enzyme	GLGB_HUMAN	GBE1	80,445	100.00%	4	4	7	8.26%	IVLDSDAAEYGGHQR	95.0%	113.0	22.1	2	0	0	2	1,630.78
									LLEIDPYLKPYAVDFQR	95.0%	36.1	20.4	0	2	0	2	2,080.11
									VALILQNVDLPN	95.0%	42.7	17.9	1	0	0	2	1,308.75
									YGWLAAPQAYVSEK	95.0%	48.1	22.3	2	0	0	2	1,582.79
Eukaryotic translation initiation factor 3 subunit L	EIF3L_HUMAN	EIF3L	66,711	100.00%	7	7	11	17.00%	LAGFLDLTEQEFR	95.0%	72.0	22.5	2	0	0	2	1,538.79
									QLEVYTSGGDPESVAGEYGR	95.0%	110.0	20.6	1	0	0	2	2,113.97
									QYEQQTYQVIPEVIK	95.0%	55.9	21.8	2	0	0	2	1,865.97
									VFSDEVQQQAQLSTIR	95.0%	59.4	22.2	1	0	0	2	1,848.95

									VSGGPSLEQR	95.0%	52.7	21.4	2	0	0	2	1,029.53
									VYEIQDIYENSWTK	95.0%	82.2	23.2	2	0	0	2	1,787.85
									VYELQASR	95.0%	47.2	22.7	1	0	0	2	965.51
Exportin-2	XPO2_HUMAN	CSE1L	110,404	100.00%	10	10	17	14.30%	ALTLPGSSENEYIMK	95.0%	62.9	21.8	2	0	0	2	1,668.82
									DLEGSDIDTR	95.0%	47.8	20.8	2	0	0	2	1,120.51
									EHDPVGQMVNNPK	95.0%	46.3	21.6	2	0	0	2	1,480.69
									GSNTIASAAADKIPGLLGVFQK	95.0%	30.8	16.9	0	1	0	2	2,158.19
									LLQTDDEEEAGLLELLK	95.0%	94.2	21.1	2	0	0	2	1,929.01
									LVLDAFALPLTNLFK	95.0%	32.3	13.2	1	0	0	2	1,674.98
									QLSDAISIIGR	95.0%	58.8	21.4	2	0	0	2	1,172.66
									SANVNEFPVLK	95.0%	43.9	23.0	2	0	0	2	1,217.65
									TGNIPALVR	95.0%	40.5	18.0	1	0	0	2	940.56
									YGALALQEIFDGIQPK	95.0%	83.9	20.7	2	0	0	2	1,762.94
Neutrophil gelatinase-associated lipocalin	NGAL_HUMAN	LCN2	22,571	100.00%	9	11	135	51.00%	ELTSELKENFIR	95.0%	73.1	21.1	8	5	0	2	1,478.79
									ITLYGR	95.0%	37.8	19.7	7	0	0	2	722.42
									MYATIYELK	95.0%	54.7	22.4	12	0	0	2	1,147.57
									MYATIYELKEDK	95.0%	64.8	22.8	6	0	0	2	1,503.74
									SYPGLTSYLVR	95.0%	79.9	22.0	17	0	0	2	1,255.67
									TFVPGCQPGEFTLGNIK	95.0%	57.1	22.3	8	0	0	2	1,864.93
									VPLQQNFQDNQFQGK	95.0%	91.3	23.0	33	2	0	2	1,790.88
									VVSTNYNQHAMVFFK	95.0%	58.1	22.5	8	0	0	2	1,784.88
									WYVVGLAGNAILR	95.0%	65.1	19.1	29	0	0	2	1,431.81
Serpin B7	SPB7_HUMAN	SERPINB7	42,888	100.00%	4	4	9	11.30%	ADLSGIASGGR	95.0%	46.0	22.8	1	0	0	2	1,003.52
									LTFQNLMEWTNPR	95.0%	51.4	22.9	2	0	0	2	1,665.81
									QLPQSTLFR	95.0%	49.0	22.3	4	0	0	2	1,089.61
									YVEVFFPQFK	95.0%	42.8	24.5	2	0	0	2	1,303.67
RNA-binding protein 8A	RBM8A_HUMAN	RBM8A	19,871	99.50%	2	2	5	17.20%	GYTLVEYETYK	95.0%	60.7	22.6	2	0	0	2	1,365.66
									MREDYDSVEQDGDEPGPQR	95.0%	51.5	14.9	0	3	0	2	2,238.92
Exportin-7	XPO7_HUMAN	XPO7	123,895	99.50%	2	2	3	2.48%	LYGDDALDNALQTFIK	95.0%	66.5	22.1	2	0	0	2	1,796.91
									NSIVNSQPPEK	95.0%	32.5	21.7	1	0	0	2	1,212.62
Coatomer subunit gamma	COPG_HUMAN	COPG	97,701	100.00%	5	5	9	8.35%	ELAPAVSVLQLFCSSPK	95.0%	42.7	21.4	1	0	0	2	1,845.98
									QEIFQEQLAAVPEFR	95.0%	38.3	22.1	2	0	0	2	1,804.92
									SIATLAITTLLK	95.0%	65.3	11.1	1	0	0	2	1,244.78
									SSPEPVALTESETEYVIR	95.0%	109.0	22.6	4	0	0	2	2,006.99
									TLEEAVGNIVK	95.0%	34.7	22.5	1	0	0	2	1,172.65
Midkine	MK_HUMAN	MDK	15,567	99.50%	2	2	4	11.90%	EFGADCK	95.0%	36.8	16.0	2	0	0	2	826.34
									YNAQCQETIR	95.0%	42.4	21.0	2	0	0	2	1,282.59
Polypeptide N-acetylgalactosaminyltransferase 1	GALT1_HUMAN	GALNT1	64,202	100.00%	7	7	17	14.70%	ATEEDSQVPSIR	95.0%	56.5	21.5	3	0	0	2	1,331.64
									ATPYTFPGGTGQIINK	95.0%	41.7	22.8	2	0	0	2	1,664.87
									GNQLWEYDPVK	95.0%	55.5	24.1	2	0	0	2	1,348.65
									HYFSLGEIR	95.0%	39.6	22.9	2	0	0	2	1,121.57
									NFFYIISPGVTK	95.0%	63.2	21.4	2	0	0	2	1,385.75
									TPTMAGGLFSIDR	95.0%	64.6	22.8	4	0	0	2	1,365.68
									VDYGDISSR	95.0%	37.7	21.0	2	0	0	2	1,011.47
AP-1 complex subunit beta-1	AP1B1_HUMAN	AP1B1	104,621	99.50%	2	2	2	6.74%	DIPNENEAQFQIR	95.0%	57.6	22.5	1	0	0	2	1,573.76
									LASQANIAQVLAELK	95.0%	58.9	17.6	2	0	0	2	1,568.90
									LQSSNIFTVAK	95.0%	44.5	21.2	1	0	0	2	1,207.67
									MEPLNNLQVAVK	95.0%	46.1	22.3	4	0	0	2	1,371.73
									NVEGQDMLYQSLK	95.0%	53.5	22.3	2	0	0	2	1,540.73
Ubiquitin carboxyl-terminal hydrolase isozyme L3	UCHL3_HUMAN	UCHL3	26,165	100.00%	4	5	18	27.40%	FLEESVSMSPEER	95.0%	89.0	20.4	5	0	0	2	1,555.70
									SQGQDVTSSVYFMK	95.0%	75.7	20.8	3	0	0	2	1,576.73

									VTHETSAHEGQTEAPSIDEK	95.0%	96.7	20.9	2	2	0	2	2,166.00
									WLPLEANPEVTNQFLK	95.0%	72.3	20.7	6	0	0	2	1,899.00
Putative tropomyosin alpha-3 chain-like protein	TPM3L_HUMAN	---	26,252	100.00%	4	5	15	24.70%	AELAESR	95.0%	35.7	24.2	1	0	0	2	775.40
									HIAEEADR	95.0%	38.5	22.0	4	0	0	2	940.45
									IQVLQQQADDAEER	95.0%	108.0	22.1	9	0	0	2	1,642.80
									IQVLQQQADDAEERAER	95.0%	59.9	22.0	1	3	0	2	1,998.98
									LKEAETR	95.0%	42.4	24.5	6	0	0	2	846.47
									LMDQNLK	95.0%	43.1	23.8	1	0	0	2	877.45
									MELQEIQLK	95.0%	56.2	23.9	5	0	0	2	1,131.61
Ribosome maturation protein SBDS	SBDS_HUMAN	SBDS	28,746	100.00%	3	3	3	12.80%	DLDEVLQTHSVFVNVSK	95.0%	38.2	21.9	0	1	0	2	1,929.99
									GEVQVSDKER	95.0%	35.4	24.0	1	0	0	2	1,146.58
									SGVEKDLDEVLQTHSVFVNVSK	95.0%	33.0	21.0	0	1	0	2	2,430.25
Cleavage stimulation factor subunit 1	CSTF1_HUMAN	CSTF1	48,341	100.00%	3	3	4	8.58%	LGMENDDTAVQYAIGR	95.0%	68.0	21.9	2	0	0	2	1,752.82
									NLLSLGHNNIVR	95.0%	36.0	19.2	1	0	0	2	1,349.77
									YTGAGLSGR	95.0%	50.3	22.2	1	0	0	2	881.45
Leucine-rich repeat flightless-interacting protein 1	LRRF1_HUMAN	LRRFIP1	89,235	100.00%	3	3	11	4.70%	EFTNQEAAEPK	95.0%	43.3	21.0	2	0	0	2	1,263.59
									IDGATQSSPAEPK	95.0%	45.4	21.7	6	0	0	2	1,300.64
									SAVEAQNEVTENPK	95.0%	84.8	21.3	3	0	0	2	1,515.73
Heat shock protein HSP 90-alpha	HS90A_HUMAN	HSP90AA1	84,645	100.00%	23	29	321	47.10%	ADLINNLGTIAK	95.0%	74.8	20.9	86	0	0	2	1,242.71
									ALLFVPR	95.0%	45.0	18.9	17	0	0	2	815.51
									APFDLFENR	95.0%	81.0	22.4	16	0	0	2	1,108.54
									APFDLFENRK	95.0%	34.0	22.8	1	0	0	2	1,236.64
									DNSTMGYMAAK	95.0%	73.2	16.0	11	0	0	2	1,204.50
									DQVANSAFVER	95.0%	78.7	22.9	32	0	0	2	1,235.60
									EDQTEYLEER	95.0%	49.1	18.8	12	0	0	2	1,311.57
									EGLELPEDEEEK	95.0%	58.8	20.1	3	0	0	2	1,416.64
									EGLELPEDEEEKK	95.0%	43.1	20.8	2	0	0	2	1,544.73
									EKYIDQEELNK	95.0%	55.1	23.3	4	0	0	2	1,408.70
									ELHINLIPNK	95.0%	34.5	20.0	1	0	0	2	1,190.69
									ELHINLIPNKQDR	95.0%	44.6	20.2	5	4	0	2	1,589.88
									ELISNSSDALDK	95.0%	80.1	23.3	18	0	0	2	1,291.64
									ELISNSSDALDKIR	95.0%	99.6	22.3	5	1	0	2	1,560.82
									EMLQQSK	95.0%	50.8	23.4	6	0	0	2	879.42
									GVVDSEDLPLNISR	95.0%	113.0	22.4	54	0	0	2	1,513.79
									HFSVEGQLEFR	95.0%	64.6	23.7	7	4	0	2	1,348.67
									HIYYITGETK	95.0%	43.0	22.1	11	0	0	2	1,224.63
									HLEINPDHSIIETLR	95.0%	85.7	21.8	10	42	0	2	1,786.95
									HNDDEQYAWESSAGGSFTVR	95.0%	133.0	17.3	7	54	0	2	2,255.96
									HSQFIGYPITLFVEK	95.0%	68.5	20.8	23	9	0	2	1,778.95
									HSQFIGYPITLFVEKER	95.0%	28.2	20.5	0	1	0	2	2,064.09
									IMKDILEK	95.0%	36.0	21.2	1	0	0	2	989.57
									IRYESLTDPSK	95.0%	47.5	22.3	2	0	0	2	1,308.68
									KHLEINPDHSIIETLR	95.0%	23.5	19.0	0	0	4	2	1,915.04
									KHSQFIGYPITLFVEK	95.0%	29.9	18.9	0	1	0	2	1,907.04
									LGIHEDSQNR	95.0%	49.0	23.1	6	9	0	2	1,168.57
									LSELLR	95.0%	44.8	23.2	17	0	0	2	730.45
									LVTSPCCIVTSTYGWTANMER	95.0%	94.9	20.9	3	0	0	2	2,462.12
									NPDDITNEEYGEFYK	95.0%	98.2	17.2	26	0	0	2	1,833.78
									RAPFDLFENR	95.0%	46.6	23.1	12	1	0	2	1,264.64
									SLTNDWEDHLAVK	95.0%	83.6	21.8	97	7	0	2	1,527.74
									TDTGEPMGR	95.0%	55.4	19.8	10	0	0	2	963.42

									TLTIVDTGIGMTK	95.0%	109.0	22.1	57	0	0	2	1,365.73
									TLVSVTK	95.0%	40.7	19.6	4	0	0	2	747.46
									VILHLK	95.0%	35.5	7.8	7	0	0	2	722.49
									VILHLKEDQTEYLEER	95.0%	69.3	22.0	7	102	0	2	2,015.05
									YESLTDPSK	95.0%	43.0	23.0	9	0	0	2	1,039.49
									YESLTDPSKLDSGK	95.0%	67.9	22.6	14	3	0	2	1,539.75
									YIDQEELNK	95.0%	60.6	23.1	21	0	0	2	1,151.56
									YYTSASGDEMVSLK	95.0%	74.2	19.8	37	0	0	2	1,566.70
Oligoribonuclease, mitochondrial	ORN_HUMAN	REXO2	26,816	99.90%	2	2	3	10.10%	ALDDISESIK	95.0%	59.6	23.8	2	0	0	2	1,090.56
									QPDELLDSMSDWCK	95.0%	43.1	19.6	1	0	0	2	1,723.73
Interleukin-1 receptor-like 1	ILRL1_HUMAN	IL1RL1	63,341	100.00%	8	11	118	18.20%	DEQGFSLFPVIGAPAQNEIK	95.0%	79.3	21.7	18	3	0	2	2,160.10
									DEQGFSLFPVIGAPAQNEIKEVEIGK	95.0%	48.1	19.8	0	8	0	2	2,815.45
									FLPAAVADSGIYTCIVR	95.0%	115.0	21.5	6	3	0	2	1,852.96
									IQQEEGQNQSFSNGLACLDMVLR	95.0%	53.7	20.8	2	1	0	2	2,653.24
									ITDFGEPR	95.0%	56.2	23.1	31	0	0	2	934.46
									QSWGLENEALIVR	95.0%	98.5	21.9	26	0	0	2	1,514.80
									SFTVKDEQGFSLFPVIGAPAQNEIK	95.0%	38.1	20.6	0	2	0	2	2,722.41
									VFASGQLLK	95.0%	61.7	18.2	18	0	0	2	962.57
T-complex protein 1 subunit zeta	TCPZ_HUMAN	CCT6A	58,007	100.00%	6	6	19	17.50%	ALQFLEEVK	95.0%	45.3	21.9	4	0	0	2	1,076.60
									DGNVLLHEMQIQHPTASLIAK	95.0%	27.8	20.5	0	1	0	2	2,331.21
									GIDPFSLDALSK	95.0%	37.9	22.9	2	0	0	2	1,262.66
									IITEGFEAAK	95.0%	46.1	21.8	4	0	0	2	1,078.58
									VATAQDDITGDGTTSNVLIIGELLK	95.0%	88.9	18.6	4	0	0	2	2,544.34
									VLAQNSGFDLQETLVK	95.0%	89.5	21.1	4	0	0	2	1,761.94
Cellular nucleic acid-binding protein	CNBP_HUMAN	CNBP	19,444	100.00%	4	5	6	25.40%	CGESGHLAK	95.0%	32.1	18.6	1	0	0	2	958.44
									CGETGHVAINCSK	95.0%	51.8	17.6	1	1	0	2	1,432.63
									GFQFVSSSLPDICYR	95.0%	48.8	22.3	2	0	0	2	1,775.84
									TSEVNCYR	95.0%	44.3	17.9	1	0	0	2	1,028.45
Gamma-synuclein	SYUG_HUMAN	SNCG	13,312	99.50%	2	3	4	13.40%	EDLRPSAPQQEGEASK	95.0%	37.3	21.5	0	1	0	2	1,741.84
									KEDLRPSAPQQEGEASK	95.0%	35.5	22.0	0	2	1	2	1,869.93
Plastin-3	PLST_HUMAN	PLS3	70,796	100.00%	16	20	80	38.60%	AESMLQQADK	95.0%	63.1	22.3	3	0	0	2	1,136.53
									ALENDPDCR	95.0%	37.8	16.5	3	0	0	2	1,089.46
									ANDDIIVNWVNR	95.0%	55.1	21.8	2	0	0	2	1,428.72
									AYFHLLNQIAPK	95.0%	43.1	21.0	0	4	0	2	1,414.78
									DGETLEELMK	95.0%	52.5	21.2	3	0	0	2	1,180.54
									HVIPMNPNTDDLFK	95.0%	32.8	21.9	0	2	0	2	1,656.81
									IDINMSGFNETDDLKR	95.0%	86.2	21.2	2	4	0	2	1,883.88
									IKVPVDWSK	95.0%	43.3	21.3	0	4	0	2	1,071.62
									INNFSADIKDSK	95.0%	61.2	22.8	4	0	0	2	1,351.69
									KLENCNYAVELGK	95.0%	63.0	22.8	2	0	0	2	1,537.77
									LSPEELLLR	95.0%	67.1	17.1	7	0	0	2	1,069.63
									MINLSVPDTIDER	95.0%	66.8	22.8	24	0	0	2	1,518.75
									NEALAALLR	95.0%	79.1	18.2	16	0	0	2	970.57
									QFVTPADVVSGNPK	95.0%	87.7	22.6	11	0	0	2	1,458.76
									RYTLNVLEDLGDGQK	95.0%	32.9	22.0	0	1	0	2	1,720.89
									SGNLTEDDKHNNAK	95.0%	48.6	20.6	1	2	0	2	1,542.72
									VYALPEDLVEVKPK	95.0%	60.2	18.7	4	2	0	2	1,599.90
									YAFVNWINK	95.0%	34.8	20.9	1	0	0	2	1,154.60
									YAVSMAR	95.0%	31.3	20.5	1	0	0	2	813.39
									YPALTKPENQDIDWTLLEGETR	95.0%	63.3	21.8	2	5	0	2	2,589.28
									YTLNVLEDLGDGQK	95.0%	90.0	22.3	7	0	0	2	1,564.79

Putative phospholipase B-like 1	PLBL1_HUMAN	PLBD1	63,240	100.00%	2	2	3	3.98%	QVIPETLLSWQR	95.0%	54.4	20.9	1	0	0	2	1,469.81
									TTGWGILEIR	95.0%	50.9	22.6	2	0	0	2	1,145.63
ATP synthase subunit beta,	ATPB_HUMAN	ATP5B	56,543	100.00%	11	12	40	30.60%	AIAELGIYPAVDPLDSTSR	95.0%	73.2	21.7	4	0	0	2	1,988.03
mitochondrial									FTQAGSEVSALLGR	95.0%	87.2	22.1	4	0	0	2	1,435.75
									IGLFGGAGVGK	95.0%	45.8	20.4	6	0	0	2	975.56
									IMDPNIVGSEHYDVAR	95.0%	42.2	21.5	0	2	0	2	1,831.87
									IMNVIGEPIDER	95.0%	56.2	22.5	4	0	0	2	1,401.71
									IPSAVGYQPTLATDMGTMQER	95.0%	53.1	21.4	2	0	0	2	2,298.08
									TIAMDGTEGLVR	95.0%	60.0	22.9	3	0	0	2	1,278.64
									TVLIMELINNVAK	95.0%	76.2	19.4	2	0	0	2	1,473.84
									VALVYGQMNEPPGAR	95.0%	79.7	23.0	4	0	0	2	1,617.81
									VLDSGAPIKIPVGPETLGR	95.0%	62.1	14.0	2	5	0	2	1,919.10
									VVDLLAPYAK	95.0%	40.6	19.3	2	0	0	2	1,088.64
Creatine kinase U-type,	KCRU_HUMAN	CKMT1A	47,020	100.00%	5	5	10	13.70%	GTGGVDTAATGGVFDISNLDR	95.0%	106.0	22.2	2	0	0	2	2,022.97
mitochondrial									HTTDLDASK	95.0%	37.8	20.9	1	0	0	2	987.47
									LGYILTCPSNLGTGLR	95.0%	38.5	20.8	2	0	0	2	1,734.92
									RGTGGVDTAATGGVFDISNLDR	95.0%	34.8	22.2	0	1	0	2	2,179.07
									VVVDALSGLK	95.0%	65.1	20.3	4	0	0	2	1,000.60
Nodal modulator 1	NOMO1_HUMAN	NOMO1	134,308	100.00%	5	5	11	5.07%	DGENYVVLLDSTLPR	95.0%	53.8	22.3	3	0	0	2	1,690.87
									EQQLAEIEAR	95.0%	56.0	23.3	2	0	0	2	1,186.61
									IQSTVTQPGGK	95.0%	30.8	23.0	1	0	0	2	1,115.61
									SSIDSEPALVLGPLK	95.0%	86.9	20.6	4	0	0	2	1,525.85
									VQVMVPEAETR	95.0%	34.7	22.8	1	0	0	2	1,274.64
Lysosome-associated membrane	LAMP1_HUMAN	LAMP1	44,865	100.00%	3	3	17	8.63%	ALQATVGNSYK	95.0%	41.2	22.8	6	0	0	2	1,151.61
glycoprotein 1									FFLQGIQLNTILPDAR	95.0%	75.9	18.0	10	0	0	2	1,846.02
									TVESITDIR	95.0%	34.8	23.3	1	0	0	2	1,033.55
Arrestin domain-containing protein 1	ARRD1_HUMAN	ARRDC1	45,963	99.90%	2	2	4	5.08%	LGAPLPFR	95.0%	42.8	19.8	2	0	0	2	870.52
									VVYSPGEPLAGTVR	95.0%	52.2	21.1	2	0	0	2	1,444.78
Transforming growth factor beta-2	TGFB2_HUMAN	TGFB2	47,731	100.00%	2	2	13	5.80%	FAGIDGTSTYTSGDQK	95.0%	108.0	20.7	7	0	0	2	1,647.75
									IELYQILK	95.0%	43.8	14.0	6	0	0	2	1,019.61
Transformer-2 protein homolog	TRA2A_HUMAN	TRA2A	32,671	99.90%	2	2	8	8.16%	GFAFVYFER	95.0%	52.2	23.2	2	0	0	2	1,135.56
alpha									YGPLSGVNVVYDQR	95.0%	56.5	22.5	6	0	0	2	1,566.79
Peptidyl-prolyl cis-trans isomerase	FKBP2_HUMAN	FKBP2	15,632	100.00%	3	4	5	28.90%	KLVIPSELGYGER	95.0%	36.8	20.3	1	1	0	2	1,460.81
FKBP2									LEDGTEFDSSLPQNQPFVFSLGTGQVIK	95.0%	28.1	21.1	0	1	0	2	3,053.51
									LVIPSELGYGER	95.0%	63.2	22.3	2	0	0	2	1,332.72
Basement membrane-specific	PGBM_HUMAN	HSPG2	468,761	100.00%	75	85	619	20.90%	AFAHLQVPER	95.0%	42.8	21.8	4	4	0	2	1,167.63
heparan sulfate proteoglycan core									AGFFGDAMK	95.0%	32.8	17.2	2	0	0	2	943.43
protein									AGLSSGFIGCVR	95.0%	46.2	23.9	2	0	0	2	1,223.62
									AHSSAGQQVAR	95.0%	45.0	21.1	2	0	0	2	1,111.56
									AMDFNGILTIR	95.0%	35.9	22.6	4	0	0	2	1,266.65
									ASYAQQPAESR	95.0%	70.6	22.3	61	0	0	2	1,207.57
									ATATSCRPCPCPYIDASR	95.0%	54.8	19.4	0	2	0	2	2,082.92
									AVTLECVSAGEPR	95.0%	97.2	22.7	8	0	0	2	1,388.68
									CAPGYYGNPSQGQPCQR	95.0%	62.3	16.4	1	0	0	2	1,939.82
									CEQCQPGYYGDAQR	95.0%	49.0	11.8	2	0	0	2	1,731.69
									CESCAPGYEGNPIQPGGK	95.0%	104.0	17.2	2	0	0	2	1,920.82
									CKNNVVGR	95.0%	34.1	24.1	1	0	0	2	946.49
									CSATGSPTPTLEWTGGPGGQLPAK	95.0%	82.8	21.8	1	0	0	2	2,370.14
									DFISLGLQDGHLVFR	95.0%	77.6	20.9	15	8	0	2	1,716.91
									EDGRPVPSGTQQR	95.0%	35.9	21.9	3	4	0	2	1,426.70
									EGGQLPPGHSVQDGVLR	95.0%	30.4	23.2	0	1	0	2	1,745.89

EGGSLPPQAR	95.0%	41.3	22.0	12	0	0	2	1,011.52
EQAWQRPDGQPATR	95.0%	42.2	22.6	3	0	0	2	1,639.79
ESDQGAYTCEAMNAR	95.0%	74.1	10.8	4	0	0	2	1,702.68
ESIQASR	95.0%	37.6	24.7	6	0	0	2	790.41
EVSEAVVDTLESEYLK	95.0%	134.0	22.5	25	0	0	2	1,810.90
FDAGSGMATIR	95.0%	85.7	21.3	29	0	0	2	1,141.53
FSSGITGCVK	95.0%	31.7	21.6	1	0	0	2	1,055.52
FTVTQR	95.0%	38.1	21.1	3	0	0	2	751.41
GHTPTQPGALNQR	95.0%	49.5	23.1	4	18	0	2	1,376.70
GMVFGIPDGVLELVPQR	95.0%	53.5	20.7	4	0	0	2	1,842.98
GPSCQDCDTGYTR	95.0%	80.5	12.6	2	0	0	2	1,516.58
GSIQVDGEELVSGR	95.0%	70.8	22.5	32	0	0	2	1,445.72
GSVYIGGAPDVATLTGGR	95.0%	119.0	21.9	9	0	0	2	1,690.88
HCTSSSWSR	94.8%	30.2	15.6	1	0	0	2	1,107.46
HQIVGSR	95.0%	30.5	19.9	1	0	0	2	796.44
HQTHGSLLR	95.0%	37.7	23.6	0	12	0	2	1,048.57
IAHVELADAGQYR	95.0%	65.1	22.3	5	4	0	2	1,442.74
IESSSPTVVEGQTLDLNCVVAR	95.0%	108.0	21.2	4	0	0	2	2,374.19
IQVVVLSASDASPPPVK	95.0%	51.7	16.4	5	0	0	2	1,706.97
ITFRPDSADGMLLYNGQK	95.0%	33.3	22.1	0	1	0	2	2,042.00
LCNECADGSFHLSTR	95.0%	31.6	18.5	0	1	0	2	1,766.76
LDGSLPPDSR	95.0%	45.2	22.1	8	0	0	2	1,056.53
LEGDTLIIPR	95.0%	78.9	20.1	26	0	0	2	1,126.65
LGTVPQFPR	95.0%	53.6	22.3	12	0	0	2	1,014.57
LLQVTPADSGEYVCR	95.0%	77.3	21.6	6	0	0	2	1,707.84
LLSGPYFWSLPSR	95.0%	50.5	21.5	6	0	0	2	1,522.81
LPAVEPTDQAQYLCR	95.0%	94.2	22.3	5	0	0	2	1,760.86
LRSPVISIDPPSSTVQQGQDASFK	95.0%	73.1	20.6	0	5	0	2	2,557.33
LSGSHSQGVAYPVR	95.0%	45.3	22.2	5	2	0	2	1,457.75
LVSEDPINDGEWHR	95.0%	71.3	21.9	7	3	0	2	1,666.78
LYIFQASPADAGQYVCR	95.0%	91.1	21.9	8	0	0	2	1,958.94
LYQASPADSGEYVCR	95.0%	97.3	19.9	9	0	0	2	1,715.77
NLVLHSARPGAPPPQPLDLQHR	95.0%	36.0	18.0	0	2	2	2	2,413.32
NPDGCLK	95.0%	32.4	17.8	2	0	0	2	803.37
RGGSLPAR	95.0%	36.5	20.9	1	0	0	2	813.47
RGSIQVDGEELVSGR	95.0%	59.7	22.6	4	0	0	2	1,601.82
RPDGQPATR	95.0%	36.8	21.6	2	0	0	1	997.52
RVPGSPTNLANR	95.0%	36.5	20.4	2	0	0	2	1,281.70
SAEPLALGR	95.0%	34.3	20.4	3	0	0	2	913.51
SIEYSPQLEDAGSR	95.0%	111.0	21.7	16	0	0	2	1,551.73
SIVPQGGSHSLR	95.0%	29.1	21.4	0	2	0	2	1,237.67
SLPEVPETIELEVR	95.0%	90.9	20.7	9	0	0	2	1,610.86
SPAYTLVWTR	95.0%	83.2	22.1	10	0	0	2	1,193.63
SPGPNVAVNAK	95.0%	78.1	21.2	22	0	0	2	1,053.57
SPLPWQHR	95.0%	35.7	23.9	1	0	0	2	1,020.54
SPVISIDPPSSTVQQGQDASFK	95.0%	121.0	22.8	9	2	0	2	2,288.14
TQVHAGR	95.0%	35.3	20.0	1	0	0	2	768.41
TSTASGLLLWQGVEVGEAGQGK	95.0%	111.0	21.2	4	2	0	2	2,188.13
VAEGQTLDLK	95.0%	74.3	23.4	9	0	0	2	1,073.58
VDSYGGSLR	95.0%	48.1	22.1	8	0	0	2	953.47
VGSSLPGR	95.0%	58.7	22.7	20	0	0	2	772.43

									VISSGSVASYVTSPQGFQFR	95.0%	105.0	22.1	13	0	0	2	2,117.07
									VPGSPTNLANR	95.0%	35.8	22.9	1	0	0	2	1,125.60
									VQVSPER	95.0%	36.5	20.5	2	0	0	2	814.44
									VTSYGGELR	95.0%	43.7	21.8	6	0	0	2	981.50
									VTVTSEGGR	95.0%	59.1	24.2	15	0	0	2	905.47
									VVPYFTQTPYSFLPLPTIK	95.0%	40.1	17.3	1	0	0	2	2,211.21
									YELGSGLAVLR	95.0%	96.4	20.8	25	0	0	2	1,177.66
									YQLGSGEAR	95.0%	63.7	22.8	8	0	0	2	980.48
Plasma alpha-L-fucosidase	FUCO2_HUMAN	FUCA2	54,050	100.00%	3	3	18	8.35%	FDPTWESLDAR	95.0%	70.1	21.0	5	0	0	2	1,336.62
									FFNANQWADIFQASGAK	95.0%	102.0	21.7	5	0	0	2	1,914.91
									YEDFGPLFTAK	95.0%	56.2	22.1	8	0	0	2	1,287.63
Beta-1,4-galactosyltransferase 1	B4GT1_HUMAN	B4GALT1	43,903	100.00%	3	3	19	14.30%	ETMLSDGLNSLTYQVLDVQR	95.0%	111.0	21.7	11	0	0	2	2,298.13
									LPQLVGVSTPLQGGSNSAAAIGQSSGELR	95.0%	37.1	19.7	0	3	0	2	2,794.47
									VAIIIPFR	95.0%	35.5	15.8	5	0	0	2	928.60
Protein canopy homolog 2	CNPY2_HUMAN	CNPY2	20,635	100.00%	3	3	7	25.80%	ALVDELEWEIAQVDPK	95.0%	56.9	21.3	3	0	0	2	1,854.95
									INPDGSQSVVEVPYAR	95.0%	66.3	21.7	2	0	0	2	1,730.87
									SEAHLTELLEEICDR	95.0%	28.7	21.8	0	2	0	2	1,814.86
Quinone oxidoreductase PIG3	QORX_HUMAN	TP53I3	35,519	100.00%	3	3	4	11.40%	EVAKPSPGEGEVLLK	95.0%	45.9	19.1	1	0	0	2	1,552.86
									GSLITSLLR	95.0%	44.6	17.3	2	0	0	2	959.59
									MAGAIPLVTAGSQK	94.7%	30.1	21.5	1	0	0	2	1,343.74
T-complex protein 1 subunit delta	TCPD_HUMAN	CCT4	57,908	100.00%	11	12	23	27.60%	AFADAMEVIPSTLAENAGLNPISTVTELR	94.7%	26.3	20.6	0	1	0	2	3,046.54
									ALIAGGGAPEIELALR	95.0%	64.2	17.6	3	0	0	2	1,550.89
									AVADAIR	95.0%	32.9	24.0	1	0	0	2	715.41
									DALSDLALHFLNK	95.0%	77.7	21.9	2	1	0	2	1,456.78
									IDDVVNTR	95.0%	36.8	23.7	2	0	0	2	931.49
									IGLIQFCLSAPK	95.0%	35.8	20.8	2	0	0	2	1,346.75
									LVIEEAER	95.0%	32.2	21.2	1	0	0	2	958.52
									TAGINVR	95.0%	36.5	24.0	2	0	0	2	730.42
									TDMDNQIVVSDYAQMDR	95.0%	90.8	17.9	2	0	0	2	2,032.86
									VIDPATATSVDLR	95.0%	74.7	22.2	4	0	0	2	1,357.73
									VVSQYSSLLSPMSVNAVMK	95.0%	63.6	22.1	2	0	0	2	2,072.04
EH domain-containing protein 2	EHD2_HUMAN	EHD2	61,145	100.00%	2	2	4	4.24%	LEGHGLPANLPR	95.0%	33.8	21.9	0	2	0	2	1,273.70
									LFELEEQDLFR	95.0%	38.3	22.9	2	0	0	2	1,438.72
Secernin-1	SCRN1_HUMAN	SCRN1	46,364	100.00%	3	4	9	9.66%	AIIESDQEQGR	95.0%	55.7	22.5	4	0	0	2	1,245.61
									EPAAEIEALLGMDLVR	95.0%	69.1	21.5	3	1	0	2	1,742.90
									SIFKPFIFVDDVK	95.0%	43.4	19.7	1	0	0	2	1,554.86
Isopentenyl-diphosphate Delta-isomerase 1	IDI1_HUMAN	IDI1	26,302	100.00%	4	4	12	23.30%	AELGIPLEEVPPEEINYLTR	95.0%	59.7	20.8	4	0	0	2	2,282.19
									AFSVFLFNTENK	95.0%	64.8	22.3	2	0	0	2	1,416.72
									IIAATFLFK	95.0%	43.7	14.5	2	0	0	2	1,023.62
									NVTLNPDPNEIK	95.0%	52.7	22.0	4	0	0	2	1,353.70
Neutral alpha-glucosidase AB	GANAB_HUMAN	GANAB	106,858	100.00%	23	28	198	26.80%	AEKDEPGAWEETFK	95.0%	66.5	21.8	3	3	0	2	1,636.75
									DENSVELTMAEGPYK	95.0%	115.0	19.7	7	0	0	2	1,698.75
									DPAEGDGAQPEETPR	95.0%	109.0	19.6	6	0	0	2	1,568.68
									DPAEGDGAQPEETPRDGDKPEETQGK	95.0%	19.8	18.0	0	0	1	2	2,753.21
									FRIDELEPR	95.0%	38.5	23.5	1	1	0	2	1,174.62
									GLLEFEHQR	95.0%	59.9	21.9	6	0	0	2	1,128.58
									KLVAIVDPHIK	95.0%	54.8	8.5	0	7	0	2	1,232.77
									LDLLEDR	95.0%	44.5	22.3	2	0	0	2	873.47
									LKVTEGGEPYR	95.0%	49.0	22.9	2	2	0	2	1,248.66
									LSFQHDPETSVLVLR	95.0%	58.0	20.7	0	26	0	2	1,740.93
									LVAIVDPHIK	95.0%	60.2	14.6	5	0	0	2	1,104.68

									MMDYLQGSGETPQTDVR	95.0%	110.0	17.3	18	0	0	2	1,959.84
									NPEPELLVR	95.0%	46.4	20.7	6	0	0	2	1,066.59
									QYASLTGTQALPPLFSLGYHQSR	95.0%	69.6	20.2	0	14	0	2	2,535.30
									REPWLLPSQHNDIIR	95.0%	38.7	20.5	0	4	0	2	1,874.00
									SGGMERPFVLAR	95.0%	39.8	23.0	0	4	0	2	1,335.68
									SIRPGLSPYR	95.0%	35.9	21.7	1	0	0	2	1,145.64
									VPDVLVADPPIAR	95.0%	41.8	17.0	2	0	0	2	1,361.78
									VSQGSKDPAEGDGAQPEETPR	95.0%	93.9	20.8	6	36	0	2	2,154.99
									VTEGGEPYR	95.0%	42.2	21.9	13	0	0	2	1,007.48
									VVIIGAGKPAAVVLQTK	95.0%	66.8	3.0	5	8	0	2	1,664.05
									WYQMGAYQPFFR	95.0%	38.7	19.8	1	0	0	2	1,609.73
									YRVPDVLVADPPIAR	95.0%	43.3	17.4	0	8	0	2	1,680.94
Serine/threonine-protein phosphatase 2A catalytic subunit beta isoform	PP2AB_HUMAN	PPP2CB	35,557	100.00%	3	3	11	11.00%	ESNVQEVR	95.0%	44.6	22.5	3	0	0	2	960.48
									SPDTNYLFMGDYVDR	95.0%	69.6	20.2	4	0	0	2	1,792.79
									YSFLQFDPAPR	95.0%	64.7	21.8	4	0	0	2	1,340.66
T-complex protein 1 subunit gamma	TCPG_HUMAN	CCT3	60,517	100.00%	9	9	12	17.10%	AMTGVEQWPYR	95.0%	38.8	21.3	1	0	0	2	1,353.63
									AVAQALEVIPR	95.0%	72.6	16.3	2	0	0	2	1,166.69
									ELGIWEPLAVK	95.0%	40.5	19.1	1	0	0	2	1,254.71
									GISDLAQHYLMR	95.0%	41.1	22.9	1	0	0	2	1,419.71
									KVQSGNINAAK	95.0%	49.2	21.6	1	0	0	2	1,129.63
									TAVETAVLLLR	95.0%	84.3	15.8	2	0	0	2	1,185.72
									TLIQNCGASTIR	95.0%	45.9	23.4	2	0	0	2	1,333.69
									VQSGNINAAK	95.0%	34.3	23.0	1	0	0	2	1,001.54
									WSSLACNIALDAVK	95.0%	71.5	22.8	1	0	0	2	1,547.79
Insulin-like growth factor-binding protein 2	IBP2_HUMAN	IGFBP2	34,796	100.00%	17	20	220	69.20%	CPPCTPER	95.0%	34.4	16.8	1	0	0	2	1,016.43
									CYPHPGSELPLQALVMGEGTCEK	95.0%	58.7	20.3	0	2	0	2	2,589.18
									DAEYGASPEQVADNGDDHSEGGLVENHVDSTMNMLGGGGSAGR	95.0%	22.5	14.1	0	0	1	2	4,318.81
									EKVTEQHR	95.0%	56.3	22.0	6	0	0	2	1,026.53
									GDPECHLFYNEQQEAR	95.0%	67.0	15.9	2	4	0	2	1,992.85
									GECWCVNPNTGK	95.0%	71.0	15.3	8	0	0	2	1,421.59
									GPLEHLYSLHIPNCDK	95.0%	30.8	22.1	0	1	0	2	1,892.93
									HGLYNLK	95.0%	39.8	22.7	20	0	0	2	844.47
									HHLGLEEPK	95.0%	51.1	22.2	1	48	0	2	1,059.56
									HHLGLEEPKK	95.0%	30.3	23.0	0	1	0	2	1,187.65
									LAACGPPPVAPPAAVAAVAGGAR	95.0%	107.0	19.3	41	4	0	2	2,041.10
									LEGEACGVYTPR	95.0%	85.4	21.6	21	0	0	2	1,351.63
									LIQGAPTIR	95.0%	43.7	15.7	31	0	0	2	968.59
									MPCAELVR	95.0%	50.5	21.6	8	0	0	2	975.48
									MSLNGQR	95.0%	36.9	22.8	4	0	0	2	805.40
									SGMKELAVFR	95.0%	44.2	23.2	0	4	0	2	1,153.60
									TPCQQELDQVLER	95.0%	108.0	21.4	12	0	0	2	1,615.78
Matrix metalloproteinase-9	MMP9_HUMAN	MMP9	78,442	100.00%	11	11	45	19.70%	AFALWSAVTPLTFTR	95.0%	65.4	20.6	2	0	0	2	1,680.91
									AVIDDAFAR	95.0%	42.8	23.5	2	0	0	2	977.51
									FQTFEGDLK	95.0%	31.6	22.1	1	0	0	2	1,084.53
									FTEGPPLHKDDVNGIR	95.0%	32.9	22.5	0	2	0	2	1,794.91
									GSRPQGPFLIADKWPALPR	95.0%	44.3	17.5	0	0	4	2	2,106.16
									LFGFCPTR	95.0%	42.5	21.6	2	0	0	2	997.49
									LGLGADVAQVTGALR	95.0%	110.0	18.9	8	0	0	2	1,440.82
									QLAEEYLYR	95.0%	42.3	20.8	2	0	0	2	1,184.60
									QLSLPETGELDSATLK	95.0%	65.6	21.9	4	0	0	2	1,701.89
									QSTLVLFPGDLR	95.0%	74.0	21.0	6	0	0	2	1,345.75

									SLGPALLLLQK	95.0%	80.3	11.5	12	0	0	2	1,152.74
LDLR chaperone MESD	MESD_HUMAN	MESDC2	26,060	100.00%	5	5	8	27.80%	DYNDADMAR	95.0%	42.3	13.2	1	0	0	2	1,086.42
									IDPSKPESILK	95.0%	43.6	19.1	2	0	0	2	1,226.70
									LLEQWEKDDDIEEGDLPEHK	95.0%	36.7	21.8	0	2	0	2	2,438.14
									RPSAPVDFSK	95.0%	44.7	23.5	2	0	0	2	1,103.59
									TLMMFVTVSGSPTEK	95.0%	38.5	21.6	1	0	0	2	1,659.80
Glutathione reductase, mitochondrial	GSHR_HUMAN	GSR	56,239	100.00%	8	9	51	23.00%	ADFDNTVAIHPTSSEELVTLR	95.0%	66.1	21.6	1	9	0	2	2,315.15
									ALLTPVAIAAGR	95.0%	74.6	14.0	20	0	0	2	1,152.71
									GHAAFTSDPKPTIEVSGK	95.0%	30.8	21.4	0	1	0	2	1,841.94
									LGGTCVNVGCVPK	95.0%	53.0	22.8	2	0	0	2	1,360.67
									LGIQTDDKGHIIVDEFQNTNVK	95.0%	43.0	20.6	0	5	0	2	2,484.27
									LNAIYQNNLTK	95.0%	55.9	21.8	6	0	0	2	1,291.70
									RAAELGAR	95.0%	56.2	21.5	3	0	0	2	843.48
									TYSTSFTPMYHAVTK	95.0%	58.8	21.8	4	0	0	2	1,733.82
Isocitrate dehydrogenase [NADP] cytoplasmic	IDHC_HUMAN	IDH1	46,643	100.00%	18	20	114	50.50%	ATDFVVPGPGK	95.0%	64.4	23.2	5	0	0	2	1,087.58
									DAAEAIKK	95.0%	37.5	23.4	2	0	0	2	845.47
									DATNDQVTK	95.0%	40.0	21.8	1	0	0	2	991.47
									FKDIFQEIYDK	95.0%	52.0	22.8	6	6	0	2	1,445.73
									GQETSTNPIASIFAWTR	95.0%	97.2	23.0	27	0	0	2	1,878.94
									GWPLYLSTK	95.0%	47.1	22.3	4	0	0	2	1,064.58
									IIWELIK	95.0%	30.7	20.2	2	0	0	2	914.57
									ISGGSVVEMQGDEMTR	95.0%	98.6	18.8	6	0	0	2	1,727.76
									KISGGSVVEMQGDEMTR	95.0%	38.4	21.3	0	2	0	2	1,855.85
									LIDDMVAQAMK	95.0%	57.0	22.5	5	0	0	2	1,266.61
									LVSGWVKPIIIGR	95.0%	58.9	12.3	2	3	0	2	1,437.89
									NILGGTVFR	95.0%	54.8	20.8	5	0	0	2	976.56
									SDYLNTFEFMDK	95.0%	81.6	17.2	10	0	0	2	1,525.65
									SEGGFIWACK	95.0%	48.1	20.5	2	0	0	2	1,154.53
									SIEDFAHSSFQMALSK	95.0%	40.6	21.6	2	0	0	2	1,813.84
									TVEAEAAHGTVTR	95.0%	76.1	22.7	10	0	0	2	1,341.68
									VEITYTPSDGTQK	95.0%	63.4	23.0	8	0	0	2	1,438.71
									VTYLVHNFEEGGGVAMGMYNQDK	95.0%	63.1	18.6	0	6	0	2	2,591.15
Quinone oxidoreductase	QOR_HUMAN	CRYZ	35,189	100.00%	6	6	20	27.40%	ILGTAGTEEGQK	95.0%	53.0	23.5	4	0	0	2	1,203.62
									KPLLPYTPGSDVAGVIEAVGDNASAFK	95.0%	33.1	19.5	0	2	0	2	2,716.42
									QGAAIGIPYFTAYR	95.0%	52.7	21.0	4	0	0	2	1,527.80
									SDIAVPIPK	95.0%	41.5	14.8	4	0	0	2	939.55
									VAEAHENIIHGSGATGK	95.0%	79.1	22.3	2	0	0	2	1,690.85
									VFEFGGPEVLK	95.0%	47.1	22.9	4	0	0	2	1,221.65
Desmoplakin	DESP_HUMAN	DSP	331,763	100.00%	28	29	115	11.60%	AELIVQPELK	95.0%	52.9	16.8	4	0	0	2	1,139.67
									AESGPDLR	95.0%	35.3	19.4	1	0	0	2	844.42
									AFIGFEGVK	95.0%	36.0	21.5	2	0	0	2	967.53
									ALLQAILQTEDMLK	95.0%	72.7	19.9	12	0	0	2	1,586.88
									ANSSATETINK	95.0%	51.2	23.4	2	0	0	2	1,135.56
									ATGSEVSQR	95.0%	44.0	23.6	1	0	0	2	934.46
									FGDSNTVMR	95.0%	49.8	18.8	4	0	0	2	1,042.46
									FLEFQYLTGGLVDPEVHGR	95.0%	47.8	21.6	0	2	0	2	2,177.10
									GFFDPNTEENLTYLQLK	95.0%	76.7	22.5	8	0	0	2	2,028.99
									GIVDSITGQR	95.0%	45.6	22.7	4	0	0	2	1,045.56
									GYFNEELSEILSDPSDDTK	95.0%	67.5	20.3	2	0	0	2	2,158.97
									IEVLEEELR	95.0%	51.7	23.7	4	0	0	2	1,129.61
									ISITEGIER	95.0%	33.5	23.0	1	0	0	2	1,017.56

									ITNLTQQLEQASIVK	95.0%	72.9	17.3	1	0	0	2	1,685.94
									LSLQDAVSQGVIDQDMATR	95.0%	78.6	22.4	1	0	0	2	2,063.01
									NQCTQVVQER	95.0%	43.1	21.7	15	0	0	2	1,261.60
									QAELDGK	95.0%	30.4	22.6	1	0	0	2	760.38
									QLQNIIQATSR	95.0%	63.8	22.2	13	0	0	2	1,271.71
									RVEEDIQQQK	95.0%	34.8	22.7	2	0	0	2	1,272.66
									SAIYQLEEEYENLLK	95.0%	69.2	21.8	6	0	0	2	1,841.92
									SGSLSLTQFADMISLK	95.0%	53.3	22.5	2	0	0	2	1,713.87
									SMVEDITGLR	95.0%	34.2	22.9	3	0	0	2	1,136.56
									STLEAETR	95.0%	36.1	24.1	4	0	0	2	906.45
									SVQNDSQAIAEVLNQLK	95.0%	99.2	21.0	7	2	0	2	1,856.97
									TLELQGLINDLQR	95.0%	71.1	20.1	3	0	0	2	1,512.84
									VTQLTDR	94.8%	30.2	24.9	1	0	0	2	832.45
									YEVTSGGGGTSR	95.0%	59.5	21.1	5	0	0	2	1,170.54
									YGDGIQLTR	95.0%	46.2	22.2	2	0	0	2	1,022.53
Protein RCC2	RCC2_HUMAN	RCC2	56,067	100.00%	8	11	42	14.20%	AGGAAVVITEPEHTK	95.0%	57.1	22.1	6	5	0	2	1,479.78
									AGGAAVVITEPEHTKER	95.0%	50.7	21.2	0	5	2	2	1,764.92
									DGQILPVPNVVVR	95.0%	60.8	15.9	7	0	0	2	1,405.82
									LFDFPGR	95.0%	37.0	21.7	4	0	0	2	851.44
									LGHAEQKDEMVPR	95.0%	42.9	22.6	1	4	0	2	1,525.74
									RVEAPR	95.0%	38.9	19.6	3	0	0	2	727.42
									VFSWGFGGYGR	95.0%	38.9	21.7	2	0	0	2	1,232.59
									VKLEGSK	95.0%	41.0	20.5	3	0	0	2	760.46
Vinculin	VINC_HUMAN	VCL	123,783	100.00%	45	48	212	47.90%	AAAVGTANK	95.0%	46.4	25.2	1	0	0	2	802.44
									AGEVINQPMMMAAR	95.0%	56.1	20.5	10	0	0	2	1,566.71
									AIPDLTAPVAAVQAAVSNLVR	95.0%	103.0	13.4	8	0	0	2	2,076.18
									ALASIDSK	95.0%	31.7	25.0	2	0	0	2	804.45
									ALASQLQDSLK	95.0%	66.3	22.9	12	0	0	2	1,173.65
									AQQVSQGLDVLTAK	95.0%	102.0	21.6	11	0	0	2	1,457.80
									AVAGNISDPGLQK	95.0%	76.8	21.1	10	0	0	2	1,269.68
									AVANSRPAK	95.0%	61.8	20.1	2	0	0	2	913.52
									DPSASPGDAGEQAIR	95.0%	84.5	20.9	9	0	0	2	1,470.68
									EAEAASIK	95.0%	46.5	24.2	1	0	0	2	818.43
									EAFQPQEPDFPPPPPDLEQLR	95.0%	79.6	21.6	4	0	0	2	2,447.19
									ELLPVLISAMK	95.0%	59.6	18.7	12	0	0	2	1,213.72
									ELTPQVVSAAR	95.0%	40.6	21.6	2	0	0	2	1,170.65
									ETVQTTEDQILK	95.0%	55.6	23.1	2	0	0	2	1,404.72
									ETVQTTEDQILKR	95.0%	67.7	22.3	2	0	0	2	1,560.82
									EVENSEDPK	95.0%	32.5	18.0	1	0	0	2	1,046.46
									EVENSEDPKFR	95.0%	43.2	21.8	2	0	0	2	1,349.63
									GEGESPQAR	95.0%	47.5	18.6	1	0	0	2	930.43
									GQGSSPVAMQK	95.0%	55.4	22.8	4	0	0	2	1,089.54
									GVGQAAIR	95.0%	55.5	18.9	4	0	0	2	771.45
									GWLRDPSASPGDAGEQAIR	95.0%	36.3	22.2	1	1	0	2	1,982.97
									IPTISTQLK	95.0%	42.5	19.3	2	0	0	2	1,000.60
									KIDAAQNWLADPNGGPEGEEQIR	95.0%	69.9	21.4	0	2	0	2	2,508.21
									LANVMMGPYR	95.0%	62.5	21.3	2	0	0	2	1,183.56
									LLAVAATAPPDAPNREEVFDER	95.0%	47.1	21.2	0	10	0	2	2,381.21
									LVQAAQMLQSDPYSVPAR	95.0%	88.1	21.7	4	1	0	2	1,990.01
									MALLMAEMSR	95.0%	76.8	22.5	4	0	0	2	1,152.56
									MLGQMTDQVADLR	95.0%	88.2	21.8	5	0	0	2	1,509.70

									MQEAMTQEVSDVFSDTTTPIK	95.0%	133.0	20.0	4	0	0	2	2,358.08
									MSAEINEIIR	95.0%	53.6	24.0	4	0	0	2	1,191.60
									MTGLVDEAIDTK	95.0%	73.7	23.2	6	0	0	2	1,292.64
									NPGNQAAYEHFETMK	95.0%	59.5	21.4	1	0	0	2	1,736.77
									NQGIEEALK	95.0%	37.8	21.2	3	0	0	2	1,001.53
									NQGIEEALKNR	95.0%	27.5	21.1	0	1	0	2	1,271.67
									QVATALQNLQTK	95.0%	81.0	21.5	11	0	0	2	1,314.74
									SFLDSGYR	95.0%	39.7	19.5	3	0	0	2	944.45
									SLGEISALTSK	95.0%	63.7	22.4	6	0	0	2	1,105.61
									STVEGIQASVK	95.0%	78.4	22.5	2	0	0	2	1,118.61
									TNISDEESEQATEMLVHNAQNLMQSVK	95.0%	77.7	17.8	0	11	0	2	3,078.40
									TNLLQVCER	95.0%	46.5	23.8	2	0	0	2	1,132.58
									VAMANIQPQMLVAGATSIAR	95.0%	155.0	19.8	5	6	0	2	2,058.08
									VDQLTAQLADLAAR	95.0%	46.4	21.2	2	0	0	2	1,484.81
									VLQLTSWDEDAWASK	95.0%	73.7	22.7	6	0	0	2	1,748.85
									VMLVNSMNTVK	95.0%	51.2	22.7	5	0	0	2	1,235.65
									WIDNPTVDDR	95.0%	51.0	20.3	2	0	0	2	1,230.58
Semaphorin-3A	SEM3A_HUMAN	SEMA3A	88,873	100.00%	3	3	6	5.58%	DGPNYQWVPYQGR	95.0%	71.6	20.8	2	0	0	2	1,579.73
									DLPDDVITFAR	95.0%	63.8	23.2	3	0	0	2	1,261.64
									QQQLYIGSTAGVAQLPLHR	95.0%	28.8	19.1	0	1	0	2	2,080.13
Heat shock 70 kDa protein 1A/1B	HSP71_HUMAN	HSPA1A	70,036	100.00%	29	40	520	54.40%	AEDEVQR	95.0%	32.6	19.3	1	0	0	2	846.40
									AFYPEEISSMVLTK	95.0%	93.3	22.3	35	0	0	2	1,630.80
									AQIHDLVLVGGSTR	95.0%	92.0	19.9	25	3	0	2	1,465.81
									ARFEELCSDLFR	95.0%	42.6	21.5	2	1	0	2	1,542.74
									ATAGDTHLGGEDFDNR	95.0%	80.2	18.3	16	24	0	2	1,675.73
									CQEVISWLDANTLAEKDEFEHK	94.7%	18.5	21.0	0	0	1	2	2,662.25
									DAGVIAGLNVLR	95.0%	93.0	18.3	46	0	0	2	1,197.70
									ELEQVCNPIISGLYQGAGGPGPGGFGAQGPK	95.0%	92.6	20.1	0	7	0	2	3,055.50
									FEELCSDLFR	95.0%	49.7	20.0	7	0	0	2	1,315.60
									FGDPVVQSDMK	95.0%	73.9	21.4	10	0	0	2	1,238.57
									HWPFQVINDGDKPK	95.0%	50.8	22.5	1	58	0	2	1,680.85
									IINEPTAAAIAYGLDR	95.0%	122.0	21.0	62	19	0	2	1,687.90
									ITITNDKGR	95.0%	64.4	22.3	20	0	0	2	1,017.57
									KFGDPVVQSDMK	95.0%	44.4	22.8	4	0	0	2	1,350.67
									LDKAQIHDLVLVGGSTR	95.0%	21.4	17.6	0	0	1	2	1,822.02
									LLQDFFNGR	95.0%	57.6	22.5	21	0	0	2	1,109.57
									LSKEEIER	95.0%	55.0	22.9	12	0	0	2	1,003.54
									LVNHFVEEFK	95.0%	41.3	23.1	6	5	0	2	1,261.66
									LVNHFVEEFKR	95.0%	42.2	22.6	2	10	0	2	1,417.76
									MVQEAEK	95.0%	49.8	23.2	3	0	0	2	834.40
									MVQEAEKYK	95.0%	32.2	23.3	3	0	0	2	1,125.56
									NALESYAFNMK	95.0%	82.7	20.3	14	0	0	2	1,287.60
									NQVALNPQNTVFDAK	95.0%	108.0	22.0	37	0	0	2	1,658.85
									NQVALNPQNTVFDAKR	95.0%	64.5	21.8	2	1	0	2	1,814.95
									NSTIPTK	95.0%	33.7	25.0	4	0	0	2	760.42
									QTQIFTTYSDNQPGVLIQVYEGER	95.0%	46.3	21.1	2	3	0	2	2,786.36
									SAVEDEGLK	95.0%	39.1	22.6	2	0	0	2	947.47
									SAVEDEGLKGK	95.0%	41.2	23.9	1	0	0	2	1,132.59
									SINPDEAVAYGAAVQAAILMGDK	95.0%	135.0	21.3	17	3	0	2	2,320.15
									STLEPVEK	95.0%	36.5	22.2	1	0	0	2	902.48
									TTPSYVAFTDTER	95.0%	106.0	21.6	97	0	0	2	1,487.70

									VEIIANDQGNR	95.0%	80.0	22.6	56	0	0	2	1,228.63
									VQVSYK	95.0%	30.9	21.0	1	0	0	2	723.40
									YKAEDEVQR	95.0%	50.8	23.2	28	25	0	2	1,137.55
SH3 domain-binding glutamic acid-rich-like protein 3	SH3L3_HUMAN	SH3BGRL3	10,420	100.00%	4	5	31	39.80%	IQYQLVDISQDNALR	95.0%	104.0	21.4	2	0	0	2	1,775.93
									IQYQLVDISQDNALRDEMR	95.0%	58.8	22.4	4	8	0	2	2,307.14
									SQQSEVTR	95.0%	45.0	23.6	7	0	0	2	934.46
									VYSTSVTGSR	95.0%	76.8	21.7	10	0	0	2	1,056.53
Collagen alpha-1(VI) chain	CO6A1_HUMAN	COL6A1	108,513	100.00%	14	17	66	16.70%	DAEEAISQTIDTIVDMIK	95.0%	67.3	22.0	2	0	0	2	2,007.98
									GDEGPPGSEGAR	95.0%	68.0	21.6	3	0	0	2	1,128.49
									GDPGEAGPQGDQGR	95.0%	65.3	18.6	6	0	0	2	1,340.58
									GDPGFEGER	95.0%	30.8	20.0	1	0	0	2	963.42
									GLEQLLVGGSHLK	95.0%	46.0	18.1	2	3	0	2	1,350.77
									IALVITDGR	95.0%	47.8	20.0	2	0	0	2	957.57
									LKPYGALVDK	95.0%	30.4	18.4	0	1	0	2	1,103.65
									LLLFSDGNSQGATPAAIEK	95.0%	88.5	22.7	12	0	0	2	1,932.01
									NVQELK	94.7%	30.1	22.9	2	0	0	2	730.41
									TAEYDVAYGESHLFR	95.0%	65.2	20.8	2	3	0	2	1,757.81
									TDPAHDVR	95.0%	32.2	21.8	2	0	0	2	910.44
									VAVVQYSGTGQQRPER	95.0%	48.9	21.8	3	7	0	2	1,774.92
									VFSVAITPDHLEPR	95.0%	47.8	21.1	3	0	0	2	1,580.84
									VPSYQALLR	95.0%	54.8	21.9	12	0	0	2	1,046.60
Glutamate--cysteine ligase catalytic subunit	GSH1_HUMAN	GCLC	72,750	100.00%	3	3	4	5.65%	LDFLIPLSK	95.0%	35.9	16.8	1	0	0	2	1,045.63
									NTPSPFIETFTEDDEASR	95.0%	109.0	19.0	2	0	0	2	2,055.91
									VVINVPIFK	95.0%	56.4	14.6	1	0	0	2	1,028.65
X-ray repair cross-complementing protein 6	XRCC6_HUMAN	XRCC6	69,828	100.00%	6	6	11	13.60%	ILELDQFK	95.0%	33.2	20.3	1	0	0	2	1,005.56
									IMATPEQVGK	95.0%	37.6	24.4	2	0	0	2	1,089.56
									NIPPYFVALVPQEEELDDQK	95.0%	34.0	22.3	2	0	0	2	2,344.17
									NIYVLQELDNPGAK	95.0%	90.9	22.1	2	0	0	2	1,573.82
									SDSFENPVLQQHFR	95.0%	34.0	22.2	0	3	0	2	1,703.81
									TFNTSTGGLLLPSDTKR	95.0%	58.9	21.2	1	0	0	2	1,807.96
Calpain small subunit 1	CPNS1_HUMAN	CAPNS1	28,299	100.00%	4	5	9	23.10%	LFAQLAGDDMEVSATELMNILNK	95.0%	140.0	21.0	2	2	0	2	2,523.25
									LGFEEFK	95.0%	42.9	20.5	1	0	0	2	869.44
									SMVAVMDSDTTGK	95.0%	47.7	16.9	1	0	0	2	1,373.59
									YSDESGNMDFDNFISCLVR	95.0%	107.0	15.2	3	0	0	2	2,268.95
Xaa-Pro aminopeptidase 1	XPP1_HUMAN	XPNPEP1	69,901	100.00%	2	2	4	5.14%	GSLTFEPLTLVPIQTK	95.0%	45.3	17.9	2	0	0	2	1,743.99
									QEALEWLIR	95.0%	43.1	21.8	2	0	0	2	1,157.63
									VTSELLR	95.0%	33.9	22.3	1	0	0	2	817.48
Proteasome subunit beta type-3	PSB3_HUMAN	PSMB3	22,932	100.00%	7	10	46	47.80%	DAVSGMGVIVHIIEK	95.0%	64.8	22.4	2	0	0	2	1,583.85
									FGIQAQMVTTDFQK	95.0%	87.6	22.5	9	0	0	2	1,629.79
									FGPYYTEPVIAGLDPK	95.0%	71.2	21.5	9	1	0	2	1,766.90
									LNLYELK	95.0%	42.7	19.6	6	0	0	2	892.51
									LYIGLAGLATDVQTVAQR	95.0%	102.0	16.2	12	4	0	2	1,889.05
									NCVAIAADRR	95.0%	41.6	23.2	1	1	0	2	1,145.58
									QIKPYTLMSMVANLLYEK	95.0%	42.5	20.9	0	1	0	2	2,174.12
Laminin subunit gamma-2	LAMC2_HUMAN	LAMC2	130,958	100.00%	23	24	286	24.60%	ADSLSSLVTR	95.0%	63.5	23.6	11	0	0	2	1,048.56
									AGYFGDPLAPNPADK	95.0%	72.7	22.5	14	0	0	2	1,532.74
									ALGSAAADAQR	95.0%	89.0	22.8	63	0	0	2	1,030.53
									ALHEGVGSGSGSPDGAVVQGLVEK	95.0%	73.5	21.7	0	6	0	2	2,250.14
									AQGGDGVVPDTELEGR	95.0%	81.4	22.0	18	0	0	2	1,599.76
									CLNCNDNTDGIHCEK	95.0%	54.1	11.8	1	0	0	2	1,849.73
									CVCKPAVTGER	95.0%	36.6	22.5	0	7	0	2	1,276.61

									EATQAEIEADR	95.0%	62.2	20.6	3	0	0	2	1,232.58
									EFDLQVDNR	95.0%	55.6	22.2	5	0	0	2	1,135.54
									ELEFDTNMDAVQMVITEAQK	95.0%	65.9	19.5	1	0	0	2	2,344.07
									EVEGELER	95.0%	40.5	21.6	2	0	0	2	960.46
									HPSAHDVILEGAGLR	95.0%	51.3	21.3	0	25	0	2	1,571.83
									HQDVFSSAQR	95.0%	66.0	21.6	21	0	0	2	1,174.56
									IQMDQFMQQLQR	95.0%	83.1	22.6	28	0	0	2	1,565.76
									ITSTFHQDVDGWK	95.0%	73.1	22.6	4	0	0	2	1,533.73
									LAESHVESASNMEQLTR	95.0%	84.9	22.3	7	18	0	2	1,901.90
									LDPVYFVAPAK	95.0%	71.5	21.2	13	0	0	2	1,219.67
									MQQAEQALQDILR	95.0%	99.2	23.0	16	0	0	2	1,559.79
									NLGNWKEEAQQLLQNGK	95.0%	39.2	21.0	0	4	0	2	1,970.01
									QCIFDR	95.0%	31.8	22.1	2	0	0	2	838.39
									SLAQEATR	95.0%	67.2	24.5	10	0	0	2	875.46
									SSAEYSVHK	95.0%	49.4	21.9	3	0	0	2	1,007.48
									VSDASDKTQQAER	95.0%	78.8	21.7	4	0	0	2	1,434.68
Spliceosome RNA helicase BAT1	UAP56_HUMAN	BAT1	48,974	100.00%	9	9	33	22.90%	DFLLKPELLR	95.0%	32.5	16.1	1	0	0	2	1,243.74
									DVQEIFR	95.0%	34.4	24.2	3	0	0	2	906.47
									ELAFQISK	95.0%	37.0	20.9	2	0	0	2	935.52
									FMQDPMEIFVDDETK	95.0%	92.6	18.3	5	0	0	2	1,844.81
									GLAITFVSDENDAK	95.0%	45.1	22.8	2	0	0	2	1,479.73
									ILVATNLFGR	95.0%	79.9	17.7	10	0	0	2	1,103.66
									LTLHGLQQYYVK	94.7%	26.4	20.0	0	1	0	2	1,462.81
									QVMMFSATLSK	95.0%	49.4	21.6	4	0	0	2	1,258.62
									VAVFFGGLSIK	95.0%	42.5	16.5	5	0	0	2	1,137.67
High mobility group protein HMG-I/HMG-Y	HMGA1_HUMAN	HMGA1	11,658	100.00%	2	2	4	22.40%	KQPPVSPGTALVGSQK	95.0%	57.4	17.8	2	0	0	2	1,593.90
									SSQPLASK	95.0%	51.6	24.1	2	0	0	2	817.44
3-ketoacyl-CoA thiolase, mitochondrial	THIM_HUMAN	ACAA2	41,906	100.00%	2	2	3	7.81%	AANDAGYFNDEMAPIEVK	95.0%	78.8	19.0	2	0	0	2	1,970.88
									DFTATDLSEFAAK	95.0%	40.6	21.4	1	0	0	2	1,415.67
Tissue factor pathway inhibitor	TFPI1_HUMAN	TFPI	34,998	100.00%	2	2	5	5.59%	FFFNIFTR	95.0%	39.7	24.0	2	0	0	2	1,091.57
									FYYNSVIGK	95.0%	38.2	24.0	3	0	0	2	1,090.56
DNA damage-binding protein 2	DDB2_HUMAN	DDB2	47,847	100.00%	2	2	3	4.22%	ASFLYSLPHR	95.0%	29.1	23.3	0	1	0	2	1,190.63
									YNLIVVGR	95.0%	64.3	19.6	2	0	0	2	933.55
Guanine nucleotide-binding protein subunit beta-2-like 1	GBLP_HUMAN	GNB2L1	35,059	100.00%	10	11	40	34.70%	DETNYGIPQR	95.0%	52.6	22.2	5	0	0	2	1,192.56
									DGQAMLWDLNEGK	95.0%	92.9	20.8	6	0	0	2	1,492.67
									DVLSVAFSSDNR	95.0%	79.2	22.1	6	0	0	2	1,309.64
									FSPNSSNPIIVSCGWDK	95.0%	49.3	20.7	2	0	0	2	1,907.90
									IIVDELKQEVISTSSK	95.0%	118.0	18.1	6	2	0	2	1,789.00
									LTRDETNYGIPQR	95.0%	46.6	22.7	2	0	0	2	1,562.79
									LWDLTTGTTTR	95.0%	55.0	22.7	6	0	0	2	1,264.65
									LWNTLGVCK	95.0%	51.4	24.2	2	0	0	2	1,090.57
									QIVSGSR	95.0%	38.3	24.2	1	0	0	2	746.42
									YWLCAATGPSIK	95.0%	44.1	22.3	2	0	0	2	1,366.68
Lactotransferrin	TRFL_HUMAN	LTF	78,164	100.00%	2	2	13	2.96%	CGLVPVLAENYK	95.0%	63.3	22.2	6	0	0	2	1,362.71
									YYGYTGAFR	95.0%	42.4	21.7	7	0	0	2	1,097.51
Galectin-3-binding protein	LG3BP_HUMAN	LGALS3BP	65,314	100.00%	24	32	2361	47.40%	AAFGQGSGPIMLDEVQCTGTEASLADCK	95.0%	49.5	19.3	0	7	0	2	2,913.31
									ASHEEVEGLVEK	95.0%	65.9	22.9	93	78	0	2	1,326.65
									AVDTWSWGER	95.0%	81.0	19.6	353	0	0	2	1,206.55
									ELSEALGQIFDSQR	95.0%	125.0	22.8	314	37	0	2	1,592.79
									GLNLTEDTYKPR	95.0%	55.4	22.9	2	3	0	2	1,406.73
									GQWGTVCDNLWDLTDASVVCR	95.0%	122.0	19.7	8	2	0	2	2,452.10

									IDITLSSVK	95.0%	65.7	19.2	37	0	0	2	975.57
									IYTSPTWSAFVTDSSWSAR	95.0%	145.0	21.4	139	10	0	2	2,162.02
									KSQLVYQSR	95.0%	57.8	20.0	58	13	0	2	1,108.61
									KTLQALEFHTVPFQLLAR	95.0%	33.3	14.3	0	0	22	2	2,112.20
									LADGGATNQGR	95.0%	95.2	20.7	110	0	0	2	1,059.52
									LASAYGAR	95.0%	61.8	23.0	78	0	0	2	808.43
									RIDITLSSVK	95.0%	51.0	16.6	88	0	0	2	1,131.67
									SDLAVPSELALLK	95.0%	90.9	18.8	132	0	0	2	1,355.78
									SLGWLK	95.0%	45.3	24.7	74	0	0	2	703.41
									SQLVYQSR	95.0%	58.7	21.4	91	0	0	2	980.52
									STHTLDLSR	95.0%	43.9	21.4	26	0	0	2	1,029.53
									STSSFPCPAGHFNGFR	95.0%	48.6	20.1	6	0	0	2	1,768.79
									TIAYENK	95.0%	42.7	22.7	13	0	0	2	838.43
									TLQALEFHTVPFQLLAR	95.0%	87.8	17.5	98	116	0	2	1,984.10
									TVIRPFYLTNSSGVD	95.0%	51.7	21.0	2	0	0	2	1,668.86
									VEIFYR	95.0%	35.5	21.1	23	0	0	2	826.45
									YSSDYFQAPSDYR	95.0%	105.0	17.6	304	0	0	2	1,598.68
									YYPYQSFQTPQHPSFLFQDK	95.0%	58.5	21.6	5	19	0	2	2,521.18
Glutamate dehydrogenase 1, mitochondrial	DHE3_HUMAN	GLUD1	61,382	100.00%	14	18	51	30.10%	ALASLMTYK	95.0%	45.8	20.8	5	0	0	2	1,013.53
									DDGSWEVIEGYR	95.0%	79.8	20.3	4	0	0	2	1,425.63
									DIVHSGLAYTMER	95.0%	57.7	21.6	4	1	0	2	1,507.72
									DSNYHLLMSVQESLER	95.0%	54.1	21.7	2	0	0	2	1,936.91
									GASIVEDK	95.0%	44.4	24.2	1	0	0	2	818.43
									GASIVEDKLVEDLR	95.0%	71.7	21.9	2	3	0	2	1,543.83
									GFIGPGIDVPAPDMSTGER	95.0%	68.8	22.6	4	0	0	2	1,931.92
									HGGTIPIVPTAEFQDR	95.0%	59.7	21.5	2	0	0	2	1,737.89
									IIAEGANGPTTPEADKIFLER	95.0%	93.0	20.8	2	3	0	2	2,242.17
									LQHGSILGFPK	95.0%	34.7	19.0	1	1	0	2	1,196.68
									MVEGFFDR	95.0%	53.6	18.1	5	0	0	2	1,016.45
									TAAYVNAIEK	95.0%	44.2	21.7	4	0	0	2	1,079.57
									YNLGLDLR	95.0%	65.0	23.1	3	0	0	2	963.53
									YSTDVSVDEVK	95.0%	77.3	21.8	4	0	0	2	1,241.59
Pre-mRNA-processing factor 19	PRP19_HUMAN	PRPF19	55,163	100.00%	4	4	7	12.30%	IWSVPNASCVQVVR	95.0%	48.4	21.7	2	0	0	2	1,614.84
									SSEQILATLK	95.0%	47.0	22.2	2	0	0	2	1,089.62
									TVPEELVKPEELSK	95.0%	56.4	20.5	2	0	0	2	1,597.87
									YIAENGTDPINNQPLSEEQLIDIK	95.0%	46.0	21.0	1	0	0	2	2,714.35
Splicing factor U2AF 65 kDa subunit	U2AF2_HUMAN	U2AF2	53,483	100.00%	2	2	12	5.47%	ELLTSFGPLK	95.0%	50.6	21.3	7	0	0	2	1,104.63
									LFIGGLPNYLNDDQVK	95.0%	53.5	21.6	5	0	0	2	1,805.94
Heterogeneous nuclear ribonucleoprotein R	HNRPR_HUMAN	HNRNPR	70,926	100.00%	3	3	19	11.20%	AGPIWDLR	95.0%	39.9	21.3	2	0	0	2	927.51
									DLYEDELVPLFEK	95.0%	43.1	21.7	4	0	0	2	1,609.80
									EAAQEAVK	95.0%	37.7	22.0	5	0	0	2	845.44
									NLATTVTEEILEK	95.0%	81.0	22.1	14	0	0	2	1,460.79
									TGYTLDVTTGQR	95.0%	43.9	22.5	2	0	0	2	1,311.65
									VTEGLVDVILYHQPDDK	95.0%	34.8	21.7	0	1	0	2	1,941.00
Proteasome subunit beta type-4	PSB4_HUMAN	PSMB4	29,187	100.00%	8	9	54	48.50%	AIHSWLTR	95.0%	40.8	19.6	2	0	0	2	983.54
									FEGGVVIAADMLGSYGSLAR	95.0%	83.1	22.5	8	0	0	2	2,029.01
									FQIATVTEK	95.0%	45.8	20.5	6	0	0	2	1,036.57
									GVEIEGPLSTETNWDIAHMISGFE	95.0%	71.0	20.0	2	0	0	2	2,648.22
									QPVLSQTEAR	95.0%	57.0	22.0	6	0	0	2	1,128.60
									QVLGQMVIDEELLGDGHSYSPR	95.0%	94.5	21.3	2	13	0	2	2,459.19
									TQNPMVTGTSVLGVK	95.0%	89.8	22.1	9	0	0	2	1,547.81

									VNNSTMLGASGDYADFQYLK	95.0%	108.0	21.3	6	0	0	2	2,210.01
Polypeptide N-acetylgalactosaminyltransferase 5	GALT5_HUMAN	GALNT5	106,250	100.00%	3	3	11	3.51%	ALLPEDSGTHQVLR	95.0%	71.7	21.8	4	0	0	2	1,535.82
									NDNPYSFPK	95.0%	41.2	20.5	4	0	0	2	1,081.50
									VEVDLDQTQR	95.0%	37.6	22.4	3	0	0	2	1,202.60
Fructose-bisphosphate aldolase C	ALDOC_HUMAN	ALDOC	39,438	100.00%	9	13	100	46.40%	ALQASALNAWR	95.0%	66.2	22.7	2	0	0	2	1,200.65
									DGADFAK	95.0%	46.1	22.6	14	0	0	2	723.33
									DNAGAATEEFIKR	95.0%	69.4	23.1	2	0	0	2	1,421.70
									GILAADESVGSMAK	95.0%	86.2	22.6	4	0	0	2	1,364.67
									GVVPLAGTDGETTTQGLDGLSER	95.0%	129.0	21.5	46	9	0	2	2,273.13
									KDGADFAK	95.0%	55.0	22.3	4	0	0	2	851.43
									QVLFSADDR	95.0%	57.3	23.3	4	0	0	2	1,050.52
									TPSALAILENANVLAR	95.0%	93.2	17.1	12	2	0	2	1,652.93
									VDKGVVPLAGTDGETTTQGLDGLSER	95.0%	84.6	20.8	2	11	0	2	2,615.32
									VLAAVYK	95.0%	45.5	18.0	18	0	0	2	763.47
									YASICQQNGIVPIVEPEILPDGDHDLK	95.0%	68.3	21.0	0	5	0	2	3,020.50
									YASICQQNGIVPIVEPEILPDGDHDLKR	95.0%	91.7	19.7	0	5	0	2	3,176.61
									YEGSGEDGGAAAQSLYIANHAY	95.0%	114.0	19.2	2	0	0	2	2,243.98
									YTPEEIAMATVTALR	95.0%	64.9	22.9	3	1	0	2	1,681.85
Heat shock 70 kDa protein 4	HSP74_HUMAN	HSPA4	94,314	100.00%	32	35	178	48.50%	AESEEMETSQAGSK	95.0%	117.0	16.4	2	0	0	2	1,483.62
									AFSDPFVEAEK	95.0%	51.8	21.5	11	0	0	2	1,239.59
									AGGIETIANEYSDR	95.0%	69.1	22.0	13	0	0	2	1,495.70
									EFSITDVVPYPISLR	95.0%	65.0	20.7	16	0	0	2	1,735.93
									ELSTTLNADEAVTR	95.0%	78.3	23.2	10	0	0	2	1,519.76
									EMLNLYIENEGK	95.0%	62.1	22.3	2	0	0	2	1,452.70
									FLEMCNDLLAR	95.0%	49.5	20.9	2	0	0	2	1,397.66
									FQESEERPK	95.0%	44.0	22.8	8	8	0	2	1,149.55
									GCALQCAILSPAFK	95.0%	37.1	22.6	1	0	0	2	1,535.77
									LEDTENWLYEDGEDQPK	95.0%	95.2	16.9	6	0	0	2	2,080.90
									LFEELGK	95.0%	30.8	20.2	1	0	0	2	835.46
									LKETAESVLK	95.0%	40.5	21.0	3	0	0	2	1,117.65
									LMNETTAVALAYGIYK	95.0%	96.9	22.0	3	0	0	2	1,773.91
									LNLQNK	95.0%	33.4	23.3	2	0	0	2	729.43
									MDQPPQAK	95.0%	47.6	19.4	4	0	0	2	930.44
									MQVDQEEPHVEEQQQTPAENK	95.0%	70.2	19.1	0	2	0	2	2,638.17
									MQVDQEEPHVEEQQQTPAENKAESEEMETSQAGSK	95.0%	42.8	13.0	0	0	2	2	4,118.77
									NAEQNGPVDGQGDNPGPQAAEQGTDTAVPSDSDK	95.0%	46.1	16.3	0	2	0	2	3,366.46
									NAVEEYVYEMR	95.0%	66.4	18.3	5	0	0	2	1,418.63
									NFTTEQVTAMLLSK	95.0%	88.8	21.9	10	0	0	2	1,582.82
									NKEDQYDHLDAADMTK	95.0%	31.3	20.2	0	2	0	2	1,893.83
									QDLPALEEKPR	95.0%	66.0	21.0	3	2	0	2	1,295.70
									QIQQYMK	95.0%	33.5	21.5	1	0	0	2	954.47
									QSLTMDPVVK	95.0%	41.8	23.0	3	0	0	2	1,117.59
									SEENEEPMETDQNAK	95.0%	60.7	12.3	1	0	0	2	1,766.70
									SEENEEPMETDQNAKEEEK	95.0%	34.1	13.8	0	1	0	2	2,281.93
									SNLAYDIVQLPTGLTGIK	95.0%	135.0	17.2	25	1	0	2	1,903.05
									STNEAMEWMNNK	95.0%	74.1	17.0	6	0	0	2	1,454.60
									SVMDATQIAGLNCLR	95.0%	88.4	22.9	4	0	0	2	1,664.81
									TSTVDLPIENQLLWQIDR	95.0%	94.1	20.6	4	0	0	2	2,141.12
									VEPPKEEQK	95.0%	31.8	20.7	1	0	0	2	1,083.57
									VLATAFDTTLGGR	95.0%	114.0	21.7	11	0	0	2	1,321.71
Tissue alpha-L-fucosidase	FUCO_HUMAN	FUCA1	53,672	100.00%	5	5	24	14.20%	DGLIVPIFQER	95.0%	41.2	21.6	2	0	0	2	1,286.71

									DLVGELGTALR	95.0%	81.9	23.1	13	0	0	2	1,143.64
									DNYPPGFSYADFGPQFTAR	95.0%	94.9	18.8	7	0	0	2	2,149.96
									FFHPEEWADLFQAAGAK	95.0%	28.5	22.7	0	1	0	2	1,963.93
									TQHFVSAK	95.0%	37.3	23.5	1	0	0	2	917.48
ERO1-like protein alpha	ERO1A_HUMAN	ERO1L	54,377	100.00%	7	7	22	18.20%	FDGILTEGEGPR	95.0%	62.5	22.0	2	0	0	2	1,290.63
									LGAVDESLSEETQK	95.0%	91.0	23.1	2	0	0	2	1,505.73
									LIANMPESGPSYEFHLTR	95.0%	38.9	22.8	0	2	0	2	2,078.00
									LLESDYFR	95.0%	41.9	21.9	2	0	0	2	1,042.52
									LQTQGLGTALK	95.0%	41.1	20.4	2	0	0	2	1,129.66
									QEIVSLFNAFGR	95.0%	88.1	22.2	10	0	0	2	1,380.73
									YLLQETWLEK	95.0%	40.7	22.0	2	0	0	2	1,322.70
Glycylpeptide N-tetradecanoyltransferase 1	NMT1_HUMAN	NMT1	56,789	100.00%	6	6	13	16.50%	AIELFSVGQGPAK	95.0%	58.9	22.0	2	0	0	2	1,316.72
									DIPVVHQLLTR	95.0%	32.2	18.1	3	0	0	2	1,290.75
									GFDVFNALDLMENK	95.0%	55.9	22.1	1	0	0	2	1,612.77
									GSETDSAQDQPVK	95.0%	32.5	19.6	2	0	0	2	1,361.62
									LGEVVNTHGPVEPDKDNIR	95.0%	48.8	21.8	0	2	0	2	2,089.07
									SYQFWDTQPVPK	95.0%	48.8	22.1	3	0	0	2	1,495.72
Glycyl-tRNA synthetase	SYG_HUMAN	GARS	83,124	100.00%	9	9	20	16.10%	APQVDVDK	95.0%	51.0	20.1	2	0	0	2	871.45
									GEFTIETEGK	95.0%	40.3	22.1	2	0	0	2	1,110.53
									LGDAVEQGVINNTVLGYFIGR	95.0%	77.5	19.2	2	0	0	2	2,235.18
									LPFAAAQIGNSFR	95.0%	73.9	21.9	4	0	0	2	1,391.74
									QQGDLVR	95.0%	36.5	21.5	1	0	0	2	815.44
									SPITGNDLSPPVSFNLMFK	95.0%	32.3	22.2	2	0	0	2	2,064.05
									TLYVEEVVPNVIEPSFGLGR	95.0%	65.3	19.2	1	0	0	2	2,218.18
									TVNVVQFEPSK	95.0%	56.8	22.5	4	0	0	2	1,247.66
									VDDSSGSIGR	95.0%	68.9	22.2	2	0	0	2	992.47
RuvB-like 2	RUVB2_HUMAN	RUVBL2	51,140	100.00%	8	8	20	21.00%	AAGVVLEMIR	95.0%	57.9	22.3	2	0	0	2	1,058.60
									AVLIAGQPGTGK	95.0%	63.3	17.6	2	0	0	2	1,111.65
									GLGLDDALEPR	95.0%	73.6	21.5	2	0	0	2	1,155.60
									LLIVSTTPYSEK	95.0%	64.2	19.9	2	0	0	2	1,350.75
									QASQGMVGQLAAR	95.0%	51.9	23.0	2	0	0	2	1,332.67
									TQGFLALFSGDTGEIK	95.0%	95.3	21.3	4	0	0	2	1,683.86
									TTEMETIYDLGTK	95.0%	74.2	22.1	4	0	0	2	1,501.71
									VYSLFLDESR	95.0%	45.2	22.8	2	0	0	2	1,228.62
Vesicular integral-membrane protein VIP36	LMAN2_HUMAN	LMAN2	40,212	100.00%	6	6	10	25.60%	DRLVPGPVFGSK	95.0%	32.0	21.7	0	2	0	2	1,271.71
									LFQLMVEHTPDEESIDWTK	95.0%	31.8	21.4	0	1	0	2	2,334.10
									LPTGYYFGASAGTGDLSDNHDIISMK	95.0%	48.7	19.3	0	2	0	2	2,746.27
									NCIDITGVR	95.0%	32.5	23.0	1	0	0	2	1,047.53
									NLHGDGIALWYTR	95.0%	30.5	22.4	1	0	0	2	1,515.77
									WTELAGCTADFR	95.0%	86.3	20.5	3	0	0	2	1,426.64
Endoplasmic reticulum aminopeptidase 2	ERAP2_HUMAN	ERAP2	110,449	100.00%	9	9	17	10.60%	AAELFSQWMESSGK	95.0%	69.6	20.0	2	0	0	2	1,570.72
									FDLGSYDIR	95.0%	50.2	22.0	2	0	0	2	1,085.53
									FPWQELR	95.0%	34.5	23.4	1	0	0	2	975.51
									ILAVTDFEPTQAR	95.0%	70.9	22.5	5	0	0	2	1,460.78
									LIELGMEGK	95.0%	33.2	23.7	1	0	0	2	989.53
									TQNLAALLHAIAR	95.0%	60.0	15.7	2	0	0	2	1,391.81
									VLSYPAHEQIALLVPEK	95.0%	40.9	17.4	1	0	0	2	1,907.06
									VSIYASPDKR	94.7%	30.1	22.5	1	0	0	2	1,135.61
									YFDIYYPLSK	95.0%	39.5	23.1	2	0	0	2	1,308.65
ATP-dependent RNA helicase A	DHX9_HUMAN	DHX9	140,944	100.00%	9	9	22	9.45%	AIEPPPLDAVIEAEHTLR	95.0%	27.5	19.8	0	1	0	2	1,971.06
									DFVNYLVR	95.0%	39.3	22.3	2	0	0	2	1,025.54

									DVVQAYPEVR	95.0%	44.8	23.6	3	0	0	2	1,175.61
									ELDALDANDELTPLGR	95.0%	49.9	21.5	4	0	0	2	1,741.86
									GMTLVTPLQLLLFASK	95.0%	38.8	13.4	2	0	0	2	1,748.00
									LGGIGQFLAK	95.0%	36.2	19.2	2	0	0	2	1,003.59
									QPAIISQLDPVNER	95.0%	59.0	20.9	3	0	0	2	1,579.84
									TTQVPQFILDDFIQNDR	95.0%	55.4	22.0	4	0	0	2	2,050.02
									YPSPFFVFGEK	95.0%	31.9	22.1	1	0	0	2	1,317.65
40S ribosomal protein S3	RS3_HUMAN	RPS3	26,671	100.00%	7	7	18	35.40%	DEILPTTPISEQK	95.0%	86.3	22.0	2	0	0	2	1,470.77
									ELAEDGYSGVEVR	95.0%	79.4	20.8	3	0	0	2	1,423.67
									FGFPEGSVELYAEK	95.0%	38.5	22.8	2	0	0	2	1,572.76
									GGKPEPPAMPQPVPTA	95.0%	45.9	23.7	5	0	0	2	1,573.81
									KPLPDHVSIVEPK	95.0%	37.4	18.8	0	2	0	2	1,458.83
									LLGGLAVR	95.0%	44.8	14.8	2	0	0	2	798.52
									TEIIILATR	95.0%	37.9	15.3	2	0	0	2	1,029.63
Carcinoembryonic antigen-related cell adhesion molecule 6	CEAM6_HUMAN	CEACAM6	37,177	99.50%	2	3	22	9.88%	EVLLLAHNLPQNR	95.0%	45.4	18.3	4	1	0	2	1,516.86
									SDPVTLNVLYGPDGPTISPSK	95.0%	99.8	21.5	17	0	0	2	2,157.11
26S proteasome non-ATPase regulatory subunit 1	PSMD1_HUMAN	PSMD1	105,821	100.00%	5	5	12	9.13%	DTSEDIEELVEPVAAHGPK	95.0%	41.5	21.6	0	2	0	2	2,035.98
									EAINLLEPMTNDPVNYVR	95.0%	54.3	22.1	4	0	0	2	2,088.04
									QDVYDLLK	95.0%	35.4	21.7	3	0	0	2	993.53
									TPEASPEPK	95.0%	47.4	21.1	2	0	0	2	955.47
									TVGTPIASVPGSTNTGTVPGSEKDSDSMETEEK	95.0%	43.7	19.0	0	1	0	2	3,324.53
Amyloid-like protein 2	APLP2_HUMAN	APLP2	86,937	100.00%	17	24	499	31.10%	ADMDQFTASISETPVDVR	95.0%	110.0	20.4	61	2	0	2	1,981.92
									AKEQLEIR	95.0%	43.5	22.0	22	0	0	2	986.56
									CLVGEFVSDVLLVPEK	95.0%	76.5	21.6	5	0	0	2	1,803.96
									EEVLQYCQEMYPELQITNVMEANQR	95.0%	42.3	17.9	0	1	0	2	3,147.41
									EMIFNAER	95.0%	43.2	22.1	8	0	0	2	1,009.48
									EWEEAELQAK	95.0%	56.6	20.9	28	0	0	2	1,232.58
									GSGVGEQDGGLIGAEEK	95.0%	82.8	22.4	7	0	0	2	1,602.76
									HYQHVLAVDPEK	95.0%	46.3	22.7	4	139	0	2	1,435.73
									LNMHVNIQTGK	95.0%	53.3	22.2	6	2	0	2	1,270.66
									MALENYLAALQSDPPRPHR	95.0%	64.2	21.4	0	49	88	2	2,195.10
									NKVDENMVIDETLDVK	95.0%	47.2	22.4	3	3	0	2	1,861.92
									QQLVETHLAR	95.0%	59.7	19.3	19	18	0	2	1,194.66
									QTLIQHFQAMVK	95.0%	36.7	22.7	3	0	0	2	1,459.77
									SCFETK	95.0%	42.0	16.2	6	0	0	2	771.33
									SQVMTHLHVIEER	95.0%	36.4	22.4	0	3	1	2	1,578.81
									VEAMLNDR	95.0%	57.2	21.9	32	0	0	2	963.46
									VPYVAQEIQEEIDELLQEQR	95.0%	62.7	21.4	0	5	0	2	2,429.22
									VSIDNWCR	95.0%	52.9	22.0	16	0	0	2	1,049.48
Eukaryotic translation initiation factor 4B	IF4B_HUMAN	EIF4B	69,136	100.00%	3	4	20	7.53%	AASIFGGAKPVDTAAR	95.0%	53.0	22.0	4	2	0	2	1,531.82
									STPKEDDSSASTSQSTR	95.0%	40.4	19.8	1	0	0	2	1,783.79
									VAPAQPSEEGPGR	95.0%	61.0	23.3	13	0	0	2	1,294.64
Proteasome subunit beta type-1	PSB1_HUMAN	PSMB1	26,473	100.00%	8	10	69	43.60%	AGGSASAMLQPLLDNQVGFK	95.0%	103.0	22.3	16	4	0	2	2,020.02
									AMTTGAIAAMLSTILYSR	95.0%	73.5	22.0	6	0	0	2	1,902.97
									DVFISAAER	95.0%	58.8	23.3	10	0	0	2	1,007.52
									DVYTGDALR	95.0%	36.7	22.0	4	0	0	2	1,009.50
									EGIREETVSLR	95.0%	46.1	23.1	3	0	0	2	1,288.69
									GAVYSFDPVGSYQR	95.0%	92.4	21.4	11	0	0	2	1,545.73
									LSEGFSIHTR	95.0%	28.6	24.1	0	1	0	2	1,146.59
									NMQNVEHVPLSLDR	95.0%	72.8	22.6	8	6	0	2	1,667.82
Kallikrein-10	KLK10_HUMAN	KLK10	30,120	100.00%	9	14	231	27.20%	ALQLPYR	95.0%	36.0	22.6	5	0	0	2	860.50

									GLTCSSITILSPK	95.0%	79.7	22.0	4	0	0	2	1,376.75
									LARPVVPGPR	95.0%	29.8	9.5	0	1	0	2	1,061.66
									RTDEHDLMLLK	95.0%	50.0	21.8	6	35	0	2	1,386.71
									TDEHDLMLLK	95.0%	41.8	22.2	1	0	0	2	1,214.61
									VGDDHLLLLQGEQLR	95.0%	88.6	19.6	28	51	0	2	1,705.92
									VGDDHLLLLQGEQLRR	95.0%	51.3	18.1	1	22	6	2	1,862.03
									YHQGSGPILPR	95.0%	43.1	21.7	10	54	0	2	1,224.65
									YMSWINK	95.0%	41.2	21.0	7	0	0	2	941.46
Thioredoxin domain-containing protein 5	TXND5_HUMAN	TXNDC5	47,611	100.00%	6	6	24	13.70%	EYVESQLQR	95.0%	45.2	23.0	2	0	0	2	1,151.57
									FVLSQAKDEL	95.0%	45.8	22.8	5	0	0	2	1,149.62
									GYPTLLLFR	95.0%	50.8	18.6	10	0	0	2	1,079.63
									GYPTLLWFR	95.0%	42.7	20.9	4	0	0	2	1,152.62
									IAEVDCTAER	95.0%	53.6	21.0	2	0	0	2	1,163.54
									TLAPTWEELSKK	95.0%	37.7	22.1	1	0	0	2	1,402.76
Parathyroid hormone-related protein	PTHR_HUMAN	PTHLH	20,176	100.00%	3	4	9	20.90%	ATSEVSPNSKPSPNTK	95.0%	70.8	22.8	2	1	0	2	1,643.82
									FGSDDEGR	95.0%	59.9	16.1	4	0	0	2	882.36
									YLTQETNKVETYK	95.0%	65.2	23.5	2	0	0	2	1,616.82
Actin-related protein 2	ARP2_HUMAN	ACTR2	44,744	100.00%	3	3	16	9.90%	DLMVGDEASELR	95.0%	86.3	20.7	5	0	0	2	1,350.62
									ILLTEPPMNPTK	95.0%	55.8	20.5	10	0	0	2	1,353.75
									SMLEVNYPMENGIVR	95.0%	70.9	21.9	1	0	0	2	1,783.84
Dihydrolipoyl dehydrogenase, mitochondrial	DLDH_HUMAN	DLD	54,159	100.00%	10	12	33	24.00%	ADGGTQVIDTK	95.0%	68.4	22.7	2	0	0	2	1,104.55
									ALTGGIAHLFK	95.0%	58.3	20.2	2	0	0	2	1,127.66
									EANLAASFGK	95.0%	32.5	23.2	1	0	0	2	1,007.52
									GIEMSEVR	95.0%	42.6	22.1	2	0	0	2	936.45
									GRIPVNTR	95.0%	31.7	16.3	1	0	0	2	912.54
									IPNIYAIGDVVAGPMLAHK	95.0%	80.4	19.6	2	10	0	2	1,995.07
									NETLGGTCLNVGCIPSK	95.0%	81.7	23.1	2	0	0	2	1,819.87
									NLGLEELGIELDPR	95.0%	70.1	21.7	4	0	0	2	1,567.83
									SEEQLKEEGIEYK	95.0%	62.4	22.8	2	3	0	2	1,581.77
									VGKFPFAANSR	95.0%	54.7	21.7	0	2	0	2	1,193.64
Nucleobindin-1	NUCB1_HUMAN	NUCB1	53,862	100.00%	28	36	231	59.90%	AKMDAEQDPNVQVDHLNLLK	95.0%	41.8	21.6	0	4	1	2	2,294.15
									APAAHPEGQLK	95.0%	52.7	23.4	10	0	0	2	1,118.60
									DLELLIQTATR	95.0%	68.5	21.1	9	0	0	2	1,272.72
									EFGDTGEGWETVEMHPAYTEEELR	95.0%	56.8	15.3	0	2	0	2	2,828.20
									EKLQAANAEDIK	95.0%	64.1	23.0	2	0	0	2	1,329.70
									ELDFVSHHVR	95.0%	71.6	23.0	4	0	0	2	1,238.63
									ELQQAVLHMEQR	95.0%	78.5	23.0	16	2	0	2	1,481.75
									EVWEELDGLDPNR	95.0%	59.3	21.9	11	0	0	2	1,571.73
									EVWEELDGLDPNRFNPK	95.0%	57.7	22.3	2	0	0	2	2,057.99
									FEEELAAR	95.0%	55.1	23.4	8	0	0	2	964.47
									FHPDTDDVPVPAPAGDQK	95.0%	76.6	21.8	5	2	0	2	1,905.90
									FHPDTDDVPVPAPAGDQKEVDTSEK	95.0%	61.9	21.2	0	2	0	2	2,694.25
									KLEEQQR	95.0%	42.3	23.7	1	0	0	2	930.50
									LDELKR	95.0%	35.9	23.5	2	0	0	2	773.45
									LLERLPEVEVPQHL	95.0%	29.1	17.8	0	1	0	2	1,671.94
									LPEVEVPQHL	95.0%	63.2	22.8	18	0	0	2	1,160.63
									LQAANAEDIK	95.0%	54.7	21.5	7	0	0	2	1,072.56
									LSQETEALGR	95.0%	80.5	23.4	15	0	0	2	1,103.57
									LVTLEEFLASTQR	95.0%	83.9	20.3	22	4	0	2	1,506.82
									MDAEQDPNVQVDHLNLLK	95.0%	121.0	21.8	4	11	0	2	2,095.01
									NEEDDMREMEEER	95.0%	29.8	9.0	0	2	0	2	1,743.64

									NVDTNQDR	95.0%	32.0	18.6	1	0	0	2	961.43
									QFEHLDPQNQHTFEAR	95.0%	35.0	21.8	3	3	0	2	1,996.93
									RFEEELAAR	95.0%	42.2	23.8	3	0	0	2	1,120.58
									VNVPGSQAQLK	95.0%	52.3	20.5	19	0	0	2	1,140.64
									YLESLGEEQR	95.0%	45.4	22.2	4	0	0	2	1,223.59
									YLESLGEEQRK	95.0%	51.5	22.6	4	1	0	2	1,351.69
									YLQEVIDVLETDGHFR	95.0%	110.0	22.8	8	18	0	2	1,933.97
ES1 protein homolog, mitochondrial	ES1_HUMAN	C21orf33	28,152	100.00%	2	2	3	13.10%	EVVEAHVDQK	95.0%	34.1	23.0	1	0	0	2	1,153.59
									ITDLANLSAANHDAAIFPGGFGAAK	95.0%	39.2	21.4	0	2	0	2	2,442.24
NIF3-like protein 1	NIF3L_HUMAN	NIF3L1	41,951	100.00%	2	2	8	6.37%	ALMQVVDFLSR	95.0%	72.8	22.2	6	0	0	2	1,294.68
									GIDGVSVTSFSAR	95.0%	81.2	22.0	2	0	0	2	1,295.66
Prolyl endopeptidase	PPCE_HUMAN	PREP	80,684	100.00%	5	5	15	11.70%	LPEADDIQYPSMLLLTADHDDR	95.0%	64.7	20.5	0	3	0	2	2,544.19
									NILQLHDLTTGALLK	95.0%	57.8	14.9	2	0	0	2	1,649.96
									SDGTETSTNLHQK	95.0%	31.5	20.8	1	0	0	2	1,417.66
									TFPLDVGSIVGYSGQK	95.0%	73.9	21.8	2	0	0	2	1,667.86
									VFLDPNILSDDGTVALR	95.0%	115.0	21.5	7	0	0	2	1,844.98
Protein arginine N-methyltransferase 1	ANM1_HUMAN	PRMT1	41,498	100.00%	7	9	30	25.20%	ANKLDHVVTIIK	95.0%	32.3	12.8	0	4	0	2	1,350.81
									ATLYVTAIEDR	95.0%	56.3	21.7	4	0	0	2	1,251.66
									DVAIKEPLVDVVDPK	95.0%	58.3	18.7	6	5	0	2	1,636.92
									TGEEIFGTIGMRPNAK	95.0%	37.6	22.1	2	1	0	2	1,736.86
									TGFSTSPESPYTHWK	95.0%	48.8	21.0	2	0	0	2	1,724.79
									VEEVELPVEK	95.0%	45.7	20.6	4	0	0	2	1,170.63
									WLAPDGLIFPDR	95.0%	38.9	21.0	2	0	0	2	1,399.74
Endoplasmin	ENPL_HUMAN	HSP90B1	92,454	100.00%	21	24	118	35.00%	DISTNYYASQK	95.0%	59.3	20.5	8	0	0	2	1,289.60
									EAESSPFVER	95.0%	44.4	21.7	6	0	0	2	1,150.54
									EEASDYLELDTIK	95.0%	88.7	22.1	4	0	0	2	1,525.73
									EESDDEAAVEEEEEEKKPK	95.0%	49.6	18.4	0	1	0	2	2,219.97
									EFEPLLNWMK	95.0%	43.0	23.1	3	0	0	2	1,322.65
									ELISNASDALDK	95.0%	92.5	23.1	15	0	0	2	1,275.64
									ELISNASDALDKIR	95.0%	108.0	21.4	10	2	0	2	1,544.83
									FAFQAEVNR	95.0%	44.4	23.1	4	0	0	2	1,081.54
									FQSSHHPTDITSLDQYVER	95.0%	86.1	21.4	0	4	2	2	2,260.06
									GLFDEYGSK	95.0%	57.2	19.4	4	0	0	2	1,015.47
									GVVDSDDLPLNVSR	95.0%	111.0	22.1	13	0	0	2	1,485.76
									IADDKYNDTFWK	95.0%	52.5	21.4	2	0	0	2	1,515.71
									IYFMAGSSR	95.0%	38.3	21.2	2	0	0	2	1,047.49
									LGVIEDHSNR	95.0%	59.3	22.4	7	2	0	2	1,139.58
									LIINSLYK	95.0%	39.8	14.3	4	0	0	2	963.59
									LISLTDENALSGNEELTVK	95.0%	75.1	21.6	22	0	0	2	2,046.06
									NLLHVTDTGVGMTR	95.0%	59.9	22.0	3	4	0	2	1,513.78
									SGTSEFLNK	95.0%	31.9	21.6	1	0	0	2	982.48
									SGYLLPDTK	95.0%	46.2	21.7	4	0	0	2	993.53
									SILFVPTSAPR	95.0%	65.1	20.6	9	0	0	2	1,187.68
									TDDEVVQREEEAIQLDGLNASQIR	95.0%	44.4	21.4	0	4	0	2	2,728.34
									VFITDDFHDMMPK	95.0%	33.4	17.4	1	0	0	2	1,627.71
									YSQFINFPIYVWSSK	95.0%	65.3	22.6	4	0	0	2	1,878.94
Peroxiredoxin-5, mitochondrial	PRDX5_HUMAN	PRDX5	22,008	100.00%	9	11	27	44.90%	ETDLLLDDSLVSIFGNR	95.0%	89.4	22.0	7	0	0	2	1,906.98
									ETDLLLDDSLVSIFGNRR	95.0%	38.5	20.7	2	1	0	2	2,063.08
									FSMVVQDGIVK	95.0%	59.7	21.7	2	0	0	2	1,238.65
									GVLFGVPGAFTPGCSK	95.0%	33.5	22.6	1	0	0	2	1,593.81
									LLADPTGAFGK	95.0%	33.8	22.9	2	0	0	2	1,089.59

									THLPGFVEQAEALK	95.0%	51.1	21.8	2	1	0	2	1,539.82
									VGDAIPAVEVFEGEPGNK	95.0%	95.1	22.1	2	0	0	2	1,827.91
									VGDAIPAVEVFEGEPGNKVNLAELFK	95.0%	37.6	19.2	0	4	0	2	2,742.44
									VNLAELFK	95.0%	57.7	19.9	3	0	0	2	933.54
Heterogeneous nuclear ribonucleoprotein A/B	ROAA_HUMAN	HNRNPAB	36,207	100.00%	3	3	34	13.30%	EVYQQQQYGSGGR	95.0%	98.9	21.0	22	0	0	2	1,499.69
									FGEVVDCTIK	95.0%	55.7	22.7	13	0	0	2	1,167.57
									GFGFILFK	95.0%	55.5	22.7	10	0	0	2	928.53
									MFVGGLSWDTSKK	95.0%	43.6	23.1	2	0	0	2	1,471.73
Carboxypeptidase A4	CBPA4_HUMAN	CPA4	47,334	100.00%	18	21	324	50.60%	APDAEELDK	95.0%	51.0	20.1	8	0	0	2	987.46
									APDAEELDKVAR	95.0%	34.7	22.0	0	2	0	2	1,313.67
									DPAITSILEK	95.0%	66.8	20.8	26	0	0	2	1,086.60
									DTGTYGFLLPANQIIPTAEETWLGLK	95.0%	39.4	18.8	0	3	0	2	2,848.48
									EWISQATAIWTAR	95.0%	76.6	23.0	69	0	0	2	1,532.79
									FFGDQVLR	95.0%	59.3	23.1	35	0	0	2	981.52
									IVSDYQR	95.0%	51.6	23.1	6	0	0	2	880.45
									KAPDAEELDK	95.0%	55.3	22.6	18	12	0	2	1,115.56
									KIVSDYQR	95.0%	40.6	20.3	8	0	0	2	1,008.55
									LSQLVNSNNLK	95.0%	65.6	22.2	26	0	0	2	1,229.69
									MDIFLLPVANPDGYVYTQTQNR	95.0%	83.5	22.0	17	35	0	2	2,571.26
									NGDEISK	95.0%	53.6	22.3	2	0	0	2	762.36
									NPGSSCIGADPNR	95.0%	49.6	19.1	1	0	0	2	1,344.60
									RPAVWLNAGIHSR	95.0%	32.0	19.9	0	8	0	2	1,476.82
									SPSSFNRPVDVLVPSVSLQAFK	95.0%	54.1	18.1	5	33	0	2	2,374.28
									SQGLEYAVTIEDLQALLDNEDDEMQHNEGQER	95.0%	74.9	16.9	0	1	0	2	3,705.65
									SVVDFIQK	95.0%	52.7	20.9	7	0	0	2	935.52
									TIMEHVR	95.0%	43.4	22.4	2	0	0	2	901.46
Galectin-1	LEG1_HUMAN	LGALS1	14,698	100.00%	2	2	4	17.80%	LNLEAINYMAADGDFK	95.0%	99.2	22.4	3	0	0	2	1,784.85
									LPDGYEFK	95.0%	31.8	20.9	1	0	0	2	968.47
Myoferlin	MYOF_HUMAN	MYOF	234,698	99.50%	2	2	4	1.16%	GPVGTVSEAQLAR	95.0%	72.2	21.1	2	0	0	2	1,284.69
									VIVESASNIPK	95.0%	48.0	21.9	2	0	0	2	1,156.66
Microtubule-associated protein RP/EB family member 1	MARE1_HUMAN	MAPRE1	29,982	100.00%	4	6	11	24.30%	FFDANYDGKDYDPVAAR	95.0%	35.2	20.1	0	1	0	2	1,963.88
									KPLTSSSAAPQRPISTQR	95.0%	36.7	19.0	0	2	1	2	1,925.06
									LEHEYIQNFK	95.0%	33.6	23.4	1	0	0	2	1,320.66
									QGQETAVAPSLVAPALNKPK	95.0%	56.1	16.1	1	5	0	2	2,019.12
Hyaluronan and proteoglycan link protein 3	HPLN3_HUMAN	HAPLN3	40,876	100.00%	2	2	3	4.44%	LTLTEAR	95.0%	30.6	23.3	1	0	0	2	803.46
									VGQLFAAWK	95.0%	49.0	21.3	2	0	0	2	1,019.57
Semaphorin-3B	SEM3B_HUMAN	SEMA3B	83,104	100.00%	6	6	14	10.90%	AASVLVGEELYSGVAADLMGR	95.0%	49.9	22.2	1	0	0	2	2,124.06
									AFLGPFAHK	95.0%	31.3	23.8	2	0	0	2	987.54
									DFPDDVIQFAR	95.0%	60.0	22.6	3	0	0	2	1,322.64
									ETAVEAAPALGR	95.0%	66.9	22.2	4	0	0	2	1,184.63
									LFVGAENHVASLNLDNISK	95.0%	46.7	21.0	0	2	0	2	2,041.07
									SAVAQIALHR	95.0%	50.2	18.2	2	0	0	2	1,065.62
									DHFLMDGQVR	95.0%	35.1	20.6	2	2	0	2	1,233.57
									GNVLLEDGK	95.0%	41.6	21.6	3	0	0	2	944.51
									GNVLLEDGKGR	95.0%	36.0	21.0	2	0	0	2	1,157.63
									GTTEGSAVCVFTMK	95.0%	63.9	20.5	4	0	0	2	1,503.68
									HVSLYQPQLATRPWIQDIEGASAK	95.0%	39.8	20.2	0	2	3	2	2,708.42
									INSSLQLPDR	95.0%	37.3	22.4	1	0	0	2	1,142.62
									ISLPLGSEERPFLR	95.0%	33.9	20.6	2	0	0	2	1,613.90
									MLLLQPQAR	95.0%	55.2	22.0	26	0	0	2	1,085.61
									SQSLRPTK	95.0%	35.3	22.5	5	0	0	2	916.52

									STALVVDGELYTGTVSSFQGNDPAISR	95.0%	37.9	21.8	0	1	0	2	2,784.37
									VPGLHHTYDVLFLGTGDGR	95.0%	41.8	21.9	0	3	0	2	2,054.05
Hsp90 co-chaperone Cdc37	CDC37_HUMAN	CDC37	44,450	100.00%	4	4	9	19.00%	EGEEAGPGDPLLEAVPK	95.0%	76.9	22.1	4	0	0	2	1,707.84
									LGPGGLDPVEVYESLPEELQK	95.0%	91.6	20.5	3	0	0	2	2,269.16
									QYMEGFNDELEAFKER	95.0%	30.7	18.2	0	1	0	2	2,021.89
									SMVNTKPEKTEEDSEEVR	95.0%	52.3	20.3	0	1	0	2	2,123.98
Obg-like ATPase 1	OLA1_HUMAN	OLA1	44,727	100.00%	6	6	21	18.20%	GGDGIKPPPIIGR	95.0%	38.8	18.7	0	1	0	2	1,276.74
									IGIVGLPNVGK	95.0%	46.3	13.2	3	0	0	2	1,066.66
									IPAFLNVVDIAGLVK	95.0%	78.3	12.8	9	0	0	2	1,568.94
									NYIVEDGDIIFFK	95.0%	45.7	23.0	1	0	0	2	1,572.80
									VPVPDER	95.0%	36.6	19.0	3	0	0	2	811.43
									YLEANMTQSALPK	95.0%	76.5	22.7	4	0	0	2	1,481.73
Nck-associated protein 1	NCKP1_HUMAN	NCKAP1	128,777	99.50%	2	2	4	2.39%	ELATVLSDQPGLLGPK	95.0%	46.1	18.2	2	0	0	2	1,637.91
									NNNQQLAQLQK	95.0%	56.2	21.4	2	0	0	2	1,298.68
Inositol polyphosphate 1-phosphatase	INPP_HUMAN	INPP1	43,981	99.50%	2	2	2	6.27%	NIFGEESNEFTNDWGEK	95.0%	86.4	16.8	1	0	0	2	2,015.86
									VASEALAR	95.0%	31.5	23.4	1	0	0	2	816.46
mRNA export factor	RAE1L_HUMAN	RAE1	40,951	100.00%	2	2	4	7.61%	GLIVYQLENQPSEFR	95.0%	32.9	21.7	1	0	0	2	1,792.92
									SSNPMMVLQLPER	95.0%	43.9	21.7	3	0	0	2	1,501.75
Extracellular matrix protein 1	ECM1_HUMAN	ECM1	60,655	100.00%	20	21	239	52.80%	ACPSHQPDISSGLELPFPPGVPTLDNIK	95.0%	33.2	20.9	0	1	0	2	2,986.50
									AWEDTLDKYCDR	95.0%	59.4	18.2	3	0	0	2	1,571.68
									ELLALIQLER	95.0%	74.2	16.6	32	0	0	2	1,197.72
									ELPSLQHPNEQK	95.0%	68.1	22.9	15	0	0	2	1,419.72
									EVGPPLPQEAVPLQK	95.0%	57.8	18.8	21	0	0	2	1,601.89
									FCEAEFSVK	95.0%	43.1	20.1	6	0	0	2	1,116.50
									FSCFQEEAPQPHYQLR	95.0%	41.2	20.3	0	6	0	2	2,036.93
									HPPSPTR	95.0%	35.5	25.0	3	0	0	2	791.42
									LDGFPPGRPSPDNLNQICLPNR	95.0%	27.3	21.6	0	1	0	2	2,477.24
									LLPAQLPAEK	95.0%	36.4	14.0	7	0	0	2	1,079.65
									LLPAQLPAEKEVGPPLPQEAVPLQK	95.0%	36.5	12.0	0	9	0	2	2,662.52
									LTFINDLCGPR	95.0%	65.5	23.2	8	0	0	2	1,305.66
									LVWEEAMSR	95.0%	61.0	22.3	30	0	0	2	1,136.54
									NLPATDPLQR	95.0%	36.0	22.1	4	0	0	2	1,124.61
									NVALVSGDTENAK	95.0%	98.4	23.3	23	0	0	2	1,317.67
									QGETLNFLEIGYSR	95.0%	111.0	22.7	57	0	0	2	1,626.81
									QHVVYGPWNLPQSSYSHLTR	95.0%	32.5	21.5	0	1	4	2	2,369.18
									QLRPEHFQEVGYAAPPSPPLSR	95.0%	29.9	20.8	0	3	0	2	2,476.27
									SLPMDHPDSSQHGPPFEGQSQVQPPPSQEATPLQQEK	95.0%	21.1	17.9	0	0	1	2	4,035.88
									SQGGWGHR	95.0%	56.4	20.1	4	0	0	2	884.41
Far upstream element-binding protein 1	FUBP1_HUMAN	FUBP1	67,543	100.00%	9	10	23	16.50%	EMVLELIR	95.0%	51.0	23.6	2	0	0	2	1,002.57
									IGGDAGTSLNSNDYGYGGQK	95.0%	85.1	19.1	2	0	0	2	1,973.88
									IGGNEGIDVPIPR	95.0%	89.8	21.4	5	0	0	2	1,336.72
									IQFKPDDGTTPER	95.0%	46.4	23.1	0	2	0	2	1,503.74
									IQIAPDSGGLPER	95.0%	77.0	21.9	2	0	0	2	1,352.72
									IQNDAGVR	95.0%	35.0	21.3	2	0	0	2	872.46
									MVMIQDGPQNTGADKPLR	95.0%	42.6	22.2	1	2	0	2	2,002.97
									RPLEDGDQPDAK	95.0%	38.3	22.1	2	0	0	2	1,340.65
									RPLEDGDQPDAKK	95.0%	33.9	21.9	0	3	0	2	1,468.74
Lysyl oxidase homolog 2	LOXL2_HUMAN	LOXL2	86,705	99.90%	2	2	3	2.58%	GGAYIGEGR	95.0%	32.0	23.3	1	0	0	2	879.43
									TPVMEGYVEVK	95.0%	43.9	22.1	2	0	0	2	1,267.62
Fructose-bisphosphate aldolase A	ALDOA_HUMAN	ALDOA	39,403	100.00%	28	38	1360	68.70%	AAQEEYVK	95.0%	60.5	21.6	10	0	0	2	937.46
									AAQEEYVKR	95.0%	92.5	22.9	59	14	0	2	1,093.56

									ADDGRPFPQVIK	95.0%	45.4	22.1	14	21	0	2	1,342.71
									ALANSLACQGK	95.0%	80.4	23.8	16	0	0	2	1,132.58
									ALQASALK	95.0%	64.5	22.3	39	0	0	2	801.48
									CPLLKPWALTFSYGR	95.0%	28.3	21.8	0	1	0	2	1,808.95
									DGADFAK	95.0%	46.1	22.6	14	0	0	2	723.33
									ELSDIAHR	95.0%	56.8	21.9	42	0	0	2	940.49
									FSHEEIAMATVTALR	95.0%	104.0	22.3	94	180	0	2	1,691.84
									FSHEEIAMATVTALRR	95.0%	33.8	21.5	0	0	12	2	1,831.95
									GGVVGIKVDK	95.0%	34.9	20.2	2	0	0	2	971.59
									GILAADESTGSIAK	95.0%	103.0	23.5	78	0	0	2	1,332.70
									GILAADESTGSIAKR	95.0%	107.0	21.5	8	4	0	2	1,488.80
									GVVPLAGTNGETTTQGLDGLSER	95.0%	140.0	21.6	20	4	0	2	2,272.14
									IGEHTPSALAIMENANVLAR	95.0%	90.9	21.2	19	283	0	2	2,107.10
									KDGADFAK	95.0%	55.0	22.3	4	0	0	2	851.43
									KELSDIAHR	95.0%	36.3	22.4	0	7	0	2	1,068.58
									LQSIGTENTEENR	95.0%	74.9	21.9	9	0	0	2	1,490.71
									LQSIGTENTEENRR	95.0%	48.8	23.0	3	7	0	2	1,646.81
									PYQYPALTPEQK	95.0%	67.2	23.1	104	0	0	1	1,434.73
									PYQYPALTPEQKK	95.0%	55.1	22.8	6	4	0	1	1,562.82
									QLLLTADDR	95.0%	76.1	23.1	27	0	0	2	1,044.57
									SKGGVVGIK	95.0%	43.9	20.9	3	0	0	2	844.53
									VDKGVVPLAGTNGETTTQGLDGLSER	95.0%	85.1	20.9	1	7	0	2	2,614.33
									VLAAVYK	95.0%	45.5	18.0	18	0	0	2	763.47
									YASICQQNGIVPIVEPEILPDGDHDLK	95.0%	68.3	21.0	0	5	0	2	3,020.50
									YASICQQNGIVPIVEPEILPDGDHDLKR	95.0%	91.7	19.7	0	5	0	2	3,176.61
									YTPSGQAGAAASESLFVSNHAY	95.0%	143.0	21.0	204	12	0	2	2,228.03
Fructose-1,6-bisphosphatase 1	F16P1_HUMAN	FBP1	36,797	100.00%	8	8	28	33.40%	AGIAHLYGIAGSTNVTGDQVK	95.0%	31.5	21.5	0	2	0	2	2,072.08
									AISSAVR	95.0%	39.7	24.8	2	0	0	2	703.41
									APVILGSPDDVLEFLK	95.0%	86.8	19.7	13	0	0	2	1,712.95
									DFDPAVTEYIQR	95.0%	67.6	22.4	4	0	0	2	1,453.70
									EAVLDVIPTDIHQR	95.0%	60.3	21.3	2	0	0	2	1,605.86
									GTGELTQLLNSLCTAVK	95.0%	70.4	21.3	2	0	0	2	1,804.95
									STDEPSEKDALQPGR	95.0%	61.9	22.2	1	0	0	2	1,629.77
									YVGSMVADVHR	95.0%	34.4	22.4	2	0	0	2	1,249.60
Src substrate cortactin	SRC8_HUMAN	CTTN	61,567	99.50%	2	2	4	4.18%	FGVQSER	95.0%	48.7	23.4	2	0	0	2	822.41
									TQTPPVSPAPQPTEER	95.0%	42.7	22.1	2	0	0	2	1,734.87
Nidogen-1	NID1_HUMAN	NID1	136,358	100.00%	21	25	145	19.00%	AAECVHR	95.0%	38.6	18.6	1	0	0	2	842.39
									AECLNPSQPSR	95.0%	39.5	21.5	1	0	0	2	1,258.59
									ALEGLQYPFAVTSYGK	95.0%	75.7	22.1	18	0	0	2	1,743.90
									ASLHGGEPTTIIR	95.0%	41.9	21.5	3	2	0	2	1,351.73
									EDLSPSITQR	95.0%	49.7	23.0	6	0	0	2	1,145.58
									EYTVTEPERDGASPSR	95.0%	46.5	22.1	8	0	0	2	1,793.83
									GNLYWTDWNR	95.0%	36.9	21.4	4	0	0	2	1,324.61
									IETSYMDGTNR	95.0%	72.1	17.2	8	0	0	2	1,302.56
									IYTYQWR	95.0%	35.0	22.0	2	0	0	2	1,029.52
									KALEGLQYPFAVTSYGK	95.0%	72.3	21.3	2	3	0	2	1,871.99
									MVYWTDITEPSIGR	95.0%	104.0	21.7	11	0	0	2	1,683.81
									NGFSITGGEFTR	95.0%	61.3	21.2	4	0	0	2	1,285.62
									NIFWTDSNLDR	95.0%	70.8	22.2	6	0	0	2	1,380.65
									NLYFTDWK	94.8%	30.2	21.2	1	0	0	2	1,086.53
									QAEVTFVGHPGNLVIK	95.0%	29.3	19.7	0	1	0	2	1,708.94

									QCVAEGSPQR	95.0%	35.8	19.8	3	0	0	2	1,131.52
									QDLGSPEGIAVDHLGR	95.0%	92.5	22.5	6	6	0	2	1,663.84
									SDIDAVYVTTNGIIATSEPPAK	95.0%	53.6	21.2	3	0	0	2	2,262.15
									VLFETDLVNPR	95.0%	71.6	22.5	29	0	0	2	1,302.71
									VYYREDLSPSITQR	95.0%	61.8	22.0	2	3	0	2	1,726.88
									YALSNSIGPVR	95.0%	81.3	22.6	12	0	0	2	1,176.64
1-phosphatidylinositol-4,5-bisphosphate phosphodiesterase beta-3	PLCB3_HUMAN	PLCB3	138,785	100.00%	5	5	9	5.51%	EAQVDAEAQR	95.0%	45.7	21.0	2	0	0	2	1,116.53
									EVVLDANTTQFK	95.0%	45.8	23.4	1	0	0	2	1,364.71
									LLDGLAQAQAEGR	95.0%	36.3	21.8	1	0	0	2	1,341.71
									LNEVLYPPLRPSQAR	95.0%	32.2	17.1	0	1	0	2	1,752.98
									LVAGQQQVLQQLAEEEPK	95.0%	88.9	20.0	4	0	0	2	2,008.07
Neurolysin, mitochondrial	NEUL_HUMAN	NLN	80,636	100.00%	2	2	2	3.27%	LVNTGLLTLR	95.0%	47.9	16.1	1	0	0	2	1,099.68
									NLNEDDTFLVFSK	95.0%	40.9	22.5	1	0	0	2	1,541.75
Tax1-binding protein 3	TX1B3_HUMAN	TAX1BP3	13,717	99.50%	2	2	3	19.40%	RSEEVVR	95.0%	40.3	23.9	1	0	0	2	874.47
									VSEGGPAEIAGLQIGDK	95.0%	93.6	21.7	2	0	0	2	1,640.85
Plexin-B2	PLXB2_HUMAN	PLXNB2	205,109	100.00%	2	2	3	1.52%	EASPNPEDGIVR	95.0%	54.0	21.7	2	0	0	2	1,283.62
									SSGGPGAGLCLFPLDK	95.0%	40.5	22.9	1	0	0	2	1,575.78
Gamma-glutamylcyclotransferase	GGCT_HUMAN	GGCT	20,990	100.00%	6	6	27	37.80%	ENGLPLEYQEK	95.0%	59.0	22.6	1	0	0	2	1,319.65
									LDFGNSQGK	95.0%	47.2	22.7	4	0	0	2	965.47
									NPSAAFFCVAR	95.0%	61.8	21.4	2	0	0	2	1,239.59
									SNLNSLDEQEGVK	95.0%	74.9	22.6	8	0	0	2	1,432.69
									SYLMTNYESAPPSPQYK	95.0%	81.0	19.9	4	0	0	2	1,991.91
									VSEEIEDIIK	95.0%	84.1	23.7	8	0	0	2	1,174.62
UPF0364 protein C6orf211	CF211_HUMAN	C6orf211	51,156	100.00%	3	3	5	9.75%	HGEEGVEAEK	95.0%	33.6	20.4	1	0	0	2	1,084.49
									LRNELQTDKPFIPLVEK	95.0%	35.8	15.9	0	2	0	2	2,040.15
									TIEDLDENQLKDEFFK	95.0%	95.6	21.7	2	0	0	2	1,983.96
Proteasome activator complex subunit 1	PSME1_HUMAN	PSME1	28,706	100.00%	9	11	41	36.90%	APLDIPVPDPVK	95.0%	47.5	18.9	2	0	0	2	1,260.72
									APLDIPVPDPVKEK	95.0%	52.0	18.5	2	3	0	2	1,517.86
									EPALNEANLSNLK	95.0%	46.5	22.0	2	0	0	2	1,412.74
									IEDGNNFGVAVQEK	95.0%	77.3	22.9	2	0	0	2	1,519.74
									ISELDAFLKEPALNEANLSNLK	95.0%	96.1	18.4	0	2	0	2	2,429.29
									IVVLLQR	95.0%	46.9	12.8	4	0	0	2	840.57
									NAYAVLYDIILK	95.0%	86.0	18.6	14	0	0	2	1,395.79
									QLVHELDEAEYR	95.0%	62.2	21.6	2	1	0	2	1,501.73
									TENLLGSYFPK	95.0%	56.0	22.0	7	0	0	2	1,268.65
Alpha-enolase	ENOA_HUMAN	ENO1	47,152	100.00%	34	47	2850	77.90%	AAVPSGASTGIYEALELR	95.0%	161.0	21.5	354	87	0	2	1,804.94
									AGYTDKVVIGMDVAASEFFR	95.0%	84.9	21.7	2	6	0	2	2,192.07
									AVEHINK	95.0%	40.7	19.5	20	0	0	2	810.45
									DATNVGDEGGFAPNILENK	95.0%	111.0	21.4	151	7	0	2	1,960.93
									DATNVGDEGGFAPNILENKEGLELLK	95.0%	77.3	21.6	0	84	0	2	2,743.38
									DYPVVSIEDPFDQDDWGAWQK	95.0%	113.0	17.1	22	4	0	2	2,510.12
									EGLELLK	95.0%	42.4	20.9	14	0	0	2	801.47
									EIFDSR	95.0%	31.7	22.0	3	0	0	2	766.37
									FTASAGIQVVGDDLTVTNPK	95.0%	128.0	21.7	277	38	0	2	2,033.06
									GNPTVEVDLFTSK	95.0%	110.0	23.0	163	0	0	2	1,406.72
									GVPLYR	95.0%	31.4	21.6	2	0	0	2	704.41
									HIADLAGNSEVILPVPAFNVINGGSHAGNK	95.0%	60.7	21.0	0	11	4	2	3,011.57
									IDKLMIEMDGTENK	95.0%	72.5	22.8	20	31	0	2	1,652.79
									IEEELGSK	95.0%	56.8	23.5	33	0	0	2	904.46
									IGAEVYHNLK	95.0%	67.0	22.8	69	30	0	2	1,143.62
									KLNVTEQEK	95.0%	52.7	23.4	53	11	0	2	1,088.60

									LAMQEFMILPVGAANFR	95.0%	114.0	21.5	297	41	0	2	1,923.98
									LAQANGWGVMVSHR	95.0%	41.4	22.1	0	3	0	2	1,525.77
									LMIEMDGTENK	95.0%	78.8	19.9	79	0	0	2	1,296.58
									LNVTEQEK	95.0%	55.3	23.7	25	0	0	2	960.50
									LNVTEQEKIDK	95.0%	42.9	22.8	3	0	0	2	1,316.71
									NFRNPLAK	95.0%	35.2	23.8	5	0	0	2	959.54
									SCNCLLLK	95.0%	47.0	22.3	13	0	0	2	1,007.50
									SGETEDTFIADLVVGLCTGQIK	95.0%	98.1	22.2	6	0	0	2	2,353.16
									SGKYDLDFK	95.0%	50.1	21.5	10	54	0	2	1,072.53
									SPDDPSR	95.0%	44.9	16.5	5	0	0	2	773.34
									TIAPALVSK	95.0%	49.6	15.9	13	0	0	2	899.56
									VNQIGSVTESLQACK	95.0%	139.0	22.8	186	2	0	2	1,633.82
									VVIGMDVAASEFFR	95.0%	117.0	22.6	349	0	0	2	1,556.78
									YDLDFK	95.0%	38.8	19.7	5	0	0	2	800.38
									YDLDFKSPDDPSR	95.0%	68.8	20.5	11	6	0	2	1,554.71
									YGKDATNVGDEGGFAPNILENK	95.0%	52.0	21.7	0	4	0	2	2,309.11
									YISPDQLADLYK	95.0%	90.8	22.9	169	0	0	2	1,425.73
									YNQLLR	95.0%	37.6	22.8	68	0	0	2	806.45
N-acetylglucosamine-1-phosphotransferase subunit gamma	GNPTG_HUMAN	GNPTG	33,956	99.90%	2	2	4	13.40%	QWDQVEQDLADELITPQGHEK	95.0%	28.6	22.0	0	1	0	2	2,479.17
									VVEEPNAFGVNNPFLPQASR	95.0%	62.4	22.1	3	0	0	2	2,185.10
Follistatin-related protein 1	FSTL1_HUMAN	FSTL1	34,967	100.00%	5	5	70	14.90%	GAQTQTEEEMTR	95.0%	82.0	17.6	41	0	0	2	1,396.60
									ICANVFCGAGR	95.0%	49.8	21.5	7	0	0	2	1,224.56
									LDSSEFLK	95.0%	43.2	22.3	5	0	0	2	938.48
									LSFQEFLK	95.0%	57.1	21.0	16	0	0	2	1,011.55
									YVQELQK	95.0%	37.7	21.5	1	0	0	2	907.49
Protocadherin Fat 1	FAT1_HUMAN	FAT1	506,251	100.00%	5	5	10	1.55%	AEISEFAPPNTPVVMVK	95.0%	37.5	22.7	1	0	0	2	1,844.95
									AFEVNPQGVLLTSSAIK	95.0%	67.6	19.3	6	0	0	2	1,773.98
									IGEETGVIETSDR	95.0%	47.4	22.3	1	0	0	2	1,405.68
									IVSGDSENLFK	95.0%	31.8	23.3	1	0	0	2	1,208.62
									VQATDADAGLNRK	95.0%	27.1	22.9	0	1	0	2	1,358.70
Annexin A10	ANX10_HUMAN	ANXA10	37,261	100.00%	10	10	50	31.80%	DKPAYFAYR	95.0%	38.2	22.4	2	0	0	2	1,130.56
									DTLMNLVQGTR	95.0%	65.0	23.6	9	0	0	2	1,247.64
									GVGTDENCLIEILASR	95.0%	92.1	22.2	4	0	0	2	1,746.87
									LYSAIHDFGFHNK	95.0%	19.6	22.6	0	0	1	2	1,548.76
									MMIAEAYQSMYGR	95.0%	102.0	16.1	6	0	0	2	1,598.67
									SEIDLLTIR	95.0%	71.2	21.9	20	0	0	2	1,059.61
									SLFHDIR	95.0%	34.0	22.6	1	0	0	2	887.47
									SYQQLR	95.0%	34.0	22.0	1	0	0	2	794.42
									TMLQMILCNK	95.0%	46.6	22.9	2	0	0	2	1,251.63
									TNGEIFQMR	95.0%	71.1	22.0	4	0	0	2	1,095.53
Adenylosuccinate synthetase isozyme 2	PURA2_HUMAN	ADSS	50,080	100.00%	6	7	17	18.60%	ELPVNAQNYVR	95.0%	37.0	23.2	2	0	0	2	1,302.68
									FIEDELQIPVK	95.0%	52.9	22.5	5	0	0	2	1,330.73
									LDGEIIPHIPANQEVLNK	95.0%	38.8	18.9	0	2	0	2	2,000.08
									LDILDMFTEIK	95.0%	89.6	21.8	2	0	0	2	1,353.70
									TLPGWNTDISNAR	95.0%	60.0	22.3	2	0	0	2	1,444.72
									VGIGAFPTEQDNEIGELLQTR	95.0%	107.0	21.0	2	2	0	2	2,287.16
Annexin A3	ANXA3_HUMAN	ANXA3	36,359	100.00%	17	20	179	48.00%	ALLTLADGR	95.0%	64.9	21.2	13	0	0	2	929.54
									ALLTLADGRR	95.0%	36.2	19.4	2	2	0	2	1,085.64
									DISQAYYTVYK	95.0%	64.5	22.6	11	0	0	2	1,350.66
									DISQAYYTVYKK	95.0%	44.0	22.8	4	0	0	2	1,478.75
									DYPDFSPSVDAEAIQK	95.0%	85.8	21.1	19	0	0	2	1,781.82

									EYQAAYGK	95.0%	48.3	18.5	3	0	0	2	929.44
									GAGTNEDALIEILTTR	95.0%	117.0	22.4	35	3	0	2	1,673.87
									GIGTDEFTLNR	95.0%	75.0	23.3	14	0	0	2	1,222.61
									HYGYSLYSAIK	95.0%	39.7	22.2	1	0	0	2	1,301.65
									MLISILTER	95.0%	66.4	21.0	23	0	0	2	1,075.62
									NTPAFLAER	95.0%	41.2	24.0	2	0	0	2	1,018.53
									QDAQILYK	95.0%	58.5	22.6	8	0	0	2	978.53
									SDTSGDYEITLLK	95.0%	87.1	21.8	14	0	0	2	1,441.71
									SEIDLLDIR	95.0%	73.7	24.9	10	0	0	2	1,073.58
									SLGDDISSETSGDFR	95.0%	83.3	18.9	5	0	0	2	1,585.70
									SLGDDISSETSGDFRK	95.0%	85.4	20.8	4	2	0	2	1,713.79
									VDEHLAK	95.0%	43.7	19.0	4	0	0	2	811.43
Proteasome subunit beta type-2	PSB2_HUMAN	PSMB2	22,820	100.00%	6	6	31	29.90%	AVELLR	95.0%	38.8	22.0	3	0	0	2	700.44
									FILNLPTFSVR	95.0%	56.0	17.0	10	0	0	2	1,306.75
									NGIHDLDNISFPK	95.0%	67.6	22.3	2	0	0	2	1,469.74
									NGYELSPTAAANFTR	95.0%	90.0	22.0	2	0	0	2	1,611.78
									VAASNIVQMK	95.0%	75.8	23.4	12	0	0	2	1,076.58
									VAASNIVQMKDDHDK	95.0%	67.9	21.6	2	0	0	2	1,686.81
Cathepsin H	CATH_HUMAN	CTSH	37,376	100.00%	8	10	161	25.10%	GIMGEDTYPYQGK	95.0%	87.2	20.1	52	0	0	2	1,458.66
									GNFVSPVK	95.0%	30.6	23.6	2	0	0	2	847.47
									GTGPYPPSVDWR	95.0%	44.8	21.6	18	0	0	2	1,331.64
									LQTFASNWR	95.0%	44.0	23.3	15	0	0	2	1,122.57
									MALNQFSDMSFAEIK	95.0%	107.0	20.7	55	0	0	2	1,731.81
									TPDKVNHAVLAVGYGEK	95.0%	61.2	20.4	1	8	3	2	1,797.95
									TYSTEEYHHR	95.0%	42.3	18.7	1	0	0	2	1,322.58
									VNHAVLAVGYGEK	95.0%	59.7	22.1	6	0	0	2	1,356.73
Cathepsin L2	CATL2_HUMAN	CTSL2	37,311	100.00%	3	3	24	10.20%	FDQNLDTK	95.0%	42.0	22.0	11	0	0	2	980.47
									NSWGPEWGSNGYVK	95.0%	42.2	20.9	2	0	0	2	1,580.71
									VFREPLFLDLPK	95.0%	42.9	18.2	0	11	0	2	1,473.85
Ras suppressor protein 1	RSU1_HUMAN	RSU1	31,524	100.00%	4	4	8	18.40%	ALYLSDNDFEILPPDIGK	95.0%	46.5	22.3	2	0	0	2	2,020.03
									EIGELTQLK	95.0%	36.7	21.9	2	0	0	2	1,030.58
									LQILSLR	95.0%	35.0	19.6	2	0	0	2	842.55
									LTVLPPELGNLDLTGQK	95.0%	48.1	15.7	2	0	0	2	1,808.02
Lipolysis-stimulated lipoprotein receptor	LSR_HUMAN	LSR	71,421	100.00%	5	5	238	10.30%	DTDSSVASEVR	95.0%	70.6	21.0	28	0	0	2	1,165.53
									ELANFDPSRPGPPSGR	95.0%	29.9	22.6	0	1	0	2	1,696.84
									IQASQQDDSMR	95.0%	77.9	19.1	102	0	0	2	1,294.57
									QGNAVTLGDYYQGR	95.0%	92.0	22.1	44	0	0	2	1,541.73
									SSSAGGQGSYVPLLR	95.0%	102.0	22.6	63	0	0	2	1,478.76
FACT complex subunit SPT16	SP16H_HUMAN	SUPT16H	119,899	100.00%	3	3	10	3.25%	AASITSEVFNK	95.0%	75.9	22.7	6	0	0	2	1,166.61
									GNENANGAPAITLLIR	95.0%	67.8	20.2	2	0	0	2	1,623.88
									LAESVEK	95.0%	38.6	25.1	2	0	0	2	775.42
ELAV-like protein 1	ELAV1_HUMAN	ELAVL1	36,075	100.00%	3	3	10	12.90%	SLFSSIGEVESAK	95.0%	51.3	22.1	3	0	0	2	1,353.69
									TNLIVNYLPQNMTQDELR	95.0%	46.6	21.8	1	0	0	2	2,162.09
									VLVDQTTGLSR	95.0%	85.5	22.3	6	0	0	2	1,188.66
Interleukin enhancer-binding factor 3	ILF3_HUMAN	ILF3	95,321	100.00%	5	5	21	7.27%	AYAALAALEK	95.0%	47.8	20.2	1	0	0	2	1,020.57
									EDITQSAQHALR	95.0%	40.7	23.4	1	0	0	2	1,368.69
									LNQLKPGLQYK	95.0%	42.6	18.4	2	0	0	2	1,301.76
									VLAGETLSVNDPPDVLDR	95.0%	83.1	21.2	7	0	0	2	1,909.99
									VLQDMGLPTGAEGR	95.0%	59.9	23.2	10	0	0	2	1,459.72
Palmitoyl-protein thioesterase 1	PPT1_HUMAN	PPT1	34,176	100.00%	4	4	34	21.20%	CPSPPMINLISVGGQHQGVFGLPR	95.0%	38.4	20.4	0	1	0	2	2,577.31
									ETIPLQETSLYTQDR	95.0%	88.7	22.6	16	0	0	2	1,793.89

									GINESYKK	95.0%	34.1	20.3	2	0	0	2	938.49
									LQQGYNAMGFSQGGQFLR	95.0%	121.0	21.8	15	0	0	2	2,001.96
Ornithine aminotransferase, mitochondrial	OAT_HUMAN	OAT	48,518	100.00%	4	4	10	12.30%	GIYLWDVEGR	95.0%	52.8	22.0	2	0	0	2	1,207.61
									LPSDVVTAVR	95.0%	54.7	19.8	4	0	0	2	1,056.61
									TVQGPPTSDDIFER	95.0%	76.6	22.5	2	0	0	2	1,561.75
									VAIAALEVLEEENLAENADK	95.0%	94.3	22.0	2	0	0	2	2,141.10
X-ray repair cross-complementing protein 5	XRCC5_HUMAN	XRCC5	82,689	100.00%	8	10	25	11.10%	ANPQVGVAFPHIK	95.0%	46.9	20.1	2	0	0	2	1,377.76
									EEASGSSVTAEEAK	95.0%	103.0	20.1	6	0	0	2	1,394.63
									EEASGSSVTAEEAKK	95.0%	75.7	21.9	4	0	0	2	1,522.72
									FSEEQR	95.0%	31.3	20.8	3	0	0	2	795.36
									LGGHGPSFPLK	95.0%	39.2	20.8	2	1	0	2	1,109.61
									VITMFVQR	95.0%	32.7	21.6	1	0	0	2	1,009.55
									YAPTEAQLNAVDALIDSMSLAK	95.0%	113.0	21.6	3	2	0	2	2,321.17
									YAYDKR	95.0%	32.0	20.5	1	0	0	2	815.41
Ribonuclease inhibitor	RINI_HUMAN	RNH1	49,956	100.00%	8	8	28	23.00%	ELCQGLGQPGSVLR	95.0%	52.8	22.1	3	0	0	2	1,513.78
									ELSLAGNELGDEGAR	95.0%	110.0	21.9	4	0	0	2	1,530.74
									ELTVSNNDINEAGVR	95.0%	112.0	22.3	4	0	0	2	1,630.80
									LDDCGLTEAR	95.0%	51.9	21.0	2	0	0	2	1,149.52
									LEDAGVR	95.0%	40.6	23.4	1	0	0	2	759.40
									LGDVGMAELCPGLLHPSSR	95.0%	57.3	22.1	0	3	0	2	2,024.99
									VNPALAELNLR	95.0%	69.9	18.2	7	0	0	2	1,209.70
									WAELLPLLQQCQVVR	95.0%	62.4	19.1	4	0	0	2	1,853.01
Dystroglycan	DAG1_HUMAN	DAG1	97,424	100.00%	7	8	63	12.40%	EGAMSAQLGYPVVGWHIANK	95.0%	49.7	22.5	1	2	0	2	2,144.06
									IPSDTFYDHEDTTTDKLK	95.0%	29.9	21.6	0	3	0	2	2,125.99
									LFDMSAFMAGPGNAK	95.0%	95.9	20.1	23	0	0	2	1,588.71
									LGCSLNQNSVPDIHGVEAPAR	95.0%	45.8	22.1	0	3	0	2	2,234.10
									LREQQLVGEK	95.0%	51.9	22.0	3	0	0	2	1,199.68
									SFSEVELHNMK	95.0%	44.5	21.4	7	0	0	2	1,336.62
									VTIPTDLIASSGDIIK	95.0%	122.0	18.2	21	0	0	2	1,642.93
Stathmin	STMN1_HUMAN	STMN1	17,285	100.00%	4	5	9	30.90%	AIEENNNFSK	95.0%	53.6	21.8	2	0	0	2	1,165.55
									ASGQAFELILSPR	95.0%	35.1	22.4	1	0	0	2	1,388.75
									DLSLEEIQK	95.0%	48.3	24.0	2	0	0	2	1,074.57
									SKESVPEFPLSPPK	95.0%	36.2	21.7	2	2	0	2	1,541.82
Selenide, water dikinase 1	SPS1_HUMAN	SEPHS1	42,893	99.50%	2	2	4	8.42%	ESFNPESYELDK	95.0%	60.4	19.1	2	0	0	2	1,457.64
									IIEVAPQVATQNVNPTPGATS	95.0%	71.3	21.3	2	0	0	2	2,107.10
Dihydropteridine reductase	DHPR_HUMAN	QDPR	25,772	100.00%	2	2	5	14.30%	MTDSFTEQADQVTAEVGK	95.0%	141.0	19.4	3	0	0	2	1,972.88
									NRPSSGSLIQVVTTEGR	95.0%	55.6	20.8	0	2	0	2	1,800.96
Polymerase I and transcript release factor	PTRF_HUMAN	PTRF	43,459	100.00%	8	10	30	22.30%	AHATTSNTVSK	95.0%	32.2	22.0	1	0	0	2	1,116.56
									GEAGDLR	95.0%	32.4	20.8	1	0	0	2	717.35
									IIGAVDQIQLTQAQLEER	95.0%	148.0	19.0	6	4	0	2	2,025.10
									IREGQVEVLK	94.9%	26.5	19.5	0	1	0	2	1,170.68
									KLEVNEAELLR	95.0%	65.7	19.9	4	3	0	2	1,313.74
									QAEMEGAVQSIQGELSK	95.0%	130.0	22.2	7	0	0	2	1,804.88
									VMIYQDEVK	95.0%	33.7	23.2	1	0	0	2	1,140.56
									VMIYQDEVKLPAK	95.0%	30.6	21.4	0	2	0	2	1,549.83
Cathepsin Z	CATZ_HUMAN	CTSZ	33,850	100.00%	5	6	52	19.50%	IVTSTYKDGK	95.0%	36.0	20.6	1	0	0	2	1,111.60
									NSWGEPWGER	95.0%	40.2	17.6	2	0	0	2	1,217.53
									NVDGVNYASITR	95.0%	80.3	22.6	26	0	0	2	1,308.65
									STYPRPHEYLSPADLPK	95.0%	36.8	22.2	0	9	2	2	1,971.00
									VGDYGSLSGR	95.0%	83.2	21.6	12	0	0	2	1,010.49
Myosin light polypeptide 6	MYL6_HUMAN	MYL6	16,912	100.00%	6	8	20	46.40%	ALGQNPTNAEVLK	95.0%	55.9	21.4	2	0	0	2	1,354.73

									EAFQLFDR	95.0%	52.8	22.6	2	0	0	2	1,025.51
									ILYSQCGDVMR	95.0%	67.4	20.5	2	0	0	2	1,357.62
									NKDQGTYEDYVEGLR	95.0%	79.1	21.5	2	2	0	2	1,786.83
									SDEMNVK	95.0%	31.0	20.9	1	0	0	2	822.37
									VLDFEHFLPMLQTVAK	95.0%	51.8	20.9	6	3	0	2	1,888.00
Plasminogen activator inhibitor 2	PAI2_HUMAN	SERPINB2	46,580	100.00%	13	14	66	36.40%	ANFSGMSER	95.0%	40.5	20.5	2	0	0	2	1,014.43
									GKIPNLLPEGSVDGDTR	95.0%	57.3	21.3	3	9	0	2	1,767.92
									GSYPDAILQAQAADK	95.0%	86.3	23.2	7	0	0	2	1,547.77
									IPNLLPEGSVDGDTR	95.0%	64.7	22.0	9	0	0	2	1,582.81
									ITNCILFFGR	95.0%	69.5	21.4	7	0	0	2	1,240.65
									LEEHYELR	95.0%	26.8	22.5	0	1	0	2	1,088.54
									LNIGYIEDLK	95.0%	73.3	21.7	10	0	0	2	1,177.65
									MAEDEVEVYIPQFK	95.0%	58.2	21.6	1	0	0	2	1,713.80
									MVLVNAVYFK	95.0%	43.0	19.4	1	0	0	2	1,183.65
									SLSSAINASTGNYLLESVNK	95.0%	106.0	21.4	8	0	0	2	2,068.06
									SMGMEDAFNK	95.0%	36.7	12.8	1	0	0	2	1,161.46
									TPVQMMYLR	95.0%	51.5	23.2	4	0	0	2	1,138.58
									YYSSEPQAVDFLECAEEAR	95.0%	110.0	17.8	3	0	0	2	2,263.98
Transcriptional activator protein Pur-beta	PURB_HUMAN	PURB	33,224	100.00%	3	3	5	24.40%	DSLGDFIEHYAQLGPSSPEQLAAGAEEGGGPR	95.0%	45.0	20.3	0	1	0	2	3,255.52
									GGGEQETQELASK	95.0%	43.6	22.4	2	0	0	2	1,333.62
									GGGGFGAGPGPGGLQSGQTIALPAQGLIEFR	95.0%	47.4	19.8	0	2	0	2	2,867.48
V-type proton ATPase subunit S1	VAS1_HUMAN	ATP6AP1	52,009	99.50%	2	2	7	6.81%	EVLTGNDEVIGQVLSTLK	95.0%	73.2	19.2	5	0	0	2	1,915.04
									LGASPLHVDLATLR	95.0%	39.7	18.3	0	2	0	2	1,462.84
Actin-related protein 2/3 complex subunit 3	ARPC3_HUMAN	ARPC3	20,530	100.00%	2	2	3	13.50%	DTDIVDEAIYYFK	95.0%	68.1	22.2	1	0	0	2	1,591.75
									LIGNMALLPIR	95.0%	61.2	15.1	2	0	0	2	1,210.73
DAZ-associated protein 1	DAZP1_HUMAN	DAZAP1	43,365	99.50%	2	2	4	7.62%	LFVGGLDWSTTQETLR	95.0%	112.0	21.8	2	0	0	2	1,822.93
									SQAPGQPGASQWGSR	95.0%	77.4	21.3	2	0	0	2	1,513.71
60 kDa SS-A/Ro ribonucleoprotein	RO60_HUMAN	TROVE2	60,654	100.00%	5	6	17	10.80%	ALLQEMPLTALLR	95.0%	77.3	16.3	8	0	0	2	1,468.86
									LGLENAEALIR	95.0%	72.8	21.6	4	0	0	2	1,198.68
									QIANSQDGYVWQVTDMNR	95.0%	75.2	21.4	1	0	0	2	2,124.98
									SFSQEGR	95.0%	35.8	20.8	1	0	0	2	810.37
									WRPDEEILK	95.0%	55.3	21.6	2	1	0	2	1,185.63
Syntenin-1	SDCB1_HUMAN	SDCBP	32,427	100.00%	3	3	7	21.50%	DSTGHVGFIFK	95.0%	36.1	22.0	4	0	0	2	1,207.61
									LYPELSQYMGLSLNEEEIR	95.0%	90.3	22.1	2	0	0	2	2,300.11
									VIQAQTAFSANPANPAILSEASAPIPHDGNLYPR	95.0%	36.5	18.8	0	1	0	2	3,530.80
Uncharacterized protein LP9056	L9056_HUMAN	LP9056	63,530	100.00%	5	5	32	9.24%	ALFPSLGTYDLEK	95.0%	48.7	22.2	9	0	0	2	1,453.76
									AMEQEFSATK	95.0%	66.7	21.4	10	0	0	2	1,141.52
									GLLYQLFR	95.0%	39.7	19.1	5	0	0	2	1,009.58
									KDHVLDIPTTQR	95.0%	29.2	20.8	0	1	0	2	1,422.77
									VSSLASASR	95.0%	75.0	23.3	7	0	0	2	877.47
EGF-containing fibulin-like extracellular matrix protein 1	FBLN3_HUMAN	EFEMP1	54,621	100.00%	11	11	120	23.10%	ADQVCINLR	95.0%	49.2	23.4	3	0	0	2	1,088.55
									ELPQSIVYK	95.0%	35.7	21.8	1	0	0	2	1,076.60
									FSCMCPQGYQVVR	95.0%	58.7	17.2	2	0	0	2	1,647.71
									GSFACQCPPGYQK	95.0%	84.6	17.2	14	0	0	2	1,499.64
									IPSNPSHR	95.0%	45.5	22.9	13	0	0	2	907.48
									LNCEDIDECR	95.0%	53.9	13.4	1	0	0	2	1,323.53
									NNFVIR	95.0%	35.2	23.7	3	0	0	2	762.43
									NPCQDPYILTPENR	95.0%	63.8	21.3	9	0	0	2	1,716.80
									QTSPVSAMLVLVK	95.0%	76.1	19.8	58	0	0	2	1,372.79
									RNPADPQR	95.0%	35.9	22.2	2	0	0	2	953.49
									SGNENGEFYLR	95.0%	53.4	19.7	14	0	0	2	1,285.58

UPF0160 protein MYG1, mitochondrial	MYG1_HUMAN	C12orf10	42,432	99.50%	2	2	4	6.91%	AMDLVQEEFLQR	95.0%	83.4	22.5	2	0	0	2	1,494.73
									FQVDPSGEIVELAK	95.0%	42.0	23.6	2	0	0	2	1,531.80
26S proteasome non-ATPase regulatory subunit 13	PSD13_HUMAN	PSMD13	42,901	100.00%	3	3	6	11.20%	LNIGDLQVTK	95.0%	42.0	21.5	3	0	0	2	1,100.63
									QMTDPNVALTFLEK	95.0%	55.7	22.9	2	0	0	2	1,622.81
									TAWGQQPDLAANEAQLLR	95.0%	106.0	21.7	1	0	0	2	1,982.01
Acyl-coenzyme A thioesterase 1	ACOT1_HUMAN	ACOT1	46,260	99.50%	2	2	4	7.36%	DGYADIVDVLNSPLEGPDQK	95.0%	91.8	22.0	2	0	0	2	2,145.04
									GPGVGLLGISK	95.0%	62.2	14.6	2	0	0	2	997.60
Glutaminyl-tRNA synthetase	SYQ_HUMAN	QARS	87,782	99.50%	2	2	6	2.58%	FDDTNPEKEEAK	95.0%	36.9	20.5	2	0	0	2	1,422.64
									LFTLTALR	95.0%	46.2	14.9	4	0	0	2	934.57
Leucine-rich alpha-2-glycoprotein	A2GL_HUMAN	LRG1	38,162	100.00%	3	3	15	11.00%	DLLLPQPDLR	95.0%	35.7	19.0	2	0	0	2	1,179.67
									TLDLGENQLETLPPDLLR	95.0%	66.7	20.0	7	0	0	2	2,037.09
									VAAGAFQGLR	95.0%	74.9	22.0	6	0	0	2	989.55
Desmoglein-2	DSG2_HUMAN	DSG2	122,276	100.00%	18	21	221	18.30%	AWITAPVALR	95.0%	38.5	18.1	5	0	0	2	1,097.65
									DGNGEVTDKPVK	95.0%	56.6	22.4	4	0	0	2	1,258.63
									DNWISVDSVTSEIK	95.0%	56.2	23.1	4	0	0	2	1,592.78
									EETPFFLLTGYALDAR	95.0%	43.0	22.1	1	0	0	2	1,842.93
									GITEPPFGIFVFNK	95.0%	79.7	20.4	17	0	0	2	1,565.84
									GNNVEKPLELR	95.0%	56.4	21.5	6	3	0	2	1,268.70
									GQIIGNFQAFDEDTGLPAHAR	95.0%	119.0	21.2	4	18	0	2	2,257.10
									ILDVNDNIPVVENK	95.0%	82.4	20.9	35	0	0	2	1,581.85
									IVAISEDYPR	95.0%	68.1	23.2	12	0	0	2	1,162.61
									IVAISEDYPRK	95.0%	30.1	21.6	0	1	0	2	1,290.71
									IVSLEPAYPPVFYLNK	95.0%	54.9	18.6	44	0	0	2	1,850.01
									LEDRDNWISVDSVTSEIK	95.0%	83.2	22.7	4	16	0	2	2,106.04
									LPDFESR	95.0%	34.5	24.0	3	0	0	2	863.43
									QAQVQIR	95.0%	37.0	20.0	2	0	0	2	842.48
									QESTSVLLQQSEK	95.0%	99.2	22.9	10	0	0	2	1,476.75
									QESTSVLLQQSEKK	95.0%	38.8	22.3	2	0	0	2	1,604.85
									VLEGMVEENQVNVEVTR	95.0%	76.8	22.5	28	0	0	2	1,944.97
									VVPSFLPVDQGGSLVGR	95.0%	55.9	19.5	2	0	0	2	1,726.95
Beta-lactamase-like protein 2	LACB2_HUMAN	LACTB2	32,789	100.00%	2	2	9	9.38%	EQQILTLFR	95.0%	55.8	20.4	7	0	0	2	1,147.65
									ILIDTGEPAIPEYISCLK	95.0%	64.9	21.7	2	0	0	2	2,032.07
SH3 domain-binding glutamic acid-rich-like protein	SH3L1_HUMAN	SH3BGRL	12,757	100.00%	6	6	11	78.10%	DIAANEENRK	95.0%	35.3	22.3	1	0	0	2	1,159.57
									ENNAVYAFLGLTAPPGSK	95.0%	52.7	22.0	2	0	0	2	1,848.95
									ENVPENSRPATGYPLPPQIFNESQYR	95.0%	53.1	20.1	0	2	0	2	3,003.46
									GDYDAFFEAR	95.0%	41.3	16.3	2	0	0	2	1,190.51
									QQDVLGFLEANK	95.0%	77.8	23.9	2	0	0	2	1,361.71
									VYIASSSGSTAIK	95.0%	45.2	21.5	2	0	0	2	1,283.68
Hsc70-interacting protein	F10A1_HUMAN	ST13	41,314	100.00%	3	3	23	10.00%	AIDLFTDAIK	95.0%	57.5	22.0	10	0	0	2	1,106.61
									AIEINPDSAQPYK	95.0%	53.8	23.0	3	0	0	2	1,445.73
									VAAIEALNDGELQK	95.0%	109.0	21.8	10	0	0	2	1,470.78
Nicotinate phosphoribosyltransferase	PNCB_HUMAN	NAPRT1	57,561	100.00%	3	3	5	7.06%	AAFVAYALAFPR	94.5%	30.0	19.5	1	0	0	2	1,296.71
									LDSGDLLQQAQEIR	95.0%	87.6	22.0	2	0	0	2	1,585.82
									SPAQYQVVLSER	95.0%	65.9	22.6	2	0	0	2	1,376.72
Matrix metalloproteinase-28	MMP28_HUMAN	MMP28	58,923	99.50%	2	2	5	5.19%	AFQWVSQLPVSGVLDR	95.0%	71.0	21.4	2	0	0	2	1,801.96
									GGLQVEPYYPR	95.0%	52.3	22.3	3	0	0	2	1,278.65
Charged multivesicular body protein 4b	CHM4B_HUMAN	CHMP4B	24,933	99.50%	2	2	4	11.20%	EALENANTNTEVLK	95.0%	45.0	23.0	2	0	0	2	1,545.78
									GGPTPQEAIQR	95.0%	45.9	22.8	2	0	0	2	1,153.60
4F2 cell-surface antigen heavy chain	4F2_HUMAN	SLC3A2	67,978	100.00%	6	6	29	13.70%	ADLLLSTQPGREEGSPLELER	95.0%	37.2	20.8	0	1	0	2	2,310.19
									GENSWFSTQVDTVATK	95.0%	56.3	21.3	2	0	0	2	1,769.83
									GQSEDPGSLLSLFR	95.0%	85.3	22.7	12	0	0	2	1,505.76

									LLTSFLPAQLLR	95.0%	40.9	12.0	8	0	0	2	1,371.84
									VAEDEAEAAAAAK	95.0%	32.0	21.8	1	0	0	2	1,245.60
									VILDLTPNYR	95.0%	50.5	21.5	5	0	0	2	1,203.67
Mitotic checkpoint protein BUB3	BUB3_HUMAN	BUB3	37,137	100.00%	6	6	9	19.80%	LNQPPEDGISSVK	95.0%	41.4	22.8	2	0	0	2	1,383.71
									LYDVPANSMR	95.0%	36.7	21.7	1	0	0	2	1,181.56
									QGYVLSSIEGR	95.0%	49.5	22.6	1	0	0	2	1,208.63
									QVTDAETKPK	95.0%	32.9	24.0	1	0	0	2	1,116.59
									VAVEYLDPSPEVQK	95.0%	59.4	23.2	2	0	0	2	1,573.81
									VLVWDLR	95.0%	33.5	22.0	2	0	0	2	900.53
Serpin B6	SPB6_HUMAN	SERPINB6	42,605	100.00%	6	7	45	23.40%	ELNMIIMLPDETTDLR	95.0%	79.4	22.4	3	0	0	2	1,935.94
									GNTAAQMAQILSFNK	95.0%	98.8	23.1	11	0	0	2	1,593.81
									IAELLSPGSVDPLTR	95.0%	76.0	19.3	14	0	0	2	1,567.87
									LVLVNAVYFR	95.0%	50.2	16.8	4	0	0	2	1,193.70
									NLGMTDAFELGK	95.0%	56.1	21.8	6	0	0	2	1,295.63
									SGGGGDIHQGFQSLLTEVNK	95.0%	86.9	21.8	2	5	0	2	2,044.01
Splicing factor, arginine/serine-rich 6	SFRS6_HUMAN	SFRS6	39,570	100.00%	5	5	21	14.00%	ALDKLDGTEINGR	95.0%	67.7	22.5	2	0	0	2	1,401.73
									LIEDKPR	95.0%	43.6	18.0	1	0	0	2	870.51
									LIVENLSSR	95.0%	52.6	21.6	11	0	0	2	1,030.59
									QAGEVTYADAHK	95.0%	51.6	21.4	3	0	0	2	1,289.61
									VIVEHAR	95.0%	44.6	18.7	4	0	0	2	823.48
L-lactate dehydrogenase B chain	LDHB_HUMAN	LDHB	36,621	100.00%	13	15	160	42.20%	EKLIAPVAEEEATVPNNK	95.0%	79.2	21.4	1	0	0	2	1,952.03
									FIIPQIVK	95.0%	42.0	16.0	3	0	0	2	957.61
									GEMMDLQHGSLFLQTPK	95.0%	53.4	21.9	2	6	0	2	1,963.93
									GLTSVINQK	95.0%	53.7	22.1	14	0	0	2	959.55
									IVADKDYSVTANSK	95.0%	97.5	21.2	13	5	0	2	1,510.78
									IVVVTAGVR	95.0%	61.8	17.0	18	0	0	2	913.58
									LIAPVAEEEATVPNNK	95.0%	88.2	20.5	20	0	0	2	1,694.90
									LKDDEVAQLK	95.0%	45.0	23.5	5	0	0	2	1,158.64
									LKGEMMDLQHGSLFLQTPK	95.0%	29.6	21.5	0	0	2	2	2,205.11
									LNLVQR	95.0%	48.6	18.8	25	0	0	2	742.46
									MVVESAYEVIK	95.0%	76.7	21.9	21	0	0	2	1,267.66
									SADTLWDIQK	95.0%	75.9	23.4	22	0	0	2	1,176.59
									SLADELALVDVLEDK	95.0%	115.0	21.6	27	0	0	2	1,629.86
									SLADELALVDVLEDKLK	95.0%	37.2	17.6	1	0	0	2	1,871.04
									VIGSGCNLDSAR	95.0%	73.9	21.7	3	0	0	2	1,248.60
Protein arginine N-methyltransferase 5	ANM5_HUMAN	PRMT5	72,667	99.50%	2	2	4	3.61%	AAILPTSIFLTNK	95.0%	48.2	16.7	3	0	0	2	1,388.82
									VPLVAPEDLR	95.0%	52.0	19.0	1	0	0	2	1,108.64
U2 small nuclear ribonucleoprotein A'	RU2A_HUMAN	SNRPA1	28,399	100.00%	3	3	6	15.70%	LTAELIEQAAQYTNAVR	95.0%	77.8	22.6	2	0	0	2	1,890.99
									NAIANASTLAEVER	95.0%	63.1	22.9	2	0	0	2	1,458.76
									SLTYLSILR	95.0%	53.4	17.0	2	0	0	2	1,065.63
Sodium/potassium-transporting ATPase subunit alpha-1	AT1A1_HUMAN	ATP1A1	112,882	100.00%	7	7	35	9.78%	AVFQANQENLPILK	95.0%	52.3	19.5	4	0	0	2	1,584.88
									GVGIISEGNETVEDIAAR	95.0%	103.0	22.0	2	0	0	2	1,829.92
									NMVPQQALVIR	95.0%	34.8	21.4	1	0	0	2	1,284.71
									QGAIVAVTGDGVNDSPALK	95.0%	87.6	20.8	4	0	0	2	1,811.95
									SPDFTNENPLETR	95.0%	89.9	20.6	5	0	0	2	1,519.70
									TSATWLALSR	95.0%	86.7	22.9	2	0	0	2	1,105.60
									VDNSSLTGESEPQTR	95.0%	89.7	21.3	17	0	0	2	1,619.75
Aminopeptidase B	AMPB_HUMAN	RNPEP	72,579	100.00%	13	14	34	28.20%	AEFGPPGPGAGSR	95.0%	39.7	22.5	4	0	0	2	1,199.58
									AFFPCFDTPAVK	95.0%	31.7	22.2	1	0	0	2	1,399.67
									AIEAVAISPWK	95.0%	65.7	20.6	2	0	0	2	1,184.67
									EEYNGVIEEFLATGEK	95.0%	72.0	22.4	3	0	0	2	1,827.87

									ETFASTASQLHSNVVNYVQQIVAPK	95.0%	30.0	20.4	0	3	0	2	2,731.41
									IEPGVDPDDTYNETPYEK	95.0%	106.0	17.9	4	0	0	2	2,081.92
									LGDTYPSISNAR	95.0%	42.5	23.2	2	0	0	2	1,293.64
									QHMDITGEENPLNK	95.0%	38.8	21.5	2	1	0	2	1,641.75
									RPLHSAQAVDVASASNFR	95.0%	33.3	21.0	0	1	0	2	1,925.99
									TYQLVYFLDK	95.0%	67.5	24.1	5	0	0	2	1,289.68
									VDIIPGFEFDR	95.0%	34.2	22.8	2	0	0	2	1,307.66
									VKIEPGVDPDDTYNETPYEK	95.0%	37.1	21.6	0	2	0	2	2,309.08
									YTLPLYHAMMGGSEVAQTLAK	95.0%	39.5	21.2	0	2	0	2	2,313.13
Delta-aminolevulinic acid dehydratase	HEM2_HUMAN	ALAD	36,277	99.50%	2	2	3	9.09%	AAVLEAMTAFR	95.0%	43.1	22.1	2	0	0	2	1,179.62
									GSAADSEESPAIEAIHLLR	95.0%	27.1	22.4	0	1	0	2	1,965.99
Eukaryotic translation initiation factor 3 subunit J	EIF3J_HUMAN	EIF3J	29,045	100.00%	4	5	9	20.50%	ETFGVNNAVYGIDAMNPSSR	95.0%	94.0	21.8	1	0	0	2	2,157.99
									LQEESDLELAK	95.0%	53.0	23.0	2	0	0	2	1,274.65
									RLEEPEEPK	95.0%	33.4	22.6	1	0	0	2	1,126.57
									VLTPEEQLADKLR	95.0%	58.9	20.6	3	2	0	2	1,511.84
Aminoacylase-1	ACY1_HUMAN	ACY1	45,866	100.00%	8	9	17	28.70%	AGFALDEGIANPTDAFTVFYSER	95.0%	58.0	21.1	1	0	0	2	2,491.18
									AVGVPALGFSPMNR	95.0%	32.6	23.3	2	0	0	2	1,431.74
									DMNLTLEPEIMPAATDNR	95.0%	68.6	20.9	1	0	0	2	2,062.94
									EGSVTSVNLTK	95.0%	50.4	22.7	2	0	0	2	1,134.60
									FMEDTAAEK	95.0%	49.7	19.1	2	0	0	2	1,057.45
									GPEEEHPSVTLFR	95.0%	62.1	22.5	2	1	0	2	1,497.73
									TVQPKPDYGAAVAFFEETAR	95.0%	45.8	21.4	0	2	0	2	2,197.09
									VVNSILAFR	95.0%	69.2	17.9	4	0	0	2	1,018.60
40S ribosomal protein SA	RSSA_HUMAN	RPSA	32,836	100.00%	6	6	38	24.10%	AIVAIENPADVSVISSR	95.0%	108.0	19.2	13	0	0	2	1,740.95
									DPEEIEKEEQAAAEK	95.0%	54.9	21.7	1	0	0	2	1,715.80
									FAAATGATPIAGR	95.0%	83.8	23.4	9	0	0	2	1,203.65
									FTPGTFTNQIQAAFR	95.0%	89.0	21.8	9	0	0	2	1,698.86
									KSDGIYIINLK	95.0%	31.2	17.4	1	0	0	2	1,263.73
									SDGIYIINLK	95.0%	59.6	21.6	5	0	0	2	1,135.64
Actin-related protein 2/3 complex subunit 2	ARPC2_HUMAN	ARPC2	34,316	100.00%	8	8	15	25.00%	ASHTAPQVLFSHR	95.0%	38.5	22.5	0	2	0	2	1,450.76
									DNTINLIHTFR	95.0%	38.6	22.5	2	0	0	2	1,343.71
									DSIVHQAGMLK	95.0%	49.2	21.8	2	0	0	2	1,198.63
									ELQAHGADELLK	95.0%	47.9	21.8	2	0	0	2	1,323.69
									ELQAHGADELLKR	95.0%	27.8	22.0	0	1	0	2	1,479.79
									IIEETLALK	95.0%	57.4	17.6	2	0	0	2	1,029.62
									MILLEVNNR	95.0%	49.8	22.8	3	0	0	2	1,101.61
									NCFASVFEK	95.0%	30.5	19.1	1	0	0	2	1,101.50
Thimet oligopeptidase	THOP1_HUMAN	THOP1	78,823	100.00%	9	10	24	14.90%	ETQENIKR	95.0%	34.1	23.2	1	0	0	2	1,017.53
									FYLDLYPR	95.0%	33.8	22.9	3	0	0	2	1,086.56
									LKPLGEQER	95.0%	34.1	20.3	0	2	0	2	1,069.60
									NILDFPQHVSPSK	95.0%	65.5	23.0	2	0	0	2	1,481.78
									QANTGLFNLR	95.0%	54.4	22.7	2	0	0	2	1,133.61
									TSQTVATFLDELAQK	95.0%	74.9	22.3	5	0	0	2	1,651.85
									VDQALHTQTDADPAEEYAR	95.0%	79.2	20.0	1	3	0	2	2,129.97
									WDLSAQQIEER	95.0%	68.5	21.3	2	0	0	2	1,374.67
									YYMNQVEETR	95.0%	53.0	18.7	3	0	0	2	1,332.59
Protein disulfide-isomerase A6	PDIA6_HUMAN	PDIA6	48,104	100.00%	6	6	19	21.80%	DGELPVEDDIDLSDVELDDLGKDEL	95.0%	66.5	20.1	2	0	0	2	2,758.27
									GESPVDYDGGR	95.0%	40.7	18.4	2	0	0	2	1,151.50
									GSTAPVGGGAFPTIVER	95.0%	97.8	22.8	2	0	0	2	1,615.84
									LAAVDATVNQVLASR	95.0%	101.0	20.6	3	0	0	2	1,527.85
									NLEPEWAAAASEVK	95.0%	31.3	22.2	1	0	0	2	1,514.75

									TGEAIVDAALSALR	95.0%	97.7	22.3	9	0	0	2	1,386.76
Delta(3,5)-Delta(2,4)-dienoyl-CoA isomerase, mitochondrial	ECH1_HUMAN	ECH1	35,798	100.00%	5	5	15	17.10%	MMADEALGSGLVSR	95.0%	98.7	21.5	6	0	0	2	1,468.68
									SPVAVQSTK	95.0%	53.3	23.0	2	0	0	2	916.51
									VIGNQSLVNELAFTAR	95.0%	79.9	20.6	2	0	0	2	1,731.94
									VNLLYSR	95.0%	47.9	20.7	4	0	0	2	864.49
									YQETFNVIER	95.0%	32.0	21.9	1	0	0	2	1,298.64
Neuropilin-2	NRP2_HUMAN	NRP2	104,843	100.00%	2	2	6	2.69%	SGEIAIDDIR	95.0%	51.3	23.8	4	0	0	2	1,088.56
									VFQANNDATEVVLNK	95.0%	72.3	22.7	2	0	0	2	1,661.85
Antileukoproteinase	SLPI_HUMAN	SLPI	14,308	100.00%	6	6	11	41.70%	AGVCPPK	95.0%	43.4	21.0	2	0	0	2	728.38
									CCMGMCGK	95.0%	50.6	0.0	3	0	0	2	1,019.32
									CLDPVDTPNPTR	95.0%	66.8	21.9	2	0	0	2	1,384.65
									SAQCLR	95.0%	37.9	23.8	1	0	0	2	734.36
									SCVSPVK	95.0%	36.6	25.0	1	0	0	2	776.40
									YKKPECQSDWQCPGK	95.0%	45.6	19.9	0	2	0	2	1,910.85
5'-nucleotidase	5NTD_HUMAN	NT5E	63,351	99.50%	2	2	3	4.18%	FPILSANIK	95.0%	31.9	17.6	2	0	0	2	1,002.60
									GPLASQISGLYLPYK	95.0%	33.4	19.3	1	0	0	2	1,606.88
AP-2 complex subunit beta	AP2B1_HUMAN	AP2B1	104,537	100.00%	3	3	8	4.27%	LASQANIAQVLAELK	95.0%	58.9	17.6	2	0	0	2	1,568.90
									MEPLNNLQVAVK	95.0%	46.1	22.3	4	0	0	2	1,371.73
									NVEGQDMLYQSLK	95.0%	53.5	22.3	2	0	0	2	1,540.73
Adenylate kinase isoenzyme 1	KAD1_HUMAN	AK1	21,617	100.00%	5	5	9	27.80%	EVQQGEEFER	95.0%	43.2	21.2	2	0	0	2	1,250.57
									GFLIDGYPR	95.0%	38.9	22.1	2	0	0	2	1,037.54
									GQLVPLETVLDMLR	95.0%	64.7	20.2	3	0	0	2	1,599.88
									IIFVVGGPGSGK	95.0%	58.8	18.7	1	0	0	2	1,130.66
									VDDNEETIK	95.0%	47.9	21.2	1	0	0	2	1,062.50
Tumor protein D54	TPD54_HUMAN	TPD52L2	22,220	100.00%	5	5	18	42.70%	GLLSDSMTDVPVDTGVAAR	95.0%	103.0	22.2	4	0	0	2	1,919.94
									LGLSTLGELK	95.0%	59.0	17.9	8	0	0	2	1,030.62
									TPAVEGLTEAEEEELR	95.0%	62.3	21.6	1	0	0	2	1,772.86
									TSAALSTVGSAISR	95.0%	70.1	22.2	4	0	0	2	1,320.71
									VVGDRENGSDNLPSSAGSGDKPLSDPAPF	95.0%	32.2	20.0	0	1	0	2	2,885.36
Ribulose-phosphate 3-epimerase	RPE_HUMAN	RPE	24,910	99.50%	2	2	3	11.40%	IGPSILNSDLANLGAECLR	95.0%	91.9	20.9	2	0	0	2	2,013.04
									SVINLLR	95.0%	35.0	21.0	1	0	0	2	814.52
14-3-3 protein epsilon	1433E_HUMAN	YWHAE	29,157	100.00%	19	24	347	71.00%	AAFDDAIAELDTLSEESYK	95.0%	121.0	20.9	21	10	0	2	2,087.97
									AASDIAMTELPPTHPIR	95.0%	81.8	22.0	36	38	0	2	1,819.94
									DNLTLWTSDMQGDGEEQNK	95.0%	115.0	18.2	7	0	0	2	2,180.94
									DNLTLWTSDMQGDGEEQNKEALQDVEDENQ	95.0%	76.9	15.2	0	5	0	2	3,467.47
									DSTLIMQLLR	95.0%	79.3	21.3	113	0	0	2	1,189.66
									EAAENSLVAYK	95.0%	67.4	23.2	31	0	0	2	1,194.60
									EALQDVEDENQ	95.0%	65.5	17.1	5	0	0	2	1,289.55
									HLIPAANTGESK	95.0%	54.5	22.5	4	1	0	2	1,237.65
									IISSIEQK	95.0%	48.1	22.6	8	0	0	2	917.53
									IISSIEQKEENK	95.0%	76.5	22.6	8	0	0	2	1,417.75
									KEAAENSLVAYK	95.0%	58.1	22.0	5	0	0	2	1,322.70
									LAEQAER	95.0%	59.4	21.6	36	0	0	2	816.42
									LAEQAERYDEMVESMK	95.0%	49.0	20.1	1	9	0	2	1,928.87
									LICCDILDVLDK	95.0%	78.4	23.3	16	0	0	2	1,476.74
									LICCDILDVLDKHLIPAANTGESK	95.0%	34.9	20.2	0	0	7	2	2,695.38
									NLLSVAYK	95.0%	51.4	19.1	49	0	0	2	907.53
									QMVETELK	95.0%	58.5	24.0	19	0	0	2	993.49
									VAGMDVELTVEER	95.0%	104.0	22.5	63	0	0	2	1,463.71
									YDEMVESMK	95.0%	49.6	13.0	11	0	0	2	1,163.46
									YDEMVESMKK	95.0%	34.3	17.9	1	1	0	2	1,275.56

									YLAEFATGNDR	95.0%	62.7	21.1	21	0	0	2	1,256.59
									YLAEFATGNDRK	95.0%	55.1	22.4	19	0	0	2	1,384.69
T-complex protein 1 subunit epsilon	TCPE_HUMAN	CCT5	59,654	100.00%	3	3	15	7.02%	IADGYEQAAR	95.0%	91.7	23.5	12	0	0	2	1,093.53
									QQISLATQMVR	95.0%	59.0	23.1	2	0	0	2	1,290.68
									WVGGPEIELIAIATGGR	95.0%	33.8	19.8	1	0	0	2	1,738.95
CD59 glycoprotein	CD59_HUMAN	CD59	14,159	99.50%	2	3	5	18.80%	FEHCNFNDVTTR	95.0%	65.5	17.8	2	1	0	2	1,539.67
									LRENELTYYCCK	95.0%	45.6	20.6	2	0	0	2	1,648.75
Metalloproteinase inhibitor 3	TIMP3_HUMAN	TIMP3	24,128	99.90%	2	2	6	9.95%	EGPFGTLVYTIK	95.0%	55.4	20.6	4	0	0	2	1,324.72
									WDQLTLSQR	95.0%	43.7	23.9	2	0	0	2	1,146.59
Amiloride-sensitive amine oxidase [copper-containing]	ABP1_HUMAN	ABP1	85,360	100.00%	3	3	6	4.39%	DTVIVWPR	95.0%	49.4	21.0	2	0	0	2	985.55
									FYGSPEELAR	95.0%	34.0	22.6	2	0	0	2	1,168.56
									VVQPTLEQTQYSWER	95.0%	84.4	22.4	2	0	0	2	1,863.92
Filaggrin	FILA_HUMAN	FLG	435,128	100.00%	4	4	6	1.75%	HAETSSGGQAASSQEQAR	95.0%	40.3	20.1	0	2	0	2	1,801.81
									LAQAYYESTR	95.0%	40.0	21.5	2	0	0	2	1,201.59
									QGSSVSQDSDSEGHSEDSER	95.0%	30.5	12.8	0	1	0	2	2,122.84
									QSGTPHAETSSGGQAASSHEQAR	94.9%	18.6	19.1	0	0	1	2	2,281.02
Heat shock protein beta-1	HSPB1_HUMAN	HSPB1	22,765	100.00%	11	11	69	59.00%	AQLGGPEAAK	95.0%	54.9	20.5	5	0	0	2	941.51
									GPSWDPFR	95.0%	33.8	21.3	2	0	0	2	961.45
									KYTLPPGVDPTQVSSSLSPEGTLTVEAPMPK	95.0%	75.0	19.5	0	11	0	2	3,242.65
									LATQSNEITIPVTFESR	95.0%	80.1	21.2	11	0	0	2	1,905.99
									LFDQAFGLPR	95.0%	86.6	22.3	21	0	0	2	1,163.62
									QLSSGVSEIR	95.0%	61.5	23.6	4	0	0	2	1,075.57
									RVPFSLLR	95.0%	37.1	15.8	2	0	0	2	987.61
									TKDGVVEITGK	95.0%	28.2	22.1	0	1	0	2	1,146.64
									VPFSLLR	95.0%	49.2	19.3	3	0	0	2	831.51
									VSLDVNHFAPDELTVK	95.0%	70.7	21.7	8	0	0	2	1,783.92
									YTLPPGVDPTQVSSSLSPEGTLTVEAPMPK	95.0%	37.4	20.7	0	1	0	2	3,114.56
Heterogeneous nuclear ribonucleoprotein F	HNRPF_HUMAN	HNRNPF	45,654	100.00%	3	4	49	15.90%	ATENDIYNFFSPLNPVR	95.0%	74.0	22.4	23	1	0	2	1,996.98
									HSGPNSADSANDGFVR	95.0%	98.5	19.3	8	3	0	2	1,630.72
									ITGEAFVQFASQELAEK	95.0%	111.0	21.4	36	0	0	2	1,867.94
									VHIEIGPDGR	95.0%	44.3	22.2	3	1	0	2	1,092.58
									YIEVFK	95.0%	32.9	19.7	2	0	0	2	798.44
Proteasome subunit alpha type-1	PSA1_HUMAN	PSMA1	29,538	100.00%	12	14	60	44.10%	AMSIGAR	95.0%	44.8	23.7	3	0	0	2	721.37
									AQPAQPADEPAEK	95.0%	60.4	21.7	10	0	0	2	1,351.65
									AQPAQPADEPAEKADEPMEH	95.0%	75.1	17.8	5	0	0	2	2,176.95
									AQSELAAHQK	95.0%	33.8	21.2	2	0	0	2	1,082.56
									ETLPAEQDLTTK	95.0%	66.5	23.0	6	0	0	2	1,345.69
									IHQIEYAMEAVK	95.0%	44.1	22.7	6	4	0	2	1,447.73
									LLCNFMR	95.0%	34.3	22.3	1	0	0	2	953.47
									LVSLIGSK	95.0%	55.4	17.2	3	0	0	2	816.52
									NQYDNDVTVWSPQGR	95.0%	86.6	24.9	12	1	0	2	1,778.81
									NVSIGIVGK	95.0%	46.5	21.7	3	0	0	2	886.54
									THAVLVALK	95.0%	36.8	13.4	1	0	0	2	951.60
									TQIPTQR	95.0%	33.5	22.7	3	0	0	2	843.47
Ras-related protein Rab-14	RAB14_HUMAN	RAB14	23,880	100.00%	9	9	21	54.00%	ADLEAQR	95.0%	63.8	22.0	3	0	0	2	802.41
									GAAGALMVYDITR	95.0%	48.8	22.5	1	0	0	2	1,337.69
									IYQNIQDGSLDLNAAESGVQHKPSAPQGGR	95.0%	41.0	20.6	0	0	1	2	3,150.56
									LQIWDTAGQER	95.0%	61.5	22.2	7	0	0	2	1,316.66
									NLTNPNTVIILIGNK	95.0%	53.4	15.3	3	0	0	2	1,623.94
									STYNHLSSWLTDAR	95.0%	30.4	22.3	0	1	0	2	1,650.79
									TGENVEDAFLEAAK	95.0%	85.2	22.9	2	0	0	2	1,493.71

									TGENVEDAFLEAAKK	95.0%	34.8	22.2	0	2	0	2	1,621.81
									YIIIGDMGVGK	95.0%	36.2	22.3	1	0	0	2	1,181.62
Vacuolar protein sorting-associated protein 26A	VP26A_HUMAN	VPS26A	38,153	100.00%	11	11	26	41.90%	EITGIGPSTTTETETIAK	95.0%	75.6	22.1	5	0	0	2	1,848.94
									ELALPGELTQSR	95.0%	67.2	21.9	5	0	0	2	1,313.71
									EYDLIVHQLATYPDVNNSIK	95.0%	68.4	20.6	1	0	0	2	2,332.18
									FESPESQASAEQPEM	95.0%	49.0	14.6	3	0	0	2	1,682.69
									HYLFYDGESVSGK	95.0%	44.3	22.0	1	0	0	2	1,501.70
									IQHMELQLIK	95.0%	28.0	19.3	0	1	0	2	1,252.71
									LFLAGYDPTPTMR	95.0%	67.8	22.5	4	0	0	2	1,497.74
									QQEIILWR	95.0%	45.7	22.1	2	0	0	2	1,085.61
									SNTHEFVNLVK	95.0%	34.4	22.5	0	1	0	2	1,287.67
									YEIMDGAPVK	95.0%	34.0	22.2	1	0	0	2	1,138.55
									YEIMDGAPVKGESIPIR	95.0%	36.3	22.5	0	2	0	2	1,890.96
GrpE protein homolog 1, mitochondrial	GRPE1_HUMAN	GRPEL1	24,261	100.00%	3	3	6	21.20%	DLLEVADVLEK	95.0%	70.7	22.2	2	0	0	2	1,243.68
									NSGQNLEEDMGQSEQKADPPATEK	95.0%	36.4	18.5	0	2	0	2	2,619.15
									TLRPALVGVVK	95.0%	34.4	7.0	0	2	0	2	1,152.75
3,2-trans-enoyl-CoA isomerase, mitochondrial	D3D2_HUMAN	DCI	32,799	100.00%	5	5	16	16.90%	AVQELWLR	95.0%	49.6	22.3	2	0	0	2	1,014.57
									DADVQNFVSFISK	95.0%	103.0	22.2	4	0	0	2	1,469.73
									ILADNPR	95.0%	38.4	19.4	2	0	0	2	798.45
									SLQMYLER	95.0%	44.7	22.0	6	0	0	2	1,055.52
									VLVEPDAGAGVAVMK	95.0%	76.3	20.8	2	0	0	2	1,455.79
Poliovirus receptor-related protein 1	PVRL1_HUMAN	PVRL1	57,140	100.00%	4	5	71	12.80%	GPINYSLAGTYICEATNPIGTR	95.0%	96.7	21.9	1	0	0	2	2,368.16
									LKGEAEYQEIR	95.0%	61.0	22.8	13	4	0	2	1,335.69
									QNVAIYNPSMGVSVLAPYR	95.0%	125.0	20.7	31	0	0	2	2,079.07
									VEFLRPSFTDGTIR	95.0%	57.0	21.5	0	22	0	2	1,637.87
Proteasome activator complex subunit 3	PSME3_HUMAN	PSME3	29,489	100.00%	3	3	6	16.10%	ITSEAEDLVANFFPK	95.0%	57.0	22.7	3	0	0	2	1,680.85
									SNQQLVDIIEK	95.0%	31.6	22.7	1	0	0	2	1,286.70
									TVESEAASYLDQISR	95.0%	82.1	21.8	2	0	0	2	1,668.81
Importin subunit beta-1	IMB1_HUMAN	KPNB1	97,153	100.00%	16	19	70	25.70%	AAVENLPTFLVELSR	95.0%	90.6	19.7	12	2	0	2	1,658.91
									ANFDKESER	95.0%	56.2	21.3	2	0	0	2	1,095.51
									ESCLEAYTGIVQGLK	95.0%	68.9	22.2	2	0	0	2	1,667.83
									GALQYLVPILTQTLTK	95.0%	51.9	12.0	5	0	0	2	1,759.04
									GDQENVHPDVMLVQPR	95.0%	48.5	22.0	2	2	0	2	1,849.89
									LAATNALLNSLEFTK	95.0%	110.0	20.1	15	0	0	2	1,605.89
									LLETTDRPDGHQNNLR	95.0%	32.9	22.5	1	1	0	2	1,878.94
									LQQVLQMESHIQSTSDR	95.0%	27.4	22.1	0	1	0	2	2,015.98
									SDYDMVDYLNELR	95.0%	77.4	18.6	2	0	0	2	1,632.72
									SNEILTAIIQGMR	95.0%	72.8	22.5	6	0	0	2	1,445.78
									SSAYESLMEIVK	95.0%	51.5	21.4	3	0	0	2	1,372.67
									TVSPDRLELEAAQK	95.0%	38.0	21.9	2	0	0	2	1,556.83
									VAALQNLVK	95.0%	45.7	16.9	3	0	0	2	955.59
									VLANPGNSQVAR	95.0%	79.6	21.5	6	0	0	2	1,225.67
									YLEVVLNTLQQASQAQVDK	95.0%	115.0	20.3	2	0	0	2	2,147.14
									YMEAFKPFLGIGLK	95.0%	28.4	21.3	0	1	0	2	1,629.87
NADP-dependent malic enzyme	MAOX_HUMAN	ME1	64,133	100.00%	5	6	11	16.10%	DMAAFNERPIIFALSNPTSK	95.0%	49.7	21.9	0	2	0	2	2,238.12
									GSEYDDFLDEFMEAVSSK	95.0%	94.9	15.1	2	0	0	2	2,084.86
									QITDNIFLTTAEVIAQQVSDK	95.0%	127.0	19.8	2	2	0	2	2,334.22
									QQLNIHGLLPPSFNSQEIQVLR	95.0%	33.2	17.3	0	1	0	2	2,531.37
									TATVYPEPQNK	95.0%	45.0	23.2	2	0	0	2	1,247.63
WD repeat-containing protein 1	WDR1_HUMAN	WDR1	66,175	100.00%	11	13	47	24.40%	AHDGGIYAISWSPDSTHLLSASGDK	95.0%	49.5	21.6	0	3	2	2	2,585.23
									DYSGQGVVK	95.0%	41.0	21.8	1	0	0	2	952.47

									FATASADGQIYIYDGK	95.0%	80.4	22.5	12	0	0	2	1,719.82
									IIGGDPK	95.0%	31.8	18.8	2	0	0	2	699.40
									KVFASLPQVER	94.9%	26.5	20.4	0	1	0	2	1,273.73
									LATGSDDNCAAFFEGPPFK	95.0%	83.3	19.3	3	0	0	2	2,043.91
									LYSILGTTLKDEGK	95.0%	77.0	19.2	4	3	0	2	1,537.85
									NIDNPALADIYTEHAHQVVVAK	95.0%	35.5	21.1	0	1	0	2	2,418.24
									VFASLPQVER	95.0%	46.9	22.7	4	0	0	2	1,145.63
									YAPSGFYIASGDVSGK	95.0%	80.0	22.1	8	0	0	2	1,618.78
									YEYQPFAGK	95.0%	35.4	21.7	3	0	0	2	1,102.52
Latent-transforming growth factor beta-binding protein 4	LTBP4_HUMAN	LTBP4	173,410	100.00%	19	21	186	14.50%	AEAAAPYTVLAQSAPR	95.0%	122.0	22.8	30	12	0	2	1,615.84
									CSCAPGYR	95.0%	47.7	14.9	2	0	0	2	970.39
									DGGCSLPILR	95.0%	41.3	23.1	18	0	0	2	1,087.56
									DVDECQLFR	95.0%	37.5	20.5	1	0	0	2	1,181.53
									EDGYSDASGFGYCFR	95.0%	85.8	13.0	3	0	0	2	1,730.68
									EICPAGPGYHYSASDLR	95.0%	64.9	20.6	2	0	0	2	1,892.86
									GCQLCPPFGSEGFR	95.0%	58.0	18.9	12	0	0	2	1,611.71
									GGYTCVCPDGFLLDSSR	95.0%	91.9	18.8	5	0	0	2	1,903.83
									GYLAPSGDLSLR	95.0%	33.3	22.9	1	0	0	2	1,248.66
									LSPQGTR	95.0%	32.3	22.6	2	0	0	2	758.42
									NPQVCGPGR	95.0%	49.8	19.9	6	0	0	2	984.47
									QICCCSR	95.0%	34.2	13.0	1	0	0	2	983.39
									RVPPPCAPGR	95.0%	38.6	23.3	0	1	0	2	1,106.59
									SSCISQHVISEAK	95.0%	27.5	21.4	0	1	0	2	1,445.71
									TQEVCCR	95.0%	34.8	16.6	1	0	0	2	952.40
									TSAGTFPGSQPQAPASPVLPARPPPPPLPR	95.0%	63.2	16.9	0	42	0	2	2,988.61
									VPPPCAPGR	95.0%	31.6	22.7	1	0	0	2	950.49
									VSGPWEEADAEAVAR	95.0%	99.1	22.0	29	0	0	2	1,586.75
									VSLSQPR	95.0%	44.1	23.1	12	0	0	2	786.45
									YNTRPLGQEPPR	95.0%	38.4	23.0	1	4	0	2	1,427.74
Alpha-actinin-1	ACTN1_HUMAN	ACTN1	103,043	100.00%	30	39	368	67.60%	AGTQIENIEEDFR	95.0%	71.5	21.9	4	0	0	2	1,521.72
									AGTQIENIEEDFRDGLK	95.0%	79.1	22.1	4	7	0	2	1,934.95
									AIMTYVSSFYHAFSGAQK	95.0%	87.6	22.3	31	13	0	2	2,023.96
									ALDFIASK	95.0%	64.7	21.2	17	0	0	2	864.48
									ASIHEAWTDGK	95.0%	48.0	20.6	20	0	0	2	1,214.58
									ATLPDADKER	95.0%	51.9	21.9	16	4	0	2	1,115.57
									CQLEINFNTLQTK	95.0%	92.7	22.9	4	0	0	2	1,608.81
									DDPLTNLNTAFDVAEK	95.0%	101.0	22.9	10	0	0	2	1,762.85
									DHSGTLGPEEFK	95.0%	30.8	20.4	1	0	0	2	1,316.61
									DLLLDPAWEK	95.0%	50.4	22.0	7	0	0	2	1,199.63
									DQALTEEHAR	95.0%	52.5	22.3	4	0	0	2	1,169.56
									DYETATLSEIK	95.0%	64.5	22.6	3	0	0	2	1,269.62
									EGLLLWCQR	95.0%	48.2	23.4	26	0	0	2	1,174.60
									ELPPDQAEYCIAR	95.0%	73.8	21.1	14	0	0	2	1,561.73
									ETADTDTADQVMASFK	95.0%	121.0	17.8	26	0	0	2	1,745.75
									FAIQDISVEETSAK	95.0%	104.0	22.3	225	0	0	2	1,537.78
									GISQEQMNEFR	95.0%	52.0	19.9	18	0	0	2	1,354.61
									GYEEWLLNEIR	95.0%	101.0	23.5	73	0	0	2	1,421.71
									HEAFESDLAAHQDR	95.0%	51.6	20.1	0	4	0	2	1,625.73
									HRPELIDYGK	95.0%	44.5	21.8	0	29	0	2	1,227.65
									HTNYTMEHIR	95.0%	41.5	20.6	0	15	0	2	1,301.61
									ICDQWDNLGALTQK	95.0%	105.0	21.8	4	0	0	2	1,661.80

									IDQLEGDHQLIQEALIFDNK	95.0%	100.0	21.2	4	16	0	2	2,339.19
									ILAGDKNYITMDELR	95.0%	30.4	21.4	0	2	0	2	1,751.90
									IMSIVDPNR	95.0%	41.5	22.9	8	0	0	2	1,044.55
									ISIEMHGTLEDQLSHLR	95.0%	32.2	21.8	0	2	0	2	1,995.00
									IVQTYHVNMAGTNPYTTITPQEINGK	95.0%	52.3	20.8	0	3	0	2	2,890.44
									KDDPLTNLNTAFDVAEK	95.0%	104.0	22.1	8	7	0	2	1,890.95
									KHEAFESDLAAHQDR	95.0%	48.4	21.5	0	6	74	2	1,753.83
									LAILGIHNEVSK	95.0%	55.3	16.9	6	0	0	2	1,293.75
									LASDLLEWIR	95.0%	101.0	22.0	38	0	0	2	1,215.67
									LDHLAEK	95.0%	45.8	19.3	13	0	0	2	825.45
									LLETIDQLYLEYAK	95.0%	81.6	19.8	10	0	0	2	1,711.92
									LMLLLEVISGER	95.0%	43.2	19.6	1	0	0	2	1,388.78
									LSNRPAFMPSEGR	95.0%	38.1	23.5	1	6	0	2	1,477.72
									LVSIGAEEIVDGNVK	95.0%	123.0	21.4	72	0	0	2	1,542.84
									MEEIGR	95.0%	30.8	23.0	3	0	0	2	734.35
									MLDAEDIVGTARPDEK	95.0%	83.3	22.3	12	10	0	2	1,775.85
									MTLGMIWTIILR	95.0%	97.4	20.2	49	0	0	2	1,479.81
									MVSDINNAWGCLEQVEK	95.0%	114.0	20.4	5	0	0	2	2,008.91
									QFGAQANVIGPWIQTK	95.0%	80.2	20.9	13	0	0	2	1,757.93
									QKDYETATLSEIK	95.0%	70.0	22.2	7	2	0	2	1,525.78
									RDQALTEEHAR	95.0%	58.8	22.2	1	13	0	2	1,325.66
									TFTAWCNSHLR	95.0%	33.2	22.0	1	0	0	2	1,392.65
									TINEVENQILTR	95.0%	86.4	22.5	61	0	0	2	1,429.77
									TIPWLENR	95.0%	36.2	20.3	2	0	0	2	1,028.55
									VEQIAAIAQELNELDYYDSPSVNAR	95.0%	75.6	22.0	1	6	0	2	2,808.37
									VGWEQLLTTIAR	95.0%	92.8	20.5	180	0	0	2	1,386.77
									VLAVNQENEQLMEDYEK	95.0%	136.0	21.1	15	0	0	2	2,067.95
									VPENTMHAMQQK	95.0%	40.7	20.6	6	0	0	2	1,429.66
									YLDIPK	95.0%	35.7	19.0	14	0	0	2	748.42
Filamin-A	FLNA_HUMAN	FLNA	280,711	100.00%	72	88	1231	39.60%	AEGPGLSR	95.0%	47.3	20.5	9	0	0	2	786.41
									AFGPGLQGGSAGSPAR	95.0%	96.4	22.4	46	0	0	2	1,429.72
									AGGPGLER	95.0%	79.5	20.7	30	0	0	2	756.40
									AGNNMLLVGVHGPR	95.0%	59.6	22.7	0	26	0	2	1,450.76
									AGQSAAGAAPGGGVDTR	95.0%	90.0	21.9	30	0	0	2	1,442.70
									AHVVPCFDASK	95.0%	32.3	22.1	1	0	0	2	1,230.59
									ALTQTGGPHVK	95.0%	44.4	21.2	21	0	0	2	1,108.61
									ANLPQSFQVDTSK	95.0%	73.7	23.0	50	0	0	2	1,434.72
									ASGPGLNTTGVPASLPVEFTIDAK	95.0%	90.5	20.0	30	1	0	2	2,342.22
									ATCAPQHGAPGPGPADASK	95.0%	42.9	21.6	0	5	0	2	1,789.83
									AWGPGLEGGVVGK	95.0%	76.9	21.7	32	0	0	2	1,226.65
									AYGPGIEPTGNMVK	95.0%	65.6	22.4	46	0	0	2	1,433.71
									CSGPGLER	95.0%	52.7	20.5	6	0	0	2	875.40
									DAGEGLLAVQITDPEGKPK	95.0%	99.3	21.1	13	8	0	2	1,938.02
									DAPQDFHPDR	95.0%	40.3	20.0	7	3	0	2	1,197.53
									DLAEDAPWK	95.0%	31.6	20.5	1	0	0	2	1,044.50
									DLAEDAPWKK	95.0%	40.9	22.5	2	0	0	2	1,172.60
									DVDIIDHHDNTYTVK	95.0%	48.7	22.5	4	0	0	2	1,784.85
									EAGAGGLAIAVEGPSK	95.0%	90.2	21.6	15	0	0	2	1,426.75
									EATTEFSVDAR	95.0%	85.5	20.8	25	0	0	2	1,225.57
									EGPYSISVLYGDEEVPR	95.0%	100.0	22.3	39	0	0	2	1,909.92
									ENGVYLIDVK	95.0%	36.9	22.8	4	0	0	2	1,149.62

FGGEHVPNSPFQVTALAGDQPSVQPPLR	95.0%	63.0	20.3	0	22	0	2	2,945.49
FNEEHIPDSPFVVPVASPSGDAR	95.0%	53.4	22.1	0	17	0	2	2,467.19
FTVETR	95.0%	33.4	20.3	2	0	0	1	752.39
FVPAEMGTHTVSVK	95.0%	37.9	22.8	2	0	0	2	1,518.76
GAGTGGLGLAVEGPSEAK	95.0%	98.9	22.4	37	0	0	2	1,570.81
GKLDVQFSGLTK	95.0%	38.5	20.4	1	0	0	2	1,292.72
GLVEPVDVVDNADGTQTVNYVPSR	95.0%	117.0	21.5	15	5	0	2	2,544.26
GQHVPGSPFQFTVGPLGEGGAHK	95.0%	20.8	21.9	0	0	1	2	2,304.15
GTVEPQLEAR	95.0%	58.9	22.7	25	0	0	2	1,099.57
IANLQTDLSDGLR	95.0%	116.0	21.5	60	0	0	2	1,415.75
IPEISIQDMTAQVTSPSGK	95.0%	107.0	21.8	11	0	0	2	2,002.02
IQQNTFTR	95.0%	42.6	22.3	12	0	0	2	1,007.53
KGEITGEVR	95.0%	40.9	23.4	2	0	0	2	988.54
LDVQFSGLTK	95.0%	57.7	22.2	4	0	0	2	1,107.61
LIALLEVLSQK	95.0%	65.7	13.4	17	0	0	2	1,226.77
LLGWIQNK	95.0%	65.3	22.2	37	0	0	2	971.57
LPQLPITNFSR	95.0%	53.8	20.0	22	0	0	2	1,285.73
LSPFMADIR	95.0%	46.8	23.2	15	0	0	2	1,049.55
LTVSSLQESGLK	95.0%	76.2	21.5	10	0	0	2	1,261.70
LVSNHSLHETSSVFVDSLTK	95.0%	52.3	21.2	0	2	0	2	2,200.13
LYSVSYLLK	95.0%	37.0	21.5	4	0	0	2	1,085.62
NDNDTFTVK	95.0%	34.9	20.3	2	0	0	2	1,053.49
NGHVGISFVPK	95.0%	29.6	20.8	0	1	0	2	1,154.63
SAGQGEVLVYVEDPAGHQEEAK	95.0%	65.6	21.5	0	18	0	2	2,313.10
SPFEVYVDK	95.0%	64.4	21.8	29	0	0	2	1,083.54
SPFEVYVDKSQGDASK	95.0%	68.7	21.7	1	5	0	2	1,756.84
SPFSVAVSPSLDLSK	95.0%	75.4	21.7	37	0	0	2	1,533.82
SPYTVTVGQACNPSACR	95.0%	67.2	20.3	1	0	0	2	1,867.84
TFSVWYVPEVTGTHK	95.0%	42.2	22.5	2	11	0	2	1,750.88
TGVAVNKPAEFTVDAK	95.0%	84.4	21.7	17	16	0	2	1,646.88
TGVELGKPTHFTVNAK	95.0%	26.9	20.8	0	1	0	2	1,698.92
TPCEEILVK	95.0%	41.3	23.2	2	0	0	2	1,088.57
VANPSGNLTETYVQDR	95.0%	120.0	22.7	36	0	0	2	1,763.86
VAQPTITDNKDGTVTVR	95.0%	72.0	21.5	15	9	0	2	1,814.96
VDVGKDQEFTVK	95.0%	64.8	22.9	20	5	0	2	1,364.71
VEPGLGADNSVVR	95.0%	58.5	22.5	11	0	0	2	1,312.69
VGSAADIPINISETDLSLLTATVVPPSGR	95.0%	31.6	15.8	0	2	0	2	2,893.55
VHGPGIQSGTTNKPNK	95.0%	53.9	22.1	4	3	0	2	1,634.86
VKAEGPGLSR	95.0%	47.0	18.9	17	0	0	2	1,013.57
VKETADFK	95.0%	32.1	20.9	1	0	0	2	937.50
VKVEPSHDASK	95.0%	43.2	21.7	0	2	0	2	1,196.63
VLPTHDASK	95.0%	44.2	19.8	2	0	0	2	967.52
VNQPASFAVSLNGAK	95.0%	67.2	22.8	4	0	0	2	1,502.80
VNVGAGSHPNK	95.0%	57.0	22.6	5	1	0	2	1,079.56
VPVHDVTDASK	95.0%	58.0	22.6	27	13	0	2	1,167.60
VQVQDNEGCPVEALVK	95.0%	101.0	22.5	12	0	0	2	1,784.89
VTAQGPGLEPSGNIANK	95.0%	102.0	21.9	60	0	0	2	1,652.86
VTVLFAGQHIAK	95.0%	69.2	17.9	11	1	0	2	1,283.75
VTYTPMAPGSYLISIK	95.0%	67.7	21.6	41	0	0	2	1,756.92
WGDEHIPGSPYR	95.0%	42.2	21.2	1	2	0	2	1,413.66
YAPSEAGLHEMDIR	95.0%	52.8	21.3	6	0	0	2	1,604.74

									YGGDEIPFSPYR	95.0%	71.6	20.3	19	0	0	2	1,400.65
									YGGPYHIGGSPFK	95.0%	27.3	22.8	0	2	0	2	1,379.68
									YGGQPVPNFPSK	95.0%	51.9	22.1	27	0	0	2	1,290.65
									YNEQHVPGSPFTAR	95.0%	48.2	22.1	5	12	0	2	1,602.77
									YTPVQQGPVGVNVTYGGDPIPK	95.0%	101.0	20.5	24	10	0	2	2,286.18
Receptor-type tyrosine-protein phosphatase F	PTPRF_HUMAN	PTPRF	212,860	100.00%	20	21	103	12.90%	AAGTEGPFQEVDGVATTR	95.0%	116.0	22.8	11	0	0	2	1,805.87
									AGLGEEFEK	95.0%	52.7	22.0	2	0	0	2	979.47
									AHTDVGPGPESSPVLVR	95.0%	68.7	22.1	2	3	0	2	1,717.89
									EHSSWDLVGLEK	95.0%	33.0	21.9	0	1	0	2	1,399.69
									FEVIEFDDGAGSVLR	95.0%	106.0	22.4	5	0	0	2	1,653.81
									GPPSEAVR	95.0%	43.1	20.2	2	0	0	2	812.43
									GSGPLSPSIQSR	95.0%	38.6	22.2	3	0	0	2	1,185.62
									GSSAGGLQHLVSIR	95.0%	65.2	21.1	2	0	0	2	1,381.76
									LENGEPR	95.0%	30.5	20.5	1	0	0	2	814.41
									NVLELSNVVR	95.0%	58.2	22.0	8	0	0	2	1,142.65
									SDMGVGVFTPTIEAR	95.0%	71.2	22.6	6	0	0	2	1,595.77
									TAQSTPSAPPQK	95.0%	43.4	21.8	8	0	0	2	1,212.62
									TDEDVPSGPPR	95.0%	39.8	21.4	3	0	0	2	1,169.54
									TGEQAPSSPPR	95.0%	71.1	22.2	20	0	0	2	1,126.55
									TGEQAPSSPPRR	95.0%	45.9	22.0	0	2	0	2	1,282.65
									TQQGVPAQPADFQAEVESDTR	95.0%	93.1	20.8	2	0	0	2	2,274.06
									VGGSMLTPR	95.0%	48.4	24.0	4	0	0	2	933.48
									VLAFTAVGDGPPSPTIQVK	95.0%	82.1	18.0	11	0	0	2	1,897.04
									YSAPANLYVR	95.0%	52.5	23.1	5	0	0	2	1,153.60
									YSIGGLSPFSEYAFR	95.0%	55.8	22.6	2	0	0	2	1,693.82
Glutathione S-transferase omega-1	GSTO1_HUMAN	GSTO1	27,549	100.00%	8	8	58	34.90%	EDPTVSALLTSEK	95.0%	76.8	23.7	4	0	0	2	1,389.71
									EFTKLEEVLTNK	95.0%	81.8	21.6	2	0	0	2	1,450.78
									GSAPPGPVPEGSIR	95.0%	72.9	23.0	7	0	0	2	1,320.69
									HEVININLK	95.0%	49.5	18.8	21	0	0	2	1,079.62
									LNECVDHTPK	95.0%	53.2	21.0	4	0	0	2	1,212.57
									MILELFSK	95.0%	40.4	19.2	8	0	0	2	980.55
									NKPEWFFK	95.0%	30.5	21.8	0	2	0	2	1,095.56
									VPSLVGSFIR	95.0%	54.3	18.8	10	0	0	2	1,074.63
Structural maintenance of chromosomes protein 3	SMC3_HUMAN	SMC3	141,529	100.00%	8	8	14	8.87%	ALDQFVNFSEQK	95.0%	76.7	23.2	1	0	0	2	1,425.70
									EENAEQQALAAK	95.0%	59.8	21.6	2	0	0	2	1,301.63
									ELGSLPQEAFEK	95.0%	42.4	22.4	1	0	0	2	1,347.68
									GALTGGYYDTR	95.0%	50.4	21.0	1	0	0	2	1,173.55
									GSGSQSSVPSVDQFTGVGIR	95.0%	82.5	21.7	2	0	0	2	1,964.97
									INQMATAPDSQR	95.0%	57.7	22.0	4	0	0	2	1,347.63
									KGDVEGSQSQDEGEGSGESER	95.0%	56.2	15.3	0	2	0	2	2,166.90
									LAQATQER	95.0%	34.4	22.9	1	0	0	2	916.49
Nuclear ubiquitous casein and cyclin-dependent kinases substrate	NUCKS_HUMAN	NUCKS1	27,279	100.00%	5	5	8	21.80%	ATVTPSPVK	95.0%	38.1	19.2	2	0	0	2	899.52
									LKATVTPSPVK	95.0%	41.0	17.1	1	0	0	2	1,140.70
									NSQEDSEDSEDKDVK	95.0%	67.6	16.5	1	0	0	2	1,724.71
									VGRPTASK	95.0%	33.4	24.0	2	0	0	2	815.47
									VVDYSQFQESDDADEDYGR	95.0%	125.0	12.8	2	0	0	2	2,237.91
Glucosamine 6-phosphate N-acetyltransferase	GNA1_HUMAN	GNPNAT1	20,731	100.00%	4	4	14	31.50%	ITLECLPQNVGFYK	95.0%	49.5	21.6	2	0	0	2	1,681.86
									LLLSTLTLLSK	95.0%	65.6	6.0	5	0	0	2	1,201.78
									MKPDETPMFDPSLLK	95.0%	31.1	22.1	1	0	0	2	1,780.85
									VLGQLTETGVVSPEQFMK	95.0%	102.0	21.9	6	0	0	2	1,979.02
14-3-3 protein sigma	1433S_HUMAN	SFN	27,757	100.00%	14	18	282	63.70%	DSTLIMQLLR	95.0%	79.3	21.3	113	0	0	2	1,189.66

									EMPPTNPIR	95.0%	50.8	21.8	13	0	0	2	1,054.54
									GAVEKGEELSCEER	95.0%	66.0	20.0	2	0	0	2	1,592.72
									GEELSCEER	95.0%	40.6	15.2	2	0	0	2	1,108.46
									KEMPPTNPIR	95.0%	34.0	21.7	3	2	0	2	1,198.63
									LAEQAER	95.0%	59.4	21.6	36	0	0	2	816.42
									LAEQAERYEDMAAFMK	95.0%	67.8	19.9	5	6	0	2	1,934.86
									MKGDYYR	95.0%	42.7	19.2	6	0	0	2	932.43
									NLLSVAYK	95.0%	51.4	19.1	49	0	0	2	907.53
									NLLSVAYKNVVGGQR	95.0%	56.6	18.5	1	0	0	2	1,617.91
									SAYQEAMDISK	95.0%	75.3	20.7	24	0	0	2	1,242.57
									SAYQEAMDISKK	95.0%	78.9	21.7	28	0	0	2	1,370.66
									SNEEGSEEKGPEVR	95.0%	105.0	19.0	39	50	0	2	1,546.70
									TTFDEAMADLHTLSEDSYK	95.0%	92.6	18.7	31	20	0	2	2,173.96
									VETELQGVCDTVLGLLDSHLIK	95.0%	32.9	19.3	0	1	0	2	2,439.28
									VLSSIEQK	95.0%	65.9	22.9	27	0	0	2	903.52
									YEDMAAFMK	95.0%	57.4	15.9	36	0	0	2	1,105.47
									YLAEVATGDDK	95.0%	95.4	21.9	13	0	0	2	1,181.57
									YLAEVATGDDKK	95.0%	51.0	22.4	6	0	0	2	1,309.66
Integrin beta-1	ITB1_HUMAN	ITGB1	88,397	100.00%	7	8	33	9.90%	IGFGSFVEK	95.0%	53.2	21.5	13	0	0	2	983.52
									LKPEDITQIQPQQLVLR	95.0%	60.0	12.3	2	5	0	2	2,019.16
									SAVTTVVNPK	95.0%	55.8	23.8	4	0	0	2	1,015.58
									SGEPQTFTLK	95.0%	34.7	24.0	3	0	0	2	1,107.57
									SLGTDLMNEMR	95.0%	42.8	22.1	3	0	0	2	1,266.58
									TVMPYISTTPAK	95.0%	32.1	22.2	2	0	0	2	1,308.69
									WDTGENPIYK	95.0%	34.3	21.7	1	0	0	2	1,222.57
Adenylate kinase 2, mitochondrial	KAD2_HUMAN	AK2	26,461	100.00%	6	6	18	31.00%	APSVPAAEPEYPK	95.0%	62.1	22.3	2	0	0	1	1,355.68
									AVLLGPPGAGK	95.0%	39.9	11.8	1	0	0	2	979.59
									LQAYHTQTTPLIEYYR	95.0%	34.0	21.5	0	3	0	2	1,997.01
									LVSDEMVVELIEK	95.0%	93.3	22.3	6	0	0	2	1,519.79
									NGFLLDGFPR	95.0%	68.0	23.1	4	0	0	2	1,135.59
									QAEMLDDLMEK	95.0%	47.0	20.2	2	0	0	2	1,322.60
Programmed cell death 6-interacting protein	PDC6I_HUMAN	PDCD6IP	96,007	100.00%	12	12	36	18.40%	ELPELLQR	95.0%	64.8	17.8	4	0	0	2	997.57
									EPSAPSIPTPAYQSSPAGGHAPTPPTPAPR	95.0%	57.3	20.2	0	3	0	2	2,936.45
									FYNELTEILVR	95.0%	66.8	21.1	2	0	0	2	1,396.75
									KADLVNR	95.0%	34.5	24.0	1	0	0	2	815.47
									LALASLGYEK	95.0%	47.8	21.2	2	0	0	2	1,064.60
									LANQAADYFGDAFK	95.0%	74.3	21.4	4	0	0	2	1,530.72
									LLDEEEATDNDLR	95.0%	110.0	20.9	5	0	0	2	1,532.71
									NLATAYDNFVELVANLK	95.0%	72.6	20.8	2	0	0	2	1,894.99
									SLLSNLDEVKK	95.0%	43.8	20.3	2	0	0	2	1,245.71
									SVIEQGGIQTVDQLIK	95.0%	115.0	18.5	6	0	0	2	1,727.95
									TMQGSEVVNVLK	95.0%	68.6	23.4	3	0	0	2	1,320.68
									YYDQICSIEPK	95.0%	48.0	21.6	2	0	0	2	1,415.65
Arylsulfatase A	ARSA_HUMAN	ARSA	53,571	99.50%	2	2	3	8.09%	DPGENYNLLGGVAGATPEVLQALK	95.0%	70.5	20.1	1	0	0	2	2,426.26
									GGLPLEEVTVAEVLAAR	95.0%	70.8	18.1	2	0	0	2	1,723.96
Activated RNA polymerase II transcriptional coactivator p15	TCP4_HUMAN	SUB1	14,378	100.00%	6	6	11	35.40%	EQISDIDDAVR	95.0%	62.8	22.3	2	0	0	2	1,260.61
									EQISDIDDAVRK	95.0%	34.8	22.6	1	0	0	2	1,388.70
									EYWMDPEGEMKPGR	95.0%	32.7	16.5	0	2	0	2	1,756.73
									GISLNPEQWSQLK	95.0%	69.9	22.1	3	0	0	2	1,499.79
									GISLNPEQWSQLKEQISDIDDAVR	95.0%	56.6	21.7	0	2	0	2	2,741.38
									VLIDIR	95.0%	38.7	19.0	1	0	0	2	728.47

Peptidyl-prolyl cis-trans isomerase FKBP3	FKBP3_HUMAN	FKBP3	25,159	100.00%	6	6	18	30.80%	AWTVEQLR	95.0%	45.8	23.3	2	0	0	2	1,002.54
									FLQEHGSDSFLAEHK	95.0%	31.8	21.7	0	4	0	2	1,744.83
									GWDEALLTMSK	95.0%	74.3	22.4	5	0	0	2	1,250.61
									SEETLDEGPPK	95.0%	50.0	20.3	2	0	0	2	1,201.56
									SEQLPK	95.0%	32.8	21.1	1	0	0	2	701.38
									TANKDHLVTAYNHLFETK	95.0%	35.7	22.0	0	0	4	2	2,102.07
Eukaryotic translation initiation factor 3 subunit F	EIF3F_HUMAN	EIF3F	37,546	100.00%	3	3	7	10.90%	FLMSLVNQVPK	95.0%	81.3	20.9	3	0	0	2	1,291.71
									VIGLSSDLQQVGGASAR	95.0%	100.0	21.3	2	0	0	2	1,657.89
									VIGTLLGTVDK	95.0%	74.5	19.2	2	0	0	2	1,115.67
Protein disulfide-isomerase	PDIA1_HUMAN	P4HB	57,100	100.00%	31	40	314	60.40%	AEGSEIR	95.0%	49.2	23.9	2	0	0	2	761.38
									EADDIVNWLK	95.0%	62.6	22.6	21	0	0	2	1,202.61
									EADDIVNWLKK	95.0%	46.8	22.5	2	0	0	2	1,330.70
									ENLLDFIK	95.0%	54.5	20.7	20	0	0	2	991.55
									FFPASADR	95.0%	40.6	21.8	1	0	0	2	910.44
									HNQLPLVIEFTEQTAPK	95.0%	62.5	19.9	11	41	0	2	1,965.04
									IKPHLMSQELPEDWDKQPVK	95.0%	36.0	20.3	0	0	16	2	2,418.25
									ILEFFGLK	95.0%	53.7	18.3	14	0	0	2	966.57
									ILEFFGLKK	95.0%	39.9	16.3	2	0	0	2	1,094.66
									ILFIFIDSDHTDNQR	95.0%	70.7	21.8	2	2	0	2	1,833.91
									ITEFCHR	95.0%	52.9	21.8	5	0	0	2	962.45
									KEECPAVR	95.0%	51.2	21.9	2	0	0	2	988.49
									KFDEGR	95.0%	43.0	23.7	1	0	0	2	751.37
									KSNFAEALAAHK	95.0%	37.7	23.2	0	2	0	2	1,286.69
									LGETYKDHENIVIAK	95.0%	36.4	21.8	2	11	0	2	1,729.91
									LITLEEEMTK	95.0%	71.1	23.0	14	0	0	2	1,222.62
									LKAEGSEIR	95.0%	38.5	23.7	2	0	0	2	1,002.56
									MDSTANEVEAVK	95.0%	84.2	20.8	26	0	0	2	1,309.59
									NFEDVAFDEK	95.0%	55.4	19.3	4	0	0	2	1,213.54
									NFEDVAFDEKK	95.0%	48.7	21.4	9	0	0	2	1,341.63
									NNFEGEVTK	95.0%	30.9	22.4	1	0	0	2	1,037.49
									NNFEGEVTKENLLDFIK	95.0%	80.7	21.4	3	6	0	2	2,010.02
									QFLQAAEAIDDIPFGITSNSDVFSK	95.0%	44.3	21.6	3	1	0	2	2,713.34
									QLAPIWDK	95.0%	45.8	19.7	2	0	0	2	970.54
									SNFAEALAAHK	95.0%	54.1	22.6	4	0	0	2	1,158.59
									TGPAATTLPDGAAAESLVESSEVAVIGFFK	95.0%	64.6	20.0	0	9	0	2	2,935.49
									THILLFLPK	95.0%	38.1	12.0	6	5	0	2	1,081.68
									TVIDYNGER	95.0%	32.4	22.7	2	0	0	2	1,066.52
									VDATEESDLAQQYGVR	95.0%	115.0	22.0	26	2	0	2	1,780.84
									YKPESEELTAER	95.0%	61.6	22.5	11	4	0	2	1,451.70
									YQLDKDGVVLFK	95.0%	65.2	20.7	7	10	0	2	1,424.78
Protein DJ-1	PARK7_HUMAN	PARK7	19,873	100.00%	11	13	65	49.70%	ALVILAK	95.0%	57.7	11.8	15	0	0	2	727.51
									DGLILTSR	95.0%	42.4	24.1	7	0	0	2	874.50
									EGPYDVVVLPGGNLGAQNLSESAAVK	95.0%	64.7	20.8	6	3	0	2	2,584.33
									EILKEQENR	95.0%	45.3	24.0	4	1	0	2	1,158.61
									GAEEMETVIPVDVMR	95.0%	68.7	23.2	18	0	0	2	1,707.79
									GAEEMETVIPVDVMRR	95.0%	44.0	22.0	0	5	0	2	1,863.89
									KEGPYDVVVLPGGNLGAQNLSESAAVK	95.0%	34.1	18.2	0	1	0	2	2,712.42
									VEKDGLILTSR	95.0%	33.5	20.4	1	0	0	2	1,230.71
									VTTHPLAK	95.0%	32.5	16.0	1	0	0	2	866.51
									VTVAGLAGK	94.7%	30.1	21.0	1	0	0	2	815.50
									VTVAGLAGKDPVQCSR	95.0%	40.3	21.4	0	2	0	2	1,657.87

Putative hydroxypyruvate isomerase	HYI_HUMAN	HYI	30,388	100.00%	4	4	7	21.70%	AEMEAVFLENLR	95.0%	32.3	21.9	1	0	0	2	1,437.70
									EFLPIVGHVQVAQVPGR	95.0%	35.3	16.4	0	2	0	2	1,846.03
									GDTVEGLSWLR	95.0%	56.8	23.3	2	0	0	2	1,232.63
									ITDPQYFLDTPQQAAAILQK	95.0%	88.9	19.9	2	0	0	2	2,261.18
Selenium-binding protein 1	SBP1_HUMAN	SELENBP1	52,374	99.90%	2	2	3	6.57%	GGPVQVLEDEELK	95.0%	64.2	22.3	1	0	0	2	1,412.73
									NTGTEAPDYLATVDVDPK	95.0%	64.1	22.2	2	0	0	2	1,905.91
Heterogeneous nuclear ribonucleoprotein A3	ROA3_HUMAN	HNRNPA3	39,577	100.00%	3	3	17	10.30%	EDSVKPGAHLTVK	95.0%	30.6	21.4	1	0	0	2	1,380.75
									IETIEVMEDR	95.0%	66.0	23.0	14	0	0	2	1,234.60
									IFVGGIK	95.0%	34.6	16.5	1	0	0	2	733.46
									IFVGGIKEDTEEYNLR	95.0%	46.2	21.4	2	0	0	2	1,882.96
Osteoclast-stimulating factor 1	OSTF1_HUMAN	OSTF1	23,770	100.00%	3	4	7	20.10%	DIVEMLFTQPNIELNQQNK	95.0%	78.9	22.3	2	0	0	2	2,274.14
									GYADIVQLLLAK	95.0%	75.4	16.7	2	0	0	2	1,303.76
									LGDTALHAAAWK	95.0%	46.0	21.6	2	1	0	2	1,253.66
Glycogen phosphorylase, brain form	PYGB_HUMAN	PYGB	96,680	100.00%	10	10	28	14.90%	GLAGLGDVAEVR	95.0%	74.4	21.4	2	0	0	2	1,156.63
									IGEEFLTDLSQLK	95.0%	70.1	22.6	3	0	0	2	1,492.79
									IGEEFLTDLSQLKK	95.0%	43.6	20.4	0	2	0	2	1,620.89
									IVNGWQVEEADDWLR	95.0%	98.5	22.0	2	0	0	2	1,829.88
									LLPLVSDEVFIR	95.0%	64.5	18.3	3	0	0	2	1,400.82
									LQDFNVGDYIEAVLDR	95.0%	119.0	22.1	3	0	0	2	1,866.92
									LVTSIGDVVNHDPVVGDR	95.0%	38.7	21.8	0	4	0	2	1,891.99
									VAIQLNDTHPALSIPELMR	95.0%	31.6	19.3	0	1	0	2	2,134.13
									VIFLENYR	95.0%	50.2	21.6	5	0	0	2	1,053.57
									VLYPNDNFFEGK	95.0%	55.6	21.8	3	0	0	2	1,442.70
Transforming growth factor-beta-induced protein ig-h3	BGH3_HUMAN	TGFBI	74,665	100.00%	21	25	175	33.70%	DGTPPIDAHTR	95.0%	36.2	23.3	3	0	0	2	1,179.58
									DLLNNHILK	95.0%	35.4	18.0	1	0	0	2	1,079.62
									EGVYTVFAPTNEAFR	95.0%	66.1	21.8	20	0	0	2	1,700.83
									ELANILK	95.0%	37.3	20.0	4	0	0	2	800.49
									FSMLVAAIQSAGLTETLNR	95.0%	107.0	21.1	14	4	0	2	2,038.06
									GDELADSALEIFK	95.0%	90.6	22.4	30	0	0	2	1,407.70
									ILGDPEALR	95.0%	60.2	18.4	7	0	0	2	983.55
									ILGDPEALRDLLNNHILK	95.0%	27.1	14.1	0	0	2	2	2,044.16
									IPSETLNR	95.0%	56.0	21.6	7	0	0	2	929.51
									LTLLAPLNSVFK	95.0%	60.9	12.0	13	0	0	2	1,315.80
									NHIIKDQLASK	95.0%	42.1	18.9	2	0	0	2	1,266.72
									NNVVSVNK	95.0%	33.5	24.0	1	0	0	2	873.48
									NNVVSVNKEPVAEPDIMATNGVVHVITNVLQPPANRPQER	95.0%	39.0	15.8	0	0	4	2	4,362.26
									QAGLGNHLSGSER	95.0%	56.8	22.4	5	4	0	2	1,325.66
									QASAFSR	95.0%	41.4	21.1	4	0	0	2	766.38
									SPYQLVLQHSR	95.0%	71.6	21.4	7	8	0	2	1,327.71
									VLTDELK	95.0%	33.1	23.0	2	0	0	2	817.47
									VLTPPMGTVMDVLK	95.0%	47.0	22.7	10	0	0	2	1,532.81
									YGTLFTMDR	95.0%	63.2	21.3	10	0	0	2	1,103.52
									YLYHGQTLETLGGK	95.0%	66.3	22.6	5	6	0	2	1,579.81
									YLYHGQTLETLGGKK	95.0%	52.3	20.3	0	2	0	2	1,707.91
Serine/threonine-protein phosphatase 2A 55 kDa regulatory subunit B alpha isoform	2ABA_HUMAN	PPP2R2A	51,675	100.00%	3	3	6	8.05%	LFEEPEDPSNR	95.0%	60.9	20.4	2	0	0	2	1,332.61
									SFFSEIISSISDVK	95.0%	65.2	23.5	2	0	0	2	1,558.80
									VVIFQQEQENK	95.0%	66.3	23.8	2	0	0	2	1,361.71
Endoplasmic reticulum aminopeptidase 1	ERAP1_HUMAN	ERAP1	107,220	100.00%	18	18	46	21.50%	ASLINNAFQLVSIGK	95.0%	101.0	17.8	3	0	0	2	1,574.89
									DMNEVETQFK	95.0%	34.8	19.0	2	0	0	2	1,256.55
									EMFDDVSYDK	95.0%	30.8	15.7	1	0	0	2	1,248.51
									ESALLFDAEK	95.0%	45.3	23.4	2	0	0	2	1,122.57

									FELGSSSIAHMVMGTTNQFSTR	95.0%	45.9	20.0	0	1	0	2	2,433.12
									GFPLITITVR	95.0%	39.9	15.6	4	0	0	2	1,116.68
									GTHTAVSSNDR	95.0%	31.7	20.3	2	0	0	2	1,144.53
									HLAISNMPLVK	95.0%	40.1	19.5	2	0	0	2	1,238.69
									ILASTQFEPTAAR	95.0%	77.1	22.3	8	0	0	2	1,404.75
									NPVGYPLAWQFLR	95.0%	36.0	21.9	2	0	0	2	1,560.83
									QTWTDEGSVSER	95.0%	48.9	19.9	1	0	0	2	1,394.62
									SDGTPFPWNK	95.0%	34.0	21.0	1	0	0	2	1,148.54
									SGIVQYLQK	95.0%	34.6	18.7	2	0	0	2	1,035.58
									TDVLILPEEVEWIK	95.0%	47.1	20.3	3	0	0	2	1,683.92
									TKTDVLILPEEVEWIK	95.0%	26.9	17.7	0	1	0	2	1,913.06
									TQEFPQILTLIGR	95.0%	67.6	19.0	8	0	0	2	1,515.85
									VGDYFFGK	95.0%	33.7	22.0	1	0	0	2	932.45
									VSVYAVPDK	95.0%	40.5	21.6	2	0	0	2	977.53
Histone H2A type 1-D	H2A1D_HUMAN	HIST1H2AD	14,090	100.00%	2	3	21	21.50%	AGLQFPVGR	95.0%	65.0	22.4	5	0	0	2	944.53
									VTIAQGGVLPNIQAVLLPK	95.0%	62.7	11.1	14	2	0	2	1,931.17
Laminin subunit beta-3	LAMB3_HUMAN	LAMB3	129,553	100.00%	31	39	345	34.70%	AAEESASQIQSSAQR	95.0%	107.0	22.4	36	2	0	2	1,562.74
									AGGAFLMAGQVAEQLR	95.0%	96.2	22.3	18	0	0	2	1,634.83
									AHAVEGQVEDVVGNLR	95.0%	95.2	21.4	5	18	0	2	1,692.87
									AVLSSPAVTEQEVAQVASAILSLR	95.0%	107.0	16.6	4	5	0	2	2,439.35
									CDCNILGSR	95.0%	34.8	17.4	1	0	0	2	1,094.47
									EGFGGLMCSAAAIR	95.0%	85.4	21.3	2	0	0	2	1,455.67
									GFNAQLQR	95.0%	54.6	23.9	5	0	0	2	933.49
									GYHPPSAYYAVSQLR	95.0%	46.4	22.7	0	3	0	2	1,708.84
									IQEVGEITNLR	95.0%	71.4	22.5	17	0	0	2	1,271.70
									ISSADPSGAFR	95.0%	62.7	23.5	4	0	0	2	1,107.54
									LEMSSLPDLTPTFNK	95.0%	79.7	21.6	11	0	0	2	1,692.85
									LETQVSASR	95.0%	82.1	23.5	18	0	0	2	990.52
									LGQSSMLGEQGAR	95.0%	85.5	22.9	21	0	0	2	1,333.65
									LLIQQVR	95.0%	46.4	13.8	2	0	0	2	869.56
									LPNVDLVLSQTK	95.0%	80.8	19.5	28	0	0	2	1,326.76
									LQAEAEEAR	95.0%	39.6	21.0	2	0	0	2	1,016.50
									MLSTAYEQSAQAAQQVSDSSR	95.0%	139.0	20.2	6	4	0	2	2,274.03
									MNEIQAIAAR	95.0%	91.6	23.2	15	0	0	2	1,116.58
									QGTVALQEAQDTMQGTSR	95.0%	102.0	21.5	6	6	0	2	1,936.90
									QLGDFWTR	95.0%	45.7	22.9	4	0	0	2	1,022.51
									QQGAEAVQAQQLAEGASEQALSAQEGFER	95.0%	87.6	20.3	0	5	0	2	3,031.44
									RLQAEAEEAR	95.0%	48.5	22.7	3	0	0	2	1,172.60
									RVEQIR	95.0%	33.5	22.6	1	0	0	2	800.47
									SADLTGLEK	95.0%	40.0	24.0	4	0	0	2	933.49
									SFNGLLTMYQR	95.0%	48.3	23.0	4	0	0	2	1,329.66
									TEAEELFGETMEMMDR	95.0%	91.0	11.1	9	0	0	2	1,950.78
									TLQGLQLDLPLEEETLSLPR	95.0%	85.7	16.5	12	1	0	2	2,265.23
									TYGDVATGCR	95.0%	47.0	18.1	1	0	0	2	1,099.48
									VAEVQQVLRPAEK	95.0%	60.8	18.2	5	1	0	2	1,466.83
									VENVASSSGPMR	95.0%	65.9	22.2	38	0	0	2	1,249.58
									VQLNLMDLVSGIPATQSQK	95.0%	123.0	19.7	15	3	0	2	2,058.09
Malate dehydrogenase, cytoplasmic	MDHC_HUMAN	MDH1	36,409	100.00%	12	12	102	43.10%	AICDHVR	95.0%	33.1	20.8	1	0	0	2	870.43
									DVIATDKEDVAFK	95.0%	93.9	23.0	11	0	0	2	1,450.74
									ELTEEKESAFEFLSSA	95.0%	75.1	21.6	28	0	0	2	1,816.85
									ENFSCLTR	95.0%	47.2	20.0	2	0	0	2	1,026.47

									EVGVYEALKDDSWLK	95.0%	74.4	21.1	8	0	0	2	1,751.89
									FVEGLPINDFSR	95.0%	63.8	22.9	12	0	0	2	1,393.71
									GEFVTTVQQR	95.0%	76.5	23.3	12	0	0	2	1,164.60
									LGVTANDVK	95.0%	61.6	23.0	15	0	0	2	916.51
									LSSAMSAAK	95.0%	39.1	21.6	1	0	0	2	881.44
									NVIIWGNHSSTQYPDVNHAK	95.0%	50.0	22.0	0	7	0	2	2,280.12
									SQGAALDK	95.0%	34.8	24.9	1	0	0	2	789.41
									VIVVGNPANTNCLTASK	95.0%	133.0	21.5	4	0	0	2	1,757.92
Annexin A5	ANXA5_HUMAN	ANXA5	35,921	100.00%	25	34	482	75.90%	ADAETLR	95.0%	42.8	24.0	2	0	0	2	775.40
									ADAETLRK	95.0%	48.4	23.2	6	0	0	2	903.49
									DLLDDLKSELTGK	95.0%	86.8	22.9	24	1	0	2	1,446.77
									DPDAGIDEAQVEQDAQALFQAGELK	95.0%	89.2	20.5	2	4	0	2	2,658.25
									ETSGNLEQLLLAVVK	95.0%	110.0	19.2	29	1	0	2	1,613.91
									FITIFGTR	95.0%	54.0	19.7	10	0	0	2	954.54
									GAGTDDHTLIR	95.0%	84.3	22.0	20	1	0	2	1,155.58
									GLGTDEESILTLLTSR	95.0%	126.0	22.1	30	5	0	2	1,704.90
									GTVTDFPGFDER	95.0%	64.6	21.2	44	0	0	2	1,340.61
									GTVTDFPGFDERADAETLR	95.0%	45.4	21.9	0	3	0	2	2,096.99
									KNFATSLYSMIK	95.0%	56.8	22.7	5	1	0	2	1,402.74
									LIVALMKPSR	95.0%	65.3	15.6	5	13	0	2	1,127.70
									LYDAYELK	95.0%	53.3	21.1	13	0	0	2	1,014.51
									MLVVLLQANR	95.0%	38.8	20.1	2	0	0	2	1,172.68
									NFATSLYSMIK	95.0%	69.9	22.0	62	0	0	2	1,290.64
									QEISAAFK	95.0%	47.2	22.0	13	0	0	2	893.47
									QVYEEEYGSSLEDDVVGDTSGYYQR	95.0%	36.5	17.2	1	0	0	2	2,888.24
									SEIDLFNIR	95.0%	68.2	23.7	64	0	0	2	1,106.58
									SEIDLFNIRK	95.0%	38.8	21.3	3	0	0	2	1,234.68
									SIPAYLAETLYYAMK	95.0%	80.6	22.7	25	3	0	2	1,749.88
									TPEELR	95.0%	40.6	20.7	1	0	0	2	744.39
									VLTEIIASR	95.0%	78.8	19.8	38	0	0	2	1,001.60
									WGTDEEK	95.0%	30.9	17.6	1	0	0	2	864.37
									WGTDEEKFITIFGTR	95.0%	78.2	22.1	19	28	0	2	1,799.90
									YMTISGFQIEETIDR	95.0%	56.9	22.0	3	0	0	2	1,818.86
GDP-L-fucose synthase	FCL_HUMAN	TSTA3	35,875	99.50%	2	2	10	9.35%	DADLTDTAQTR	95.0%	78.7	20.7	9	0	0	2	1,206.56
									VVADGAGLPGEDWVFVSSK	95.0%	32.2	22.0	1	0	0	2	1,932.97
Ran-specific GTPase-activating protein	RANG_HUMAN	RANBP1	23,293	100.00%	3	3	9	16.40%	FASENDLPEWK	95.0%	57.4	21.4	2	0	0	2	1,335.62
									TLEEDEEELFK	95.0%	49.3	21.3	6	0	0	2	1,381.64
									VAEKLEALSVK	95.0%	29.3	17.4	0	1	0	2	1,186.70
Ephrin type-A receptor 2	EPHA2_HUMAN	EPHA2	108,249	100.00%	4	4	8	4.30%	FADIVSILDK	95.0%	50.8	21.7	4	0	0	2	1,120.63
									LNVEER	95.0%	34.7	23.4	2	0	0	2	759.40
									VLEDDPEATYTTSGGK	95.0%	52.7	20.9	1	0	0	2	1,682.78
									YSEPPHGLTR	95.0%	27.0	22.0	0	1	0	2	1,156.58
CAP-Gly domain-containing linker protein 1	CLIP1_HUMAN	CLIP1	162,232	99.50%	2	2	2	1.95%	LTNLQENLSEVSQVK	95.0%	47.7	21.5	1	0	0	2	1,701.90
									SQQLSALQEENVK	95.0%	51.8	21.8	1	0	0	2	1,473.76
Serine/threonine-protein phosphatase 4 catalytic subunit	PP4C_HUMAN	PPP4C	35,062	99.50%	2	2	4	9.77%	EILVEESNVQR	95.0%	37.7	22.9	2	0	0	2	1,315.69
									VGGDVPETNYLFMGDFVDR	95.0%	70.8	20.9	2	0	0	2	2,130.98
6-phosphogluconolactonase	6PGL_HUMAN	PGLS	27,530	100.00%	8	8	19	49.20%	ELPAAVAPAGPASLAR	95.0%	43.7	19.6	3	0	0	2	1,490.83
									ILEDQEENPLPAALVQPHTGK	95.0%	62.6	20.6	0	2	0	2	2,299.19
									IVAPISDSPKPPPQR	95.0%	59.0	18.2	2	0	0	2	1,601.90
									LCWFLDEAAAR	95.0%	56.5	22.3	1	0	0	2	1,351.65
									LPIPESQVITINPELPVEEAAEDYAK	95.0%	28.7	19.7	0	1	0	2	2,865.48

									LVPFDHAESTYGLYR	95.0%	35.5	22.5	2	0	0	2	1,767.87
									TVIFVATGEGK	95.0%	47.7	22.0	2	0	0	2	1,121.62
									VTLTLPVLNAAR	95.0%	68.2	12.6	6	0	0	2	1,267.77
Small nuclear ribonucleoprotein Sm D3	SMD3_HUMAN	SNRPD3	13,899	99.50%	2	2	4	15.10%	FLILPDMLK	95.0%	31.4	19.8	3	0	0	2	1,089.64
									VAQLEQVYIR	95.0%	75.2	22.7	1	0	0	2	1,218.68
Heterogeneous nuclear ribonucleoprotein H	HNRH1_HUMAN	HNRNPH1	49,212	100.00%	6	9	90	18.70%	ATENDIYNFFSPLNPVR	95.0%	74.0	22.4	23	1	0	2	1,996.98
									GLPWSCSADEVQR	95.0%	34.1	20.8	1	0	0	2	1,504.69
									HTGPNSPDTANDGFVR	95.0%	97.0	20.9	12	12	0	2	1,684.77
									SNNVEMDWVLK	95.0%	40.3	21.6	1	0	0	2	1,350.64
									STGEAFVQFASQEIAEK	95.0%	104.0	22.0	36	0	0	2	1,841.89
									VHIEIGPDGR	95.0%	44.3	22.2	3	1	0	2	1,092.58
D-3-phosphoglycerate dehydrogenase	SERA_HUMAN	PHGDH	56,633	99.50%	2	2	2	3.19%	DLPLLLFR	95.0%	41.0	19.3	1	0	0	2	986.60
									TLGILGLGR	95.0%	30.7	18.9	1	0	0	2	899.57
Glyoxalase domain-containing protein 4	GLOD4_HUMAN	GLOD4	34,776	100.00%	6	7	20	22.00%	ELPDLEDLMK	95.0%	46.2	21.8	2	0	0	2	1,202.60
									GGVDHAAAFGR	95.0%	55.9	21.5	4	1	0	2	1,057.52
									ILTPLVSLDTPGK	95.0%	60.9	15.6	8	0	0	2	1,353.80
									IYEKDEEK	95.0%	31.1	22.7	1	0	0	2	1,053.51
									LLDDAMAADKSDEWFAK	95.0%	33.1	20.4	0	1	0	2	1,941.89
									VTLAVSDLQK	95.0%	53.3	21.0	3	0	0	2	1,073.62
Isoleucyl-tRNA synthetase, cytoplasmic	SYIC_HUMAN	IARS	144,484	100.00%	3	3	4	2.06%	FLIQNVLR	95.0%	33.8	16.9	2	0	0	2	1,002.61
									LESDYEILER	95.0%	34.5	22.8	1	0	0	2	1,266.62
									LLILMEAR	95.0%	41.6	20.4	1	0	0	2	974.57
Myosin-Ic	MYO1C_HUMAN	MYO1C	121,709	100.00%	2	2	2	3.29%	GEELLSPLNLEQAAYAR	95.0%	34.5	22.0	1	0	0	2	1,873.97
									VLQALGSEPIQYAVPVVK	95.0%	47.5	15.6	1	0	0	2	1,911.10
Interleukin-18	IL18_HUMAN	IL18	22,309	100.00%	2	2	6	8.81%	SDIIFFQR	95.0%	51.8	21.9	3	0	0	2	1,025.54
									TIFIISMYK	95.0%	37.1	22.6	3	0	0	2	1,131.61
Calsyntenin-1	CSTN1_HUMAN	CLSTN1	109,774	100.00%	30	36	929	28.70%	AASEFESSEGVFLFPELR	95.0%	85.6	22.3	188	2	0	2	2,014.98
									AMQHISYLNSR	95.0%	75.3	23.0	21	14	0	2	1,319.65
									ATEDVLVK	95.0%	65.0	23.6	20	0	0	2	874.49
									ATVHIQVNDVNEYAPVFK	95.0%	78.9	21.9	25	50	0	2	2,044.05
									ATVIEGK	95.0%	44.9	25.5	16	0	0	2	717.41
									EGLDLQVLEDSGR	95.0%	108.0	22.2	124	0	0	2	1,430.71
									EKLDCELQK	95.0%	32.4	23.9	1	0	0	2	1,162.58
									EPFTISVWMR	95.0%	42.3	22.2	16	0	0	2	1,281.63
									ETILCSSDK	95.0%	36.5	21.2	1	0	0	2	1,052.49
									ETILCSSDKTDMNR	95.0%	65.2	19.8	2	0	0	2	1,685.75
									GNLAGLTLR	95.0%	67.2	20.8	80	0	0	2	914.54
									GPDGTNVK	95.0%	61.1	20.0	4	0	0	2	787.40
									GPDGTNVKK	95.0%	36.8	21.6	3	0	0	2	915.49
									GVQIQAHPSQLVLTLEGEDLGELDK	95.0%	64.9	19.8	0	20	0	2	2,689.41
									IHGQNVPFDAVVVDK	95.0%	101.0	21.5	35	28	0	2	1,637.87
									IISTITR	95.0%	55.9	17.9	44	0	0	2	803.50
									IPDGVVSVSPK	95.0%	74.8	19.4	98	0	0	2	1,097.62
									IPDGVVSVSPKEPFTISVWMR	95.0%	46.4	21.3	0	2	0	2	2,360.23
									ISIKPTCTPGWQGWNNR	95.0%	42.5	22.2	1	11	0	2	2,014.99
									ISLSGVHHFAR	95.0%	78.3	21.0	6	16	0	2	1,223.66
									LDCELQK	95.0%	37.1	22.4	6	0	0	2	905.44
									LIFLFR	95.0%	44.1	12.8	17	0	0	2	808.51
									LTVTAYDCGK	95.0%	49.2	22.3	8	0	0	2	1,127.54
									QFPTPGIR	95.0%	44.6	23.1	7	0	0	2	915.51
									QYDSILR	95.0%	51.9	22.4	17	0	0	2	894.47

									SLLDRK	95.0%	32.3	23.2	3	0	0	2	731.44
									STGEGVIR	95.0%	53.5	24.3	16	0	0	2	818.44
									VIDCLYTCK	95.0%	64.3	21.1	24	0	0	2	1,171.55
									YISNEFK	95.0%	35.7	20.2	2	0	0	2	900.45
									YRPAEFHWK	94.6%	26.2	23.6	0	1	0	2	1,233.62
Non-histone chromosomal protein HMG-14	HMGN1_HUMAN	HMGN1	10,641	99.50%	2	2	4	29.00%	TEESPASDEAGEKEAK	94.9%	26.5	19.9	0	1	0	2	1,677.75
									VSSAEGAAKEEPK	95.0%	70.8	22.5	3	0	0	2	1,302.65
Calumenin	CALU_HUMAN	CALU	37,090	100.00%	3	3	5	8.25%	EQFVEFR	95.0%	33.2	21.2	1	0	0	2	954.47
									TFDQLTPEESK	95.0%	33.0	22.4	2	0	0	2	1,294.62
									WIYEDVER	95.0%	33.1	21.6	2	0	0	2	1,109.53
Tumor-associated calcium signal transducer 2	TACD2_HUMAN	TACSTD2	35,692	100.00%	3	3	13	12.10%	AAGDVDIGDAAYYFER	95.0%	99.6	20.0	9	0	0	2	1,732.78
									DIKGESLFQGR	94.6%	30.1	23.3	2	0	0	2	1,249.65
									TLIYYLDEIPPK	95.0%	39.4	20.6	2	0	0	2	1,464.80
Collagen alpha-1(XVII) chain	COHA1_HUMAN	COL17A1	150,404	100.00%	5	7	22	4.28%	GAMGPAGPDGHQGPR	95.0%	73.6	20.2	2	3	0	2	1,420.64
									GEQGLTGMPGIR	95.0%	41.9	23.3	2	0	0	2	1,231.61
									LLSTDASHSR	95.0%	55.3	21.8	5	4	0	2	1,086.55
									LQGMAPAAGADLDK	95.0%	60.0	22.3	4	0	0	2	1,373.67
									SELISYLTSPDVR	95.0%	44.1	22.6	2	0	0	2	1,479.77
Nucleoside diphosphate kinase A	NDKA_HUMAN	NME1	17,131	100.00%	4	6	9	65.80%	DRPFFAGLVK	95.0%	47.4	20.3	2	1	0	2	1,149.64
									FMQASEDLLK	95.0%	61.9	22.9	2	0	0	2	1,181.59
									GDFCIQVGR	95.0%	51.1	22.8	4	0	0	2	1,051.50
									GLVGEIIK	95.0%	36.3	16.7	2	0	0	2	828.52
									GLVGEIIKR	95.0%	52.3	16.7	5	0	0	2	984.62
									NIIHGSDSVESAEK	95.0%	73.1	21.9	2	1	0	2	1,485.72
									TFIAIKPDGVQR	95.0%	34.9	19.8	2	0	0	2	1,344.76
									VMLGETNPADSKPGTIR	95.0%	88.7	22.3	4	8	0	2	1,785.92
									YMHSGPVVAMVWEGLNVVK	95.0%	45.3	21.4	0	1	0	2	2,116.07
T-complex protein 1 subunit beta	TCPB_HUMAN	CCT2	57,472	100.00%	11	12	21	26.50%	AAHSEGNTTAGLDMR	95.0%	40.3	18.6	0	2	0	2	1,546.69
									DASLMVTNDGATILK	95.0%	43.9	22.2	1	0	0	2	1,564.79
									EAVAMESYAK	95.0%	44.8	20.5	4	0	0	2	1,114.51
									GATQQILDEAER	95.0%	86.1	22.1	2	0	0	2	1,330.66
									IGVNQPK	95.0%	32.4	20.0	1	0	0	2	755.44
									ILIANTGMDTDKIK	95.0%	44.7	21.6	1	0	0	2	1,548.83
									LGGSLADSYLDEGFLLDK	95.0%	85.8	22.6	2	0	0	2	1,912.95
									LGGSLADSYLDEGFLLDKK	95.0%	38.9	22.0	0	2	0	2	2,041.05
									LKGSGNLEAIHIIK	94.9%	18.6	14.3	0	0	1	2	1,492.89
									SLHDALCVLAQTVK	95.0%	31.2	20.8	1	0	0	2	1,554.83
									VQDDEVGDGTTSVTVLAAELLR	95.0%	141.0	21.9	2	2	0	2	2,288.16
Peptidyl-prolyl cis-trans isomerase H	PPIH_HUMAN	PPIH	19,190	100.00%	3	4	10	13.00%	IENVPTGPNNKPK	95.0%	29.8	21.1	0	2	0	2	1,407.76
									IIDGLLVMR	95.0%	55.0	19.9	4	0	0	2	1,029.61
									KIENVPTGPNNKPK	95.0%	63.0	19.5	2	2	0	2	1,535.85
Ubiquitin-conjugating enzyme E2 L3	UB2L3_HUMAN	UBE2L3	17,844	99.50%	2	3	8	24.00%	IEINFPAEYPFKPPK	95.0%	32.4	21.4	0	1	0	2	1,789.95
									TDQVIQSLIALVNDPQPEHPLR	95.0%	92.6	19.2	2	5	0	2	2,483.33
Polypyrimidine tract-binding protein 1	PTBP1_HUMAN	PTBP1	57,205	100.00%	7	8	24	20.00%	DYGNSPLHR	95.0%	31.7	21.7	2	0	0	2	1,058.50
									HQNVQLPR	95.0%	43.5	21.4	2	0	0	2	991.54
									IAIPGLAGAGNSVLLVSNLNPER	95.0%	144.0	14.0	7	2	0	2	2,275.28
									NFQNIFPPSATLHLSNIPPSVSEEDLK	95.0%	29.3	20.6	0	1	0	2	2,994.52
									NNQFQALLQYADPVSAQHAK	95.0%	51.2	21.6	0	6	0	2	2,243.12
									VLFSSNGGVVK	95.0%	48.2	21.1	2	0	0	2	1,106.62
									VTNLLMLK	95.0%	47.7	19.5	2	0	0	2	931.57
Nicotinamide	NAMPT_HUMAN	NAMPT	55,505	100.00%	3	3	9	8.55%	AVPEGFVIPR	95.0%	33.9	19.5	1	0	0	2	1,084.62

phosphoribosyltransferase									GTDTVAGLALIK	95.0%	59.0	21.6	2	0	0	2	1,158.67
									VIQGDGVDINTLQEIVEGMK	95.0%	126.0	20.7	6	0	0	2	2,158.11
Probable aminopeptidase NPEPL1	PEPL1_HUMAN	NPEPL1	55,843	99.50%	2	2	6	5.16%	ELGIIPTIIRDEELK	95.0%	34.5	15.6	1	0	0	2	1,739.00
									TVEINNTDAEGR	95.0%	59.6	21.7	5	0	0	2	1,318.62
Glucose-6-phosphate 1-dehydrogenase	G6PD_HUMAN	G6PD	59,240	100.00%	18	22	114	41.20%	CISEVQANNVVLGQYVGNPDGEGEATK	95.0%	70.8	20.9	0	2	0	2	2,848.34
									DGLLPENTFIVGYAR	95.0%	79.2	22.8	11	0	0	2	1,664.87
									EMVQNLMVLR	95.0%	64.1	22.9	8	0	0	2	1,232.65
									GGYFDEFGIIR	95.0%	54.9	21.8	9	0	0	2	1,273.62
									GPTEADELMK	95.0%	40.2	21.9	3	0	0	2	1,106.50
									GPTEADELMKR	95.0%	51.0	22.9	4	0	0	2	1,262.61
									GYLDDPTVPR	95.0%	45.3	23.1	5	0	0	2	1,132.56
									IFGPIWNR	95.0%	41.0	24.8	6	0	0	2	1,002.55
									IIVEKPFGR	95.0%	34.0	18.6	2	0	0	2	1,058.64
									KPGMFFNPEESELDLTYGNR	95.0%	43.0	19.3	0	4	0	2	2,360.09
									LFYLALPPTVYEAVTK	95.0%	48.6	17.6	7	0	0	2	1,825.02
									LKLEDFFAR	95.0%	43.6	20.3	7	10	0	2	1,138.63
									LNSHMNALHLGSQANR	95.0%	61.4	22.6	1	1	2	2	1,778.87
									LPDAYER	95.0%	45.2	24.0	5	0	0	2	863.43
									LSNHISSLFR	95.0%	33.4	23.7	1	2	0	2	1,173.64
									NSYVAGQYDDAASYQR	95.0%	98.6	19.3	19	0	0	2	1,807.79
									QSEPFFK	95.0%	33.2	21.5	2	0	0	2	882.44
									VGFQYEGTYK	95.0%	37.1	22.9	3	0	0	2	1,191.57
Eukaryotic translation initiation factor 2 subunit 1	IF2A_HUMAN	EIF2S1	36,095	100.00%	7	7	15	23.80%	AGLNCSTENMPIK	95.0%	42.6	19.7	2	0	0	2	1,450.67
									GVFNVQMEPK	95.0%	38.4	23.2	2	0	0	2	1,164.57
									INLIAPPR	95.0%	45.0	13.0	2	0	0	2	893.56
									LTPQAVK	95.0%	33.3	15.4	1	0	0	2	756.46
									TEGLSVLSQAMAVIK	95.0%	37.7	20.3	1	0	0	2	1,546.85
									VVTDTDETELAR	95.0%	78.6	23.8	6	0	0	2	1,348.66
									YVMTTTTLER	94.8%	30.2	22.2	1	0	0	2	1,214.61
Proteasome subunit alpha type-7	PSA7_HUMAN	PSMA7	27,869	100.00%	12	15	93	45.60%	AITVFSPDGHLFQVEYAQEAVK	95.0%	56.5	21.7	2	5	0	2	2,449.24
									AITVFSPDGHLFQVEYAQEAVKK	95.0%	35.1	20.7	0	2	0	2	2,577.34
									ALLEVVQSGGK	95.0%	81.3	22.7	12	0	0	2	1,100.63
									DIVVLGVEK	95.0%	45.2	19.8	2	0	0	2	971.58
									GRDIVVLGVEK	95.0%	63.9	19.2	6	0	0	2	1,184.70
									GSTAVGVR	95.0%	37.6	24.2	1	0	0	2	746.42
									ILNPEEIEK	95.0%	34.7	20.8	3	0	0	2	1,084.59
									KGSTAVGVR	95.0%	43.8	21.7	1	0	0	2	874.51
									LTVEDPVTVEYITR	95.0%	102.0	22.1	17	2	0	2	1,634.86
									LYQTDPSGTYHAWK	95.0%	70.9	21.8	4	1	0	2	1,666.79
									NIELAVMR	95.0%	40.2	24.5	4	0	0	2	945.52
									NYTDEAIETDDLTIK	95.0%	136.0	21.2	31	0	0	2	1,740.82
Semaphorin-7A	SEM7A_HUMAN	SEMA7A	74,806	100.00%	5	5	21	8.86%	AAAIQTMSLDAER	95.0%	61.3	22.1	3	0	0	2	1,392.68
									GDQGGESSLSVSK	95.0%	66.7	22.9	6	0	0	2	1,250.59
									LQDVFLLPDPSGQWR	95.0%	51.4	22.5	4	0	0	2	1,770.92
									VSLAPNSR	95.0%	32.3	22.6	3	0	0	2	843.47
									VYLFDFPEGK	95.0%	55.8	22.2	5	0	0	2	1,214.61
Density-regulated protein	DENR_HUMAN	DENR	22,074	100.00%	4	4	11	21.70%	LDADYPLR	95.0%	46.0	23.6	4	0	0	2	962.49
									QEAGISEGQGTAGEEEEK	95.0%	82.1	18.8	3	0	0	2	1,848.81
									WPEVDDDSIEDLGEVK	95.0%	95.8	20.5	2	0	0	2	1,845.84
									WPEVDDDSIEDLGEVKK	95.0%	42.7	21.5	0	2	0	2	1,973.93
ADP-ribose pyrophosphatase,	NUDT9_HUMAN	NUDT9	39,108	99.50%	2	2	2	7.43%	EFGEEALNSLQK	95.0%	40.9	22.7	1	0	0	2	1,364.67

mitochondrial									WADPQISESNFSPK	95.0%	48.9	22.0	1	0	0	2	1,605.76
Glycogen debranching enzyme	GDE_HUMAN	AGL	174,750	100.00%	11	11	19	8.88%	AVTVYTNYPFPGETFNR	95.0%	68.9	21.2	2	0	0	2	1,975.96
									FPSLPILSPALMDVPYR	95.0%	44.0	19.8	1	0	0	2	1,932.03
									IPFASLASR	95.0%	31.6	21.2	1	0	0	2	961.55
									LEQGYELQFR	94.8%	30.2	21.9	1	0	0	2	1,282.64
									LGISSLIR	95.0%	43.2	20.1	2	0	0	2	858.54
									LGPTLQGK	95.0%	30.9	17.9	1	0	0	2	813.48
									LTLAELNQILYR	95.0%	49.8	17.5	2	0	0	2	1,446.83
									SDPNQHLTIIQDPEYR	94.8%	26.4	21.6	0	1	0	2	1,925.94
									SGGGYIVVDPILR	95.0%	64.2	20.8	3	0	0	2	1,345.75
									SGSLAVDNADPILK	95.0%	64.4	21.5	3	0	0	2	1,399.74
									YTWNDVGQLVEK	95.0%	59.1	22.4	2	0	0	2	1,451.72
High mobility group protein B2	HMGB2_HUMAN	HMGB2	24,017	100.00%	8	11	42	33.00%	HPDSSVNFAEFSK	95.0%	36.0	21.1	1	0	0	2	1,464.68
									IKSEHPGLSIGDTAK	95.0%	62.0	21.0	2	2	0	2	1,552.83
									KLGEMWSEQSAK	95.0%	47.2	22.2	2	0	0	2	1,409.67
									LGEMWSEQSAK	95.0%	65.6	20.6	2	0	0	2	1,265.58
									MSSYAFFVQTCR	95.0%	104.0	19.7	19	0	0	2	1,512.66
									SEHPGLSIGDTAK	95.0%	47.2	22.5	5	1	0	2	1,311.65
									SKFEDMAK	95.0%	32.1	20.0	1	0	0	2	955.46
									YEKDIAAYR	95.0%	50.4	22.0	5	2	0	2	1,128.57
Phosphatidylethanolamine-binding protein 1	PEBP1_HUMAN	PEBP1	21,039	100.00%	8	9	55	59.40%	GNDISSGTVLSDYVGSGPPK	95.0%	121.0	22.7	14	0	0	2	1,949.95
									LYEQLSGK	95.0%	58.7	20.9	12	0	0	2	937.50
									LYTLVLTDPDAPSR	95.0%	97.2	22.5	13	0	0	2	1,560.83
									LYTLVLTDPDAPSRK	95.0%	64.7	20.2	0	3	0	2	1,688.92
									NRPTSISWDGLDSGK	95.0%	79.3	22.9	6	3	0	2	1,632.80
									VLTPTQVK	95.0%	30.4	17.2	1	0	0	2	885.54
									WSGPLSLQEVDEQPQHPLHVTYAGAAVDELGK	95.0%	26.1	19.2	0	0	2	2	3,471.72
									YVWLVYEQDRPLK	95.0%	38.1	21.3	1	0	0	2	1,708.91
Protein FAM49B	FA49B_HUMAN	FAM49B	36,731	100.00%	11	13	37	42.00%	AWGAVVPLVGK	95.0%	48.5	17.6	3	0	0	2	1,096.65
									DAEGILEDLQSYR	95.0%	84.7	22.3	4	0	0	2	1,508.72
									DQPPNSVEGLLNALR	95.0%	81.7	21.5	6	0	0	2	1,622.85
									EAIQHPADEK	95.0%	39.8	23.2	1	0	0	2	1,137.55
									EAIQHPADEKLQEK	95.0%	74.0	22.8	2	2	0	2	1,635.83
									GLLGALTSTPYSPTQHLER	95.0%	59.2	21.3	2	2	0	2	2,041.07
									HLNDETTSK	95.0%	45.4	19.7	1	0	0	2	1,044.50
									INNVPAEGENEVNNELANR	95.0%	94.3	21.6	2	0	0	2	2,096.00
									MSLFYAEATPMLK	95.0%	65.6	22.7	5	0	0	2	1,533.73
									MTNPAIQNDFSYYR	95.0%	99.5	21.5	4	0	0	2	1,735.78
									VMLETPEYR	95.0%	40.3	23.0	3	0	0	2	1,137.56
Uncharacterized glycosyltransferase AER61	AER61_HUMAN	AER61	61,994	100.00%	3	3	5	5.69%	EAVFSLLPR	95.0%	65.3	20.8	2	0	0	2	1,031.59
									ELGFLDQLR	95.0%	44.9	23.2	2	0	0	2	1,090.59
									ILNQNELVNALK	95.0%	45.6	17.6	1	0	0	2	1,368.79
F-actin-capping protein subunit alpha-2	CAZA2_HUMAN	CAPZA2	32,931	100.00%	3	3	10	27.60%	EGAAHAFAQYNLDQFTPVK	95.0%	36.8	22.3	0	2	0	2	2,107.03
									FIIHAPPGEFNEVFNDVR	95.0%	36.6	21.8	0	2	0	2	2,101.05
									FTITPSTTQVVGILK	95.0%	78.8	14.1	6	0	0	2	1,604.93
									IQVHYYEDGNVQLVSHK	95.0%	40.7	22.2	0	11	4	2	2,029.01
									LLLNNDNLLR	95.0%	72.5	19.2	14	0	0	2	1,197.70
60S ribosomal protein L7	RL7_HUMAN	RPL7	29,210	100.00%	2	2	4	10.10%	IALTDNALIAR	95.0%	70.4	20.1	2	0	0	2	1,170.68
									IVEPYIAWGYPNLK	95.0%	55.3	20.3	2	0	0	2	1,662.89
Leucyl-tRNA synthetase, cytoplasmic	SYLC_HUMAN	LARS	134,453	99.50%	2	2	3	1.96%	FDDPLLGPR	95.0%	35.2	22.0	1	0	0	2	1,029.54
									STGNFLTLTQAIDK	95.0%	61.7	21.7	2	0	0	2	1,508.80

Semaphorin-5A	SEM5A_HUMAN	SEMA5A	120,597	100.00%	2	2	4	2.42%	LADPNLLEVGR	95.0%	54.9	19.9	2	0	0	2	1,196.66
									NAVDFSQLTFDPGQK	95.0%	69.2	22.4	2	0	0	2	1,666.81
Metalloproteinase inhibitor 2	TIMP2_HUMAN	TIMP2	24,382	100.00%	7	9	36	25.90%	AVSEKEVDSGNDIYGNPIK	95.0%	103.0	22.8	2	2	0	2	2,035.00
									EVDSGNDIYGNPIK	95.0%	80.1	22.6	3	0	0	2	1,520.72
									EVDSGNDIYGNPIKR	95.0%	91.5	22.2	10	2	0	2	1,676.82
									EYLIAGK	95.0%	34.0	21.8	5	0	0	2	793.45
									GAAPPKQEFLDIEDP	95.0%	48.1	22.6	7	0	0	2	1,626.80
									IQYEIK	95.0%	40.9	21.8	4	0	0	2	793.45
									SDGSCAWYR	95.0%	35.0	15.4	1	0	0	2	1,101.44
Glyceraldehyde-3-phosphate dehydrogenase	G3P_HUMAN	GAPDH	36,035	100.00%	14	19	204	51.90%	AGAHLQGGAK	95.0%	69.1	21.2	15	0	0	2	909.49
									GALQNIIPASTGAAK	95.0%	95.3	20.5	25	0	0	2	1,411.79
									IISNASCTTNCLAPLAK	95.0%	93.8	22.3	4	0	0	2	1,833.92
									LISWYDNEFGYSNR	95.0%	97.8	21.3	6	0	0	2	1,763.80
									LTGMAFR	95.0%	46.0	22.0	8	0	0	2	811.41
									LVINGNPITIFQER	95.0%	86.9	20.6	22	0	0	2	1,613.90
									LVINGNPITIFQERDPSK	95.0%	62.0	18.6	2	1	0	2	2,041.11
									RVIISAPSADAPMFVMGVNHEK	95.0%	47.1	21.7	0	4	0	2	2,401.20
									VIHDNFGIVEGLMTTVHAITATQK	95.0%	45.6	20.2	0	13	21	2	2,611.36
									VIISAPSADAPMFVMGVNHEK	95.0%	90.5	21.7	6	2	0	2	2,213.11
									VPTANVSVVDLTCR	95.0%	119.0	22.9	20	2	0	2	1,530.80
									VVDLMAHMASK	95.0%	47.8	22.7	2	0	0	2	1,201.61
									VVDLMAHMASKE	95.0%	61.5	22.5	18	0	0	2	1,330.65
									WGDAGAEYVVESTGVFTTMEK	95.0%	133.0	20.1	21	12	0	2	2,293.03
T-complex protein 1 subunit eta	TCPH_HUMAN	CCT7	59,350	100.00%	14	15	37	37.00%	ALEIIPR	95.0%	43.5	15.4	2	0	0	2	811.50
									EGTDSSQGIPQLVSNISACQVIAEAVR	95.0%	32.8	20.6	0	1	0	2	2,829.41
									GGAEQFMEETER	95.0%	63.8	16.7	6	0	0	2	1,399.58
									LLDVVHPAAK	95.0%	39.3	17.0	2	0	0	2	1,062.63
									LPIGDVATQYFADR	95.0%	96.5	22.8	2	0	0	2	1,565.80
									MVVDAVMMLDDLLQLK	95.0%	73.3	21.5	4	0	0	2	1,881.94
									QLCDNAGFDATNILNK	95.0%	57.9	23.1	2	0	0	2	1,793.85
									QVKPYVEEGLHPQIIIR	94.7%	18.5	14.3	0	0	1	2	2,019.14
									SLHDAIMIVR	94.6%	30.0	21.3	1	0	0	2	1,170.63
									SQDAEVGDGTTSVTLLAAEFLK	95.0%	135.0	22.4	5	1	0	2	2,252.13
									STVDAPTAAGR	95.0%	56.2	21.6	5	0	0	2	1,045.53
									TATQLAVNK	95.0%	44.0	22.8	2	0	0	2	945.54
									TFSYAGFEMQPK	95.0%	68.7	19.4	2	0	0	2	1,421.64
									VQGGALEDSQLVAGVAFK	95.0%	51.0	21.5	1	0	0	2	1,788.95
Eukaryotic translation initiation factor 3 subunit D	EIF3D_HUMAN	EIF3D	63,956	100.00%	3	4	8	8.94%	IFHTVTTTDDPVIR	95.0%	57.5	21.9	2	2	0	2	1,614.85
									LGDDIDLIVR	95.0%	75.9	21.7	2	0	0	2	1,128.63
									YNFPNPNPFVEDDMDKNEIASVAYR	95.0%	45.1	19.0	0	2	0	2	2,961.34
Ras GTPase-activating protein-binding protein 2	G3BP2_HUMAN	G3BP2	54,102	99.50%	2	2	3	6.22%	LPNFGFVVFDDSEPVQR	95.0%	33.3	22.2	1	0	0	2	1,965.97
									VEAKPEVQSQPPR	95.0%	33.4	21.3	0	2	0	2	1,464.78
GMP synthase [glutamine-hydrolyzing]	GUAA_HUMAN	GMPS	76,699	100.00%	4	4	7	7.94%	ELGLPEELVSR	95.0%	43.2	20.9	1	0	0	2	1,241.67
									EPPTDVTPTFLTTGVLSTLR	95.0%	88.5	19.6	2	0	0	2	2,145.14
									SGNIVAGIANESK	95.0%	46.8	23.2	1	0	0	2	1,259.66
									TLNMTTSPEEK	95.0%	37.2	22.6	3	0	0	2	1,266.59
Rho GDP-dissociation inhibitor 1	GDIR1_HUMAN	ARHGDIA	23,190	100.00%	3	5	28	22.10%	AEEYEFLTPVEEAPK	95.0%	82.3	22.6	19	0	0	2	1,751.84
									IDKTDYMVGSYGPR	95.0%	83.1	22.3	2	4	0	2	1,601.76
									SIQEIQELDKDDESLR	95.0%	90.4	22.2	2	1	0	2	1,917.94
Uridine diphosphate glucose pyrophosphatase	NUD14_HUMAN	NUDT14	24,100	100.00%	4	4	6	21.60%	IEGASVGR	95.0%	34.7	24.3	1	0	0	2	788.43
									QFRPAVYAGEVER	95.0%	31.5	22.3	0	1	0	2	1,521.78

									QTMFYTEVTDAQR	95.0%	54.0	19.2	2	0	0	2	1,605.72
									VATYWSGVGLTGSR	95.0%	79.5	22.2	2	0	0	2	1,453.74
Malignant T cell-amplified sequence 1	MCTS1_HUMAN	MCTS1	20,538	100.00%	4	6	7	31.50%	LYPAAVDTIVAIMAEGK	95.0%	82.7	20.9	2	1	0	2	1,761.95
									MSAEDIEK	95.0%	32.0	20.3	1	0	0	2	938.41
									NQLIEQFPGIEPWLNQIMPK	95.0%	50.0	20.3	1	0	0	2	2,411.24
									YPFILPHQQVDK	95.0%	34.0	21.1	1	1	0	2	1,484.79
Interleukin-1 receptor antagonist protein	IL1RA_HUMAN	IL1RN	20,037	99.90%	2	2	4	19.20%	LQLEAVNITDLSENR	95.0%	67.1	22.2	2	0	0	2	1,714.90
									NNQLVAGYLQGPNVNLEEK	95.0%	109.0	21.6	2	0	0	2	2,100.07
Serpin B3	SPB3_HUMAN	SERPINB3	44,548	100.00%	8	9	22	25.10%	DLSMIVLLPNEIDGLQK	95.0%	57.2	19.3	4	0	0	2	1,898.03
									DNTAQQIK	95.0%	53.8	23.3	1	0	0	2	917.47
									FYQTSVESVDFANAPEESR	95.0%	93.2	19.8	2	0	0	2	2,175.98
									GLVLSGVLHK	95.0%	48.7	12.3	2	0	0	2	1,022.64
									INSWVESQTNEK	95.0%	73.8	21.6	2	0	0	2	1,434.69
									LMEWTSLQNMR	95.0%	54.2	22.4	5	0	0	2	1,408.67
									VDLHLPR	95.0%	49.4	20.0	3	0	0	2	849.49
									VLHFDQVTENTTGK	95.0%	68.3	22.9	2	1	0	2	1,588.80
Serine/threonine-protein phosphatase PP1-alpha catalytic subunit	PP1A_HUMAN	PPP1CA	37,496	100.00%	6	6	11	19.70%	EIFLSQPILLELEAPLK	95.0%	56.9	13.6	3	0	0	2	1,953.13
									IKYPENFFLLR	95.0%	31.7	18.6	0	1	0	2	1,439.81
									LNLDSIIGR	95.0%	47.5	20.9	2	0	0	2	1,000.58
									NVQLTENEIR	95.0%	39.4	23.7	1	0	0	2	1,215.63
									TFTDCFNCLPIAAIVDEK	95.0%	74.1	21.4	2	0	0	2	2,113.99
									YPENFFLLR	95.0%	55.5	21.1	2	0	0	2	1,198.63
Alanyl-tRNA synthetase, cytoplasmic	SYAC_HUMAN	AARS	106,795	100.00%	9	9	15	13.10%	ASEWVQQVSGLMDGK	95.0%	41.4	22.6	1	0	0	2	1,634.79
									AVFDETYPDPVR	95.0%	47.0	22.0	2	0	0	2	1,408.68
									EIADLGEALATAVIPQWQK	95.0%	53.0	18.8	2	0	0	2	2,053.10
									GLEVTDDSPK	95.0%	38.0	23.0	2	0	0	2	1,060.52
									IVAVTGAEAQK	95.0%	40.7	21.2	2	0	0	2	1,086.62
									MALELLTQEFGIPIER	95.0%	37.4	21.2	2	0	0	2	1,875.99
									MSNYDTDLFVPYFEAIQK	95.0%	42.6	20.3	1	0	0	2	2,197.02
									QIWQNLGLDDTK	95.0%	31.2	22.4	1	0	0	2	1,430.73
									VGAEDADGIDMAYR	95.0%	72.0	19.2	2	0	0	2	1,498.65
Alcohol dehydrogenase class 4 mu/sigma chain	ADH7_HUMAN	ADH7	41,463	100.00%	2	2	3	7.25%	ISEGFELLNSGQSIR	95.0%	87.8	22.6	2	0	0	2	1,649.85
									MLTYDPMLLFTGR	95.0%	53.2	22.6	1	0	0	2	1,573.78
Pyruvate dehydrogenase phosphatase regulatory subunit, mitochondrial	PDPR_HUMAN	PDPR	99,349	99.50%	2	2	3	3.41%	INAGLNVIGIPSEIISPK	95.0%	36.1	12.8	1	0	0	2	1,835.06
									TGSIFLAQTQDR	95.0%	51.3	22.8	2	0	0	2	1,336.69
Probable ATP-dependent RNA helicase DDX17	DDX17_HUMAN	DDX17	72,355	100.00%	3	3	7	6.00%	APILIATDVASR	95.0%	57.3	18.5	5	0	0	2	1,226.71
									ELAQQVQQVADDYGK	95.0%	53.1	22.9	1	0	0	2	1,691.82
									VLEEANQAINPK	95.0%	52.7	21.3	1	0	0	2	1,325.71
Na(+)/H(+) exchange regulatory cofactor NHE-RF1	NHRF1_HUMAN	SLC9A3R1	38,850	100.00%	4	4	18	19.80%	AQEAPGQAEPPAAAEVQGAGNENEPR	95.0%	75.9	21.0	0	2	0	2	2,588.20
									EALAEAALESPRPALVR	95.0%	35.6	19.1	0	1	0	2	1,792.99
									LLVVDPETDEQLQK	95.0%	68.0	21.1	9	0	0	2	1,626.86
									SVDPDSPAEASGLR	95.0%	51.7	21.7	6	0	0	2	1,400.67
Phosphoglucomutase-1	PGM1_HUMAN	PGM1	61,433	100.00%	22	27	112	46.60%	ADNFEYSDPVDGSISR	95.0%	108.0	18.2	5	0	0	2	1,771.78
									DLEALMFDR	95.0%	48.1	21.8	7	0	0	2	1,109.53
									EAIQLIAR	95.0%	45.7	19.3	2	0	0	2	913.55
									FFGNLMDASK	95.0%	60.0	20.5	4	0	0	2	1,129.54
									FNISNGGPAPEAITDK	95.0%	104.0	22.5	2	0	0	2	1,630.81
									IAAANGIGR	95.0%	47.2	20.0	2	0	0	2	842.48
									IALYETPTGWK	95.0%	31.5	22.7	1	0	0	2	1,278.67
									IDAMHGVVGPYVK	95.0%	46.1	22.5	2	2	0	2	1,385.73
									INQDPQVMLAPLISIALK	95.0%	97.5	16.3	20	1	0	2	1,980.12

									LSGTGSAGATIR	95.0%	81.2	23.2	23	0	0	2	1,090.59
									LVIGQNGILSTPAVSCIIR	95.0%	47.1	15.8	2	0	0	2	2,011.14
									LYIDSYEKDVAK	95.0%	73.0	22.5	2	1	0	2	1,443.74
									QEATLVVGGDGR	95.0%	84.8	22.5	8	0	0	2	1,201.62
									QFSANDK	95.0%	41.8	22.1	2	0	0	2	809.38
									QFSANDKVYTVEK	95.0%	28.0	22.5	0	1	0	2	1,528.76
									SGEHDFGAAFDGDGDRNMILGK	95.0%	33.2	18.8	0	2	2	2	2,325.02
									SIFDFSALK	95.0%	39.5	20.7	1	0	0	2	1,027.55
									SMPTSGALDR	95.0%	31.6	21.4	1	0	0	2	1,050.49
									TQAYQDQKPGTSGLR	95.0%	69.5	23.0	3	4	0	2	1,649.83
									VDLGVLGK	95.0%	50.7	20.0	4	0	0	2	800.49
									VSQLQER	95.0%	55.2	23.4	2	0	0	2	859.46
									YDYEEVEAEGANK	95.0%	112.0	16.8	6	0	0	2	1,516.64
Kallikrein-11	KLK11_HUMAN	KLK11	31,041	100.00%	2	2	18	7.45%	LLCGATLIAPR	95.0%	85.1	20.6	9	0	0	2	1,184.68
									YVDWIQETMK	95.0%	52.6	20.8	9	0	0	2	1,328.62
Basic leucine zipper and W2 domain-containing protein 2	BZW2_HUMAN	BZW2	48,146	99.50%	2	2	6	6.68%	DTLVQGLNEAGDDLEAVAK	95.0%	123.0	21.7	3	0	0	2	1,957.97
									LLELFPVNR	95.0%	53.4	20.4	3	0	0	2	1,100.65
26S protease regulatory subunit 6A	PRS6A_HUMAN	PSMC3	49,187	100.00%	4	4	5	13.70%	LAGPQLVQMFIGDGAK	95.0%	34.6	21.7	1	0	0	2	1,660.87
									QTYFLPVIGLVDAEK	95.0%	60.1	19.1	2	0	0	2	1,692.92
									TMLELLNQLDGFQPNTQVK	95.0%	64.5	21.4	1	0	0	2	2,205.12
									VDILDPALLR	95.0%	32.9	13.8	1	0	0	2	1,124.67
Cadherin-1	CADH1_HUMAN	CDH1	97,440	100.00%	14	16	158	18.40%	DTANWLEINPDTGAISTR	95.0%	120.0	22.2	41	0	0	2	1,973.96
									FKVGTDGVITVK	95.0%	52.7	17.4	2	26	0	2	1,263.73
									GLDFEAK	95.0%	41.4	23.6	2	0	0	2	779.39
									GQVPENEANVVITTLK	95.0%	97.7	20.3	31	2	0	2	1,711.92
									MALEVGDYK	95.0%	64.9	21.1	9	0	0	2	1,025.50
									NDVAPTLMSVPR	95.0%	34.7	22.3	2	0	0	2	1,299.67
									NMFTINR	95.0%	30.6	21.9	2	0	0	2	895.45
									NTGVISVVTTGLDR	95.0%	121.0	20.8	10	0	0	2	1,431.78
									TAYFSLDTR	95.0%	71.1	21.2	4	0	0	2	1,073.53
									VFYSITGQGADTPPVGVFIIER	95.0%	115.0	20.2	14	0	0	2	2,366.24
									VGTDGVITVK	95.0%	80.0	21.7	5	0	0	2	988.57
									VNFEDCTGR	95.0%	60.1	17.8	3	0	0	2	1,097.47
									VTEPLDR	95.0%	34.0	21.0	4	0	0	2	829.44
									YLPRPANPDEIGNFIDENLK	95.0%	30.2	21.4	0	1	0	2	2,315.17
Cystatin-B	CYTB_HUMAN	CSTB	11,121	100.00%	3	3	12	45.90%	SQVVAGTNYFIK	95.0%	53.9	21.1	4	0	0	2	1,326.71
									VFQSLPHENKPLTLSNYQTNK	95.0%	81.3	20.6	2	0	0	2	2,458.27
									VHVGDEDFVHLR	95.0%	63.5	22.6	0	6	0	2	1,422.71
Splicing factor, proline- and glutamine-rich	SFPQ_HUMAN	SFPQ	76,132	100.00%	4	4	6	6.65%	FATHAAALSVR	95.0%	42.2	23.3	0	2	0	2	1,143.63
									LFVGNLPADITEDEFK	95.0%	66.3	22.7	2	0	0	2	1,807.91
									LFVGNLPADITEDEFKR	95.0%	28.8	22.0	0	1	0	2	1,964.01
									MGGGGAMNMGDPYGSGGQK	94.9%	30.3	10.0	1	0	0	2	1,819.71
Beta-hexosaminidase subunit alpha	HEXA_HUMAN	HEXA	60,672	100.00%	11	12	70	25.10%	ALLSAPWYLNR	95.0%	57.9	22.2	12	0	0	2	1,303.72
									DFYVVEPLAFEGTPEQK	95.0%	72.9	21.8	19	0	0	2	1,968.96
									DLLFGSGSWPRPYLTGK	95.0%	36.6	21.7	1	0	0	2	1,893.99
									EDIPVNYMK	95.0%	30.5	22.0	1	0	0	2	1,124.53
									GLETFSQLVWK	95.0%	50.5	21.0	7	0	0	2	1,307.70
									GSYNPVTHIYTAQDVK	95.0%	69.2	22.5	4	1	0	2	1,792.89
									GYVVWQEVFDNK	95.0%	54.2	22.1	2	0	0	2	1,483.72
									IQPDTIIQVWR	95.0%	50.4	19.0	8	0	0	2	1,368.76
									LTSDLTFAYER	95.0%	83.6	22.1	4	0	0	2	1,315.65

									SNPEIQDFMR	95.0%	52.0	19.6	8	0	0	2	1,252.56
									TEIEDFPR	95.0%	37.6	22.1	3	0	0	2	1,006.48
NAD(P)H dehydrogenase [quinone] 1	NQO1_HUMAN	NQO1	30,851	100.00%	5	6	12	21.20%	ALIVLAHSER	95.0%	63.9	18.3	2	0	0	2	1,108.65
									EAAAAALKK	95.0%	33.0	21.8	1	0	0	2	872.52
									EGHLSPDIVAEQK	95.0%	67.4	22.7	3	0	0	2	1,422.72
									IQILEGWK	95.0%	43.9	23.1	2	0	0	2	986.57
									LKDPANFQYPAESVLAYK	95.0%	54.7	21.2	1	3	0	2	2,054.06
Splicing factor, arginine/serine-rich 4	SFRS4_HUMAN	SFRS4	56,662	100.00%	3	3	8	11.90%	GESENAGTNQETR	95.0%	54.6	18.0	1	0	0	2	1,392.60
									LDGTEVNGR	95.0%	53.3	22.7	3	0	0	2	960.48
									LIVENLSSR	95.0%	52.6	21.6	11	0	0	2	1,030.59
									LVEDKPGSR	95.0%	59.5	22.0	4	0	0	2	1,000.54
									QAGEVTYADAHK	95.0%	51.6	21.4	3	0	0	2	1,289.61
									VIVEHAR	95.0%	44.6	18.7	4	0	0	2	823.48
Proliferation-associated protein 2G4	PA2G4_HUMAN	PA2G4	43,769	100.00%	21	23	195	54.30%	AAHLCAEAALR	95.0%	45.9	22.1	0	7	0	2	1,182.61
									AFFSEVER	95.0%	57.6	21.1	12	0	0	2	984.48
									ALLQSSASR	95.0%	61.4	22.0	16	0	0	2	932.52
									EGEFVAQFK	95.0%	47.2	21.9	9	0	0	2	1,054.52
									FDAMPFTLR	95.0%	51.3	21.5	8	0	0	2	1,113.54
									FTVLLMPNGPMR	95.0%	59.7	22.7	6	0	0	2	1,391.72
									GDAMIMEETGK	95.0%	63.8	19.7	19	0	0	2	1,213.51
									HELLQPFNVLYEK	95.0%	72.8	21.5	6	8	0	2	1,629.86
									ITSGPFEPDLYK	95.0%	76.7	22.5	7	0	0	2	1,366.69
									ITSGPFEPDLYKSEMEVQDAELK	95.0%	49.8	21.6	0	8	0	2	2,626.26
									LVKPGNQNTQVTEAWNK	95.0%	63.5	21.0	6	7	0	2	1,927.00
									MGGDIANR	95.0%	67.6	21.4	7	0	0	2	849.39
									MGVVECAK	95.0%	30.8	21.8	1	0	0	2	909.42
									RFDAMPFTLR	95.0%	31.1	21.6	1	0	0	2	1,269.64
									SDQDYILK	95.0%	62.0	22.0	11	0	0	2	981.49
									SEMEVQDAELK	95.0%	78.2	20.6	10	0	0	2	1,294.58
									SLVEASSSGVSVLSLCEK	95.0%	86.1	22.3	14	0	0	2	1,851.94
									TAENATSGETLEENEAGD	95.0%	104.0	16.0	10	0	0	2	1,837.76
									TIIQNPTDQQK	95.0%	51.4	22.0	17	0	0	2	1,285.68
									TIIQNPTDQQKK	95.0%	53.7	21.1	4	0	0	2	1,413.77
									VAHSFNCTPIEGMLSHQLK	95.0%	28.6	21.5	0	1	0	2	2,185.05
Cytosol aminopeptidase	AMPL_HUMAN	LAP3	56,150	100.00%	6	6	10	16.00%	EKEDDVPQFTSAGENFDK	95.0%	32.7	19.2	0	1	0	2	2,055.92
									GITFDSGGISIK	95.0%	40.7	22.0	1	0	0	2	1,194.64
									GSPNANEPPLVFVGK	95.0%	48.0	22.4	2	0	0	2	1,525.80
									GVLFASGQNLAR	95.0%	46.7	23.0	2	0	0	2	1,232.68
									QLMETPANEMTPTR	95.0%	59.7	20.9	2	0	0	2	1,650.75
									TIQVDNTDAEGR	95.0%	62.5	21.7	2	0	0	2	1,318.62
Phosphoribosylformylglycinamidine synthase	PUR4_HUMAN	PFAS	144,706	100.00%	8	8	14	6.35%	ELSDPAGAIIYTSR	95.0%	77.4	23.0	2	0	0	2	1,492.76
									EPGGPSPR	95.0%	32.1	20.2	1	0	0	2	796.40
									FGEPVLAGFAR	95.0%	62.2	23.0	3	0	0	2	1,163.62
									FQQQQGLR	95.0%	33.8	23.9	1	0	0	2	1,004.53
									LNFSTPTSTNIVSVCR	95.0%	68.5	22.0	2	0	0	2	1,795.90
									SLGLQLPDGQR	95.0%	51.1	20.8	2	0	0	2	1,183.64
									VGPGPALMLR	95.0%	32.2	18.8	2	0	0	2	1,010.58
									VGTETVR	95.0%	40.9	25.0	1	0	0	2	761.42
Ubiquitin	UBIQ_HUMAN	RPS27A	8,547	100.00%	5	6	102	69.70%	ESTLHLVLR	95.0%	46.9	18.4	11	0	0	2	1,067.62
									IQDKEGIPPDQQR	95.0%	73.5	21.4	13	3	0	2	1,523.78
									QLEDGR	95.0%	31.8	20.8	1	0	0	2	717.35

									TITLEVEPSDTIENVK	95.0%	86.7	21.8	71	0	0	2	1,787.93
									TLSDYNIQK	95.0%	44.2	22.3	3	0	0	2	1,081.55
Cathepsin D	CATD_HUMAN	CTSD	44,535	100.00%	14	16	473	38.60%	AIGAVPLIQGEYMIPCEK	95.0%	82.5	22.0	18	0	0	2	2,005.01
									DPDAQPGGELMLGGTDSK	95.0%	114.0	20.3	56	0	0	2	1,803.81
									FDGILGMAYPR	95.0%	84.1	22.0	74	0	0	2	1,239.62
									ISVNNVLPVFDNLMQQK	95.0%	61.3	20.6	110	20	0	2	1,959.04
									LVDQNIFSFYLSR	95.0%	78.4	22.6	39	0	0	2	1,601.83
									QPGITFIAAK	95.0%	41.5	20.5	7	0	0	2	1,045.60
									QVFGEATK	95.0%	34.4	22.7	1	0	0	2	879.46
									RTMSEVGGSVEDLIAK	95.0%	57.0	22.8	1	7	0	2	1,707.86
									TMSEVGGSVEDLIAK	95.0%	95.3	23.2	67	0	0	2	1,535.76
									VGFAEAAR	95.0%	64.6	25.0	32	0	0	2	820.43
									VGFAEAARL	95.0%	50.2	22.9	3	0	0	2	933.52
									VSTLPAITLK	95.0%	35.2	13.8	3	0	0	2	1,042.65
									YSQAVPAVTEGPIPEVLK	95.0%	98.8	19.4	34	0	0	2	1,898.03
									YYTVFDRDNNR	95.0%	31.4	20.3	1	0	0	2	1,462.67
Histidyl-tRNA synthetase, cytoplasmic	SYHC_HUMAN	HARS	57,394	100.00%	7	7	16	16.90%	AQLGPDESK	95.0%	45.6	21.2	2	0	0	2	944.47
									ASAELIEEEVAK	95.0%	74.5	23.2	4	0	0	2	1,288.66
									DQGGELLSLR	95.0%	58.1	23.7	2	0	0	2	1,087.58
									HGAEVIDTPVFELK	95.0%	55.7	21.6	2	0	0	2	1,554.82
									IFSIVEQR	95.0%	71.9	20.5	2	0	0	2	991.56
									IGDYVQQHGGVSLVEQLLQDPK	95.0%	49.2	20.3	0	2	0	2	2,423.26
									LVSELWDAGIK	95.0%	66.1	22.6	2	0	0	2	1,230.67
Leukotriene A-4 hydrolase	LKHA4_HUMAN	LTA4H	69,269	100.00%	12	12	34	29.30%	DGETPDPEDPSR	95.0%	35.3	16.3	6	0	0	2	1,314.55
									DLSSHQLNEFLAQTLQR	95.0%	36.9	21.8	0	2	0	2	2,000.02
									EDDLNSFNATDLK	95.0%	90.2	20.8	2	0	0	2	1,481.68
									ELVALMSAIR	95.0%	66.7	21.6	4	0	0	2	1,102.63
									GSPMEISLPIALSK	95.0%	75.6	21.5	4	0	0	2	1,458.79
									HFNALGGWGELQNSVK	95.0%	37.0	22.3	0	1	0	2	1,756.88
									LVVDLTDIDPDVAYSSVPYEK	95.0%	99.6	21.9	3	0	0	2	2,338.17
									MQEVYNFNAINNSEIR	95.0%	110.0	20.7	2	0	0	2	1,957.91
									SAYEFSETESMLK	95.0%	58.1	18.6	2	0	0	2	1,521.68
									SLSNVIAHEISHSWTGNLVTNK	95.0%	57.2	20.4	0	4	0	2	2,407.24
									SSALQWLTPEQTSGK	95.0%	60.9	23.3	2	0	0	2	1,632.82
									WEDAIPLALK	95.0%	37.4	19.7	2	0	0	2	1,155.64
60S acidic ribosomal protein P0	RLA0_HUMAN	RPLP0	34,256	100.00%	5	5	18	23.00%	AFLADPSAFVAAAPVAAATTAAPAAAAAPAK	95.0%	47.2	18.3	0	1	0	2	2,752.47
									GHLENNPALEK	95.0%	41.4	23.5	1	0	0	2	1,221.62
									GNVGFVFTK	95.0%	49.2	21.8	2	0	0	2	968.52
									IIQLLDDYPK	95.0%	76.7	22.0	6	0	0	2	1,217.68
									TSFFQALGITTK	95.0%	103.0	21.7	8	0	0	2	1,313.71
Cathepsin L1	CATL1_HUMAN	CTSL1	37,546	100.00%	6	7	70	22.20%	HSFTMAMNAFGDMTSEEFR	95.0%	44.7	13.4	0	2	0	2	2,240.91
									LYGMNEEGWR	95.0%	55.8	20.5	8	0	0	2	1,254.56
									MIELHNQEYR	95.0%	54.0	22.5	9	1	0	2	1,332.64
									NSWGEEWGMGGYVK	95.0%	96.3	17.6	4	0	0	2	1,615.69
									QVMNGFQNR	95.0%	60.7	21.6	2	0	0	2	1,109.52
									VFQEPLFYEAPR	95.0%	78.0	22.0	44	0	0	2	1,495.76
Pirin	PIR_HUMAN	PIR	32,096	100.00%	5	5	13	18.30%	MNPGDLQWMTAGR	95.0%	77.8	20.2	4	0	0	2	1,492.67
									MVEPQYQELK	95.0%	34.2	22.5	2	0	0	2	1,280.62
									NLDPFLLFDEFK	95.0%	38.8	23.0	1	0	0	2	1,497.76
									SEEIPKPSK	95.0%	39.7	22.3	4	0	0	2	1,014.55
									TPTLYLDFK	95.0%	41.0	21.0	2	0	0	2	1,097.59

Peroxiredoxin-4	PRDX4_HUMAN	PRDX4	30,523	100.00%	5	6	15	29.20%	DYGVYLEDSGHTLR	95.0%	76.8	21.9	2	0	0	2	1,624.76
									GLFIIDDK	95.0%	47.4	20.5	11	0	0	2	920.51
									GLFIIDDKGILR	95.0%	53.2	15.6	9	5	0	2	1,359.80
									IPLLSDLTHQISK	95.0%	55.2	17.1	3	3	0	2	1,464.84
									LVQAFQYTDK	95.0%	58.8	21.3	2	0	0	2	1,212.63
									QITLNDLPVGR	95.0%	53.0	19.9	4	0	0	2	1,225.69
									SVDETLR	95.0%	53.7	24.7	2	0	0	2	819.42
									VSVADHSLHLSK	95.0%	59.9	21.8	1	0	0	2	1,292.70
Bifunctional aminoacyl-tRNA synthetase	SYEP_HUMAN	EPRS	170,575	100.00%	3	3	9	2.84%	DQDLEPGAPSMGAK	95.0%	46.5	20.2	3	0	0	2	1,431.64
									LTVAENEAETK	95.0%	63.4	23.2	2	0	0	2	1,204.61
									NQGGGLSSSGAGEGQGPK	95.0%	77.1	21.3	4	0	0	2	1,587.74
Eukaryotic translation initiation factor 3 subunit B	EIF3B_HUMAN	EIF3B	92,465	100.00%	6	6	30	9.46%	AQAVSEDAGGNEGR	95.0%	108.0	18.6	15	0	0	2	1,360.61
									GTQGVVTNFEIFR	95.0%	65.3	22.1	4	0	0	2	1,467.76
									ITNDFYPEEDGKTK	95.0%	32.8	20.8	1	0	0	2	1,656.78
									MAQELYMEQK	95.0%	47.5	17.6	2	0	0	2	1,302.57
									TSIFWNDVKDPVSIEER	95.0%	27.0	22.9	0	1	0	2	2,035.01
									VTLMQLPTR	95.0%	65.4	22.3	7	0	0	2	1,058.60
Protocadherin-7	PCDH7_HUMAN	PCDH7	116,054	100.00%	6	7	14	7.76%	LDASEGGGGTNPGGR	95.0%	59.6	19.2	2	0	0	2	1,344.61
									LFEIDPTSGVVSLVGK	95.0%	88.6	20.1	2	0	0	2	1,660.92
									RLDASEGGGGTNPGGR	95.0%	45.3	21.1	1	4	0	2	1,500.72
									SVYEADLAENSAPGTPILQLR	95.0%	61.6	20.9	1	0	0	2	2,244.15
									VATVLATDADSGK	95.0%	52.3	23.2	2	0	0	2	1,247.65
									YELLQEPGGGGSGGESR	95.0%	62.4	22.9	2	0	0	2	1,692.78
Coatomer subunit delta	COPD_HUMAN	ARCN1	57,193	100.00%	6	6	24	13.70%	GVQLQTHPNVDKK	95.0%	45.2	20.8	1	0	0	2	1,463.80
									IEGLLAAFPK	95.0%	43.6	20.4	2	0	0	2	1,058.63
									LFTAESLIGLK	95.0%	64.9	16.7	6	0	0	2	1,191.70
									NTLEWCLPVIDAK	95.0%	55.3	23.1	1	0	0	2	1,558.79
									SFPVNSDVGVLK	95.0%	38.1	22.3	1	0	0	2	1,261.68
									VTQVDGNSPVR	95.0%	71.0	22.1	13	0	0	2	1,171.61
Plastin-2	PLSL_HUMAN	LCP1	70,274	100.00%	25	27	141	46.90%	AACLPLPGYR	95.0%	40.5	24.2	2	0	0	2	1,117.58
									ALENDPDCR	95.0%	37.8	16.5	3	0	0	2	1,089.46
									AYYHLLEQVAPK	95.0%	52.5	22.6	2	5	0	2	1,431.76
									EGESLEDLMK	95.0%	41.0	22.0	1	0	0	2	1,150.53
									EKDDIQR	95.0%	31.1	22.3	1	0	0	2	903.45
									FSLVGIGGQDLNEGNR	95.0%	86.3	22.3	8	0	0	2	1,675.84
									GDEEGVPAVVIDMSGLR	95.0%	54.7	21.4	7	0	0	2	1,743.86
									GSVSDEEMMELR	95.0%	64.4	15.8	1	0	0	2	1,414.58
									IGNFSTDIK	95.0%	48.0	21.6	2	0	0	2	994.52
									IGNFSTDIKDSK	95.0%	38.9	21.7	1	0	0	2	1,324.68
									IKVPVDWNR	95.0%	40.1	20.3	0	8	0	2	1,126.64
									ISFDEFIK	95.0%	45.9	20.5	4	0	0	2	998.52
									KLENCNYAVELGK	95.0%	63.0	22.8	2	0	0	2	1,537.77
									LSPEELLLR	95.0%	67.1	17.1	7	0	0	2	1,069.63
									MINLSVPDTIDER	95.0%	66.8	22.8	24	0	0	2	1,518.75
									NEALIALLR	95.0%	77.8	16.0	6	0	0	2	1,012.62
									NWMNSLGVNPR	95.0%	48.9	22.1	2	0	0	2	1,287.63
									QFVTATDVVR	95.0%	65.2	23.3	11	0	0	2	1,135.61
									TENLNDDEKLNNAK	95.0%	87.2	21.4	7	2	0	2	1,617.77
									VDTDGNGYISFNELNDLFK	95.0%	76.0	21.6	2	0	0	2	2,161.01
									VNDDIIVNWVNETLR	95.0%	97.8	21.8	26	0	0	2	1,799.93
									VYALPEDLVEVNPK	95.0%	52.8	20.8	2	0	0	2	1,585.85

									YAFVNWINK	95.0%	34.8	20.9	1	0	0	2	1,154.60
									YPALHKPENQDIDWGALEGETR	95.0%	75.4	22.0	0	2	0	2	2,539.22
									YTLNILEEIGGGQK	95.0%	100.0	22.2	2	0	0	2	1,534.81
Histone-binding protein RBBP4	RBBP4_HUMAN	RBBP4	47,638	100.00%	6	6	26	16.50%	IGEEQSPEDAEDGPPELLFIHGGHTAK	95.0%	62.8	20.3	0	2	0	2	2,873.36
									LMIWDTR	95.0%	34.4	23.5	2	0	0	2	934.48
									LNVWDLSK	95.0%	43.3	22.5	3	0	0	2	974.53
									TPSSDVLVFDYTK	95.0%	89.5	23.1	5	0	0	2	1,471.73
									TVALWDLR	95.0%	52.7	23.2	13	0	0	2	973.55
									VINEEYK	95.0%	32.2	22.2	1	0	0	2	894.46
Fatty acid synthase	FAS_HUMAN	FASN	273,409	100.00%	14	14	62	6.69%	AAEQYTPK	95.0%	36.0	23.4	1	0	0	2	907.45
									DNLEFFLAGIGR	95.0%	44.6	21.6	2	0	0	2	1,351.70
									EDGLAQQQTQLNLR	95.0%	78.7	22.0	4	0	0	2	1,613.83
									FPQLDSTSFANSR	95.0%	70.1	22.0	9	0	0	2	1,469.70
									GLVQALQTK	95.0%	34.6	20.0	3	0	0	2	957.57
									GNAGQSNYGFANSAMER	95.0%	79.3	16.4	1	0	0	2	1,789.76
									LPEDPLLSGLLDSPALK	95.0%	87.2	17.3	7	0	0	2	1,778.00
									LQVVDQPLPVR	95.0%	56.2	17.2	8	0	0	2	1,263.74
									SEGVVAVLLTK	95.0%	53.5	19.2	4	0	0	2	1,115.67
									SLLVNPEGPTLMR	95.0%	50.8	21.4	4	0	0	2	1,442.77
									TPEAVQK	95.0%	31.6	19.8	1	0	0	2	772.42
									VLEALLPLK	95.0%	53.6	8.5	7	0	0	2	995.65
									VLQGDLVMNVYR	95.0%	63.9	22.3	2	0	0	2	1,422.74
									VVVQVLAEEPEAVLK	95.0%	70.3	15.1	9	0	0	2	1,622.94
									ISSSDLSLGHVTR	95.0%	35.2	22.2	1	0	0	2	1,371.72
									SSGFPVHLLPDIAEPGSVAGR	95.0%	51.0	21.1	0	2	0	2	2,106.10
Hepatoma-derived growth factor	HDGF_HUMAN	HDGF	26,771	100.00%	9	12	59	42.50%	AGDLLEDSPK	95.0%	54.7	21.2	1	0	0	2	1,044.52
									DLFPYEESKEK	95.0%	35.4	22.0	2	0	0	2	1,384.66
									EAATLEVERPLPMEVEK	95.0%	42.0	22.3	2	0	0	2	1,957.00
									EAENPEGEEK	95.0%	42.8	16.1	2	0	0	2	1,131.48
									EAENPEGEEKEAATLEVERPLPMEVEK	95.0%	49.0	20.9	0	2	2	2	3,053.46
									GFSEGLWEIENNPTVK	95.0%	68.6	23.4	17	1	0	2	1,819.89
									GPPQEEEEEEDEEEEATKEDAEAPGIR	95.0%	75.4	14.6	0	7	0	2	3,042.28
									IDEMPEAAVK	95.0%	60.5	21.9	15	0	0	2	1,118.54
									KGFSEGLWEIENNPTVK	95.0%	92.6	21.8	3	5	0	2	1,947.98
Caprin-1	CAPR1_HUMAN	CAPRIN1	78,346	100.00%	3	3	14	4.23%	LDDYQER	95.0%	41.3	20.1	2	0	0	2	938.42
									LNQDQLDAVSK	95.0%	60.8	23.2	5	0	0	2	1,230.63
									YQEVTNNLEFAK	95.0%	52.2	22.4	7	0	0	2	1,455.71
Cytoplasmic FMR1-interacting protein 1	CYFP1_HUMAN	CYFIP1	145,169	100.00%	6	6	9	5.67%	AVGPSSTQLYMVR	95.0%	47.8	22.7	1	0	0	2	1,424.72
									LGTPQQIAIAR	95.0%	51.3	16.8	2	0	0	2	1,167.69
									NVIQSVLQAIR	95.0%	43.2	17.3	1	0	0	2	1,240.74
									SLLQGTILQYVK	95.0%	41.5	15.7	1	0	0	2	1,362.80
									SSLEGPTILDIEK	95.0%	50.5	22.0	2	0	0	2	1,401.75
									TVLPFSQEFQR	95.0%	42.1	21.6	2	0	0	2	1,351.70
Niban-like protein 1	NIBL1_HUMAN	FAM129B	82,666	99.50%	2	2	6	3.27%	FQELIFEDFAR	95.0%	54.6	22.6	2	0	0	2	1,414.70
									VQQVQPAMQAVIR	95.0%	65.7	21.3	4	0	0	2	1,483.81
Aldo-keto reductase family 1 member C3	AK1C3_HUMAN	AKR1C3	36,827	100.00%	14	16	233	44.60%	AIDGLDR	94.6%	30.0	23.4	1	0	0	2	759.40
									HIDSAHLYNNEEQVGLAIR	95.0%	82.9	22.0	0	56	21	2	2,179.09
									IADGSVKR	95.0%	45.2	23.3	1	0	0	2	845.48
									LAIEAGFR	95.0%	46.2	23.1	52	0	0	2	876.49
									LNDGHFMPVLGFGTYAPPEVPR	95.0%	71.0	20.9	1	6	0	2	2,430.19
									QLEMILNKPGLK	95.0%	56.0	17.6	9	0	0	2	1,383.80

									REDIFYTSK	95.0%	38.6	21.6	15	0	0	2	1,158.58
									RQLEMILNKPGLK	95.0%	28.1	14.5	0	2	0	2	1,539.90
									RTPALIALR	95.0%	37.6	10.0	2	0	0	2	1,010.65
									SIGVSNFNR	95.0%	53.8	22.6	6	0	0	2	993.51
									SKDIVLVAYSALGSQR	95.0%	60.2	18.8	0	26	0	2	1,706.94
									TPALIALR	95.0%	70.5	14.8	31	0	0	2	854.55
									WVDPNSPVLLEDPVLCALAK	95.0%	86.9	21.1	2	0	0	2	2,236.17
									YQLQR	95.0%	30.5	22.5	2	0	0	2	707.38
Importin-5	IPO5_HUMAN	IPO5	123,614	100.00%	8	9	18	10.60%	EGFVEYTEQVVK	95.0%	44.9	22.4	1	0	0	2	1,427.71
									LLSSAFDEVYPALPSDVQTAIK	95.0%	48.1	20.3	1	0	0	2	2,364.23
									LVLEQVVTSIASVADTAEEK	95.0%	108.0	19.7	2	1	0	2	2,102.12
									NLIDEDGNNQWPEGLK	95.0%	60.5	22.4	1	0	0	2	1,841.87
									NTTAAEEAR	95.0%	37.3	21.3	2	0	0	2	962.45
									SLVEIADTVPK	95.0%	59.9	22.7	4	0	0	2	1,171.66
									VCDIAAELAR	95.0%	32.8	23.3	2	0	0	2	1,117.57
									VIAALLQTMEDQGNQR	95.0%	81.9	22.3	4	0	0	2	1,802.91
NKG2D ligand 2	N2DL2_HUMAN	ULBP2	27,351	100.00%	5	6	66	15.90%	DIQLENYTPK	95.0%	59.5	23.4	6	0	0	2	1,220.62
									DIQLENYTPKEPLTLQAR	95.0%	94.6	20.8	7	6	0	2	2,129.12
									EPLTLQAR	95.0%	37.6	19.0	5	0	0	2	927.53
									EVVDILTEQLR	95.0%	87.0	22.4	38	0	0	2	1,314.73
									MWTTVHPGAR	95.0%	38.2	21.1	4	0	0	2	1,171.57
Guanine nucleotide-binding protein G(i) subunit alpha-2	GNAI2_HUMAN	GNAI2	40,434	99.50%	2	2	5	7.32%	IAQSDYIPTQQDVLR	95.0%	52.5	22.2	2	0	0	2	1,746.90
									LLLLGAGESGK	95.0%	57.8	20.1	3	0	0	2	1,057.63
Endoplasmic reticulum mannosyl-oligosaccharide 1,2-alpha-mannosidase	MA1B1_HUMAN	MAN1B1	79,564	100.00%	4	4	10	9.30%	ADSYYEYLLK	95.0%	41.7	22.7	2	0	0	2	1,264.61
									IPYSDVNIGTGVAHPPR	95.0%	35.2	21.5	0	2	0	2	1,792.93
									QETQLLEDYVEAIEGVR	95.0%	81.6	22.0	2	0	0	2	1,991.99
									VPSGGYSSINNVQDPQKPEPR	95.0%	40.8	22.0	0	4	0	2	2,269.12
Copine-1	CPNE1_HUMAN	CPNE1	59,041	100.00%	6	6	14	12.80%	DIVQFVPYR	95.0%	45.7	22.5	2	0	0	2	1,136.61
									EALAQTVLAEVPTQLVSYFR	95.0%	76.4	17.4	3	0	0	2	2,235.20
									FGIYDIDNK	95.0%	42.5	22.2	2	0	0	2	1,084.53
									FQNAPR	95.0%	31.9	23.0	1	0	0	2	732.38
									LYGPTNFAPIINHVAR	95.0%	36.9	20.6	0	2	0	2	1,782.97
									SDPFLEFFR	95.0%	71.8	21.0	4	0	0	2	1,157.56
Growth arrest-specific protein 6	GAS6_HUMAN	GAS6	79,658	100.00%	3	3	13	4.85%	GQSEVSAAQLQER	95.0%	110.0	22.7	5	0	0	2	1,402.69
									IAVAGDLFQPER	95.0%	74.3	22.5	4	0	0	2	1,315.70
									LLDLDEAAYK	95.0%	50.4	22.0	4	0	0	2	1,150.60
Splicing factor, arginine/serine-rich 3	SFRS3_HUMAN	SFRS3	19,312	100.00%	4	5	24	28.70%	AFGYYGPLR	95.0%	54.3	22.3	8	0	0	2	1,043.53
									NPPGFAFVEFEDPR	95.0%	79.2	22.1	7	0	0	2	1,621.77
									NPPGFAFVEFEDPRDAADAVR	95.0%	54.6	21.4	3	5	0	2	2,320.10
									VYVGNLGNNGNKTELER	95.0%	27.7	22.6	0	1	0	2	1,876.95
Calreticulin	CALR_HUMAN	CALR	48,125	100.00%	14	20	158	44.40%	AKIDDPTDSKPEDWDKPEHIPDPDAK	95.0%	37.9	20.5	0	3	1	2	2,959.40
									EQFLDGDGWTSR	95.0%	66.6	19.7	29	0	0	2	1,410.63
									FYALSASFEPFSNK	95.0%	103.0	21.9	13	0	0	2	1,607.77
									FYGDEEKDK	95.0%	47.9	18.4	4	3	0	2	1,130.50
									GLQTSQDAR	95.0%	55.9	23.0	5	0	0	2	975.49
									IDDPTDSKPEDWDKPEHIPDPDAK	95.0%	31.7	19.5	0	1	1	2	2,760.26
									IDNSQVESGSLEDDWDFLPPK	95.0%	137.0	20.2	11	0	0	2	2,391.10
									IDNSQVESGSLEDDWDFLPPKK	95.0%	97.1	21.5	6	51	0	2	2,519.19
									IKDPDASKPEDWDER	95.0%	66.3	21.8	5	2	1	2	1,800.84
									KPEDWDEEMDGEWEPPVIQNPEYK	95.0%	63.1	17.1	0	13	0	2	2,976.29
									KVHVIFNYK	95.0%	28.1	18.3	0	1	0	2	1,147.66

									LFPNSLDQTDMHGDSEYNIMFGPDICGPGTK	95.0%	56.1	15.6	0	2	0	2	3,488.51
									NVLINK	95.0%	30.6	22.0	1	0	0	2	700.44
									QIDNPDYK	95.0%	51.7	22.1	5	0	0	2	992.47
Tubulointerstitial nephritis antigen-like	TINAL_HUMAN	TINAGL1	52,369	100.00%	16	17	194	48.00%	ELAPGLHLR	95.0%	44.1	18.5	7	0	0	2	1,005.58
									ELMENGPVQALMEVHEDFFLYK	95.0%	27.5	21.0	0	1	0	2	2,671.24
									GGIYSHTPVSLGRPER	95.0%	33.0	21.2	1	0	0	2	1,725.90
									GVNECDIESFVLGVWGR	95.0%	43.6	21.8	3	0	0	2	1,936.92
									GVVSDHCYPFSGR	95.0%	62.7	19.9	4	0	0	2	1,480.66
									HGTHSVK	95.0%	42.6	22.6	4	0	0	2	765.40
									ITGWGEETLPDGR	95.0%	90.1	22.2	51	0	0	2	1,430.69
									IYPVLGTYWDNCNR	95.0%	78.4	21.4	11	0	0	2	1,770.83
									LDGAWWFLR	95.0%	73.5	23.1	75	0	0	2	1,163.60
									LGSNDKEIMK	95.0%	34.3	22.8	3	11	0	2	1,150.58
									LGTIRPSSSVMNMHEIYTVLNPGEVLPTAFEASEK	95.0%	35.0	19.3	0	1	0	2	3,849.90
									QATAHCPNSYVNNNDIYQVTPVYR	95.0%	55.6	21.0	0	2	0	2	2,824.31
									RELAPGLHLR	95.0%	56.2	16.8	0	3	0	2	1,161.69
									VGMEDMGHH	95.0%	38.2	12.0	3	0	0	2	1,028.39
									YCQEQDLCCR	95.0%	50.7	9.5	5	0	0	2	1,431.55
									YWTAANSWGPAWGER	95.0%	82.9	22.3	9	0	0	2	1,751.79
Adenosine kinase	ADK_HUMAN	ADK	40,529	100.00%	4	4	6	11.30%	ELFDELVK	95.0%	34.3	20.9	1	0	0	2	992.53
									IVIFTQGR	95.0%	33.7	19.6	3	0	0	2	933.55
									VAQWMIQQPHK	95.0%	27.6	22.7	0	1	0	2	1,365.71
									YSLKPNDQILAEDK	95.0%	32.2	22.2	0	1	0	2	1,633.84
Pigment epithelium-derived factor	PEDF_HUMAN	SERPINF1	46,326	100.00%	7	7	23	19.90%	DTDTGALLFIGK	95.0%	81.0	22.3	6	0	0	2	1,250.66
									ELLDTVTAPQK	95.0%	41.5	22.6	4	0	0	2	1,214.66
									LAAAVSNFGYDLYR	95.0%	72.0	23.1	2	0	0	2	1,559.79
									LDLQEINNWVQAQMK	95.0%	56.2	22.3	2	0	0	2	1,845.92
									LQSLFDSPDFSK	95.0%	69.2	22.7	6	0	0	2	1,383.68
									LTQVEHR	95.0%	63.7	21.0	1	0	0	2	882.48
									TSLEDFYLDEER	95.0%	61.2	19.6	2	0	0	2	1,516.68
Reticulocalbin-2	RCN2_HUMAN	RCN2	36,860	99.50%	2	2	4	12.30%	LSEEEILENPDLFLTSEATDYGR	95.0%	45.2	21.0	1	0	0	2	2,641.25
									WDPTANEDPEWILVEK	95.0%	52.1	20.8	3	0	0	2	1,941.92
DNA damage-binding protein 1	DDB1_HUMAN	DDB1	126,952	100.00%	21	22	64	19.70%	ALVSEWKEPQAK	95.0%	53.9	21.7	2	0	0	2	1,385.74
									ALYYLQIHPQELR	95.0%	34.2	20.5	0	1	0	2	1,643.89
									DSAATTDEER	95.0%	57.8	16.5	4	0	0	2	1,094.46
									EATADDLIK	95.0%	32.6	23.3	2	0	0	2	975.50
									EMLGGEIIPR	95.0%	63.9	22.0	8	0	0	2	1,114.59
									GDFILVGDLMR	95.0%	46.7	22.8	2	0	0	2	1,235.65
									IAVMELFRPK	95.0%	66.8	18.3	2	0	0	2	1,203.69
									IEVQDTSGGTTALRPSASTQALSSSVSSSK	95.0%	47.2	20.3	0	5	0	2	2,952.48
									IVVFQYSDGK	95.0%	63.5	21.7	4	0	0	2	1,155.61
									LEELHVIDVK	95.0%	54.0	18.6	2	0	0	2	1,194.67
									LPSFELLHK	95.0%	32.9	19.5	1	1	0	2	1,083.62
									LQTVAEK	95.0%	30.7	23.7	1	0	0	2	788.45
									LVFSNVNLK	95.0%	49.2	17.6	4	0	0	2	1,033.60
									LVSQEPK	95.0%	33.2	21.1	1	0	0	2	800.45
									LYEWTTEK	95.0%	30.6	20.5	1	0	0	2	1,069.52
									QGQGQLVTCSGAFK	95.0%	78.1	22.1	2	0	0	2	1,480.72
									TVPLYESPR	95.0%	37.0	22.3	1	0	0	2	1,061.56
									VTLGTQPTVLR	95.0%	74.0	19.2	15	0	0	2	1,184.70
									VVEELTR	95.0%	39.9	23.4	3	0	0	2	845.47

									YLAIAPPIIK	95.0%	31.1	16.0	1	0	0	2	1,098.69
									YLLGDMEGR	95.0%	42.5	23.0	1	0	0	2	1,053.50
Protein FAM3C	FAM3C_HUMAN	FAM3C	24,663	100.00%	11	13	412	54.60%	AIQDGTIVLMGTYDDGATK	95.0%	145.0	22.3	143	1	0	2	1,984.95
									GINVALANGK	95.0%	52.1	20.2	3	0	0	2	956.55
									ICLEDNVLMSGVK	95.0%	77.2	23.2	10	0	0	2	1,493.73
									LIADLGSTSITNLGFR	95.0%	149.0	19.5	133	8	0	2	1,677.92
									MASGAANVVGPK	95.0%	92.1	21.8	60	0	0	2	1,101.57
									MDASLGNLFAR	95.0%	81.6	23.3	6	0	0	2	1,194.59
									SALDTAAR	95.0%	64.1	23.6	9	0	0	2	804.42
									SPFEQHIK	95.0%	57.2	20.8	25	0	0	2	985.51
									TGEVLDTK	95.0%	48.8	24.6	6	0	0	2	862.45
									TKSPFEQHIK	95.0%	38.0	21.1	0	6	0	2	1,214.65
									YFDMWGGDVAPFIEFLK	95.0%	62.0	21.2	2	0	0	2	2,050.96
60S ribosomal protein L22	RL22_HUMAN	RPL22	14,769	99.50%	2	2	3	18.80%	AGNLGGGVVTIER	95.0%	48.1	22.9	1	0	0	2	1,242.68
									ITVTSEVPFSK	95.0%	58.1	22.0	2	0	0	2	1,207.66
Sortilin	SORT_HUMAN	SORT1	92,052	99.50%	2	2	2	2.29%	ADLGALELWR	95.0%	64.7	22.6	1	0	0	2	1,143.62
									IYSFGLGGR	95.0%	30.5	21.5	1	0	0	2	969.52
Heterogeneous nuclear ribonucleoprotein A1	ROA1_HUMAN	HNRNPA1	38,729	100.00%	10	14	257	39.50%	DYFEQYGK	95.0%	47.3	19.2	9	0	0	2	1,049.46
									EDSQRPGAHLTVK	95.0%	59.3	22.1	22	0	0	2	1,437.75
									EDTEEHHLR	95.0%	44.3	20.9	5	0	0	2	1,165.52
									GFAFVTFDDHDSVDK	95.0%	78.1	20.1	25	2	0	2	1,699.76
									GFGFVTYATVEEVDAAMNARPHK	95.0%	32.7	22.0	0	0	7	2	2,526.21
									IEVIEIMTDR	95.0%	72.2	23.6	33	0	0	2	1,234.64
									IFVGGIK	95.0%	34.6	16.5	1	0	0	2	733.46
									IFVGGIKEDTEEHHLR	95.0%	41.6	21.8	0	17	1	2	1,879.97
									KLFIGGLSFETTDESLR	95.0%	47.9	21.6	2	1	0	2	1,913.00
									LFIGGLSFETTDESLR	95.0%	124.0	21.4	99	0	0	2	1,784.91
									NQGGYGGSSSSSSYGSGR	95.0%	128.0	15.7	14	0	0	2	1,694.70
									SESPKEPEQLR	95.0%	49.8	21.9	18	20	0	2	1,299.65
									SSGPYGGGGQYFAKPR	95.0%	99.9	21.7	4	1	0	2	1,628.78
Basic leucine zipper and W2 domain-containing protein 1	BZW1_HUMAN	BZW1	48,027	100.00%	4	4	10	8.83%	AEVLSEEPILK	95.0%	73.6	20.2	4	0	0	2	1,227.68
									DINAVAASLR	95.0%	43.6	21.3	2	0	0	2	1,029.57
									GFSESER	95.0%	35.8	17.7	1	0	0	2	811.36
									LMELFPANK	95.0%	34.2	23.6	3	0	0	2	1,062.57
Testin	TES_HUMAN	TES	47,978	100.00%	4	4	17	10.90%	EGDPAIYAER	95.0%	41.7	22.6	1	0	0	2	1,120.53
									NVMILTNPVAAK	95.0%	70.3	20.3	6	0	0	2	1,270.72
									QPVAGSEGAQYR	95.0%	36.2	23.0	2	0	0	2	1,262.61
									STPAAVGAMEDK	95.0%	48.5	22.0	8	0	0	2	1,192.55
Fascin	FSCN1_HUMAN	FSCN1	54,512	100.00%	25	32	511	61.30%	DVPWGVDSLITLAFQDQR	95.0%	51.9	21.6	1	0	0	2	2,060.05
									FLIVAHDDGR	95.0%	57.9	22.4	17	11	0	2	1,142.60
									GDHAGVLK	95.0%	50.2	18.9	3	0	0	2	796.43
									KVTGTLDANR	95.0%	44.6	23.2	4	1	0	2	1,074.59
									LINRPIIVFR	95.0%	46.0	10.4	4	33	0	2	1,240.79
									LSCFAQTVSPAEK	95.0%	99.6	21.8	9	0	0	2	1,437.70
									LVARPEPATGYTLEFR	95.0%	65.3	20.7	24	69	0	2	1,819.97
									NGQLAASVETAGDSELFLMK	95.0%	82.5	22.1	20	0	0	2	2,097.02
									QGMDLSANQDEETDQETFQLEIDR	95.0%	48.1	16.5	0	5	0	2	2,828.22
									QGMDLSANQDEETDQETFQLEIDRDTK	95.0%	83.5	16.8	0	10	0	2	3,172.39
									QIWTLEQPPDEAGSAAVCLR	95.0%	90.4	21.6	4	0	0	2	2,241.10
									SSYDVFQLEFNDGAYNIK	95.0%	115.0	21.5	34	0	0	2	2,109.98
									VGKDELFALEQSCAQVVLQAANER	95.0%	72.7	20.8	0	10	0	2	2,675.35

									VNASASSLK	95.0%	62.8	22.9	8	0	0	2	876.48
									VNASASSLKK	95.0%	51.6	18.9	7	0	0	2	1,004.57
									VTGTLDANR	95.0%	60.7	23.6	12	0	0	2	946.50
									WSLQSEAHR	95.0%	57.7	22.0	21	1	0	2	1,113.54
									YAHLSARPADEIAVDR	95.0%	18.8	22.0	0	0	1	2	1,783.91
									YFGGTEDR	95.0%	51.8	17.0	8	0	0	2	944.41
									YLAADKDGNVTCER	95.0%	81.6	21.0	2	0	0	2	1,611.74
									YLAPSGPSGTLK	95.0%	44.2	22.5	3	0	0	2	1,190.64
									YLKGDHAGVLK	95.0%	47.3	22.3	3	9	0	2	1,200.67
									YLTAEAFGFK	95.0%	45.8	23.8	12	0	0	2	1,146.58
									YSVQTADHR	95.0%	66.2	22.2	24	1	0	2	1,076.51
									YWTLTATGGVQSTASSK	95.0%	149.0	22.4	140	0	0	2	1,757.87
N(G),N(G)-dimethylarginine dimethylaminohydrolase 1	DDAH1_HUMAN	DDAH1	31,104	100.00%	7	8	29	33.00%	ALPESLGQHALR	95.0%	37.3	20.8	2	0	0	2	1,291.71
									DYAVSTVPVADGLHLK	95.0%	76.4	21.8	3	0	0	2	1,684.89
									GAEILADTFK	95.0%	51.5	23.3	2	0	0	2	1,064.56
									SAKGEEVDVAR	95.0%	47.0	23.5	2	4	0	2	1,160.59
									SFCSMAGPNLIAIGSSESAQK	95.0%	73.3	21.6	2	0	0	2	2,155.02
									TPEEYPESAK	95.0%	61.9	21.0	12	0	0	2	1,150.53
									VDGLLTCCSVLINK	95.0%	81.4	22.7	2	0	0	2	1,591.82
Proteasome subunit beta type-6	PSB6_HUMAN	PSMB6	25,340	100.00%	6	7	28	21.30%	DGSSGGVIR	95.0%	76.0	23.6	4	0	0	2	847.43
									LAAIAESGVER	95.0%	98.9	23.0	8	0	0	2	1,115.61
									LTPIHDR	95.0%	31.2	20.7	1	0	0	2	851.47
									QVLLGDQIPK	95.0%	34.5	17.8	1	0	0	2	1,110.65
									TTTGSYIANR	95.0%	67.8	22.0	10	0	0	2	1,083.54
									VTDKLTPIHDR	95.0%	48.7	21.0	2	2	0	2	1,294.71
Ribosyldihydronicotinamide dehydrogenase [quinone]	NQO2_HUMAN	NQO2	25,935	99.50%	2	3	5	13.00%	SLASDITDEQKK	95.0%	62.2	23.0	2	0	0	2	1,334.68
									VLAPQISFAPEIASEEER	95.0%	78.4	21.4	2	1	0	2	1,986.02
Alpha-centractin	ACTZ_HUMAN	ACTR1A	42,597	100.00%	3	3	4	12.50%	AQYYLPDGSTIEIGPSR	95.0%	35.4	22.1	1	0	0	2	1,866.92
									DQLQTFSEEHPVLLTEAPLNPR	95.0%	39.9	20.3	0	2	0	2	2,534.29
									EYEEDGAR	95.0%	33.1	16.2	1	0	0	2	968.40
Amyloid beta A4 protein	A4_HUMAN	APP	86,923	100.00%	16	19	237	22.60%	AVIQHFQEKVESLEQEAANER	95.0%	51.5	20.6	0	11	0	2	2,455.22
									CAPFFYGGCGGNR	95.0%	31.8	13.4	2	0	0	2	1,462.60
									CLVGEFVSDALLVPDK	95.0%	83.1	22.8	3	0	0	2	1,761.91
									EQNYSDDVLANMISEPR	95.0%	109.0	19.6	27	2	0	2	1,996.89
									ISYGNDALMPSLTETK	95.0%	90.6	21.8	16	0	0	2	1,739.85
									LALENYITALQAVPPRPR	95.0%	85.2	14.1	2	59	0	2	2,022.15
									MDAEFR	95.0%	32.5	17.9	2	0	0	2	768.34
									MSQVMR	95.0%	37.9	20.2	2	0	0	2	767.35
									MSQVMREWEEAER	95.0%	33.3	18.7	0	1	0	2	1,712.74
									QQLVETHMAR	95.0%	47.2	21.9	2	2	0	2	1,212.62
									SQVMTHLR	95.0%	31.2	21.4	2	0	0	2	971.51
									THPHFVIPYR	95.0%	36.9	21.4	0	28	0	2	1,266.67
									VEAMLNDR	95.0%	57.2	21.9	32	0	0	2	963.46
									VESLEQEAANER	95.0%	79.8	21.3	33	0	0	2	1,374.65
									WDSDPSGTK	95.0%	40.1	19.2	3	0	0	2	992.43
									WYFDVTEGK	95.0%	43.8	19.8	8	0	0	2	1,144.53
Thioredoxin domain-containing protein 17	TXD17_HUMAN	TXNDC17	13,922	100.00%	5	6	16	43.90%	DPNNDFR	95.0%	32.0	18.1	2	0	0	2	877.38
									SWCPDCVQAEPVVR	95.0%	84.4	19.9	2	0	0	2	1,702.77
									TIFAYFTGSK	95.0%	43.0	23.8	3	0	0	2	1,134.58
									VTAVPTLLK	95.0%	40.8	11.8	4	0	0	2	941.60
									YEEVSVSGFEEFHR	95.0%	54.4	20.3	2	3	0	2	1,714.77

Complement C3	CO3_HUMAN	C3	187,131	100.00%	33	35	98	25.60%	ADIGCTPGSGK	95.0%	45.8	21.2	1	0	0	2	1,062.49
									AEDLVGK	95.0%	41.6	24.4	2	0	0	2	731.39
									AGDFLEANYMNLQR	95.0%	71.1	21.5	7	0	0	2	1,657.76
									APSTWLTAYVVK	95.0%	53.1	20.4	2	0	0	2	1,335.73
									AYYENSPQQVFSTEFEVK	95.0%	74.1	21.3	3	0	0	2	2,166.00
									EYVLPSFEVIVEPTEK	95.0%	81.1	21.1	4	0	0	2	1,878.97
									FYYIYNEK	95.0%	37.2	22.6	2	0	0	2	1,139.54
									GYTQQLAFR	95.0%	48.3	21.5	3	0	0	2	1,083.56
									ILLQGTPVAQMTEDAVDAER	95.0%	119.0	21.9	10	0	0	2	2,173.08
									IPIEDGSGEVVLSR	95.0%	100.0	22.0	2	0	0	2	1,470.78
									ISLPESLKR	95.0%	32.7	18.6	1	0	0	2	1,042.63
									KQELSEAEQATR	95.0%	73.7	22.9	2	0	0	2	1,389.70
									LVAYYTLIGASGQR	95.0%	54.1	20.5	3	0	0	2	1,511.82
									NTMILEICTR	95.0%	34.6	22.8	1	0	0	2	1,266.62
									QELSEAEQATR	95.0%	58.8	22.3	2	0	0	2	1,261.60
									QLANGVDR	95.0%	68.4	21.3	2	0	0	2	872.46
									QNQELK	95.0%	39.3	23.4	1	0	0	2	759.40
									QPSSAFAAFVK	95.0%	31.7	22.6	1	0	0	2	1,152.61
									SEETKENEGFTVTAEGK	95.0%	31.4	21.5	0	1	0	2	1,855.86
									SGQSEDRQPVPGQQMTLK	95.0%	49.3	21.6	2	0	0	2	2,001.97
									SGSDEVQVGQQR	95.0%	71.4	21.1	10	0	0	2	1,289.61
									SNLDEDIIAEENIVSR	95.0%	91.5	22.3	4	0	0	2	1,816.89
									SSLSVPYVIVPLK	95.0%	54.2	12.0	9	0	0	2	1,401.84
									TIYTPGSTVLYR	95.0%	37.9	21.1	1	0	0	2	1,370.73
									TMQALPYSTVGNSNNYLHLSVLR	95.0%	34.3	20.6	0	2	0	2	2,594.30
									TVMVNIENPEGIPVK	95.0%	50.9	21.5	2	0	0	2	1,655.87
									VFLDCCNYITELR	95.0%	65.7	20.9	2	0	0	2	1,702.79
									VHQYFNVELIQPGAVK	95.0%	32.3	19.8	0	2	0	2	1,841.99
									VLLDGVQNPR	95.0%	52.4	20.2	2	0	0	2	1,110.63
									VPVAVQGEDTVQSLTQGDGVAK	95.0%	90.8	21.4	3	2	0	2	2,198.13
									VQLSNDFDEYIMAIEQTIK	95.0%	133.0	21.2	2	0	0	2	2,273.10
									VTIKPAPETEK	95.0%	40.1	19.3	1	2	0	2	1,212.68
									YYTYLIMNK	95.0%	37.5	22.5	2	0	0	2	1,224.60
Sushi repeat-containing protein SRPX2	SRPX2_HUMAN	SRPX2	52,954	100.00%	4	4	26	11.00%	DSADGTITR	95.0%	53.9	22.9	6	0	0	2	935.44
									EQQLSANIIEELR	95.0%	62.1	21.8	1	0	0	2	1,542.81
									HVTIIELVGQPPQEVGR	95.0%	34.5	18.1	0	4	0	2	1,872.03
									LLIISAPDPSNR	95.0%	55.1	17.4	15	0	0	2	1,295.73
Fumarylacetoacetase	FAAA_HUMAN	FAH	46,358	100.00%	4	4	9	10.50%	ASSVVVSGTPIR	95.0%	79.4	22.4	4	0	0	2	1,172.66
									HLFTGPVLSK	95.0%	34.2	18.6	2	0	0	2	1,098.63
									LGEPIPISK	95.0%	34.9	14.9	1	0	0	2	953.57
									VFLQNLLSVSQAR	95.0%	84.4	18.4	2	0	0	2	1,474.84
Splicing factor, arginine/serine-rich 7	SFRS7_HUMAN	SFRS7	27,350	100.00%	2	2	6	13.90%	NPPGFAFVEFEDPR	95.0%	79.2	22.1	7	0	0	2	1,621.77
									NPPGFAFVEFEDPRDAEDAVR	95.0%	36.5	20.5	0	4	0	2	2,378.11
									VYVGNLGTGAGK	95.0%	42.8	22.8	2	0	0	2	1,135.61
Rho GDP-dissociation inhibitor 2	GDIR2_HUMAN	ARHGDIB	22,970	100.00%	10	12	74	62.70%	APEPHVEEDDDDELDSK	95.0%	103.0	16.2	4	4	0	2	1,939.80
									APEPHVEEDDDDELDSKLNYKPPPQK	95.0%	21.0	20.4	0	0	1	2	3,005.40
									APNVVVTR	95.0%	52.3	18.1	13	0	0	2	855.51
									ATFMVGSYGPRPEEYEFLTPVEEAPK	95.0%	61.1	21.8	0	12	0	2	2,960.40
									ELQEMDKDDESLIK	95.0%	64.9	23.0	11	1	0	2	1,692.80
									LNYKPPPQK	95.0%	28.2	19.8	0	4	0	2	1,084.62
									LTLVCESAPGPITMDLTGDLEALKK	95.0%	31.3	20.8	0	3	0	2	2,688.38

									TGVKVDK	95.0%	35.2	22.4	1	0	0	2	746.44
									TLLGDGPVVTDPK	95.0%	73.8	20.9	15	0	0	2	1,311.72
									YVQHTYR	95.0%	37.0	22.1	5	0	0	2	966.48
Peroxiredoxin-1	PRDX1_HUMAN	PRDX1	22,093	100.00%	14	17	183	55.30%	ADEGISFR	95.0%	59.6	22.6	12	0	0	2	894.43
									AEEFKK	95.0%	30.6	23.1	1	0	0	2	751.40
									ATAVMPDGQFK	95.0%	58.5	22.6	19	0	0	2	1,180.57
									DISLSDYK	95.0%	37.4	21.6	5	0	0	2	940.46
									GLFIIDDK	95.0%	47.4	20.5	11	0	0	2	920.51
									GLFIIDDKGILR	95.0%	53.2	15.6	9	5	0	2	1,359.80
									IGHPAPNFK	95.0%	45.3	21.7	1	0	0	2	980.53
									KQGGLGPMNIPLVSDPK	95.0%	54.7	18.2	3	1	0	2	1,750.95
									LVQAFQFTDK	95.0%	67.5	21.2	54	0	0	2	1,196.63
									QGGLGPMNIPLVSDPK	95.0%	108.0	22.0	17	0	0	2	1,638.85
									QGGLGPMNIPLVSDPKR	95.0%	62.5	19.6	3	3	0	2	1,778.96
									QITVNDLPVGR	95.0%	82.9	21.0	23	0	0	2	1,211.68
									SVDETLR	95.0%	53.7	24.7	2	0	0	2	819.42
									TIAQDYGVLK	95.0%	60.4	21.7	14	0	0	2	1,107.61
Proteasome subunit alpha type-3	PSA3_HUMAN	PSMA3	28,416	100.00%	10	11	77	37.60%	AVENSSTAIGIR	95.0%	92.8	23.2	11	0	0	2	1,217.65
									DIREEAEK	95.0%	39.4	22.9	4	0	0	2	989.49
									EEASNFR	95.0%	45.5	21.0	4	0	0	2	852.39
									ESLKEEDESDDDNM	95.0%	77.1	8.5	14	0	0	2	1,671.62
									HEIVPK	95.0%	34.8	19.9	3	0	0	2	722.42
									HVGMAVAGLLADAR	95.0%	81.5	21.8	6	8	0	2	1,396.74
									LYEEGSNKR	95.0%	48.6	21.4	4	0	0	2	1,095.54
									SLADIAR	95.0%	31.3	25.5	1	0	0	2	745.42
									SNFGYNIPLK	95.0%	58.0	22.3	12	0	0	2	1,152.61
									VFQVEYAMK	95.0%	57.8	21.9	10	0	0	2	1,130.56
Tyrosine-protein kinase-like 7	PTK7_HUMAN	PTK7	118,374	100.00%	6	6	13	7.38%	ADGSSLPEWVTDNAGTLHFAR	95.0%	37.8	22.0	0	1	0	2	2,244.07
									DDVTGEEAR	95.0%	48.7	15.9	2	0	0	2	991.43
									HPASEAEIQPQTQVTLR	95.0%	29.8	22.5	0	2	0	2	1,904.98
									QPSSQDALQGR	95.0%	47.2	21.8	4	0	0	2	1,186.58
									VFTAGSEER	95.0%	35.6	21.3	1	0	0	2	995.48
									VVLAPQDVVVAR	95.0%	48.0	14.5	3	0	0	2	1,265.76
Syntaxin-12	STX12_HUMAN	STX12	31,625	99.50%	2	2	2	10.50%	ELGSLPLPLSTSEQR	95.0%	37.3	20.8	1	0	0	2	1,626.87
									QLEADILDVNQIFK	95.0%	45.5	21.1	1	0	0	2	1,645.88
Apolipoprotein B-100	APOB_HUMAN	APOB	515,554	100.00%	2	2	4	0.48%	IEGNLIFDPNNYLPK	95.0%	31.6	21.7	2	0	0	2	1,746.91
									NLLVALK	95.0%	41.8	12.0	2	0	0	2	770.51
