# Supplementary material for: Secretome-Based Identification of ULBP2 as a Novel Serum Marker for Pancreatic Cancer Detection
Source: PLoS One. 2011 May 20;6(5):e20029. doi: 10.1371/journal.pone.0020029 (PMC3098863; doi:10.1371/journal.pone.0020029)
Supplement: Table S2 — List of proteins identified in the MIA PaCa-2 conditioned medium. (PDF) [file pone.0020029.s006.pdf]

Supporting Table S2. List of proteins identified in the MIA PaCa-2 conditioned medium

Protein name	Accession No.	Gene symbol	Molecular weight (Da)	Protein probability	No. of unique peptides	No. of unique spectra	Spectral counts	Sequence coverage	Peptide sequence	Peptide probability	Mascot ion score	Mascot identity score	No. of identified spectra: 2+ spectra	3+ spectra	4+ spectra	No. of tryptic termini	Calculated MH+
Non-POU domain-containing octamer-binding protein	NONO_HUMAN	NONO	54,214	100.00%	2	2	5	6.16%	FAQPGSFEYEYAMR	95.0%	49.0	18.6	1	0	0	2	1,711.74
									MGQMAMGGAMGINNR	95.0%	73.6	12.8	4	0	0	2	1,602.65
Apolipoprotein A-I-binding protein	AIBP_HUMAN	APOA1BP	31,657	100.00%	5	5	17	20.10%	GLTVPIASIDIPSGWDVEK	95.0%	52.0	20.2	1	0	0	2	1,997.06
									GNAGGIQPDLLISLTAPK	95.0%	84.2	17.8	7	0	0	2	1,764.99
									KSATQFTGR	95.0%	56.4	21.8	2	0	0	2	995.53
									LFGYEPTIYYPK	95.0%	50.3	24.0	6	0	0	2	1,490.76
									SATQFTGR	95.0%	37.2	21.4	1	0	0	2	867.43
COP9 signalosome complex subunit 5	CSN5_HUMAN	COPS5	37,562	100.00%	7	8	15	29.90%	GSFMLGLETHDR	95.0%	33.2	21.4	1	0	0	2	1,378.64
									GYKPPDEGPSEYQTIPLNK	95.0%	34.0	22.1	1	2	0	2	2,133.05
									ISALALLK	95.0%	40.8	14.1	3	0	0	2	828.56
									LEQSEAQLGR	95.0%	31.8	22.3	1	0	0	2	1,130.58
									SGGNLEVMGLMLGK	95.0%	57.2	21.9	3	0	0	2	1,437.71
									TTIEAIHGLMSQVIK	95.0%	37.9	20.8	2	0	0	2	1,656.90
									VDGETMIIMDSFALPVEGTETR	95.0%	61.5	21.1	2	0	0	2	2,443.14
Multifunctional protein ADE2	PUR6_HUMAN	PAICS	47,062	100.00%	9	12	36	22.60%	ASILNTWISLK	95.0%	50.3	18.6	5	0	0	2	1,245.72
									DLKEVTPEGLQMVK	95.0%	58.6	22.6	3	2	0	2	1,602.84
									DQITAGNAAR	95.0%	57.6	21.4	4	0	0	2	1,016.51
									EVTPEGLQMVK	95.0%	45.6	23.9	4	0	0	2	1,246.64
									EVYELLDSPGK	95.0%	42.1	23.3	2	0	0	2	1,249.63
									IKAEYEGDGIPTVFVAVAGR	95.0%	39.8	20.0	0	5	0	2	2,092.11
									ITSCIFQLLQEAGIK	95.0%	82.5	20.5	2	0	0	2	1,720.93
									TKEVYELLDSPGK	95.0%	74.4	21.8	2	2	0	2	1,478.77
									VTSAHKGPDETLR	95.0%	48.8	21.9	2	0	3	2	1,410.73
Antithrombin-III	ANT3_HUMAN	SERPINC1	52,586	100.00%	2	2	4	6.03%	ATEDEGSEQKIPEATNR	95.0%	29.3	21.5	0	2	0	2	1,874.87
									TSDQIHFFFAK	95.0%	49.0	22.1	2	0	0	2	1,340.66
Matrilin-2	MATN2_HUMAN	MATN2	106,819	100.00%	4	4	11	6.49%	AIEEELQEIASEPTNK	95.0%	47.0	21.3	3	0	0	2	1,800.89
									QFVTGIIDSLTISPK	95.0%	70.9	17.2	4	0	0	2	1,618.91
									SLGEENFEVVK	95.0%	43.0	22.7	2	0	0	2	1,250.63
									VIMIVTDGRPQDSVAEVAAK	95.0%	32.5	20.4	0	2	0	2	2,115.11
Histone H2B type 1-C/E/F/G/I	H2B1C_HUMAN	HIST1H2BC	13,919	100.00%	7	7	49	52.40%	AMGIMNSFVNDIFER	95.0%	113.0	21.3	33	0	0	2	1,759.81
									EIQTAVR	95.0%	33.4	23.8	2	0	0	2	816.46
									IAGEASR	95.0%	45.8	24.5	3	0	0	2	703.37
									KESYSVYVYK	95.0%	54.8	23.1	2	0	0	2	1,265.64
									LAHYNKR	95.0%	34.6	21.7	1	0	0	2	901.50
									LLLPGELAK	95.0%	42.0	13.8	7	0	0	2	953.60
									QVHPDTGISSK	95.0%	41.9	22.4	0	1	0	2	1,168.60
Heterogeneous nuclear ribonucleoproteins A2/B1	ROA2_HUMAN	HNRNPA2B1	37,412	100.00%	17	23	316	51.00%	EDTEEHHLR	95.0%	47.4	20.9	2	0	0	2	1,165.52
									EESGKPGAHVTVK	95.0%	55.8	22.6	17	0	0	2	1,338.70
									GFGFVTFDDHDPVDK	95.0%	83.4	20.5	18	6	0	2	1,695.77
									GFGFVTFDDHDPVDKIVLQK	95.0%	60.2	21.6	2	30	0	2	2,277.16
									GGGGNFGPGPGSNFR	95.0%	56.6	21.1	31	0	0	2	1,377.63
									GGNFGFGDSR	95.0%	61.0	18.6	26	0	0	2	1,013.44
									GGSDGYGSGR	95.0%	78.2	16.1	3	0	0	2	912.38
									IDTIEIITDR	95.0%	101.0	23.1	22	0	0	2	1,188.65
									KLFIGGLSFETTEESLR	95.0%	90.8	20.7	2	3	0	2	1,927.02
									LFIGGLSFETTEESLR	95.0%	126.0	21.7	80	0	0	2	1,798.92
									LFVGGIKEDTEEHHLR	95.0%	50.5	21.8	0	11	1	2	1,879.97

									LTDCVVMR	95.0%	40.3	21.6	1	0	0	2	1,009.48
									LTDCVVMRDPASK	95.0%	48.1	22.7	3	0	0	2	1,491.73
									NMGGPYGGGNYGPGGSGGSGGYGGR	95.0%	162.0	14.1	13	6	0	2	2,189.91
									QEMQEVQSSR	95.0%	63.6	19.7	23	0	0	2	1,237.55
									TLETVPLER	95.0%	43.8	20.9	12	0	0	2	1,057.59
									YHTINGHNAEVR	95.0%	36.1	22.0	0	2	2	2	1,410.69
Glutaredoxin-3	GLRX3_HUMAN	GLRX3	37,415	100.00%	10	10	17	29.60%	ASVMLFMK	95.0%	41.7	20.8	4	0	0	2	958.47
									ELKENGELLPILR	95.0%	66.7	15.6	2	0	0	2	1,523.88
									ELPQVSFVK	95.0%	36.8	21.6	2	0	0	2	1,046.59
									ENGELLPILR	95.0%	47.5	19.1	2	0	0	2	1,153.66
									GELVGGLDIVK	95.0%	48.6	21.6	1	0	0	2	1,099.64
									HASSGSFLPSANEHLK	94.7%	25.7	22.8	0	1	0	2	1,681.83
									HNIQFSSFDIFSDEEVR	95.0%	27.3	21.3	0	1	0	2	2,069.96
									LEAEGVPEVSEK	95.0%	54.6	22.8	1	0	0	2	1,286.65
									LEAEGVPEVSEKYEISSVPTFLFFK	95.0%	30.5	20.6	0	1	0	2	2,845.46
									YEISSVPTFLFFK	95.0%	41.6	22.5	2	0	0	2	1,577.83
Laminin subunit gamma-1	LAMC1_HUMAN	LAMC1	177,583	100.00%	8	8	60	7.15%	AFDITYVR	95.0%	40.6	21.7	2	0	0	2	984.52
									EAQQALGSAAADATEAK	95.0%	111.0	22.7	7	0	0	2	1,631.79
									LNTFGDEVFNDPK	95.0%	76.8	22.7	7	0	0	2	1,495.71
									LSAEDLVLEGAGLR	95.0%	124.0	21.5	18	0	0	2	1,442.79
									NTIEETGNLAEQAR	95.0%	102.0	22.1	5	0	0	2	1,545.75
									QDIAVISDSYFPR	95.0%	75.5	22.5	5	0	0	2	1,510.75
									TFAEVTDLDNEVNNMLK	95.0%	87.9	21.5	5	0	0	2	1,968.92
									VSVPLIAQGNSYPSETTVK	95.0%	94.2	20.5	11	0	0	2	1,990.05
Flap endonuclease 1	FEN1_HUMAN	FEN1	42,576	99.90%	2	2	5	9.47%	EAHQLFLEPEVLDPESVELK	95.0%	72.0	20.8	0	4	0	2	2,322.19
									QLQQAQAAGAEQEVEK	95.0%	95.8	22.1	1	0	0	2	1,727.86
Cell growth regulator with EF hand domain protein 1	CGRE1_HUMAN	CGREF1	31,887	100.00%	7	7	26	42.90%	ENGEEAKELPGETLESK	95.0%	49.2	22.0	1	0	0	2	1,859.89
									GEAGGQAEAEGDAPGPR	95.0%	98.3	19.0	6	0	0	2	1,568.69
									HVEPGEPLAPSPQEPQAVGR	95.0%	51.8	22.3	0	4	0	2	2,095.06
									RESLDPVQEPGGQAEADGDVPGPR	95.0%	83.0	21.6	0	5	0	2	2,476.17
									SPLRQETQEAPGPR	95.0%	33.3	22.6	0	3	0	2	1,565.80
									TEVQLEHLSR	95.0%	67.3	21.8	6	0	0	2	1,211.64
									VLETQDLNGDGLMTPAELINFPGVALR	95.0%	54.3	19.1	0	1	0	2	2,899.49
Phosphoserine phosphatase	SERB_HUMAN	PSPH	24,990	100.00%	5	5	8	28.90%	FYFNGEYAGFDETQPTAESGGK	95.0%	97.6	15.4	2	0	0	2	2,415.04
									LALIQPSR	95.0%	32.4	15.3	2	0	0	2	897.55
									LIAEQPPHLTPGIR	94.8%	25.7	17.6	0	1	0	2	1,541.88
									NVQVFLISGGFR	95.0%	75.7	20.3	2	0	0	2	1,336.74
									SIVEHVASK	95.0%	38.4	19.8	1	0	0	2	969.54
Asparagine synthetase [glutamine-hydrolyzing]	ASNS_HUMAN	ASNS	64,354	100.00%	2	2	4	4.10%	ELYLFDVLR	95.0%	59.0	20.5	2	0	0	2	1,167.64
									LAVVDPLFGMQPIR	95.0%	59.1	20.6	2	0	0	2	1,571.86
Complement C1r subcomponent	C1R_HUMAN	C1R	80,102	100.00%	10	12	66	16.90%	FCGQLGSPLGNPPGK	95.0%	75.9	22.4	2	0	0	2	1,528.76
									FCGQLGSPLGNPPGKK	95.0%	30.3	21.7	0	1	0	2	1,656.85
									IAHDLR	95.0%	42.4	19.2	5	0	0	2	724.41
									LGNHPIR	95.0%	38.0	20.5	4	0	0	2	806.46
									MGNFPWQVFTNIHGR	95.0%	53.2	23.2	1	9	0	2	1,819.87
									QRPPDLDTSSNAVDLLFFTDESGDSR	95.0%	71.3	20.5	0	12	0	2	2,882.34
									TLDEFTIIQNLQPQYQFR	95.0%	93.5	22.2	14	2	0	2	2,254.15
									VSVHPDYR	95.0%	42.2	21.5	2	0	0	2	972.49
									WILTAAHTLYPK	95.0%	57.5	20.0	2	0	0	2	1,413.79
									YTTTMGVNTYK	95.0%	60.2	21.4	12	0	0	2	1,294.60
40S ribosomal protein S2	RS2_HUMAN	RPS2	31,307	100.00%	6	7	40	18.80%	GTGIVSAPVPK	95.0%	46.1	17.0	8	0	0	2	1,025.60

									LSIVPVR	94.7%	30.2	12.8	1	0	0	2	783.51
									LSIVPVRR	95.0%	42.6	10.0	2	0	0	2	939.61
									SLEEIYLFSLPIK	95.0%	50.9	18.5	14	0	0	2	1,551.87
									SPYQEFTDHLVK	95.0%	61.2	22.7	7	6	0	2	1,463.72
									TYSYLTPDLWK	95.0%	50.4	22.2	2	0	0	2	1,386.69
Protein CDV3 homolog	CDV3_HUMAN	CDV3	27,317	99.50%	2	2	3	14.30%	KTPQGPPEIYSDTQFPSLQSTAK	95.0%	39.5	21.3	0	2	0	2	2,520.26
									LQLDNQYAVLENQK	95.0%	61.9	22.3	1	0	0	2	1,675.87
Eukaryotic translation initiation factor 3 subunit A	EIF3A_HUMAN	EIF3A	166,557	100.00%	12	12	48	11.90%	AVEDIHGLFSLSK	95.0%	59.4	21.6	2	0	0	2	1,415.75
									EDAPIGPHLQSMPSEQIR	95.0%	49.7	22.2	0	4	0	2	2,020.98
									EQPEKEPELQQYVPQLQNNTILR	95.0%	55.4	20.8	0	4	0	2	2,794.44
									FNVLQYVVPEVK	95.0%	56.1	19.0	7	0	0	2	1,434.80
									IGLINDMVR	95.0%	47.5	23.8	4	0	0	2	1,046.57
									LESLNIQR	95.0%	51.6	23.0	4	0	0	2	972.55
									LLDMDGIIVEK	95.0%	55.3	22.4	4	0	0	2	1,261.67
									LLQQVSQIYQSIEFSR	95.0%	84.3	20.8	4	0	0	2	1,939.03
									LTSLVPFVDAFQLER	95.0%	33.7	20.3	2	0	0	2	1,734.94
									NQLTAMSSVLAK	95.0%	50.0	22.7	4	0	0	2	1,278.67
									TLSFGSDLNYATR	95.0%	80.4	22.6	4	0	0	2	1,444.71
									VLLATLSIPITPER	95.0%	47.9	12.3	5	0	0	2	1,522.92
Crk-like protein	CRKL_HUMAN	CRKL	33,759	100.00%	5	7	13	23.80%	IGDQEFDHLPALLEFYK	95.0%	42.8	22.8	1	1	0	2	2,035.02
									IHYLDTTTLIEPAPR	95.0%	72.4	20.7	2	1	0	2	1,739.93
									TALALEVGDIVK	95.0%	77.9	19.7	4	0	0	2	1,228.72
									TLYDFPGNDAEDLPFK	95.0%	74.4	22.1	3	0	0	2	1,841.86
									VSHYIINSLPNR	95.0%	60.2	21.1	1	0	0	2	1,412.77
Calpain-2 catalytic subunit	CAN2_HUMAN	CAPN2	79,995	100.00%	8	8	15	15.00%	DREAAEGLGSHER	95.0%	32.4	21.5	0	2	0	2	1,426.67
									FADDQLIIDFDNFVR	95.0%	79.4	22.8	2	0	0	2	1,827.89
									IMVDMLDSDGSGK	95.0%	72.4	19.2	4	0	0	2	1,399.61
									MGEDMHTIGFGIYEVPEELSGQTNIHLSK	95.0%	22.2	19.8	0	0	1	2	3,264.52
									NFFLTNR	95.0%	31.0	21.8	1	0	0	2	911.47
									SDTFINLR	95.0%	58.3	22.7	2	0	0	2	965.51
									SGTMNSYEMR	95.0%	37.4	11.5	1	0	0	2	1,207.47
									YLNQDYEALR	95.0%	37.5	21.4	2	0	0	2	1,284.62
Eukaryotic initiation factor 4A-III	IF4A3_HUMAN	EIF4A3	46,854	100.00%	4	4	11	18.00%	EANFTVSSMHGDMPQK	95.0%	35.1	17.9	1	0	0	2	1,810.77
									GIYAYGFEKPSAIQQR	95.0%	93.3	21.2	4	4	0	2	1,827.94
									GRDVIAQSQSGTGK	95.0%	76.8	23.3	2	0	0	2	1,403.72
									KLDYGQHVVAGTPGR	95.0%	42.8	21.9	0	4	0	2	1,597.85
									MLVLDEADEMLNK	95.0%	68.0	22.1	4	0	0	2	1,552.72
Probable methylthioribulose-1-phosphate dehydratase	MTNB_HUMAN	APIP	27,107	100.00%	5	5	16	31.80%	AAVMATLLFPGR	95.0%	47.5	21.3	4	0	0	2	1,262.69
									HGDEIYIAPSGVQK	95.0%	84.9	22.6	2	0	0	2	1,513.77
									KVGLDPSQLPVGENGIV	95.0%	30.5	19.6	1	0	0	2	1,721.94
									MAHAMNEYPDSCAVLVR	95.0%	27.2	19.0	0	1	0	2	1,995.87
									YDDMLVVPIIENTPEEK	95.0%	50.7	22.1	8	0	0	2	2,020.98
T-complex protein 1 subunit alpha	TCPA_HUMAN	TCP1	60,327	100.00%	16	19	71	37.40%	AFHNEAQVNPER	95.0%	62.3	21.5	4	2	0	2	1,411.67
									AFHNEAQVNPERK	95.0%	33.3	21.8	0	2	3	2	1,539.77
									DDKHGSYEDAVHSGALND	95.0%	63.2	17.2	1	4	0	2	1,929.82
									EQLAIAEFAR	95.0%	61.6	23.5	7	0	0	2	1,147.61
									EVGDGTTSVVIIAAELLK	95.0%	32.0	18.9	1	0	0	2	1,815.01
									FATEAAITILR	95.0%	68.2	18.5	4	0	0	2	1,205.69
									LGVQVVITDPEKLDQIR	95.0%	31.7	15.6	0	2	0	2	1,923.09
									LLEVEHPAAK	95.0%	57.2	21.1	2	0	0	2	1,106.62
									MLVDDIGDVTITNDGATILK	95.0%	187.0	21.9	2	0	0	2	2,120.08

									QAGVFEPTIVK	95.0%	34.5	21.8	1	0	0	2	1,188.66
									SLLVIPNTLAVNAAQDSTDLVAK	95.0%	98.2	15.4	23	0	0	2	2,353.30
									SQNVMAAASIANIVK	95.0%	78.1	22.6	3	0	0	2	1,532.81
									SSLGPVGLDK	95.0%	50.2	21.5	2	0	0	2	972.54
									STGETIR	95.0%	33.8	22.5	1	0	0	2	763.40
									YINENLIVNTDELGR	95.0%	97.0	22.5	4	0	0	2	1,762.90
									YPVNSVNILK	95.0%	32.0	20.6	3	0	0	2	1,146.65
Serpin B9	SPB9_HUMAN	SERPINB9	42,386	100.00%	13	16	110	43.10%	ADLSAMSAER	95.0%	87.8	20.7	13	0	0	2	1,066.48
									AFQSLLTEVNK	95.0%	86.3	21.1	20	0	0	2	1,249.68
									AGTQYLLR	95.0%	58.5	21.1	4	0	0	2	921.52
									AQLLELPYAR	95.0%	59.0	21.9	23	0	0	2	1,173.66
									ESCLQFYHAELK	95.0%	52.3	22.0	3	0	0	2	1,524.72
									GNTATQMAQALSLNTEEDIHR	95.0%	52.3	20.9	0	4	0	2	2,316.09
									HLGIVDAFQQGK	95.0%	62.7	21.4	3	6	0	2	1,312.70
									IEELLPGSSIDAETR	95.0%	93.6	23.1	6	0	0	2	1,629.83
									INQEEQRPVQMMYQEATFK	95.0%	52.5	20.7	1	7	0	2	2,402.11
									LAHVGEVR	95.0%	56.3	19.0	7	0	0	2	880.50
									LQEDYDMESVLR	95.0%	55.3	20.6	4	0	0	2	1,513.68
									LVLVNAIYFK	95.0%	71.6	14.0	11	0	0	2	1,179.71
									STEVEVLLPK	95.0%	58.2	21.8	5	0	0	2	1,114.64
									TEGKIEELLPGSSIDAETR	95.0%	76.4	22.3	2	2	0	2	2,045.04
Integrin alpha-3	ITA3_HUMAN	ITGA3	118,740	100.00%	7	7	15	8.44%	AAFVSEQQQK	95.0%	39.5	22.6	3	0	0	2	1,135.57
									ARPVINIVHK	95.0%	31.6	7.8	0	1	0	2	1,146.71
									EAGNPGSLFGYSVALHR	95.0%	81.7	22.7	2	0	0	2	1,774.89
									FAGSESAVFHGFFSMPEMR	95.0%	38.6	18.3	0	2	0	2	2,165.94
									GNSYMIQR	95.0%	38.8	20.0	2	0	0	2	984.46
									LELLLMDNLR	95.0%	49.6	22.2	2	0	0	2	1,245.69
									LQSFFGGTVMGESGMK	95.0%	102.0	22.6	3	0	0	2	1,707.77
Hydroxyacylglutathione hydrolase, mitochondrial	GLO2_HUMAN	HAGH	33,788	99.50%	2	3	7	7.79%	ALLEVLGR	95.0%	32.5	17.1	1	0	0	2	870.54
									TVQQHAGETDPVTTMR	95.0%	62.1	21.7	3	3	0	2	1,786.84
Major vault protein	MVP_HUMAN	MVP	99,308	100.00%	23	25	60	33.70%	ALQPLEEGEDEEK	95.0%	58.8	21.1	2	0	0	2	1,486.69
									AQALAIETEAELQR	95.0%	59.8	21.4	1	0	0	2	1,542.81
									AQLELEVSK	95.0%	51.2	22.3	2	0	0	2	1,016.56
									AQQLAEVEVK	95.0%	59.7	23.1	2	0	0	2	1,114.61
									AQQLAEVEVKK	95.0%	34.6	20.9	2	0	0	2	1,242.71
									DITPLQVVLPNTALHLK	95.0%	50.6	13.6	2	1	0	2	1,872.10
									DLAVAGPEMQVK	95.0%	64.2	23.0	2	0	0	2	1,273.65
									DQAVFPQNGLVVSSVDVQSVEPVDQR	95.0%	39.8	21.3	0	2	0	2	2,812.41
									ELLELEALSMAVESTGTAK	95.0%	99.1	22.1	8	2	0	2	2,008.02
									ELPPGVEELLNK	95.0%	64.1	20.4	2	0	0	2	1,337.73
									GAVASVTFDDFHK	95.0%	77.8	23.2	2	0	0	2	1,393.68
									GPLEYVPSAK	94.7%	30.2	22.9	1	0	0	2	1,060.57
									HADLEIR	95.0%	49.7	21.5	4	0	0	2	853.45
									ILDQSEAEK	95.0%	54.3	22.6	5	0	0	2	1,032.52
									IPPYHYIHVLDQNSNVSR	95.0%	46.0	22.0	0	2	0	2	2,152.09
									KELLELEALSMAVESTGTAK	95.0%	31.5	21.0	0	1	0	2	2,136.11
									LAQDPFPLYPGEVLEK	95.0%	67.6	20.4	6	0	0	2	1,815.95
									LLQSLGLK	95.0%	41.1	17.9	1	0	0	2	871.56
									QAIPLDENEGIYVQDVK	95.0%	83.2	22.0	2	0	0	2	1,930.98
									QMTEAIGPSTIR	95.0%	66.8	22.8	2	0	0	2	1,319.66
									TAVFGFETSEAK	95.0%	82.6	22.0	2	0	0	2	1,286.63

									VASGPSPGEGISPQSAQAPQAPGDNHVVPVLR	95.0%	86.5	20.3	0	2	0	2	3,119.59
									VPHNAAVQVYDYR	95.0%	35.2	22.8	0	2	0	2	1,531.77
Ras-related protein Rab-11B	RB11B_HUMAN	RAB11B	24,471	100.00%	4	4	17	25.20%	GAVGALLVYDIAK	95.0%	74.1	17.9	8	0	0	2	1,289.75
									NNLSFIETSALDSTNVEEAFK	95.0%	114.0	21.4	4	0	0	2	2,329.12
									STIGVEFATR	95.0%	54.4	22.0	2	0	0	2	1,080.57
									VVLIGDSGVGK	95.0%	59.7	19.8	3	0	0	2	1,043.61
THO complex subunit 6 homolog	THOC6_HUMAN	THOC6	37,517	99.50%	2	2	5	10.30%	AVPLAVPLGQTEVFQALQR	95.0%	62.2	16.4	3	0	0	2	2,037.15
									TSLEVPEINALLLVPK	95.0%	38.4	12.8	2	0	0	2	1,736.02
Putative thymosin beta-4-like protein 1	TMSL1_HUMAN	TMSL1	5,053	100.00%	2	3	4	54.50%	ETIEQEKQAGES	95.0%	36.5	21.6	1	0	0	2	1,348.62
									TETQEKNPLPSK	95.0%	44.9	22.4	2	1	0	2	1,371.71
10 kDa heat shock protein, mitochondrial	CH10_HUMAN	HSPE1	10,914	100.00%	4	5	8	45.10%	GKGGEIQPVSVK	95.0%	37.2	20.6	1	0	0	2	1,198.68
									KFLPLFDR	95.0%	30.6	19.3	1	0	0	2	1,035.60
									VLQATVVAVGSGSK	95.0%	104.0	19.1	2	0	0	2	1,315.76
									VVLDDKDYFLFR	95.0%	53.3	22.1	2	2	0	2	1,529.80
Alpha-1,6-mannosylglycoprotein 6-beta-N-acetylglucosaminyltransferase A	MGT5A_HUMAN	MGAT5	84,527	100.00%	11	12	34	17.90%	ALAEENR	95.0%	47.2	22.2	3	0	0	2	802.41
									DLQFLLR	95.0%	39.2	20.8	6	0	0	2	904.53
									INVADIINGAQEK	95.0%	90.7	21.5	2	0	0	2	1,384.74
									ISASLAELK	95.0%	52.0	20.3	3	0	0	2	931.55
									NPYEEADHNSLAEIR	95.0%	30.1	20.7	0	1	0	2	1,757.81
									NTDFFIGKPTLR	95.0%	51.8	20.9	4	0	0	2	1,408.76
									NVVDGPYAGVMTAYDLK	95.0%	104.0	21.7	2	0	0	2	1,828.88
									TDFNILYSMMK	95.0%	54.1	20.1	6	0	0	2	1,394.63
									TLAVLLDNILQR	95.0%	70.9	16.1	3	0	0	2	1,368.82
									VLDSFGTEPEFNHANYAQSK	95.0%	51.3	20.5	1	1	0	2	2,254.04
									VLVHLGLLTK	95.0%	34.0	8.5	2	0	0	2	1,092.71
Insulin-degrading enzyme	IDE_HUMAN	IDE	117,956	100.00%	7	7	19	8.05%	AFIPQLLSR	95.0%	34.3	18.7	1	0	0	2	1,044.62
									ESLDDLTNLVVK	95.0%	57.5	22.8	4	0	0	2	1,345.72
									NEFIPTNFEILPLEK	95.0%	51.4	21.6	4	0	0	2	1,803.95
									NLYVTFPIPDLQK	95.0%	66.4	20.8	5	0	0	2	1,547.85
									SIEDMTEEAFQK	95.0%	37.3	19.3	1	0	0	2	1,443.63
									VEAFLITMEK	95.0%	71.4	22.1	2	0	0	2	1,196.62
									VLLISDPTTDK	95.0%	45.2	22.6	2	0	0	2	1,201.67
Vasodilator-stimulated phosphoprotein	VASP_HUMAN	VASP	39,811	100.00%	4	6	30	15.80%	SGGGGLMEEMNAMLAR	95.0%	62.1	16.5	4	0	0	2	1,671.71
									TPKDESANQEEPEAR	95.0%	54.1	20.5	4	8	0	2	1,700.77
									VKEEIIEAFVQELR	95.0%	42.5	19.7	0	2	0	2	1,702.94
									VSKQEEASGGPTAPK	95.0%	73.5	22.2	4	8	0	2	1,485.76
Synaptic vesicle membrane protein VAT-1 homolog	VAT1_HUMAN	VAT1	41,902	100.00%	3	3	5	12.50%	GVDIVMDPLGGSDTAK	95.0%	101.0	22.3	2	0	0	2	1,590.77
									LQSRPAAPPAPGPGQLTLR	95.0%	27.0	16.9	0	1	0	2	1,927.09
									VVTYGMANLLTGPK	95.0%	63.1	21.6	2	0	0	2	1,479.79
Electron transfer flavoprotein subunit alpha, mitochondrial	ETFA_HUMAN	ETFA	35,062	99.50%	2	2	4	8.71%	GLLPEELTPLILATQK	95.0%	67.7	12.8	2	0	0	2	1,736.02
									LEVAPISDIIAIK	95.0%	41.8	12.0	2	0	0	2	1,381.83
UPF0587 protein C1orf123	CA123_HUMAN	C1orf123	18,031	99.50%	2	2	8	25.00%	ATLENITNLRPVGEDFR	95.0%	39.8	21.3	0	4	0	2	1,945.01
									ENSIEILSSTIKPYNAEDNENFK	95.0%	68.9	21.5	0	4	0	2	2,655.28
DNA fragmentation factor subunit alpha	DFFA_HUMAN	DFFA	36,505	99.50%	2	2	3	9.97%	AAFGEEVDAVDTGISR	95.0%	47.8	22.4	1	0	0	2	1,636.78
									ETSSDVALASHILTALR	95.0%	30.5	20.0	0	2	0	2	1,783.96
Podocalyxin-like protein 1	PODXL_HUMAN	PODXL	58,617	100.00%	4	4	11	7.89%	ATFNPAQDK	95.0%	34.2	23.3	1	0	0	2	991.49
									LASVPGSQTVVVK	95.0%	57.0	18.3	6	0	0	2	1,284.75
									LGDQGPPEEAEDR	95.0%	71.4	20.1	3	0	0	2	1,412.63
									LPAKDVYER	95.0%	26.0	23.3	0	1	0	2	1,090.59
Biotinidase	BTD_HUMAN	BTD	61,115	100.00%	7	8	84	20.40%	GDMFLVANLGTK	95.0%	91.1	22.7	13	0	0	2	1,281.65
									HVVYPTAWMNQLPLLAAIEIQK	95.0%	36.3	18.1	0	2	0	2	2,551.37

									LSSGLVTAALYGR	95.0%	100.0	18.9	21	0	0	2	1,307.73
									QEALELMNQNLDIYEQQVMTAAQK	95.0%	73.9	21.7	0	6	0	2	2,840.34
									SHLIIAQVAK	95.0%	54.4	14.8	13	3	0	2	1,079.66
									TSIYPFLDFMPSPQVVR	95.0%	79.6	21.8	20	0	0	2	2,013.02
									VDLITFDTPFAGR	95.0%	80.2	23.3	6	0	0	2	1,451.75
40S ribosomal protein S5	RS5_HUMAN	RPS5	22,859	100.00%	4	4	14	22.10%	QAVDVSPLR	95.0%	68.8	19.2	2	0	0	2	984.55
									TIAECLADELINAAK	95.0%	100.0	23.2	9	0	0	2	1,631.83
									VNQAIWLLCTGAR	95.0%	72.8	21.5	1	0	0	2	1,501.80
									YLPHSAGR	95.0%	37.5	20.7	2	0	0	2	900.47
Pre-mRNA branch site protein p14	PM14_HUMAN	SF3B14	14,568	99.50%	2	2	2	16.00%	ITAEEMYDIFGK	95.0%	41.8	21.4	1	0	0	2	1,416.67
									VGNTPETR	95.0%	45.3	21.8	1	0	0	2	873.44
Importin-4	IPO4_HUMAN	IPO4	118,701	99.50%	2	2	3	2.41%	LLPVLLSTAQEADPEVR	95.0%	52.2	18.5	1	0	0	2	1,851.02
									SLILTALQR	95.0%	39.0	17.0	2	0	0	2	1,014.63
Membrane-bound transcription factor site-1 protease	MBTP1_HUMAN	MBTPS1	117,732	100.00%	9	11	32	11.70%	ALSGTSVASPVVAGAVTLLVSTVQK	95.0%	81.9	12.3	2	8	0	2	2,355.35
									ALSGTSVASPVVAGAVTLLVSTVQKR	95.0%	38.5	10.8	0	3	0	2	2,511.45
									DQGLEVLKQETAVVENVPILGLYQIPAEGGGR	95.0%	30.0	16.1	0	1	0	2	3,392.81
									FPEDGVVITQTFK	95.0%	54.3	21.7	2	0	0	2	1,480.77
									MKPDIVTYGAGVR	95.0%	54.1	22.4	1	0	0	2	1,422.74
									QETAVVENVPILGLYQIPAEGGGR	95.0%	96.8	19.9	2	1	0	2	2,510.33
									QRPPSGAGSVTPER	95.0%	52.1	22.4	0	8	0	2	1,438.74
									RLPGVNMFEQGHGK	95.0%	41.8	22.6	0	2	0	2	1,585.79
									VAVFDTGLSEK	95.0%	54.2	22.4	2	0	0	2	1,165.61
Epididymis-specific alpha-mannosidase	MA2B2_HUMAN	MAN2B2	113,961	100.00%	7	7	19	10.40%	ASALLYAGESMFTR	95.0%	43.8	22.6	2	0	0	2	1,532.74
									AYAANVYTSVVEELAR	95.0%	103.0	22.3	3	0	0	2	1,755.89
									DMYATHLASGMLGMR	95.0%	37.2	18.2	0	2	0	2	1,701.74
									FIAVEQEFFR	95.0%	67.9	21.8	4	0	0	2	1,285.66
									IEQEYQAGPLELNR	95.0%	72.3	22.8	3	0	0	2	1,659.83
									QGPISDNYLFTPGK	95.0%	64.2	22.7	3	0	0	2	1,536.77
									SALALQHRPVVLFGDLAGTAPK	95.0%	62.7	14.5	0	2	0	2	2,261.28
Exportin-1	XPO1_HUMAN	XPO1	123,371	100.00%	21	25	71	27.80%	AIIASNIMYIVGQYPR	95.0%	78.4	20.8	4	0	0	2	1,824.97
									AVGHPFVIQLGR	95.0%	56.4	17.6	3	3	0	2	1,293.74
									EFAGEDTSDLFLEER	95.0%	110.0	20.3	5	0	0	2	1,757.79
									EPEVLSTMAIIVNK	95.0%	76.5	22.3	4	0	0	2	1,559.84
									ETLVYLTHLDYVDTER	95.0%	84.7	22.1	4	2	0	2	1,966.98
									FLVTVIK	95.0%	35.0	10.4	2	0	0	2	819.53
									ISTSLNPGNPVNNQIFLQEYVANLLK	95.0%	41.2	18.6	0	1	0	2	2,886.54
									IYLDMLNVYK	95.0%	51.2	22.2	4	0	0	2	1,287.67
									LDINLLDNVVNCLYHGEGAQQR	95.0%	60.0	21.5	0	2	0	2	2,541.25
									LFVTGLFSLNQDIPAFK	95.0%	49.4	18.9	2	0	0	2	1,910.04
									LISTLIYK	95.0%	31.1	12.8	1	0	0	2	950.59
									LLSEEVFDFSSGQITQVK	95.0%	87.9	21.6	1	2	0	2	2,027.03
									LNMILVQILK	95.0%	46.4	15.1	4	0	0	2	1,200.74
									LVLDSIIWAFK	95.0%	36.4	16.9	1	0	0	2	1,304.76
									MAKPEEVLVVENDQGEVVR	95.0%	50.9	21.8	0	3	0	2	2,157.09
									MAQEVLTHLK	95.0%	48.5	21.7	4	0	0	2	1,185.63
									NVDILKDPETVK	95.0%	48.8	20.9	4	1	0	2	1,370.75
									PAIMTMLADHAAR	95.0%	30.2	21.9	0	1	0	1	1,429.69
									SAFPHLQDAQVK	95.0%	40.4	22.0	3	0	0	2	1,340.70
									YMLLPNQVWDSIIQQATK	95.0%	73.3	21.6	2	0	0	2	2,164.11
									YYGLQILENVIK	95.0%	80.8	18.7	8	0	0	2	1,452.81
Desmoglein-1	DSG1_HUMAN	DSG1	113,731	100.00%	8	8	53	11.70%	ALNSMGQDLERPLELR	94.7%	25.6	21.6	0	1	0	2	1,857.95

									EGGLNMNFMESYFCQK	95.0%	36.3	12.3	1	0	0	2	1,986.80
									EMQDLGGGER	95.0%	45.3	16.5	2	0	0	2	1,107.47
									ISGVGIDQPPYGIFVINQK	95.0%	71.8	18.6	21	0	0	2	2,045.11
									QEPSDSPMFIINR	95.0%	54.7	22.7	3	0	0	2	1,549.73
									TMNNFLDREQYGQYALAVR	95.0%	57.7	21.5	0	1	0	2	2,305.10
									YQGTILSIDDNLQR	95.0%	78.7	22.7	5	0	0	2	1,635.83
									YVMGNNPADLLAVDSR	95.0%	96.2	22.9	19	0	0	2	1,750.84
40S ribosomal protein S19	RS19_HUMAN	RPS19	16,043	100.00%	5	5	9	27.60%	DVNQQEFVR	95.0%	52.6	23.5	3	0	0	2	1,134.55
									IAGQVAAANK	95.0%	54.6	20.0	1	0	0	2	942.54
									IAGQVAAANKK	95.0%	30.7	20.8	0	1	0	2	1,070.63
									LKVPEWVDTVK	95.0%	33.9	20.0	0	2	0	2	1,313.75
									VLQALEGLK	95.0%	60.5	16.2	2	0	0	2	970.59
Poliovirus receptor-related protein 2	PVRL2_HUMAN	PVRL2	57,724	100.00%	3	3	6	7.62%	MGPSFPSPKPGSER	95.0%	36.4	22.5	0	2	0	2	1,489.71
									VEHESFEEPALIPVTLSVR	95.0%	42.9	20.4	0	3	0	2	2,152.13
									VQVLPEVR	95.0%	30.6	16.6	1	0	0	2	939.56
Peroxisomal multifunctional enzyme type 2	DHB4_HUMAN	HSD17B4	79,670	99.50%	2	2	3	3.94%	GALVVVNDLGGDFK	95.0%	65.7	21.8	1	0	0	2	1,403.75
									LGLLGLANSLAIEGR	95.0%	88.5	16.1	2	0	0	2	1,496.88
Bleomycin hydrolase	BLMH_HUMAN	BLMH	52,545	99.50%	2	2	7	5.49%	IGPITPLEFYR	95.0%	56.9	21.0	5	0	0	2	1,305.72
									LYTVEYLSNMVGGR	95.0%	88.2	22.8	2	0	0	2	1,617.79
Protein-glutamine gamma-glutamyltransferase 2	TGM2_HUMAN	TGM2	77,311	100.00%	2	2	11	4.22%	ALLVEPVINSYLLAER	95.0%	87.3	15.6	6	0	0	2	1,800.03
									YLLNLNLEPFSEK	95.0%	79.9	21.6	5	0	0	2	1,579.84
Urokinase-type plasminogen activator	UROK_HUMAN	PLAU	48,490	100.00%	10	12	125	22.00%	DKPGVYTR	95.0%	33.8	23.3	1	0	0	2	935.50
									DYSADTLAHHNDIALLK	95.0%	76.9	22.7	9	28	0	2	1,896.95
									EDYIVYLGR	95.0%	52.4	21.4	2	0	0	2	1,127.57
									FEVENLILHK	95.0%	41.8	20.8	11	0	0	2	1,241.69
									KEDYIVYLGR	95.0%	59.8	22.0	18	2	0	2	1,255.67
									KPSSPPEELK	95.0%	62.6	21.8	23	0	0	2	1,111.60
									LNSNTQGEMKFEVENLILHK	95.0%	42.1	21.4	0	6	0	2	2,360.19
									SDALQLGLGK	95.0%	86.8	22.2	23	0	0	2	1,001.56
									SHTKEENGLAL	95.0%	35.3	22.5	1	0	0	2	1,198.61
									VSHFLPWIR	95.0%	35.2	20.4	0	1	0	2	1,154.65
COP9 signalosome complex subunit 1	CSN1_HUMAN	GPS1	55,520	100.00%	2	2	3	7.13%	EGSQGELTPANSQSR	95.0%	46.1	20.7	2	0	0	2	1,560.73
									MLDEMKDNLLLDMYLAPHVR	95.0%	28.4	21.7	0	0	1	2	2,465.19
Heterogeneous nuclear ribonucleoprotein L	HNRPL_HUMAN	HNRNPL	64,115	100.00%	7	8	29	15.80%	AITHLNNNFMFGQK	95.0%	47.9	22.9	2	0	0	2	1,650.81
									ISRPGDSDDSR	95.0%	39.7	20.6	3	0	0	2	1,204.56
									NPNGPYPYTLK	95.0%	33.6	23.9	2	0	0	2	1,263.64
									SDALETLGFLNHYQMK	95.0%	56.5	23.1	6	3	0	2	1,882.90
									SKPGAAMVEMADGYAVDR	95.0%	50.0	19.4	0	5	0	2	1,899.86
									TPASPVVHIR	95.0%	55.7	20.1	0	5	0	2	1,076.62
									VFNVFCLYGNVEK	95.0%	65.2	23.1	3	0	0	2	1,588.78
U5 small nuclear ribonucleoprotein 200 kDa helicase	U520_HUMAN	SNRNP200	244,496	100.00%	15	15	36	8.94%	DTLGLFLR	95.0%	38.8	19.9	3	0	0	2	934.54
									IVALSSSLSNAK	95.0%	60.0	19.2	3	0	0	2	1,189.68
									KPVIVFVPSR	95.0%	56.1	13.2	4	0	0	2	1,141.71
									LPDMLNAEIVLGNVQNAK	95.0%	93.2	20.6	2	0	0	2	1,955.03
									LTAIDILTTCAADIQR	95.0%	78.0	21.3	2	0	0	2	1,774.94
									LYDLNHNEIGELIR	95.0%	44.6	21.8	0	3	0	2	1,698.88
									MQLSAELQSDTEEILSK	95.0%	113.0	22.6	4	0	0	2	1,937.94
									NALLQLTDSQIADVAR	95.0%	107.0	20.6	5	0	0	2	1,727.93
									NQVLVFVHSR	95.0%	36.7	21.8	1	0	0	2	1,198.67
									SGGPVVVLVQLER	95.0%	48.8	14.1	1	0	0	2	1,352.79
									SLVQEMVGSFGK	95.0%	63.6	22.0	1	0	0	2	1,297.65

									SPTLYGISHDDLK	95.0%	36.8	22.8	1	0	0	2	1,445.73
									VFSLSSEFK	95.0%	42.7	22.1	2	0	0	2	1,043.54
									VVLLTGETSTDLK	95.0%	104.0	20.5	2	0	0	2	1,375.77
									YAQAGFEGFK	95.0%	38.5	21.4	2	0	0	2	1,117.53
Golgi membrane protein 1	GOLM1_HUMAN	GOLM1	45,315	100.00%	23	29	227	60.30%	DLSENNDQR	95.0%	50.2	18.5	11	0	0	2	1,090.48
									DQLVIPDGQEEEQEAAGEGR	95.0%	101.0	20.9	18	0	0	2	2,169.99
									DTINLLDQR	95.0%	65.9	23.8	26	0	0	2	1,087.58
									EQCEERIEEVTK	95.0%	32.0	21.4	1	0	0	2	1,549.72
									EQLDKIQSSHNFQLESVNK	95.0%	39.7	21.7	0	4	0	2	2,244.13
									EQVVEDRPVGGR	95.0%	44.1	22.3	8	0	0	2	1,340.69
									FSYDLSQCINQMK	95.0%	86.8	19.0	4	0	0	2	1,649.73
									GFGGAGELGQTPQVQAALSVSQENPEMEGPER	95.0%	78.1	21.1	0	6	0	2	3,286.53
									GNEAVASR	95.0%	42.0	21.3	3	0	0	2	803.40
									GNVLGNSK	95.0%	37.5	24.3	3	0	0	2	788.43
									IEEVTKK	95.0%	31.3	22.8	2	0	0	2	846.49
									IQSSHNFQLESVNK	95.0%	91.6	22.5	14	17	0	2	1,630.82
									KFSYDLSQCINQMK	95.0%	52.1	23.3	3	2	0	2	1,777.83
									KNEFQGELEK	95.0%	57.5	23.5	12	4	0	2	1,221.61
									LPQEPGR	95.0%	48.7	18.9	5	0	0	2	796.43
									LQAAGLPHTEVPQGK	95.0%	73.5	22.2	4	2	0	2	1,545.84
									LQQDVLQFQK	95.0%	58.8	22.1	16	0	0	2	1,246.68
									LYQDEK	95.0%	30.5	22.0	2	0	0	2	795.39
									NEFQGELEK	95.0%	42.8	22.0	2	0	0	2	1,093.52
									NIDVFNVEDQKR	95.0%	64.8	23.2	15	4	0	2	1,476.74
									QQLQALSEPQPR	95.0%	88.5	22.4	16	0	0	2	1,394.74
									QVEKEETNEIQVVNEEPQR	95.0%	91.6	22.0	6	9	0	2	2,298.12
									VLQDQLK	95.0%	40.0	19.8	8	0	0	2	843.49
Septin-2	SEPT2_HUMAN	SEPT2	41,470	100.00%	7	7	26	30.70%	ASIPFSVVGSNQLIEAK	95.0%	66.9	20.3	4	0	0	2	1,759.96
									ILDEIEEHNIK	95.0%	59.8	22.6	2	0	0	2	1,352.71
									MQAQMQMQMQGGDGDGGALGHHV	95.0%	56.7	12.0	0	2	0	2	2,447.98
									STLINSLFLTDLYPER	95.0%	72.2	20.3	7	0	0	2	1,882.00
									TIISYIDEQFER	95.0%	78.5	22.6	8	0	0	2	1,513.75
									TMLITHMQDLQEVTQDLHYENFR	95.0%	21.9	21.0	0	0	1	2	2,894.35
									VNIVPVIAK	95.0%	34.6	10.4	2	0	0	2	952.62
Peptidyl-prolyl cis-trans isomerase FKBP1A	FKB1A_HUMAN	FKBP1A	11,933	99.50%	2	2	4	25.00%	GVQVETISPGDGR	95.0%	48.5	21.8	2	0	0	1	1,314.67
									GWEEGVAQMSVGQR	95.0%	82.3	21.1	2	0	0	2	1,549.71
Protein disulfide-isomerase A3	PDIA3_HUMAN	PDIA3	56,767	100.00%	28	33	142	57.20%	DGEEAGAYDGPR	95.0%	65.9	14.9	4	0	0	2	1,236.51
									DLLIAYYDVDYEK	95.0%	50.8	22.2	3	0	0	2	1,619.78
									DPNIVIAK	95.0%	30.4	16.4	1	0	0	2	869.51
									EATNPPVIQEEKPK	95.0%	63.0	21.6	8	0	0	2	1,579.83
									ELSDFISYLQR	95.0%	57.8	22.5	19	0	0	2	1,370.70
									FEDKTVAYTEQK	95.0%	64.6	22.0	2	0	0	2	1,458.71
									FIQENIFGICPHMTEDNKDLIQGK	95.0%	36.4	21.6	0	1	0	2	2,863.38
									FISDKDASIVGFFDDSFSEAHSEFLK	95.0%	52.2	20.9	0	1	0	2	2,938.38
									FLQDYFDGNLK	95.0%	73.5	21.8	2	0	0	2	1,359.66
									FLQDYFDGNLKR	95.0%	57.1	22.5	3	1	0	2	1,515.76
									FVMQEEFSR	95.0%	46.9	18.5	16	0	0	2	1,188.54
									GFPTIYFSPANK	95.0%	62.7	22.5	5	0	0	2	1,341.68
									GFPTIYFSPANKK	95.0%	35.2	21.2	2	0	0	2	1,469.78
									IFRDGEEAGAYDGPR	95.0%	59.2	22.5	2	2	0	2	1,652.77
									KTFSHELSDFGLESTAGEIPVVAIR	95.0%	29.1	20.4	0	0	7	2	2,703.40

									LAPEYEAAATR	95.0%	91.7	23.5	12	0	0	2	1,191.60
									LKGIVPLAK	95.0%	31.3	8.5	1	0	0	2	938.64
									LNFAVASR	95.0%	55.8	22.6	2	0	0	2	877.49
									LSKDPNIVIAK	95.0%	56.8	16.6	3	5	0	2	1,197.72
									MDATANDVPSPYEVR	95.0%	93.6	20.8	8	0	0	2	1,680.75
									QAGPASVPLR	95.0%	51.0	18.9	2	0	0	2	995.56
									RLAPEYEAAATR	95.0%	54.0	23.2	2	1	0	2	1,347.70
									SEPIPESNDGPVK	95.0%	50.2	21.9	6	0	0	2	1,368.66
									TADGIVSHLKK	95.0%	46.2	18.5	2	0	0	2	1,168.67
									TFSHELSDFGLESTAGEIPVVAIR	95.0%	69.4	21.6	1	2	0	2	2,575.30
									TVAYTEQK	95.0%	49.6	21.9	2	0	0	2	939.48
									VVVAENFDEIVNNENK	95.0%	96.0	22.6	2	0	0	2	1,832.90
									YGVSGYPTLK	95.0%	53.5	21.9	12	0	0	2	1,084.57
Semaphorin-6B	SEM6B_HUMAN	SEMA6B	95,267	100.00%	5	6	35	7.32%	DYLNHYPVFVGSGPGR	95.0%	59.2	22.3	4	7	0	2	1,777.87
									EIAMEFNYLEK	95.0%	60.9	21.8	10	0	0	2	1,402.66
									LTPAEGADDLNIQR	95.0%	82.1	22.7	9	0	0	2	1,512.77
									TLFIGDRDNLYR	95.0%	36.7	21.7	2	0	0	2	1,482.77
									VELEPPTSTELR	95.0%	39.7	21.8	3	0	0	2	1,370.72
60S ribosomal protein L11	RL11_HUMAN	RPL11	20,235	100.00%	3	3	6	17.40%	ISKEEAMR	95.0%	37.0	22.4	2	0	0	2	979.49
									VLEQLTGQTPVFSK	95.0%	63.2	20.7	3	0	0	2	1,546.85
									YDGIILPGK	95.0%	38.2	21.1	1	0	0	2	975.55
Histidine triad nucleotide-binding protein 1	HINT1_HUMAN	HINT1	13,784	99.50%	2	2	3	27.00%	AQVARPGGDTIFGK	95.0%	53.4	22.5	2	0	0	2	1,416.76
									CLAFHDISPQAPTHFLVIPK	95.0%	32.2	20.4	0	0	1	2	2,291.20
14-3-3 protein gamma	1433G_HUMAN	YWHAG	28,285	100.00%	13	16	75	68.00%	ATVVESSEK	95.0%	58.5	23.5	3	0	0	2	949.48
									AYSEAHEISK	95.0%	64.9	22.7	8	0	0	2	1,134.54
									DNLTLWTSDQQDDDGGEGNN	95.0%	68.2	11.5	1	0	0	2	2,193.88
									DSTLIMQLLR	95.0%	78.2	22.5	32	0	0	2	1,205.66
									EHMQPTHPIR	95.0%	36.8	22.7	1	0	0	2	1,261.61
									LAEQAER	95.0%	56.5	21.6	15	0	0	2	816.42
									LAEQAERYDDMAAAMK	95.0%	60.6	18.6	4	2	0	2	1,844.82
									MKGDYYR	95.0%	35.8	19.2	2	0	0	2	932.43
									MVDREQLVQK	95.0%	35.9	23.1	1	0	0	2	1,261.66
									NCSETQYESK	95.0%	48.9	14.3	2	0	0	2	1,245.51
									NLLSVAYK	95.0%	51.2	19.1	26	0	0	2	907.53
									NLLSVAYKNVVGAR	95.0%	85.3	17.9	2	4	0	2	1,503.86
									NVTELNEPLSNEER	95.0%	101.0	21.5	22	0	0	2	1,643.79
									RATVVESSEK	95.0%	47.7	23.7	4	0	0	2	1,105.59
									TAFDDAIAELDTLNEDSYK	95.0%	109.0	20.4	4	1	0	2	2,130.97
									VISSIEQK	95.0%	73.7	23.0	12	0	0	2	903.52
									YDDMAAAMK	95.0%	54.2	12.3	4	0	0	2	1,047.41
									YLAEVATGEK	95.0%	59.7	22.4	11	0	0	2	1,080.56
									YLAEVATGEKR	95.0%	68.4	22.3	6	1	0	2	1,236.66
Deoxyuridine 5'-triphosphate nucleotidohydrolase, mitochondrial	DUT_HUMAN	DUT	26,689	100.00%	3	4	7	15.50%	ARPAEVGGMQLR	95.0%	26.7	22.2	0	1	0	2	1,300.68
									IFYPEIEEVQALDDTER	95.0%	105.0	21.8	2	0	0	2	2,066.99
									LSEHATAPTR	95.0%	32.6	21.9	1	3	0	2	1,082.56
Regulation of nuclear pre-mRNA domain-containing protein 1B	RPR1B_HUMAN	RPRD1B	36,883	100.00%	4	5	8	18.10%	EFESVLVDAFSHVAR	95.0%	63.1	22.7	2	2	0	2	1,705.85
									IASLPQEVQDVSLLEK	95.0%	81.0	19.4	2	0	0	2	1,768.97
									KLTFLYLANDVIQNSK	95.0%	38.7	18.5	0	1	0	2	1,867.03
									SVYGGEFIQQLK	95.0%	39.6	21.5	1	0	0	2	1,368.72
Sialate O-acetylesterase	SIAE_HUMAN	SIAE	58,297	100.00%	8	8	45	20.10%	DKQTVAYR	95.0%	35.1	21.4	1	0	0	2	980.52
									ELSNTAAYQSVR	95.0%	93.5	22.2	6	0	0	2	1,338.67

									FASYINNDMVLQK	95.0%	62.7	22.3	8	0	0	2	1,558.76
									FFPFGLVQLSSDLSK	95.0%	96.7	21.8	21	0	0	2	1,684.90
									MPNTFMAVAMDLCDRDSPFGSIHPR	95.0%	45.5	18.3	0	0	1	2	2,913.28
									QGQETIMK	95.0%	35.8	23.2	2	0	0	2	950.46
									QGSIPYDSVTGPSK	95.0%	61.1	23.2	3	0	0	2	1,435.71
									SSDDGFPQIR	95.0%	47.8	22.1	3	0	0	2	1,121.52
Far upstream element-binding protein 2	FUBP2_HUMAN	KHSRP	73,129	100.00%	6	6	34	12.50%	IGGGIDVPVPR	95.0%	83.6	18.6	11	0	0	2	1,079.62
									IGQQPQQPGAPPQQDYTK	95.0%	65.2	22.4	4	0	0	2	1,980.98
									IQNDAGVR	95.0%	37.2	21.3	3	0	0	2	872.46
									MMLDDIVSR	95.0%	37.4	21.1	1	0	0	2	1,111.51
									SGPPGPPGGPGMPPGGR	95.0%	59.0	21.6	4	0	0	2	1,487.71
									SVSLTGAPESVQK	95.0%	72.1	23.2	11	0	0	2	1,302.69
									VQISPDSGGLPER	95.0%	45.0	22.1	3	0	0	2	1,354.70
Histone H4	H4_HUMAN	HIST1H4A	11,350	100.00%	7	10	65	52.40%	DAVTYTEHAK	95.0%	40.4	23.0	2	0	0	2	1,134.54
									DNIQGITKPAIR	95.0%	61.6	18.8	9	2	0	2	1,325.75
									ISGLIYEETR	95.0%	66.9	21.7	4	0	0	2	1,180.62
									KTVTAMDVVYALK	95.0%	44.2	19.9	1	1	0	2	1,438.80
									TVTAMDVVYALK	95.0%	58.6	21.2	8	0	0	2	1,310.70
									TVTAMDVVYALKR	95.0%	75.9	20.4	4	2	0	2	1,466.80
									VFLENVIR	95.0%	54.3	20.3	32	0	0	2	989.58
Spectrin beta chain, brain 1	SPTB2_HUMAN	SPTBN1	274,595	100.00%	24	25	47	13.30%	AFEDEMSGR	95.0%	40.2	14.0	1	0	0	2	1,057.43
									DTGNIGQER	95.0%	37.0	20.3	1	0	0	2	989.47
									EAEKLESEHPDQAQAILSR	95.0%	39.5	21.7	0	1	0	2	2,151.07
									EGMQLISEKPETEAVVK	95.0%	26.0	21.9	0	1	0	2	1,903.97
									EIQGHQPR	95.0%	30.5	22.8	1	0	0	2	964.50
									FANSLVGVQQQLQAFNTYR	95.0%	107.0	21.7	1	2	0	2	2,184.12
									FESLEPEMNNQASR	95.0%	54.7	18.5	2	0	0	2	1,667.73
									GNLEVLLFTIQSK	95.0%	39.7	16.7	2	0	0	2	1,461.83
									HLLGVEDLLQK	95.0%	50.4	18.9	2	0	0	2	1,264.73
									IVSSSDVGHDEYSTQSLVK	95.0%	41.3	22.9	0	2	0	2	2,050.99
									LEMNLGLQK	95.0%	31.9	22.8	1	0	0	2	1,061.57
									LFDANK	95.0%	36.7	22.6	1	0	0	2	707.37
									LLDPEDISVDHPDEK	95.0%	61.5	23.2	2	0	0	2	1,721.82
									LLEVLSGER	95.0%	32.8	23.8	1	0	0	2	1,015.58
									LQQFLR	95.0%	30.4	21.2	1	0	0	2	804.47
									LVSQDNFGFDLPAVEAATK	95.0%	87.6	22.1	11	0	0	2	2,022.02
									MLTAQDMSYDEAR	95.0%	91.2	14.8	2	0	0	2	1,562.65
									QALQDTLALYK	95.0%	48.6	21.3	2	0	0	2	1,263.70
									TALPAQSAATLPAR	95.0%	66.9	18.8	2	0	0	2	1,367.76
									TQTAIASEDMPNTLTEAEK	95.0%	114.0	21.3	3	0	0	2	2,065.96
									VAVVNQIAR	95.0%	36.5	15.4	1	0	0	2	969.58
									VDSIDDR	95.0%	33.2	22.3	1	0	0	2	819.39
									VIESTQDLGNDLAGVMALQR	95.0%	36.5	22.0	0	1	0	2	2,146.08
									VLDNAIETEK	95.0%	63.1	24.2	2	0	0	2	1,131.59
									VLVLSQDYGK	95.0%	68.8	22.0	1	0	0	2	1,121.62
Serine-threonine kinase receptor-associated protein	STRAP_HUMAN	STRAP	38,421	100.00%	3	3	9	12.30%	IYDLNKPEAEPK	95.0%	56.5	21.7	2	0	0	2	1,416.74
									TVDFTQDSNYLLTGGQDK	95.0%	107.0	21.8	5	0	0	2	2,001.94
									YDYNSGEELESYK	95.0%	94.0	14.5	2	0	0	2	1,596.67
Protein transport protein Sec23A	SC23A_HUMAN	SEC23A	86,145	100.00%	9	10	29	17.80%	AETEEGPDVLR	95.0%	62.3	21.5	2	0	0	2	1,215.59
									GAIQFVTQYQHSSGQR	95.0%	82.2	22.6	2	2	0	2	1,806.89
									GPQVQQPPPSNR	95.0%	66.8	23.7	6	0	0	2	1,304.67

									HLLQAPVDDAQEILHSR	95.0%	74.1	21.5	0	3	0	2	1,942.01
									IDMNLTDLLGELQR	95.0%	84.7	22.7	4	0	0	2	1,646.84
									IMMFIGGPATQGPGMVVGDELK	95.0%	92.9	21.4	1	0	0	2	2,296.10
									MVVPVAALFTPLK	95.0%	52.9	16.4	5	0	0	2	1,401.82
									SGYQDMPEYENFR	95.0%	72.5	14.0	2	0	0	2	1,651.67
									SSFLQVFNNSPDESSYYR	95.0%	113.0	19.8	2	0	0	2	2,139.96
Transmembrane protein 132A	T132A_HUMAN	TMEM132A	110,089	99.50%	2	2	2	4.50%	AEELVNTAPLTGVPQHVPVR	95.0%	27.5	18.0	0	1	0	2	2,127.16
									SPLSDSILGEQALAVTDDKVSVLELR	95.0%	34.9	18.3	0	1	0	2	2,755.47
Interstitial collagenase	MMP1_HUMAN	MMP1	53,990	100.00%	27	34	431	58.20%	ADVDHAIEK	95.0%	71.1	21.0	5	0	0	2	997.50
									AFQLWSNVTPLTFTK	95.0%	98.6	20.1	13	0	0	2	1,752.93
									CGVPDVAQFVLTEGNPR	95.0%	94.2	22.2	2	1	0	2	1,858.91
									DGFFYFFHGTR	95.0%	57.1	21.1	12	7	0	2	1,393.63
									DIYSSFGFPR	95.0%	82.4	21.1	49	0	0	2	1,188.57
									DNSPFDGPGGNLAHAFQPGPGIGGDAHFDEDER	95.0%	48.8	16.5	0	2	0	2	3,393.48
									EYNLHR	95.0%	37.5	21.0	1	0	0	2	831.41
									GEVMFFK	95.0%	32.9	20.9	2	0	0	2	873.42
									GEVMFFKDR	95.0%	35.6	21.2	3	0	0	2	1,144.55
									HIDAALSEENTGK	95.0%	96.3	22.5	51	6	0	2	1,384.67
									IENYTPDLPR	95.0%	56.8	23.0	22	0	0	2	1,217.62
									ILTLQK	95.0%	32.6	18.1	1	0	0	2	715.47
									LKQMQEFFGLK	95.0%	43.6	21.9	9	4	0	2	1,384.73
									LTFDAITTIR	95.0%	95.7	19.7	66	0	0	2	1,150.65
									MIAHDFPGIGHK	95.0%	51.1	22.3	6	5	0	2	1,338.66
									NSGPVVEK	95.0%	41.0	21.0	8	0	0	2	829.44
									QMQEFFGLK	95.0%	52.8	20.8	16	0	0	2	1,143.55
									RNSGPVVEK	95.0%	33.8	21.0	2	0	0	2	985.54
									SMDPGYPK	95.0%	34.8	18.5	4	0	0	2	910.40
									SQNPVQPIGPQTPK	95.0%	70.9	21.9	45	0	0	2	1,490.80
									TYFFVANK	95.0%	52.4	24.1	20	0	0	2	989.51
									VDAVFMK	95.0%	46.1	20.8	3	0	0	2	825.42
									VSEGQADIMISFVR	95.0%	91.9	22.7	31	0	0	2	1,567.78
									VTGKPDAETLK	95.0%	52.7	23.5	21	2	0	2	1,158.64
									WEQTHLTYR	95.0%	53.1	22.7	6	0	0	2	1,233.60
									YDEYKR	95.0%	34.3	19.6	3	0	0	2	873.41
									YWAVQGQNVLHGYPK	95.0%	65.9	22.7	2	1	0	2	1,759.89
Myosin regulatory light chain 12A	ML12A_HUMAN	MYL12A	19,777	100.00%	4	4	6	29.20%	ATSNVFAMFDQSQIQEFK	95.0%	94.8	21.7	2	0	0	2	2,090.99
									FTDEEVDELYR	95.0%	54.3	19.4	2	0	0	2	1,415.63
									GNFNYIEFTR	95.0%	42.1	21.7	1	0	0	2	1,260.60
									LNGTDPEDVIR	95.0%	35.3	22.8	1	0	0	2	1,228.62
ATP-dependent DNA helicase Q1	RECQ1_HUMAN	RECQL	73,441	100.00%	3	3	5	5.70%	FRPLQLETINVTMAGK	95.0%	42.9	19.7	0	1	0	2	1,833.99
									VAGVVAPTLPR	95.0%	35.4	15.2	3	0	0	2	1,079.66
									VKDILQNVFK	95.0%	37.4	16.9	0	1	0	2	1,203.71
Guanine nucleotide-binding protein G(k) subunit alpha	GNAI3_HUMAN	GNAI3	40,515	99.50%	2	2	2	12.10%	EYQLNDSASYYLNDLDR	95.0%	108.0	19.6	1	0	0	2	2,078.93
									ISQSNYIPTQQDVLR	95.0%	65.8	22.5	1	0	0	2	1,761.91
									LLLLGAGESGK	95.0%	61.9	20.1	6	0	0	2	1,057.63
ADP-ribosylation factor 1	ARF1_HUMAN	ARF1	20,680	100.00%	3	4	8	27.60%	ILMVGLDAAGK	95.0%	45.0	23.4	2	0	0	2	1,103.61
									LGEIVTTIPTIGFNVETVEYK	95.0%	62.9	18.5	2	0	0	2	2,323.24
									MLAEDELRDAVLLVFANK	95.0%	62.0	20.6	2	2	0	2	2,063.08
Beta-mannosidase	MANBA_HUMAN	MANBA	100,879	100.00%	10	10	32	14.10%	FQSAVLYAAQQSK	95.0%	84.3	22.3	4	0	0	2	1,440.75
									FSDNGFLMTEK	95.0%	71.7	19.0	2	0	0	2	1,304.58
									GSPGLSFYFK	95.0%	53.4	23.1	3	0	0	2	1,102.56

									ILFNEVTIGETDNMFNR	95.0%	104.0	22.1	4	0	0	2	2,028.97
									LLLQSVVDANMNTLR	95.0%	86.8	21.0	4	0	0	2	1,702.92
									LPQSTDPLR	95.0%	32.6	21.2	3	0	0	2	1,026.56
									LQTQQTYSIELQPGKR	95.0%	31.3	20.6	0	1	0	2	1,890.01
									TVELIEEPIK	95.0%	68.6	20.5	3	0	0	2	1,170.66
									VNLILEGVDTVSK	95.0%	116.0	19.5	4	0	0	2	1,386.78
									YSFDITNVVR	95.0%	67.4	21.4	4	0	0	2	1,213.62
N-alpha-acetyltransferase 10, NatA catalytic subunit	NAA10_HUMAN	NAA10	26,441	99.50%	2	2	3	14.00%	HVVLGAIENKVESK	95.0%	33.7	19.2	0	2	0	2	1,522.86
									MEEDPDDVPHGHITSLAVK	95.0%	22.3	22.0	0	0	1	2	2,105.98
Low-density lipoprotein receptor	LDLR_HUMAN	LDLR	95,357	100.00%	5	5	14	7.33%	AHGVSSYDTVISR	95.0%	45.5	23.2	1	0	0	2	1,391.69
									AVGSIAYLFFTNR	95.0%	62.2	22.3	6	0	0	2	1,458.77
									LAHPFSLAVFEDK	95.0%	29.2	22.2	0	2	0	2	1,473.77
									NVVALDTEVASNR	95.0%	64.4	22.2	4	0	0	2	1,387.72
									SEYTSLIPNLR	95.0%	32.8	22.9	1	0	0	2	1,292.69
Plasminogen activator inhibitor 1 RNA-binding protein	PAIRB_HUMAN	SERBP1	44,948	100.00%	5	5	68	18.90%	EAGGGGVGGPGAK	95.0%	72.8	21.0	3	0	0	2	1,013.50
									FDQLFDDESDPFEVLK	95.0%	95.5	20.6	13	0	0	2	1,943.89
									RPDQQLQGEGK	95.0%	54.7	22.1	35	0	0	2	1,255.64
									SAAQAAAQTNSNAAGK	95.0%	109.0	22.8	16	0	0	2	1,460.71
									TDKSSASAPDVDDPEAFPALA	95.0%	47.0	20.8	1	0	0	2	2,103.97
Endothelial protein C receptor	EPCR_HUMAN	PROCR	26,653	100.00%	2	3	24	10.90%	TLAFPLTIR	95.0%	43.8	12.6	5	0	0	2	1,031.63
									TQSGLQSYLLQFHGLVR	95.0%	70.2	20.4	7	12	0	2	1,947.05
Protein KIAA1967	K1967_HUMAN	KIAA1967	102,885	100.00%	3	3	5	3.79%	FAEFQYLQPGPPR	95.0%	41.8	22.4	2	0	0	2	1,549.78
									ILLTLGIR	95.0%	37.4	9.0	1	0	0	2	898.61
									VLLLSSPGLEELYR	95.0%	45.3	19.0	2	0	0	2	1,588.90
Coiled-coil domain-containing protein 25	CCD25_HUMAN	CCDC25	24,461	100.00%	2	3	3	10.10%	KAQIQEMK	95.0%	33.7	22.9	1	0	0	2	991.52
									TADMDVGQIGFHR	95.0%	39.2	20.6	1	1	0	2	1,462.68
Protein S100-A9	S10A9_HUMAN	S100A9	13,224	100.00%	3	5	27	37.70%	LGHPDTLNQGEFK	95.0%	55.0	22.7	6	1	0	2	1,455.72
									NIETIINTFHQYSVK	95.0%	79.2	21.3	16	0	0	2	1,806.94
									VIEHIMEDLDTNADK	95.0%	74.2	20.8	2	2	0	2	1,758.82
Syndecan-4	SDC4_HUMAN	SDC4	21,624	100.00%	5	7	165	25.30%	AGSGSQVPTEPK	95.0%	66.9	22.3	45	0	0	2	1,157.58
									ETEVIDPQDLLEGR	95.0%	81.9	22.6	61	0	0	2	1,613.80
									ISPVEESEDVSNK	95.0%	90.9	21.8	11	0	0	2	1,432.68
									KLEENEVIPK	95.0%	49.1	21.6	33	1	0	2	1,198.67
									RISPVEESEDVSNK	95.0%	72.3	23.1	4	10	0	2	1,588.78
Protein argonaute-2	AGO2_HUMAN	EIF2C2	97,190	100.00%	2	2	2	3.26%	AQPVIEFVCEVLDFK	95.0%	33.5	23.0	1	0	0	2	1,793.92
									YAQGADSVEPMFR	95.0%	30.9	19.9	1	0	0	2	1,486.66
Pentraxin-related protein PTX3	PTX3_HUMAN	PTX3	41,958	100.00%	6	7	76	16.80%	ALAAVLEELR	95.0%	63.3	20.5	12	0	0	2	1,084.64
									LFIMLENSQMR	95.0%	63.8	21.5	2	0	0	2	1,413.69
									LTSALDELLQATR	95.0%	110.0	20.7	13	0	0	2	1,430.79
									MEGAEAQRPEEAGR	95.0%	41.1	18.4	5	34	0	2	1,546.69
									MLLQATDDVLR	95.0%	70.2	23.5	7	0	0	2	1,290.67
									MLLQATDDVLRGELQR	95.0%	31.2	21.7	0	3	0	2	1,873.98
Protein transport protein Sec24C	SC24C_HUMAN	SEC24C	118,307	100.00%	6	6	19	7.95%	AVITSLLDQIPEMFADTR	95.0%	80.3	21.6	9	0	0	2	2,036.04
									ETETVFVPVIQAGMEALK	95.0%	46.6	21.8	4	0	0	2	1,978.02
									SDVLQPGAEVTTDDR	95.0%	39.6	22.2	1	0	0	2	1,602.76
									SLLDFLPR	95.0%	31.4	21.6	1	0	0	2	960.55
									SPVESTTEPPAVR	95.0%	42.1	22.1	2	0	0	2	1,369.70
									TLFQPQTGAYQTLAK	95.0%	43.3	20.6	2	0	0	2	1,666.88
Transketolase	TKT_HUMAN	TKT	67,861	100.00%	23	31	278	45.60%	AVELAANTK	95.0%	60.3	23.0	2	0	0	2	916.51
									IIALDGDTK	95.0%	49.7	23.1	1	0	0	2	945.53
									ILATPPQEDAPSVDIANIR	95.0%	102.0	20.1	33	9	0	2	2,020.07

									ILTVEDHYYEGGIGEAVSSAVVGEPGITVTHLAVNR	95.0%	19.5	18.9	0	0	2	2	3,752.91
									ISSDLDGHPVPK	95.0%	59.4	22.7	5	9	0	2	1,264.65
									KAYGQALAK	95.0%	58.0	20.5	4	0	0	2	949.55
									KILATPPQEDAPSVDIANIR	95.0%	49.8	17.9	0	2	0	2	2,148.17
									KISSDLDGHPVPK	95.0%	63.0	21.3	2	5	0	2	1,392.75
									KLILDSAR	95.0%	38.2	19.8	4	0	0	2	915.56
									LDNLVAILDINR	95.0%	93.4	17.6	7	1	0	2	1,368.79
									LGQSDPAPLQHQMDIYQK	95.0%	42.2	22.4	5	4	0	2	2,085.01
									LILDSAR	95.0%	36.8	24.2	2	0	0	2	787.47
									MFGIDRDAIAQAVR	95.0%	42.9	22.2	0	12	0	2	1,578.81
									MPSLPSYK	95.0%	39.9	21.8	5	0	0	2	938.47
									NMAEQIIQEIYSQIQSK	95.0%	126.0	21.7	17	48	0	2	2,039.01
									NSTFSEIFKK	95.0%	39.5	22.6	4	0	0	2	1,200.63
									SGKPAELLK	95.0%	50.4	21.3	2	0	0	2	942.56
									SKDDQVTVIGAGVTLHEALAAAELLK	95.0%	32.8	15.9	0	3	0	2	2,649.45
									SVPTSTVFYPSDGVATEK	95.0%	95.9	22.4	40	0	0	2	1,884.92
									TSRPENAIIYNNNEDFQVGQAK	95.0%	78.3	21.6	2	22	0	2	2,508.21
									TVPFCSTFAAFFTR	95.0%	75.2	22.2	7	0	0	2	1,651.79
									VLDPFTIKPLDR	95.0%	58.5	19.2	12	5	0	2	1,413.81
									VLDPFTIKPLDRK	95.0%	46.1	13.4	2	0	0	2	1,541.91
Splicing factor, arginine/serine-rich 9	SFRS9_HUMAN	SFRS9	25,525	99.50%	2	2	3	9.50%	HGLVPFAFVR	95.0%	31.9	21.9	1	0	0	2	1,142.65
									IYVGNLPTDVR	95.0%	41.2	22.0	2	0	0	2	1,246.68
Calretinin	CALB2_HUMAN	CALB2	31,524	100.00%	7	7	24	25.50%	ELENFFQELEK	95.0%	58.2	22.6	2	0	0	2	1,425.69
									EMNIQQLTNYR	95.0%	65.2	22.3	4	0	0	2	1,425.68
									GFLSDLLKK	95.0%	49.4	17.1	4	0	0	2	1,020.61
									LLPVQENFLLK	95.0%	52.7	16.3	4	0	0	2	1,313.78
									LQEYTQTILR	95.0%	55.4	20.8	4	0	0	2	1,264.69
									SDNFGEK	95.0%	44.8	19.4	4	0	0	2	796.35
									SGYIEANELK	95.0%	31.2	22.8	2	0	0	2	1,123.56
Cystathionine beta-synthase	CBS_HUMAN	CBS	60,569	99.90%	2	2	5	3.81%	AAQELQEGQR	95.0%	62.2	21.1	4	0	0	2	1,129.56
									SNDEEAFTFAR	95.0%	68.4	17.7	1	0	0	2	1,286.57
Putative phospholipase B-like 2	PLBL2_HUMAN	PLBD2	65,455	100.00%	8	8	22	16.50%	AFIPGGPSPGSR	95.0%	40.8	21.9	1	0	0	2	1,142.60
									IKPSLGSGSCSALIK	95.0%	34.8	20.0	0	1	0	2	1,517.84
									LASDGATWADIFKR	95.0%	70.0	22.1	1	0	0	2	1,550.80
									LTLLQLK	95.0%	36.7	14.1	4	0	0	2	828.56
									SVLLDVSAGQLLMVDGR	95.0%	32.9	21.4	1	0	0	2	1,788.95
									VLTILEQIPGMVVVADK	95.0%	111.0	15.7	11	0	0	2	1,841.05
									VLTILEQIPGMVVVADKTSELYQK	95.0%	51.7	16.5	0	2	0	2	2,690.47
									VTSMSLAR	95.0%	38.9	23.2	1	0	0	2	880.46
Eukaryotic translation initiation factor 6	IF6_HUMAN	EIF6	26,580	100.00%	5	7	16	31.40%	ETEEILADVLK	95.0%	57.8	22.1	1	0	0	2	1,259.67
									HGLLVPNNTTDQELQHIR	95.0%	58.6	20.5	0	3	2	2	2,085.08
									LNEAQPSTIATSMR	95.0%	47.8	22.7	2	0	0	2	1,534.75
									NSLPDTVQIR	95.0%	36.5	22.4	1	0	0	2	1,142.62
									TSIEDQDELSSLLQVPLVAGTVNR	95.0%	74.9	19.2	3	4	0	2	2,584.35
Cytosolic non-specific dipeptidase	CNDP2_HUMAN	CNDP2	52,862	100.00%	13	14	39	37.10%	EGGSIPVTLTFQEATGK	95.0%	61.6	22.9	4	0	0	2	1,734.89
									GSTDDKGPVAGWINALEAYQK	95.0%	36.1	22.5	0	2	0	2	2,220.09
									LPDGSEIPLPPILLGR	95.0%	49.3	13.4	3	0	0	2	1,686.98
									LVPNMTPEVVGEQVTSYLTK	95.0%	81.5	21.5	6	2	0	2	2,221.14
									MLAAYLYEVSQLKD	95.0%	54.0	22.9	2	0	0	2	1,659.83
									MMEVAAADVK	95.0%	36.5	21.2	2	0	0	2	1,096.50
									NVMLLPVGSADDGAHSQNEK	95.0%	32.1	21.8	0	1	0	2	2,097.99

									QKLPDGSEIPLPPILLGR	95.0%	27.0	13.0	0	1	0	2	1,943.13
									QLGGSVELVDIGK	95.0%	72.8	22.4	4	0	0	2	1,314.73
									SPNEFK	95.0%	47.2	23.4	4	0	0	2	721.35
									TGQEIPVNVR	95.0%	44.1	20.0	4	0	0	2	1,112.61
									YNYIEGTK	95.0%	32.4	21.1	1	0	0	2	987.48
									YPSLSLHGIEGAFSGSGAK	95.0%	36.2	21.8	0	3	0	2	1,877.94
Aspartate aminotransferase, cytoplasmic	AATC_HUMAN	GOT1	46,230	100.00%	13	16	74	37.80%	APPSVFAEVPQAQPVLVFK	95.0%	93.7	18.1	8	2	0	1	2,024.12
									EPESILQVLSQMEK	95.0%	77.7	22.7	9	0	0	2	1,646.83
									HIYLLPSGR	94.9%	30.4	19.7	1	0	0	2	1,055.60
									IANDNSLNHEYLPILGLAEFR	95.0%	75.6	21.1	2	7	0	2	2,399.24
									IGADFLAR	95.0%	58.3	24.4	7	0	0	2	862.48
									INVSGLTTK	95.0%	44.6	20.4	2	0	0	2	932.54
									KVNLGVGAYR	95.0%	43.0	17.9	0	1	0	2	1,076.62
									LALGDDSPALK	95.0%	47.5	20.4	3	0	0	2	1,099.60
									NFGLYNER	95.0%	36.9	21.5	2	0	0	2	1,012.49
									NLDYVATSIHEAVTK	95.0%	96.5	21.2	8	2	0	2	1,660.85
									QVEYLVNEK	95.0%	33.2	23.5	2	0	0	2	1,121.58
									VGGVQSLGGTGALR	95.0%	77.7	22.2	8	0	0	2	1,271.71
									VGNLTVVGKEPESILQVLSQMEK	95.0%	45.7	17.5	0	10	0	2	2,514.35
Ubiquitin-like modifier-activating enzyme 1	UBA1_HUMAN	UBA1	117,832	100.00%	32	39	683	35.80%	AAVATFLQSVQVPEFTPK	95.0%	101.0	19.3	68	13	0	2	1,933.04
									AENYDIPSADR	95.0%	59.7	21.2	17	0	0	2	1,250.57
									AEVSQPR	95.0%	36.6	20.5	6	0	0	2	786.41
									ALPAVQQNNLDEDLIR	95.0%	109.0	22.0	33	0	0	2	1,808.95
									ALPAVQQNNLDEDLIRK	95.0%	103.0	19.2	5	0	0	2	1,937.05
									DEFEGLFK	95.0%	30.9	19.9	1	0	0	2	984.47
									DEFEGLFKQPAENVNQYLTDPK	95.0%	64.0	21.2	0	2	0	2	2,582.24
									ERLDQPMTEIVSR	95.0%	29.0	23.1	0	2	0	2	1,589.80
									FEVQGLQPNGEEMTLK	95.0%	69.3	22.1	4	0	0	2	1,835.89
									GLGVEIAK	95.0%	35.1	20.0	2	0	0	2	786.47
									GNVQVVIPFLTESYSSSQDPPEK	95.0%	95.1	21.8	17	2	0	2	2,521.25
									IHVSDQELQSANASVDDSRLEELK	95.0%	74.1	21.9	0	3	2	2	2,683.32
									IYDDDFFQNLDGVANALDNVDAR	95.0%	96.2	19.7	14	0	0	2	2,600.19
									KLAYVAAGDLAPINAFIGGLAAQEVMK	95.0%	57.9	17.4	0	4	0	2	2,747.48
									KPLLESGTLGTK	95.0%	58.8	19.0	12	0	0	2	1,243.73
									LAGTQPLEVLEAVQR	95.0%	94.9	18.1	50	25	0	2	1,623.91
									LAYVAAGDLAPINAFIGGLAAQEVMK	95.0%	75.0	18.8	3	37	0	2	2,619.39
									LDQPMTEIVSR	95.0%	68.3	23.4	30	0	0	2	1,304.65
									LKSDTAAAAVR	95.0%	70.5	21.7	21	22	0	2	1,102.62
									LQTSSVLVSGLR	95.0%	88.6	17.9	38	0	0	2	1,259.73
									LVVADTR	95.0%	41.0	24.6	2	0	0	2	773.45
									NEEDAAELVALAQAVNAR	95.0%	145.0	21.6	59	41	0	2	1,883.95
									NGSEADIDEGLYSR	95.0%	105.0	19.8	2	0	0	2	1,525.68
									NIILGGVK	95.0%	32.7	16.5	1	0	0	2	813.52
									QFLDYFK	95.0%	46.6	23.1	2	0	0	2	960.48
									QFLFRPWDVTK	95.0%	43.9	21.3	2	0	0	2	1,436.77
									QPAENVNQYLTDPK	95.0%	87.2	22.6	22	0	0	2	1,616.79
									RLQTSSVLVSGLR	95.0%	28.3	14.6	0	1	0	2	1,415.83
									SLVASLAEPDFVVTDFAK	95.0%	88.3	22.0	62	0	0	2	1,909.00
									VGPDTER	95.0%	51.1	20.1	9	0	0	2	773.38
									YDGQVAVFGSDLQEK	95.0%	101.0	21.6	40	0	0	2	1,655.79
									YFLVGAGAIGCELLK	95.0%	92.0	21.1	7	0	0	2	1,610.86

Proteasome subunit beta type-5	PSB5_HUMAN	PSMB5	28,463	100.00%	13	15	91	48.70%	AIYQATYR	95.0%	34.7	20.8	3	0	0	2	985.51
									ATAGAYIASQTVK	95.0%	96.1	21.1	8	0	0	2	1,280.69
									ATAGAYIASQTVKK	95.0%	60.3	19.7	2	0	0	2	1,408.78
									DAYSGGAVNLYHVR	95.0%	88.7	23.0	8	2	0	2	1,521.75
									GPGLYYVDSEGNR	95.0%	71.6	21.4	4	0	0	2	1,426.66
									GYSYDLEVEQAYDLAR	95.0%	93.0	21.4	19	0	0	2	1,891.87
									HGVIVAADSR	95.0%	72.6	20.7	12	0	0	2	1,024.55
									ISGATFSVGSGSVYAYGVMDR	95.0%	76.2	21.5	2	0	0	2	2,140.00
									ISVAAASK	95.0%	33.6	22.4	1	0	0	2	746.44
									LLANMVYQYK	95.0%	73.4	22.5	10	0	0	2	1,258.65
									RAIYQATYR	95.0%	43.8	20.9	6	0	0	2	1,141.61
									RGPGLYYVDSEGNR	95.0%	32.9	22.3	0	4	0	2	1,582.76
									VSSDNVADLHEK	95.0%	85.5	22.1	6	4	0	2	1,313.63
Golgi apparatus protein 1	GSLG1_HUMAN	GLG1	134,536	100.00%	4	4	16	3.73%	IIIQESALDYR	95.0%	49.2	22.1	1	0	0	2	1,320.72
									LLELQYFISR	95.0%	71.6	20.3	11	0	0	2	1,281.72
									MTAIIFSDYR	95.0%	34.8	22.7	1	0	0	2	1,232.60
									VAELSSDDFHLDR	95.0%	39.9	21.0	0	3	0	2	1,503.71
Dynamin-1-like protein	DNM1L_HUMAN	DNM1L	81,861	100.00%	4	4	7	10.50%	IFSPNVVNLTLVDLPGMTK	95.0%	69.8	18.8	2	0	0	2	2,074.13
									LGIIGVVNR	95.0%	55.9	15.3	2	0	0	2	940.59
									LQDVFNTVGADIIQLPQIVVVGTQSSGK	95.0%	57.0	17.2	0	1	0	2	2,926.59
									TLESVDPLGGLNTIDILTAIR	95.0%	109.0	14.8	2	0	0	2	2,211.22
Galectin-7	LEG7_HUMAN	LGALS7	15,057	99.50%	2	2	8	19.90%	GQPFEVLIIASDDGFK	95.0%	101.0	21.7	2	0	0	2	1,735.89
									LDTSEVVFNSK	95.0%	92.4	23.0	6	0	0	2	1,238.63
Coactosin-like protein	COTL1_HUMAN	COTL1	15,927	100.00%	8	9	23	61.30%	AGGANYDAQTE	95.0%	37.0	17.0	2	0	0	2	1,096.45
									ELEEDFIK	95.0%	37.6	22.2	2	0	0	2	1,022.50
									FALITWIGENVSGLQR	95.0%	79.9	20.8	5	2	0	2	1,803.98
									FTTGDAMSK	95.0%	34.8	17.5	1	0	0	2	973.43
									FTTGDAMSKR	95.0%	44.4	21.4	3	0	0	2	1,113.54
									LFAFVR	95.0%	34.9	16.3	6	0	0	2	752.45
									TGTDKTLVK	95.0%	54.6	19.1	1	0	0	2	962.55
									YDGSTIVPGEQGAEYQHFIQQCTDDVR	95.0%	32.0	17.0	0	1	0	2	3,113.39
Aspartate aminotransferase, mitochondrial	AATM_HUMAN	GOT2	47,459	100.00%	14	16	72	35.80%	ASAELALGENSEVLK	95.0%	73.0	22.2	3	0	0	2	1,530.80
									DAGMQLQGYR	95.0%	68.4	21.4	6	0	0	2	1,154.53
									DDNGKPYVLPSVR	95.0%	30.6	22.8	1	0	0	2	1,459.75
									EFSIYMTK	95.0%	37.0	21.4	1	0	0	2	1,034.49
									FVTVQTISGTGALR	95.0%	110.0	20.9	19	0	0	2	1,449.81
									IAAAILNTPDLR	95.0%	75.1	18.0	8	0	0	2	1,267.74
									IAAAILNTPDLRK	95.0%	66.3	15.9	5	4	0	2	1,395.83
									IGASFLQR	95.0%	66.6	21.7	4	0	0	2	891.51
									ILIRPMYSNPPLNGAR	95.0%	35.6	20.3	0	3	0	2	1,827.99
									ISVAGVTSSNVGYLAHAIHQVTK	95.0%	40.6	17.8	0	4	6	2	2,352.27
									MNLGVGAYR	95.0%	41.6	22.1	3	0	0	2	996.49
									MNLGVGAYRDDNGKPYVLPSVR	95.0%	40.9	21.6	0	2	0	2	2,437.23
									NLDKEYLPIGGLAEFCK	95.0%	27.4	21.8	0	1	0	2	1,967.00
									NMGLYGER	95.0%	45.7	20.5	2	0	0	2	955.43
40S ribosomal protein S28	RS28_HUMAN	RPS28	7,823	100.00%	2	2	4	23.20%	EGDVLTLLESER	95.0%	65.4	23.9	2	0	0	2	1,360.70
									GPVREGDVLTLLESER	95.0%	43.0	21.1	0	2	0	2	1,769.94
Nascent polypeptide-associated complex subunit alpha	NACA_HUMAN	NACA	23,365	100.00%	8	8	51	38.10%	ALKNNSNDIVNAIMELTM	95.0%	72.9	21.8	2	0	0	2	2,022.98
									DIELVMSQANVSR	95.0%	77.7	23.2	4	0	0	2	1,461.74
									IEDLSQQAQLAAAEK	95.0%	122.0	22.4	11	0	0	2	1,614.83
									NILFVITKPDVYK	95.0%	77.7	15.8	11	0	0	2	1,549.90

									NNSNDIVNAIMELTM	95.0%	68.9	20.2	5	0	0	2	1,710.77
									QVTGVTR	95.0%	44.8	24.2	2	0	0	2	760.43
									SKNILFVITKPDVYK	95.0%	46.3	13.4	0	2	0	2	1,765.03
									SPASDTYIVFGEAK	95.0%	99.0	22.5	14	0	0	2	1,484.73
Coatomer subunit beta'	COPB2_HUMAN	COPB2	102,471	100.00%	4	4	9	5.41%	AAESLADPTEYENLFPGLK	95.0%	38.1	21.8	4	0	0	2	2,065.01
									DNNQFASASLDR	95.0%	76.9	20.2	1	0	0	2	1,337.61
									LPEAAFLAR	95.0%	45.0	22.5	2	0	0	2	987.56
									VFNYNTLER	95.0%	51.7	21.9	2	0	0	2	1,155.58
G-protein coupled receptor 126	GP126_HUMAN	GPR126	136,681	100.00%	8	8	119	7.62%	CLLNNALPVK	95.0%	37.3	20.2	2	0	0	2	1,141.64
									GDIYNFR	95.0%	31.6	21.7	1	0	0	2	884.43
									ISVVIQNILR	95.0%	89.6	12.3	42	0	0	2	1,154.73
									LLLGSNQNEIVSLK	95.0%	103.0	17.9	35	0	0	2	1,527.87
									NDGIIYR	95.0%	31.6	23.8	1	0	0	2	850.44
									SGYFLSISDSK	95.0%	91.9	23.0	12	0	0	2	1,203.59
									SISIPELSAFTLCFEATK	95.0%	55.8	22.3	1	0	0	2	2,014.02
									VILPQTSDAYQVSVAK	95.0%	77.4	20.4	25	0	0	2	1,718.93
Prefoldin subunit 2	PFD2_HUMAN	PFDN2	16,630	99.50%	2	2	4	16.90%	GAVSAEQVIAGFNR	95.0%	80.6	23.0	2	0	0	2	1,418.74
									IIETLTQQLQAK	95.0%	51.7	18.1	2	0	0	2	1,385.80
26S proteasome non-ATPase regulatory subunit 7	PSD7_HUMAN	PSMD7	37,008	100.00%	6	10	29	27.80%	DIKDTTVGTLSQR	95.0%	81.6	22.1	2	2	0	2	1,433.76
									DLGLPTEAYISVEEVHDDGTPTSK	95.0%	38.8	20.6	1	1	0	2	2,573.23
									ITNQVHGLK	95.0%	36.7	18.1	2	0	0	2	1,009.58
									SVVALHNLINNK	95.0%	66.9	18.6	2	0	0	2	1,321.76
									TNDQMVVVYLASLIR	95.0%	99.8	21.2	8	2	0	2	1,737.92
									VVVHPLVLLSVVDHFNR	95.0%	58.6	14.0	0	6	3	2	1,943.12
AP-3 complex subunit delta-1	AP3D1_HUMAN	AP3D1	130,144	99.50%	2	2	3	3.04%	DVPVAEEVSALFAGELNPVAPK	95.0%	48.5	20.2	1	0	0	2	2,252.18
									LLESGDLSMSSIK	95.0%	54.5	23.0	2	0	0	2	1,395.70
Poly(ADP-ribose) glycohydrolase ARH3	ARHL2_HUMAN	ADPRHL2	38,929	100.00%	4	4	8	15.20%	ALVQSLLAK	95.0%	48.1	15.3	2	0	0	2	942.60
									HVQSLEPDPGTPGSER	95.0%	58.1	23.4	2	0	0	2	1,705.81
									TEALYYTDDTAMAR	95.0%	86.2	19.0	2	0	0	2	1,636.72
									VAGISLAYSSVQDVQK	95.0%	86.9	21.8	2	0	0	2	1,664.89
Methionine adenosyltransferase 2 subunit beta	MAT2B_HUMAN	MAT2B	37,534	100.00%	6	7	21	27.80%	AVLENNLGAAVLR	95.0%	102.0	19.1	4	0	0	2	1,339.77
									IPILYGEVEKLEESAVTVMFDK	95.0%	38.9	20.3	0	2	0	2	2,526.31
									LETLGIGQR	95.0%	68.5	22.0	6	0	0	2	986.56
									LVEEEVNIPNRR	95.0%	35.8	21.0	2	1	0	2	1,467.79
									RPDVVENQPDAASQLNVDASGNLAK	95.0%	72.1	22.0	0	2	0	2	2,608.30
									VLVTGATGLLGR	95.0%	107.0	16.0	4	0	0	2	1,156.71
Programmed cell death protein 10	PDC10_HUMAN	PDCD10	24,685	100.00%	2	2	4	10.80%	LIHQTNLILQTFK	95.0%	38.8	17.3	2	0	0	2	1,568.92
									VNLSAAQTLR	95.0%	57.8	21.0	2	0	0	2	1,072.61
Calcyclin-binding protein	CYBP_HUMAN	CACYBP	26,192	100.00%	9	12	44	50.00%	AELLDNEKPAAVVAPITTGYTVK	95.0%	36.6	17.9	0	2	0	2	2,400.30
									EKPSYDTETDPSEGLMNVLK	95.0%	48.8	21.9	0	2	0	2	2,269.06
									ISNYGWDQSDKFVK	95.0%	26.4	21.8	0	2	0	2	1,686.81
									IYEDGDDDMKR	95.0%	31.4	16.0	1	2	0	2	1,372.57
									KAELLDNEKPAAVVAPITTGYTVK	95.0%	63.5	14.9	0	6	3	2	2,528.40
									SFDLLVK	95.0%	45.4	17.2	7	0	0	2	821.48
									SYSMIVNNLLKPISVEGSSK	95.0%	72.1	20.1	4	10	0	2	2,182.14
									TDTVLILCR	95.0%	76.7	23.0	3	0	0	2	1,090.59
									WDYLTQVEK	95.0%	51.7	22.8	2	0	0	2	1,181.58
Axin interactor, dorsalization-associated protein	AIDA_HUMAN	AIDA	35,007	99.50%	2	2	4	6.86%	LPSEPGMTLLTIR	95.0%	64.1	22.1	2	0	0	2	1,443.79
									VPGTLLPR	95.0%	47.0	14.3	2	0	0	2	852.53
Proteasome subunit alpha type-6	PSA6_HUMAN	PSMA6	27,382	100.00%	9	11	50	44.30%	AINQGGLTSVAVR	95.0%	96.5	20.8	12	0	0	2	1,285.72
									GSSAGFDR	95.0%	50.4	18.3	2	0	0	2	796.36

									HITIFSPEGR	95.0%	48.9	21.5	8	0	0	2	1,156.61
									ILTEAEIDAHLVALAERD	95.0%	92.8	20.1	6	1	0	2	1,979.05
									ITENIGCVMTGMTADSR	95.0%	101.0	17.9	1	0	0	2	1,887.83
									LLDSSTVTHLFK	95.0%	76.1	21.4	7	4	0	2	1,360.75
									LYQVEYAFK	95.0%	60.7	23.4	7	0	0	2	1,160.60
									QTESTSFLEK	95.0%	41.9	22.7	1	0	0	2	1,169.57
									YGYEIPVDMLCK	95.0%	41.5	20.4	1	0	0	2	1,503.69
26S proteasome non-ATPase regulatory subunit 2	PSMD2_HUMAN	PSMD2	100,184	100.00%	16	20	57	27.00%	APVQPQQSPAAAPGGTDEKPSGK	95.0%	67.1	21.6	0	2	0	2	2,218.11
									AVPLALALISVSNPR	95.0%	33.4	11.8	0	2	0	2	1,520.92
									DKAPVQPQQSPAAAPGGTDEKPSGK	95.0%	55.0	21.4	0	2	2	2	2,461.23
									DPNNLFMVR	95.0%	63.3	23.2	4	0	0	2	1,121.54
									EDVLTLLLPVMGDSK	95.0%	76.1	21.4	4	0	0	2	1,645.87
									FAADIISVLAMTMSGER	95.0%	82.2	22.0	3	2	0	2	1,843.89
									FGGSGSQVDSAR	95.0%	70.9	21.6	2	0	0	2	1,167.54
									FSHDADPEVSYNSIFAMGMVGSGTNNAR	95.0%	54.9	16.5	0	2	0	2	3,006.30
									HLAGEVAK	95.0%	44.0	18.9	3	0	0	2	824.46
									LNILDTLSK	95.0%	52.2	19.3	5	0	0	2	1,016.60
									MLVTFDEELRPLPVSVR	95.0%	53.2	19.9	0	2	0	2	2,017.08
									MNLASSFVNGFVNAAFGQDK	95.0%	142.0	21.8	4	4	0	2	2,133.01
									NECDPALALLSDYVLHNSNTMR	95.0%	28.9	20.2	0	1	0	2	2,549.18
									SSTTSMTSVPKPLK	95.0%	42.7	22.3	3	0	0	2	1,479.77
									TITGFQTHTTPVLLAHGER	95.0%	47.9	20.5	0	2	0	2	2,079.10
									VGQAVDVVGQAGKPK	95.0%	85.4	18.1	4	4	0	2	1,452.82
3'(2'),5'-bisphosphate nucleotidase 1	BPNT1_HUMAN	BPNT1	33,375	100.00%	4	5	10	17.90%	AIAGVINQPYYNYEAGPDAVLGR	95.0%	85.7	21.5	2	1	0	2	2,451.23
									IIQLIEGK	95.0%	44.3	14.9	2	0	0	2	913.57
									LTDIHGNVLQYHK	95.0%	28.1	22.3	0	1	0	2	1,537.81
									LVASAYSIAQK	95.0%	78.8	20.1	4	0	0	2	1,150.65
Lactadherin	MFGM_HUMAN	MFGE8	43,105	100.00%	3	3	11	10.10%	EVTGIITQGAR	95.0%	66.3	22.2	3	0	0	2	1,144.63
									NAVHVNLFETPVEAQYVR	95.0%	57.2	21.5	0	4	0	2	2,086.07
									NLFETPILAR	95.0%	49.0	21.9	4	0	0	2	1,173.66
Hsp70-binding protein 1	HPBP1_HUMAN	HSPBP1	39,456	100.00%	4	4	11	12.70%	EQEAGLLQFLR	95.0%	71.7	22.7	2	0	0	2	1,303.70
									LDGFSVLMR	95.0%	54.9	22.3	6	0	0	2	1,053.54
									SAFLLQNLLVGHPEHK	95.0%	29.6	17.9	0	1	0	2	1,802.99
									YLEAGAAGLR	95.0%	45.2	23.0	2	0	0	2	1,020.55
Endothelin-converting enzyme 1	ECE1_HUMAN	ECE1	87,147	100.00%	2	2	3	4.55%	NEIVFPAGILQAPFYTR	95.0%	65.4	19.6	2	0	0	2	1,936.03
									TPESSHEGLITDPHSPSR	95.0%	51.1	21.7	0	1	0	2	1,946.92
Myotrophin	MTPN_HUMAN	MTPN	12,877	99.50%	2	2	5	25.40%	GPDGLTAFEATDNQAIK	95.0%	90.2	22.9	3	0	0	2	1,747.85
									NGDLDEVKDYVAK	95.0%	57.9	22.7	2	0	0	2	1,465.72
Probable ATP-dependent RNA helicase DDX6	DDX6_HUMAN	DDX6	54,401	100.00%	6	6	13	17.80%	DNIQAMVIVPTR	95.0%	38.1	23.1	1	0	0	2	1,372.73
									GVTQYYAYVTER	95.0%	69.9	22.7	3	0	0	2	1,449.70
									QILLYSATFPLSVQK	95.0%	66.7	16.2	1	0	0	2	1,707.97
									SGAYLIPLLER	95.0%	55.3	19.2	4	0	0	2	1,231.71
									SIEEQLGTEIKPIPSNIDK	95.0%	45.4	19.1	0	2	0	2	2,111.12
									SLYVAEYHSEPVEDEKP	95.0%	50.8	21.2	2	0	0	2	1,991.92
DnaJ homolog subfamily A member 1	DNJA1_HUMAN	DNAJA1	44,851	100.00%	3	3	4	12.80%	ITFHGEGDQEPGLEPGDIIIVLDQK	95.0%	46.3	20.6	0	1	0	2	2,720.38
									QISQAYEVLSDAK	95.0%	63.0	22.8	1	0	0	2	1,451.74
									TIVITSHPGQIVK	95.0%	60.0	14.6	2	0	0	2	1,392.82
Ubiquitin thioesterase OTUB1	OTUB1_HUMAN	OTUB1	31,267	100.00%	7	7	18	33.60%	AFGFSHLEALLDDSKELQR	95.0%	63.4	21.4	0	2	0	2	2,176.10
									EYAEDDNIYQQK	95.0%	42.9	18.6	2	0	0	2	1,515.66
									FFEHFIEGGR	95.0%	40.2	22.2	2	0	0	2	1,238.60
									GEGGTTNPHIFPEGSEPK	95.0%	42.2	21.5	1	0	0	2	1,853.87

									IQQEIAVQNPLVSER	95.0%	107.0	20.4	4	0	0	2	1,723.93
									LELSVLYK	95.0%	31.4	14.6	1	0	0	2	964.57
									LLTSGYLQR	95.0%	55.1	19.2	6	0	0	2	1,050.59
60S ribosomal protein L10a	RL10A_HUMAN	RPL10A	24,814	100.00%	7	9	30	24.00%	AGKFPSLLTHNENMVAK	95.0%	38.0	21.9	0	2	1	2	1,872.96
									AVDIPHMDIEALK	95.0%	51.3	22.9	2	0	0	2	1,467.75
									AVDIPHMDIEALKK	95.0%	54.8	20.7	2	0	0	2	1,595.85
									DTLYEAVR	95.0%	52.7	22.5	6	0	0	2	966.49
									FPSLLTHNENMVAK	95.0%	48.1	23.0	2	0	0	2	1,616.81
									KYDAFLASESLIK	95.0%	83.2	21.5	6	7	0	2	1,484.80
									YDAFLASESLIK	95.0%	54.2	21.5	2	0	0	2	1,356.71
Macrophage colony-stimulating factor 1	CSF1_HUMAN	CSF1	60,101	100.00%	4	4	131	10.80%	AFLLVQDIMEDTMR	95.0%	96.0	22.3	44	0	0	2	1,713.82
									DNTPNAIAIVQLQELSLR	95.0%	120.0	18.5	23	0	0	2	1,995.09
									SHSSGSVLPLGELEGR	95.0%	46.9	21.6	0	9	0	2	1,624.83
									TFYETPLQLLEK	95.0%	78.8	21.1	55	0	0	2	1,481.79
Clusterin	CLUS_HUMAN	CLU	52,477	100.00%	16	18	264	35.60%	ASSIIDELFQDR	95.0%	90.5	23.5	62	0	0	2	1,393.70
									ELDESLQVAER	95.0%	65.8	22.5	41	0	0	2	1,288.64
									EPQDTYHYLPFSLPHR	95.0%	62.9	22.0	6	0	0	2	1,999.97
									FMETVAEK	95.0%	58.3	19.0	22	0	0	2	970.46
									IDSLLENDR	95.0%	40.0	22.9	4	0	0	2	1,074.54
									IDSLLENDRQQTHMLDVMQDHFSR	95.0%	20.3	20.1	0	0	1	2	2,960.36
									KTLLSNLEEAK	95.0%	41.8	20.3	0	4	0	2	1,245.71
									KYNELLK	95.0%	33.2	19.1	7	0	0	2	907.53
									LFDSDPITVTVPVEVSR	95.0%	108.0	21.7	50	1	0	2	1,873.99
									QQTHMLDVMQDHFSR	95.0%	32.1	18.4	0	3	0	2	1,904.84
									RPHFFFPK	95.0%	37.8	23.9	0	14	0	2	1,075.58
									SGSGLVGR	95.0%	79.4	23.2	6	0	0	2	732.40
									TLLSNLEEAK	95.0%	68.1	23.1	7	0	0	2	1,117.61
									TLLSNLEEAKK	95.0%	50.6	20.4	5	0	0	2	1,245.71
									VTTVASHTSDSDVPSGVTEVVVK	95.0%	93.0	21.6	8	18	0	2	2,314.18
									YVNKEIQNAVNGVK	95.0%	105.0	21.8	5	0	0	2	1,575.85
ATP-citrate synthase	ACLY_HUMAN	ACLY	120,825	100.00%	28	31	132	31.40%	AFDSGIIPMEFVNK	95.0%	96.9	22.2	7	0	0	2	1,583.78
									AGKDLVSSLTSGLLTIGDR	95.0%	101.0	17.6	3	3	0	2	1,903.05
									DEPSVAAMVYPFTGDHK	95.0%	85.1	21.0	4	0	0	2	1,879.85
									DGVYVLDLAAK	95.0%	63.7	22.5	11	0	0	2	1,163.63
									DLVSSLTSGLLTIGDR	95.0%	81.8	20.7	17	1	0	2	1,646.90
									DYQGPLKEHEVTIFVR	95.0%	35.5	21.6	0	1	0	2	1,931.00
									EAYPEEAYIADLDAK	95.0%	89.0	21.3	9	0	0	2	1,697.79
									FICTTSAIQNR	95.0%	68.4	22.8	2	0	0	2	1,310.65
									GGPNYQEGLR	95.0%	51.7	23.7	2	0	0	2	1,090.53
									GQELIYAGMPITEVFK	95.0%	79.4	21.9	1	0	0	2	1,811.93
									IGNTGGMLDNILASK	95.0%	71.6	22.6	7	0	0	2	1,519.78
									LGLVGVNLTLDGVK	95.0%	86.0	16.0	2	0	0	2	1,397.84
									LGQEATVGK	95.0%	74.8	23.9	6	0	0	2	902.49
									LTLLNPK	95.0%	31.7	15.8	2	0	0	2	798.51
									LYRPGSVAYVSR	95.0%	47.4	20.7	4	2	0	2	1,367.74
									MIVVLGEIGGTEEYK	95.0%	67.4	22.6	1	0	0	2	1,653.84
									QHFPATPLLDYALEVEK	95.0%	42.7	21.6	0	5	0	2	1,971.02
									SAYDSTMETMNYAQIR	95.0%	77.6	16.3	2	0	0	2	1,912.81
									SGGMSNELNNIISR	95.0%	75.5	21.5	4	0	0	2	1,507.72
									SINNPDMR	95.0%	30.9	20.6	1	0	0	2	962.44
									SMGFIGHYLDQK	95.0%	40.0	21.6	2	0	0	2	1,411.67

									TASFSESR	95.0%	41.5	20.2	2	0	0	2	884.41
									TIAIIAEGIPEALTR	95.0%	129.0	17.0	24	0	0	2	1,567.91
									TILSLMTR	95.0%	43.8	21.4	2	0	0	2	950.53
									TTDGVYEGVAIGGDR	95.0%	61.8	22.1	2	0	0	2	1,509.72
									TTDGVYEGVAIGGDRYPGSTFMDHVLR	95.0%	50.5	20.5	0	1	0	2	2,929.38
									WGDIEFPPPFGR	95.0%	42.2	22.5	1	0	0	2	1,417.69
									YQDTPGVK	95.0%	31.4	23.4	1	0	0	2	907.45
Ribose-phosphate pyrophosphokinase 1	PRPS1_HUMAN	PRPS1	34,817	100.00%	3	5	14	13.20%	IQVIDISMILAEAIR	95.0%	93.4	17.5	9	0	0	2	1,700.96
									MVLVGDVKDR	95.0%	41.8	23.4	1	1	0	2	1,147.61
									VYAILTHGIFSGPAISR	95.0%	85.4	20.3	1	2	0	2	1,802.00
Ras GTPase-activating protein-binding protein 1	G3BP1_HUMAN	G3BP1	52,145	100.00%	5	6	33	14.40%	EAGEQGDIEPR	95.0%	39.7	20.8	4	0	0	2	1,200.55
									LNVEEK	95.0%	34.3	24.4	2	0	0	2	731.39
									LPNFGFVVFDDSEPVQK	95.0%	77.1	21.8	12	2	0	2	1,937.97
									QYYTLLNQAPDMLHR	95.0%	26.9	22.6	0	1	0	2	1,878.92
									SSSPAPADIAQTVQEDLR	95.0%	106.0	22.0	12	0	0	2	1,884.93
U1 small nuclear ribonucleoprotein A	SNRPA_HUMAN	SNRPA	31,262	100.00%	4	5	9	21.60%	EVSSATNALR	95.0%	51.5	23.1	3	0	0	2	1,047.54
									GQAFVIFK	95.0%	45.7	20.3	3	0	0	2	909.52
									KAVQGGGATPVVGAVQGPVPGMPPMTQAPR	95.0%	45.9	20.2	0	1	0	2	2,887.49
									SMQGFPFYDKPMR	95.0%	33.0	20.1	1	1	0	2	1,635.73
Dipeptidyl peptidase 1	CATC_HUMAN	CTSC	51,824	100.00%	6	8	30	11.00%	AINAIQK	95.0%	53.0	21.8	4	0	0	2	757.46
									KVGTASENVYYVNTAHLK	95.0%	33.0	21.5	0	1	0	2	1,830.97
									KVVVYLQK	95.0%	37.9	11.1	1	0	0	2	976.62
									MKEDCFR	95.0%	40.8	15.1	1	0	0	2	1,001.42
									NVHGINFVSPVR	95.0%	57.4	20.3	5	7	0	2	1,338.73
									VGTASENVYYVNTAHLK	95.0%	85.1	22.2	4	7	0	2	1,702.88
Dynactin subunit 2	DCTN2_HUMAN	DCTN2	44,214	100.00%	3	3	7	8.98%	LLGPDAAINLTDPDGALAK	95.0%	89.9	20.0	4	0	0	2	1,865.00
									VAELEKR	95.0%	54.8	23.7	2	0	0	2	844.49
									VHQLYETIQR	95.0%	37.6	23.2	1	0	0	2	1,286.69
Transferrin receptor protein 1	TFR1_HUMAN	TFRC	84,856	100.00%	24	28	185	36.20%	AAAEVAGQFVIK	95.0%	60.1	21.5	22	0	0	2	1,203.67
									AFTYINLDK	95.0%	59.0	21.8	4	0	0	2	1,084.57
									AVLGTSNFK	95.0%	60.1	20.6	2	0	0	2	936.52
									DENLALYVENQFR	95.0%	91.7	22.5	4	0	0	2	1,610.78
									DSAQNSVIIVDK	95.0%	84.4	23.4	6	0	0	2	1,288.68
									EAGSQKDENLALYVENQFR	95.0%	79.5	21.6	2	2	0	2	2,211.07
									EMGLSLQWLYSAR	95.0%	33.6	22.7	1	0	0	2	1,569.77
									GFVEPDHYVVVGAQR	95.0%	93.4	22.7	4	8	0	2	1,672.84
									ILNIFGVIK	95.0%	64.4	12.3	16	0	0	2	1,016.65
									LAQMFSDMVLK	95.0%	63.8	21.6	10	0	0	2	1,314.64
									LAVDEEENADNNTK	95.0%	75.1	18.6	1	0	0	2	1,561.70
									LDSTDFTGTIK	95.0%	70.5	22.2	5	0	0	2	1,197.60
									LLNENSYVPR	95.0%	65.9	23.9	2	0	0	2	1,204.63
									LSEKLDSTDFTGTIK	95.0%	64.7	21.5	2	0	0	2	1,654.85
									LTHDVELNLDYER	95.0%	63.6	22.6	2	3	0	2	1,616.79
									LTTDFGNAEK	95.0%	41.2	22.3	2	0	0	2	1,095.53
									LTVSNVLK	95.0%	37.4	18.1	2	0	0	2	873.54
									LVYLVENPGGYVAYSK	95.0%	111.0	22.0	11	0	0	2	1,771.93
									SGVGTALLLK	95.0%	76.3	17.6	10	0	0	2	958.59
									SSGLPNIPVQTISR	95.0%	63.9	19.6	15	0	0	2	1,468.81
									VANAESLNAIGVLIYMDQTK	95.0%	52.8	21.5	1	0	0	2	2,166.11
									VEYHFLSPYVSPK	95.0%	56.9	22.5	2	8	0	2	1,565.80
									VSASPLLYTLIEK	95.0%	111.0	17.0	28	0	0	2	1,433.83

									YNSQLLSFVR	95.0%	63.8	21.7	10	0	0	2	1,226.65
Bullous pemphigoid antigen 1	BPA1_HUMAN	DST	860,658	100.00%	5	5	7	1.10%	FFLGNQFGDSQQLR	95.0%	44.6	22.3	1	0	0	2	1,656.81
									LASMSPIGTDLETVK	95.0%	47.8	22.3	1	0	0	2	1,577.81
									MQSSADLIIQEFMDLR	95.0%	67.4	21.4	1	0	0	2	1,928.91
									TGPQLLELSPGEGFSIQEK	95.0%	69.8	21.9	2	0	0	2	2,030.05
									TLEQALQLAR	95.0%	48.6	22.0	2	0	0	2	1,142.65
									VLQEDILLR	95.0%	35.1	19.5	1	0	0	2	1,098.65
Glucosidase 2 subunit beta	GLU2B_HUMAN	PRKCSH	59,408	100.00%	9	11	115	16.30%	AQQEQELAADAFK	95.0%	93.0	22.9	12	0	0	2	1,448.70
									ERESLQQMAEVTR	95.0%	38.0	23.1	1	0	0	2	1,592.77
									ESLQQMAEVTR	95.0%	54.8	22.5	11	0	0	2	1,307.63
									ETMVTSTTEPSR	95.0%	67.0	21.1	33	0	0	2	1,354.62
									LVSQKPK	95.0%	35.7	18.6	1	0	0	2	799.50
									LWEEQLAAAK	94.8%	30.3	23.7	1	0	0	2	1,158.62
									SLEDQVEMLR	95.0%	68.6	22.4	12	0	0	2	1,235.59
									SLKDMEESIR	95.0%	39.2	22.1	1	3	0	2	1,223.59
									TVKEEAEKPER	95.0%	49.1	23.0	6	34	0	2	1,315.69
Ras-related protein Rab-1A	RAB1A_HUMAN	RAB1A	22,660	100.00%	3	3	5	27.30%	EFADSLGIPFLETSAK	95.0%	32.8	23.1	1	0	0	2	1,724.87
									LQIWDTAGQER	95.0%	55.4	22.2	2	0	0	2	1,316.66
									MGPGATAGGAEK	95.0%	36.3	21.2	2	0	0	2	1,062.49
									NATNVEQSFMTMAAEIK	95.0%	108.0	20.4	2	0	0	2	1,916.87
Plasma serine protease inhibitor	IPSP_HUMAN	SERPINA5	45,685	100.00%	3	3	9	9.36%	AAAATGTIFTFR	95.0%	63.6	21.1	3	0	0	2	1,226.65
									GFQQLLQELNQPR	95.0%	55.5	22.2	4	0	0	2	1,570.83
									MQILEGLGLNLQK	95.0%	88.0	21.1	2	0	0	2	1,472.82
Succinyl-CoA ligase [GDP-forming]	SUCB2_HUMAN	SUCLG2	46,494	99.50%	2	2	4	4.40%	INFDDNAEFR	95.0%	52.9	20.4	2	0	0	2	1,240.56
subunit beta, mitochondrial									SQAADQITK	95.0%	36.5	24.0	2	0	0	2	961.50
Heterogeneous nuclear	HNRPK_HUMAN	HNRNPK	50,961	100.00%	9	11	101	26.30%	ENTQTTIK	95.0%	39.4	23.4	2	0	0	2	934.48
ribonucleoprotein K									GSYGDLGGPIITTQVTIPK	95.0%	101.0	19.4	9	0	0	2	1,917.03
									IDEPLEGSEDR	95.0%	34.1	19.2	1	0	0	2	1,259.58
									IILDLISESPIK	95.0%	90.4	14.9	41	0	0	2	1,340.80
									IITITGTQDQIQNAQYLLQNSVK	95.0%	74.8	17.2	1	8	0	2	2,589.39
									NAGAVIGK	95.0%	40.0	23.3	2	0	0	2	729.43
									RPAEDMEEEQAFKR	95.0%	45.7	22.0	0	2	0	2	1,751.80
									TDYNASVSVPDSSGPER	95.0%	94.8	19.4	26	0	0	2	1,780.80
									VVLIGGKPDR	95.0%	41.8	12.6	5	4	0	2	1,053.64
Dipeptidyl peptidase 3	DPP3_HUMAN	DPP3	82,574	100.00%	12	17	39	25.90%	EVDGEGKPYYEVR	95.0%	59.1	22.8	2	2	0	2	1,540.73
									FPEDGPELEEILTQLATADAR	95.0%	125.0	21.7	2	1	0	2	2,315.14
									GEFEGFVAVVNK	95.0%	81.8	21.4	2	0	0	2	1,295.66
									GPIVESYIGFIESYR	95.0%	92.0	22.5	2	0	0	2	1,729.88
									LAQDFLDSQNLSAYNTR	95.0%	123.0	21.6	4	0	0	2	1,955.95
									LASVLGSEPSLDSEVTSK	95.0%	62.8	22.6	4	0	0	2	1,818.93
									LEGSDVQLLEYEASAAGLIR	95.0%	125.0	22.0	3	2	0	2	2,134.10
									LVASAEQLLK	95.0%	43.9	20.6	2	0	0	2	1,071.64
									LYAYHLSR	95.0%	32.8	22.4	1	0	0	2	1,022.54
									NVSLGNVLAVAYATQR	95.0%	121.0	20.0	2	2	0	2	1,675.91
									VILGSEAAQQHPEEVR	95.0%	86.9	21.7	2	3	0	2	1,762.91
									VLLEAGEGLVTITPTTGSDGRPDAR	95.0%	77.9	19.8	0	3	0	2	2,525.32
Branched-chain-amino-acid	BCAT2_HUMAN	BCAT2	44,270	100.00%	3	4	6	8.67%	LGGNYGPTVLVQQEALK	95.0%	74.7	19.0	2	0	0	2	1,786.97
aminotransferase, mitochondrial									LGGNYGPTVLVQQEALKR	95.0%	81.7	18.8	1	1	0	2	1,943.07
									NLHIPTMENGPELILR	95.0%	35.3	21.2	0	2	0	2	1,862.98
Secretogranin-2	SCG2_HUMAN	SCG2	70,925	100.00%	17	18	126	38.90%	AGTEALPDGLSVEDILNLLGMESAANQK	95.0%	57.1	21.5	0	14	0	2	2,872.43
									ALEYIENLR	95.0%	60.7	22.5	13	0	0	2	1,120.60

									EHLNQGSSQETDKLAPVSK	95.0%	31.9	22.0	0	1	0	2	2,068.03
									ELDLPVDLDDISEADLDHPDLFQNR	95.0%	43.3	21.4	0	1	0	2	2,894.37
									FPSPEMIR	95.0%	30.7	22.5	2	0	0	2	992.49
									IILEALR	95.0%	35.9	15.1	8	0	0	2	827.54
									LFEKPLDSQSIYQLIEISR	95.0%	92.4	18.3	2	17	0	2	2,279.23
									LVNAAGSGR	95.0%	57.6	22.7	16	0	0	2	844.46
									NLQIPPEDLIEMLK	95.0%	39.3	21.5	3	0	0	2	1,668.89
									QAENEPQSAPK	95.0%	47.6	22.1	2	0	0	2	1,198.57
									QMAYENLNDKDQELGEYLAR	95.0%	35.7	21.2	0	1	0	2	2,416.11
									RLVNAAGSGR	95.0%	39.1	21.1	4	0	0	2	1,000.57
									SGQLGIQEEDLR	95.0%	79.3	22.2	18	0	0	2	1,344.68
									TNEIVEEQYTPQSLATLESVFQELGK	95.0%	50.2	20.4	0	3	0	2	2,953.47
									TSYFPNPYNQEK	95.0%	52.7	20.6	6	0	0	2	1,487.68
									VLEYLNQEK	95.0%	56.0	23.3	4	0	0	2	1,135.60
									YPEIINSNQVK	95.0%	56.8	23.1	11	0	0	2	1,304.69
Collagen alpha-1(V) chain	CO5A1_HUMAN	COL5A1	183,545	100.00%	3	3	6	3.16%	FLGSNDEEMSYDNNPYIR	95.0%	44.1	16.0	1	0	0	2	2,179.92
									LLSYVDAEGNPVGVVQMTFLR	95.0%	102.0	20.5	2	0	0	2	2,324.20
									QLYPASAFPEDFSILTTVK	95.0%	74.3	21.1	3	0	0	2	2,127.10
Keratinocyte proline-rich protein	KPRP_HUMAN	KPRP	64,115	100.00%	2	2	17	2.94%	GRPAVCQPQGR	95.0%	38.1	22.7	0	7	0	2	1,225.62
									LQLFPR	95.0%	38.3	23.2	10	0	0	2	773.47
Granulins	GRN_HUMAN	GRN	63,522	100.00%	7	10	75	10.80%	APAHLSLPDPQALK	95.0%	48.5	20.4	2	2	0	2	1,457.81
									APAHLSLPDPQALKR	95.0%	54.6	18.8	3	17	12	2	1,613.91
									ASLSHPR	95.0%	58.8	20.6	9	0	0	2	767.42
									CITPTGTHPLAK	95.0%	34.3	21.6	2	0	0	2	1,295.68
									EVVSAQPATFLAR	95.0%	75.3	22.3	24	0	0	2	1,388.75
									LPAHTVGDVK	95.0%	44.3	20.0	3	0	0	2	1,036.58
									SPHVGVK	95.0%	42.8	16.6	1	0	0	2	723.42
Polyadenylate-binding protein 1	PABP1_HUMAN	PABPC1	70,653	100.00%	15	16	114	28.10%	ALDTMNFDVIK	95.0%	72.7	21.7	15	0	0	2	1,282.64
									EFSPFGTITSAK	95.0%	51.4	22.1	10	0	0	2	1,284.65
									FGPALSVK	95.0%	41.6	21.5	7	0	0	2	818.48
									FSPAGPILSIR	95.0%	61.2	21.0	11	0	0	2	1,157.67
									GFGFVSFER	95.0%	48.9	22.4	12	0	0	2	1,045.51
									ITGMLLEIDNSELLHMLESPESLR	95.0%	76.6	20.6	0	4	0	2	2,772.38
									IVATKPLYVALAQR	95.0%	75.2	10.4	6	12	0	2	1,542.94
									KEFSPFGTITSAK	95.0%	45.0	21.2	3	0	0	2	1,412.74
									LFPLIQAMHPTLAGK	95.0%	51.9	18.5	4	0	0	2	1,652.92
									NLDDGIDDERLR	95.0%	46.2	22.4	1	0	0	2	1,430.69
									QAHLTNQYMQR	95.0%	28.2	21.8	0	2	0	2	1,405.66
									SGVGNIFIK	95.0%	64.0	19.9	10	0	0	2	934.54
									SKVDEAVAVLQAHQAK	95.0%	35.2	19.6	0	2	0	2	1,693.92
									SLGYAYVNFQQPADAER	95.0%	104.0	21.2	11	0	0	2	1,928.91
									YQGVNLYVK	95.0%	39.9	20.9	4	0	0	2	1,083.58
FACT complex subunit SSRP1	SSRP1_HUMAN	SSRP1	81,060	100.00%	4	5	16	7.62%	ASSGLLYPLER	95.0%	32.3	22.7	1	0	0	2	1,205.65
									FDEISFVNFAR	95.0%	78.3	22.6	6	0	0	2	1,344.66
									FYVPPTQEDGVDPVEAFAQNVLSK	95.0%	41.8	21.8	1	4	0	2	2,650.30
									LFDFVNAK	95.0%	41.5	20.9	4	0	0	2	953.51
Protein SET	SET_HUMAN	SET	33,471	100.00%	7	8	91	27.90%	EFHLNESGDPSSK	95.0%	76.6	20.4	27	0	0	2	1,446.65
									IDFYFDENPYFENK	95.0%	69.7	19.2	22	0	0	2	1,840.81
									KPRPPPALGPEETSASAGLPK	95.0%	85.5	19.5	0	2	0	2	2,100.15
									LNEQASEEILK	95.0%	59.5	23.3	4	0	0	2	1,273.66
									LNEQASEEILKVEQK	95.0%	87.2	21.5	3	4	0	2	1,757.93

									LRQPFFQK	95.0%	35.7	20.8	3	0	0	2	1,063.61
									VEVTEFEDIK	95.0%	80.0	23.2	26	0	0	2	1,208.61
Poly(rC)-binding protein 1	PCBP1_HUMAN	PCBP1	37,480	100.00%	3	4	28	12.40%	ESTGAQVQVAGDMLPNSTER	95.0%	97.3	21.7	12	2	0	2	2,105.98
									IITLTGPTNAIFK	95.0%	85.4	16.6	13	0	0	2	1,388.82
									INISEGNCPER	95.0%	42.1	20.3	1	0	0	2	1,288.60
Flavin reductase	BLVRB_HUMAN	BLVRB	22,101	100.00%	5	6	22	42.70%	LPSEGPRPAHVVVGDVLQAADVDK	95.0%	50.4	19.9	0	2	0	2	2,469.31
									LQAVTDDHIR	95.0%	43.8	22.7	4	2	0	2	1,167.61
									NDLSPTTVMSEGAR	95.0%	78.5	20.9	6	0	0	2	1,493.69
									TVAGQDAVIVLLGTR	95.0%	105.0	18.1	6	0	0	2	1,512.88
									YVAVMPPHIGDQPLTGAYTVTLDGR	95.0%	54.3	21.0	0	2	0	2	2,687.35
Core histone macro-H2A.1	H2AY_HUMAN	H2AFY	39,601	100.00%	5	6	18	22.00%	AASADSTTEGTPADGFTVLSTK	95.0%	117.0	22.1	5	0	0	2	2,127.01
									GVTIASGGVLPNIHPELLAK	95.0%	45.4	14.6	0	2	0	2	1,986.14
									NGPLEVAGAAVSAGHGLPAK	95.0%	92.0	20.3	3	2	0	2	1,815.97
									QTAAQLILK	95.0%	39.7	17.8	1	0	0	2	985.60
									SIAFPSIGSGR	95.0%	42.4	23.7	5	0	0	2	1,091.59
Ubiquilin-4	UBQL4_HUMAN	UBQLN4	63,836	100.00%	3	3	5	12.80%	ALSNLESIPGGYNALR	95.0%	43.0	22.1	1	0	0	2	1,674.88
									AQQDQLVLIFAGK	95.0%	41.9	19.9	2	0	0	2	1,430.80
									HMIMANPQMQQLMER	95.0%	34.7	19.3	0	2	0	2	1,921.84
									LQLPVFLQQMQNPESLSILTNPR	95.0%	28.1	17.9	0	1	0	2	2,682.43
									NPAMMQEMMR	95.0%	33.6	10.4	1	0	0	2	1,302.50
HLA class I histocompatibility antigen, A-11 alpha chain	1A11_HUMAN	HLA-A	40,919	100.00%	5	6	63	19.50%	DGEDQTQDTELVETRPAGDGTFQK	95.0%	67.7	20.9	1	22	0	2	2,637.19
									FDSDAASQR	95.0%	66.2	20.5	16	0	0	2	996.44
									FIAVGYVDDTQFVR	95.0%	97.6	22.9	10	0	0	2	1,629.83
									WAAVVVPSGEEQR	95.0%	58.6	23.2	12	0	0	2	1,427.73
									YLENGKETLQR	95.0%	52.7	23.7	2	0	0	2	1,350.70
Group XV phospholipase A2	PAG15_HUMAN	PLA2G15	46,641	100.00%	3	3	7	10.20%	APNENGPYFLALR	95.0%	51.1	23.1	2	0	0	2	1,461.75
									HPPVVLVPGDLGNQLEAK	95.0%	32.2	18.8	1	0	0	2	1,883.04
									TFSLEFLDPSK	95.0%	50.7	21.4	4	0	0	2	1,283.65
CD109 antigen	CD109_HUMAN	CD109	161,674	100.00%	21	25	191	18.50%	ADGNQLTLEER	95.0%	68.2	22.4	10	0	0	2	1,245.61
									ALSEFAALMNTER	95.0%	81.8	21.8	12	0	0	2	1,468.71
									DYIDGVYDNAEYAER	95.0%	81.1	18.8	4	0	0	2	1,792.77
									ELSYMVVSR	95.0%	32.5	22.0	1	0	0	2	1,099.55
									IEFPILEDSSELQLK	95.0%	93.6	21.6	28	1	0	2	1,760.93
									IPVQLVFK	95.0%	56.4	17.0	6	0	0	2	943.60
									ISVFIQTDK	95.0%	56.3	21.5	8	0	0	2	1,050.58
									ISVTQPDSIVGIVAVDK	95.0%	106.0	18.3	26	0	0	2	1,740.97
									IVTLFSDFKPYK	95.0%	69.7	20.9	7	0	0	2	1,457.80
									LKELSYMVVSR	95.0%	41.3	21.2	4	0	0	2	1,340.72
									NNVVITVTQR	95.0%	47.3	20.7	4	0	0	2	1,143.65
									NSLGGFASTQDTTVALK	95.0%	89.3	21.8	6	0	0	2	1,709.87
									SPVTLTAYIVTSLLGYR	95.0%	99.8	17.9	2	3	0	2	1,854.04
									SSMAVHSLFK	95.0%	38.6	23.3	1	0	0	2	1,122.56
									SYSQSILLDLTDNR	95.0%	109.0	22.1	15	0	0	2	1,624.82
									TLSFSFPPNTVTGSER	95.0%	64.3	21.9	13	0	0	2	1,739.86
									TLTLPSLPLNSADEIYELR	95.0%	93.9	19.6	16	0	0	2	2,145.14
									TNIQVTVTGPSSPSPVK	95.0%	105.0	20.3	6	0	0	2	1,711.92
									VGSPFELVVSGNK	95.0%	91.9	22.7	3	0	0	2	1,332.72
									VGSPFELVVSGNKR	95.0%	87.5	20.1	7	2	0	2	1,488.82
									VQITAIGDVLGPSINGLASLIR	95.0%	94.5	11.8	3	3	0	2	2,207.28
Serine/threonine-protein phosphatase 2A activator	PTPA_HUMAN	PPP2R4	40,650	100.00%	9	10	36	34.10%	FGSLLPIHPVTSG	95.0%	63.0	20.5	7	0	0	2	1,324.73
									FPVIQHFK	95.0%	37.4	23.0	2	0	0	2	1,015.57

									KEIHTVPDMGK	95.0%	48.1	22.1	5	4	0	2	1,270.65
									KLTFEYR	95.0%	31.4	20.2	2	0	0	2	956.52
									LDEEAENLVATVVPTHLAAAVPEVAVYLK	95.0%	63.5	16.7	0	4	0	2	3,061.65
									LVALLNTLDR	95.0%	75.2	19.1	5	0	0	2	1,127.68
									QPPPDSSEEAPPATQNFIIPK	95.0%	38.8	21.3	1	0	0	2	2,263.13
									VDDQIAIVFK	95.0%	56.2	21.1	4	0	0	2	1,147.64
									WIDETPPVDQPSR	95.0%	58.6	23.2	2	0	0	2	1,539.74
Heat shock cognate 71 kDa protein	HSP7C_HUMAN	HSPA8	70,882	100.00%	40	58	1094	64.10%	AMTKDNNLLGK	95.0%	27.6	23.6	0	1	0	2	1,220.63
									ARFEELNADLFR	95.0%	62.1	22.1	10	37	0	2	1,480.75
									ATVEDEK	95.0%	32.0	23.2	1	0	0	2	791.38
									CNEIINWLDK	95.0%	54.0	24.2	2	0	0	2	1,304.63
									DAGTIAGLNVLR	95.0%	110.0	22.0	101	0	0	2	1,199.68
									DNNLLGK	95.0%	43.6	23.7	2	0	0	2	773.42
									EIAEAYLGK	95.0%	58.3	21.7	4	0	0	2	993.53
									FDDAVVQSDMK	95.0%	78.5	19.8	8	0	0	2	1,270.56
									FEELNADLFR	95.0%	65.6	22.7	35	0	0	2	1,253.62
									GPAVGIDLGTTYSCVGVFQHGK	95.0%	55.2	21.6	0	52	0	2	2,263.12
									GTLDPVEK	95.0%	36.0	21.5	4	0	0	2	858.46
									HWPFMVVNDAGRPK	95.0%	44.7	22.6	0	6	0	2	1,669.83
									IINEPTAAAIAYGLDK	95.0%	110.0	21.5	26	2	0	2	1,659.90
									IINEPTAAAIAYGLDKK	95.0%	120.0	16.6	21	25	0	2	1,787.99
									ITITNDKGR	95.0%	44.0	22.1	2	0	0	2	1,017.57
									LDKSQIHDIVLVGGSTR	95.0%	79.6	19.3	4	16	2	2	1,838.01
									LLQDFFNGK	95.0%	55.9	22.1	22	0	0	2	1,081.57
									LSKEDIER	95.0%	62.3	23.4	30	0	0	2	989.53
									LYQSAGGMPGGMPGGFPGGGAPPSGGASSGPTIEEVD	95.0%	55.2	18.1	0	1	0	2	3,378.49
									MKEIAEAYLGK	95.0%	79.0	22.1	20	6	0	2	1,268.66
									MVNHFIAEFK	95.0%	41.4	22.9	12	2	0	2	1,251.62
									MVNHFIAEFKR	95.0%	50.5	22.5	2	2	0	2	1,407.72
									MVQEAEKYK	95.0%	36.9	22.3	2	0	0	2	1,141.56
									NQTAEKEEFEHQQK	95.0%	67.0	21.1	10	18	0	2	1,745.81
									NQVAMNPTNTVFDAK	95.0%	111.0	22.6	59	0	0	2	1,665.79
									NQVAMNPTNTVFDAKR	95.0%	75.3	22.5	2	1	0	2	1,821.89
									NSLESYAFNMK	95.0%	81.8	19.5	56	0	0	2	1,319.59
									NTTIPTK	95.0%	41.5	24.6	2	0	0	2	774.44
									QTQTFTTYSDNQPGVLIQVYEGER	95.0%	77.3	21.4	8	24	0	2	2,774.33
									RFDDAVVQSDMK	95.0%	66.1	21.4	17	3	0	2	1,426.66
									RNTTIPTK	95.0%	33.7	22.1	1	0	0	2	930.54
									SFYPEEVSSMVLTK	95.0%	98.5	22.3	56	5	0	2	1,632.78
									SINPDEAVAYGAAVQAAILSGDK	95.0%	127.0	21.5	53	6	0	2	2,260.15
									SQIHDIVLVGGSTR	95.0%	97.5	20.9	31	8	0	2	1,481.81
									STAGDTHLGGEDFDNR	95.0%	77.7	17.6	46	34	0	2	1,691.73
									TTPSYVAFTDTER	95.0%	102.0	21.7	73	0	0	2	1,487.70
									TVTNAVVTVPAYFNDSQR	95.0%	78.5	21.7	40	15	0	2	1,982.00
									VEIIANDQGNR	95.0%	74.6	22.6	42	0	0	2	1,228.63
									VQVEYK	95.0%	34.6	22.1	1	0	0	2	765.41
									VQVEYKGETK	95.0%	55.8	22.5	21	2	0	2	1,180.62
Methylthioribose-1-phosphate isomerase	MTNA_HUMAN	MRI1	39,132	100.00%	2	2	3	7.05%	EAEREGATEEAVR	95.0%	28.0	21.7	0	1	0	2	1,446.68
									GSLQILDQLLLPK	95.0%	68.2	13.8	2	0	0	2	1,437.87
Chromobox protein homolog 3	CBX3_HUMAN	CBX3	20,794	100.00%	4	5	14	22.40%	IIGATDSSGELMFLMK	95.0%	99.3	22.2	7	0	0	2	1,744.85
									KVEEAEPEEFVVEK	95.0%	63.9	22.9	2	1	0	2	1,661.83

									SLSDSESDDSK	95.0%	61.1	16.3	2	0	0	2	1,169.48
									VEEAEPEEFVVEK	95.0%	50.8	22.3	2	0	0	2	1,533.73
Elongation factor 1-gamma	EF1G_HUMAN	EEF1G	50,101	100.00%	12	14	110	22.70%	AKDPFAHLPK	95.0%	40.2	21.2	1	3	0	2	1,123.63
									ALIAAQYSGAQVR	95.0%	93.2	21.4	19	0	0	2	1,347.74
									EYFSWEGAFQHVGK	95.0%	52.5	21.5	1	0	0	2	1,684.78
									FAETQPK	95.0%	36.6	23.8	8	0	0	2	820.42
									ILGLLDAYLK	95.0%	68.1	13.0	44	0	0	2	1,118.68
									KFAETQPK	95.0%	32.1	22.3	2	0	0	2	948.52
									KLDPGSEETQTLVR	95.0%	86.2	22.2	5	6	0	2	1,572.82
									LDPGSEETQTLVR	95.0%	74.4	22.3	2	0	0	2	1,444.73
									MAQFDAK	95.0%	34.9	18.9	3	0	0	2	826.38
									STFVLDEFKR	95.0%	51.3	21.3	7	0	0	2	1,241.65
									TFLVGER	95.0%	42.4	22.7	4	0	0	2	821.45
									TPEFLR	95.0%	34.4	23.4	5	0	0	2	762.42
Purine nucleoside phosphorylase	PNPH_HUMAN	PNP	32,100	100.00%	14	19	82	58.80%	ANHEEVLAAGK	95.0%	74.8	23.4	5	3	0	2	1,138.59
									DHINLPGFSGQNPLR	95.0%	57.1	22.6	2	2	0	2	1,664.85
									DHINLPGFSGQNPLRGPNDER	95.0%	32.7	20.9	0	0	2	2	2,333.14
									FEVGDIMLIR	95.0%	64.0	22.8	8	0	0	2	1,208.64
									FGDRFPAMSDAYDR	95.0%	67.2	19.4	3	4	0	2	1,647.72
									LEQFVSILMASIPLPDK	95.0%	58.0	18.6	4	2	0	2	1,901.05
									LEQFVSILMASIPLPDKAS	95.0%	73.1	20.2	9	2	0	2	2,075.11
									LGADAVGMSTVPEVIVAR	95.0%	84.9	21.1	9	0	0	2	1,800.95
									LTQAQIFDYGEIPNFPR	95.0%	84.7	21.7	4	0	0	2	2,009.01
									LVFGFLNGR	95.0%	56.3	21.3	4	0	0	2	1,022.58
									STVPGHAGR	95.0%	31.7	22.1	1	0	0	2	881.46
									VFGFSLITNK	95.0%	74.7	20.0	11	0	0	2	1,125.63
									VFHLLGVDTLVVTNAAGGLNPK	95.0%	27.9	15.4	0	1	0	2	2,235.25
									VIMDYESLEK	95.0%	65.4	22.2	6	0	0	2	1,242.59
Vimentin	VIME_HUMAN	VIM	53,635	100.00%	52	71	1607	83.00%	DGQVINETSQHHDDLE	95.0%	72.6	19.6	38	0	0	2	1,836.80
									DNLAEDIMR	95.0%	50.5	21.1	3	0	0	2	1,076.50
									EEAENTLQSFR	95.0%	66.7	21.5	34	0	0	2	1,323.62
									EEAENTLQSFRQDVDNASLAR	95.0%	59.1	20.5	0	4	0	2	2,393.13
									EKLQEEMLQR	95.0%	54.2	22.9	20	0	0	2	1,319.66
									EMEENFAVEAANYQDTIGR	95.0%	123.0	18.3	70	18	0	2	2,202.96
									ETNLDSLPLVDTHSK	95.0%	78.7	21.4	31	6	0	2	1,668.84
									ETNLDSLPLVDTHSKR	95.0%	71.3	21.9	9	3	2	2	1,824.95
									EYQDLLNVK	95.0%	68.1	23.3	55	0	0	2	1,121.58
									FADLSEAANR	95.0%	85.1	23.5	50	0	0	2	1,093.53
									FADLSEAANRNNDALR	95.0%	41.3	22.1	2	8	0	2	1,776.86
									FANYIDK	95.0%	50.7	21.3	21	0	0	2	870.44
									FANYIDKVR	95.0%	76.8	22.5	19	14	0	2	1,125.61
									FLEQQNK	95.0%	48.0	24.2	53	0	0	2	906.47
									GTNESLER	95.0%	57.8	22.5	25	0	0	2	905.43
									HLREYQDLLNVK	95.0%	27.5	21.5	0	1	0	2	1,527.83
									ILLAELEQLK	95.0%	73.3	16.6	111	0	0	2	1,169.71
									ILLAELEQLKGQGK	95.0%	87.8	15.3	52	45	0	2	1,539.91
									ISLPLPNFSSLNLR	95.0%	114.0	17.2	110	11	0	2	1,570.90
									KLLEGEESR	95.0%	57.1	23.8	24	22	0	2	1,060.56
									KVESLQEEIAFLK	95.0%	118.0	19.1	33	47	0	2	1,533.85
									LGDLYEEEMR	95.0%	68.1	19.8	67	0	0	2	1,270.56
									LGDLYEEEMRELR	95.0%	37.5	21.8	2	4	0	2	1,668.79

									LLEGEESR	95.0%	54.4	23.5	31	0	0	2	932.47
									LLQDSVDFSLADAINTEFK	95.0%	118.0	21.4	14	0	0	2	2,126.07
									LQDEIQNMK	95.0%	43.2	23.7	6	0	0	2	1,134.55
									LQDEIQNMKEEMAR	95.0%	68.7	22.5	42	13	0	2	1,734.82
									LQEEMLQR	95.0%	52.1	23.3	11	0	0	2	1,062.53
									LQEEMLQREEAENTLQSFR	95.0%	67.3	21.8	7	35	0	2	2,367.13
									MALDIEIATYR	95.0%	66.9	22.8	64	0	0	2	1,311.66
									MALDIEIATYRK	95.0%	63.3	21.0	7	3	0	2	1,439.76
									MFGGPGTASRPSSSR	95.0%	29.8	21.2	0	2	0	2	1,510.71
									NLQEAEEWYK	95.0%	55.5	21.3	7	0	0	2	1,309.61
									NNDALR	95.0%	33.6	19.5	2	0	0	2	702.35
									QDVDNASLAR	95.0%	88.3	22.4	89	0	0	2	1,088.53
									QESTEYR	95.0%	36.6	18.3	2	0	0	2	912.41
									QMREMEENFAVEAANYQDTIGR	95.0%	73.7	17.3	0	11	0	2	2,634.16
									QQYESVAAK	95.0%	56.4	21.9	12	0	0	2	1,023.51
									QVDQLTNDK	95.0%	56.2	23.0	14	0	0	2	1,060.53
									QVDQLTNDKAR	95.0%	52.0	22.2	10	2	0	2	1,287.67
									QVQSLTCEVDALKGTNESLER	95.0%	33.1	21.7	0	3	0	2	2,377.17
									RQVDQLTNDK	95.0%	49.8	23.6	4	0	0	2	1,216.63
									SLYASSPGGVYATR	95.0%	85.6	22.1	19	0	0	2	1,428.71
									SRLGDLYEEEMR	95.0%	39.6	20.7	3	2	0	2	1,513.70
									SSVPGVR	95.0%	49.2	22.7	10	0	0	2	701.39
									SYVTTSTR	95.0%	42.4	21.1	2	0	0	2	914.46
									TNEKVELQELNDR	95.0%	88.6	22.5	33	46	0	2	1,587.80
									TVETRDGQVINETSQHHDDLE	95.0%	61.7	19.2	0	4	0	2	2,423.11
									TYSLGSALRPSTSR	95.0%	35.2	22.7	2	2	0	2	1,495.79
									VELQELNDR	95.0%	58.1	21.4	11	0	0	2	1,115.57
									VESLQEEIAFLK	95.0%	85.8	21.5	4	0	0	2	1,405.76
									VEVERDNLAEDIMR	95.0%	57.5	22.4	23	41	0	2	1,704.82
AP-2 complex subunit mu	AP2M1_HUMAN	AP2M1	49,638	100.00%	4	5	9	10.60%	LNYSDHDVIK	95.0%	51.9	23.5	2	0	0	2	1,203.60
									MIGGLFIYNHK	95.0%	49.4	22.4	2	0	0	2	1,308.68
									QNVNAAMVFEFLYK	95.0%	60.7	23.3	2	0	0	2	1,689.83
									SNFKPSLLAQK	95.0%	35.1	20.0	1	2	0	2	1,232.70
Asparaginyl-tRNA synthetase, cytoplasmic	SYNC_HUMAN	NARS	62,926	100.00%	7	11	25	17.30%	EDGTFYEFGEDIPEAPER	95.0%	67.5	18.3	2	0	0	2	2,100.90
									EPFPTIYVDSQKENER	95.0%	48.8	22.0	2	1	0	2	1,951.94
									IFDSEEILAGYKR	95.0%	33.0	21.8	0	1	0	2	1,540.80
									KEDGTFYEFGEDIPEAPER	95.0%	75.2	20.1	1	2	0	2	2,229.00
									LTESVDVLMPNVGEIVGGSMR	95.0%	75.7	22.5	2	1	0	2	2,235.10
									NLMFLVLR	95.0%	41.5	18.9	8	0	0	2	1,021.59
									SPAGSIVHELNPNFQPPK	95.0%	66.0	22.7	2	3	0	2	1,932.00
UTP--glucose-1-phosphate uridylyltransferase	UGPA_HUMAN	UGP2	56,924	100.00%	9	10	35	24.80%	EFPTVPLVK	95.0%	41.2	21.0	1	0	0	2	1,029.60
									GGTLTQYEGK	95.0%	51.8	21.9	2	0	0	2	1,053.52
									GLPDNISSVLNK	95.0%	65.1	22.2	4	0	0	2	1,256.69
									IQRPPEDSIQPYEK	95.0%	54.4	22.0	3	1	0	2	1,699.87
									LVEIAQVPK	95.0%	68.2	12.8	4	0	0	2	996.61
									NENTFLDLTVQQIEHLNK	95.0%	92.2	21.6	4	0	0	2	2,156.10
									SFENSLGINVPR	95.0%	62.2	23.1	3	0	0	2	1,332.69
									TLDGGLNVIQLETAVGAAIK	95.0%	103.0	15.7	12	0	0	2	1,983.11
									TYNTDVPLVLMNSFNTDEDTKK	95.0%	30.7	21.5	1	0	0	2	2,561.21
Cullin-4B	CUL4B_HUMAN	CUL4B	103,969	100.00%	10	11	27	13.10%	EAVEAIQNSTSIK	95.0%	77.2	23.2	2	0	0	2	1,389.72
									ETVEEQASTTER	95.0%	64.8	20.9	2	0	0	2	1,379.63

									GLNNLLDENR	95.0%	53.5	21.7	2	0	0	2	1,157.59
									IQDLSLLYQLFSR	95.0%	84.6	19.2	3	0	0	2	1,595.88
									LEEEADRLITYLDQTTQK	95.0%	31.2	21.6	0	1	0	2	2,166.09
									LITYLDQTTQK	95.0%	49.9	21.0	2	0	0	2	1,323.72
									SIFLFLDR	95.0%	34.4	19.7	1	0	0	2	1,010.57
									SLLSMLSDLQIYQDSFEQR	95.0%	92.2	21.0	2	0	0	2	2,289.11
									TIDGILLLIER	95.0%	56.3	15.1	9	0	0	2	1,255.76
									TLSHNLLVSEVYNQLK	95.0%	49.4	19.0	1	2	0	2	1,858.01
Ras-related protein Rab-5C	RAB5C_HUMAN	RAB5C	23,465	100.00%	4	4	8	23.60%	GVDLQENNPASR	95.0%	66.2	21.7	2	0	0	2	1,299.63
									LVLLGESAVGK	95.0%	45.0	20.1	2	0	0	2	1,085.66
									QASPNIVIALAGNK	95.0%	74.9	18.2	2	0	0	2	1,395.80
									TAMNVNEIFMAIAK	95.0%	60.0	22.3	2	0	0	2	1,584.78
Endophilin-A2	SH3G1_HUMAN	SH3GL1	41,473	99.50%	2	2	4	6.52%	QAVQILDELAEK	95.0%	80.3	21.8	2	0	0	2	1,356.74
									TIEYLQPNPASR	95.0%	67.1	22.4	2	0	0	2	1,388.72
Ras-related C3 botulinum toxin substrate 1	RAC1_HUMAN	RAC1	21,433	100.00%	4	4	6	17.20%	KLTPITYPQGLAMAK	95.0%	36.7	18.6	1	0	0	2	1,647.91
									LTPITYPQGLAMAK	95.0%	53.0	21.0	2	0	0	2	1,519.82
									TVFDEAIR	95.0%	37.3	22.3	1	0	0	2	950.49
									YLECSALTQR	95.0%	44.7	21.9	2	0	0	2	1,240.60
Metallothionein-1G	MT1G_HUMAN	MT1G	6,123	99.50%	2	2	4	32.30%	CAQGCICK	95.0%	36.9	16.0	2	0	0	2	996.41
									SCCSCCPVGCAK	95.0%	42.3	7.8	2	0	0	2	1,445.51
Histone H3.1t	H31T_HUMAN	HIST3H3	15,491	100.00%	2	2	8	13.20%	EIAQDFKTDLR	95.0%	35.8	22.8	2	0	0	2	1,335.69
									STELLIR	95.0%	50.5	21.9	6	0	0	2	831.49
Probable global transcription activator SNF2L1	SMCA1_HUMAN	SMARCA1	122,592	99.50%	2	2	2	1.99%	LLNILMQLR	95.0%	32.7	18.8	1	0	0	2	1,129.68
									TLQTIALLGYLK	95.0%	45.4	10.8	1	0	0	2	1,333.81
Ubiquilin-1	UBQL1_HUMAN	UBQLN1	62,502	100.00%	6	7	13	14.40%	ALSNLESIPGGYNALR	95.0%	43.0	22.1	1	0	0	2	1,674.88
									FQQQLEQLSAMGFLNR	95.0%	97.1	21.8	1	2	0	2	1,925.95
									NPAMMQEMMR	95.0%	33.6	10.4	1	0	0	2	1,302.50
									NPEISHMLNNPDIMR	95.0%	34.5	20.8	0	4	0	2	1,812.84
									QLIMANPQMQQLIQR	95.0%	48.0	21.4	2	0	0	2	1,843.95
									SHTDQLVLIFAGK	95.0%	31.5	20.8	0	2	0	2	1,428.79
Porphobilinogen deaminase	HEM3_HUMAN	HMBS	39,313	99.50%	2	3	7	8.59%	GPQLAAQNLGISLANLLLSK	95.0%	120.0	12.8	4	2	0	2	2,021.18
									IQTDSVVATLK	95.0%	32.1	21.4	1	0	0	2	1,174.67
Annexin A2	ANXA2_HUMAN	ANXA2	38,588	100.00%	18	24	137	52.50%	AEDGSVIDYELIDQDAR	95.0%	94.0	21.0	7	0	0	2	1,908.88
									AYTNFDAER	95.0%	41.3	19.6	2	0	0	2	1,086.49
									AYTNFDAERDALNIETAIK	95.0%	55.0	22.1	2	5	0	2	2,155.07
									DALNIETAIK	95.0%	59.8	24.8	6	0	0	2	1,087.60
									GLGTDEDSLIEIICSR	95.0%	80.4	22.2	6	0	0	2	1,777.86
									GVDEVTIVNILTNR	95.0%	102.0	21.4	20	5	0	2	1,542.85
									LSLEGDHSTPPSAYGSVK	95.0%	75.2	22.5	2	1	0	2	1,844.90
									QDIAFAYQR	95.0%	67.4	22.3	7	0	0	2	1,111.55
									RAEDGSVIDYELIDQDAR	95.0%	79.6	22.3	3	6	0	2	2,064.98
									SALSGHLETVILGLLK	95.0%	72.3	11.1	6	4	0	2	1,650.98
									SLYYYIQQDTK	95.0%	67.1	23.0	5	0	0	2	1,421.70
									SLYYYIQQDTKGDYQK	95.0%	33.2	21.6	0	1	0	2	2,012.96
									SYSPYDMLESIR	95.0%	66.6	21.3	9	0	0	2	1,476.67
									SYSPYDMLESIRK	95.0%	54.4	23.0	9	0	0	2	1,588.77
									TDLEKDIISDTSGDFR	95.0%	73.1	22.2	3	3	0	2	1,811.87
									TKGVDEVTIVNILTNR	95.0%	36.2	18.1	0	3	0	2	1,771.99
									TNQELQEINR	95.0%	65.4	23.5	12	0	0	2	1,244.62
									TPAQYDASELK	95.0%	48.0	23.0	10	0	0	2	1,222.60
60S ribosomal protein L9	RL9_HUMAN	RPL9	21,846	100.00%	3	3	7	15.60%	FLDGIYVSEK	95.0%	53.4	20.7	2	0	0	2	1,170.60

									KFLDGIYVSEK	95.0%	34.9	21.6	1	0	0	2	1,298.70
									TILSNQTVDIPENVDITLK	95.0%	87.9	19.1	4	0	0	2	2,113.14
Aldo-keto reductase family 1 member C1	AK1C1_HUMAN	AKR1C1	36,771	99.50%	2	2	6	16.70%	LAIEAGFR	95.0%	42.8	23.1	6	0	0	2	876.49
									LNDGHFMPVLGFGTYAPAEVPK	95.0%	52.9	20.8	0	4	0	2	2,376.17
									QNVQVFEFQLTSEEMK	95.0%	86.1	21.3	2	0	0	2	1,972.93
									TPALIALR	95.0%	56.9	14.8	4	0	0	2	854.55
BRO1 domain-containing protein BROX	BROX_HUMAN	BROX	46,460	100.00%	4	4	7	16.10%	ATAPVSFNYYGVVTGPSASK	95.0%	112.0	22.0	2	0	0	2	2,016.01
									HAPGLIAALAYETANFYQK	95.0%	49.6	21.8	0	2	0	2	2,078.07
									IPTEAPQLELK	95.0%	43.5	19.0	2	0	0	2	1,238.70
									LLELFTDLSCNPEMMK	95.0%	42.2	20.5	1	0	0	2	1,972.91
Sialidase-1	NEUR1_HUMAN	NEU1	45,449	99.50%	2	2	6	4.58%	GTLLAFAEAR	95.0%	83.2	21.9	4	0	0	2	1,048.58
									IPLITATPR	95.0%	42.6	14.8	2	0	0	2	981.61
60S ribosomal protein L12	RL12_HUMAN	RPL12	17,801	100.00%	5	5	16	38.20%	CTGGEVGATSALAPK	95.0%	95.6	22.6	2	0	0	2	1,418.70
									HSGNITFDEIVNIAR	95.0%	39.6	22.1	0	4	0	2	1,685.86
									IGPLGLSPK	95.0%	49.8	11.5	5	0	0	2	881.55
									KVGDDIAK	95.0%	47.1	23.4	2	0	0	2	845.47
									QAQIEVVPSASALIIK	95.0%	74.3	12.8	3	0	0	2	1,666.97
Ras-related protein Rab-27B	RB27B_HUMAN	RAB27B	24,591	100.00%	4	4	7	21.60%	ADLPDQR	95.0%	32.0	20.8	1	0	0	2	814.41
									AVETLLDLIMK	95.0%	57.0	21.3	4	0	0	2	1,261.71
									LLALGDSGVGK	95.0%	31.9	21.5	1	0	0	2	1,029.59
									YGIPYFETSAATGQNVEK	95.0%	70.5	21.3	1	0	0	2	1,974.95
Glutaminyl-peptide cyclotransferase-like protein	QPCTL_HUMAN	QPCTL	42,908	99.50%	2	2	5	7.33%	HLAQLMESIPHSPGPTR	95.0%	34.8	21.6	0	2	0	2	1,886.95
									VPLIGSLPEAR	95.0%	53.6	16.2	3	0	0	2	1,151.68
Inter-alpha-trypsin inhibitor heavy chain H4	ITIH4_HUMAN	ITIH4	103,340	99.50%	2	2	6	1.94%	FAHTVVTSR	95.0%	43.6	23.9	1	0	0	2	1,017.55
									LGVYELLLK	95.0%	50.6	7.0	5	0	0	2	1,047.65
UV excision repair protein RAD23 homolog A	RD23A_HUMAN	RAD23A	39,591	100.00%	5	5	12	23.70%	ASYNNPHR	95.0%	39.0	19.1	3	0	0	2	958.45
									DQPQFQNMR	95.0%	40.6	19.8	2	0	0	2	1,179.52
									EDKSPSEESAPTTSPESVSGSVPSSGSSGR	95.0%	52.1	17.9	0	3	0	2	2,922.31
									ILSDDVPIR	95.0%	47.5	19.7	2	0	0	2	1,027.58
									QVIQQNPALLPALLQQLGQENPQLLQQISR	95.0%	56.2	11.8	0	2	0	2	3,378.89
Inositol monophosphatase 1	IMPA1_HUMAN	IMPA1	30,171	100.00%	3	3	6	13.70%	LQVSQQEDITK	95.0%	57.6	23.3	2	0	0	2	1,288.68
									SLLVTELGSSR	95.0%	57.4	21.9	2	0	0	2	1,161.65
									SSPVDLVTATDQKVEK	95.0%	78.5	21.5	2	0	0	2	1,716.90
Exosome complex exonuclease RRP42	EXOS7_HUMAN	EXOSC7	31,817	100.00%	3	3	6	13.10%	GGDDLGTEIANTLYR	95.0%	90.1	22.7	4	0	0	2	1,594.77
									LGHTDILVGVK	95.0%	26.0	16.9	0	1	0	2	1,151.68
									VYIVHGVQEDLR	95.0%	27.3	21.3	0	1	0	2	1,427.76
Mps one binder kinase activator-like 1A	MOL1A_HUMAN	MOBKL1A	25,074	100.00%	2	2	5	10.60%	ELAPLQELIEK	95.0%	51.8	18.5	4	0	0	2	1,282.73
									HAEATLGSGNLR	95.0%	52.6	22.0	1	0	0	2	1,225.63
Carbohydrate sulfotransferase 14	CHSTE_HUMAN	CHST14	42,980	99.90%	2	2	8	5.59%	ALLQDVLPK	95.0%	51.1	12.8	4	0	0	2	996.61
									VLAGVLDSVDVR	95.0%	57.5	20.8	4	0	0	2	1,242.71
Actin-related protein 2/3 complex subunit 1B	ARC1B_HUMAN	ARPC1B	40,932	99.50%	2	3	14	8.33%	ASSEGGTAAGAGLDSLHK	95.0%	94.6	21.8	8	2	0	2	1,628.79
									NSVSQISVLSGGK	95.0%	80.4	22.0	4	0	0	2	1,275.69
Adapter molecule crk	CRK_HUMAN	CRK	33,813	100.00%	6	7	25	29.30%	ALFDFNGNDEEDLPFK	95.0%	72.4	21.0	3	0	0	2	1,870.85
									DSSTSPGDYVLSVSENSR	95.0%	91.3	19.2	4	0	0	2	1,899.86
									IGDQEFDSLPALLEFYK	95.0%	78.3	22.4	5	0	0	2	1,984.99
									IHYLDTTTLIEPVSR	95.0%	80.3	20.8	4	4	0	2	1,757.94
									QEAVALLQGQR	95.0%	59.0	20.4	2	0	0	2	1,212.67
									TALALEVGELVK	95.0%	81.4	19.5	3	0	0	2	1,242.73
Proline synthase co-transcribed bacterial homolog protein	PROSC_HUMAN	PROSC	30,326	100.00%	5	5	16	21.50%	APLEVAQEH	95.0%	40.0	24.0	2	0	0	2	993.50
									LMAVPNLFMLETVDSVK	95.0%	63.1	21.5	5	0	0	2	1,938.99
									TFGENYVQELLEK	95.0%	78.9	23.1	5	0	0	2	1,569.78

									VMVQINTSGEESK	95.0%	70.5	22.1	2	0	0	2	1,437.69
									VQQAVAR	95.0%	49.0	19.8	2	0	0	2	771.45
Nucleolin	NUCL_HUMAN	NCL	76,598	100.00%	30	37	827	40.70%	AAVTPGKK	94.6%	30.1	20.0	1	0	0	2	771.47
									ALELTGLK	95.0%	48.3	20.7	14	0	0	2	844.51
									AVTTPGKK	95.0%	36.1	22.1	2	0	0	2	801.48
									EAMEDGEIDGNK	95.0%	56.6	14.1	3	0	0	2	1,323.54
									EAMEDGEIDGNKVTLDWAKPK	95.0%	40.6	21.3	0	1	0	2	2,362.12
									EVFEDAAEIR	95.0%	73.4	23.5	17	0	0	2	1,178.57
									FGYVDFESAEDLEK	95.0%	98.5	19.0	47	0	0	2	1,648.74
									GFGFVDFNSEEDAK	95.0%	81.8	17.3	41	0	0	2	1,561.68
									GIAYIEFK	95.0%	45.1	20.2	30	0	0	2	940.51
									GLSEDTTEETLK	95.0%	71.1	22.4	2	0	0	2	1,322.63
									GLSEDTTEETLKESFDGSVR	95.0%	69.8	21.6	2	8	0	2	2,200.03
									GQNQDYR	95.0%	38.4	19.4	6	0	0	2	880.39
									GYAFIEFASFEDAK	95.0%	82.2	21.0	4	0	0	2	1,594.74
									IVTDRETGSSK	95.0%	62.1	22.9	4	0	0	2	1,192.62
									KFGYVDFESAEDLEK	95.0%	98.2	22.0	24	18	0	2	1,776.83
									KGAAIPAK	95.0%	46.5	18.1	5	0	0	2	755.48
									LELQGPR	95.0%	34.9	20.2	4	0	0	2	812.46
									NDLAVVDVR	95.0%	80.2	21.6	53	0	0	2	1,000.54
									NSTWSGESK	95.0%	33.1	20.8	2	0	0	2	995.44
									QGTEIDGR	95.0%	75.6	22.0	40	0	0	2	875.42
									QKVEGTEPTTAFNLFVGNLNFNK	95.0%	72.5	20.8	0	29	0	2	2,568.31
									SISLYYTGEK	95.0%	57.1	23.7	46	0	0	2	1,160.58
									TEADAEKTFEEK	95.0%	71.7	21.3	40	9	0	2	1,397.64
									TGISDVFAK	95.0%	74.7	20.9	64	0	0	2	937.50
									TLVLSNLSYSATEETLQEVFEK	95.0%	135.0	21.0	36	36	0	2	2,501.27
									VAVATPAKK	95.0%	35.8	16.3	2	0	0	2	884.56
									VEGTEPTTAFNLFVGNLNFNK	95.0%	108.0	21.5	78	58	0	2	2,312.16
									VFGNEIKLEKPK	95.0%	28.8	17.5	0	1	1	2	1,401.81
									VTQDELK	95.0%	40.1	24.5	6	0	0	2	832.44
									VTQDELKEVFEDAAEIR	95.0%	131.0	22.1	47	46	0	2	1,991.99
60S ribosomal protein L30	RL30_HUMAN	RPL30	12,767	100.00%	4	4	9	51.30%	KSEIEYYAMLAK	95.0%	46.2	23.0	3	0	0	2	1,445.74
									LVILANNCPALR	95.0%	51.5	18.4	2	0	0	2	1,353.77
									TGVHHYSGNNIELGTACGK	95.0%	55.6	21.4	0	2	0	2	2,014.94
									VCTLAIIDPGDSDIIR	95.0%	95.8	21.6	2	0	0	2	1,757.91
40S ribosomal protein S15	RS15_HUMAN	RPS15	17,023	100.00%	4	5	8	37.20%	DMIILPEMVGSMVGVYNGK	95.0%	72.6	22.1	3	0	0	2	2,069.01
									EAPPMEKPEVVK	95.0%	42.1	22.2	2	0	0	2	1,369.70
									GVDLDQLLDMSYEQLMQLYSAR	95.0%	26.9	21.0	0	1	0	2	2,620.23
									KEAPPMEKPEVVK	95.0%	27.4	21.4	0	1	1	2	1,497.80
Vesicle-associated membrane protein-associated protein A	VAPA_HUMAN	VAPA	27,876	99.50%	2	2	2	11.20%	FKGPFTDVVTTNLK	95.0%	53.8	20.4	1	0	0	2	1,566.85
									HEQILVLDPPTDLK	95.0%	40.1	20.3	1	0	0	2	1,617.89
Peptidyl-glycine alpha-amidating monooxygenase	AMD_HUMAN	PAM	108,315	100.00%	3	3	10	3.91%	EGPVLILGR	95.0%	46.1	14.6	4	0	0	2	953.58
									GSGGLNLGNFFASR	95.0%	64.3	22.3	2	0	0	2	1,396.70
									IPVDEEAFVIDFKPR	95.0%	63.9	21.5	0	4	0	2	1,774.94
40S ribosomal protein S18	RS18_HUMAN	RPS18	17,701	100.00%	7	8	20	38.80%	AGELTEDEVER	95.0%	65.0	20.0	2	0	0	2	1,247.58
									IAFAITAIK	95.0%	47.1	11.8	2	0	0	2	947.59
									IPDWFLNR	95.0%	43.0	24.3	4	0	0	2	1,060.56
									RAGELTEDEVER	95.0%	55.9	21.7	2	2	0	2	1,403.68
									VITIMQNPR	95.0%	59.7	24.1	4	0	0	2	1,087.59
									VLNTNIDGR	95.0%	51.1	23.0	2	0	0	2	1,001.54

									YSQVLANGLDNK	95.0%	56.3	22.7	2	0	0	2	1,321.68
Hematological and neurological expressed 1-like protein	HN1L_HUMAN	HN1L	20,046	99.50%	2	2	4	14.70%	GSGIFDESTPVQTR	95.0%	47.6	23.4	2	0	0	2	1,493.72
									TSDIFGSPVTATSR	95.0%	52.8	22.6	2	0	0	2	1,438.72
Plasma membrane calcium-transporting ATPase 4	AT2B4_HUMAN	ATP2B4	137,906	99.90%	2	2	9	2.82%	EASDIILTDDNFTSIVK	95.0%	57.2	22.9	4	0	0	2	1,880.95
									YGDLLPADGILIQGNDLK	95.0%	64.6	20.8	5	0	0	2	1,915.02
Triosephosphate isomerase	TPIS_HUMAN	TPI1	26,651	100.00%	21	32	597	91.20%	DCGATWVVLGHSER	95.0%	76.4	21.4	4	4	0	2	1,586.74
									EAGITEK	95.0%	47.2	24.5	2	0	0	2	747.39
									ELASQPDVDGFLVGGASLKPEFVDIINAK	95.0%	75.8	18.4	0	54	0	2	3,029.58
									ELASQPDVDGFLVGGASLKPEFVDIINAKQ	95.0%	85.8	18.1	0	4	0	2	3,157.64
									FFVGGNWK	95.0%	33.5	20.8	1	0	0	2	954.48
									HVFGESDELIGQK	95.0%	78.1	22.8	33	33	0	2	1,458.72
									IAVAAQNCYK	95.0%	52.3	23.3	13	0	0	2	1,137.57
									IIYGGSVTGATCK	95.0%	115.0	22.4	8	0	0	2	1,326.67
									KQSLGELIGTLNAAK	95.0%	96.9	17.3	15	12	0	2	1,542.89
									LDEREAGITEK	95.0%	34.5	22.8	1	4	0	2	1,260.64
									QSLGELIGTLNAAK	95.0%	110.0	20.6	37	0	0	2	1,414.79
									RHVFGESDELIGQK	95.0%	77.6	22.6	6	13	1	2	1,614.82
									SNVSDAVAQSTR	95.0%	107.0	22.7	53	0	0	2	1,234.60
									TATPQQAQEVHEK	95.0%	77.0	22.9	95	11	0	2	1,466.72
									VAHALAEGLGVIACIGEK	95.0%	119.0	20.1	24	42	0	2	1,807.97
									VIADNVK	95.0%	33.0	23.6	1	0	0	2	758.44
									VIADNVKDWSK	95.0%	46.5	23.3	4	0	0	2	1,274.67
									VPADTEVVCAPPTAYIDFAR	95.0%	91.1	21.8	6	3	0	2	2,192.07
									VTNGAFTGEISPGMIK	95.0%	114.0	22.5	15	2	0	2	1,637.82
									VVFEQTK	95.0%	42.8	21.9	5	0	0	2	850.47
									VVLAYEPVWAIGTGK	95.0%	95.4	19.3	88	3	0	2	1,602.89
Heterogeneous nuclear ribonucleoproteins C1/C2	HNRPC_HUMAN	HNRNPC	33,653	100.00%	10	10	72	30.40%	AAVAGEDGR	95.0%	33.6	20.7	1	0	0	2	845.41
									GFAFVQYVNER	95.0%	74.2	22.3	10	0	0	2	1,329.66
									KSDVEAIFSK	95.0%	44.2	22.0	2	0	0	2	1,123.60
									MIAGQVLDINLAAEPK	95.0%	110.0	19.8	14	0	0	2	1,682.92
									NDKSEEEQSSSSVK	95.0%	76.7	19.9	5	0	0	2	1,553.69
									QKVDSLLENLEK	95.0%	52.1	20.9	5	0	0	2	1,415.77
									SDVEAIFSK	95.0%	37.6	21.5	2	0	0	2	995.51
									VDSLLENLEK	95.0%	50.1	24.0	3	0	0	2	1,159.62
									VFIGNLNTLVVK	95.0%	82.1	13.6	29	0	0	2	1,316.79
									VPPPPPIAR	95.0%	35.9	18.1	1	0	0	2	943.57
Ephrin type-A receptor 7	EPHA7_HUMAN	EPHA7	112,080	100.00%	3	3	7	5.11%	AFTAAGYGNYSPR	95.0%	41.3	21.1	3	0	0	2	1,374.64
									IDTIAADESFTQGDLGER	95.0%	83.1	21.4	2	0	0	2	1,937.91
									STSASINNLKPGTVYVFQIR	95.0%	33.8	18.5	0	2	0	2	2,195.18
Protocadherin-1	PCDH1_HUMAN	PCDH1	114,726	100.00%	8	8	19	9.72%	DMNDNAPTIEIR	95.0%	65.8	20.0	3	0	0	2	1,404.64
									GLFTISPETGEIQVK	95.0%	64.4	22.0	2	0	0	2	1,618.87
									LEVGAPYLR	95.0%	40.9	21.8	2	0	0	2	1,017.57
									NTGLITVQGPVDREDLSTLR	95.0%	47.7	19.7	0	3	0	2	2,184.16
									TGDIFTTETSIDR	95.0%	68.5	22.3	1	0	0	2	1,455.70
									VQDGGSPPR	94.8%	30.3	21.1	1	0	0	2	912.45
									VTVLDTNDNAPK	95.0%	67.7	22.9	2	0	0	2	1,286.66
									YFLQTTTPLDYEK	95.0%	80.6	22.7	5	0	0	2	1,618.80
Poliovirus receptor	PVR_HUMAN	PVR	45,284	100.00%	3	4	24	11.30%	HGESGSMAVFHQTQGPSYSESK	95.0%	42.3	18.0	0	5	0	2	2,367.03
									VLAKPQNTAEVQK	95.0%	56.2	19.0	12	1	0	2	1,425.81
									VQLTGEPVPMAR	95.0%	42.9	22.0	6	0	0	2	1,313.69
S-adenosylmethionine synthase	METK2_HUMAN	MAT2A	43,643	100.00%	8	10	41	23.30%	AAVDYQK	95.0%	36.2	22.5	4	0	0	2	794.40

isoform type-2									DLDLKKPIYQR	95.0%	37.5	20.3	1	2	0	2	1,388.79
									FVIGGPQGDAGLTGR	95.0%	99.4	22.7	11	0	0	2	1,444.76
									HIGYDDSSK	95.0%	48.5	19.8	4	0	0	2	1,021.46
									NFDLRPGVIVR	94.8%	25.8	19.6	0	1	0	2	1,285.74
									TGMILLAGEITSR	95.0%	64.7	22.1	4	0	0	2	1,377.74
									TQVTVQYMQDR	95.0%	49.5	22.1	3	0	0	2	1,384.65
									YLDEDTIYHLQPSGR	95.0%	63.6	22.0	6	5	0	2	1,806.87
Phenylalanyl-tRNA synthetase beta chain	SYFB_HUMAN	FARSB	66,101	100.00%	9	10	18	18.30%	ALVAIGTHDLDTLSGPFTYTAK	95.0%	35.4	20.7	0	1	0	2	2,291.19
									ASEGPAFFPGR	95.0%	77.0	22.5	2	0	0	2	1,135.55
									DLLFQALGR	95.0%	51.7	22.0	2	0	0	2	1,032.58
									IRPFAVAAVLR	95.0%	42.8	11.1	0	2	0	2	1,212.76
									LFEISDIVIK	95.0%	54.3	17.5	2	0	0	2	1,176.69
									LGVLHPDVITK	95.0%	35.8	16.7	1	0	0	2	1,191.71
									NPGFEIIHGLLDR	95.0%	72.4	21.0	2	2	0	2	1,480.79
									TTLLPGLLK	95.0%	39.2	7.8	2	0	0	2	955.62
									TYTIANQFPLNK	95.0%	49.0	21.8	2	0	0	2	1,409.74
Protein phosphatase methylesterase 1	PPME1_HUMAN	PPME1	42,298	99.50%	2	2	4	5.96%	LLLLAGVDR	95.0%	52.8	16.6	2	0	0	2	969.61
									VKNPEDLSAETMAK	95.0%	57.6	22.3	2	0	0	2	1,548.76
Peptidyl-prolyl cis-trans isomerase FKBP10	FKB10_HUMAN	FKBP10	64,228	100.00%	8	9	18	13.40%	ASPAGGPLEDVVIER	95.0%	74.7	21.9	3	0	0	2	1,509.79
									DGEVPPEEFSTFIK	95.0%	32.9	22.7	1	0	0	2	1,594.76
									EVQMGDFVR	95.0%	37.9	22.4	3	0	0	2	1,096.51
									IIIPPFLAYGEK	95.0%	40.8	18.1	1	0	0	2	1,360.79
									ITVDELK	95.0%	31.3	23.0	3	0	0	2	817.47
									KIIIPPFLAYGEK	95.0%	36.8	12.8	1	2	0	2	1,488.88
									MVQDGDFVR	95.0%	30.5	20.8	2	0	0	2	1,082.49
									TIGDMFQNQDR	95.0%	33.4	18.6	2	0	0	2	1,340.59
Filaggrin-2	FILA2_HUMAN	FLG2	248,034	100.00%	4	4	34	2.80%	FSNSSSSNEFSK	95.0%	61.4	16.0	5	0	0	2	1,320.57
									HQEEESETEEDEEDTPGHK	95.0%	40.8	10.4	0	1	0	2	2,254.89
									SGQSSYGQHSSGSSQSSGYGQHGSR	95.0%	36.2	14.1	0	3	0	2	2,500.05
									SVVTVIDVFYK	95.0%	55.7	19.9	25	0	0	2	1,269.71
Putative pre-mRNA-splicing factor ATP-dependent RNA helicase DHX15	DHX15_HUMAN	DHX15	90,917	100.00%	6	6	38	8.18%	EAMNDPLLER	95.0%	41.1	21.6	1	0	0	2	1,203.57
									EVDDLGPEVGDIK	95.0%	67.8	22.4	2	0	0	2	1,385.68
									FTDILVR	94.5%	30.0	19.7	1	0	0	2	863.50
									SNLGSVVLQLK	95.0%	75.3	18.6	3	0	0	2	1,157.69
									TLATDILMGVLK	95.0%	77.0	19.0	30	0	0	2	1,290.73
									YGVIILDEAHER	95.0%	37.5	21.9	0	1	0	2	1,414.73
Alpha-actinin-4	ACTN4_HUMAN	ACTN4	104,839	100.00%	50	64	483	65.50%	ACLISLGYDVENDRQGEAEFNR	95.0%	45.2	20.6	0	1	0	2	2,556.18
									AGTQIENIDEDFR	95.0%	71.9	21.0	1	0	0	2	1,507.70
									AGTQIENIDEDFRDGLK	95.0%	77.7	22.0	4	3	0	2	1,920.93
									AIMTYVSSFYHAFSGAQK	95.0%	101.0	22.3	15	4	0	2	2,023.96
									ALDFIASK	95.0%	55.2	21.2	12	0	0	2	864.48
									ASFNHFDKDHGGALGPEEFK	95.0%	46.4	20.3	0	3	2	2	2,203.02
									ASIHEAWTDGK	95.0%	30.5	20.7	1	0	0	2	1,214.58
									CQLEINFNTLQTK	95.0%	96.1	22.9	4	0	0	2	1,608.81
									DDPVTNLNNAFEVAEK	95.0%	103.0	22.0	7	0	0	2	1,775.85
									DGLAFNALIHR	95.0%	52.6	21.6	8	5	0	2	1,226.66
									DYETATLSDIK	95.0%	62.3	21.7	9	0	0	2	1,255.61
									EAILAIHK	95.0%	47.5	15.2	7	0	0	2	894.54
									EGLLLWCQR	95.0%	46.4	23.2	3	0	0	2	1,174.60
									ELPPDQAEYCIAR	95.0%	53.2	20.9	7	0	0	2	1,561.73
									ETTDTDTADQVIASFK	95.0%	95.8	21.3	23	0	0	2	1,741.81

									FAIQDISVEETSAK	95.0%	97.4	22.4	36	0	0	2	1,537.78
									GISQEQMQEFR	95.0%	63.8	20.9	20	0	0	2	1,368.62
									GYEEWLLNEIR	95.0%	75.4	23.5	4	0	0	2	1,421.71
									HRDYETATLSDIK	95.0%	56.5	22.9	4	6	0	2	1,548.77
									HTNYTMEHIR	95.0%	43.8	20.0	2	4	0	2	1,317.60
									IAESNHIK	95.0%	34.2	20.1	1	0	0	2	911.50
									ICDQWDALGSLTHSR	95.0%	54.5	20.8	0	4	0	2	1,758.82
									ISIEMNGTLEDQLSHLK	95.0%	80.3	22.4	4	8	0	2	1,943.98
									KDDPVTNLNNAFEVAEK	95.0%	114.0	21.7	7	10	0	2	1,903.94
									LASDLLEWIR	95.0%	95.8	22.0	4	0	0	2	1,215.67
									LDHLAEK	95.0%	38.2	19.3	7	0	0	2	825.45
									LMLLLEVISGER	95.0%	80.2	19.6	7	0	0	2	1,388.78
									LSGSNPYTTVTPQIINSK	95.0%	110.0	21.3	24	0	0	2	1,920.01
									LSNRPAFMPSEGK	95.0%	37.8	22.9	3	5	0	2	1,449.72
									LVSIGAEEIVDGNAK	95.0%	101.0	22.0	30	0	0	2	1,514.81
									MAPYQGPDAVPGALDYK	95.0%	92.8	22.1	9	0	0	2	1,808.85
									MLDAEDIVNTARPDEK	95.0%	84.2	22.0	6	8	0	2	1,832.87
									MTLGMIWTIILR	95.0%	93.8	20.2	13	0	0	2	1,479.81
									NFITAEELR	95.0%	45.9	23.3	2	0	0	2	1,092.57
									NVNVQNFHISWK	95.0%	61.1	22.4	2	0	0	2	1,485.76
									QFASQANVVGPWIQTK	95.0%	103.0	21.9	4	0	0	2	1,773.93
									QGEAEFNR	95.0%	31.6	21.5	1	0	0	2	950.43
									QLEAIDQLHLEYAK	95.0%	95.2	21.7	6	0	0	2	1,670.88
									RQFASQANVVGPWIQTK	95.0%	28.2	20.6	0	1	0	2	1,930.03
									SIVDYKPNLDLLEQQHQLIQEALIFDNK	95.0%	41.6	18.9	0	2	0	2	3,324.75
									STLPDADRER	95.0%	44.0	22.8	6	7	0	2	1,159.57
									TINEVENQILTR	95.0%	76.3	22.2	26	0	0	2	1,429.77
									TIPWLEDRVPQK	95.0%	33.0	20.8	1	0	0	2	1,481.81
									TIQEMQQK	95.0%	43.1	22.5	2	0	0	2	1,021.50
									VEQIAAIAQELNELDYYDSHNVNTR	95.0%	96.7	20.8	0	11	1	2	2,905.40
									VGWEQLLTTIAR	95.0%	86.1	20.5	33	0	0	2	1,386.77
									VLAGDKNFITAEELR	95.0%	89.5	20.5	4	7	0	2	1,675.90
									VLAVNQENEHLMEDYEK	95.0%	70.5	20.4	6	6	0	2	2,076.95
									VQQLVPK	95.0%	42.1	15.4	7	0	0	2	811.50
									YLDIPK	95.0%	34.2	19.0	3	0	0	2	748.42
Cohesin subunit SA-2	STAG2_HUMAN	STAG2	141,314	99.50%	2	2	2	2.11%	FALTFGLDQLK	95.0%	42.4	19.4	1	0	0	2	1,252.69
									ITELFAVALPQLLAK	95.0%	37.0	10.8	1	0	0	2	1,626.98
Splicing factor, arginine/serine-rich 2	SFRS2_HUMAN	SFRS2	25,459	99.90%	2	2	14	11.30%	DAEDAMDAMDGAVLDGR	95.0%	104.0	13.8	4	0	0	2	1,783.71
									VGDVYIPR	95.0%	61.8	22.8	10	0	0	2	918.51
3-hydroxyisobutyrate dehydrogenase, mitochondrial	3HIDH_HUMAN	HIBADH	35,312	100.00%	4	5	7	16.10%	DFSSVFQFLR	95.0%	32.8	22.3	1	0	0	2	1,245.63
									DLGLAQDSATSTK	95.0%	64.0	23.4	1	0	0	2	1,306.65
									SPILLGSLAHQIYR	95.0%	73.6	17.7	1	2	0	2	1,567.90
									TPVGFIGLGNMGNPMAK	95.0%	55.6	22.4	2	0	0	2	1,735.85
Bone morphogenetic protein 1	BMP1_HUMAN	BMP1	111,231	100.00%	13	14	156	16.40%	AAAFLGDIALDEEDLR	95.0%	87.8	22.4	2	0	0	2	1,718.86
									AFQVQQAVDLRR	94.6%	25.6	20.7	0	1	0	2	1,430.79
									AGFAVNFFK	95.0%	72.8	21.6	35	0	0	2	1,000.53
									DGHSESSTLIGR	95.0%	54.7	20.9	7	0	0	2	1,258.60
									DYGHIQSPNYPDDYRPSK	95.0%	45.5	20.1	0	6	7	2	2,151.97
									ENIQPGQEYNFLK	95.0%	74.2	23.0	13	0	0	2	1,579.78
									FYSDNSVQR	95.0%	55.5	20.3	11	0	0	2	1,115.51
									GDIAQAR	95.0%	51.4	21.9	7	0	0	2	730.38

									GGGPQAISIGK	95.0%	52.4	18.9	6	0	0	2	984.55
									GIFLDTIVPK	95.0%	56.4	18.2	24	0	0	2	1,102.65
									KPEPVLATGSR	95.0%	44.8	20.2	2	0	0	2	1,154.65
									LPEPIVSTDSR	95.0%	62.1	22.4	22	0	0	2	1,213.64
									MEPQEVESLGETYDFDSIMHYAR	95.0%	67.6	16.6	0	13	0	2	2,779.19
UPF0568 protein C14orf166	CN166_HUMAN	C14orf166	28,051	100.00%	5	5	10	25.80%	AGVMALANLLQIQR	95.0%	72.4	19.5	2	0	0	2	1,513.85
									HDDYLVMLK	95.0%	36.9	22.7	1	0	0	2	1,149.56
									INEAIVAVQAIIADPK	95.0%	75.7	15.3	1	0	0	2	1,664.96
									LTQDAVAK	95.0%	37.7	23.4	2	0	0	2	845.47
									NAEPLINLDVNNPDFK	95.0%	99.3	22.0	4	0	0	2	1,812.91
Isochorismatase domain-containing protein 1	ISOC1_HUMAN	ISOC1	32,219	100.00%	4	4	8	19.80%	FSMVLPEVEAALAEIPGVR	95.0%	44.3	21.8	2	0	0	2	2,044.08
									GLGSTVQEIDLTGVK	95.0%	84.8	21.4	2	0	0	2	1,516.82
									ILGIPVIVTEQYPK	95.0%	49.9	15.2	3	0	0	2	1,569.93
									YFGDIISVGQR	95.0%	35.2	22.7	1	0	0	2	1,254.65
Tripeptidyl-peptidase 2	TPP2_HUMAN	TPP2	138,335	100.00%	2	2	6	2.00%	GTLTEAFPVLGGK	95.0%	61.4	21.1	5	0	0	2	1,289.71
									LSTMETGTGLIR	95.0%	40.4	22.8	1	0	0	2	1,294.67
Protein S100-A4	S10A4_HUMAN	S100A4	11,711	100.00%	7	8	14	48.50%	ALDVMVSTFHK	95.0%	53.3	22.9	2	0	0	2	1,247.65
									ELPSFLGK	95.0%	30.6	20.3	1	0	0	2	890.50
									LMSNLDSNR	95.0%	51.0	21.7	2	0	0	2	1,065.50
									LNKSELK	95.0%	40.0	21.9	1	0	0	2	831.49
									RTDEAAFQK	95.0%	47.7	22.8	2	0	0	2	1,065.53
									SELKELLTR	95.0%	46.3	19.8	3	1	0	2	1,088.63
									TDEAAFQK	95.0%	57.4	21.8	2	0	0	2	909.43
Torsin-1B	TOR1B_HUMAN	TOR1B	37,963	100.00%	3	3	5	11.30%	DLEPVLSVGVFNNK	95.0%	73.9	22.1	2	0	0	2	1,530.82
									NFVSQIVAENLHPK	95.0%	93.0	21.0	2	0	0	2	1,595.85
									VAEEMTFFPR	95.0%	45.3	22.1	1	0	0	2	1,226.59
Glyoxylate reductase/hydroxypyruvate reductase	GRHPR_HUMAN	GRHPR	35,651	100.00%	7	7	16	32.00%	GDVVNQDDLYQALASGK	95.0%	86.8	22.3	2	0	0	2	1,792.87
									GEPMPSELKL	95.0%	30.8	22.6	1	0	0	2	1,116.56
									IAAAGLDVTSPEPLPTNHPLLTLK	95.0%	60.9	14.8	0	4	0	2	2,468.38
									ILDAAGANLK	95.0%	58.0	18.6	4	0	0	2	985.57
									NTMSLLAANNLLAGLR	95.0%	66.3	20.4	2	0	0	2	1,687.92
									RLPEAIEEVK	95.0%	43.9	19.6	1	0	0	2	1,183.67
									VISTMSVGIDHLALDEIK	95.0%	48.0	20.8	2	0	0	2	1,957.03
Vacuolar protein sorting-associated protein 45	VPS45_HUMAN	VPS45	65,060	100.00%	5	5	8	11.40%	ENVDYIIQELR	95.0%	69.7	22.4	2	0	0	2	1,391.72
									LKENLYPYLGPSTLR	94.6%	25.5	19.1	0	1	0	2	1,763.97
									SFLEEVLASGLHSR	95.0%	47.2	22.5	0	2	0	2	1,544.81
									TEVPPLLLILDR	95.0%	46.9	11.5	1	0	0	2	1,378.83
									TTQGLTALLLSLK	95.0%	50.4	13.2	2	0	0	2	1,358.83
COP9 signalosome complex subunit 3	CSN3_HUMAN	COPS3	47,857	100.00%	4	4	10	14.20%	AMDQEITVNPQFVQK	95.0%	94.3	23.1	4	0	0	2	1,763.86
									DGMVSFHDNPEK	95.0%	47.7	16.1	2	0	0	2	1,391.59
									TFLTLSLQDMASR	95.0%	67.5	21.6	2	0	0	2	1,498.76
									YATDTFAGLCHQLTNALVER	95.0%	49.8	22.4	0	2	0	2	2,280.11
Heterogeneous nuclear ribonucleoprotein D-like	HNRDL_HUMAN	HNRPDL	46,421	100.00%	3	3	8	12.90%	DAASVDKVLELK	95.0%	43.9	22.4	2	0	0	2	1,287.72
									DLTEYLSR	95.0%	42.3	21.8	4	0	0	2	996.50
									FGEVVDCTIK	95.0%	62.7	22.7	10	0	0	2	1,167.57
									GFGFVLFK	95.0%	46.7	22.9	31	0	0	2	914.51
									VFVGGLSPDTSEEQIK	95.0%	54.1	22.6	2	0	0	2	1,705.87
Polypeptide N-acetylgalactosaminyltransferase 2	GALT2_HUMAN	GALNT2	64,715	100.00%	9	9	26	23.10%	EIILVDDYSNDPEDGALLGK	95.0%	108.0	22.1	5	0	0	2	2,176.07
									FNQVESDKLR	95.0%	27.8	23.5	0	2	0	2	1,235.64
									NFYYAAVPSAR	95.0%	43.7	21.7	4	0	0	2	1,258.62
									NVPYGNIQSR	95.0%	31.0	24.1	1	0	0	2	1,147.59

									QHPYTFPGGSGTVFAR	95.0%	37.7	22.6	0	1	0	2	1,721.84
									TPMIAGGLFVMDK	95.0%	77.1	21.9	4	0	0	2	1,411.70
									VDLPATSVVITFHNEAR	95.0%	33.3	19.9	0	3	0	2	1,868.99
									VVSPIIDVINMDNFQYVGASADLK	95.0%	40.5	21.2	0	2	0	2	2,624.33
									WYLENVYPELR	95.0%	43.9	22.3	4	0	0	2	1,481.74
Inositol-3-phosphate synthase 1	INO1_HUMAN	ISYNA1	61,050	100.00%	6	6	21	16.70%	APLVPPGSPVVNALFR	95.0%	64.7	15.8	4	0	0	2	1,633.94
									FCEVIPGLNDTAENLLR	95.0%	42.3	22.6	1	0	0	2	1,960.98
									SVLVDFLIGSGLK	95.0%	57.9	15.3	4	0	0	2	1,347.79
									TMSIVSYNHLGNNDGENLSAPLQFR	95.0%	55.2	21.1	0	5	0	2	2,793.33
									VFVGGDDFK	95.0%	45.3	21.6	6	0	0	2	983.48
									VGPVAATYPMLNK	95.0%	43.0	22.9	1	0	0	2	1,376.72
Protein phosphatase 1G	PPM1G_HUMAN	PPM1G	59,254	100.00%	4	4	11	9.34%	ALEDAFLAIDAK	95.0%	70.0	23.2	2	0	0	2	1,276.68
									NLPPEEQMISALPDIK	95.0%	62.7	21.6	4	0	0	2	1,810.93
									NTAELQPESGKR	95.0%	30.7	23.0	1	0	0	2	1,329.68
									QLIVANAGDSR	95.0%	39.0	22.7	4	0	0	2	1,143.61
Semaphorin-3C	SEM3C_HUMAN	SEMA3C	85,190	100.00%	14	15	94	23.60%	EFPDDVVTFIR	95.0%	77.3	23.5	16	0	0	2	1,337.67
									EGPNHQLISYQGR	95.0%	64.6	21.7	6	3	0	2	1,498.74
									IIATSQGLLIR	95.0%	94.1	17.0	4	0	0	2	1,184.74
									ILLMDEDQDR	95.0%	51.1	22.0	6	0	0	2	1,263.59
									KQQLYVSSNEGVSQVSLHR	95.0%	63.8	21.4	0	2	0	2	2,159.12
									MAGKDPTHGCGNFVR	95.0%	32.6	20.5	0	2	0	2	1,662.75
									NAAEIVQYGVK	95.0%	63.3	22.7	8	0	0	2	1,191.64
									NHPLMYNSIYPIHK	95.0%	29.8	22.5	0	1	0	2	1,742.87
									SEDQVFMIDSK	95.0%	87.8	19.8	5	0	0	2	1,314.59
									SPQASIK	95.0%	46.6	22.9	6	0	0	2	730.41
									VLDSEMVAVVTDK	95.0%	64.1	23.6	2	0	0	2	1,421.72
									VYLTFDELR	95.0%	63.8	21.7	24	0	0	2	1,155.61
									WLSEPMFVDAHVIPDGTDPNDAK	95.0%	33.6	20.2	0	3	0	2	2,570.19
									YHVLFLGTDR	95.0%	39.0	22.8	6	0	0	2	1,220.64
Translin	TSN_HUMAN	TSN	26,165	100.00%	7	12	26	30.30%	EAVTEILGIEPDR	95.0%	65.5	22.2	2	0	0	2	1,441.75
									EAVTEILGIEPDREK	95.0%	62.8	21.6	5	2	0	2	1,698.89
									EILTLLQGVHQGAGFQDIPK	95.0%	87.0	17.9	2	3	0	2	2,164.18
									EILTLLQGVHQGAGFQDIPKR	95.0%	55.5	16.8	0	2	0	2	2,320.28
									KVEEVVYDLSIR	95.0%	77.7	20.8	3	1	0	2	1,449.80
									KVVQSLEQTAR	95.0%	66.6	21.5	2	1	0	2	1,258.71
									TKFPAEQYYR	95.0%	37.7	22.7	1	2	0	2	1,302.65
SUMO-conjugating enzyme UBC9	UBC9_HUMAN	UBE2I	17,990	100.00%	2	2	3	16.50%	KDHPFGFVAVPTK	95.0%	42.9	21.3	0	1	0	2	1,442.78
									MLFKDDYPSSPPK	95.0%	60.9	22.7	2	0	0	2	1,540.74
Neuroligin-2	NLGN2_HUMAN	NLGN2	93,818	99.50%	2	2	3	3.33%	LGVLGFLSTGDQAAK	95.0%	70.8	20.3	2	0	0	2	1,476.81
									TGDPNQPVPQDTK	95.0%	33.6	22.7	1	0	0	2	1,396.67
Cleavage and polyadenylation specificity factor subunit 6	CPSF6_HUMAN	CPSF6	59,193	99.50%	2	3	10	7.08%	AVSDASAGDYGSAIETLVTAISLIK	95.0%	139.0	19.9	6	2	0	2	2,452.28
									TPLSEAEFEEIMNR	95.0%	98.8	21.0	2	0	0	2	1,681.77
Serpin B12	SPB12_HUMAN	SERPINB12	46,260	99.50%	2	2	6	4.20%	IGFIEEVK	95.0%	34.0	19.1	2	0	0	2	934.53
									TQTILFYGR	95.0%	34.9	19.7	4	0	0	2	1,098.59
cAMP-dependent protein kinase type II-alpha regulatory subunit	KAP2_HUMAN	PRKAR2A	45,501	99.90%	2	2	3	5.69%	MFESFIESVPLLK	95.0%	41.0	22.2	2	0	0	2	1,555.81
									MKIVDVIGEK	94.6%	30.1	22.2	1	0	0	2	1,147.64
Nucleosome assembly protein 1-like 4	NP1L4_HUMAN	NAP1L4	42,806	100.00%	3	3	5	14.90%	AAATAEEPDPK	95.0%	51.7	21.8	1	0	0	2	1,099.53
									FYEEVHDLER	95.0%	47.7	21.0	5	0	0	2	1,336.62
									LDNVPHTPSSYIETLPK	95.0%	29.8	21.0	0	2	0	2	1,910.99
									NVDMLSELVQEYDEPILK	95.0%	73.2	21.9	2	0	0	2	2,151.05
Hexokinase-1	HXK1_HUMAN	HK1	102,470	100.00%	16	17	34	20.20%	ASGVEGADVVK	95.0%	34.7	22.7	1	0	0	2	1,031.54

									FLSQIESDRLALLQVR	95.0%	38.3	16.6	0	2	0	2	1,888.07
									GAALITAVGVR	95.0%	91.5	18.9	2	0	0	2	1,027.63
									GAAMVTAVAYR	95.0%	67.8	22.5	2	0	0	2	1,125.57
									GDFIALDLGGSSFR	95.0%	69.0	22.3	2	0	0	2	1,454.73
									GKFNTSDVSAIEK	95.0%	82.8	22.0	2	0	0	2	1,395.71
									ITPELLTR	95.0%	38.0	18.2	1	0	0	2	942.56
									LALLQVR	95.0%	34.0	15.2	2	0	0	2	812.54
									LSDETLIDIMTR	95.0%	76.9	22.7	2	0	0	2	1,422.72
									LVDEYSLNAGK	95.0%	78.9	23.3	2	0	0	2	1,208.62
									MISGMYLGEIVR	95.0%	66.9	21.5	2	0	0	2	1,400.69
									MLPTFVR	95.0%	36.8	23.5	1	0	0	2	879.48
									MVSGMYLGELVR	95.0%	45.6	21.5	2	0	0	2	1,386.68
									SANLVAATLGAILNR	95.0%	108.0	17.8	3	4	0	2	1,483.86
									TPDGTENGDFLALDLGGTNFR	95.0%	101.0	21.3	2	0	0	2	2,210.04
									TTVGVDGSLYK	95.0%	45.8	22.1	2	0	0	2	1,139.60
Thioredoxin reductase 1, cytoplasmic	TRXR1_HUMAN	TXNRD1	70,889	100.00%	20	32	158	40.50%	ALEGTLSELAAETDLPVVFVK	95.0%	87.7	18.1	2	0	0	2	2,202.19
									EKIYSAER	95.0%	34.3	21.3	1	0	0	2	995.52
									FLIATGERPR	95.0%	50.5	22.0	5	0	0	2	1,159.66
									GFDQDMANK	95.0%	46.1	17.5	2	0	0	2	1,041.43
									IGEHMEEHGIK	95.0%	46.4	21.5	9	4	0	2	1,295.61
									IGLETVGVK	95.0%	47.2	19.7	4	0	0	2	915.55
									IPVTDEEQTNVPYIYAIGDILEDKVELTPVAIQAGR	95.0%	95.1	16.7	0	4	3	2	3,970.07
									KLMHQAALLGQALQDSR	95.0%	90.1	20.5	2	6	2	2	1,896.01
									KVMVLDFVTPTPLGTR	95.0%	91.8	18.4	2	3	0	2	1,789.99
									KVVYENAYGQFIGPHR	95.0%	85.0	21.6	1	2	0	2	1,877.97
									LMHQAALLGQALQDSR	95.0%	106.0	21.3	2	2	0	2	1,767.92
									LYAGSTVK	95.0%	30.9	20.3	1	0	0	2	838.47
									MIEAVQNHIGSLNWGYR	95.0%	45.2	22.1	0	1	0	2	2,003.98
									QFVPIKVEQIEAGTPGR	95.0%	73.2	18.3	2	4	0	2	1,869.02
									SYDYDLIIIGGGSGGLAAAK	95.0%	106.0	22.0	27	1	0	2	1,941.00
									VEQIEAGTPGR	95.0%	66.7	22.4	2	0	0	2	1,156.60
									VMVLDFVTPTPLGTR	95.0%	89.5	21.0	17	1	0	2	1,661.89
									VVAQSTNSEEIIEGEYNTVMLAIGR	95.0%	42.7	21.3	0	3	0	2	2,739.35
									VVGFHVLGPNAGEVTQGFAAALK	95.0%	92.4	18.8	4	34	0	2	2,282.23
									VVYENAYGQFIGPHR	95.0%	76.5	22.9	3	2	0	2	1,749.87
Neural cell adhesion molecule L1	L1CAM_HUMAN	L1CAM	139,985	100.00%	6	7	20	6.21%	AQLLVVGSPGPVPR	95.0%	77.3	15.4	2	0	0	2	1,389.82
									DLQELGDSDKYFIEDGR	95.0%	31.4	21.6	0	2	0	2	1,999.93
									LGTAMSHEIR	95.0%	50.2	22.4	1	0	0	2	1,130.56
									LVLSDLHLLTQSQVR	95.0%	114.0	14.6	4	4	0	2	1,721.99
									LVVFPTDDISLK	95.0%	38.5	20.4	3	0	0	2	1,346.76
									VGEEDDGEYR	95.0%	59.6	14.3	4	0	0	2	1,168.48
UPF0027 protein C22orf28	CV028_HUMAN	C22orf28	55,192	100.00%	4	4	5	14.10%	GLGHQVATDALVAMEK	95.0%	34.2	21.5	0	1	0	2	1,655.84
									LVMEEAPESYK	95.0%	32.2	21.5	1	0	0	2	1,311.61
									QIGNVAALPGIVHR	95.0%	39.6	17.0	0	2	0	2	1,444.84
									TNLDESDVQPVKEQLAQAMFDHIPVGVGSK	95.0%	46.5	21.0	0	1	0	2	3,268.62
cAMP-dependent protein kinase type I-alpha regulatory subunit	KAP0_HUMAN	PRKAR1A	42,964	100.00%	2	2	3	7.35%	LTVADALEPVQFEDGQK	95.0%	59.6	22.2	2	0	0	2	1,859.94
									SENEEFVEVGR	95.0%	51.0	20.7	1	0	0	2	1,294.59
COP9 signalosome complex subunit 6	CSN6_HUMAN	COPS6	36,145	100.00%	3	3	10	16.80%	ASEAGEVPFNHEILR	95.0%	37.4	21.5	0	2	0	2	1,668.83
									MTATGSGENSTVAEHLIAQHSAIK	95.0%	27.6	22.3	0	1	0	2	2,469.20
									SQEGRPVQVIGALIGK	95.0%	38.2	15.3	0	7	0	2	1,651.95
Inosine-5'-monophosphate	IMDH2_HUMAN	IMPDH2	55,788	99.50%	2	2	5	6.03%	DKYPNLQVIGGNVVTAAQAK	95.0%	39.5	18.7	0	1	0	2	2,086.13

dehydrogenase 2									NLIDAGVDALR	95.0%	56.5	21.8	4	0	0	2	1,156.63
Complement factor B	CFAB_HUMAN	CFB	85,515	100.00%	24	29	236	31.00%	ALFVSEEEK	95.0%	32.7	22.4	1	0	0	2	1,051.53
									ALFVSEEEKK	95.0%	51.3	22.3	12	0	0	2	1,179.63
									CLVNLIEK	95.0%	50.9	24.0	4	0	0	2	988.55
									DAQYAPGYDK	95.0%	51.7	19.4	9	0	0	2	1,127.50
									DISEVVTPR	95.0%	60.2	21.5	6	0	0	2	1,015.54
									DNEQHVFK	95.0%	32.6	19.5	2	0	0	2	1,016.48
									EAGIPEFYDYDVALIK	95.0%	70.4	22.4	27	0	0	2	1,842.92
									EELLPAQDIK	95.0%	36.6	21.1	5	0	0	2	1,155.63
									EKLQDEDLGFL	95.0%	67.5	23.2	21	0	0	2	1,306.65
									GDSGGPLIVHK	95.0%	53.1	21.5	11	0	0	2	1,079.59
									HVIILMTDGLHNMGGDPITVIDEIR	95.0%	54.9	20.7	0	5	1	2	2,791.41
									ISVIRPSK	95.0%	50.2	18.9	8	0	0	2	899.57
									KCLVNLIEK	95.0%	36.2	20.8	1	0	0	2	1,116.65
									KDNEQHVFK	95.0%	31.6	22.8	1	1	0	2	1,144.58
									KEAGIPEFYDYDVALIK	95.0%	58.2	21.8	4	6	0	2	1,971.01
									LEDSVTYHCSR	95.0%	65.3	19.1	5	0	0	2	1,366.61
									LLQEGQALEYVCPSGFYPYPVQTR	95.0%	44.3	20.6	2	3	0	2	2,815.38
									QLNEINYEDHK	95.0%	51.6	22.2	5	0	0	2	1,402.66
									VASYGVKPR	95.0%	62.3	20.8	15	0	0	2	976.56
									VKDISEVVTPR	95.0%	85.0	20.9	13	2	0	2	1,242.71
									VSEADSSNADWVTK	95.0%	96.5	20.6	4	0	0	2	1,508.69
									VSVGGEKR	95.0%	66.3	23.7	12	0	0	2	831.47
									YGLVTYATYPK	95.0%	81.6	22.8	48	0	0	2	1,275.66
									YGQTIRPICLPCTEGTTR	95.0%	51.9	21.5	0	2	0	2	2,123.04
F-actin-capping protein subunit alpha-1	CAZA1_HUMAN	CAPZA1	32,905	100.00%	13	16	60	67.50%	DVQDSLTVSNEAQTAK	95.0%	110.0	23.4	6	0	0	2	1,705.82
									EASDPQPEEADGGLK	95.0%	82.9	19.7	6	0	0	2	1,542.69
									EGAAHAFAQYNMDQFTPVK	95.0%	30.3	20.6	0	1	0	2	2,140.98
									ESCDSALR	95.0%	34.6	18.3	2	0	0	2	937.41
									FITHAPPGEFNEVFNDVR	95.0%	56.0	21.7	4	2	0	2	2,089.01
									FTITPPTAQVVGVLK	95.0%	52.6	15.2	3	2	0	2	1,570.92
									IEGYEDQVLITEHGDLGNSR	95.0%	43.9	22.0	0	2	0	2	2,245.07
									IIENAENEYQTAISENYQTMSDTTFK	95.0%	69.9	18.1	0	4	0	2	3,056.37
									IQVHYYEDGNVQLVSHK	95.0%	40.0	22.3	0	10	1	2	2,029.01
									KEASDPQPEEADGGLK	95.0%	36.1	22.1	0	2	0	2	1,670.79
									LLLNNDNLLR	95.0%	76.8	19.2	12	0	0	2	1,197.70
									TIDGQQTIIACIESHQFQPK	95.0%	71.5	22.1	2	0	0	2	2,314.15
									VSDEEKVR	95.0%	39.4	24.0	1	0	0	2	961.50
Cystatin-C	CYTC_HUMAN	CST3	15,781	100.00%	9	12	120	62.30%	AFCSFQIYAVPWQGTMTLSK	95.0%	53.2	21.2	4	0	0	2	2,351.12
									ALDFAVGEYNK	95.0%	64.6	22.9	46	0	0	2	1,226.61
									ASNDMYHSR	95.0%	70.2	18.1	2	1	0	2	1,080.45
									KQIVAGVNYFLDVELGR	95.0%	109.0	18.1	17	18	0	2	1,921.05
									LVGGPMDASVEEEGVR	95.0%	90.6	21.7	6	0	0	2	1,644.79
									LVGGPMDASVEEEGVRR	95.0%	48.4	21.3	4	1	0	2	1,800.89
									QIVAGVNYFLDVELGR	95.0%	90.8	20.8	17	0	0	2	1,792.96
									RALDFAVGEYNK	95.0%	34.8	23.0	2	0	0	2	1,382.71
									TQPNLDNCPFHDQPHLK	95.0%	67.6	21.7	2	0	0	2	2,060.96
Insulin-like growth factor-binding protein 6	IBP6_HUMAN	IGFBP6	25,304	100.00%	6	9	898	26.70%	APAVAEENPK	95.0%	75.2	20.9	358	0	0	2	1,025.53
									GAQTLYVPNCDHR	95.0%	64.9	20.9	24	8	0	2	1,530.71
									HLDSVLQQLQTEVYR	95.0%	133.0	21.8	339	142	0	2	1,828.96
									NPGTSTTPSQPNSAGVQDTEMGPCR	95.0%	42.3	16.7	0	2	0	2	2,605.13

									NPGTSTTPSQPNSAGVQDTEMGPCRR	95.0%	40.2	19.3	0	4	0	2	2,761.23
									RHLDSVLQQLQTEVYR	95.0%	58.1	20.3	0	19	2	2	1,985.06
Small nuclear ribonucleoprotein Sm D2	SMD2_HUMAN	SNRPD2	13,509	100.00%	5	5	10	40.70%	EEEEFNTGPLSVLTQSVK	95.0%	58.0	22.6	2	0	0	2	2,006.99
									GDSVIVVLR	95.0%	51.2	20.0	2	0	0	2	957.57
									NNTQVLINCR	95.0%	53.2	23.4	2	0	0	2	1,231.62
									REEEEFNTGPLSVLTQSVK	95.0%	43.5	22.2	0	2	0	2	2,163.09
									SEMTPEELQK	95.0%	32.5	21.8	2	0	0	2	1,191.56
Small nuclear ribonucleoprotein-associated proteins B and B'	RSMB_HUMAN	SNRPB	24,593	100.00%	6	6	28	27.50%	GENLVSMTVEGPPPK	95.0%	109.0	22.1	8	0	0	2	1,570.78
									GENLVSMTVEGPPPKDTGIAR	95.0%	51.5	22.0	0	2	0	2	2,184.10
									GVGGPSQQVMTPQGR	95.0%	87.8	22.2	4	0	0	2	1,514.74
									MLQHIDYR	95.0%	43.0	22.6	2	0	0	2	1,091.53
									VLGLVLLR	95.0%	61.8	6.0	8	0	0	2	882.61
									VPLAGAAGGPGIGR	95.0%	83.7	20.6	4	0	0	2	1,192.68
Thrombospondin-3	TSP3_HUMAN	THBS3	104,182	100.00%	11	16	210	15.80%	AVAQPGLQLK	95.0%	44.8	16.4	25	0	0	2	1,024.62
									AVTSVSGPGEHLR	95.0%	61.9	22.4	27	2	0	2	1,309.69
									EDGKVHAVNLQQAGLADGR	95.0%	28.7	22.2	0	1	1	2	1,978.01
									GTQVSGVGIDYAR	95.0%	84.7	22.3	35	0	0	2	1,322.67
									IILGGSMAR	95.0%	51.6	23.0	4	0	0	2	933.52
									LGDQHAGLPALAPIPPAEVDGLEIR	95.0%	65.1	17.2	2	39	0	2	2,549.37
									LYEGPQLVADSGVIIDTSMR	95.0%	88.6	21.7	11	1	0	2	2,180.09
									NTIMECQVCGFHEQR	95.0%	29.9	16.9	0	1	0	2	1,924.81
									QGGVLFGLYSR	95.0%	79.0	21.3	32	0	0	2	1,196.64
									TALLTAGDIYLLSTFR	95.0%	77.9	19.6	19	0	0	2	1,754.97
									VHAVNLQQAGLADGR	95.0%	65.2	22.4	4	6	0	2	1,548.82
Nucleoprotein TPR	TPR_HUMAN	TPR	267,271	100.00%	24	24	61	14.90%	ASTALSNEQQAR	95.0%	58.7	22.6	8	0	0	2	1,275.63
									EGVQGPLNVSLSEEGK	95.0%	73.2	22.2	1	0	0	2	1,642.83
									FEVAQVESLR	95.0%	70.2	23.4	2	0	0	2	1,177.62
									FLADQQSEIDGLK	95.0%	68.8	23.5	2	0	0	2	1,463.74
									GAILSEEELAAMSPTAAAVAK	95.0%	75.7	21.8	2	0	0	2	2,046.04
									GQNLLLTNLQTIQGILER	95.0%	96.1	15.7	5	0	0	2	2,024.15
									ILLSQTTGVAIPLHASSLDDVSLASTPK	95.0%	49.8	14.8	0	3	0	2	2,834.55
									ISTQLDFASK	95.0%	36.9	22.3	1	0	0	2	1,109.58
									KLELDILPLQEANAELSEK	95.0%	52.1	17.9	0	8	0	2	2,153.17
									LDELQASDVSVK	95.0%	70.4	24.3	2	0	0	2	1,303.67
									LEQDLQQMQAK	95.0%	50.0	23.1	1	0	0	2	1,347.66
									LESALTELEQLR	95.0%	40.4	21.4	2	0	0	2	1,401.76
									LESALTELEQLRK	95.0%	50.4	20.3	1	0	0	2	1,529.85
									LQEQVTDLR	95.0%	70.3	23.2	4	0	0	2	1,101.59
									LSQELEYLTEDVK	95.0%	67.9	22.4	1	0	0	2	1,566.79
									NLDVQLLDTKR	95.0%	32.2	21.5	1	0	0	2	1,314.74
									NLQEQTVQLQSELSR	95.0%	97.0	21.9	2	0	0	2	1,772.91
									QEQIINTMTQDLR	95.0%	44.6	22.6	1	0	0	2	1,605.79
									RPSTSQTVSTPAPVPVIESTEAIEAK	95.0%	59.9	19.3	0	4	0	2	2,695.42
									SLESQVENLQK	95.0%	59.8	23.5	3	0	0	2	1,274.66
									SNASLTNNQNLIQSLKEDLNK	95.0%	36.2	20.3	0	2	0	2	2,344.21
									SQEQILEILR	95.0%	52.1	24.8	1	0	0	2	1,228.69
									TLSSVQNEVQEALQR	95.0%	94.9	22.2	3	0	0	2	1,701.88
									TSTSNVEQYQAMVTSLEESLNKEK	94.6%	25.6	21.0	0	1	0	2	2,732.29
Acidic leucine-rich nuclear phosphoprotein 32 family member B	AN32B_HUMAN	ANP32B	28,771	100.00%	4	5	23	31.50%	DISTLEPLKK	95.0%	35.5	20.8	2	0	0	2	1,143.66
									IFGGLDMLAEK	95.0%	64.8	22.4	9	0	0	2	1,209.62
									IFGGLDMLAEKLPNLTHLNLSGNK	95.0%	61.1	18.4	0	3	0	2	2,595.40

									IHLELR	95.0%	36.5	15.4	4	0	0	2	780.47
									KLELSENR	95.0%	41.1	23.6	3	0	0	2	988.54
									LKDISTLEPLK	95.0%	50.4	17.5	6	3	0	2	1,256.75
									LLPQLTYLDGYDR	95.0%	49.9	21.9	2	0	0	2	1,566.82
									LPNLTHLNLSGNK	95.0%	65.5	20.1	4	4	0	2	1,420.79
									SLDLFNCEVTNLNDYR	95.0%	91.4	20.6	3	0	0	2	1,972.91
NEDD8-activating enzyme E1 catalytic subunit	UBA3_HUMAN	UBA3	51,835	100.00%	4	4	8	12.50%	ELGLVDGQELAVADVTTPQTVLFK	95.0%	88.0	18.8	1	0	0	2	2,543.36
									IQDFNDTFYR	95.0%	50.1	20.6	3	0	0	2	1,318.61
									LQEVLDYLTNSASLQMK	95.0%	126.0	21.9	3	0	0	2	1,969.00
									TRPNLSK	94.6%	30.1	24.0	1	0	0	2	815.47
Histone deacetylase 2	HDAC2_HUMAN	HDAC2	55,348	100.00%	2	2	3	5.94%	TFNLPLLMLGGGGYTIR	95.0%	37.3	20.7	1	0	0	2	1,838.98
									YGEYFPGTGDLR	95.0%	34.0	20.2	2	0	0	2	1,374.63
Nuclear autoantigenic sperm protein	NASP_HUMAN	NASP	85,218	100.00%	3	3	15	6.09%	EAQLYAAQAHLK	95.0%	39.7	22.0	2	0	0	2	1,342.71
									EIEELKELLPEIR	95.0%	47.3	18.8	4	0	0	2	1,610.90
									HLVMGDIPAAVNAFQEAASLLGK	95.0%	43.2	20.9	0	9	0	2	2,368.23
Puromycin-sensitive aminopeptidase	PSA_HUMAN	NPEPPS	103,261	100.00%	32	39	258	41.90%	AFFESHPAPSAER	95.0%	46.7	21.1	4	2	0	2	1,445.68
									AGIISTVEVLK	95.0%	75.4	18.2	24	0	0	2	1,129.68
									AQELDALDNSHPIEVSVGHPSEVDEIFDAISYSK	95.0%	82.9	21.0	0	2	1	2	3,711.77
									ATFDISLVVPK	95.0%	56.6	19.1	4	0	0	2	1,189.68
									ATLEEAR	95.0%	44.9	24.9	4	0	0	2	789.41
									DAESIHQYLLQR	95.0%	63.2	21.4	7	2	0	2	1,472.75
									DLSLPPVDR	95.0%	34.0	20.0	2	0	0	2	1,011.55
									DVFSPIGER	95.0%	34.8	22.7	1	0	0	2	1,019.52
									DYFNVPYPLPK	95.0%	65.2	22.3	11	0	0	2	1,352.69
									ETALLIDPK	95.0%	50.2	21.7	6	0	0	2	999.57
									FALEVAAK	95.0%	36.9	20.1	4	0	0	2	848.49
									FIKDNWEELYNR	95.0%	27.8	22.4	0	2	0	2	1,626.79
									FKDHVEGK	95.0%	35.2	23.3	1	0	0	2	959.50
									HGDGTTLDIMLK	95.0%	53.1	22.5	4	0	0	2	1,316.65
									IDFVGELNDK	95.0%	62.0	23.2	5	0	0	2	1,149.58
									IDFVGELNDKMK	95.0%	61.8	23.0	1	2	0	2	1,424.71
									KPYPDDENLVEVK	95.0%	73.8	23.1	5	2	0	2	1,545.78
									LGLQNDLFSLAR	95.0%	92.7	21.6	12	0	0	2	1,346.74
									LGWDPKPGEGHLDALLR	95.0%	32.9	21.3	0	2	1	2	1,873.99
									LNLGTVGFYR	95.0%	69.0	21.8	15	0	0	2	1,139.62
									LSVEGFAVDK	95.0%	55.3	23.3	8	0	0	2	1,064.56
									QMGFPLIYVEAEQVEDDR	95.0%	84.8	21.1	2	2	0	2	2,155.00
									QMGFPLIYVEAEQVEDDRLLR	95.0%	34.5	21.0	0	4	0	2	2,537.27
									SPVYLTVLK	95.0%	54.2	14.0	10	0	0	2	1,019.61
									TQYSSAMLESLLPGIR	95.0%	64.6	22.4	13	0	0	2	1,781.91
									VALSNMNVIDR	95.0%	69.4	23.6	8	0	0	2	1,247.64
									VLGATLLPDLIQK	95.0%	82.0	11.8	32	0	0	2	1,380.85
									VLTFALSEEVRPQDTVSVIGGVAGGSK	95.0%	60.5	17.4	0	28	0	2	2,716.45
									VTLSFPSTLQTGTGTLK	95.0%	70.7	17.9	10	0	0	2	1,750.96
									YAAVTQFEATDAR	95.0%	94.9	21.7	8	0	0	2	1,442.69
									YQGGFLISR	95.0%	62.7	21.0	6	0	0	2	1,040.55
									YTTPSGEVR	95.0%	41.3	22.2	1	0	0	2	1,009.50
Complement component C7	CO7_HUMAN	C7	93,499	99.50%	2	2	3	4.39%	DGFVQDEGTMFPVGK	95.0%	36.6	20.3	1	0	0	2	1,642.74
									GGGAGFISGLSYLELDNPAGNK	95.0%	78.6	21.5	2	0	0	2	2,137.06
60S ribosomal protein L3	RL3_HUMAN	RPL3	46,092	100.00%	5	5	6	17.40%	AGMTHIVR	95.0%	34.5	21.4	1	0	0	2	900.47
									HGSLGFLPR	95.0%	56.1	20.8	2	0	0	2	983.54

									LEQQVPVNQVFGQDEMIDVIGVTK	94.7%	25.6	20.5	0	1	0	2	2,702.37
									SINPLGGFVHYGEVTNDFVMLK	95.0%	28.7	22.2	0	1	0	2	2,453.22
									VAFSVAR	95.0%	30.7	21.1	1	0	0	2	749.43
Trans-Golgi network integral membrane protein 2	TGON2_HUMAN	TGOLN2	51,095	100.00%	3	3	22	9.38%	DHSKPISNPSDNKELPK	95.0%	22.3	21.8	0	0	2	2	1,905.97
									SGAEAQTPEDSPNR	95.0%	63.3	19.5	14	0	0	2	1,458.65
									SSAEAQTPEDTPNK	95.0%	74.1	20.6	6	0	0	2	1,474.67
Aflatoxin B1 aldehyde reductase member 2	ARK72_HUMAN	AKR7A2	39,571	100.00%	3	3	7	12.80%	ALQAAYGASAPSVTSAALR	95.0%	88.2	20.8	2	0	0	2	1,804.96
									FYAYNPLAGGLLTGK	95.0%	56.7	21.2	3	0	0	2	1,584.84
									VASVLGTMEMGR	95.0%	61.4	22.1	2	0	0	2	1,282.61
Neurosecretory protein VGF	VGF_HUMAN	VGF	67,240	100.00%	14	19	150	29.80%	AYQGVAAPFPK	95.0%	78.7	22.4	20	0	0	2	1,148.61
									ESAREEEEAEQER	95.0%	64.1	17.2	6	13	0	2	1,591.68
									FGEGVSSPK	95.0%	46.3	23.4	5	0	0	2	907.45
									GLQEAAEER	95.0%	46.6	20.0	3	0	0	2	1,002.49
									LADLASDLLLQYLLQGGAR	95.0%	89.7	16.0	19	15	0	2	2,030.13
									LHLPADDVVSIIEEVEEK	95.0%	69.7	21.7	6	23	0	2	2,035.06
									LHLPADDVVSIIEEVEEKR	95.0%	28.7	19.8	0	1	1	2	2,191.16
									LQEQEELENYIEHVLLR	95.0%	36.2	22.0	0	4	0	2	2,155.10
									NSEPQDEGELFQGVDPR	95.0%	85.8	19.8	13	0	0	2	1,916.86
									QQETAAAETETR	95.0%	32.8	21.3	2	0	0	2	1,334.62
									RPESALLGGSEAGER	95.0%	70.6	22.3	4	6	0	2	1,528.77
									THLGEALAPLSK	95.0%	54.7	20.7	5	0	0	2	1,236.70
									VGEEDEEAAEAEAEAEEAER	95.0%	126.0	14.9	2	0	0	2	2,162.89
									VNLESPGPER	95.0%	36.4	22.9	2	0	0	2	1,097.56
Transitional endoplasmic reticulum ATPase	TERA_HUMAN	VCP	89,307	100.00%	34	40	278	44.30%	AFEEAEK	95.0%	30.9	21.0	1	0	0	2	823.38
									AIANECQANFISIK	95.0%	83.4	22.6	2	0	0	2	1,578.80
									AIGVKPPR	95.0%	42.1	14.6	1	0	0	2	837.53
									AVANETGAFFFLINGPEIMSK	95.0%	92.5	21.7	10	2	0	2	2,272.13
									DVDLEFLAK	95.0%	63.6	23.0	10	0	0	2	1,049.55
									ELQELVQYPVEHPDK	95.0%	83.2	22.2	4	2	0	2	1,823.92
									ESIESEIRR	95.0%	32.3	23.1	1	0	0	2	1,118.58
									EVDIGIPDATGR	95.0%	65.7	21.9	4	0	0	2	1,242.63
									GDDLSTAILK	95.0%	59.2	23.2	2	0	0	2	1,032.56
									GDIFLVR	95.0%	48.1	20.5	7	0	0	2	819.47
									GGNIGDGGGAADR	95.0%	99.8	19.6	15	0	0	2	1,116.50
									GILLYGPPGTGK	95.0%	65.4	21.2	15	0	0	2	1,172.67
									GVLFYGPPGCGK	95.0%	43.3	22.9	2	0	0	2	1,251.62
									IVSQLLTLMDGLK	95.0%	112.0	17.9	20	0	0	2	1,446.82
									KGDIFLVR	95.0%	48.8	17.6	6	0	0	2	947.57
									KYEMFAQTLQQSR	95.0%	92.6	22.2	4	0	0	2	1,645.80
									LAGESESNLR	95.0%	57.0	23.0	3	0	0	2	1,075.54
									LAGESESNLRK	95.0%	64.9	23.6	10	1	0	2	1,203.63
									LDQLIYIPLPDEK	95.0%	77.6	20.2	20	0	0	2	1,556.86
									LGDVISIQPCPDVK	95.0%	57.0	22.0	1	0	0	2	1,540.80
									LIVDEAINEDNSVVSLSQPK	95.0%	115.0	21.0	13	2	0	2	2,170.12
									MDELQLFR	95.0%	62.4	21.7	4	0	0	2	1,067.52
									NAPAIIFIDELDAIAPK	95.0%	106.0	18.6	64	10	0	2	1,811.00
									NAPAIIFIDELDAIAPKR	95.0%	57.3	17.9	2	0	0	2	1,967.10
									NVFIIGATNRPDIIDPAILRPGR	95.0%	52.2	13.2	0	4	0	2	2,518.43
									QAAPCVLFFDELDSIAK	95.0%	58.8	21.7	8	2	0	2	1,923.95
									QTNPSAMEVEEDDPVPEIR	95.0%	78.2	20.3	4	0	0	2	2,171.98
									QTNPSAMEVEEDDPVPEIRR	95.0%	40.5	21.0	0	2	0	2	2,328.08

									RIVSQLLTLMDGLK	95.0%	54.0	16.0	2	0	0	2	1,602.93
									SVSDNDIR	95.0%	34.3	22.5	2	0	0	2	905.43
									VINQILTEMDGMSTK	95.0%	88.8	22.1	9	0	0	2	1,711.82
									VINQILTEMDGMSTKK	95.0%	52.5	22.6	4	0	0	2	1,839.92
									WALSQSNPSALR	95.0%	80.3	22.8	2	0	0	2	1,329.69
									YEMFAQTLQQSR	95.0%	42.6	20.9	1	0	0	2	1,517.71
Growth-regulated alpha protein	GROA_HUMAN	CXCL1	11,284	100.00%	4	6	10	38.30%	ACLNPASPIVK	95.0%	30.9	20.9	1	0	0	2	1,169.64
									CQCLQTLQGIHPK	95.0%	44.3	22.5	2	2	0	2	1,582.78
									KACLNPASPIVK	95.0%	62.9	19.3	2	0	0	2	1,297.73
									SPGPHCAQTEVIATLK	95.0%	44.4	22.7	2	1	0	2	1,708.87
Peptidyl-prolyl cis-trans isomerase FKBP4	FKBP4_HUMAN	FKBP4	51,788	100.00%	28	31	134	68.00%	AEASSGDHPTDTEMKEEQK	95.0%	59.0	16.9	4	25	0	2	2,089.90
									ALELDSNNEK	95.0%	42.6	21.1	4	0	0	2	1,132.55
									ATESGAQSAPLPMEGVDISPK	95.0%	83.8	21.6	10	0	0	2	2,101.01
									DKFSFDLGK	95.0%	33.0	21.8	1	0	0	2	1,056.54
									EGTGTEMPMIGDR	95.0%	53.7	17.2	15	0	0	2	1,425.60
									FDSSLDR	94.8%	30.2	20.9	1	0	0	2	839.39
									FDSSLDRK	95.0%	31.4	22.3	2	0	0	2	967.49
									FEIGEGENLDLPYGLER	95.0%	102.0	22.1	18	0	0	2	1,950.95
									FQIPPNAELK	94.8%	30.3	22.0	2	0	0	2	1,156.64
									FSFDLGK	95.0%	30.9	20.9	1	0	0	2	813.41
									FSFDLGKGEVIK	95.0%	38.2	21.2	2	0	0	2	1,339.73
									GEAHLAVNDFELAR	95.0%	42.1	22.1	0	2	0	2	1,541.77
									GEDLTEEEDGGIIR	95.0%	35.0	21.0	1	0	0	2	1,532.71
									GEGYAKPNEGAIVEVALEGYYKDK	95.0%	30.1	21.9	0	1	0	2	2,600.29
									GEHSIVYLKPSYAFGSVGK	95.0%	49.2	20.9	2	3	5	2	2,039.06
									LAEEENKAK	95.0%	37.6	22.7	1	0	0	2	1,031.54
									LASHLNLAMCHLK	95.0%	36.2	20.8	0	1	0	2	1,523.78
									LQAFSAAIESCNK	95.0%	90.0	22.2	2	0	0	2	1,438.70
									LYANMFER	95.0%	49.0	19.4	4	0	0	2	1,059.49
									MEKGEHSIVYLKPSYAFGSVGK	95.0%	32.5	21.1	0	0	2	2	2,443.23
									QDEGVLK	95.0%	36.0	23.0	2	0	0	2	788.42
									RGEAHLAVNDFELAR	95.0%	76.5	22.4	0	6	0	2	1,697.87
									SNTAGSQSQVETEA	95.0%	97.7	17.3	7	0	0	2	1,408.62
									TAEEMKATESGAQSAPLPMEGVDISPK	95.0%	49.0	20.6	0	3	0	1	2,806.31
									TQLAVCQQR	95.0%	41.5	23.1	2	0	0	2	1,103.56
									VFVHYTGWLLDGTK	95.0%	66.1	22.7	0	2	0	2	1,635.85
									VGEVCHITCKPEYAYGSAGSPPK	95.0%	25.3	20.5	0	0	1	2	2,507.17
									VLQLYPNNK	95.0%	45.0	22.5	2	0	0	2	1,088.61
Melanoma-derived growth regulatory protein	MIA_HUMAN	MIA	14,491	99.50%	2	2	3	14.50%	EDQTLKPGK	95.0%	32.0	21.5	2	0	0	2	1,015.54
									LGYFPSSIVR	95.0%	39.1	20.3	1	0	0	2	1,138.63
Eukaryotic initiation factor 4A-I	IF4A1_HUMAN	EIF4A1	46,137	100.00%	15	20	77	43.10%	DFTVSAMHGDMDQK	95.0%	47.9	14.1	3	0	0	2	1,613.66
									EELTLEGIR	95.0%	42.6	23.1	2	0	0	2	1,059.57
									GFKDQIYDIFQK	95.0%	78.1	22.6	4	1	0	2	1,501.77
									GIDVQQVSLVINYDLPTNR	95.0%	77.2	20.3	2	0	0	2	2,144.14
									GIYAYGFEKPSAIQQR	95.0%	93.3	21.2	4	4	0	2	1,827.94
									GVAINMVTEEDKR	95.0%	42.3	23.4	2	0	0	2	1,477.73
									GYDVIAQAQSGTGK	95.0%	77.8	22.7	2	0	0	2	1,394.69
									KEELTLEGIR	95.0%	45.7	22.7	5	2	0	2	1,187.66
									LNSNTQVVLLSATMPSDVLEVTK	95.0%	89.0	19.5	4	1	0	2	2,475.30
									LNSNTQVVLLSATMPSDVLEVTKK	95.0%	78.2	18.5	0	3	0	2	2,603.40
									LQMEAPHIIVGTPGR	95.0%	60.6	21.7	4	5	0	2	1,634.87

									MFVLDEADEMLSR	95.0%	95.8	19.4	8	0	0	2	1,587.70
									QFYINVER	95.0%	34.1	22.3	1	0	0	2	1,068.55
									VFDMLNR	95.0%	31.5	22.0	1	0	0	2	910.45
									VLITTDLLAR	95.0%	67.3	17.6	19	0	0	2	1,114.68
SAP domain-containing ribonucleoprotein	SARNP_HUMAN	SARNP	23,653	100.00%	4	4	7	22.90%	FGISSVPTK	95.0%	36.4	20.9	1	0	0	2	935.52
									FGIVTSSAGTGTTEDTEAK	95.0%	93.5	21.5	2	0	0	2	1,871.89
									FGLNVSSISR	95.0%	58.8	22.0	2	0	0	2	1,079.58
									ITSEIPQTER	95.0%	50.4	22.8	2	0	0	2	1,173.61
Ribosome biogenesis protein WDR12	WDR12_HUMAN	WDR12	47,691	99.50%	2	2	3	8.98%	SIMTIVGHTDVVK	95.0%	46.3	21.7	2	0	0	2	1,415.76
									YAVDDVPFSIPAASEIADLSNIINK	95.0%	26.8	20.2	0	1	0	2	2,662.36
Eukaryotic translation initiation factor 5A-1	IF5A1_HUMAN	EIF5A	16,815	100.00%	7	9	26	48.10%	KYEDICPSTHNMDVPNIK	95.0%	38.6	21.6	0	4	0	2	2,161.01
									LPEGDLGKEIEQK	95.0%	67.2	21.4	2	0	0	2	1,455.77
									NDFQLIGIQDGYLSLLQDSGEVR	95.0%	80.6	21.2	2	2	0	2	2,580.30
									NGFVVLK	95.0%	49.2	19.9	4	0	0	2	776.47
									RNDFQLIGIQDGYLSLLQDSGEVR	95.0%	35.8	21.0	0	1	0	2	2,736.40
									VHLVGIDIFTGK	95.0%	61.1	19.2	5	2	0	2	1,298.75
									VHLVGIDIFTGKK	95.0%	40.5	15.8	0	4	0	2	1,426.84
Tripeptidyl-peptidase 1	TPP1_HUMAN	TPP1	61,230	100.00%	12	18	114	35.90%	ADPEEELSLTFALR	95.0%	77.1	23.3	25	0	0	2	1,590.80
									AYPDVAALSDGYWVVSNR	95.0%	94.5	22.0	3	2	0	2	1,982.96
									ILSGRPPLGFLNPR	95.0%	48.8	13.8	1	8	0	2	1,536.90
									LFGGNFAHQASVAR	95.0%	109.0	23.1	4	4	0	2	1,474.76
									LSELVQAVSDPSSPQYGK	95.0%	110.0	22.1	14	0	0	2	1,904.96
									LYQQHGAGLFDVTR	95.0%	81.7	22.6	4	7	0	2	1,604.82
									QAELLLPGAEFHHYVGGPTETHVVR	95.0%	63.0	20.5	0	4	5	2	2,757.41
									QRPEPQVTGTVGLHLGVTPSVIR	95.0%	48.7	15.8	0	3	0	2	2,441.36
									SPHPYQLPQALAPHVDFVGGLHR	95.0%	46.1	19.6	0	2	10	2	2,536.32
									VNTELMK	95.0%	45.9	23.8	4	0	0	2	850.43
									WLLAAGAQK	95.0%	37.2	22.5	1	0	0	2	957.55
									YLTLENVADLVRPSPLTLHTVQK	95.0%	64.4	14.1	0	13	0	2	2,607.45
6-phosphogluconate dehydrogenase, decarboxylating	6PGD_HUMAN	PGD	53,124	100.00%	19	24	108	37.30%	AGQAVDDFIEK	95.0%	53.9	23.1	7	0	0	2	1,192.59
									CLSSLKDER	95.0%	30.5	22.8	1	0	0	2	1,107.55
									DAFDRNPELQNLLLDDFFK	95.0%	36.5	22.1	0	2	0	2	2,310.14
									FQDTDGK	95.0%	31.1	19.3	1	0	0	2	810.36
									FQDTDGKHLLPK	95.0%	60.7	20.8	2	1	0	2	1,398.74
									FQFDGDKK	95.0%	39.4	21.1	1	0	0	2	984.48
									GILFVGSGVSGGEEGAR	95.0%	106.0	23.0	31	0	0	2	1,591.81
									HEMLPASLIQAQR	95.0%	56.1	22.1	3	1	0	2	1,509.78
									IISYAQGFMLLR	95.0%	79.1	22.3	11	0	0	2	1,427.77
									LVPLLDTGDIIIDGGNSEYR	95.0%	141.0	21.5	15	2	0	2	2,160.12
									LVPLLDTGDIIIDGGNSEYRDTTR	95.0%	45.2	20.5	1	1	0	2	2,633.34
									NPELQNLLLDDFFK	95.0%	95.4	22.4	2	0	0	2	1,705.88
									SFLEDIR	95.0%	36.7	22.7	2	0	0	2	879.46
									SFLEDIRK	95.0%	32.1	21.5	2	0	0	2	1,007.55
									TIFQGIAAK	95.0%	40.7	18.9	4	0	0	2	948.55
									TVSKVDDFLANEAK	95.0%	80.2	22.6	3	2	0	2	1,536.79
									VDDFLANEAK	95.0%	76.4	23.9	7	0	0	2	1,121.55
									VVGAQSLKEMVSK	95.0%	58.9	21.0	2	0	0	2	1,391.76
									YGPSLMPGGNK	95.0%	75.5	22.3	4	0	0	2	1,136.54
Chloride intracellular channel protein 4	CLIC4_HUMAN	CLIC4	28,756	100.00%	4	5	10	22.90%	HPESNTAGMDIFAK	95.0%	35.9	20.5	1	0	0	2	1,533.70
									KPADLQNLAPGTHPPFITFNSEVK	95.0%	28.6	20.3	0	1	0	2	2,621.37
									NSRPEANEALER	95.0%	53.1	21.9	6	1	0	2	1,385.68

									YLTNAYSR	95.0%	32.3	20.5	1	0	0	2	987.49
Serpin H1	SERPH_HUMAN	SERPINH1	46,424	100.00%	13	16	47	44.00%	AATLAER	95.0%	45.7	25.1	2	0	0	2	731.41
									AVAISLPK	95.0%	34.7	15.8	2	0	0	2	798.51
									AVLSAEQLRDEEVHAGLGELLR	95.0%	46.6	19.1	0	1	0	2	2,405.28
									DQAVENILVSPVVVASSLGLVSLGGK	95.0%	82.8	14.1	4	6	0	2	2,551.43
									DTQSGSLLFIGR	95.0%	64.5	23.0	4	0	0	2	1,293.68
									GVVEVTHDLQK	95.0%	36.9	21.1	2	0	0	2	1,224.66
									HLAGLGLTEAIDKNK	95.0%	64.2	18.5	0	4	0	2	1,579.88
									LFYADHPFIFLVR	95.0%	63.9	19.4	2	1	0	2	1,637.88
									LQIVEMPLAHK	95.0%	52.5	20.4	3	0	0	2	1,294.72
									LSSLIILMPHHVEPLER	95.0%	44.6	17.4	0	5	2	2	2,000.10
									LYGPSSVSFADDFVR	95.0%	85.4	21.8	4	0	0	2	1,659.80
									SAGLAFSLYQAMAK	95.0%	81.2	22.3	3	0	0	2	1,473.74
									TGLYNYYDDEKEK	95.0%	70.1	20.9	2	0	0	2	1,637.73
Phosphoglycerate mutase 1	PGAM1_HUMAN	PGAM1	28,787	100.00%	13	20	92	64.60%	ALPFWNEEIVPQIK	95.0%	69.5	20.6	13	0	0	2	1,683.91
									AMEAVAAQGK	95.0%	72.1	23.2	5	0	0	2	991.49
									FSGWYDADLSPAGHEEAK	95.0%	66.4	19.7	2	1	0	2	1,979.88
									HGESAWNLENR	95.0%	28.7	21.2	0	1	0	2	1,312.60
									HLEGLSEEAIMELNLPTGIPIVYELDK	95.0%	61.1	19.8	0	7	0	2	3,039.56
									HYGGLTGLNK	95.0%	44.6	23.1	9	0	0	2	1,059.56
									KAMEAVAAQGK	95.0%	74.6	23.4	4	0	0	2	1,119.58
									NLKPIKPMQFLGDEETVR	95.0%	49.7	20.3	2	6	5	2	2,131.12
									RSYDVPPPPMEPDHPFYSNISK	95.0%	40.4	20.6	0	2	3	2	2,589.21
									RVLIAAHGNSLR	95.0%	51.3	14.8	0	3	0	2	1,306.77
									SYDVPPPPMEPDHPFYSNISK	95.0%	66.1	19.8	4	2	0	2	2,433.11
									VLIAAHGNSLR	95.0%	60.3	17.0	11	7	0	2	1,150.67
									YADLTEDQLPSCESLKDTIAR	95.0%	69.6	21.0	2	3	0	2	2,425.16
cAMP-dependent protein kinase catalytic subunit alpha	KAPCA_HUMAN	PRKACA	40,573	100.00%	4	4	8	14.20%	DNSNLYMVMEYVPGGEMFSHLR	95.0%	30.8	17.6	0	1	0	2	2,637.14
									FSEPHAR	95.0%	39.9	19.9	2	0	0	2	843.41
									ILQAVNFPFLVK	95.0%	61.9	15.3	4	0	0	2	1,388.83
									KVEAPFIPK	95.0%	32.4	20.7	1	0	0	2	1,028.61
Ras-related protein Rab-2A	RAB2A_HUMAN	RAB2A	23,528	100.00%	4	4	9	25.50%	GAAGALLVYDITR	95.0%	52.0	21.0	2	0	0	2	1,319.73
									IQEGVFDINNEANGIK	95.0%	82.3	22.7	2	0	0	2	1,760.88
									TASNVEEAFINTAK	95.0%	104.0	23.5	3	0	0	2	1,494.74
									YIIIGDTGVGK	95.0%	52.0	21.6	2	0	0	2	1,135.64
Tyrosyl-tRNA synthetase, cytoplasmic	SYYC_HUMAN	YARS	59,127	100.00%	7	7	14	21.20%	AMLESIGVPLEK	95.0%	74.1	23.1	4	0	0	2	1,302.70
									IITVEKHPDADSLYVEK	95.0%	26.0	21.2	0	1	0	2	1,957.03
									NLQEVLGEEK	95.0%	31.8	23.8	1	0	0	2	1,158.60
									NSEPEEVIPSR	95.0%	40.7	21.8	3	0	0	2	1,256.61
									QVEHPLLSGLLYPGLQALDEEYLK	95.0%	26.3	20.7	0	1	0	2	2,725.45
									QVEPLDPPAGSAPGEHVFVK	95.0%	35.8	22.2	0	2	0	2	2,074.06
									TVVSGLVQFVPKEELQDR	95.0%	46.8	19.0	0	2	0	2	2,044.11
Ferritin light chain	FRIL_HUMAN	FTL	20,003	100.00%	5	6	14	25.10%	AAMALEK	95.0%	39.9	23.3	2	0	0	2	749.39
									KLNQALLDLHALGSAR	95.0%	25.1	14.8	0	0	2	2	1,719.99
									LGGPEAGLGEYLFER	95.0%	67.6	22.8	5	0	0	2	1,607.81
									LNQALLDLHALGSAR	95.0%	79.0	17.2	2	2	0	2	1,591.89
									LTLKHD	94.6%	30.1	18.1	1	0	0	2	726.42
Alpha-1-antitrypsin	A1AT_HUMAN	SERPINA1	46,720	100.00%	11	13	34	36.40%	FLENEDRR	95.0%	27.0	23.6	0	1	0	2	1,078.53
									GTEAAGAMFLEAIPMSIPPEVK	95.0%	81.3	22.1	1	0	0	2	2,291.13
									ITPNLAEFAFSLYR	95.0%	97.1	21.6	8	0	0	2	1,641.86
									LQHLENELTHDIITK	95.0%	41.9	21.5	0	2	2	2	1,803.96

									LSITGTYDLK	95.0%	49.8	21.1	3	0	0	2	1,110.60
									QINDYVEK	95.0%	37.9	22.2	2	0	0	2	1,008.50
									SASLHLPK	95.0%	31.7	16.5	1	0	0	2	852.49
									SVLGQLGITK	95.0%	61.8	17.7	4	0	0	2	1,015.62
									TDTSHHDQDHPTFNK	95.0%	44.4	19.7	0	2	0	2	1,779.77
									TLNQPDSQLQLTTGNGLFLSEGLK	95.0%	78.4	19.3	2	2	0	2	2,574.34
									VFSNGADLSGVTEEAPLK	95.0%	111.0	22.3	4	0	0	2	1,833.92
Proteasome subunit alpha type-2	PSA2_HUMAN	PSMA2	25,881	100.00%	9	14	55	53.80%	AANGVVLATEKK	95.0%	43.7	21.0	2	0	0	2	1,200.70
									GYSFSLTTFSPSGK	95.0%	105.0	22.7	2	0	0	2	1,478.72
									HIGLVYSGMGPDYR	95.0%	61.3	22.3	4	4	0	2	1,580.75
									KLAQQYYLVYQEPIPTAQLVQR	95.0%	73.3	15.7	0	1	0	2	2,649.44
									LAQQYYLVYQEPIPTAQLVQR	95.0%	62.6	18.9	2	2	0	2	2,521.35
									LTPTEVKDYLAAIA	95.0%	34.1	20.3	1	0	0	2	1,504.83
									LVQIEYALAAVAGGAPSVGIK	95.0%	71.8	14.0	3	13	0	2	2,027.15
									SVHKVEPITK	95.0%	45.2	17.3	5	5	0	2	1,137.66
									YNEDLELEDAIHTAILTLK	95.0%	140.0	21.0	4	7	0	2	2,201.13
Eukaryotic translation initiation factor 4 gamma 1	IF4G1_HUMAN	EIF4G1	175,476	100.00%	6	6	33	5.75%	AALSEEELEKK	95.0%	40.2	23.8	4	0	0	2	1,246.65
									EAALPPVSPLK	95.0%	56.8	17.6	4	0	0	2	1,121.66
									EAVGDLLDAFK	95.0%	61.6	23.2	8	0	0	2	1,177.61
									EFLPEGQDIGAFVAEQK	95.0%	80.2	22.0	9	0	0	2	1,877.93
									GSSGGSGAKPSDAASEAARPATSTLNR	95.0%	31.0	21.5	0	1	0	2	2,503.21
									VEYTLGEESEAPGQR	95.0%	120.0	21.3	7	0	0	2	1,664.78
Glutathione synthetase	GSHB_HUMAN	GSS	52,368	100.00%	17	18	65	40.70%	AIEHADGGVAAGVAVLDNPYPV	95.0%	86.7	22.0	4	0	0	2	2,135.08
									AIENELLAR	95.0%	63.9	20.8	10	0	0	2	1,028.57
									ALAEGVLLR	95.0%	75.0	16.5	9	0	0	2	941.58
									ASYILMEK	95.0%	38.0	21.4	2	0	0	2	970.49
									AWELYGSPNALVLLIAQEK	95.0%	50.8	19.6	1	0	0	2	2,115.15
									DGYMPR	95.0%	30.7	17.6	1	0	0	2	754.32
									EGGGNNLYGEEMVQALK	95.0%	74.3	21.9	6	0	0	2	1,808.85
									EGIAQTVFLGLNR	95.0%	92.1	20.7	7	0	0	2	1,417.78
									ILSNNPSK	95.0%	43.0	22.3	2	0	0	2	872.48
									KVQQELSRPGMLEMLLPGQPEAVAR	95.0%	41.9	19.3	0	1	0	2	2,809.47
									QDDFTAR	95.0%	37.4	21.0	2	0	0	2	852.39
									QIEINTISASFGGLASR	95.0%	140.0	21.6	6	1	0	2	1,763.93
									QQLEELAR	95.0%	46.9	21.4	5	0	0	2	986.53
									QYSLQNWEAR	95.0%	38.9	22.5	1	0	0	2	1,294.62
									SADGSPALK	95.0%	41.3	22.0	4	0	0	2	845.44
									TLVMNK	95.0%	39.5	22.9	1	0	0	2	721.39
									VQQELSRPGMLEMLLPGQPEAVAR	95.0%	41.4	20.8	0	2	0	2	2,681.38
N-acetyl-D-glucosamine kinase	NAGK_HUMAN	NAGK	37,359	99.50%	2	2	3	10.80%	HIVAVLPEIDPVLFQGK	95.0%	40.9	12.3	0	2	0	2	1,875.07
									IVFDSIDNLEAAPHDIGYVK	95.0%	39.4	21.5	0	1	0	2	2,216.12
Vitamin K-dependent protein S	PROS_HUMAN	PROS1	75,105	100.00%	11	13	68	17.30%	EAVMDINKPGPLFKPENGLLETK	95.0%	41.0	18.8	0	2	0	2	2,556.34
									FRLPEISR	95.0%	38.6	22.4	7	0	0	2	1,017.58
									FSAEFDFR	95.0%	52.5	19.9	7	0	0	2	1,018.46
									IETISHEDLQR	95.0%	37.7	22.4	1	0	0	2	1,340.68
									KVESELIKPINPR	95.0%	54.1	15.6	1	10	4	2	1,522.90
									NNLELSTPLK	95.0%	58.0	22.4	1	0	0	2	1,128.63
									NNLELSTPLKIETISHEDLQR	95.0%	53.9	20.8	0	9	0	2	2,450.29
									QSTNAYPDLR	95.0%	77.8	23.1	2	0	0	2	1,164.57
									SFQTGLFTAAR	95.0%	77.2	21.3	11	0	0	2	1,198.62
									SQDILLSVENTVIYR	95.0%	84.8	20.0	9	0	0	2	1,749.94

									VYFAGFPR	95.0%	40.2	20.1	4	0	0	2	956.50
Exostosin-1	EXT1_HUMAN	EXT1	86,239	100.00%	2	3	5	4.96%	ASISTENFRPNFDVSIPLFSK	95.0%	44.5	20.8	0	1	0	2	2,369.21
									IAESYQNILAAIEGSR	95.0%	74.7	22.2	3	1	0	2	1,734.90
MARCKS-related protein	MRP_HUMAN	MARCKSL1	19,511	100.00%	2	2	25	14.40%	AAATPESQEPQAK	95.0%	59.5	22.7	20	0	0	2	1,327.65
									GDVTAEEAAGASPAK	95.0%	60.8	22.4	5	0	0	2	1,373.66
Coatomer subunit epsilon	COPE_HUMAN	COPE	34,465	100.00%	8	8	18	33.10%	APPAPGPASGGSGEVDELFDVK	95.0%	109.0	22.3	4	0	0	1	2,097.01
									AYLAQR	95.0%	38.2	21.0	1	0	0	2	721.40
									DSIVAELDREMSR	95.0%	53.6	21.9	2	0	0	2	1,536.73
									ENDFDRLVLQYAPSA	95.0%	34.1	22.1	2	0	0	2	1,737.84
									FGVVLDEIKPSSAPELQAVR	95.0%	62.8	18.3	0	2	0	2	2,155.18
									KFGVVLDEIKPSSAPELQAVR	95.0%	55.6	16.1	0	4	0	2	2,283.27
									LQDAYYIFQEMADK	95.0%	80.8	22.2	2	0	0	2	1,750.80
									MFADYLAHESR	95.0%	32.3	20.1	0	1	0	2	1,355.61
Cleavage and polyadenylation specificity factor subunit 5	CPSF5_HUMAN	NUDT21	26,210	100.00%	6	8	14	27.30%	GVTQFGNK	95.0%	35.8	23.8	1	0	0	2	850.44
									LFLVQLQEK	95.0%	53.8	18.8	1	0	0	2	1,117.66
									LPGGELNPGEDEVEGLK	95.0%	54.5	22.3	2	0	0	2	1,752.87
									LPGGELNPGEDEVEGLKR	95.0%	81.0	22.6	1	2	0	2	1,908.97
									TINLYPLTNYTFGTK	95.0%	65.1	22.4	3	0	0	2	1,745.91
									YIQQTKPLTLER	95.0%	36.8	19.5	2	2	0	2	1,489.84
Signal transducer and activator of transcription 3	STAT3_HUMAN	STAT3	88,052	100.00%	3	3	6	7.40%	GLSIEQLTTLAEK	95.0%	106.0	20.5	2	0	0	2	1,402.78
									LLQTAATAAQQGGQANHPTAAVVTEK	95.0%	74.6	20.0	0	2	0	2	2,576.34
									SIVSELAGLLSAMEYVQK	95.0%	55.2	20.5	2	0	0	2	1,954.02
40S ribosomal protein S7	RS7_HUMAN	RPS7	22,110	100.00%	10	11	29	47.40%	AIIIFVPVPQLK	95.0%	65.2	9.5	6	0	0	2	1,337.86
									AQQNNVEHKVETFSGVYK	95.0%	30.3	22.9	0	2	0	2	2,078.03
									DVNFEFPEFQL	95.0%	42.7	21.2	4	0	0	2	1,384.64
									EIEVGGGR	95.0%	38.4	21.6	2	0	0	2	816.42
									HVVFIAQR	95.0%	31.1	17.2	1	0	0	2	969.56
									LTGKDVNFEFPEFQL	95.0%	46.7	22.3	2	0	0	2	1,783.89
									TLTAVHDAILEDLVFPSEIVGK	95.0%	77.4	18.6	3	4	0	2	2,367.28
									TLTAVHDAILEDLVFPSEIVGKR	95.0%	56.0	15.7	0	3	0	2	2,523.38
									VETFSGVYKK	95.0%	31.6	22.4	1	0	0	2	1,157.62
									VKLDGSR	95.0%	33.8	24.1	1	0	0	2	774.45
Gamma-enolase	ENOG_HUMAN	ENO2	47,252	100.00%	5	5	15	27.90%	AAVPSGASTGIYEALELR	95.0%	166.0	21.4	297	64	0	2	1,804.94
									DATNVGDEGGFAPNILENSEALELVK	95.0%	52.7	21.8	2	0	0	2	2,702.32
									GNPTVEVDLYTAK	95.0%	75.0	23.0	2	0	0	2	1,406.72
									IEEELGDEAR	95.0%	55.4	21.1	2	0	0	2	1,160.54
									LGAEVYHTLK	95.0%	63.8	23.2	3	0	0	2	1,130.62
									SGETEDTFIADLVVGLCTGQIK	95.0%	83.2	22.3	7	1	0	2	2,353.16
									YDLDFK	95.0%	34.7	19.7	4	0	0	2	800.38
									YITGDQLGALYQDFVR	95.0%	126.0	22.1	6	0	0	2	1,858.93
Dipeptidyl peptidase 9	DPP9_HUMAN	DPP9	98,246	100.00%	9	10	21	14.40%	AGKPYQLQIYPNER	95.0%	47.6	22.5	1	2	0	2	1,676.88
									ELVQPFSSLFPK	95.0%	40.1	20.9	4	0	0	2	1,391.76
									ENSLLYSEIPK	95.0%	38.9	23.2	2	0	0	2	1,292.67
									HPTVLFVYGGPQVQLVNNSFK	95.0%	34.6	19.3	0	2	0	2	2,344.25
									LAEFQTDSQGK	95.0%	51.0	22.3	2	0	0	2	1,223.59
									LSGPDDDPLHK	95.0%	39.3	22.4	0	1	0	2	1,193.58
									NQMGQVEIEDQVEGLQFVAEK	95.0%	34.5	21.0	0	1	0	2	2,407.15
									SAGVATFVIQEEFDR	95.0%	71.7	22.1	2	0	0	2	1,668.82
									YGFIDLSR	95.0%	44.1	20.8	4	0	0	2	970.50
Coronin-1C	COR1C_HUMAN	CORO1C	53,232	100.00%	3	3	9	10.10%	AIFLADGNVFTTGFSR	95.0%	92.0	22.1	5	0	0	2	1,715.88
									TTDTASVQNEAK	95.0%	58.4	22.5	1	0	0	2	1,264.60

									YFEITDESPYVHYLNTFSSK	95.0%	38.0	20.9	0	3	0	2	2,440.13
Heterogeneous nuclear ribonucleoprotein Q	HNRPQ_HUMAN	SYNCRIP	69,586	100.00%	15	17	160	30.20%	AIEALKEFNEDGALAVLQQFK	95.0%	48.7	20.0	0	10	0	2	2,334.23
									AMEEMNGKDLEGENIEIVFAKPPDQK	95.0%	19.6	20.0	0	0	1	2	2,964.40
									DLFEDELVPLFEK	95.0%	81.0	23.1	34	0	0	2	1,593.81
									DSDLSHVQNK	95.0%	57.6	21.5	9	0	0	2	1,142.54
									EAAQEAVK	95.0%	45.9	22.0	17	0	0	2	845.44
									EFNEDGALAVLQQFK	95.0%	61.1	22.8	8	0	0	2	1,708.85
									EQILEEFSK	95.0%	43.6	23.4	3	0	0	2	1,122.57
									KYGGPPPDSVYSGQQPSVGTEIFVGK	95.0%	38.8	21.0	0	2	0	2	2,694.34
									LFVGSIPK	95.0%	35.3	18.4	2	0	0	2	860.52
									LMMDPLTGLNR	95.0%	55.9	22.9	24	0	0	2	1,292.63
									NLANTVTEEILEK	95.0%	95.0	22.1	8	0	0	2	1,473.78
									TGYTLDVTTGQR	95.0%	77.1	22.4	16	0	0	2	1,311.65
									TKEQILEEFSK	95.0%	63.9	21.8	6	1	0	2	1,351.71
									VADSSKGPDEAK	95.0%	52.0	22.8	6	0	0	2	1,203.59
									VTEGLTDVILYHQPDDK	95.0%	86.0	22.4	4	9	0	2	1,942.98
5'-nucleotidase domain-containing protein 1	NT5D1_HUMAN	NT5DC1	51,830	100.00%	7	8	15	24.80%	DIVAAIQHNYK	95.0%	64.2	21.7	2	0	0	2	1,271.67
									GLALDLEDGNFLK	95.0%	79.8	23.2	2	0	0	2	1,404.74
									ISTYSTIAIPSIEAIAELPLDYK	95.0%	49.0	18.7	1	0	0	2	2,508.35
									MMTPEVLAEAYGK	95.0%	56.8	20.7	2	0	0	2	1,471.68
									SQRPEESEPLEK	95.0%	66.3	22.3	2	2	0	2	1,428.70
									TAGYYPNPPLVLSSDETLISK	95.0%	79.6	20.9	2	0	0	2	2,265.17
									YNLPESAPLIYNSFAQFLVK	95.0%	76.2	20.5	2	0	0	2	2,314.21
Exostosin-like 2	EXTL2_HUMAN	EXTL2	37,449	100.00%	6	7	23	24.50%	APDELWNSLGPHPIPVIFK	95.0%	38.9	20.0	0	2	0	2	2,130.14
									LVNIYDSMPLR	95.0%	64.0	22.1	8	0	0	2	1,336.69
									TSGIFVKPVNMDNLEK	95.0%	30.6	21.9	1	1	0	2	1,807.93
									VIVVWNNIGEK	95.0%	50.1	20.3	2	0	0	2	1,270.72
									YLELFQR	95.0%	39.6	21.8	7	0	0	2	968.52
									YSNIMISQFGFPYANYK	95.0%	78.8	21.3	2	0	0	2	2,058.96
Nardilysin	NRDC_HUMAN	NRD1	131,558	100.00%	11	12	23	11.70%	AFTTTLNLLPYHK	95.0%	37.1	20.9	2	0	0	2	1,518.83
									ANLVLLSGANEGK	95.0%	93.3	21.9	3	0	0	2	1,285.71
									GSLSNAGDPEIVK	95.0%	56.3	22.9	2	0	0	2	1,286.66
									IENLTEEAFNTQVTALIK	95.0%	45.1	20.6	1	0	0	2	2,034.08
									LAHEIEALK	95.0%	34.0	18.8	1	0	0	2	1,023.58
									LGPEKR	95.0%	38.3	20.4	1	0	0	2	699.42
									LLILEYAR	95.0%	49.5	15.1	3	0	0	2	990.60
									LQNGLQALLISDLSNMEGK	95.0%	88.7	21.7	2	2	0	2	2,060.07
									TVFQFDVQR	95.0%	47.1	22.2	4	0	0	2	1,139.59
									TYFNILIKPETLAK	95.0%	29.6	15.9	0	1	0	2	1,650.95
									YPDENGFDAFLK	95.0%	63.6	20.9	1	0	0	2	1,415.65
Aldose reductase	ALDR_HUMAN	AKR1B1	35,836	100.00%	2	2	5	5.38%	REELFIVSK	95.0%	43.2	20.6	3	0	0	2	1,120.64
									TTAQVLIR	95.0%	41.8	18.5	2	0	0	2	901.55
Isoleucyl-tRNA synthetase, mitochondrial	SYIM_HUMAN	IARS2	113,776	100.00%	12	12	21	14.00%	ASPLLPANHVTMAK	95.0%	38.2	21.8	1	0	0	2	1,465.78
									DSFLGSIPGK	95.0%	44.0	22.5	2	0	0	2	1,020.54
									EAQNLSAMEIR	95.0%	42.8	22.5	2	0	0	2	1,277.62
									ELSNFYFSIIK	95.0%	48.7	23.9	2	0	0	2	1,360.72
									EMTADVIELK	95.0%	46.8	23.1	2	0	0	2	1,164.58
									EPPYGADVLR	95.0%	33.9	22.5	2	0	0	2	1,116.57
									FIPGSALNGMVEMMDR	95.0%	50.2	20.5	2	0	0	2	1,815.81
									FLINLEGGDIR	95.0%	62.1	22.1	1	0	0	2	1,246.68
									FPLLKPSPK	95.0%	35.4	17.2	2	0	0	2	1,026.64

									QQPDTELEIQQK	95.0%	45.4	22.0	2	0	0	2	1,456.73
									SGDLYVLAADK	95.0%	30.5	23.0	1	0	0	2	1,151.60
									TKDEYLINSQTTEHIVK	95.0%	41.1	22.3	0	2	0	2	2,019.04
Tumor necrosis factor receptor superfamily member 6B	TNF6B_HUMAN	TNFRSF6B	32,661	100.00%	9	12	232	29.00%	AVIDFVAFQDISIK	95.0%	44.1	18.4	3	0	0	2	1,565.86
									AVIDFVAFQDISIKR	95.0%	71.2	17.8	3	0	0	2	1,721.96
									HYTQFWNYLER	95.0%	38.6	21.6	1	0	0	2	1,556.73
									LLQALEAPEGWGPTPR	95.0%	74.8	21.4	20	4	0	2	1,734.92
									LTELLGAQDGALLVR	95.0%	110.0	17.7	164	3	0	2	1,568.90
									MPGLER	95.0%	35.6	23.5	2	0	0	2	718.36
									RLTELLGAQDGALLVR	95.0%	81.9	14.3	11	17	0	2	1,725.00
									VPGAEECER	95.0%	39.7	17.4	2	0	0	2	1,046.46
									YCNVLCGEREEEAR	95.0%	34.5	17.8	0	2	0	2	1,784.77
Aconitate hydratase, mitochondrial	ACON_HUMAN	ACO2	85,410	100.00%	5	6	15	9.87%	AKDINQEVYNFLATAGAK	95.0%	44.0	21.6	0	3	0	2	1,953.01
									DINQEVYNFLATAGAK	95.0%	87.7	22.1	4	0	0	2	1,753.88
									IVYGHLDDPASQEIER	95.0%	41.2	22.1	1	2	0	2	1,841.90
									NDANPETHAFVTSPEIVTALAIAGTLK	95.0%	53.3	19.1	0	3	0	2	2,780.45
									QGLLPLTFADPADYNK	95.0%	77.7	22.2	2	0	0	2	1,762.90
Cathepsin B	CATB_HUMAN	CTSB	37,803	100.00%	14	22	212	38.10%	DIMAEIYK	95.0%	32.0	21.3	2	0	0	2	998.49
									HYGYNSYSVSNSEK	95.0%	102.0	19.1	6	2	0	2	1,634.71
									HYGYNSYSVSNSEKDIMAEIYK	95.0%	46.3	18.6	0	4	2	2	2,614.18
									ICEPGYSPTYK	95.0%	51.2	20.4	4	0	0	2	1,314.60
									LCGTFLGGPKPPQR	95.0%	61.8	21.3	4	2	0	2	1,527.81
									LPASFDAR	95.0%	53.2	24.5	11	0	0	2	876.46
									NGPVEGAFSVYSDFLLYK	95.0%	121.0	21.6	44	0	0	2	2,005.99
									QDKHYGYNSYSVSNSEK	95.0%	31.3	17.6	0	2	0	2	2,005.89
									SGVYQHVTGEMMGGHAIR	95.0%	64.2	21.5	6	22	1	2	1,961.90
									SRPSFHPLSDELVNYVNK	95.0%	71.5	21.8	0	10	6	2	2,102.07
									SRPSFHPLSDELVNYVNKR	95.0%	64.2	20.5	0	5	25	2	2,258.17
									TDQYWEKI	95.0%	40.4	21.6	1	0	0	2	1,082.52
									VMFTEDLK	95.0%	39.1	21.3	5	0	0	2	998.49
									VMFTEDLKLPASFDAR	95.0%	82.2	22.7	15	33	0	2	1,855.93
Lamin-B1	LMNB1_HUMAN	LMNB1	66,392	100.00%	21	21	87	35.70%	AGGPTTPLSPTR	95.0%	40.7	22.3	4	0	0	2	1,154.62
									ALDDTAR	95.0%	34.9	23.9	1	0	0	2	761.38
									ALYETELADAR	95.0%	71.9	22.8	9	0	0	2	1,251.62
									ALYETELADARR	95.0%	31.4	22.4	1	0	0	2	1,407.72
									DAALATALGDKK	95.0%	70.1	23.3	8	0	0	2	1,173.65
									EELMESR	94.8%	30.3	20.0	1	0	0	2	909.40
									IESLSSQLSNLQK	95.0%	68.1	21.8	1	0	0	2	1,446.78
									IGDTSVSYK	95.0%	31.5	20.4	1	0	0	2	969.49
									IQELEDLLAK	95.0%	66.5	22.9	9	0	0	2	1,171.66
									KLLEGEEER	95.0%	38.7	23.0	1	0	0	2	1,102.57
									KSMYEEEINETR	95.0%	64.5	20.3	1	0	0	2	1,544.69
									LAQALHEMR	95.0%	38.4	22.0	1	0	0	2	1,084.56
									LLEGEEER	95.0%	34.4	22.0	2	0	0	2	974.48
									LQEKEELR	95.0%	43.5	22.3	2	0	0	2	1,044.57
									LREYEAALNSK	95.0%	48.1	22.9	5	0	0	2	1,293.68
									LSSEMNTSTVNSAR	95.0%	95.6	21.1	18	0	0	2	1,512.70
									LYKEELEQTYHAK	95.0%	34.1	22.7	0	1	0	2	1,651.83
									MRIESLSSQLSNLQK	95.0%	27.6	21.5	0	1	0	2	1,749.92
									NSQGEEVAQR	95.0%	66.7	20.0	9	0	0	2	1,117.52
									QLADETLLK	95.0%	46.3	23.3	2	0	0	2	1,030.58

									RALDDTAR	95.0%	38.3	23.8	2	0	0	2	917.48
									SMYEEEINETR	95.0%	69.0	17.3	5	0	0	2	1,416.60
									TTIPEEEEEEEEAAGVVVEEELFHQQGTPR	95.0%	97.2	19.5	0	5	0	2	3,411.57
Endoplasmic reticulum resident protein 29	ERP29_HUMAN	ERP29	28,977	100.00%	7	7	20	39.80%	DGDFENPVPYTGAVK	95.0%	73.8	22.5	3	0	0	2	1,608.75
									ESYPVFYLFR	95.0%	31.2	23.7	2	0	0	2	1,320.66
									FDTQYPYGEK	95.0%	40.8	18.9	2	0	0	2	1,247.56
									GALPLDTVTFYK	95.0%	71.5	20.6	2	0	0	2	1,324.72
									ILDQGEDFPASEMTR	95.0%	93.6	21.1	4	0	0	2	1,724.78
									LAENSASSDDLLVAEVGISDYGDKLNMELSEK	95.0%	129.0	19.5	0	2	0	2	3,428.63
									SLNILTAFQK	95.0%	74.5	18.1	5	0	0	2	1,134.65
NSFL1 cofactor p47	NSF1C_HUMAN	NSFL1C	40,555	100.00%	6	6	12	26.20%	ASSSILIDESEPTTNIQIR	95.0%	96.7	22.1	2	0	0	2	2,074.07
									DLIHDQDEDEEEEEGQR	95.0%	30.7	14.5	0	2	0	2	2,085.85
									EANLLNAVIVQR	95.0%	69.4	18.8	2	0	0	2	1,339.77
									LGSTAPQVLSTSSPAQQAENEAK	95.0%	79.4	22.1	2	0	0	2	2,314.15
									SPNELVDDLFK	95.0%	62.5	23.4	2	0	0	2	1,276.64
									SYQDPSNAQFLESIR	95.0%	96.6	21.5	2	0	0	2	1,754.84
Spectrin alpha chain, brain	SPTA2_HUMAN	SPTAN1	284,525	100.00%	27	28	82	15.80%	ALINADELASDVAGAEALLDR	95.0%	92.9	21.2	4	1	0	2	2,127.09
									DLAALEDKVK	95.0%	35.0	21.4	1	0	0	2	1,101.62
									DLASVQALLR	95.0%	79.2	21.2	8	0	0	2	1,085.63
									DLSSVQTLLTK	95.0%	68.1	21.0	4	0	0	2	1,204.68
									EAIVTSEELGQDLEHVEVLQK	95.0%	61.4	20.7	0	1	0	2	2,366.21
									ELPTAFDYVEFTR	95.0%	85.9	22.6	3	0	0	2	1,587.77
									FLADFR	95.0%	32.1	20.3	1	0	0	2	768.40
									GKDLIGVQNLLK	95.0%	34.5	14.9	1	0	0	2	1,297.78
									GNAMVEEGHFAAEDVK	95.0%	31.4	19.7	0	1	0	2	1,719.76
									GVIDMGNSLIER	95.0%	54.6	22.9	3	0	0	2	1,319.66
									IAALQAFADQLIAAGHYAK	95.0%	34.4	19.8	0	3	0	2	1,972.07
									ITALDEFATK	95.0%	49.8	22.3	6	0	0	2	1,108.59
									KVEDLFLTFAK	95.0%	72.3	18.8	3	0	0	2	1,310.74
									LFGAAEVQR	95.0%	55.1	24.1	6	0	0	2	990.54
									LGESQTLQQFSR	95.0%	76.6	22.9	4	0	0	2	1,393.71
									LIQEQHPEEELIK	95.0%	62.5	21.7	5	0	0	2	1,605.85
									LIQSHPESAEDLQEK	95.0%	26.4	22.2	0	1	0	2	1,723.85
									LLVGSEDYGR	95.0%	42.0	22.6	2	0	0	2	1,108.56
									LQTASDESYKDPTNIQSK	95.0%	31.3	21.8	0	1	0	2	2,024.98
									MTLVASEDYGDTLAAIQGLLK	95.0%	110.0	21.3	3	0	0	2	2,225.14
									NQALNTDNYGHDLASVQALQR	95.0%	33.3	22.3	0	2	0	2	2,328.13
									QQVAPTDDETGKELVLALYDYQEK	95.0%	28.7	21.4	0	1	0	2	2,753.35
									SADESGQALLAAGHYASDEVR	95.0%	59.4	21.6	0	7	0	2	2,147.00
									SSLSSAQADFNQLAELDR	95.0%	130.0	21.5	6	0	0	2	1,951.94
									TATDEAYKDPSNLQGK	95.0%	32.6	22.2	1	0	0	2	1,737.83
									VLETAEDIQER	95.0%	57.4	22.5	2	0	0	2	1,302.65
									VNEVNQFAAK	95.0%	46.5	23.7	1	0	0	2	1,119.58
Stress-70 protein, mitochondrial	GRP75_HUMAN	HSPA9	73,663	100.00%	18	20	91	37.00%	AMQDAEVSK	94.7%	30.2	20.1	1	0	0	2	994.45
									AQFEGIVTDLIR	95.0%	83.6	21.2	9	0	0	2	1,361.74
									DAGQISGLNVLR	95.0%	59.4	22.9	8	0	0	2	1,242.68
									EQQIVIQSSGGLSKDDIENMVK	95.0%	53.2	21.5	0	4	0	2	2,434.21
									ETGVDLTKDNMALQR	95.0%	59.1	22.5	4	0	0	2	1,706.84
									KDSETGENIR	95.0%	53.0	23.0	1	0	0	2	1,148.55
									LLGQFTLIGIPPAPR	95.0%	42.1	10.4	3	0	0	2	1,592.95
									LYSPSQIGAFVLMK	95.0%	58.5	21.8	5	3	0	2	1,569.84

									MKETAENYLGHTAK	95.0%	28.7	23.3	0	2	2	2	1,608.77
									NAVITVPAYFNDSQR	95.0%	58.1	22.8	6	0	0	2	1,694.85
									NTTIPTK	95.0%	41.5	24.6	2	0	0	2	774.44
									QAVTNPNNTFYATK	95.0%	94.0	22.6	4	0	0	2	1,568.77
									RYDDPEVQK	95.0%	45.0	22.8	3	0	0	2	1,149.55
									SDIGEVILVGGMTR	95.0%	63.5	23.0	3	0	0	2	1,462.76
									STNGDTFLGGEDFDQALLR	95.0%	107.0	21.7	5	0	0	2	2,055.96
									TTPSVVAFTADGER	95.0%	111.0	22.6	6	0	0	2	1,450.72
									VINEPTAAALAYGLDK	95.0%	117.0	21.2	6	0	0	2	1,645.88
									VLENAEGAR	95.0%	52.6	21.0	8	0	0	2	958.50
									VQQTVQDLFGR	95.0%	80.3	23.4	8	0	0	2	1,290.68
Splicing factor 3A subunit 1	SF3A1_HUMAN	SF3A1	88,868	100.00%	4	4	15	9.08%	ASKPLPPAPAPDEYLVSPITGEK	95.0%	37.5	19.4	0	2	0	2	2,377.27
									LNGQVLVFTLPLTDQVSVIK	95.0%	50.3	9.0	3	0	0	2	2,184.26
									VMQQQQQTTQQQLPQK	95.0%	81.6	21.9	7	0	0	2	1,957.98
									VQAQVIQETIVPK	95.0%	43.1	16.7	3	0	0	2	1,452.84
Heterogeneous nuclear ribonucleoprotein G	HNRPG_HUMAN	RBMX	42,316	100.00%	10	12	29	25.10%	AIKVEQATKPSFESGR	95.0%	37.5	21.2	0	2	2	2	1,747.93
									ALEAVFGK	95.0%	32.9	20.9	1	0	0	2	834.47
									GFAFVTFESPADAK	95.0%	98.0	22.3	5	0	0	2	1,486.72
									GFAFVTFESPADAKDAAR	95.0%	35.8	21.7	0	1	0	2	1,899.92
									GGHMDDGGYSMNFNMSSSR	95.0%	34.1	6.0	0	1	0	2	2,097.77
									IVEVLLMK	95.0%	60.8	17.0	3	0	0	2	960.58
									LFIGGLNTETNEK	95.0%	72.9	22.3	6	0	0	2	1,435.74
									RGPPPPPR	95.0%	51.6	23.0	2	0	0	2	873.51
									VEQATKPSFESGR	95.0%	65.2	23.2	2	2	0	2	1,435.72
									YDDYSSSR	95.0%	39.7	14.1	2	0	0	2	992.40
Eukaryotic translation initiation factor 4H	IF4H_HUMAN	EIF4H	27,368	100.00%	4	4	12	21.80%	EEVVQKEQE	95.0%	52.5	21.6	5	0	0	2	1,117.54
									GSNMDFREPTEEER	95.0%	45.9	16.8	0	4	0	2	1,712.72
									TGPPMGSR	95.0%	38.0	20.9	1	0	0	2	818.38
									TVATPLNQVANPNSAIFGGARPR	95.0%	38.6	19.1	0	2	0	2	2,351.26
Splicing factor, arginine/serine-rich 1	SFRS1_HUMAN	SFRS1	27,727	100.00%	6	6	32	30.60%	DGTGVVEFVR	95.0%	52.2	23.5	10	0	0	2	1,078.55
									EAGDVCYADVYR	95.0%	56.8	17.0	2	0	0	2	1,417.61
									GGPPFAFVEFEDPRDAEDAVYGR	95.0%	39.2	20.5	0	5	0	2	2,541.17
									IYVGNLPPDIR	95.0%	50.6	21.6	7	0	0	2	1,256.70
									SHEGETAYIR	94.6%	30.1	21.0	1	0	0	2	1,162.55
									TKDIEDVFYK	95.0%	65.9	22.2	7	0	0	2	1,257.64
Vasorin	VASN_HUMAN	VASN	71,696	100.00%	3	3	6	6.09%	LAGLGLQQLDEGLFSR	95.0%	99.8	19.6	3	0	0	2	1,716.93
									SLTLGIEPVSPTSLR	95.0%	58.9	18.8	2	0	0	2	1,569.89
									YLQGSSVQLR	95.0%	43.3	22.5	1	0	0	2	1,150.62
Casein kinase II subunit alpha'	CSK22_HUMAN	CSNK2A2	41,197	100.00%	5	5	9	17.40%	HLVSPEALDLLDK	95.0%	66.9	20.8	2	0	0	2	1,449.80
									QLYQILTDFDIR	95.0%	63.2	21.4	2	0	0	2	1,524.81
									TPALVFEYINNTDFK	95.0%	80.4	22.4	2	0	0	2	1,771.89
									VLGTEELYGYLK	95.0%	32.5	21.0	1	0	0	2	1,384.74
									VYAEVNSLR	95.0%	58.9	22.4	2	0	0	2	1,050.56
Haloacid dehalogenase-like hydrolase domain-containing protein 2	HDHD2_HUMAN	HDHD2	28,519	100.00%	3	3	8	16.20%	DGLALGPGPFVTALEYATDTK	95.0%	88.9	21.8	4	0	0	2	2,136.09
									LLLDGAPLIAIHK	95.0%	29.5	7.0	0	2	0	2	1,373.85
									TFFLEALR	95.0%	48.5	20.7	2	0	0	2	996.55
Junction plakoglobin	PLAK_HUMAN	JUP	81,728	100.00%	11	11	45	20.40%	ALMGSPQLVAAVVR	95.0%	75.4	20.2	9	0	0	2	1,427.80
									HLTSNSPR	95.0%	31.0	21.8	1	0	0	2	911.47
									HPEAEMAQNSVR	95.0%	33.3	20.6	1	0	0	2	1,384.63
									LIILANGGPQALVQIMR	95.0%	92.0	13.2	8	0	0	2	1,823.06
									LLNDEDPVVVTK	95.0%	50.1	21.2	7	0	0	2	1,341.73

									LNYGIPAIVK	95.0%	60.1	18.1	10	0	0	2	1,087.65
									LVQLLVK	95.0%	32.2	10.0	1	0	0	2	812.56
									NLALCPANHAPLQEAAVIPR	95.0%	41.3	19.4	0	1	0	2	2,155.14
									SAIVHLINYQDDAELATR	95.0%	45.7	21.8	0	2	0	2	2,029.04
									VLSVCPSNKPAIVEAGGMQALGK	95.0%	27.3	20.1	0	1	0	2	2,342.22
									VSVELTNSLFK	95.0%	61.5	20.6	4	0	0	2	1,236.68
Alpha-1-antichymotrypsin	AACT_HUMAN	SERPINA3	47,635	100.00%	32	47	6153	71.40%	ADLSGITGAR	95.0%	105.0	24.3	463	0	0	2	960.51
									AKWEMPFDPQDTHQSR	95.0%	65.8	20.8	5	18	0	2	1,988.89
									AVLDVFEEGTEASAATAVK	95.0%	145.0	22.3	1052	234	0	2	1,907.96
									DEELSCTVVELK	95.0%	101.0	22.0	12	0	0	2	1,421.68
									DLDSQTMMVLVNYIFFK	95.0%	88.9	21.5	251	90	0	2	2,096.01
									DSLEFR	95.0%	52.8	22.0	61	0	0	2	766.37
									DYNLNDILLQLGIEEAFTSK	95.0%	157.0	21.0	68	24	0	2	2,296.17
									EIGELYLPK	95.0%	52.3	20.9	94	0	0	2	1,061.59
									EQLSLLDR	95.0%	71.7	22.4	218	0	0	2	973.53
									EQLSLLDRFTEDAK	95.0%	80.0	22.6	100	31	0	2	1,664.85
									FNRPFLMIIVPTDTQNIFFMSK	95.0%	73.3	20.1	0	238	0	2	2,691.37
									FTEDAKR	95.0%	33.6	23.2	5	0	0	2	866.44
									GKITDLIK	95.0%	50.3	16.8	15	0	0	2	887.56
									GTHVDLGLASANVDFAFSLYK	95.0%	136.0	21.8	133	119	0	2	2,225.12
									ITDLIK	95.0%	41.0	20.0	54	0	0	2	702.44
									ITDLIKDLDSQTMMVLVNYIFFK	95.0%	64.4	20.2	2	18	0	2	2,779.43
									ITLLSALVETR	95.0%	123.0	15.7	207	4	0	2	1,215.73
									KLINDYVK	95.0%	57.5	19.7	174	0	0	2	992.58
									LINDYVK	95.0%	53.6	21.2	48	0	0	2	864.48
									LYGSEAFATDFQDSAAAK	95.0%	140.0	21.7	1083	6	0	2	1,891.87
									LYGSEAFATDFQDSAAAKK	95.0%	85.7	21.0	170	286	0	2	2,019.97
									MEEVEAMLLPETLK	95.0%	125.0	22.9	23	0	0	2	1,664.81
									MEEVEAMLLPETLKR	95.0%	67.6	22.5	14	19	0	2	1,820.91
									NLAVSQVVHK	95.0%	63.1	18.9	344	0	0	2	1,094.63
									NLAVSQVVHKAVLDVFEEGTEASAATAVK	95.0%	57.7	18.1	0	1	0	2	2,983.57
									QLVLKAPDK	95.0%	41.9	14.8	1	0	0	2	1,011.62
									RLYGSEAFATDFQDSAAAK	95.0%	125.0	21.7	190	220	0	2	2,047.97
									TLNQSSDELQLSMGNAMFVK	95.0%	117.0	21.3	4	2	0	2	2,245.05
									WEMPFDPQDTHQSR	95.0%	61.6	16.9	12	7	0	2	1,789.76
									WRDSLEFR	95.0%	40.7	22.4	18	7	0	2	1,108.55
									WVMVPMMSLHHLTIPYFR	95.0%	24.6	21.7	0	0	5	2	2,306.13
									YTGNASALFILPDQDKMEEVEAMLLPETLK	95.0%	67.6	20.2	0	3	0	2	3,398.68
Alpha-galactosidase A	AGAL_HUMAN	GLA	48,750	100.00%	6	7	37	19.30%	ALLQDKDVIAINQDPLGK	95.0%	96.6	17.6	7	10	0	2	1,951.09
									LQADPQRFPHGIR	95.0%	28.4	21.7	0	1	0	2	1,534.82
									QEIGGPR	95.0%	40.8	20.7	3	0	0	2	756.40
									SHINPTGTVLLQLENTMQMSLK	95.0%	46.8	21.5	0	3	0	2	2,487.26
									SILDWTSFNQER	95.0%	71.0	22.1	1	0	0	2	1,495.72
									SYTIAVASLGK	95.0%	78.8	19.6	12	0	0	2	1,109.62
CD44 antigen	CD44_HUMAN	CD44	81,535	100.00%	7	8	113	12.40%	ALSIGFETCR	95.0%	65.0	22.6	12	0	0	2	1,153.57
									ESSETPDQFMTADETR	95.0%	112.0	10.4	11	0	0	2	1,859.76
									FAGVFHVEK	95.0%	42.8	22.9	31	0	0	2	1,033.55
									LVINSGNGAVEDR	95.0%	68.7	21.9	6	0	0	2	1,343.69
									TEAADLCK	95.0%	45.0	20.9	8	0	0	2	907.42
									TNPEDIYPSNPTDDDVSSGSSSER	95.0%	79.6	15.3	4	0	0	2	2,569.08
									YGFIEGHVVIPR	95.0%	72.6	22.5	28	13	0	2	1,386.75

Reticulon-4	RTN4_HUMAN	RTN4	129,917	99.50%	2	2	3	1.76%	GPLPAAPPVAPER	95.0%	34.3	21.7	2	0	0	2	1,271.71
									SDEGHPFR	95.0%	35.7	18.1	1	0	0	2	944.42
Ubiquitin fusion degradation protein 1 homolog	UFD1_HUMAN	UFD1L	34,483	100.00%	2	2	6	8.14%	FQPQSPDFLDITNPK	95.0%	54.6	22.2	5	0	0	2	1,746.87
									LNITYPMLFK	95.0%	33.4	21.6	1	0	0	2	1,255.68
40S ribosomal protein S25	RS25_HUMAN	RPS25	13,725	100.00%	3	3	8	16.00%	AALQELLSK	95.0%	62.3	20.0	4	0	0	2	972.57
									DKLNNLVLFDK	95.0%	36.3	20.0	2	0	0	2	1,318.74
									LNNLVLFDK	95.0%	50.5	20.9	2	0	0	2	1,075.62
Calpain-1 catalytic subunit	CAN1_HUMAN	CAPN1	81,875	100.00%	10	10	26	18.10%	APSDLYQIILK	95.0%	64.3	18.9	4	0	0	2	1,260.72
									DMETIGFAVYEVPPELVGQPAVHLK	95.0%	71.5	21.6	0	3	0	2	2,755.40
									GHAYSVTGAK	95.0%	38.7	23.9	1	0	0	2	990.50
									KAPSDLYQIILK	95.0%	65.7	16.7	4	0	0	2	1,388.82
									LPPGEYVVVPSTFEPNKEGDFVLR	95.0%	33.0	20.5	0	1	0	2	2,689.39
									LYELIITR	95.0%	44.9	14.6	3	0	0	2	1,020.61
									QLAGEDMEISVK	95.0%	55.9	22.4	2	0	0	2	1,335.65
									RPTELLSNPQFIVDGATR	95.0%	53.0	21.2	0	3	0	2	2,014.07
									SGSMSAYEMR	95.0%	62.8	12.0	2	0	0	2	1,150.45
									YLGQDYEQLR	95.0%	48.3	21.4	3	0	0	2	1,284.62
Serpin B8	SPB8_HUMAN	SERPINB8	42,750	100.00%	5	5	11	20.10%	ADFSGMSTEK	95.0%	52.4	14.5	3	0	0	2	1,088.46
									ISEVLDAGTVDPLTK	95.0%	107.0	22.6	4	0	0	2	1,557.84
									LEESYDLEPFLR	95.0%	43.7	23.0	1	0	0	2	1,510.74
									LGMIDAFDEAK	95.0%	38.2	21.4	2	0	0	2	1,225.58
									LVLVNAIYFK	95.0%	71.6	14.0	11	0	0	2	1,179.71
									SKVQVFLPR	95.0%	30.0	17.0	0	1	0	2	1,073.65
									TGTQYLLR	95.0%	66.4	20.4	8	0	0	2	951.53
60S ribosomal protein L18	RL18_HUMAN	RPL18	21,617	100.00%	5	5	18	25.50%	ILTFDQLALDSPK	95.0%	99.2	21.9	8	0	0	2	1,460.80
									TAVVVGTITDDVR	95.0%	91.1	22.0	4	0	0	2	1,345.73
									TNRPPLSLSR	95.0%	36.5	18.3	0	2	0	2	1,140.65
									TNSTFNQVVLK	95.0%	66.2	21.8	2	0	0	2	1,250.67
									TNSTFNQVVLKR	95.0%	56.5	20.7	2	0	0	2	1,406.78
Cytochrome c	CYC_HUMAN	CYCS	11,731	100.00%	7	8	22	56.20%	ADLIAYLK	95.0%	51.7	17.4	3	0	0	2	906.53
									ADLIAYLKK	95.0%	46.9	13.2	3	0	0	2	1,034.63
									GIIWGEDTLMEYLENPK	95.0%	85.5	22.5	2	0	0	2	2,023.97
									KTGQAPGYSYTAANK	95.0%	76.4	22.1	3	2	0	2	1,556.77
									MIFVGIK	95.0%	31.2	20.4	1	0	0	2	807.48
									TGPNLHGLFGR	95.0%	81.7	21.1	4	0	0	2	1,168.62
									TGQAPGYSYTAANK	95.0%	73.4	21.7	4	0	0	2	1,428.68
Twinfilin-1	TWF1_HUMAN	TWF1	40,266	99.50%	2	3	7	9.14%	HQTLQGVAFPISR	95.0%	46.9	20.1	1	4	0	2	1,453.79
									YLLSQSSPAPLTAAEEELR	95.0%	96.6	21.4	2	0	0	2	2,075.07
Superoxide dismutase [Cu-Zn]	SODC_HUMAN	SOD1	15,917	100.00%	4	5	11	29.90%	ADDLGKGGNEESTK	95.0%	54.8	21.3	2	1	0	2	1,420.66
									AVCVLKGDGPVQGIINFEQK	95.0%	35.4	20.5	0	1	0	2	2,172.15
									GDGPVQGIINFEQK	95.0%	82.9	23.1	5	0	0	2	1,501.77
									HVGDLGNVTADK	95.0%	59.2	22.7	2	0	0	2	1,225.62
Amidophosphoribosyltransferase	PUR1_HUMAN	PPAT	57,381	100.00%	3	3	9	6.77%	EAPTAYSLLIMHR	95.0%	44.7	22.5	2	0	0	2	1,517.78
									EVLPGEIVEISR	95.0%	51.7	20.1	4	0	0	2	1,340.74
									IVLVDDSIVR	95.0%	40.7	21.2	3	0	0	2	1,128.66
Small nuclear ribonucleoprotein E	RUXE_HUMAN	SNRPE	10,786	100.00%	3	3	9	29.30%	GDNITLLQSVSN	95.0%	53.3	22.7	2	0	0	2	1,260.64
									IMLKGDNITLLQSVSN	95.0%	37.5	20.9	1	0	0	2	1,761.94
									VMVQPINLIFR	95.0%	62.4	18.5	6	0	0	2	1,345.77
Transforming growth factor beta receptor type 3	TGBR3_HUMAN	TGFBR3	93,411	100.00%	11	13	74	16.70%	GTTGLPQEVHVLNLR	95.0%	71.0	20.5	5	1	0	2	1,633.90
									IIAPNSIGFGK	95.0%	58.0	21.0	9	0	0	2	1,116.64
									ILLDPGALPALQNPPIR	95.0%	42.5	14.9	3	0	0	2	1,798.06

									KEYGAVTSFTELK	95.0%	98.6	22.1	4	0	0	2	1,472.76
									LATGVSR	95.0%	45.3	24.8	2	0	0	2	703.41
									NFLSLNYLAEYLQPK	95.0%	83.6	21.1	12	0	0	2	1,812.95
									NLILILK	95.0%	31.3	7.0	3	0	0	2	826.58
									SIRDDIPSTQGNLVK	95.0%	93.5	20.8	6	2	0	2	1,642.88
									TAGQGPGQLQR	95.0%	70.4	22.0	17	0	0	2	1,112.58
									VGEDQVFPPK	95.0%	50.9	22.0	8	0	0	2	1,115.57
									WALDNGYSPITSYTMAPVANR	95.0%	97.6	21.3	2	0	0	2	2,343.11
AP-2 complex subunit alpha-1	AP2A1_HUMAN	AP2A1	107,531	100.00%	4	4	7	8.80%	ALLLSTYIK	95.0%	44.6	12.8	2	0	0	2	1,021.63
									DFLTPPLLSVR	95.0%	32.6	20.3	2	0	0	2	1,257.72
									EMGEAFAADIPR	95.0%	34.2	20.5	1	0	0	2	1,322.61
									FFQPTEMAAQDFFQR	95.0%	83.3	21.8	2	0	0	2	1,878.85
									GLAVFISDIR	95.0%	71.8	20.0	4	0	0	2	1,090.63
									VGGYILGEFGNLIAGDPR	95.0%	86.4	21.6	7	0	0	2	1,847.97
									YGGAPQALTLK	95.0%	52.4	21.7	2	0	0	2	1,118.62
GDP-fucose protein O-fucosyltransferase 1	OFUT1_HUMAN	POFUT1	43,938	100.00%	4	4	6	15.20%	DLQGRPSSFFGMDRPPK	95.0%	37.8	22.0	0	1	0	2	1,950.95
									FGNQADHFLGSLAFAK	95.0%	51.0	23.0	0	2	0	2	1,722.86
									STAAPLTMTMCLPDLK	95.0%	54.3	22.1	1	0	0	2	1,781.85
									VISLEDFMEK	95.0%	46.7	22.8	2	0	0	2	1,226.60
40S ribosomal protein S13	RS13_HUMAN	RPS13	17,205	100.00%	6	7	14	39.10%	DSHGVAQVR	95.0%	45.4	21.7	2	0	0	2	968.49
									GLAPDLPEDLYHLIK	95.0%	61.1	20.0	2	0	0	2	1,693.92
									GLSQSALPYR	95.0%	40.2	23.8	2	0	0	2	1,091.59
									GLTPSQIGVILR	95.0%	64.6	16.6	2	0	0	2	1,253.76
									KGLTPSQIGVILR	95.0%	71.0	11.1	2	2	0	2	1,381.85
									LTSDDVKEQIYK	95.0%	79.1	22.6	2	0	0	2	1,438.74
Programmed cell death protein 5	PDCD5_HUMAN	PDCD5	14,267	100.00%	5	5	12	39.20%	AVENYLIQMAR	95.0%	60.4	22.7	4	0	0	2	1,307.68
									HGDPGDAAQQEAK	95.0%	55.7	19.9	2	0	0	2	1,323.59
									NSILAQVLDQSAR	95.0%	87.4	21.7	2	0	0	2	1,414.77
									VSEQGLIEILK	95.0%	46.9	19.7	2	0	0	2	1,228.72
									VSEQGLIEILKK	95.0%	55.0	15.9	2	0	0	2	1,356.81
U1 small nuclear ribonucleoprotein 70 kDa	RU17_HUMAN	SNRNP70	51,540	100.00%	5	5	22	12.80%	DPIPYLPPLEK	95.0%	40.1	20.9	3	0	0	2	1,281.71
									EFEDPRDAPPPTR	95.0%	28.9	21.3	0	1	0	2	1,526.72
									EFEVYGPIKR	95.0%	38.8	21.7	6	0	0	2	1,237.66
									VNYDTTESK	95.0%	45.6	21.0	10	0	0	2	1,056.48
									YDERPGPSPLPHR	95.0%	28.6	22.6	0	2	0	2	1,520.76
Apoptosis-inducing factor 1, mitochondrial	AIFM1_HUMAN	AIFM1	66,884	100.00%	12	15	65	27.90%	AALSASEGEEVPQDK	95.0%	79.4	21.6	4	0	0	2	1,530.73
									ALGTEVIQLFPEK	95.0%	58.0	20.1	7	0	0	2	1,444.81
									ALGTEVIQLFPEKGNMGK	95.0%	38.7	21.0	1	3	0	2	1,948.02
									DGEQHEDLNEVAK	95.0%	63.4	19.6	4	2	0	2	1,483.67
									IIKDGEQHEDLNEVAK	95.0%	35.2	22.1	0	3	0	2	1,837.93
									KVETDHIVAAVGLEPNVELAK	95.0%	35.6	17.5	0	2	0	2	2,232.22
									LNDGSQITYEK	95.0%	71.2	22.4	7	0	0	2	1,267.62
									SATEQSGTGIR	95.0%	80.7	23.4	8	0	0	2	1,106.54
									SIYFQPPSFYVSAQDLPHIENGGVAVLTGK	95.0%	39.4	19.2	0	2	0	2	3,234.65
									TGGLEIDSDFGGFR	95.0%	86.8	21.2	6	0	0	2	1,470.69
									VLIVSEDPELPYMRPPLSK	95.0%	47.8	18.8	3	6	0	2	2,199.17
									VMPNAIVQSVGVSSGK	95.0%	95.3	22.9	7	0	0	2	1,588.84
Lupus La protein	LA_HUMAN	SSB	46,821	100.00%	7	8	61	24.00%	FASDDEHDEHDENGATGPVK	95.0%	28.3	14.3	0	2	0	2	2,169.90
									GSIFVVFDSIESAK	95.0%	72.5	21.6	10	0	0	2	1,498.78
									IIEDQQESLNK	95.0%	78.5	23.6	4	0	0	2	1,316.67
									LTTDFNVIVEALSK	95.0%	105.0	20.1	36	0	0	2	1,549.85

									QKLEEDAEMK	95.0%	33.3	21.7	1	0	0	2	1,236.58
									SPSKPLPEVTDEYKNDVK	95.0%	33.6	21.9	0	4	3	2	2,046.04
									YKETDLLILFK	95.0%	37.7	17.0	1	0	0	2	1,382.79
Platelet-activating factor acetylhydrolase IB subunit gamma	PA1B3_HUMAN	PAFAH1B3	25,716	100.00%	3	3	7	11.30%	AALAGHPR	95.0%	35.9	20.1	2	0	0	2	792.45
									AIVQLVNER	95.0%	50.7	19.5	2	0	0	2	1,041.61
									VVVLGLLPR	95.0%	65.7	3.0	3	0	0	2	965.65
Calsyntenin-3	CSTN3_HUMAN	CLSTN3	106,080	100.00%	7	9	40	9.10%	ATGEGLIR	95.0%	42.6	23.8	1	0	0	2	816.46
									EGLDYRDFESLGK	95.0%	40.0	21.7	2	0	0	2	1,528.73
									ESLLLDTTSLQQR	95.0%	78.9	21.6	4	0	0	2	1,503.80
									IEYAPGAGSLALFPGIR	95.0%	108.0	20.6	9	7	0	2	1,731.94
									LHGSGVPFEAVILDK	95.0%	58.5	19.8	2	4	0	2	1,581.86
									LQYSGER	95.0%	32.8	22.7	1	0	0	2	852.42
									VNDVNEFAPVFVER	95.0%	77.1	22.7	10	0	0	2	1,634.82
Gelsolin	GELS_HUMAN	GSN	85,680	100.00%	34	49	1671	48.10%	AGALNSNDAFVLK	95.0%	90.4	22.9	157	0	0	2	1,319.70
									AGKEPGLQIWR	95.0%	49.6	19.8	2	9	0	2	1,254.70
									AQPVQVAEGSEPDGFWEALGGK	95.0%	117.0	21.1	45	7	0	2	2,272.09
									AVEVLPK	95.0%	57.1	14.5	70	0	0	2	755.47
									DPDQTDGLGLSYLSSHIANVER	95.0%	122.0	21.7	13	13	0	2	2,387.15
									DSQEEEKTEALTSAK	95.0%	105.0	22.9	85	63	0	2	1,665.78
									EGGQTAPASTR	95.0%	51.5	20.8	4	0	0	2	1,074.52
									EPAHLMSLFGGKPMIIYK	95.0%	47.7	22.0	8	0	3	2	2,064.07
									EPGLQIWR	95.0%	34.4	20.8	1	0	0	2	998.54
									EVQGFESATFLGYFK	95.0%	126.0	22.3	397	0	0	2	1,722.84
									GASQAGAPQGR	95.0%	77.8	20.8	34	0	0	2	999.50
									GGVASGFK	95.0%	56.0	22.5	17	0	0	2	722.38
									HVVPNEVVVQR	95.0%	68.5	20.0	51	28	0	2	1,275.72
									KGGVASGFK	95.0%	63.6	21.0	20	0	0	2	850.48
									KMDAHPPR	95.0%	46.4	22.0	1	0	0	2	967.48
									LKATQVSK	95.0%	46.0	17.8	7	0	0	2	874.54
									MDAHPPR	95.0%	30.6	22.3	1	0	0	2	823.39
									NWRDPDQTDGLGLSYLSSHIANVER	95.0%	90.7	21.3	0	4	7	2	2,843.37
									QTQVSVLPEGGETPLFK	95.0%	90.3	21.2	123	1	0	2	1,829.97
									RTPITVVK	95.0%	39.8	17.0	7	0	0	2	913.58
									RYIETDPANR	95.0%	34.7	23.6	0	2	0	2	1,234.62
									SEDCFILDHGK	95.0%	31.3	19.8	1	0	0	2	1,320.59
									SEDCFILDHGKDGK	95.0%	48.1	19.7	3	2	0	2	1,620.73
									TASDFITK	95.0%	58.8	20.8	15	0	0	2	882.46
									TEALTSAK	95.0%	35.5	23.6	1	0	0	2	820.44
									TGAQELLR	95.0%	78.8	23.7	68	0	0	2	887.50
									TPITVVK	95.0%	52.7	14.9	11	0	0	2	757.48
									TPSAAYLWVGTGASEAEK	95.0%	119.0	22.4	20	0	0	2	1,837.90
									VHVSEEGTEPEAMLQVLGPKPALPAGTEDTAK	95.0%	80.4	19.6	0	19	26	2	3,317.66
									VHVSEEGTEPEAMLQVLGPKPALPAGTEDTAKEDAANR	95.0%	67.4	19.1	0	2	28	2	3,973.95
									VPEARPNSMVVEHPEFLK	95.0%	60.5	22.1	3	69	25	2	2,095.06
									VPFDAATLHTSTAMAAQHGMDDDGTGQK	95.0%	71.1	18.7	0	69	41	2	2,905.27
									VSNGAGTMSVSLVADENPFAQGALK	95.0%	131.0	21.9	28	28	0	2	2,479.21
									YIETDPANR	95.0%	59.1	22.9	32	0	0	2	1,078.52
Peptidyl-prolyl cis-trans isomerase A	PPIA_HUMAN	PPIA	17,995	100.00%	13	18	262	75.20%	EGMNIVEAMER	95.0%	53.5	21.3	13	0	0	2	1,278.58
									FEDENFILK	95.0%	53.0	22.8	2	0	0	2	1,154.57
									HTGPGILSMANAGPNTNGSQFFICTAK	95.0%	73.3	21.6	0	4	0	2	2,807.32
									IIPGFMCQGGDFTR	95.0%	60.8	21.2	5	0	0	2	1,614.74

									KITIADCGQLE	95.0%	46.6	23.8	2	0	0	2	1,247.63
									MVNPTVFFDIAVDGEPLGR	95.0%	114.0	22.6	4	2	0	2	2,077.04
									SIYGEKFEDENFILK	95.0%	88.8	22.5	39	2	0	2	1,831.91
									TEWLDGK	95.0%	35.9	23.0	2	0	0	2	848.42
									TEWLDGKHVVFGK	95.0%	29.5	22.2	0	2	0	2	1,515.80
									VKEGMNIVEAMER	95.0%	93.5	22.9	23	8	0	2	1,521.74
									VNPTVFFDIAVDGEPLGR	95.0%	110.0	21.5	50	22	0	1	1,946.00
									VSFELFADK	95.0%	63.0	20.9	20	0	0	2	1,055.54
									VSFELFADKVPK	95.0%	67.9	20.3	46	16	0	2	1,379.76
Complement C1r subcomponent-like protein	C1RL_HUMAN	C1RL	53,480	100.00%	4	4	13	11.90%	GQESSTDIK	95.0%	36.7	22.4	1	0	0	2	964.46
									GSEAINAPGDNPAK	95.0%	42.3	22.4	7	0	0	2	1,340.64
									LGNFPWQAFTSIHGR	95.0%	36.8	22.1	0	4	0	2	1,730.88
									VVVHPDYR	95.0%	32.2	21.6	1	0	0	2	984.53
									WILTAAHTIYPK	95.0%	57.5	20.0	2	0	0	2	1,413.79
Transcription intermediary factor 1-beta	TIF1B_HUMAN	TRIM28	88,531	100.00%	3	3	7	5.51%	DIVENYFMR	95.0%	31.7	19.4	1	0	0	2	1,202.55
									IVAERPGTNSTGPAPMAPPR	95.0%	37.0	22.5	0	2	0	2	2,035.04
									LSPPYSSPQEFAQDVGR	95.0%	77.4	23.0	4	0	0	2	1,877.90
26S proteasome non-ATPase regulatory subunit 3	PSMD3_HUMAN	PSMD3	60,962	100.00%	6	8	33	14.80%	AVQGFFTSNNATR	95.0%	60.8	22.5	3	0	0	2	1,412.69
									FNQVLDQFGEK	95.0%	48.9	22.3	4	0	0	2	1,324.65
									HDADGQATLLNLLLR	95.0%	107.0	19.8	4	7	0	2	1,649.90
									LQLDSPEDAEFIVAK	95.0%	64.8	22.9	7	0	0	2	1,674.86
									SLMPYFLLTQAVR	94.7%	30.1	20.8	1	0	0	2	1,554.84
									VYEFLDKLDVVR	95.0%	74.3	20.1	2	5	0	2	1,495.82
Enhancer of rudimentary homolog	ERH_HUMAN	ERH	12,241	100.00%	4	4	7	36.50%	ADTQTYQPYNK	95.0%	30.5	20.1	1	0	0	2	1,328.61
									ADTQTYQPYNKDWIK	95.0%	39.5	22.4	2	0	0	2	1,870.90
									IYVLLR	95.0%	36.6	13.2	2	0	0	2	776.50
									TYADYESVNECMEGVCK	95.0%	83.9	8.5	2	0	0	2	2,070.81
ATPase ASNA1	ASNA_HUMAN	ASNA1	38,776	100.00%	2	2	4	8.33%	GMNFSVVVFDTAPTGHTLR	95.0%	44.9	21.8	0	2	0	2	2,065.02
									LLNFPTIVER	95.0%	45.3	19.4	2	0	0	2	1,201.69
Proteasome subunit alpha type-4	PSA4_HUMAN	PSMA4	29,467	100.00%	8	9	43	34.10%	ATCIGNNSAAAVSMLK	95.0%	79.4	22.7	2	0	0	2	1,623.78
									LLDEVFFSEK	95.0%	74.2	22.6	17	0	0	2	1,226.63
									LNEDMACSVAGITSDANVLTNELR	95.0%	133.0	21.5	2	0	0	2	2,609.22
									LSAEKVEIATLTR	95.0%	84.2	18.5	2	3	0	2	1,430.82
									QAYTQFGGK	95.0%	50.6	20.9	3	0	0	2	999.49
									SALALAIK	95.0%	59.0	19.3	6	0	0	2	786.51
									TTIFSPEGR	95.0%	38.8	23.2	4	0	0	2	1,007.52
									VEIATLTR	95.0%	48.3	20.5	4	0	0	2	902.53
N(G),N(G)-dimethylarginine dimethylaminohydrolase 2	DDAH2_HUMAN	DDAH2	29,626	100.00%	4	4	8	27.40%	EFFVGLSK	95.0%	32.9	20.6	1	0	0	2	926.50
									GGGDLPNSQEALQK	95.0%	68.5	22.3	2	0	0	2	1,413.70
									GVPESLASGEGAGAGLPALDLAK	95.0%	109.0	20.8	2	0	0	2	2,080.09
									IVEIGDENATLDGTDVLFTGR	95.0%	111.0	22.5	2	0	0	2	2,235.11
									TVVAGSSDAAQK	95.0%	66.7	23.0	2	0	0	2	1,133.58
Peptidyl-prolyl cis-trans isomerase B	PPIB_HUMAN	PPIB	23,725	100.00%	13	18	85	55.60%	DFMIQGGDFTR	95.0%	62.9	19.3	2	0	0	2	1,286.58
									DKPLKDVIIADCGK	95.0%	76.7	20.9	2	1	0	2	1,571.85
									DTNGSQFFITTVK	95.0%	91.0	22.1	4	0	0	2	1,457.73
									HYGPGWVSMANAGK	95.0%	54.2	21.5	4	2	0	2	1,474.69
									IEVEKPFAIAK	95.0%	38.1	17.3	2	2	0	2	1,244.73
									IEVEKPFAIAKE	95.0%	64.3	20.4	8	4	0	2	1,373.77
									IGDEDVGR	95.0%	45.8	20.4	2	0	0	2	860.41
									LKHYGPGWVSMANAGK	95.0%	32.5	22.0	0	1	0	2	1,731.86
									SIYGERFPDENFK	95.0%	55.2	22.0	3	0	0	2	1,601.76

									TVDNFVALATGEK	95.0%	102.0	22.7	23	0	0	2	1,364.71
									VIFGLFGK	95.0%	59.7	18.7	11	0	0	2	880.53
									VIKDFMIQGGDFTR	95.0%	78.7	22.4	4	7	0	2	1,642.83
									VLEGMEVVR	95.0%	58.8	23.1	3	0	0	2	1,031.56
Thioredoxin	THIO_HUMAN	TXN	11,719	100.00%	5	6	16	42.90%	EKLEATINELV	95.0%	42.8	22.7	4	0	0	2	1,258.69
									MIKPFFHSLSEK	95.0%	56.4	22.5	4	2	0	2	1,479.77
									TAFQEALDAAGDK	95.0%	85.5	22.7	2	0	0	2	1,336.64
									VGEFSGANK	95.0%	44.2	22.5	2	0	0	2	908.45
									VGEFSGANKEK	95.0%	37.5	23.1	2	0	0	2	1,165.59
Transportin-1	TNPO1_HUMAN	TNPO1	102,341	100.00%	8	8	18	9.80%	ALVMLLEVR	95.0%	65.1	19.9	4	0	0	2	1,059.62
									ATVGILITTIASK	95.0%	49.7	12.0	1	0	0	2	1,287.79
									ESQSPDTTIQR	95.0%	39.4	22.1	2	0	0	2	1,261.60
									FSDQFPLPLK	95.0%	45.5	21.9	2	0	0	2	1,191.64
									FSDQFPLPLKER	95.0%	38.5	22.0	0	1	0	2	1,476.79
									GDVEEDETIPDSEQDIRPR	95.0%	36.0	20.3	0	2	0	2	2,200.00
									QSSFALLGDLTK	95.0%	37.2	21.9	2	0	0	2	1,279.69
									TLLENTAITIGR	95.0%	68.3	20.0	4	0	0	2	1,301.74
Serine/threonine-protein phosphatase 5	PPP5_HUMAN	PPP5C	56,862	100.00%	7	7	12	22.40%	AASNMALGK	95.0%	33.4	23.4	1	0	0	2	878.44
									AEGYEVAHGGR	95.0%	47.4	19.6	1	0	0	2	1,145.53
									AFLEENNLDYIIR	95.0%	86.0	21.8	2	0	0	2	1,609.82
									FYSQAIELNPSNAIYYGNR	95.0%	101.0	21.8	2	0	0	2	2,220.07
									GNHETDNMNQIYGFEGEVK	95.0%	47.4	17.1	0	2	0	2	2,197.95
									SVVDSLDIESMTIEDEYSGPK	95.0%	118.0	19.8	2	0	0	2	2,330.06
									VLIMHGGLFSEDGVTLDDIR	95.0%	39.8	21.6	0	2	0	2	2,203.11
Small nuclear ribonucleoprotein Sm D1	SMD1_HUMAN	SNRPD1	13,264	100.00%	2	4	10	27.70%	NREPVQLETLSIR	95.0%	56.8	20.1	2	2	0	2	1,554.86
									YFILPDSLPLDTLLVDVEPK	95.0%	77.8	17.0	4	2	0	2	2,287.25
N-acetylgalactosamine kinase	GALK2_HUMAN	GALK2	50,361	99.50%	2	2	2	5.02%	LIEFSPLR	95.0%	36.5	20.4	1	0	0	2	974.57
									TQILSPNTQDVLIFK	95.0%	30.9	19.1	1	0	0	2	1,716.95
Ubiquitin-like domain-containing CTD phosphatase 1	UBCP1_HUMAN	UBLCP1	36,788	100.00%	5	5	8	14.80%	EIAKLDDFLDLNHK	95.0%	39.5	21.6	0	2	0	2	1,670.88
									LDDFLDLNHK	95.0%	26.1	22.2	0	1	0	2	1,229.62
									MKELGVSTNANYK	95.0%	59.4	22.0	2	0	0	2	1,470.73
									NFLMNPQNGLK	95.0%	36.1	23.5	1	0	0	2	1,291.65
									TLTGVLPER	95.0%	39.8	18.6	2	0	0	2	985.57
L-lactate dehydrogenase A chain	LDHA_HUMAN	LDHA	36,671	100.00%	17	21	321	47.00%	DLADELALVDVIEDK	95.0%	135.0	21.3	76	6	0	2	1,657.85
									DLADELALVDVIEDKLK	95.0%	81.4	18.5	15	1	0	2	1,899.03
									DQLIYNLLK	95.0%	61.7	20.2	10	0	0	2	1,119.64
									DQLIYNLLKEEQTPQNK	95.0%	103.0	21.9	10	9	0	2	2,074.08
									DYNVTANSK	95.0%	64.8	21.6	7	0	0	2	1,011.47
									FIIPNVVK	95.0%	44.9	17.9	4	0	0	2	929.58
									GEMMDLQHGSLFLR	95.0%	54.8	21.6	4	4	0	2	1,665.77
									LKGEMMDLQHGSLFLR	95.0%	33.9	22.2	0	0	20	2	1,906.95
									LLIVSNPVDILTYVAWK	95.0%	86.5	13.0	3	0	0	2	1,944.12
									LNLVQR	95.0%	39.7	18.8	28	0	0	2	742.46
									LVIITAGAR	95.0%	73.7	17.0	41	0	0	2	913.58
									NVNIFK	95.0%	35.0	22.0	5	0	0	2	734.42
									QVVESAYEVIK	95.0%	79.3	21.8	19	0	0	2	1,264.68
									RVHPVSTMIK	95.0%	29.4	19.4	0	3	0	2	1,183.66
									SADTLWGIQK	95.0%	52.2	23.0	5	0	0	2	1,118.58
									VIGSGCNLDSAR	95.0%	69.6	21.7	6	0	0	2	1,248.60
									VTLTSEEEAR	95.0%	79.2	23.7	45	0	0	2	1,134.56
Cullin-3	CUL3_HUMAN	CUL3	88,914	100.00%	5	5	7	7.55%	ALVSEEGEGKNPVDYIQGLLDLK	95.0%	56.2	20.6	0	2	0	2	2,487.30

									DMSISNTTMDEFR	95.0%	39.7	13.2	1	0	0	2	1,578.64
									FLLESFNNDR	95.0%	38.8	22.3	1	0	0	2	1,254.61
									NPVDYIQGLLDLK	95.0%	47.6	20.9	1	0	0	2	1,487.81
									SPEYLSLFIDDK	95.0%	73.1	22.2	2	0	0	2	1,426.71
Nucleophosmin	NPM_HUMAN	NPM1	32,557	100.00%	12	17	389	42.20%	ADKDYHFK	95.0%	41.1	21.9	3	0	0	2	1,023.49
									DELHIVEAEAMNYEGSPIK	95.0%	87.5	21.7	4	11	0	2	2,161.01
									GPSSVEDIK	95.0%	64.7	21.7	32	0	0	2	931.47
									GPSSVEDIKAK	95.0%	72.4	23.1	8	4	0	2	1,130.61
									LLSISGK	95.0%	35.0	22.0	2	0	0	2	717.45
									MQASIEK	95.0%	41.2	23.9	8	0	0	2	822.40
									MSVQPTVSLGGFEITPPVVLR	95.0%	119.0	19.2	137	29	0	2	2,243.21
									MTDQEAIQDLWQWR	95.0%	66.6	24.6	3	0	0	2	1,835.84
									SKGQESFK	95.0%	31.8	21.5	1	0	0	2	910.46
									TVSLGAGAKDELHIVEAEAMNYEGSPIK	95.0%	95.8	20.8	0	97	7	2	2,945.46
									VDNDENEHQLSLR	95.0%	89.3	21.7	28	1	0	2	1,568.73
									VTLATLK	95.0%	41.8	15.1	14	0	0	2	745.48
Lysosomal alpha-glucosidase	LYAG_HUMAN	GAA	105,301	100.00%	16	18	113	23.10%	AGYIIPLQGPGLTTTESR	95.0%	93.5	20.8	20	0	0	2	1,874.00
									DAQAHPGRPR	95.0%	37.6	23.5	1	0	0	2	1,104.57
									DFPAMVQELHQGGR	95.0%	52.2	20.9	6	0	0	2	1,600.75
									DGFRDFPAMVQELHQGGR	95.0%	28.8	22.3	0	1	1	2	2,075.97
									ELSGSSPVLEETHPAHQQGASRPGPR	95.0%	55.8	20.9	0	1	0	2	2,724.35
									GAYTQVIFLAR	95.0%	72.7	19.5	14	0	0	2	1,238.69
									LDVMMETENR	95.0%	51.2	18.4	13	0	0	2	1,269.55
									NHNSLLSLPQEPYSFSEPAQQAMR	95.0%	73.2	21.0	0	17	0	2	2,760.31
									QQPMALAVALTK	95.0%	42.0	21.9	5	0	0	2	1,286.71
									RYEVPLETPHVHSR	95.0%	39.1	22.0	0	4	0	2	1,719.89
									STFAGHGR	95.0%	46.6	21.0	5	0	0	2	832.41
									VTSEGAGLQLQK	95.0%	75.9	23.0	11	0	0	2	1,230.67
									VTVLGVATAPQQVLSNGVPVSNFTYSPDTK	95.0%	26.6	18.8	0	2	0	2	3,089.62
									YEVPLETPHVHSR	95.0%	41.0	23.0	2	0	1	2	1,563.79
									YMMIVDPAISSSGPAGSYRPYDEGLR	95.0%	37.3	19.9	0	4	0	2	2,864.32
									YMMIVDPAISSSGPAGSYRPYDEGLRR	95.0%	34.3	20.4	0	0	5	2	3,020.42
Sec1 family domain-containing protein 1	SCFD1_HUMAN	SCFD1	72,364	99.50%	2	2	4	4.21%	FGQDIISPLLSVK	95.0%	52.7	19.1	2	0	0	2	1,416.81
									LTSAVSSLPELLEK	95.0%	59.0	20.3	2	0	0	2	1,486.84
Lamin-B2	LMNB2_HUMAN	LMNB2	67,672	100.00%	6	7	24	12.70%	ALELENDRLLLK	95.0%	41.6	17.1	2	0	0	2	1,426.83
									ALYESELADAR	95.0%	53.8	22.2	5	0	0	2	1,237.61
									GLESDVAELR	95.0%	79.8	23.8	2	0	0	2	1,088.56
									ISEKEEVTTR	95.0%	64.1	23.0	7	4	0	2	1,191.62
									KLLEGEEER	95.0%	38.7	23.0	1	0	0	2	1,102.57
									LLEGEEER	95.0%	34.4	22.0	2	0	0	2	974.48
									LQEKEELR	95.0%	43.5	22.3	2	0	0	2	1,044.57
									MAQALEELR	95.0%	36.7	23.3	2	0	0	2	1,076.54
									VLDETAR	95.0%	38.7	25.0	2	0	0	2	803.43
Eukaryotic initiation factor 4A-II	IF4A2_HUMAN	EIF4A2	46,386	100.00%	5	6	11	34.60%	DFTVSALHGDMDQK	95.0%	49.3	18.9	2	0	0	2	1,579.71
									DFTVSALHGDMDQKER	95.0%	31.0	21.8	0	1	0	2	1,864.85
									ETQALVLAPTR	95.0%	46.1	21.1	2	0	0	2	1,198.68
									GFKDQIYEIFQK	95.0%	38.2	22.5	0	1	0	2	1,515.79
									GIDVQQVSLVINYDLPTNR	95.0%	77.2	20.3	2	0	0	2	2,144.14
									GIYAYGFEKPSAIQQR	95.0%	93.3	21.2	4	4	0	2	1,827.94
									GYDVIAQAQSGTGK	95.0%	77.8	22.7	2	0	0	2	1,394.69
									LQAEAPHIVVGTPGR	95.0%	67.4	20.5	2	3	0	2	1,544.86

									MFVLDEADEMLSR	95.0%	95.8	19.4	8	0	0	2	1,587.70
									QFYINVER	95.0%	34.1	22.3	1	0	0	2	1,068.55
									VFDMLNR	95.0%	31.5	22.0	1	0	0	2	910.45
									VLITTDLLAR	95.0%	67.3	17.6	19	0	0	2	1,114.68
Seizure 6-like protein 2	SE6L2_HUMAN	SEZ6L2	97,484	100.00%	4	4	20	6.81%	EGDMLTLFDGDGPSAR	95.0%	84.1	19.0	6	0	0	2	1,696.75
									LLLHFQSPR	95.0%	40.8	17.3	3	0	0	2	1,110.64
									SGGSPLSPVIYDSDMDDVPER	95.0%	106.0	19.8	7	0	0	2	2,252.00
									TASDAGFPVGSHVQYR	95.0%	49.0	23.1	0	4	0	2	1,691.81
Aspartyl aminopeptidase	DNPEP_HUMAN	DNPEP	52,411	100.00%	7	7	17	22.70%	GTPEPGPLNAVDER	95.0%	45.7	22.5	1	0	0	2	1,451.71
									LEQQLVHVERPILR	95.0%	43.4	12.8	0	2	0	2	1,730.01
									LLQAGFSELKETEK	95.0%	56.9	20.9	1	0	0	2	1,592.85
									NINENFGPNTEMHLVPILATAIQEELEK	95.0%	57.5	20.9	0	4	0	2	3,180.59
									VKVPLQDLMVR	95.0%	51.2	19.1	0	3	0	2	1,313.76
									VLDLGSPQLAMHSIR	95.0%	65.7	21.3	2	0	0	2	1,652.88
									YASNAVSEALIR	95.0%	68.9	22.9	4	0	0	2	1,293.68
Xaa-Pro dipeptidase	PEPD_HUMAN	PEPD	54,530	100.00%	5	5	19	11.60%	AVYEAVLR	95.0%	45.6	21.0	2	0	0	2	920.52
									FEVNNTILHPEIVECR	95.0%	51.9	21.7	0	2	0	2	1,969.98
									TDMELEVLR	95.0%	73.5	24.4	2	0	0	2	1,121.55
									TVEEIEACMAGCDK	95.0%	101.0	15.8	2	0	0	2	1,628.66
									VPLALFALNR	95.0%	73.7	16.9	11	0	0	2	1,113.68
Heterogeneous nuclear ribonucleoprotein D0	HNRPD_HUMAN	HNRNPD	38,417	100.00%	6	8	169	22.80%	ESESVDKVMDQK	95.0%	45.4	20.5	12	4	0	2	1,410.64
									EYFGGFGEVESIELPMDNK	95.0%	56.6	20.2	3	0	0	2	2,176.98
									FGEVVDCTLK	95.0%	62.7	22.7	10	0	0	2	1,167.57
									GFGFVLFK	95.0%	46.7	22.9	31	0	0	2	914.51
									IDASKNEEDEGHSNSSPR	95.0%	93.9	18.9	17	60	0	2	1,971.86
									IFVGGLSPDTPEEK	95.0%	88.4	23.0	32	0	0	2	1,488.76
Filamin-B	FLNB_HUMAN	FLNB	278,141	100.00%	62	71	582	34.60%	AAGSGELGVTMK	95.0%	70.5	23.0	12	0	0	2	1,136.56
									ADIEMPFDPSK	95.0%	59.2	19.9	11	0	0	2	1,265.57
									AGGPGLER	95.0%	75.8	20.7	19	0	0	2	756.40
									AGPGTLSVTIEGPSK	95.0%	93.4	21.5	14	0	0	2	1,413.76
									AHGPGLEGGLVGKPAEFTIDTK	95.0%	35.4	18.8	0	1	11	2	2,194.15
									APLNVQFNSPLPGDAVK	95.0%	72.6	20.8	22	0	0	2	1,766.94
									APSVATVGSICDLNLK	95.0%	88.4	21.6	4	0	0	2	1,644.86
									DAGEGLLAVQITDQEGKPK	95.0%	97.9	21.6	10	1	0	2	1,969.02
									DAGYGGISLAVEGPSK	95.0%	70.2	23.1	6	0	0	2	1,520.76
									DGTYAVTYVPLTAGMYTLTMK	95.0%	70.0	21.3	5	6	0	2	2,328.11
									DLDIIDNYDYSHTVK	95.0%	76.3	21.0	10	0	0	2	1,810.85
									EAFTNKPNVFTVVTR	95.0%	48.0	20.9	0	5	0	2	1,722.92
									EATTDFTVDSRPLTQVGGDHIK	95.0%	60.1	21.7	0	12	0	2	2,387.18
									FADEHVPGSPFTVK	95.0%	29.5	21.8	0	6	0	2	1,530.76
									FNDEHIPESPYLVPVIAPSDDAR	95.0%	30.9	21.5	0	1	0	2	2,581.26
									FVPQEMGVHTVSVK	95.0%	74.2	23.6	9	0	0	2	1,573.80
									GAGIGGLGITVEGPSESK	95.0%	82.9	22.0	20	0	0	2	1,628.85
									GIEPTGNMVK	95.0%	45.0	22.4	2	0	0	2	1,061.53
									GLVEPVNVVDNGDGTHTVTYTPSQEGPYMVSVK	95.0%	83.1	19.2	0	11	0	2	3,505.68
									GQHVTGSPFQFTVGPLGEGGAHK	95.0%	34.1	22.0	0	0	8	2	2,308.15
									IAGPGLGSGVR	95.0%	72.0	18.3	10	0	0	2	983.56
									IFAQDGEGQR	95.0%	49.8	22.8	10	0	0	2	1,120.54
									IFFAGDTIPK	95.0%	57.4	21.2	24	0	0	2	1,108.60
									IGNLQTDLSDGLR	95.0%	66.0	22.5	6	0	0	2	1,401.73
									IKVFGPGIEGK	95.0%	44.1	18.6	0	6	0	2	1,144.67

									IPEINSSDMSAHVTSPSGR	95.0%	52.9	21.3	0	18	0	2	2,000.93
									KGEITGEVHMPSGK	95.0%	42.4	21.8	0	4	0	2	1,485.74
									LDVTILSPSR	95.0%	85.0	22.7	15	0	0	2	1,100.63
									LDVTILSPSRK	95.0%	33.2	18.7	0	2	0	2	1,228.73
									LIALLEVLSQK	95.0%	62.2	13.4	21	0	0	2	1,226.77
									LKPGAPLKPK	95.0%	27.7	7.0	0	1	0	2	1,048.69
									LPNNHIGISFIPR	95.0%	47.8	17.7	0	9	0	2	1,477.83
									LTVMSLQESGLK	95.0%	60.2	21.9	2	0	0	2	1,321.70
									LVSPGSANETSSILVESVTR	95.0%	123.0	21.4	7	0	0	2	2,046.07
									NGNHVANSPVSIMVVQSEIGDAR	95.0%	44.8	22.1	0	1	0	2	2,410.18
									NTVELLVEDK	95.0%	31.3	24.0	1	0	0	2	1,159.62
									QMQLENVSVALEFLDR	95.0%	40.9	22.5	1	0	0	2	1,907.95
									SPFEVQVGPEAGMQK	95.0%	89.8	21.2	18	0	0	2	1,619.77
									SPFTVGVAAPLDLSK	95.0%	102.0	21.2	18	0	0	2	1,501.83
									TATPEIVDNKDGTVTVR	95.0%	81.8	20.9	2	0	0	2	1,815.95
									TFEMSDFIVDTR	95.0%	63.2	21.3	10	0	0	2	1,476.67
									TGEEVGFVVDAK	95.0%	81.1	22.7	7	0	0	2	1,250.63
									VEVGKDQEFTVDTR	95.0%	53.3	22.8	4	11	0	2	1,622.80
									VFGPGIEGK	95.0%	42.1	22.7	2	0	0	2	903.49
									VKAEGPGLSK	95.0%	32.2	18.6	2	0	0	2	985.57
									VKVDPSHDASK	95.0%	27.9	21.9	0	2	0	2	1,182.61
									VLFASQEIPASPFR	95.0%	82.0	22.5	23	0	0	2	1,561.84
									VLQSFTVDSSK	95.0%	64.8	22.6	2	0	0	2	1,210.63
									VLSEDEEDVDFDIIHNANDTFTVK	95.0%	46.8	20.4	0	3	0	2	2,765.28
									VMYTPMAPGNYLISVK	95.0%	68.1	22.3	11	0	0	2	1,815.90
									VNIGQGSHPQK	95.0%	36.8	23.4	3	1	0	2	1,164.61
									VNQPASFAIR	95.0%	51.5	23.5	8	0	0	2	1,102.60
									VPVKDVVDPSK	95.0%	59.7	19.4	6	0	0	2	1,182.67
									VSYFPTVPGVYIVSTK	95.0%	51.0	20.3	17	0	0	2	1,756.95
									VTASGPGLSSYGVPASLPVDFAIDAR	95.0%	59.7	20.3	4	6	0	2	2,547.31
									VTEAEIVPMGK	95.0%	53.4	23.2	8	0	0	2	1,189.61
									VVASGPGLEHGK	95.0%	51.1	21.6	9	6	0	2	1,150.62
									YADEEIPR	95.0%	48.3	22.1	9	0	0	2	992.47
									YGGELVPHFPAR	95.0%	37.8	21.7	4	8	0	2	1,342.69
									YGGPNHIVGSPFK	95.0%	49.5	22.3	5	0	0	2	1,372.70
									YMIGVTYGGDDIPLSPYR	95.0%	85.6	21.0	16	0	0	2	2,032.97
									YTPTQQGNMQVLVTYGGDPIPK	95.0%	96.7	21.0	9	4	0	2	2,423.19
60S ribosomal protein L5	RL5_HUMAN	RPL5	34,346	100.00%	11	14	94	38.40%	DIICQIAYAR	95.0%	53.0	23.2	11	0	0	2	1,222.63
									ENPVYEK	95.0%	31.9	22.3	2	0	0	2	878.43
									GAVDGGLSIPHSTK	95.0%	90.0	22.6	10	5	0	2	1,338.70
									GAVDGGLSIPHSTKR	95.0%	29.4	21.6	0	1	0	2	1,494.80
									HIMGQNVADYMR	95.0%	42.0	19.9	11	0	0	2	1,466.65
									IEGDMIVCAAYAHELPK	95.0%	55.7	22.4	1	4	0	2	1,932.92
									NSVTPDMMEEMYK	95.0%	62.2	12.0	7	0	0	2	1,622.64
									NSVTPDMMEEMYKK	95.0%	33.8	14.9	3	0	0	2	1,750.73
									RFPGYDSESK	95.0%	44.4	21.2	7	0	0	2	1,185.55
									VGLTNYAAAYCTGLLLAR	95.0%	102.0	21.1	7	0	0	2	1,927.01
									YLMEEDEDAYKK	95.0%	61.2	17.6	10	15	0	2	1,549.67
Single-stranded DNA-binding protein, mitochondrial	SSBP_HUMAN	SSBP1	17,242	99.90%	2	2	4	20.30%	NPVTIFSLATNEMWR	95.0%	50.1	22.9	2	0	0	2	1,794.89
									SGDSEVYQLGDVSQK	95.0%	105.0	21.4	2	0	0	2	1,611.75
Pre-mRNA-processing-splicing	PRP8_HUMAN	PRPF8	273,591	100.00%	5	5	15	3.08%	AISAANLHLR	95.0%	33.8	18.3	1	0	0	2	1,065.62

factor 8									AQIAGYLYGVSPPDNPQVK	95.0%	63.4	21.8	2	0	0	2	2,017.04
									EAVVNTQELLDLLVK	95.0%	77.4	18.5	1	0	0	2	1,683.95
									ISLIQIFR	95.0%	61.3	13.8	10	0	0	2	989.61
									TDMIQALGGVEGILEHTLFK	95.0%	26.8	20.3	0	1	0	2	2,188.13
Ubiquitin-conjugating enzyme E2 N	UBE2N_HUMAN	UBE2N	17,121	100.00%	3	3	11	23.70%	LELFLPEEYPMAAPK	95.0%	47.5	22.4	4	0	0	2	1,763.89
									LLAEPVPGIK	95.0%	36.9	11.8	2	0	0	2	1,036.64
									TNEAQAIETAR	95.0%	64.3	23.2	5	0	0	2	1,203.60
Glycoprotein endo-alpha-1,2-mannosidase-like protein	MANEL_HUMAN	MANEAL	51,301	100.00%	3	3	11	8.10%	APDGLPALGPGLELAPFER	95.0%	60.4	19.8	4	0	0	2	1,920.02
									ISASYPR	95.0%	37.3	23.6	1	0	0	2	793.42
									RPEGAPAPAAR	95.0%	44.4	20.7	6	0	0	2	1,092.59
Transcriptional activator protein Pur-alpha	PURA_HUMAN	PURA	34,893	100.00%	3	4	5	18.30%	GPGLGSTQGQTIALPAQGLIEFR	95.0%	110.0	18.7	2	1	0	2	2,311.24
									IAEVGAGGNK	95.0%	31.3	21.6	1	0	0	2	915.49
									LIDDYGVEEEPAELPEGTSLTVDNKR	95.0%	49.1	21.3	0	1	0	2	2,889.40
Poly(U)-binding-splicing factor PUF60	PUF60_HUMAN	PUF60	59,858	100.00%	3	3	11	12.30%	AVTPPMPLLTPATPGGLPPAAAVAAAAATAK	95.0%	33.0	14.6	0	2	0	2	2,810.55
									LGLPPLTPEQQEALQK	95.0%	49.2	17.9	3	0	0	2	1,761.98
									VGRPSNIGQAQPIIDQLAEEAR	95.0%	74.7	19.4	0	6	0	2	2,362.25
NEDD8-activating enzyme E1 regulatory subunit	ULA1_HUMAN	NAE1	60,230	100.00%	3	3	5	8.80%	LLQSIGQAPESISEK	95.0%	31.6	21.5	1	0	0	2	1,599.86
									NENGAPEDEENFEEAIK	95.0%	63.5	16.9	2	0	0	2	1,934.83
									YPGVSNYQVEEDIGK	95.0%	73.3	21.3	2	0	0	2	1,697.80
Ceruloplasmin	CERU_HUMAN	CP	122,190	100.00%	13	13	52	16.50%	ALYLQYTDETFR	95.0%	75.3	23.4	3	0	0	2	1,519.74
									DIASGLIGPLIICK	95.0%	58.8	19.3	2	0	0	2	1,469.84
									DIFTGLIGPMK	95.0%	46.5	22.9	4	0	0	2	1,207.64
									DLYSGLIGPLIVCR	95.0%	54.3	21.0	2	0	0	2	1,575.86
									EVGPTNADPVCLAK	95.0%	33.7	22.3	1	0	0	2	1,470.73
									EYTDASFTNR	95.0%	56.5	17.3	4	0	0	2	1,203.53
									GAYPLSIEPIGVR	95.0%	62.9	21.6	17	0	0	2	1,371.76
									IYHSHIDAPK	95.0%	36.4	23.4	0	3	0	2	1,180.61
									LISVDTEHSNIYLQNGPDR	95.0%	49.4	21.8	0	2	0	2	2,171.07
									MFTTAPDQVDKEDEDFQESNK	95.0%	52.7	15.7	0	5	0	2	2,490.06
									MYYSAVDPTKDIFTGLIGPMK	95.0%	27.3	21.8	0	2	0	2	2,379.16
									NNEGTYYSPNYNPQSR	95.0%	51.8	17.5	3	0	0	2	1,903.82
									QSEDSTFYLGER	95.0%	97.5	20.1	4	0	0	2	1,431.64
Beta-2-microglobulin	B2MG_HUMAN	B2M	13,697	100.00%	5	10	81	42.90%	IEKVEHSDLSFSK	95.0%	90.8	23.0	2	2	3	2	1,518.78
									IQVYSR	95.0%	41.1	21.9	13	0	0	2	765.43
									SNFLNCYVSGFHPSDIEVDLLK	95.0%	41.1	21.5	5	8	0	2	2,554.23
									VEHSDLSFSK	95.0%	59.0	22.8	9	3	0	2	1,148.56
									VNHVTLSQPK	95.0%	53.2	20.5	25	11	0	2	1,122.63
Adenosylhomocysteinase	SAHH_HUMAN	AHCY	47,699	100.00%	19	27	185	41.40%	ALDIAENEMPGLMR	95.0%	88.7	21.8	6	1	0	2	1,591.75
									ATDVMIAGK	95.0%	65.0	23.3	4	0	0	2	921.47
									DGPLNMILDDGGDLTNLIHTK	95.0%	96.0	22.1	6	33	0	2	2,268.12
									ESLIDGIK	95.0%	35.8	23.6	2	0	0	2	874.49
									ESLIDGIKR	95.0%	46.2	20.9	5	0	0	2	1,030.59
									GCAQALR	95.0%	45.0	24.6	2	0	0	2	775.39
									GISEETTTGVHNLYK	95.0%	81.0	23.4	15	5	0	2	1,648.82
									IILLAEGR	95.0%	60.2	16.3	11	0	0	2	884.56
									KALDIAENEMPGLMR	95.0%	65.2	21.8	3	5	0	2	1,719.84
									KLDEAVAEAHLGK	95.0%	95.3	21.3	2	6	0	2	1,380.75
									LDEAVAEAHLGK	95.0%	69.4	22.2	2	2	0	2	1,252.65
									SKFDNLYGCR	95.0%	36.3	19.8	2	0	0	2	1,259.58
									VADIGLAAWGR	95.0%	85.9	22.3	2	0	0	2	1,128.62
									VAVVAGYGDVGK	95.0%	88.5	21.8	7	0	0	2	1,134.62

									VNIKPQVDR	95.0%	39.8	18.7	3	2	0	2	1,068.62
									VPAINVNDSVTK	95.0%	87.3	22.2	32	0	0	2	1,256.69
									WLNENAVEK	95.0%	32.3	22.6	1	0	0	2	1,102.55
									YPQLLPGIR	95.0%	49.6	20.4	24	0	0	2	1,056.62
									YPVGVHFLPK	95.0%	35.6	21.0	1	1	0	2	1,156.65
Aspartyl-tRNA synthetase, cytoplasmic	SYDC_HUMAN	DARS	57,119	100.00%	10	10	37	24.80%	ATVNQDTR	95.0%	35.3	23.2	2	0	0	2	904.45
									EAGVEMGDEDDLSTPNEK	95.0%	74.8	14.9	3	0	0	2	1,951.81
									FGAPPHAGGGIGLER	95.0%	47.8	22.1	0	3	0	2	1,435.74
									FQTEIQTVNK	95.0%	51.8	23.3	5	0	0	2	1,207.63
									GFVEIQTPK	95.0%	36.9	22.4	2	0	0	2	1,018.56
									IYVISLAEPR	95.0%	55.1	19.4	6	0	0	2	1,160.67
									LPLQLDDAVRPEAEGEEEGR	95.0%	40.2	22.4	0	5	0	2	2,223.09
									NNAYLAQSPQLYK	95.0%	80.7	22.7	5	0	0	2	1,509.77
									VFSIGPVFR	95.0%	44.7	19.4	5	0	0	2	1,021.58
									VTMLFLGLHNVR	95.0%	26.4	20.8	0	1	0	2	1,415.78
Nuclear migration protein nudC	NUDC_HUMAN	NUDC	38,226	100.00%	6	8	23	21.10%	ELTDEEAER	95.0%	39.2	19.2	3	0	0	2	1,091.49
									FMDQHPEMDFSK	95.0%	33.5	12.6	1	1	0	2	1,543.62
									LKPNLGNGADLPNYR	95.0%	53.3	21.2	1	4	0	2	1,641.87
									LSDLDSETR	95.0%	48.2	22.6	2	0	0	2	1,035.50
									LVSSDPEINTK	95.0%	56.3	23.9	9	0	0	2	1,202.63
									TDFFIGGEEGMAEK	95.0%	46.7	18.5	2	0	0	2	1,546.67
	PLOD3_HUMAN	PLOD3	84,769	100.00%	18	20	133	32.50%	FLNSGGFIGFATTIHQIVR	95.0%	38.7	18.9	0	4	0	2	2,078.12
									GIFLHLSNQHEFGR	95.0%	36.4	21.9	0	4	0	2	1,654.85
									GRDPVNPEK	95.0%	31.5	22.1	1	0	0	2	1,011.52
									IFQNLNGALDEVVLK	95.0%	103.0	19.6	15	0	0	2	1,672.93
									KFVQSGSR	95.0%	43.0	21.1	2	0	0	2	908.50
									LAGGYENVPTVDIHMK	95.0%	63.9	22.4	4	5	0	2	1,759.87
									LLLLDYPPDR	95.0%	61.5	22.3	21	0	0	2	1,214.68
									LLVITVATAETEGYLR	95.0%	56.0	18.1	5	0	0	2	1,748.98
									LSLNLDHK	95.0%	50.7	17.4	4	0	0	2	939.53
									LVGPEEALSPGEAR	95.0%	68.7	21.7	13	0	0	2	1,424.74
									LYLDPGLR	95.0%	51.0	21.7	6	0	0	2	946.54
									NVAYDTLPIVVHGNGPTK	95.0%	106.0	21.0	4	0	0	2	1,895.00
									QVGYEDQWLQLLR	95.0%	55.3	23.2	2	0	0	2	1,647.85
									SEDYVELVQR	95.0%	71.5	22.2	18	0	0	2	1,237.61
									TYVGPMTESLFPGYHTK	95.0%	52.7	21.8	4	5	0	2	1,943.92
									VFLAVFVEQPTPFLPR	95.0%	83.8	18.2	9	0	0	2	1,860.04
									VGVWNVPYISQAYVIR	95.0%	42.5	19.6	1	0	0	2	1,864.01
									YKDDDDDQLFYTR	95.0%	86.7	19.9	6	0	0	2	1,693.73
	TLN1_HUMAN	TLN1	269,747	100.00%	24	27	127	16.50%	AVAEQIPLLVQGVR	95.0%	59.7	14.1	6	0	0	2	1,492.89
									AVSSAIAQLLGEVAQGNENYAGIAAR	95.0%	28.5	20.5	0	1	0	2	2,573.33
									DLDQASLAAVSQQLAPR	95.0%	49.5	21.4	2	0	0	2	1,782.94
									EVANSTANLVK	95.0%	40.8	23.5	1	0	0	2	1,145.62
									GLAGAVSELLR	95.0%	81.2	20.6	15	0	0	2	1,085.63
									GSQAQPDSPSAQLALIAASQSFLQPGGK	95.0%	42.2	19.6	0	5	0	2	2,754.41
									GVAALTSDPAVQAIVLDTASDVLDK	95.0%	27.4	20.0	0	1	0	2	2,469.31
									GVGAAATAVTQALNELLQHVK	95.0%	36.1	16.6	0	3	0	2	2,091.16
									ILAQATSDLVNAIK	95.0%	78.7	18.0	13	0	0	2	1,456.84
									IPEAPAGPPSDFGLFLSDDDPKK	95.0%	31.3	21.8	0	1	0	2	2,413.19
									LAQAAQSSVATITR	95.0%	104.0	21.1	5	0	0	2	1,416.78
									LGAASLGAEDPETQVVLINAVK	95.0%	81.6	18.3	4	0	0	2	2,195.19

									LLAALLEDEGGSGRPLLQAAK	95.0%	56.7	16.2	1	9	0	2	2,122.19
									LNEAAAGLNQAATELVQASR	95.0%	128.0	21.1	6	1	0	2	2,027.05
									NGNLPEFGDAISTASK	95.0%	50.3	23.1	1	0	0	2	1,620.79
									NLGTALAELR	95.0%	64.0	21.6	6	0	0	2	1,057.60
									QAAASATQTIAAAQHAASTPK	95.0%	40.6	21.6	0	4	0	2	1,995.03
									QEDVIATANLSR	95.0%	42.5	23.7	2	0	0	2	1,316.68
									TLAESALQLLYTAK	95.0%	77.7	20.0	19	0	0	2	1,521.85
									TLSHPQQMALLDQTK	95.0%	31.2	21.5	0	1	0	2	1,726.88
									TMLESAGGLIQTAR	95.0%	84.4	23.4	3	0	0	2	1,463.75
									VAGSVTELIQAAEAMK	95.0%	75.2	22.1	2	0	0	2	1,633.85
									VLVQNAAGSQEK	95.0%	58.4	22.2	5	0	0	2	1,243.66
									VSQMAQYFEPLTLAAVGAASK	95.0%	77.6	21.3	6	4	0	2	2,198.12
60S ribosomal protein L24	RL24_HUMAN	RPL24	17,762	99.50%	2	2	4	13.40%	AITGASLADIMAK	95.0%	79.6	22.8	2	0	0	2	1,277.68
									VFQFLNAK	95.0%	41.0	20.9	2	0	0	2	966.54
Nucleotide exchange factor SIL1	SIL1_HUMAN	SIL1	52,068	100.00%	4	4	18	12.10%	LLVILATEQPLTAK	95.0%	77.2	11.5	6	0	0	2	1,509.93
									MFAEEEAELTQEMSPEK	95.0%	81.1	16.2	1	0	0	2	2,030.86
									VQVEAIEGGALQK	95.0%	67.2	20.4	2	0	0	2	1,341.74
									VVTLLYDLVTEK	95.0%	77.0	17.3	9	0	0	2	1,392.80
RuvB-like 1	RUVB1_HUMAN	RUVBL1	50,211	100.00%	14	19	79	41.40%	ALESSIAPIVIFASNR	95.0%	94.9	19.8	6	8	0	2	1,687.94
									AQTEGINISEEALNHLGEIGTK	95.0%	102.0	21.3	2	4	0	2	2,324.17
									AVLLAGPPGTGK	95.0%	53.5	16.7	2	0	0	2	1,080.64
									EACGVIVELIK	95.0%	51.3	22.9	2	0	0	2	1,230.68
									GLGLDESGLAK	95.0%	58.8	23.1	6	0	0	2	1,059.57
									GTEDITSPHGIPLDLLDR	95.0%	72.9	22.2	4	6	0	2	1,949.00
									KTEVLMENFR	95.0%	66.9	22.4	2	1	0	2	1,282.65
									LDPSIFESLQK	95.0%	67.3	23.2	4	0	0	2	1,276.68
									QAASGLVGQENAR	95.0%	64.1	22.1	6	0	0	2	1,300.66
									TALALAIAQELGSK	95.0%	120.0	17.9	9	0	0	2	1,385.80
									TISHVIIGLK	95.0%	42.5	10.8	4	2	0	2	1,080.68
									TMLYTPQEMK	95.0%	41.9	19.9	4	0	0	2	1,273.58
									VPFCPMVGSEVYSTEIK	95.0%	74.1	21.6	1	0	0	2	1,958.92
									YSVQLLTPANLLAK	95.0%	60.5	16.2	6	0	0	2	1,530.89
Cysteinyl-tRNA synthetase, cytoplasmic	SYCC_HUMAN	CARS	85,458	100.00%	7	7	18	11.10%	APVDITGQFEK	95.0%	47.2	23.7	4	0	0	2	1,204.62
									DNILPELGVR	95.0%	32.0	20.3	2	0	0	2	1,125.63
									EQKVPEILQLSDALR	95.0%	37.7	19.0	0	1	0	2	1,738.97
									FLNEFFLNVK	95.0%	44.7	21.5	5	0	0	2	1,270.68
									IQHAVQLATEPLEK	95.0%	43.4	20.2	1	0	0	2	1,576.87
									SYISFDILR	95.0%	35.9	21.7	3	0	0	2	1,113.59
									VSEYVPEIVNFVQK	95.0%	66.5	21.0	2	0	0	2	1,650.87
V-type proton ATPase catalytic subunit A	VATA_HUMAN	ATP6V1A	68,287	100.00%	9	11	18	20.90%	AVETTAQSDNK	95.0%	54.6	22.5	2	0	0	2	1,163.55
									DMGYHVSMMADSTSR	95.0%	35.3	12.6	1	0	0	2	1,735.67
									EILQEEEDLAEIVQLVGK	95.0%	102.0	19.3	2	0	0	2	2,055.09
									LAEMPADSGYPAYLGAR	95.0%	77.8	21.8	2	0	0	2	1,797.85
									LPANHPLLTGQR	95.0%	38.5	20.6	1	2	0	2	1,316.74
									QVRPVTEK	95.0%	36.0	20.2	1	0	0	2	956.55
									TALVANTSNMPVAAR	95.0%	111.0	23.5	2	0	0	2	1,531.79
									VGHSELVGEIIR	95.0%	62.3	19.6	2	2	0	2	1,308.73
									VGSHITGGDIYGIVSENSLIK	95.0%	34.0	20.3	0	1	0	2	2,159.14
Elongation factor 1-delta	EF1D_HUMAN	EEF1D	31,104	100.00%	6	8	58	29.90%	ATAPQTQHVSPMR	95.0%	42.3	23.4	2	3	0	2	1,439.71
									FYEQMNGPVAGASR	95.0%	86.0	20.6	6	0	0	2	1,542.70
									GVVQELQQAISK	95.0%	81.9	20.9	20	0	0	2	1,299.73

									IASLEVENQSLR	95.0%	95.5	22.1	15	0	0	2	1,358.73
									LVPVGYGIR	95.0%	42.2	19.5	3	0	0	2	973.58
									SLAGSSGPGASSGTSGDHGELVVR	95.0%	116.0	21.3	1	8	0	2	2,185.05
14-3-3 protein eta	1433F_HUMAN	YWHAH	28,202	100.00%	9	11	35	55.30%	AVTELNEPLSNEDR	95.0%	92.4	22.5	4	0	0	2	1,586.77
									AVTELNEPLSNEDRNLLSVAYK	95.0%	62.1	20.7	2	4	0	2	2,475.27
									DSTLIMQLLR	95.0%	78.2	22.5	32	0	0	2	1,205.66
									ELETVCNDVLSLLDK	95.0%	75.7	22.8	4	0	0	2	1,747.88
									EQMQPTHPIR	95.0%	30.2	22.1	0	2	0	2	1,252.61
									LAEQAER	95.0%	56.5	21.6	15	0	0	2	816.42
									LAEQAERYDDMASAMK	95.0%	68.7	17.6	0	4	0	2	1,860.81
									MKGDYYR	95.0%	35.8	19.2	2	0	0	2	932.43
									NLLSVAYK	95.0%	51.2	19.1	26	0	0	2	907.53
									NLLSVAYKNVVGAR	95.0%	85.3	17.9	2	4	0	2	1,503.86
									NSVVEASEAAYK	95.0%	54.1	22.3	2	0	0	2	1,267.62
									QAFDDAIAELDTLNEDSYK	95.0%	117.0	21.6	1	0	0	2	2,157.98
									VISSIEQK	95.0%	73.7	23.0	12	0	0	2	903.52
									YDDMASAMK	95.0%	58.0	14.0	6	0	0	2	1,031.42
									YLAEVASGEKK	95.0%	53.6	22.0	4	2	0	2	1,194.64
40S ribosomal protein S9	RS9_HUMAN	RPS9	22,575	100.00%	10	11	31	32.00%	HIDFSLR	95.0%	33.8	22.6	2	0	0	2	887.47
									IEDFLER	95.0%	34.9	22.4	2	0	0	2	921.47
									IGVLDEGK	95.0%	31.9	23.1	1	0	0	2	830.46
									KQVVNIPSFIVR	95.0%	50.4	14.8	1	0	0	2	1,399.84
									LDYILGLK	95.0%	43.4	11.1	3	0	0	2	934.56
									LFEGNALLR	95.0%	61.3	22.9	8	0	0	2	1,032.58
									LIGEYGLR	95.0%	59.1	21.0	3	0	0	2	920.52
									MKLDYILGLK	95.0%	36.5	17.9	3	1	0	2	1,209.69
									QVVNIPSFIVR	95.0%	45.9	18.5	5	0	0	2	1,271.75
									RLFEGNALLR	95.0%	67.3	18.6	2	0	0	2	1,188.69
Early endosome antigen 1	EEA1_HUMAN	EEA1	162,450	100.00%	11	11	21	10.30%	AAQLATEIADIK	95.0%	62.3	22.3	2	0	0	2	1,243.69
									EAQNDLEQVLR	95.0%	53.4	21.8	2	0	0	2	1,314.67
									IQAGEGETAVLNQLQEK	95.0%	105.0	21.2	4	0	0	2	1,827.95
									IQNLEALLQK	95.0%	50.2	18.2	3	0	0	2	1,169.69
									ITTQLDQVTAK	95.0%	61.6	22.1	1	0	0	2	1,217.67
									LQQQLTQAAQELAAEK	95.0%	87.4	21.4	1	0	0	2	1,769.94
									LQQQLTQAAQELAAEKEK	95.0%	32.2	20.5	0	1	0	2	2,027.08
									LTMQITALNENLGTVK	95.0%	71.9	21.1	2	0	0	2	1,761.94
									NHTLQEQVTQLTEK	95.0%	64.9	20.7	1	0	0	2	1,668.86
									TELLQRPGIEDVAVLKK	95.0%	28.5	10.8	0	1	0	2	1,909.11
									VLSLETSVNELNSQLNESK	95.0%	106.0	21.4	3	0	0	2	2,104.08
Interleukin enhancer-binding factor 2	ILF2_HUMAN	ILF2	43,045	100.00%	13	19	119	38.20%	AQDPSEVLTMLTNETGFEISSSDATVK	95.0%	58.9	20.2	0	11	0	2	2,886.36
									GTMTTGHNVADLVVILK	95.0%	58.9	20.4	2	4	0	2	1,784.96
									ILITTVPPNLR	95.0%	43.6	11.8	10	0	0	2	1,236.77
									ILPTLEAVAALGNK	95.0%	116.0	14.5	30	3	0	2	1,409.84
									KLDPELHLDIK	95.0%	33.5	18.6	0	3	0	2	1,320.75
									LDPELHLDIK	95.0%	50.7	21.4	1	0	0	2	1,192.66
									NQDLAPNSAEQASILSLVTK	95.0%	115.0	20.6	12	10	0	2	2,099.10
									QPLALNVAYR	95.0%	59.1	21.8	5	0	0	2	1,144.65
									RNQDLAPNSAEQASILSLVTK	95.0%	72.2	19.4	0	2	0	2	2,255.20
									VKPAPDETSFSEALLK	95.0%	69.0	21.7	4	1	0	2	1,731.92
									VKPAPDETSFSEALLKR	95.0%	89.4	20.7	2	1	5	2	1,888.02
									VLQSALAAIR	95.0%	82.0	18.1	12	0	0	2	1,041.64

									WFEENASQSTVK	95.0%	36.0	21.9	1	0	0	2	1,425.67
Pyruvate kinase isozymes M1/M2	KPYM_HUMAN	PKM2	57,920	100.00%	28	42	786	57.30%	AGKPVICATQMLESMIK	95.0%	54.0	22.5	2	2	0	2	1,908.96
									APIIAVTR	95.0%	61.8	14.3	11	0	0	2	840.53
									ASDVHEVR	95.0%	67.8	21.1	24	0	0	2	912.45
									EAEAAIYHLQLFEELRR	95.0%	57.2	21.7	1	51	0	2	2,088.09
									FGVEQDVDMVFASFIR	95.0%	120.0	22.3	77	30	0	2	1,875.90
									GADFLVTEVENGGSLGSK	95.0%	139.0	22.1	41	0	0	2	1,779.88
									GADFLVTEVENGGSLGSKK	95.0%	56.8	22.5	1	1	0	2	1,907.97
									GDLGIEIPAEK	95.0%	59.7	21.5	47	0	0	2	1,141.61
									GDYPLEAVR	95.0%	51.6	23.3	23	0	0	2	1,019.52
									GSGTAEVELK	95.0%	39.3	23.3	3	0	0	2	990.51
									GSGTAEVELKK	95.0%	54.9	22.3	17	2	0	2	1,118.61
									GVNLPGAAVDLPAVSEK	95.0%	87.6	20.1	25	0	0	2	1,636.89
									GVNLPGAAVDLPAVSEKDIQDLK	95.0%	68.0	17.8	3	10	0	2	2,349.27
									IENHEGVR	95.0%	53.9	22.6	24	0	0	2	953.48
									ITLDNAYMEK	95.0%	66.5	21.9	28	0	0	2	1,213.58
									IYVDDGLISLQVK	95.0%	102.0	19.4	65	0	0	2	1,462.82
									KGVNLPGAAVDLPAVSEK	95.0%	94.8	17.8	5	2	0	2	1,764.99
									LAPITSDPTEATAVGAVEASFK	95.0%	144.0	21.2	66	12	0	2	2,175.12
									LDIDSPPITAR	95.0%	67.4	21.5	42	0	0	2	1,197.65
									LNFSHGTHEYHAETIK	95.0%	68.5	22.1	2	8	1	2	1,883.90
									MQHLIAR	95.0%	38.8	20.8	7	0	0	2	884.48
									NTGIICTIGPASR	95.0%	74.5	23.3	8	0	0	2	1,359.71
									QKGADFLVTEVENGGSLGSK	95.0%	99.3	22.2	2	2	0	2	2,036.03
									RFDEILEASDGIMVAR	95.0%	54.1	22.6	9	29	0	2	1,837.91
									SVETLKEMIK	95.0%	45.5	21.4	15	0	0	2	1,193.65
									TATESFASDPILYRPVAVALDTK	95.0%	63.7	19.1	7	65	0	2	2,465.29
									TATESFASDPILYRPVAVALDTKGPEIR	95.0%	61.0	18.1	0	5	1	2	3,017.60
									VNFAMNVGK	95.0%	55.3	22.0	10	0	0	2	995.50
40S ribosomal protein S10	RS10_HUMAN	RPS10	18,880	100.00%	3	4	12	18.80%	DYLHLPPEIVPATLR	95.0%	33.2	18.5	2	2	0	2	1,733.96
									HPELADK	95.0%	39.0	22.9	2	0	0	2	809.42
									IAIYELLFK	95.0%	54.1	15.3	6	0	0	2	1,109.66
Haloacid dehalogenase-like hydrolase domain-containing protein	HDD1A_HUMAN	HDHD1A	25,232	99.50%	2	3	6	14.90%	ALEAAQIIIDVLQLPMSK	95.0%	61.6	17.4	2	0	0	2	1,969.10
									LKEVFPTAALMPGAEK	95.0%	60.5	21.3	2	2	0	2	1,717.92
Sulfatase-modifying factor 2	SUMF2_HUMAN	SUMF2	33,825	99.90%	2	3	4	10.60%	EATVKPFAIDIFPVTNK	95.0%	33.9	18.2	1	1	0	2	1,890.04
									MGNTPDSASDNLGFR	95.0%	54.8	18.5	2	0	0	2	1,597.69
Tyrosine-protein kinase receptor UFO	UFO_HUMAN	AXL	98,317	100.00%	3	3	15	4.59%	APLQGTLLGYR	95.0%	74.4	21.2	6	0	0	2	1,188.67
									LAYQGQDTPEVLMDIGLR	95.0%	70.1	22.9	6	0	0	2	2,035.02
									TATITVLPQQPR	95.0%	37.0	16.8	3	0	0	2	1,324.76
Exosome complex exonuclease RRP4	EXOS2_HUMAN	EXOSC2	32,771	100.00%	4	4	7	21.20%	HLVVPGDTITTDTGFMR	95.0%	30.7	22.2	0	1	0	2	1,875.93
									LDSVLLLSSMNLPGGELR	95.0%	98.6	20.6	2	0	0	2	1,930.03
									LGQGVLVQVSPSLVK	95.0%	76.5	14.0	2	0	0	2	1,523.92
									YIGEVGDIVVGR	95.0%	57.3	22.7	2	0	0	2	1,276.69
Ubiquilin-2	UBQL2_HUMAN	UBQLN2	65,679	100.00%	4	5	10	15.50%	FQQQLEQLNAMGFLNR	95.0%	117.0	21.6	2	1	0	2	1,952.97
									GPAAAQGSAAAPAEPK	95.0%	64.7	23.0	3	0	0	2	1,393.71
									NPAMMQEMMR	95.0%	33.6	10.4	1	0	0	2	1,302.50
									QLIMANPQMQQLIQR	95.0%	48.0	21.4	2	0	0	2	1,843.95
									SQNRPQGQSTQPSNAAGTNTTSASTPR	95.0%	73.5	21.8	0	2	0	2	2,744.30
									SQTDQLVLIFAGK	95.0%	67.3	20.7	2	0	0	2	1,419.79
Ceroid-lipofuscinosis neuronal protein 5	CLN5_HUMAN	CLN5	41,480	100.00%	3	3	7	8.10%	ITYEEIPLPIR	95.0%	62.0	19.9	4	0	0	2	1,343.76
									LAEFGAEFK	94.6%	30.1	21.5	1	0	0	2	1,011.52

									YGDLLGHLK	95.0%	36.5	23.1	2	0	0	2	1,015.56
GTP-binding nuclear protein Ran	RAN_HUMAN	RAN	24,405	100.00%	6	7	20	33.80%	FNVWDTAGQEK	95.0%	58.0	21.6	1	0	0	2	1,294.61
									HLTGEFEKK	95.0%	31.2	23.5	0	4	0	2	1,088.57
									KYVATLGVEVHPLVFHTNR	95.0%	38.4	17.9	0	0	1	2	2,180.20
									LVLVGDGGTGK	95.0%	43.5	21.5	4	0	0	2	1,015.58
									NLQYYDISAK	95.0%	46.7	22.5	5	0	0	2	1,214.61
									SNYNFEKPFLWLAR	95.0%	38.9	21.5	2	3	0	2	1,784.91
Dipeptidyl peptidase 2	DPP2_HUMAN	DPP7	54,325	100.00%	6	8	19	14.00%	ASHPEDPASVVEAR	95.0%	42.7	22.3	2	1	0	2	1,464.71
									DLFLQGAYDTVR	95.0%	86.7	22.1	3	0	0	2	1,397.71
									DLTQLFMFAR	95.0%	41.7	21.9	1	0	0	2	1,257.63
									DVTADFEGQSPK	95.0%	73.1	21.4	5	0	0	2	1,293.60
									GALLVFAEHR	95.0%	52.9	19.8	3	2	0	2	1,112.62
									SLPFGAQSTQR	95.0%	51.0	23.2	2	0	0	2	1,191.61
DnaJ homolog subfamily B member 11	DJB11_HUMAN	DNAJB11	40,497	100.00%	8	10	20	25.70%	DGMEYPFIGEGEPHVDGEPGDLR	95.0%	38.0	17.2	0	2	0	2	2,532.10
									FQDLGAAYEVLSDSEK	95.0%	147.0	21.8	3	1	0	2	1,771.84
									FQDLGAAYEVLSDSEKR	95.0%	98.8	21.9	2	2	0	2	1,927.94
									GEGLPNFDNNNIK	95.0%	40.8	21.8	2	0	0	2	1,431.69
									KGEGLPNFDNNNIK	95.0%	67.5	23.0	2	0	0	2	1,559.78
									LALQLHPDRNPDDPQAQEK	95.0%	51.7	22.0	0	2	0	2	2,185.10
									TLEVEIEPGVR	95.0%	49.5	20.8	2	0	0	2	1,241.67
									TTQLGPGR	95.0%	49.9	21.7	2	0	0	2	829.45
Proteasome activator complex subunit 2	PSME2_HUMAN	PSME2	27,384	100.00%	7	10	36	35.60%	AFYAELYHIISSNLEK	95.0%	90.6	22.0	2	5	0	2	1,897.97
									ALVHERDEAAYGELR	95.0%	70.8	22.4	1	4	0	2	1,728.87
									AMVLDLR	95.0%	36.2	24.7	2	0	0	2	817.46
									GEEKPSMY	95.0%	35.9	16.7	1	0	0	2	956.40
									IEDGNDFGVAIQEK	95.0%	99.0	22.7	4	0	0	2	1,534.74
									QNLFQEAEEFLYR	95.0%	85.8	21.7	10	1	0	2	1,686.81
									TKVEAFQTTISK	95.0%	74.2	19.5	6	0	0	2	1,352.74
Perilipin-3	PLIN3_HUMAN	PLIN3	47,028	100.00%	8	8	27	28.80%	DTVATQLSEAVDATR	95.0%	48.6	22.9	1	0	0	2	1,576.78
									GLDKLEENLPILQQPTEK	95.0%	26.6	17.7	0	2	0	2	2,065.12
									IATSLDGFDVASVQQQR	95.0%	104.0	22.4	6	0	0	2	1,834.93
									LGQMVLSGVDTVLGK	95.0%	74.8	20.6	3	0	0	2	1,532.84
									QLQGPEKEPPKPEQVESR	95.0%	30.3	21.6	0	0	2	2	2,076.07
									SVVTGGVQSVMGSR	95.0%	94.2	23.0	6	0	0	2	1,379.70
									TLTAAAVSGAQPILSK	95.0%	73.3	18.3	4	0	0	2	1,527.87
									VSGAQEMVSSAK	95.0%	66.9	22.7	3	0	0	2	1,209.58
Phospholipid transfer protein	PLTP_HUMAN	PLTP	54,723	100.00%	17	23	308	36.10%	AGALQLLLVGDK	95.0%	103.0	16.6	29	0	0	2	1,197.72
									AGALQLLLVGDKVPHDLDMLLR	95.0%	51.4	14.1	0	22	1	2	2,403.34
									ATYFGSIVLLSPAVIDSPLK	95.0%	77.3	13.8	31	9	0	2	2,091.17
									AVEPQLQEEER	95.0%	58.8	22.7	33	0	0	2	1,327.65
									DPVASTSNLDMDFR	95.0%	89.1	18.4	5	0	0	2	1,583.70
									FLEQELETITIPDLR	95.0%	69.9	21.5	23	8	0	2	1,816.97
									GAFFPLTER	95.0%	34.7	22.1	2	0	0	2	1,037.54
									IYSNHSALESLALIPLQAPLK	95.0%	30.9	15.6	0	1	0	2	2,278.28
									KVYDFLSTFITSGMR	95.0%	68.6	22.4	6	4	0	2	1,780.89
									MHAAFGGTFK	95.0%	36.3	21.3	6	0	0	2	1,082.51
									MHAAFGGTFKK	95.0%	30.8	22.8	0	3	0	2	1,210.60
									SSVDELVGIDYSLMK	95.0%	86.7	22.4	2	0	0	2	1,671.82
									SSVDELVGIDYSLMKDPVASTSNLDMDFR	95.0%	71.0	19.8	0	19	1	2	3,236.50
									TGLELSRDPAGR	95.0%	38.0	21.5	0	9	0	2	1,271.67
									TMLQIGVMPMLNER	95.0%	87.7	22.3	22	0	0	2	1,680.81

									VPHDLDMLLR	95.0%	36.3	22.1	0	6	0	2	1,224.64
									VYDFLSTFITSGMR	95.0%	82.9	22.1	54	12	0	2	1,652.80
Argininosuccinate lyase	ARLY_HUMAN	ASL	51,641	100.00%	3	4	8	9.70%	FVGAVDPIMEK	95.0%	37.6	23.3	1	0	0	2	1,221.62
									INVLPLGSGAIAGNPLGVDR	95.0%	94.3	16.7	2	0	0	2	1,933.09
									LNSNDEDIHTANER	95.0%	95.5	19.7	2	3	0	2	1,627.73
UPF0556 protein C19orf10	CS010_HUMAN	C19orf10	18,777	100.00%	4	5	8	27.20%	ESDVPLKTEEFEVTK	95.0%	67.6	22.7	2	1	0	2	1,750.88
									GAEIEYAMAYSK	95.0%	60.7	20.5	2	0	0	2	1,348.61
									SYLYFTQFK	95.0%	45.7	22.6	2	0	0	2	1,196.60
									TAVAHRPGAFK	95.0%	28.2	20.5	0	1	0	2	1,154.64
COP9 signalosome complex subunit 2	CSN2_HUMAN	COPS2	51,582	100.00%	2	2	7	4.97%	NYDESGSPR	95.0%	38.4	18.2	1	0	0	2	1,024.43
									SINSILDYISTSK	95.0%	58.3	21.8	6	0	0	2	1,440.76
Nuclear transport factor 2	NTF2_HUMAN	NUTF2	14,461	100.00%	2	2	3	18.90%	ADEDPIMGFHQMFLLK	95.0%	30.7	21.1	2	0	0	2	1,907.90
									LSSLPFQK	95.0%	36.9	20.6	1	0	0	2	919.53
Tropomyosin beta chain	TPM2_HUMAN	TPM2	32,834	100.00%	8	10	54	22.50%	AEFAER	95.0%	35.5	21.9	4	0	0	2	722.35
									IQLVEEELDR	95.0%	72.3	22.3	6	0	0	2	1,243.65
									IQLVEEELDRAQER	95.0%	46.6	21.9	9	5	0	2	1,727.89
									KLVILEGELER	95.0%	29.4	16.8	0	3	0	2	1,298.77
									LATALQKLEEAEK	95.0%	37.8	20.8	0	2	0	2	1,443.81
									LEEAEKAADESER	95.0%	76.7	21.7	11	11	0	2	1,476.68
									SVAKLEK	95.0%	32.4	21.7	1	0	0	2	774.47
									YEEVAR	95.0%	38.4	22.0	2	0	0	2	766.37
Phosphoglucomutase-2	PGM2_HUMAN	PGM2	68,268	100.00%	2	3	5	5.23%	DLTTGYDDSQPDKK	95.0%	69.8	20.3	2	0	0	2	1,582.72
									ELNELVSAIEEHFFQPQK	95.0%	60.9	21.6	2	1	0	2	2,158.08
Nucleolysin TIAR	TIAR_HUMAN	TIAL1	41,572	99.50%	2	2	3	6.40%	FEDVVNQSSPK	95.0%	31.4	22.7	1	0	0	2	1,249.61
									QTFSPFGQIMEIR	95.0%	38.1	23.2	2	0	0	2	1,569.77
40S ribosomal protein S17	RS17_HUMAN	RPS17	15,533	100.00%	3	4	6	38.50%	DNYVPEVSALDQEIIEVDPDTK	95.0%	30.8	21.5	1	0	0	2	2,489.19
									LLDFGSLSNLQVTQPTVGMNFK	95.0%	115.0	21.2	3	1	0	2	2,409.25
									LQEEERER	95.0%	37.3	21.9	1	0	0	2	1,088.53
Serotransferrin	TRFE_HUMAN	TF	77,032	99.50%	2	3	16	2.72%	CLVEKGDVAFVK	95.0%	64.1	21.9	2	4	0	2	1,364.72
									GDVAFVK	95.0%	50.4	22.7	10	0	0	1	735.40
Phosphopantothenate--cysteine ligase	PPCS_HUMAN	PPCS	33,988	99.90%	2	2	4	7.72%	IQSSGGPLQITMK	95.0%	54.9	22.7	2	0	0	2	1,375.73
									LETDPAIVINR	95.0%	51.1	19.9	2	0	0	2	1,240.69
EF-hand domain-containing protein D2	EFHD2_HUMAN	EFHD2	26,680	100.00%	2	2	3	9.17%	DGFIDLMELK	95.0%	38.6	23.5	1	0	0	2	1,180.59
									LSEIDVSSEGVK	95.0%	50.7	23.4	2	0	0	2	1,262.65
14-3-3 protein theta	1433T_HUMAN	YWHAQ	27,747	100.00%	17	19	138	69.40%	AKLAEQAER	95.0%	33.6	22.6	4	0	0	2	1,015.55
									AVTEQGAELSNEER	95.0%	111.0	22.3	37	1	0	2	1,532.72
									DSTLIMQLLR	95.0%	78.2	22.5	32	0	0	2	1,205.66
									EKVESELR	95.0%	49.4	23.4	8	0	0	2	989.53
									EMQPTHPIR	95.0%	54.3	21.9	17	0	0	2	1,124.55
									KEMQPTHPIR	95.0%	50.7	22.5	2	4	0	2	1,252.65
									KQTIDNSQGAYQEAFDISK	95.0%	103.0	22.1	4	0	0	2	2,143.03
									LAEQAER	95.0%	56.5	21.6	15	0	0	2	816.42
									LAEQAERYDDMATCMK	95.0%	38.6	17.6	0	3	0	2	1,947.83
									LQLIKDYR	94.9%	25.8	18.6	0	1	0	2	1,048.62
									MKGDYFR	95.0%	39.1	19.2	2	0	0	2	932.43
									NLLSVAYK	95.0%	51.2	19.1	26	0	0	2	907.53
									QTIDNSQGAYQEAFDISK	95.0%	131.0	21.1	9	0	0	2	2,014.94
									QTIDNSQGAYQEAFDISKK	95.0%	69.1	22.3	0	9	0	2	2,143.03
									SICTTVLELLDK	95.0%	78.9	21.8	13	0	0	2	1,391.75
									TAFDEAIAELDTLNEDSYK	95.0%	114.0	21.6	12	4	0	2	2,144.99
									TELIQK	95.0%	42.1	23.5	3	0	0	2	731.43

									VESELR	95.0%	34.0	25.4	2	0	0	2	732.39
									VISSIEQK	95.0%	73.7	23.0	12	0	0	2	903.52
									YDDMATCMK	95.0%	42.8	6.0	3	0	0	2	1,166.42
									YLAEVACGDDR	95.0%	67.4	18.5	3	0	0	2	1,268.56
									YLAEVACGDDRK	95.0%	58.8	21.5	4	0	0	2	1,396.65
									YLIANATNPESK	95.0%	80.2	23.2	8	0	0	2	1,320.68
Protein S100-A6	S10A6_HUMAN	S100A6	10,162	99.90%	2	2	13	16.70%	LMEDLDR	95.0%	40.4	20.9	4	0	0	2	907.42
									LQDAEIAR	95.0%	69.6	21.6	9	0	0	2	915.49
Protein mago nashi homolog 2	MGN2_HUMAN	MAGOHB	17,259	100.00%	2	2	4	14.20%	IGSLIDVNQSK	95.0%	70.8	23.3	2	0	0	2	1,173.65
									VFYYLVQDLK	95.0%	67.6	22.8	2	0	0	2	1,287.70
CTP synthase 1	PYRG1_HUMAN	CTPS	66,673	100.00%	6	6	10	12.90%	FSDSYASVIK	95.0%	54.7	22.1	2	0	0	2	1,116.56
									GIIASSVGTILK	95.0%	75.3	12.8	3	0	0	2	1,158.71
									KLYGDADYLEER	95.0%	34.8	22.3	0	1	0	2	1,471.71
									RLDLPIER	95.0%	38.5	17.6	1	0	0	2	1,011.60
									VPLLLEEQGVVDYFLR	95.0%	60.7	18.2	1	0	0	2	1,890.04
									YIDSADLEPITSQEEPVR	95.0%	105.0	22.0	2	0	0	2	2,062.00
Ubiquitin-conjugating enzyme E2 variant 1	UB2V1_HUMAN	UBE2V1	16,477	100.00%	3	3	5	19.70%	LLEELEEGQK	95.0%	61.3	22.8	2	0	0	2	1,187.62
									WTGMIIGPPR	95.0%	45.5	20.5	1	0	0	2	1,127.60
									YPEAPPFVR	95.0%	40.5	24.0	2	0	0	2	1,075.56
Platelet-activating factor acetylhydrolase IB subunit alpha	LIS1_HUMAN	PAFAH1B1	46,619	100.00%	5	5	15	16.60%	GHTDSVQDISFDHSGK	95.0%	52.1	20.7	0	6	0	2	1,729.78
									LNEAKEEFTSGGPLGQK	95.0%	38.5	22.3	0	1	0	2	1,804.91
									SNGYEEAYSVFKK	95.0%	52.1	22.3	1	0	0	2	1,521.72
									TAPYVVTGSVDQTVK	95.0%	75.9	22.1	5	0	0	2	1,564.82
									YALSGHR	95.0%	32.0	22.6	2	0	0	2	803.42
Trifunctional purine biosynthetic protein adenosine-3	PUR2_HUMAN	GART	107,750	100.00%	10	10	22	13.30%	AFAHITGGGLLENIPR	95.0%	30.0	19.7	0	1	0	2	1,665.91
									AIAFLQQPR	95.0%	45.5	21.7	6	0	0	2	1,043.60
									ENLISALEEAK	95.0%	40.6	23.9	1	0	0	2	1,216.64
									ENLISALEEAKK	95.0%	44.3	21.6	2	0	0	2	1,344.74
									FGDPECQVILPLLK	95.0%	51.7	21.4	2	0	0	2	1,628.87
									GVEITGFPEAQALGLEVFHAGTALK	95.0%	26.0	18.3	0	1	0	2	2,555.35
									MLNIHPSLLPSFK	95.0%	50.4	20.5	2	0	0	2	1,512.83
									TVDGMQQEGTPYTGILYAGIMLTK	95.0%	35.8	21.3	0	3	0	2	2,619.27
									VDLGGFAGLFDLK	95.0%	66.4	21.2	2	0	0	2	1,351.73
									VLAVTAIR	95.0%	49.0	19.6	2	0	0	2	842.55
BRCA2 and CDKN1A-interacting protein	BCCIP_HUMAN	BCCIP	35,962	100.00%	3	3	18	15.00%	APVNTAELTDLLIQQNHIGSVIK	95.0%	63.8	15.9	0	7	0	2	2,474.36
									FLNDTTKPVGLLLSER	95.0%	44.0	17.5	0	7	0	2	1,803.00
									LLQQLFLK	95.0%	46.1	10.4	4	0	0	2	1,002.64
Chitinase-3-like protein 2	CH3L2_HUMAN	CHI3L2	43,484	100.00%	4	4	7	9.49%	DKSEVMLYQTINSLK	95.0%	67.2	21.3	2	0	0	2	1,784.91
									LQDQQVPYAVK	95.0%	51.4	22.4	2	0	0	2	1,288.69
									QMIDNSYQVEK	95.0%	45.6	20.6	2	0	0	2	1,370.63
									SEVMLYQTINSLK	95.0%	50.3	22.3	1	0	0	2	1,541.79
Spermidine synthase	SPEE_HUMAN	SRM	33,807	100.00%	7	7	28	25.50%	AAFVLPEFAR	95.0%	40.6	21.6	6	0	0	2	1,120.62
									FLPGMAIGYSSSK	95.0%	67.2	22.4	4	0	0	2	1,373.68
									KVLIIGGGDGGVLR	95.0%	56.2	13.2	0	4	0	2	1,353.82
									NPSTNFQEPVQPLTQQQVAQMQLK	95.0%	40.2	21.4	0	4	0	2	2,770.38
									VLIIGGGDGGVLR	95.0%	63.2	17.6	6	0	0	2	1,225.73
									YQDILVFR	95.0%	54.9	21.6	3	0	0	2	1,053.57
									YYNSDVHR	95.0%	37.2	20.0	0	1	0	2	1,053.48
Sulfhydryl oxidase 1	QSOX1_HUMAN	QSOX1	82,561	100.00%	28	35	755	37.10%	AAPGQEPPEHMAELQR	95.0%	69.2	21.9	11	0	0	2	1,776.83
									AHFSPSNIILDFPAAGSAAR	95.0%	108.0	21.6	23	63	0	2	2,042.05
									AKEVLPAIR	95.0%	37.1	12.8	6	0	0	2	996.62

									ALAEDVK	95.0%	41.5	24.5	3	0	0	2	745.41
									DFNIPGFPTVR	95.0%	55.0	22.9	24	0	0	2	1,262.65
									DVQNVAAAPELAMGALELESR	95.0%	99.2	22.4	9	0	0	2	2,200.09
									EVALDLSQHK	95.0%	58.8	22.1	24	0	0	2	1,139.61
									EVLPAIR	95.0%	33.6	16.6	1	0	0	2	797.49
									FGVTDFPSCYLLFR	95.0%	43.3	22.4	1	0	0	2	1,721.84
									FPVLEGQR	95.0%	50.9	22.9	26	0	0	2	945.52
									IEVGRFPVLEGQR	95.0%	54.7	20.0	19	23	0	2	1,499.83
									IPYSFFK	95.0%	37.2	24.3	4	0	0	2	901.48
									IYMADLESALHYILR	95.0%	91.0	22.2	65	18	0	2	1,823.94
									KEGAVLAK	95.0%	39.3	21.0	4	0	0	2	815.50
									LAGAPSEDPQFPK	95.0%	81.3	22.0	45	0	0	2	1,356.68
									LDVPVWDVEATLNFLK	95.0%	70.6	21.1	3	4	0	2	1,859.00
									LEEIDGFFAR	95.0%	53.4	22.8	15	0	0	2	1,196.60
									NGSGAVFPVAGADVQTLR	95.0%	99.4	22.4	22	0	0	2	1,758.91
									NKIPYSFFK	95.0%	49.0	22.8	0	4	0	2	1,143.62
									NNEEYLALIFEK	95.0%	102.0	22.2	80	1	0	2	1,482.75
									RDVQNVAAAPELAMGALELESR	95.0%	82.1	21.8	0	11	0	2	2,356.19
									RVLNTEANVVR	95.0%	43.3	20.1	10	4	0	2	1,270.72
									SALYSPSDPLTLLQADTVR	95.0%	117.0	21.4	111	8	0	2	2,047.07
									SFYTAYLQR	95.0%	47.3	22.9	27	0	0	2	1,148.57
									TALDDRK	95.0%	37.5	24.3	5	0	0	2	818.44
									VLNTEANVVR	95.0%	61.0	23.0	34	0	0	2	1,114.62
									VLNTEANVVRK	95.0%	49.9	21.4	5	0	0	2	1,242.72
									VPVLMESR	95.0%	55.0	23.1	42	0	0	2	946.50
Acidic leucine-rich nuclear phosphoprotein 32 family member E	AN32E_HUMAN	ANP32E	30,675	100.00%	4	5	14	23.90%	CPNLTYLNLSGNK	95.0%	57.8	23.1	1	0	0	2	1,493.74
									EEIQDEEDDDDYVEEGEEEEEEEEEGGLRGEK	95.0%	85.4	7.8	0	1	0	2	3,658.43
									IKDLSTVEALQNLK	95.0%	104.0	17.9	3	6	0	2	1,571.90
									INLELR	95.0%	42.7	21.8	3	0	0	2	757.46
Nuclease-sensitive element-binding protein 1	YBOX1_HUMAN	YBX1	35,906	100.00%	7	8	65	42.00%	AADPPAENSSAPEAEQGGAE	95.0%	51.5	17.9	1	0	0	2	1,897.81
									EDGNEEDKENQGDETQGQQPPQR	95.0%	56.7	15.8	0	10	0	2	2,628.11
									GAEAANVTGPGGVPVQGSK	95.0%	117.0	22.6	26	0	0	2	1,695.87
									NDTKEDVFVHQTAIK	95.0%	40.7	22.7	0	3	0	2	1,744.89
									NEGSESAPEGQAQQR	95.0%	63.8	18.9	4	0	0	2	1,587.70
									NYQQNYQNSESGEKNEGSESAPEGQAQQR	95.0%	91.8	15.6	0	14	0	2	3,257.40
									RPQYSNPPVQGEVMEGADNQGAGEQGRPVR	95.0%	77.0	20.6	0	2	5	2	3,239.53
Eukaryotic peptide chain release factor subunit 1	ERF1_HUMAN	ETF1	49,015	100.00%	6	6	31	15.60%	FHTEALTALLSDDSK	95.0%	34.2	23.5	0	1	0	2	1,647.82
									FTVDLPK	95.0%	44.3	20.5	6	0	0	2	819.46
									GFGGIGGILR	95.0%	56.8	20.1	7	0	0	2	946.55
									LSVLGAITSVQQR	95.0%	77.1	17.8	6	0	0	2	1,371.80
									TELSQSDMFDQR	95.0%	72.5	18.4	6	0	0	2	1,472.63
									YFDEISQDTGK	95.0%	55.8	18.5	5	0	0	2	1,302.59
Nuclear cap-binding protein subunit 1	NCBP1_HUMAN	NCBP1	91,823	99.50%	2	2	4	2.53%	ATNDEIFSILK	95.0%	65.0	22.3	2	0	0	2	1,250.66
									NLFLVIFQR	95.0%	51.8	17.2	2	0	0	2	1,149.68
Splicing factor 3A subunit 3	SF3A3_HUMAN	SF3A3	58,833	100.00%	6	7	15	16.40%	ENPSEEAQNLVEFTDEEGYGR	95.0%	110.0	17.8	2	0	0	2	2,413.04
									SALLALGLK	95.0%	61.9	13.4	2	0	0	2	885.58
									SLESLDTSLFAK	95.0%	95.9	22.1	4	0	0	2	1,310.68
									STLRDQINSDHR	95.0%	27.6	21.7	0	1	0	2	1,441.71
									VKPLQDQNELFGK	95.0%	57.2	21.8	2	2	0	2	1,515.82
									YLEMLLEYLQDYTDR	95.0%	81.8	22.0	2	0	0	2	1,980.93
Proliferating cell nuclear antigen	PCNA_HUMAN	PCNA	28,751	100.00%	12	14	74	60.50%	AEDNADTLALVFEAPNQEK	95.0%	106.0	21.7	14	1	0	2	2,074.99

									ATPLSSTVTLSMSADVPLVVEYK	95.0%	69.5	19.8	9	2	0	2	2,424.26
									DLSHIGDAVVISCAK	95.0%	56.8	22.5	1	0	0	2	1,584.81
									FSASGELGNGNIK	95.0%	100.0	23.2	8	0	0	2	1,293.64
									IADMGHLK	95.0%	32.4	21.0	3	0	0	2	900.46
									LSQTSNVDKEEEAVTIEMNEPVQLTFALR	95.0%	88.0	20.8	0	20	0	2	3,307.64
									LVQGSILKK	95.0%	45.9	11.8	2	0	0	2	985.64
									MPSGEFAR	94.8%	30.3	20.6	1	0	0	2	910.41
									NLAMGVNLTSMSK	95.0%	63.1	21.9	6	0	0	2	1,397.68
									SEGFDTYR	95.0%	34.0	15.7	3	0	0	2	974.42
									YLNFFTK	95.0%	37.5	23.6	3	0	0	2	932.49
									YYLAPK	94.6%	30.1	22.2	1	0	0	2	754.41
Semaphorin-4C	SEM4C_HUMAN	SEMA4C	92,605	99.50%	2	2	4	3.24%	DLPAEQPGSFLYDAR	95.0%	73.2	22.8	2	0	0	2	1,678.81
									LQALVVMAAQPR	95.0%	55.3	20.0	2	0	0	2	1,312.74
Myosin-XVIIIa	MY18A_HUMAN	MYO18A	233,102	100.00%	5	5	8	2.97%	DGFSLASQLK	95.0%	33.0	22.8	1	0	0	2	1,065.56
									IISNLFLGR	95.0%	62.4	16.9	2	0	0	2	1,032.62
									NLTLFQAACR	95.0%	38.5	23.1	2	0	0	2	1,193.61
									TFVQELER	95.0%	39.0	22.6	1	0	0	2	1,021.53
									VVSLEAELQDISSQESKDEASLAK	95.0%	32.7	21.7	0	2	0	2	2,576.29
Actin-related protein 2/3 complex subunit 4	ARPC4_HUMAN	ARPC4	19,649	100.00%	3	3	8	17.90%	AENFFILR	95.0%	48.4	21.7	4	0	0	2	1,009.55
									ELLLQPVTISR	95.0%	55.4	17.1	2	0	0	2	1,268.76
									VLIEGSINSVR	95.0%	50.7	20.0	2	0	0	2	1,186.68
Actin-related protein 3	ARP3_HUMAN	ACTR3	47,354	100.00%	7	8	31	23.90%	AEPEDHYFLLTEPPLNTPENR	95.0%	30.0	21.8	0	1	0	2	2,482.19
									DITYFIQQLLR	95.0%	66.2	19.1	12	0	0	2	1,409.78
									DREVGIPPEQSLETAK	95.0%	81.0	21.9	4	0	0	2	1,768.91
									GVDDLDFFIGDEAIEKPTYATK	95.0%	78.3	22.4	2	4	0	2	2,444.19
									HNPVFGVMS	95.0%	31.3	19.6	1	0	0	2	1,003.47
									LSEELSGGR	95.0%	63.3	23.5	3	0	0	2	947.48
									NIVLSGGSTMFR	95.0%	88.5	22.2	4	0	0	2	1,297.66
Adenylyl cyclase-associated protein 1	CAP1_HUMAN	CAP1	51,838	100.00%	11	15	67	29.30%	AGAAPYVQAFDSLLAGPVAEYLK	95.0%	94.9	20.0	9	2	0	2	2,351.23
									EIGGDVQK	95.0%	53.2	22.0	4	0	0	2	845.44
									EMNDAAMFYTNR	95.0%	60.1	14.8	4	0	0	2	1,494.60
									INSITVDNCK	95.0%	41.1	23.2	2	0	0	2	1,163.57
									LEAVSHTSDMHR	95.0%	59.2	20.5	6	2	0	2	1,398.64
									LSDLLAPISEQIK	95.0%	67.9	18.0	18	0	0	2	1,426.82
									LSDLLAPISEQIKEVITFR	95.0%	38.8	15.3	0	1	0	2	2,172.23
									NSLDCEIVSAK	95.0%	34.2	22.5	2	0	0	2	1,235.59
									SGPKPFSAPKPQTSPSPK	95.0%	56.6	20.3	2	1	0	2	1,837.98
									VENQENVSNLVIEDTELK	95.0%	117.0	22.4	11	2	0	2	2,073.04
									VPTISINK	95.0%	41.0	20.4	1	0	0	2	871.53
26S protease regulatory subunit 4	PRS4_HUMAN	PSMC1	49,168	100.00%	2	2	2	9.09%	GVILYGPPGTGK	95.0%	38.2	22.0	2	0	0	2	1,158.65
									IETLDPALIRPGR	95.0%	38.1	16.9	0	1	0	2	1,450.84
									TMLELLNQLDGFDSR	95.0%	44.3	22.5	1	0	0	2	1,767.86
Dihydropyrimidinase-related protein 2	DPYL2_HUMAN	DPYSL2	62,276	100.00%	5	5	9	12.90%	GSPLVVISQGK	95.0%	34.3	18.5	2	0	0	2	1,084.64
									ILDLGITGPEGHVLSRPEEVEAEAVNR	95.0%	29.7	19.0	0	1	0	2	2,900.51
									MDENQFVAVTSTNAAK	95.0%	110.0	21.0	3	0	0	2	1,741.81
									QIGENLIVPGGVK	95.0%	31.5	18.5	1	0	0	2	1,323.76
									VFNLYPR	95.0%	33.5	20.8	2	0	0	2	908.50
SUMO-activating enzyme subunit 1	SAE1_HUMAN	SAE1	38,432	100.00%	4	4	16	19.10%	AQNLNPMVDVK	95.0%	52.5	23.7	4	0	0	2	1,244.63
									GLTMLDHEQVTPEDPGAQFLIR	95.0%	43.9	21.7	0	4	0	2	2,483.22
									NDVLDSLGISPDLLPEDFVR	95.0%	95.0	21.9	4	0	0	2	2,214.13
									VSQGVEDGPDTKR	95.0%	43.4	22.2	4	0	0	2	1,387.68

Insulin-like growth factor-binding protein 4	IBP4_HUMAN	IGFBP4	27,916	99.50%	2	2	8	4.65%	LPGGLEPK	95.0%	34.6	16.2	2	0	0	2	810.47
									TGVKLPGGLEPK	95.0%	59.4	15.7	0	6	0	2	1,195.71
60S ribosomal protein L19	RL19_HUMAN	RPL19	23,449	99.50%	2	2	5	9.18%	LLADQAEAR	95.0%	47.7	21.4	4	0	0	2	986.53
									TLSKEEETK	95.0%	32.9	23.3	1	0	0	2	1,064.55
Prothymosin alpha	PTMA_HUMAN	PTMA	12,185	99.50%	2	3	4	12.60%	AAEDDEDDDVDTK	95.0%	82.6	10.0	2	0	0	2	1,437.55
									AAEDDEDDDVDTKK	95.0%	72.5	16.5	1	1	0	2	1,565.65
Importin-7	IPO7_HUMAN	IPO7	119,502	100.00%	15	17	77	16.80%	AFAVGVQQVLLK	95.0%	92.7	14.3	14	0	0	2	1,272.77
									AIFQTIQNR	95.0%	56.3	22.6	2	0	0	2	1,090.60
									DGALHMIGSLAEILLK	95.0%	67.1	19.4	4	5	0	2	1,696.93
									ENIVEAIIHSPELIR	95.0%	75.3	20.0	9	11	0	2	1,732.96
									ETENDDLTNVIQK	95.0%	71.3	22.5	2	0	0	2	1,518.73
									EYNEFAEVFLK	95.0%	64.8	21.6	11	0	0	2	1,388.67
									FIQLLSDQSDQSVLIQK	95.0%	123.0	19.0	2	0	0	2	1,962.06
									FKSDQNLQTALELTR	95.0%	27.7	21.8	0	1	0	2	1,763.93
									FSAPVVPSSFNFGGPAPGMN	95.0%	62.8	21.6	2	0	0	2	1,995.93
									GIDQCIPLFVEAALER	95.0%	71.0	22.3	3	0	0	2	1,830.94
									GTMDPALR	95.0%	36.3	23.1	2	0	0	2	876.43
									QLQDIATLADQR	95.0%	50.6	22.3	2	0	0	2	1,371.72
									QLQDIATLADQRR	95.0%	40.2	21.9	0	2	0	2	1,527.82
									SDQNLQTALELTR	95.0%	80.9	22.9	3	0	0	2	1,488.77
									YGSPGNVSK	95.0%	44.5	22.5	2	0	0	2	908.45
Apoptosis inhibitor 5	API5_HUMAN	API5	57,545	100.00%	4	4	21	10.40%	DAYQVILDGVK	95.0%	50.8	22.6	4	0	0	2	1,220.65
									ELPQFATGENLPR	95.0%	72.7	22.4	8	0	0	2	1,471.75
									EVEELILTESK	95.0%	31.6	23.7	1	0	0	2	1,289.68
									GTLGGLFSQILQGEDIVR	95.0%	107.0	19.9	8	0	0	2	1,903.03
NADPH--cytochrome P450 reductase	NCPR_HUMAN	POR	76,673	100.00%	3	3	6	8.12%	ATTPVIMVGPGTGVAPFIGFIQER	95.0%	58.1	19.1	0	2	0	2	2,474.31
									FAVFGLGNK	95.0%	46.5	22.2	2	0	0	2	952.53
									TNVLYELAQYASEPSEQELLRK	95.0%	53.7	20.7	0	2	0	2	2,581.32
Lactoylglutathione lyase	LGUL_HUMAN	GLO1	20,761	100.00%	7	8	31	42.40%	CDFPIMK	95.0%	32.5	20.0	1	0	0	2	926.41
									DFLLQQTMLR	95.0%	69.6	22.4	9	0	0	2	1,280.67
									FSLYFLAYEDKNDIPK	95.0%	62.5	22.2	2	3	0	2	1,962.99
									GLAFIQDPDGYWIEILNPNK	95.0%	105.0	20.8	4	0	0	2	2,303.17
									KSLDFYTR	95.0%	38.0	22.7	2	0	0	2	1,029.54
									RFEELGVK	95.0%	46.0	21.6	3	0	0	2	977.54
									VLGMTLIQK	95.0%	69.7	19.8	7	0	0	2	1,018.60
Signal recognition particle 9 kDa protein	SRP09_HUMAN	SRP9	10,094	100.00%	3	3	5	34.90%	PQYQTWEEFSR	95.0%	71.7	20.1	1	0	0	1	1,470.67
									TDQAQDVK	95.0%	38.8	22.0	2	0	0	2	904.44
									VTDDLVCLVYK	95.0%	73.7	22.4	2	0	0	2	1,324.68
PEST proteolytic signal-containing nuclear protein	PCNP_HUMAN	PCNP	18,907	100.00%	3	4	7	23.60%	AGAAGGPEEEAEKPVK	95.0%	49.0	21.9	2	0	0	2	1,539.77
									FGFAIGSQTTK	95.0%	45.4	21.5	2	0	0	2	1,156.60
									SAEEEAADLPTKPTK	95.0%	44.6	22.5	1	2	0	2	1,586.79
Rab GDP dissociation inhibitor alpha	GDIA_HUMAN	GDI1	50,566	100.00%	9	9	17	45.20%	FDLGQDVIDFTGHALALYR	95.0%	73.5	21.7	1	3	0	2	2,151.09
									FLMANGQLVK	95.0%	42.1	22.7	2	0	0	2	1,136.61
									FLVFVANFDENDPK	95.0%	88.4	22.1	2	0	0	2	1,654.81
									FQLLEGPPESMGR	95.0%	51.5	22.8	3	0	0	2	1,476.72
									IKLYSESLAR	95.0%	38.7	18.7	0	4	0	2	1,179.67
									KFDLGQDVIDFTGHALALYR	95.0%	45.7	20.9	0	1	0	2	2,279.18
									KQNDVFGEAEQ	95.0%	49.2	20.9	1	0	0	2	1,264.58
									LYSESLAR	95.0%	54.3	20.3	6	0	0	2	938.49
									MAGTAFDFENMKR	95.0%	36.9	18.4	2	0	0	2	1,549.68
									MLLYTEVTR	95.0%	55.7	21.8	8	0	0	2	1,141.59

									NPYYGGESSSITPLEELYK	95.0%	126.0	22.0	1	0	0	2	2,147.02
									NPYYGGESSSITPLEELYKR	95.0%	31.3	21.8	0	2	0	2	2,303.12
									SPYLYPLYGLGELPQGFAR	95.0%	115.0	21.6	17	1	0	2	2,141.11
									TFEGVDPQTTSMR	95.0%	59.1	19.3	2	0	0	2	1,484.67
									VPSTETEALASNLMGMFEK	95.0%	81.2	20.7	2	0	0	2	2,086.97
									YIAIASTTVETTDPEKEVEPALELLEPIDQK	95.0%	76.9	18.7	0	2	0	2	3,442.77
Calcium-binding protein 39	CAB39_HUMAN	CAB39	39,853	100.00%	6	8	17	20.20%	DVAQIFNNILR	95.0%	58.0	22.0	1	0	0	2	1,302.72
									FQNDRTEDEQFNDEK	95.0%	90.8	16.6	2	2	0	2	1,914.81
									LLGELLLDR	95.0%	66.0	19.1	2	0	0	2	1,041.63
									LLHSENYVTK	95.0%	28.0	23.3	0	2	0	2	1,203.64
									LLSAEFLEQHYDR	95.0%	66.3	22.9	3	3	0	2	1,620.80
									NIQFEAFHVFK	95.0%	42.0	23.0	2	0	0	2	1,379.71
Arginyl-tRNA synthetase, cytoplasmic	SYRC_HUMAN	RARS	75,364	100.00%	15	17	46	28.20%	AAYPDLENPPLLVTPSQQAK	95.0%	63.9	20.5	2	0	0	2	2,152.13
									DFVSEQLTSLLVNGVQLPALGENK	95.0%	43.0	19.0	0	1	0	2	2,571.37
									DFVSEQLTSLLVNGVQLPALGENKK	95.0%	112.0	17.4	0	4	0	2	2,699.46
									GFDILGIKPVQR	95.0%	51.0	18.6	4	2	0	2	1,342.79
									GNTAAYLLYAFTR	95.0%	48.3	22.9	2	0	0	2	1,460.75
									IYDALDVSLIER	95.0%	89.5	21.7	2	0	0	2	1,406.75
									LANIDEEMLQK	95.0%	37.7	23.0	2	0	0	2	1,319.65
									LLQQEEEIK	95.0%	44.1	24.1	2	0	0	2	1,129.61
									LMDLLGEGLKR	95.0%	31.5	22.4	0	1	0	2	1,260.70
									LNDYIFSFDK	95.0%	66.1	22.7	2	0	0	2	1,261.61
									MNDIVKEFEDR	95.0%	40.5	21.1	4	0	0	2	1,411.65
									SDGGYTYDTSDLAAIK	95.0%	106.0	21.2	4	0	0	2	1,676.77
									STIIGESISR	95.0%	74.1	21.6	4	0	0	2	1,062.58
									VEIAGPGFINVHLR	95.0%	75.9	19.3	2	3	0	2	1,521.85
									VIVDFSSPNIAK	95.0%	70.6	21.1	5	0	0	2	1,289.71
Fibulin-1	FBLN1_HUMAN	FBLN1	77,194	100.00%	9	10	31	15.90%	AITPPHPASQANIIFDITEGNLR	95.0%	72.8	19.7	0	8	0	2	2,475.30
									EFTRPEEIIFLR	95.0%	42.7	20.7	2	1	0	2	1,549.84
									GYHLNEEGTR	95.0%	39.1	19.3	4	0	0	2	1,175.54
									IIEVEEEQEDPYLNDR	95.0%	89.3	21.1	1	0	0	2	1,990.92
									LEMNYVVGGVVSHR	95.0%	41.0	23.1	0	2	0	2	1,575.80
									MCVDVNECQR	95.0%	51.3	15.2	2	0	0	2	1,326.52
									SQETGDLDVGGLQETDK	95.0%	129.0	22.4	4	0	0	2	1,791.83
									SQETGDLDVGGLQETDKIIEVEEEQEDPYLNDR	95.0%	62.2	19.3	0	3	0	2	3,763.73
									TGYYFDGISR	95.0%	76.0	21.8	4	0	0	2	1,178.55
Histone acetyltransferase type B catalytic subunit	HAT1_HUMAN	HAT1	49,496	100.00%	3	3	10	13.40%	LLVTDMSDAEQYR	95.0%	89.2	21.5	4	0	0	2	1,556.73
									VSQMLILTPFQGQGHGAQLLETVHR	95.0%	27.5	18.5	0	0	2	2	2,776.46
									YYTEFPTVLDITAEDPSK	95.0%	106.0	21.2	4	0	0	2	2,089.00
Inorganic pyrophosphatase	IPYR_HUMAN	PPA1	32,643	100.00%	6	7	23	21.80%	AAPFSLEYR	95.0%	58.3	22.0	8	0	0	2	1,053.54
									DKDFAIDIIK	95.0%	44.7	21.7	6	0	0	2	1,177.65
									MEIATKDPLNPIK	95.0%	38.2	22.1	2	1	0	2	1,485.80
									VIAINVDDPDAANYNDINDVK	95.0%	139.0	22.2	2	0	0	2	2,288.10
									VIAINVDDPDAANYNDINDVKR	95.0%	28.1	22.0	0	3	0	2	2,444.21
									YVANLFPYK	95.0%	31.7	21.7	1	0	0	2	1,114.59
Cartilage-associated protein	CRTAP_HUMAN	CRTAP	46,546	100.00%	3	3	9	9.48%	SLPGAEDYIKDLETK	95.0%	67.6	22.3	1	0	0	2	1,678.85
									SYESLFIR	95.0%	33.4	20.9	1	0	0	2	1,014.53
									TSITDMELALPDFFK	95.0%	64.0	20.5	7	0	0	2	1,743.85
Translationally-controlled tumor protein	TCTP_HUMAN	TPT1	19,578	100.00%	5	8	22	55.80%	DLISHDEMFSDIYK	95.0%	63.6	20.4	6	4	0	2	1,728.78
									EDGVTPYMIFFK	95.0%	38.7	21.7	2	0	0	2	1,462.69
									GKLEEQRPER	95.0%	33.4	21.1	2	1	0	2	1,241.66

									TEGNIDDSLIGGNASAEGPEGEGTESTVITGVDIVMNHHLQETSFTK	95.0%	23.1	16.3	0	0	1	2	4,873.26
									VKPFMTGAAEQIK	95.0%	36.5	21.6	4	2	0	2	1,435.76
Actin, cytoplasmic 1	ACTB_HUMAN	ACTB	41,720	100.00%	24	35	2080	60.80%	AGFAGDDAPR	95.0%	81.3	20.8	169	0	0	2	976.45
									AVFPSIVGRPR	95.0%	70.1	19.1	70	0	0	2	1,198.71
									CDVDIRK	95.0%	39.3	23.7	3	0	0	2	905.45
									DLTDYLMK	95.0%	55.4	20.2	70	0	0	2	1,014.48
									DLYANTVLSGGTTMYPGIADR	95.0%	126.0	21.7	161	22	0	2	2,231.07
									DSYVGDEAQSK	95.0%	82.1	19.0	70	0	0	2	1,198.52
									DSYVGDEAQSKR	95.0%	74.2	21.6	100	21	0	2	1,354.62
									EITALAPSTMK	95.0%	73.5	23.8	106	0	0	2	1,177.61
									EKLCYVALDFEQEMATAASSSSLEK	95.0%	28.5	21.6	0	2	0	2	2,823.31
									GYSFTTTAER	95.0%	82.0	20.3	251	0	0	2	1,132.53
									HQGVMVGMGQK	95.0%	48.2	21.1	9	12	0	2	1,203.56
									HQGVMVGMGQKDSYVGDEAQSK	95.0%	41.4	20.3	0	2	3	2	2,383.07
									IIAPPER	95.0%	39.1	17.6	8	0	0	2	795.47
									IIAPPERK	95.0%	41.0	16.0	11	0	0	2	923.57
									IKIIAPPER	95.0%	36.8	12.0	4	0	0	2	1,036.65
									IWHHTFYNELR	95.0%	50.6	22.4	0	6	0	2	1,515.75
									KDLYANTVLSGGTTMYPGIADR	95.0%	104.0	21.4	7	31	0	2	2,359.16
									LCYVALDFEQEMATAASSSSLEK	95.0%	122.0	20.1	56	103	0	2	2,566.17
									MQKEITALAPSTMK	95.0%	52.1	22.6	2	0	0	2	1,580.80
									QEYDESGPSIVHR	95.0%	64.3	21.4	39	52	0	2	1,516.70
									QEYDESGPSIVHRK	95.0%	57.8	21.8	4	2	0	2	1,644.80
									SYELPDGQVITIGNER	95.0%	116.0	22.7	268	4	0	2	1,790.89
									TTGIVMDSGDGVTHTVPIYEGYALPHAILR	95.0%	76.0	20.0	0	131	112	2	3,183.62
									VAPEEHPVLLTEAPLNPK	95.0%	92.5	18.4	94	75	0	2	1,954.07
Serine/threonine-protein phosphatase 2A 65 kDa regulatory subunit A alpha isoform	2AAA_HUMAN	PPP2R1A	65,292	100.00%	11	14	57	27.20%	AISHEHSPSDLEAHFVPLVK	95.0%	46.3	21.0	0	2	3	2	2,213.14
									DKAVESLR	95.0%	34.2	23.6	1	0	0	2	917.51
									IGPILDNSTLQSEVKPILEK	95.0%	69.6	14.6	3	2	0	2	2,194.23
									LSTIALALGVER	95.0%	65.8	16.2	2	0	0	2	1,242.74
									MAGDPVANVR	95.0%	46.6	22.0	4	0	0	2	1,045.51
									QLSQSLLPAIVELAEDAK	95.0%	81.6	18.7	18	6	0	2	1,925.06
									SALASVIMGLSPILGK	95.0%	80.1	15.6	5	0	0	2	1,572.90
									SEIIPMFSNLASDEQDSVR	95.0%	88.8	21.4	2	0	0	2	2,154.00
									TDLVPAFQNLMK	95.0%	47.8	22.4	2	0	0	2	1,392.72
									VLAMSGDPNYLHR	95.0%	57.0	22.1	3	0	0	2	1,488.73
									YMVADKFTELQK	95.0%	56.2	23.2	4	0	0	2	1,488.74
Carboxypeptidase E	CBPE_HUMAN	CPE	53,134	100.00%	5	5	12	17.40%	ELLVIELSDNPGVHEPGEPEFK	95.0%	40.1	20.6	0	2	0	2	2,448.23
									IHIMPSLNPDGFEK	95.0%	58.8	22.5	2	0	0	2	1,613.80
									LTASAPGYLAITK	95.0%	85.2	19.5	4	0	0	2	1,305.74
									VAVPYSPAAGVDFELESFSER	95.0%	61.4	21.5	2	0	0	2	2,270.10
									YIGNMHGNEAVGR	95.0%	36.7	21.5	2	0	0	2	1,433.66
Collagen alpha-2(IV) chain	CO4A2_HUMAN	COL4A2	167,540	100.00%	4	4	15	2.92%	EGFPGPPGFIGPR	95.0%	46.9	21.2	1	0	0	2	1,327.68
									GLPGEVLGAQPGPR	95.0%	67.2	21.1	7	0	0	2	1,347.74
									IAVQPGTVGPQGR	95.0%	44.3	20.3	1	0	0	2	1,279.71
									SVSIGYLLVK	95.0%	38.2	14.8	6	0	0	2	1,078.65
Galectin-3	LEG3_HUMAN	LGALS3	26,135	100.00%	7	11	75	31.20%	GNDVAFHFNPR	95.0%	73.6	21.9	9	0	0	2	1,273.61
									IALDFQR	95.0%	49.4	24.4	5	0	0	2	862.48
									IQVLVEPDHFK	95.0%	54.6	20.6	8	6	0	2	1,324.73
									KLNEISK	95.0%	44.6	21.9	2	0	0	2	831.49
									MLITILGTVKPNANR	95.0%	79.0	14.6	9	23	0	2	1,640.95

									QSVFPFESGKPFK	95.0%	42.5	22.1	1	6	0	2	1,497.77
									VAVNDAHLLQYNHR	95.0%	102.0	22.6	2	4	0	2	1,649.85
40S ribosomal protein S4, X isoform	RS4X_HUMAN	RPS4X	29,581	100.00%	16	18	40	52.10%	DANGNSFATR	95.0%	67.9	20.2	2	0	0	2	1,052.48
									ERHPGSFDVVHVK	95.0%	25.4	22.4	0	0	1	2	1,506.78
									GIPHLVTHDAR	95.0%	52.7	22.9	2	1	0	2	1,215.66
									GNKPWISLPR	95.0%	32.1	19.7	2	0	0	2	1,167.66
									HPGSFDVVHVK	95.0%	45.0	22.9	0	2	0	2	1,221.64
									IGVITNR	95.0%	43.8	24.4	2	0	0	2	772.47
									LRECLPLIIFLR	95.0%	42.5	12.3	2	4	0	2	1,542.92
									LSNIFVIGK	95.0%	61.1	15.1	6	0	0	2	990.60
									LTIAEER	95.0%	35.5	23.6	1	0	0	2	831.46
									TDITYPAGFMDVISIDK	95.0%	72.0	22.6	6	0	0	2	1,901.92
									TGENFR	95.0%	38.0	21.8	1	0	0	2	723.34
									TIRYPDPLIK	95.0%	31.7	18.8	0	2	0	2	1,215.71
									VNDTIQIDLETGK	95.0%	61.5	22.6	2	0	0	2	1,445.75
									VRTDITYPAGFMDVISIDK	95.0%	42.4	21.7	0	1	0	2	2,157.09
									YALTGDEVK	95.0%	33.1	21.9	1	0	0	2	995.51
									YALTGDEVKK	95.0%	40.0	21.2	2	0	0	2	1,123.60
Importin subunit alpha-4	IMA4_HUMAN	KPNA4	57,869	100.00%	3	4	11	11.50%	DAQVVQVVLDGLSNILK	95.0%	93.7	16.7	8	1	0	2	1,811.03
									IEQLQNHENEDIYK	95.0%	45.5	21.8	1	0	0	2	1,772.85
									VQNTSLEAIVQNASSDNQGIQLSAVQAAR	95.0%	40.5	19.8	0	1	0	2	3,012.54
Serine protease HTRA1	HTRA1_HUMAN	HTRA1	51,269	100.00%	7	7	24	14.40%	ESTLNMVVR	95.0%	30.9	23.4	1	0	0	2	1,048.55
									IAPAVVHIELFR	95.0%	51.1	15.4	2	0	0	2	1,364.81
									IDHQGKLPVLLLGR	95.0%	31.9	10.8	0	1	0	2	1,558.94
									LHRPPVIVLQR	95.0%	45.5	9.0	0	3	0	2	1,327.83
									LPVLLLGR	95.0%	53.6	6.0	9	0	0	2	880.60
									VTAGISFAIPSDK	95.0%	44.6	21.8	2	0	0	2	1,305.71
									YNFIADVVEK	95.0%	54.4	22.0	6	0	0	2	1,197.62
Dermcidin	DCD_HUMAN	DCD	11,266	100.00%	4	5	28	35.50%	DAVEDLESVGK	95.0%	46.9	23.0	3	0	0	2	1,161.56
									ENAGEDPGLAR	95.0%	76.3	20.9	21	0	0	2	1,128.53
									GAVHDVKDVLDSVL	95.0%	33.3	21.5	1	0	0	2	1,466.79
									LGKDAVEDLESVGK	95.0%	44.9	22.5	2	1	0	2	1,459.76
Vacuolar protein sorting-associated protein 35	VPS35_HUMAN	VPS35	91,692	100.00%	13	14	41	23.00%	AELAELPLR	95.0%	45.9	17.7	2	0	0	2	1,011.58
									ENDAVTIQVLNQLIQK	95.0%	70.1	18.8	2	0	0	2	1,826.00
									ILVGTNLVR	95.0%	58.8	16.7	2	0	0	2	984.62
									IPVDTYNNILTVLK	95.0%	95.9	17.7	11	0	0	2	1,602.91
									IREDLPNLESSEETEQINK	95.0%	49.1	21.8	0	2	0	2	2,244.10
									LFDIFSQQVATVIQSR	95.0%	94.2	20.4	4	1	0	2	1,852.00
									LNLEHIATSSAVSK	95.0%	25.9	21.1	0	1	0	2	1,469.80
									LSQLEGVNVER	95.0%	68.1	21.9	4	0	0	2	1,243.66
									NIIIALIDR	95.0%	32.2	14.1	1	0	0	2	1,040.65
									PTTQQSPQDEQEKLLDEAIQAVK	95.0%	50.3	21.3	0	4	0	1	2,596.31
									SEDPDQQYLILNTAR	95.0%	92.9	22.5	3	0	0	2	1,762.86
									VADLYELVQYAGNIIPR	95.0%	41.6	20.8	0	2	0	2	1,934.04
									VLETTVEIFNK	95.0%	71.4	20.9	2	0	0	2	1,292.71
Eukaryotic translation initiation factor 5	IF5_HUMAN	EIF5	49,205	99.90%	2	2	34	5.57%	AMGPLVLTEVLFNEK	95.0%	91.1	21.3	29	0	0	2	1,676.89
									VNILFDFVK	95.0%	36.5	19.5	5	0	0	2	1,094.63
Protein S100-A10	S10AA_HUMAN	S100A10	11,186	99.50%	2	2	4	27.80%	EFPGFLENQKDPLAVDK	95.0%	53.7	22.4	2	0	0	2	1,946.99
									FAGDKGYLTK	95.0%	39.8	22.3	2	0	0	2	1,099.58
Ras-related protein Rab-7a	RAB7A_HUMAN	RAB7A	23,472	100.00%	4	4	9	21.30%	ATIGADFLTK	95.0%	34.4	20.2	1	0	0	2	1,036.57
									DEFLIQASPR	95.0%	32.8	23.1	1	0	0	2	1,175.61

									EAINVEQAFQTIAR	95.0%	92.2	22.3	4	0	0	2	1,589.83
									FQSLGVAFYR	95.0%	43.6	22.0	3	0	0	2	1,187.62
Polyadenylate-binding protein 4	PABP4_HUMAN	PABPC4	70,766	100.00%	2	2	3	11.60%	ALDTMNFDVIK	95.0%	72.7	21.7	15	0	0	2	1,282.64
									EFSPFGSITSAK	95.0%	60.2	21.6	2	0	0	2	1,270.63
									FSPAGPVLSIR	95.0%	33.6	21.1	1	0	0	2	1,143.65
									ITGMLLEIDNSELLHMLESPESLR	95.0%	76.6	20.6	0	4	0	2	2,772.38
									SLGYAYVNFQQPADAER	95.0%	104.0	21.2	11	0	0	2	1,928.91
Legumain	LGMN_HUMAN	LGMN	49,393	100.00%	10	14	85	25.40%	APLTGHSCYPEALLHFR	95.0%	34.3	21.8	0	0	3	2	1,968.98
									ASSPVPLPPVTHLDLTPSPDVPLTIMK	95.0%	52.6	17.4	0	7	0	2	2,838.53
									DYTGEDVTPQNFLAVLR	95.0%	79.8	22.0	27	0	0	2	1,937.96
									IVSLLAASEAEVEQLLSER	95.0%	107.0	18.8	8	6	0	2	2,057.11
									KASSPVPLPPVTHLDLTPSPDVPLTIMK	95.0%	70.7	15.4	0	4	3	2	2,966.63
									KIVSLLAASEAEVEQLLSER	95.0%	108.0	16.4	4	1	0	2	2,185.21
									KLMNTNDLEESR	95.0%	78.3	21.5	9	3	0	2	1,465.70
									LMNTNDLEESR	95.0%	52.3	20.4	2	0	0	2	1,337.60
									QLTEEIQR	95.0%	47.1	22.3	4	0	0	2	1,016.54
									VMQFQGMK	95.0%	47.0	19.3	4	0	0	2	1,000.46
CTP synthase 2	PYRG2_HUMAN	CTPS2	65,661	99.50%	2	2	3	4.61%	LHLPIGDSASNLLFK	95.0%	29.1	18.6	0	1	0	2	1,624.91
									VPVLLEEQSIVK	95.0%	79.7	15.6	2	0	0	2	1,353.80
ATP-dependent RNA helicase DDX1	DDX1_HUMAN	DDX1	82,415	100.00%	9	10	17	17.20%	DQLSVLENGVDIVVGTPGR	95.0%	74.4	20.4	2	0	0	2	1,968.04
									ELAEQTLNNIK	95.0%	62.8	22.8	2	0	0	2	1,272.68
									ELLIIGGVAAR	95.0%	56.8	16.7	2	0	0	2	1,111.68
									GEDSVPDTVHHVVVPVNPK	95.0%	29.2	21.8	0	1	0	2	2,025.04
									GIDIHGVPYVINVTLPDEK	95.0%	41.4	19.9	1	4	0	2	2,079.11
									ILKGEYAVR	95.0%	28.0	18.6	0	1	0	2	1,048.62
									MHNQIPQVTSDGKR	95.0%	49.5	22.6	0	2	0	2	1,626.80
									TGAFSIPVIQIVYETLK	95.0%	49.9	16.0	1	0	0	2	1,879.06
									TGASVLNK	95.0%	40.3	23.5	1	0	0	2	789.45
Proactivator polypeptide	SAP_HUMAN	PSAP	58,094	100.00%	13	15	242	29.00%	EIVDSYLPVILDIIK	95.0%	101.0	14.5	83	12	0	2	1,730.00
									EMPMQTLVPAK	95.0%	34.9	23.2	2	0	0	2	1,276.63
									GCSFLPDPYQK	95.0%	42.2	21.0	9	0	0	2	1,311.60
									GEMSRPGEVCSALNLCESLQK	95.0%	38.7	20.7	0	2	0	2	2,381.09
									HLAELNHQK	95.0%	40.3	23.6	8	26	0	2	1,089.58
									LGPGMADICK	95.0%	60.4	21.5	9	0	0	2	1,077.51
									LPALTVHVTQPK	95.0%	43.0	16.0	0	8	0	2	1,303.77
									LVGYLDR	94.5%	30.0	21.5	1	0	0	2	835.47
									NVIPALELVEPIK	95.0%	47.0	11.5	17	0	0	2	1,434.86
									NVIPALELVEPIKK	95.0%	79.8	9.0	38	0	0	2	1,562.95
									NYISQYSEIAIQMMMHMQPK	95.0%	32.6	16.8	0	2	0	2	2,507.11
									QEILAALEK	95.0%	59.6	19.3	11	0	0	2	1,014.58
									SDVYCEVCEFLVK	95.0%	90.1	19.8	14	0	0	2	1,647.74
Myosin-9	MYH9_HUMAN	MYH9	226,520	100.00%	46	53	248	30.30%	AGVLAHLEEER	95.0%	52.1	22.8	2	0	0	2	1,223.64
									ALEEAMEQK	95.0%	49.8	22.6	5	0	0	2	1,064.49
									ALELDSNLYR	95.0%	57.7	23.1	4	0	0	2	1,193.62
									ALEQQVEEMKTQLEELEDELQATEDAK	95.0%	105.0	20.0	0	1	0	2	3,163.48
									ANLQIDQINTDLNLER	95.0%	128.0	22.0	8	0	0	2	1,869.97
									DFSALESQLQDTQELLQEENR	95.0%	114.0	20.8	8	7	0	2	2,493.17
									ELEDATETADAMNR	95.0%	91.6	17.1	9	0	0	2	1,581.67
									ELESQISELQEDLESER	95.0%	95.3	21.4	7	0	0	2	2,033.95
									EMEAELEDERK	95.0%	51.7	20.0	1	0	0	2	1,394.61
									EQADFAIEALAK	95.0%	70.6	22.8	7	0	0	2	1,305.67

									HSQAVEELAEQLEQTKR	95.0%	55.3	22.2	0	3	0	2	1,996.01
									IAEFTTNLTEEEEK	95.0%	77.4	22.0	5	0	0	2	1,653.79
									IAEFTTNLTEEEEKSK	95.0%	92.1	22.3	2	2	0	2	1,868.91
									IAQLEEELEEEQGNTELINDR	95.0%	84.7	20.5	4	1	0	2	2,472.17
									IAQLEEQLDNETK	95.0%	101.0	22.3	7	0	0	2	1,530.77
									IAQLEEQLDNETKER	95.0%	30.9	22.1	0	1	0	2	1,815.91
									IIGLDQVAGMSETALPGAFK	95.0%	91.1	22.4	10	0	0	2	2,034.06
									IMGIPEEEQMGLLR	95.0%	65.8	23.0	8	0	0	2	1,647.81
									IRELESQISELQEDLESER	95.0%	43.2	21.6	0	1	0	2	2,303.14
									KEEELQAALAR	95.0%	55.9	22.8	2	0	0	2	1,257.68
									KFDQLLAEEK	95.0%	34.1	22.6	1	0	0	2	1,220.65
									KLEGDSTDLSDQIAELQAQIAELK	95.0%	39.7	20.6	0	1	0	2	2,615.34
									KLQAQMK	95.0%	39.1	22.8	1	0	0	2	846.49
									KQELEEICHDLEAR	95.0%	27.7	21.9	0	2	0	2	1,769.85
									LDPHLVLDQLR	95.0%	47.1	18.8	4	10	0	2	1,318.75
									LQEMEGTVK	95.0%	33.8	23.4	1	0	0	2	1,050.51
									LQQELDDLLVDLDHQR	95.0%	104.0	22.1	4	15	0	2	1,949.99
									LQVELDNVTGLLSQSDSK	95.0%	137.0	21.4	16	0	0	2	1,946.01
									MQQNIQELEEQLEEEESAR	95.0%	62.7	19.0	0	5	0	2	2,349.05
									NLPIYSEEIVEMYK	95.0%	63.5	20.6	7	0	0	2	1,743.85
									NMDPLNDNIATLLHQSSDK	95.0%	31.0	21.7	0	2	0	2	2,142.01
									NTDQASMPDNTAAQK	95.0%	70.0	17.6	1	0	0	2	1,607.70
									QAQQERDELADEIANSSGK	95.0%	68.4	20.7	0	5	0	2	2,088.98
									QLLQANPILEAFGNAK	95.0%	94.9	19.3	25	0	0	2	1,726.95
									QTLENERGELANEVK	95.0%	31.7	22.4	1	0	0	2	1,729.87
									RGDLPFVVPR	95.0%	32.3	19.7	0	1	0	2	1,155.66
									TDLLLEPYNK	95.0%	60.8	22.9	4	0	0	2	1,205.64
									TEMEDLMSSKDDVGK	95.0%	55.2	17.0	1	3	0	2	1,716.73
									TQLEELEDELQATEDAK	95.0%	114.0	21.0	3	0	0	2	1,961.92
									VIQYLAYVASSHK	95.0%	68.6	20.7	3	0	0	2	1,478.80
									VISGVLQLGNIVFK	95.0%	94.8	10.4	12	0	0	2	1,486.90
									VISGVLQLGNIVFKK	95.0%	45.2	9.0	4	0	0	2	1,614.99
									VNKDDIQK	95.0%	37.0	23.0	1	0	0	2	959.52
									VSHLLGINVTDFTR	95.0%	63.4	21.1	2	5	0	2	1,571.85
									VVFQEFR	95.0%	36.9	22.4	1	0	0	2	924.49
									YEILTPNSIPK	95.0%	54.2	22.1	2	0	0	2	1,274.70
Glypican-1	GPC1_HUMAN	GPC1	61,663	100.00%	6	6	36	18.60%	GFSLSDVPQAEISGEHLR	95.0%	30.0	21.8	0	2	0	2	1,941.97
									SFVQGLGVASDVVR	95.0%	63.1	21.3	8	0	0	2	1,433.78
									TLQATFPGAFGELYTQNAR	95.0%	102.0	23.2	10	0	0	2	2,085.04
									VLQAMLATQLR	95.0%	65.1	20.4	12	0	0	2	1,259.71
									VNPQGPGPEEK	95.0%	32.1	23.3	1	0	0	2	1,151.57
									YLPEVMGDGLANQINNPEVEVDITKPDMTIR	95.0%	104.0	19.8	0	3	0	2	3,503.70
Profilin-1	PROF1_HUMAN	PFN1	15,036	100.00%	8	11	175	57.10%	CYEMASHLR	95.0%	43.3	18.5	5	3	0	2	1,182.50
									DSLLQDGEFSMDLR	95.0%	110.0	20.3	81	0	0	2	1,641.74
									SSFYVNGLTLGGQK	95.0%	89.8	22.4	2	0	0	2	1,470.76
									STGGAPTFNVTVTK	95.0%	75.3	23.0	6	0	0	2	1,379.72
									TDKTLVLLMGK	95.0%	65.3	17.6	8	5	0	2	1,218.71
									TFVNITPAEVGVLVGK	95.0%	106.0	17.5	39	0	0	2	1,643.94
									TFVNITPAEVGVLVGKDR	95.0%	96.1	17.2	10	6	0	2	1,915.07
									TLVLLMGK	95.0%	61.5	17.6	10	0	0	2	874.54
Transcription initiation factor IIA	TF2AA_HUMAN	GTF2A1	41,494	99.50%	2	2	3	7.45%	DIFLDDGVDEQVLMELK	95.0%	45.7	21.9	2	0	0	2	1,994.96

subunit 1									SVIEDVINDVR	95.0%	44.1	23.0	1	0	0	2	1,258.66
ADP-ribosylation factor-like protein	ARL3_HUMAN	ARL3	20,438	99.50%	2	2	3	18.10%	ILLLGLDNAGK	95.0%	57.2	17.2	2	0	0	2	1,126.68
3									QDLLTAAPASEIAEGLNLHTIR	95.0%	29.5	18.3	0	1	0	2	2,333.25
Macrophage migration inhibitory	MIF_HUMAN	MIF	12,459	99.50%	2	2	20	17.40%	LLCGLLAER	95.0%	62.5	22.1	7	0	0	2	1,044.59
factor									PMFIVNTNVPR	95.0%	66.5	22.9	13	0	0	1	1,287.69
Pyridoxal kinase	PDXK_HUMAN	PDXK	35,084	100.00%	2	2	5	12.20%	GQVLNSDELQELYEGLR	95.0%	96.6	22.6	2	0	0	2	1,962.98
									VVPLADIITPNQFEAELLSGR	95.0%	53.0	17.7	3	0	0	2	2,282.24
Coproporphyrinogen-III oxidase,	HEM6_HUMAN	CPOX	50,134	100.00%	4	4	8	10.80%	ATSLGRPEEEEDELAHR	95.0%	52.8	21.8	0	2	0	2	1,938.92
mitochondrial									FGLFTPGSR	95.0%	40.0	23.1	2	0	0	2	981.52
									IESILMSLPLTAR	95.0%	83.3	19.3	2	0	0	2	1,459.82
									YVEFNLLYDR	95.0%	50.9	22.7	2	0	0	2	1,331.66
Beta-galactosidase	BGAL_HUMAN	GLB1	76,060	100.00%	6	6	33	11.40%	AGATLDLLVENMGR	95.0%	54.6	22.8	3	0	0	2	1,475.75
									AYVAVDGIPQGVLER	95.0%	49.8	22.0	5	0	0	2	1,586.85
									SLYPLTFIQVK	95.0%	60.0	17.5	7	0	0	2	1,308.76
									SSDPDYLAAVDK	95.0%	55.1	22.1	4	0	0	2	1,280.60
									TEAVASSLYDILAR	95.0%	98.3	21.2	10	0	0	2	1,508.80
									VNYGAYINDFK	95.0%	48.4	22.8	4	0	0	2	1,303.63
Nucleosome assembly protein 1-like	NP1L1_HUMAN	NAP1L1	45,357	100.00%	4	4	13	14.30%	FYEEVHDLER	95.0%	47.7	21.0	5	0	0	2	1,336.62
1									GIPEFWLTVFK	95.0%	41.1	20.6	2	0	0	2	1,336.73
									LDGLVETPTGYIESLPR	95.0%	65.4	21.2	5	0	0	2	1,859.98
									NVDLLSDMVQEHDEPILK	95.0%	52.7	22.0	1	0	0	2	2,095.04
A disintegrin and metalloproteinase	ATS7_HUMAN	ADAMTS7	184,072	100.00%	5	6	14	4.39%	DAPAFYELQYR	95.0%	62.3	21.4	2	0	0	2	1,372.65
with thrombospondin motifs 7									DAVVDGTPCYQVR	95.0%	45.5	21.6	1	0	0	2	1,479.69
									IQEVAEAANFLALR	95.0%	116.0	20.8	6	2	0	2	1,544.84
									SCSPPSHGAPSR	95.0%	50.8	19.2	2	0	0	2	1,239.55
									VPETQPLAPSLAEAGPPADPLVVR	95.0%	88.5	18.3	1	0	0	2	2,424.31
V-type proton ATPase subunit E 1	VATE1_HUMAN	ATP6V1E1	26,128	100.00%	3	4	7	16.80%	ARDDLITDLLNEAK	95.0%	95.0	22.0	3	2	0	2	1,586.84
									IQMSNLMNQAR	95.0%	42.9	22.0	1	0	0	2	1,337.63
									LDLIAQQMMPEVR	95.0%	33.2	22.8	1	0	0	2	1,575.79
26S proteasome non-ATPase	PSD11_HUMAN	PSMD11	47,448	100.00%	7	7	17	21.80%	AELRDDPIISTHLAK	95.0%	41.0	20.3	0	2	0	2	1,678.91
regulatory subunit 11									EQSILELGSLLAK	95.0%	73.2	19.9	6	0	0	2	1,400.80
									IMLNTPEDVQALVSGK	95.0%	87.1	22.1	2	0	0	2	1,730.90
									LYDNLLEQNLIR	95.0%	62.1	20.5	1	0	0	2	1,503.82
									TGQAAELGGLLK	95.0%	52.3	22.4	3	0	0	2	1,157.65
									VQIEHISSLIK	94.9%	25.9	17.2	0	1	0	2	1,266.74
									YQEALHLGSQLLR	95.0%	46.8	21.4	0	2	0	2	1,527.83
Cullin-1	CUL1_HUMAN	CUL1	89,663	100.00%	8	9	15	15.10%	DGEDLMDESVLK	95.0%	44.7	19.2	2	0	0	2	1,366.61
									ESFESQFLADTER	95.0%	94.7	19.2	2	0	0	2	1,558.70
									ESTEFLQQNPVTEYMK	95.0%	91.2	20.8	2	0	0	2	1,959.90
									FTAFYASR	95.0%	31.9	22.8	1	0	0	2	962.47
									GQTPGGAQFVGLELYK	95.0%	47.9	23.0	1	0	0	2	1,664.87
									HQQLLGEVLTQLSSR	95.0%	41.1	19.7	0	2	0	2	1,708.93
									LVHQNSASDDAEASMISK	95.0%	62.7	21.4	0	2	0	2	1,918.88
									NPEEAELEDTLNQVMVVFK	95.0%	92.8	21.3	2	1	0	2	2,221.07
Dedicator of cytokinesis protein 11	DOC11_HUMAN	DOCK11	237,660	100.00%	3	3	9	1.64%	FVFEAPYTLSGK	95.0%	32.4	22.6	2	0	0	2	1,358.70
									LTGLSEISLR	95.0%	51.8	19.8	3	0	0	2	1,088.63
									NEASALLYLLMR	95.0%	67.1	21.8	4	0	0	2	1,409.75
Thioredoxin-like protein 1	TXNL1_HUMAN	TXNL1	32,233	100.00%	11	12	22	44.60%	GYMDLMPFINK	95.0%	50.0	20.7	2	0	0	2	1,360.63
									IDQYQGADAVGLEEK	95.0%	77.9	21.5	2	0	0	2	1,635.79
									IFINLPR	95.0%	48.4	20.3	1	0	0	2	872.54
									IKQHLENDPGSNEDTDIPK	95.0%	72.4	22.1	0	2	0	2	2,150.04

									ISYFTFIGTPVQATNMNDFK	95.0%	80.7	22.3	2	0	0	2	2,310.11
									ISYFTFIGTPVQATNMNDFKR	95.0%	30.3	21.1	0	2	0	2	2,466.21
									QHLENDPGSNEDTDIPK	95.0%	35.5	19.5	0	2	0	2	1,908.86
									SEPTQALELTEDDIKEDGIVPLR	95.0%	51.1	20.9	0	2	0	2	2,568.31
									SMDFEEAER	95.0%	33.3	14.1	1	0	0	2	1,129.45
									VGVKPVGSDPDFQPELSGAGSR	95.0%	90.8	22.0	2	2	0	1	2,199.10
									VRIDQYQGADAVGLEEK	95.0%	48.7	22.3	0	2	0	2	1,890.96
Radixin	RADI_HUMAN	RDX	68,548	100.00%	6	8	19	31.90%	AFAAQEDLEK	95.0%	45.9	24.1	2	0	0	2	1,121.55
									APDFVFYAPR	95.0%	83.9	21.7	44	0	0	2	1,182.59
									EKEELMER	95.0%	33.3	21.6	1	0	0	2	1,079.50
									ENPLQFK	95.0%	32.2	24.5	1	0	0	2	875.46
									FFPEDVSEELIQEITQR	95.0%	76.7	22.3	5	3	0	2	2,080.02
									FVIKPIDK	95.0%	36.6	17.2	7	0	0	2	959.59
									IAQDLEMYGVNYFEIK	95.0%	83.4	23.1	2	0	0	2	1,948.94
									IGFPWSEIR	95.0%	62.2	23.4	19	0	0	2	1,104.58
									KAPDFVFYAPR	95.0%	54.9	22.3	12	1	0	2	1,310.69
									KENPLQFK	95.0%	31.6	22.1	1	0	0	2	1,003.56
									KPDTIEVQQMK	95.0%	48.7	23.4	4	0	0	2	1,332.68
									LFFLQVK	95.0%	41.5	18.9	14	0	0	2	894.55
									LKQIEEQTIK	94.8%	25.8	20.0	0	1	0	2	1,229.71
									NISFNDKK	94.8%	30.3	22.7	1	0	0	2	965.51
									NQEQLAAELAEFTAK	95.0%	139.0	22.9	2	2	0	2	1,662.83
									PKPINVR	95.0%	31.9	12.6	1	0	0	1	823.52
									QLFDQVVK	95.0%	56.5	21.4	9	0	0	2	976.55
									QLQALSSELAQAR	95.0%	101.0	22.0	2	0	0	2	1,414.77
									RKPDTIEVQQMK	95.0%	53.8	22.3	13	38	0	2	1,488.78
									VTTMDAELEFAIQPNTTGK	95.0%	114.0	22.4	28	6	0	2	2,082.01
S-formylglutathione hydrolase	ESTD_HUMAN	ESD	31,446	100.00%	3	3	9	15.20%	AFSGYLGTDQSK	95.0%	61.0	21.9	4	0	0	2	1,273.61
									FAVYLPPK	95.0%	39.0	21.4	4	0	0	2	934.54
									MYSYVTEELPQLINANFPVDPQR	95.0%	28.3	21.7	0	1	0	2	2,740.33
Alpha-2-macroglobulin	A2MG_HUMAN	A2M	163,273	100.00%	5	5	28	3.73%	ALLAYAFALAGNQDK	95.0%	66.0	20.9	9	0	0	2	1,565.83
									ATVLNYLPK	95.0%	44.0	18.4	6	0	0	2	1,018.59
									FEVQVTVPK	94.8%	30.3	22.3	1	0	0	2	1,046.59
									LPPNVVEESAR	95.0%	65.8	22.4	10	0	0	2	1,210.64
									SSGSLLNNAIK	95.0%	31.5	22.5	2	0	0	2	1,103.61
GMP reductase 2	GMPR2_HUMAN	GMPR2	37,857	100.00%	2	2	4	6.90%	FSLFTAVHK	95.0%	31.7	21.8	2	0	0	2	1,049.58
									TVEVPFKGDVEHTIR	95.0%	37.2	21.3	0	2	0	2	1,726.91
Sialic acid synthase	SIAS_HUMAN	NANS	40,290	100.00%	2	2	3	9.75%	GRPMVISSGMQSMDTMK	95.0%	60.8	18.1	0	2	0	2	1,919.83
									GSDHSASLEPGELAELVR	95.0%	46.8	22.3	0	1	0	2	1,866.92
Peptidase M20 domain-containing protein 2	P20D2_HUMAN	PM20D2	47,759	100.00%	2	2	3	5.50%	GGAHDYYNVLPNK	95.0%	36.7	21.8	2	0	0	2	1,447.70
									LQEEQFVNAVE	95.0%	36.5	22.4	1	0	0	2	1,305.63
Eukaryotic translation initiation factor 2 subunit 3	IF2G_HUMAN	EIF2S3	51,092	100.00%	4	5	17	10.80%	GVTIKPTVDDD	95.0%	49.1	23.0	2	0	0	2	1,159.59
									NEVLMVNIGSLSTGGR	95.0%	108.0	22.5	4	0	0	2	1,662.85
									QDLTTLDVTK	95.0%	48.1	23.4	2	0	0	2	1,133.61
									VGQEIEVRPGIVSK	95.0%	51.4	17.8	4	5	0	2	1,510.86
Phosphatidylinositol transfer protein beta isoform	PIPNB_HUMAN	PITPNB	31,522	99.90%	2	4	11	12.50%	MIAPEGSLVFHEK	95.0%	47.0	22.3	2	4	0	2	1,473.74
									SQVEPADYKADEDPALFQSVK	95.0%	73.4	22.3	1	4	0	2	2,337.13
Cytosolic acyl coenzyme A thioester hydrolase	BACH_HUMAN	ACOT7	41,777	100.00%	8	10	14	27.90%	IMRPDDANVAGNVHGGTILK	95.0%	30.5	21.7	0	1	1	2	2,094.08
									LMDEVAGIVAAR	95.0%	80.9	23.7	2	0	0	2	1,260.66
									MIEEAGAIISTR	95.0%	34.8	23.3	1	0	0	2	1,306.67
									SLPVPQLVPETEDEK	95.0%	61.8	21.8	2	0	0	2	1,680.87

									SMEIEVLVDADPVVDSSQK	95.0%	100.0	21.8	1	0	0	2	2,077.00
									SMEIEVLVDADPVVDSSQKR	95.0%	43.6	21.9	0	1	0	2	2,233.10
									TNIVTASVDAINFHDK	95.0%	66.0	22.7	2	2	0	2	1,744.89
									VLEVPPVVYSR	95.0%	39.2	20.3	1	0	0	2	1,257.72
Renin receptor	RENR_HUMAN	ATP6AP2	38,991	100.00%	7	8	23	29.40%	ANSVFEDLSVTLR	95.0%	65.6	22.9	3	0	0	2	1,450.75
									DHSPDLYSLELAGLDEIGKR	95.0%	42.4	21.4	0	4	2	2	2,228.12
									FADDMYSLYGGNAVVELVTVK	95.0%	71.0	22.0	1	0	0	2	2,307.12
									ILVDALQK	95.0%	33.6	15.9	1	0	0	2	899.56
									IPDVAALSMGFSVK	95.0%	73.4	22.5	4	0	0	2	1,450.76
									LFQENSVLSSLPLNSLSR	95.0%	114.0	19.9	6	0	0	2	2,004.08
									YGEDSEQFR	95.0%	66.7	17.2	2	0	0	2	1,130.48
D-dopachrome decarboxylase	DOPD_HUMAN	DDT	12,694	100.00%	7	10	24	56.80%	ELALGQDR	95.0%	38.9	22.5	1	0	0	2	901.47
									FFPLESWQIGK	95.0%	57.3	21.8	3	0	0	2	1,351.71
									LCAAAASILGKPADR	95.0%	61.7	21.1	2	2	0	2	1,513.82
									PFLELDTNLPANR	95.0%	76.7	22.1	3	0	0	1	1,499.79
									PFLELDTNLPANRVPAGLEK	95.0%	60.7	18.1	1	2	0	1	2,194.19
									RLCAAAASILGKPADR	95.0%	34.4	20.5	0	2	0	2	1,669.92
									SHSAHFFEFLTK	95.0%	40.1	22.8	2	6	0	2	1,450.71
Kallikrein-6	KLK6_HUMAN	KLK6	26,838	100.00%	7	13	396	35.20%	AVIHPDYDAASHDQDIMLLR	95.0%	114.0	22.3	12	28	65	2	2,280.11
									EKPGVYTNVCR	95.0%	32.8	23.3	1	0	0	2	1,322.65
									ESSQEQSSVVR	95.0%	75.9	21.9	36	0	0	2	1,235.59
									KPNLQVFLGK	95.0%	77.0	16.7	37	30	0	2	1,143.69
									LSELIQPLPLER	95.0%	72.2	15.3	162	3	0	2	1,407.82
									QRESSQEQSSVVR	95.0%	42.1	23.8	2	7	0	2	1,519.75
									TADGDFPDTIQCAYIHLVSR	95.0%	93.0	21.5	6	7	0	2	2,279.08
Platelet-activating factor acetylhydrolase IB subunit beta	PA1B2_HUMAN	PAFAH1B2	25,552	99.50%	2	3	13	12.20%	ELFSPLHALNFGIGGDTTR	95.0%	74.9	22.1	2	2	0	2	2,045.05
									IIVLGLLPR	95.0%	49.9	4.8	9	0	0	2	993.68
T-complex protein 1 subunit theta	TCPQ_HUMAN	CCT8	59,603	100.00%	32	35	72	58.40%	AIADTGANVVVTGGK	95.0%	108.0	21.8	3	0	0	2	1,372.74
									ANEVISK	95.0%	60.0	25.0	4	0	0	2	760.42
									APGFAQMLK	95.0%	40.6	23.3	2	0	0	2	978.51
									AVDDGVNTFK	95.0%	45.8	22.4	4	0	0	2	1,065.52
									DIDEVSSLLR	95.0%	61.7	23.9	4	0	0	2	1,146.60
									DMLEAGILDTYLGK	95.0%	117.0	23.4	2	0	0	2	1,554.77
									EDGAISTIVLR	95.0%	51.3	22.9	2	0	0	2	1,173.65
									ELEVQHPAAK	95.0%	37.8	22.4	1	0	0	2	1,121.60
									ETEGDVTSVK	95.0%	36.2	22.2	2	0	0	2	1,064.51
									ETEGDVTSVKDAK	95.0%	61.2	22.9	2	0	0	2	1,378.67
									FAEAFEAIPR	95.0%	76.6	21.5	4	0	0	2	1,150.59
									GSTDNLMDDIER	95.0%	63.0	18.1	2	0	0	2	1,381.59
									HEKEDGAISTIVLR	95.0%	43.0	21.1	0	1	0	2	1,567.84
									HFSGLEEAVYR	95.0%	42.2	23.3	2	1	0	2	1,307.64
									IAVYSCPFDGMITETK	95.0%	60.7	21.9	1	0	0	2	1,847.86
									ILGSGISSSSVLHGMVFK	95.0%	79.3	20.7	2	0	0	2	1,834.97
									ILGSGISSSSVLHGMVFKK	95.0%	20.1	18.6	0	0	1	2	1,963.07
									KFAEAFEAIPR	95.0%	51.1	21.7	1	0	0	2	1,278.68
									LATNAAVTVLR	95.0%	99.0	18.7	2	0	0	2	1,128.67
									LFVTNDAATILR	95.0%	85.7	20.1	5	0	0	2	1,333.75
									LVPGGGATEIELAK	95.0%	61.7	19.5	3	0	0	2	1,354.76
									LYAVHQEGNK	95.0%	35.6	22.6	2	0	0	2	1,158.59
									MVINHLEK	95.0%	33.3	20.9	1	0	0	2	999.53
									NLRDIDEVSSLLR	95.0%	57.0	21.7	1	2	0	2	1,529.83

									NVGLDIEAEVPAVK	95.0%	90.6	20.3	2	0	0	2	1,453.79
									QYGNEVFLAK	95.0%	32.9	22.6	1	0	0	2	1,168.60
									TAEELMNFSK	95.0%	63.5	20.5	2	0	0	2	1,185.55
									TAEELMNFSKGEENLMDAQVK	95.0%	63.1	20.9	0	2	0	2	2,416.10
									TVGATALPR	95.0%	37.5	19.6	2	0	0	2	885.52
									VADMALHYANK	95.0%	37.7	22.1	1	1	0	2	1,248.60
									VDQIIMAKPAGGPKPPSGK	95.0%	41.7	18.9	0	2	0	2	1,907.04
									YNIMLVR	95.0%	43.4	22.1	2	0	0	2	924.50
Lysosomal alpha-mannosidase	MA2B1_HUMAN	MAN2B1	113,727	100.00%	6	6	22	7.81%	DLFSTFTITR	95.0%	62.9	22.6	4	0	0	2	1,200.63
									ELVDYFLNVATAQGR	95.0%	88.3	22.4	5	0	0	2	1,695.87
									FQVIVYNPLGR	95.0%	53.6	19.6	4	0	0	2	1,305.73
									HLVLLDTAQAAAAGHR	95.0%	72.8	20.3	0	2	0	2	1,643.90
									IYITDGNMQLTVLTDR	95.0%	103.0	22.3	3	0	0	2	1,868.94
									LPVSEGVFVVK	95.0%	54.2	19.1	4	0	0	2	1,173.69
Citrate synthase, mitochondrial	CISY_HUMAN	CS	51,696	100.00%	3	3	6	8.15%	GLVYETSVLDPDEGIR	95.0%	67.9	22.9	2	0	0	2	1,762.89
									IVPNVLLEQGK	95.0%	51.9	15.3	2	0	0	2	1,209.72
									VVPGYGHAVLR	95.0%	49.8	20.5	2	0	0	2	1,167.66
Structural maintenance of chromosomes protein 1A	SMC1A_HUMAN	SMC1A	143,220	99.50%	2	2	4	1.87%	SNLMDAISFVLGEK	95.0%	73.2	22.1	3	0	0	2	1,539.77
									TALFEEISR	95.0%	37.3	21.9	1	0	0	2	1,065.56
Tryptophanyl-tRNA synthetase, cytoplasmic	SYWC_HUMAN	WARS	53,150	100.00%	26	39	399	64.80%	AGNASKDEIDSAVK	95.0%	85.3	22.0	14	10	0	2	1,404.70
									ALIEVLQPLIAEHQAR	95.0%	89.8	16.4	19	45	0	2	1,801.03
									DIIACGFDINK	95.0%	68.0	22.4	9	0	0	2	1,265.62
									DLTLDQAYSYAVENAK	95.0%	120.0	21.3	47	0	0	2	1,800.87
									DMNQVLDAYENK	95.0%	92.1	19.4	18	0	0	2	1,455.64
									DMNQVLDAYENKKPFYLYTGR	95.0%	46.0	21.4	0	7	3	2	2,581.24
									DTIEEHR	95.0%	37.2	20.2	6	0	0	2	899.42
									DYTSGAMLTGELK	95.0%	73.3	20.6	3	0	0	2	1,401.66
									DYTSGAMLTGELKK	95.0%	69.5	22.3	8	0	0	2	1,529.75
									EVTDEIVKEFMTPR	95.0%	44.3	22.0	3	3	0	2	1,709.84
									GIDYDKLIVR	95.0%	73.4	20.3	26	5	0	2	1,191.67
									GIFFSHR	95.0%	32.3	24.1	1	0	0	2	863.45
									GIFGFTDSDCIGK	95.0%	72.3	20.4	2	0	0	2	1,416.65
									GPSSEAMHVGHLIPFIFTK	95.0%	45.9	21.3	3	27	2	2	2,068.07
									HAFSGGR	95.0%	40.2	15.9	3	0	0	2	731.36
									HAFSGGRDTIEEHR	95.0%	23.9	21.5	0	0	2	2	1,611.76
									IDKELINR	95.0%	52.7	20.9	14	0	0	2	1,000.58
									IGYPKPALLHSTFFPALQGAQTK	95.0%	43.6	17.2	0	3	5	2	2,485.36
									ISFPAIQAAPSFSNSFPQIFR	95.0%	88.2	21.5	14	2	0	2	2,325.20
									KALIEVLQPLIAEHQAR	95.0%	63.9	12.3	0	6	3	2	1,929.13
									KLSFDFQ	95.0%	61.9	21.5	19	0	0	2	884.45
									KPFYLYTGR	95.0%	31.2	23.2	1	3	0	2	1,144.62
									MSASDPNSSIFLTDTAK	95.0%	118.0	21.6	37	0	0	2	1,800.83
									PNSEPASLLELFNSIATQGELVR	95.0%	149.0	20.7	8	7	0	1	2,485.29
									TFIFSDLDYMGMSSGFYK	95.0%	127.0	18.1	6	0	0	2	2,140.92
									WLQDVFNVPLVIQMTDDEK	95.0%	79.6	21.5	3	2	0	2	2,306.14
Protocadherin gamma-C3	PCDGK_HUMAN	PCDHGC3	101,061	100.00%	3	3	5	4.82%	FPLESAHDPDVGSNSLQTYELSR	95.0%	48.1	21.6	0	2	0	2	2,562.21
									LEISEAVAPGTR	95.0%	30.5	22.7	1	0	0	2	1,242.67
									VLEDAPSGTR	95.0%	33.5	20.8	2	0	0	2	1,044.53
Cytoplasmic dynein 1 heavy chain 1	DYHC1_HUMAN	DYNC1H1	532,388	100.00%	10	10	33	3.06%	ASVVTLPVYLNFTR	95.0%	40.3	18.1	1	0	0	2	1,579.88
									DAATIMQPYFTSNGLVTK	95.0%	44.3	22.8	1	0	0	2	1,972.97
									DLFQVAFNR	95.0%	48.0	22.5	9	0	0	2	1,109.57

									IAFIMDESNVLDSGFLER	95.0%	98.2	22.1	4	0	0	2	2,072.00
									IMFEVQDLK	95.0%	45.2	23.5	6	0	0	2	1,138.58
									IQGLTVEQAEAVVR	95.0%	82.6	20.1	3	0	0	2	1,512.84
									LSLSNAISTALPLTQLR	95.0%	64.8	15.8	2	0	0	2	1,798.04
									SLLQALNEVK	95.0%	57.7	21.5	4	0	0	2	1,114.65
									TMTLFSALR	95.0%	31.6	20.1	1	0	0	2	1,055.56
									VLQLYQITQINHGLMMVGPSGSGK	95.0%	46.8	21.2	0	2	0	2	2,603.33
Leukocyte elastase inhibitor	ILEU_HUMAN	SERPINB1	42,726	100.00%	15	17	75	44.10%	ADLSGMSGAR	95.0%	65.7	20.2	3	0	0	2	980.45
									EATTNAPFR	95.0%	39.1	22.9	1	0	0	2	1,006.50
									FAYGYIEDLK	95.0%	54.1	23.3	4	0	0	2	1,218.60
									FQSLNADINKR	95.0%	56.4	22.3	4	0	0	2	1,305.69
									GQTEGKIPELLASGMVDNMTK	95.0%	44.9	22.3	0	3	0	2	2,251.10
									HNSSGSILFLGR	95.0%	72.9	22.9	5	0	0	2	1,287.68
									IEEQLTLEK	95.0%	62.6	23.7	4	0	0	2	1,102.60
									IPELLASGMVDNMTK	95.0%	92.4	22.7	3	0	0	2	1,650.81
									KIEEQLTLEK	95.0%	62.4	21.7	4	0	0	2	1,230.69
									LGVQDLFNSSK	95.0%	82.7	23.1	6	0	0	2	1,207.63
									LVLVNAIYFK	95.0%	71.6	14.0	11	0	0	2	1,179.71
									TYGADLASVDFQHASEDAR	95.0%	102.0	19.9	4	4	0	2	2,052.93
									TYGADLASVDFQHASEDARK	95.0%	65.2	20.6	0	4	0	2	2,181.02
									TYNFLPEFLVSTQK	95.0%	60.3	22.8	9	2	0	2	1,686.87
									VLELPYQGEELSMVILLPDDIEDESTGLKK	95.0%	78.5	19.6	0	4	0	2	3,389.73
Calmodulin	CALM_HUMAN	CALM1	16,820	99.50%	2	4	8	22.10%	EAFSLFDKDGDGTITTK	95.0%	77.5	22.6	2	2	0	2	1,844.89
									VFDKDGNGYISAAELR	95.0%	90.6	22.5	2	2	0	2	1,754.87
Lysosomal protective protein	PPGB_HUMAN	CTSA	54,450	100.00%	7	9	93	15.80%	DTVVVQDLGNIFTR	95.0%	98.0	22.5	33	0	0	2	1,576.83
									EFSHIAFLTIK	95.0%	42.7	21.0	7	0	0	2	1,305.72
									GAGHMVPTDKPLAAFTMFSR	95.0%	43.2	21.3	0	11	2	2	2,166.05
									QYSGYLK	95.0%	30.7	20.9	1	0	0	2	858.44
									SMNSQYLK	95.0%	45.4	19.2	2	0	0	2	986.46
									YEKDTVVVQDLGNIFTR	95.0%	84.0	21.2	2	10	0	2	1,997.03
									YGDSGEQIAGFVK	95.0%	102.0	21.8	25	0	0	2	1,370.66
Hornerin	HORN_HUMAN	HRNR	282,355	100.00%	5	9	240	2.95%	GEQHGSSSGSSSSYGQHGSGSR	95.0%	41.9	14.8	0	3	6	2	2,122.88
									GPYESGSGHSSGLGHR	95.0%	98.4	20.6	17	100	23	2	1,584.72
									GSGSGQSPSSGQHGTGFGR	95.0%	60.5	18.8	2	0	0	2	1,747.78
									QSLGHGQHGSGSGQSPSPSR	95.0%	63.6	21.6	0	84	4	2	1,947.90
									QSPSYGR	95.0%	36.8	21.3	1	0	0	1	794.38
NAD-dependent malic enzyme, mitochondrial	MAOM_HUMAN	ME2	65,428	99.90%	2	2	3	3.94%	ALTSQLTDEELAQGR	95.0%	82.4	22.9	2	0	0	2	1,631.82
									LFTPDVIR	95.0%	33.5	21.6	1	0	0	2	960.55
Phosphoserine aminotransferase	SERC_HUMAN	PSAT1	40,405	100.00%	14	18	71	38.60%	ALELNMLSLK	95.0%	70.2	21.5	10	0	0	2	1,147.64
									ASLYNAVTIEDVQK	95.0%	88.4	22.0	9	0	0	2	1,550.81
									DDLLGFALR	95.0%	58.0	23.9	8	0	0	2	1,019.55
									ELLAVPDNYK	95.0%	34.8	23.4	2	0	0	2	1,161.62
									FGTINIVHPK	95.0%	55.4	18.9	2	2	0	2	1,125.64
									FGVIFAGAQK	95.0%	64.9	20.6	6	0	0	2	1,037.58
									GAVLVCDMSSNFLSKPVDVSK	95.0%	49.9	21.9	0	2	0	2	2,269.12
									GDDALEKR	95.0%	48.2	22.3	2	0	0	2	903.45
									GVGISVLEMSHR	95.0%	48.6	22.3	3	1	0	2	1,300.67
									IINNTENLVR	95.0%	66.2	23.8	6	0	0	2	1,185.66
									KFGTINIVHPK	95.0%	41.2	17.1	1	4	0	2	1,253.74
									LPHSVLLEIQK	95.0%	57.0	16.4	2	6	0	2	1,276.76
									LPHSVLLEIQKELLDYK	95.0%	31.4	13.6	0	0	2	2	2,038.16

									QVVNFGPGPAK	95.0%	41.0	21.1	3	0	0	2	1,113.61
Dihydropyrimidine dehydrogenase [NADP+]	DPYD_HUMAN	DPYD	111,386	100.00%	23	26	57	27.80%	AAFIGIGLPEPNK	95.0%	53.2	20.1	2	0	0	2	1,326.74
									ADVVISAFGSVLSDPK	95.0%	89.2	22.3	5	0	0	2	1,604.85
									AMSIPQIR	95.0%	36.7	24.2	2	0	0	2	931.50
									CEFLPFLSPR	95.0%	38.3	22.9	1	0	0	2	1,265.64
									DSADIESILALNPR	95.0%	60.0	22.4	2	0	0	2	1,513.79
									EGGANGVTATNTVSGLMGLK	95.0%	93.5	22.3	2	0	0	2	1,892.94
									FINPFGLASATPATSTSMIR	95.0%	79.9	21.7	4	1	0	2	2,098.06
									GTTSGPMYGPGQSSFLNIELISEK	95.0%	61.5	21.6	1	2	0	2	2,529.22
									HTTLGER	95.0%	33.6	22.0	1	0	0	2	813.42
									IVAMQFVR	95.0%	36.6	21.2	3	0	0	2	979.54
									KLPSFGPYLEQR	95.0%	43.1	21.5	0	2	0	2	1,434.77
									LENNFDDIK	95.0%	53.6	22.7	2	0	0	2	1,107.53
									LGYSDITIFEK	95.0%	78.8	21.7	2	0	0	2	1,285.67
									LKEQNVAFSPLK	95.0%	53.5	20.3	2	0	0	2	1,373.78
									LPSFGPYLEQR	95.0%	48.1	22.2	2	0	0	2	1,306.68
									LTPNVTDIVSIAR	95.0%	87.6	18.9	8	0	0	2	1,398.80
									MSEAYSAK	95.0%	31.4	16.4	1	0	0	2	902.39
									QAVQIPFFAK	95.0%	52.7	19.8	1	0	0	2	1,148.65
									QEYVGGLSTSEIPQFR	95.0%	69.9	22.5	2	0	0	2	1,810.90
									SFITSIANK	95.0%	31.4	19.8	1	0	0	2	980.54
									SLSVNEMTLSTLK	95.0%	90.6	22.4	2	0	0	2	1,438.75
									TFSLDKDIVTNVSPR	95.0%	114.0	21.2	2	2	0	2	1,691.90
									TTYGGVSGTAIRPIALR	95.0%	36.8	18.7	0	2	0	2	1,732.97
Scavenger mRNA-decapping enzyme DcpS	DCPS_HUMAN	DCPS	38,592	100.00%	2	2	4	11.30%	IVFENPDPSDGFVLIPDLK	95.0%	71.2	20.3	2	0	0	2	2,115.10
									VNEASGDGDGEDAVVILEK	95.0%	121.0	21.5	2	0	0	2	1,916.91
Acylamino-acid-releasing enzyme	ACPH_HUMAN	APEH	81,206	100.00%	11	12	40	22.10%	ALDVSASDDEIAR	95.0%	74.5	24.4	4	0	0	2	1,361.66
									GSTGFGQDSILSLPGNVGHQDVK	95.0%	86.5	21.6	2	4	0	2	2,313.15
									LLTIDQDLMVAQFSTPSLPPTLK	95.0%	43.2	17.6	1	0	0	2	2,544.36
									MGFAVLLVNYR	95.0%	72.0	22.1	5	0	0	2	1,298.69
									QVLLSEPEEAAALYR	95.0%	105.0	22.6	2	0	0	2	1,688.89
									QYLVFHDGDSVVFAGPAGNSVETR	95.0%	41.7	21.2	0	2	0	2	2,565.24
									SALYYVDLIGGK	95.0%	78.8	21.6	6	0	0	2	1,298.70
									SFNLSALEK	95.0%	40.3	21.4	2	0	0	2	1,008.54
									TPLLLMLGQEDR	95.0%	92.6	22.4	8	0	0	2	1,401.74
									VGFLPSAGK	95.0%	33.3	22.3	1	0	0	2	875.50
									VTSVVVDVVPR	95.0%	74.1	18.1	3	0	0	2	1,169.69
Hydroxyacyl-coenzyme A dehydrogenase, mitochondrial	HCDH_HUMAN	HADH	34,260	100.00%	5	6	13	21.70%	DTPGFIVNR	95.0%	36.1	24.1	2	0	0	2	1,018.53
									FAGLHFFNPVPVMK	94.5%	25.5	23.0	0	1	0	2	1,619.84
									GDASKEDIDTAMK	95.0%	49.6	20.9	2	0	0	2	1,396.63
									LGAGYPMGPFELLDYVGLDTTK	95.0%	66.5	20.5	3	1	0	2	2,373.17
									TFESLVDFSK	95.0%	63.1	22.5	4	0	0	2	1,172.58
Kinectin	KTN1_HUMAN	KTN1	156,258	100.00%	20	20	73	19.30%	AHVQEVAQHNLK	95.0%	38.5	22.2	0	2	0	2	1,373.73
									ALKEEIGNVQLEK	95.0%	38.0	21.1	0	6	0	2	1,470.82
									AQQSLELIQSK	95.0%	62.0	22.6	2	0	0	2	1,244.69
									DAVSNTTNQLESK	95.0%	77.4	22.2	9	0	0	2	1,406.68
									IHVSYQETQQMQMK	95.0%	27.9	20.4	0	1	0	2	1,782.82
									ILNDQNK	95.0%	30.6	22.5	1	0	0	2	844.45
									LLEEQLQHEISNK	95.0%	69.4	21.7	2	0	0	2	1,580.83
									LMQLMESEQK	95.0%	33.8	21.3	1	0	0	2	1,268.59
									LQALANEQAAAAHELEK	95.0%	33.4	21.7	0	3	0	2	1,806.94

									LQTLVSEQPNKDVVEQMEK	95.0%	51.9	21.9	0	5	0	2	2,231.12
									QMQSSFTSSEQELER	95.0%	71.7	18.8	1	0	0	2	1,802.79
									QQQVEAVELEAK	95.0%	36.8	22.2	1	0	0	2	1,371.71
									SVEELLEAELLK	95.0%	73.5	21.5	9	0	0	2	1,372.76
									TAEHEAAQQDLQSK	95.0%	44.6	22.3	1	0	0	2	1,555.74
									TQLLQDVQDENK	95.0%	69.1	22.3	5	0	0	2	1,430.71
									TVEELLETGLIQVATK	95.0%	33.5	19.1	0	1	0	2	1,743.97
									TVEELLETGLIQVATKEEELNAIR	95.0%	51.9	16.9	0	8	0	2	2,698.45
									VNKEESLQMQVQDILEQNEALK	95.0%	66.4	21.0	0	5	0	2	2,602.30
									VQELQNLLK	95.0%	51.3	20.5	9	0	0	2	1,084.64
									WLQDLQEENESLK	95.0%	51.8	22.9	1	0	0	2	1,631.79
Attractin	ATRN_HUMAN	ATRN	158,518	100.00%	10	11	39	7.98%	DLDMFINASK	95.0%	67.5	21.8	6	0	0	2	1,169.55
									EQYAVVGHSAHIVTLK	95.0%	38.9	20.7	0	2	0	2	1,751.94
									LTGSSGFVTDGPGNYK	95.0%	93.5	22.2	10	0	0	2	1,599.77
									LTLTPWVGLR	95.0%	53.4	19.1	2	0	0	2	1,155.69
									NHNALLASLTTQK	95.0%	66.2	20.5	1	5	0	2	1,410.77
									SEAACLAAGPGIR	95.0%	71.7	21.8	4	0	0	2	1,272.64
									SVNNVVVR	95.0%	36.9	22.9	2	0	0	2	886.51
									YDVDTQMWTILK	95.0%	68.4	21.9	3	0	0	2	1,528.74
									YGHSLALYK	95.0%	27.3	21.4	0	2	0	2	1,051.56
									YQGNPLR	95.0%	31.4	24.0	2	0	0	2	847.44
Complement component 1 Q subcomponent-binding protein,	C1QBP_HUMAN	C1QBP	31,345	99.50%	2	4	17	12.10%	AFVDFLSDEIKEER	95.0%	69.2	23.7	2	5	0	2	1,697.84
									VEEQEPELTSTPNFVVEVIK	95.0%	94.8	20.6	8	2	0	2	2,287.17
Adenylosuccinate lyase	PUR8_HUMAN	ADSL	54,873	100.00%	6	6	19	19.20%	ASLPTLGFTHFQPAQLTTVGK	95.0%	26.1	19.1	0	1	0	2	2,214.19
									FLEEEVYPLLKPYESVMK	95.0%	47.7	21.9	0	4	0	2	2,230.14
									GTTGTQASFLQLFEGDDHKVEQLDK	95.0%	72.9	21.5	0	2	0	2	2,764.34
									NALDLLLPK	95.0%	57.2	12.8	4	0	0	2	996.61
									SNLENIDFK	95.0%	47.8	23.3	4	0	0	2	1,079.54
									VLSQQAASVVK	95.0%	46.4	20.4	4	0	0	2	1,129.66
CAD protein	PYR1_HUMAN	CAD	242,965	100.00%	10	10	29	6.97%	EATAGNPGGQTVR	95.0%	39.3	21.7	2	0	0	2	1,257.62
									EELSALVAPAFAHTSQVLVDK	95.0%	28.3	18.9	0	1	0	2	2,225.18
									LALGIPLPELR	95.0%	35.5	7.8	3	0	0	2	1,191.75
									LFVEALGQIGPAPPLK	95.0%	44.0	14.8	1	0	0	2	1,649.96
									MAEIGEHVAPSEAANSLEQAQAAAER	95.0%	59.3	21.4	0	1	0	2	2,696.26
									MALLATVLGRF	95.0%	60.3	19.0	5	0	0	2	1,207.69
									TLGVDLVALATR	95.0%	74.2	18.7	8	0	0	2	1,228.73
									VIMGEEVEPVGLMTGSGVVGVK	95.0%	93.4	21.3	2	0	0	2	2,219.13
									VLGTSPEAIDSAENR	95.0%	78.7	22.7	1	0	0	2	1,558.77
									VPQFSFSR	95.0%	40.5	22.9	5	0	0	2	967.50
Serum albumin	ALBU_HUMAN	ALB	69,349	100.00%	6	7	131	8.37%	KVPQVSTPTLVEVSR	95.0%	84.6	16.1	40	51	0	2	1,639.94
									LVNEVTEFAK	95.0%	61.5	22.8	16	0	0	2	1,149.62
									LVTDLTK	95.0%	53.2	21.1	2	0	0	2	789.47
									VPQVSTPTLVEVSR	95.0%	63.7	20.6	2	0	0	2	1,511.84
									YICENQDSISSK	95.0%	37.7	19.6	1	0	0	2	1,443.64
									YLYEIAR	95.0%	39.4	21.0	19	0	0	2	927.49
Transaldolase	TALDO_HUMAN	TALDO1	37,524	100.00%	18	19	95	45.40%	AAQASDLEK	95.0%	64.8	23.5	4	0	0	2	932.47
									ALAGCDFLTISPK	95.0%	84.9	22.6	6	0	0	2	1,392.72
									FAADAVK	95.0%	38.8	23.0	1	0	0	2	721.39
									IHLDEK	95.0%	33.2	18.8	1	0	0	2	754.41
									ILDWHVANTDKK	95.0%	38.9	20.3	1	0	0	2	1,439.76
									KFAADAVK	95.0%	56.2	20.7	2	0	0	2	849.48

									LFVLFGAEILK	95.0%	46.6	12.6	4	0	0	2	1,249.76
									LGGSQEDQIK	95.0%	59.1	22.9	6	0	0	2	1,074.54
									LIELYK	95.0%	46.9	13.8	7	0	0	2	778.47
									LLGELLQDNAK	95.0%	82.9	21.3	20	0	0	2	1,213.68
									LSDGIRK	95.0%	41.7	22.2	3	0	0	2	788.46
									LSFDKDAMVAR	95.0%	46.2	22.0	4	2	0	2	1,268.63
									LSSTWEGIQAGK	95.0%	80.0	23.3	4	0	0	2	1,276.65
									MESALDQLK	95.0%	46.4	23.1	7	0	0	2	1,050.51
									RLIELYK	95.0%	33.1	14.9	1	0	0	2	934.57
									SYEPLEDPGVK	95.0%	48.2	22.7	6	0	0	2	1,233.60
									TIVMGASFR	95.0%	59.9	22.1	11	0	0	2	997.51
									VSTEVDAR	95.0%	62.6	23.6	5	0	0	2	876.44
Actin-like protein 6A	ACL6A_HUMAN	ACTL6A	47,443	100.00%	7	7	13	27.50%	ELFQEMNIELVPPYMIASK	95.0%	49.3	21.6	2	0	0	2	2,284.12
									LIANNTTVER	95.0%	57.5	23.8	2	0	0	2	1,130.62
									LKIPEGLFDPSNVK	95.0%	28.1	19.9	0	1	0	2	1,556.87
									QGGPTYYIDTNALR	95.0%	79.7	22.7	2	0	0	2	1,568.77
									SEASLHPVLMSEAPWNTR	95.0%	39.4	21.7	0	2	0	2	2,040.98
									SPLAGDFITMQCR	95.0%	81.7	21.3	2	0	0	2	1,511.70
									STGLILDSGATHTTAIPVHDGYVLQQGIVK	95.0%	64.2	16.9	0	2	0	2	3,091.64
Di-N-acetylchitobiase	DIAC_HUMAN	CTBS	43,743	100.00%	3	4	16	7.79%	ATYIQNYR	95.0%	34.6	22.0	2	0	0	2	1,028.52
									ETTDSFHR	95.0%	44.9	19.8	6	0	0	2	992.44
									GDVSLKDIIDPAFR	95.0%	60.3	22.3	2	6	0	2	1,545.83
UDP-glucose:glycoprotein glucosyltransferase 1	UGGG1_HUMAN	UGGT1	177,177	100.00%	10	10	18	9.20%	EISYENTQISR	95.0%	40.6	22.0	2	0	0	2	1,339.65
									FLFVDADQIVR	95.0%	49.3	21.1	2	0	0	2	1,322.71
									GQYQGLSQDPNSLSNLDQDLPNNMIHQVPIK	95.0%	39.3	20.5	0	1	0	2	3,479.69
									IEYQFFEDR	95.0%	38.5	20.6	1	0	0	2	1,246.57
									ILASPVELALVVMK	95.0%	89.2	14.3	2	0	0	2	1,498.89
									ILETTTFFQR	95.0%	57.6	22.0	2	0	0	2	1,255.67
									LGIEGLSLHNVLK	95.0%	49.7	14.3	2	0	0	2	1,392.82
									LNIQPSEADYAVDIR	95.0%	87.9	22.9	2	0	0	2	1,703.86
									VEEDVASDLVMK	95.0%	66.0	22.7	2	0	0	2	1,350.65
									YVLEPEISFTSDNSFAK	95.0%	81.7	21.7	2	0	0	2	1,946.94
Multiple epidermal growth factor-like domains protein 8	MEGF8_HUMAN	MEGF8	303,074	100.00%	7	7	26	3.97%	APQTVELPAVAGHTLTAR	95.0%	49.0	18.2	0	2	0	2	1,832.00
									FLDTGVVQSDR	95.0%	68.7	23.4	3	0	0	2	1,236.62
									GAMYLLGGLTAGGVTR	95.0%	82.3	21.6	2	0	0	2	1,552.82
									GPDTENMEEVGR	95.0%	86.8	15.4	11	0	0	2	1,349.56
									GPLLASLSGSTRPPPIEASSGK	95.0%	40.0	18.1	0	4	0	2	2,122.15
									SASVGPPMEESVAHAVAAVGSR	95.0%	37.2	22.3	0	3	0	2	2,125.03
									SFHAAAYVPAGR	95.0%	35.6	23.6	1	0	0	2	1,246.63
Transportin-3	TNPO3_HUMAN	TNPO3	104,186	100.00%	5	6	19	8.88%	IQTSFYELPTDSHASLR	95.0%	48.6	22.1	0	2	0	2	1,964.97
									NPQFLDPVLGYLMK	95.0%	41.5	22.0	2	0	0	2	1,650.86
									SLDSFLLSPEAAVGLLK	95.0%	89.9	17.5	7	0	0	2	1,759.98
									SVDPENNPTLVEVLEGVVR	95.0%	69.5	20.2	4	2	0	2	2,066.08
									VIQEIWPVLSETLNK	95.0%	32.1	18.1	2	0	0	2	1,768.99
Elongation factor 1-beta	EF1B_HUMAN	EEF1B2	24,746	100.00%	2	2	10	14.70%	SPAGLQVLNDYLADK	95.0%	101.0	22.2	9	0	0	2	1,603.83
									SSILLDVKPWDDETDMAK	95.0%	31.5	22.4	0	1	0	2	2,079.00
N-acetylgalactosamine-6-sulfatase	GALNS_HUMAN	GALNS	58,009	100.00%	10	11	50	21.10%	AIDGLNLLPTLLQGR	95.0%	80.1	13.0	10	4	0	2	1,593.93
									ARPNIPVYR	95.0%	40.1	21.5	3	0	0	2	1,085.62
									DPGERFPLSFASAEYQEALSR	95.0%	66.8	21.7	0	4	0	2	2,370.14
									FPLSFASAEYQEALSR	95.0%	112.0	22.0	4	0	0	2	1,815.89
									LMDRPIFYYR	95.0%	32.6	23.1	0	4	0	2	1,389.70

									LPLIFHLGR	95.0%	57.7	12.3	6	0	0	2	1,065.66
									NAYTPQEIVGGIPDSEQLLPELLK	95.0%	61.4	19.6	4	0	0	2	2,624.38
									NAYTPQEIVGGIPDSEQLLPELLKK	95.0%	56.2	17.6	0	6	0	2	2,752.48
									NGFYTTNAHAR	95.0%	32.3	21.7	1	0	0	2	1,251.59
									YYEEFPINLK	95.0%	50.9	22.4	4	0	0	2	1,315.66
Soluble calcium-activated nucleotidase 1	CANT1_HUMAN	CANT1	44,822	100.00%	11	11	39	29.40%	AVPWVILSDGDGTVEK	95.0%	91.2	22.1	2	0	0	2	1,685.88
									FIPNTDDQIIVALK	95.0%	91.0	20.5	4	0	0	2	1,586.88
									FLLPETK	95.0%	32.0	19.5	1	0	0	2	847.49
									GMELSDLIVFNGK	95.0%	83.8	22.7	5	0	0	2	1,438.73
									GRGMELSDLIVFNGK	95.0%	29.9	22.3	0	1	0	2	1,651.85
									GYLTLSDSGDK	95.0%	32.0	20.8	1	0	0	2	1,155.55
									IAVIADLDTESR	95.0%	88.2	23.2	6	0	0	2	1,302.69
									IGSVKYEGIEFI	95.0%	53.2	22.0	4	0	0	2	1,354.73
									LYSVDDR	95.0%	39.7	23.4	3	0	0	2	867.42
									TGVVYQIEGSK	95.0%	70.3	22.5	4	0	0	2	1,180.62
									VASYIMAFTLDGR	95.0%	98.3	23.4	8	0	0	2	1,459.73
Mitogen-activated protein kinase 1	MK01_HUMAN	MAPK1	41,374	100.00%	8	9	17	24.20%	ALDLLDK	95.0%	31.2	21.8	1	0	0	2	787.46
									APEIMLNSK	95.0%	49.1	23.9	3	0	0	2	1,018.52
									APTIEQMKDVYIVQDLMETDLYK	95.0%	37.8	21.8	0	2	0	2	2,775.35
									DVYIVQDLMETDLYK	95.0%	42.0	22.5	1	0	0	2	1,860.89
									GQVFDVGPR	95.0%	44.2	23.2	2	0	0	2	974.51
									LKELIFEETAR	95.0%	52.8	20.3	2	2	0	2	1,348.75
									NYLLSLPHK	95.0%	34.1	20.0	2	0	0	2	1,084.62
									VADPDHDHTGFLTEYVATR	95.0%	60.3	21.6	0	2	0	2	2,144.01
Protein TFG	TFG_HUMAN	TFG	43,430	99.50%	2	2	2	9.75%	LLDSLEPPGEPGPSTNIPENDTVDGREEK	95.0%	39.2	19.9	0	1	0	2	3,105.49
									LLSNDEVTIK	95.0%	37.2	23.0	1	0	0	2	1,131.63
Protein-L-isoaspartate(D-aspartate) O-methyltransferase	PIMT_HUMAN	PCMT1	24,633	100.00%	8	10	28	59.00%	ALDVGSGSGILTACFAR	95.0%	91.3	22.4	2	0	0	2	1,694.85
									ELVDDSINNVR	95.0%	53.6	22.8	3	0	0	2	1,273.64
									LILPVGPAGGNQMLEQYDK	95.0%	78.6	21.7	3	0	0	2	2,059.05
									MGYAEEAPYDAIHVGAAAPVVPQALIDQLKPGGR	95.0%	97.4	20.0	0	2	4	2	3,520.79
									MKPLMGVIYVPLTDKEK	95.0%	41.4	20.0	0	2	2	2	1,994.07
									SGGASHSELIHNLR	95.0%	63.9	23.5	0	4	0	2	1,477.75
									TDKVFEVMLATDR	95.0%	46.5	22.6	0	2	0	2	1,540.77
									VQLVVGDGR	95.0%	52.0	20.0	4	0	0	2	942.54
Tropomyosin alpha-4 chain	TPM4_HUMAN	TPM4	28,504	99.50%	2	2	4	34.30%	AEFAER	95.0%	35.5	21.9	4	0	0	2	722.35
									HIAEEADR	95.0%	40.4	22.0	5	0	0	2	940.45
									IQALQQQADEAEDR	95.0%	71.8	21.9	2	0	0	2	1,614.77
									IQALQQQADEAEDRAQGLQR	95.0%	38.1	22.0	0	2	0	2	2,268.13
									IQLVEEELDR	95.0%	72.3	22.3	6	0	0	2	1,243.65
									IQLVEEELDRAQER	95.0%	46.6	21.9	9	5	0	2	1,727.89
									KLVILEGELER	95.0%	29.4	16.8	0	3	0	2	1,298.77
									LATALQKLEEAEK	95.0%	37.8	20.8	0	2	0	2	1,443.81
									LEEAEKAADESER	95.0%	76.7	21.7	11	11	0	2	1,476.68
									YEEVAR	95.0%	38.4	22.0	2	0	0	2	766.37
Phosphoglycerate kinase 1	PGK1_HUMAN	PGK1	44,597	100.00%	29	43	527	71.20%	ACANPAAGSVILLENLR	95.0%	108.0	20.4	2	0	0	2	1,768.94
									AHSSMVGVNLPQK	95.0%	64.1	22.6	27	4	0	2	1,383.71
									ALESPERPFLAILGGAK	95.0%	78.0	17.2	37	32	0	2	1,769.00
									ALMDEVVK	95.0%	52.5	22.6	7	0	0	2	920.48
									DCVGPEVEK	95.0%	48.7	19.0	4	0	0	2	1,032.47
									DVLFLK	95.0%	39.7	15.7	3	0	0	2	734.45
									ELNYFAK	95.0%	54.0	21.5	5	0	0	2	884.45

									FHVEEEGKGK	95.0%	44.3	22.2	14	10	0	2	1,159.57
									IQLINNMLDK	95.0%	49.8	23.4	2	0	0	2	1,217.66
									IQLINNMLDKVNEMIIGGGMAFTFLK	95.0%	51.6	20.0	0	6	0	2	2,958.51
									ITLPVDFVTADKFDENAK	95.0%	117.0	21.1	58	12	0	2	2,023.04
									KYAEAVTR	95.0%	43.1	20.8	2	0	0	2	937.51
									LGDVYVNDAFGTAHR	95.0%	96.3	22.5	22	40	0	2	1,634.79
									LTLDKLDVK	95.0%	50.9	17.7	4	0	0	2	1,044.63
									NNQITNNQR	95.0%	50.6	21.5	7	0	0	2	1,101.54
									SLLGKDVLFLK	95.0%	59.1	9.0	7	3	0	2	1,232.76
									SVVLMSHLGRPDGVPMPDK	95.0%	32.9	21.9	1	1	0	2	2,067.04
									SVVLMSHLGRPDGVPMPDKYSLEPVAVELK	95.0%	43.2	18.8	0	3	4	2	3,295.71
									TGQATVASGIPAGWMGLDCGPESSKK	95.0%	33.3	21.3	0	2	0	2	2,621.23
									VADKIQLINNMLDK	95.0%	76.3	21.0	2	2	0	2	1,630.88
									VDFNVPMK	95.0%	51.3	22.8	9	0	0	2	965.48
									VDFNVPMKNNQITNNQR	95.0%	45.1	22.8	2	4	0	2	2,048.00
									VLNNMEIGTSLFDEEGAK	95.0%	98.8	21.5	61	2	0	2	1,982.94
									VLPGVDALSNI	95.0%	63.7	19.4	45	0	0	2	1,097.62
									VNEMIIGGGMAFTFLK	95.0%	85.8	22.5	4	1	0	2	1,759.88
									VSHVSTGGGASLELLEGK	95.0%	98.2	21.5	17	14	0	2	1,740.91
									WNTEDKVSHVSTGGGASLELLEGK	95.0%	67.8	21.2	0	27	1	2	2,514.25
									YAEAVTR	95.0%	49.6	22.9	12	0	0	2	809.42
									YSLEPVAVELK	95.0%	75.1	21.6	5	0	0	2	1,247.69
Nucleobindin-2	NUCB2_HUMAN	NUCB2	50,179	100.00%	15	20	121	37.90%	AATSDLEHYDK	95.0%	62.7	20.3	12	0	0	2	1,249.57
									AATSDLEHYDKTR	95.0%	94.2	21.7	3	2	0	2	1,506.72
									ADIEEIK	95.0%	41.3	22.0	3	0	0	2	817.43
									AKLDSLQDIGMDHQALLK	95.0%	54.0	21.5	0	8	0	2	2,012.05
									ELDLVSHHVR	95.0%	59.3	23.5	4	0	0	2	1,204.64
									EVWEETDGLDPNDFDPK	95.0%	72.3	17.0	2	0	0	2	2,005.87
									IEPPDTGLYYDEYLK	95.0%	68.7	21.0	13	0	0	2	1,815.87
									KADELQK	95.0%	38.0	24.1	1	0	0	2	831.46
									LDSLQDIGMDHQALLK	95.0%	46.3	21.7	1	6	0	2	1,812.92
									LHDVNSDGFLDEQELEALFTK	95.0%	92.6	21.9	4	15	0	2	2,420.16
									LVTLEEFLK	95.0%	50.0	19.0	20	0	0	2	1,091.64
									NEEDDMVEMEEER	95.0%	86.3	4.8	4	0	0	2	1,686.61
									QVIDVLETDKHFR	95.0%	28.9	21.7	0	3	0	2	1,599.85
									VQNIHPVESAK	95.0%	54.2	22.7	8	4	0	2	1,221.66
									VYDPKNEEDDMVEMEEER	95.0%	59.8	12.6	1	7	0	2	2,288.92
Procollagen-lysine,2-oxoglutarate 5-dioxygenase 1	PLOD1_HUMAN	PLOD1	83,535	100.00%	26	31	322	42.50%	AQFDLAFVVR	95.0%	73.9	21.4	74	0	0	2	1,165.64
									AQVEEFLAQHGSEYQSVK	95.0%	101.0	22.3	7	0	0	2	2,049.99
									FLGSGGFIGYAPNLSK	95.0%	111.0	21.9	25	0	0	2	1,627.85
									FLLEYIAPMTEK	95.0%	95.3	22.4	29	0	0	2	1,470.76
									GDAKPEDNLLVLTVATK	95.0%	36.1	18.5	0	3	0	2	1,783.98
									GELQSSDLFHHSK	95.0%	59.2	21.7	5	0	0	2	1,484.71
									HTLGHLLSLDSYR	95.0%	45.2	21.6	0	8	0	2	1,511.80
									IFLDPEKR	95.0%	46.9	21.8	6	0	0	2	1,017.57
									IFQNLDGALDEVVLK	95.0%	116.0	20.4	30	4	0	2	1,673.91
									IQALGLGEDWNVEK	95.0%	81.8	21.4	2	0	0	2	1,571.81
									IQGGYENVPTIDIHMNQIGFER	95.0%	61.1	21.8	2	12	0	2	2,547.23
									LDPDMAFCANIR	95.0%	64.9	20.8	4	0	0	2	1,438.65
									LETKYPVVSDGK	95.0%	44.2	21.4	1	0	0	2	1,335.72
									LLIQQNK	95.0%	31.5	18.9	1	0	0	2	856.53

									LQLNYLGNYIPR	95.0%	63.7	20.5	21	0	0	2	1,463.80
									LTHYHEGLPTTR	95.0%	50.1	22.2	4	4	0	2	1,424.73
									LVGPEVR	95.0%	42.3	17.2	9	0	0	2	769.46
									LYPGYYTR	95.0%	35.4	23.0	2	0	0	2	1,032.52
									NLAYDTLPVLIHGNGPTK	95.0%	94.5	19.8	9	2	0	2	1,923.03
									QQDVFMFLTNR	95.0%	63.5	22.0	15	0	0	2	1,414.68
									SAQFFNYK	95.0%	56.8	22.6	9	0	0	2	1,004.48
									SEDYVDIVQGR	95.0%	94.9	22.3	18	0	0	2	1,280.61
									SQVVFSAEELIYPDR	95.0%	80.3	22.1	9	0	0	2	1,752.88
									SQVVFSAEELIYPDRR	95.0%	55.2	22.5	1	3	0	2	1,908.98
									VGVWNVPYISNIYLIK	95.0%	30.6	16.3	1	0	0	2	1,878.05
									YPVVSDGKR	95.0%	46.1	23.8	2	0	0	2	1,020.55
Threonyl-tRNA synthetase, cytoplasmic	SYTC_HUMAN	TARS	83,420	100.00%	11	12	65	16.50%	ADMETLQR	95.0%	43.3	21.0	2	0	0	2	979.45
									AILGSVER	95.0%	31.8	23.7	2	0	0	2	844.49
									ETLLAMFK	95.0%	32.3	20.7	1	0	0	2	968.51
									GAYIYNALIEFIR	95.0%	80.2	20.9	14	0	0	2	1,542.83
									GFQEVVTPNIFNSR	95.0%	84.1	23.2	8	0	0	2	1,607.82
									IYGISFPDPK	95.0%	56.4	22.2	13	0	0	2	1,136.60
									LADFGVLHR	95.0%	61.9	20.3	3	0	0	2	1,027.57
									MGGEEKPIGAGEEK	95.0%	54.1	20.6	3	2	0	2	1,447.67
									NELSGALTGLTR	95.0%	87.3	22.5	11	0	0	2	1,231.66
									QLENSLNEFGEK	95.0%	69.6	22.5	2	0	0	2	1,407.68
									TVYSVFGFSFK	95.0%	56.4	22.7	4	0	0	2	1,281.65
Nuclear mitotic apparatus protein 1	NUMA1_HUMAN	NUMA1	238,242	100.00%	14	14	33	9.27%	AALMESQGQQQEER	95.0%	74.8	19.7	4	0	0	2	1,620.73
									AQELGHSQSALASAQR	95.0%	42.5	22.9	0	3	0	2	1,653.83
									AVQAQGGESQQEAQR	95.0%	75.3	22.1	4	0	0	2	1,586.75
									DSALETLQGQLEEK	95.0%	80.9	22.6	4	0	0	2	1,560.78
									DSAQTSVTQAQR	95.0%	67.4	22.5	3	0	0	2	1,291.62
									EAEQMGNELER	95.0%	36.9	17.7	1	0	0	2	1,321.57
									GEVLGDVLQLETLK	95.0%	37.8	19.8	1	0	0	2	1,513.85
									LEILQQQLQVANEAR	95.0%	90.2	18.8	4	0	0	2	1,752.96
									LLQAETASNSAR	95.0%	65.5	23.4	2	0	0	2	1,260.65
									LQAQLNELQAQLSQK	95.0%	92.7	19.7	2	0	0	2	1,711.93
									NSLISSLEEEVSILNR	95.0%	62.4	21.3	1	0	0	2	1,802.95
									QFLEVELDQAR	95.0%	61.5	22.5	2	0	0	2	1,347.69
									SLEAQVAHADQQLR	95.0%	32.6	22.9	1	0	0	2	1,565.80
									VEFATLQEALAHALTEK	95.0%	32.8	20.5	0	1	0	2	1,870.99
Disintegrin and metalloproteinase domain-containing protein 15	ADA15_HUMAN	ADAM15	87,726	100.00%	5	5	13	7.00%	EPLEPQVLQDDLPISLK	95.0%	59.3	20.3	1	0	0	2	1,934.05
									EPLEPQVLQDDLPISLKK	95.0%	41.2	17.1	2	0	0	2	2,062.14
									GLVVLTPER	95.0%	34.1	16.2	2	0	0	2	983.59
									SYTLEQGPGDLQGPPIISR	95.0%	82.5	21.0	4	0	0	2	2,028.04
									VLQTSLPEPLR	95.0%	50.6	17.7	4	0	0	2	1,252.73
Lysosomal acid lipase/cholesteryl ester hydrolase	LICH_HUMAN	LIPA	45,402	100.00%	8	9	102	28.80%	DLFGDKEFLPQSAFLK	95.0%	64.0	21.5	35	9	0	2	1,854.96
									EFLPQSAFLK	95.0%	44.6	21.6	2	0	0	2	1,179.64
									FQAFDWGSSAK	95.0%	79.0	20.0	10	0	0	2	1,243.57
									IINLMR	95.0%	41.3	23.2	9	0	0	2	775.45
									LPDHLIK	95.0%	43.3	17.6	10	0	0	2	835.50
									MFFALGPVASVAFCTSPMAK	95.0%	103.0	21.6	8	0	0	2	2,164.03
									TGQEQVYYVGHSQGTTIGFIAFSQIPELAK	95.0%	43.2	20.2	0	16	0	2	3,269.65
									VDVYTTHSPAGTSVQNMLHWSQAVK	95.0%	36.1	20.9	0	3	0	2	2,772.34
Eukaryotic translation initiation	EIF3I_HUMAN	EIF3I	36,484	100.00%	6	6	13	28.00%	DMTMFVTASK	95.0%	41.8	19.1	5	0	0	2	1,162.51

factor 3 subunit I									EGDLLFTVAK	95.0%	38.7	20.8	1	0	0	2	1,092.59
									GHFGPINSVAFHPDGK	95.0%	44.8	22.8	0	2	0	2	1,679.83
									QINDIQLSR	95.0%	46.8	22.0	2	0	0	2	1,086.59
									SYSSGGEDGYVR	95.0%	66.0	16.7	2	0	0	2	1,276.54
									TERPVNSAALSPNYDHVVLGGGQEAMDVTTTSTR	95.0%	30.0	20.2	0	0	1	2	3,589.72
Septin-7	SEPT7_HUMAN	SEPT7	50,662	100.00%	7	8	26	19.90%	DVTNNVHYENYR	95.0%	69.5	20.6	2	0	0	2	1,523.69
									FEDYLNAESR	95.0%	73.0	20.2	2	0	0	2	1,243.56
									LKDSEAELQR	95.0%	42.1	23.1	1	2	0	2	1,188.62
									MEMEMEQVFEMK	95.0%	67.3	10.8	2	0	0	2	1,625.62
									SPLAQMEEERR	95.0%	33.5	24.2	0	4	0	2	1,361.65
									STLINSLFLTDLYSPEYPGPSHR	95.0%	75.4	21.7	0	2	0	2	2,607.31
									VNIIPLIAK	95.0%	40.3	9.5	11	0	0	2	980.65
Tropomyosin alpha-3 chain	TPM3_HUMAN	TPM3	32,802	100.00%	5	5	16	33.50%	AADAEAEVASLNR	95.0%	66.5	21.8	3	0	0	2	1,316.64
									AADAEAEVASLNRR	95.0%	27.0	21.3	0	1	0	2	1,472.75
									AEFAER	95.0%	35.5	21.9	4	0	0	2	722.35
									HIAEEADR	95.0%	40.4	22.0	5	0	0	2	940.45
									IQLVEEELDR	95.0%	72.3	22.3	6	0	0	2	1,243.65
									IQLVEEELDRAQER	95.0%	46.6	21.9	9	5	0	2	1,727.89
									KLVIIEGDLER	95.0%	53.3	18.3	0	2	0	2	1,284.75
									LATALQKLEEAEK	95.0%	37.8	20.8	0	2	0	2	1,443.81
									LEEAEKAADESER	95.0%	76.7	21.7	11	11	0	2	1,476.68
									MELQEIQLK	95.0%	56.4	23.8	5	0	0	2	1,131.61
									SVAKLEK	95.0%	32.4	21.7	1	0	0	2	774.47
									YEEVAR	95.0%	38.4	22.0	2	0	0	2	766.37
Importin-9	IPO9_HUMAN	IPO9	115,946	100.00%	7	7	30	11.30%	EALVDTLTGILSPVQEVR	95.0%	61.5	18.7	10	0	0	2	1,940.07
									EVTDTQMPLVAPVILPEMYK	95.0%	71.5	21.6	2	0	0	2	2,306.17
									HLQEAEQTK	95.0%	41.9	22.1	2	0	0	2	1,083.54
									IAAQDLLLAVATDFQNESAAALAAAATR	95.0%	49.5	18.6	0	2	0	2	2,786.47
									LIINELSNVMEANAAR	95.0%	88.6	22.3	2	0	0	2	1,773.92
									TSEFTAAFVGR	95.0%	86.2	22.4	2	0	0	2	1,185.59
									YSNDPVVASLAQDIFK	95.0%	102.0	21.8	10	0	0	2	1,766.90
Platelet-derived growth factor C	PDGFC_HUMAN	PDGFC	39,011	99.90%	2	2	22	8.41%	VVDLNLLTEEVR	95.0%	114.0	20.3	21	0	0	2	1,399.78
									YDFVEVEEPSDGTILGR	95.0%	49.1	21.3	1	0	0	2	1,925.91
Alpha-mannosidase 2	MA2A1_HUMAN	MAN2A1	131,128	100.00%	31	36	165	29.80%	AGLSHMLIQR	95.0%	52.0	21.1	1	7	0	2	1,141.62
									DKTQYIFNNMVLK	95.0%	60.6	23.1	3	1	0	2	1,629.83
									DSVINLSESVEDGPK	95.0%	96.2	22.9	4	0	0	2	1,588.77
									EGSFPQGQLSMLQEK	95.0%	53.4	22.5	3	0	0	2	1,694.81
									FDQTGLMK	95.0%	47.6	20.0	8	0	0	2	955.46
									FLSSSLYTALTEAR	95.0%	107.0	23.1	24	0	0	2	1,558.81
									FYTDLNGYQIQPR	95.0%	58.8	22.4	6	0	0	2	1,614.79
									GLEQGIQDNK	95.0%	48.5	21.8	5	0	0	2	1,101.55
									GLEQGIQDNKITANLFR	95.0%	34.0	20.0	0	2	0	2	1,917.02
									GQSMFPVLSGDFFTYADR	95.0%	86.4	20.0	2	0	0	2	2,053.93
									IIGNSAFLLILK	95.0%	65.7	9.5	3	0	0	2	1,301.82
									ILESASSNSHLADYVLYK	95.0%	93.5	21.4	2	3	0	2	2,010.02
									IMESHLR	95.0%	33.5	22.4	1	0	0	2	901.46
									IQFGTLSDFFDALDK	95.0%	66.4	22.5	5	0	0	2	1,716.85
									ITANLFR	95.0%	39.5	21.8	4	0	0	2	834.48
									LFHSLMVLEK	95.0%	38.6	22.8	0	7	0	2	1,232.67
									LLAENNEIISNIR	95.0%	78.4	21.3	9	0	0	2	1,498.82
									LPLQANVYPMTTMAYIQDAK	95.0%	56.0	21.7	7	2	0	2	2,300.13

									LTYDSYSPDTFLEMDLK	95.0%	102.0	20.0	2	0	0	2	2,053.93
									NKVEDSGIFTIK	95.0%	54.2	21.7	7	0	0	2	1,350.73
									NLGLFQHHDAITGTAK	95.0%	62.1	21.2	2	0	0	2	1,722.89
									NYQQLFDYMNSQSK	95.0%	75.5	20.2	4	0	0	2	1,781.78
									REGSFPQGQLSMLQEK	95.0%	41.6	23.0	2	4	0	2	1,850.91
									SAVNTEEEKK	95.0%	44.7	23.7	5	0	0	2	1,134.56
									SQDSLPQK	95.0%	38.3	22.5	3	0	0	2	902.46
									TFNDYFR	95.0%	41.9	21.3	3	0	0	2	962.44
									TQYIFNNMVLK	95.0%	47.3	21.8	2	0	0	2	1,386.71
									VEDSGIFTIK	95.0%	51.1	22.3	2	0	0	2	1,108.59
									VGNGHSNEAALILHR	95.0%	45.4	22.6	0	2	0	2	1,587.84
									VLLAPLGDDFR	95.0%	58.8	22.0	9	0	0	2	1,215.67
									YLVVYNPLEQDR	95.0%	52.5	23.0	9	0	0	2	1,508.78
Glucose-6-phosphate isomerase	G6PI_HUMAN	GPI	63,130	100.00%	27	33	513	59.70%	AVLHVALR	95.0%	50.1	9.0	12	0	0	2	878.56
									DPQFQK	95.0%	39.5	23.8	3	0	0	2	762.38
									DVMPEVNK	95.0%	39.4	22.3	1	0	0	2	947.45
									EWFLQAAK	95.0%	44.5	22.8	5	0	0	2	992.52
									FAAYFQQGDMESNGK	95.0%	88.9	19.7	10	0	0	2	1,708.73
									FNHFSLTLNTNHGHILVDYSK	95.0%	18.4	21.8	0	0	1	2	2,457.23
									HFVALSTNTTK	95.0%	58.8	23.1	16	0	0	2	1,218.65
									ILLANFLAQTEALMR	95.0%	100.0	19.2	88	75	0	2	1,719.95
									INYTEGR	95.0%	53.6	22.7	4	0	0	2	852.42
									KELQAAGK	94.9%	30.3	23.7	1	0	0	2	844.49
									KIEPELDGSAQVTSHDASTNGLINFIK	95.0%	59.1	19.3	0	25	4	2	2,884.47
									LFDANK	95.0%	36.7	22.6	1	0	0	2	707.37
									LFDANKDR	95.0%	50.6	23.6	1	0	0	2	978.50
									LTPFMLGALVAMYEHK	95.0%	72.8	21.9	8	0	0	2	1,852.93
									MIPCDFLIPVQTQHPIR	95.0%	34.7	21.7	0	1	0	2	2,081.07
									MIPCDFLIPVQTQHPIRK	95.0%	25.9	20.4	0	0	2	2	2,209.16
									MLVDLAK	95.0%	39.1	22.7	13	0	0	2	805.45
									NLVTEDVMR	95.0%	60.0	23.6	9	0	0	2	1,076.54
									SNTPILVDGK	95.0%	56.0	20.5	4	0	0	2	1,043.57
									SNTPILVDGKDVMPEVNK	95.0%	78.8	22.3	12	7	0	2	1,972.01
									SPEDLER	95.0%	49.1	19.5	12	0	0	2	845.40
									TFTTQETITNAETAK	95.0%	128.0	21.9	73	0	0	2	1,655.81
									TFTTQETITNAETAKEWFLQAAK	95.0%	68.0	21.1	0	2	0	2	2,629.32
									TITDVINIGIGGSDLGPLMVTEALKPYSSGGPR	95.0%	121.0	18.6	0	27	0	2	3,344.74
									TLAQLNPESSLFIIASK	95.0%	107.0	16.8	75	2	0	2	1,832.02
									VDHQTGPIVWGEPGTNGQHAFYQLIHQGTK	95.0%	23.0	20.6	0	0	1	2	3,315.63
									VFEGNRPTNSIVFTK	95.0%	69.6	21.5	9	3	0	2	1,708.90
									VWYVSNIDGTHIAK	95.0%	78.0	22.8	4	3	0	2	1,602.83
Acidic leucine-rich nuclear phosphoprotein 32 family member A	AN32A_HUMAN	ANP32A	28,568	100.00%	12	14	42	40.60%	CPNLTHLNLSGNK	95.0%	45.2	23.2	0	2	0	2	1,467.74
									DLSTIEPLKK	95.0%	35.5	20.8	2	0	0	2	1,143.66
									ELVLDNSR	95.0%	37.4	23.2	5	0	0	2	945.50
									IHLELR	95.0%	36.5	15.4	4	0	0	2	780.47
									IKDLSTIEPLK	95.0%	50.4	17.5	6	3	0	2	1,256.75
									KLELSDNR	95.0%	37.4	22.8	2	0	0	2	974.53
									LLPQLTYLDGYDR	95.0%	49.9	21.9	2	0	0	2	1,566.82
									LLPQLTYLDGYDRDDK	95.0%	47.7	22.8	2	0	0	2	1,924.97
									SLDLFNCEVTNLNDYR	95.0%	91.4	20.6	3	0	0	2	1,972.91
									SLDLFNCEVTNLNDYRENVFK	95.0%	47.0	21.0	0	2	0	2	2,590.22

									TPSDVKELVLDNSR	95.0%	56.3	22.2	2	2	0	2	1,572.82
									VSGGLEVLAEK	95.0%	83.9	22.9	5	0	0	2	1,101.62
Retinal dehydrogenase 1	AL1A1_HUMAN	ALDH1A1	54,845	100.00%	29	38	701	56.70%	ANNTFYGLSAGVFTK	95.0%	114.0	23.2	81	1	0	2	1,589.80
									DRLLLATMESMNGGK	95.0%	32.5	22.4	0	1	0	2	1,667.81
									EAGFPPGVVNIVPGYGPTAGAAISSHMDIDK	95.0%	71.4	21.3	0	31	0	2	3,083.52
									EAGFPPGVVNIVPGYGPTAGAAISSHMDIDKVAFTGSTEVGK	95.0%	42.5	17.6	0	0	4	2	4,160.07
									EEIFGPVQQIMK	95.0%	68.9	23.4	22	0	0	2	1,434.73
									ELGEYGFHEYTEVK	95.0%	69.1	20.5	50	1	0	2	1,700.78
									GYFVQPTVFSNVTDEMR	95.0%	103.0	20.0	108	2	0	2	2,005.93
									IAKEEIFGPVQQIMK	95.0%	88.1	20.5	24	29	0	2	1,746.95
									IFINNEWHDSVSGK	95.0%	88.9	22.2	20	4	0	2	1,645.80
									IFVEESIYDEFVR	95.0%	82.1	22.8	53	0	0	2	1,645.81
									IGPALSCGNTVVVKPAEQTPLTALHVASLIK	95.0%	81.8	13.2	0	5	5	2	3,184.78
									ILDLIESGK	95.0%	66.8	21.0	11	0	0	2	987.57
									ILDLIESGKK	95.0%	61.2	19.2	35	11	0	2	1,115.67
									KYILGNPLTPGVTQGPQIDK	95.0%	54.9	17.6	0	2	0	2	2,139.18
									LADLIER	95.0%	44.0	21.9	8	0	0	2	829.48
									LADLIERDR	95.0%	39.5	22.6	1	2	0	2	1,100.61
									LECGGGPWGNK	95.0%	43.8	19.7	2	0	0	2	1,174.53
									LLLATMESMNGGK	95.0%	85.5	22.6	20	0	0	2	1,396.68
									LYSNAYLNDLAGCIK	95.0%	98.9	22.3	23	0	0	2	1,714.85
									QAFQIGSPWR	95.0%	57.0	22.2	7	0	0	2	1,189.61
									RANNTFYGLSAGVFTK	95.0%	66.3	23.3	2	6	0	2	1,745.90
									RVTLELGGK	95.0%	32.6	21.1	1	0	0	2	972.58
									SLDDVIK	95.0%	50.2	23.7	5	0	0	2	789.44
									SLDDVIKR	95.0%	61.6	22.6	25	0	0	2	945.54
									TIPIDGNFFTYTR	95.0%	70.2	23.0	36	0	0	2	1,544.78
									VAFTGSTEVGK	95.0%	72.2	21.4	21	0	0	2	1,095.57
									VTLELGGK	95.0%	45.5	22.6	21	0	0	2	816.48
									YILGNPLTPGVTQGPQIDK	95.0%	84.0	19.3	5	0	0	2	2,011.09
									YILGNPLTPGVTQGPQIDKEQYDK	95.0%	38.9	20.5	0	16	0	2	2,674.37
Casein kinase II subunit alpha	CSK21_HUMAN	CSNK2A1	45,127	100.00%	5	7	24	15.30%	FNDILGR	95.0%	43.7	24.1	2	0	0	2	834.45
									FVHSENQHLVSPEALDFLDK	95.0%	36.9	21.8	0	1	3	2	2,325.15
									GGPNIITLADIVK	95.0%	39.5	17.2	2	0	0	2	1,310.77
									GGPNIITLADIVKDPVSR	95.0%	91.8	16.5	6	7	0	2	1,865.05
									TPALVFEHVNNTDFK	95.0%	85.8	22.0	3	0	0	2	1,731.87
Hypoxia up-regulated protein 1	HYOU1_HUMAN	HYOU1	111,319	100.00%	5	6	13	6.21%	AANSLEAFIFETQDK	95.0%	63.5	22.5	4	1	0	2	1,683.82
									DAVVYPILVEFTR	95.0%	36.8	20.1	4	0	0	2	1,521.83
									FFGDSAASMAIK	95.0%	41.4	21.8	2	0	0	2	1,260.59
									LPATEKPVLLSK	95.0%	43.7	14.0	1	0	0	2	1,295.79
									TLGGLEMELR	95.0%	36.0	23.8	1	0	0	2	1,134.58
14-3-3 protein beta/alpha	1433B_HUMAN	YWHAB	28,065	100.00%	7	10	69	60.20%	AKLAEQAER	95.0%	33.6	22.6	4	0	0	2	1,015.55
									AVTEQGHELSNEER	95.0%	96.5	21.6	15	7	0	2	1,598.74
									DSTLIMQLLR	95.0%	78.2	22.5	32	0	0	2	1,205.66
									EMQPTHPIR	95.0%	54.3	21.9	17	0	0	2	1,124.55
									KEMQPTHPIR	95.0%	50.7	22.5	2	4	0	2	1,252.65
									LAEQAER	95.0%	56.5	21.6	15	0	0	2	816.42
									LAEQAERYDDMAAAMK	95.0%	60.6	18.6	4	2	0	2	1,844.82
									MKGDYFR	95.0%	39.1	19.2	2	0	0	2	932.43
									NLLSVAYK	95.0%	51.2	19.1	26	0	0	2	907.53
									NLLSVAYKNVVGAR	95.0%	85.3	17.9	2	4	0	2	1,503.86

									QTTVSNSQQAYQEAFEISK	95.0%	124.0	22.0	9	0	0	2	2,159.03
									QTTVSNSQQAYQEAFEISKK	95.0%	68.9	22.1	1	6	0	2	2,287.12
									SELVQK	95.0%	37.6	25.8	2	0	0	2	703.40
									TAFDEAIAELDTLNEESYK	95.0%	113.0	21.5	7	2	0	2	2,159.00
									VISSIEQK	95.0%	73.7	23.0	12	0	0	2	903.52
									YDDMAAAMK	95.0%	54.2	12.3	4	0	0	2	1,047.41
									YLIPNATQPESK	95.0%	66.0	23.7	6	0	0	2	1,360.71
									YLSEVASGDNK	95.0%	64.3	21.7	14	0	0	2	1,182.56
Phosphoglycolate phosphatase	PGP_HUMAN	PGP	33,989	99.90%	2	2	5	8.72%	GETAVPGAPEALR	95.0%	36.3	21.9	1	0	0	2	1,267.66
									TILTLTGVSTLGDVK	95.0%	92.8	16.8	4	0	0	2	1,517.88
Intercellular adhesion molecule 5	ICAM5_HUMAN	ICAM5	97,097	100.00%	3	3	30	4.22%	ASLTLTLLR	95.0%	70.9	12.8	4	0	0	2	987.62
									SDGGAVLALGLLGPVTR	95.0%	68.6	16.1	15	0	0	2	1,595.91
									SGELGAVIEGLLR	95.0%	86.4	19.9	11	0	0	2	1,313.74
Biliverdin reductase A	BIEA_HUMAN	BLVRA	33,411	100.00%	9	10	25	35.80%	FGFPAFSGISR	95.0%	71.3	22.1	2	0	0	2	1,185.61
									FGVVVVGVGR	95.0%	83.9	18.2	3	0	0	2	988.59
									GSLLFTAGPLEEER	95.0%	56.5	23.0	3	0	0	2	1,518.78
									ILHCLGLAEEIQK	95.0%	29.6	20.1	0	2	0	2	1,523.83
									LLGQFSEK	95.0%	33.8	21.2	1	0	0	2	921.50
									LLGQFSEKELAAEK	95.0%	61.9	21.0	2	0	0	2	1,562.84
									NIFLKDQNIFVQK	95.0%	53.5	18.4	2	0	0	2	1,606.90
									NPHPSSAFLNLIGFVSR	95.0%	52.7	20.5	1	6	0	2	1,855.98
									SGSLENVPNVGVNK	95.0%	79.0	21.0	3	0	0	2	1,413.73
Replication protein A 70 kDa DNA-binding subunit	RFA1_HUMAN	RPA1	68,121	100.00%	2	2	2	4.71%	AAGPSLSHTSGGTQSK	95.0%	37.4	21.9	1	0	0	2	1,485.73
									VVPIASLTPYQSK	95.0%	30.8	19.5	1	0	0	2	1,402.79
Hypoxanthine-guanine phosphoribosyltransferase	HPRT_HUMAN	HPRT1	24,562	100.00%	8	9	59	42.70%	DLNHVCVISETGK	95.0%	42.4	22.7	2	0	0	2	1,471.72
									FFADLLDYIK	95.0%	75.1	22.4	20	0	0	2	1,244.66
									NVLIVEDIIDTGK	95.0%	87.0	21.2	11	0	0	2	1,428.80
									SVGYKPDFVGFEIPDK	95.0%	32.9	22.5	0	1	0	2	1,797.91
									TMQTLLSLVR	95.0%	75.6	20.3	7	0	0	2	1,177.66
									VASLLVK	95.0%	36.5	19.1	2	0	0	2	729.49
									VFIPHGLIMDR	95.0%	35.4	21.4	3	8	0	2	1,313.70
									VIGGDDLSTLTGK	95.0%	98.5	22.5	5	0	0	2	1,275.68
Plectin-1	PLEC1_HUMAN	PLEC1	531,766	100.00%	49	50	186	12.00%	AELELELGR	95.0%	48.5	20.9	1	0	0	2	1,029.56
									AKLEQLFQDEVAK	95.0%	56.8	22.1	1	0	0	2	1,518.82
									ALQALEELR	95.0%	43.4	21.5	3	0	0	2	1,042.59
									AQLEPVASPAK	95.0%	39.5	19.3	4	0	0	2	1,110.62
									AQVEQELTTLR	95.0%	72.9	23.1	13	0	0	2	1,287.69
									ASDSELER	95.0%	38.4	21.1	1	0	0	2	906.42
									DSQDAGGFGPEDR	95.0%	64.9	14.9	2	0	0	2	1,350.56
									EAEGQLQK	95.0%	32.1	22.5	1	0	0	2	902.46
									EAQAVPATLPELEATK	95.0%	42.5	19.9	1	0	0	2	1,667.89
									EQELQQTLQQEQSVLDQLR	95.0%	72.9	21.5	3	0	0	2	2,313.17
									GGAEGELQALR	95.0%	86.0	22.4	7	0	0	2	1,100.57
									GIYQSLEGAVQAGQLK	95.0%	71.7	21.2	2	0	0	2	1,661.89
									GTQGAEEVLR	95.0%	39.0	22.9	2	0	0	2	1,059.54
									IISLETYNLLR	95.0%	52.2	17.6	2	0	0	2	1,334.77
									LAAIGEATR	95.0%	44.9	24.1	3	0	0	2	901.51
									LEDLLQDAQDEKEQLNEYK	95.0%	52.5	21.6	0	3	0	2	2,321.12
									LFNAIIHR	95.0%	57.5	18.6	2	0	0	2	983.58
									LKAEAELLQQQK	95.0%	38.3	18.9	1	0	0	2	1,398.80
									LLDPEDVDVPQPDEK	95.0%	60.6	22.5	4	0	0	2	1,708.83

									LLFNDVQTLK	95.0%	61.9	20.0	4	0	0	2	1,190.68
									LQAEEVAQQK	95.0%	57.1	23.1	12	0	0	2	1,143.60
									LQLEATER	95.0%	32.8	22.5	1	0	0	2	959.52
									LQNVQIALDYLR	95.0%	85.0	19.5	4	0	0	2	1,445.81
									LRAETEQGEQQR	95.0%	32.7	22.7	1	2	0	2	1,444.71
									MQEEVVR	95.0%	33.3	22.2	1	0	0	2	906.44
									NDDIADGNPK	95.0%	43.7	17.8	2	0	0	2	1,058.48
									QLAEEDLAQQR	95.0%	56.9	22.0	3	0	0	2	1,300.65
									QLAEGTAQQR	95.0%	49.4	20.5	3	0	0	2	1,101.57
									QLEMSAEAER	95.0%	50.4	20.6	4	0	0	2	1,179.53
									QLLEEELAR	95.0%	48.9	24.2	4	0	0	2	1,100.60
									QQEELLAEENQR	95.0%	45.2	22.3	1	0	0	2	1,486.71
									QSSEAEIQAK	95.0%	32.5	23.8	1	0	0	2	1,090.54
									QTNLENLDQAFSVAER	95.0%	129.0	23.8	6	0	0	2	1,834.89
									QVEEEILALK	95.0%	55.4	22.9	6	0	0	2	1,171.66
									QVQVALETAQR	95.0%	40.5	22.9	2	0	0	2	1,242.68
									RQEQIQAMPLADSQAVR	95.0%	40.8	22.3	0	1	0	2	1,956.99
									SAEAELQSKR	94.8%	30.2	23.1	1	0	0	2	1,118.58
									SIITYVSSLYDAMPR	95.0%	51.7	21.8	4	0	0	2	1,731.86
									SIQEELQQLR	95.0%	45.6	22.3	9	0	0	2	1,243.66
									SKEQAELEAAR	95.0%	30.6	23.5	0	3	0	2	1,231.63
									SLAAEEEAAR	95.0%	69.9	21.3	7	0	0	2	1,046.51
									SLQEEHVAVAQLR	95.0%	40.2	22.1	0	3	0	2	1,479.79
									SLSAIYLEK	95.0%	45.0	20.6	4	0	0	2	1,023.57
									SNAEDTLR	95.0%	44.2	22.5	1	0	0	2	905.43
									SQVEEELFSVR	95.0%	83.6	22.9	14	0	0	2	1,322.66
									VLALPEPSPAAPTLR	95.0%	63.3	16.7	9	0	0	2	1,531.89
									VQSGSESVIQEYVDLR	95.0%	93.1	22.2	13	0	0	2	1,808.90
									VTLVQTLEIQR	95.0%	53.3	18.5	1	0	0	2	1,299.76
									WQAVLAQTDVR	95.0%	59.0	23.2	3	0	0	2	1,286.69
Receptor-type tyrosine-protein phosphatase S	PTPRS_HUMAN	PTPRS	217,075	100.00%	23	26	149	18.60%	DFLPVDPSASNGR	95.0%	33.9	21.3	1	0	0	2	1,374.67
									EDQLPSGFPNIDMGPQLK	95.0%	90.6	21.9	2	0	0	2	2,001.96
									ETELPAAAEPGAENALTLQGLKPDTAYDLQVR	95.0%	65.9	20.8	0	2	0	2	3,381.72
									EVNGIPPTTTQILLEALEK	95.0%	26.0	15.8	0	1	0	2	2,066.14
									GAVLGRPTLSVQQTPEGSLLAR	95.0%	51.8	15.8	0	8	0	2	2,250.26
									GGLGEEAAEVLSIPEDTPR	95.0%	43.8	21.5	0	2	0	2	1,939.96
									HNVDDSLLTTVGSLLEDETYTVR	95.0%	30.1	21.0	0	1	0	2	2,577.27
									IQYNGLTLDVDGR	95.0%	83.6	23.2	1	0	0	2	1,463.75
									ITTVAHTEVGPGPESSPVVVR	95.0%	80.0	20.3	2	12	0	2	2,132.14
									LTVLREDQLPSGFPNIDMGPQLK	95.0%	45.8	19.2	0	3	0	2	2,584.35
									SPQGLGAFTPVVR	95.0%	66.9	21.2	17	0	0	2	1,328.73
									SQDGPYQIKEDITTTR	95.0%	26.0	22.6	0	1	0	2	1,851.91
									SRGGLGEEAAEVLSIPEDTPR	95.0%	40.9	21.9	0	2	0	2	2,183.09
									TDEDVPSAPPR	95.0%	60.6	21.2	2	0	0	2	1,183.56
									TFDPTTSYVVEDLKPNTEYAFR	95.0%	53.9	21.5	0	6	0	2	2,593.25
									TGEQAPASAPR	95.0%	79.9	22.1	40	0	0	2	1,084.54
									TLQSKPSAPPQDVK	95.0%	48.8	20.1	2	1	0	2	1,495.81
									TQQGVPGQPMNLR	95.0%	37.8	21.8	7	0	0	2	1,441.72
									VLAFTSVGDGPLSDPIQVK	95.0%	89.2	20.4	27	0	0	2	1,943.05
									WEPPAGTAEDQVLGYR	95.0%	77.9	21.8	2	0	0	2	1,788.86
									WMQGAEDLTPEDDMPVGR	95.0%	86.8	17.2	2	0	0	2	2,078.88

									YRPLGSEDPEPK	95.0%	44.2	21.4	1	2	0	2	1,387.69
									YSSPANLYVR	95.0%	55.9	23.1	2	0	0	2	1,169.60
Malate dehydrogenase, mitochondrial	MDHM_HUMAN	MDH2	35,486	100.00%	20	24	247	65.40%	AGAGSATLSMAYAGAR	95.0%	107.0	22.1	37	0	0	2	1,454.71
									AKAGAGSATLSMAYAGAR	95.0%	39.6	22.2	0	2	0	2	1,669.83
									ANTFVAELK	95.0%	64.2	21.7	6	0	0	2	992.54
									FVFSLVDAMNGK	95.0%	83.1	22.4	8	0	0	2	1,327.67
									GCDVVVIPAGVPR	95.0%	73.9	21.4	2	0	0	2	1,338.72
									GYLGPEQLPDCLK	95.0%	61.8	23.3	2	0	0	2	1,489.74
									HGVYNPNK	95.0%	36.8	21.1	2	0	0	2	928.46
									IFGVTTLDIVR	95.0%	66.5	17.3	42	0	0	2	1,233.72
									IQEAGTEVVK	95.0%	48.0	23.4	9	0	0	2	1,073.58
									KGEDFVK	95.0%	44.2	22.4	3	0	0	2	822.44
									LTLYDIAHTPGVAADLSHIETK	95.0%	98.1	18.7	2	33	2	2	2,365.24
									MISDAIPELK	95.0%	56.8	23.6	23	0	0	2	1,116.60
									NSPLVSR	95.0%	43.0	22.4	2	0	0	2	772.43
									SQETECTYFSTPLLLGK	95.0%	106.0	22.3	2	0	0	2	1,973.95
									TIIPLISQCTPK	95.0%	53.9	19.9	2	0	0	2	1,370.77
									VAVLGASGGIGQPLSLLLK	95.0%	130.0	3.0	26	2	0	2	1,793.09
									VAVLGASGGIGQPLSLLLKNSPLVSR	95.0%	33.0	10.0	0	2	0	2	2,546.50
									VDFPQDQLTALTGR	95.0%	101.0	22.8	25	0	0	2	1,560.80
									VNVPVIGGHAGK	95.0%	48.7	17.7	3	6	0	2	1,147.66
									VSSFEEK	95.0%	39.8	21.5	4	0	0	2	825.40
Cytoplasmic aconitate hydratase	ACOC_HUMAN	ACO1	98,383	100.00%	8	9	16	16.80%	ANYLASPPLVIAYAIAGTIR	95.0%	83.9	15.7	1	0	0	2	2,074.17
									DFNDPSQDPDFTQVVELDLK	95.0%	72.3	20.6	2	0	0	2	2,322.08
									FVEFFGPGVAQLSIADR	95.0%	98.8	21.5	2	0	0	2	1,852.96
									GPFLLGIK	95.0%	37.6	21.2	1	0	0	2	844.53
									QAPQTIHLPSGEILDVFDAAER	95.0%	45.8	20.8	0	2	0	2	2,407.23
									SNLVGMGVIPLEYLPGENADALGLTGQER	95.0%	38.0	20.4	0	1	0	2	3,029.53
									VILQDFTGVPAVVDFAAMR	95.0%	100.0	21.0	3	2	0	2	2,065.08
									YQQAGLPLIVLAGK	95.0%	73.7	15.6	2	0	0	2	1,470.87
Elongation factor 2	EF2_HUMAN	EEF2	95,322	100.00%	35	41	533	46.00%	AGIIASAR	95.0%	61.7	23.9	2	0	0	2	758.45
									ALLELQLEPEELYQTFQR	95.0%	88.8	20.9	19	4	0	2	2,220.16
									ARPFPDGLAEDIDKGEVSAR	95.0%	108.0	22.1	0	49	11	2	2,143.08
									AYLPVNESFGFTADLR	95.0%	97.1	21.9	50	0	0	2	1,799.90
									CLYASVLTAQPR	95.0%	73.1	22.3	4	0	0	2	1,378.72
									EDLYLKPIQR	95.0%	42.0	19.9	2	0	0	2	1,274.71
									EGIPALDNFLDKL	95.0%	79.3	22.3	92	0	0	2	1,444.77
									ETVSEESNVLCLSK	95.0%	88.6	22.6	5	0	0	2	1,594.76
									FSVSPVVR	95.0%	32.7	20.6	3	0	0	2	890.51
									GEGQLGPAER	95.0%	51.5	21.0	39	0	0	2	1,013.50
									GGGQIIPTAR	95.0%	52.1	20.3	8	0	0	2	969.55
									GHVFEESQVAGTPMFVVK	95.0%	119.0	22.4	12	5	0	2	1,977.97
									GPLMMYISK	95.0%	40.7	20.8	3	0	0	2	1,071.52
									GVQYLNEIK	95.0%	57.5	21.4	6	0	0	2	1,063.58
									IKPVLMMNK	95.0%	26.2	22.3	0	1	0	2	1,105.61
									IMGPNYTPGK	95.0%	47.4	23.0	4	0	0	2	1,093.54
									IMGPNYTPGKK	95.0%	41.9	22.4	0	10	0	2	1,221.63
									KEDLYLKPIQR	95.0%	52.9	18.1	2	16	0	2	1,402.81
									KIWCFGPDGTGPNILTDITK	95.0%	44.1	21.3	0	2	0	2	2,233.13
									NMSVIAHVDHGK	95.0%	55.8	22.8	7	0	0	2	1,323.65
									NPADLPK	95.0%	33.0	18.8	2	0	0	2	754.41

									QFAEMYVAK	95.0%	43.6	21.1	2	0	0	2	1,102.52
									RCLYASVLTAQPR	95.0%	28.7	21.7	0	2	0	2	1,534.82
									SDPVVSYR	95.0%	40.4	22.6	7	0	0	2	922.46
									STAISLFYELSENDLNFIK	95.0%	122.0	22.1	6	0	0	2	2,204.11
									STLTDSLVCK	95.0%	47.3	23.6	2	0	0	2	1,123.57
									TFCQLILDPIFK	95.0%	59.2	21.4	16	0	0	2	1,494.80
									TGTITTFEHAHNMR	95.0%	58.8	20.5	6	12	1	2	1,631.76
									VFDAIMNFK	95.0%	44.6	20.8	15	0	0	2	1,100.55
									VFDAIMNFKK	95.0%	38.0	22.6	3	0	0	2	1,228.64
									VFSGLVSTGLK	95.0%	90.0	18.8	58	0	0	2	1,107.64
									VNFTVDQIR	95.0%	80.2	23.8	30	0	0	1	1,091.59
									WLPAGDALLQMITIHLPSPVTAQK	95.0%	57.1	16.3	0	10	0	2	2,616.42
									YEWDVAEAR	95.0%	57.7	19.6	3	0	0	2	1,138.52
									YVEPIEDVPCGNIVGLVGVDQFLVK	95.0%	48.8	19.3	0	2	0	2	2,759.43
Cullin-associated NEDD8-dissociated protein 1	CAND1_HUMAN	CAND1	136,363	100.00%	19	23	144	20.20%	ADVFHAYLSLLK	95.0%	31.7	20.6	0	2	0	2	1,376.76
									ALTLIAGSPLK	95.0%	73.4	17.4	13	0	0	2	1,083.68
									AVAALLTIPEAEK	95.0%	75.1	17.6	8	0	0	2	1,325.77
									CLDAVVSTR	95.0%	41.2	23.7	1	0	0	2	1,020.51
									DLLDTVLPHLYNETK	95.0%	62.3	22.3	4	0	0	2	1,770.93
									EGPAVVGQFIQDVK	95.0%	61.6	21.9	12	0	0	2	1,486.79
									FMATNDLMTELQK	95.0%	69.3	20.2	3	0	0	2	1,573.72
									FTISDHPQPIDPLLK	95.0%	47.4	20.8	2	4	0	2	1,720.93
									HEMLPEFYK	95.0%	44.3	22.6	2	0	0	2	1,209.56
									IDLRPVLGEGVPILASFLR	95.0%	51.6	7.8	0	5	0	2	2,065.22
									ISGSILNELIGLVR	95.0%	95.3	15.2	10	0	0	2	1,483.89
									ITSEALLVTQQLVK	95.0%	74.0	14.3	5	2	0	2	1,542.91
									LGTLSALDILIK	95.0%	76.7	13.0	17	0	0	2	1,256.78
									LTLIDPETLLPR	95.0%	61.1	15.3	26	0	0	2	1,380.81
									MLTFLMLVR	95.0%	58.3	20.3	9	0	0	2	1,155.63
									MLTGPVYSQSTALTHK	95.0%	66.6	22.9	2	2	0	2	1,749.88
									SVILEAFSSPSEEVK	95.0%	82.4	22.5	8	0	0	2	1,621.83
									TVSPALISR	95.0%	33.6	21.0	2	0	0	2	943.56
									VIRPLDQPSSFDATPYIK	95.0%	54.7	20.6	2	3	0	2	2,047.09
Proteasome subunit beta type-8	PSB8_HUMAN	PSMB8	30,337	100.00%	3	3	5	14.50%	FQHGVIAAVDSR	95.0%	41.3	22.2	2	0	0	2	1,299.68
									KGPGLYYVDEHGTR	95.0%	60.8	22.9	0	2	0	2	1,591.79
									VESTDVSDLLHQYR	95.0%	31.9	22.7	0	1	0	2	1,661.81
Protein ERGIC-53	LMAN1_HUMAN	LMAN1	57,531	100.00%	4	5	11	10.40%	GAGMPGQHGQITQQELDTVVK	95.0%	43.5	21.7	0	2	0	2	2,210.09
									GHPDLQGQPAEEIFESVGDR	95.0%	98.9	20.6	1	4	0	2	2,181.02
									YVSSLTEEISK	95.0%	52.8	22.2	2	0	0	2	1,255.64
									YVSSLTEEISKR	95.0%	72.4	21.7	2	0	0	2	1,411.74
Protein MEMO1	MEMO1_HUMAN	MEMO1	33,716	100.00%	4	5	10	18.20%	HPIGVLLNAITELQK	95.0%	55.0	12.3	2	3	0	2	1,645.96
									IFILGPSHHVPLSR	95.0%	31.6	15.4	0	1	0	2	1,572.90
									MGMSIIEQLDPVSFSNYLK	95.0%	79.3	22.5	2	0	0	2	2,204.06
									TGMFER	95.0%	39.5	17.2	2	0	0	2	756.34
Heterogeneous nuclear ribonucleoprotein M	HNRPM_HUMAN	HNRNPM	77,499	99.50%	2	2	5	3.70%	AFITNIPFDVK	95.0%	54.8	20.4	4	0	0	2	1,264.69
									VGEVTYVELLMDAEGK	95.0%	53.6	22.3	1	0	0	2	1,768.87
60S ribosomal protein L4	RL4_HUMAN	RPL4	47,681	100.00%	4	5	9	11.20%	APIRPDIVNFVHTNLR	95.0%	35.2	15.9	0	2	1	2	1,862.04
									IEEVPELPLVVEDK	95.0%	33.3	20.0	1	0	0	2	1,608.87
									NIPGITLLNVSK	95.0%	33.9	17.1	2	0	0	2	1,268.76
									SPEIQR	95.0%	48.4	20.7	3	0	0	2	729.39
Glycogen phosphorylase, liver form	PYGL_HUMAN	PYGL	97,134	100.00%	28	35	115	47.00%	AAPGYHMAK	95.0%	38.3	21.3	1	0	0	2	961.46

									APNDFNLR	95.0%	59.2	24.1	6	0	0	2	946.47
									DFNVGDYIQAVLDR	95.0%	91.8	22.8	24	2	0	2	1,624.80
									DFSELEPDKFQNK	95.0%	43.7	21.7	1	0	0	2	1,596.75
									DIINMLFYHDR	95.0%	36.7	22.4	3	0	0	2	1,452.69
									DYYFALAHTVR	95.0%	57.9	22.7	4	1	0	2	1,355.67
									EYAQNIWNVEPSDLK	95.0%	39.5	22.8	2	0	0	2	1,805.87
									EYYEALPELK	95.0%	45.1	22.6	2	0	0	2	1,254.63
									FSQFLETEYK	95.0%	71.6	22.9	4	0	0	2	1,291.62
									GAGTVFDAFPDQVAIQLNDTHPALAIPELMR	95.0%	54.3	20.0	0	3	0	2	3,323.67
									GIVGVENVAELK	95.0%	60.6	21.0	2	0	0	2	1,227.70
									GIVGVENVAELKK	95.0%	46.1	17.7	2	0	0	2	1,355.79
									HLEIIYEINQK	95.0%	55.2	21.9	4	0	0	2	1,399.76
									IDDVAALDKK	95.0%	28.3	24.4	0	2	0	2	1,087.60
									IFVDIEKLPWSK	95.0%	40.5	18.7	0	2	0	2	1,474.83
									IGEDYVKDLSQLTK	95.0%	72.8	22.3	2	0	0	2	1,608.85
									IHSDIVK	95.0%	32.6	17.9	1	0	0	2	811.47
									INPSSMFDVQVK	95.0%	71.4	22.3	4	0	0	2	1,380.68
									LHSFLGDDVFLR	95.0%	62.8	22.8	4	5	0	2	1,418.74
									LITSVADVVNNDPMVGSK	95.0%	106.0	21.5	7	1	0	2	1,874.95
									LVIDQIDNGFFSPK	95.0%	93.6	22.3	4	0	0	2	1,592.83
									MSLIEEEGSKR	95.0%	41.7	23.0	2	1	0	2	1,294.63
									NLAENISR	95.0%	38.8	22.9	3	0	0	2	916.49
									SRPEFMLPVHFYGK	95.0%	43.0	22.4	0	4	0	2	1,723.86
									TFAYTNHTVLPEALER	95.0%	80.2	22.1	2	4	0	2	1,861.94
									TNGITPR	95.0%	37.1	22.6	1	0	0	2	758.42
									TQQHYYDK	95.0%	41.2	21.1	1	0	0	2	1,082.49
									VIFLENYR	95.0%	52.4	21.0	16	0	0	2	1,053.57
									VIPATDLSEQISTAGTEASGTGNMK	95.0%	93.5	21.3	2	2	0	2	2,494.20
									VLYPNDNFFEGK	95.0%	64.3	21.8	9	0	0	2	1,442.70
									VSQLYMNPK	95.0%	31.4	22.0	1	0	0	2	1,095.55
									WLLLCNPGLAELIAEK	95.0%	49.1	19.4	2	0	0	2	1,840.00
									YEYGIFNQK	95.0%	44.2	22.4	2	0	0	2	1,161.56
40S ribosomal protein S12	RS12_HUMAN	RPS12	14,497	100.00%	4	4	8	35.60%	ESQAKDVIEEYFK	95.0%	66.9	22.9	2	0	0	2	1,585.78
									LGEWVGLCK	95.0%	52.6	23.9	2	0	0	2	1,061.55
									LVEALCAEHQINLIK	95.0%	33.9	18.5	0	2	0	2	1,750.95
									TALIHDGLAR	95.0%	32.4	18.9	0	2	0	2	1,066.60
Moesin	MOES_HUMAN	MSN	67,804	100.00%	40	51	357	56.80%	AKFYPEDVSEELIQDITQR	95.0%	107.0	21.5	2	3	0	2	2,281.14
									ALELEQER	95.0%	46.5	23.3	11	0	0	2	987.51
									ALTSELANAR	95.0%	85.4	22.9	9	0	0	2	1,045.56
									ALTSELANARDESK	95.0%	89.3	23.3	3	0	0	2	1,504.76
									AMLENEK	95.0%	33.7	22.5	2	0	0	2	850.40
									APDFVFYAPR	95.0%	83.9	21.7	44	0	0	2	1,182.59
									AQMVQEDLEK	95.0%	60.3	21.6	16	0	0	2	1,206.57
									AQMVQEDLEKTR	95.0%	43.4	22.9	1	0	0	2	1,463.72
									AQQELEEQTR	95.0%	50.4	22.4	1	0	0	2	1,231.59
									AQQELEEQTRR	95.0%	26.1	22.4	0	1	0	2	1,387.69
									EDAVLEYLK	95.0%	48.5	23.2	4	0	0	2	1,079.56
									EKEELMER	95.0%	33.3	21.6	1	0	0	2	1,079.50
									ESPLLFK	95.0%	35.4	20.7	2	0	0	2	833.48
									EVWFFGLQYQDTK	95.0%	83.1	22.0	3	0	0	2	1,660.80
									FVIKPIDK	95.0%	36.6	17.2	7	0	0	2	959.59

									FYPEDVSEELIQDITQR	95.0%	102.0	22.3	13	12	0	2	2,082.00
									GMLREDAVLEYLK	95.0%	37.9	21.3	0	3	0	2	1,552.80
									IAQDLEMYGVNYFSIK	95.0%	111.0	22.4	4	0	0	2	1,906.93
									IGFPWSEIR	95.0%	62.2	23.4	19	0	0	2	1,104.58
									ISQLEMAR	95.0%	58.0	23.4	15	0	0	2	963.49
									KAPDFVFYAPR	95.0%	54.9	22.3	12	1	0	2	1,310.69
									KAQQELEEQTR	95.0%	76.1	22.6	5	6	0	2	1,359.69
									KESPLLFK	95.0%	33.8	17.6	2	0	0	2	961.57
									KPDTIEVQQMK	95.0%	48.7	23.4	4	0	0	2	1,332.68
									KTANDMIHAENMR	95.0%	60.1	19.3	3	3	0	2	1,562.71
									KTQEQLALEMAELTAR	95.0%	91.8	22.3	2	6	0	2	1,847.95
									LFFLQVK	95.0%	41.5	18.9	14	0	0	2	894.55
									LKQIEEQTK	95.0%	38.5	22.3	1	0	0	2	1,116.63
									NISFNDKK	94.8%	30.3	22.7	1	0	0	2	965.51
									QEAEEAKEALLQASR	95.0%	75.2	22.7	2	2	0	2	1,672.85
									QLFDQVVK	95.0%	56.5	21.4	9	0	0	2	976.55
									QRIDEFESM	95.0%	43.8	18.1	2	0	0	2	1,170.51
									RALELEQER	95.0%	39.2	23.3	2	0	0	2	1,143.61
									RKPDTIEVQQMK	95.0%	53.8	22.3	13	38	0	2	1,488.78
									SGYLAGDK	95.0%	61.7	23.4	2	0	0	2	810.40
									TAMSTPHVAEPAENEQDEQDENGAEASADLR	95.0%	46.9	15.2	0	2	0	2	3,328.42
									TANDMIHAENMR	95.0%	50.1	16.9	5	1	0	2	1,434.61
									TQEQLALEMAELTAR	95.0%	95.3	21.6	6	2	0	2	1,719.86
									VTAQDVR	95.0%	38.7	24.3	1	0	0	2	788.43
									VTTMDAELEFAIQPNTTGK	95.0%	114.0	22.4	28	6	0	2	2,082.01
Eukaryotic translation initiation factor 3 subunit H	EIF3H_HUMAN	EIF3H	39,913	100.00%	4	5	9	19.60%	EGTGSTATSSSSTAGAAGK	95.0%	64.7	20.3	1	0	0	2	1,627.74
									LFMAQALQEYNN	95.0%	55.5	21.3	2	0	0	2	1,457.67
									NLQLLMDRVDEMSQDIVK	95.0%	33.1	21.9	0	2	0	2	2,179.07
									SAVADKHELLSLASSNHLGK	95.0%	65.8	20.0	0	2	2	2	2,077.10
Splicing factor 3B subunit 3	SF3B3_HUMAN	SF3B3	135,561	100.00%	20	22	136	21.00%	AVMISAIEK	95.0%	30.6	22.6	1	0	0	2	977.53
									FLAVGLVDNTVR	95.0%	71.0	20.3	20	0	0	2	1,303.74
									HIANYISGIQTIGHR	95.0%	67.8	21.0	0	7	0	2	1,679.90
									ILELLRPDPNTGK	95.0%	34.8	17.6	2	0	0	2	1,465.84
									ITLETDEDMVTEIR	95.0%	70.2	21.9	1	0	0	2	1,680.80
									IVILEYQPSK	95.0%	58.0	19.1	6	0	0	2	1,189.68
									IVPGQFLAVDPK	95.0%	38.1	19.0	3	0	0	2	1,283.74
									LGAVFNQVAFPLQYTPR	95.0%	96.6	20.2	29	0	0	2	1,921.03
									LPPNTNDEVDEDPTGNK	95.0%	67.6	19.6	4	0	0	2	1,854.84
									LTISSPLEAHK	95.0%	35.1	20.3	2	1	0	2	1,195.67
									MFLYNLTLQR	95.0%	47.7	22.1	5	0	0	2	1,314.69
									MQGQEAVLAMSSR	95.0%	81.7	20.3	4	0	0	2	1,439.66
									NENQLIIFADDTYPR	95.0%	112.0	22.2	9	2	0	2	1,808.88
									NFGDQPDIR	95.0%	44.0	22.3	1	0	0	2	1,061.50
									NVSEELDRTPPEVSK	95.0%	53.2	22.4	2	0	0	2	1,699.85
									QDELGER	95.0%	32.1	19.3	1	0	0	2	846.40
									SMFFFLAQTEQGDIFK	95.0%	121.0	21.9	4	0	0	2	1,924.92
									SVAGGFVYTYK	95.0%	67.8	23.8	2	0	0	2	1,191.60
									TPVEEVPAAIAPFQGR	95.0%	85.9	21.9	13	0	0	2	1,681.89
									TVLDPVTGDLSDTR	95.0%	91.9	22.8	17	0	0	2	1,488.75
Protocadherin alpha-4	PCDA4_HUMAN	PCDHA4	102,276	99.50%	2	2	5	3.06%	DINDNPPVFPATQK	95.0%	30.9	22.1	1	0	0	2	1,555.78
									IAQDLGLELAELVPR	95.0%	66.4	17.6	4	0	0	2	1,636.93

Proteasome assembly chaperone 1	PSMG1_HUMAN	PSMG1	32,836	100.00%	3	3	6	15.30%	AGTEDEEEEEEGRR	95.0%	29.6	14.6	0	2	0	2	1,635.67
									LDLITVEAFKPILSTR	95.0%	37.7	13.4	0	2	0	2	1,816.06
									TSESTGSLPSPFLR	95.0%	50.3	22.6	2	0	0	2	1,478.75
Lamina-associated polypeptide 2, isoform alpha	LAP2A_HUMAN	TMPO	75,476	100.00%	5	5	25	13.00%	GPPDFSSDEEREPTPVLGSGAAAAGR	95.0%	82.3	21.1	0	3	0	2	2,570.21
									PEFLEDPSVLTK	95.0%	60.8	23.2	4	0	0	1	1,374.72
									QEDKDDLDVTELTNEDLLDQLVK	95.0%	44.6	21.3	0	3	0	2	2,688.31
									SSTPLPTISSSAENTR	95.0%	81.3	23.2	7	0	0	2	1,647.82
									YGVNPGPIVGTTR	95.0%	51.3	22.4	8	0	0	2	1,330.71
Ephrin-A5	EFNA5_HUMAN	EFNA5	26,279	99.50%	2	2	2	9.21%	EYFYISSAIPDNGR	95.0%	52.8	21.7	1	0	0	2	1,631.77
									TIGVHDR	95.0%	32.1	21.0	1	0	0	2	797.43
Phospholipase D3	PLD3_HUMAN	PLD3	54,688	100.00%	2	2	4	4.29%	LFVVPADEAQAR	95.0%	65.6	22.5	3	0	0	2	1,315.70
									SQLEAIFLR	95.0%	32.6	21.0	1	0	0	2	1,076.61
LIM and SH3 domain protein 1	LASP1_HUMAN	LASP1	29,699	100.00%	3	4	6	14.90%	GFSVVADTPELQR	95.0%	64.0	23.4	1	0	0	2	1,418.73
									LKQQSELQSQVR	95.0%	68.8	22.1	2	1	0	2	1,443.79
									MGPSGGEGMEPERR	95.0%	28.0	16.3	0	2	0	2	1,521.64
Major prion protein	PRIO_HUMAN	PRNP	27,643	99.50%	2	2	12	9.09%	VVEQMCITQYER	95.0%	70.2	21.6	6	0	0	2	1,571.72
									YPGQGSPGGNR	95.0%	47.6	20.5	6	0	0	2	1,089.51
4-trimethylaminobutyraldehyde dehydrogenase	AL9A1_HUMAN	ALDH9A1	53,784	100.00%	4	5	9	9.11%	EILDKFTEEVVK	95.0%	64.5	21.8	4	0	0	2	1,449.78
									EVNLAVQNAK	95.0%	46.8	20.8	1	0	0	2	1,085.60
									GIKPVTLELGGK	95.0%	37.0	15.3	1	1	0	2	1,211.74
									VEPADASGTEK	95.0%	40.6	21.3	2	0	0	2	1,103.52
Elongation factor 1-alpha 1	EF1A1_HUMAN	EEF1A1	50,123	100.00%	16	20	817	42.20%	DGNASGTTLLEALDCILPPTRPTDKPLR	95.0%	60.5	19.6	0	6	0	2	3,021.57
									EHALLAYTLGVK	95.0%	65.7	20.5	84	15	0	2	1,314.74
									EVSTYIK	95.0%	31.1	21.6	1	0	0	2	839.45
									FEKEAAEMGK	95.0%	27.6	20.3	0	1	0	2	1,155.54
									IGGIGTVPVGR	95.0%	90.3	16.1	86	0	0	2	1,025.61
									KLEDGPK	95.0%	30.8	19.8	2	0	0	2	786.44
									LPLQDVYK	95.0%	60.1	21.1	31	0	0	2	975.55
									QLIVGVNK	95.0%	34.2	17.1	2	0	0	2	870.54
									QTVAVGVIK	95.0%	35.5	18.7	2	0	0	2	914.57
									SGDAAIVDMVPGKPMCVESFSDYPPLGR	95.0%	60.2	20.0	0	4	0	2	3,027.39
									STTTGHLIYK	95.0%	51.8	21.3	35	0	0	2	1,120.60
									THINIVVIGHVDSGK	95.0%	84.4	20.2	10	353	0	2	1,588.88
									VETGVLKPGMVVTFAPVNVTTEVK	95.0%	56.2	16.4	7	69	0	2	2,531.38
									YEEIVK	95.0%	34.7	20.6	4	0	0	2	780.41
									YEEIVKEVSTYIK	95.0%	54.4	21.4	1	0	0	2	1,600.85
									YYVTIIDAPGHR	95.0%	66.5	22.9	46	58	0	2	1,404.73
Septin-9	SEPT9_HUMAN	SEPT9	65,384	100.00%	2	2	2	4.78%	APVDFGYVGIDSILEQMR	95.0%	35.1	22.3	1	0	0	2	2,026.00
									YLQEEVNINR	95.0%	39.8	23.5	1	0	0	2	1,277.65
26S proteasome non-ATPase regulatory subunit 6	PSMD6_HUMAN	PSMD6	45,515	100.00%	13	15	27	34.40%	ANEDELK	95.0%	39.8	20.5	1	0	0	2	818.39
									DNNMAPYYEALCK	95.0%	37.9	16.2	1	0	0	2	1,604.67
									GAEILEVLHSLPAVR	95.0%	49.3	16.6	0	2	0	2	1,603.92
									IDKVNEIVETNRPDSK	95.0%	60.0	20.8	2	2	0	2	1,856.97
									IGLFYMDNDLITR	95.0%	75.3	22.4	3	0	0	2	1,586.79
									IHAYSQLLESYR	95.0%	34.0	22.6	0	2	0	2	1,479.76
									LDIVFYLLR	95.0%	36.2	16.4	2	0	0	2	1,151.68
									NLGESEIRDAMMAK	94.8%	30.3	20.8	1	0	0	2	1,596.74
									PLENLEEEGLPK	95.0%	64.6	22.1	2	0	0	1	1,367.71
									PLENLEEEGLPKNPDLR	95.0%	55.7	22.6	0	2	0	1	1,963.01
									RLDEELEDAEK	95.0%	52.3	22.6	2	0	0	2	1,346.64
									TVALGHR	95.0%	35.2	21.0	1	0	0	2	753.44

									VNEIVETNRPDSK	95.0%	55.9	23.2	2	2	0	2	1,500.77
Chitinase domain-containing protein 1	CHID1_HUMAN	CHID1	44,923	100.00%	7	7	22	24.20%	EPVVGAR	95.0%	30.9	20.6	1	0	0	2	727.41
									FTQISPVWLQLK	95.0%	63.9	18.1	3	0	0	2	1,459.83
									HVVFYPTLK	95.0%	35.1	21.5	2	0	0	2	1,103.63
									LLALLVIPPAITPGTDQLGMFTHK	95.0%	29.7	14.1	0	3	0	2	2,562.44
									NVLDSEDEIEELSK	95.0%	103.0	21.6	4	0	0	2	1,619.77
									SQFSDKPVQDR	95.0%	60.5	22.7	7	0	0	2	1,306.64
									VGLIHMLTHLAEALHQAR	95.0%	23.1	19.3	0	0	2	2	2,026.10
Macrophage-capping protein	CAPG_HUMAN	CAPG	38,500	100.00%	8	9	48	28.20%	ANAQAAALYK	95.0%	58.8	23.2	5	0	0	2	1,020.55
									AQVEIVTDGEEPAEMIQVLGPKPALK	95.0%	57.4	19.5	0	7	0	2	2,778.46
									DLALAIR	95.0%	35.1	20.0	2	0	0	2	771.47
									EGNPEEDLTADK	95.0%	74.6	19.1	4	0	0	2	1,317.58
									QAALQVAEGFISR	95.0%	91.2	22.5	8	0	0	2	1,389.75
									SNILER	94.7%	30.3	25.1	1	0	0	2	731.41
									VSDATGQMNLTK	95.0%	77.6	22.4	18	0	0	2	1,280.62
									YQEGGVESAFHK	95.0%	53.6	21.4	2	1	0	2	1,351.63
Protein disulfide-isomerase A4	PDIA4_HUMAN	PDIA4	72,916	100.00%	36	43	225	52.70%	DFPEYTFAIADEEDYAGEVK	95.0%	92.1	18.9	2	0	0	2	2,309.01
									DKDPPIPVAK	95.0%	36.7	18.6	2	1	0	2	1,079.61
									DLGLSESGEDVNAAILDESGK	95.0%	99.3	22.2	1	0	0	2	2,119.00
									DLGLSESGEDVNAAILDESGKK	95.0%	130.0	21.5	3	4	0	2	2,247.10
									EFVTAFK	95.0%	36.0	21.5	2	0	0	2	841.45
									EKYGIVDYMIEQSGPPSK	95.0%	32.0	21.7	0	3	0	2	2,056.99
									EVSQPDWTPPPEVTLVLTK	95.0%	44.7	20.3	2	0	0	2	2,136.12
									FAMEPEEFDSDTLR	95.0%	81.2	17.2	12	0	0	2	1,702.73
									FDVSGYPTIK	95.0%	75.7	22.6	24	0	0	2	1,126.58
									FEGGDRDLEHLSK	95.0%	41.9	21.8	2	0	0	2	1,502.72
									FHHTFSTEIAK	95.0%	36.2	22.4	0	2	0	2	1,317.66
									FIEEHATK	95.0%	58.7	22.8	2	0	0	2	974.49
									GESDPAYQQYQDAANNLR	95.0%	116.0	19.1	6	0	0	2	2,039.91
									GESDPAYQQYQDAANNLREDYK	95.0%	41.6	18.2	0	2	0	2	2,575.13
									GQAVDYEGSR	95.0%	58.9	20.5	4	0	0	2	1,081.49
									IDATSASVLASR	95.0%	117.0	22.6	22	0	0	2	1,190.64
									KGQAVDYEGSR	95.0%	70.4	23.2	3	2	0	2	1,209.59
									MDATANDVPSDR	95.0%	79.6	17.8	25	0	0	2	1,307.55
									MDATANDVPSDRYK	95.0%	40.2	18.8	1	0	0	2	1,598.71
									QLEPVYNSLAK	95.0%	57.5	22.3	3	0	0	2	1,261.68
									QVQEFLKDGDDVIIIGVFK	95.0%	34.3	18.0	0	2	0	2	2,163.17
									RFDVSGYPTIK	95.0%	40.6	21.7	6	2	0	1	1,282.68
									RSPPIPLAK	95.0%	31.5	9.0	1	0	0	2	978.61
									SHMMDVQGSTQDSAIK	95.0%	70.0	20.8	2	1	0	2	1,766.77
									SHMMDVQGSTQDSAIKDFVLK	95.0%	46.2	21.4	0	7	0	2	2,369.11
									TFDSIVMDPK	95.0%	70.8	21.8	8	0	0	2	1,168.56
									TFDSIVMDPKK	95.0%	45.9	21.9	2	0	0	2	1,296.65
									TQEEIVAK	95.0%	49.6	23.6	3	0	0	2	917.49
									VDATAETDLAK	95.0%	72.3	23.3	10	0	0	2	1,133.57
									VDATAETDLAKR	95.0%	68.9	24.2	4	1	0	2	1,289.67
									VEGFPTIYFAPSGDK	95.0%	59.1	22.1	4	0	0	2	1,627.80
									VEGFPTIYFAPSGDKK	95.0%	59.0	22.1	2	2	0	2	1,755.90
									VSQGQLVVMQPEK	95.0%	88.3	22.4	16	0	0	2	1,458.76
									YALPLVGHR	95.0%	55.7	18.3	10	0	0	2	1,025.59
									YGIVDYMIEQSGPPSK	95.0%	92.5	21.9	10	0	0	2	1,799.85

									YKVEGFPTIYFAPSGDK	95.0%	53.5	22.2	2	0	0	2	1,918.96
Metalloproteinase inhibitor 1	TIMP1_HUMAN	TIMP1	23,153	100.00%	11	15	1371	76.30%	ACTCVPPHPQTAFCNSDLVIR	95.0%	28.0	21.5	0	1	0	2	2,386.11
									EPGLCTWQSLR	95.0%	39.7	22.8	7	0	0	2	1,346.65
									FVGTPEVNQTTLYQR	95.0%	84.4	22.0	9	0	0	2	1,752.89
									FVYTPAMESVCGYFHR	95.0%	100.0	20.1	173	16	0	2	1,963.88
									GFQALGDAADIR	95.0%	109.0	23.5	702	2	0	2	1,233.62
									HLACLPR	95.0%	39.0	22.9	28	0	0	2	866.47
									LQDGLLHITTCSFVAPWNSLSLAQR	95.0%	61.4	19.5	0	52	0	2	2,827.46
									LQSGTHCLWTDQLLQGSEK	95.0%	96.1	21.9	14	33	0	2	2,201.07
									MYKGFQALGDAADIR	95.0%	70.0	22.4	8	12	0	2	1,671.82
									SEEFLIAGK	95.0%	65.6	21.7	309	0	0	2	993.53
									TYTVGCEECTVFPCLSIPCK	95.0%	75.6	18.5	5	0	0	2	2,421.06
Complement factor I	CFAI_HUMAN	CFI	65,702	100.00%	3	3	7	5.83%	AQLGDLPWQVAIK	95.0%	37.2	18.7	1	0	0	2	1,438.81
									HGNTDSEGIVEVK	95.0%	61.4	22.6	1	0	0	2	1,384.67
									IVIEYVDR	95.0%	36.5	20.8	5	0	0	2	1,006.56
Signal recognition particle 14 kDa protein	SRP14_HUMAN	SRP14	14,553	99.50%	2	2	4	19.90%	KGTVEGFEPADNK	95.0%	47.6	22.9	2	0	0	2	1,391.68
									VLLESEQFLTELTR	95.0%	94.9	20.6	2	0	0	1	1,677.91
Lysyl-tRNA synthetase	SYK_HUMAN	KARS	68,032	100.00%	13	14	60	22.80%	FELFVMK	95.0%	31.4	21.8	2	0	0	2	929.48
									HITGSYK	95.0%	36.8	24.0	4	0	0	2	805.42
									LIFYDLR	95.0%	43.1	19.0	7	0	0	2	939.53
									LPETNLFETEETR	95.0%	53.1	22.6	1	0	0	2	1,578.77
									LPETNLFETEETRK	95.0%	68.0	23.0	5	6	0	2	1,706.86
									LQVMANSR	95.0%	34.6	23.3	2	0	0	2	934.48
									MLVVGGIDR	94.8%	30.3	23.5	1	0	0	2	975.53
									RGDIIGVQGNPGK	95.0%	53.6	20.3	3	0	0	2	1,310.72
									VAMFLTDSNNIK	95.0%	50.7	22.9	6	0	0	2	1,368.68
									VNGEDPYPHK	95.0%	26.1	21.2	0	1	0	2	1,155.54
									VTYHPDGPEGQAYDVDFTPPFR	95.0%	52.2	20.6	0	5	0	2	2,508.15
									YLDLILNDFVR	95.0%	59.6	20.8	14	0	0	2	1,380.75
									YSHLQPGDHLTDITLK	95.0%	33.6	21.7	0	3	0	2	1,837.95
Thioredoxin-dependent peroxide reductase, mitochondrial	PRDX3_HUMAN	PRDX3	27,675	100.00%	4	5	15	21.10%	DYGVLLEGSGLALR	95.0%	73.1	21.4	2	0	0	2	1,462.79
									GLFIIDPNGVIK	95.0%	54.0	18.3	7	0	0	2	1,285.75
									HLSVNDLPVGR	95.0%	49.0	21.9	2	0	0	2	1,206.66
									NGGLGHMNIALLSDLTK	95.0%	50.8	22.0	2	2	0	2	1,769.92
Ezrin	EZRI_HUMAN	EZR	69,397	100.00%	27	32	207	56.70%	AKEAQDDLVK	95.0%	49.6	24.0	2	0	0	2	1,116.59
									AKEELER	95.0%	34.7	24.3	2	0	0	2	874.46
									AKFYPEDVAEELIQDITQK	95.0%	53.6	21.9	2	8	0	2	2,237.13
									ALQLEEER	95.0%	48.7	22.7	16	0	0	2	987.51
									APDFVFYAPR	95.0%	83.9	21.7	44	0	0	2	1,182.59
									AQEEAERLEADR	95.0%	41.9	21.3	10	3	0	2	1,416.67
									DDKLTPK	94.9%	30.4	22.1	1	0	0	2	816.45
									DNAMLEYLK	95.0%	44.1	22.4	7	0	0	2	1,112.53
									EGILSDEIYCPPETAVLLGSYAVQAK	95.0%	50.1	21.3	0	2	0	2	2,823.41
									EKEELMLR	95.0%	33.9	24.5	1	0	0	2	1,063.55
									ELSEQIQR	95.0%	44.4	22.9	7	0	0	2	1,002.52
									ENPLQFK	95.0%	32.2	24.5	1	0	0	2	875.46
									FVIKPIDK	95.0%	36.6	17.2	7	0	0	2	959.59
									FYPEDVAEELIQDITQK	95.0%	97.8	21.9	31	21	0	2	2,038.00
									GMLKDNAMLEYLK	95.0%	43.1	22.8	3	0	0	2	1,557.77
									IALLEEAR	95.0%	43.9	18.6	9	0	0	2	914.53
									IAQDLEMYGINYFEIK	95.0%	86.2	22.1	23	0	0	2	1,962.95

									IGFPWSEIR	95.0%	62.2	23.4	19	0	0	2	1,104.58
									ILQLCMGNHELYMR	95.0%	46.8	21.7	1	0	0	2	1,809.85
									KAPDFVFYAPR	95.0%	54.9	22.3	12	1	0	2	1,310.69
									KENPLQFK	95.0%	31.6	22.1	1	0	0	2	1,003.56
									KPDTIEVQQMK	95.0%	48.7	23.4	4	0	0	2	1,332.68
									KVSAQEVR	95.0%	37.7	22.5	1	0	0	2	916.52
									LFFLQVK	95.0%	41.5	18.9	14	0	0	2	894.55
									NISFNDKK	94.8%	30.3	22.7	1	0	0	2	965.51
									PKPINVR	95.0%	31.9	12.6	1	0	0	1	823.52
									QAVDQIK	95.0%	51.1	21.7	4	0	0	2	801.45
									QLFDQVVK	95.0%	56.5	21.4	9	0	0	2	976.55
									QLLTLSSELSQAR	95.0%	113.0	21.6	6	0	0	2	1,445.80
									QQLETEK	95.0%	33.5	23.5	2	0	0	2	875.45
									QRIDEFEAL	95.0%	36.0	23.7	3	0	0	2	1,120.56
									RKPDTIEVQQMK	95.0%	53.8	22.3	13	38	0	2	1,488.78
									SGYLSSER	95.0%	48.3	20.6	1	0	0	2	898.43
									SQEQLAAELAEYTAK	95.0%	128.0	22.5	24	2	0	2	1,651.82
									THNDIIHNENMR	95.0%	66.5	21.4	4	0	1	2	1,509.69
									VSAQEVR	95.0%	46.2	24.3	7	0	0	2	788.43
									VTTMDAELEFAIQPNTTGK	95.0%	114.0	22.4	28	6	0	2	2,082.01
Latent-transforming growth factor beta-binding protein 3	LTBP3_HUMAN	LTBP3	139,337	99.50%	2	2	7	2.99%	GYTQDNNIVNYGIPAHR	95.0%	49.3	22.9	0	5	0	2	1,931.94
									TGALSTGALPPLAPEGDSVASK	95.0%	65.8	21.2	2	0	0	2	2,039.07
Annexin A1	ANXA1_HUMAN	ANXA1	38,698	100.00%	20	26	248	60.70%	AAYLQETGKPLDETLK	95.0%	81.2	21.4	2	0	0	2	1,776.94
									AAYLQETGKPLDETLKK	95.0%	59.6	18.3	4	9	0	2	1,905.03
									ALTGHLEEVVLALLK	95.0%	84.3	10.4	19	0	0	2	1,605.96
									ALYEAGER	95.0%	53.1	22.5	8	0	0	2	908.45
									CATSKPAFFAEK	95.0%	62.0	21.0	2	0	0	2	1,356.66
									CLTAIVK	95.0%	32.9	22.0	1	0	0	2	804.47
									DITSDTSGDFR	95.0%	63.1	19.3	9	0	0	2	1,213.53
									DLAKDITSDTSGDFR	95.0%	59.5	22.3	3	0	0	2	1,640.78
									GDRSEDFGVNEDLADSDAR	95.0%	73.4	17.8	3	5	0	2	2,067.89
									GGPGSAVSPYPTFNPSSDVAALHK	95.0%	105.0	22.1	2	17	0	2	2,356.16
									GLGTDEDTLIEILASR	95.0%	118.0	21.5	50	8	0	2	1,702.89
									GTDVNVFNTILTTR	95.0%	93.4	22.0	22	0	0	2	1,550.82
									GVDEATIIDILTK	95.0%	96.8	20.9	29	0	0	2	1,387.77
									GVDEATIIDILTKR	95.0%	81.3	19.1	9	7	0	2	1,543.87
									KGTDVNVFNTILTTR	95.0%	108.0	20.2	4	2	0	2	1,678.91
									MYGISLCQAILDETKGDYEK	95.0%	50.1	20.5	0	2	0	2	2,350.10
									NALLSLAK	95.0%	46.9	20.9	1	0	0	2	829.51
									SEDFGVNEDLADSDAR	95.0%	131.0	18.0	6	0	0	2	1,739.74
									SEIDMNDIK	95.0%	43.6	21.2	5	0	0	2	1,080.49
									TPAQFDADELR	95.0%	76.5	22.1	19	0	0	2	1,262.60
Bifunctional purine biosynthesis protein PURH	PUR9_HUMAN	ATIC	64,599	100.00%	14	15	27	32.10%	ALFEEVPELLTEAEKK	95.0%	42.4	21.0	2	0	0	2	1,845.99
									APGQLALFSVSDK	95.0%	61.1	22.3	2	0	0	1	1,332.72
									DVSELTGFPEMLGGR	95.0%	42.6	21.8	2	0	0	2	1,623.77
									EVSDGIIAPGYEEEALTILSK	95.0%	54.6	21.0	2	0	0	2	2,234.14
									GVSQMPLR	95.0%	33.2	24.1	1	0	0	2	903.47
									HVSPAGAAVGIPLSEDEAK	95.0%	103.0	22.3	2	2	0	2	1,847.95
									LDFNLIR	95.0%	42.4	20.6	2	0	0	2	890.51
									LTEVSISSDAFFPFR	95.0%	64.1	22.0	1	0	0	2	1,715.86
									NIPEDNADMAR	95.0%	31.8	18.1	1	0	0	2	1,261.55

									NLTALGLNLVASGGTAK	95.0%	116.0	18.2	2	0	0	2	1,599.91
									SLFSNVVTK	95.0%	39.3	19.5	2	0	0	2	994.56
									TLFGLHLSQK	95.0%	36.8	20.9	2	0	0	2	1,143.65
									VVACNLYPFVK	95.0%	31.9	22.0	2	0	0	2	1,309.70
									YGMNPHQTPAQLYTLQPK	95.0%	50.1	22.4	0	2	0	2	2,103.03
Reticulocalbin-1	RCN1_HUMAN	RCN1	38,873	100.00%	4	5	12	13.30%	AADLNGDLTATR	95.0%	80.3	22.9	1	0	0	2	1,217.61
									IDNDGDGFVTTEELK	95.0%	118.0	22.5	3	0	0	2	1,652.77
									IVDRIDNDGDGFVTTEELK	95.0%	83.4	21.7	1	3	0	2	2,136.05
									TFDQLTPDESKER	95.0%	59.1	22.7	4	0	0	2	1,565.75
Alpha-aminoadipic semialdehyde dehydrogenase	AL7A1_HUMAN	ALDH7A1	58,470	100.00%	11	11	40	24.90%	DLPLAQGIK	95.0%	44.9	14.8	2	0	0	2	954.56
									GAPTTSLISVAVTK	95.0%	101.0	19.2	4	0	0	2	1,344.77
									GEVITTYCPANNEPIAR	95.0%	81.5	22.4	2	0	0	2	1,904.92
									IQVLGSLVSLEMGK	95.0%	118.0	19.9	9	0	0	2	1,489.83
									MIGGPILPSER	95.0%	51.7	20.6	6	0	0	2	1,169.64
									QASVADYEETVK	95.0%	38.1	22.4	1	0	0	2	1,339.64
									QASVADYEETVKK	95.0%	45.8	22.9	2	0	0	2	1,467.73
									QAVSMFLGAVEEAKK	95.0%	42.1	21.8	0	2	0	2	1,623.84
									QGLSSSIFTK	95.0%	36.5	21.2	4	0	0	2	1,067.57
									VGNPWDPNVLYGPLHTK	95.0%	45.8	21.8	0	2	0	2	1,906.98
									VNLLSFTGSTQVGK	95.0%	96.7	20.4	6	0	0	2	1,450.79
26S protease regulatory subunit 7	PRS7_HUMAN	PSMC2	48,618	100.00%	5	5	9	16.20%	ALDEGDIALLK	95.0%	61.7	22.3	2	0	0	2	1,157.64
									FDDGAGGDNEVQR	95.0%	65.0	16.7	2	0	0	2	1,379.58
									FVVDLSDQVAPTDIEEGMR	95.0%	65.3	22.4	1	0	0	2	2,137.01
									GVLLFGPPGTGK	95.0%	34.5	22.1	1	0	0	2	1,142.66
									TMLELINQLDGFDPR	95.0%	55.2	22.4	3	0	0	2	1,777.88
Poly [ADP-ribose] polymerase 1	PARP1_HUMAN	PARP1	113,070	100.00%	17	20	66	23.50%	AMVEYEIDLQK	95.0%	68.7	21.8	2	0	0	2	1,354.66
									GGAAVDPDSGLEHSAHVLEK	95.0%	80.9	22.1	2	0	0	2	1,988.97
									GGSDDSSKDPIDVNYEK	95.0%	68.7	19.6	4	1	0	2	1,825.81
									HPDVEVDGFSELR	95.0%	55.1	22.5	2	0	0	2	1,499.71
									IAPPEAPVTGYMFGK	95.0%	36.5	23.3	3	0	0	2	1,593.80
									KFYPLEIDYGQDEEAVK	95.0%	28.9	21.8	0	1	0	2	2,043.99
									KPPLLNNADSVQAK	95.0%	46.7	19.0	2	0	0	2	1,494.83
									KTAEAGGVTGK	95.0%	43.0	23.2	2	0	0	2	1,018.55
									MAIMVQSPMFDGK	95.0%	60.6	19.5	2	0	0	2	1,502.67
									NREELGFRPEYSASQLK	95.0%	34.7	21.7	0	1	2	2	2,024.02
									QQVPSGESAILDR	95.0%	40.3	21.9	1	0	0	2	1,399.72
									SKLPKPVQDLIK	95.0%	35.6	10.4	0	2	0	2	1,365.85
									TLGDFAAEYAK	95.0%	57.9	22.6	4	0	0	2	1,185.58
									TTNFAGILSQGLR	95.0%	96.5	21.6	24	0	0	2	1,377.75
									VFSATLGLVDIVK	95.0%	82.8	12.8	3	0	0	2	1,361.80
									VVDRDSEEAEIIR	95.0%	51.9	22.8	2	3	0	2	1,530.78
									VVSEDFLQDVSASTK	95.0%	98.1	22.5	3	0	0	2	1,624.81
Multimerin-2	MMRN2_HUMAN	MMRN2	104,390	100.00%	7	7	36	10.60%	QLDGSSLQALQNAVDAVSLAVDAHK	95.0%	97.1	19.9	0	7	0	2	2,550.32
									QLHSAFAALLEDALR	95.0%	37.7	20.6	0	5	0	2	1,654.89
									REEELQYTLEDMR	95.0%	30.4	20.6	0	1	0	2	1,727.79
									SISELQADVDTK	95.0%	68.9	23.3	8	0	0	2	1,305.65
									SLSGTAFGGFLMFK	95.0%	53.1	23.0	3	0	0	2	1,478.74
									SQVQALDDEVGALK	95.0%	101.0	22.0	11	0	0	2	1,472.76
									VQDSAVAR	95.0%	35.3	23.0	1	0	0	2	845.45
DNA replication complex GINS protein SLD5	SLD5_HUMAN	GINS4	26,029	99.50%	2	2	3	14.80%	TIAPLVASGAVQLI	95.0%	48.8	14.3	2	0	0	2	1,352.82
									TRPEGEPSSLSPEELAFAR	95.0%	38.0	22.1	0	1	0	2	2,073.03

Nucleoside diphosphate kinase B	NDKB_HUMAN	NME2	17,280	100.00%	11	14	69	73.70%	ASEEHLK	95.0%	46.7	20.3	3	0	0	2	813.41
									DRPFFPGLVK	95.0%	40.8	20.6	3	0	0	2	1,175.66
									EISLWFKPEELVDYK	95.0%	37.1	21.0	2	0	0	2	1,895.98
									GDFCIQVGR	95.0%	65.9	22.8	4	0	0	2	1,051.50
									GLVGEIIK	95.0%	49.3	16.7	4	0	0	2	828.52
									GLVGEIIKR	95.0%	53.2	16.7	5	0	0	2	984.62
									NIIHGSDSVK	95.0%	41.6	21.7	5	0	0	2	1,069.56
									SAEKEISLWFKPEELVDYK	95.0%	26.9	20.7	0	0	2	2	2,311.19
									TFIAIKPDGVQR	95.0%	36.9	19.8	1	1	0	2	1,344.76
									VMLGETNPADSKPGTIR	95.0%	88.0	22.1	6	16	0	2	1,785.92
									YMNSGPVVAMVWEGLNVVK	95.0%	114.0	22.5	13	4	0	2	2,109.05
Activator of 90 kDa heat shock protein ATPase homolog 1	AHSA1_HUMAN	AHSA1	38,256	100.00%	5	5	18	21.90%	EAMGIYISTLK	95.0%	66.4	21.0	2	0	0	2	1,241.65
									ETFLTSPEELYR	95.0%	47.5	22.2	2	0	0	2	1,484.73
									FHMVDGNVSGEFTDLVPEK	95.0%	29.9	22.1	0	1	0	2	2,136.99
									LDGEASINNR	95.0%	45.0	22.4	1	0	0	2	1,088.53
									VFTTQELVQAFTHAPATLEADR	95.0%	77.3	21.2	0	12	0	2	2,445.24
60S ribosomal protein L8	RL8_HUMAN	RPL8	28,007	99.50%	2	3	5	6.61%	ASGNYATVISHNPETK	95.0%	67.7	22.3	2	1	0	2	1,688.82
									ASGNYATVISHNPETKK	95.0%	32.2	22.4	0	2	0	2	1,816.92
Vascular endothelial growth factor C	VEGFC_HUMAN	VEGFC	46,865	100.00%	4	5	12	10.70%	DLEEQLR	95.0%	31.7	22.5	1	0	0	2	902.46
									EQANLNSR	95.0%	42.4	21.0	1	0	0	2	931.46
									FAAAHYNTEILK	95.0%	69.2	22.3	6	2	0	2	1,377.72
									SVSSVDELMTVLYPEYWK	95.0%	49.7	21.8	2	0	0	2	2,162.04
Protein S100-A11	S10AB_HUMAN	S100A11	11,723	100.00%	5	7	56	47.60%	CIESLIAVFQK	95.0%	75.0	21.2	4	0	0	2	1,307.70
									DGYNYTLSK	95.0%	52.0	20.6	7	0	0	2	1,060.50
									NQKDPGVLDR	95.0%	42.7	21.6	1	5	0	2	1,141.60
									TEFLSFMNTELAAFTK	95.0%	102.0	22.6	35	0	0	2	1,865.90
									YAGKDGYNYTLSK	95.0%	81.7	22.0	2	2	0	2	1,479.71
Carbonyl reductase [NADPH] 1	CBR1_HUMAN	CBR1	30,357	100.00%	11	13	54	57.80%	DVCTELLPLIKPQGR	95.0%	36.0	19.7	0	1	0	2	1,738.95
									EYGGLDVLVNNAGIAFK	95.0%	85.0	21.6	10	0	0	2	1,779.93
									FHQLDIDDLQSIR	95.0%	62.9	21.8	2	7	0	2	1,599.81
									GIGLAIVR	95.0%	45.1	14.8	2	0	0	2	798.52
									GQAAVQQLQAEGLSPR	95.0%	118.0	21.8	7	3	0	2	1,652.87
									IGVTVLSR	95.0%	65.1	20.9	4	0	0	2	844.53
									LFSGDVVLTAR	95.0%	72.6	20.8	4	0	0	2	1,177.66
									SETITEEELVGLMNK	95.0%	58.8	22.7	6	0	0	2	1,708.83
									SPEEGAETPVYLALLPPDAEGPHGQFVSEK	95.0%	81.9	20.3	0	6	0	2	3,164.54
									VADPTPFHIQAEVTMK	95.0%	35.1	22.1	1	0	0	2	1,799.90
									VVNVSSIMSVR	95.0%	94.0	22.4	1	0	0	2	1,206.65
Lysosomal Pro-X carboxypeptidase	PCP_HUMAN	PRCP	55,783	100.00%	10	16	65	23.40%	ALGSLHLPTNPTSLPAVAK	95.0%	27.8	15.3	0	1	0	2	1,887.07
									AMLVFAEHR	95.0%	45.6	23.6	5	2	0	2	1,089.55
									DITDTLVAVTISEGAHHLDLR	95.0%	64.6	19.8	0	11	4	2	2,276.19
									HLNFLTSEQALADFAELIK	95.0%	111.0	20.8	3	1	0	2	2,160.13
									IVTTDFR	95.0%	44.8	20.8	3	0	0	2	851.46
									NALDPMSVLLAR	95.0%	56.0	22.7	8	0	0	2	1,315.70
									TKNALDPMSVLLAR	95.0%	43.8	20.8	2	1	0	2	1,544.85
									VDHFGFNTVK	95.0%	37.5	23.3	4	4	0	2	1,163.59
									YYGESLPFGDNSFK	95.0%	67.1	20.9	8	0	0	2	1,623.73
									YYGESLPFGDNSFKDSR	95.0%	57.3	19.3	4	4	0	2	1,981.89
Heat shock protein HSP 90-beta	HS90B_HUMAN	HSP90AB1	83,249	100.00%	42	58	2014	49.00%	ADHGEPIGR	95.0%	45.9	22.0	13	0	0	2	951.47
									ADLINNLGTIAK	95.0%	72.6	20.9	155	0	0	2	1,242.71
									ALLFIPR	95.0%	46.5	17.1	28	0	0	2	829.53

									APFDLFENK	95.0%	48.8	22.7	10	0	0	2	1,080.54
									APFDLFENKK	95.0%	50.3	22.5	1	3	0	2	1,208.63
									DNSTMGYMMAK	95.0%	68.9	9.5	23	0	0	2	1,296.49
									EDQTEYLEER	95.0%	54.4	18.8	36	0	0	2	1,311.57
									EGLELPEDEEEK	95.0%	49.5	19.9	8	0	0	2	1,416.64
									EGLELPEDEEEKK	95.0%	47.7	21.2	7	0	0	2	1,544.73
									EKYIDQEELNK	95.0%	54.8	23.7	11	1	0	2	1,408.70
									ELISNASDALDK	95.0%	97.2	23.1	53	0	0	2	1,275.64
									ELISNASDALDKIR	95.0%	109.0	21.4	34	13	0	2	1,544.83
									ELKIDIIPNPQER	95.0%	54.9	18.5	5	0	0	2	1,564.87
									EMLQQSK	95.0%	46.5	23.4	7	0	0	2	879.42
									EQVANSAFVER	95.0%	70.0	23.5	66	0	0	2	1,249.62
									GVVDSEDLPLNISR	95.0%	117.0	22.4	116	0	0	2	1,513.79
									HFSVEGQLEFR	95.0%	85.9	23.7	175	150	0	2	1,348.67
									HLEINPDHPIVETLR	95.0%	75.5	20.9	4	34	2	2	1,782.95
									HNDDEQYAWESSAGGSFTVR	95.0%	28.5	17.6	0	3	0	2	2,255.96
									HSQFIGYPITLYLEK	95.0%	76.6	21.8	42	54	0	2	1,808.96
									IDIIPNPQER	95.0%	51.3	20.9	32	0	0	2	1,194.65
									IEDVGSDEEDDSGK	95.0%	93.9	15.1	4	0	0	2	1,494.61
									IEDVGSDEEDDSGKDK	95.0%	103.0	17.3	6	10	0	2	1,737.73
									IRYESLTDPSK	95.0%	47.4	22.2	3	6	0	2	1,308.68
									KHLEINPDHPIVETLR	95.0%	38.7	18.4	0	2	10	2	1,911.05
									KHSQFIGYPITLYLEK	95.0%	32.8	18.6	0	2	0	2	1,937.05
									LGIHEDSTNR	95.0%	54.6	22.7	26	47	0	2	1,141.56
									LGIHEDSTNRR	95.0%	35.7	21.8	0	5	0	2	1,297.66
									LSELLR	95.0%	42.5	23.4	35	0	0	2	730.45
									NPDDITQEEYGEFYK	95.0%	98.5	17.9	65	0	0	2	1,847.80
									RAPFDLFENK	95.0%	54.7	22.6	32	0	0	2	1,236.64
									SIYYITGESK	95.0%	63.8	23.7	46	0	0	2	1,160.58
									SLTNDWEDHLAVK	95.0%	83.3	22.0	4	2	0	2	1,527.74
									SLVSVTK	95.0%	38.2	18.8	1	0	0	2	733.45
									TLTLVDTGIGMTK	95.0%	117.0	22.3	121	0	0	2	1,365.73
									VFIMDSCDELIPEYLNFIR	95.0%	40.8	21.3	1	0	0	2	2,390.14
									VILHLK	95.0%	39.2	7.8	6	0	0	2	722.49
									VILHLKEDQTEYLEER	95.0%	75.7	21.9	52	216	1	2	2,015.05
									YESLTDPSK	95.0%	49.8	22.6	19	0	0	2	1,039.49
									YESLTDPSKLDSGK	95.0%	77.4	22.6	40	2	0	2	1,539.75
									YHTSQSGDEMTSLSEYVSR	95.0%	97.5	17.9	5	87	0	2	2,192.94
									YIDQEELNK	95.0%	60.7	23.2	72	0	0	2	1,151.56
60 kDa heat shock protein, mitochondrial	CH60_HUMAN	HSPD1	61,038	100.00%	27	37	136	59.50%	ALMLQGVDLLADAVAVTMGPK	95.0%	105.0	20.9	10	26	0	2	2,145.13
									APGFGDNRK	95.0%	34.6	23.3	1	0	0	2	961.49
									CEFQDAYVLLSEK	95.0%	88.1	21.8	2	0	0	2	1,601.75
									CIPALDSLTPANEDQK	95.0%	94.3	22.0	2	0	0	2	1,771.85
									DMAIATGGAVFGEEGLTLNLEDVQPHDLGK	95.0%	42.7	21.4	0	2	0	2	3,113.51
									GVMLAVDAVIAELK	95.0%	73.6	19.5	1	0	0	2	1,444.81
									GVMLAVDAVIAELKK	95.0%	85.9	15.7	8	2	0	2	1,572.90
									GYISPYFINTSK	95.0%	61.3	23.5	3	0	0	2	1,389.71
									IGIEIIK	95.0%	47.1	10.8	2	0	0	2	785.51
									IGIEIIKR	95.0%	38.8	13.0	2	0	0	2	941.62
									IMQSSSEVGYDAMAGDFVNMVEK	95.0%	79.1	16.6	2	2	0	2	2,556.09
									ISSIQSIVPALEIANAHR	95.0%	111.0	17.8	2	14	0	2	1,919.07

									KISSIQSIVPALEIANAHR	95.0%	78.2	14.9	2	2	2	2	2,047.17
									KPLVIIAEDVDGEALSTLVLNR	95.0%	31.8	13.4	0	2	0	2	2,365.33
									LSDGVAVLK	95.0%	47.8	20.6	3	0	0	2	901.54
									LVQDVANNTNEEAGDGTTTATVLAR	95.0%	141.0	21.8	1	1	0	2	2,560.25
									NAGVEGSLIVEK	95.0%	66.8	23.5	3	0	0	2	1,215.66
									QSKPVTTPEEIAQVATISANGDKEIGNIISDAMK	95.0%	48.0	19.7	0	2	0	2	3,571.82
									TLKIPAMTIAK	95.0%	34.2	16.7	0	1	0	2	1,202.72
									TLNDELEIIEGMK	95.0%	75.0	22.9	3	0	0	2	1,520.75
									TLNDELEIIEGMKFDR	95.0%	68.3	22.1	2	1	0	2	1,938.95
									VGEVIVTK	95.0%	42.3	20.7	4	0	0	2	844.51
									VGEVIVTKDDAMLLK	95.0%	53.4	20.8	2	2	0	2	1,646.90
									VGGTSDVEVNEK	95.0%	74.7	22.4	3	0	0	2	1,233.60
									VGGTSDVEVNEKK	95.0%	70.9	23.5	4	5	0	2	1,361.69
									VGLQVVAVK	95.0%	77.6	13.0	2	0	0	2	912.59
									VTDALNATR	95.0%	76.3	24.0	8	0	0	2	960.51
High mobility group protein B1	HMGB1_HUMAN	HMGB1	24,877	100.00%	9	13	45	40.90%	FKDPNAPK	95.0%	39.5	23.5	5	0	0	2	916.49
									GEHPGLSIGDVAK	95.0%	51.9	22.8	3	2	0	2	1,279.66
									GEHPGLSIGDVAKK	95.0%	39.0	21.0	0	2	0	2	1,407.76
									HPDASVNFSEFSK	95.0%	45.0	21.3	4	0	0	2	1,464.68
									HPDASVNFSEFSKK	95.0%	49.1	23.0	0	2	0	2	1,592.77
									IKGEHPGLSIGDVAK	95.0%	57.0	19.9	4	12	3	2	1,520.84
									KHPDASVNFSEFSK	95.0%	50.9	23.0	2	3	0	2	1,592.77
									LGEMWNNTAADDKQPYEK	95.0%	55.0	20.4	0	2	0	2	2,125.95
									MSSYAFFVQTCR	95.0%	92.3	19.7	7	0	0	2	1,512.66
									TYIPPKGETK	95.0%	31.9	22.6	1	0	0	2	1,133.62
									YEKDIAAYR	95.0%	55.9	22.3	10	9	0	2	1,128.57
Kinesin-like protein KIF23	KIF23_HUMAN	KIF23	110,041	100.00%	3	3	4	5.00%	ALLQEFDNAVLSK	95.0%	38.3	21.5	2	0	0	2	1,447.78
									ELFDVVANPLVNDLIHGK	95.0%	68.8	18.8	1	0	0	2	1,993.08
									LVQAPLDADGDNVLQEK	95.0%	80.4	21.8	1	0	0	2	1,824.93
Prostaglandin reductase 1	PTGR1_HUMAN	PTGR1	35,853	100.00%	7	9	31	28.60%	EYIIEGFENMPAAFMGMLK	95.0%	92.7	20.9	4	0	0	2	2,239.01
									HFVGYPTNSDFELK	95.0%	61.5	22.0	2	0	0	2	1,653.79
									LGFDVVFNYK	95.0%	53.7	22.4	3	0	0	2	1,201.63
									LKEGDTMMGQQVAK	95.0%	55.3	22.2	4	5	0	2	1,567.75
									MEAFVVYR	95.0%	52.0	20.1	4	0	0	2	1,030.50
									TGPLPPGPPPEIVIYQELR	95.0%	80.4	17.6	5	2	0	2	2,073.14
									VVGAVGSDEK	95.0%	47.6	23.7	2	0	0	2	960.50
Out at first protein homolog	OAF_HUMAN	OAF	30,670	100.00%	3	3	9	10.60%	ALILGELEK	95.0%	47.5	17.4	7	0	0	2	985.59
									ASEQAELPR	95.0%	40.6	20.6	1	0	0	2	1,000.51
									SYSFDFYVPQR	95.0%	33.0	21.9	1	0	0	2	1,408.65
Small nuclear ribonucleoprotein G-like protein	RUXGL_HUMAN	---	8,526	99.90%	2	2	5	17.10%	GNSIIMLEALER	95.0%	38.1	23.3	2	0	0	2	1,345.72
									GNSIIMLEALERV	95.0%	87.5	22.2	3	0	0	2	1,460.78
Ras GTPase-activating-like protein IQGAP1	IQGA1_HUMAN	IQGAP1	189,241	100.00%	42	47	202	35.20%	ALQSPALGLR	95.0%	40.4	16.9	4	0	0	2	1,025.61
									ATFYGEQVDYYK	95.0%	75.4	20.0	4	0	0	2	1,483.67
									DSLHEKFPDAGEDELLK	95.0%	28.2	22.6	0	2	0	2	1,942.94
									EEIQSSISGVTAAYNR	95.0%	136.0	22.0	4	0	0	2	1,724.85
									EQLSDMMMINK	95.0%	36.5	17.5	2	0	0	2	1,387.59
									FALGIFAINEAVESGDVGK	95.0%	116.0	21.3	4	0	0	2	1,937.00
									FDVPGDENAEMDAR	95.0%	74.2	14.1	3	0	0	2	1,581.65
									FLSAIVSSVDK	95.0%	77.3	20.5	2	0	0	2	1,165.65
									FLSAIVSSVDKIPYGMR	95.0%	42.0	20.7	1	0	0	2	1,899.01
									FMDSVIFTLYNYASNQR	95.0%	114.0	21.7	2	0	0	2	2,084.98

									FQPGETLTEILETPATSEQEAEHQR	95.0%	82.6	20.3	0	10	0	2	2,841.35
									GVLLEIEDLQVNQFK	95.0%	75.3	20.4	4	0	0	2	1,744.95
									IFYPETTDIYDR	95.0%	37.9	22.5	1	0	0	2	1,532.73
									IFYPETTDIYDRK	95.0%	65.3	22.8	2	0	0	2	1,660.82
									IIGNLLYYR	95.0%	62.0	20.1	8	0	0	2	1,124.65
									ILAIGLINEALDEGDAQK	95.0%	137.0	20.0	30	17	0	2	1,883.01
									ITLQDVVSHSK	95.0%	55.4	21.4	2	1	0	2	1,226.67
									LAAVALINAAIQK	95.0%	84.7	12.6	12	0	0	2	1,295.81
									LAVGDNNSK	94.9%	30.4	23.3	1	0	0	2	917.47
									LEGVLAEVAQHYQDTLIR	95.0%	93.8	19.3	6	3	0	2	2,055.09
									LGLAPQIQDLYGK	95.0%	69.8	20.3	6	0	0	2	1,415.79
									LGNFFSPK	95.0%	47.9	21.8	6	0	0	2	909.48
									LIFQMPQNK	95.0%	30.5	22.9	1	0	0	2	1,134.60
									LPYDVTPEQALAHEEVK	95.0%	53.2	21.3	2	5	0	2	1,938.98
									LQQTYAALNSK	95.0%	59.7	22.4	3	0	0	2	1,236.66
									LTAEEMDER	95.0%	76.5	18.8	4	0	0	2	1,109.48
									LTELGTVDPK	95.0%	58.8	27.2	2	0	0	2	1,072.59
									MFLGDNAHLSIINEYLSQSYQK	95.0%	49.7	21.8	0	4	0	2	2,587.25
									MREEVITLIR	95.0%	28.4	21.4	0	1	0	2	1,275.71
									NKEQLSDMMMINK	95.0%	40.7	18.4	0	3	0	2	1,629.73
									NPNAMLVNLEEPLASTYQDILYQAK	95.0%	55.1	20.6	1	4	0	2	2,851.42
									NVIFEISPTEEVGDFEVK	95.0%	89.4	22.4	5	0	0	2	2,052.02
									QSGQTDPLQKEELQSGVDAANSAAQQYQR	95.0%	82.2	20.7	0	1	0	2	3,147.49
									SNQQLENDLNLMDIK	95.0%	85.2	22.5	6	0	0	2	1,790.86
									TLINAEDPPMVVVR	95.0%	70.1	21.6	3	0	0	2	1,569.83
									TLQALQIPAAK	95.0%	76.7	15.3	6	0	0	2	1,153.69
									TVLELMNPEAQLPQVYPFAADLYQK	95.0%	42.4	20.3	0	2	0	2	2,894.47
									VDFTEEEINNMK	95.0%	68.0	19.2	2	0	0	2	1,484.66
									VDFTEEEINNMKTELEK	95.0%	29.2	21.6	0	1	0	2	2,084.97
									VDQIQEIVTGNPTVIK	95.0%	90.2	19.0	6	0	0	2	1,753.97
									YMNPAIVAPDAFDIIDLSAGGQLTTDQR	95.0%	35.0	21.6	0	1	0	2	3,008.47
									YQELINDIAR	95.0%	44.6	23.7	2	0	0	2	1,234.64
Glutathione S-transferase P	GSTP1_HUMAN	GSTP1	23,339	100.00%	8	11	134	52.40%	AFLASPEYVNLPINGNGKQ	95.0%	75.8	21.6	3	0	0	2	2,032.05
									ALPGQLKPFETLLSQNQGGK	95.0%	79.5	18.1	10	22	0	2	2,126.16
									DQQEAALVDMVNDGVEDLR	95.0%	115.0	20.6	14	3	0	2	2,132.98
									FQDGDLTLYQSNTILR	95.0%	148.0	21.5	44	1	0	2	1,883.95
									PPYTVVYFPVR	95.0%	68.5	21.2	27	0	0	1	1,337.73
									TLGLYGKDQQEAALVDMVNDGVEDLR	95.0%	49.8	21.3	0	4	0	2	2,865.39
									YISLIYTNYEAGK	95.0%	70.0	22.5	2	0	0	2	1,534.78
									YISLIYTNYEAGKDDYVK	95.0%	102.0	22.1	4	0	0	2	2,155.06
Seryl-tRNA synthetase, cytoplasmic	SYSC_HUMAN	SARS	58,761	100.00%	5	6	10	14.40%	FKDPGLVDQLVK	95.0%	33.7	19.7	2	1	0	2	1,358.77
									IEQFVYSSPHDNK	95.0%	50.4	22.0	2	0	0	2	1,563.74
									KEPVGDDESVPENVLSFDDLTADALANLK	95.0%	59.1	21.0	0	1	0	2	3,101.52
									LLIDEAILK	95.0%	37.5	12.3	3	0	0	2	1,027.64
									VDKGGDPALIR	95.0%	31.0	19.9	0	1	0	2	1,140.64
Fibronectin	FINC_HUMAN	FN1	262,581	99.50%	2	2	3	1.13%	LGVRPSQGGEAPR	95.0%	28.0	21.0	0	2	0	2	1,323.71
									SYTITGLQPGTDYK	95.0%	49.3	21.8	1	0	0	2	1,543.76
14-3-3 protein zeta/delta	1433Z_HUMAN	YWHAZ	27,728	100.00%	22	28	288	68.60%	AKLAEQAER	95.0%	33.6	22.6	4	0	0	2	1,015.55
									DICNDVLSLLEK	95.0%	88.7	23.9	26	0	0	2	1,418.72
									DSTLIMQLLR	95.0%	78.2	22.5	32	0	0	2	1,205.66
									EKIETELR	95.0%	46.8	23.0	6	0	0	2	1,017.56

									EMQPTHPIR	95.0%	54.3	21.9	17	0	0	2	1,124.55
									FLIPNASQAESK	95.0%	57.6	23.1	7	0	0	2	1,304.69
									GIVDQSQQAYQEAFEISK	95.0%	106.0	21.8	47	1	0	2	2,040.99
									GIVDQSQQAYQEAFEISKK	95.0%	79.4	22.3	3	2	0	2	2,169.08
									IETELR	95.0%	31.7	25.0	1	0	0	2	760.42
									KEMQPTHPIR	95.0%	50.7	22.5	2	4	0	2	1,252.65
									KGIVDQSQQAYQEAFEISK	95.0%	37.2	22.4	0	3	0	2	2,169.08
									LAEQAER	95.0%	56.5	21.6	15	0	0	2	816.42
									LAEQAERYDDMAACMK	95.0%	48.8	16.1	0	3	0	2	1,933.81
									MKGDYYR	95.0%	35.8	19.2	2	0	0	2	932.43
									NLLSVAYK	95.0%	51.2	19.1	26	0	0	2	907.53
									NLLSVAYKNVVGAR	95.0%	85.3	17.9	2	4	0	2	1,503.86
									SVTEQGAELSNEER	95.0%	119.0	20.6	24	0	0	2	1,548.71
									TAFDEAIAELDTLSEESYK	95.0%	101.0	20.6	11	3	0	2	2,131.99
									VVSSIEQKTEGAEK	95.0%	76.5	22.4	3	0	0	2	1,504.79
									YDDMAACMK	95.0%	54.1	4.8	3	0	0	2	1,136.41
									YLAEVAAGDDK	95.0%	61.4	23.1	4	0	0	2	1,151.56
									YLAEVAAGDDKK	95.0%	71.4	22.7	23	10	0	2	1,279.65
Agrin	AGRIN_HUMAN	AGRN	214,820	100.00%	49	62	830	27.70%	AAAVSSGFDGAIQLVSLGGR	95.0%	123.0	21.1	73	6	0	2	1,875.99
									AIVDVHFDPTTAFR	95.0%	60.8	22.3	18	19	0	2	1,588.81
									ALEPQGLLLYNGNAR	95.0%	45.1	21.0	1	3	0	2	1,628.88
									ALQSNHFELSLR	95.0%	65.1	22.1	11	9	0	2	1,414.74
									AYGTGFVGCLR	95.0%	61.5	21.2	7	0	0	2	1,200.58
									AYHTLR	95.0%	35.9	24.2	1	0	0	2	760.41
									DFLALALLDGR	95.0%	72.1	21.3	10	0	0	2	1,203.67
									DQCPEPCR	95.0%	33.5	12.0	1	0	0	2	1,061.41
									EPLYVGGAPDFSK	95.0%	64.8	22.7	7	0	0	2	1,379.68
									ESLLDGGNK	95.0%	50.5	23.5	2	0	0	2	932.47
									FDTGSGPAVLTSAVPVEPGQWHR	95.0%	76.8	20.8	2	15	0	2	2,408.20
									FGALCEAETGR	95.0%	79.1	20.8	11	0	0	2	1,210.55
									FHCQCPPGR	95.0%	40.5	17.9	2	0	0	2	1,158.49
									FNAVCLSR	95.0%	60.9	23.2	11	0	0	2	966.48
									GAPEGTVCGSDGADYPGECQLLR	95.0%	56.0	16.1	1	0	0	2	2,409.05
									GDFVSLALR	95.0%	62.3	21.4	15	0	0	2	977.54
									GIVTDGR	95.0%	33.1	24.0	2	0	0	2	717.39
									GKDFLALALLDGR	95.0%	112.0	19.9	42	0	0	2	1,388.79
									GLHTFAR	95.0%	35.5	20.7	3	0	0	2	801.44
									GLYVAAQGACR	95.0%	65.0	23.1	6	0	0	2	1,165.58
									GMLCGFGAVCEPNAEGPGR	95.0%	90.4	17.4	3	0	0	2	1,994.85
									LALEFR	95.0%	38.3	21.6	7	0	0	2	748.44
									LELGIGPGAATR	95.0%	79.8	20.2	44	0	0	2	1,154.65
									LLDVNNQR	95.0%	57.3	20.9	20	0	0	2	971.53
									LRDLGPGK	95.0%	54.4	18.1	4	0	0	2	855.51
									MALEVVFLAR	95.0%	88.4	20.4	124	0	0	2	1,164.65
									QIQVSR	95.0%	35.7	24.0	1	0	0	2	730.42
									QLLTPEHVLR	95.0%	45.9	17.8	3	4	0	2	1,205.70
									QVDVTSFAGHPCTR	95.0%	89.5	20.9	2	3	0	2	1,574.74
									RLEFR	95.0%	32.6	19.0	4	0	0	2	720.42
									SADGLTASCLCPATCR	95.0%	72.9	18.9	2	0	0	2	1,739.75
									SAGDVDTLAFDGR	95.0%	105.0	21.5	55	0	0	2	1,323.62
									SELFGETAR	95.0%	43.7	22.9	7	0	0	2	1,009.50

									SFLAFPTLR	95.0%	60.4	20.3	61	0	0	2	1,051.59
									SIESTLDDLFR	95.0%	77.9	22.0	52	0	0	2	1,295.65
									SREPVTLGAWTR	95.0%	54.8	22.3	2	1	0	2	1,372.73
									STVPVNTNR	95.0%	45.5	22.4	7	0	0	2	987.52
									TDGKGDFVSLALR	95.0%	83.4	22.2	11	1	0	2	1,378.73
									TEATQGLVLWSGK	95.0%	93.7	22.3	8	0	0	2	1,389.74
									TFVEYLNAVTESEK	95.0%	116.0	22.4	53	0	0	2	1,629.80
									TFVGAGLR	95.0%	55.5	21.6	8	0	0	2	820.47
									TPSLDAEGSNCPATK	95.0%	71.5	20.6	2	0	0	2	1,547.70
									VCGSDGVTYGNECQLK	95.0%	94.6	18.5	2	0	0	2	1,786.77
									VFQGVLELEGVEGQELFYTPEMADPK	95.0%	52.7	21.2	0	4	0	2	2,941.42
									VLGAPVPAFEGR	95.0%	63.6	21.0	25	0	0	2	1,212.67
									VLGESPVPHTVLNLK	95.0%	69.1	16.3	6	1	0	2	1,602.92
									VLGESPVPHTVLNLKEPLYVGGAPDFSK	95.0%	62.5	18.1	0	10	8	2	2,963.59
									VTCDGAYRPVCAQDGR	95.0%	46.3	19.9	2	2	0	2	1,824.81
									VVISGFGDPLICDNQVSTGDTR	95.0%	83.5	21.1	2	1	0	2	2,350.14
Beta-1,4-galactosyltransferase 4	B4GT4_HUMAN	B4GALT4	40,024	100.00%	4	6	10	14.80%	DKGNEVNAER	95.0%	40.3	20.8	1	0	0	2	1,131.54
									ISRPLPEVGK	95.0%	31.6	16.3	1	1	0	2	1,095.65
									LIFKPDLTLEEVQAENPK	95.0%	68.2	19.5	2	3	0	2	2,084.13
									YSGYFGGVTALSR	95.0%	89.2	22.4	2	0	0	2	1,377.68
Kinesin-1 heavy chain	KINH_HUMAN	KIF5B	109,668	100.00%	4	4	9	4.67%	ISFLENNLEQLTK	95.0%	57.8	22.1	4	0	0	2	1,548.83
									LFVQDLATR	95.0%	38.2	23.2	1	0	0	2	1,062.59
									LYLVDLAGSEK	95.0%	50.0	22.0	2	0	0	2	1,207.66
									SLTEYLQNVEQK	95.0%	55.6	23.0	2	0	0	2	1,451.74
Rab GDP dissociation inhibitor beta	GDIB_HUMAN	GDI2	50,648	100.00%	27	36	147	60.00%	DLGTESQIFISR	95.0%	83.4	22.6	10	0	0	2	1,365.70
									EIRPALELLEPIEQK	95.0%	37.6	16.3	0	4	0	2	1,778.01
									EPEKEIRPALELLEPIEQK	95.0%	34.3	17.9	0	1	1	2	2,261.24
									FDLGQDVIDFTGHALALYR	95.0%	73.5	21.7	1	3	0	2	2,151.09
									FKIPGSPPESMGR	95.0%	51.0	23.4	2	1	0	2	1,418.71
									FLMANGQLVK	95.0%	42.1	22.7	2	0	0	2	1,136.61
									FLVYVANFDEKDPR	95.0%	46.1	22.6	1	2	0	2	1,712.87
									FVSISDLLVPK	95.0%	67.9	16.5	7	0	0	2	1,217.71
									IKLYSESLAR	95.0%	38.7	18.7	0	4	0	2	1,179.67
									IPGSPPESMGR	95.0%	45.2	21.1	2	0	0	2	1,143.55
									IYKVPSTEAEALASSLMGLFEK	95.0%	70.3	20.5	2	5	0	2	2,400.24
									KFDLGQDVIDFTGHALALYR	95.0%	45.7	20.9	0	1	0	2	2,279.18
									LSAIYGGTYMLNKPIEEIIVQNGK	95.0%	62.0	19.1	0	5	0	2	2,667.41
									LYSESLAR	95.0%	54.3	20.3	6	0	0	2	938.49
									MLLYTEVTR	95.0%	55.7	21.8	8	0	0	2	1,141.59
									MTGSEFDFEEMK	95.0%	88.8	9.0	2	0	0	2	1,482.58
									MTGSEFDFEEMKR	95.0%	70.5	16.3	4	0	0	2	1,638.68
									NPYYGGESASITPLEDLYK	95.0%	104.0	22.1	2	0	0	2	2,117.01
									NPYYGGESASITPLEDLYKR	95.0%	104.0	21.6	2	3	0	2	2,273.11
									SEGEIAR	95.0%	36.7	23.9	1	0	0	2	761.38
									SPYLYPLYGLGELPQGFAR	95.0%	115.0	21.6	17	1	0	2	2,141.11
									TFEGIDPK	95.0%	49.5	23.4	4	0	0	2	906.46
									TYDATTHFETTCDDIK	95.0%	31.3	17.8	0	1	0	2	1,917.82
									VICILSHPIK	95.0%	54.9	17.7	2	2	0	2	1,179.69
									VPSTEAEALASSLMGLFEK	95.0%	95.9	22.8	22	11	0	2	1,996.00
									VPSTEAEALASSLMGLFEKR	95.0%	26.0	21.8	0	1	0	2	2,152.10
									VTEGSFVYK	95.0%	67.7	21.7	4	0	0	2	1,029.53

Annexin A6	ANXA6_HUMAN	ANXA6	75,860	100.00%	9	10	28	18.70%	ALIEILATR	95.0%	59.4	18.3	4	0	0	2	999.62
									DAFVAIVQSVK	95.0%	95.1	20.8	7	0	0	2	1,176.66
									EDAQVAAEILEIADTPSGDK	95.0%	45.8	22.1	1	0	0	2	2,072.00
									GFGSDKEAILDIITSR	95.0%	50.7	20.8	0	4	0	2	1,721.91
									GLGTDEDTIIDIITHR	95.0%	107.0	22.1	3	3	0	2	1,768.91
									GSIHDFPGFDPNQDAEALYTAMK	95.0%	52.9	20.0	0	2	0	2	2,540.14
									SELDMLDIR	95.0%	34.7	23.4	1	0	0	2	1,107.54
									SLHQAIEGDTSGDFLK	95.0%	48.2	22.7	2	0	0	2	1,717.84
									TNAEIR	95.0%	30.4	24.5	1	0	0	2	703.37
Glycogenin-1	GLYG_HUMAN	GYG1	39,366	99.50%	2	2	4	6.86%	GALVLGSSLK	95.0%	42.7	18.1	2	0	0	2	944.58
									LVVLATPQVSDSMR	95.0%	67.4	22.2	2	0	0	2	1,531.82
Prefoldin subunit 3	PFD3_HUMAN	VBP1	22,641	99.50%	2	2	3	13.70%	KLDEQYQK	95.0%	36.8	21.9	1	0	0	2	1,051.54
									LHLGIPEAVFVEDVDSFMK	95.0%	47.9	21.8	0	2	0	2	2,162.08
DNA-(apurinic or apyrimidinic site) lyase	APEX1_HUMAN	APEX1	35,538	100.00%	8	9	46	32.40%	EAAGEGPALYEDPPDQK	95.0%	80.4	20.7	3	0	0	2	1,786.81
									EGYSGVGLLSR	95.0%	77.0	22.9	7	0	0	2	1,137.59
									GAVAEDGDELRTEPEAK	95.0%	74.0	21.6	2	2	0	2	1,786.85
									LDYFLLSHSLLPALCDSK	95.0%	36.0	21.0	1	0	0	2	2,092.08
									NAGFTPQER	95.0%	42.4	22.5	4	0	0	2	1,019.49
									NDKEAAGEGPALYEDPPDQK	95.0%	36.4	20.9	0	1	0	2	2,143.98
									QGFGELLQAVPLADSFR	95.0%	83.0	21.7	23	0	0	2	1,847.97
									TSPSGKPATLK	95.0%	37.7	22.7	3	0	0	2	1,086.62
High mobility group protein B3	HMGB3_HUMAN	HMGB3	22,963	100.00%	2	2	5	13.50%	KNPEVPVNFAEFSK	94.6%	30.1	22.5	1	0	0	2	1,605.83
									STNPGISIGDVAK	95.0%	75.7	23.0	4	0	0	2	1,258.66
Aminoacyl tRNA synthase complex-interacting multifunctional protein 2	AIMP2_HUMAN	AIMP2	35,331	99.50%	2	2	3	9.06%	DIVINANPASPPLSLLVLHR	95.0%	35.0	12.6	0	1	0	2	2,139.23
									FLFSLFGQK	95.0%	35.8	22.6	2	0	0	2	1,086.60
Semaphorin-4D	SEM4D_HUMAN	SEMA4D	96,133	99.50%	2	2	2	3.48%	DHPLMDDSVTPIDNRPR	95.0%	27.2	21.3	0	1	0	2	1,993.94
									YMQSTTVEQSHTK	95.0%	42.8	21.0	1	0	0	2	1,555.71
Aldehyde dehydrogenase family 16 member A1	A16A1_HUMAN	ALDH16A1	85,109	100.00%	4	4	9	6.48%	DSSGNLHGYVAEGGAK	95.0%	62.8	20.9	2	0	0	2	1,561.72
									EALLVANGTPR	95.0%	70.7	20.5	2	0	0	2	1,140.64
									GLDGAVDMGAR	95.0%	53.4	20.7	2	0	0	2	1,077.50
									VQAQGHTLQVAGLR	95.0%	47.0	18.5	0	3	0	2	1,477.82
Adenosine deaminase	ADA_HUMAN	ADA	40,747	100.00%	3	3	6	12.90%	ELLDLLYK	95.0%	42.2	20.0	1	0	0	2	1,006.58
									GIALPANTAEGLLNVIGMDKPLTLPDFLAK	95.0%	48.8	16.9	0	2	0	2	3,108.70
									IAYEFVEMK	95.0%	41.5	21.8	3	0	0	2	1,145.56
Cofilin-1	COF1_HUMAN	CFL1	18,485	100.00%	10	12	100	70.50%	AVLFCLSEDKK	95.0%	38.1	22.6	2	0	0	2	1,309.68
									CTLAEK	95.0%	35.4	23.4	1	0	0	2	721.36
									EILVGDVGQTVDDPYATFVK	95.0%	112.0	21.6	2	0	0	2	2,166.10
									HELQANCYEEVKDR	95.0%	37.6	21.0	0	1	0	2	1,790.81
									KEDLVFIFWAPESAPLK	95.0%	68.3	19.5	2	2	0	2	1,990.07
									KSSTPEEVK	95.0%	39.3	23.4	1	0	0	2	1,004.53
									LGGSAVISLEGKPL	95.0%	93.9	18.0	62	0	0	2	1,340.78
									MIYASSK	95.0%	47.3	20.3	2	0	0	2	815.40
									NIILEEGKEILVGDVGQTVDDPYATFVK	95.0%	88.3	19.1	0	14	1	2	3,062.59
									YALYDATYETK	95.0%	60.5	22.0	10	0	0	2	1,337.63
WD repeat-containing protein 5	WDR5_HUMAN	WDR5	36,571	100.00%	3	3	8	12.60%	TLIDDDNPPVSFVK	95.0%	70.2	23.2	2	0	0	2	1,559.80
									TLPAHSDPVSAVHFNR	95.0%	42.2	22.6	0	4	0	2	1,747.89
									YILAATLDNTLK	95.0%	47.1	20.0	2	0	0	2	1,335.75
Eukaryotic translation initiation factor 3 subunit K	EIF3K_HUMAN	EIF3K	25,042	100.00%	3	3	6	18.80%	ENAYDLEANLAVLK	95.0%	60.2	23.0	2	0	0	2	1,562.81
									WLLAEMLGDLSDSQLK	95.0%	76.4	22.4	2	0	0	2	1,834.93
									YNPENLATLER	95.0%	63.1	22.7	2	0	0	2	1,319.66
60S acidic ribosomal protein P2	RLA2_HUMAN	RPLP2	11,647	100.00%	5	6	11	84.30%	ILDSVGIEADDDRLNK	95.0%	71.1	22.4	2	0	0	2	1,772.90

									KEESEESDDDMGFGLFD	95.0%	62.8	10.0	2	0	0	2	1,965.76
									LASVPAGGAVAVSAAPGSAAPAAGSAPAAAEEK	95.0%	29.6	20.4	0	2	0	2	2,774.43
									NIEDVIAQGIGK	95.0%	91.2	22.2	2	0	0	2	1,256.69
									YVASYLLAALGGNSSPSAK	95.0%	73.6	21.2	2	1	0	2	1,868.98
UV excision repair protein RAD23 homolog B	RD23B_HUMAN	RAD23B	43,153	100.00%	5	6	11	19.10%	IDIDPEETVK	94.7%	30.2	22.6	1	0	0	2	1,158.59
									NFVVVMVTKPK	95.0%	46.2	18.3	2	0	0	2	1,277.73
									NQPQFQQMR	94.8%	30.3	22.0	1	0	0	2	1,192.55
									QEKPAEKPAETPVATSPTATDSTSGDSSR	95.0%	69.7	20.9	0	1	0	2	2,945.40
									QIIQQNPSLLPALLQQIGR	95.0%	90.8	11.1	4	2	0	2	2,130.24
Serglycin	SRGN_HUMAN	SRGN	17,635	100.00%	9	10	383	41.80%	CNPDSNSANCLEEK	95.0%	89.7	13.2	8	0	0	2	1,637.65
									CNPDSNSANCLEEKGPMFELLPGESNK	95.0%	44.6	15.3	0	7	0	2	3,053.33
									GPMFELLPGESNK	95.0%	87.8	22.2	121	0	0	2	1,434.69
									GPMFELLPGESNKIPR	95.0%	79.0	22.0	29	75	0	2	1,800.93
									IQDLNR	95.0%	44.7	22.6	24	0	0	2	758.42
									LRTDLFPK	95.0%	39.7	20.3	15	0	0	2	989.58
									NLPSDSQDLGQHGLEEDFML	95.0%	79.3	19.3	82	0	0	2	2,261.00
									TDLFPK	95.0%	32.3	21.6	17	0	0	2	720.39
									TRIQDLNR	95.0%	41.7	21.5	5	0	0	2	1,015.57
Alpha-soluble NSF attachment protein	SNAA_HUMAN	NAPA	33,216	100.00%	5	5	12	24.40%	AIDIYEQVGTNAMDSPLLK	95.0%	111.0	22.5	2	0	0	2	2,094.04
									EAEAMALLAEAER	95.0%	82.2	22.5	3	0	0	2	1,419.68
									NSQSFFSGLFGGSSK	95.0%	82.3	22.2	2	0	0	2	1,549.73
									VAGYAALLEQYQK	95.0%	76.7	22.0	2	0	0	2	1,453.77
									YEELFPAFSDSR	95.0%	42.8	20.8	3	0	0	2	1,460.67
Cleavage stimulation factor subunit 3	CSTF3_HUMAN	CSTF3	82,906	100.00%	4	4	7	8.37%	FLAFESNIGDLASILK	95.0%	75.4	19.7	2	0	0	2	1,737.94
									GVEAVGSYAENQR	95.0%	83.2	22.0	2	0	0	2	1,379.66
									LAAIIPDPVVAPSIVPVLK	95.0%	41.0	3.0	2	0	0	2	1,912.19
									LFSDEAANIYER	95.0%	32.1	21.2	1	0	0	2	1,427.68
26S proteasome non-ATPase regulatory subunit 5	PSMD5_HUMAN	PSMD5	56,179	100.00%	8	9	31	21.20%	ILTLSQIGR	95.0%	64.3	17.4	3	0	0	2	1,000.62
									IVENSDAVTEILNNAELLK	95.0%	114.0	19.9	10	0	0	2	2,085.11
									LEAPLEELR	95.0%	40.8	21.1	1	0	0	2	1,069.59
									LGPLFSLLNENHR	95.0%	60.8	20.8	4	1	0	2	1,509.82
									LMFNSPGFVEYVVDR	95.0%	98.1	22.1	5	0	0	2	1,788.86
									QQAAELR	95.0%	31.5	21.5	1	0	0	2	815.44
									TIAEIFGNPNYLR	95.0%	64.1	22.5	3	0	0	2	1,507.79
									TYLSEGPYYVKPVSTTAVEGAE	95.0%	55.3	21.6	3	0	0	2	2,361.15
General vesicular transport factor p115	USO1_HUMAN	USO1	107,880	100.00%	7	7	12	9.56%	DSEQVAELKQELATLK	95.0%	40.0	21.6	1	0	0	2	1,801.96
									EQDLQLEELR	95.0%	47.2	22.1	2	0	0	2	1,272.64
									GVMGGQSAGPQHTEAETIQK	95.0%	32.5	21.0	0	1	0	2	2,041.96
									IVAFENAFER	95.0%	58.6	22.1	2	0	0	2	1,195.61
									LMDLLADSR	95.0%	55.0	23.7	2	0	0	2	1,049.53
									NDGVLLLQALTR	95.0%	77.7	18.2	2	0	0	2	1,312.76
									QSEDLGSQFTEIFIK	95.0%	88.1	21.6	2	0	0	2	1,741.87
Alpha-N-acetylglucosaminidase	ANAG_HUMAN	NAGLU	82,150	100.00%	15	21	118	33.20%	AAAVSEAEADFYEQNSR	95.0%	111.0	20.0	5	0	0	2	1,857.83
									AGGVLAYELLPALDEVLASDSR	95.0%	99.7	19.6	14	5	0	2	2,259.19
									ALAAKPGLDTYSLGGGGAAR	95.0%	72.0	21.6	4	7	0	2	1,845.98
									FLLGSWLEQAR	95.0%	57.4	22.1	4	0	0	2	1,319.71
									KDPVPDLAAWVTSFAAR	95.0%	53.4	21.2	0	2	0	2	1,843.97
									LFLEALVDSVAQGIPFQQHQFDK	95.0%	69.8	19.4	0	3	0	2	2,630.36
									LLGPGPAADFSVSVER	95.0%	102.0	21.9	9	0	0	2	1,614.85
									LLLTSAPSLATSPAFR	95.0%	87.8	17.9	17	2	0	2	1,644.93
									LLVLDLFAESQPVYTR	95.0%	80.2	19.3	6	0	0	2	1,864.02

									LPRPLPAVPGELTEATPNR	95.0%	55.0	17.2	2	6	0	2	2,028.12
									NVFQLEQAFVLSK	95.0%	94.2	20.8	8	0	0	2	1,522.83
									QAVQELVSLYYEEAR	95.0%	119.0	22.1	7	4	0	2	1,797.90
									QLAGLVANYYTPR	95.0%	88.8	22.1	4	0	0	2	1,465.78
									SFGMTPVLPAFAGHVPEAVTR	95.0%	63.4	21.4	3	2	0	2	2,200.12
									YDLLDLTR	95.0%	53.0	21.4	4	0	0	2	1,008.54
COP9 signalosome complex subunit 7b	CSN7B_HUMAN	COPS7B	29,605	100.00%	3	3	5	16.70%	ATASSSAQEMEQQLAER	95.0%	91.0	20.6	2	0	0	2	1,852.83
									ESLPELSTAQQNK	95.0%	65.9	21.9	1	0	0	2	1,444.73
									TQQQVEAEVTNIKK	95.0%	53.2	21.4	0	2	0	2	1,615.87
Lysozyme C	LYSC_HUMAN	LYZ	16,519	99.50%	2	2	10	14.20%	ATNYNAGDR	95.0%	36.9	19.3	4	0	0	2	981.44
									STDYGIFQINSR	95.0%	89.6	21.6	6	0	0	2	1,400.68
Stress-induced-phosphoprotein 1	STIP1_HUMAN	STIP1	62,624	100.00%	18	23	67	34.60%	AAALEAMK	95.0%	33.2	24.4	1	0	0	2	820.42
									ALSVGNIDDALQCYSEAIK	95.0%	121.0	22.0	3	0	0	2	2,067.01
									AMADPEVQQIMSDPAMR	95.0%	74.5	18.1	3	1	0	2	1,937.84
									DPQALSEHLK	95.0%	42.6	22.9	2	0	0	2	1,137.59
									DPQALSEHLKNPVIAQK	95.0%	42.9	19.2	0	3	0	2	1,888.03
									EAADGYQR	95.0%	39.2	20.9	2	0	0	2	909.41
									EGLQNMEAR	95.0%	51.1	20.0	8	0	0	2	1,063.48
									ELIEQLR	95.0%	31.4	20.0	2	0	0	2	900.52
									ETKPEPMEEDLPENKK	95.0%	34.4	22.1	2	2	0	2	1,929.91
									IGNSYFKEEK	95.0%	54.6	22.4	6	1	0	2	1,214.61
									ILKEQER	95.0%	41.3	23.4	1	0	0	2	915.53
									LAYINPDLALEEK	95.0%	61.9	22.4	3	0	0	2	1,488.80
									LDPHNHVLYSNR	95.0%	42.3	22.4	2	2	0	2	1,464.73
									LMDVGLIAIR	95.0%	70.8	20.9	9	0	0	2	1,116.65
									NPVIAQK	95.0%	37.2	17.2	2	0	0	2	769.46
									TLLSDPTYR	95.0%	30.5	22.8	1	0	0	2	1,065.56
									TYEEGLKHEANNPQLK	95.0%	62.0	22.0	2	8	0	2	1,870.93
									YKDAIHFYNK	95.0%	38.8	21.5	1	0	0	2	1,298.65
ADP-sugar pyrophosphatase	NUDT5_HUMAN	NUDT5	24,310	100.00%	6	7	21	30.10%	EQTADGVAVIPVLQR	95.0%	89.5	19.5	6	1	0	2	1,595.88
									HANAKPFEVPFLK	95.0%	31.9	20.5	0	5	0	2	1,497.82
									KEQTADGVAVIPVLQR	95.0%	80.5	16.9	1	0	0	2	1,723.97
									LDALVAEEHLTVDAR	95.0%	38.5	21.5	0	3	0	2	1,651.87
									QYIISEELISEGK	95.0%	59.9	22.6	3	0	0	2	1,508.79
									VYSYALALK	95.0%	47.7	19.3	2	0	0	2	1,027.58
KDEL motif-containing protein 2	KDEL2_HUMAN	KDELC2	58,556	100.00%	3	3	3	6.11%	DFASFPSINLQQMLK	95.0%	48.4	22.6	1	0	0	2	1,754.88
									EGQLMAR	95.0%	33.2	23.6	1	0	0	2	820.40
									LMGFFDFFK	95.0%	31.2	21.8	1	0	0	2	1,167.56
40S ribosomal protein S6	RS6_HUMAN	RPS6	28,664	100.00%	7	8	20	26.50%	DIPGLTDTTVPR	95.0%	48.3	21.2	5	0	0	2	1,284.68
									ISGGNDKQGFPMK	95.0%	36.4	21.9	2	0	0	2	1,394.67
									LFNLSKEDDVR	95.0%	49.6	22.8	2	2	0	2	1,335.69
									LIEVDDER	95.0%	52.2	23.5	4	0	0	2	988.50
									NKEEAAEYAK	95.0%	36.5	22.2	1	0	0	2	1,152.55
									RLSSLR	95.0%	39.3	19.7	2	0	0	2	731.45
									TFYEKR	95.0%	30.5	20.9	2	0	0	2	843.44
Lamin-A/C	LMNA_HUMAN	LMNA	74,123	100.00%	32	42	317	42.00%	AAYEAELGDAR	95.0%	86.6	22.3	12	0	0	2	1,165.55
									AAYEAELGDARK	95.0%	61.2	23.2	8	0	0	2	1,293.64
									AQHEDQVEQYKK	95.0%	53.9	21.8	14	16	4	2	1,502.72
									EAALSTALSEKR	95.0%	69.3	22.0	9	2	0	2	1,275.69
									EGDLIAAQAR	95.0%	83.4	22.1	6	0	0	2	1,043.55
									IDSLSAQLSQLQK	95.0%	90.1	20.7	7	0	0	2	1,430.79

									IRIDSLSAQLSQLQK	95.0%	96.0	17.2	5	6	0	2	1,699.97
									ITESEEVVSR	95.0%	90.3	23.4	16	0	0	2	1,148.58
									KLESTESR	95.0%	41.6	22.1	1	0	0	2	949.50
									KLLEGEEER	95.0%	38.7	23.0	1	0	0	2	1,102.57
									LADALQELR	95.0%	83.5	21.4	12	0	0	2	1,028.57
									LALDMEIHAYR	95.0%	48.7	22.3	2	0	0	2	1,347.67
									LAVYIDR	95.0%	42.0	20.6	8	0	0	2	849.48
									LEAALGEAK	95.0%	38.8	21.7	4	0	0	2	901.50
									LEAALGEAKK	95.0%	40.0	21.5	4	0	0	2	1,029.59
									LKDLEALLNSK	95.0%	59.0	19.0	12	0	0	2	1,243.73
									LLEGEEER	95.0%	34.4	22.0	2	0	0	2	974.48
									LQEKEDLQELNDR	95.0%	84.3	22.0	14	15	0	2	1,629.81
									LQTMKEELDFQK	95.0%	49.2	22.7	11	5	0	2	1,525.76
									MQQQLDEYQELLDIK	95.0%	113.0	22.4	16	0	0	2	1,909.92
									NIYSEELR	95.0%	46.2	22.0	1	0	0	2	1,023.51
									NIYSEELRETK	95.0%	37.0	22.2	1	0	0	2	1,381.70
									NSNLVGAAHEELQQSR	95.0%	103.0	22.1	13	8	0	2	1,752.86
									QLQDEMLR	95.0%	41.0	22.7	3	0	0	2	1,048.51
									SGAQASSTPLSPTR	95.0%	84.2	22.9	17	0	0	2	1,359.69
									SLETENAGLR	95.0%	73.4	23.6	19	0	0	2	1,089.55
									TALINSTGEEVAMR	95.0%	89.3	22.9	14	0	0	2	1,507.74
									TALINSTGEEVAMRK	95.0%	56.9	22.7	2	0	0	2	1,635.84
									TLEGELHDLR	95.0%	65.0	22.4	10	1	0	2	1,182.61
									TLEGELHDLRGQVAK	95.0%	49.3	19.5	2	3	0	2	1,665.89
									VAVEEVDEEGK	95.0%	50.4	22.0	1	0	0	2	1,203.57
									VAVEEVDEEGKFVR	95.0%	72.9	22.6	8	2	0	2	1,605.81
Arginase-1	ARGI1_HUMAN	ARG1	34,718	100.00%	4	4	17	16.10%	GGVEEGPTVLR	95.0%	50.7	21.1	1	0	0	2	1,113.59
									TGLLSGLDIMEVNPSLGK	95.0%	97.5	21.1	9	0	0	2	1,859.98
									TIGIIGAPFSK	95.0%	41.9	19.5	2	0	0	2	1,103.65
									VMEETLSYLLGR	95.0%	52.6	22.0	5	0	0	2	1,426.73
60S ribosomal protein L6	RL6_HUMAN	RPL6	32,711	100.00%	3	5	51	15.60%	ASITPGTILIILTGR	95.0%	92.1	9.0	33	5	0	2	1,525.93
									QLASGLLLVTGPLVLNR	95.0%	92.0	6.0	10	2	0	2	1,764.08
									VLATVTKPVGGDK	95.0%	32.2	18.3	1	0	0	2	1,284.75
N-acetylglucosamine-6-sulfatase	GNS_HUMAN	GNS	62,066	100.00%	8	8	47	18.30%	AFQNVFAPR	95.0%	53.6	23.0	9	0	0	2	1,049.55
									IQEPNTFPAILR	95.0%	65.3	20.0	17	0	0	2	1,398.77
									QLYEFDIK	95.0%	37.0	20.9	1	0	0	2	1,055.54
									RPNVVLLLTDDQDEVLGGMTPLKK	95.0%	56.0	17.9	0	2	0	2	2,667.44
									SDVLVEYQGEGR	95.0%	55.4	21.6	2	0	0	2	1,351.65
									TPGVFDPGYR	95.0%	45.5	22.4	4	0	0	2	1,108.54
									TQMDGMSLLPILR	95.0%	53.5	22.8	8	0	0	2	1,506.77
									WQTLLSVDDLVEK	95.0%	66.6	22.9	4	0	0	2	1,545.82
Vacuolar protein sorting-associated protein 29	VPS29_HUMAN	VPS29	20,488	99.50%	2	3	5	12.60%	GDFDENLNYPEQK	95.0%	52.0	19.3	2	0	0	2	1,568.69
									TLAGDVHIVR	95.0%	53.5	19.0	2	1	0	2	1,080.62
COP9 signalosome complex subunit 4	CSN4_HUMAN	COPS4	46,252	100.00%	8	8	16	25.40%	AIQLSGAEQLEALK	95.0%	64.4	21.1	2	0	0	2	1,470.82
									ATTADGSSILDR	95.0%	81.9	23.2	2	0	0	2	1,206.60
									AVIEHNLLSASK	95.0%	60.6	20.8	2	0	0	2	1,281.72
									IASQMITEGR	95.0%	52.2	24.1	3	0	0	2	1,121.56
									LYLEDDDPVQAEAYINR	95.0%	95.9	22.3	2	0	0	2	2,023.96
									MLATLFKDER	95.0%	45.3	22.0	1	0	0	2	1,239.64
									NAAQVLVGIPLETGQK	95.0%	113.0	18.2	2	0	0	2	1,637.92
									VISFEEQVASIR	95.0%	80.4	22.0	2	0	0	2	1,377.74

Clathrin heavy chain 1	CLH1_HUMAN	CLTC	191,601	100.00%	39	49	282	31.00%	AFMTADLPNELIELLEK	95.0%	85.3	22.5	21	0	0	2	1,963.01
									AHMGMFTELAILYSK	95.0%	49.9	20.3	1	2	0	2	1,743.85
									ALEHFTDLYDIK	95.0%	36.5	22.3	1	0	0	2	1,464.74
									ALEHFTDLYDIKR	95.0%	56.9	22.5	0	3	0	2	1,620.84
									DAMQYASESK	95.0%	72.7	16.5	2	0	0	2	1,145.48
									EAIDSYIK	95.0%	31.3	22.3	1	0	0	2	938.48
									FNALFAQGNYSEAAK	95.0%	67.6	22.3	2	0	0	2	1,630.79
									GQFSTDELVAEVEK	95.0%	110.0	23.1	5	0	0	2	1,551.75
									GQFSTDELVAEVEKR	95.0%	86.5	22.6	3	1	0	2	1,707.86
									HELIEFR	95.0%	36.7	21.1	3	0	0	2	943.50
									HNIMDFAMPYFIQVMK	95.0%	49.8	20.0	0	1	0	2	2,032.93
									ISGETIFVTAPHEATAGIIGVNR	95.0%	84.0	18.9	1	8	0	2	2,353.25
									IVLDNSVFSEHR	95.0%	89.2	21.6	2	3	0	2	1,415.73
									IYIDSNNNPER	95.0%	48.7	22.2	7	0	0	2	1,334.63
									KAVDVFFPPEAQNDFPVAMQISEK	95.0%	43.8	21.0	0	2	0	2	2,723.34
									KFNALFAQGNYSEAAK	95.0%	83.8	22.4	2	3	0	2	1,758.88
									LAELEEFINGPNNAHIQQVGDR	95.0%	60.8	21.4	0	16	0	2	2,464.22
									LASTLVHLGEYQAAVDGAR	95.0%	58.6	21.6	1	11	0	2	1,971.03
									LEKHELIEFR	95.0%	36.3	20.8	0	2	0	2	1,313.72
									LHIIEVGTPPTGNQPFPK	95.0%	58.6	20.2	2	6	0	2	1,945.06
									LLEMNLMHAPQVADAILGNQMFTHYDR	95.0%	32.8	20.1	0	0	4	2	3,176.50
									LLLPWLEAR	95.0%	37.2	15.7	4	0	0	2	1,110.67
									LLYNNVSNFGR	95.0%	63.1	22.4	19	0	0	2	1,296.67
									LPVVIGGLLDVDCSEDVIK	95.0%	103.0	19.7	4	0	0	2	2,041.09
									LTDQLPLIIVCDR	95.0%	34.0	19.8	1	0	0	2	1,555.85
									NLQNLLILTAIK	95.0%	106.0	10.0	38	0	0	2	1,353.85
									NNLAGAEELFAR	95.0%	104.0	23.5	29	0	0	2	1,304.66
									NNRPSEGPLQTR	95.0%	41.7	23.2	1	4	0	2	1,368.70
									RPISADSAIMNPASK	95.0%	59.7	23.3	4	1	0	2	1,573.80
									SVDPTLALSVYLR	95.0%	58.4	19.9	2	0	0	2	1,433.80
									SVNESLNNLFITEEDYQALR	95.0%	104.0	21.6	2	2	0	2	2,355.15
									TLQIFNIEMK	95.0%	74.0	22.0	15	0	0	2	1,252.66
									TSIDAYDNFDNISLAQR	95.0%	109.0	22.0	15	0	0	2	1,942.91
									VGEQAQVVIIDMNDPSNPIR	95.0%	87.4	22.2	2	0	0	2	2,211.11
									VIQCFAETGQVQK	95.0%	75.5	22.6	5	0	0	2	1,507.76
									VMEYINR	95.0%	38.8	22.1	7	0	0	2	940.46
									VSQPIEGHAASFAQFK	95.0%	60.2	22.6	1	0	0	2	1,716.87
									VVGAMQLYSVDR	95.0%	56.5	22.3	4	0	0	2	1,353.68
									WLLLTGISAQQNR	95.0%	79.5	20.0	6	0	0	2	1,499.83
Tubulin-specific chaperone A	TBCA_HUMAN	TBCA	12,837	100.00%	5	6	11	38.00%	AEDGENYDIKK	95.0%	36.5	21.4	2	0	0	2	1,281.60
									LEAAYLDLQR	95.0%	74.1	22.6	2	0	0	2	1,191.64
									LVLDSVKLEA	95.0%	50.1	20.8	2	0	0	2	1,086.64
									QAEILQESR	95.0%	36.5	22.9	1	0	0	2	1,073.56
									RLEAAYLDLQR	95.0%	51.1	21.2	2	2	0	2	1,347.74
Annexin A4	ANXA4_HUMAN	ANXA4	35,866	99.50%	2	2	3	10.00%	GLGTDEDAIISVLAYR	95.0%	77.5	21.3	1	0	0	2	1,692.88
									SETSGSFEDALLAIVK	95.0%	98.3	21.7	2	0	0	2	1,666.85
Transcription elongation factor A protein 1	TCEA1_HUMAN	TCEA1	33,953	100.00%	3	3	3	11.60%	EESTSSGNVSNR	95.0%	45.8	19.7	1	0	0	2	1,266.56
									NAAGALDLLK	95.0%	37.2	18.6	1	0	0	2	985.57
									NIPMTLELLQSTR	95.0%	49.2	22.3	1	0	0	2	1,531.82
Fumarate hydratase, mitochondrial	FUMH_HUMAN	FH	54,620	100.00%	14	15	27	36.50%	AAAEVNQDYGLDPK	95.0%	75.0	21.9	2	0	0	2	1,490.71
									AIEMLGGELGSK	95.0%	53.3	23.5	2	0	0	2	1,220.62

									IANAIMK	95.0%	48.8	23.2	2	0	0	2	776.43
									IANDIR	95.0%	32.5	22.7	2	0	0	2	701.39
									IEYDTFGELK	95.0%	42.8	22.4	2	0	0	2	1,214.59
									IGGVTER	95.0%	50.4	25.1	2	0	0	2	731.41
									IPVHPNDHVNK	95.0%	36.1	21.3	1	2	0	2	1,269.67
									IYELAAGGTAVGTGLNTR	95.0%	93.7	21.6	2	0	0	2	1,763.93
									LMNESLMLVTALNPHIGYDK	95.0%	61.4	21.9	0	2	0	2	2,291.14
									SGLGELILPENEPGSSIMPGK	95.0%	62.8	22.2	2	0	0	2	2,141.08
									SKEFAQIIK	95.0%	34.5	19.4	2	0	0	2	1,063.62
									THTQDAVPLTLGQEFSGYVQQVK	95.0%	52.4	21.8	0	2	0	2	2,546.29
									VAALTGLPFVTAPNK	95.0%	68.5	18.0	1	0	0	2	1,498.86
									VPNDKYYGAQTVR	95.0%	26.0	21.6	0	1	0	2	1,510.77
Proteasome subunit beta type-7	PSB7_HUMAN	PSMB7	29,948	100.00%	9	11	32	35.40%	ATEGMVVADKNCSK	95.0%	45.5	21.0	2	0	0	2	1,525.70
									DGIVLGADTR	95.0%	59.2	22.3	4	0	0	2	1,016.54
									FRPDMEEEEAK	95.0%	52.4	17.9	10	3	0	2	1,380.61
									GTTAVLTEK	95.0%	33.9	23.1	1	0	0	2	919.51
									ITPLEIEVLEETVQTMDTS	95.0%	94.5	22.1	6	1	0	2	2,164.06
									LDFLRPYTVPNK	95.0%	29.4	20.4	0	1	0	2	1,462.81
									LDFLRPYTVPNKK	95.0%	39.0	15.8	0	2	0	2	1,590.90
									LPYVTMGSGSLAAMAVFEDK	95.0%	51.8	22.2	1	0	0	2	2,119.01
									NKLDFLRPYTVPNK	95.0%	31.7	18.9	0	1	0	2	1,704.94
Coatomer subunit alpha	COPA_HUMAN	COPA	138,331	100.00%	5	6	31	5.47%	LLELGPKPEVAQQTR	95.0%	45.2	16.6	0	6	0	2	1,678.95
									LLHDQVGVIQFGPYK	94.5%	25.5	20.8	0	1	0	2	1,713.93
									SILLSVPLLVVDNK	95.0%	65.1	11.5	6	0	0	2	1,509.93
									TALNLFFK	95.0%	60.4	20.2	12	0	0	2	953.55
									VTTVTEIGKDVIGLR	95.0%	74.3	14.5	5	1	0	2	1,600.93
Beta-hexosaminidase subunit beta	HEXB_HUMAN	HEXB	63,095	100.00%	15	18	90	26.80%	DMDDAYDR	95.0%	35.7	8.5	2	0	0	2	1,016.36
									DVRDMDDAYDR	95.0%	44.5	14.1	7	0	0	2	1,386.56
									EISEVFPDQFIHLGGDEVEFK	95.0%	121.0	21.3	3	6	0	2	2,435.18
									GIAAQPLYAGYCNHENM	95.0%	59.5	18.3	2	0	0	2	1,924.83
									GILIDTSR	95.0%	41.4	24.1	2	0	0	2	874.50
									GSIVWQEVFDDK	95.0%	51.7	22.5	2	0	0	2	1,422.69
									GSYSLSHVYTPNDVR	95.0%	93.1	22.6	10	6	0	2	1,694.81
									KLESFYIQK	95.0%	44.6	20.5	3	0	0	2	1,155.64
									LESFYIQK	95.0%	31.8	20.7	1	0	0	2	1,027.55
									MVIEYAR	95.0%	32.5	22.1	3	0	0	2	897.45
									TLDAMAFNK	95.0%	56.7	20.1	5	0	0	2	1,026.49
									VEPLDFGGTQK	95.0%	46.4	23.7	4	0	0	2	1,190.61
									VLDIIATINK	95.0%	68.5	17.5	18	0	0	2	1,099.67
									VLPEFDTPGHTLSWGK	95.0%	34.4	22.0	1	0	0	2	1,783.90
									YYKVEPLDFGGTQK	95.0%	70.6	22.7	8	7	0	2	1,644.83
F-actin-capping protein subunit beta	CAPZB_HUMAN	CAPZB	31,334	100.00%	6	7	36	19.90%	KLEVEANNAFDQYR	95.0%	113.0	22.4	2	3	0	2	1,696.83
									LEVEANNAFDQYR	95.0%	80.0	22.0	2	0	0	2	1,568.73
									LVEDMENK	95.0%	31.1	20.7	1	0	0	2	993.46
									RLPPQQIEK	95.0%	36.1	18.3	4	0	0	2	1,108.65
									SGSGTMNLGGSLTR	95.0%	90.2	21.7	11	0	0	2	1,337.65
									STLNEIYFGK	95.0%	76.7	22.8	13	0	0	2	1,171.60
Superoxide dismutase [Mn], mitochondrial	SODM_HUMAN	SOD2	24,705	100.00%	3	3	6	16.70%	AIWNVINWENVTER	95.0%	89.1	22.6	2	0	0	2	1,743.88
									GDVTAQIALQPALK	95.0%	75.1	17.5	2	0	0	2	1,424.81
									GELLEAIKR	95.0%	48.0	19.6	2	0	0	2	1,028.61
Nucleolar RNA helicase 2	DDX21_HUMAN	DDX21	87,328	100.00%	3	3	5	6.13%	EGAFSNFPISEETIK	95.0%	38.8	21.8	1	0	0	2	1,668.81

									GAVEALAAALAHISGATSVDQR	95.0%	35.7	19.8	0	2	0	2	2,108.11
									TFSFAIPLIEK	95.0%	35.8	18.6	2	0	0	2	1,265.71
Heat shock protein 105 kDa	HS105_HUMAN	HSPH1	96,848	100.00%	22	25	68	29.60%	AFNDPFIQK	95.0%	45.0	23.1	2	0	0	2	1,079.55
									DISTTLNADEAVAR	95.0%	80.5	22.7	2	0	0	2	1,475.73
									DLLNMYIETEGK	95.0%	71.2	21.6	2	0	0	2	1,441.69
									EKENLSYDLVPLK	95.0%	72.8	21.4	2	0	0	2	1,547.83
									ENLSYDLVPLK	95.0%	69.3	22.2	2	0	0	2	1,290.69
									FQEAEERPK	95.0%	45.9	23.1	5	5	0	2	1,133.56
									FVVQNVSAQK	95.0%	45.6	22.6	2	0	0	2	1,119.62
									GPFELEAFYSDPQGVPYPEAK	95.0%	71.1	21.6	2	0	0	2	2,341.10
									IEVPLYSLLEQTHLK	95.0%	74.7	17.8	2	1	0	2	1,783.00
									LKETAENSLK	95.0%	36.5	22.8	2	2	0	2	1,132.62
									LMNDMTAVALNYGIYK	95.0%	101.0	22.8	2	0	0	2	1,848.89
									MFEELGQR	95.0%	48.6	20.9	2	0	0	2	1,025.47
									NAVEEYVYEFR	95.0%	61.5	21.5	5	0	0	2	1,418.66
									NHAAPFSK	95.0%	35.6	20.7	3	0	0	2	871.44
									NQQITHANNTVSNFK	95.0%	26.7	21.9	0	1	0	2	1,715.85
									NQQITHANNTVSNFKR	95.0%	34.4	22.2	0	2	0	2	1,871.95
									QAYVDKLEELMK	95.0%	55.2	22.5	2	0	0	2	1,482.75
									RGPFELEAFYSDPQGVPYPEAK	95.0%	39.9	21.2	0	2	0	2	2,497.20
									SLDQDPVVR	95.0%	37.5	20.8	2	0	0	2	1,028.54
									VEDVSAVEIVGGATR	95.0%	102.0	22.9	2	0	0	2	1,501.79
									VLGTAFDPFLGGK	95.0%	83.1	21.1	13	0	0	2	1,321.72
									VMYMGEEHLFSVEQITAMLLTK	95.0%	41.5	21.3	0	2	0	2	2,618.26
									YNHIDESEMK	95.0%	33.6	16.4	2	0	0	2	1,281.54
SEC14-like protein 2	S14L2_HUMAN	SEC14L2	46,128	100.00%	3	3	5	10.90%	AGEMTEVLPNQR	95.0%	38.6	21.8	1	0	0	2	1,360.65
									HISPDQVPVEYGGTMTDPDGNPK	95.0%	74.2	20.3	0	2	0	2	2,470.12
									INYGGDIPR	95.0%	48.2	23.3	2	0	0	2	1,004.52
40S ribosomal protein S26	RS26_HUMAN	RPS26	12,998	100.00%	3	3	4	33.90%	DISEASVFDAYVLPK	95.0%	46.9	22.6	1	0	0	2	1,653.84
									FRPAGAAPRPPPKPM	95.0%	23.3	21.0	0	0	1	2	1,605.87
									NIVEAAAVR	95.0%	45.2	20.0	2	0	0	2	942.54
Proteasome-associated protein ECM29 homolog	ECM29_HUMAN	ECM29	204,278	100.00%	7	7	13	4.88%	AGEQLAPFLPQLVPR	95.0%	63.6	16.7	4	0	0	2	1,635.92
									FQEFSNIVIPLIK	95.0%	51.4	16.7	2	0	0	2	1,547.88
									IVAISCAADILK	95.0%	31.1	20.3	1	0	0	2	1,273.72
									LMSALLSGLTDR	95.0%	51.2	22.3	1	0	0	2	1,292.69
									TEALSVIELLLK	95.0%	70.6	12.0	2	0	0	2	1,328.80
									TLMSSGQMAPSSSNK	95.0%	91.1	18.4	2	0	0	2	1,557.69
									YLLLLAAGDPR	95.0%	46.4	19.4	1	0	0	2	1,201.69
Ataxin-10	ATX10_HUMAN	ATXN10	53,473	100.00%	3	3	5	9.05%	LSGVMVPAPIQDLEALR	95.0%	43.1	19.5	3	0	0	2	1,824.99
									NLTEDNSQNQDLIAK	95.0%	52.8	21.7	1	0	0	2	1,702.83
									SPELVQAMFPK	95.0%	32.8	23.3	1	0	0	2	1,262.65
Polymerase delta-interacting protein 2	PDIP2_HUMAN	POLDIP2	42,015	99.50%	2	2	3	6.52%	LENLDSDVVQLR	95.0%	78.1	22.2	1	0	0	2	1,400.74
									VLETVGVFEVPK	95.0%	60.3	20.0	2	0	0	2	1,316.75
Farnesyl pyrophosphate synthase	FPPS_HUMAN	FDPS	48,259	100.00%	9	12	49	24.60%	ATPEQYQILK	95.0%	52.9	22.5	4	0	0	2	1,190.64
									EVLEYNAIGGK	95.0%	61.0	23.3	2	0	0	2	1,192.62
									GLTVVVAFR	95.0%	47.2	17.9	10	0	0	2	961.58
									IGTDIQDNK	95.0%	40.4	22.8	5	0	0	2	1,003.51
									KQDADSLQR	95.0%	41.2	24.3	3	0	0	2	1,060.54
									LKEVLEYNAIGGK	95.0%	74.6	19.2	4	8	0	2	1,433.80
									QDFVQHFSQIVR	95.0%	51.1	23.2	2	6	0	2	1,503.77
									TAFYSFYLPIAAAMYMAGIDGEK	95.0%	42.1	20.8	1	0	0	2	2,562.19

									VLTEDEMGHPEIGDAIAR	95.0%	71.2	21.6	2	2	0	2	1,968.93
Superkiller viralicidic activity 2-like 2	SK2L2_HUMAN	SKIV2L2	117,790	100.00%	3	3	4	4.03%	ALFATETFAMGINMPAR	95.0%	46.5	22.3	2	0	0	2	1,872.90
									DVDFEGTDEPIFGK	95.0%	42.9	20.6	1	0	0	2	1,568.71
									EYPFILDAFQR	95.0%	42.8	22.5	1	0	0	2	1,398.71
CLIP-associating protein 1	CLAP1_HUMAN	CLASP1	169,438	100.00%	3	3	5	2.73%	AQIGTVLPSLIDR	95.0%	49.1	17.2	2	0	0	2	1,382.80
									LQVGQELIDYFSDK	95.0%	77.1	22.2	1	0	0	2	1,654.83
									VVLLGMDILSALVTR	95.0%	102.0	14.3	2	0	0	2	1,615.95
Sorbitol dehydrogenase	DHSO_HUMAN	SORD	38,307	100.00%	8	11	21	30.50%	ALEAFETFKK	95.0%	53.4	21.8	2	0	0	2	1,183.64
									AMGAAQVVVTDLSATR	95.0%	136.0	22.4	2	0	0	2	1,605.83
									GGVTLGHK	95.0%	54.5	20.1	1	0	0	2	768.44
									IGNFIVK	95.0%	42.5	17.9	2	0	0	2	790.48
									KPMVLGHEASGTVEK	95.0%	74.8	22.0	2	1	1	2	1,598.82
									LENYPIPEPGPNEVLLR	95.0%	64.6	20.6	3	0	0	2	1,950.03
									SGGNLVLVGLGSEMTTVPLLHAAIR	95.0%	49.9	16.4	0	4	0	2	2,521.38
									SVNVKPLVTHR	95.0%	35.5	16.0	1	2	0	2	1,249.74
Histone-arginine methyltransferase CARM1	CARM1_HUMAN	CARM1	63,442	100.00%	2	2	3	3.42%	LLTIGDANGEIQR	95.0%	62.6	22.0	2	0	0	2	1,399.75
									SNNLTDR	95.0%	36.3	23.0	1	0	0	2	819.40
Disintegrin and metalloproteinase domain-containing protein 10	ADA10_HUMAN	ADAM10	84,125	100.00%	7	8	29	11.60%	AIDTIYQTTDFSGIR	95.0%	96.8	22.3	11	0	0	2	1,700.85
									EAVIAQISSHVK	95.0%	45.9	20.8	4	0	0	2	1,281.72
									FEGFIQTR	95.0%	48.5	22.4	2	0	0	2	997.51
									FPNIGVEK	95.0%	39.3	22.7	6	0	0	2	903.49
									GGTFYVEPAER	95.0%	31.0	21.9	1	0	0	2	1,225.59
									SLNTGIITVQNYGSHVPPK	95.0%	38.6	21.0	1	3	0	2	2,025.08
									TITLQPGSPCNDFR	95.0%	35.0	22.3	1	0	0	2	1,605.77
DCN1-like protein 1	DCNL1_HUMAN	DCUN1D1	30,108	100.00%	2	2	4	10.40%	LDVATDNFFQNPELYIR	95.0%	90.2	23.4	2	0	0	2	2,055.02
									MEQELKEPGR	95.0%	32.1	22.7	0	2	0	2	1,232.59
Alcohol dehydrogenase [NADP+]	AK1A1_HUMAN	AKR1A1	36,556	100.00%	7	7	22	27.10%	AVPREELFVTSK	95.0%	36.4	21.2	2	0	0	2	1,375.76
									DPDEPVLLEEPVVLALAEK	95.0%	96.0	20.7	6	0	0	2	2,076.11
									GLEVTAYSPLGSSDR	95.0%	69.6	23.3	3	0	0	2	1,551.77
									GLVQALGLSNFNSR	95.0%	75.0	22.0	2	0	0	2	1,475.80
									SPAQILLR	95.0%	60.6	15.3	3	0	0	2	897.55
									VFDFTFSPEEMK	95.0%	66.9	20.2	4	0	0	2	1,492.67
									YALSVGYR	95.0%	43.0	20.9	2	0	0	2	928.49
Disintegrin and metalloproteinase domain-containing protein 9	ADAM9_HUMAN	ADAM9	90,538	100.00%	4	6	24	4.52%	DLLPEDFVVYTYNK	95.0%	55.8	22.0	3	0	0	2	1,715.85
									MDDVYKEPLK	95.0%	53.0	23.2	6	1	0	2	1,253.61
									NKDLLPEDFVVYTYNK	95.0%	92.1	21.7	8	4	0	2	1,957.99
									QVSYVIQAEGK	95.0%	35.8	23.2	2	0	0	2	1,221.65
Galactosylgalactosylxylosylprotein 3-beta-glucuronosyltransferase 3	B3GA3_HUMAN	B3GAT3	37,104	100.00%	5	5	16	18.80%	AAAEQLR	95.0%	45.7	22.6	2	0	0	2	758.42
									FEGPQVQDGR	95.0%	56.6	21.2	2	0	0	2	1,132.54
									ISQLQAELR	95.0%	67.3	21.3	4	0	0	2	1,057.60
									LSQTLSLVPR	95.0%	68.3	19.4	6	0	0	2	1,113.66
									RPPPAPAQPPEPEALPTIYVVTPTYAR	95.0%	28.6	16.8	0	2	0	2	2,928.56
Proteasome subunit alpha type-5	PSA5_HUMAN	PSMA5	26,393	100.00%	7	8	40	38.60%	AIGSASEGAQSSLQEVYHK	95.0%	97.7	21.9	2	2	0	2	1,961.96
									EELEEVIKDI	95.0%	54.3	23.4	1	0	0	2	1,216.63
									GVNTFSPEGR	95.0%	35.8	23.1	2	0	0	2	1,063.52
									ITSPLMEPSSIEK	95.0%	75.6	22.9	5	0	0	2	1,447.74
									LFQVEYAIEAIK	95.0%	94.7	19.5	18	0	0	2	1,423.78
									LNATNIELATVQPGQNFHMFTK	95.0%	55.0	21.2	0	2	0	2	2,490.25
									SSLIILK	95.0%	67.5	12.0	8	0	0	2	773.51
Annexin A11	ANX11_HUMAN	ANXA11	54,374	100.00%	4	4	6	9.31%	DAQELYAAGENR	95.0%	61.0	20.8	2	0	0	2	1,336.61
									FNAVLCSR	95.0%	41.4	23.2	1	0	0	2	966.48

									SLYHDISGDTSGDYRK	95.0%	37.6	20.8	0	1	0	2	1,813.84
									TPVLFDIYEIK	95.0%	46.7	20.4	2	0	0	2	1,337.74
Ribonucleoside-diphosphate reductase large subunit	RIR1_HUMAN	RRM1	90,056	99.50%	2	2	5	3.79%	EQGPYETYEGSPVSK	95.0%	75.3	20.1	4	0	0	2	1,670.76
									VLSGEFQIVNPHLLK	94.8%	25.7	15.9	0	1	0	2	1,693.96
78 kDa glucose-regulated protein	GRP78_HUMAN	HSPA5	72,317	100.00%	30	40	311	49.70%	AKFEELNMDLFR	95.0%	66.6	22.2	13	9	0	2	1,528.75
									ALSSQHQAR	95.0%	44.9	21.6	1	0	0	2	997.52
									DAGTIAGLNVMR	95.0%	74.3	23.5	20	0	0	2	1,233.63
									DNHLLGTFDLTGIPPAPR	95.0%	101.0	22.2	11	17	0	2	1,934.01
									EFFNGKEPSR	95.0%	52.3	22.4	3	0	0	2	1,210.59
									ELEEIVQPIISK	95.0%	68.8	18.9	8	0	0	2	1,397.79
									FEELNMDLFR	95.0%	53.5	20.8	4	0	0	2	1,329.62
									IDTRNELESYAYSLK	95.0%	32.9	22.3	0	2	0	2	1,801.90
									IEIESFYEGEDFSETLTR	95.0%	120.0	20.4	6	0	0	2	2,164.99
									IINEPTAAAIAYGLDK	95.0%	110.0	21.5	26	2	0	2	1,659.90
									IINEPTAAAIAYGLDKR	95.0%	108.0	19.3	8	20	0	2	1,816.00
									IQQLVK	95.0%	32.3	19.0	1	0	0	2	728.47
									ITITNDQNR	95.0%	46.1	23.3	9	0	0	2	1,074.55
									ITPSYVAFTPEGER	95.0%	88.8	22.4	20	0	0	2	1,566.78
									KSDIDEIVLVGGSTR	95.0%	106.0	22.3	4	3	0	2	1,588.85
									KSQIFSTASDNQPTVTIK	95.0%	68.6	20.6	1	0	0	2	1,965.03
									KVTHAVVTVPAYFNDAQR	95.0%	51.3	20.2	0	4	2	2	2,016.07
									LTPEEIER	95.0%	33.5	21.2	1	0	0	2	986.52
									LYGSAGPPPTGEEDTAEKDEL	95.0%	116.0	21.0	15	0	0	2	2,175.99
									MKETAEAYLGK	95.0%	44.4	21.9	3	3	0	2	1,256.62
									NELESYAYSLK	95.0%	82.9	22.3	7	0	0	2	1,316.64
									NQIGDKEK	95.0%	33.0	24.1	1	0	0	2	931.49
									NQLTSNPENTVFDAK	95.0%	104.0	23.0	20	0	0	2	1,677.81
									NQLTSNPENTVFDAKR	95.0%	101.0	22.2	4	1	0	2	1,833.91
									SDIDEIVLVGGSTR	95.0%	58.2	22.7	4	0	0	2	1,460.76
									SQIFSTASDNQPTVTIK	95.0%	94.2	21.9	19	1	0	2	1,836.93
									TFAPEEISAMVLTK	95.0%	94.6	21.7	25	0	0	2	1,552.79
									TKPYIQVDIGGGQTK	95.0%	93.8	20.8	6	2	0	2	1,604.87
									VEIIANDQGNR	95.0%	74.6	22.6	42	0	0	2	1,228.63
									VLEDSDLKK	95.0%	40.1	23.4	5	0	0	2	1,046.57
									VTHAVVTVPAYFNDAQR	95.0%	87.3	22.2	4	17	0	2	1,887.97
									VYEGERPLTK	95.0%	38.6	22.6	7	0	0	2	1,191.64
Aminoacyl tRNA synthase complex-interacting multifunctional protein 1	AIMP1_HUMAN	AIMP1	34,335	100.00%	7	10	20	30.80%	AQTMSNSGIK	95.0%	34.0	21.9	1	0	0	2	1,052.50
									GAEADQIIEYLK	95.0%	85.2	22.6	2	0	0	2	1,349.70
									ITFDAFPGEPDKELNPK	95.0%	50.4	22.3	2	4	0	2	1,917.96
									KHPDADSLYVEEVDVGEIAPR	94.8%	17.7	21.9	0	0	1	2	2,339.15
									KQQSIAGSADSKPIDVSR	95.0%	36.4	21.1	0	1	1	2	1,886.99
									QQSIAGSADSKPIDVSR	95.0%	40.2	22.4	0	2	0	2	1,758.90
									TVVSGLVNHVPLEQMQNR	95.0%	71.7	21.7	2	4	0	2	2,037.06
Peroxiredoxin-2	PRDX2_HUMAN	PRDX2	21,874	100.00%	12	14	98	45.50%	AEDFRK	95.0%	32.1	23.3	1	0	0	2	765.39
									ATAVVDGAFK	95.0%	61.0	22.6	7	0	0	2	978.53
									ATAVVDGAFKEVK	95.0%	62.1	21.3	4	0	0	2	1,334.73
									EGGLGPLNIPLLADVTR	95.0%	81.4	16.6	32	2	0	2	1,734.98
									GLFIIDGK	95.0%	36.2	18.1	2	0	0	2	862.50
									IGKPAPDFK	95.0%	33.0	24.2	2	0	0	2	972.55
									KEGGLGPLNIPLLADVTR	95.0%	116.0	14.9	12	19	0	2	1,863.07
									LSEDYGVLK	95.0%	51.0	22.7	6	0	0	2	1,023.54

									LSEDYGVLKTDEGIAYR	95.0%	39.8	22.3	0	1	0	2	1,928.96
									QITVNDLPVGR	95.0%	82.6	20.9	28	0	0	2	1,211.68
									RLSEDYGVLK	95.0%	48.0	21.9	2	0	0	2	1,179.64
									SVDEALR	95.0%	43.2	24.9	2	0	0	2	789.41
									TDEGIAYR	95.0%	53.9	21.2	6	0	0	2	924.44
Gamma-glutamyl hydrolase	GGH_HUMAN	GGH	35,948	100.00%	7	9	59	27.00%	FFNVLTTNTDGK	95.0%	68.7	22.1	9	0	0	2	1,356.68
									KPIIGILMQK	95.0%	43.6	15.1	4	0	0	2	1,156.71
									LDLTEKDYEILFK	95.0%	68.6	21.0	8	7	0	2	1,626.86
									NLDGISHAPNAVK	95.0%	58.8	22.1	4	0	0	2	1,335.70
									SINGILFPGGSVDLR	95.0%	58.6	20.8	6	0	0	2	1,544.84
									TAFYLAEFFVNEAR	95.0%	94.2	23.1	14	1	0	2	1,677.83
									YYIAASYVK	95.0%	50.7	23.9	6	0	0	2	1,077.56
Ribose-5-phosphate isomerase	RPIA_HUMAN	RPIA	33,251	99.50%	2	2	3	11.60%	MIPGVVDTGLFINMAER	95.0%	68.5	22.6	2	0	0	2	1,894.94
									NNQVLGIGSGSTIVHAVQR	95.0%	35.2	19.5	0	1	0	2	1,950.05
Enoyl-CoA hydratase, mitochondrial	ECHM_HUMAN	ECHS1	31,370	100.00%	3	3	6	19.70%	ALNALCDGLIDELNQALK	95.0%	86.1	21.8	2	0	0	2	1,971.02
									AQFAQPEILIGTIPGAGGTQR	95.0%	105.0	18.9	2	0	0	2	2,125.14
									TFEEDPAVGAIVLTGGDK	95.0%	89.9	22.3	2	0	0	2	1,818.91
26S proteasome non-ATPase regulatory subunit 12	PSD12_HUMAN	PSMD12	52,888	100.00%	5	5	11	14.00%	GSLESPATDVFGSTEEGEKR	95.0%	25.9	21.3	0	1	0	2	2,095.98
									LFTTMELMR	95.0%	44.4	21.9	2	0	0	2	1,173.56
									LNSLMSLVNK	95.0%	47.9	22.5	2	0	0	2	1,134.62
									LQEVIETLLSLEK	95.0%	69.0	18.5	4	0	0	2	1,514.87
									MEVDYSATVDQR	95.0%	72.8	18.7	2	0	0	2	1,429.63
Basigin	BASI_HUMAN	BSG	42,182	100.00%	3	3	3	10.60%	FFVSSSQGR	95.0%	32.5	20.5	1	0	0	2	1,014.50
									GGVVLKEDALPGQK	94.9%	25.8	17.5	0	1	0	2	1,410.80
									SELHIENLNMEADPGQYR	95.0%	30.1	21.0	0	1	0	2	2,131.97
UMP-CMP kinase	KCY_HUMAN	CMPK1	22,205	100.00%	7	8	22	44.40%	FLIDGFPR	95.0%	55.5	22.0	7	0	0	2	964.53
									IQTYLQSTKPIIDLYEEMGK	95.0%	32.8	20.9	0	3	0	2	2,386.22
									KNPDSQYGELIEK	95.0%	89.9	23.0	4	0	0	2	1,520.76
									MKPLVVFVLGGPGAGK	95.0%	69.8	16.4	1	1	0	2	1,585.91
									NPDSQYGELIEK	95.0%	34.1	21.9	1	0	0	2	1,392.66
									SVDEVFDEVVQIFDKEG	95.0%	49.0	21.6	3	0	0	2	1,954.93
									YGYTHLSAGELLR	95.0%	51.4	22.6	0	2	0	2	1,479.76
Glucosamine-6-phosphate isomerase 1	GNPI1_HUMAN	GNPDA1	32,651	100.00%	9	10	42	47.80%	AAGGIELFVGGIGPDGHIAFNEPGSSLVSR	95.0%	42.7	20.3	0	2	0	2	2,924.49
									AIEEGVNHMWTVSAFQQHPR	95.0%	35.1	21.6	0	1	0	2	2,353.11
									EVMILITGAHK	95.0%	45.9	21.6	4	0	0	2	1,227.68
									IIQFNPGPEK	95.0%	50.8	22.9	6	0	0	2	1,142.62
									LVDPLYSIK	95.0%	32.5	16.1	1	0	0	2	1,047.61
									TFNMDEYVGLPR	95.0%	78.1	21.3	9	0	0	2	1,457.67
									TLAMDTILANAR	95.0%	74.8	23.3	6	0	0	2	1,289.69
									VPTMALTVGVGTVMDAR	95.0%	107.0	22.6	6	5	0	2	1,749.89
									YFTLGLPTGSTPLGCYK	95.0%	74.8	22.1	2	0	0	2	1,874.94
Stromelysin-3	MMP11_HUMAN	MMP11	54,601	100.00%	4	4	8	7.99%	ALEGFPR	95.0%	35.8	24.5	2	0	0	2	789.43
									ALMSAFYTFR	95.0%	41.0	22.3	2	0	0	2	1,222.59
									GGQLQPGYPALASR	95.0%	43.9	22.1	3	0	0	2	1,414.74
									QTMAEALK	95.0%	31.6	23.8	1	0	0	2	907.46
Galactocerebrosidase	GALC_HUMAN	GALC	77,019	100.00%	4	4	11	7.45%	EFDGIGAVSGGGATSR	95.0%	98.6	22.0	3	0	0	2	1,480.70
									LLVNYPEPYR	95.0%	52.0	22.5	3	0	0	2	1,263.67
									MLNYQGLQR	95.0%	60.9	22.7	3	0	0	2	1,138.57
									VVDVIGAHYPGTHSAK	95.0%	41.1	21.8	0	2	0	2	1,650.86
Spermine synthase	SPSY_HUMAN	SMS	41,252	100.00%	7	8	19	20.80%	DVLILGGGDGGILCEIVK	95.0%	84.8	20.4	2	0	0	2	1,827.99
									ELSQDSTGR	95.0%	43.1	22.2	2	0	0	2	992.47

									HSTLDFMLGAK	95.0%	47.5	22.8	4	0	0	2	1,235.61
									LILDLSMK	95.0%	34.6	20.3	2	0	0	2	948.54
									LVEYDIDEVVYDEDSPYQNIK	95.0%	103.0	20.7	2	2	0	2	2,546.18
									MKELSQDSTGR	95.0%	55.5	21.6	2	0	0	2	1,267.60
									RLPPIVR	95.0%	33.1	11.5	3	0	0	2	850.56
Eukaryotic translation initiation factor 3 subunit E	EIF3E_HUMAN	EIF3E	52,205	100.00%	12	13	21	31.70%	ETIDNNSVSSPLQSLQQR	95.0%	114.0	22.3	2	0	0	2	2,016.00
									HLVFPLLEFLSVK	95.0%	29.6	12.3	0	2	0	2	1,541.91
									LDLLSDTNMVDFAMDVYK	95.0%	115.0	20.2	2	0	0	2	2,121.97
									LFIFETFCR	95.0%	40.9	23.5	1	0	0	2	1,232.61
									LGHVVMGNNAVSPYQQVIEK	95.0%	37.6	21.5	0	1	0	2	2,199.12
									LKETIDNNSVSSPLQSLQQR	95.0%	52.6	20.5	0	2	0	2	2,257.18
									LNMTPEEAER	95.0%	46.4	20.3	2	0	0	2	1,205.55
									MLFDYLADK	95.0%	39.8	20.2	2	0	0	2	1,131.54
									NLYSDDIPHALR	95.0%	61.8	21.8	2	1	0	2	1,413.71
									QEYLDTLYR	95.0%	34.1	21.8	1	0	0	2	1,200.59
									QLQAETEPIVK	95.0%	45.6	20.8	2	0	0	2	1,255.69
									SQMLAMNIEK	95.0%	33.4	22.9	1	0	0	2	1,196.57
45 kDa calcium-binding protein	CAB45_HUMAN	SDF4	41,789	100.00%	11	15	117	30.10%	AVDPDGDGHVSWDEYK	95.0%	58.0	17.5	1	0	0	2	1,789.77
									DLGGFDEDAEPR	95.0%	80.0	15.6	10	0	0	2	1,320.57
									DLGGFDEDAEPRR	95.0%	42.3	21.2	6	2	0	2	1,476.67
									GFHQEVFLGK	95.0%	46.4	23.4	8	0	0	2	1,161.61
									GHSEKEVADAIR	95.0%	39.8	22.7	0	7	0	2	1,311.67
									LEMDGHLNR	95.0%	43.0	19.9	7	2	0	2	1,100.52
									LMVIFSK	95.0%	43.2	20.2	7	0	0	2	853.49
									QMIAVADENQNHHLEPEEVLK	95.0%	53.7	22.4	0	12	1	2	2,444.19
									TAEHFQEAMEESK	95.0%	72.6	18.1	33	10	0	2	1,536.66
									VDVNTDR	95.0%	47.2	23.3	9	0	0	2	818.40
									VDVNTDRK	95.0%	33.2	23.6	2	0	0	2	946.50
Plasminogen activator inhibitor 1	PAI1_HUMAN	SERPINE1	45,042	100.00%	16	21	414	52.00%	ELMGPWNKDEISTTDAIFVQR	95.0%	43.8	20.8	0	1	0	2	2,466.20
									FSLETEVDLR	95.0%	74.9	23.3	99	0	0	2	1,208.62
									GAVDQLTR	95.0%	49.0	23.4	8	0	0	2	859.46
									GMISNLLGK	95.0%	69.1	22.8	14	0	0	2	948.52
									HNPTGTVLFMGQVMEP	95.0%	103.0	20.9	17	0	0	2	1,789.83
									IDDKGMAPALR	95.0%	57.8	23.9	5	3	0	2	1,202.62
									KPLENLGMTDMFR	95.0%	61.7	21.5	15	15	0	2	1,583.76
									LVQGFMPHFFR	95.0%	56.0	22.9	18	0	0	2	1,394.70
									MAPEEIIMDRPFLFVVR	95.0%	33.9	21.7	0	6	0	2	2,095.07
									QFQADFTSLSDQEPLHVAQALQK	95.0%	80.3	21.2	1	52	0	2	2,601.30
									QVDFSEVER	95.0%	73.0	22.2	33	0	0	2	1,108.53
									SDGSTVSVPMMAQTNK	95.0%	62.2	20.1	38	0	0	2	1,684.75
									TPFPDSSTHR	95.0%	44.2	20.7	12	20	0	2	1,144.54
									VFQQVAQASK	95.0%	79.8	22.9	32	0	0	2	1,105.60
									VFQQVAQASKDR	95.0%	60.9	22.7	4	4	0	2	1,376.73
									VKIEVNESGTVASSSTAVIVSAR	95.0%	40.4	18.5	0	17	0	2	2,304.24
Peroxiredoxin-6	PRDX6_HUMAN	PRDX6	25,018	100.00%	12	16	99	48.20%	DFTPVCTTELGR	95.0%	55.1	21.2	3	0	0	2	1,395.66
									DGDSVMVLPTIPEEEAK	95.0%	50.1	21.8	7	0	0	2	1,845.88
									DGDSVMVLPTIPEEEAKK	95.0%	42.7	22.5	0	2	0	2	1,973.97
									ELAILLGMLDPAEK	95.0%	70.4	21.1	2	0	0	2	1,528.83
									ELAILLGMLDPAEKDEK	95.0%	63.1	20.8	12	2	0	2	1,900.99
									LKLSILYPATTGR	95.0%	49.6	14.8	2	3	0	2	1,432.85
									LPFPIIDDR	95.0%	59.1	20.9	19	0	0	2	1,085.60

									LSILYPATTGR	95.0%	57.9	19.5	6	0	0	2	1,191.67
									PGGLLLGDVAPNFEANTTVGR	95.0%	112.0	20.3	21	4	0	1	2,098.09
									VATPVDWKDGDSVMVLPTIPEEEAK	95.0%	28.4	21.1	0	1	0	2	2,742.36
									VVFVFGPDK	95.0%	39.3	21.8	4	0	0	2	1,007.56
									VVFVFGPDKK	95.0%	57.7	18.9	10	1	0	2	1,135.65
Developmentally-regulated GTP-binding protein 1	DRG1_HUMAN	DRG1	40,526	100.00%	3	3	5	12.30%	GGGGGGPGEGFDVAK	95.0%	51.2	20.4	2	0	0	2	1,261.58
									IQLLDLPGIIEGAK	95.0%	76.2	11.1	2	0	0	2	1,479.88
									SDATADDLIDVVEGNR	95.0%	94.5	21.7	1	0	0	2	1,689.79
Tetratricopeptide repeat protein 38	TTC38_HUMAN	TTC38	52,770	99.50%	2	2	3	4.26%	LFDATLTQYVK	95.0%	70.8	21.6	2	0	0	2	1,298.70
									VLELLLPIR	95.0%	59.9	8.5	1	0	0	2	1,065.70
Pyridoxine-5'-phosphate oxidase	PNPO_HUMAN	PNPO	29,970	99.90%	2	2	4	8.81%	EAFEETHLTSLDPVK	95.0%	58.0	22.0	2	0	0	2	1,715.85
									FFTNFESR	95.0%	52.3	21.5	2	0	0	2	1,047.49
C-1-tetrahydrofolate synthase, cytoplasmic	C1TC_HUMAN	MTHFD1	101,544	100.00%	10	10	19	13.60%	AAQAPSSFQLLYDLK	95.0%	85.6	21.5	2	0	0	2	1,651.87
									GALALAQAVQR	95.0%	91.7	17.2	2	0	0	2	1,097.64
									GVPTGFILPIR	95.0%	85.7	17.5	2	0	0	2	1,169.70
									IYGADDIELLPEAQHK	94.7%	25.7	22.1	0	1	0	2	1,811.92
									MFGIPVVVAVNAFK	95.0%	70.5	20.4	2	0	0	2	1,507.83
									TDPTTLTDEEINR	95.0%	69.9	21.0	2	0	0	2	1,504.71
									TDTESELDLISR	95.0%	68.7	22.5	2	0	0	2	1,378.67
									TPVPSDIDISR	95.0%	55.2	21.8	2	0	0	2	1,199.63
									VLLSALER	95.0%	46.8	18.9	2	0	0	2	900.55
									YVVVTGITPTPLGEGK	95.0%	87.7	20.1	2	0	0	2	1,630.91
Prolactin-inducible protein	PIP_HUMAN	PIP	16,555	100.00%	3	3	6	26.00%	FYTIEILKVE	95.0%	39.5	19.3	2	0	0	2	1,254.70
									TVQIAAVVDVIR	95.0%	70.4	17.2	2	0	0	2	1,283.77
									TYLISSIPLQGAFNYK	95.0%	56.2	21.0	2	0	0	2	1,814.97
Complement C1s subcomponent	C1S_HUMAN	C1S	76,666	100.00%	14	16	151	21.80%	EPTMYVGSTSVQTSR	95.0%	83.7	20.9	9	0	0	2	1,658.77
									GDSGGAFAVQDPNDK	95.0%	83.9	19.0	3	0	0	2	1,477.66
									GDSGGAFAVQDPNDKTK	95.0%	65.6	24.1	4	0	0	2	1,706.80
									GFQVVVTLR	95.0%	58.3	17.6	4	0	0	2	1,018.61
									IIGGSDADIK	95.0%	36.5	23.1	2	0	0	2	988.53
									LKDPVK	95.0%	32.2	15.6	1	0	0	2	699.44
									LLEVPEGR	95.0%	34.9	19.3	2	0	0	2	912.52
									LQVIFK	95.0%	37.9	15.6	7	0	0	2	747.48
									SDFSNEER	95.0%	57.6	14.9	9	0	0	2	983.41
									SNALDIIFQTDLTGQK	95.0%	116.0	22.1	45	1	0	2	1,763.92
									SSNNPHSPIVEEFQVPYNK	95.0%	51.7	21.3	3	21	0	2	2,186.05
									TMQENSTPRED	95.0%	48.6	16.1	3	0	0	2	1,323.55
									TNFDNDIALVR	95.0%	74.9	23.5	33	0	0	2	1,277.65
									VEDPESTLFGSVIR	95.0%	116.0	23.2	4	0	0	2	1,548.79
Coatomer subunit beta	COPB_HUMAN	COPB1	107,128	100.00%	5	5	11	8.39%	LVEKPSPLTLAPHDFANIK	95.0%	18.6	17.1	0	0	1	2	2,090.17
									NVTVQPDDPISFMQLTAK	95.0%	67.2	22.4	1	0	0	2	2,020.01
									TNNVSEHEDTDKYR	95.0%	48.3	21.5	0	4	0	2	1,707.76
									VLQDLVMDILR	95.0%	33.4	21.5	3	0	0	2	1,330.74
									YEAAGTLVTLSSAPTAIK	95.0%	87.7	20.9	2	0	0	2	1,792.97
Staphylococcal nuclease domain-containing protein 1	SND1_HUMAN	SND1	101,981	100.00%	10	11	36	16.80%	DTNGENIAESLVAEGLATR	95.0%	121.0	22.0	3	1	0	2	1,959.96
									DYVAPTANLDQK	95.0%	37.6	23.6	3	0	0	2	1,334.66
									EADGSETPEPFAAEAK	95.0%	104.0	19.0	5	0	0	2	1,648.73
									LGTLSPAFSTR	95.0%	39.0	21.8	2	0	0	2	1,149.63
									NDIASHPPVEGSYAPR	95.0%	49.6	21.6	0	2	0	2	1,709.82
									NLPGLVQEGEPFSEEATLFTK	95.0%	89.2	21.7	8	0	0	2	2,306.16
									QFLPFLQR	95.0%	36.2	20.2	5	0	0	2	1,048.59

									VITEYLNAQESAK	95.0%	71.0	21.8	2	0	0	2	1,465.75
									VMQVLNADAIVVK	95.0%	63.0	20.0	3	0	0	2	1,415.79
									VNVTVDYIRPASPATETVPAFSER	95.0%	70.1	21.1	0	2	0	2	2,619.34
Monocyte differentiation antigen CD14	CD14_HUMAN	CD14	40,059	100.00%	7	11	111	23.50%	AFPALTSLDLSDNPGLGER	95.0%	107.0	22.7	16	0	0	2	1,973.00
									ATVNPSAPR	95.0%	54.5	20.8	9	0	0	2	912.49
									FPAIQNLALR	95.0%	59.6	20.4	4	0	0	2	1,142.67
									ITGTMPPLPLEATGLALSSLR	95.0%	97.5	17.1	26	6	0	2	2,154.18
									LKELTLEDLK	95.0%	60.2	18.7	7	1	0	2	1,201.70
									LTVGAAQVPAQLLVGALR	95.0%	97.5	11.8	31	1	0	2	1,777.07
									RLTVGAAQVPAQLLVGALR	95.0%	45.7	10.4	2	8	0	2	1,933.17
Cadherin-5	CADH5_HUMAN	CDH5	87,499	99.50%	2	2	4	3.70%	KPLIGTVLAMDPDAAR	95.0%	65.9	20.4	0	2	0	2	1,683.91
									VDAETGDVFAIER	95.0%	54.2	22.9	2	0	0	2	1,421.69
Chloride intracellular channel protein 1	CLIC1_HUMAN	CLIC1	26,905	100.00%	13	14	100	54.40%	FLDGNELTLADCNLLPK	95.0%	85.5	22.1	5	0	0	2	1,932.97
									GFTIPEAFR	95.0%	40.8	22.3	1	0	0	2	1,037.54
									GVTFNVTTVDTK	95.0%	97.5	21.6	10	0	0	2	1,281.67
									GVTFNVTTVDTKR	95.0%	43.5	21.1	2	0	0	2	1,437.77
									IGNCPFSQR	95.0%	46.0	22.6	2	0	0	2	1,078.51
									KFLDGNELTLADCNLLPK	95.0%	82.0	21.7	2	1	0	2	2,061.07
									LAALNPESNTAGLDIFAK	95.0%	87.9	21.4	36	0	0	2	1,844.98
									LFMVLWLK	95.0%	49.6	18.3	7	0	0	2	1,065.62
									LHIVQVVCK	95.0%	42.4	17.9	2	0	0	2	1,095.64
									NSNPALNDNLEK	95.0%	66.4	21.8	18	0	0	2	1,328.64
									VLDNYLTSPLPEEVDETSAEDEGVSQR	95.0%	37.1	19.3	0	2	0	2	2,992.39
									VLDNYLTSPLPEEVDETSAEDEGVSQRK	95.0%	62.9	20.1	0	4	0	2	3,120.49
									YLSNAYAR	95.0%	43.7	20.3	8	0	0	2	957.48
Cystathionine gamma-lyase	CGL_HUMAN	CTH	44,491	100.00%	4	5	9	9.63%	DVLGISDTLIR	95.0%	41.5	21.8	2	0	0	2	1,201.68
									LLEAAITPETK	95.0%	52.8	23.5	2	0	0	2	1,185.67
									NDRDVLGISDTLIR	95.0%	40.1	22.0	1	2	0	2	1,586.85
									QGAPGQHSGFEYSR	95.0%	33.6	19.9	0	2	0	2	1,520.69
Basal cell adhesion molecule	BCAM_HUMAN	BCAM	67,386	100.00%	13	15	88	24.80%	AGAAGTAEATAR	95.0%	75.2	23.2	6	0	0	2	1,046.52
									EASGLLSLTSTLYLR	95.0%	86.8	19.2	2	0	0	2	1,623.90
									GDGSPSPEYTLFR	95.0%	61.1	22.3	7	0	0	2	1,425.67
									GRSPPYQLDSQGR	95.0%	28.3	22.8	0	1	0	2	1,460.72
									LASAEMQGSELQVTMHDTR	95.0%	44.9	20.2	1	6	0	2	2,135.97
									LEVPVEMNPEGYMTSR	95.0%	87.1	20.2	8	0	0	2	1,883.85
									LNVFAKPEATEVSPNK	95.0%	58.6	21.4	7	3	0	2	1,743.93
									LSVPPLVEVMR	95.0%	60.2	20.8	6	0	0	2	1,255.71
									SPPYQLDSQGR	95.0%	65.1	22.9	9	0	0	2	1,247.60
									TAEIEPK	95.0%	38.5	21.9	3	0	0	2	787.42
									VAYLDPLELSEGK	95.0%	62.0	22.5	17	0	0	2	1,433.75
									VEDYDAADDVQLSK	95.0%	120.0	20.7	11	0	0	2	1,567.71
									VTSALSR	95.0%	35.9	22.2	1	0	0	2	733.42
Guanine nucleotide-binding protein G(I)/G(S)/G(T) subunit beta-1	GBB1_HUMAN	GNB1	37,360	99.50%	2	2	3	6.18%	LIIWDSYTTNK	95.0%	34.6	21.7	1	0	0	2	1,353.71
									LLVSASQDGK	95.0%	37.5	22.3	2	0	0	2	1,017.56
Transgelin-2	TAGL2_HUMAN	TAGLN2	22,374	100.00%	11	13	61	63.30%	DDGLFSGDPNWFPK	95.0%	55.7	20.0	2	0	0	2	1,594.72
									DDGLFSGDPNWFPKK	95.0%	36.9	22.7	1	0	0	2	1,722.81
									DGTVLCELINALYPEGQAPVK	95.0%	79.6	20.5	2	0	0	2	2,287.16
									DGTVLCELINALYPEGQAPVKK	95.0%	55.6	21.1	1	6	0	2	2,415.26
									GASQAGMTGYGMPR	95.0%	72.2	18.2	13	0	0	2	1,415.60
									IQASTMAFK	95.0%	36.5	22.4	1	0	0	2	1,012.51
									NFSDNQLQEGK	95.0%	65.6	21.0	9	0	0	2	1,279.59

									NVIGLQMGTNR	95.0%	91.0	24.1	4	0	0	2	1,218.63
									QMEQISQFLQAAER	95.0%	100.0	22.2	8	2	0	2	1,694.82
									TLMNLGGLAVAR	95.0%	78.6	21.5	8	0	0	2	1,215.69
									YGINTTDIFQTVDLWEGK	95.0%	107.0	22.3	4	0	0	2	2,100.03
Exportin-T	XPOT_HUMAN	XPOT	109,949	100.00%	6	7	21	8.11%	ANVEAIMLAVMK	95.0%	70.6	22.5	2	0	0	2	1,321.69
									DLQEFIPLINQITAK	95.0%	78.0	18.6	5	2	0	2	1,742.97
									LAQVSPELLLASVR	95.0%	79.2	14.3	6	0	0	2	1,495.88
									NAQEALQAIETK	95.0%	79.8	23.0	2	0	0	2	1,315.69
									QMNPFIEDILNR	95.0%	54.2	23.0	2	0	0	2	1,505.74
									YSELTTVQQQLIR	95.0%	73.9	20.8	2	0	0	2	1,578.85
1,4-alpha-glucan-branching enzyme	GLGB_HUMAN	GBE1	80,445	100.00%	18	23	74	29.80%	AGLLFIFNFHPSK	95.0%	89.5	21.3	8	9	0	2	1,490.82
									CIAYAESHDQALVGDK	95.0%	28.6	21.8	0	2	0	2	1,776.82
									EFKDEDWNMGDIVYTLTNR	95.0%	42.2	18.6	0	2	0	2	2,362.07
									FLLSNIR	95.0%	40.8	19.6	1	0	0	2	862.52
									GYESFGVHR	95.0%	49.3	22.3	5	0	0	2	1,051.50
									HFTCNVLPR	95.0%	44.6	21.5	2	0	0	2	1,143.57
									IVLDSDAAEYGGHQR	95.0%	116.0	22.2	3	1	0	2	1,630.78
									IYESHVGISSHEGK	95.0%	80.4	21.7	2	3	0	2	1,542.76
									LAMAIPDKWIQLLK	95.0%	33.9	15.6	1	3	0	2	1,655.96
									LLEIDPYLKPYAVDFQR	95.0%	67.5	20.2	2	7	0	2	2,080.11
									NIGENEGGIDKFSR	95.0%	56.2	23.4	2	0	0	2	1,535.75
									QFHLTDDDLLR	95.0%	49.6	22.0	2	0	0	2	1,372.69
									RQFHLTDDDLLR	95.0%	35.7	21.8	0	3	0	2	1,528.79
									SGEILYR	95.0%	43.0	22.6	3	0	0	2	837.45
									VALILQNVDLPN	95.0%	88.2	17.9	6	0	0	2	1,308.75
									VGTALPGK	95.0%	46.2	18.5	4	0	0	2	742.45
									YGWLAAPQAYVSEK	95.0%	46.0	22.3	2	0	0	2	1,582.79
									YKQFSQILK	95.0%	31.1	20.0	1	0	0	2	1,154.66
Eukaryotic translation initiation factor 3 subunit L	EIF3L_HUMAN	EIF3L	66,711	100.00%	5	6	21	11.20%	LAGFLDLTEQEFR	95.0%	84.8	22.4	4	0	0	2	1,538.79
									LHSLLGDYYQAIK	95.0%	51.3	21.9	2	3	0	2	1,520.81
									MLGYFSLVGLLR	95.0%	73.0	20.9	5	0	0	2	1,384.77
									QYEQQTYQVIPEVIK	95.0%	62.7	21.9	3	0	0	2	1,865.97
									VSGGPSLEQR	95.0%	62.4	21.7	4	0	0	2	1,029.53
Exportin-2	XPO2_HUMAN	CSE1L	110,404	100.00%	25	31	121	32.20%	AADEEAFEDNSEEYIR	95.0%	85.8	15.8	2	0	0	2	1,887.79
									AADEEAFEDNSEEYIRR	95.0%	42.2	18.7	0	2	0	2	2,043.89
									ALTLPGSSENEYIMK	95.0%	83.8	22.0	5	0	0	2	1,668.82
									ANIVHLMLSSPEQIQK	95.0%	68.2	20.8	2	2	0	2	1,823.97
									ATIELCSTHANDASALR	95.0%	29.9	22.1	0	1	0	2	1,829.88
									DLEGSDIDTR	95.0%	53.0	20.8	3	0	0	2	1,120.51
									EHDPVGQMVNNPK	95.0%	53.9	21.4	5	4	0	2	1,480.69
									FLESVEGNQNYPLLLLTLLEK	95.0%	87.9	17.1	2	1	0	2	2,433.33
									FQSGDFHVINGVLR	95.0%	78.2	22.5	1	1	0	2	1,588.82
									GSNTIASAAADKIPGLLGVFQK	95.0%	85.4	17.2	3	11	0	2	2,158.19
									IIIPEIQK	94.6%	30.1	13.8	1	0	0	2	953.60
									ILFSSLILISK	95.0%	78.4	6.0	5	0	0	2	1,233.78
									LLQAFLER	95.0%	53.6	20.3	5	0	0	2	989.58
									LLQTDDEEEAGLLELLK	95.0%	132.0	21.1	21	2	0	2	1,929.01
									LLTECPPMMDTEYTK	95.0%	47.0	17.1	3	0	0	2	1,860.81
									LVLDAFALPLTNLFK	95.0%	60.4	13.2	5	0	0	2	1,674.98
									NLFEDQNTLTSICEK	95.0%	85.0	21.1	4	0	0	2	1,811.85
									QIFILLFQR	95.0%	56.9	14.5	4	0	0	2	1,177.71

									QLSDAISIIGR	95.0%	83.6	21.4	4	0	0	2	1,172.66
									SANVNEFPVLK	95.0%	45.9	23.0	2	0	0	2	1,217.65
									SQDNVIK	95.0%	32.2	25.0	2	0	0	2	803.43
									TGNIPALVR	95.0%	55.0	18.0	4	0	0	2	940.56
									VIVPNMEFR	95.0%	31.0	23.3	1	0	0	2	1,120.58
									YDEEFQR	95.0%	33.7	16.8	1	0	0	2	986.42
									YGALALQEIFDGIQPK	95.0%	88.3	20.7	12	0	0	2	1,762.94
RNA-binding protein 8A	RBM8A_HUMAN	RBM8A	19,871	99.50%	2	3	8	17.20%	GYTLVEYETYK	95.0%	54.2	22.7	2	0	0	2	1,365.66
									MREDYDSVEQDGDEPGPQR	95.0%	79.5	14.8	2	4	0	2	2,238.92
1,2-dihydroxy-3-keto-5-methylthiopentene dioxygenase	MTND_HUMAN	ADI1	21,481	100.00%	2	4	7	15.10%	GDMVTLPAGIYHR	95.0%	34.6	22.5	2	2	0	2	1,445.72
									LDADKYENDPELEK	95.0%	60.6	22.4	2	1	0	2	1,678.78
Exportin-7	XPO7_HUMAN	XPO7	123,895	100.00%	10	10	16	13.80%	AALSGSYVNFGVFR	95.0%	63.5	22.5	2	0	0	2	1,487.76
									DGLEDPLEDTGLVQQQLDQLSTIGR	95.0%	35.2	21.6	0	1	0	2	2,740.36
									DSSLFDIFTLSCNLLK	95.0%	52.9	21.8	1	0	0	2	1,872.94
									LLLSIPHSDLLDYPK	95.0%	30.7	17.7	0	1	0	2	1,723.96
									LQFDVSSPNGILLFR	95.0%	65.6	19.1	2	0	0	2	1,705.93
									LYGDDALDNALQTFIK	95.0%	90.5	22.1	3	0	0	2	1,796.91
									NSIVNSQPPEK	95.0%	40.3	21.8	2	0	0	2	1,212.62
									NYVLNYLATRPK	95.0%	47.5	18.4	1	0	0	2	1,451.80
									TLQLLNDLSIGYSSVR	95.0%	55.0	18.8	1	0	0	2	1,778.97
									TNNPLPLEQR	95.0%	44.0	22.6	2	0	0	2	1,181.63
Coatomer subunit gamma	COPG_HUMAN	COPG	97,701	100.00%	5	5	21	8.24%	FGAQNEEMLPSILVLLK	95.0%	57.9	20.4	3	0	0	2	1,918.04
									LLLLDTVTMQVTAR	95.0%	34.9	18.1	1	0	0	2	1,589.89
									SIATLAITTLLK	95.0%	81.8	11.1	12	0	0	2	1,244.78
									SSPEPVALTESETEYVIR	95.0%	109.0	22.6	2	0	0	2	2,006.99
									TLEEAVGNIVK	95.0%	52.5	22.4	3	0	0	2	1,172.65
N-acetyllactosaminide beta-1,3-N-acetylglucosaminyltransferase	B3GN1_HUMAN	B3GNT1	47,101	100.00%	5	5	20	12.30%	EPGEFALLR	95.0%	38.6	22.7	8	0	0	2	1,031.55
									TALASGGVLDASGDYR	95.0%	93.8	22.2	8	0	0	2	1,552.76
									VAMHLVCPSR	95.0%	27.4	22.5	0	1	0	2	1,185.59
									VPTFDER	95.0%	34.8	24.0	1	0	0	2	863.43
									YEAAVPDPR	95.0%	36.7	22.4	2	0	0	2	1,017.50
Polypeptide N-acetylgalactosaminyltransferase 1	GALT1_HUMAN	GALNT1	64,202	99.50%	2	2	4	5.01%	ATPYTFPGGTGQIINK	95.0%	55.2	22.8	2	0	0	2	1,664.87
									NFFYIISPGVTK	95.0%	69.6	21.2	2	0	0	2	1,385.75
AP-1 complex subunit beta-1	AP1B1_HUMAN	AP1B1	104,621	100.00%	4	4	5	14.10%	DIPNENEAQFQIR	95.0%	42.9	22.0	2	0	0	2	1,573.76
									KLAPPLVTLLSAEPELQYVALR	95.0%	37.5	11.1	0	1	0	2	2,421.41
									KPTETQELVQQVLSLATQDSDNPDLR	95.0%	39.9	21.2	0	1	0	2	2,925.48
									LAPPLVTLLSAEPELQYVALR	95.0%	31.9	11.1	0	1	0	2	2,293.32
									LASQANIAQVLAELK	95.0%	98.8	17.7	17	1	0	2	1,568.90
									LSHANSAVVLSAVK	95.0%	47.0	18.2	2	0	0	2	1,395.80
									LVYLYLMNYAK	95.0%	63.4	23.1	3	0	0	2	1,406.74
									MEPLNNLQVAVK	95.0%	55.9	22.1	8	0	0	2	1,371.73
									NVEGQDMLYQSLK	95.0%	69.1	22.6	8	0	0	2	1,540.73
									YNDPIYVK	95.0%	46.8	21.6	2	0	0	2	1,011.52
Ubiquitin carboxyl-terminal hydrolase isozyme L3	UCHL3_HUMAN	UCHL3	26,165	100.00%	4	4	6	24.30%	FLEESVSMSPEER	95.0%	49.4	20.4	1	0	0	2	1,555.70
									SQGQDVTSSVYFMK	95.0%	63.2	20.2	2	0	0	2	1,592.73
									VTHETSAHEGQTEAPSIDEK	95.0%	50.2	21.0	0	2	0	2	2,166.00
									YLENYDAIR	95.0%	51.2	21.6	1	0	0	2	1,156.56
U3 small nucleolar RNA-associated protein 15 homolog	UTP15_HUMAN	UTP15	58,399	99.50%	2	2	3	6.56%	LNPDLFITGSYDHTVK	95.0%	66.9	22.2	1	0	0	2	1,819.92
									VDFSPQPPYNYAVTASSR	95.0%	42.4	22.1	2	0	0	2	1,998.96
Putative tropomyosin alpha-3 chain-like protein	TPM3L_HUMAN	---	26,252	100.00%	4	5	13	21.50%	AELAESR	95.0%	39.2	24.2	2	0	0	2	775.40
									HIAEEADR	95.0%	40.4	22.0	5	0	0	2	940.45

									IQVLQQQADDAEER	95.0%	103.0	22.1	2	0	0	2	1,642.80
									IQVLQQQADDAEERAER	95.0%	57.9	21.9	2	6	0	2	1,998.98
									LMDQNLK	95.0%	36.4	23.8	1	0	0	2	877.45
									MELQEIQLK	95.0%	56.4	23.8	5	0	0	2	1,131.61
Ribosome maturation protein SBDS	SBDS_HUMAN	SBDS	28,746	100.00%	3	3	4	16.00%	GEVQVSDKER	95.0%	34.2	24.1	1	0	0	2	1,146.58
									LTNVAVVR	95.0%	30.7	21.0	1	0	0	2	871.54
									SGVEKDLDEVLQTHSVFVNVSK	95.0%	61.6	21.0	0	2	0	2	2,430.25
Heat shock protein HSP 90-alpha	HS90A_HUMAN	HSP90AA1	84,645	100.00%	26	36	753	57.10%	ADLINNLGTIAK	95.0%	72.6	20.9	155	0	0	2	1,242.71
									ALLFVPR	95.0%	49.5	18.9	54	0	0	2	815.51
									APFDLFENR	95.0%	64.2	22.4	27	0	0	2	1,108.54
									APFDLFENRK	95.0%	41.6	22.7	2	0	0	2	1,236.64
									DNSTMGYMAAK	95.0%	42.3	13.6	3	0	0	2	1,220.49
									DQVANSAFVER	95.0%	79.0	22.9	85	0	0	2	1,235.60
									EDQTEYLEER	95.0%	54.4	18.8	36	0	0	2	1,311.57
									EGLELPEDEEEK	95.0%	49.5	19.9	8	0	0	2	1,416.64
									EGLELPEDEEEKK	95.0%	47.7	21.2	7	0	0	2	1,544.73
									EKYIDQEELNK	95.0%	54.8	23.7	11	1	0	2	1,408.70
									ELHINLIPNKQDR	95.0%	58.6	20.2	10	8	2	2	1,589.88
									ELISNSSDALDK	95.0%	83.1	23.2	37	0	0	2	1,291.64
									ELISNSSDALDKIR	95.0%	103.0	22.4	35	4	0	2	1,560.82
									EMLQQSK	95.0%	46.5	23.4	7	0	0	2	879.42
									ESEDKPEIEDVGSDEEEEKK	95.0%	34.9	18.1	0	1	0	2	2,321.02
									FYEQFSK	95.0%	32.0	21.3	1	0	0	2	948.45
									GVVDSEDLPLNISR	95.0%	117.0	22.4	116	0	0	2	1,513.79
									HFSVEGQLEFR	95.0%	85.9	23.7	175	150	0	2	1,348.67
									HGLEVIYMIEPIDEYCVQQLK	95.0%	49.6	21.0	0	5	0	2	2,593.27
									HIYYITGETK	95.0%	52.8	22.1	21	0	0	2	1,224.63
									HLEINPDHSIIETLR	95.0%	89.7	21.9	11	43	1	2	1,786.95
									HNDDEQYAWESSAGGSFTVR	95.0%	28.5	17.6	0	3	0	2	2,255.96
									HSQFIGYPITLFVEK	95.0%	85.5	20.8	70	23	0	2	1,778.95
									HSQFIGYPITLFVEKER	95.0%	73.8	20.1	0	14	0	2	2,064.09
									IRYESLTDPSK	95.0%	47.4	22.2	3	6	0	2	1,308.68
									KHLEINPDHSIIETLR	95.0%	36.0	18.9	0	5	27	2	1,915.04
									KHSQFIGYPITLFVEK	95.0%	44.0	18.5	0	17	2	2	1,907.04
									LGIHEDSQNR	95.0%	52.2	23.1	22	21	0	2	1,168.57
									LGIHEDSQNRK	95.0%	32.4	22.0	0	2	0	2	1,296.67
									LGLGIDEDDPTADDTSAAVTEEMPPLEGDDDTSR	95.0%	38.6	15.4	0	1	0	2	3,563.54
									LSELLR	95.0%	42.5	23.4	35	0	0	2	730.45
									NPDDITNEEYGEFYK	95.0%	98.4	17.3	70	0	0	2	1,833.78
									RAPFDLFENR	95.0%	54.2	23.3	39	7	0	2	1,264.64
									SLTNDWEDHLAVK	95.0%	83.3	22.0	4	2	0	2	1,527.74
									TDTGEPMGR	95.0%	44.3	17.0	8	0	0	2	979.42
									TLTIVDTGIGMTK	95.0%	117.0	22.3	121	0	0	2	1,365.73
									TLVSVTK	95.0%	30.5	19.6	1	0	0	2	747.46
									VFIMDNCEELIPEYLNFIR	95.0%	50.0	21.9	3	0	0	2	2,431.17
									VILHLK	95.0%	39.2	7.8	6	0	0	2	722.49
									VILHLKEDQTEYLEER	95.0%	75.7	21.9	52	216	1	2	2,015.05
									YESLTDPSK	95.0%	49.8	22.6	19	0	0	2	1,039.49
									YESLTDPSKLDSGK	95.0%	77.4	22.6	40	2	0	2	1,539.75
									YIDQEELNK	95.0%	60.7	23.2	72	0	0	2	1,151.56
									YYTSASGDEMVSLK	95.0%	87.0	19.8	71	0	0	2	1,566.70

T-complex protein 1 subunit zeta	TCPZ_HUMAN	CCT6A	58,007	100.00%	13	15	54	31.80%	AGMSSLKG	95.0%	49.4	22.0	2	0	0	2	766.38
									ALQFLEEVK	95.0%	58.9	21.7	5	0	0	2	1,076.60
									DGNVLLHEMQIQHPTASLIAK	95.0%	40.8	20.5	0	2	0	2	2,331.21
									GIDPFSLDALSK	95.0%	64.6	22.9	12	0	0	2	1,262.66
									GLQDVLR	95.0%	31.2	21.7	2	0	0	2	800.46
									IITEGFEAAK	95.0%	55.3	21.8	4	0	0	2	1,078.58
									KQDEPIDLFMIEIMEMK	95.0%	49.7	21.9	1	2	0	2	2,158.01
									QADLYISEGLHPR	95.0%	26.6	21.6	0	1	0	2	1,498.77
									QDEPIDLFMIEIMEMK	95.0%	65.4	20.7	1	0	0	2	2,029.92
									TEVNSGFFYK	95.0%	36.1	22.6	2	0	0	2	1,191.57
									VATAQDDITGDGTTSNVLIIGELLK	95.0%	122.0	18.6	7	4	0	2	2,544.34
									VHAELADVLTEAVVDSILAIK	95.0%	30.8	15.9	0	2	0	2	2,206.23
									VLAQNSGFDLQETLVK	95.0%	84.8	21.4	7	0	0	2	1,761.94
Cellular nucleic acid-binding protein	CNBP_HUMAN	CNBP	19,444	100.00%	3	3	6	18.60%	CGESGHLAK	95.0%	42.5	18.6	2	0	0	2	958.44
									CGESGHLAR	95.0%	42.1	17.8	2	0	0	2	986.45
									GFQFVSSSLPDICYR	95.0%	52.1	22.3	2	0	0	2	1,775.84
Plastin-3	PLST_HUMAN	PLS3	70,796	100.00%	8	9	16	19.50%	AESMLQQADK	95.0%	63.0	22.3	2	0	0	2	1,136.53
									AYFHLLNQIAPK	95.0%	42.3	21.0	0	2	0	2	1,414.78
									HVIPMNPNTDDLFK	95.0%	34.5	21.9	1	0	0	2	1,656.81
									LSPEELLLR	95.0%	81.2	18.9	2	0	0	2	1,069.63
									MINLSVPDTIDER	95.0%	66.0	22.9	4	0	0	2	1,518.75
									NEALAALLR	95.0%	58.5	18.2	2	0	0	2	970.57
									QFVTPADVVSGNPK	95.0%	67.8	22.9	2	0	0	2	1,458.76
									SGNLTEDDKHNNAK	95.0%	42.3	20.9	0	2	0	2	1,542.72
									VYALPEDLVEVKPK	95.0%	56.2	18.9	2	1	0	2	1,599.90
									YTLNVLEDLGDGQK	95.0%	91.5	22.3	2	0	0	2	1,564.79
Putative phospholipase B-like 1	PLBL1_HUMAN	PLBD1	63,240	100.00%	6	8	20	15.20%	GTLYIVEQIPTYVEYSEQTDVLR	95.0%	47.3	21.1	1	1	0	2	2,716.37
									GTLYIVEQIPTYVEYSEQTDVLRK	95.0%	27.2	21.0	0	1	0	2	2,844.47
									LGLDYSYDLAPR	95.0%	93.8	23.0	7	0	0	2	1,382.70
									TLHQGMPEVYNFDFITMKPILK	95.0%	35.8	21.0	0	4	4	2	2,654.34
									TTGWGILEIR	95.0%	54.5	22.6	1	0	0	2	1,145.63
									YNSGTYNNQYMVLDLK	95.0%	65.5	20.3	1	0	0	2	1,938.89
ATP synthase subunit beta, mitochondrial	ATPB_HUMAN	ATP5B	56,543	100.00%	14	15	50	38.40%	ADKLAEEHSS	95.0%	38.7	20.0	1	0	0	2	1,086.51
									AIAELGIYPAVDPLDSTSR	95.0%	70.1	21.7	4	0	0	2	1,988.03
									DQEGQDVLLFIDNIFR	95.0%	51.5	21.8	1	0	0	2	1,921.97
									FLSQPFQVAEVFTGHMGK	95.0%	32.2	21.4	0	2	0	2	2,039.01
									FTQAGSEVSALLGR	95.0%	110.0	22.3	4	0	0	2	1,435.75
									IGLFGGAGVGK	95.0%	51.3	20.5	4	0	0	2	975.56
									IMNVIGEPIDER	95.0%	53.3	22.5	5	0	0	2	1,401.71
									IPSAVGYQPTLATDMGTMQER	95.0%	54.4	21.5	1	0	0	2	2,298.08
									TIAMDGTEGLVR	95.0%	51.1	22.9	5	0	0	2	1,278.64
									TVLIMELINNVAK	95.0%	91.8	19.5	8	0	0	2	1,473.84
									VALTGLTVAEYFR	95.0%	77.4	19.9	4	0	0	2	1,439.79
									VALVYGQMNEPPGAR	95.0%	55.5	23.0	4	0	0	2	1,617.81
									VLDSGAPIKIPVGPETLGR	95.0%	57.0	12.6	2	4	0	2	1,919.10
									VVDLLAPYAK	95.0%	42.2	19.3	1	0	0	2	1,088.64
Creatine kinase U-type, mitochondrial	KCRU_HUMAN	CKMT1A	47,020	100.00%	5	5	9	23.50%	GTGGVDTAATGGVFDISNLDR	95.0%	123.0	22.2	2	0	0	2	2,022.97
									LSEMTEAEQQQLIDDHFLFDKPVSPLLTAAGMAR	95.0%	28.2	20.0	0	0	2	2	3,833.87
									SGYFDER	95.0%	31.6	14.9	1	0	0	2	873.37
									TVGMVAGDEETYEVFADLFDPVIQER	95.0%	46.6	20.3	0	2	0	2	2,946.37
									VVVDALSGLK	95.0%	68.2	19.3	2	0	0	2	1,000.60

Xylosyltransferase 2	XYLT2_HUMAN	XYLT2	96,750	99.50%	2	2	14	3.24%	NFGGLLGPLDEPVAVQR	95.0%	72.1	20.3	10	0	0	2	1,781.96
									TNEELVAFLSK	95.0%	46.2	22.4	4	0	0	2	1,250.66
Nodal modulator 1	NOMO1_HUMAN	NOMO1	134,308	100.00%	7	7	16	7.86%	DGENYVVLLDSTLPR	95.0%	68.2	22.2	2	0	0	2	1,690.87
									EGYVLTAVEGTIGDFK	95.0%	53.7	21.8	2	0	0	2	1,698.86
									FLLFSSLVTK	95.0%	42.7	17.4	1	0	0	2	1,154.68
									GQPLGPAGVQVSLR	95.0%	44.5	18.9	1	0	0	2	1,378.78
									SSIDSEPALVLGPLK	95.0%	74.8	20.6	4	0	0	2	1,525.85
									VNAMTFTFDNVLPGK	95.0%	81.7	22.7	2	0	0	2	1,669.83
									VQVMVPEAETR	95.0%	44.1	22.9	4	0	0	2	1,274.64
Lysosome-associated membrane glycoprotein 1	LAMP1_HUMAN	LAMP1	44,865	99.50%	2	2	13	6.47%	ALQATVGNSYK	95.0%	35.9	23.2	1	0	0	2	1,151.61
									FFLQGIQLNTILPDAR	95.0%	73.1	17.9	12	0	0	2	1,846.02
Cathepsin O	CATO_HUMAN	CTSO	35,940	99.50%	2	2	5	8.10%	GYSAYDFSDQEDEMAK	95.0%	115.0	11.1	4	0	0	2	1,871.73
									TGSTPYWIVR	95.0%	32.8	22.3	1	0	0	2	1,179.62
Heme-binding protein 1	HEBP1_HUMAN	HEBP1	21,079	100.00%	2	2	6	16.40%	FATVEVTDKPVDEALR	95.0%	44.6	21.8	0	2	0	2	1,789.93
									IPNQFQSDPPAPSDK	95.0%	67.8	22.6	4	0	0	2	1,640.79
26S protease regulatory subunit 8	PRS8_HUMAN	PSMC5	45,609	100.00%	7	7	15	23.90%	EVIELPVKHPELFEALGIAQPK	95.0%	28.9	14.6	0	1	0	2	2,457.38
									GVLLYGPPGTGK	95.0%	38.2	22.0	2	0	0	2	1,158.65
									IAELMPGASGAEVK	95.0%	57.5	23.6	2	0	0	2	1,388.71
									IDILDSALLRPGR	95.0%	31.0	16.0	1	0	0	2	1,438.84
									LEGGSGGDSEVQR	95.0%	70.1	19.8	6	0	0	2	1,290.59
									VPDSTYEMIGGLDK	95.0%	93.7	22.2	2	0	0	2	1,540.72
									VSGSELVQK	95.0%	33.0	22.9	1	0	0	2	946.52
Uroporphyrinogen decarboxylase	DCUP_HUMAN	UROD	40,769	100.00%	5	6	11	16.60%	EAGLAPVPMIIFAK	95.0%	43.4	20.4	3	0	0	2	1,472.82
									FALPYIR	95.0%	38.7	19.7	3	0	0	2	879.51
									GPSFPEPLREEQDLER	95.0%	39.2	22.0	1	0	0	2	1,898.93
									LRDPEVVASELGYVFQAITLTR	95.0%	28.3	16.6	0	1	0	2	2,477.34
									LREAGLAPVPMIIFAK	95.0%	56.2	14.3	2	1	0	2	1,742.00
Basement membrane-specific heparan sulfate proteoglycan core protein	PGBM_HUMAN	HSPG2	468,761	100.00%	90	103	813	27.80%	AASGPGPEQEASFTVTVPPSEGSSYR	95.0%	73.4	21.2	2	0	0	2	2,608.22
									AELLVTEAPSKPITVTVEEQR	95.0%	67.9	17.4	0	23	0	2	2,310.26
									AFAHLQVPER	95.0%	59.6	21.5	10	3	0	2	1,167.63
									AGFFGDAMK	95.0%	51.0	17.1	5	0	0	2	959.43
									AGLSSGFIGCVR	95.0%	69.4	23.9	4	0	0	2	1,223.62
									AHSSAGQQVAR	95.0%	45.2	21.0	1	0	0	2	1,111.56
									AMDFNGILTIR	95.0%	62.4	22.6	24	0	0	2	1,266.65
									AQASAQLVIQALPSVLINIR	95.0%	29.2	6.0	0	1	0	2	2,105.24
									AQIHGGILR	95.0%	38.9	15.2	2	0	0	2	964.57
									ASYAQQPAESR	95.0%	72.9	22.1	54	0	0	2	1,207.57
									ATATSCRPCPCPYIDASR	95.0%	47.5	19.6	0	2	0	2	2,082.92
									AVLHVHGGGGPR	95.0%	47.6	21.8	0	5	0	2	1,156.63
									AVTLECVSAGEPR	95.0%	101.0	22.2	6	0	0	2	1,388.68
									CESCAPGYEGNPIQPGGK	95.0%	89.3	17.2	4	0	0	2	1,920.82
									CFCMGVSR	95.0%	44.1	14.1	4	0	0	2	1,032.41
									CRPVNQEIVR	95.0%	27.5	21.6	0	2	0	2	1,270.67
									EDGRPVPSGTQQR	95.0%	32.4	21.8	1	3	0	2	1,426.70
									EFREVSEAVVDTLESEYLK	95.0%	44.6	21.1	0	1	0	2	2,243.11
									EGGQLPPGHSVQDGVLR	95.0%	38.0	23.2	0	7	0	2	1,745.89
									EGGSLPPQAR	95.0%	36.4	22.1	5	0	0	2	1,011.52
									EHLLMALADLDELLIR	95.0%	27.2	20.0	0	2	0	2	1,881.02
									EHLLMALAGIDTLLIR	95.0%	91.2	16.2	4	12	0	2	1,795.02
									EQAWQRPDGQPATR	95.0%	43.7	22.2	1	0	0	2	1,639.79
									ESDQGAYTCEAMNAR	95.0%	54.5	12.0	1	0	0	2	1,718.68

ESIQASR	95.0%	55.6	23.3	2	0	0	2	790.41
ETSLIVTIQGSGSSHVPSVSPPIR	95.0%	36.5	18.1	0	6	0	2	2,448.31
EVSEAVVDTLESEYLK	95.0%	120.0	22.4	37	0	0	2	1,810.90
FDAGSGMATIR	95.0%	84.9	21.0	26	0	0	2	1,141.53
FLGDKVTSYGGELR	95.0%	89.5	21.8	6	0	0	2	1,541.80
FSSGITGCVK	95.0%	38.5	22.2	2	0	0	2	1,055.52
GGSLPSHHQTR	95.0%	43.1	22.7	2	0	0	2	1,176.59
GHTPTQPGALNQR	95.0%	47.8	23.1	0	17	0	2	1,376.70
GMLEPVQRPDVVLVGAGYR	95.0%	67.1	21.1	1	18	0	2	2,072.10
GMVFGIPDGVLELVPQR	95.0%	77.9	20.9	25	1	0	2	1,842.98
GSIQVDGEELVSGR	95.0%	71.8	22.5	6	0	0	2	1,445.72
GSVYIGGAPDVATLTGGR	95.0%	107.0	21.9	10	0	0	2	1,690.88
HLISTHFAPGDFQGFALVNPQR	95.0%	41.7	20.5	0	3	0	2	2,452.25
HPTPLALGHFHTVTLLR	95.0%	34.4	17.1	0	0	3	2	1,910.08
HQIVGSR	95.0%	36.4	19.9	5	0	0	2	796.44
HQTHGSLLR	95.0%	44.8	23.1	0	25	0	2	1,048.57
HQVHGPLLR	95.0%	29.9	19.8	0	3	0	2	1,056.61
IAHVELADAGQYR	95.0%	70.3	22.6	4	13	0	2	1,442.74
IESSSPTVVEGQTLDLNCVVAR	95.0%	150.0	21.2	5	3	0	2	2,374.19
IPGDQVVSVVFIK	95.0%	69.1	17.4	27	0	0	2	1,400.82
IQVVVLSASDASPPPVK	95.0%	84.0	16.4	10	6	0	2	1,706.97
ITFRPDSADGMLLYNGQK	95.0%	42.8	21.7	0	5	0	2	2,042.00
LDGSLPPDSR	95.0%	45.0	22.1	13	0	0	2	1,056.53
LDVEFKPLAPDGVLLFSGGK	95.0%	51.0	16.9	1	13	0	2	2,102.15
LEGDTLIIPR	95.0%	84.4	20.1	15	0	0	2	1,126.65
LGTVPQFPR	95.0%	51.5	22.3	13	0	0	2	1,014.57
LHHVSPADSGEYVCR	95.0%	47.3	21.8	0	2	0	2	1,726.80
LHLVSPADSGEYVCR	95.0%	26.1	21.7	0	1	0	2	1,702.82
LLQVTPADSGEYVCR	95.0%	94.2	21.6	6	0	0	2	1,707.84
LLSGPYFWSLPSR	95.0%	47.4	21.5	6	0	0	2	1,522.81
LPAVEPTDQAQYLCR	95.0%	101.0	22.2	7	0	0	2	1,760.86
LRFDQPDDFK	95.0%	30.9	22.5	1	0	0	2	1,280.63
LRLPQVSPADSGEYVCR	95.0%	44.3	22.6	0	1	0	2	1,946.98
LRSPVISIDPPSSTVQQGQDASFK	95.0%	87.3	20.6	0	6	0	2	2,557.33
LSGSHSQGVAYPVR	95.0%	69.7	22.1	14	4	0	2	1,457.75
LVSEDPINDGEWHR	95.0%	66.4	21.9	2	0	0	2	1,666.78
LYIFQASPADAGQYVCR	95.0%	115.0	21.9	8	0	0	2	1,958.94
LYQASPADSGEYVCR	95.0%	90.2	19.8	5	0	0	2	1,715.77
MASVGLSDIAMDTTVTHATSHGR	95.0%	59.8	20.7	0	8	15	2	2,390.11
NELLHFER	95.0%	40.4	21.2	11	0	0	2	1,057.54
NLVLHSARPGAPPPQPLDLQHR	95.0%	39.5	17.9	0	2	0	2	2,413.32
QPDFISFGLVGGRPEFR	95.0%	31.7	21.6	0	6	0	2	1,921.99
RCESCAPGYEGNPIQPGGK	95.0%	41.9	18.2	0	3	0	2	2,076.92
RLGTVPQFPR	95.0%	32.6	19.8	2	0	0	2	1,170.67
RVPGSPTNLANR	95.0%	27.0	20.4	0	1	0	2	1,281.70
SGPVEDFVSLAMVGGHLEFR	95.0%	27.9	21.9	0	1	0	2	2,163.05
SIEYSPQLEDAGSR	95.0%	95.6	21.8	18	0	0	2	1,551.73
SIVPQGGSHSLR	95.0%	26.7	21.4	0	1	0	2	1,237.67
SLPEVPETIELEVR	95.0%	79.2	20.7	7	0	0	2	1,610.86
SPAYTLVWTR	95.0%	83.1	21.8	4	0	0	2	1,193.63
SPGPNVAVNAK	95.0%	76.8	21.2	12	0	0	2	1,053.57

									SPVISIDPPSSTVQQGQDASFK	95.0%	119.0	22.8	9	4	0	2	2,288.14
									SQSVRPGADVTFICTAK	95.0%	38.8	22.8	0	3	0	2	1,836.93
									SYEIMFR	95.0%	37.3	20.0	4	0	0	2	961.45
									TSTASGLLLWQGVEVGEAGQGK	95.0%	91.4	21.1	1	0	0	2	2,188.13
									VAEGQTLDLK	95.0%	65.4	23.4	7	0	0	2	1,073.58
									VDSYGGSLR	95.0%	55.3	22.1	11	0	0	2	953.47
									VGGHLRPGIVQSGGVVR	95.0%	75.3	15.4	0	13	0	2	1,687.97
									VGSSLPGR	95.0%	55.7	22.7	23	0	0	2	772.43
									VISSGSVASYVTSPQGFQFR	95.0%	110.0	21.7	19	3	0	2	2,117.07
									VTSYGGELR	95.0%	41.7	21.8	13	0	0	2	981.50
									VTVTSEGGR	95.0%	59.0	24.2	11	0	0	2	905.47
									VVGSSGTQEASVLVTIQQR	95.0%	76.5	19.8	7	0	0	2	1,959.05
									VVPYFTQTPYSFLPLPTIK	95.0%	41.4	16.4	2	0	0	2	2,211.21
									YELGSGLAVLR	95.0%	93.8	20.8	13	0	0	2	1,177.66
									YQLGSGEAR	95.0%	32.6	23.1	1	0	0	2	980.48
Plasma alpha-L-fucosidase	FUCO2_HUMAN	FUCA2	54,050	100.00%	13	14	89	30.00%	AILGATEVK	95.0%	51.7	20.6	4	0	0	2	901.54
									DIVKELEVAIR	95.0%	53.5	18.3	3	0	0	2	1,284.75
									DNYPPSFKYEDFGPLFTAK	95.0%	28.3	21.6	0	2	0	2	2,236.06
									EAGISDYLTIEELVK	95.0%	83.3	22.3	34	0	0	2	1,679.87
									FDPTWESLDAR	95.0%	78.1	21.0	3	0	0	2	1,336.62
									FFNANQWADIFQASGAK	95.0%	109.0	21.8	8	0	0	2	1,914.91
									GTVVTNDR	95.0%	35.1	24.1	1	0	0	2	861.44
									LVYAIFLK	95.0%	50.0	13.6	5	0	0	2	966.60
									VNGEAIYETHTWR	95.0%	33.8	22.4	0	2	0	2	1,575.76
									WPTSGQLFLGHPK	95.0%	72.9	21.1	2	4	0	2	1,467.78
									YEDFGPLFTAK	95.0%	68.8	22.1	18	0	0	2	1,287.63
									YNPGHLLPHK	95.0%	38.2	22.7	2	0	0	2	1,175.63
									YVEFMKDNYPPSFK	95.0%	53.7	21.5	1	0	0	2	1,780.83
Quinone oxidoreductase PIG3	QORX_HUMAN	TP53I3	35,519	100.00%	5	5	22	20.50%	EVAKPSPGEGEVLLK	95.0%	64.8	18.5	4	0	0	2	1,552.86
									GSLITSLLR	95.0%	71.5	17.3	6	0	0	2	959.59
									MAGAIPLVTAGSQK	95.0%	56.5	22.7	6	0	0	2	1,359.73
									QMLVNAFTEQILPHFSTEGPQR	95.0%	52.0	21.3	0	4	0	2	2,559.27
									VAASALNR	95.0%	56.1	24.7	2	0	0	2	801.46
Carnitine O-acetyltransferase	CACP_HUMAN	CRAT	70,841	100.00%	3	3	6	7.51%	LPVPPLQQSLDHYLK	95.0%	42.3	17.2	0	3	0	2	1,747.97
									LSPDAFIQMALQLAYYR	95.0%	71.7	22.4	2	0	0	2	2,016.03
									QPVVIYSSPGVMLPK	95.0%	40.3	21.0	1	0	0	2	1,630.89
T-complex protein 1 subunit delta	TCPD_HUMAN	CCT4	57,908	100.00%	13	15	75	31.00%	ALIAGGGAPEIELALR	95.0%	79.6	17.6	18	0	0	2	1,550.89
									AYILNLVK	95.0%	42.1	13.4	4	0	0	2	933.58
									DALSDLALHFLNK	95.0%	92.8	21.8	20	3	0	2	1,456.78
									ETLLNSATTSLNSK	95.0%	71.6	21.8	2	0	0	2	1,478.77
									GIHPTIISESFQK	95.0%	31.2	21.9	1	0	0	2	1,456.78
									IDDVVNTR	95.0%	43.6	23.7	2	0	0	2	931.49
									IGLIQFCLSAPK	95.0%	54.4	20.6	2	0	0	2	1,346.75
									LTEYSR	95.0%	38.7	18.8	1	0	0	2	768.39
									LVIEEAER	95.0%	31.3	21.2	1	0	0	2	958.52
									MIQDGKGDVTITNDGATILK	95.0%	48.7	21.5	0	2	0	2	2,106.08
									TDMDNQIVVSDYAQMDR	95.0%	96.8	18.0	4	0	0	2	2,032.86
									VIDPATATSVDLR	95.0%	78.1	22.2	11	0	0	2	1,357.73
									VVSQYSSLLSPMSVNAVMK	95.0%	98.6	21.8	2	2	0	2	2,072.04
Ubiquitin-conjugating enzyme E2 K	UBE2K_HUMAN	UBE2K	22,389	100.00%	4	4	7	24.00%	GEIAGPPDTPYEGGR	95.0%	50.5	21.2	1	0	0	2	1,515.71
									IPETYPFNPPK	95.0%	51.8	23.0	2	0	0	2	1,302.67

									NAVIVALSSK	95.0%	45.0	18.5	2	0	0	2	1,001.60
									VDLVDENFTELR	95.0%	38.7	22.9	2	0	0	2	1,449.72
Ephrin type-A receptor 4	EPHA4_HUMAN	EPHA4	109,843	100.00%	8	9	83	13.80%	FTVIQLVGMLR	95.0%	43.2	18.8	3	0	0	2	1,292.74
									GLNPLTSYVFHVR	95.0%	83.5	22.5	13	8	0	2	1,502.81
									IDTIAADESFTQVDIGDR	95.0%	103.0	22.0	2	0	0	2	1,965.94
									NLAQFPDTITGADTSSLVEVR	95.0%	132.0	21.2	21	0	0	2	2,234.13
									TAAGYGDFSEPLEVTTNTVPSR	95.0%	114.0	21.0	8	0	0	2	2,312.10
									VYPANEVTLLDSR	95.0%	75.8	22.7	22	0	0	2	1,476.77
									YNPNPDQSVSVTVTTNQAAPSSIALVQAK	95.0%	43.5	19.5	0	4	0	2	3,000.53
									YYEKDQNER	95.0%	44.5	19.3	2	0	0	2	1,244.55
Serine/threonine-protein phosphatase 2A catalytic subunit beta isoform	PP2AB_HUMAN	PPP2CB	35,557	100.00%	4	4	14	19.10%	ESNVQEVR	95.0%	44.7	22.5	5	0	0	2	960.48
									GAGYTFGQDISETFNHANGLTLVSR	95.0%	28.1	21.2	0	1	0	2	2,655.28
									SPDTNYLFMGDYVDR	95.0%	92.5	21.0	6	0	0	2	1,808.78
									YSFLQFDPAPR	95.0%	67.3	22.1	2	0	0	2	1,340.66
Neutral alpha-glucosidase AB	GANAB_HUMAN	GANAB	106,858	100.00%	14	18	78	18.00%	DENSVELTMAEGPYK	95.0%	83.2	19.7	2	0	0	2	1,698.75
									DPAEGDGAQPEETPR	95.0%	42.8	19.6	1	0	0	2	1,568.68
									FRIDELEPR	95.0%	32.1	23.4	2	0	0	2	1,174.62
									KLVAIVDPHIK	95.0%	34.6	8.5	0	2	0	2	1,232.77
									LDLLEDR	95.0%	39.7	22.3	2	0	0	2	873.47
									LKVTEGGEPYR	95.0%	37.6	22.9	1	1	0	2	1,248.66
									LSFQHDPETSVLVLR	95.0%	55.5	20.7	2	10	0	2	1,740.93
									LVAIVDPHIK	95.0%	52.4	14.1	4	0	0	2	1,104.68
									MMDYLQGSGETPQTDVR	95.0%	110.0	16.8	8	0	0	2	1,959.84
									NPEPELLVR	95.0%	40.6	20.4	3	0	0	2	1,066.59
									QYASLTGTQALPPLFSLGYHQSR	95.0%	64.6	20.2	0	7	0	2	2,535.30
									VSQGSKDPAEGDGAQPEETPR	95.0%	79.7	20.8	1	17	0	2	2,154.99
									VVIIGAGKPAAVVLQTK	95.0%	75.4	3.0	4	9	0	2	1,664.05
									YRVPDVLVADPPIAR	95.0%	36.1	17.4	0	2	0	2	1,680.94
T-complex protein 1 subunit gamma	TCPG_HUMAN	CCT3	60,517	100.00%	12	13	35	24.80%	AVAQALEVIPR	95.0%	68.6	16.5	8	0	0	2	1,166.69
									DVTHPR	95.0%	34.3	18.9	1	0	0	2	724.37
									EILSEVER	95.0%	48.5	23.3	2	0	0	2	974.52
									EIQVQHPAAK	95.0%	45.3	22.4	2	0	0	2	1,120.61
									GISDLAQHYLMR	95.0%	49.6	22.8	2	0	0	2	1,419.71
									IVLLDSSLEYK	95.0%	57.5	20.4	6	0	0	2	1,279.72
									KISIPVDISDSDMMLNIINSSITTK	95.0%	30.1	20.5	0	1	0	2	2,767.41
									KVQSGNINAAK	95.0%	50.8	23.3	2	1	0	2	1,129.63
									MLLDPMGGIVMTNDGNAILR	95.0%	88.2	21.7	2	0	0	2	2,179.06
									NLQDAMQVCR	95.0%	40.3	20.9	2	0	0	2	1,250.56
									TAVETAVLLLR	95.0%	77.5	15.8	5	0	0	2	1,185.72
									VQSGNINAAK	95.0%	48.4	23.0	1	0	0	2	1,001.54
Glutathione reductase, mitochondrial	GSHR_HUMAN	GSR	56,239	100.00%	4	4	15	9.96%	ADFDNTVAIHPTSSEELVTLR	95.0%	70.9	21.7	0	2	0	2	2,315.15
									ALLTPVAIAAGR	95.0%	81.9	14.0	8	0	0	2	1,152.71
									LNAIYQNNLTK	95.0%	44.6	21.8	3	0	0	2	1,291.70
									RAAELGAR	95.0%	54.8	21.5	2	0	0	2	843.48
Isocitrate dehydrogenase [NADP] cytoplasmic	IDHC_HUMAN	IDH1	46,643	100.00%	15	16	64	39.10%	ATDFVVPGPGK	95.0%	81.9	23.1	2	0	0	2	1,087.58
									ATDFVVPGPGKVEITYTPSDGTQK	95.0%	38.7	20.8	0	2	0	2	2,507.27
									DATNDQVTK	95.0%	32.4	21.8	1	0	0	2	991.47
									DIFQEIYDK	95.0%	30.7	22.2	1	0	0	2	1,170.57
									FKDIFQEIYDK	95.0%	57.4	22.8	5	5	0	2	1,445.73
									GQETSTNPIASIFAWTR	95.0%	80.1	22.8	4	0	0	2	1,878.94
									ISGGSVVEMQGDEMTR	95.0%	97.1	18.8	6	0	0	2	1,727.76

									KISGGSVVEMQGDEMTR	95.0%	58.5	21.3	1	0	0	2	1,855.85
									LIDDMVAQAMK	95.0%	54.3	22.9	4	0	0	2	1,266.61
									NILGGTVFR	95.0%	47.1	20.8	4	0	0	2	976.56
									SDYLNTFEFMDK	95.0%	84.7	17.2	5	0	0	2	1,525.65
									SIEDFAHSSFQMALSK	95.0%	47.5	21.6	4	0	0	2	1,813.84
									TVEAEAAHGTVTR	95.0%	91.5	22.7	9	0	0	2	1,341.68
									VEITYTPSDGTQK	95.0%	67.3	22.9	5	0	0	2	1,438.71
									VTYLVHNFEEGGGVAMGMYNQDK	95.0%	49.3	18.3	0	6	0	2	2,591.15
Quinone oxidoreductase	QOR_HUMAN	CRYZ	35,189	100.00%	9	12	34	33.10%	DLSLLSHGGR	95.0%	52.0	21.1	4	0	0	2	1,054.56
									GTIEINPR	95.0%	42.4	20.6	4	0	0	2	899.50
									ILGTAGTEEGQK	95.0%	55.7	23.5	3	0	0	2	1,203.62
									KPLLPYTPGSDVAGVIEAVGDNASAFK	95.0%	48.7	19.3	0	4	0	2	2,716.42
									KPLLPYTPGSDVAGVIEAVGDNASAFKK	95.0%	41.5	17.9	0	1	1	2	2,844.52
									QGAAIGIPYFTAYR	95.0%	42.4	21.0	2	0	0	2	1,527.80
									SDIAVPIPK	95.0%	54.7	14.8	2	0	0	2	939.55
									VAEAHENIIHGSGATGK	95.0%	81.4	22.6	3	4	2	2	1,690.85
									VFEFGGPEVLK	95.0%	57.6	22.9	4	0	0	2	1,221.65
Desmoplakin	DESP_HUMAN	DSP	331,763	100.00%	21	23	130	10.90%	AELIVQPELK	95.0%	52.7	17.6	9	0	0	2	1,139.67
									ALLQAILQTEDMLK	95.0%	94.8	19.9	37	0	0	2	1,602.88
									AQQIHSQTSQQYPLYDLDLGK	95.0%	35.7	21.5	0	3	0	2	2,433.21
									AVTGYNDPETGNIISLFQAMNK	95.0%	96.0	21.7	2	2	0	2	2,399.16
									FGDSNTVMR	95.0%	40.5	17.5	5	0	0	2	1,042.46
									GFFDPNTEENLTYLQLK	95.0%	73.8	22.3	8	0	0	2	2,028.99
									GYFNEELSEILSDPSDDTK	95.0%	68.2	20.0	3	0	0	2	2,158.97
									HQNQNTIQELLQNCSDCLMR	95.0%	58.7	20.4	0	3	0	2	2,518.12
									IEVLEEELR	95.0%	66.2	24.0	6	0	0	2	1,129.61
									ITNLTQQLEQASIVK	95.0%	65.2	17.3	1	0	0	2	1,685.94
									LLEAQIASGGVVDPVNSVFLPKDVALAR	95.0%	43.7	12.8	0	1	0	2	2,878.60
									LLEAQIATGGIIDPK	95.0%	58.7	17.0	1	0	0	2	1,538.88
									LLQLQEQMR	95.0%	43.7	23.5	6	0	0	2	1,174.63
									QLQNIIQATSR	95.0%	61.9	22.2	9	0	0	2	1,271.71
									SAIYQLEEEYENLLK	95.0%	78.0	22.0	3	0	0	2	1,841.92
									SVEEVASEIQPFLR	95.0%	31.6	22.3	1	0	0	2	1,603.83
									SVQNDSQAIAEVLNQLK	95.0%	64.6	20.8	5	1	0	2	1,856.97
									TLELQGLINDLQR	95.0%	74.1	19.7	7	0	0	2	1,512.84
									TMIQSPSGVILQEAADVHAR	95.0%	64.8	21.9	0	9	0	2	2,139.09
									YGDGIQLTR	95.0%	74.9	22.2	5	0	0	2	1,022.53
									YIELLTR	95.0%	38.1	19.1	3	0	0	2	907.53
Protein RCC2	RCC2_HUMAN	RCC2	56,067	100.00%	9	14	31	20.70%	AGGAAVVITEPEHTK	95.0%	54.4	22.1	2	2	0	2	1,479.78
									AGGAAVVITEPEHTKER	95.0%	63.3	21.4	2	3	2	2	1,764.92
									DGQILPVPNVVVR	95.0%	55.6	15.9	2	0	0	2	1,405.82
									LFDFPGR	95.0%	40.9	21.7	3	0	0	2	851.44
									LGHAEQKDEMVPR	95.0%	47.6	22.6	2	2	0	2	1,525.74
									MGQLGLGNQTDAVPSPAQIMYNGQPITK	95.0%	58.7	20.6	0	2	0	2	2,961.45
									RVEAPR	95.0%	41.7	19.6	3	0	0	2	727.42
									TKDGQILPVPNVVVR	95.0%	44.8	13.8	2	2	0	2	1,634.96
									TLDGIFSEQVAMGYSHSLVIAR	95.0%	62.4	21.1	0	2	0	2	2,410.21
Vinculin	VINC_HUMAN	VCL	123,783	100.00%	49	56	447	49.50%	AAAVGTANK	95.0%	37.6	25.2	2	0	0	2	802.44
									AASDELSK	95.0%	50.0	24.1	3	0	0	2	820.41
									AAVHLEGK	95.0%	46.2	18.9	3	0	0	2	824.46
									AGEVINQPMMMAAR	95.0%	59.9	20.5	25	0	0	2	1,566.71

									AIPDLTAPVAAVQAAVSNLVR	95.0%	126.0	14.3	38	3	0	2	2,076.18
									ALASQLQDSLK	95.0%	74.7	22.9	20	0	0	2	1,173.65
									ALASQLQDSLKDLK	95.0%	82.5	19.7	2	0	0	2	1,529.85
									AQQVSQGLDVLTAK	95.0%	108.0	21.3	17	0	0	2	1,457.80
									AVAGNISDPGLQK	95.0%	74.3	21.1	19	0	0	2	1,269.68
									CDRVDQLTAQLADLAAR	95.0%	60.6	22.3	1	2	0	2	1,915.97
									DPSASPGDAGEQAIR	95.0%	80.8	21.0	16	0	0	2	1,470.68
									DYLIDGSR	95.0%	33.5	21.9	2	0	0	2	938.46
									EAEAASIK	95.0%	45.3	24.2	4	0	0	2	818.43
									EAFQPQEPDFPPPPPDLEQLR	95.0%	114.0	21.4	4	0	0	2	2,447.19
									ELLPVLISAMK	95.0%	61.3	18.9	11	0	0	2	1,229.72
									ELTPQVVSAAR	95.0%	67.0	22.0	20	0	0	2	1,170.65
									ETVQTTEDQILK	95.0%	66.5	23.1	2	0	0	2	1,404.72
									ETVQTTEDQILKR	95.0%	77.0	22.4	3	0	0	2	1,560.82
									EVENSEDPK	95.0%	48.4	17.8	2	0	0	2	1,046.46
									GILSGTSDLLLTFDEAEVR	95.0%	76.8	21.3	3	0	0	2	2,036.06
									GILSGTSDLLLTFDEAEVRK	95.0%	98.6	19.8	2	1	0	2	2,164.15
									GQGSSPVAMQK	95.0%	52.2	21.5	9	0	0	2	1,105.53
									GVGQAAIR	95.0%	56.2	19.8	2	0	0	2	771.45
									IPTISTQLK	95.0%	37.2	19.3	9	0	0	2	1,000.60
									LANVMMGPYR	95.0%	67.3	21.3	6	0	0	2	1,183.56
									LLAVAATAPPDAPNREEVFDER	95.0%	50.4	21.3	2	11	0	2	2,381.21
									LVQAAQMLQSDPYSVPAR	95.0%	91.3	21.7	10	2	0	2	1,990.01
									MALLMAEMSR	95.0%	55.0	20.1	11	0	0	2	1,200.54
									MLGQMTDQVADLR	95.0%	103.0	21.8	16	0	0	2	1,509.70
									MQEAMTQEVSDVFSDTTTPIK	95.0%	150.0	19.4	12	2	0	2	2,390.07
									MSAEINEIIR	95.0%	76.6	24.0	20	0	0	2	1,191.60
									MTGLVDEAIDTK	95.0%	86.4	23.3	22	0	0	2	1,308.64
									NPGNQAAYEHFETMK	95.0%	54.7	20.7	5	0	0	2	1,752.77
									NQGIEEALK	95.0%	48.2	22.1	5	0	0	2	1,001.53
									PVFHTR	95.0%	36.6	19.5	2	0	0	1	756.42
									QVATALQNLQTK	95.0%	78.8	21.5	12	0	0	2	1,314.74
									SFLDSGYR	95.0%	41.8	19.5	2	0	0	2	944.45
									SLGEISALTSK	95.0%	83.8	22.4	15	0	0	2	1,105.61
									SLLDASEEAIKK	95.0%	57.0	22.3	3	0	0	2	1,303.71
									STVEGIQASVK	95.0%	81.7	22.6	8	0	0	2	1,118.61
									TDAGFTLR	95.0%	55.3	23.1	8	0	0	2	880.45
									TIESILEPVAQQISHLVIMHEEGEVDGK	94.6%	25.6	20.5	0	1	0	2	3,117.58
									TNISDEESEQATEMLVHNAQNLMQSVK	95.0%	81.0	17.8	0	15	0	2	3,078.40
									VAMANIQPQMLVAGATSIAR	95.0%	126.0	21.7	16	1	0	2	2,074.08
									VDQLTAQLADLAAR	95.0%	81.6	21.5	2	0	0	2	1,484.81
									VGKETVQTTEDQILK	95.0%	65.7	21.1	2	0	0	2	1,688.91
									VMLVNSMNTVK	95.0%	49.2	22.3	8	0	0	2	1,267.64
									VMLVNSMNTVKELLPVLISAMK	95.0%	34.7	17.6	0	2	0	2	2,478.34
									VREAFQPQEPDFPPPPPDLEQLR	95.0%	33.8	20.9	0	1	0	2	2,702.36
Semaphorin-3A	SEM3A_HUMAN	SEMA3A	88,873	100.00%	27	38	558	43.50%	ACAECCLAR	95.0%	35.8	15.1	3	0	0	2	1,110.45
									ALVYWQFQR	95.0%	54.0	22.5	10	0	0	2	1,210.64
									DFAIFR	95.0%	45.6	20.3	13	0	0	2	768.40
									DGPNYQWVPYQGR	95.0%	61.2	21.0	7	0	0	2	1,579.73
									DHIFSFDLVNIK	95.0%	78.8	22.6	39	7	0	2	1,447.76
									DHIFSFDLVNIKDFQK	95.0%	87.8	21.8	3	14	0	2	1,966.01

									DLPDDVITFAR	95.0%	65.5	23.2	89	0	0	2	1,261.64
									ENAIDGEHSGK	95.0%	63.0	20.9	2	0	0	2	1,156.52
									EPTAISAMELSTK	95.0%	81.7	23.4	15	0	0	2	1,393.69
									FISAHLISESDNPEDDKVYFFFR	95.0%	70.9	21.4	0	8	10	2	2,776.33
									GSAVCMYSMSDVR	95.0%	64.5	14.9	7	0	0	2	1,494.60
									IGQICK	95.0%	40.0	24.0	2	0	0	2	718.39
									IVWPVSYTR	95.0%	42.5	21.6	2	0	0	2	1,120.62
									LENSHFENGR	95.0%	76.4	19.2	4	6	0	2	1,202.56
									LLTASLLIDGELYSGTAADFMGR	95.0%	89.9	21.4	7	17	0	2	2,430.22
									NDFGGHR	95.0%	43.3	16.7	3	0	0	2	802.36
									NPVVYGVFTTSSNIFK	95.0%	124.0	22.0	132	20	0	2	1,772.92
									QQQLYIGSTAGVAQLPLHR	95.0%	92.4	19.1	2	35	0	2	2,080.13
									RVFLGPYAHR	95.0%	40.5	21.5	0	2	0	2	1,215.68
									SHPAMYNPVFPMNNRPIVIK	95.0%	33.6	20.7	0	7	10	2	2,357.19
									TDQGLLLR	95.0%	54.4	23.4	25	0	0	2	915.53
									TDVNYQFTQIVVDR	95.0%	81.3	23.2	4	0	0	2	1,697.85
									TFGGFDSTKDLPDDVITFAR	95.0%	101.0	21.5	2	9	0	2	2,202.07
									TKEMSNSMTPSQK	95.0%	30.5	18.6	1	0	0	2	1,500.67
									VDAEDGQYDVMFIGTDVGTVLK	95.0%	119.0	21.2	7	2	0	2	2,388.13
									VFLGPYAHR	95.0%	52.1	23.4	24	2	0	2	1,059.57
									VTLEVIDTEHLEELLHKDDDGDGSK	95.0%	63.0	22.1	0	6	0	2	2,807.36
Heat shock 70 kDa protein 1A/1B	HSP71_HUMAN	HSPA1A	70,036	100.00%	14	18	76	38.10%	AFYPEEISSMVLTK	95.0%	92.8	22.3	6	0	0	2	1,630.80
									AQIHDLVLVGGSTR	95.0%	64.6	19.8	2	0	0	2	1,465.81
									ATAGDTHLGGEDFDNR	95.0%	74.8	18.3	3	2	0	2	1,675.73
									DAGVIAGLNVLR	95.0%	102.0	17.9	9	0	0	2	1,197.70
									FGDPVVQSDMK	95.0%	70.8	22.0	2	0	0	2	1,238.57
									HWPFQVINDGDKPK	95.0%	31.0	22.3	0	1	0	2	1,680.85
									IINEPTAAAIAYGLDR	95.0%	122.0	21.1	8	4	0	2	1,687.90
									ITITNDKGR	95.0%	44.0	22.1	2	0	0	2	1,017.57
									LLQDFFNGR	95.0%	53.8	22.5	3	0	0	2	1,109.57
									LVNHFVEEFKR	95.0%	33.6	22.6	0	5	0	2	1,417.76
									MKEIAEAYLGYPVTNAVITVPAYFNDSQR	95.0%	39.8	19.8	0	2	0	2	3,276.63
									MVQEAEKYK	95.0%	36.9	22.3	2	0	0	2	1,141.56
									NALESYAFNMK	95.0%	77.1	20.6	8	0	0	2	1,303.60
									NQVALNPQNTVFDAK	95.0%	92.1	22.0	6	0	0	2	1,658.85
									SINPDEAVAYGAAVQAAILMGDK	95.0%	112.0	21.4	3	2	0	2	2,320.15
									TTPSYVAFTDTER	95.0%	102.0	21.7	73	0	0	2	1,487.70
									VEIIANDQGNR	95.0%	74.6	22.6	42	0	0	2	1,228.63
									YKAEDEVQR	95.0%	46.6	23.5	5	5	0	2	1,137.55
SH3 domain-binding glutamic acid-rich-like protein 3	SH3L3_HUMAN	SH3BGRL3	10,420	100.00%	3	3	7	39.80%	IQYQLVDISQDNALRDEMR	95.0%	49.1	21.8	0	4	0	2	2,323.14
									SQQSEVTR	95.0%	35.1	23.6	1	0	0	2	934.46
									VYSTSVTGSR	95.0%	72.7	22.1	2	0	0	2	1,056.53
Collagen alpha-1(VI) chain	CO6A1_HUMAN	COL6A1	108,513	100.00%	27	32	537	34.60%	DAEEAISQTIDTIVDMIK	95.0%	121.0	22.0	74	7	0	2	2,007.98
									DQLLPPSPNNR	95.0%	45.0	22.5	2	0	0	2	1,250.65
									DTTPLNVLCSPGIQVVSVGIK	95.0%	78.2	18.2	2	0	0	2	2,197.19
									ENYAELLEDAFLK	95.0%	82.0	23.5	2	0	0	2	1,554.77
									FEPGQSYAGVVQYSHSQMQEHVSLR	95.0%	26.6	20.9	0	1	0	2	2,880.34
									GAPGPAGPPGDPGLMGER	95.0%	82.0	21.7	4	0	0	2	1,648.78
									GDEGPPGSEGAR	95.0%	66.0	21.6	20	0	0	2	1,128.49
									GDPGEAGPQGDQGR	95.0%	55.5	18.6	15	0	0	2	1,340.58
									GDPGFEGER	95.0%	45.5	20.0	4	0	0	2	963.42

									GLEQLLVGGSHLK	95.0%	83.4	18.1	34	28	0	2	1,350.77
									GPEGPQGPPGHQGPPGPDECEILDIIMK	95.0%	65.0	20.6	0	2	0	2	2,938.37
									GVFHQTVSR	95.0%	59.4	23.9	21	0	0	2	1,030.54
									IALVITDGR	95.0%	72.3	20.0	36	0	0	2	957.57
									KGLEQLLVGGSHLK	95.0%	39.4	14.0	0	2	0	2	1,478.87
									LKPYGALVDK	95.0%	40.5	18.4	0	13	0	2	1,103.65
									LLLFSDGNSQGATPAAIEK	95.0%	140.0	22.9	64	0	0	2	1,932.01
									LSIIATDHTYR	95.0%	69.4	23.4	11	0	0	2	1,289.69
									LSRDELVK	95.0%	38.2	22.1	1	0	0	2	959.55
									NNVEQVCCSFECQPAR	95.0%	79.0	13.8	1	0	0	2	1,997.83
									NVQELK	95.0%	40.9	22.9	3	0	0	2	730.41
									QVNEPHIR	95.0%	31.5	22.1	1	0	0	2	992.53
									TAEYDVAYGESHLFR	95.0%	95.4	20.7	17	22	0	2	1,757.81
									TDPAHDVR	95.0%	35.1	21.8	2	0	0	2	910.44
									VAVVQYSGTGQQRPER	95.0%	51.3	22.0	8	19	0	2	1,774.92
									VFSVAITPDHLEPR	95.0%	78.8	21.3	26	47	0	2	1,580.84
									VPSYQALLR	95.0%	66.1	21.9	47	0	0	2	1,046.60
									YLIVVTDGHPLEGYK	95.0%	30.3	21.7	0	1	0	2	1,703.90
Inter-alpha-trypsin inhibitor heavy chain H1	ITIH1_HUMAN	ITIH1	101,372	99.50%	2	2	2	2.41%	AAISGENAGLVR	95.0%	41.4	21.9	1	0	0	2	1,157.63
									LDAQASFLPK	95.0%	38.5	22.7	1	0	0	2	1,089.59
Valyl-tRNA synthetase	SYVC_HUMAN	VARS	140,460	100.00%	6	6	9	6.96%	DPGVITYDLPTPPGEK	95.0%	46.0	21.5	1	0	0	2	1,698.86
									IETMLGDVAVAVHPK	95.0%	31.4	20.7	0	1	0	2	1,595.85
									ITPAHDQNDYEVGQR	95.0%	26.0	21.6	0	1	0	2	1,742.81
									LLSPFMPFVTEELFQR	95.0%	46.5	21.1	2	0	0	2	1,970.01
									LSAAVTEAFVR	95.0%	68.5	20.7	2	0	0	2	1,163.64
									SSAQDPQAVLGALGR	95.0%	61.8	22.1	2	0	0	2	1,469.77
X-ray repair cross-complementing protein 6	XRCC6_HUMAN	XRCC6	69,828	100.00%	18	22	94	39.90%	DIISIAEDEDLR	95.0%	65.2	22.8	3	0	0	2	1,388.69
									DSLIFLVDASK	95.0%	77.5	21.9	8	0	0	2	1,207.66
									DTGIFLDLMHLK	95.0%	50.8	23.4	14	1	0	2	1,418.74
									ELVYPPDYNPEGK	95.0%	49.9	22.8	3	0	0	2	1,520.73
									FDDPGLMLMGFKPLVLLK	95.0%	31.6	19.0	0	3	0	2	2,066.11
									IISSDRDLLAVVFYGTEK	95.0%	37.4	19.6	0	3	0	2	2,026.09
									IMATPEQVGK	95.0%	53.6	23.3	6	0	0	2	1,089.56
									IMLFTNEDNPHGNDSAK	95.0%	40.1	20.5	0	2	0	2	1,918.86
									IQVTPPGFQLVFLPFADDK	95.0%	96.3	19.8	3	0	0	2	2,132.14
									KQELLEALTK	95.0%	49.6	19.9	4	0	0	2	1,172.69
									LGSLVDEFK	95.0%	70.4	23.3	4	0	0	2	1,007.54
									NIPPYFVALVPQEEELDDQK	95.0%	42.1	22.1	1	0	0	2	2,344.17
									NIYVLQELDNPGAK	95.0%	129.0	22.7	16	0	0	2	1,573.82
									NLEALALDLMEPEQAVDLTLPK	95.0%	91.1	19.8	4	1	0	2	2,439.27
									SDSFENPVLQQHFR	95.0%	64.8	22.1	4	3	0	2	1,703.81
									SQIYGSR	95.0%	31.3	21.9	1	0	0	2	810.41
									TFNTSTGGLLLPSDTK	95.0%	77.2	22.3	4	0	0	2	1,651.85
									TFNTSTGGLLLPSDTKR	95.0%	74.9	21.2	5	1	0	2	1,807.96
Polypeptide N-acetylgalactosaminyltransferase 10	GLT10_HUMAN	GALNT10	68,975	99.50%	2	2	2	7.13%	ERQPDGTPGGSGAAVAPAAGQGSHSR	95.0%	23.6	21.2	0	0	1	2	2,418.15
									VGNGEQGRPYPMTDAER	95.0%	26.5	20.6	0	1	0	2	1,892.86
Calpain small subunit 1	CPNS1_HUMAN	CAPNS1	28,299	100.00%	4	4	7	25.00%	ILGGVISAISEAAAQYNPEPPPPR	95.0%	61.7	20.0	0	1	0	2	2,447.29
									LFAQLAGDDMEVSATELMNILNK	95.0%	48.7	21.6	0	3	0	2	2,555.24
									LGFEEFK	95.0%	37.2	20.5	2	0	0	2	869.44
									SMVAVMDSDTTGK	95.0%	33.4	16.9	1	0	0	2	1,373.59
Xaa-Pro aminopeptidase 1	XPP1_HUMAN	XPNPEP1	69,901	99.90%	2	2	5	5.30%	GSLTFEPLTLVPIQTK	95.0%	71.5	17.9	4	0	0	2	1,743.99

									TLSLDEVYLIDSGAQYK	95.0%	54.3	21.5	1	0	0	2	1,914.97
Proteasome subunit beta type-3	PSB3_HUMAN	PSMB3	22,932	100.00%	10	12	50	50.20%	DAVSGMGVIVHIIEK	95.0%	42.2	22.4	1	0	0	2	1,583.85
									DAVSGMGVIVHIIEKDK	95.0%	18.1	20.5	0	0	1	2	1,826.97
									FGIQAQMVTTDFQK	95.0%	88.3	22.5	6	0	0	2	1,629.79
									FGPYYTEPVIAGLDPK	95.0%	64.5	21.6	5	0	0	2	1,766.90
									FRLNLYELK	95.0%	36.9	19.6	0	2	0	2	1,195.68
									LNLYELK	95.0%	44.3	19.6	4	0	0	2	892.51
									LYIGLAGLATDVQTVAQR	95.0%	127.0	16.8	17	8	0	2	1,889.05
									NCVAIAADRR	95.0%	34.5	22.7	1	2	0	2	1,145.58
									QIKPYTLMSMVANLLYEK	95.0%	26.2	20.9	0	1	0	2	2,174.12
									RFGPYYTEPVIAGLDPK	95.0%	31.2	21.0	0	2	0	2	1,923.00
Spliceosome RNA helicase BAT1	UAP56_HUMAN	BAT1	48,974	100.00%	11	13	67	30.10%	DFLLKPELLR	95.0%	32.4	16.1	2	1	0	2	1,243.74
									DVQEIFR	95.0%	34.9	24.2	2	0	0	2	906.47
									ELAFQISK	95.0%	41.0	20.9	3	0	0	2	935.52
									FMQDPMEIFVDDETK	95.0%	76.7	16.5	2	0	0	2	1,876.80
									GLAITFVSDENDAK	95.0%	104.0	22.8	12	0	0	2	1,479.73
									GSYVSIHSSGFR	95.0%	58.3	22.0	2	0	0	2	1,296.63
									ILVATNLFGR	95.0%	80.8	17.7	18	0	0	2	1,103.66
									LTLHGLQQYYVK	95.0%	41.0	20.4	2	4	0	2	1,462.81
									QVMMFSATLSK	95.0%	44.4	21.5	3	0	0	2	1,274.61
									VAVFFGGLSIK	95.0%	61.4	16.5	9	0	0	2	1,137.67
									VNIAFNYDMPEDSDTYLHR	95.0%	44.9	19.0	0	7	0	2	2,316.02
3-hydroxyisobutyryl-CoA hydrolase, mitochondrial	HIBCH_HUMAN	HIBCH	43,466	100.00%	11	11	22	30.30%	AGIATHFVDSEK	95.0%	69.5	22.3	2	0	0	2	1,274.64
									AVLIDKDQSPK	95.0%	59.9	21.3	2	0	0	2	1,213.68
									ENIASVLENYHTESK	95.0%	63.5	22.8	2	0	0	2	1,733.83
									EVTEEDLNNHFK	95.0%	51.9	21.4	2	0	0	2	1,474.68
									IAPVFFR	95.0%	31.4	19.4	1	0	0	2	849.50
									LAMLEEDLLALK	95.0%	71.0	20.8	2	0	0	2	1,374.76
									LGYFLALTGFR	95.0%	69.4	21.5	4	0	0	2	1,257.70
									SLGSSDLKF	95.0%	46.7	21.7	2	0	0	2	953.49
									SPSKENIASVLENYHTESK	95.0%	54.5	22.0	0	2	0	2	2,133.05
									TLQEVLTMEYR	95.0%	70.2	22.5	2	0	0	2	1,398.69
									WEQDPETFLIIIK	95.0%	38.9	22.0	1	0	0	2	1,631.87
3-ketoacyl-CoA thiolase, mitochondrial	THIM_HUMAN	ACAA2	41,906	100.00%	6	7	18	29.50%	AANDAGYFNDEMAPIEVK	95.0%	87.8	18.8	4	0	0	2	1,970.88
									DFTATDLSEFAAK	95.0%	97.6	21.4	2	0	0	2	1,415.67
									DGTVTAGNASGVADGAGAVIIASEDAVKK	95.0%	28.3	20.8	0	1	0	2	2,644.34
									TNVNGGAIALGHPLGGSGSR	95.0%	30.0	21.5	0	2	0	2	1,834.95
									TPFGAYGGLLK	95.0%	54.1	21.2	4	0	0	2	1,123.62
									VSPETVDSVIMGNVLQSSSDAIYLAR	95.0%	47.8	21.2	2	3	0	2	2,767.38
Tissue factor pathway inhibitor	TFPI1_HUMAN	TFPI	34,998	100.00%	6	6	117	22.00%	FFFNIFTR	95.0%	53.2	24.0	67	0	0	2	1,091.57
									FYYNSVIGK	95.0%	49.5	24.0	22	0	0	2	1,090.56
									IAYEEIFVK	95.0%	51.0	21.7	16	0	0	2	1,111.60
									KGFIQR	95.0%	37.4	19.5	3	0	0	2	748.45
									TTLQQEKPDFCFLEEDPGICR	95.0%	43.8	20.4	0	4	0	2	2,583.19
									YSGCGGNENNFTSK	95.0%	99.2	14.0	5	0	0	2	1,534.62
Guanine nucleotide-binding protein subunit beta-2-like 1	GBLP_HUMAN	GNB2L1	35,059	100.00%	8	10	44	30.30%	DETNYGIPQR	95.0%	64.7	22.1	7	0	0	2	1,192.56
									DGQAMLWDLNEGK	95.0%	60.2	20.9	2	0	0	2	1,492.67
									DVLSVAFSSDNR	95.0%	84.2	22.1	13	0	0	2	1,309.64
									GHNGWVTQIATTPQFPDMILSASR	95.0%	45.5	21.0	0	2	0	2	2,643.30
									IIVDELKQEVISTSSK	95.0%	90.9	18.1	10	3	0	2	1,789.00
									LTRDETNYGIPQR	95.0%	44.2	22.8	2	1	0	2	1,562.79

									LWDLTTGTTTR	95.0%	53.0	22.7	2	0	0	2	1,264.65
									QIVSGSR	95.0%	34.4	24.2	2	0	0	2	746.42
Lactotransferrin	TRFL_HUMAN	LTF	78,164	100.00%	5	5	148	8.87%	CGLVPVLAENYK	95.0%	39.9	22.0	2	0	0	2	1,362.71
									CLAENAGDVAFVK	95.0%	67.5	22.5	14	0	0	2	1,336.66
									DGAGDVAFIR	95.0%	69.8	24.5	2	0	0	2	1,020.51
									IDSGLYLGSGYFTAIQNLR	95.0%	84.4	21.7	1	0	0	2	2,088.08
									YYGYTGAFR	95.0%	51.5	21.7	129	0	0	2	1,097.51
Nucleolar and coiled-body phosphoprotein 1	NOLC1_HUMAN	NOLC1	73,586	100.00%	4	5	12	13.00%	AAESSSDSSDSDSSEDDEAPSKPAGTTK	95.0%	78.6	11.1	0	3	0	2	2,758.13
									AASSSSSSSSSSSSDDSEEEKAAATPK	95.0%	60.2	17.2	0	2	0	2	2,577.09
									SSDSDSDSSSEDEPPKNQKPK	95.0%	54.9	17.8	0	1	0	2	2,263.98
									VVPSDLYPLVLGFLR	95.0%	48.9	14.8	5	1	0	2	1,687.98
Galectin-3-binding protein	LG3BP_HUMAN	LGALS3BP	65,314	100.00%	24	30	1286	43.90%	AAFGQGSGPIMLDEVQCTGTEASLADCK	95.0%	56.0	17.5	0	4	0	2	2,929.30
									ASHEEVEGLVEK	95.0%	69.9	22.9	76	55	0	2	1,326.65
									AVDTWSWGER	95.0%	53.1	19.8	6	0	0	2	1,206.55
									ELSEALGQIFDSQR	95.0%	120.0	22.8	187	32	0	2	1,592.79
									GLNLTEDTYKPR	95.0%	67.6	23.1	2	0	0	2	1,406.73
									IDITLSSVK	95.0%	63.2	19.2	16	0	0	2	975.57
									IYTSPTWSAFVTDSSWSAR	95.0%	121.0	21.5	8	0	0	2	2,162.02
									KSQLVYQSR	95.0%	53.0	20.0	15	2	0	2	1,108.61
									KTLQALEFHTVPFQLLAR	95.0%	42.9	14.0	0	18	16	2	2,112.20
									LADGGATNQGR	95.0%	92.6	20.7	86	0	0	2	1,059.52
									LASAYGAR	95.0%	62.2	23.0	28	0	0	2	808.43
									RIDITLSSVK	95.0%	60.6	16.6	70	0	0	2	1,131.67
									SDLAVPSELALLK	95.0%	88.8	18.8	134	0	0	2	1,355.78
									SLGWLK	95.0%	35.8	24.7	4	0	0	2	703.41
									SQLVYQSR	95.0%	55.8	21.4	22	0	0	2	980.52
									STHTLDLSR	95.0%	45.2	21.4	32	0	0	2	1,029.53
									STSSFPCPAGHFNGFR	95.0%	55.8	20.1	4	0	0	2	1,768.79
									TIAYENK	95.0%	40.7	22.7	9	0	0	2	838.43
									TLQALEFHTVPFQLLAR	95.0%	96.9	17.5	112	120	0	2	1,984.10
									TVIRPFYLTNSSGVD	95.0%	54.1	21.0	3	0	0	2	1,668.86
									VEIFYR	95.0%	34.2	21.1	9	0	0	2	826.45
									YSSDYFQAPSDYR	95.0%	109.0	17.6	164	0	0	2	1,598.68
									YYPYQSFQTPQHPSFLFQDK	95.0%	59.4	21.3	9	35	0	2	2,521.18
									YYPYQSFQTPQHPSFLFQDKR	95.0%	54.0	20.9	0	8	0	2	2,677.28
Gamma-interferon-inducible lysosomal thiol reductase	GILT_HUMAN	IFI30	29,131	99.50%	2	3	26	6.90%	GMQLMHANAQR	95.0%	53.2	20.7	7	1	0	2	1,272.59
									TGNLYLR	95.0%	68.5	21.4	18	0	0	2	836.46
Phosphatidylinositol transfer protein alpha isoform	PIPNA_HUMAN	PITPNA	31,788	99.50%	2	3	6	13.30%	MLAPEGALNIHEK	95.0%	33.4	22.5	0	2	0	2	1,438.74
									VILPVSVDEYQVGQLYSVAEASK	95.0%	89.4	19.9	2	2	0	2	2,494.31
Glutamate dehydrogenase 1, mitochondrial	DHE3_HUMAN	GLUD1	61,382	100.00%	8	8	23	19.40%	ALASLMTYK	95.0%	34.2	20.8	2	0	0	2	1,013.53
									GASIVEDKLVEDLR	95.0%	74.2	21.9	2	0	0	2	1,543.83
									GFIGPGIDVPAPDMSTGER	95.0%	41.7	22.6	1	0	0	2	1,931.92
									HGGTIPIVPTAEFQDR	95.0%	59.1	21.7	4	0	0	2	1,737.89
									IIAEGANGPTTPEADKIFLER	95.0%	50.5	20.6	0	4	0	2	2,242.17
									TAAYVNAIEK	95.0%	44.3	22.6	2	0	0	2	1,079.57
									YNLGLDLR	95.0%	60.5	23.1	4	0	0	2	963.53
									YSTDVSVDEVK	95.0%	71.5	22.0	4	0	0	2	1,241.59
Pre-mRNA-processing factor 19	PRP19_HUMAN	PRPF19	55,163	100.00%	6	7	12	19.00%	FIASTGMDR	95.0%	31.7	19.7	1	0	0	2	1,013.47
									ILTGGADKNVVVFDK	95.0%	74.9	19.0	2	1	0	2	1,575.87
									KVTSVVFHPSQDLVFSASPDATIR	95.0%	53.8	19.3	0	2	0	2	2,601.37
									SSEQILATLK	95.0%	49.3	22.2	2	0	0	2	1,089.62

									TVPEELVKPEELSK	95.0%	61.4	20.7	2	0	0	2	1,597.87
									YIAENGTDPINNQPLSEEQLIDIK	95.0%	38.2	20.8	0	2	0	2	2,714.35
Splicing factor U2AF 65 kDa subunit	U2AF2_HUMAN	U2AF2	53,483	100.00%	5	5	18	13.50%	AMQGLTGR	95.0%	50.6	23.1	4	0	0	2	849.43
									ELLTSFGPLK	95.0%	58.5	21.0	5	0	0	2	1,104.63
									LFIGGLPNYLNDDQVK	95.0%	60.4	21.6	4	0	0	2	1,805.94
									NFAFLEFR	95.0%	34.8	22.3	1	0	0	2	1,043.53
									SVDETTQAMAFDGIIFQGQSLK	95.0%	107.0	21.6	4	0	0	2	2,402.16
Collagen alpha-2(V) chain	CO5A2_HUMAN	COL5A2	144,892	100.00%	38	45	746	35.90%	DGEVGPSGPVGPPGLAGER	95.0%	101.0	22.5	20	0	0	2	1,747.86
									EGNPGPLGPIGPPGVR	95.0%	52.2	21.4	24	0	0	2	1,513.81
									EGPPGLR	95.0%	36.8	17.9	1	0	0	2	725.39
									GANDLDIK	95.0%	51.3	22.0	6	0	0	2	845.44
									GANDLDIKAEGNIR	95.0%	66.1	22.8	7	6	0	2	1,485.77
									GAPGKDGEVGPSGPVGPPGLAGER	95.0%	69.0	21.5	1	0	0	2	2,158.09
									GDPGTVGPPGPVGER	95.0%	59.8	23.3	17	0	0	2	1,391.69
									GEAGPTGPMGAMGPLGPR	95.0%	64.3	21.4	16	0	0	2	1,684.78
									GENGPTGAVGFAGPQGPDGQPGVK	95.0%	75.2	22.0	13	2	0	2	2,194.05
									GEPGPHGIQGPIGPPGEEGK	95.0%	37.5	22.3	0	5	0	2	1,909.94
									GEPGPHGIQGPIGPPGEEGKR	95.0%	18.3	22.0	0	0	1	2	2,066.04
									GIVGMPGQR	95.0%	47.3	22.8	14	0	0	2	930.48
									GLPGPLGPPGPAGPTGEK	95.0%	48.8	20.9	3	0	0	2	1,598.85
									GLPGPQGPR	95.0%	36.6	21.3	2	0	0	2	878.49
									GLTGNPGVQGPEGK	95.0%	65.9	22.4	49	0	0	2	1,310.67
									GMPGLPGPAGTPGK	95.0%	44.0	22.7	5	0	0	2	1,252.64
									GPAGPPGGPGDKGDPGEDGQPGPDGPPGPAGTTGQR	95.0%	87.1	18.3	0	10	0	2	3,204.46
									GPEGPPGKPGEDGEPGR	95.0%	67.6	21.4	39	56	0	2	1,632.76
									GPEGPQGQR	95.0%	39.4	21.8	2	0	0	2	925.45
									GPIGPPGR	95.0%	33.6	19.7	2	0	0	2	750.43
									GPPGPVGPSGK	95.0%	58.5	22.7	31	0	0	2	949.51
									GRPGPAGPPGSQGPR	95.0%	63.2	22.5	16	0	0	2	1,387.72
									GSQFAYGDHQSPNTAITQMTFLR	95.0%	73.6	20.6	3	105	0	2	2,586.20
									GSQGDPGRPGEPGLPGAR	95.0%	37.2	22.7	0	5	0	2	1,704.84
									GSSGDPGKPGEAGNAGVPGQR	95.0%	30.8	22.8	0	2	0	2	1,894.90
									GTQGPPGATGFPGSAGR	95.0%	101.0	22.2	8	0	0	2	1,514.74
									LGPLGAPGEDGRPGPPGSIGIR	95.0%	33.8	19.3	0	11	0	2	2,070.11
									LGPQGAPGQR	95.0%	64.0	21.2	47	0	0	2	980.53
									NGNPGEVGFAGSPGAR	95.0%	89.2	21.6	14	0	0	2	1,486.70
									NSVGYMDDQAK	95.0%	57.6	17.6	40	0	0	2	1,243.53
									QSGEYWIDPNQGSVEDAIK	95.0%	73.8	20.8	6	0	0	2	2,135.99
									SGLGSQVGLMPGSVGPVGPR	95.0%	81.2	21.1	56	3	0	2	1,867.97
									SLSSQIETMR	95.0%	74.9	22.1	65	0	0	2	1,167.57
									SPDNKPVWYGLDMNR	95.0%	47.0	22.5	2	8	0	2	1,807.84
									TVFEYR	95.0%	31.7	20.9	1	0	0	2	814.41
									VGPPGPAGAPGPAGPLGEPGK	95.0%	82.9	20.8	12	1	0	2	1,779.94
									VGPPGPAGAPGPAGPLGEPGKEGPPGLR	95.0%	42.3	18.1	0	8	0	2	2,486.32
									YIVLQDTCSK	95.0%	33.1	22.7	1	0	0	2	1,226.61
Heterogeneous nuclear ribonucleoprotein R	HNRPR_HUMAN	HNRNPR	70,926	100.00%	3	3	20	10.60%	EAAQEAVK	95.0%	45.9	22.0	17	0	0	2	845.44
									EFNEEGALSVLQQFK	95.0%	67.9	22.5	2	0	0	2	1,738.87
									LFVGSIPK	95.0%	35.3	18.4	2	0	0	2	860.52
									LMMDPLSGQNR	95.0%	53.9	20.8	4	0	0	2	1,293.59
									NLATTVTEEILEK	95.0%	85.7	22.1	14	0	0	2	1,460.79
									TGYTLDVTTGQR	95.0%	77.1	22.4	16	0	0	2	1,311.65

Proteasome subunit beta type-4	PSB4_HUMAN	PSMB4	29,187	100.00%	8	11	47	47.30%	EVLEKQPVLSQTEAR	95.0%	44.1	20.1	1	2	0	2	1,726.93
									FEGGVVIAADMLGSYGSLAR	95.0%	112.0	22.4	20	0	0	2	2,029.01
									FQIATVTEK	95.0%	48.2	20.5	2	0	0	2	1,036.57
									GVEIEGPLSTETNWDIAHMISGFE	95.0%	66.4	19.8	2	0	0	2	2,648.22
									QPVLSQTEAR	95.0%	50.9	22.0	5	0	0	2	1,128.60
									QVLGQMVIDEELLGDGHSYSPR	95.0%	84.1	21.3	2	6	0	2	2,459.19
									TQNPMVTGTSVLGVK	95.0%	82.7	22.3	3	0	0	2	1,547.81
									VNNSTMLGASGDYADFQYLK	95.0%	114.0	21.3	2	2	0	2	2,210.01
Fructose-bisphosphate aldolase C	ALDOC_HUMAN	ALDOC	39,438	100.00%	6	9	65	33.80%	DGADFAK	95.0%	44.3	22.6	7	0	0	2	723.33
									GILAADESVGSMAK	95.0%	98.6	22.6	2	0	0	2	1,364.67
									GVVPLAGTDGETTTQGLDGLSER	95.0%	132.0	21.5	29	5	0	2	2,273.13
									KDGADFAK	95.0%	31.2	22.3	2	0	0	2	851.43
									QVLFSADDR	95.0%	35.8	23.0	2	0	0	2	1,050.52
									TPSALAILENANVLAR	95.0%	75.1	17.1	4	2	0	2	1,652.93
									VDKGVVPLAGTDGETTTQGLDGLSER	95.0%	95.0	20.6	2	17	0	2	2,615.32
									VLAAVYK	95.0%	48.2	18.0	20	0	0	2	763.47
									YASICQQNGIVPIVEPEILPDGDHDLK	95.0%	55.5	20.8	0	4	0	2	3,020.50
									YASICQQNGIVPIVEPEILPDGDHDLKR	95.0%	82.0	19.4	0	4	3	2	3,176.61
									YTPEEIAMATVTALR	95.0%	89.9	22.6	2	0	0	2	1,681.85
Heat shock 70 kDa protein 4	HSP74_HUMAN	HSPA4	94,314	100.00%	38	46	443	51.30%	AFSDPFVEAEK	95.0%	68.1	21.5	12	0	0	2	1,239.59
									AGGIETIANEYSDR	95.0%	71.0	22.1	22	0	0	2	1,495.70
									EDIYAVEIVGGATR	95.0%	86.9	23.1	6	0	0	2	1,492.76
									EFSITDVVPYPISLR	95.0%	68.0	20.7	44	0	0	2	1,735.93
									ELSTTLNADEAVTR	95.0%	87.1	23.2	16	0	0	2	1,519.76
									EMLNLYIENEGK	95.0%	69.3	22.0	9	0	0	2	1,468.70
									FLEMCNDLLAR	95.0%	51.2	20.9	5	0	0	2	1,397.66
									FQESEERPK	95.0%	49.4	22.8	32	28	0	2	1,149.55
									FVSEDDRNSFTLK	95.0%	64.2	22.1	6	0	0	2	1,557.76
									LFEELGK	95.0%	43.7	20.3	2	0	0	2	835.46
									LKETAESVLK	95.0%	49.2	21.0	4	4	0	2	1,117.65
									LMNETTAVALAYGIYK	95.0%	128.0	22.1	22	2	0	2	1,773.91
									LMNETTAVALAYGIYKQDLPALEEKPR	95.0%	63.2	19.0	0	0	2	2	3,050.59
									LNLQNK	95.0%	34.0	23.3	4	0	0	2	729.43
									MDQPPQAK	95.0%	50.7	19.4	4	0	0	2	930.44
									MQVDQEEPHVEEQQQTPAENK	95.0%	65.1	19.0	0	6	0	2	2,638.17
									MQVDQEEPHVEEQQQTPAENKAESEEMETSQAGSK	95.0%	33.6	13.0	0	0	5	2	4,118.77
									NAEQNGPVDGQGDNPGPQAAEQGTDTAVPSDSDK	95.0%	33.3	16.0	0	3	0	2	3,366.46
									NAVEEYVYEMR	95.0%	71.6	18.3	22	0	0	2	1,418.63
									NFTTEQVTAMLLSK	95.0%	114.0	22.3	24	0	0	2	1,598.81
									NHAAPFSK	95.0%	35.6	20.7	3	0	0	2	871.44
									NKEDQYDHLDAADMTK	95.0%	83.8	17.5	4	7	1	2	1,909.82
									QDLPALEEKPR	95.0%	63.3	21.0	6	2	0	2	1,295.70
									QIQQYMK	95.0%	33.2	21.5	1	0	0	2	954.47
									QSLTMDPVVK	95.0%	46.2	23.9	11	0	0	2	1,133.59
									QVYVDK	95.0%	30.6	23.1	1	0	0	2	751.40
									SEENEEPMETDQNAK	95.0%	68.8	12.0	5	0	0	2	1,766.70
									SEENEEPMETDQNAKEEEK	95.0%	55.6	13.8	2	3	0	2	2,281.93
									SKEIEAK	95.0%	39.3	25.0	1	0	0	2	804.45
									SNLAYDIVQLPTGLTGIK	95.0%	109.0	17.2	62	3	0	2	1,903.05
									SVLEQTK	95.0%	38.5	25.0	2	0	0	2	804.45
									SVMDATQIAGLNCLR	95.0%	74.7	22.9	1	0	0	2	1,664.81

									TSTVDLPIENQLLWQIDR	95.0%	94.1	20.6	5	0	0	2	2,141.12
									VLATAFDTTLGGR	95.0%	105.0	21.7	33	0	0	2	1,321.71
									VREFSITDVVPYPISLR	95.0%	56.4	16.5	2	0	0	2	1,991.10
									VTPQSDGSSSK	95.0%	41.2	22.2	2	0	0	2	1,092.52
									VTYMEEER	95.0%	49.0	17.5	1	0	0	2	1,072.46
									VTYMEEERNFTTEQVTAMLLSK	95.0%	46.6	21.3	0	1	0	2	2,652.25
Tissue alpha-L-fucosidase	FUCO_HUMAN	FUCA1	53,672	100.00%	2	2	8	4.72%	DLVGELGTALR	95.0%	79.8	23.1	6	0	0	2	1,143.64
									ITMLGIQGDLK	95.0%	55.3	22.5	2	0	0	2	1,204.66
Protein transport protein Sec24A	SC24A_HUMAN	SEC24A	119,734	100.00%	5	5	9	7.50%	DALVNAVIDSLSAYR	95.0%	81.9	22.5	3	0	0	2	1,606.84
									MSVFQTQLPTLGVGALKPR	95.0%	26.5	17.2	0	2	0	2	2,059.14
									SSVLSNQQPGLMVPFSLR	95.0%	53.3	21.7	1	0	0	2	1,976.03
									TLETQSALGPALQAAFK	95.0%	44.2	20.3	1	0	0	2	1,745.94
									TYINPFVSFLDQR	95.0%	65.8	21.8	2	0	0	2	1,599.82
L-aminoadipate-semialdehyde dehydrogenase-phosphopantetheinyl transferase	ADPPT_HUMAN	AASDHPPT	35,759	100.00%	3	4	8	11.00%	GSIPEFFHIMK	95.0%	32.8	23.1	1	0	0	2	1,321.66
									HQDVPSQDDSKPTQR	95.0%	45.0	22.4	1	4	0	2	1,737.82
									IGQFVFAR	95.0%	64.1	20.6	2	0	0	2	937.53
ERO1-like protein alpha	ERO1A_HUMAN	ERO1L	54,377	100.00%	4	4	7	12.60%	FDGILTEGEGPR	95.0%	33.6	22.0	1	0	0	2	1,290.63
									LIANMPESGPSYEFHLTR	95.0%	37.9	22.5	0	2	0	2	2,078.00
									QEIVSLFNAFGR	95.0%	71.7	22.2	3	0	0	2	1,380.73
									VLPFFERPDFQLFTGNK	95.0%	39.0	21.2	1	0	0	2	2,055.07
Glycylpeptide N-tetradecanoyltransferase 1	NMT1_HUMAN	NMT1	56,789	100.00%	3	3	8	8.87%	DIPVVHQLLTR	95.0%	39.5	18.3	2	0	0	2	1,290.75
									GFDVFNALDLMENK	95.0%	78.3	21.4	5	0	0	2	1,628.76
									LGEVVNTHGPVEPDKDNIR	95.0%	48.0	21.6	0	1	0	2	2,089.07
RuvB-like 2	RUVB2_HUMAN	RUVBL2	51,140	100.00%	12	13	51	32.60%	ALESDMAPVLIMATNR	95.0%	118.0	23.1	8	3	0	2	1,763.87
									DYDAMGSQTK	95.0%	60.4	14.3	2	0	0	2	1,131.46
									EYQDAFLFNELKGETMDTS	95.0%	70.7	18.5	4	0	0	2	2,253.99
									GLGLDDALEPR	95.0%	73.1	21.5	2	0	0	2	1,155.60
									GTEVQVDDIKR	95.0%	27.9	23.2	0	1	0	2	1,259.66
									GTSYQSPHGIPIDLLDR	95.0%	49.7	22.1	4	0	0	2	1,868.95
									LLIVSTTPYSEK	95.0%	64.5	19.9	4	0	0	2	1,350.75
									LLIVSTTPYSEKDTK	95.0%	80.0	20.2	4	0	0	2	1,694.92
									QASQGMVGQLAAR	95.0%	61.0	23.1	2	0	0	2	1,332.67
									TQGFLALFSGDTGEIK	95.0%	98.1	21.3	6	0	0	2	1,683.86
									TTEMETIYDLGTK	95.0%	80.4	20.5	6	0	0	2	1,517.70
									VYSLFLDESR	95.0%	51.5	22.8	5	0	0	2	1,228.62
Glycyl-tRNA synthetase	SYG_HUMAN	GARS	83,124	100.00%	11	14	69	19.60%	AQVSGQSAR	95.0%	42.6	22.4	1	0	0	2	903.46
									GEFTIETEGK	95.0%	57.8	21.7	4	0	0	2	1,110.53
									LGDAVEQGVINNTVLGYFIGR	95.0%	97.9	19.5	8	4	0	2	2,235.18
									LPFAAAQIGNSFR	95.0%	71.2	21.9	8	0	0	2	1,391.74
									QQGDLVR	95.0%	37.0	21.5	1	0	0	2	815.44
									SPITGNDLSPPVSFNLMFK	95.0%	72.4	22.0	6	0	0	2	2,080.04
									TFFSFPAVVAPFK	95.0%	52.3	21.9	18	0	0	2	1,457.78
									TLYVEEVVPNVIEPSFGLGR	95.0%	106.0	19.2	5	2	0	2	2,218.18
									TVNVVQFEPSK	95.0%	61.8	22.5	4	0	0	2	1,247.66
									VDDSSGSIGR	95.0%	75.1	22.2	4	0	0	2	992.47
									YPLFEGQETGKK	95.0%	39.6	22.5	2	2	0	2	1,396.71
Vesicular integral-membrane protein VIP36	LMAN2_HUMAN	LMAN2	40,212	100.00%	10	13	163	36.20%	DHDTFLAVR	95.0%	37.8	22.5	11	0	0	2	1,073.54
									DNFHGLAIFLDTYPNDETTER	95.0%	122.0	20.2	9	34	0	2	2,468.14
									DRLVPGPVFGSK	95.0%	57.2	21.7	11	9	0	2	1,271.71
									LFQLMVEHTPDEESIDWTK	95.0%	41.0	21.2	0	6	0	2	2,334.10
									LPTGYYFGASAGTGDLSDNHDIISMK	95.0%	102.0	19.2	0	51	0	2	2,746.27

									LTPDER	95.0%	31.4	20.3	1	0	0	2	730.37
									NCIDITGVR	95.0%	55.5	23.0	10	0	0	2	1,047.53
									NLHGDGIALWYTR	95.0%	46.8	22.3	2	1	0	2	1,515.77
									NRDHDTFLAVR	95.0%	42.7	21.9	0	16	0	2	1,343.68
									WTELAGCTADFR	95.0%	81.4	20.5	2	0	0	2	1,426.64
Endoplasmic reticulum aminopeptidase 2	ERAP2_HUMAN	ERAP2	110,449	100.00%	3	3	4	3.65%	ILAVTDFEPTQAR	95.0%	61.6	22.6	2	0	0	2	1,460.78
									LNIPTDVLK	95.0%	33.0	16.7	1	0	0	2	1,012.60
									TQNLAALLHAIAR	95.0%	57.6	15.7	1	0	0	2	1,391.81
ATP-dependent RNA helicase A	DHX9_HUMAN	DHX9	140,944	100.00%	13	13	82	13.10%	AAMEALVVEVTK	95.0%	40.1	23.2	2	0	0	2	1,276.68
									AIEPPPLDAVIEAEHTLR	94.8%	30.3	20.0	1	0	0	2	1,971.06
									DFVNYLVR	95.0%	50.4	21.8	4	0	0	2	1,025.54
									DVVQAYPEVR	95.0%	50.2	23.4	5	0	0	2	1,175.61
									ELDALDANDELTPLGR	95.0%	89.0	21.6	6	0	0	2	1,741.86
									GISHVIVDEIHER	95.0%	52.3	21.9	0	2	0	2	1,503.79
									GMTLVTPLQLLLFASK	95.0%	73.3	13.4	33	0	0	2	1,748.00
									LGGIGQFLAK	95.0%	65.0	18.4	6	0	0	2	1,003.59
									QPAIISQLDPVNER	95.0%	38.8	20.9	2	0	0	2	1,579.84
									TPLHEIALSIK	95.0%	40.9	14.6	0	4	0	2	1,221.72
									TTQVPQFILDDFIQNDR	95.0%	67.2	22.1	10	0	0	2	2,050.02
									VFDPVPVGVTK	94.6%	30.1	21.8	1	0	0	2	1,157.66
									YPSPFFVFGEK	95.0%	49.0	22.2	6	0	0	2	1,317.65
40S ribosomal protein S3	RS3_HUMAN	RPS3	26,671	100.00%	15	15	68	67.90%	AELNEFLTR	95.0%	67.0	23.1	8	0	0	2	1,092.57
									DEILPTTPISEQK	95.0%	61.1	22.0	2	0	0	2	1,470.77
									ELAEDGYSGVEVR	95.0%	89.6	20.9	11	0	0	2	1,423.67
									ELTAVVQKR	95.0%	44.2	19.4	2	0	0	2	1,043.62
									FGFPEGSVELYAEK	95.0%	61.2	22.7	6	0	0	2	1,572.76
									FIMESGAK	95.0%	34.6	21.3	1	0	0	2	898.43
									FVDGLMIHSGDPVNYYVDTAVR	95.0%	46.0	21.1	0	2	0	2	2,484.19
									GGKPEPPAMPQPVPTA	95.0%	55.1	22.5	9	0	0	2	1,589.80
									GLCAIAQAESLR	95.0%	36.3	23.2	2	0	0	2	1,288.67
									IMLPWDPTGK	95.0%	42.6	22.6	2	0	0	2	1,173.60
									KFVADGIFK	95.0%	44.8	20.4	2	0	0	2	1,024.58
									KPLPDHVSIVEPK	95.0%	44.9	18.7	2	0	0	2	1,458.83
									KPLPDHVSIVEPKDEILPTTPISEQK	95.0%	53.1	16.1	0	2	0	2	2,910.58
									LLGGLAVR	95.0%	67.8	14.8	8	0	0	2	798.52
									TEIIILATR	95.0%	65.4	15.3	9	0	0	2	1,029.63
26S proteasome non-ATPase regulatory subunit 1	PSMD1_HUMAN	PSMD1	105,821	100.00%	14	16	33	23.90%	AAVESLGFILFR	95.0%	74.2	18.8	5	0	0	2	1,322.75
									DTSEDIEELVEPVAAHGPK	95.0%	71.8	21.6	2	2	0	2	2,035.98
									EAINLLEPMTNDPVNYVR	95.0%	83.4	21.6	3	0	0	2	2,104.04
									EALQLMATYLPK	95.0%	47.7	21.7	2	0	0	2	1,393.74
									LLHVAVSDVNDDVRR	95.0%	38.4	20.8	0	2	2	2	1,707.91
									MITSAAGIISLLDEDEPQLK	95.0%	79.5	21.6	1	0	0	2	2,160.11
									NAQAIEDMVGYAQETQHEK	95.0%	26.1	20.7	0	1	0	2	2,177.98
									QDVYDLLK	95.0%	49.0	21.7	2	0	0	2	993.53
									QFAALVASK	95.0%	31.2	20.0	1	0	0	2	934.54
									TILESNDVPGMLAYSLK	95.0%	77.4	22.2	2	0	0	2	1,866.95
									TNLYQDDAVTGEAAGLALGLVMLGSK	95.0%	48.4	21.0	0	2	0	2	2,623.33
									TPEASPEPK	95.0%	47.3	21.5	2	0	0	2	955.47
									TVGTPIASVPGSTNTGTVPGSEKDSDSMETEEK	95.0%	50.4	19.3	0	2	0	2	3,324.53
									VSTAVLSITAK	95.0%	68.0	18.5	2	0	0	2	1,089.65
40S ribosomal protein S15a	RS15A_HUMAN	RPS15A	14,822	100.00%	4	4	11	30.80%	HGYIGEFEIIDDHR	95.0%	57.4	21.8	0	4	0	2	1,700.80

									IVVNLTGR	95.0%	54.7	21.0	3	0	0	2	871.54
									MNVLADALK	95.0%	41.5	22.7	2	0	0	2	974.53
									WQNNLLPSR	95.0%	32.8	21.0	2	0	0	2	1,127.60
60S ribosomal protein L7a	RL7A_HUMAN	RPL7A	29,978	100.00%	2	2	13	10.90%	AGVNTVTTLVENK	95.0%	93.4	22.0	11	0	0	2	1,345.73
									LKVPPAINQFTQALDR	95.0%	44.8	17.6	0	2	0	2	1,811.02
Amyloid-like protein 2	APLP2_HUMAN	APLP2	86,937	100.00%	10	18	141	19.30%	ADMDQFTASISETPVDVR	95.0%	103.0	19.6	30	1	0	2	1,997.91
									CLVGEFVSDVLLVPEK	95.0%	59.4	21.6	7	0	0	2	1,803.96
									EMIFNAER	95.0%	33.3	20.9	1	0	0	2	1,025.47
									HYQHVLAVDPEK	95.0%	64.9	22.5	8	11	0	2	1,435.73
									LNMHVNIQTGK	95.0%	42.3	22.2	1	12	0	2	1,270.66
									MALENYLAALQSDPPRPHR	95.0%	65.9	21.4	0	20	22	2	2,195.10
									QQLVETHLAR	95.0%	43.0	19.4	2	5	0	2	1,194.66
									QTLIQHFQAMVK	95.0%	34.6	22.2	2	0	0	2	1,459.77
									SQVMTHLHVIEER	95.0%	44.1	21.9	1	2	2	2	1,594.80
									VEAMLNDR	95.0%	59.7	21.9	23	0	0	2	963.46
									VPYVAQEIQEEIDELLQEQR	95.0%	104.0	21.4	2	12	0	2	2,429.22
Proteasome subunit beta type-1	PSB1_HUMAN	PSMB1	26,473	100.00%	10	12	70	52.30%	AGGSASAMLQPLLDNQVGFK	95.0%	87.1	22.3	9	4	0	2	2,020.02
									AMTTGAIAAMLSTILYSR	95.0%	105.0	22.2	30	0	0	2	1,902.97
									DVFISAAER	95.0%	52.8	23.3	3	0	0	2	1,007.52
									DVYTGDALR	95.0%	32.9	22.0	2	0	0	2	1,009.50
									EGIREETVSLR	95.0%	48.2	23.1	2	0	0	2	1,288.69
									FFPYYVYNIIGGLDEEGK	95.0%	51.8	22.5	1	0	0	2	2,124.03
									GAVYSFDPVGSYQR	95.0%	94.5	21.2	6	0	0	2	1,545.73
									LSEGFSIHTR	95.0%	64.3	23.7	5	0	0	2	1,146.59
									LVKDVFISAAER	95.0%	54.9	19.4	2	0	0	2	1,347.76
									NMQNVEHVPLSLDR	95.0%	72.2	22.6	5	1	0	2	1,667.82
26S proteasome non-ATPase regulatory subunit 14	PSDE_HUMAN	PSMD14	34,559	100.00%	2	2	8	9.35%	AVAVVVDPIQSVK	95.0%	66.6	14.9	7	0	0	2	1,324.78
									AVEEEDKMTPEQLAIK	95.0%	52.2	22.0	1	0	0	2	1,846.91
U4/U6.U5 tri-snRNP-associated protein 2	SNUT2_HUMAN	USP39	65,364	100.00%	3	3	4	8.85%	ANDYANAVLQALSNVPPLR	95.0%	54.1	20.3	1	0	0	2	2,026.07
									EQLIIPQVPLFNILAK	95.0%	31.1	10.8	1	0	0	2	1,836.10
									NPTIVNFPITNVDLR	95.0%	51.2	19.9	2	0	0	2	1,712.93
Thioredoxin domain-containing protein 5	TXND5_HUMAN	TXNDC5	47,611	100.00%	4	4	15	7.87%	DLESLREYVESQLQR	95.0%	41.5	21.6	1	0	0	2	1,864.94
									EYVESQLQR	95.0%	40.5	23.3	3	0	0	2	1,151.57
									FVLSQAKDEL	95.0%	43.5	22.8	1	0	0	2	1,149.62
									GYPTLLLFR	95.0%	62.9	18.6	10	0	0	2	1,079.63
Actin-related protein 2	ARP2_HUMAN	ACTR2	44,744	100.00%	6	7	25	19.30%	DLMVGDEASELR	95.0%	74.5	20.7	4	0	0	2	1,350.62
									GYAFNHSADFETVR	95.0%	70.5	20.6	2	2	0	2	1,613.73
									HIVLSGGSTMYPGLPSR	95.0%	80.6	22.1	2	0	0	2	1,787.91
									ILLTEPPMNPTK	95.0%	58.1	20.8	7	0	0	2	1,369.74
									LNIDTR	95.0%	39.8	25.1	4	0	0	2	731.41
									SMLEVNYPMENGIVR	95.0%	70.2	21.8	4	0	0	2	1,783.84
Dihydrolipoyl dehydrogenase, mitochondrial	DLDH_HUMAN	DLD	54,159	100.00%	8	10	20	19.40%	ADGGTQVIDTK	95.0%	50.3	23.2	4	0	0	2	1,104.55
									ALTGGIAHLFK	95.0%	49.8	20.2	1	0	0	2	1,127.66
									IPNIYAIGDVVAGPMLAHK	95.0%	77.5	19.6	2	2	0	2	1,995.07
									NLGLEELGIELDPR	95.0%	77.8	21.7	2	0	0	2	1,567.83
									NQVTATK	94.7%	30.2	25.0	1	0	0	2	761.42
									SEEQLKEEGIEYK	95.0%	59.9	22.8	2	2	0	2	1,581.77
									VCHAHPTLSEAFR	95.0%	40.8	22.5	0	2	0	2	1,524.74
									VGKFPFAANSR	95.0%	51.7	21.6	0	2	0	2	1,193.64
Nucleobindin-1	NUCB1_HUMAN	NUCB1	53,862	100.00%	32	48	519	64.90%	AKMDAEQDPNVQVDHLNLLK	95.0%	66.4	21.4	2	17	5	2	2,294.15
									APAAHPEGQLK	95.0%	54.0	23.4	46	1	0	2	1,118.60

									DLAQYDAAHHEEFK	95.0%	68.6	20.5	2	0	0	2	1,673.76
									DLAQYDAAHHEEFKR	95.0%	82.6	21.4	2	1	0	2	1,829.86
									DLELLIQTATR	95.0%	89.7	21.1	37	0	0	2	1,272.72
									EKLQAANAEDIK	95.0%	69.1	22.9	4	0	0	2	1,329.70
									ELDFVSHHVR	95.0%	63.3	22.9	6	1	0	2	1,238.63
									ELQQAVLHMEQR	95.0%	67.1	23.0	37	1	0	2	1,481.75
									EVWEELDGLDPNRFNPK	95.0%	29.7	21.9	0	2	0	2	2,057.99
									FEEELAAR	95.0%	47.5	23.4	19	0	0	2	964.47
									FHPDTDDVPVPAPAGDQK	95.0%	73.6	22.0	10	5	0	2	1,905.90
									FHPDTDDVPVPAPAGDQKEVDTSEK	95.0%	58.2	21.0	0	12	1	2	2,694.25
									KLEEQQR	95.0%	47.2	23.4	1	0	0	2	930.50
									LLERLPEVEVPQHL	95.0%	73.0	18.1	2	9	0	2	1,671.94
									LPEVEVPQHL	95.0%	63.1	22.8	33	0	0	2	1,160.63
									LQAANAEDIK	95.0%	62.3	21.6	17	0	0	2	1,072.56
									LQAANAEDIKSGK	95.0%	27.1	22.7	0	1	0	2	1,344.71
									LSQETEALGR	95.0%	90.7	23.3	42	0	0	2	1,103.57
									LVTLEEFLASTQR	95.0%	80.1	20.3	17	17	0	2	1,506.82
									LVTLEEFLASTQRK	95.0%	76.4	17.5	8	0	0	2	1,634.91
									MDAEQDPNVQVDHLNLLK	95.0%	115.0	21.8	6	20	0	2	2,095.01
									NEEDDMREMEEER	95.0%	28.4	9.0	0	4	0	2	1,743.64
									NVDTNQDR	95.0%	57.7	18.6	2	0	0	2	961.43
									QFEHLDPQNQHTFEAR	95.0%	50.7	21.7	2	7	1	2	1,996.93
									RFEEELAAR	95.0%	48.7	24.3	10	0	0	2	1,120.58
									TFFILHDINSDGVLDEQELEALFTK	95.0%	47.0	20.6	0	2	0	2	2,894.45
									VNVPGSQAQLK	95.0%	73.3	20.2	27	0	0	2	1,140.64
									YEMLKEHER	95.0%	32.5	22.7	1	0	0	2	1,250.58
									YLESLGEEQR	95.0%	51.4	22.3	4	0	0	2	1,223.59
									YLESLGEEQRK	95.0%	64.4	22.9	7	1	0	2	1,351.69
									YLQEVIDVLETDGHFR	95.0%	123.0	22.8	27	38	0	2	1,933.97
									YLQEVIDVLETDGHFREK	95.0%	27.3	22.0	0	1	1	2	2,191.10
ES1 protein homolog, mitochondrial	ES1_HUMAN	C21orf33	28,152	100.00%	3	3	6	21.30%	EVVEAHVDQK	95.0%	39.9	22.3	2	0	0	2	1,153.59
									GGAEVQIFAPDVPQMHVIDHTK	95.0%	32.2	22.3	0	2	0	2	2,405.19
									ITDLANLSAANHDAAIFPGGFGAAK	95.0%	44.6	21.4	0	2	0	2	2,442.24
Putative deoxyribose-phosphate aldolase	DEOC_HUMAN	DERA	35,214	100.00%	4	4	9	19.50%	ASMIAMMAGSDFIK	95.0%	68.3	20.1	2	0	0	2	1,520.68
									AVTFIDLTTLSGDDTSSNIQR	95.0%	94.5	22.0	1	0	0	2	2,254.12
									ETVNATFPVAIVMLR	95.0%	50.4	20.6	2	0	0	2	1,676.90
									IGASTLLSDIER	95.0%	88.0	22.2	4	0	0	2	1,274.70
NIF3-like protein 1	NIF3L_HUMAN	NIF3L1	41,951	100.00%	6	6	13	24.90%	ALMQVVDFLSR	95.0%	68.1	22.2	5	0	0	2	1,294.68
									GFLSDLRDMLDSHLENK	95.0%	27.5	21.3	0	1	0	2	2,005.97
									GIDGVSVTSFSAR	95.0%	89.4	22.0	2	0	0	2	1,295.66
									INIILSETDRDPLQVV	95.0%	52.4	18.1	2	0	0	2	1,825.01
									TEILSLEKPLLLHTGMGR	95.0%	39.9	18.1	0	2	0	2	2,024.12
									VEFNVNYTQDLDKVMSAVK	95.0%	30.1	22.1	0	1	0	2	2,216.09
40S ribosomal protein S16	RS16_HUMAN	RPS16	16,428	100.00%	7	10	22	38.40%	EIKDILIQYDR	95.0%	67.1	20.2	2	2	0	2	1,405.77
									FAGVDIR	95.0%	47.0	24.3	2	0	0	2	777.43
									GGGHVAQIYAIR	95.0%	56.0	20.8	2	1	0	2	1,241.68
									GPLQSVQVFGR	95.0%	64.4	22.5	2	0	0	2	1,187.65
									LLEPVLLLGK	95.0%	47.4	7.8	6	0	0	2	1,094.72
									PSKGPLQSVQVFGR	95.0%	54.8	20.0	2	2	0	1	1,499.83
									VKGGGHVAQIYAIR	95.0%	33.0	17.0	0	1	0	2	1,468.84
Glucosylceramidase	GLCM_HUMAN	GBA	59,700	100.00%	7	7	24	15.50%	ATLGETHR	95.0%	60.5	22.2	3	0	0	2	884.46

									NDLDAVALMHPDGSAVVVVLNR	95.0%	69.1	21.0	0	5	0	2	2,321.19
									NFVDSPIIVDITK	95.0%	84.4	21.0	6	0	0	2	1,460.80
									NFVDSPIIVDITKDTFYK	95.0%	85.9	20.3	1	0	0	2	2,115.10
									SYFSEEGIGYNIIR	95.0%	84.2	23.2	3	0	0	2	1,647.80
									VPMASCDFSIR	95.0%	48.5	21.6	2	0	0	2	1,298.59
									VVLTDPEAAK	95.0%	48.8	21.7	4	0	0	2	1,042.58
Prolyl endopeptidase	PPCE_HUMAN	PREP	80,684	100.00%	11	11	16	21.70%	ELPDVLER	95.0%	44.1	19.4	2	0	0	2	970.52
									FIATLQYIVGR	95.0%	54.9	18.8	2	0	0	2	1,280.74
									GIDASDYQTVQIFYPSK	95.0%	44.5	22.8	1	0	0	2	1,931.94
									GMFYNSYPQQDGK	95.0%	62.9	16.4	1	0	0	2	1,550.66
									LPEADDIQYPSMLLLTADHDDR	95.0%	69.3	20.7	0	2	0	2	2,544.19
									MTELYDYPK	95.0%	42.0	19.3	1	0	0	2	1,175.53
									NILQLHDLTTGALLK	95.0%	82.8	14.9	2	0	0	2	1,649.96
									SDGTETSTNLHQK	95.0%	52.4	20.8	1	0	0	2	1,417.66
									TFPLDVGSIVGYSGQK	95.0%	77.9	21.8	2	0	0	2	1,667.86
									VFLDPNILSDDGTVALR	95.0%	98.9	21.5	1	0	0	2	1,844.98
									YFYFYNTGLQNQR	95.0%	73.9	22.0	1	0	0	2	1,713.80
Protein arginine N-methyltransferase 1	ANM1_HUMAN	PRMT1	41,498	100.00%	7	11	51	18.30%	ANKLDHVVTIIK	95.0%	58.7	12.8	2	6	0	2	1,350.81
									ATLYVTAIEDR	95.0%	82.2	21.6	11	0	0	2	1,251.66
									DVAIKEPLVDVVDPK	95.0%	53.5	18.7	8	6	0	2	1,636.92
									EPLVDVVDPK	95.0%	30.5	21.6	1	0	0	2	1,110.60
									GKVEEVELPVEK	95.0%	58.6	21.4	3	3	0	2	1,355.74
									TGEEIFGTIGMRPNAK	95.0%	45.7	22.4	4	4	0	2	1,736.86
									VEEVELPVEK	95.0%	54.2	20.7	3	0	0	2	1,170.63
Endoplasmin	ENPL_HUMAN	HSP90B1	92,454	100.00%	21	24	135	32.80%	DISTNYYASQK	95.0%	55.7	20.0	8	0	0	2	1,289.60
									EAESSPFVER	95.0%	35.4	22.1	1	0	0	2	1,150.54
									EEASDYLELDTIK	95.0%	85.0	22.1	7	0	0	2	1,525.73
									ELISNASDALDK	95.0%	97.2	23.1	53	0	0	2	1,275.64
									ELISNASDALDKIR	95.0%	109.0	21.4	34	13	0	2	1,544.83
									FAFQAEVNR	95.0%	57.1	23.1	5	0	0	2	1,081.54
									FQSSHHPTDITSLDQYVER	95.0%	72.3	21.4	0	8	1	2	2,260.06
									GLFDEYGSK	95.0%	35.8	19.4	2	0	0	2	1,015.47
									GVVDSDDLPLNVSR	95.0%	91.3	22.1	19	0	0	2	1,485.76
									IYFMAGSSR	95.0%	50.7	21.2	2	0	0	2	1,047.49
									KSDYIK	95.0%	31.7	18.9	2	0	0	2	753.41
									LGVIEDHSNR	95.0%	63.5	22.2	4	4	0	2	1,139.58
									LIINSLYK	95.0%	48.2	14.3	8	0	0	2	963.59
									LISLTDENALSGNEELTVK	95.0%	67.2	21.6	18	0	0	2	2,046.06
									LSLNIDPDAK	95.0%	55.1	22.9	2	0	0	2	1,085.58
									NLLHVTDTGVGMTR	95.0%	66.4	22.7	6	4	0	2	1,529.77
									SGTSEFLNK	95.0%	37.1	21.6	1	0	0	2	982.48
									SGYLLPDTK	95.0%	36.5	22.1	2	0	0	2	993.53
									SILFVPTSAPR	95.0%	64.8	20.5	23	0	0	2	1,187.68
									TDDEVVQREEEAIQLDGLNASQIR	95.0%	57.1	21.4	0	3	0	2	2,728.34
									TFEINPR	95.0%	31.3	24.5	1	0	0	2	876.46
									VFITDDFHDMMPK	95.0%	51.2	17.4	2	0	0	2	1,627.71
									YSQFINFPIYVWSSK	95.0%	72.4	22.6	2	0	0	2	1,878.94
Peroxiredoxin-5, mitochondrial	PRDX5_HUMAN	PRDX5	22,008	100.00%	6	6	8	32.20%	ETDLLLDDSLVSIFGNR	95.0%	71.8	22.0	1	0	0	2	1,906.98
									ETDLLLDDSLVSIFGNRR	95.0%	34.9	20.9	1	0	0	2	2,063.08
									LLADPTGAFGK	95.0%	31.0	22.9	1	0	0	2	1,089.59
									THLPGFVEQAEALK	95.0%	66.1	21.8	2	0	0	2	1,539.82

									VGDAIPAVEVFEGEPGNKVNLAELFK	95.0%	37.6	19.2	0	1	0	2	2,742.44
									VNLAELFK	95.0%	59.5	19.9	2	0	0	2	933.54
Heterogeneous nuclear ribonucleoprotein A/B	ROAA_HUMAN	HNRNPAB	36,207	100.00%	7	7	54	25.00%	DLKDYFTK	94.9%	30.3	21.8	1	0	0	2	1,029.53
									EVYQQQQYGSGGR	95.0%	87.8	21.1	27	0	0	2	1,499.69
									EYFGEFGEIEAIELPMDPK	95.0%	47.5	20.4	2	0	0	2	2,230.03
									FGEVVDCTIK	95.0%	62.7	22.7	10	0	0	2	1,167.57
									GFGFILFK	95.0%	45.0	22.7	21	0	0	2	928.53
									GFVFITFK	95.0%	35.1	23.9	1	0	0	2	958.54
									KFHTVSGSK	95.0%	37.6	24.1	1	0	0	2	990.54
									VLDQKEHR	95.0%	31.2	20.9	1	0	0	2	1,024.55
Galectin-1	LEG1_HUMAN	LGALS1	14,698	100.00%	5	6	46	42.20%	FNAHGDANTIVCNSK	95.0%	71.4	20.6	2	1	0	2	1,647.76
									LNLEAINYMAADGDFK	95.0%	124.0	22.1	35	0	0	2	1,800.85
									LPDGYEFK	95.0%	35.2	20.9	2	0	0	2	968.47
									SFVLNLGK	95.0%	49.6	18.1	4	0	0	2	877.51
									VRGEVAPDAK	95.0%	32.7	19.4	2	0	0	2	1,041.57
Protein argonaute-1	AGO1_HUMAN	EIF2C1	97,197	99.50%	2	2	2	6.42%	MLHEALVSGQIPVPLESVQALDVAMR	95.0%	50.9	20.1	0	1	0	2	2,835.48
									NASYNLDPYIQEFGIK	95.0%	52.1	22.5	1	0	0	2	1,871.92
									YAQGADSVEPMFR	95.0%	30.9	19.9	1	0	0	2	1,486.66
Microtubule-associated protein RP/EB family member 1	MARE1_HUMAN	MAPRE1	29,982	100.00%	3	4	7	17.90%	KPLTSSSAAPQRPISTQR	95.0%	34.6	19.0	0	1	2	2	1,925.06
									LEHEYIQNFK	95.0%	33.8	23.4	2	0	0	2	1,320.66
									QGQETAVAPSLVAPALNKPK	95.0%	66.0	16.1	0	2	0	2	2,019.12
Protein phosphatase 1 regulatory subunit 7	PP1R7_HUMAN	PPP1R7	41,548	100.00%	7	7	28	25.30%	AIENIDTLTNLESLFLGK	95.0%	106.0	19.3	8	0	0	2	1,991.07
									ELDLYDNQIK	95.0%	43.8	22.7	4	0	0	2	1,250.63
									GAGQQQSQEMMEVDR	95.0%	78.0	16.7	2	0	0	2	1,725.72
									IEGLQNLVNLR	95.0%	55.9	18.6	8	0	0	2	1,268.73
									LQNLDALTNLTVLSMQSNR	95.0%	119.0	21.1	2	0	0	2	2,147.11
									SLETVYLER	95.0%	41.1	21.1	3	0	0	2	1,109.58
									VMLALPSVR	95.0%	36.0	22.8	1	0	0	2	1,001.58
Semaphorin-4B	SEM4B_HUMAN	SEMA4B	92,176	100.00%	4	4	17	6.01%	GTTEGSAVCVFTMK	95.0%	66.4	20.5	2	0	0	2	1,503.68
									MLLLQPQAR	95.0%	49.3	22.0	13	0	0	2	1,085.61
									SQSLRPTK	95.0%	37.5	22.5	1	0	0	2	916.52
									VPGLHHTYDVLFLGTGDGR	95.0%	33.0	21.9	0	1	0	2	2,054.05
AP-1 complex subunit gamma-1	AP1G1_HUMAN	AP1G1	91,336	100.00%	6	6	19	12.20%	IGYLGAMLLLDER	95.0%	41.0	22.0	1	0	0	2	1,479.79
									NLIMSGYSPEHDVSGISDPFLQVR	95.0%	31.4	21.0	0	1	0	2	2,677.29
									NVGNAILYETVLTIMDIK	95.0%	73.3	19.7	7	0	0	2	2,023.08
									TFQLQLLSPSSSIVPAFNTGTITQVIK	95.0%	28.4	14.3	0	1	0	2	2,890.59
									VLAINILGR	95.0%	60.5	10.8	8	0	0	2	968.63
									YVALTSLLK	95.0%	32.8	13.2	1	0	0	2	1,007.61
Hsp90 co-chaperone Cdc37	CDC37_HUMAN	CDC37	44,450	100.00%	4	6	26	16.10%	ASEAKEGEEAGPGDPLLEAVPK	95.0%	29.1	22.4	0	1	0	2	2,194.09
									EGEEAGPGDPLLEAVPK	95.0%	53.6	22.2	6	0	0	2	1,707.84
									LGPGGLDPVEVYESLPEELQK	95.0%	96.2	20.5	14	1	0	2	2,269.16
									SMVNTKPEKTEEDSEEVR	95.0%	35.1	20.4	0	3	1	2	2,123.98
Obg-like ATPase 1	OLA1_HUMAN	OLA1	44,727	100.00%	12	15	81	37.90%	AFEDDDITHVEGSVDPIR	95.0%	73.8	21.1	2	0	0	2	2,014.94
									FNTPQQPK	95.0%	32.0	23.8	1	0	0	2	959.50
									GGDGIKPPPIIGR	95.0%	38.1	18.7	0	4	0	2	1,276.74
									HLFLTSKPMVYLVNLSEK	95.0%	38.8	18.6	0	2	0	2	2,135.16
									IGIVGLPNVGK	95.0%	55.1	13.0	11	0	0	2	1,066.66
									IPAFLNVVDIAGLVK	95.0%	103.0	12.8	35	1	0	2	1,568.94
									KGGDGIKPPPIIGR	95.0%	48.6	14.8	2	4	0	2	1,404.83
									LQELSAEER	95.0%	52.4	22.9	2	0	0	2	1,074.54
									NYIVEDGDIIFFK	95.0%	82.7	22.8	7	0	0	2	1,572.80

									YDPGALVIPFSGALELK	95.0%	38.0	19.8	2	0	0	2	1,789.97
									YEDFKEEGSENAVK	95.0%	79.8	19.8	2	1	0	2	1,644.74
									YLEANMTQSALPK	95.0%	72.7	22.7	5	0	0	2	1,481.73
Nck-associated protein 1	NCKP1_HUMAN	NCKAP1	128,777	100.00%	6	8	13	8.07%	AINQIAAALFTIHK	95.0%	66.0	16.6	2	1	0	2	1,510.87
									LVVENVDVLTQMR	95.0%	77.1	22.3	2	0	0	2	1,531.82
									NNNQQLAQLQK	95.0%	36.7	21.4	1	0	0	2	1,298.68
									SIVGMTMYNQATQEIAKPSELLTSVR	95.0%	37.7	19.8	0	2	0	2	2,899.45
									SLSDALISLQMVYPR	95.0%	53.5	21.8	2	1	0	2	1,708.89
									SLSELLGPYGMK	95.0%	63.5	22.2	2	0	0	2	1,310.67
mRNA export factor	RAE1L_HUMAN	RAE1	40,951	100.00%	2	2	4	7.61%	GLIVYQLENQPSEFR	95.0%	36.7	21.6	2	0	0	2	1,792.92
									SSNPMMVLQLPER	95.0%	61.5	22.4	2	0	0	2	1,533.74
Extracellular matrix protein 1	ECM1_HUMAN	ECM1	60,655	100.00%	8	8	40	15.60%	ELLALIQLER	95.0%	76.8	16.6	16	0	0	2	1,197.72
									ELPSLQHPNEQK	95.0%	44.5	22.9	5	0	0	2	1,419.72
									EVGPPLPQEAVPLQK	95.0%	50.6	18.3	3	0	0	2	1,601.89
									LLPAQLPAEK	95.0%	33.9	16.0	1	0	0	2	1,079.65
									LLPAQLPAEKEVGPPLPQEAVPLQK	95.0%	33.2	12.0	0	3	0	2	2,662.52
									NLPATDPLQR	95.0%	33.6	22.1	1	0	0	2	1,124.61
									NVALVSGDTENAK	95.0%	107.0	23.3	7	0	0	2	1,317.67
									QGETLNFLEIGYSR	95.0%	83.3	22.8	4	0	0	2	1,626.81
Far upstream element-binding protein 1	FUBP1_HUMAN	FUBP1	67,543	100.00%	6	6	21	10.90%	EMVLELIR	95.0%	35.5	23.0	4	0	0	2	1,018.56
									IGGNEGIDVPIPR	95.0%	79.5	21.4	4	0	0	2	1,336.72
									IQFKPDDGTTPER	95.0%	26.3	23.1	0	2	0	2	1,503.74
									IQIAPDSGGLPER	95.0%	55.0	21.3	4	0	0	2	1,352.72
									IQNDAGVR	95.0%	37.2	21.3	3	0	0	2	872.46
									SVQAGNPGGPGPGGR	95.0%	70.1	23.4	4	0	0	2	1,307.65
Lysyl oxidase homolog 2	LOXL2_HUMAN	LOXL2	86,705	100.00%	19	27	254	22.40%	AILSTYR	95.0%	46.9	18.5	5	0	0	2	823.47
									AYKPEQPLVR	95.0%	57.2	22.3	11	11	0	2	1,200.67
									EAVTGSR	95.0%	40.4	24.7	3	0	0	2	719.37
									ELGFGSAK	95.0%	35.9	23.3	1	0	0	2	808.42
									ELGYVEAK	95.0%	49.6	22.1	6	0	0	2	908.47
									FDNSLINQIENLNIQVEDIR	95.0%	124.0	20.5	21	18	0	2	2,387.22
									FEHFSGLLNNQLSPQ	95.0%	73.1	22.2	9	0	0	2	1,730.85
									FSSQIHNNGQSDFRPK	95.0%	68.1	22.5	4	23	1	2	1,861.89
									GGAYIGEGR	95.0%	41.6	23.3	3	0	0	2	879.43
									KAYKPEQPLVR	95.0%	38.3	19.1	2	4	0	2	1,328.77
									KFEHFSGLLNNQLSPQ	95.0%	76.0	22.2	16	2	0	2	1,858.95
									LGPQVSLDPMK	95.0%	39.6	22.5	2	0	0	2	1,200.63
									LGQGIGPIHLNEIQCTGNEK	95.0%	54.7	22.6	0	5	0	2	2,178.10
									LRGGAYIGEGR	95.0%	56.7	22.4	4	2	0	2	1,148.62
									RTPVMEGYVEVK	95.0%	39.3	22.2	1	5	0	2	1,423.73
									TPVMEGYVEVK	95.0%	58.1	22.1	59	0	0	2	1,267.62
									VEVLVER	95.0%	47.4	19.8	23	0	0	2	843.49
									VVCGMFGFPGER	95.0%	54.2	20.3	8	0	0	2	1,371.62
									VVMSGVK	95.0%	38.6	22.5	5	0	0	2	735.41
Fructose-bisphosphate aldolase A	ALDOA_HUMAN	ALDOA	39,403	100.00%	31	43	1201	74.70%	AAQEEYVK	95.0%	59.9	21.4	9	0	0	2	937.46
									AAQEEYVKR	95.0%	92.2	22.9	41	12	0	2	1,093.56
									ADDGRPFPQVIK	95.0%	45.4	22.1	18	22	0	2	1,342.71
									ALANSLACQGK	95.0%	62.7	24.0	8	0	0	2	1,132.58
									ALQASALK	95.0%	57.3	22.3	15	0	0	2	801.48
									DGADFAK	95.0%	44.3	22.6	7	0	0	2	723.33
									ELSDIAHR	95.0%	56.8	21.9	42	0	0	2	940.49

									FSHEEIAMATVTALR	95.0%	105.0	22.4	83	95	0	2	1,691.84
									FSHEEIAMATVTALRR	95.0%	31.9	22.3	0	1	6	2	1,847.94
									GGVVGIKVDK	95.0%	56.4	20.2	12	0	0	2	971.59
									GILAADESTGSIAK	95.0%	96.3	23.1	68	0	0	2	1,332.70
									GILAADESTGSIAKR	95.0%	97.0	21.1	18	2	0	2	1,488.80
									GVVPLAGTNGETTTQGLDGLSER	95.0%	117.0	21.8	12	4	0	2	2,272.14
									IGEHTPSALAIMENANVLAR	95.0%	86.1	21.3	66	206	0	2	2,123.09
									IVAPGKGILAADESTGSIAK	95.0%	27.9	18.2	0	1	0	2	1,898.06
									KDGADFAK	95.0%	31.2	22.3	2	0	0	2	851.43
									KELSDIAHR	95.0%	39.1	22.4	0	14	0	2	1,068.58
									LQSIGTENTEENR	95.0%	98.6	21.9	8	0	0	2	1,490.71
									LQSIGTENTEENRR	95.0%	35.7	22.9	12	3	0	2	1,646.81
									MPYQYPALTPEQK	95.0%	55.9	22.9	1	0	0	2	1,581.76
									PYQYPALTPEQK	95.0%	65.4	23.1	85	0	0	1	1,434.73
									PYQYPALTPEQKK	95.0%	53.2	22.8	13	5	0	1	1,562.82
									QLLLTADDR	95.0%	62.0	23.1	13	0	0	2	1,044.57
									RTVPPAVTGITFLSGGQSEEEASINLNAINK	95.0%	35.0	17.7	0	1	0	2	3,213.68
									SKGGVVGIK	95.0%	48.6	20.9	5	0	0	2	844.53
									TVPPAVTGITFLSGGQSEEEASINLNAINK	95.0%	64.4	19.9	0	75	0	2	3,057.57
									VDKGVVPLAGTNGETTTQGLDGLSER	95.0%	90.8	20.6	2	11	0	2	2,614.33
									VLAAVYK	95.0%	48.2	18.0	20	0	0	2	763.47
									YASICQQNGIVPIVEPEILPDGDHDLK	95.0%	55.5	20.8	0	4	0	2	3,020.50
									YASICQQNGIVPIVEPEILPDGDHDLKR	95.0%	82.0	19.4	0	4	3	2	3,176.61
									YTPSGQAGAAASESLFVSNHAY	95.0%	142.0	20.9	163	9	0	2	2,228.03
Acetyl-CoA acetyltransferase, mitochondrial	THIL_HUMAN	ACAT1	45,182	100.00%	6	6	19	21.80%	EAYMGNVLQGGEGQAPTR	95.0%	94.9	21.5	2	0	0	2	1,893.88
									FGNEVIPVTVTVK	95.0%	41.5	19.1	1	0	0	2	1,402.79
									LGSIAIQGAIEK	95.0%	47.8	20.8	1	0	0	2	1,199.70
									NEQDAYAINSYTR	95.0%	96.9	20.5	2	0	0	2	1,544.70
									TPIGSFLGSLSLLPATK	95.0%	86.0	16.2	12	0	0	2	1,701.98
									VNINGGAVSLGHPIGMSGAR	95.0%	30.8	21.8	0	1	0	2	1,922.99
Calmodulin-like protein 5	CALL5_HUMAN	CALML5	15,875	99.50%	2	2	4	28.80%	AFSAVDTDGNGTINAQELGAALK	95.0%	117.0	21.6	3	0	0	2	2,263.12
									AMAGLGQPLPQEELDAMIR	95.0%	61.3	22.2	1	0	0	2	2,072.02
Nicastrin	NICA_HUMAN	NCSTN	78,394	100.00%	3	3	4	4.51%	ALADVATVLGR	95.0%	32.5	21.2	1	0	0	2	1,085.63
									APDVTTLPR	95.0%	31.8	20.3	1	0	0	2	969.54
									NQVEDLLATLEK	95.0%	46.1	22.4	2	0	0	2	1,372.73
1-phosphatidylinositol-4,5-bisphosphate phosphodiesterase beta-3	PLCB3_HUMAN	PLCB3	138,785	100.00%	4	4	7	5.02%	EAQVDAEAQR	95.0%	46.0	21.3	1	0	0	2	1,116.53
									LNEVLYPPLRPSQAR	95.0%	33.1	17.3	0	2	0	2	1,752.98
									LVAGQQQVLQQLAEEEPK	95.0%	83.3	20.0	2	0	0	2	2,008.07
									SFDPFTEVIVDGIVANALR	95.0%	71.3	20.7	2	0	0	2	2,063.08
Neurolysin, mitochondrial	NEUL_HUMAN	NLN	80,636	100.00%	10	10	18	17.90%	AELGALPDDFIDSLEK	95.0%	49.1	22.5	4	0	0	2	1,732.87
									DGSPIADDLLEK	95.0%	46.7	22.2	2	0	0	2	1,272.63
									EFILNLK	95.0%	31.7	18.8	1	0	0	2	876.52
									EVMSPLQAMSSYTVAGR	95.0%	55.0	21.7	1	0	0	2	1,858.87
									FDIEMSMR	95.0%	40.9	17.5	2	0	0	2	1,060.44
									LVNTGLLTLR	95.0%	90.4	16.1	1	0	0	2	1,099.68
									NLILKPGGSLDGMDMLHNFLK	95.0%	25.9	20.3	0	1	0	2	2,345.20
									NLNEDDTFLVFSK	95.0%	73.2	22.5	1	0	0	2	1,541.75
									TRTEELIVQTK	95.0%	36.2	20.3	1	0	0	2	1,317.74
									VTAFLDDLSQK	95.0%	57.2	22.8	4	0	0	2	1,236.65
Plexin-B2	PLXB2_HUMAN	PLXNB2	205,109	100.00%	4	4	7	2.94%	DLVLSGDLGSLYAMTQDK	95.0%	47.7	21.1	1	0	0	2	1,941.95
									EASPNPEDGIVR	95.0%	38.4	21.7	3	0	0	2	1,283.62

									FMEPVTMQESGTFAFR	95.0%	93.5	20.3	2	0	0	2	1,909.85
									VLYAVFSR	95.0%	34.8	19.7	1	0	0	2	954.54
Gamma-glutamylcyclotransferase	GGCT_HUMAN	GGCT	20,990	100.00%	7	7	20	43.60%	ENGLPLEYQEK	95.0%	44.8	22.6	2	0	0	2	1,319.65
									LDFGNSQGK	95.0%	35.9	22.6	4	0	0	2	965.47
									LKAIEPNDYTGK	95.0%	28.7	22.4	0	2	0	2	1,348.71
									SGMYVVIEVK	95.0%	32.7	22.1	1	0	0	2	1,140.60
									SNLNSLDEQEGVK	95.0%	73.5	22.6	4	0	0	2	1,432.69
									SYLMTNYESAPPSPQYK	95.0%	89.5	20.1	4	0	0	2	1,991.91
									VSEEIEDIIK	95.0%	73.8	23.3	3	0	0	2	1,174.62
Proteasome activator complex subunit 1	PSME1_HUMAN	PSME1	28,706	100.00%	14	16	62	55.80%	APLDIPVPDPVK	95.0%	37.3	18.9	1	0	0	2	1,260.72
									APLDIPVPDPVKEK	95.0%	51.5	18.5	2	1	0	2	1,517.86
									EPALNEANLSNLK	95.0%	37.8	22.0	2	0	0	2	1,412.74
									IEDGNNFGVAVQEK	95.0%	81.5	23.1	3	0	0	2	1,519.74
									ISELDAFLKEPALNEANLSNLK	95.0%	87.6	18.5	0	2	0	2	2,429.29
									IVVLLQR	95.0%	45.6	12.8	4	0	0	2	840.57
									LEGFHTQISK	95.0%	38.1	23.1	2	0	0	2	1,159.61
									LMVMEIR	95.0%	47.4	22.6	8	0	0	2	923.47
									NAYAVLYDIILK	95.0%	90.5	18.6	23	0	0	2	1,395.79
									QLVHELDEAEYR	95.0%	70.0	21.6	2	2	0	2	1,501.73
									TENLLGSYFPK	95.0%	62.4	22.0	7	0	0	2	1,268.65
									VDVFREDLCTK	95.0%	32.7	22.4	1	0	0	2	1,381.68
									VQPEAQAK	95.0%	32.8	19.9	1	0	0	2	870.47
									YFSERGDAVTK	95.0%	36.8	21.9	1	0	0	2	1,272.62
UPF0364 protein C6orf211	CF211_HUMAN	C6orf211	51,156	100.00%	3	4	6	11.60%	IHEAIIQSPPIDYFDVFK	95.0%	29.3	20.8	0	1	0	2	2,132.11
									LRNELQTDKPFIPLVEK	95.0%	35.8	15.9	0	2	0	2	2,040.15
									TIEDLDENQLKDEFFK	95.0%	66.3	21.7	2	1	0	2	1,983.96
Alpha-enolase	ENOA_HUMAN	ENO1	47,152	100.00%	31	45	2002	67.70%	AAVPSGASTGIYEALELR	95.0%	166.0	21.4	297	64	0	2	1,804.94
									AAVPSGASTGIYEALELRDNDK	95.0%	92.0	21.9	2	0	0	2	2,277.14
									AGYTDKVVIGMDVAASEFFR	95.0%	90.9	21.8	23	88	0	2	2,192.07
									AVEHINK	95.0%	42.5	19.5	26	0	0	2	810.45
									DATNVGDEGGFAPNILENK	95.0%	98.0	21.5	112	3	0	2	1,960.93
									DATNVGDEGGFAPNILENKEGLELLK	95.0%	78.5	21.6	0	72	0	2	2,743.38
									EGLELLK	95.0%	47.1	20.9	10	0	0	2	801.47
									FTASAGIQVVGDDLTVTNPK	95.0%	119.0	21.7	186	36	0	2	2,033.06
									FTASAGIQVVGDDLTVTNPKR	95.0%	95.8	20.3	1	7	0	2	2,189.16
									GNPTVEVDLFTSK	95.0%	110.0	22.9	136	0	0	2	1,406.72
									GVPLYR	95.0%	31.4	21.6	4	0	0	2	704.41
									HIADLAGNSEVILPVPAFNVINGGSHAGNK	95.0%	64.8	21.0	0	2	0	2	3,011.57
									IDKLMIEMDGTENK	95.0%	70.7	21.8	16	31	0	2	1,668.78
									IEEELGSK	95.0%	45.6	23.8	5	0	0	2	904.46
									IGAEVYHNLK	95.0%	68.1	22.8	116	48	0	2	1,143.62
									KLNVTEQEK	95.0%	48.3	23.4	16	4	0	2	1,088.60
									LAMQEFMILPVGAANFR	95.0%	112.0	21.3	145	30	0	2	1,923.98
									LMIEMDGTENK	95.0%	79.9	18.5	58	0	0	2	1,312.58
									LNVTEQEK	95.0%	55.2	23.7	5	0	0	2	960.50
									LNVTEQEKIDK	95.0%	64.4	22.8	5	1	0	2	1,316.71
									NFRNPLAK	95.0%	48.2	22.2	2	0	0	2	959.54
									SGETEDTFIADLVVGLCTGQIK	95.0%	83.2	22.3	7	1	0	2	2,353.16
									SGKYDLDFK	95.0%	37.0	21.5	7	16	0	2	1,072.53
									TIAPALVSK	95.0%	50.6	15.9	7	0	0	2	899.56
									VNQIGSVTESLQACK	95.0%	116.0	22.8	40	0	0	2	1,633.82

									VVIGMDVAASEFFR	95.0%	117.0	22.6	159	0	0	2	1,556.78
									YDLDFK	95.0%	34.7	19.7	4	0	0	2	800.38
									YDLDFKSPDDPSR	95.0%	48.6	20.3	8	8	0	2	1,554.71
									YGKDATNVGDEGGFAPNILENK	95.0%	117.0	21.8	2	4	0	2	2,309.11
									YISPDQLADLYK	95.0%	87.5	22.9	144	0	0	2	1,425.73
									YNQLLR	95.0%	37.7	22.8	44	0	0	2	806.45
N-acetylglucosamine-1-phosphotransferase subunit gamma	GNPTG_HUMAN	GNPTG	33,956	100.00%	5	7	23	13.80%	DPSPVSGPVHLFR	95.0%	34.5	22.1	1	3	0	2	1,407.74
									MKVVEEPNAFGVNNPFLPQASR	95.0%	38.8	21.0	0	1	0	2	2,460.23
									RDPSPVSGPVHLFR	95.0%	58.5	21.3	0	4	0	2	1,563.84
									SPEQLR	95.0%	48.9	20.7	6	0	0	2	729.39
									VVEEPNAFGVNNPFLPQASR	95.0%	83.6	22.1	6	2	0	2	2,185.10
Follistatin-related protein 1	FSTL1_HUMAN	FSTL1	34,967	100.00%	3	3	17	9.09%	GAQTQTEEEMTR	95.0%	73.2	17.7	6	0	0	2	1,396.60
									LDSSEFLK	95.0%	49.5	22.3	4	0	0	2	938.48
									LSFQEFLK	95.0%	54.8	21.0	7	0	0	2	1,011.55
UDP-N-acetylhexosamine pyrophosphorylase	UAP1_HUMAN	UAP1	58,752	100.00%	15	15	82	36.00%	AIEGFNQSSHQK	95.0%	55.3	22.5	5	0	0	2	1,345.65
									ALAAQNIVEDMEQR	95.0%	106.0	21.8	6	0	0	2	1,603.78
									DVVNVYEPQLQHHVAQK	95.0%	54.5	22.5	1	0	0	2	2,004.03
									ENVIFFQQGMLPAMSFDGK	95.0%	105.0	21.2	6	0	0	2	2,191.02
									EVLGSATR	95.0%	39.3	24.9	2	0	0	2	832.45
									FVFDIFQFAK	95.0%	58.6	22.3	23	0	0	2	1,261.66
									GMYDVGLPSR	95.0%	47.3	21.5	4	0	0	2	1,110.53
									KENVIFFQQGMLPAMSFDGK	95.0%	44.9	21.7	0	1	0	2	2,319.12
									KIPYVDTQGQLIKPDKPNGIK	95.0%	31.6	13.8	0	0	1	2	2,352.33
									LLFNAGNIANHFFTVPFLR	95.0%	26.2	19.1	0	1	0	2	2,191.18
									LQQVAEK	95.0%	55.3	21.5	7	0	0	2	815.46
									NADSQNGKDNPTTAR	95.0%	29.8	20.6	0	1	0	2	1,588.73
									TLFQIQAER	95.0%	58.4	22.3	6	0	0	2	1,105.60
									VAVLLLAGGQGTR	95.0%	87.6	17.4	14	0	0	2	1,254.75
									VSMAPDGNGGLYR	95.0%	59.7	21.5	4	0	0	2	1,352.63
Cysteine and histidine-rich domain-containing protein 1	CHRD1_HUMAN	CHORDC1	37,472	99.90%	2	2	6	9.04%	FQEHIIQAPKPVEAIK	95.0%	30.7	16.7	0	4	0	2	1,848.04
									RPSPDEPMTNLELK	95.0%	51.9	22.2	2	0	0	2	1,642.81
Adenylosuccinate synthetase isozyme 2	PURA2_HUMAN	ADSS	50,080	100.00%	6	7	25	19.10%	DGVYFLYEALHGPPK	95.0%	41.6	22.5	1	0	0	2	1,705.86
									ELPVNAQNYVR	95.0%	64.8	23.2	4	0	0	2	1,302.68
									FIEDELQIPVK	95.0%	66.6	22.5	4	0	0	2	1,330.73
									LDGEIIPHIPANQEVLNK	95.0%	39.0	18.7	0	4	0	2	2,000.08
									LDILDMFTEIK	95.0%	77.0	21.8	4	0	0	2	1,353.70
									VGIGAFPTEQDNEIGELLQTR	95.0%	109.0	21.0	4	4	0	2	2,287.16
Methylosome protein 50	MEP50_HUMAN	WDR77	36,705	100.00%	2	2	3	7.89%	ETPPPLVPPAAR	95.0%	40.4	21.0	1	0	0	2	1,244.70
									SDGALLLGASSLSGR	95.0%	93.0	22.6	2	0	0	2	1,403.75
Proteasome subunit beta type-2	PSB2_HUMAN	PSMB2	22,820	100.00%	8	11	26	32.80%	AVELLR	95.0%	36.1	22.0	4	0	0	2	700.44
									FILNLPTFSVR	95.0%	48.1	17.0	2	0	0	2	1,306.75
									IIDKNGIHDLDNISFPK	95.0%	62.9	20.8	2	2	0	2	1,939.03
									MRNGYELSPTAAANFTR	95.0%	45.3	21.8	0	2	0	2	1,914.91
									NGIHDLDNISFPK	95.0%	71.9	22.2	2	1	0	2	1,469.74
									NGYELSPTAAANFTR	95.0%	92.2	22.0	2	0	0	2	1,611.78
									VAASNIVQMK	95.0%	83.1	23.4	6	0	0	2	1,076.58
									VAASNIVQMKDDHDK	95.0%	62.0	21.7	2	1	0	2	1,686.81
Cathepsin H	CATH_HUMAN	CTSH	37,376	100.00%	6	8	72	18.80%	GIMGEDTYPYQGK	95.0%	76.8	19.0	23	0	0	2	1,474.65
									GNFVSPVK	95.0%	32.2	23.6	2	0	0	2	847.47
									MALNQFSDMSFAEIK	95.0%	99.4	21.9	20	0	0	2	1,763.80
									TPDKVNHAVLAVGYGEK	95.0%	85.8	20.4	4	8	5	2	1,797.95

									TYSTEEYHHR	95.0%	58.2	19.3	6	0	0	2	1,322.58
									VNHAVLAVGYGEK	95.0%	70.8	22.1	4	0	0	2	1,356.73
Dynamin-like 120 kDa protein, mitochondrial	OPA1_HUMAN	OPA1	111,616	100.00%	4	4	8	6.56%	AKNEILDEVISLSQVTPK	95.0%	117.0	17.9	2	0	0	2	1,984.10
									ALGYFAVVTGK	95.0%	33.0	20.3	2	0	0	2	1,125.63
									LDAFIEALHQEK	95.0%	37.7	21.8	1	0	0	2	1,413.74
									MVLVDLPGVINTVTSGMAPDTK	95.0%	90.4	21.4	3	0	0	2	2,290.17
Cathepsin L2	CATL2_HUMAN	CTSL2	37,311	100.00%	6	9	78	29.00%	AVATVGPISVAMDAGHSSFQFYK	95.0%	114.0	21.5	10	1	0	2	2,399.17
									FDQNLDTK	95.0%	37.8	22.0	8	0	0	2	980.47
									HGFTMAMNAFGDMTNEEFR	95.0%	87.9	13.4	6	6	0	2	2,253.90
									MIELHNGEYSQGK	95.0%	58.9	21.1	8	0	0	2	1,521.70
									NLDHGVLVVGYGFEGANSNNSK	95.0%	68.2	21.6	0	6	0	2	2,291.11
									VFREPLFLDLPK	95.0%	40.1	18.2	5	28	0	2	1,473.85
Ras suppressor protein 1	RSU1_HUMAN	RSU1	31,524	100.00%	3	3	6	15.90%	ALYLSDNDFEILPPDIGK	95.0%	31.9	22.3	1	0	0	2	2,020.03
									EIGELTQLK	95.0%	36.0	21.9	2	0	0	2	1,030.58
									LTVLPPELGNLDLTGQK	95.0%	45.9	15.7	3	0	0	2	1,808.02
FACT complex subunit SPT16	SP16H_HUMAN	SUPT16H	119,899	100.00%	6	7	19	6.88%	AASITSEVFNK	95.0%	81.0	22.7	2	0	0	2	1,166.61
									GNENANGAPAITLLIR	95.0%	75.6	20.2	4	0	0	2	1,623.88
									LAESVEK	95.0%	45.3	25.1	2	0	0	2	775.42
									NEGNIFPNPEATFVK	95.0%	61.0	22.3	3	0	0	2	1,676.83
									NLGFGMGIEFR	95.0%	44.0	21.9	4	0	0	2	1,256.61
									VMEIVDADEKVR	95.0%	63.1	22.9	2	2	0	2	1,419.72
Prostaglandin-H2 D-isomerase	PTGDS_HUMAN	PTGDS	21,011	99.50%	2	2	7	17.40%	AQGFTEDTIVFLPQTDK	95.0%	49.7	22.4	2	0	0	2	1,909.96
									TMLLQPAGSLGSYSYR	95.0%	107.0	22.5	5	0	0	2	1,759.87
ELAV-like protein 1	ELAV1_HUMAN	ELAVL1	36,075	100.00%	5	5	40	24.80%	NVALLSQLYHSPAR	95.0%	74.8	20.8	4	0	0	2	1,568.85
									SEAEEAITSFNGHKPPGSSEPITVK	95.0%	73.0	22.0	0	5	0	2	2,612.28
									SLFSSIGEVESAK	95.0%	80.2	22.1	12	0	0	2	1,353.69
									TNLIVNYLPQNMTQDELR	95.0%	77.5	23.3	7	0	0	2	2,178.09
									VLVDQTTGLSR	95.0%	78.8	22.3	12	0	0	2	1,188.66
Vacuolar protein sorting-associated protein VTA1 homolog	VTA1_HUMAN	VTA1	33,862	100.00%	3	3	3	13.00%	LMDQLEALKK	95.0%	36.9	22.5	1	0	0	2	1,204.66
									MFLYADNEDR	95.0%	32.1	17.1	1	0	0	2	1,289.55
									YAGSALQYEDVSTAVQNLQK	95.0%	90.6	22.1	1	0	0	2	2,185.08
Interleukin enhancer-binding factor 3	ILF3_HUMAN	ILF3	95,321	100.00%	13	14	116	24.80%	AYAALAALEK	95.0%	87.7	20.2	15	0	0	2	1,020.57
									DSSKGEDSAEETEAKPAVVAPAPVVEAVSTPSAAFPSDATAEQGPILTK	95.0%	18.5	17.1	0	0	1	2	4,852.39
									EATDAIGHLDR	95.0%	64.7	22.6	10	0	0	2	1,197.59
									EDITQSAQHALR	95.0%	67.4	23.4	14	0	0	2	1,368.69
									FVMEVEVDGQK	95.0%	68.3	22.4	6	0	0	2	1,296.61
									HSSVYPTQEELEAVQNMVSHTER	95.0%	39.3	19.6	0	3	1	2	2,687.24
									LAAFGQLHK	95.0%	51.6	20.4	8	0	0	2	984.56
									LFPDTPLALDANK	95.0%	67.3	22.3	8	0	0	2	1,414.76
									LNQLKPGLQYK	95.0%	38.1	18.4	5	0	0	2	1,301.76
									LVSQTGPVHAPIFTMSVEVDGNSFEASGPSK	95.0%	55.7	20.8	0	2	0	2	3,204.55
									VLAGETLSVNDPPDVLDR	95.0%	103.0	21.2	24	0	0	2	1,909.99
									VLGMDPLPSK	95.0%	50.4	27.5	5	0	0	2	1,072.57
									VLQDMGLPTGAEGR	95.0%	69.4	23.2	14	0	0	2	1,459.72
Carbonic anhydrase-related protein 11	CAH11_HUMAN	CA11	36,221	100.00%	3	4	32	14.00%	GTLYNTGR	95.0%	41.9	22.2	2	0	0	2	881.45
									LLSQNPPSQIFQSLSGNSRPLQPLAHR	95.0%	55.7	17.3	0	4	13	2	2,985.60
									VLYDPFLPPLR	95.0%	57.5	19.4	13	0	0	2	1,329.76
Palmitoyl-protein thioesterase 1	PPT1_HUMAN	PPT1	34,176	100.00%	7	9	69	29.10%	CPSPPMINLISVGGQHQGVFGLPR	95.0%	54.8	20.6	0	3	0	2	2,577.31
									ETIPLQETSLYTQDR	95.0%	98.4	22.4	11	3	0	2	1,793.89
									GINESYKK	95.0%	31.3	20.3	1	0	0	2	938.49
									IPGIYVLSLEIGK	95.0%	67.1	12.0	13	0	0	2	1,401.84

									KIPGIYVLSLEIGK	95.0%	67.1	8.5	0	19	0	2	1,529.93
									KTLNAGAYSK	95.0%	31.4	20.0	1	0	0	2	1,052.57
									LQQGYNAMGFSQGGQFLR	95.0%	126.0	21.2	17	1	0	2	2,017.96
Myosin-10	MYH10_HUMAN	MYH10	228,987	100.00%	13	13	36	12.60%	AGVLAHLEEER	95.0%	52.1	22.8	2	0	0	2	1,223.64
									ALELDPNLYR	95.0%	41.9	23.2	4	0	0	2	1,203.64
									ELQAQIAELQEDFESEK	95.0%	87.9	22.4	4	0	0	2	2,006.96
									EQADFAVEALAK	95.0%	32.4	24.1	1	0	0	2	1,291.65
									IVFQEFR	95.0%	35.4	21.5	1	0	0	2	938.51
									IVGLDQVTGMTETAFGSAYK	95.0%	119.0	21.3	7	0	0	2	2,104.03
									KFDQLLAEEK	95.0%	34.1	22.6	1	0	0	2	1,220.65
									KLQAQMK	95.0%	39.1	22.8	1	0	0	2	846.49
									LDPHLVLDQLR	95.0%	47.1	18.8	4	10	0	2	1,318.75
									MQAHIQDLEEQLDEEEGAR	95.0%	54.3	19.7	0	1	0	2	2,257.00
									NLPIYSENIIEMYR	95.0%	82.8	22.6	4	0	0	2	1,770.87
									NMDPLNDNVATLLHQSSDR	95.0%	37.6	21.6	0	1	0	2	2,156.00
									NTDQASMPENTVAQK	95.0%	35.6	20.1	1	0	0	2	1,649.74
									QLLQANPILESFGNAK	95.0%	68.0	20.0	2	0	0	2	1,742.94
									SDLLLEGFNNYR	95.0%	58.5	22.1	1	0	0	2	1,440.71
									SLEAEILQLQEELASSER	95.0%	36.4	22.1	2	0	0	2	2,045.04
									TQLEELEDELQATEDAK	95.0%	114.0	21.0	3	0	0	2	1,961.92
									VVSSVLQFGNISFK	95.0%	76.9	18.9	7	0	0	2	1,524.84
X-ray repair cross-complementing protein 5	XRCC5_HUMAN	XRCC5	82,689	100.00%	13	15	49	20.40%	ANPQVGVAFPHIK	95.0%	53.8	20.0	6	0	0	2	1,377.76
									DKPSGDTAAVFEEGGDVDDLLDMI	95.0%	40.6	19.4	1	0	0	2	2,525.12
									EEASGSSVTAEEAK	95.0%	94.9	20.4	2	0	0	2	1,394.63
									EEASGSSVTAEEAKK	95.0%	73.3	21.9	8	0	0	2	1,522.72
									FSEEQR	95.0%	32.2	20.3	1	0	0	2	795.36
									HIEIFTDLSSR	95.0%	50.0	23.4	2	0	0	2	1,317.68
									HLMLPDFDLLEDIESK	95.0%	71.5	21.9	5	10	0	2	1,930.95
									KYAPTEAQLNAVDALIDSMSLAK	95.0%	57.6	21.0	0	3	0	2	2,465.26
									LGGHGPSFPLK	95.0%	39.4	20.8	1	4	0	2	1,109.61
									TDTLEDLFPTTK	95.0%	58.4	22.6	2	0	0	2	1,380.69
									VITMFVQR	95.0%	45.8	21.6	1	0	0	2	1,009.55
									YAPTEAQLNAVDALIDSMSLAK	95.0%	108.0	21.5	2	0	0	2	2,337.17
									YGSDIVPFSK	95.0%	59.9	21.9	1	0	0	2	1,112.56
Ribonuclease inhibitor	RINI_HUMAN	RNH1	49,956	100.00%	4	4	19	10.40%	ELSLAGNELGDEGAR	95.0%	109.0	21.6	7	0	0	2	1,530.74
									ELTVSNNDINEAGVR	95.0%	116.0	22.3	5	0	0	2	1,630.80
									LEDAGVR	95.0%	40.2	23.4	2	0	0	2	759.40
									VNPALAELNLR	95.0%	88.1	17.7	5	0	0	2	1,209.70
THO complex subunit 4	THOC4_HUMAN	THOC4	26,871	100.00%	3	3	5	16.70%	AAVHYDR	95.0%	38.3	21.0	2	0	0	2	831.41
									QYNGVPLDGRPMNIQLVTSQIDAQR	95.0%	33.3	20.3	0	1	0	2	2,829.43
									SLGTADVHFER	95.0%	53.2	23.1	2	0	0	2	1,231.61
Dystroglycan	DAG1_HUMAN	DAG1	97,424	100.00%	7	7	88	12.00%	EGAMSAQLGYPVVGWHIANK	95.0%	52.0	22.5	0	5	0	2	2,144.06
									IDLLHR	95.0%	40.2	17.9	11	0	0	2	766.46
									IPSDTFYDHEDTTTDKLK	95.0%	30.1	21.5	0	1	0	2	2,125.99
									LFDMSAFMAGPGNAK	95.0%	97.7	20.1	18	0	0	2	1,588.71
									LGCSLNQNSVPDIHGVEAPAR	95.0%	57.0	22.0	0	4	0	2	2,234.10
									SFSEVELHNMK	95.0%	48.0	21.4	12	0	0	2	1,336.62
									VTIPTDLIASSGDIIK	95.0%	131.0	18.2	37	0	0	2	1,642.93
Exportin-4	XPO4_HUMAN	XPO4	130,125	99.50%	2	2	3	2.00%	IPQLPEDLFK	95.0%	35.5	22.1	1	0	0	2	1,199.67
									SPPLNFLSSPVQR	95.0%	60.6	21.3	2	0	0	2	1,441.78
Stathmin	STMN1_HUMAN	STMN1	17,285	100.00%	9	10	16	55.70%	AIEENNNFSK	95.0%	38.2	22.0	2	0	0	2	1,165.55

									ASGQAFELILSPR	95.0%	44.6	22.4	2	0	0	2	1,388.75
									DLSLEEIQK	95.0%	50.5	24.6	1	0	0	2	1,074.57
									EHEKEVLQK	95.0%	32.3	22.1	1	0	0	2	1,139.61
									ESKDPADETEAD	95.0%	42.5	13.4	1	0	0	2	1,306.53
									KLEAAEER	95.0%	63.9	23.2	2	0	0	2	945.50
									LEAAEER	95.0%	39.5	21.7	1	0	0	2	817.41
									SHEAEVLK	95.0%	33.0	20.7	2	0	0	2	912.48
									SKESVPEFPLSPPK	95.0%	41.7	21.7	2	2	0	2	1,541.82
Selenide, water dikinase 1	SPS1_HUMAN	SEPHS1	42,893	100.00%	6	6	13	27.00%	DKVMPLIIQGFK	95.0%	33.1	18.9	1	0	0	2	1,404.79
									ESFNPESYELDK	95.0%	61.0	19.2	4	0	0	2	1,457.64
									HGGLSLVQTTDYIYPIVDDPYMMGR	95.0%	42.5	20.4	0	1	0	2	2,873.35
									IIEVAPQVATQNVNPTPGATS	95.0%	62.7	21.4	2	0	0	2	2,107.10
									LVVTQEDVELAYQEAMMNMAR	95.0%	65.1	20.1	0	3	0	2	2,489.14
									NEVSFVIHNLPVLAK	95.0%	39.0	17.1	0	2	0	2	1,679.95
Polymerase I and transcript release factor	PTRF_HUMAN	PTRF	43,459	99.50%	2	2	3	8.97%	IIGAVDQIQLTQAQLEER	95.0%	98.7	18.8	2	0	0	2	2,025.10
									QAEMEGAVQSIQGELSK	95.0%	57.2	22.8	1	0	0	2	1,820.87
Dihydropteridine reductase	DHPR_HUMAN	QDPR	25,772	100.00%	4	6	9	28.70%	EGGLLTLAGAK	95.0%	50.8	19.6	2	0	0	2	1,029.59
									MTDSFTEQADQVTAEVGK	95.0%	141.0	19.4	2	0	0	2	1,972.88
									NRPSSGSLIQVVTTEGR	95.0%	57.4	20.8	1	2	0	2	1,800.96
									NSGMPPGAAAIAVLPVTLDTPMNR	95.0%	90.4	21.6	1	1	0	2	2,425.22
Cathepsin Z	CATZ_HUMAN	CTSZ	33,850	100.00%	7	10	134	25.70%	GDGLAPLGR	95.0%	49.6	20.4	9	0	0	2	855.47
									IVTSTYK	95.0%	33.6	17.8	3	0	0	2	811.46
									IVTSTYKDGK	95.0%	56.9	20.6	10	0	0	2	1,111.60
									MMAEIYANGPISCGIMATER	95.0%	88.3	18.9	1	1	0	2	2,262.99
									NVDGVNYASITR	95.0%	91.1	22.9	23	0	0	2	1,308.65
									STYPRPHEYLSPADLPK	95.0%	56.6	22.2	5	35	12	2	1,971.00
									VGDYGSLSGR	95.0%	96.4	21.6	35	0	0	2	1,010.49
Neogenin	NEO1_HUMAN	NEO1	159,999	100.00%	11	13	30	10.70%	DVVASLVSTR	95.0%	66.1	21.8	7	0	0	2	1,046.58
									GMGPMSEAVQFR	95.0%	61.0	19.4	2	0	0	2	1,341.59
									GYAIGYGIGSPHAQTIK	95.0%	70.7	22.2	1	1	0	2	1,732.90
									HGPGVSTPDVAVR	95.0%	57.8	23.2	2	0	0	2	1,291.68
									KSDVTETLVSGTQLSQLIEGLDR	95.0%	27.4	19.3	0	2	0	2	2,489.31
									LIVAGLPR	95.0%	40.5	12.6	2	0	0	2	838.55
									NEEALDTESSER	95.0%	48.8	17.9	2	0	0	2	1,379.59
									QLLPDGSLFISNVVHSK	95.0%	57.7	19.8	1	2	0	2	1,854.01
									SGSAPQSPGASIR	95.0%	61.2	22.0	4	0	0	2	1,214.61
									VETQPEVQLPGPAPNLR	95.0%	41.8	20.5	1	0	0	2	1,844.99
									VLPDPEVISDLVFLK	95.0%	52.4	17.9	3	0	0	2	1,683.96
Myosin light polypeptide 6	MYL6_HUMAN	MYL6	16,912	100.00%	5	7	17	41.70%	ALGQNPTNAEVLK	95.0%	61.5	21.7	2	0	0	2	1,354.73
									EAFQLFDR	95.0%	42.3	22.7	2	0	0	2	1,025.51
									ILYSQCGDVMR	95.0%	49.9	20.5	2	0	0	2	1,357.62
									NKDQGTYEDYVEGLR	95.0%	98.1	21.5	2	2	0	2	1,786.83
									VLDFEHFLPMLQTVAK	95.0%	49.6	21.2	4	3	0	2	1,888.00
5'-3' exoribonuclease 2	XRN2_HUMAN	XRN2	108,568	100.00%	2	2	7	3.37%	AALEEVYPDLTPEETRR	95.0%	29.0	21.8	0	3	0	2	1,988.99
									ELTMASLPFTFDVER	95.0%	64.8	22.1	4	0	0	2	1,771.86
Transcriptional activator protein Pur-beta	PURB_HUMAN	PURB	33,224	100.00%	4	4	9	27.20%	DSLGDFIEHYAQLGPSSPEQLAAGAEEGGGPR	95.0%	47.7	20.2	0	2	0	2	3,255.52
									GGGEQETQELASK	95.0%	48.0	22.4	2	0	0	2	1,333.62
									GGGGFGAGPGPGGLQSGQTIALPAQGLIEFR	95.0%	58.2	19.8	0	2	0	2	2,867.48
									VSEVKPSYR	95.0%	47.1	22.9	3	0	0	2	1,064.57
Tubulin-folding cofactor B	TBCB_HUMAN	TBCB	27,308	100.00%	2	2	2	8.61%	AQQEAEAAQR	95.0%	54.0	20.4	1	0	0	2	1,101.53
									YTISQEAYDQR	95.0%	59.4	20.0	1	0	0	2	1,373.63

V-type proton ATPase subunit S1	VAS1_HUMAN	ATP6AP1	52,009	100.00%	6	7	21	18.50%	EVLTGNDEVIGQVLSTLK	95.0%	83.0	19.2	5	0	0	2	1,915.04
									LGASPLHVDLATLR	95.0%	72.1	18.3	2	4	0	2	1,462.84
									LPYTASSGLMAPR	95.0%	73.3	23.0	4	0	0	2	1,379.70
									LSIEDFTAYGGVFGNK	95.0%	39.1	22.7	2	0	0	2	1,717.84
									NVLLFLQDK	95.0%	50.2	20.7	2	0	0	2	1,089.63
									SEDVPYTAALTAVRPSR	95.0%	38.5	21.3	2	0	0	2	1,832.95
Actin-related protein 2/3 complex subunit 3	ARPC3_HUMAN	ARPC3	20,530	100.00%	2	2	2	15.20%	ETKDTDIVDEAIYYFK	95.0%	66.1	22.3	1	0	0	2	1,949.94
									VFDPQNDKPSK	95.0%	38.2	22.3	1	0	0	2	1,274.64
60 kDa SS-A/Ro ribonucleoprotein	RO60_HUMAN	TROVE2	60,654	100.00%	6	7	18	13.20%	ALDAAFYK	95.0%	33.4	21.5	1	0	0	2	898.47
									ALLQEMPLTALLR	95.0%	59.1	18.9	4	0	0	2	1,484.85
									GGMALALAVTK	95.0%	57.0	21.8	2	0	0	2	1,047.59
									IPTHLFTFIQFK	95.0%	42.0	18.1	2	1	0	2	1,491.84
									LGLENAEALIR	95.0%	82.5	21.1	5	0	0	2	1,198.68
									LSHLKPSSEGLAIVTK	95.0%	75.7	15.2	0	3	0	2	1,679.97
Ankyrin repeat and FYVE domain-containing protein 1	ANFY1_HUMAN	ANKFY1	128,384	100.00%	3	3	5	3.51%	ELDLSDANPEVTMTMLR	95.0%	52.2	21.3	1	0	0	2	1,966.91
									GDLFAATFLIK	95.0%	39.7	19.2	2	0	0	2	1,195.67
									RLESIATTLVSHK	95.0%	37.8	18.6	0	2	0	2	1,454.83
Histone-binding protein RBBP7	RBBP7_HUMAN	RBBP7	47,802	100.00%	3	3	9	16.50%	EMFEDTVEER	95.0%	56.3	17.1	3	0	0	2	1,300.54
									IGEEQSAEDAEDGPPELLFIHGGHTAK	95.0%	89.1	20.6	0	5	0	2	2,847.34
									TPSSDVLVFDYTK	95.0%	104.0	23.0	12	0	0	2	1,471.73
									TVALWDLR	95.0%	60.2	23.2	4	0	0	2	973.55
									YMPQNPHIIATK	95.0%	37.2	22.0	1	0	0	2	1,428.73
Syntenin-1	SDCB1_HUMAN	SDCBP	32,427	100.00%	4	5	15	29.20%	DSTGHVGFIFK	95.0%	41.4	22.1	3	0	0	2	1,207.61
									LYPELSQYMGLSLNEEEIR	95.0%	81.9	22.1	3	0	0	2	2,300.11
									SIDNGIFVQLVQANSPASLVGLR	95.0%	126.0	15.9	5	3	0	2	2,398.31
									VIQAQTAFSANPANPAILSEASAPIPHDGNLYPR	95.0%	83.6	19.1	0	1	0	2	3,530.80
60S ribosomal protein L27	RL27_HUMAN	RPL27	15,780	100.00%	3	4	6	19.10%	VKFEER	95.0%	39.7	23.3	1	0	0	2	807.44
									VYNYNHLMPTR	95.0%	38.0	21.1	2	1	0	2	1,423.68
									YSVDIPLDK	95.0%	34.3	23.0	2	0	0	2	1,049.55
DnaJ homolog subfamily C member 9	DNJC9_HUMAN	DNAJC9	29,892	100.00%	2	2	4	11.90%	ISLEDIQAFEK	95.0%	75.3	23.2	2	0	0	2	1,292.67
									NIIQQAIDAGEVPSYNAFVK	95.0%	77.9	20.5	2	0	0	2	2,177.12
UPF0160 protein MYG1, mitochondrial	MYG1_HUMAN	C12orf10	42,432	100.00%	3	3	6	12.50%	AMDLVQEEFLQR	95.0%	90.8	22.8	2	0	0	2	1,494.73
									FQVDPSGEIVELAK	95.0%	71.5	23.5	2	0	0	2	1,531.80
									LLAQLLGTSEEDSMVGTLYDK	95.0%	92.6	21.7	2	0	0	2	2,299.14
26S proteasome non-ATPase regulatory subunit 13	PSD13_HUMAN	PSMD13	42,901	100.00%	9	10	24	28.70%	ALSVGLVK	95.0%	40.6	17.9	1	0	0	2	786.51
									ETIEDVEEMLNNLPGVTSVHSR	95.0%	61.1	21.4	0	2	0	2	2,485.19
									ITVNEVELLVMK	95.0%	96.8	19.5	6	0	0	2	1,403.78
									LNIGDLQVTK	95.0%	75.4	21.9	3	0	0	2	1,100.63
									LYENFISEFEHR	95.0%	61.3	21.6	1	2	0	2	1,583.75
									QLTFEEIAK	95.0%	40.3	23.0	2	0	0	2	1,078.58
									QMTDPNVALTFLEK	95.0%	74.5	22.8	3	0	0	2	1,622.81
									VLDLQQIK	95.0%	51.3	16.9	2	0	0	2	956.58
									YYQTIGNHASYYK	95.0%	51.0	21.3	2	0	0	2	1,607.75
Glutaminyl-tRNA synthetase	SYQ_HUMAN	QARS	87,782	100.00%	6	7	14	9.55%	ATGILLYGLASR	95.0%	67.4	17.7	4	0	0	2	1,234.72
									FDDTNPEKEEAK	95.0%	54.3	20.5	2	1	0	2	1,422.64
									GFHQVPFAPIVFIER	95.0%	44.7	19.6	0	2	0	2	1,756.95
									HTGYVIELQHVVK	95.0%	52.2	20.6	0	2	0	2	1,522.84
									LFTLTALR	95.0%	42.7	14.9	2	0	0	2	934.57
									LVMEDGKMDPVAYR	95.0%	30.8	21.4	0	1	0	2	1,655.78
60S ribosomal protein L17	RL17_HUMAN	RPL17	21,379	100.00%	2	2	3	13.00%	EQIVPKPEEEVAQK	95.0%	42.6	21.0	2	0	0	2	1,623.86
									YSLDPENPTK	95.0%	51.8	22.4	1	0	0	2	1,163.56

Leucine-rich alpha-2-glycoprotein	A2GL_HUMAN	LRG1	38,162	100.00%	5	5	28	16.10%	ALGHLDLSGNR	95.0%	34.7	22.5	0	3	0	2	1,152.61
									DLLLPQPDLR	95.0%	45.7	19.0	4	0	0	2	1,179.67
									GPLQLER	95.0%	45.3	20.3	1	0	0	2	812.46
									TLDLGENQLETLPPDLLR	95.0%	66.4	19.9	8	0	0	2	2,037.09
									VAAGAFQGLR	95.0%	86.6	22.8	12	0	0	2	989.55
Desmoglein-2	DSG2_HUMAN	DSG2	122,276	100.00%	3	3	9	3.94%	GITEPPFGIFVFNK	95.0%	53.7	20.4	4	0	0	2	1,565.84
									ILDVNDNIPVVENK	95.0%	45.9	21.0	1	0	0	2	1,581.85
									IVSLEPAYPPVFYLNK	95.0%	40.2	18.7	4	0	0	2	1,850.01
Hsc70-interacting protein	F10A1_HUMAN	ST13	41,314	100.00%	7	7	70	20.10%	AIDLFTDAIK	95.0%	75.3	21.9	23	0	0	2	1,106.61
									AIEINPDSAQPYK	95.0%	60.5	22.5	17	0	0	2	1,445.73
									KVNELR	95.0%	36.2	23.8	1	0	0	2	758.45
									LAILYAK	95.0%	37.0	13.6	3	0	0	2	791.50
									LDYDEDASAMLK	95.0%	69.1	19.8	2	0	0	2	1,386.61
									QDPSVLHTEEMR	95.0%	35.2	20.5	1	0	0	2	1,457.67
									VAAIEALNDGELQK	95.0%	109.0	22.3	23	0	0	2	1,470.78
4F2 cell-surface antigen heavy chain	4F2_HUMAN	SLC3A2	67,978	100.00%	3	3	13	6.03%	GQSEDPGSLLSLFR	95.0%	85.4	22.8	9	0	0	2	1,505.76
									LKLEPHEGLLLR	95.0%	27.1	10.8	0	1	0	2	1,417.85
									LLTSFLPAQLLR	95.0%	45.3	12.0	3	0	0	2	1,371.84
Serpin B6	SPB6_HUMAN	SERPINB6	42,605	100.00%	19	24	375	65.40%	ADFSGMSQTDLSLSK	95.0%	100.0	19.5	30	0	0	2	1,602.73
									ELNMIIMLPDETTDLR	95.0%	103.0	22.6	15	1	0	2	1,935.94
									FCADHPFLFFIQHSK	95.0%	31.2	22.2	0	2	0	2	1,893.91
									FKLEESYDMESVLR	95.0%	69.8	22.6	17	41	0	2	1,761.84
									FYQAEMEELDFISAVEK	95.0%	117.0	20.3	60	0	0	2	2,064.95
									GNTAAQMAQILSFNK	95.0%	95.8	21.6	20	0	0	2	1,609.80
									IAELLSPGSVDPLTR	95.0%	79.5	19.4	30	0	0	2	1,567.87
									LDMMDEEEVEVSLPR	95.0%	82.8	21.1	23	0	0	2	1,823.80
									LEESYDMESVLR	95.0%	51.0	20.5	1	0	0	2	1,486.67
									LVLVNAVYFR	95.0%	73.2	16.8	33	0	0	2	1,193.70
									NEEKPVQMMFK	95.0%	35.4	21.3	5	0	0	2	1,412.66
									NLGMTDAFELGK	95.0%	62.2	22.1	12	0	0	2	1,311.63
									SCDFLSSFR	95.0%	47.2	18.2	2	0	0	2	1,118.49
									SFVEVNEEGTEAAAATAAIMMMR	95.0%	111.0	19.1	9	14	0	2	2,477.10
									SGGGGDIHQGFQSLLTEVNK	95.0%	107.0	21.8	8	22	0	2	2,044.01
									TGTQYLLR	95.0%	66.4	20.4	8	0	0	2	951.53
									TVEKELTYEK	95.0%	45.0	21.7	11	0	0	2	1,239.65
									TYIGEIFTQILVLPYVGK	95.0%	77.1	15.3	4	0	0	2	2,054.16
									VSKNEEKPVQMMFK	95.0%	47.6	22.5	4	3	0	2	1,726.85
Mitotic checkpoint protein BUB3	BUB3_HUMAN	BUB3	37,137	100.00%	4	5	9	17.40%	LNQPPEDGISSVK	95.0%	55.7	22.4	2	0	0	2	1,383.71
									MHDLNTDQENLVGTHDAPIR	95.0%	51.5	20.6	0	2	2	2	2,292.07
									VAVEYLDPSPEVQK	95.0%	47.2	23.3	2	0	0	2	1,573.81
									VYTLSVSGDR	95.0%	51.0	21.6	1	0	0	2	1,096.56
Glutaminyl-peptide cyclotransferase	QPCT_HUMAN	QPCT	40,860	100.00%	7	10	82	27.10%	DHSLEGR	95.0%	31.7	20.4	1	0	0	2	813.39
									LQAIEHELHELGLLK	95.0%	68.3	17.2	10	13	1	2	1,742.98
									MASTPHPPGAR	95.0%	59.9	22.6	26	9	0	2	1,137.55
									SFSNIISTLNPTAK	95.0%	93.8	22.2	15	0	0	2	1,492.80
									VFVGATDSAVPCAMMLELAR	95.0%	80.8	21.5	2	0	0	2	2,170.03
									YFQNYSYGGVIQDDHIPFLR	94.8%	25.7	22.0	0	1	0	2	2,432.17
									YPGSPGSYAAR	95.0%	57.9	21.7	4	0	0	2	1,125.53
Splicing factor, arginine/serine-rich 6	SFRS6_HUMAN	SFRS6	39,570	100.00%	3	3	10	8.72%	LIVENLSSR	95.0%	60.6	21.8	8	0	0	2	1,030.59
									QAGEVTYADAHKER	95.0%	32.6	21.8	0	1	0	2	1,574.76
									VIVEHAR	95.0%	31.9	18.7	1	0	0	2	823.48

Protein arginine N-methyltransferase 5	ANM5_HUMAN	PRMT5	72,667	100.00%	7	8	14	12.90%	AAILPTSIFLTNK	95.0%	78.4	16.7	2	0	0	2	1,388.82
									AAILPTSIFLTNKK	95.0%	34.2	12.6	1	0	0	2	1,516.91
									DPMIDNNR	95.0%	38.1	17.6	1	0	0	2	990.43
									VPEEEKDTNVQVLMVLGAGR	95.0%	41.6	21.1	0	2	0	2	2,200.13
									VPLVAPEDLR	95.0%	48.8	19.0	2	0	0	2	1,108.64
									VPLVAPEDLRDDIIENAPTTHTEEYSGEEK	95.0%	95.1	19.3	0	2	1	2	3,367.62
									YSQYQQAIYK	95.0%	47.1	22.8	3	0	0	2	1,291.63
U2 small nuclear ribonucleoprotein A'	RU2A_HUMAN	SNRPA1	28,399	100.00%	2	2	2	9.02%	NAIANASTLAEVER	95.0%	79.1	23.0	1	0	0	2	1,458.76
									SLTYLSILR	95.0%	56.4	15.7	1	0	0	2	1,065.63
Acetyl-CoA acetyltransferase, cytosolic	THIC_HUMAN	ACAT2	41,332	100.00%	9	13	21	40.60%	AGHFDKEIVPVLVSTR	95.0%	43.5	18.7	0	2	1	2	1,767.98
									ATVAPEDVSEVIFGHVLAAGCGQNPVR	95.0%	43.3	21.2	0	1	0	2	2,793.40
									EDQDKVAVLSQNR	95.0%	53.6	22.6	2	1	0	2	1,501.76
									ELGLNPEK	95.0%	30.9	19.6	1	0	0	2	899.48
									ILVTLLHTLER	95.0%	67.4	11.8	2	4	0	2	1,307.81
									IVSWSQVGVEPSIMGIGPIPAIK	95.0%	49.1	17.5	1	0	0	2	2,394.31
									KGLIEVK	95.0%	30.9	17.9	1	0	0	2	786.51
									LKPYFLTDGTGTVTPANASGINDGAAAVVLMK	95.0%	62.0	17.8	0	2	0	2	3,208.66
									TIIGSFNGALAAVPVQDLGSTVIK	95.0%	119.0	14.8	2	1	0	2	2,371.32
Translin-associated protein X	TSNAX_HUMAN	TSNAX	33,095	100.00%	6	7	21	25.50%	HMLADVFSVK	95.0%	31.0	23.5	1	0	0	2	1,162.59
									QVYDGFSFIGNTGPYEVSK	95.0%	79.6	21.4	2	0	0	2	2,108.00
									SFQQELDAR	95.0%	34.9	23.5	2	0	0	2	1,093.53
									SLISMDEINK	95.0%	41.8	23.5	2	0	0	2	1,165.58
									TIFLLHR	95.0%	33.4	19.1	1	0	0	2	899.55
									VTPVDYLLGVADLTGELMR	95.0%	105.0	21.6	11	2	0	2	2,078.08
Sodium/potassium-transporting ATPase subunit alpha-1	AT1A1_HUMAN	ATP1A1	112,882	100.00%	5	5	17	7.23%	GVGIISEGNETVEDIAAR	95.0%	94.1	22.0	1	0	0	2	1,829.92
									MSINAEEVVVGDLVEVK	95.0%	80.1	21.7	5	0	0	2	1,846.95
									NMVPQQALVIR	95.0%	50.7	21.4	4	0	0	2	1,284.71
									SPDFTNENPLETR	95.0%	76.8	20.4	4	0	0	2	1,519.70
									VDNSSLTGESEPQTR	95.0%	65.8	21.2	3	0	0	2	1,619.75
Aminopeptidase B	AMPB_HUMAN	RNPEP	72,579	100.00%	14	15	40	29.40%	AEFGPPGPGAGSR	95.0%	54.2	22.4	4	0	0	2	1,199.58
									AFFPCFDTPAVK	95.0%	38.1	22.2	1	0	0	2	1,399.67
									EEYNGVIEEFLATGEK	95.0%	95.4	22.4	4	0	0	2	1,827.87
									ETFASTASQLHSNVVNYVQQIVAPK	95.0%	28.1	20.3	0	2	0	2	2,731.41
									GLSGTAVLDLR	95.0%	73.3	21.8	4	0	0	2	1,101.63
									IEPGVDPDDTYNETPYEK	95.0%	72.6	18.7	4	0	0	2	2,081.92
									LGDTYPSISNAR	95.0%	43.7	23.2	2	0	0	2	1,293.64
									LQVLLTYR	95.0%	37.7	15.3	3	0	0	2	1,005.61
									QHMDITGEENPLNK	95.0%	47.1	21.6	2	0	0	2	1,641.75
									RPLHSAQAVDVASASNFR	95.0%	27.2	20.9	0	1	0	2	1,925.99
									TYQLVYFLDK	95.0%	67.8	24.0	4	0	0	2	1,289.68
									VDIIPGFEFDR	95.0%	56.8	22.3	2	0	0	2	1,307.66
									VKIEPGVDPDDTYNETPYEK	95.0%	50.4	21.4	1	2	0	2	2,309.08
									YTLPLYHAMMGGSEVAQTLAK	95.0%	40.8	21.3	0	4	0	2	2,313.13
Delta-aminolevulinic acid dehydratase	HEM2_HUMAN	ALAD	36,277	99.50%	2	2	3	8.79%	GSAADSEESPAIEAIHLLR	95.0%	31.5	22.4	0	2	0	2	1,965.99
									LAEVALAYAK	95.0%	37.6	17.6	1	0	0	2	1,048.60
KH domain-containing, RNA-binding, signal transduction-	KHDR1_HUMAN	KHDRBS1	48,210	100.00%	2	3	9	4.97%	LQEETGAK	95.0%	40.9	23.5	3	0	0	2	875.45
									SGSMDPSGAHPSVR	95.0%	39.8	18.6	4	2	0	2	1,400.62
Adenine phosphoribosyltransferase	APT_HUMAN	APRT	19,591	99.50%	2	2	5	14.40%	LAPVPFFSLLQYE	95.0%	47.8	21.3	3	0	0	2	1,523.82
									SFPDFPTPGVVFR	95.0%	42.2	21.8	2	0	0	2	1,465.75
Aminoacylase-1	ACY1_HUMAN	ACY1	45,866	100.00%	10	11	20	35.50%	AGFALDEGIANPTDAFTVFYSER	95.0%	91.3	21.1	2	0	0	2	2,491.18
									AVGVPALGFSPMNR	95.0%	43.0	23.4	2	0	0	2	1,431.74

									DMNLTLEPEIMPAATDNR	95.0%	83.0	20.7	2	0	0	2	2,062.94
									EGSVTSVNLTK	95.0%	43.6	22.8	2	0	0	2	1,134.60
									FMEDTAAEK	95.0%	48.4	18.5	2	0	0	2	1,057.45
									GPEEEHPSVTLFR	95.0%	60.4	22.3	2	2	0	2	1,497.73
									GVDIYTR	95.0%	30.7	22.0	1	0	0	2	823.43
									LEGGVAYNVIPATMSASFDFR	95.0%	81.1	22.0	2	0	0	2	2,261.09
									TVQPKPDYGAAVAFFEETAR	95.0%	42.8	21.3	0	1	0	2	2,197.09
									VVNSILAFR	95.0%	65.8	17.6	2	0	0	2	1,018.60
40S ribosomal protein SA	RSSA_HUMAN	RPSA	32,836	100.00%	10	12	127	44.10%	ADHQPLTEASYVNLPTIALCNTDSPLR	95.0%	65.0	21.4	0	5	0	2	2,996.48
									AIVAIENPADVSVISSR	95.0%	124.0	19.2	35	2	0	2	1,740.95
									DPEEIEKEEQAAAEK	95.0%	78.3	21.8	2	0	0	2	1,715.80
									FAAATGATPIAGR	95.0%	109.0	23.4	28	0	0	2	1,203.65
									FLAAGTHLGGTNLDFQMEQYIYK	95.0%	64.9	21.4	0	4	0	2	2,633.27
									FTPGTFTNQIQAAFR	95.0%	91.1	21.5	22	0	0	2	1,698.86
									KSDGIYIINLK	95.0%	60.0	17.3	6	1	0	2	1,263.73
									LLVVTDPR	95.0%	55.4	16.1	13	0	0	2	912.55
									SDGIYIINLK	95.0%	70.3	21.6	8	0	0	2	1,135.64
									SDGIYIINLKR	95.0%	25.9	18.9	0	1	0	2	1,291.74
Glutathione S-transferase theta-2	GSTT2_HUMAN	GSTT2	27,489	99.50%	2	2	3	11.50%	SKEFLQINSLGK	95.0%	63.5	19.6	1	0	0	2	1,363.76
									TLPTPSPEAYQAMLLR	95.0%	59.5	21.9	2	0	0	2	1,803.93
Actin-related protein 2/3 complex subunit 2	ARPC2_HUMAN	ARPC2	34,316	100.00%	8	8	23	25.70%	ASHTAPQVLFSHR	95.0%	36.2	22.9	0	3	0	2	1,450.76
									AYIHTR	95.0%	38.8	24.2	2	0	0	2	760.41
									DNTINLIHTFR	95.0%	33.3	22.5	0	2	0	2	1,343.71
									DSIVHQAGMLK	95.0%	33.9	22.0	3	0	0	2	1,214.62
									ELQAHGADELLK	95.0%	54.3	21.8	4	0	0	2	1,323.69
									ELQAHGADELLKR	95.0%	41.7	21.8	0	2	0	2	1,479.79
									MILLEVNNR	95.0%	39.5	22.7	3	0	0	2	1,117.60
									YFQFQEEGKEGENR	95.0%	47.2	20.0	0	4	0	2	1,760.79
Thimet oligopeptidase	THOP1_HUMAN	THOP1	78,823	100.00%	14	17	49	22.90%	DAASGEVVGK	95.0%	49.8	23.5	2	0	0	2	932.47
									DSLRPEAAR	95.0%	32.3	21.2	1	0	0	2	1,014.53
									ETQENIKR	95.0%	40.7	23.2	1	0	0	2	1,017.53
									FKQEGVLNSK	95.0%	50.0	21.8	2	3	0	2	1,149.63
									FYLDLYPR	95.0%	42.1	22.9	3	0	0	2	1,086.56
									LKPLGEQER	95.0%	38.0	20.3	0	3	0	2	1,069.60
									LSEFDVEMSMR	95.0%	50.5	16.6	2	0	0	2	1,375.59
									NILDFPQHVSPSK	95.0%	68.7	23.0	2	0	0	2	1,481.78
									QANTGLFNLR	95.0%	67.2	22.7	3	0	0	2	1,133.61
									TSQTVATFLDELAQK	95.0%	119.0	22.3	9	5	0	2	1,651.85
									VDQALHTQTDADPAEEYAR	95.0%	82.2	20.5	2	5	0	2	2,129.97
									VGMDYR	95.0%	44.4	17.2	2	0	0	2	756.34
									VYDQVGTQEFEDVSYESTLK	95.0%	85.2	20.4	1	0	0	2	2,337.08
									YYMNQVEETR	95.0%	46.4	17.9	3	0	0	2	1,348.58
Protein disulfide-isomerase A6	PDIA6_HUMAN	PDIA6	48,104	100.00%	15	16	98	45.20%	AATALKDVVK	95.0%	45.6	17.7	5	0	0	2	1,015.62
									ALDLFSDNAPPPELLEIINEDIAK	95.0%	59.7	20.5	0	2	0	2	2,637.37
									ALDLFSDNAPPPELLEIINEDIAKR	95.0%	56.1	18.9	0	2	0	2	2,793.47
									DGELPVEDDIDLSDVELDDLGKDEL	95.0%	71.5	20.3	2	0	0	2	2,758.27
									GESPVDYDGGR	95.0%	65.1	18.4	6	0	0	2	1,151.50
									GSFSEQGINEFLR	95.0%	80.6	22.2	7	0	0	2	1,483.72
									GSTAPVGGGAFPTIVER	95.0%	97.0	22.6	2	0	0	2	1,615.84
									IFQKGESPVDYDGGR	95.0%	41.5	22.3	0	1	0	2	1,667.80
									KDVIELTDDSFDK	95.0%	75.4	22.8	1	0	0	2	1,524.74

									LAAVDATVNQVLASR	95.0%	120.0	20.6	19	0	0	2	1,527.85
									NRPEDYQGGR	95.0%	35.8	20.6	1	0	0	2	1,191.55
									NSYLEVLLK	95.0%	63.9	20.6	9	0	0	2	1,078.62
									TGEAIVDAALSALR	95.0%	106.0	22.3	37	0	0	2	1,386.76
									TRSDIVSR	95.0%	36.0	23.2	1	0	0	2	933.51
									VGAVDADKHHSLGGQYGVQGFPTIK	95.0%	89.8	20.4	0	2	1	2	2,581.32
Delta(3,5)-Delta(2,4)-dienoyl-CoA isomerase, mitochondrial	ECH1_HUMAN	ECH1	35,798	100.00%	4	4	10	14.30%	MMADEALGSGLVSR	95.0%	97.4	21.5	2	0	0	2	1,468.68
									VIGNQSLVNELAFTAR	95.0%	95.0	20.6	4	0	0	2	1,731.94
									VNLLYSR	95.0%	35.5	20.7	2	0	0	2	864.49
									YQETFNVIER	95.0%	43.4	21.9	2	0	0	2	1,298.64
5'-nucleotidase	5NTD_HUMAN	NT5E	63,351	100.00%	8	9	40	25.30%	ETPFLSNPGTNLVFEDEITALQPEVDKLK	95.0%	33.4	19.0	0	2	0	2	3,244.66
									FPILSANIK	95.0%	39.7	17.6	6	0	0	2	1,002.60
									GPLASQISGLYLPYK	95.0%	86.3	19.3	6	0	0	2	1,606.88
									HDSGDQDINVVSTYISK	95.0%	104.0	22.5	2	0	0	2	1,877.89
									VILPNFLANGGDGFQMIKDELLR	95.0%	31.7	18.8	0	3	0	2	2,576.36
									VLPVGDEVVGIVGYTSK	95.0%	132.0	20.0	10	2	0	2	1,731.95
									YDAMALGNHEFDNGVEGLIEPLLK	95.0%	43.5	21.7	0	5	0	2	2,661.29
									YPFIVTSDDGR	95.0%	52.7	21.8	4	0	0	2	1,269.61
Collagen alpha-1(XVIII) chain	COIA1_HUMAN	COL18A1	178,170	100.00%	14	17	188	12.10%	ADDILASPPR	95.0%	34.9	21.8	3	0	0	2	1,054.55
									AVGLAGTFR	95.0%	41.2	21.7	8	0	0	2	891.51
									DFQPVLHLVALNSPLSGGMR	95.0%	73.8	21.3	9	32	0	2	2,167.13
									ELLREETGAALKPR	95.0%	58.1	18.9	1	19	0	2	1,582.89
									GADFQCFQQAR	95.0%	59.3	20.3	2	0	0	2	1,327.59
									GLELEPGAGLFVAQAGGADPDKFQGVIAELK	95.0%	44.7	18.2	0	4	0	2	3,097.62
									GQPGPPGPQGPPGIGYEGR	95.0%	61.0	22.5	3	0	0	2	1,817.89
									GTDNEVAALQPPVVQLHDSNPYPR	95.0%	52.7	21.1	0	23	0	2	2,617.30
									IFSFDGK	95.0%	38.9	20.9	4	0	0	2	813.41
									IFSFDGKDVLR	95.0%	68.8	21.0	9	0	0	2	1,296.70
									KVQLEAR	95.0%	31.6	21.7	2	0	0	2	843.51
									LQDLYSIVR	95.0%	73.3	21.1	24	0	0	2	1,106.62
									LSGVQDGHQDISLLYTEPGAGQTHTAASFR	95.0%	86.2	20.5	0	4	9	2	3,156.54
									TEAPSATGQASSLLGGR	95.0%	130.0	22.6	32	0	0	2	1,602.81
AP-2 complex subunit beta	AP2B1_HUMAN	AP2B1	104,537	100.00%	12	14	62	17.40%	DIPNENELQFQIK	95.0%	59.3	22.9	4	0	0	2	1,587.80
									DSDYYNMLLK	95.0%	37.2	19.1	1	0	0	2	1,277.57
									KLAPPLVTLLSGEPEVQYVALR	95.0%	44.1	12.8	0	2	0	2	2,393.38
									KPSETQELVQQVLSLATQDSDNPDLR	95.0%	49.9	20.6	0	4	0	2	2,911.47
									LAPPLVTLLSGEPEVQYVALR	95.0%	102.0	14.0	5	3	0	2	2,265.29
									LASQANIAQVLAELK	95.0%	98.8	17.7	17	1	0	2	1,568.90
									LHDINAQMVEDQGFLDSLR	95.0%	64.2	21.8	0	2	0	2	2,217.06
									LSHANSAVVLSAVK	95.0%	47.0	18.2	2	0	0	2	1,395.80
									LVYLYLMNYAK	95.0%	63.4	23.1	3	0	0	2	1,406.74
									MEPLNNLQVAVK	95.0%	55.9	22.1	8	0	0	2	1,371.73
									NVEGQDMLYQSLK	95.0%	69.1	22.6	8	0	0	2	1,540.73
									YNDPIYVK	95.0%	46.8	21.6	2	0	0	2	1,011.52
Adenylate kinase isoenzyme 1	KAD1_HUMAN	AK1	21,617	100.00%	8	8	26	46.40%	ATEPVIAFYEK	95.0%	38.1	22.0	2	0	0	2	1,267.66
									ATEPVIAFYEKR	95.0%	31.8	21.6	0	2	0	2	1,423.76
									EVQQGEEFER	95.0%	49.3	21.2	2	0	0	2	1,250.57
									GFLIDGYPR	95.0%	39.5	22.4	2	0	0	2	1,037.54
									GQLVPLETVLDMLR	95.0%	86.5	20.2	8	0	0	2	1,599.88
									IGQPTLLLYVDAGPETMTQR	95.0%	91.3	21.4	2	0	0	2	2,219.14
									IIFVVGGPGSGK	95.0%	84.6	18.8	4	0	0	2	1,130.66

									YGYTHLSTGDLLR	95.0%	46.5	21.7	0	4	0	2	1,495.75
Tumor protein D54	TPD54_HUMAN	TPD52L2	22,220	100.00%	3	3	7	20.90%	GLLSDSMTDVPVDTGVAAR	95.0%	85.4	22.5	2	0	0	2	1,919.94
									LGLSTLGELK	95.0%	50.8	17.9	3	0	0	2	1,030.62
									TSAALSTVGSAISR	95.0%	71.9	22.2	2	0	0	2	1,320.71
Ribulose-phosphate 3-epimerase	RPE_HUMAN	RPE	24,910	99.50%	2	2	4	11.40%	IGPSILNSDLANLGAECLR	95.0%	102.0	20.9	2	0	0	2	2,013.04
									SVINLLR	95.0%	34.7	21.0	2	0	0	2	814.52
14-3-3 protein epsilon	1433E_HUMAN	YWHAE	29,157	100.00%	17	23	350	63.50%	AAFDDAIAELDTLSEESYK	95.0%	110.0	20.8	21	4	0	2	2,087.97
									AASDIAMTELPPTHPIR	95.0%	83.8	22.0	24	28	0	2	1,835.93
									DSTLIMQLLR	95.0%	78.2	22.5	32	0	0	2	1,205.66
									EAAENSLVAYK	95.0%	68.6	23.3	34	0	0	2	1,194.60
									EALQDVEDENQ	95.0%	53.0	16.9	2	0	0	2	1,289.55
									HLIPAANTGESK	95.0%	41.8	22.5	3	3	0	2	1,237.65
									IISSIEQK	95.0%	38.3	22.6	3	0	0	2	917.53
									IISSIEQKEENK	95.0%	72.6	22.6	9	0	0	2	1,417.75
									KEAAENSLVAYK	95.0%	70.2	22.0	6	0	0	2	1,322.70
									LAEQAER	95.0%	56.5	21.6	15	0	0	2	816.42
									LAEQAERYDEMVESMK	95.0%	52.7	18.5	2	9	0	2	1,960.86
									LICCDILDVLDK	95.0%	85.2	23.2	11	0	0	2	1,476.74
									LICCDILDVLDKHLIPAANTGESK	95.0%	45.3	20.2	0	8	16	2	2,695.38
									NLLSVAYK	95.0%	51.2	19.1	26	0	0	2	907.53
									QMVETELK	95.0%	62.4	24.0	16	0	0	2	993.49
									VAGMDVELTVEER	95.0%	111.0	22.5	104	0	0	2	1,463.71
									YDEMVESMK	95.0%	52.1	12.6	12	0	0	2	1,163.46
									YDEMVESMKK	95.0%	31.5	17.8	1	0	0	2	1,291.56
									YLAEFATGNDR	95.0%	79.4	21.1	12	0	0	2	1,256.59
									YLAEFATGNDRK	95.0%	50.2	22.4	17	5	0	2	1,384.69
DnaJ homolog subfamily C member 13	DJC13_HUMAN	DNAJC13	254,421	99.90%	2	2	3	1.20%	DSLLASLLDGVR	95.0%	34.4	22.0	1	0	0	2	1,258.70
									IVDGPDPENIILILK	95.0%	42.1	13.8	2	0	0	2	1,648.95
T-complex protein 1 subunit epsilon	TCPE_HUMAN	CCT5	59,654	100.00%	13	15	64	35.30%	AFADALEVIPMALSENSGMNPIQTMTEVR	95.0%	41.0	19.8	0	1	0	2	3,183.50
									AVTIFIR	95.0%	37.3	14.5	2	0	0	2	819.51
									DVDFELIKVEGK	95.0%	59.1	21.7	2	0	0	2	1,391.74
									EKFEEMIQQIK	95.0%	45.3	22.9	2	0	0	2	1,438.73
									GVIVDKDFSHPQMPK	95.0%	66.0	22.3	2	2	0	2	1,713.86
									IADGYEQAAR	95.0%	85.2	23.6	10	0	0	2	1,093.53
									IDDIRKPGESEE	95.0%	39.0	21.7	2	0	0	2	1,387.67
									ISDSVLVDIKDTEPLIQTAK	95.0%	77.5	17.2	2	2	0	2	2,185.20
									KQQISLATQMVR	95.0%	54.9	21.0	2	0	0	2	1,418.78
									LGFAGLVQEISFGTTK	95.0%	110.0	19.2	29	0	0	2	1,667.90
									MMVDKDGDVTVTNDGATILSMMDVDHQIAK	95.0%	80.7	18.7	0	0	2	2	3,314.49
									QQISLATQMVR	95.0%	52.8	23.1	2	0	0	2	1,290.68
									WVGGPEIELIAIATGGR	95.0%	79.4	19.8	2	0	0	2	1,738.95
Elongation factor 1-alpha 2	EF1A2_HUMAN	EEF1A2	50,453	100.00%	4	5	41	33.30%	EGNASGVSLLEALDTILPPTRPTDKPLR	95.0%	69.7	15.9	0	3	0	2	2,960.61
									EHALLAYTLGVK	95.0%	65.7	20.5	84	15	0	2	1,314.74
									FEKEAAEMGK	95.0%	27.6	20.3	0	1	0	2	1,155.54
									IGGIGTVPVGR	95.0%	90.3	16.1	86	0	0	2	1,025.61
									KLEDNPK	95.0%	48.5	20.3	2	0	0	2	843.46
									LPLQDVYK	95.0%	60.1	21.1	31	0	0	2	975.55
									QLIVGVNK	95.0%	34.2	17.1	2	0	0	2	870.54
									QTVAVGVIK	95.0%	35.5	18.7	2	0	0	2	914.57
									STTTGHLIYK	95.0%	51.8	21.3	35	0	0	2	1,120.60
									THINIVVIGHVDSGK	95.0%	84.4	20.2	10	353	0	2	1,588.88

									VETGILRPGMVVTFAPVNITTEVK	95.0%	63.3	14.8	0	24	0	2	2,587.42
									YYITIIDAPGHR	95.0%	61.0	22.7	4	8	0	2	1,418.74
Pregnancy-specific beta-1-glycoprotein 9	PSG9_HUMAN	PSG9	48,256	100.00%	3	3	6	7.51%	LPIPYITINNLNPR	95.0%	67.7	16.1	3	0	0	2	1,637.94
									NPVSASR	95.0%	34.3	21.9	1	0	0	2	730.38
									SDPVTLNLLPK	95.0%	37.0	18.9	2	0	0	2	1,196.69
Heterogeneous nuclear ribonucleoprotein H3	HNRH3_HUMAN	HNRNPH3	36,910	99.50%	2	2	11	8.38%	ATENDIANFFSPLNPIR	95.0%	67.5	22.3	10	0	0	2	1,918.97
									STGEAFVQFASK	95.0%	36.8	21.9	1	0	0	2	1,271.63
Heat shock protein beta-1	HSPB1_HUMAN	HSPB1	22,765	100.00%	9	10	59	58.50%	AQLGGPEAAKSDETAAK	95.0%	63.1	23.0	2	0	0	2	1,643.82
									KYTLPPGVDPTQVSSSLSPEGTLTVEAPMPK	95.0%	62.4	19.5	0	13	0	2	3,242.65
									LATQSNEITIPVTFESR	95.0%	85.6	21.4	12	0	0	2	1,905.99
									LFDQAFGLPR	95.0%	74.8	22.2	17	0	0	2	1,163.62
									QLSSGVSEIR	95.0%	60.2	23.5	1	0	0	2	1,075.57
									RVPFSLLR	95.0%	35.0	15.8	4	0	0	2	987.61
									TKDGVVEITGK	95.0%	51.8	22.4	1	0	0	2	1,146.64
									VPFSLLR	95.0%	42.6	19.3	2	0	0	2	831.51
									VSLDVNHFAPDELTVK	95.0%	67.3	21.7	4	3	0	2	1,783.92
C-type mannose receptor 2	MRC2_HUMAN	MRC2	166,637	99.50%	2	2	14	1.83%	GTDVREPDDSPQGR	95.0%	39.9	21.0	0	5	0	2	1,528.70
									TLGDQLSLLLGAR	95.0%	72.6	19.4	9	0	0	2	1,356.79
40S ribosomal protein S24	RS24_HUMAN	RPS24	15,406	100.00%	2	2	4	20.30%	TTGFGMIYDSLDYAK	95.0%	59.2	21.3	2	0	0	2	1,681.78
									TTPDVIFVFGFR	95.0%	59.0	20.8	2	0	0	2	1,398.74
Proteasome subunit alpha type-1	PSA1_HUMAN	PSMA1	29,538	100.00%	17	21	75	57.00%	ALRETLPAEQDLTTK	95.0%	57.7	20.2	1	2	0	2	1,685.91
									AMSIGAR	95.0%	44.4	23.7	4	0	0	2	721.37
									AQPAQPADEPAEK	95.0%	52.5	21.7	4	0	0	2	1,351.65
									AQPAQPADEPAEKADEPMEH	95.0%	68.0	17.8	6	0	0	2	2,176.95
									AQSELAAHQK	95.0%	46.5	21.2	5	0	0	2	1,082.56
									AQSELAAHQKK	95.0%	31.2	22.1	1	0	0	2	1,210.65
									ETLPAEQDLTTK	95.0%	48.4	23.0	10	0	0	2	1,345.69
									HMSEFMECNLNELVK	95.0%	64.2	18.1	2	2	0	2	1,912.82
									IHQIEYAMEAVK	95.0%	52.0	22.8	6	5	0	2	1,447.73
									ILHVDNHIGISIAGLTADAR	95.0%	48.0	18.3	0	8	0	2	2,086.14
									KAQPAQPADEPAEK	95.0%	42.7	22.9	2	0	0	2	1,479.74
									LVSLIGSK	95.0%	42.6	17.2	4	0	0	2	816.52
									NQYDNDVTVWSPQGR	95.0%	74.0	25.0	2	0	0	2	1,778.81
									NVSIGIVGK	95.0%	34.9	21.7	1	0	0	2	886.54
									RAQSELAAHQK	95.0%	27.9	20.9	0	1	0	2	1,238.66
									THAVLVALKR	95.0%	44.0	9.5	2	6	0	2	1,107.70
									TQIPTQR	95.0%	37.7	22.7	1	0	0	2	843.47
Heterogeneous nuclear ribonucleoprotein F	HNRPF_HUMAN	HNRNPF	45,654	100.00%	3	3	17	13.50%	ATENDIYNFFSPLNPVR	95.0%	85.2	22.4	29	0	0	2	1,996.98
									HSGPNSADSANDGFVR	95.0%	73.7	19.3	3	0	0	2	1,630.72
									ITGEAFVQFASQELAEK	95.0%	109.0	21.4	13	0	0	2	1,867.94
									YIEVFK	95.0%	32.4	19.7	1	0	0	2	798.44
Histone H1.3	H13_HUMAN	HIST1H1D	22,333	100.00%	4	4	7	15.40%	ALAAAGYDVEK	95.0%	45.4	24.2	2	0	0	2	1,107.57
									ASGPPVSELITK	95.0%	37.9	21.6	2	0	0	2	1,198.67
									KASGPPVSELITK	95.0%	49.0	19.5	2	0	0	2	1,326.76
									SGVSLAALKK	95.0%	47.2	16.1	1	0	0	2	973.60
Ras-related protein Rab-14	RAB14_HUMAN	RAB14	23,880	99.90%	2	2	4	12.10%	LQIWDTAGQER	95.0%	55.4	22.2	2	0	0	2	1,316.66
									NLTNPNTVIILIGNK	95.0%	47.1	15.3	2	0	0	2	1,623.94
Vacuolar protein sorting-associated protein 26A	VP26A_HUMAN	VPS26A	38,153	100.00%	9	11	19	36.70%	EITGIGPSTTTETETIAK	95.0%	75.2	22.1	2	0	0	2	1,848.94
									ELALPGELTQSR	95.0%	60.1	21.9	1	0	0	2	1,313.71
									EYDLIVHQLATYPDVNNSIK	95.0%	87.2	20.6	2	0	0	2	2,332.18
									FESPESQASAEQPEM	95.0%	63.1	14.6	3	0	0	2	1,682.69

									LFLAGYDPTPTMR	95.0%	67.7	22.6	3	0	0	2	1,497.74
									SNTHEFVNLVK	95.0%	41.6	22.4	0	2	0	2	1,287.67
									YEIMDGAPVK	95.0%	46.1	22.2	1	0	0	2	1,138.55
									YEIMDGAPVKGESIPIR	95.0%	82.8	22.4	2	1	0	2	1,890.96
									YFLNLVLVDEEDRR	95.0%	37.9	21.1	1	1	0	2	1,780.92
GrpE protein homolog 1, mitochondrial	GRPE1_HUMAN	GRPEL1	24,261	100.00%	3	4	6	17.50%	DLLEVADVLEK	95.0%	61.3	22.2	2	0	0	2	1,243.68
									NSGQNLEEDMGQSEQK	95.0%	38.7	18.2	1	0	0	2	1,809.76
									TLRPALVGVVK	95.0%	31.1	7.0	1	2	0	2	1,152.75
3,2-trans-enoyl-CoA isomerase, mitochondrial	D3D2_HUMAN	DCI	32,799	100.00%	3	3	5	11.90%	DADVQNFVSFISK	95.0%	81.7	22.1	2	0	0	2	1,469.73
									SLQMYLER	95.0%	40.3	22.2	1	0	0	2	1,055.52
									VLVEPDAGAGVAVMK	95.0%	79.2	21.9	2	0	0	2	1,471.78
Proteasome activator complex subunit 3	PSME3_HUMAN	PSME3	29,489	100.00%	3	3	10	15.00%	NQYVTLHDMILK	95.0%	39.9	22.9	2	0	0	2	1,490.77
									SNQQLVDIIEK	95.0%	67.3	22.7	4	0	0	2	1,286.70
									TVESEAASYLDQISR	95.0%	90.0	21.8	4	0	0	2	1,668.81
Importin subunit beta-1	IMB1_HUMAN	KPNB1	97,153	100.00%	19	26	206	30.00%	AAVENLPTFLVELSR	95.0%	99.6	19.7	48	4	0	2	1,658.91
									ANFDKESER	95.0%	49.5	21.3	3	0	0	2	1,095.51
									ESCLEAYTGIVQGLK	95.0%	86.5	22.2	2	0	0	2	1,667.83
									GALQYLVPILTQTLTK	95.0%	81.4	12.0	27	9	0	2	1,759.04
									GDQENVHPDVMLVQPR	95.0%	47.4	22.0	4	4	0	2	1,849.89
									LAATNALLNSLEFTK	95.0%	114.0	20.1	37	0	0	2	1,605.89
									LLETTDRPDGHQNNLR	95.0%	53.0	22.6	1	4	0	2	1,878.94
									LQQVLQMESHIQSTSDR	95.0%	96.3	22.0	2	6	0	2	2,015.98
									NSLTSKDPDIK	95.0%	32.8	23.5	1	0	0	2	1,217.64
									SDYDMVDYLNELR	95.0%	73.0	17.6	3	0	0	2	1,648.72
									SNEILTAIIQGMR	95.0%	69.2	22.6	5	1	0	2	1,461.77
									SSAYESLMEIVK	95.0%	76.5	21.4	5	0	0	2	1,372.67
									TTLVIMER	95.0%	38.7	23.6	2	0	0	2	978.53
									TVSPDRLELEAAQK	95.0%	61.9	22.1	4	0	0	2	1,556.83
									VAALQNLVK	95.0%	47.0	16.9	9	0	0	2	955.59
									VLANPGNSQVAR	95.0%	67.1	21.5	17	0	0	2	1,225.67
									VQHQDALQISDVVMASLLR	95.0%	35.9	20.0	0	1	0	2	2,139.12
									YLEVVLNTLQQASQAQVDK	95.0%	110.0	20.7	4	0	0	2	2,147.14
									YMEAFKPFLGIGLK	95.0%	48.7	21.4	1	2	0	2	1,629.87
NADP-dependent malic enzyme	MAOX_HUMAN	ME1	64,133	100.00%	5	6	10	15.20%	DMAAFNERPIIFALSNPTSK	95.0%	44.6	21.9	0	2	0	2	2,238.12
									GSEYDDFLDEFMEAVSSK	95.0%	90.0	15.1	2	0	0	2	2,084.86
									QITDNIFLTTAEVIAQQVSDK	95.0%	89.3	20.5	1	2	0	2	2,334.22
									QITDNIFLTTAEVIAQQVSDKHLEEGR	95.0%	34.8	19.5	0	0	2	2	3,055.57
									QQLNIHGLLPPSFNSQEIQVLR	95.0%	34.0	17.3	0	1	0	2	2,531.37
Leucine-rich repeat-containing protein 59	LRC59_HUMAN	LRRC59	34,913	100.00%	2	2	5	8.14%	LVNLQHLDLLNNK	95.0%	43.2	18.2	1	0	0	2	1,533.88
									LVTLPVSFAQLK	95.0%	46.8	12.0	4	0	0	2	1,315.80
Abhydrolase domain-containing protein 14B	ABHEB_HUMAN	ABHD14B	22,328	100.00%	4	5	11	26.20%	AVAIDLPGLGHSK	95.0%	54.9	20.3	2	0	0	2	1,277.72
									FSVLLLHGIR	95.0%	61.1	15.3	2	2	0	2	1,154.71
									INAANYASVK	95.0%	31.6	22.4	1	0	0	2	1,050.56
									TPALIVYGDQDPMGQTSFEHLK	95.0%	39.7	21.3	0	4	0	2	2,463.19
WD repeat-containing protein 1	WDR1_HUMAN	WDR1	66,175	100.00%	4	5	19	6.77%	KVFASLPQVER	95.0%	38.8	20.4	1	0	0	2	1,273.73
									LYSILGTTLKDEGK	95.0%	61.4	19.2	2	4	0	2	1,537.85
									VFASLPQVER	95.0%	53.5	22.7	6	0	0	2	1,145.63
									YAPSGFYIASGDVSGK	95.0%	116.0	22.1	6	0	0	2	1,618.78
Latent-transforming growth factor beta-binding protein 4	LTBP4_HUMAN	LTBP4	173,410	100.00%	4	5	15	2.96%	AEAAAPYTVLAQSAPR	95.0%	104.0	22.8	6	2	0	2	1,615.84
									GYLAPSGDLSLRR	95.0%	33.5	21.3	1	0	0	2	1,404.76
									VSLSQPR	95.0%	34.4	23.0	5	0	0	2	786.45

									YNTRPLGQEPPR	95.0%	27.2	23.0	0	1	0	2	1,427.74
Filamin-A	FLNA_HUMAN	FLNA	280,711	100.00%	33	35	189	20.40%	AFGPGLQGGSAGSPAR	95.0%	78.1	22.4	21	0	0	2	1,429.72
									AGGPGLER	95.0%	75.8	20.7	19	0	0	2	756.40
									AGNNMLLVGVHGPR	95.0%	45.0	22.7	0	4	0	2	1,450.76
									ANLPQSFQVDTSK	95.0%	47.3	23.2	4	0	0	2	1,434.72
									ASGPGLNTTGVPASLPVEFTIDAK	95.0%	78.8	20.0	5	1	0	2	2,342.22
									AYGPGIEPTGNMVK	95.0%	50.9	22.6	9	0	0	2	1,449.71
									DAGEGLLAVQITDPEGKPK	95.0%	29.0	21.1	0	2	0	2	1,938.02
									EGPYSISVLYGDEEVPR	95.0%	79.7	22.3	5	0	0	2	1,909.92
									FGGEHVPNSPFQVTALAGDQPSVQPPLR	95.0%	25.9	20.3	0	1	0	2	2,945.49
									FNEEHIPDSPFVVPVASPSGDAR	95.0%	36.6	22.0	0	6	0	2	2,467.19
									FVPAEMGTHTVSVK	95.0%	35.2	22.6	2	0	0	2	1,518.76
									GAGTGGLGLAVEGPSEAK	95.0%	87.7	22.4	6	0	0	2	1,570.81
									GKLDVQFSGLTK	95.0%	37.7	20.4	1	0	0	2	1,292.72
									GTVEPQLEAR	95.0%	47.8	21.9	2	0	0	2	1,099.57
									IANLQTDLSDGLR	95.0%	72.7	21.9	6	0	0	2	1,415.75
									LDVQFSGLTK	95.0%	48.1	22.1	2	0	0	2	1,107.61
									LIALLEVLSQK	95.0%	62.2	13.4	21	0	0	2	1,226.77
									LPQLPITNFSR	95.0%	37.0	20.0	4	0	0	2	1,285.73
									LSPFMADIR	95.0%	37.0	23.2	4	0	0	2	1,065.54
									QMQLENVSVALEFLDR	95.0%	40.9	22.5	1	0	0	2	1,907.95
									SAGQGEVLVYVEDPAGHQEEAK	95.0%	57.5	21.4	0	8	0	2	2,313.10
									SPFEVYVDK	95.0%	58.7	22.3	7	0	0	2	1,083.54
									SPFSVAVSPSLDLSK	95.0%	32.6	21.3	1	0	0	2	1,533.82
									TGVAVNKPAEFTVDAK	95.0%	73.8	21.6	9	7	0	2	1,646.88
									TGVELGKPTHFTVNAK	95.0%	18.2	20.8	0	0	1	2	1,698.92
									VANPSGNLTETYVQDR	95.0%	44.8	22.7	2	0	0	2	1,763.86
									VDVGKDQEFTVK	95.0%	61.2	22.8	5	0	0	2	1,364.71
									VEPGLGADNSVVR	95.0%	33.1	22.6	3	0	0	2	1,312.69
									VGSAADIPINISETDLSLLTATVVPPSGR	95.0%	29.2	15.8	0	1	0	2	2,893.55
									VPVHDVTDASK	95.0%	31.0	22.7	2	0	0	2	1,167.60
									VTAQGPGLEPSGNIANK	95.0%	87.1	21.9	28	0	0	2	1,652.86
									VTVLFAGQHIAK	95.0%	51.1	18.1	7	0	0	2	1,283.75
									VTYTPMAPGSYLISIK	95.0%	54.4	21.3	9	0	0	2	1,756.92
									YGGDEIPFSPYR	95.0%	36.9	20.4	1	0	0	2	1,400.65
									YGGQPVPNFPSK	95.0%	56.2	22.1	4	0	0	2	1,290.65
									YTPVQQGPVGVNVTYGGDPIPK	95.0%	101.0	20.5	9	0	0	2	2,286.18
Alpha-actinin-1	ACTN1_HUMAN	ACTN1	103,043	100.00%	28	39	284	62.30%	ACLISLGYDIGNDPQGEAEFAR	95.0%	124.0	21.2	4	0	0	2	2,396.12
									AGTQIENIEEDFRDGLK	95.0%	75.3	22.1	4	3	0	2	1,934.95
									AIMTYVSSFYHAFSGAQK	95.0%	101.0	22.3	15	4	0	2	2,023.96
									ALDFIASK	95.0%	55.2	21.2	12	0	0	2	864.48
									ASIHEAWTDGK	95.0%	30.5	20.7	1	0	0	2	1,214.58
									ATLPDADKER	95.0%	56.1	21.9	17	2	0	2	1,115.57
									CQLEINFNTLQTK	95.0%	96.1	22.9	4	0	0	2	1,608.81
									DDPLTNLNTAFDVAEK	95.0%	111.0	23.1	13	0	0	2	1,762.85
									DHSGTLGPEEFK	95.0%	48.4	20.4	4	0	0	2	1,316.61
									DQALTEEHAR	95.0%	44.2	22.3	1	0	0	2	1,169.56
									DYETATLSEIK	95.0%	68.4	22.6	2	0	0	2	1,269.62
									EGLLLWCQR	95.0%	46.4	23.2	3	0	0	2	1,174.60
									ELPPDQAEYCIAR	95.0%	53.2	20.9	7	0	0	2	1,561.73
									ETADTDTADQVMASFK	95.0%	121.0	17.7	7	0	0	2	1,745.75

									FAIQDISVEETSAK	95.0%	97.4	22.4	36	0	0	2	1,537.78
									GISQEQMNEFR	95.0%	56.1	19.8	16	0	0	2	1,354.61
									GYEEWLLNEIR	95.0%	75.4	23.5	4	0	0	2	1,421.71
									HRPELIDYGK	95.0%	46.9	21.4	0	13	0	2	1,227.65
									HTNYTMEHIR	95.0%	43.8	20.0	2	4	0	2	1,317.60
									IDQLEGDHQLIQEALIFDNK	95.0%	90.5	21.4	5	20	0	2	2,339.19
									ILAGDKNYITMDELR	95.0%	53.3	21.6	2	1	0	2	1,767.90
									IMSIVDPNR	95.0%	43.0	24.9	4	0	0	2	1,060.55
									ISIEMHGTLEDQLSHLR	95.0%	63.7	22.1	0	8	6	2	1,995.00
									IVQTYHVNMAGTNPYTTITPQEINGK	95.0%	59.2	20.8	0	4	0	2	2,906.44
									KDDPLTNLNTAFDVAEK	95.0%	109.0	21.8	6	4	0	2	1,890.95
									LAILGIHNEVSK	95.0%	75.4	16.9	11	1	0	2	1,293.75
									LASDLLEWIR	95.0%	95.8	22.0	4	0	0	2	1,215.67
									LDHLAEK	95.0%	38.2	19.3	7	0	0	2	825.45
									LLETIDQLYLEYAK	95.0%	90.3	19.8	19	0	0	2	1,711.92
									LMLLLEVISGER	95.0%	80.2	19.6	7	0	0	2	1,388.78
									LSNRPAFMPSEGR	95.0%	36.5	23.5	0	5	0	2	1,477.72
									LVSIGAEEIVDGNVK	95.0%	114.0	21.4	35	0	0	2	1,542.84
									MLDAEDIVGTARPDEK	95.0%	87.3	22.2	11	11	0	2	1,775.85
									MTLGMIWTIILR	95.0%	93.8	20.2	13	0	0	2	1,479.81
									NVNIQNFHISWK	95.0%	54.6	22.0	1	0	0	2	1,499.78
									QFGAQANVIGPWIQTK	95.0%	75.1	20.6	4	0	0	2	1,757.93
									QKDYETATLSEIK	95.0%	56.0	22.2	4	2	0	2	1,525.78
									RDQALTEEHAR	95.0%	63.7	22.2	2	13	0	2	1,325.66
									TINEVENQILTR	95.0%	76.3	22.2	26	0	0	2	1,429.77
									VEQIAAIAQELNELDYYDSPSVNAR	95.0%	67.7	21.9	2	5	0	2	2,808.37
									VGWEQLLTTIAR	95.0%	86.1	20.5	33	0	0	2	1,386.77
									VLAVNQENEQLMEDYEK	95.0%	109.0	21.1	10	0	0	2	2,067.95
									VPENTMHAMQQK	95.0%	44.6	20.0	2	0	0	2	1,445.65
									YLDIPK	95.0%	34.2	19.0	3	0	0	2	748.42
Receptor-type tyrosine-protein phosphatase F	PTPRF_HUMAN	PTPRF	212,860	100.00%	36	44	256	24.60%	AAGTEGPFQEVDGVATTR	95.0%	114.0	22.7	16	0	0	2	1,805.87
									AGLGEEFEK	95.0%	55.3	22.0	4	0	0	2	979.47
									AGLGEEFEKEIR	95.0%	42.3	22.6	1	1	0	2	1,377.70
									AHTDVGPGPESSPVLVR	95.0%	80.7	22.1	7	6	0	2	1,717.89
									ELPGELLGYR	95.0%	49.9	23.4	2	0	0	2	1,146.62
									FDLSMPHVQDPSLVR	95.0%	31.1	21.8	0	1	0	2	1,756.87
									FEVIEFDDGAGSVLR	95.0%	111.0	22.4	12	0	0	2	1,653.81
									FTLTGLKPDTTYDIK	95.0%	80.0	20.7	5	1	0	2	1,712.91
									GPPSEAVR	95.0%	48.5	20.7	6	0	0	2	812.43
									GSSAGGLQHLVSIR	95.0%	68.9	21.1	2	1	0	2	1,381.76
									GYQVTYVR	95.0%	51.1	20.8	4	0	0	2	985.51
									HVVDGISR	95.0%	38.8	21.0	2	0	0	2	882.48
									IISYTVVFR	95.0%	56.3	18.1	9	0	0	2	1,097.64
									IQLSWLLPPQER	95.0%	37.5	18.0	1	0	0	2	1,479.83
									KLIADLQPNTEYSFVLMNR	95.0%	34.2	20.4	0	1	0	2	2,268.17
									LIADLQPNTEYSFVLMNR	95.0%	101.0	21.5	6	2	0	2	2,140.08
									LSVLEEEQLPPGFPSIDMGPQLK	95.0%	47.0	20.9	2	0	0	2	2,540.30
									NVLELSNVVR	95.0%	67.8	22.0	14	0	0	2	1,142.65
									SDMGVGVFTPTIEAR	95.0%	78.1	22.6	13	0	0	2	1,595.77
									TAPDLLPHKPLPASAYIEDGR	95.0%	23.9	20.0	0	0	2	2	2,261.19
									TAQSTPSAPPQK	95.0%	53.6	21.8	11	0	0	2	1,212.62

									TDEDVPSGPPR	95.0%	60.6	21.5	12	0	0	2	1,169.54
									TGEQAPSSPPR	95.0%	76.4	22.2	31	0	0	2	1,126.55
									TGEQAPSSPPRR	95.0%	39.4	22.0	0	2	0	2	1,282.65
									TQQGVPAQPADFQAEVESDTR	95.0%	111.0	20.9	8	2	0	2	2,274.06
									TSVLLSWEVPDSYK	95.0%	63.9	22.1	2	0	0	2	1,623.83
									VGGSMLTPR	95.0%	58.8	24.0	7	0	0	2	933.48
									VLAFTAVGDGPPSPTIQVK	95.0%	91.5	18.0	25	1	0	2	1,897.04
									VLAVNSIGR	95.0%	55.5	20.2	2	0	0	2	928.56
									VMCVSMGSTTVR	95.0%	55.1	19.1	2	0	0	2	1,359.61
									VPEDQTGLSGGVASFVCQATGEPKPR	95.0%	93.7	20.4	0	2	0	2	2,687.31
									VTFDPTSSYTLEDLKPDTLYR	95.0%	85.6	21.9	2	3	0	2	2,461.21
									WFYIVVVPIDR	95.0%	32.9	19.5	2	0	0	2	1,406.78
									WMMGAEELTKEDEMPVGR	95.0%	47.2	18.3	0	2	0	2	2,156.93
									YSAPANLYVR	95.0%	76.2	23.1	9	0	0	2	1,153.60
									YSIGGLSPFSEYAFR	95.0%	70.1	22.5	10	0	0	2	1,693.82
Glutathione S-transferase omega-1	GSTO1_HUMAN	GSTO1	27,549	100.00%	7	7	20	27.40%	EFTKLEEVLTNK	95.0%	66.2	21.0	2	0	0	2	1,450.78
									GIRHEVININLK	95.0%	33.3	14.5	0	2	0	2	1,405.83
									GSAPPGPVPEGSIR	95.0%	50.4	23.1	6	0	0	2	1,320.69
									HEVININLK	95.0%	36.5	17.9	2	0	0	2	1,079.62
									KLLPDDPYEK	95.0%	32.6	23.5	1	0	0	2	1,217.64
									MILELFSK	95.0%	37.8	20.5	3	0	0	2	996.54
									VPSLVGSFIR	95.0%	51.2	18.8	4	0	0	2	1,074.63
Structural maintenance of chromosomes protein 3	SMC3_HUMAN	SMC3	141,529	100.00%	7	7	9	8.46%	EENAEQQALAAK	95.0%	35.6	21.6	1	0	0	2	1,301.63
									ELGSLPQEAFEK	95.0%	34.4	22.4	1	0	0	2	1,347.68
									GSGSQSSVPSVDQFTGVGIR	95.0%	54.2	21.8	1	0	0	2	1,964.97
									INQMATAPDSQR	95.0%	41.9	22.0	1	0	0	2	1,347.63
									KGDVEGSQSQDEGEGSGESER	95.0%	56.7	15.1	0	2	0	2	2,166.90
									MNLPGEVTFLPLNK	95.0%	83.9	23.0	2	0	0	2	1,588.84
									SIMELMNVLELR	94.9%	30.3	22.7	1	0	0	2	1,479.76
Nuclear ubiquitous casein and cyclin-dependent kinases substrate	NUCKS_HUMAN	NUCKS1	27,279	100.00%	3	3	7	23.50%	DSGSDEDFLMEDDDDSDYGSSK	95.0%	119.0	0.0	3	0	0	2	2,444.87
									LKATVTPSPVK	95.0%	39.5	17.3	3	0	0	2	1,140.70
									TSTSPPPEKSGDEGSEDEAPSGED	95.0%	38.0	11.1	1	0	0	2	2,404.98
Glucosamine 6-phosphate N-acetyltransferase	GNA1_HUMAN	GNPNAT1	20,731	100.00%	3	3	9	23.40%	ITLECLPQNVGFYK	95.0%	59.1	21.6	2	0	0	2	1,681.86
									LLLSTLTLLSK	95.0%	84.1	6.0	3	0	0	2	1,201.78
									VLGQLTETGVVSPEQFMK	95.0%	96.8	22.0	4	0	0	2	1,963.02
14-3-3 protein sigma	1433S_HUMAN	SFN	27,757	100.00%	3	4	14	31.00%	AKLAEQAER	95.0%	33.6	22.6	4	0	0	2	1,015.55
									DSTLIMQLLR	95.0%	78.2	22.5	32	0	0	2	1,205.66
									LAEQAER	95.0%	56.5	21.6	15	0	0	2	816.42
									LAEQAERYEDMAAFMK	95.0%	26.0	19.4	0	1	0	2	1,934.86
									MKGDYYR	95.0%	35.8	19.2	2	0	0	2	932.43
									NLLSVAYK	95.0%	51.2	19.1	26	0	0	2	907.53
									SAYQEAMDISKK	95.0%	37.2	21.9	1	0	0	2	1,386.66
									SNEEGSEEKGPEVR	95.0%	55.1	19.2	4	8	0	2	1,546.70
									VLSSIEQK	95.0%	73.7	23.0	12	0	0	2	903.52
Exportin-5	XPO5_HUMAN	XPO5	136,297	100.00%	4	4	7	4.73%	AGGFVVGYTSSGNPIFR	95.0%	73.8	22.3	2	0	0	2	1,728.87
									AVTVMMDPNSTQR	95.0%	60.1	20.7	1	0	0	2	1,481.67
									DPLLLAIIPK	95.0%	43.0	6.0	2	0	0	2	1,092.70
									VLSDVDAFIAYVGTDQK	95.0%	64.1	22.1	2	0	0	2	1,840.93
Integrin beta-1	ITB1_HUMAN	ITGB1	88,397	100.00%	7	9	42	12.00%	IGFGSFVEK	95.0%	52.1	21.5	8	0	0	2	983.52
									LKPEDITQIQPQQLVLR	95.0%	93.2	12.0	5	8	0	2	2,019.16
									LSENNIQTIFAVTEEFQPVYK	95.0%	32.6	20.9	0	2	0	2	2,470.25

									NVLSLTNKGEVFNELVGK	95.0%	70.9	19.1	1	6	0	2	1,961.07
									SAVTTVVNPK	95.0%	51.4	21.5	4	0	0	2	1,015.58
									SGEPQTFTLK	95.0%	36.2	23.2	4	0	0	2	1,107.57
									SLGTDLMNEMR	95.0%	59.7	19.2	4	0	0	2	1,298.57
Adenylate kinase 2, mitochondrial	KAD2_HUMAN	AK2	26,461	100.00%	11	13	48	56.90%	AMVASGSELGK	95.0%	35.8	22.8	2	0	0	2	1,065.53
									AMVASGSELGKK	95.0%	51.9	22.8	3	3	0	2	1,193.62
									APSVPAAEPEYPK	95.0%	56.7	22.3	2	0	0	1	1,355.68
									AVLLGPPGAGK	95.0%	47.4	11.8	6	0	0	2	979.59
									GIHSAIDASQTPDVVFASILAAFSK	95.0%	57.3	18.8	0	4	0	2	2,545.33
									LDSVIEFSIPDSLLIR	95.0%	55.0	18.1	3	0	0	2	1,817.01
									LKATMDAGK	95.0%	42.0	23.2	2	0	0	2	950.50
									LQAYHTQTTPLIEYYR	95.0%	76.3	21.5	4	3	0	2	1,997.01
									LVSDEMVVELIEK	95.0%	81.4	22.3	10	0	0	2	1,519.79
									NGFLLDGFPR	95.0%	64.0	23.0	3	0	0	2	1,135.59
									QAEMLDDLMEK	95.0%	60.1	18.4	3	0	0	2	1,354.59
Programmed cell death 6-interacting protein	PDC6I_HUMAN	PDCD6IP	96,007	100.00%	15	15	44	24.80%	DTIVLLCKPEPELNAAIPSANPAK	95.0%	34.6	19.5	0	1	0	2	2,561.37
									ELPELLQR	95.0%	40.2	17.8	1	0	0	2	997.57
									EPSAPSIPTPAYQSSPAGGHAPTPPTPAPR	95.0%	41.4	20.0	0	2	0	2	2,936.45
									FLTALAQDGVINEEALSVTELDR	95.0%	136.0	20.8	2	0	0	2	2,504.29
									FTDLFEK	95.0%	36.5	19.5	2	0	0	2	899.45
									FYNELTEILVR	95.0%	80.6	20.6	6	0	0	2	1,396.75
									LALASLGYEK	95.0%	43.1	21.7	2	0	0	2	1,064.60
									LLDEEEATDNDLR	95.0%	101.0	21.0	4	0	0	2	1,532.71
									NIQVSHQEFSK	95.0%	42.8	22.2	2	0	0	2	1,316.66
									NLATAYDNFVELVANLK	95.0%	76.9	20.8	5	0	0	2	1,894.99
									SLLSNLDEVKK	95.0%	45.3	20.4	2	0	0	2	1,245.71
									STPVNVPISQK	95.0%	36.2	20.0	1	0	0	2	1,169.65
									SVIEQGGIQTVDQLIK	95.0%	112.0	18.5	8	0	0	2	1,727.95
									TMQGSEVVNVLK	95.0%	63.4	23.4	4	0	0	2	1,320.68
									YYDQICSIEPK	95.0%	56.0	21.2	2	0	0	2	1,415.65
Ubiquitin carboxyl-terminal hydrolase 14	UBP14_HUMAN	USP14	56,052	100.00%	4	4	16	10.30%	AQLFALTGVQPAR	95.0%	83.6	20.8	4	0	0	2	1,371.78
									ASGEMASAQYITAALR	95.0%	103.0	21.9	4	0	0	2	1,655.81
									LPAYLTIQMVR	95.0%	60.9	20.1	6	0	0	2	1,320.74
									RVEIMEEESEQ	95.0%	52.1	20.1	2	0	0	2	1,394.61
Arylsulfatase A	ARSA_HUMAN	ARSA	53,571	100.00%	3	3	8	10.80%	DPGENYNLLGGVAGATPEVLQALK	95.0%	84.7	20.1	2	0	0	2	2,426.26
									GGLPLEEVTVAEVLAAR	95.0%	94.8	18.1	5	0	0	2	1,723.96
									QSLFFYPSYPDEVR	95.0%	59.0	22.6	1	0	0	2	1,747.83
Activated RNA polymerase II transcriptional coactivator p15	TCP4_HUMAN	SUB1	14,378	100.00%	3	3	6	18.90%	EQISDIDDAVR	95.0%	62.8	22.2	2	0	0	2	1,260.61
									GISLNPEQWSQLK	95.0%	55.4	22.0	2	0	0	2	1,499.79
									GISLNPEQWSQLKEQISDIDDAVR	95.0%	60.1	21.7	0	2	0	2	2,741.38
Ubiquitin carboxyl-terminal hydrolase 5	UBP5_HUMAN	USP5	95,770	100.00%	2	2	2	2.68%	FASFPDYLVIQIK	95.0%	37.3	20.3	1	0	0	2	1,540.84
									SSENPNEVFR	95.0%	37.9	21.4	1	0	0	2	1,178.54
Cullin-2	CUL2_HUMAN	CUL2	86,967	100.00%	2	2	3	4.56%	FVQLINTVLNGDQHFMSALDK	95.0%	27.3	22.4	0	1	0	2	2,406.21
									KLMVEPLQAILIR	95.0%	35.1	12.3	0	2	0	2	1,539.93
Methionyl-tRNA synthetase, cytoplasmic	SYMC_HUMAN	MARS	101,100	99.90%	2	2	4	3.00%	GFVLQDTVEQLR	95.0%	66.3	22.6	3	0	0	2	1,404.75
									NNSELLNNLGNFINR	95.0%	71.2	22.2	1	0	0	2	1,731.88
Serine/arginine repetitive matrix protein 1	SRRM1_HUMAN	SRRM1	102,319	99.90%	2	2	4	2.65%	HRPSPPATPPPK	95.0%	51.1	21.0	0	2	0	2	1,281.71
									VNLEVIKPWITK	95.0%	40.3	13.2	0	2	0	2	1,439.86
Peptidyl-prolyl cis-trans isomerase FKBP3	FKBP3_HUMAN	FKBP3	25,159	100.00%	10	14	29	43.30%	ARLEIEPEWAYGK	95.0%	37.2	22.1	0	1	0	2	1,561.80
									AWTVEQLR	95.0%	33.2	23.8	1	0	0	2	1,002.54
									ETKSEETLDEGPPK	95.0%	66.4	21.8	2	1	0	2	1,559.74

									FKGTESISK	95.0%	40.0	20.6	2	0	0	2	996.54
									FLQEHGSDSFLAEHK	95.0%	74.8	21.6	2	5	1	2	1,744.83
									GWDEALLTMSK	95.0%	43.1	22.7	1	0	0	2	1,266.60
									SEETLDEGPPK	95.0%	71.2	20.3	5	0	0	2	1,201.56
									SEETLDEGPPKYTK	95.0%	65.1	22.0	2	1	0	2	1,593.77
									SEQLPK	95.0%	33.6	21.1	2	0	0	2	701.38
									TANKDHLVTAYNHLFETK	95.0%	27.0	22.1	0	0	3	2	2,102.07
Eukaryotic translation initiation factor 3 subunit F	EIF3F_HUMAN	EIF3F	37,546	100.00%	9	10	18	33.60%	EAPNPIHLTVDTSLQNGR	95.0%	36.2	22.2	0	1	0	2	1,962.00
									FLMSLVNQVPK	95.0%	70.6	20.9	2	0	0	2	1,291.71
									IGVDLIMK	95.0%	55.3	22.3	2	0	0	2	904.52
									IQDALSTVLQYAEDVLSGK	95.0%	82.7	20.8	1	0	0	2	2,050.07
									LHPVILASIVDSYER	95.0%	90.5	19.3	2	2	0	2	1,711.94
									TMGVMFTPLTVK	95.0%	63.4	22.0	2	0	0	2	1,356.69
									VIGLSSDLQQVGGASAR	95.0%	99.9	21.3	2	0	0	2	1,657.89
									VIGTLLGTVDK	95.0%	62.9	19.2	2	0	0	2	1,115.67
									VSADNTVGR	95.0%	74.5	23.9	2	0	0	2	918.46
Protein disulfide-isomerase	PDIA1_HUMAN	P4HB	57,100	100.00%	23	32	170	50.00%	AEGSEIR	95.0%	52.9	23.9	2	0	0	2	761.38
									EADDIVNWLK	95.0%	40.0	22.6	3	0	0	2	1,202.61
									ENLLDFIK	95.0%	45.7	20.7	5	0	0	2	991.55
									HNQLPLVIEFTEQTAPK	95.0%	67.2	19.8	2	16	0	2	1,965.04
									ILEFFGLK	95.0%	44.3	18.3	17	0	0	2	966.57
									ILEFFGLKK	95.0%	38.5	16.3	2	0	0	2	1,094.66
									ILFIFIDSDHTDNQR	95.0%	78.1	22.1	3	3	0	2	1,833.91
									KFDEGR	95.0%	32.5	23.7	1	0	0	2	751.37
									KSNFAEALAAHK	95.0%	44.1	23.2	2	1	0	2	1,286.69
									LGETYKDHENIVIAK	95.0%	33.5	21.9	0	2	0	2	1,729.91
									LITLEEEMTK	95.0%	64.1	23.5	13	0	0	2	1,222.62
									MDSTANEVEAVK	95.0%	87.1	20.7	9	0	0	2	1,309.59
									NFEDVAFDEK	95.0%	55.7	19.3	2	0	0	2	1,213.54
									NFEDVAFDEKK	95.0%	40.6	21.4	6	0	0	2	1,341.63
									NNFEGEVTKENLLDFIK	95.0%	86.4	21.4	2	9	0	2	2,010.02
									QFLQAAEAIDDIPFGITSNSDVFSK	95.0%	48.2	21.4	1	2	0	2	2,713.34
									SNFAEALAAHK	95.0%	69.8	22.5	2	0	0	2	1,158.59
									TGPAATTLPDGAAAESLVESSEVAVIGFFK	95.0%	84.3	19.6	0	12	0	2	2,935.49
									THILLFLPK	95.0%	49.6	12.0	9	1	0	2	1,081.68
									TVIDYNGER	95.0%	44.2	23.3	2	0	0	2	1,066.52
									VDATEESDLAQQYGVR	95.0%	117.0	21.6	11	2	0	2	1,780.84
									YKPESEELTAER	95.0%	49.4	22.1	15	1	0	2	1,451.70
									YQLDKDGVVLFK	95.0%	63.3	20.7	3	9	0	2	1,424.78
Collagen alpha-2(I) chain	CO1A2_HUMAN	COL1A2	129,297	100.00%	71	90	2343	68.60%	AGEDGHPGKPGRPGER	95.0%	36.5	22.3	0	15	0	2	1,616.79
									AGVMGPPGSR	95.0%	63.9	21.3	45	0	0	2	944.46
									AQPENIPAK	95.0%	39.0	19.8	4	0	0	2	967.52
									AVILQGSNDVELVAEGNSR	95.0%	117.0	21.6	243	92	0	2	1,971.01
									DGNPGNDGPPGR	95.0%	80.7	20.6	18	0	0	2	1,152.50
									DYEVDATLK	95.0%	61.9	22.9	21	0	0	2	1,053.51
									EGPVGLPGIDGRPGPIGPAGAR	95.0%	52.5	19.3	9	12	0	2	2,040.10
									EMATQLAFMR	95.0%	82.1	20.7	101	0	0	2	1,229.57
									FTYTVLVDGCSK	95.0%	85.7	23.3	10	0	0	2	1,389.67
									GAAGIPGGK	95.0%	41.7	20.6	7	0	0	2	727.41
									GAAGLPGVAGAPGLPGPR	95.0%	68.9	19.2	48	2	0	2	1,514.84
									GAPGAVGAPGPAGATGDR	95.0%	78.8	23.1	23	0	0	2	1,478.74

GAPGAVGAPGPAGATGDRGEAGAAGPAGPAGPR	95.0%	55.8	21.5	0	42	8	2	2,695.33
GAPGPDGNNGAQGPPGPQGVQGGK	95.0%	93.3	21.2	16	15	0	2	2,114.00
GASGPAGVR	95.0%	56.4	19.9	22	0	0	2	771.41
GDGGPPGMTGFPGAAGR	95.0%	81.5	19.4	67	0	0	2	1,517.68
GEAGAAGPAGPAGPR	95.0%	46.9	22.9	6	0	0	2	1,235.61
GEIGAVGNAGPAGPAGPR	95.0%	140.0	22.7	67	0	0	2	1,547.79
GEIGNPGR	95.0%	46.9	21.1	16	0	0	2	799.41
GEKGEPGLR	95.0%	33.1	21.2	1	0	0	2	942.50
GENGVVGPTGPVGAAGPAGPNGPPGPAGSR	95.0%	91.9	21.3	1	4	0	2	2,551.27
GEPGAPGENGTPGQTGAR	95.0%	93.6	22.3	24	0	0	2	1,652.76
GEPGNIGFPGPK	95.0%	70.3	23.1	36	0	0	2	1,169.60
GEPGSAGPQGPPGPSGEEGK	95.0%	78.2	21.9	16	0	0	2	1,791.82
GEPGSAGPQGPPGPSGEEGKR	95.0%	27.7	22.5	0	1	0	2	1,947.92
GEPGVVGAVGTAGPSGPSGLPGER	95.0%	95.8	21.5	5	0	0	2	2,105.06
GERGPPGESGAAGPTGPIGSR	95.0%	101.0	22.4	9	24	0	2	1,906.94
GERGPPGPPGR	95.0%	38.1	23.5	0	8	0	2	1,076.56
GESGNKGEPGSAGPQGPPGPSGEEGK	95.0%	117.0	19.5	11	65	0	2	2,364.07
GETGPSGPVGPAGAVGPR	95.0%	106.0	22.8	64	0	0	2	1,562.79
GEVGPAGPNGFAGPAGAAGQPGAK	95.0%	53.9	22.8	1	0	0	2	2,035.00
GFPGTPGLPGFK	95.0%	48.9	23.5	10	0	0	2	1,174.63
GHAGLAGAR	95.0%	42.8	21.7	9	0	0	2	809.44
GHNGLDGLKGQPGAPGVK	95.0%	26.5	21.4	0	1	0	2	1,701.90
GIPGPVGAAGATGAR	95.0%	93.7	21.8	36	0	0	2	1,251.68
GLHGEFGLPGPAGPR	95.0%	75.7	23.3	26	0	0	2	1,461.76
GLPGADGR	95.0%	34.3	21.1	3	0	0	2	742.39
GLPGSPGNIGPAGK	95.0%	60.5	22.7	25	0	0	2	1,221.66
GLPGVAGAVGEPGPLGIAGPPGAR	95.0%	131.0	17.1	77	14	0	2	2,067.14
GLVGEPGPAGSK	95.0%	63.1	21.2	14	0	0	2	1,068.57
GLVGEPGPAGSKGESGNK	95.0%	57.5	22.4	2	0	0	2	1,640.82
GPAGDRGPR	95.0%	34.8	21.3	1	0	0	2	882.45
GPAGPSGPAGK	95.0%	60.7	22.6	8	0	0	2	895.46
GPNGDAGRPGEPGLMGPR	95.0%	41.7	22.2	0	16	0	2	1,750.83
GPNGEAGSAGPPGPPGLR	95.0%	76.5	22.5	7	0	0	2	1,587.79
GPPGAAGAPGPQGFQGPAGEPGEPGQTGPAGAR	95.0%	60.8	20.8	0	30	1	2	2,895.38
GPPGAVGSPGVNGAPGEAGRDGNPGNDGPPGR	95.0%	43.8	20.5	0	2	0	2	2,837.33
GPPGESGAAGPTGPIGSR	95.0%	102.0	22.3	54	0	0	2	1,564.77
GPSGPPGPDGNK	95.0%	43.8	22.1	2	0	0	2	1,079.51
GPSGPPGPDGNKGEPGVVGAVGTAGPSGPSGLPGER	95.0%	81.0	20.8	0	27	0	2	3,165.56
GPSGPQGIR	95.0%	52.8	21.5	26	0	0	2	868.46
GQPGAPGVKGEPGAPGENGTPGQTGAR	95.0%	43.9	22.3	0	4	0	2	2,444.19
GSDGSVGPVGPAGPIGSAGPPGFPGAPGPK	95.0%	130.0	21.3	8	12	0	2	2,541.27
GVGLGPGPMGLMGPR	95.0%	69.9	23.2	75	0	0	2	1,427.71
GVVGPQGAR	95.0%	46.1	22.2	10	0	0	2	840.47
GYPGNIGPVGAAGAPGPHGPVGPAGK	95.0%	81.8	21.4	7	17	0	2	2,252.16
HGNRGETGPSGPVGPAGAVGPR	95.0%	97.0	21.9	4	73	64	2	2,027.02
HVWLGETINAGSQFEYNVEGVTSK	95.0%	66.6	21.5	0	15	0	2	2,665.29
NGDKGHAGLAGAR	95.0%	48.7	23.6	0	5	0	2	1,223.62
NSIAYMDEETGNLK	95.0%	91.6	19.8	20	0	0	2	1,600.72
NSIAYMDEETGNLKK	95.0%	78.9	21.8	58	18	0	2	1,712.82
RGPNGEAGSAGPPGPPGLR	95.0%	70.2	22.6	4	8	0	2	1,743.89
SAPSLRPK	95.0%	33.3	18.1	3	0	0	2	855.51

									SLNNQIETLLTPEGSR	95.0%	102.0	22.2	112	8	0	2	1,771.92
									SLNNQIETLLTPEGSRK	95.0%	60.5	20.7	2	6	0	2	1,900.01
									TGEVGAVGPPGFAGEK	95.0%	83.9	21.6	35	0	0	2	1,472.74
									TGHPGTVGPAGIR	95.0%	48.1	22.8	4	35	0	2	1,219.66
									TGPPGPSGISGPPGPPGPAGK	95.0%	109.0	22.1	49	0	0	2	1,781.92
									TIIEYK	95.0%	37.0	18.3	11	0	0	2	766.44
									VGAPGPAGAR	95.0%	67.3	20.3	95	0	0	2	852.47
									VYCDFSTGETCIR	95.0%	75.9	17.2	11	0	0	2	1,607.68
Protein DJ-1	PARK7_HUMAN	PARK7	19,873	100.00%	13	15	93	72.50%	ALVILAK	95.0%	55.3	11.8	15	0	0	2	727.51
									APLVLKD	95.0%	44.8	14.5	19	0	0	2	755.47
									DGLILTSR	95.0%	51.6	24.1	8	0	0	2	874.50
									EGPYDVVVLPGGNLGAQNLSESAAVK	95.0%	51.8	20.8	6	5	0	2	2,584.33
									EILKEQENR	95.0%	43.8	23.7	6	1	0	2	1,158.61
									GAEEMETVIPVDVMR	95.0%	66.1	23.3	14	0	0	2	1,707.79
									GAEEMETVIPVDVMRR	95.0%	46.8	21.9	0	5	0	2	1,863.89
									GLIAAICAGPTALLAHEIGFGSK	95.0%	45.7	18.1	0	4	0	2	2,267.22
									KEGPYDVVVLPGGNLGAQNLSESAAVK	95.0%	53.1	18.1	0	3	0	2	2,712.42
									MMNGGHYTYSENRVEK	95.0%	27.6	18.1	0	1	0	2	1,947.83
									VEKDGLILTSR	95.0%	63.4	20.4	4	0	0	2	1,230.71
									VTTHPLAK	95.0%	32.4	16.0	1	0	0	2	866.51
									VTVAGLAGKDPVQCSR	95.0%	36.1	21.6	0	1	0	2	1,657.87
Putative hydroxypyruvate isomerase	HYI_HUMAN	HYI	30,388	99.50%	2	2	3	11.60%	AEMEAVFLENLR	95.0%	40.9	21.9	1	0	0	2	1,437.70
									ITDPQYFLDTPQQAAAILQK	95.0%	82.7	19.9	2	0	0	2	2,261.18
Heterogeneous nuclear ribonucleoprotein A3	ROA3_HUMAN	HNRNPA3	39,577	100.00%	5	7	22	16.90%	EDSVKPGAHLTVK	95.0%	30.9	21.4	1	0	0	2	1,380.75
									IETIEVMEDR	95.0%	51.7	23.2	5	0	0	2	1,250.59
									IFVGGIKEDTEEYNLR	95.0%	87.6	21.5	3	2	0	2	1,882.96
									SSGSPYGGGYGSGGGSGGYGSR	95.0%	143.0	15.3	7	0	0	2	1,910.79
									YGKIETIEVMEDR	95.0%	55.8	22.0	1	3	0	2	1,598.77
Epididymal secretory protein E1	NPC2_HUMAN	NPC2	16,552	100.00%	5	5	16	43.70%	AVVHGILMGVPVPFPIPEPDGCK	95.0%	26.0	20.3	0	1	0	2	2,445.27
									EVNVSPCPTQPCQLSK	95.0%	66.7	21.2	2	0	0	2	1,843.87
									LPVKSEYPSIK	95.0%	50.1	18.9	5	0	0	2	1,260.72
									SGINCPIQK	95.0%	37.6	22.7	2	0	0	2	1,016.52
									TYSYLNKLPVK	95.0%	55.8	18.9	6	0	0	2	1,325.75
Cochlin	COCH_HUMAN	COCH	59,465	100.00%	7	7	20	13.50%	EFTGLEPIVSDVIR	95.0%	64.0	22.8	9	0	0	2	1,574.84
									ENVLAVIR	95.0%	42.7	19.3	1	0	0	2	913.55
									FFTVDAGVR	95.0%	42.2	22.2	2	0	0	2	1,011.53
									GVISNSGGPVR	95.0%	39.2	22.8	2	0	0	2	1,042.56
									LMLEFVSNIAK	95.0%	51.9	20.5	2	0	0	2	1,280.69
									TEFSFTDYSTK	95.0%	55.5	19.6	2	0	0	2	1,325.59
									TFEISDIGAK	95.0%	35.9	22.3	2	0	0	2	1,080.56
Glycogen phosphorylase, brain form	PYGB_HUMAN	PYGB	96,680	100.00%	34	42	200	44.80%	AAPGYHMAK	95.0%	38.3	21.3	1	0	0	2	961.46
									ARPEYMLPVHFYGR	95.0%	49.2	22.1	0	5	2	2	1,751.87
									DFYELEPEK	95.0%	35.6	21.6	2	0	0	2	1,169.54
									DFYELEPEKFQNK	95.0%	52.3	21.7	3	0	0	2	1,686.80
									DYFFALAHTVR	95.0%	51.5	22.6	4	1	0	2	1,339.68
									EYYDHLPELK	95.0%	43.7	22.5	2	0	0	2	1,306.63
									FSAFLEK	95.0%	36.7	21.5	2	0	0	2	841.45
									GLAGLGDVAEVR	95.0%	91.9	21.4	12	0	0	2	1,156.63
									GLAGLGDVAEVRK	95.0%	63.4	20.5	2	0	0	2	1,284.73
									HLDHVAALFPGDVDR	95.0%	20.5	22.6	0	0	2	2	1,661.84
									HLEIIYAINQR	95.0%	31.5	20.2	0	2	0	2	1,369.76

									IGEEFLTDLSQLK	95.0%	80.7	22.6	9	0	0	2	1,492.79
									IGEEFLTDLSQLKK	95.0%	85.3	20.4	6	9	0	2	1,620.89
									IHSEIVK	95.0%	30.7	16.0	1	0	0	2	825.48
									INMAHLCVIGSHAVNGVAR	95.0%	28.4	22.8	0	1	0	2	2,035.03
									INPSSMFDVHVK	95.0%	47.8	22.6	2	0	0	2	1,389.68
									KLLPLVSDEVFIR	95.0%	48.9	12.6	1	2	0	2	1,528.91
									LLPLVSDEVFIR	95.0%	87.0	17.4	27	0	0	2	1,400.82
									LQDFNVGDYIEAVLDR	95.0%	112.0	22.3	29	1	0	2	1,866.92
									LVTSIGDVVNHDPVVGDR	95.0%	86.3	21.6	4	8	0	2	1,891.99
									NLAENISR	95.0%	38.8	22.9	3	0	0	2	916.49
									QAVDQISSGFFSPK	95.0%	88.8	22.5	2	0	0	2	1,510.75
									QLLNCLHVVTLYNR	95.0%	35.2	20.5	0	2	0	2	1,742.94
									TCAYTNHTVLPEALER	95.0%	36.0	22.4	0	2	0	2	1,874.91
									TNGITPR	95.0%	37.1	22.6	1	0	0	2	758.42
									TVMIGGK	95.0%	36.0	22.9	1	0	0	2	721.39
									VAIQLNDTHPALSIPELMR	95.0%	73.4	19.2	2	4	0	2	2,134.13
									VEDVEALDR	95.0%	52.0	22.7	3	0	0	2	1,045.52
									VEHTPDGVK	95.0%	32.0	21.8	1	0	0	2	981.50
									VIFLENYR	95.0%	52.4	21.0	16	0	0	2	1,053.57
									VIPAADLSQQISTAGTEASGTGNMK	95.0%	101.0	21.9	4	3	0	2	2,463.20
									VLYPNDNFFEGK	95.0%	64.3	21.8	9	0	0	2	1,442.70
									WLDTQVVLAMPYDTPVPGYK	95.0%	80.5	21.4	2	0	0	2	2,309.15
									YEFGIFNQK	95.0%	43.0	22.4	5	0	0	2	1,145.56
Transforming growth factor-beta-induced protein ig-h3	BGH3_HUMAN	TGFBI	74,665	100.00%	5	6	17	9.08%	EGVYTVFAPTNEAFR	95.0%	73.6	21.8	6	0	0	2	1,700.83
									GDELADSALEIFK	95.0%	87.1	22.4	6	0	0	2	1,407.70
									ILGDPEALR	94.6%	30.1	18.4	1	0	0	2	983.55
									SPYQLVLQHSR	95.0%	46.7	21.6	2	0	0	2	1,327.71
									YLYHGQTLETLGGK	95.0%	53.6	22.6	1	1	0	2	1,579.81
Serine/threonine-protein phosphatase 2A 55 kDa regulatory subunit B alpha isoform	2ABA_HUMAN	PPP2R2A	51,675	100.00%	5	5	27	14.30%	LFEEPEDPSNR	95.0%	52.1	20.3	3	0	0	2	1,332.61
									NAAQFLLSTNDK	95.0%	33.4	22.6	1	0	0	2	1,321.68
									SFFSEIISSISDVK	95.0%	81.2	23.5	18	0	0	2	1,558.80
									VPVFRPMDLMVEASPR	95.0%	28.3	22.2	0	3	0	2	1,875.95
									VVIFQQEQENK	95.0%	53.9	23.4	2	0	0	2	1,361.71
Endoplasmic reticulum aminopeptidase 1	ERAP1_HUMAN	ERAP1	107,220	100.00%	10	10	26	12.90%	ASLINNAFQLVSIGK	95.0%	109.0	17.8	5	0	0	2	1,574.89
									DMNEVETQFK	95.0%	32.8	18.9	2	0	0	2	1,256.55
									EMFDDVSYDK	95.0%	34.9	14.0	2	0	0	2	1,264.50
									ESALLFDAEK	95.0%	55.8	23.4	1	0	0	2	1,122.57
									FELGSSSIAHMVMGTTNQFSTR	95.0%	62.1	20.3	0	2	0	2	2,433.12
									GFPLITITVR	95.0%	57.6	15.7	5	0	0	2	1,116.68
									ILASTQFEPTAAR	95.0%	75.5	22.5	3	0	0	2	1,404.75
									TQEFPQILTLIGR	95.0%	72.3	19.0	4	0	0	2	1,515.85
									VGDYFFGK	95.0%	34.0	22.0	1	0	0	2	932.45
									YQFSLSSTEK	95.0%	32.0	22.4	1	0	0	2	1,189.57
Histone H2A type 1-D	H2A1D_HUMAN	HIST1H2AD	14,090	100.00%	4	5	57	30.00%	AGLQFPVGR	95.0%	65.7	22.4	17	0	0	2	944.53
									NDEELNK	95.0%	30.9	20.4	1	0	0	2	861.40
									NDEELNKLLGK	95.0%	51.7	22.7	3	0	0	2	1,272.68
									VTIAQGGVLPNIQAVLLPK	95.0%	89.9	11.1	30	6	0	2	1,931.17
Zinc transporter ZIP10	S39AA_HUMAN	SLC39A10	94,114	100.00%	3	4	47	3.85%	GHQDLDPDNEGELR	95.0%	86.5	19.2	10	6	0	2	1,594.71
									LLTNLGLGER	95.0%	82.6	20.6	21	0	0	2	1,085.63
									LSFFGLEK	95.0%	43.3	20.2	10	0	0	2	940.51
40S ribosomal protein S3a	RS3A_HUMAN	RPS3A	29,927	100.00%	10	11	55	40.50%	APAMFNIR	95.0%	58.7	22.6	10	0	0	2	935.48

									ATGDETGAKVER	95.0%	41.1	22.8	2	0	0	2	1,233.61
									EVQTNDLKEVVNK	95.0%	77.7	22.6	8	0	0	2	1,515.80
									LFCVGFTK	95.0%	41.8	21.0	2	0	0	2	971.50
									LIPDSIGKDIEK	95.0%	48.5	19.9	5	0	0	2	1,327.75
									LITEDVQGK	95.0%	36.5	24.9	2	0	0	2	1,002.55
									LMELHGEGSSSGK	95.0%	69.7	19.6	9	5	0	2	1,347.62
									MMEIMTR	95.0%	34.8	16.3	3	0	0	2	959.40
									TTDGYLLR	95.0%	54.6	20.3	8	0	0	2	938.49
									VFEVSLADLQNDEVAFR	95.0%	50.2	22.0	1	0	0	2	1,951.98
Malate dehydrogenase, cytoplasmic	MDHC_HUMAN	MDH1	36,409	100.00%	14	16	135	45.80%	AICDHVR	95.0%	42.2	20.8	2	0	0	2	870.43
									DLDVAILVGSMPR	95.0%	60.7	22.5	9	0	0	2	1,401.74
									DVIATDKEDVAFK	95.0%	91.0	23.1	9	1	0	2	1,450.74
									ELTEEKESAFEFLSSA	95.0%	66.3	21.5	30	0	0	2	1,816.85
									EVGVYEALKDDSWLK	95.0%	75.7	21.1	3	0	0	2	1,751.89
									FVEGLPINDFSR	95.0%	60.4	23.2	24	0	0	2	1,393.71
									GEFVTTVQQR	95.0%	77.0	23.3	16	0	0	2	1,164.60
									KLSSAMSAAK	95.0%	50.1	22.1	4	0	0	2	993.54
									LGVTANDVK	95.0%	63.9	23.0	15	0	0	2	916.51
									LSSAMSAAK	95.0%	47.9	21.6	2	0	0	2	881.44
									NVIIWGNHSSTQYPDVNHAK	95.0%	43.4	21.8	0	3	0	2	2,280.12
									SQGAALDK	95.0%	38.7	24.9	2	0	0	2	789.41
									SQGAALDKYAK	95.0%	63.9	23.2	12	1	0	2	1,151.61
									VIVVGNPANTNCLTASK	95.0%	99.7	21.4	2	0	0	2	1,757.92
Annexin A5	ANXA5_HUMAN	ANXA5	35,921	100.00%	9	9	20	33.80%	ADAETLRK	95.0%	32.3	23.2	1	0	0	2	903.49
									DLLDDLKSELTGK	95.0%	71.0	22.9	3	0	0	2	1,446.77
									ETSGNLEQLLLAVVK	95.0%	71.4	19.2	3	0	0	2	1,613.91
									GLGTDEESILTLLTSR	95.0%	87.0	22.7	4	0	0	2	1,704.90
									GTVTDFPGFDER	95.0%	35.3	20.7	2	0	0	2	1,340.61
									NFATSLYSMIK	95.0%	48.0	22.2	2	0	0	2	1,290.64
									SEIDLFNIR	95.0%	52.6	23.9	2	0	0	2	1,106.58
									SIPAYLAETLYYAMK	95.0%	39.3	22.9	1	0	0	2	1,749.88
									VLTEIIASR	95.0%	52.9	19.8	2	0	0	2	1,001.60
Transforming growth factor beta-1	TGFB1_HUMAN	TGFB1	44,324	100.00%	3	3	10	11.50%	DNTLQVDINGFTTGR	95.0%	59.6	22.7	1	0	0	2	1,650.81
									EAVPEPVLLSR	95.0%	44.3	18.7	5	0	0	2	1,209.68
									VAGESAEPEPEPEADYYAK	95.0%	65.8	19.2	4	0	0	2	2,051.91
Serine/threonine-protein phosphatase 4 catalytic subunit	PP4C_HUMAN	PPP4C	35,062	99.50%	2	2	4	9.77%	EILVEESNVQR	95.0%	44.6	22.9	2	0	0	2	1,315.69
									VGGDVPETNYLFMGDFVDR	95.0%	76.9	20.6	2	0	0	2	2,146.98
40S ribosomal protein S14	RS14_HUMAN	RPS14	16,255	100.00%	3	3	6	17.20%	IEDVTPIPSDSTR	95.0%	48.9	22.3	2	0	0	2	1,429.72
									TKTPGPGAQSALR	95.0%	52.8	20.5	0	2	0	2	1,283.71
									TPGPGAQSALR	95.0%	66.8	21.3	2	0	0	2	1,054.56
Eukaryotic translation initiation factor 5B	IF2P_HUMAN	EIF5B	138,813	100.00%	6	6	9	6.80%	AQVMEVK	95.0%	31.2	23.5	1	0	0	2	820.42
									DPIVMGVTVEAGQVK	95.0%	93.2	23.0	2	0	0	2	1,558.82
									HFEATDILVSK	95.0%	54.7	22.9	2	0	0	2	1,259.66
									ILPQYIFNSR	95.0%	31.8	21.0	1	0	0	2	1,250.69
									IPGMLIIDTPGHESFSNLR	95.0%	50.7	20.9	0	2	0	2	2,113.08
									LKEGDTIIVPGVEGPIVTQIR	95.0%	45.9	13.4	0	1	0	2	2,234.28
Ribonuclease T2	RNT2_HUMAN	RNASET2	29,463	100.00%	4	5	56	14.80%	ELDLNSVLLK	95.0%	54.7	20.8	34	0	0	2	1,143.66
									LGIKPSINYYQVADFK	95.0%	58.1	19.8	6	12	0	2	1,856.00
									LGIKPSINYYQVADFKDALAR	95.0%	29.6	16.8	0	0	3	2	2,382.28
									VYGVIPK	95.0%	38.5	18.3	1	0	0	2	775.47
6-phosphogluconolactonase	6PGL_HUMAN	PGLS	27,530	100.00%	10	11	34	54.70%	ELPAAVAPAGPASLAR	95.0%	53.6	19.7	3	0	0	2	1,490.83

									FALGLSGGSLVSMLAR	95.0%	99.2	20.2	8	0	0	2	1,594.86
									ILEDQEENPLPAALVQPHTGK	95.0%	76.4	20.6	1	3	0	2	2,299.19
									IVAPISDSPKPPPQR	95.0%	74.9	18.2	2	0	0	2	1,601.90
									LLTVPFEK	95.0%	31.8	17.2	2	0	0	2	946.56
									LPIPESQVITINPELPVEEAAEDYAK	95.0%	29.9	19.6	0	1	0	2	2,865.48
									LPIPESQVITINPELPVEEAAEDYAKK	95.0%	64.3	17.8	0	2	0	2	2,993.57
									LVPFDHAESTYGLYR	95.0%	68.4	22.3	2	0	0	2	1,767.87
									TVIFVATGEGK	95.0%	73.3	21.9	5	0	0	2	1,121.62
									VTLTLPVLNAAR	95.0%	67.9	12.6	5	0	0	2	1,267.77
Small nuclear ribonucleoprotein Sm D3	SMD3_HUMAN	SNRPD3	13,899	99.50%	2	2	7	15.10%	FLILPDMLK	95.0%	36.5	19.8	3	0	0	2	1,089.64
									VAQLEQVVYIR	95.0%	74.8	22.7	4	0	0	2	1,218.68
Heterogeneous nuclear ribonucleoprotein H	HNRH1_HUMAN	HNRNPH1	49,212	100.00%	3	4	65	11.10%	ATENDIYNFFSPLNPVR	95.0%	85.2	22.4	29	0	0	2	1,996.98
									HTGPNSPDTANDGFVR	95.0%	94.1	20.9	8	3	0	2	1,684.77
									STGEAFVQFASQEIAEK	95.0%	108.0	22.0	25	0	0	2	1,841.89
Glyoxalase domain-containing protein 4	GLOD4_HUMAN	GLOD4	34,776	100.00%	7	9	28	26.20%	ELPDLEDLMK	95.0%	52.4	22.1	2	0	0	2	1,218.59
									GGVDHAAAFGR	95.0%	70.7	21.5	4	3	0	2	1,057.52
									ILTPLVSLDTPGK	95.0%	71.2	15.6	7	0	0	2	1,353.80
									IYEKDEEK	95.0%	41.2	23.1	2	0	0	2	1,053.51
									LGNDFMGITLASSQAVSNAR	95.0%	113.0	22.0	2	2	0	2	2,068.01
									SLPQSDPVLK	95.0%	31.0	19.7	2	0	0	2	1,083.61
									VTLAVSDLQK	95.0%	70.8	22.2	4	0	0	2	1,073.62
26S proteasome non-ATPase regulatory subunit 8	PSMD8_HUMAN	PSMD8	29,989	100.00%	3	3	4	15.20%	HPVSLEQYLMEGSYNK	95.0%	40.4	22.3	0	1	0	2	1,910.90
									ILFTEATR	95.0%	34.5	21.1	1	0	0	2	950.53
									LVLLELNFLPTTGTK	95.0%	54.7	12.8	2	0	0	2	1,658.97
C-X-C motif chemokine 5	CXCL5_HUMAN	CXCL5	11,955	100.00%	5	5	9	44.70%	CVCLQTTQGVHPK	95.0%	34.4	21.8	0	1	0	2	1,527.74
									EICLDPEAPFLK	95.0%	34.5	23.3	2	0	0	2	1,431.72
									EICLDPEAPFLKK	95.0%	32.6	23.3	1	0	0	2	1,559.81
									ILDGGNKEN	95.0%	43.3	21.2	2	0	0	2	959.48
									MISNLQVFAIGPQCSK	95.0%	94.3	22.5	3	0	0	2	1,808.90
Myosin-Ic	MYO1C_HUMAN	MYO1C	121,709	100.00%	5	5	7	6.21%	DGTIDFTPGSELLITK	95.0%	43.9	21.8	2	0	0	2	1,706.89
									GEELLSPLNLEQAAYAR	95.0%	69.3	22.0	1	0	0	2	1,873.97
									QLLLTPNAVVIVEDAK	95.0%	35.7	14.6	2	0	0	2	1,723.00
									TSFLLNLR	95.0%	33.0	18.1	1	0	0	2	963.56
									YLGLLENLR	95.0%	33.5	20.0	1	0	0	2	1,090.63
Isoleucyl-tRNA synthetase, cytoplasmic	SYIC_HUMAN	IARS	144,484	100.00%	12	12	36	12.10%	APLKPYPVSPSDK	95.0%	32.1	21.0	0	1	0	2	1,398.76
									EIVVIHQDPEALK	95.0%	40.1	21.3	1	0	0	2	1,490.82
									FLIQNVLR	95.0%	42.3	16.9	8	0	0	2	1,002.61
									GSELEITLTR	95.0%	57.8	22.5	1	0	0	2	1,118.61
									LESDYEILER	95.0%	45.4	22.8	2	0	0	2	1,266.62
									LFLNETQTQEITEDIPVK	95.0%	115.0	21.6	4	0	0	2	2,118.10
									LMAPYTPFLTELMYQNLK	95.0%	56.7	21.7	3	0	0	2	2,205.10
									LYLINSPVVR	95.0%	40.8	17.2	4	0	0	2	1,173.70
									NVIVNGLVLASDGQK	95.0%	88.9	19.4	4	0	0	2	1,526.85
									QLSSEELEQFQK	95.0%	59.7	22.6	4	0	0	2	1,465.72
									TESAVSQMQSVIELGR	95.0%	70.3	22.6	2	0	0	2	1,750.86
									VENMVDQLLR	95.0%	68.2	23.7	2	0	0	2	1,232.63
Calsyntenin-1	CSTN1_HUMAN	CLSTN1	109,774	100.00%	21	26	551	22.90%	AASEFESSEGVFLFPELR	95.0%	78.5	22.3	92	5	0	2	2,014.98
									AMQHISYLNSR	95.0%	56.0	22.2	7	4	0	2	1,335.65
									ATEDVLVK	95.0%	65.3	23.6	15	0	0	2	874.49
									ATVHIQVNDVNEYAPVFK	95.0%	92.2	22.2	19	37	0	2	2,044.05
									ATVIEGK	95.0%	45.2	25.5	13	0	0	2	717.41

									EGLDLQVLEDSGR	95.0%	97.2	22.1	81	0	0	2	1,430.71
									EPFTISVWMR	95.0%	35.7	22.2	3	0	0	2	1,281.63
									GNLAGLTLR	95.0%	67.2	20.8	52	0	0	2	914.54
									GPDGTNVK	95.0%	32.3	20.0	1	0	0	2	787.40
									GVQIQAHPSQLVLTLEGEDLGELDK	95.0%	91.7	19.7	1	31	0	2	2,689.41
									IHGQNVPFDAVVVDK	95.0%	97.9	21.6	38	21	0	2	1,637.87
									IISTITR	95.0%	51.2	17.8	19	0	0	2	803.50
									IPDGVVSVSPK	95.0%	75.1	18.2	65	0	0	2	1,097.62
									ISLSGVHHFAR	95.0%	77.2	20.9	10	0	0	2	1,223.66
									LIFLFR	95.0%	44.0	12.8	16	0	0	2	808.51
									LTVTAYDCGK	95.0%	33.0	22.3	2	0	0	2	1,127.54
									QFPTPGIR	95.0%	34.2	23.1	1	0	0	2	915.51
									QYDSILR	95.0%	49.8	22.4	6	0	0	2	894.47
									SLLDRK	95.0%	36.2	23.2	2	0	0	2	731.44
									STGEGVIR	95.0%	49.8	24.3	4	0	0	2	818.44
									VIDCLYTCK	95.0%	54.1	21.0	6	0	0	2	1,171.55
40S ribosomal protein S21	RS21_HUMAN	RPS21	9,094	100.00%	3	3	8	44.60%	DHASIQMNVAEVDKVTGR	95.0%	68.5	22.4	0	3	0	2	1,985.97
									MGESDDSILR	95.0%	48.2	18.5	3	0	0	2	1,138.51
									TYAICGAIR	95.0%	34.0	21.8	2	0	0	2	1,024.53
Nuclear receptor-binding protein	NRBP_HUMAN	NRBP1	59,827	100.00%	2	2	3	5.42%	NMDTSAVLAEIPAGPGR	95.0%	54.8	22.4	2	0	0	2	1,714.84
									TPTPEPAEVETR	95.0%	34.2	22.9	1	0	0	2	1,326.65
Ferritin heavy chain	FRIH_HUMAN	FTH1	21,208	100.00%	4	5	24	24.60%	ELGDHVTNLR	95.0%	37.8	22.9	1	0	0	2	1,153.60
									IFLQDIK	95.0%	41.6	18.8	6	0	0	2	876.52
									MGAPESGLAEYLFDK	95.0%	65.7	20.9	4	0	0	2	1,643.76
									QNYHQDSEAAINR	95.0%	50.4	19.9	5	8	0	2	1,545.70
Nucleoside diphosphate kinase A	NDKA_HUMAN	NME1	17,131	100.00%	5	8	14	70.40%	DRPFFAGLVK	95.0%	49.0	20.5	2	2	0	2	1,149.64
									FMQASEDLLK	95.0%	45.3	22.9	2	0	0	2	1,181.59
									FMQASEDLLKEHYVDLK	95.0%	27.2	22.7	0	1	0	2	2,082.02
									GDFCIQVGR	95.0%	65.9	22.8	4	0	0	2	1,051.50
									GLVGEIIK	95.0%	49.3	16.7	4	0	0	2	828.52
									GLVGEIIKR	95.0%	53.2	16.7	5	0	0	2	984.62
									NIIHGSDSVESAEK	95.0%	74.8	21.9	2	2	0	2	1,485.72
									TFIAIKPDGVQR	95.0%	36.9	19.8	1	1	0	2	1,344.76
									VMLGETNPADSKPGTIR	95.0%	88.0	22.1	6	16	0	2	1,785.92
									YMHSGPVVAMVWEGLNVVK	95.0%	53.8	22.1	1	2	0	2	2,148.06
T-complex protein 1 subunit beta	TCPB_HUMAN	CCT2	57,472	100.00%	20	27	110	50.10%	AAHSEGNTTAGLDMR	95.0%	72.4	18.7	6	4	0	2	1,546.69
									AGADEERAETAR	95.0%	39.0	20.8	2	0	0	2	1,275.59
									DASLMVTNDGATILK	95.0%	86.7	22.3	4	0	0	2	1,564.79
									EALLSSAVDHGSDEVK	95.0%	70.7	22.2	3	2	0	2	1,656.81
									EAVAMESYAK	95.0%	53.0	19.8	8	0	0	2	1,114.51
									GATQQILDEAER	95.0%	97.4	22.0	7	0	0	2	1,330.66
									IGVNQPK	95.0%	32.2	20.0	2	0	0	2	755.44
									ILIANTGMDTDKIK	95.0%	56.6	21.6	6	0	0	2	1,548.83
									LALVTGGEIASTFDHPELVK	95.0%	63.1	19.6	1	1	0	2	2,097.12
									LAVEAVLR	95.0%	64.1	17.1	6	0	0	2	870.54
									LGGSLADSYLDEGFLLDK	95.0%	76.4	22.5	2	0	0	2	1,912.95
									LGGSLADSYLDEGFLLDKK	95.0%	82.3	22.4	1	7	0	2	2,041.05
									LKGSGNLEAIHIIK	95.0%	51.0	14.5	0	2	0	2	1,492.89
									LTSFIGAIAIGDLVK	95.0%	100.0	14.3	10	0	0	2	1,517.89
									MLPTIIADNAGYDSADLVAQLR	95.0%	122.0	21.4	3	2	0	2	2,363.19
									QDLMNIAGTTLSSK	95.0%	56.1	23.5	2	0	0	2	1,494.75

									QVLLSAAEAAEVILR	95.0%	89.9	15.8	3	0	0	2	1,582.92
									SLHDALCVLAQTVK	95.0%	73.7	20.7	2	0	0	2	1,554.83
									TPGKEAVAMESYAK	95.0%	60.4	21.7	1	1	0	2	1,497.73
									VQDDEVGDGTTSVTVLAAELLR	95.0%	154.0	21.8	13	9	0	2	2,288.16
Peptidyl-prolyl cis-trans isomerase H	PPIH_HUMAN	PPIH	19,190	99.50%	2	2	3	13.00%	IIDGLLVMR	94.9%	30.4	20.9	1	0	0	2	1,045.61
									KIENVPTGPNNKPK	95.0%	36.7	19.5	0	2	0	2	1,535.85
Ubiquitin-conjugating enzyme E2 L3	UB2L3_HUMAN	UBE2L3	17,844	100.00%	5	7	16	36.40%	ADLAEEYSKDR	95.0%	48.2	22.2	1	0	0	2	1,296.61
									IEINFPAEYPFKPPK	95.0%	45.7	21.3	2	4	0	2	1,789.95
									NAEEFTKK	95.0%	31.3	22.5	1	0	0	2	966.49
									TDQVIQSLIALVNDPQPEHPLR	95.0%	110.0	19.2	2	4	0	2	2,483.33
									TDQVIQSLIALVNDPQPEHPLRADLAEEYSK	95.0%	55.9	18.8	0	2	0	2	3,489.79
Heterogeneous nuclear ribonucleoprotein U	HNRPU_HUMAN	HNRNPU	90,567	100.00%	8	8	27	16.50%	AEGGGGGGRPGAPAAGDGKTEQK	95.0%	57.7	21.8	0	6	0	2	2,024.98
									AELMER	95.0%	38.0	22.7	2	0	0	2	764.36
									DIDIHEVR	95.0%	39.9	22.4	4	0	0	2	996.51
									EKPYFPIPEEYTFIQNVPLEDR	95.0%	47.5	21.0	0	6	0	2	2,724.36
									LQAALDDEEAGGRPAMEPGNGSLDLGGDSAGR	95.0%	54.8	18.1	0	2	0	2	3,142.44
									NFILDQTNVSAAAQR	95.0%	44.6	23.6	1	0	0	2	1,647.85
									SSGPTSLFAVTVAPPGAR	95.0%	41.0	21.6	2	0	0	2	1,714.91
									YNILGTNTIMDK	95.0%	50.4	23.0	4	0	0	2	1,398.69
Polypyrimidine tract-binding protein 1	PTBP1_HUMAN	PTBP1	57,205	100.00%	14	16	100	35.40%	DYGNSPLHR	95.0%	36.2	21.6	4	0	0	2	1,058.50
									EGQEDQGLTK	95.0%	39.0	20.8	3	0	0	2	1,104.52
									EGQEDQGLTKDYGNSPLHR	95.0%	52.6	21.6	0	1	0	2	2,144.00
									ENALVQMADGNQAQLAMSHLNGHK	95.0%	21.3	21.6	0	0	2	2	2,609.22
									GQPIYIQFSNHK	95.0%	51.9	23.5	3	0	0	2	1,431.74
									HQNVQLPR	95.0%	46.4	21.4	7	0	0	2	991.54
									IAIPGLAGAGNSVLLVSNLNPER	95.0%	122.0	14.0	27	13	0	2	2,275.28
									KLPIDVTEGEVISLGLPFGK	95.0%	140.0	14.6	5	0	0	2	2,112.20
									LPIDVTEGEVISLGLPFGK	95.0%	90.2	17.6	2	0	0	2	1,984.10
									LSLDGQNIYNACCTLR	95.0%	73.0	21.2	1	0	0	2	1,897.89
									NFQNIFPPSATLHLSNIPPSVSEEDLK	95.0%	41.3	20.6	0	6	0	2	2,994.52
									NNQFQALLQYADPVSAQHAK	95.0%	99.4	21.5	2	14	0	2	2,243.12
									VLFSSNGGVVK	95.0%	46.0	21.1	5	0	0	2	1,106.62
									VTNLLMLK	95.0%	52.0	18.6	5	0	0	2	947.56
Nicotinamide phosphoribosyltransferase	NAMPT_HUMAN	NAMPT	55,505	100.00%	11	14	71	29.10%	AVPEGFVIPR	95.0%	42.3	20.0	4	0	0	2	1,084.62
									DVYKEHFQDDVFNEK	95.0%	39.0	21.3	0	3	0	2	1,912.87
									GTDTVAGLALIK	95.0%	75.9	21.6	12	0	0	2	1,158.67
									KFPVTENSK	95.0%	33.2	21.9	1	0	0	2	1,049.56
									STQAPLIIRPDSGNPLDTVLK	95.0%	61.8	16.1	5	1	0	2	2,235.24
									SYSFDEIRK	95.0%	39.0	22.2	4	0	0	2	1,144.56
									VIQGDGVDINTLQEIVEGMK	95.0%	121.0	21.8	14	2	0	2	2,174.10
									VLEILGK	95.0%	38.6	13.0	3	0	0	2	771.50
									YDGHLPIEIK	95.0%	37.2	22.1	2	1	0	2	1,184.63
									YEETVFYGLQYILNK	95.0%	74.3	21.9	8	0	0	2	1,879.95
									YLLETSGNLDGLEYK	95.0%	104.0	22.5	11	0	0	2	1,714.85
Gem-associated protein 5	GEMI5_HUMAN	GEMIN5	168,573	100.00%	3	3	6	3.51%	IMALGNEDGSIEIFQIPNLK	95.0%	98.7	21.0	2	0	0	2	2,218.14
									TVIESSPESPVTITEPYR	95.0%	89.6	22.0	2	0	0	2	2,005.01
									VGPGAGESPGTPPFR	95.0%	41.4	23.1	2	0	0	2	1,425.71
Glucose-6-phosphate 1-dehydrogenase	G6PD_HUMAN	G6PD	59,240	100.00%	8	9	19	19.80%	DGLLPENTFIVGYAR	95.0%	52.2	22.8	5	0	0	2	1,664.87
									GGYFDEFGIIR	95.0%	56.1	21.9	4	0	0	2	1,273.62
									GPTEADELMKR	95.0%	53.4	22.9	2	0	0	2	1,262.61
									GYLDDPTVPR	95.0%	36.0	22.6	2	0	0	2	1,132.56

									KPGMFFNPEESELDLTYGNR	95.0%	29.6	19.3	0	1	0	2	2,360.09
									LFYLALPPTVYEAVTK	95.0%	51.8	17.0	1	0	0	2	1,825.02
									LKLEDFFAR	95.0%	35.8	20.3	2	1	0	2	1,138.63
									LSNHISSLFR	94.6%	30.1	23.7	1	0	0	2	1,173.64
Probable aminopeptidase NPEPL1	PEPL1_HUMAN	NPEPL1	55,843	100.00%	3	3	8	7.07%	ELGIIPTIIRDEELK	95.0%	56.6	15.8	3	0	0	2	1,739.00
									GFGGIYGVGK	95.0%	40.8	20.9	2	0	0	2	954.51
									TVEINNTDAEGR	95.0%	60.6	21.7	3	0	0	2	1,318.62
Importin subunit alpha-2	IMA2_HUMAN	KPNA2	57,845	100.00%	3	3	7	10.80%	LLGASELPIVTPALR	95.0%	66.2	11.8	4	0	0	2	1,549.93
									NKNPAPPIDAVEQILPTLVR	95.0%	30.6	14.1	0	2	0	2	2,185.23
									QDQIQQVVNHGLVPFLVSVLSK	95.0%	34.4	15.2	0	1	0	2	2,448.36
Eukaryotic translation initiation factor 2 subunit 1	IF2A_HUMAN	EIF2S1	36,095	100.00%	10	10	40	37.80%	ENAEVDGDDDAEEMEAK	95.0%	89.4	6.0	4	0	0	2	1,882.71
									ENAEVDGDDDAEEMEAKAED	95.0%	100.0	4.8	3	0	0	2	2,197.82
									FPEVEDVVMVNVR	95.0%	82.6	23.1	4	0	0	2	1,548.77
									GVFNVQMEPK	95.0%	51.3	22.8	4	0	0	2	1,164.57
									HVAEVLEYTKDEQLESLFQR	95.0%	79.6	21.6	0	4	0	2	2,434.23
									INLIAPPR	95.0%	41.8	13.0	4	0	0	2	893.56
									RPGYGAYDAFK	95.0%	36.2	22.1	2	0	0	2	1,244.61
									TEGLSVLSQAMAVIK	95.0%	63.4	20.2	7	0	0	2	1,562.85
									VVTDTDETELAR	95.0%	93.4	24.0	7	0	0	2	1,348.66
									YVMTTTTLER	95.0%	30.8	22.5	1	0	0	2	1,230.60
Proteasome subunit alpha type-7	PSA7_HUMAN	PSMA7	27,869	100.00%	12	14	49	46.00%	AITVFSPDGHLFQVEYAQEAVK	95.0%	60.8	21.6	2	4	0	2	2,449.24
									AITVFSPDGHLFQVEYAQEAVKK	95.0%	34.2	20.8	0	1	0	2	2,577.34
									ALLEVVQSGGK	95.0%	73.9	22.7	6	0	0	2	1,100.63
									DIVVLGVEKK	95.0%	46.8	18.0	1	0	0	2	1,099.67
									GRDIVVLGVEK	95.0%	62.7	19.2	3	0	0	2	1,184.70
									GSTAVGVR	95.0%	40.8	24.2	1	0	0	2	746.42
									ILNPEEIEK	95.0%	32.1	20.8	1	0	0	2	1,084.59
									KGSTAVGVR	95.0%	41.5	21.7	2	0	0	2	874.51
									LTVEDPVTVEYITR	95.0%	94.7	22.1	8	2	0	2	1,634.86
									LYQTDPSGTYHAWK	95.0%	61.7	21.8	2	0	0	2	1,666.79
									NIELAVMR	95.0%	48.8	23.7	3	0	0	2	961.51
									NYTDEAIETDDLTIK	95.0%	126.0	21.2	13	0	0	2	1,740.82
Cytokine receptor-like factor 1	CRLF1_HUMAN	CRLF1	46,284	99.50%	2	2	12	6.16%	LAGLKPGTVYFVQVR	95.0%	49.9	14.6	0	4	0	2	1,647.96
									VGGLEDQLSVR	95.0%	62.2	22.6	8	0	0	2	1,172.63
Density-regulated protein	DENR_HUMAN	DENR	22,074	99.50%	2	2	7	12.10%	LDADYPLR	95.0%	57.1	23.6	4	0	0	2	962.49
									WPEVDDDSIEDLGEVK	95.0%	75.9	20.6	3	0	0	2	1,845.84
Phenylalanyl-tRNA synthetase alpha chain	SYFA_HUMAN	FARSA	57,547	100.00%	3	3	7	7.68%	LDAEPRPPPTQEAA	95.0%	37.4	23.3	1	0	0	2	1,491.74
									SLQALGEVIEAELR	95.0%	93.3	21.1	4	0	0	2	1,527.84
									VVDSMEDEVQR	95.0%	61.2	19.7	2	0	0	2	1,322.59
Melanotransferrin	TRFM_HUMAN	MFI2	80,223	100.00%	4	4	6	7.99%	CLAEGAGDVAFVK	95.0%	43.4	22.5	1	0	0	2	1,336.66
									HSTVLENTDGK	95.0%	31.2	21.6	1	0	0	2	1,200.59
									LFSHEGSSFQMFSSEAYGQK	95.0%	44.9	18.7	0	2	0	2	2,283.00
									MFDSSNYHGQDLLFK	95.0%	66.9	20.5	2	0	0	2	1,817.82
116 kDa U5 small nuclear ribonucleoprotein component	U5S1_HUMAN	EFTUD2	109,420	100.00%	8	8	20	12.00%	AFIPAIDSFGFETDLR	95.0%	68.5	21.7	3	0	0	2	1,798.90
									FFDDPMLLELAK	95.0%	62.6	22.2	4	0	0	2	1,454.72
									GGGQIIPTAR	95.0%	52.1	20.3	8	0	0	2	969.55
									IAVEPVNPSELPK	95.0%	51.3	19.3	3	0	0	2	1,392.77
									ILDAVVAQEPLHR	95.0%	36.4	20.2	0	3	0	2	1,460.82
									IYADTFGDINYQEFAK	95.0%	65.4	22.6	1	0	0	2	1,894.89
									SFVEFILEPLYK	95.0%	46.6	21.3	3	0	0	2	1,484.80
									STPVTVVLPDTK	95.0%	44.0	21.2	2	0	0	2	1,256.71

									VLSGTIHAGQPVK	95.0%	52.8	17.9	1	0	0	2	1,306.75
mRNA turnover protein 4 homolog	MRT4_HUMAN	MRTO4	27,543	99.50%	2	2	3	16.30%	AAFTVSLDPGPLEQFPHSMEPQLR	95.0%	27.1	21.9	0	1	0	2	2,683.32
									SPSDEYKDNLHQVSK	95.0%	31.4	22.0	0	2	0	2	1,746.83
High mobility group protein B2	HMGB2_HUMAN	HMGB2	24,017	100.00%	8	11	42	28.70%	DKQPYEQK	95.0%	33.0	22.0	2	0	0	2	1,035.51
									HPDSSVNFAEFSKK	95.0%	34.8	23.0	0	1	0	2	1,592.77
									IKSEHPGLSIGDTAK	95.0%	75.1	21.2	3	3	0	2	1,552.83
									KHPDSSVNFAEFSK	95.0%	32.9	23.0	0	1	0	2	1,592.77
									MSSYAFFVQTCR	95.0%	92.3	19.7	7	0	0	2	1,512.66
									SEHPGLSIGDTAK	95.0%	55.2	22.5	2	2	0	2	1,311.65
									SEHPGLSIGDTAKK	95.0%	56.6	22.1	2	0	0	2	1,439.75
									YEKDIAAYR	95.0%	55.9	22.3	10	9	0	2	1,128.57
Heat shock 70 kDa protein 13	HSP13_HUMAN	HSPA13	51,911	100.00%	14	16	54	31.20%	IFTAEELEAEIGR	95.0%	84.7	23.4	3	0	0	2	1,477.75
									IFTAEELEAEIGRYPFK	95.0%	54.6	21.4	3	1	0	2	2,013.03
									ILVPIQQVLK	95.0%	38.3	6.0	4	0	0	2	1,150.76
									KLFDTLNEDLFQK	95.0%	88.8	21.7	4	3	0	2	1,610.84
									LFDTLNEDLFQK	95.0%	84.1	22.3	4	0	0	2	1,482.75
									LGGQDFNQR	95.0%	72.3	23.4	9	0	0	2	1,034.50
									LKEMAEAYLGMPVANAVISVPAEFDLK	95.0%	45.8	19.4	0	4	0	2	2,938.49
									LLQYLYK	95.0%	31.4	18.4	1	0	0	2	940.55
									LSSADDHR	95.0%	49.4	19.9	3	0	0	2	900.42
									QAVEMVK	95.0%	34.5	24.4	1	0	0	2	820.42
									QIYQTYGFVPSR	95.0%	62.5	22.8	4	0	0	2	1,458.74
									SGESQVLFETEISR	95.0%	86.7	22.6	4	0	0	2	1,581.78
									VINEPTAAAMAYGLHK	95.0%	88.3	22.6	4	0	0	2	1,701.86
									VNSGFGR	95.0%	36.2	23.0	2	0	0	2	736.37
Phosphatidylethanolamine-binding protein 1	PEBP1_HUMAN	PEBP1	21,039	100.00%	9	12	72	69.00%	APVAGTCYQAEWDDYVPK	95.0%	128.0	20.1	2	0	0	2	2,069.93
									GNDISSGTVLSDYVGSGPPK	95.0%	120.0	22.7	19	0	0	2	1,949.95
									LYEQLSGK	95.0%	56.5	20.9	14	0	0	2	937.50
									LYTLVLTDPDAPSR	95.0%	78.4	22.5	16	0	0	2	1,560.83
									LYTLVLTDPDAPSRK	95.0%	63.2	20.3	2	2	0	2	1,688.92
									NRPTSISWDGLDSGK	95.0%	77.2	22.9	5	3	0	2	1,632.80
									VLTPTQVK	94.7%	30.2	17.2	1	0	0	2	885.54
									WSGPLSLQEVDEQPQHPLHVTYAGAAVDELGK	95.0%	53.8	19.3	0	3	1	2	3,471.72
									YVWLVYEQDRPLK	95.0%	62.1	21.0	4	0	0	2	1,708.91
Protein FAM49B	FA49B_HUMAN	FAM49B	36,731	100.00%	9	13	42	36.70%	DAEGILEDLQSYR	95.0%	90.9	22.5	6	0	0	2	1,508.72
									DQPPNSVEGLLNALR	95.0%	93.7	21.5	6	0	0	2	1,622.85
									EAIQHPADEKLQEK	95.0%	68.1	22.8	4	4	0	2	1,635.83
									GLLGALTSTPYSPTQHLER	95.0%	70.4	20.8	2	4	0	2	2,041.07
									INNVPAEGENEVNNELANR	95.0%	111.0	21.6	4	1	0	2	2,096.00
									MSLFYAEATPMLK	95.0%	91.8	22.6	2	0	0	2	1,533.73
									MTNPAIQNDFSYYR	95.0%	81.6	21.4	4	0	0	2	1,735.78
									VLKDQPPNSVEGLLNALR	95.0%	69.9	17.0	2	2	0	2	1,963.10
									VMLETPEYR	95.0%	36.2	22.6	1	0	0	2	1,153.56
Importin-11	IPO11_HUMAN	IPO11	112,520	100.00%	3	3	5	4.10%	IFQNMSPLLELSSENLR	95.0%	89.6	21.9	1	0	0	2	2,007.02
									LTVDDFEFR	95.0%	45.0	21.5	2	0	0	2	1,141.55
									VTEDEEPPTEQDKR	95.0%	29.9	20.9	0	2	0	2	1,672.77
F-actin-capping protein subunit alpha-2	CAZA2_HUMAN	CAPZA2	32,931	100.00%	3	3	10	27.60%	FIIHAPPGEFNEVFNDVR	95.0%	42.0	21.8	0	3	0	2	2,101.05
									FTITPSTTQVVGILK	95.0%	89.1	14.1	6	0	0	2	1,604.93
									IDGQQTIIACIESHQFQAK	95.0%	31.1	21.9	0	1	0	2	2,187.09
									IQVHYYEDGNVQLVSHK	95.0%	40.0	22.3	0	10	1	2	2,029.01
									LLLNNDNLLR	95.0%	76.8	19.2	12	0	0	2	1,197.70

Translation initiation factor eIF-2B subunit beta	EI2BB_HUMAN	EIF2B2	38,972	100.00%	2	2	3	8.26%	AVTGTHTLALAAK	95.0%	31.6	19.0	2	0	0	2	1,253.72
									MTAAQPSETTVGNMVR	95.0%	95.0	21.0	1	0	0	2	1,724.79
Beta-glucuronidase	BGLR_HUMAN	GUSB	74,715	100.00%	3	3	7	6.14%	ADFSDNR	95.0%	40.3	17.1	1	0	0	2	824.35
									SLDPSRPVTFVSNSNYAADK	95.0%	49.0	22.1	0	3	0	2	2,168.06
									SLLEQYHLGLDQK	95.0%	56.5	22.2	3	0	0	2	1,543.81
60S ribosomal protein L7	RL7_HUMAN	RPL7	29,210	100.00%	5	5	15	22.60%	AGNFYVPAEPK	95.0%	45.4	22.5	2	0	0	2	1,192.60
									IALTDNALIAR	95.0%	79.1	20.1	6	0	0	2	1,170.68
									LAFVIR	95.0%	34.1	13.8	4	0	0	2	718.46
									QIFNGTFVK	95.0%	36.5	21.6	1	0	0	2	1,053.57
									TTHFVEGGDAGNREDQINR	95.0%	29.5	20.9	0	0	2	2	2,115.98
Leucyl-tRNA synthetase, cytoplasmic	SYLC_HUMAN	LARS	134,453	100.00%	9	9	19	10.50%	FDDPLLGPR	95.0%	52.9	22.7	2	0	0	2	1,029.54
									GFYEGIMLVDGFK	95.0%	58.8	21.9	2	0	0	2	1,491.72
									GTGVVTSVPSDSPDDIAALR	95.0%	67.9	22.3	2	0	0	2	1,956.99
									MIDAGDALIYMEPEK	95.0%	110.0	20.4	2	0	0	2	1,727.79
									QTGEGVGPQEYTLLK	95.0%	61.8	23.1	2	0	0	2	1,619.83
									SFITTDVNPYYDSFVR	95.0%	79.0	22.0	3	0	0	2	1,923.91
									VDIGDTIIYLVH	95.0%	58.8	21.8	2	0	0	2	1,357.74
									VFASELNAGIIK	95.0%	67.6	20.6	2	0	0	2	1,261.72
									VIYVLPMLTIK	95.0%	49.7	13.8	2	0	0	2	1,305.79
Metalloproteinase inhibitor 2	TIMP2_HUMAN	TIMP2	24,382	100.00%	15	19	204	54.10%	AEGDGKMHITLCDFIVPWDTLSTTQK	95.0%	29.1	21.6	0	1	0	2	2,979.42
									AVSEKEVDSGNDIYGNPIK	95.0%	78.5	22.7	4	3	0	2	2,035.00
									EVDSGNDIYGNPIK	95.0%	111.0	22.8	9	0	0	2	1,520.72
									EVDSGNDIYGNPIKR	95.0%	94.8	22.2	38	10	0	2	1,676.82
									EYLIAGK	95.0%	38.3	21.8	17	0	0	2	793.45
									FFACIK	95.0%	31.2	21.1	1	0	0	2	785.40
									FFACIKR	95.0%	32.8	21.0	1	0	0	2	941.50
									GAAPPKQEFLDIEDP	95.0%	53.1	22.6	43	0	0	2	1,626.80
									GPEKDIEFIYTAPSSAVCGVSLDVGGK	95.0%	58.9	21.1	0	2	0	2	2,796.38
									IQYEIK	95.0%	50.8	21.8	45	0	0	2	793.45
									KEYLIAGK	95.0%	52.2	19.8	7	0	0	2	921.54
									MHITLCDFIVPWDTLSTTQK	95.0%	74.8	21.9	4	8	0	2	2,422.18
									MHITLCDFIVPWDTLSTTQKK	95.0%	38.3	21.3	0	2	1	2	2,550.27
									RIQYEIK	95.0%	34.8	20.5	2	0	0	2	949.55
									SDGSCAWYR	95.0%	70.7	15.4	6	0	0	2	1,101.44
Ganglioside GM2 activator	SAP3_HUMAN	GM2A	20,821	100.00%	4	4	10	19.70%	IESVLSSSGK	95.0%	47.3	21.0	1	0	0	2	1,006.54
									IESVLSSSGKR	95.0%	64.1	21.9	3	0	0	2	1,162.64
									SEFVVPDLELPSWLTTGNYR	95.0%	54.6	21.5	1	0	0	2	2,323.16
									VDLVLEK	95.0%	43.6	19.5	5	0	0	2	815.49
Receptor-type tyrosine-protein phosphatase eta	PTPRJ_HUMAN	PTPRJ	145,924	100.00%	2	2	4	1.94%	TPSSTGPSPVFDIK	95.0%	45.1	23.0	2	0	0	2	1,432.73
									VITEPIPVSDLR	95.0%	46.8	18.6	2	0	0	2	1,338.76
Glyceraldehyde-3-phosphate dehydrogenase	G3P_HUMAN	GAPDH	36,035	100.00%	17	27	751	65.10%	AGAHLQGGAK	95.0%	55.0	21.2	9	0	0	2	909.49
									AGAHLQGGAKR	95.0%	33.5	19.4	1	0	0	2	1,065.59
									GALQNIIPASTGAAK	95.0%	95.5	20.6	58	0	0	2	1,411.79
									GILGYTEHQVVSSDFNSDTHSSTFDAGAGIALNDHFVK	95.0%	21.2	18.5	0	0	3	2	4,036.90
									IISNASCTTNCLAPLAK	95.0%	95.8	22.5	6	0	0	2	1,833.92
									LISWYDNEFGYSNR	95.0%	95.3	21.3	14	0	0	2	1,763.80
									LTGMAFR	95.0%	41.6	22.4	10	0	0	2	811.41
									LVINGNPITIFQER	95.0%	88.0	20.6	24	1	0	2	1,613.90
									LVINGNPITIFQERDPSK	95.0%	59.4	18.6	3	2	0	2	2,041.11
									RVIISAPSADAPMFVMGVNHEK	95.0%	59.5	21.7	2	13	1	2	2,401.20
									VIHDNFGIVEGLMTTVHAITATQK	95.0%	89.6	20.4	19	287	176	2	2,611.36

									VIISAPSADAPMFVMGVNHEK	95.0%	55.9	22.0	10	7	0	2	2,245.10
									VIISAPSADAPMFVMGVNHEKYDNSLK	95.0%	34.1	20.5	0	3	0	2	2,965.44
									VPTANVSVVDLTCR	95.0%	119.0	22.8	6	2	0	2	1,530.80
									VVDLMAHMASK	95.0%	46.8	22.4	2	0	0	2	1,233.60
									VVDLMAHMASKE	95.0%	63.7	22.3	42	5	0	2	1,362.64
									WGDAGAEYVVESTGVFTTMEK	95.0%	131.0	20.1	36	9	0	2	2,293.03
LanC-like protein 1	LANC1_HUMAN	LANCL1	45,267	100.00%	2	2	2	8.52%	IPQSHIQQICETILTSGENLAR	95.0%	26.9	20.4	0	1	0	2	2,508.29
									SLAEGYFDAAGR	95.0%	84.8	21.1	1	0	0	2	1,256.59
T-complex protein 1 subunit eta	TCPH_HUMAN	CCT7	59,350	100.00%	18	21	77	44.40%	AEKDNAEIR	95.0%	41.3	21.6	2	0	0	2	1,045.53
									AIKNDSVVAGGGAIEMELSK	95.0%	42.4	21.7	0	3	0	2	2,005.03
									ALEIIPR	95.0%	44.4	15.4	4	0	0	2	811.50
									ATISNDGATILK	95.0%	54.6	22.4	4	0	0	2	1,203.66
									EGTDSSQGIPQLVSNISACQVIAEAVR	95.0%	29.2	20.8	0	1	0	2	2,829.41
									GGAEQFMEETER	95.0%	54.4	16.7	6	0	0	2	1,399.58
									INALTAASEAACLIVSVDETIKNPR	95.0%	35.0	19.3	0	1	0	2	2,656.40
									LLDVVHPAAK	95.0%	51.2	17.0	2	0	0	2	1,062.63
									LPIGDVATQYFADR	95.0%	91.5	23.1	4	0	0	2	1,565.80
									MVVDAVMMLDDLLQLK	95.0%	111.0	21.5	13	3	0	2	1,881.94
									NDSVVAGGGAIEMELSK	95.0%	80.7	22.1	3	0	0	2	1,692.81
									QVKPYVEEGLHPQIIIR	95.0%	21.9	14.5	0	0	2	2	2,019.14
									SLHDAIMIVR	95.0%	32.1	21.2	1	1	0	2	1,170.63
									SQDAEVGDGTTSVTLLAAEFLK	95.0%	156.0	22.1	8	4	0	2	2,252.13
									STVDAPTAAGR	95.0%	63.8	22.0	10	0	0	2	1,045.53
									TATQLAVNK	95.0%	34.7	22.6	2	0	0	2	945.54
									TFSYAGFEMQPK	95.0%	67.2	19.4	2	0	0	2	1,421.64
									VPEEDLKR	95.0%	45.1	20.7	1	0	0	2	985.53
Abhydrolase domain-containing protein 11	ABHDB_HUMAN	ABHD11	34,672	100.00%	3	3	5	14.90%	AINIADELPR	95.0%	42.7	20.3	2	0	0	2	1,111.61
									LADEQLSSVIQDMAVR	95.0%	33.2	22.9	1	0	0	2	1,790.90
									LLDGEAALPAVVFLHGLFGSK	95.0%	31.8	16.0	0	2	0	2	2,154.20
Eukaryotic translation initiation factor 3 subunit D	EIF3D_HUMAN	EIF3D	63,956	100.00%	3	3	6	7.66%	DSSVEVR	95.0%	32.9	23.0	2	0	0	2	791.39
									LGDDIDLIVR	95.0%	87.2	22.4	2	0	0	2	1,128.63
									YNFPNPNPFVEDDMDKNEIASVAYR	95.0%	31.2	19.0	0	2	0	2	2,961.34
Ras GTPase-activating protein-binding protein 2	G3BP2_HUMAN	G3BP2	54,102	100.00%	2	3	5	7.26%	FMQTFVLAPEGSVPNK	95.0%	36.4	22.7	1	0	0	2	1,780.89
									LNVEEK	95.0%	34.3	24.4	2	0	0	2	731.39
									VEAKPEVQSQPPR	95.0%	36.2	21.5	1	3	0	2	1,464.78
GMP synthase [glutamine-hydrolyzing]	GUAA_HUMAN	GMPS	76,699	100.00%	9	10	39	19.50%	DEPDWESLIFLAR	95.0%	31.5	22.6	1	0	0	2	1,590.78
									ELFVQSEIFPLETPAFAIK	95.0%	64.3	18.4	3	0	0	2	2,179.17
									ELGLPEELVSR	95.0%	52.0	20.8	3	0	0	2	1,241.67
									EPPTDVTPTFLTTGVLSTLR	95.0%	105.0	20.0	9	1	0	2	2,145.14
									ISQMPVILTPLHFDRDPLQK	95.0%	25.5	17.3	0	0	3	2	2,364.28
									LMQITSLHSLNAFLLPIK	95.0%	38.6	14.6	0	2	0	2	2,055.17
									SGNIVAGIANESK	95.0%	79.1	23.2	4	0	0	2	1,259.66
									TLNMTTSPEEK	95.0%	46.0	22.7	6	0	0	2	1,266.59
									VVYIFGPPVK	95.0%	44.6	19.1	7	0	0	2	1,118.66
Rho GDP-dissociation inhibitor 1	GDIR1_HUMAN	ARHGDIA	23,190	100.00%	5	7	26	25.50%	AEEYEFLTPVEEAPK	95.0%	77.9	22.6	9	0	0	2	1,751.84
									IDKTDYMVGSYGPR	95.0%	74.8	22.3	5	4	0	2	1,601.76
									SIQEIQELDKDDESLR	95.0%	74.7	22.1	2	2	0	2	1,917.94
									TDYMVGSYGPR	95.0%	48.0	18.4	2	0	0	2	1,261.55
									YIQHTYR	95.0%	33.4	22.8	2	0	0	2	980.50
Malignant T cell-amplified sequence 1	MCTS1_HUMAN	MCTS1	20,538	99.50%	2	2	3	16.00%	LYPAAVDTIVAIMAEGK	95.0%	68.9	20.6	2	0	0	2	1,761.95
									YPFILPHQQVDK	95.0%	30.0	21.1	0	1	0	2	1,484.79

Immunoglobulin superfamily member 8	IGSF8_HUMAN	IGSF8	65,015	99.50%	2	2	2	3.92%	SDLAVEAGAPYAER	95.0%	35.8	23.2	1	0	0	2	1,448.70
									VVAGEVQVQR	95.0%	53.5	20.6	1	0	0	2	1,084.61
Serpin B3	SPB3_HUMAN	SERPINB3	44,548	100.00%	4	4	6	13.60%	DLSMIVLLPNEIDGLQK	95.0%	57.8	20.2	2	0	0	2	1,914.03
									FMFDLFQQFR	95.0%	40.7	22.0	2	0	0	2	1,394.66
									FYQTSVESVDFANAPEESR	95.0%	101.0	19.8	1	0	0	2	2,175.98
									VDLHLPR	95.0%	46.4	20.0	1	0	0	2	849.49
Serine/threonine-protein phosphatase PP1-alpha catalytic subunit	PP1A_HUMAN	PPP1CA	37,496	100.00%	6	7	26	21.20%	AHQVVEDGYEFFAK	95.0%	68.2	22.2	3	2	0	2	1,639.78
									EIFLSQPILLELEAPLK	95.0%	76.9	13.6	11	0	0	2	1,953.13
									IKYPENFFLLR	95.0%	45.5	18.6	0	4	0	2	1,439.81
									NVQLTENEIR	95.0%	60.2	23.7	2	0	0	2	1,215.63
									YGQFSGLNPGGRPITPPR	95.0%	38.7	21.5	0	3	0	2	1,914.00
									YPENFFLLR	95.0%	52.0	21.3	1	0	0	2	1,198.63
Sepiapterin reductase	SPRE_HUMAN	SPR	28,032	100.00%	4	4	9	25.70%	DMLFQVLALEEPNVR	95.0%	62.4	22.0	2	0	0	2	1,789.92
									LLLINNAGSLGDVSK	95.0%	81.6	19.0	2	0	0	2	1,513.86
									VLNYAPGPLDTDMQQLAR	95.0%	31.8	22.2	1	0	0	2	2,018.00
									VPADLGAEAGLQQLLGALR	95.0%	116.0	16.7	4	0	0	2	1,892.06
Alanyl-tRNA synthetase, cytoplasmic	SYAC_HUMAN	AARS	106,795	100.00%	10	11	40	14.80%	AVFDETYPDPVR	95.0%	63.6	22.7	4	0	0	2	1,408.68
									GLEVTDDSPK	95.0%	55.0	21.9	2	0	0	2	1,060.52
									GLVVDMDGFEEER	95.0%	71.7	19.8	2	0	0	2	1,511.67
									GLVVDMDGFEEERK	95.0%	54.8	21.7	1	0	0	2	1,639.76
									MALELLTQEFGIPIER	95.0%	68.8	21.2	13	0	0	2	1,875.99
									MHSPQTSAMLFTVDNEAGK	95.0%	62.2	20.9	0	2	0	2	2,095.94
									MSNYDTDLFVPYFEAIQK	95.0%	77.0	20.7	6	0	0	2	2,197.02
									QFIDSNPNQPLVILEMESGASAK	95.0%	52.2	21.3	1	2	0	2	2,504.23
									TITVALADGGRPDNTGR	95.0%	28.7	22.0	0	1	0	2	1,713.89
									VGAEDADGIDMAYR	95.0%	89.6	19.2	6	0	0	2	1,498.65
60S ribosomal protein L13	RL13_HUMAN	RPL13	24,244	100.00%	4	4	8	19.40%	EAAEQDVEK	95.0%	41.1	19.3	2	0	0	2	1,018.47
									KGDSSAEELK	95.0%	41.2	23.4	3	0	0	2	1,063.53
									LATQLTGPVMPVR	95.0%	47.5	20.0	2	0	0	2	1,398.78
									TIGISVDPR	95.0%	31.5	20.5	1	0	0	2	957.54
Prolyl 3-hydroxylase 1	P3H1_HUMAN	LEPRE1	83,377	100.00%	6	6	22	10.50%	AVGFSSGTENPHGVK	95.0%	66.8	22.4	1	0	0	2	1,486.73
									DLSFFGGLLR	95.0%	66.7	22.4	14	0	0	2	1,124.61
									EGGPLLYEGISLTMNSK	95.0%	68.4	22.4	2	0	0	2	1,824.91
									LTNVAATSGDGYR	95.0%	51.0	22.4	1	0	0	2	1,324.65
									SPYNYLQVAYFK	95.0%	49.8	22.4	2	0	0	2	1,492.75
									TAIEEVQAER	95.0%	62.6	23.0	2	0	0	2	1,145.58
Procollagen galactosyltransferase 1	GT251_HUMAN	GLT25D1	71,620	100.00%	5	6	13	9.97%	MLPVDEFLPVMFDK	95.0%	46.3	21.8	2	0	0	2	1,712.83
									NAAHALPTTLGALER	95.0%	47.0	21.7	2	2	0	2	1,534.83
									NSDVLQSPLDSAARDEL	95.0%	85.0	22.1	2	0	0	2	1,829.89
									TPAYIPIR	95.0%	40.4	22.1	1	0	0	2	930.54
									VLIALLAR	95.0%	54.2	3.0	4	0	0	2	868.60
Phosphoglucomutase-1	PGM1_HUMAN	PGM1	61,433	100.00%	12	13	25	27.90%	ADNFEYSDPVDGSISR	95.0%	89.7	18.2	2	0	0	2	1,771.78
									AIGGIILTASHNPGGPNGDFGIK	95.0%	54.7	20.3	0	2	0	2	2,206.16
									DLEALMFDR	95.0%	45.3	21.5	2	0	0	2	1,125.53
									EAIQLIAR	95.0%	39.8	19.3	2	0	0	2	913.55
									FFGNLMDASK	95.0%	61.5	18.9	2	0	0	2	1,145.53
									FNISNGGPAPEAITDK	95.0%	92.5	22.5	2	0	0	2	1,630.81
									INQDPQVMLAPLISIALK	95.0%	104.0	16.3	3	0	0	2	1,980.12
									LYIDSYEKDVAK	95.0%	57.1	22.7	1	0	0	2	1,443.74
									SIFDFSALK	95.0%	38.2	21.7	2	0	0	2	1,027.55
									TQAYQDQKPGTSGLR	95.0%	70.2	23.1	2	1	0	2	1,649.83

									VDLGVLGK	95.0%	40.1	20.0	2	0	0	2	800.49
									YDYEEVEAEGANK	95.0%	93.8	16.6	2	0	0	2	1,516.64
Cation-independent mannose-6-phosphate receptor	MPRI_HUMAN	IGF2R	274,256	100.00%	14	15	45	6.38%	ATLITFLCDR	95.0%	57.8	22.1	4	0	0	2	1,209.63
									DGAGNSFDLSSLSR	95.0%	85.8	21.6	6	0	0	2	1,425.66
									DGIIVLK	95.0%	32.8	14.9	1	0	0	2	757.48
									DIDTLRDPGSQLR	95.0%	28.5	22.5	0	1	0	2	1,485.77
									DQGSFTEVVSISNLGMAK	95.0%	85.1	22.2	4	0	0	2	1,898.92
									FLHQDIDSGQGIR	95.0%	54.7	22.2	2	6	0	2	1,485.75
									FVCNDDVYSGPLK	95.0%	38.9	21.1	1	0	0	2	1,513.70
									GHQAFDVGQPR	95.0%	63.6	21.4	2	0	0	2	1,211.59
									LSYYDGMIQLNYR	95.0%	39.5	21.6	1	0	0	2	1,651.78
									RYDLSALVR	95.0%	36.4	19.6	1	0	0	2	1,092.62
									SFSLGDIYFK	95.0%	50.9	24.0	2	0	0	2	1,176.59
									TTTGDVQVLGLVHTQK	95.0%	80.2	20.0	2	0	0	2	1,696.92
									VPIDGPPIDIGR	95.0%	67.6	21.5	3	0	0	2	1,248.70
									YDLSALVR	95.0%	54.2	20.6	9	0	0	2	936.52
Basic leucine zipper and W2 domain-containing protein 2	BZW2_HUMAN	BZW2	48,146	100.00%	5	5	16	21.00%	ADVLSEEAILK	95.0%	88.8	22.7	2	0	0	2	1,187.65
									DTLVQGLNEAGDDLEAVAK	95.0%	140.0	22.0	5	0	0	2	1,957.97
									EGIAASFAVK	95.0%	42.3	21.7	2	0	0	2	992.54
									IVVLFYK	95.0%	33.4	14.1	1	0	0	2	881.55
									KEELVAEQALK	95.0%	38.6	21.7	1	0	0	2	1,257.71
									LLELFPVNR	95.0%	46.2	20.4	6	0	0	2	1,100.65
									YADTLFDILVAGSMLAPGGTR	95.0%	70.3	21.9	0	1	0	2	2,184.10
26S protease regulatory subunit 6A	PRS6A_HUMAN	PSMC3	49,187	99.50%	2	2	9	7.74%	QTYFLPVIGLVDAEK	95.0%	71.2	19.2	8	0	0	2	1,692.92
									TMLELLNQLDGFQPNTQVK	95.0%	49.0	21.3	1	0	0	2	2,205.12
26S protease regulatory subunit S10B	PRS10_HUMAN	PSMC6	44,157	99.50%	2	2	11	7.46%	ALQSVGQIVGEVLK	95.0%	85.3	16.6	8	0	0	2	1,440.84
									EVIELPLTNPELFQR	95.0%	44.5	19.4	3	0	0	2	1,797.98
Cystatin-B	CYTB_HUMAN	CSTB	11,121	100.00%	3	6	14	45.90%	SQVVAGTNYFIK	95.0%	66.4	21.1	4	0	0	2	1,326.71
									VFQSLPHENKPLTLSNYQTNK	95.0%	75.6	20.8	2	2	0	2	2,458.27
									VHVGDEDFVHLR	95.0%	58.4	22.8	2	2	2	2	1,422.71
Vacuolar protein sorting-associated protein 26B	VP26B_HUMAN	VPS26B	39,138	99.50%	2	2	3	7.14%	FEGTTSLGEVR	95.0%	55.3	22.6	2	0	0	2	1,195.60
									LFLAGYELTPTMR	95.0%	40.3	21.5	1	0	0	2	1,527.79
Splicing factor 3B subunit 1	SF3B1_HUMAN	SF3B1	145,817	100.00%	12	14	30	14.30%	AAGLATMISTMRPDIDNMDEYVR	95.0%	32.3	19.8	0	2	0	2	2,618.19
									AFAVVASALGIPSLLPFLK	95.0%	37.4	7.8	1	0	0	2	1,914.15
									AIGPHDVLATLLNNLK	95.0%	96.6	15.1	6	2	0	2	1,688.97
									AIGYLIPLMDAEYANYYTR	95.0%	63.0	22.2	1	0	0	2	2,253.09
									ATVNTFGYIAK	95.0%	53.2	21.4	2	0	0	2	1,184.63
									EFGAGPLFNQILPLLMSPTLEDQER	95.0%	44.4	20.8	0	2	0	2	2,831.43
									EVMLILIR	95.0%	30.9	18.5	1	0	0	2	1,002.60
									ILVVIEPLLIDEDYYAR	95.0%	95.9	17.5	2	0	0	2	2,034.12
									QLVDTTVELANK	95.0%	48.8	22.7	2	0	0	2	1,330.72
									SLVEIIEHGLVDEQQK	95.0%	57.1	21.1	4	2	0	2	1,836.97
									TEILPPFFK	95.0%	34.9	21.7	2	0	0	2	1,091.61
									VPELNVQNGVLK	95.0%	43.8	17.9	1	0	0	2	1,309.75
Splicing factor, proline- and glutamine-rich	SFPQ_HUMAN	SFPQ	76,132	100.00%	8	9	26	13.30%	FATHAAALSVR	95.0%	49.4	22.7	1	4	0	2	1,143.63
									FGQGGAGPVGGQGPR	95.0%	61.9	22.5	4	0	0	2	1,341.67
									LFVGNLPADITEDEFK	95.0%	47.9	22.7	2	0	0	2	1,807.91
									LFVGNLPADITEDEFKR	95.0%	33.9	22.1	0	4	0	2	1,964.01
									MGGGGAMNMGDPYGSGGQK	95.0%	65.9	10.4	2	0	0	2	1,819.71
									QGPGPGGPK	95.0%	33.6	22.0	4	0	0	2	794.42
									SPPPGMGLNQNR	95.0%	43.6	21.8	1	0	0	2	1,283.62

									YGEPGEVFINK	95.0%	46.2	22.5	4	0	0	2	1,252.62
Beta-hexosaminidase subunit alpha	HEXA_HUMAN	HEXA	60,672	100.00%	11	12	52	24.40%	ALLSAPWYLNR	95.0%	51.3	21.0	5	0	0	2	1,303.72
									DFYVVEPLAFEGTPEQK	95.0%	77.2	21.6	12	0	0	2	1,968.96
									EDIPVNYMK	95.0%	38.7	21.9	4	0	0	2	1,124.53
									EVIEYAR	95.0%	43.2	22.7	2	0	0	2	879.46
									GLLLDTSR	95.0%	41.4	24.1	2	0	0	2	874.50
									GSYNPVTHIYTAQDVK	95.0%	72.8	22.6	4	0	0	2	1,792.89
									GYVVWQEVFDNK	95.0%	57.4	22.2	2	0	0	2	1,483.72
									HYLPLSSILDTLDVMAYNK	95.0%	84.2	21.5	4	7	0	2	2,209.12
									KGSYNPVTHIYTAQDVK	95.0%	49.6	22.3	0	1	0	2	1,920.98
									LTSDLTFAYER	95.0%	74.5	22.1	3	0	0	2	1,315.65
									SNPEIQDFMR	95.0%	56.4	19.6	6	0	0	2	1,252.56
									TEIEDFPR	95.0%	33.9	22.1	2	0	0	2	1,006.48
NAD(P)H dehydrogenase [quinone] 1	NQO1_HUMAN	NQO1	30,851	100.00%	4	5	16	18.20%	ALIVLAHSER	95.0%	64.0	17.2	2	0	0	2	1,108.65
									EAAAAALKK	95.0%	49.5	21.8	2	0	0	2	872.52
									EGHLSPDIVAEQK	95.0%	69.7	22.4	6	0	0	2	1,422.72
									LKDPANFQYPAESVLAYK	95.0%	81.2	21.2	2	4	0	2	2,054.06
40S ribosomal protein S20	RS20_HUMAN	RPS20	13,355	100.00%	3	3	5	22.70%	DTGKTPVEPEVAIHR	95.0%	46.1	21.8	0	2	0	2	1,648.87
									LIDLHSPSEIVK	95.0%	41.2	19.3	2	0	0	2	1,350.76
									TPVEPEVAIHR	95.0%	30.2	22.8	0	1	0	2	1,247.67
Proliferation-associated protein 2G4	PA2G4_HUMAN	PA2G4	43,769	100.00%	17	20	74	39.10%	AFFSEVER	95.0%	55.3	21.1	4	0	0	2	984.48
									ALLQSSASR	95.0%	53.4	22.0	6	0	0	2	932.52
									EGEFVAQFK	95.0%	44.5	20.9	2	0	0	2	1,054.52
									FDAMPFTLR	95.0%	44.7	21.4	2	0	0	2	1,113.54
									FTVLLMPNGPMR	95.0%	54.4	22.8	4	0	0	2	1,407.71
									GDAMIMEETGK	95.0%	61.5	19.7	6	0	0	2	1,213.51
									HELLQPFNVLYEK	95.0%	70.6	21.2	7	1	0	2	1,629.86
									ITSGPFEPDLYK	95.0%	66.2	22.3	2	0	0	2	1,366.69
									ITSGPFEPDLYKSEMEVQDAELK	95.0%	36.7	21.0	1	2	0	2	2,642.26
									MGGDIANR	95.0%	56.0	21.4	5	0	0	2	849.39
									MGVVECAK	95.0%	31.8	21.8	1	0	0	2	909.42
									SDQDYILK	95.0%	54.6	21.9	6	0	0	2	981.49
									SDQDYILKEGDLVK	95.0%	58.5	22.8	2	1	0	2	1,622.83
									SEMEVQDAELK	95.0%	74.0	20.6	4	0	0	2	1,294.58
									TAENATSGETLEENEAGD	95.0%	94.6	16.0	4	0	0	2	1,837.76
									TIIQNPTDQQK	95.0%	56.7	22.1	11	0	0	2	1,285.68
									TIIQNPTDQQKK	95.0%	47.7	21.1	3	0	0	2	1,413.77
Splicing factor 3A subunit 2	SF3A2_HUMAN	SF3A2	49,237	100.00%	3	3	6	10.60%	MEKPPAPPSLPAGPPGVK	95.0%	27.9	21.4	0	1	0	2	1,785.96
									QLALETIDINKDPYFMK	95.0%	32.0	21.4	0	1	0	2	2,055.05
									TGSGGVASSSESNR	95.0%	80.4	20.3	4	0	0	2	1,295.58
Cytosol aminopeptidase	AMPL_HUMAN	LAP3	56,150	100.00%	4	4	14	11.40%	EKEDDVPQFTSAGENFDK	94.9%	25.8	19.0	0	1	0	2	2,055.92
									GSPNANEPPLVFVGK	95.0%	51.0	22.2	6	0	0	2	1,525.80
									GVLFASGQNLAR	95.0%	62.6	23.0	3	0	0	2	1,232.68
									QLMETPANEMTPTR	95.0%	67.0	21.0	4	0	0	2	1,650.75
Collagen alpha-3(VI) chain	CO6A3_HUMAN	COL6A3	343,645	100.00%	8	8	21	3.31%	AAPLQGMLPGLLAPLR	95.0%	32.3	14.8	1	0	0	2	1,633.95
									ALILVGLER	95.0%	67.2	13.2	4	0	0	2	983.63
									ALNLGYALDYAQR	95.0%	34.3	21.8	1	0	0	2	1,467.76
									ISLSPEYVFSVSTFR	95.0%	36.6	21.8	1	0	0	2	1,731.90
									LLTPITTLTSEQIQK	95.0%	50.5	14.6	4	0	0	2	1,685.97
									LSDAGITPLFLTR	95.0%	61.0	19.4	7	0	0	2	1,403.79
									SSGIVSLGVGDR	95.0%	31.1	23.1	1	0	0	2	1,146.61

									VPQIAFVITGGK	95.0%	38.0	17.2	2	0	0	2	1,229.73
Serine hydroxymethyltransferase, cytosolic	GLYC_HUMAN	SHMT1	53,066	100.00%	5	5	7	16.10%	ALSEALTELGYK	95.0%	46.3	22.0	1	0	0	2	1,294.69
									ISATSIFFESMPYK	95.0%	66.9	22.5	2	0	0	2	1,636.79
									IVTGGSDNHLILVDLR	95.0%	26.4	17.7	0	1	0	2	1,721.96
									MLAQPLKDSDVEVYNIIK	95.0%	27.6	20.0	0	1	0	2	2,092.10
									VNPDTGYINYDQLEENAR	95.0%	88.8	21.5	2	0	0	2	2,110.97
Phosphoribosylformylglycinamidine synthase	PUR4_HUMAN	PFAS	144,706	100.00%	14	16	42	15.20%	AFSITQGLLK	95.0%	39.5	19.0	3	0	0	2	1,077.63
									EAPEPGMEVVK	95.0%	31.8	21.1	1	0	0	2	1,201.58
									ELSDPAGAIIYTSR	95.0%	82.6	22.9	7	0	0	2	1,492.76
									FGEPVLAGFAR	95.0%	79.3	22.3	8	0	0	2	1,163.62
									GQLHVDGQK	95.0%	30.5	22.1	1	0	0	2	981.51
									GVAFVGGFSYADVLGSAK	95.0%	72.5	22.6	4	0	0	2	1,744.89
									KEFFLQR	95.0%	35.5	21.8	1	0	0	2	967.54
									KPPMLQPLALPPGLSVHQALER	95.0%	47.9	14.1	0	2	1	2	2,408.35
									LNFSTPTSTNIVSVCR	95.0%	79.6	21.8	2	0	0	2	1,795.90
									LSFAHPPSAEVEAIALATLHDR	95.0%	41.5	19.8	0	2	0	2	2,345.23
									LVHSLFESIMSTQESSNPNNVLK	95.0%	39.2	21.6	0	3	0	2	2,590.28
									NPSTVEAFDLAQSNSEHSR	95.0%	111.0	21.1	1	2	0	2	2,088.96
									SLGLQLPDGQR	95.0%	49.9	21.4	2	0	0	2	1,183.64
									VGPGPALMLR	95.0%	42.7	19.9	2	0	0	2	1,026.58
Ubiquitin	UBIQ_HUMAN	RPS27A	8,547	100.00%	4	6	108	61.80%	ESTLHLVLR	95.0%	56.2	17.8	7	1	0	2	1,067.62
									IQDKEGIPPDQQR	95.0%	63.7	21.1	22	2	0	2	1,523.78
									TITLEVEPSDTIENVK	95.0%	91.8	21.8	70	0	0	2	1,787.93
									TLSDYNIQK	95.0%	46.9	22.4	6	0	0	2	1,081.55
Cathepsin D	CATD_HUMAN	CTSD	44,535	100.00%	13	17	231	34.20%	DPDAQPGGELMLGGTDSK	95.0%	104.0	20.5	24	0	0	2	1,803.81
									FDGILGMAYPR	95.0%	67.2	21.9	26	0	0	2	1,255.61
									ISVNNVLPVFDNLMQQK	95.0%	71.5	22.3	44	9	0	2	1,975.03
									LVDQNIFSFYLSR	95.0%	86.0	22.3	24	0	0	2	1,601.83
									QPGITFIAAK	95.0%	42.8	20.5	6	0	0	2	1,045.60
									QVFGEATKQPGITFIAAK	95.0%	65.8	19.1	2	2	0	2	1,906.04
									RTMSEVGGSVEDLIAK	95.0%	53.5	22.8	3	2	0	2	1,707.86
									TMSEVGGSVEDLIAK	95.0%	92.9	23.0	24	0	0	2	1,551.76
									VGFAEAAR	95.0%	64.7	25.0	24	0	0	2	820.43
									VGFAEAARL	95.0%	56.5	22.9	7	0	0	2	933.52
									VSTLPAITLK	95.0%	43.5	16.8	6	0	0	2	1,042.65
									YSQAVPAVTEGPIPEVLK	95.0%	96.2	19.4	25	1	0	2	1,898.03
									YYTVFDRDNNR	95.0%	35.7	20.3	2	0	0	2	1,462.67
Histidyl-tRNA synthetase, cytoplasmic	SYHC_HUMAN	HARS	57,394	100.00%	13	13	61	26.70%	AALEELVK	95.0%	33.9	20.8	2	0	0	2	872.51
									ASAELIEEEVAK	95.0%	96.4	23.2	4	0	0	2	1,288.66
									DQGGELLSLR	95.0%	75.0	23.7	6	0	0	2	1,087.58
									EKVFDVIIR	95.0%	48.5	19.1	3	0	0	2	1,118.66
									HGAEVIDTPVFELK	95.0%	74.2	21.5	7	0	0	2	1,554.82
									IFSIVEQR	95.0%	76.5	20.4	5	0	0	2	991.56
									IGDYVQQHGGVSLVEQLLQDPK	95.0%	53.5	20.4	0	15	0	2	2,423.26
									ISFDLSLAR	95.0%	71.6	21.8	2	0	0	2	1,021.57
									QALEGLGDLK	95.0%	48.3	21.6	2	0	0	2	1,043.57
									REDLVEEIK	95.0%	31.3	24.2	2	0	0	2	1,130.61
									TTETQVLVASAQK	95.0%	101.0	22.1	6	0	0	2	1,375.74
									TTETQVLVASAQKK	95.0%	72.4	18.7	2	0	0	2	1,503.84
									YDGLVGMFDPK	95.0%	47.0	21.7	5	0	0	2	1,241.59
Leukotriene A-4 hydrolase	LKHA4_HUMAN	LTA4H	69,269	100.00%	15	18	58	30.40%	ASMHPVTAMLVGK	95.0%	59.2	22.6	2	4	0	2	1,373.69

									DFLYSYFK	95.0%	36.7	22.5	1	0	0	2	1,082.52
									DGETPDPEDPSR	95.0%	37.8	16.4	4	0	0	2	1,314.55
									DGETPDPEDPSRK	95.0%	50.3	19.0	2	1	0	2	1,442.64
									DLSSHQLNEFLAQTLQR	95.0%	94.2	21.8	2	4	0	2	2,000.02
									EDDLNSFNATDLK	95.0%	90.5	20.6	6	0	0	2	1,481.68
									ELVALMSAIR	95.0%	60.1	21.6	2	0	0	2	1,118.62
									GSPMEISLPIALSK	95.0%	65.9	21.5	6	0	0	2	1,458.79
									LTYTAEVSVPK	95.0%	44.3	22.0	2	0	0	2	1,207.66
									LVVDLTDIDPDVAYSSVPYEK	95.0%	94.8	21.9	5	0	0	2	2,338.17
									MQEVYNFNAINNSEIR	95.0%	113.0	20.3	4	0	0	2	1,957.91
									SAYEFSETESMLK	95.0%	89.8	18.6	8	0	0	2	1,537.67
									TFGETHPFTK	95.0%	28.5	23.2	0	1	0	2	1,164.57
									TLTGTAALTVQSQEDNLR	95.0%	93.1	21.7	2	0	0	2	1,917.99
									VVINGQEVK	95.0%	46.3	18.6	2	0	0	2	985.57
60S acidic ribosomal protein P0	RLA0_HUMAN	RPLP0	34,256	100.00%	9	10	63	40.70%	AFLADPSAFVAAAPVAAATTAAPAAAAAPAK	95.0%	51.5	18.3	1	2	0	2	2,752.47
									GHLENNPALEK	95.0%	56.3	23.5	13	0	0	2	1,221.62
									GNVGFVFTK	95.0%	51.2	21.7	8	0	0	2	968.52
									GTIEILSDVQLIK	95.0%	58.7	17.7	10	0	0	2	1,428.83
									IIQLLDDYPK	95.0%	71.3	22.0	6	0	0	2	1,217.68
									SNYFLK	95.0%	42.4	21.0	4	0	0	2	771.40
									TSFFQALGITTK	95.0%	99.9	21.7	9	0	0	2	1,313.71
									VEAKEESEESDEDMGFGLFD	95.0%	58.2	14.6	1	0	0	2	2,278.92
									VLALSVETDYTFPLAEK	95.0%	84.2	20.5	9	0	0	2	1,896.00
Cathepsin L1	CATL1_HUMAN	CTSL1	37,546	100.00%	6	8	90	18.30%	EKGYVTPVK	95.0%	39.8	20.5	2	0	0	2	1,020.57
									GKVFQEPLFYEAPR	95.0%	62.2	21.4	2	0	0	2	1,680.88
									HSFTMAMNAFGDMTSEEFR	95.0%	83.6	11.5	4	15	0	2	2,256.90
									MIELHNQEYR	95.0%	62.9	21.9	11	2	0	2	1,348.63
									QVMNGFQNR	95.0%	40.3	21.7	1	0	0	2	1,109.52
									VFQEPLFYEAPR	95.0%	88.1	21.8	53	0	0	2	1,495.76
Cysteine-rich secretory protein LCCL domain-containing 2	CRLD2_HUMAN	CRISPLD2	55,901	99.50%	2	3	6	6.24%	AAIHYGILDDKGGLVDITR	95.0%	30.5	19.5	0	1	3	2	2,027.09
									TSAVNYMTQVVR	95.0%	42.0	22.6	2	0	0	2	1,384.69
Microtubule-associated protein 4	MAP4_HUMAN	MAP4	120,988	100.00%	2	2	2	2.95%	TTTLSGTAPAAGVVPSR	95.0%	45.2	20.3	1	0	0	2	1,585.86
									VGSLDNVGHLPAGGAVK	95.0%	35.0	21.6	0	1	0	2	1,590.86
Peroxiredoxin-4	PRDX4_HUMAN	PRDX4	30,523	100.00%	6	8	22	32.10%	DYGVYLEDSGHTLR	95.0%	96.1	21.9	3	0	0	2	1,624.76
									GLFIIDDK	95.0%	49.5	20.5	10	0	0	2	920.51
									GLFIIDDKGILR	95.0%	64.0	15.4	23	9	0	2	1,359.80
									IPLLSDLTHQISK	95.0%	57.1	17.1	3	4	0	2	1,464.84
									LVQAFQYTDK	95.0%	64.7	21.3	2	0	0	2	1,212.63
									QGGLGPIRIPLLSDLTHQISK	95.0%	57.6	12.0	0	2	0	2	2,243.29
									QITLNDLPVGR	95.0%	60.2	19.9	5	0	0	2	1,225.69
									SVDETLR	95.0%	46.7	24.7	2	0	0	2	819.42
									VSVADHSLHLSK	95.0%	66.9	21.8	2	1	0	2	1,292.70
Bifunctional aminoacyl-tRNA synthetase	SYEP_HUMAN	EPRS	170,575	100.00%	9	10	25	8.00%	AIQGGTSHHLGQNFSK	95.0%	69.6	22.6	2	0	0	2	1,681.84
									DQDLEPGAPSMGAK	95.0%	61.8	20.2	4	0	0	2	1,431.64
									LTVAENEAETK	95.0%	66.8	23.3	4	0	0	2	1,204.61
									MFEIVFEDPKIPGEK	95.0%	47.4	22.8	2	1	0	2	1,794.90
									NQGGGLSSSGAGEGQGPK	95.0%	75.9	21.4	5	0	0	2	1,587.74
									SGKTELAEPIAIRPTSETVMYPAYAK	95.0%	19.9	19.5	0	0	1	2	2,839.46
									TELAEPIAIRPTSETVMYPAYAK	95.0%	35.4	20.0	0	2	0	2	2,567.31
									THMVVANTMEDFQK	95.0%	79.8	20.1	2	0	0	2	1,682.75
									YYTLFGR	95.0%	43.8	23.9	2	0	0	2	919.47

Eukaryotic translation initiation factor 3 subunit B	EIF3B_HUMAN	EIF3B	92,465	100.00%	9	9	29	16.30%	AQAVSEDAGGNEGR	95.0%	108.0	18.6	4	0	0	2	1,360.61
									DRPQEADGIDSVIVVDNVPQVGPDR	95.0%	35.0	21.0	0	3	0	2	2,690.34
									FAVLHGEAPR	95.0%	39.1	21.6	1	0	0	2	1,096.59
									GTQGVVTNFEIFR	95.0%	63.2	22.1	7	0	0	2	1,467.76
									GTYLATFHQR	95.0%	53.2	23.7	2	0	0	2	1,193.61
									GYIFLEYASPAHAVDAVK	95.0%	53.8	21.5	0	5	0	2	1,951.00
									ITNDFYPEEDGKTK	95.0%	29.5	20.8	0	1	0	2	1,656.78
									MTLDTLSIYETPSMGLLDKK	95.0%	34.4	22.5	0	3	0	2	2,288.14
									VTLMQLPTR	95.0%	48.3	23.2	3	0	0	2	1,074.60
Cytoskeleton-associated protein 5	CKAP5_HUMAN	CKAP5	225,484	100.00%	4	4	5	2.61%	DAAFEALGTALK	95.0%	55.6	22.5	2	0	0	2	1,206.64
									TALAATNPAVR	95.0%	37.9	21.0	1	0	0	2	1,084.61
									TSAQVVLDGLVDKIGDVK	95.0%	40.6	17.9	0	1	0	2	1,857.03
									VNMPAKPAPPTK	95.0%	28.1	20.9	0	1	0	2	1,266.69
Coatomer subunit delta	COPD_HUMAN	ARCN1	57,193	100.00%	4	4	13	9.39%	LFTAESLIGLK	95.0%	85.0	16.9	2	0	0	2	1,191.70
									MHAPPINMESVHMK	95.0%	39.9	20.2	0	4	0	2	1,669.75
									SFPVNSDVGVLK	95.0%	47.6	22.3	2	0	0	2	1,261.68
									VTQVDGNSPVR	95.0%	76.8	22.1	5	0	0	2	1,171.61
Plastin-2	PLSL_HUMAN	LCP1	70,274	99.90%	2	2	6	3.51%	LSPEELLLR	95.0%	81.2	18.9	2	0	0	2	1,069.63
									MINLSVPDTIDER	95.0%	66.0	22.9	4	0	0	2	1,518.75
Angiotensin-converting enzyme	ACE_HUMAN	ACE	149,701	100.00%	5	5	20	4.67%	AALPAQELEEYNK	95.0%	57.6	22.7	4	0	0	2	1,475.74
									AILQFYPK	95.0%	49.2	20.3	4	0	0	2	979.56
									DMVGLDALDAQPLLK	95.0%	65.2	22.1	2	0	0	2	1,614.84
									SMYETPSLEQDLER	95.0%	80.1	19.1	6	0	0	2	1,713.76
									TLGSANLPLAK	95.0%	53.9	20.5	4	0	0	2	1,084.64
Histone-binding protein RBBP4	RBBP4_HUMAN	RBBP4	47,638	100.00%	3	4	23	11.30%	IGEEQSPEDAEDGPPELLFIHGGHTAK	95.0%	76.8	20.2	0	5	2	2	2,873.36
									TPSSDVLVFDYTK	95.0%	104.0	23.0	12	0	0	2	1,471.73
									TVALWDLR	95.0%	60.2	23.2	4	0	0	2	973.55
Fatty acid synthase	FAS_HUMAN	FASN	273,409	100.00%	41	48	400	23.30%	AAEQYTPK	95.0%	39.4	23.4	5	0	0	2	907.45
									ALGLGVEQLPVVFEDVVLHQATILPK	95.0%	36.7	9.0	0	2	0	2	2,785.59
									DLVEAVAHILGIR	95.0%	70.3	15.9	4	2	0	2	1,405.82
									DNLEFFLAGIGR	95.0%	76.1	21.6	11	0	0	2	1,351.70
									DTVTISGPQAPVFEFVEQLR	95.0%	73.2	20.9	2	0	0	2	2,233.15
									DTVTISGPQAPVFEFVEQLRK	95.0%	53.5	19.8	3	1	0	2	2,361.25
									EDGLAQQQTQLNLR	95.0%	90.7	22.0	11	0	0	2	1,613.83
									EQGVTFPSGDIQEQLIR	95.0%	89.6	22.3	9	0	0	2	1,916.97
									FDASFFGVHPK	95.0%	67.8	22.9	12	4	0	2	1,251.62
									FPQLDSTSFANSR	95.0%	89.4	22.0	25	0	0	2	1,469.70
									GLVQALQTK	95.0%	53.1	20.0	12	0	0	2	957.57
									GNAGQSNYGFANSAMER	95.0%	98.1	16.6	5	0	0	2	1,789.76
									GTPLISPLIK	95.0%	39.1	6.0	3	0	0	2	1,038.66
									GVDLVLNSLAEEK	95.0%	55.5	22.6	2	0	0	2	1,386.75
									HGLYLPTR	95.0%	34.3	19.6	3	0	0	2	956.53
									HSQDLAFLSMLNDIAAVPATAMPFR	95.0%	45.0	21.4	0	5	0	2	2,748.35
									IPGLLSPHPLLQLSYTATDR	95.0%	56.8	16.5	0	3	0	2	2,192.21
									LFDHPESPTPNPTEPLFLAQAEVYK	95.0%	73.7	21.2	0	18	0	2	2,840.41
									LHLSGIDANPNALFPPVEFPAPR	95.0%	58.7	19.6	0	9	0	2	2,472.30
									LPEDPLLSGLLDSPALK	95.0%	105.0	17.2	25	0	0	2	1,778.00
									LQVVDQPLPVR	95.0%	65.8	17.2	24	0	0	2	1,263.74
									MVVPGLDGAQIPR	95.0%	42.5	20.7	2	0	0	2	1,368.73
									RPTPQDSPIFLPVDDTSFR	95.0%	60.5	21.2	0	13	0	2	2,188.10
									SDEAVKPFGLK	95.0%	52.6	22.5	7	1	0	2	1,190.64

									SEGVVAVLLTK	95.0%	72.5	19.2	29	0	0	2	1,115.67
									SLLVNPEGPTLMR	95.0%	54.7	21.6	15	0	0	2	1,442.77
									SLYQSAGVAPESFEYIEAHGTGTK	95.0%	39.0	21.9	0	7	0	2	2,542.21
									SNMGHPEPASGLAALAK	95.0%	49.0	22.6	3	9	0	2	1,666.82
									TGGMAFHSYFMEAIAPPLLQELKK	95.0%	26.5	21.0	0	1	0	2	2,711.36
									TGTVSLEVR	95.0%	39.2	22.8	2	0	0	2	961.53
									TLLEGSGLESIISIIHSSLAEPR	95.0%	66.3	17.2	0	2	0	2	2,422.32
									TPEAVQK	95.0%	45.0	19.8	7	0	0	2	772.42
									VGDPQELNGITR	95.0%	83.3	21.5	13	0	0	2	1,298.67
									VLEALLPLK	95.0%	68.2	8.5	21	0	0	2	995.65
									VLEALLPLKGLEER	95.0%	49.7	12.3	2	2	0	2	1,579.94
									VLQGDLVMNVYR	95.0%	77.4	22.3	18	0	0	2	1,422.74
									VSVHVIEGDHR	95.0%	35.2	23.2	0	3	0	2	1,247.65
									VTAIHIDPATHR	95.0%	32.4	22.6	0	2	0	2	1,330.72
									VVVQVLAEEPEAVLK	95.0%	94.6	15.1	34	2	0	2	1,622.94
									VYATILNAGTNTDGFK	95.0%	43.6	21.7	1	0	0	2	1,684.85
									YSGTLNLDR	95.0%	42.2	21.6	4	0	0	2	1,038.52
Hepatoma-derived growth factor	HDGF_HUMAN	HDGF	26,771	100.00%	10	12	52	42.90%	AGDLLEDSPK	95.0%	75.9	21.2	4	0	0	2	1,044.52
									DLFPYEESKEK	95.0%	35.1	21.9	7	0	0	2	1,384.66
									EAATLEVERPLPMEVEK	95.0%	44.5	22.1	2	0	0	2	1,957.00
									EAENPEGEEK	95.0%	41.6	16.9	2	0	0	2	1,131.48
									EAENPEGEEKEAATLEVERPLPMEVEK	95.0%	58.5	20.7	0	8	1	2	3,069.46
									GFSEGLWEIENNPTVK	95.0%	79.8	23.4	6	0	0	2	1,819.89
									GPPQEEEEEEDEEEEATKEDAEAPGIR	95.0%	72.2	14.6	0	3	0	2	3,042.28
									IDEMPEAAVK	95.0%	59.2	22.6	15	0	0	2	1,118.54
									KGFSEGLWEIENNPTVK	95.0%	73.8	21.7	1	2	0	2	1,947.98
									RAGDLLEDSPK	95.0%	36.7	22.5	1	0	0	2	1,200.62
Caprin-1	CAPR1_HUMAN	CAPRIN1	78,346	100.00%	4	5	14	5.78%	LNQDQLDAVSK	95.0%	54.3	23.2	3	0	0	2	1,230.63
									TVLELQYVLDK	95.0%	70.7	19.8	3	0	0	2	1,320.74
									TVLELQYVLDKLGDDEVR	95.0%	32.3	20.1	1	1	0	2	2,105.11
									YQEVTNNLEFAK	95.0%	51.4	22.4	6	0	0	2	1,455.71
Cytoplasmic FMR1-interacting protein 1	CYFP1_HUMAN	CYFIP1	145,169	100.00%	9	9	14	8.14%	DFVSEAYLITLGK	95.0%	49.2	21.1	1	0	0	2	1,455.77
									EFFLELTMGR	95.0%	48.3	21.3	2	0	0	2	1,258.61
									LADQIFAYYK	95.0%	53.8	23.9	1	0	0	2	1,231.64
									NVIQSVLQAIR	95.0%	46.5	17.3	3	0	0	2	1,240.74
									SLLQGTILQYVK	95.0%	46.6	15.7	2	0	0	2	1,362.80
									SSLEGPTILDIEK	95.0%	56.3	21.9	2	0	0	2	1,401.75
									TMLESLIADK	95.0%	35.5	22.4	1	0	0	2	1,136.59
									TVEVLEPEVTK	95.0%	40.2	22.0	1	0	0	2	1,243.68
									YLTLDGFDAMFR	95.0%	38.7	21.9	1	0	0	2	1,464.68
Receptor-type tyrosine-protein phosphatase zeta	PTPRZ_HUMAN	PTPRZ1	254,569	100.00%	5	5	10	2.12%	AIIDGVESVSR	95.0%	77.8	23.8	4	0	0	2	1,145.62
									DSATNQIR	95.0%	37.2	23.2	2	0	0	2	904.45
									QSPINIDEDLTQVNVNLK	95.0%	75.9	22.2	1	0	0	2	2,040.06
									QSPINIDEDLTQVNVNLKK	95.0%	31.8	18.9	0	2	0	2	2,168.16
									VSGGVSEMVFK	95.0%	40.6	22.0	1	0	0	2	1,155.57
Niban-like protein 1	NIBL1_HUMAN	FAM129B	82,666	100.00%	5	6	23	11.30%	FQELIFEDFAR	95.0%	59.3	22.6	8	0	0	2	1,414.70
									ILTSVDQYLELIGNSLPGTTAK	95.0%	48.2	17.6	1	3	0	2	2,333.26
									NHVQPYIPSILEALMVPTSQGFTEVR	95.0%	61.1	19.7	0	5	0	2	2,942.51
									VEGPAFTDAIR	95.0%	83.0	23.4	1	0	0	2	1,175.61
									VQQVQPAMQAVIR	95.0%	61.3	21.6	5	0	0	2	1,483.81
AP-1 complex subunit mu-1	AP1M1_HUMAN	AP1M1	48,570	100.00%	7	7	13	24.60%	AHFGLPSVEAEDKEGKPPISVK	95.0%	36.9	19.7	0	0	2	2	2,335.23

									FEIPYFTTSGIQVR	95.0%	85.3	21.4	2	0	0	2	1,657.86
									HNNLYLVATSK	95.0%	53.1	22.1	2	0	0	2	1,259.67
									STANNVEIHIPVPNDADSPK	95.0%	38.6	21.9	0	2	0	2	2,118.05
									TISFIPPDGEFELMSYR	95.0%	32.1	21.6	1	0	0	2	2,017.96
									VFLSGMPELR	95.0%	49.3	23.3	2	0	0	2	1,164.61
									VVQVFSEYFK	95.0%	55.8	23.6	2	0	0	2	1,245.65
Fumarylacetoacetate hydrolase domain-containing protein 1	FAHD1_HUMAN	FAHD1	24,825	100.00%	3	3	4	30.40%	IITLEEGDIILTGTPK	95.0%	44.3	18.9	1	0	0	2	1,712.97
									QEGETSSMIFSIPYIISYVSK	95.0%	46.1	21.7	1	0	0	2	2,395.17
									SAVLSEPVLFLKPSTAYAPEGSPILMPAYTR	95.0%	53.0	17.2	0	2	0	2	3,321.74
Aldo-keto reductase family 1 member C3	AK1C3_HUMAN	AKR1C3	36,827	100.00%	2	2	10	4.95%	LAIEAGFR	95.0%	42.8	23.1	6	0	0	2	876.49
									TPALIALR	95.0%	56.9	14.8	4	0	0	2	854.55
Importin-5	IPO5_HUMAN	IPO5	123,614	100.00%	13	16	66	17.50%	EFQQYLPVVMGPLMK	95.0%	50.5	22.2	4	0	0	2	1,811.91
									EGFVEYTEQVVK	95.0%	64.0	22.1	4	0	0	2	1,427.71
									FLFDSVSSQNVGLR	95.0%	84.8	21.8	4	0	0	2	1,568.81
									FMQDASDVMQLLLK	95.0%	87.6	22.5	4	0	0	2	1,670.81
									FVPYYDLFMPSLK	95.0%	49.0	23.0	4	0	0	2	1,635.81
									LLSSAFDEVYPALPSDVQTAIK	95.0%	58.4	20.2	4	0	0	2	2,364.23
									LVLEQVVTSIASVADTAEEK	95.0%	96.6	19.7	7	14	0	2	2,102.12
									QAEETYENIPGQSK	95.0%	64.6	21.5	1	0	0	2	1,593.74
									SELLMIIQMETQSSMR	95.0%	99.9	21.0	2	2	0	2	1,944.91
									SLLIPYLDNLVK	95.0%	37.1	14.3	1	0	0	2	1,387.82
									SLVEIADTVPK	95.0%	78.9	22.7	4	0	0	2	1,171.66
									VIAALLQTMEDQGNQR	95.0%	105.0	22.3	4	0	0	2	1,802.91
									VSDILHSIFSSYK	95.0%	65.3	22.7	4	3	0	2	1,495.78
Guanine nucleotide-binding protein G(i) subunit alpha-2	GNAI2_HUMAN	GNAI2	40,434	100.00%	3	3	10	11.80%	AVVYSNTIQSIMAIVK	95.0%	85.4	18.6	2	0	0	2	1,752.96
									IAQSDYIPTQQDVLR	95.0%	59.1	22.2	2	0	0	2	1,746.90
									LLLLGAGESGK	95.0%	61.9	20.1	6	0	0	2	1,057.63
NKG2D ligand 2	N2DL2_HUMAN	ULBP2	27,351	99.50%	2	2	9	11.80%	DIQLENYTPKEPLTLQAR	95.0%	29.4	20.2	0	1	0	2	2,129.12
									EVVDILTEQLR	95.0%	68.6	22.7	8	0	0	2	1,314.73
Protein S100-A7	S10A7_HUMAN	S100A7	11,454	99.50%	2	2	6	21.80%	GTNYLADVFEK	95.0%	54.1	21.9	4	0	0	2	1,256.62
									SIIGMIDMFHK	95.0%	39.3	22.7	2	0	0	2	1,323.64
AP-2 complex subunit alpha-2	AP2A2_HUMAN	AP2A2	103,945	100.00%	9	9	22	16.10%	ALLLSTYIK	95.0%	44.6	12.8	2	0	0	2	1,021.63
									FFQPTEMASQDFFQR	95.0%	61.1	21.0	2	0	0	2	1,894.84
									GLAVFISDIR	95.0%	71.8	20.0	4	0	0	2	1,090.63
									IIGFGSALLEEVDPNPANFVGAGIIHTK	95.0%	59.6	18.3	0	1	0	2	2,879.53
									LSTVASTDILATVLEEMPPFPER	95.0%	26.8	21.2	0	1	0	2	2,532.29
									NNGVLFENQLLQIGLK	95.0%	68.4	18.2	1	0	0	2	1,800.00
									QLSNPQQEVQNIFK	95.0%	60.7	22.7	2	0	0	2	1,672.87
									VGGYILGEFGNLIAGDPR	95.0%	86.4	21.6	7	0	0	2	1,847.97
									YGGTFQNVSVQLPITLNK	95.0%	99.3	19.0	2	0	0	2	1,979.06
Endoplasmic reticulum mannosyl-oligosaccharide 1,2-alpha-mannosidase	MA1B1_HUMAN	MAN1B1	79,564	100.00%	5	5	11	10.30%	ADSYYEYLLK	95.0%	43.3	22.7	6	0	0	2	1,264.61
									DVDVNLFESTIR	95.0%	47.7	22.4	1	0	0	2	1,407.71
									KAEDFGNR	95.0%	44.4	22.5	2	0	0	2	936.45
									QMETGLSPEIVHFNLYPQPGR	95.0%	40.0	21.9	0	1	0	2	2,429.19
									VPSGGYSSINNVQDPQKPEPR	95.0%	26.1	21.9	0	1	0	2	2,269.12
40S ribosomal protein S8	RS8_HUMAN	RPS8	24,188	100.00%	7	8	19	32.70%	ADGYVLEGK	95.0%	50.6	22.6	4	0	0	2	951.48
									ELEFYLR	95.0%	35.6	21.4	1	0	0	2	969.50
									IIDVVYNASNNELVR	95.0%	67.0	21.7	3	2	0	2	1,718.91
									ISSLLEEQFQQGK	95.0%	76.7	22.3	3	0	0	2	1,506.78
									KYELGRPAANTK	95.0%	53.1	21.4	2	0	0	2	1,347.74
									NCIVLIDSTPYR	95.0%	77.5	22.7	2	0	0	2	1,450.74

									YELGRPAANTK	95.0%	31.2	23.7	2	0	0	2	1,219.64
Mucin-1	MUC1_HUMAN	MUC1	122,081	99.50%	2	2	3	1.75%	DISEMFLQIYK	95.0%	35.3	22.6	1	0	0	2	1,402.69
									QGGFLGLSNIK	95.0%	48.5	21.9	2	0	0	2	1,133.63
N-alpha-acetyltransferase 25, NatB auxiliary subunit	NAA25_HUMAN	NAA25	112,280	100.00%	11	12	22	13.00%	DIQYPFLGPVPTR	95.0%	36.4	22.6	1	0	0	2	1,502.80
									DTSEYIIQAYK	95.0%	70.9	22.5	2	0	0	2	1,330.65
									FINQLLGVVPLSTPTEDK	95.0%	83.0	18.5	2	0	0	2	1,971.08
									IPEFIAFR	95.0%	51.4	21.0	2	0	0	2	992.56
									LALPADIR	95.0%	44.2	16.4	1	0	0	2	868.53
									LISGLPSLNHPVEPK	95.0%	38.4	18.5	2	0	0	2	1,600.91
									LLLQQLEATLETGK	95.0%	78.7	17.8	2	0	0	2	1,556.89
									LLLQQLEATLETGKR	95.0%	81.6	16.3	2	2	0	2	1,712.99
									SLLDQLKDVFSK	95.0%	59.0	20.1	2	0	0	2	1,392.77
									VQSSYLHSLLEMGELLK	95.0%	33.0	22.0	0	2	0	2	1,963.02
									YQEALDVIR	95.0%	36.3	23.7	2	0	0	2	1,106.58
Splicing factor, arginine/serine-rich 3	SFRS3_HUMAN	SFRS3	19,312	99.50%	2	3	7	18.30%	AFGYYGPLR	95.0%	42.8	22.3	2	0	0	2	1,043.53
									NPPGFAFVEFEDPRDAADAVR	95.0%	36.3	21.4	1	4	0	2	2,320.10
Poly(rC)-binding protein 2	PCBP2_HUMAN	PCBP2	38,563	100.00%	2	2	22	16.40%	AITIAGIPQSIIECVK	95.0%	51.3	19.0	4	0	0	2	1,712.96
									ESTGAQVQVAGDMLPNSTER	95.0%	97.3	21.7	12	2	0	2	2,105.98
									IITLAGPTNAIFK	95.0%	69.5	16.1	18	0	0	2	1,358.81
									INISEGNCPER	95.0%	42.1	20.3	1	0	0	2	1,288.60
Platelet-derived growth factor D	PDGFD_HUMAN	PDGFD	42,831	100.00%	3	3	10	8.92%	IAEFDTVEDLLK	95.0%	71.2	22.5	4	0	0	2	1,392.73
									LANVVFFPR	95.0%	66.2	19.8	5	0	0	2	1,062.61
									SDDYFVAKPGFK	95.0%	40.2	22.2	1	0	0	2	1,373.67
Calreticulin	CALR_HUMAN	CALR	48,125	100.00%	10	13	25	36.50%	AKIDDPTDSKPEDWDKPEHIPDPDAK	95.0%	21.7	20.5	0	0	1	2	2,959.40
									EQFLDGDGWTSR	95.0%	57.1	19.7	2	0	0	2	1,410.63
									FYALSASFEPFSNK	95.0%	120.0	21.9	2	0	0	2	1,607.77
									FYGDEEKDK	95.0%	47.8	18.4	4	1	0	2	1,130.50
									GLQTSQDAR	95.0%	56.3	23.0	2	0	0	2	975.49
									HEQNIDCGGGYVK	95.0%	52.3	19.5	1	0	0	2	1,476.65
									IDNSQVESGSLEDDWDFLPPKK	95.0%	72.5	21.6	2	2	0	2	2,519.19
									IKDPDASKPEDWDER	95.0%	68.0	21.8	2	0	2	2	1,800.84
									KPEDWDEEMDGEWEPPVIQNPEYK	95.0%	30.4	17.2	0	2	0	2	2,976.29
									VHVIFNYK	95.0%	33.2	21.3	2	0	0	2	1,019.57
Pigment epithelium-derived factor	PEDF_HUMAN	SERPINF1	46,326	100.00%	21	26	861	46.90%	ALYYDLISSPDIHGTYK	95.0%	91.6	21.9	68	32	0	2	1,955.98
									ALYYDLISSPDIHGTYKELLDTVTAPQK	95.0%	58.2	19.2	0	4	0	2	3,151.62
									DTDTGALLFIGK	95.0%	110.0	22.3	143	0	0	2	1,250.66
									EIPDEISILLLGVAHFK	95.0%	50.1	15.8	2	0	0	2	1,894.07
									ELLDTVTAPQK	95.0%	70.3	22.4	57	0	0	2	1,214.66
									IAQLPLTGSMSIIFFLPLK	95.0%	113.0	12.8	59	7	0	2	2,105.21
									IKSSFVAPLEK	95.0%	32.3	18.3	0	2	0	2	1,218.71
									KTSLEDFYLDEER	95.0%	83.8	20.9	32	40	0	2	1,644.78
									LAAAVSNFGYDLYR	95.0%	97.7	23.0	101	0	0	2	1,559.79
									LDLQEINNWVQAQMK	95.0%	84.6	22.4	9	1	0	2	1,845.92
									LKLSYEGEVTK	95.0%	37.8	20.5	2	2	0	2	1,266.69
									LQSLFDSPDFSK	95.0%	82.1	22.8	129	0	0	2	1,383.68
									LSYEGEVTK	95.0%	56.9	22.1	29	0	0	2	1,025.52
									LTQVEHR	95.0%	67.2	21.0	22	0	0	2	882.48
									SLQEMK	95.0%	39.1	23.2	3	0	0	2	751.37
									SSFVAPLEK	95.0%	43.5	21.6	7	0	0	2	977.53
									SYGTRPR	95.0%	36.3	23.0	2	0	0	2	836.44
									TESIIHR	95.0%	42.2	20.4	5	0	0	2	855.47

									TSLEDFYLDEER	95.0%	98.2	19.6	36	0	0	2	1,516.68
									TVQAVLTVPK	95.0%	70.7	15.2	51	0	0	2	1,055.65
									VLTGNPR	95.0%	46.5	19.5	16	0	0	2	756.44
Collagen alpha-2(VI) chain	CO6A2_HUMAN	COL6A2	108,563	100.00%	4	4	14	4.91%	DIASTPHELYR	95.0%	48.3	21.9	2	0	0	2	1,301.65
									NDYATMLPDSTEIDQDTINR	95.0%	97.4	18.8	1	0	0	2	2,328.03
									NLQGISSFR	95.0%	49.6	22.6	6	0	0	2	1,021.54
									VFAVVITDGR	95.0%	55.8	20.2	5	0	0	2	1,076.61
									GPPGLAGAPGLR	95.0%	50.3	19.8	1	0	0	2	1,062.61
									GPVGPSGPPGK	95.0%	37.1	22.7	1	0	0	2	949.51
									INTDEIMTSLK	95.0%	54.9	22.9	1	0	0	2	1,280.64
									SGDRGESGPAGPAGAPGPAGSR	95.0%	27.6	20.7	0	2	0	2	1,907.90
									TVFEYR	95.0%	31.7	20.9	1	0	0	2	814.41
DNA damage-binding protein 1	DDB1_HUMAN	DDB1	126,952	100.00%	30	35	108	30.80%	ALYYLQIHPQELR	95.0%	79.8	20.9	4	12	0	2	1,643.89
									DPNTYFIVGTAMVYPEEAEPK	95.0%	95.6	20.7	2	0	0	2	2,387.11
									EATADDLIK	95.0%	38.6	23.9	1	0	0	2	975.50
									EMLGGEIIPR	95.0%	78.9	23.2	4	0	0	2	1,130.59
									ETDDTLVLSFVGQTR	95.0%	86.0	22.3	2	0	0	2	1,680.84
									GAVYSMVEFNGK	95.0%	43.1	21.2	1	0	0	2	1,317.62
									GDFILVGDLMR	95.0%	54.7	21.8	4	0	0	2	1,251.64
									GESKDLLFILTAK	95.0%	67.9	17.5	2	1	0	2	1,434.82
									IEVQDTSGGTTALRPSASTQALSSSVSSSK	95.0%	75.8	20.2	0	3	0	2	2,952.48
									IGRPSETGIIGIIDPECR	94.6%	25.6	21.4	0	1	0	2	1,983.03
									IVVFQYSDGK	95.0%	60.8	21.7	13	0	0	2	1,155.61
									KTEPATGFIDGDLIESFLDISRPK	95.0%	45.4	19.8	0	2	1	2	2,649.38
									LEELHVIDVK	95.0%	68.5	18.6	4	0	0	2	1,194.67
									LFMLLLEK	95.0%	46.5	18.1	3	0	0	2	1,022.60
									LFMLLLEKEEQMDGTVTLK	95.0%	34.8	21.3	2	0	0	2	2,270.17
									LGKDPNTYFIVGTAMVYPEEAEPK	95.0%	54.6	21.0	0	5	0	2	2,685.31
									LLASINSTVR	95.0%	62.4	20.2	1	0	0	2	1,073.63
									LPSFELLHK	95.0%	45.3	19.2	3	1	0	2	1,083.62
									LVFSNVNLK	95.0%	49.8	19.2	6	0	0	2	1,033.60
									LVSQEPK	95.0%	38.6	21.1	2	0	0	2	800.45
									MQEVVANLQYDDGSGMKR	95.0%	29.0	19.4	0	2	0	2	2,072.94
									QSGESIDIITR	95.0%	73.8	23.8	2	0	0	2	1,218.63
									SDPNRETDDTLVLSFVGQTR	95.0%	27.1	21.5	0	1	0	2	2,250.10
									SFHTER	95.0%	32.6	22.4	1	0	0	2	776.37
									SVLLLAYKPMEGNFEEIAR	95.0%	50.3	20.0	3	10	0	2	2,196.14
									TKGDFILVGDLMR	95.0%	27.0	21.4	0	1	0	2	1,480.78
									VTLGTQPTVLR	95.0%	66.5	18.8	5	0	0	2	1,184.70
									VVEELTR	95.0%	31.0	23.4	1	0	0	2	845.47
									YLAIAPPIIK	95.0%	33.3	16.0	1	0	0	2	1,098.69
									YLLGDMEGR	95.0%	38.4	21.4	1	0	0	2	1,069.50
Protein FAM3C	FAM3C_HUMAN	FAM3C	24,663	100.00%	11	11	39	49.80%	AIQDGTIVLMGTYDDGATK	95.0%	106.0	22.3	5	0	0	2	1,984.95
									AIQDGTIVLMGTYDDGATKLNDEAR	95.0%	48.3	21.4	0	2	0	2	2,683.29
									GINVALANGK	95.0%	53.3	20.2	2	0	0	2	956.55
									ICLEDNVLMSGVK	95.0%	68.1	23.1	2	0	0	2	1,493.73
									LIADLGSTSITNLGFR	95.0%	108.0	19.4	6	0	0	2	1,677.92
									MASGAANVVGPK	95.0%	78.9	22.3	14	0	0	2	1,101.57
									MDASLGNLFAR	95.0%	52.9	22.3	2	0	0	2	1,210.59
									SALDTAAR	95.0%	50.5	23.6	2	0	0	2	804.42
									SPFEQHIK	95.0%	41.7	20.6	1	0	0	2	985.51

									TGEVLDTK	95.0%	36.0	24.2	1	0	0	2	862.45
									TKSPFEQHIK	95.0%	36.3	22.6	0	2	0	2	1,214.65
Parathymosin	PTMS_HUMAN	PTMS	11,512	99.50%	2	3	5	11.80%	AAEEEDEADPK	95.0%	35.7	15.4	1	0	0	2	1,203.50
									AAEEEDEADPKR	95.0%	64.0	19.2	2	2	0	2	1,359.60
N-sulphoglucosamine sulphohydrolase	SPHM_HUMAN	SGSH	56,678	100.00%	4	4	13	9.16%	ADLAAQYTTVGR	95.0%	59.3	23.6	2	0	0	2	1,265.65
									FAQLLEMLR	95.0%	57.4	22.8	4	0	0	2	1,136.61
									MDQGVGLVLQELR	95.0%	94.4	22.3	4	0	0	2	1,473.77
									SLPLLLSQAGVR	95.0%	67.8	15.8	3	0	0	2	1,253.76
60S ribosomal protein L22	RL22_HUMAN	RPL22	14,769	100.00%	3	3	6	19.50%	AGNLGGGVVTIER	95.0%	69.4	22.9	2	0	0	2	1,242.68
									ITVTSEVPFSK	95.0%	48.1	22.0	2	0	0	2	1,207.66
									ITVTSEVPFSKR	95.0%	30.0	19.6	0	2	0	2	1,363.76
Basic leucine zipper and W2 domain-containing protein 1	BZW1_HUMAN	BZW1	48,027	100.00%	6	6	20	17.90%	AEVLSEEPILK	95.0%	53.5	20.2	6	0	0	2	1,227.68
									DINAVAASLR	95.0%	46.9	21.9	5	0	0	2	1,029.57
									IVVLFYK	95.0%	33.4	14.1	1	0	0	2	881.55
									KEELVAEQAIK	95.0%	38.6	21.7	1	0	0	2	1,257.71
									LMELFPANK	95.0%	56.8	23.7	6	0	0	2	1,078.56
									YAETLFDILVAGGMLAPGGTLADDMMR	95.0%	29.3	20.2	0	1	0	2	2,876.35
Sortilin	SORT_HUMAN	SORT1	92,052	99.50%	2	2	3	3.49%	DPIYFTGLASEPGAR	95.0%	58.3	23.2	1	0	0	2	1,593.79
									TEFGMAIGPENSGK	95.0%	38.5	20.5	2	0	0	2	1,453.66
Heterogeneous nuclear ribonucleoprotein A1	ROA1_HUMAN	HNRNPA1	38,729	100.00%	10	14	264	39.50%	DYFEQYGK	95.0%	42.3	18.1	1	0	0	2	1,049.46
									EDSQRPGAHLTVK	95.0%	53.4	22.1	24	0	0	2	1,437.75
									EDTEEHHLR	95.0%	47.4	20.9	2	0	0	2	1,165.52
									GFAFVTFDDHDSVDK	95.0%	66.5	20.1	8	3	0	2	1,699.76
									GFGFVTYATVEEVDAAMNARPHK	95.0%	47.4	22.0	0	0	52	2	2,526.21
									IEVIEIMTDR	95.0%	69.0	23.3	25	0	0	2	1,234.64
									IFVGGIKEDTEEHHLR	95.0%	50.5	21.8	0	11	1	2	1,879.97
									KLFIGGLSFETTDESLR	95.0%	92.4	21.7	2	2	0	2	1,913.00
									LFIGGLSFETTDESLR	95.0%	128.0	21.2	43	0	0	2	1,784.91
									NQGGYGGSSSSSSYGSGR	95.0%	136.0	15.8	54	0	0	2	1,694.70
									SESPKEPEQLR	95.0%	40.2	21.9	14	6	0	2	1,299.65
									SSGPYGGGGQYFAKPR	95.0%	113.0	21.7	18	12	0	2	1,628.78
60S ribosomal protein L15	RL15_HUMAN	RPL15	24,129	100.00%	3	4	5	17.20%	FFEVILIDPFHK	95.0%	34.0	19.7	1	1	0	2	1,504.82
									SLQSVAEER	95.0%	38.7	23.2	2	0	0	2	1,018.52
									VLNSYWVGEDSTYK	95.0%	73.4	21.6	1	0	0	2	1,660.79
UDP-glucuronic acid decarboxylase 1	UXS1_HUMAN	UXS1	47,560	99.50%	2	2	3	9.29%	TNTIGTLNMLGLAK	95.0%	59.9	21.1	1	0	0	2	1,462.79
									VVSNFILQALQGEPLTVYGSGSQTR	95.0%	44.0	20.0	0	2	0	2	2,664.40
N(G),N(G)-dimethylarginine dimethylaminohydrolase 1	DDAH1_HUMAN	DDAH1	31,104	100.00%	9	9	17	32.30%	ALPESLGQHALR	95.0%	45.5	20.8	2	0	0	2	1,291.71
									DENATLDGGDVLFTGR	95.0%	81.7	21.8	1	0	0	2	1,679.79
									DYAVSTVPVADGLHLK	95.0%	64.2	21.8	2	0	0	2	1,684.89
									EFFVGLSK	95.0%	32.9	20.6	1	0	0	2	926.50
									GAEILADTFK	95.0%	45.0	21.8	2	0	0	2	1,064.56
									GAEILADTFKDYAVSTVPVADGLHLK	95.0%	33.5	18.9	0	2	0	2	2,730.44
									LQLNIVEMKDENATLDGGDVLFTGR	95.0%	28.6	21.0	0	1	0	2	2,764.38
									SAKGEEVDVAR	95.0%	37.5	23.3	1	0	0	2	1,160.59
									TPEEYPESAK	95.0%	57.9	21.2	5	0	0	2	1,150.53
Proteasome subunit beta type-6	PSB6_HUMAN	PSMB6	25,340	100.00%	5	6	26	21.30%	DGSSGGVIR	95.0%	64.9	23.6	4	0	0	2	847.43
									LAAIAESGVER	95.0%	79.0	23.0	2	0	0	2	1,115.61
									QVLLGDQIPK	95.0%	53.0	17.8	2	0	0	2	1,110.65
									TTTGSYIANR	95.0%	85.0	22.3	8	0	0	2	1,083.54
									VTDKLTPIHDR	95.0%	61.0	21.0	3	7	0	2	1,294.71
Eukaryotic translation initiation	EIF3M_HUMAN	EIF3M	42,486	100.00%	3	3	8	14.40%	ALKDPNAFLFDHLLTLKPVK	95.0%	29.1	11.5	0	0	2	2	2,280.31

factor 3 subunit M									FYQNNKDFIDSLGLLHEQNMAK	95.0%	26.6	21.3	0	0	2	2	2,641.27
									LLTFMGMAVENK	95.0%	74.4	22.5	4	0	0	2	1,385.68
Ribosyldihydronicotinamide dehydrogenase [quinone]	NQO2_HUMAN	NQO2	25,935	100.00%	7	8	16	37.20%	ATDKDITGTLSNPEVFNYGVETHEAYK	95.0%	77.5	21.1	0	1	0	2	2,999.43
									LALLSVTTGGTAEMYTK	95.0%	83.0	22.3	2	0	0	2	1,771.92
									SLASDITDEQK	95.0%	52.3	22.2	2	0	0	2	1,206.59
									SLASDITDEQKK	95.0%	74.4	23.0	2	0	0	2	1,334.68
									VLAPQISFAPEIASEEER	95.0%	90.3	21.5	3	2	0	2	1,986.02
									VLAPQISFAPEIASEEERK	95.0%	26.3	20.8	0	1	0	2	2,114.11
									VLIVYAHQEPK	95.0%	56.5	18.6	3	0	0	2	1,296.73
Alpha-centractin	ACTZ_HUMAN	ACTR1A	42,597	100.00%	5	5	13	24.70%	AGFAGDQIPK	95.0%	57.6	22.6	2	0	0	2	1,003.52
									DQLQTFSEEHPVLLTEAPLNPR	95.0%	55.3	20.3	0	2	0	2	2,534.29
									TLFSNIVLSGGSTLFK	95.0%	107.0	19.6	3	0	0	2	1,683.93
									TTGVVLDSGDGVTHAVPIYEGFAMPHSIMR	95.0%	57.6	20.7	0	2	0	2	3,189.54
									VMAGALEGDIFIGPK	95.0%	84.9	22.4	4	0	0	2	1,533.80
Amyloid beta A4 protein	A4_HUMAN	APP	86,923	100.00%	21	34	855	27.00%	AVIQHFQEK	95.0%	37.5	21.7	14	0	0	2	1,099.59
									AVIQHFQEKVESLEQEAANER	95.0%	128.0	20.8	2	49	3	2	2,455.22
									CLVGEFVSDALLVPDK	95.0%	87.3	22.6	17	0	0	2	1,761.91
									EQNYSDDVLANMISEPR	95.0%	124.0	19.6	51	5	0	2	1,996.89
									GLTTRPGSGLTNIK	95.0%	62.0	20.2	20	0	0	2	1,414.80
									GLTTRPGSGLTNIKTEEISEVK	95.0%	46.3	18.3	0	4	2	2	2,330.26
									ISYGNDALMPSLTETK	95.0%	94.4	22.3	85	0	0	2	1,755.85
									LALENYITALQAVPPRPR	95.0%	84.2	14.0	31	172	12	2	2,022.15
									LNMHMNVQNGK	95.0%	32.0	20.6	1	0	0	2	1,317.60
									QQLVETHMAR	95.0%	52.3	22.9	21	9	0	2	1,228.61
									RLALENYITALQAVPPRPR	95.0%	47.4	11.1	0	26	17	2	2,178.25
									SQVMTHLR	95.0%	42.2	22.1	20	0	0	2	987.50
									STNLHDYGMLLPCGIDK	95.0%	63.4	20.8	3	3	0	2	1,949.91
									STNLHDYGMLLPCGIDKFR	95.0%	39.5	22.4	0	3	0	2	2,253.08
									TEEISEVK	95.0%	44.3	24.1	8	0	0	2	934.47
									TEEISEVKMDAEFR	95.0%	73.9	21.2	5	5	0	2	1,699.79
									THPHFVIPYR	95.0%	37.3	21.4	4	103	0	2	1,266.67
									VEAMLNDR	95.0%	59.7	21.9	23	0	0	2	963.46
									VESLEQEAANER	95.0%	82.4	21.6	106	0	0	2	1,374.65
									WYFDVTEGK	95.0%	42.3	19.8	12	0	0	2	1,144.53
									YLETPGDENEHAHFQK	95.0%	81.7	20.3	11	7	1	2	1,914.86
Phosphoacetylglucosamine mutase	AGM1_HUMAN	PGM3	59,834	99.50%	2	2	2	5.54%	AFVRPSGTEDVVR	95.0%	30.6	22.7	1	0	0	2	1,432.75
									QAVTPPGLQEAINDLVK	95.0%	33.9	20.0	1	0	0	2	1,792.98
Fumarylacetoacetase	FAAA_HUMAN	FAH	46,358	100.00%	6	6	18	18.40%	ASSVVVSGTPIR	95.0%	56.0	21.9	4	0	0	2	1,172.66
									HLFTGPVLSK	95.0%	41.2	18.9	2	0	0	2	1,098.63
									IGVAIGDQILDLSIIK	95.0%	87.8	10.0	5	0	0	2	1,668.00
									LGEPIPISK	95.0%	47.2	14.9	2	0	0	2	953.57
									LLDMELEMAFFVGPGNR	95.0%	47.3	21.7	1	0	0	2	1,970.94
									VFLQNLLSVSQAR	95.0%	82.5	18.3	4	0	0	2	1,474.84
ProSAAS	PCSK1_HUMAN	PCSK1N	27,355	99.50%	2	2	4	11.20%	ARAEAQEAEDQQAR	95.0%	42.6	22.5	0	2	0	2	1,572.74
									ILAGSADSEGVAAPR	95.0%	102.0	21.6	2	0	0	2	1,413.73
Peroxiredoxin-1	PRDX1_HUMAN	PRDX1	22,093	100.00%	19	24	226	67.30%	ADEGISFR	95.0%	59.4	22.6	14	0	0	2	894.43
									AEEFKK	95.0%	30.6	23.1	1	0	0	2	751.40
									ATAVMPDGQFK	95.0%	52.2	22.6	22	0	0	2	1,180.57
									ATAVMPDGQFKDISLSDYK	95.0%	75.7	22.5	4	3	0	2	2,086.02
									DISLSDYK	95.0%	49.0	21.6	6	0	0	2	940.46
									DISLSDYKGK	94.6%	30.1	22.3	1	0	0	2	1,125.58

									GLFIIDDK	95.0%	49.5	20.5	10	0	0	2	920.51
									GLFIIDDKGILR	95.0%	64.0	15.4	23	9	0	2	1,359.80
									HGEVCPAGWKPGSDTIKPDVQK	95.0%	40.8	22.4	0	2	2	2	2,406.19
									IGHPAPNFK	95.0%	33.6	20.8	1	0	0	2	980.53
									KQGGLGPMNIPLVSDPK	95.0%	70.4	18.3	4	2	0	2	1,750.95
									LVQAFQFTDK	95.0%	62.7	22.0	43	0	0	2	1,196.63
									QGGLGPMNIPLVSDPK	95.0%	91.8	22.0	15	0	0	2	1,638.85
									QGGLGPMNIPLVSDPKR	95.0%	52.3	19.6	4	4	0	2	1,778.96
									QITVNDLPVGR	95.0%	82.6	20.9	28	0	0	2	1,211.68
									RTIAQDYGVLK	95.0%	43.1	21.1	1	0	0	2	1,263.71
									SVDETLR	95.0%	46.7	24.7	2	0	0	2	819.42
									TIAQDYGVLK	95.0%	71.8	22.1	24	0	0	2	1,107.61
									TIAQDYGVLKADEGISFR	95.0%	26.1	21.8	0	1	0	2	1,983.02
Proteasome subunit alpha type-3	PSA3_HUMAN	PSMA3	28,416	100.00%	11	12	59	42.40%	AVENSSTAIGIR	95.0%	78.6	23.2	5	0	0	2	1,217.65
									DIREEAEK	95.0%	31.2	22.9	1	0	0	2	989.49
									EEASNFR	95.0%	34.2	21.0	2	0	0	2	852.39
									ESLKEEDESDDDNM	95.0%	86.1	7.8	16	0	0	2	1,671.62
									HEIVPK	95.0%	32.8	19.7	1	0	0	2	722.42
									HVGMAVAGLLADAR	95.0%	83.2	21.8	6	7	0	2	1,396.74
									IIYIVHDEVKDK	95.0%	56.1	20.4	4	0	0	2	1,471.82
									LYEEGSNKR	95.0%	46.5	21.7	4	0	0	2	1,095.54
									SLADIAREEASNFR	95.0%	28.7	23.0	0	1	0	2	1,578.79
									SNFGYNIPLK	95.0%	58.2	22.6	4	0	0	2	1,152.61
									VFQVEYAMK	95.0%	64.0	21.9	8	0	0	2	1,130.56
Tyrosine-protein kinase-like 7	PTK7_HUMAN	PTK7	118,374	100.00%	6	7	23	7.85%	ADGSSLPEWVTDNAGTLHFAR	95.0%	38.8	22.0	0	3	0	2	2,244.07
									DDVTGEEAR	95.0%	50.9	15.9	3	0	0	2	991.43
									FAQGSSLSFAAVDR	95.0%	65.4	22.7	1	0	0	2	1,455.72
									HPASEAEIQPQTQVTLR	95.0%	103.0	22.5	3	5	0	2	1,904.98
									QPSSQDALQGR	95.0%	54.4	22.0	2	0	0	2	1,186.58
									VVLAPQDVVVAR	95.0%	69.4	14.5	6	0	0	2	1,265.76
