# Supplementary material for: Secretome-Based Identification of ULBP2 as a Novel Serum Marker for Pancreatic Cancer Detection
Source: PLoS One. 2011 May 20;6(5):e20029. doi: 10.1371/journal.pone.0020029 (PMC3098863; doi:10.1371/journal.pone.0020029)
Supplement: Table S3 — Correlation between clinicopathological features and BIGH3 expression in tissue sections from 31 pancreatic cancer patients. (PDF) [file pone.0020029.s007.pdf]

Supporting Table S3. Correlation between clinicopathological features and BIGH3 expression in tissue sections from 31 pancreatic cancer patients

Characteristics	Patient No.	IHC score (Mean ± SD)[a]	*p*-value
Gender			
Male	19	0.63 ± 0.50	0.141[b]
Female	12	0.39 ± 0.37	
Age (years)			
< 64[c]	16	0.52 ± 0.42	1.000[b]
≥ 64	15	0.55 ± 0.51	
Histological grade[d]			
Well differentiation	12	0.53 ± 0.36	0.115[e]
Moderate differentiation	13	0.56 ± 0.53	
Poor differentiation	4	0.17 ± 0.19	
Overall stage			
stage I	6	0.67 ± 0.56	0.085[e]
stage II	23	0.55 ± 0.43	
stage IV	2	0.00 ± 0.00	
Tumor-node-metastasis (TNM)-T classification			
TNM-T2	6	0.67 ± 0.56	0.575[b]
TNM-T3	25	0.51 ± 0.44	
TNM-N classification			
TNM-N0	13	0.56 ± 0.39	0.557[b]
TNM-N1	18	0.52 ± 0.51	
TNM-M classification			
No metastasis	29	0.57 ± 0.45	0.026[b]
Distant metastasis	2	0.00 ± 0.00	

[a] Intensity and percentage scores of cell staining were multiplied and then divided by 3 to get the IHC scores.

[b] The *p*-values were determined using Wilcoxon test.

[c] Median.

[d] Histological grade information not available in 2 patients.

[e] The *p*-values were determined using Kruskal-Wallis test.
