# Supplementary material for: Secretome-Based Identification of ULBP2 as a Novel Serum Marker for Pancreatic Cancer Detection
Source: PLoS One. 2011 May 20;6(5):e20029. doi: 10.1371/journal.pone.0020029 (PMC3098863; doi:10.1371/journal.pone.0020029)
Supplement: Table S4 — Correlation between clinicopathological features and ULBP2 expression in tissue sections from 67 pancreatic cancer patients. (PDF) [file pone.0020029.s008.pdf]

Supporting Table S4. Correlation between clinicopathological features and ULBP2 expression in tissue sections from 67 pancreatic cancer patients

Characteristics	Patient No.	IHC score (Mean ± SD)[a]	*p*-value
Gender			
Male	44	2.64 ± 0.54	0.180[b]
Female	23	2.83 ± 0.36	
Age (years)			
< 64[c]	33	2.61 ± 0.55	0.089[b]
≥ 64	34	2.80 ± 0.41	
Histological grade[d]			
Well differentiation	23	2.71 ± 0.59	0.746[e]
Moderate differentiation	31	2.68 ± 0.44	
Poor differentiation	10	2.70 ± 0.48	
Overall stage			
stage I	9	2.52 ± 0.82	0.593[e]
stage II	56	2.73 ± 0.43	
stage IV	2	3.00 ± 0.00	
Tumor-node-metastasis (TNM)-T classification			
TNM-T2	9	2.52 ± 0.82	0.653[b]
TNM-T3	58	2.74 ± 0.42	
TNM-N classification			
TNM-N0	25	2.69 ± 0.59	0.806[b]
TNM-N1	42	2.72 ± 0.43	
TNM-M classification			
No metastasis	65	2.70 ± 0.50	0.479[b]
Distant metastasis	2	3.00 ± 0.00	

[a] Intensity and percentage scores of cell staining were multiplied and then divided by 3 to get the IHC scores.

[b] The *p*-values were determined using Wilcoxon test.

[c] Median.

[d] Histological grade information not available in 3 patients.

[e] The *p*-values were determined using Kruskal-Wallis test.
