# Supplementary material for: Secretome-Based Identification of ULBP2 as a Novel Serum Marker for Pancreatic Cancer Detection
Source: PLoS One. 2011 May 20;6(5):e20029. doi: 10.1371/journal.pone.0020029 (PMC3098863; doi:10.1371/journal.pone.0020029)
Supplement: Table S5 — Correlation of serum BIGH3, ULBP2, and CA19-9 levels with clinicopathologic characteristics in 154 pancreatic cancer patients. (PDF) [file pone.0020029.s009.pdf]

Supporting Table S5. Correlation of serum BIGH3, ULBP2, and CA19-9 levels with clinicopathologic characteristics in 154 pancreatic cancer patients

Characteristics	No.	BIGH3 (μg/mL) Mean ± SD	*p*-value	ULBP2 (pg/mL) Mean ± SD	*p*-value	CA19-9 (U/mL) Mean ± SD	*p*-value
Gender							
Male	111	1.8 ± 1.6	0.582[a]	199.5 ± 168.4	0.877[a]	64.7 ± 24.6	0.097[a]
Female	43	2.1 ± 1.9		202.0 ± 170.8		60.1 ± 23.7	
Age (years)							
< 70[b]	75	2.1 ± 2.0	0.335[a]	200.4 ± 182.9	0.788[a]	60.2 ± 26.0	0.082[a]
≥ 70	79	1.6 ± 1.3		199.9 ± 154.9		66.4 ± 22.5	
Histological grade[c]							
Well differentiation	12	2.3 ± 2.1	0.522[d]	148.8 ± 101.0	0.377[d]	70.0 ± 13.7	0.553[d]
Moderate differentiation	102	1.9 ± 1.7		198.7 ± 173.1		59.8 ± 26.0	
Poor differentiation	14	1.6 ± 1.4		134.4 ± 99.9		58.7 ± 25.2	
Overall stage[c]							
Stage I-II	106	2.0 ± 1.7	0.663[a]	181.2 ± 158.8	0.394[a]	60.7 ± 24.4	0.538[a]
Stage III-IV	22	1.7 ± 1.6		215.3 ± 178.5		60.4 ± 28.9	
Tumor-node-metastasis (TNM)-T classification[c]							
TNM-T1	7	1.5 ± 0.8	0.077[d]	251.0 ± 150.0	0.418[d]	62.1 ± 20.5	0.536[d]
TNM-T2	27	1.4 ± 1.0		194.0 ± 192.9		54.8 ± 28.4	
TNM-T3	77	2.2 ± 1.8		175.1 ± 150.5		61.8 ± 24.0	
TNM-T4	17	1.7 ± 1.9		204.0 ± 171.1		63.9 ± 26.8	
TNM-N classification[c]							
TNM-N0	57	1.8 ± 1.8	0.179[a]	191.6 ± 155.2	0.383[a]	62.1 ± 25.5	0.384[a]
TNM-N1	71	2.0 ± 1.6		183.4 ± 168.5		59.4 ± 24.9	
TNM-M classification[c]							
No metastasis	125	1.9 ± 1.7	0.953[a]	190.5 ± 162.4	0.016[a]	60.9 ± 25.2	0.278[a]
Distant metastasis	3	1.4 ± 0.7		40.8 ± 27.7		48.5 ± 22.1	

[a] The *p*-values were determined using Wilcoxon test.

[b] Median.

[c] Information of histological grade, overall stage and TNM stage not available in 26 patients.

[d] The *p*-values were determined using Kruskal-Wallis test.
