# Supplementary material for: Secretome-Based Identification of ULBP2 as a Novel Serum Marker for Pancreatic Cancer Detection
Source: PLoS One. 2011 May 20;6(5):e20029. doi: 10.1371/journal.pone.0020029 (PMC3098863; doi:10.1371/journal.pone.0020029)
Supplement: Table S6 — Expression profiles of candidate PC markers in the Human Protein Atlas (HPA) database. (PDF) [file pone.0020029.s010.pdf]

Supporting Table S6. Expression profiles of candidate PC markers in the Human Protein Atlas (HPA) database

Protein name (Gene symbol)	HPA Ab[a]	No. of positive IHC staining[b]				
		3＋	2＋	1＋	－	Positive rate (%)
Alpha-soluble NSF attachment protein (NAPA)	CAB010100	4	8	0	0	100.0
Ras-related protein Rab-14 (RAB14)	HPA026419	0	4	3	2	77.8
60S ribosomal protein L22 (RPL22)	NA	NA	NA	NA	NA	NA
Transcriptional activator protein Pur-beta (PURB)	NA	NA	NA	NA	NA	NA
Ceruloplasmin (CP)	HPA001834	0	2	8	2	83.3
	CAB008591	0	3	4	4	63.6
Complement C1s subcomponent (C1S)	HPA018852	0	2	3	7	41.7
	CAB016722	0	0	0	11	0.0
Annexin A11 (ANXA11)	HPA027545	1	8	3	0	100.0
	CAB004851	0	8	2	0	100.0
ERO1-like protein alpha (ERO1L)	HPA026653	9	3	0	0	100.0
	HPA030053	8	3	0	0	100.0
	CAB034294	3	7	0	0	100.0

[a] ID of antibodies in the Human Protein Atlas (HPA) database (http://www.proteinatlas.org/).
[b] Immunohistochemical staining scores in the HPA: 3＋, strong staining; 2＋, moderate staining; 1＋, weak staining; －, negative staining; NA, not available.
