# Supplementary material for: Secretome-Based Identification of ULBP2 as a Novel Serum Marker for Pancreatic Cancer Detection
Source: PLoS One. 2011 May 20;6(5):e20029. doi: 10.1371/journal.pone.0020029 (PMC3098863; doi:10.1371/journal.pone.0020029)
Supplement: Materials and Methods S1 — (PDF) [file pone.0020029.s011.pdf]

Materials and Methods S1

Harvest of conditioned media from cell lines

The BxPC-3 and MIA PaCa-2 cells were grown to confluence in tissue culture dishes, washed with serum-free media, and incubated in serum-free media for 24 h. The supernatants were then harvested and centrifuged to eliminate the intact cells after which they were concentrated and desalted by centrifugation in Amicon Ultra-15 tubes (5 kDa molecular weight cutoff; Millipore, MA, USA). The cells left on the dishes were washed twice with phosphate-buffered saline (PBS) and lysed in hypotonic buffer (10 mM Tris, pH 7.4, 1 mM EDTA, 1 mM EGTA, 50 mM NaCl, 50 mM NaF, 20 mM $Na_4P_2O_7$, 1 mM Na_3VO_4, 1 mM PMSF, 1 mM benzamidine, 0.5 μg/mL leupeptin, and 1% Triton-X100) on ice for 15 min. The cell lysates were collected and then sonicated on ice followed by centrifugation at 10,000g for 25 min at 4°C. The resulting supernatants were used as the cell extracts. The protein concentrations of the conditioned media and cell extracts were determined using the BCA protein assay reagent from Pierce (Rockford, IL, USA).

Generation of secretome datasets by the GeLC-MS/MS strategy

One-dimensional SDS-PAGE and in-gel digest of proteins - Protein (50 μg) was resolved on 10% SDS-PAGE and stained by 0.5% Coomassie Brillant Blue G-250

(AppliChem GmbH, Darmstadt, Germany). The whole gel lane was cut into 40 pieces and subjected to in-gel tryptic digestion, essentially as previously described [1,2]. Briefly, the gel pieces were destained in 10% methanol (Mallinckrodt Baker, NJ, USA), then dehydrated in acetonitrile (Mallinckrodt Baker, NJ, USA) and dried in a SpeedVac. The proteins were reduced with 25 mM NH_4HCO_3 containing 10 mM dithiothreitol (Biosynth AG, Switzerland) at 60°C for 30 min and alkylated with 55 mM iodoacetamide (Amersham Biosciences, UK) at room temperature for 30 min. After reduction and alkylation, proteins were digested by sequencing grade modified porcine trypsin (20 μg/mL; Promega, Madison, WI, USA) overnight at 37°C. Peptides were extracted with acetonitrile and dried in a SpeedVac.

Reverse phase liquid chromatography-tandem mass spectrometry - Each peptide mixture was reconstituted in HPLC buffer A (0.1% formic acid; Sigma, St. Louis, MO, USA), loaded into a trap column (Zorbax 300SB-C18, 0.3 x 5 mm, Agilent Technologies, Wilmington, DE, USA) at a flow rate of 0.2 μl/min in HPLC buffer A, and separated on a resolving 10-cm analytical C_{18} column (inner diameter, 75 m) with a 15-μm tip (New Objective, Woburn, MA). The peptides were eluted by a linear gradient of 0-10% HPLC buffer B (99.9% acetonitrile containing 0.1% formic acid) for 3 min, 10-30% buffer B for 35 min, 30-35% buffer B for 4 min, 35-50% buffer B for 1 min, 50-95% buffer B for 1 min, and 95% buffer B for 8 min at a flow rate of

0.25 µl/min across the analytical column.

The LC setup was coupled on-line to a 2-D linear ion trap mass spectrometer LTQ-Orbitrap (Thermo Fisher, San Jose, CA, USA) operated using Xcalibur 2.0 software (Thermo Fisher). Intact peptides were detected in the Orbitrap at a resolution of 30,000. Internal calibration was performed using the ion signal of (Si(CH3)2O)6H+ at m/z 445.120025 as a lock mass [3]. The data-dependent procedure that alternated between one MS scan followed by six MS/MS scans for the six most abundant precursor ions in the MS survey scan was applied. The m/z values selected for MS/MS were dynamically excluded for 180 sec. The electrospray voltage applied was 1.8 kV. Both MS and MS/MS spectra were acquired using the one microscan with a maximum fill-time of 1000 and 100 ms for MS and MS/MS analysis, respectively. Automatic gain control was used to prevent over-filling of the ion trap, 5 x 10^4 ions were accumulated in the ion trap for generation of MS/MS spectra. For MS scans, the m/z scan range was 350 to 2,000 Da.

Database searching and bioinformatics - Tandem mass spectra were analyzed using Mascot algorithm (version 2.1, Matrix Science, London, UK). Mascot was set up to search the SwissProt version 57.0 database (selected for Homo sapiens, 20367 entries) assuming the digestion enzyme trypsin. Mascot was searched with a fragment ion mass tolerance of 0.50 Da and a parent ion tolerance of 10.0 ppm. Search

parameters included differential amino acid mass shifts for oxidized methionine (+15.99 Da) and fixed modification for carbamidomethyl cysteine (+57 Da). We measured the false-positive rate of protein identification by searching a random database, in which every sequence entry from the “normal” database was randomly shuffled. The number of hits from each search was categorized based on score, and for each scoring interval, the false-positive rate was calculated as number of random hits/(number of random hits + number of normal hits), and the false positive rate for the peptide sequence matches using the criteria described above was estimated to be < 1% in this study.

The validation of protein identification was performed with the Scaffold software (version 2.1.03, Proteome Software Inc., Portland, OR). The Scaffold software includes a peptide probability score program, PeptideProphet, that aids in the assignment of peptide MS spectrum [4] and the ProteinProphet program that assigns and groups peptides to a unique protein or a protein family if the peptide is shared among several isoforms [5]. ProteinProphet allows filtering of large scale data sets with assessment of predictable sensitivity and false positive identification error rates. In this study, peptide identifications were accepted if they could reach at greater than 90.0% probability; protein identifications were accepted if they could be judged at greater than 99.9% probability and contained at least 3 identified peptides.

Immunohistochemistry

Tissue sections were deparaffinized with xylene, dehydrated with ethanol, heated in 0.01 M citrate buffer (pH 6.0), and treated with blocking buffer (Dako, CA, USA) for 5 minutes at room temperature. The sections were then incubated with anti-BIGH3 (1:100 dilution) or anti-ULBP2 antibodies (1:20 dilution) for 16 hours at 4°C. N-Histofine Simple Stain (Nichirei, Japan) was then applied to slide-mounted samples and incubated for 50 minutes at room temperature, followed by treatment with the substrate, DAB chromogen (Novocastra/Leica Microsystems, IL, USA). Slides were counterstained with hematoxylin.

ELISA for ULBP2

ULBP2 levels in tested samples were determined using a ULBP2 ELISA developed in our laboratory. A goat polyclonal anti-ULBP2 antibody (R&D), diluted to 6 μg/mL in 1X phosphate-buffered saline (PBS), was coated onto a 96-well microplate as the capture antibody. The wells were blocked with 1X PBS containing 1% BSA, and washed three times with 1X PBS between steps. Recombinant ULBP2 protein (R&D) was applied at concentrations ranging from 3.91 to 250 ng/mL to create a standard curve. Protein standards or test samples were incubated in the wells

for 2 hours, followed by incubation with biotin-conjugated detection antibodies (R&D) and AP-conjugated streptavidin. The chromogenic substrate, MUP, was then added and enzymatic reaction products were subsequently measured using a SpectraMax M5 Microplate Reader (MDS, Inc. Toronto, Canada).

Bead-based suspension immunoassay for the detection of serum ULBP2

We have previously established the bead-based immunoassay to determine the plasma MIP-3α level in nasopharyngeal carcinoma patients [6]. Using the similar technique, the ULBP2 bead-based immunoassay was developed to measure serum ULBP2 concentration. Briefly, filter-bottom 96-well microplates (Millipore, MA, USA) were blocked with assay buffer (Bio-Rad Laboratories, Taipei, Taiwan) for 10 minutes and incubated with goat anti-ULBP2 antibody (R&D Systems, MN, USA), which was covalently coupled to COOH beads (Bio-Rad Laboratories) using the Bio-Plex Amine Coupling Kit (Bio-Rad Laboratories) before use. After washing with assay buffer (Bio-Rad Laboratories), 50 μl of serum samples or protein standards (recombinant ULBP2, R&D Systems) were then added to the microplates and incubated for 1 hour at room temperature in the dark. After washing, the samples in the wells were treated with biotin-conjugated goat anti-ULBP2 antibody (R&D Systems) for 1 hour at room temperature in the dark, followed by treatment with

phycoerythrin-conjugated streptavidin (Bio-Rad Laboratories) for 10 minutes. After washing, the beads were suspended in assay buffer and analyzed using the Bio-Plex 200 system (Bio-Rad Laboratories). Serum ULBP2 concentration were calculated from standard curves using Bio-Plex Manager software version 4.2 (Bio-Rad Laboratories).

References

1. Wang CL, Wang CI, Liao PC, Chen CD, Liang Y, et al. (2009) Discovery of retinoblastoma-associated binding protein 46 as a novel prognostic marker for distant metastasis in nonsmall cell lung cancer by combined analysis of cancer cell secretome and pleural effusion proteome. J Proteome Res 8: 4428-4440.

2. Wu CC, Hsu CW, Chen CD, Yu CJ, Chang KP, et al. (2010) Candidate serological biomarkers for cancer identified from the secretomes of 23 cancer cell lines and the human protein atlas. Mol Cell Proteomics 9: 1100-1117.

3. Olsen JV, de Godoy LM, Li G, Macek B, Mortensen P, et al. (2005) Parts per million mass accuracy on an Orbitrap mass spectrometer via lock mass injection into a C-trap. Mol Cell Proteomics 4: 2010-2021.

4. Keller A, Nesvizhskii AI, Kolker E, Aebersold R (2002) Empirical statistical model to estimate the accuracy of peptide identifications made by MS/MS and database

search. Anal Chem 74: 5383-5392.

5. Nesvizhskii AI, Keller A, Kolker E, Aebersold R (2003) A statistical model for identifying proteins by tandem mass spectrometry. Anal Chem 75: 4646-4658.

6. Chang KP, Chang YT, Wu CC, Liu YL, Chen MC, et al. (2011) Multiplexed immunobead-based profiling of cytokine markers for detection of nasopharyngeal carcinoma and prognosis of patient survival. Head Neck In press.
